# Treg cells as a protective factor for Hashimoto`s thyroiditis: a Mendelian randomization study

**DOI:** 10.3389/fendo.2024.1347695

**Published:** 2024-03-08

**Authors:** Jinzhou Guo, Gao Si, Fuchun Si

**Affiliations:** ^1^ Academy of Zhongjing, Henan University of Chinese Medicine, Zhengzhou, China; ^2^ Laboratory of Traditional Chinese Medicine (TCM) Syndrome and Prescription Signaling, Academy of Zhongjing, Zhengzhou, China; ^3^ Henan Key Laboratory of Traditional Chinese Medicine (TCM) Syndrome and Prescription Signaling, Henan International Joint, Zhengzhou, China; ^4^ Department of Orthopedic, Peking University Third Hospital, Beijing, China

**Keywords:** Hashimoto`s thyroiditis, Treg cells, immunity, causal inference, MR analysis

## Abstract

**Background and objectives:**

Hashimoto’s thyroiditis (HT), a chronic autoimmune disorder impacting thyroid function, is a growing public health concern. The relationship between Treg cells and HT has been extensively studied, with Treg cells considered crucial in suppressing HT progression. However, these studies have mainly been observational, limiting our understanding of Treg cells’ impact on HT risk. Leveraging large datasets, we utilized Mendelian randomization (MR) analysis to examine the causal association between Treg cell biomarkers and HT, providing additional validation for these relationships.

**Methods:**

Comprehensive two-sample Mendelian randomization analysis was performed to determine the causal association between Treg cells signatures and HT in this study. Based on publicly available genetic data, we explored causal associations between 165 Treg cells signatures and HT risk.

**Results:**

The European cohort study has identified five Treg cell phenotypes that causally protect against HT risk. Resting Treg %CD4 (OR = 0.975, 95% CI = 0.954~0.998, *P* = 0.030); CD4 on resting Treg (OR = 0.938, 95% CI = 0.882~0.997, *P* = 0.041; CD28- CD8dim %CD8dim (OR = 0.983, 95% CI = 0.969~0.998, *P* = 0.030); CD25 on CD39+ resting Treg (OR = 0.926, 95% CI = 0.864~0.991, *P* = 0.026); 5) CD28 on activated & secreting Treg (OR = 0.969, 95% CI = 0.942~0.996, *P* = 0.025). The Asian cohort study has identified four Treg cell phenotypes negatively correlated with the risk of HT. CD25hi %T cell (OR = 0.635, 95% CI = 0.473~852, *P* = 0.002); CD4 Treg %CD4 (OR = 0.829, 95% CI = 0.687~1.000, *P* = 0.050); CD127-CD8br %T cell (OR = 0.463, 95% CI =0.311~0.687, *P*< 0.001); CD3 on resting Treg (OR = 0.786, 95% CI = 0.621~0.994, *P* = 0.044).

**Conclusion:**

Our study has demonstrated the close connection between Treg cells and HT by genetic means, thus providing foundational basis for future research.

## 1 Introduction

Hashimoto’s Thyroiditis (HT) is a common chronic autoimmune disease affecting thyroid function (1, 2), named after the Japanese scientist Hashimoto, who first described it as a type of autoimmune thyroiditis typically resulting in hypothyroidism (3). The primary pathological changes include extensive enlargement of the thyroid gland, follicular hyperplasia, and the presence of plasma cells and lymphocytes (4). The incidence of HT has been rapidly increasing worldwide in recent years (4), with rates ranging between 27 and 448 per 100,000 (5). Consequently, HT has become a critical public health issue. And studies have shown that, HT is a known risk factor for thyroid cancer development (6). Therefore, Precise diagnosis and effective treatment of HT are essential for enhancing patients’ quality of life and reducing their risk of thyroid cancer (7).

Despite the ongoing debate regarding the etiology and pathogenesis of HT, there is a prevailing agreement that autoimmunity maintains a crucial role in its pathogenesis (2, 8). Recently, regulatory T cells (Treg) have emerged as crucial factors in HT. Treg cells, a CD4 + T cell population, exert immunosuppressive effects on excessive immune responses (9–11). Animal experiments (12–14) indicate that the lack of Tregs could result in the spontaneous advancement of autoimmune thyroiditis. Clinical evidence (10, 15) further substantiates that Treg frequency in circulation is reduced among HT patients. These findings suggest that a decrease in Treg numbers or dysfunction leads to impaired immune tolerance towards autoantigenic stimuli, contributing to the development of Hashimoto’s thyroiditis. Consequently, Treg immunomodulation has emerged as a crucial matter in recent HT immunology research. However, conflicting findings exist in other studies. Glick et al. reported no significant difference in CD4+CD25+ Tregs frequency in HT (16). Even one study found a significantly higher proportion of CD4+Foxp3+ Tregs in autoimmune thyroid disease (AITD) patients (17). The variation may be ascribed to the dual regulatory function of the immune system and the heterogeneity induced by the multiple Treg cell subtypes (18). Therefore, investigating the association between Treg cell subtypes and HT could potentially have a crucial role in precise immunotherapy for HT.

Mendelian randomization (MR) utilizes single nucleotide polymorphisms (SNPs) as instrumental variables (IVs) to infer potential causal relationships between exposure and outcomes (19, 20). follows the Mendelian gamete random allocation principle as well as the principle of free combination. This approach effectively mitigates the impact of confounding factors and reverse causation commonly encountered in conventional observational studies (21). MR is widely used in genetic epidemiology (22). With the large datasets currently available, MR analysis may be a good tool for exploring novel biomarkers in Treg cells that have a causal impact on the risk of Hashimoto’s thyroiditis. Therefore, this study employs a two-sample Mendelian randomization approach, utilizing large-scale GWAS databases to explore the causal relationship between immune cell phenotypes and HT risk, To our knowledge, there haven’t been previous studies using MR to assess the causal impact of Treg cell traits on the risk of HT.

## 2 Materials and methods

### 2.1 Study design

The current study applied a two-sample MR method to analyze causal relationships between 165 Treg traits and HT. Therefore, to ensure the validity of causal inference, the IVs relies on three fundamental assumptions: 1) Genetic variants have a direct connection with the exposure; 2) Genetic variants are not associated with potential confounding factors between exposure and outcome; and 3) Genetic variants do not influence the outcome through pathways unrelated to the exposure. All MR analyses were conducted using publicly available summary statistics, thus eliminating the need for additional ethical approval or informed consent.

### 2.2 Data source

The GWAS summary statistics for a diverse range of Treg traits were retrieved from the GWAS Catalog (23), the GWAS Catalog Accession Numbers are detailed in **Supplementary Table 1**. The original GWAS for immune traits utilized data from 3,757 European individuals, these datasets encompass an extensive collection of 165 Treg traits. The GWAS summary statistics for HT were sourced from the GWAS Catalog, encompassing datasets from both European (accession number GCST90018855) (24) and Asian populations (accession number GCST90018635). The initial GWAS analysis for the European cohort involved 395,640 individuals, including 15,654 cases and 379,986 controls. Likewise, the original GWAS analysis for the Asian cohort included 173,193 participants of East Asian descent, predominantly of Japanese origin, with 537 cases and 172,656 controls.

### 2.3 Instrumental variable selection

Genetic variants were utilized as IVs in this study. Significant SNPs that were associated with the Treg cell phenotype with genome-wide significance (*P*<1×10^-5^) (25) were selected based on recent research. To minimize bias due to linkage disequilibrium (LD), the following conditions were imposed: R^2^< 0.001 and distance = 10,000 kb (26). IVs with an F< 10 were excluded to ensure strong correlation of SNPs with HT risk (**Supplementary Table 2**). The formula for calculating *F* is: *F* = ${\text{R}}^{2}\times \frac{\text{K}-2}{1-{\text{R}}^{2}}$ , where K is the sample size, *R* ^2^ is the proportion of variance explained by SNPs in the exposure database (27). The formula for calculating R^2^ is: ${\text{R}}^{2}=\frac{2\times \beta \times \text{eaf}\times (1-\text{eaf})}{2\times \beta^{2}\times \text{eaf}\times (1-\text{eaf})+2\times {\text{se}}^{2}\times \text{K}\times \text{eaf}\times (1-\text{eaf})}$ , where eaf is the effect allele frequency, *β* is the allele effect value, K is the sample size, and se is the Standard Error. The exposure and outcome datasets were merged, and incompatible alleles and palindromic SNPs were eliminated. The phenotypes associated with the remaining SNPs were identified utilizing the Human Genotype-Phenotype Association Database (http://www.phenoscanner.medschl.cam.ac.uk/) (28). The remaining SNPs were ultimately utilized as instrumental variables for exposure.

### 2.4 Statistical analysis

The univariable MR analysis was performed using the R packages “TwoSampleMR”. Our analysis primarily utilized the inverse variance weighting (IVW) method (29). We employed four additional methods to assess the reliability and consistency of our results. These methods include MR-Egger regression (30), the weighted median estimator (WME) (31), mode-based simple estimation (32), and mode-based weighted estimation. All causal estimates were converted to odds ratios (ORs) for the outcome which was a dichotomous phenotype.

**Figure 1:**
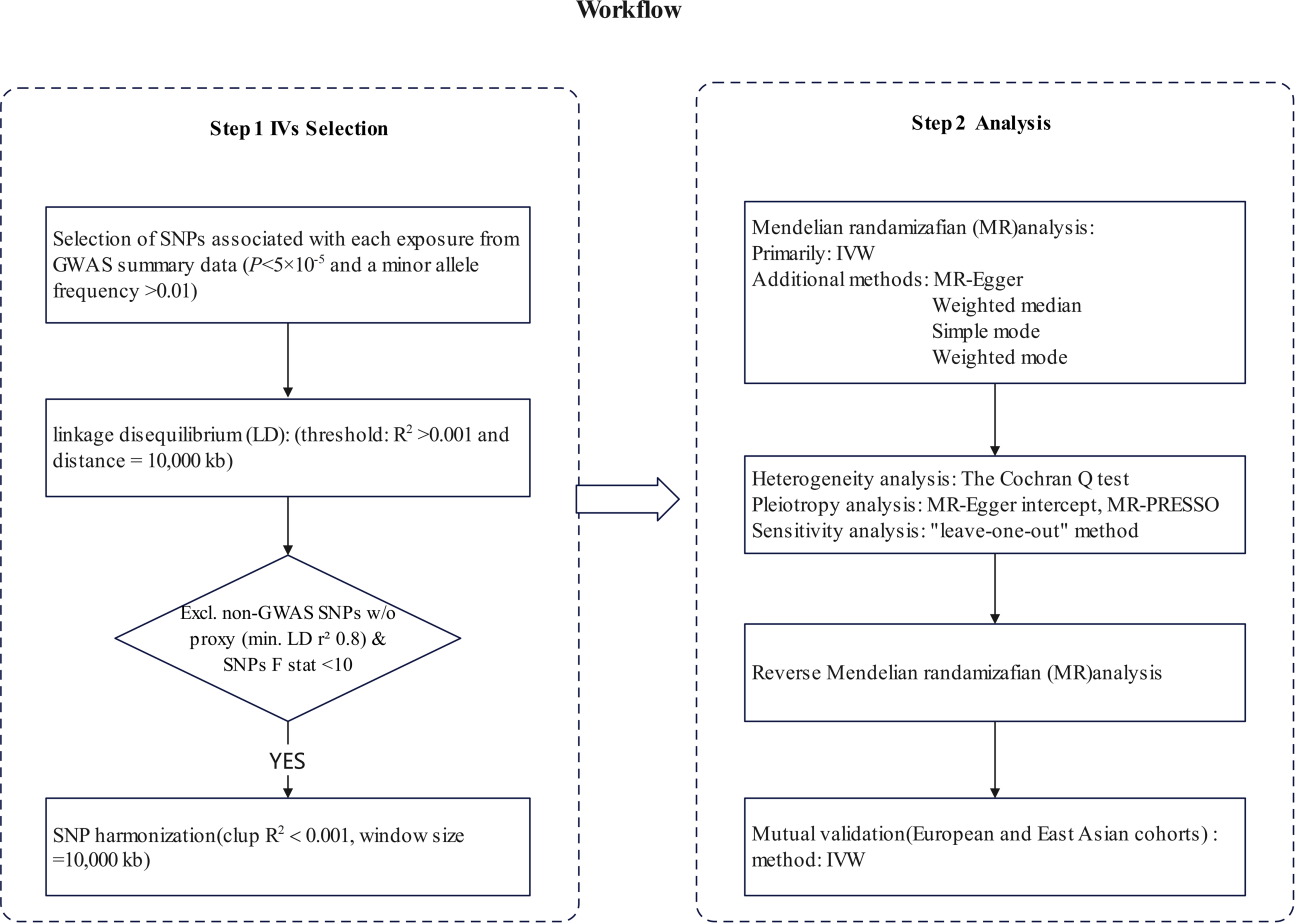
The steps of Mendelian randomization (MR)analysis. SNP, single-nucleotide polymorphism; IVW, inverse variance weighting; MR-PRESSO, Mendelian Randomization Pleiotropy RESidual Sum and Outlier.

Heterogeneity among the individual estimates of genetic variation was evaluated using The Cochran Q test. No significant heterogeneity was observed when *P* > 0.05. We used the MR-Egger intercept to assess the effect of horizontal pleiotropy (33). Sensitivity analysis was conducted using the “leave-one-out” method to assess the impact of individual SNPs on the causal relationship. The Mendelian Randomization Pleiotropy RESidual Sum and Outlier (MR-PRESSO) method were utilized as indicators to evaluate and correct the level of pleiotropy (34), the MR-PRESSO was conducted using the R package “MRPRESSO” (**Figure 1**).

## 3 Results

### 3.1 The MR analysis of results from European cohorts

**Figure 2:**
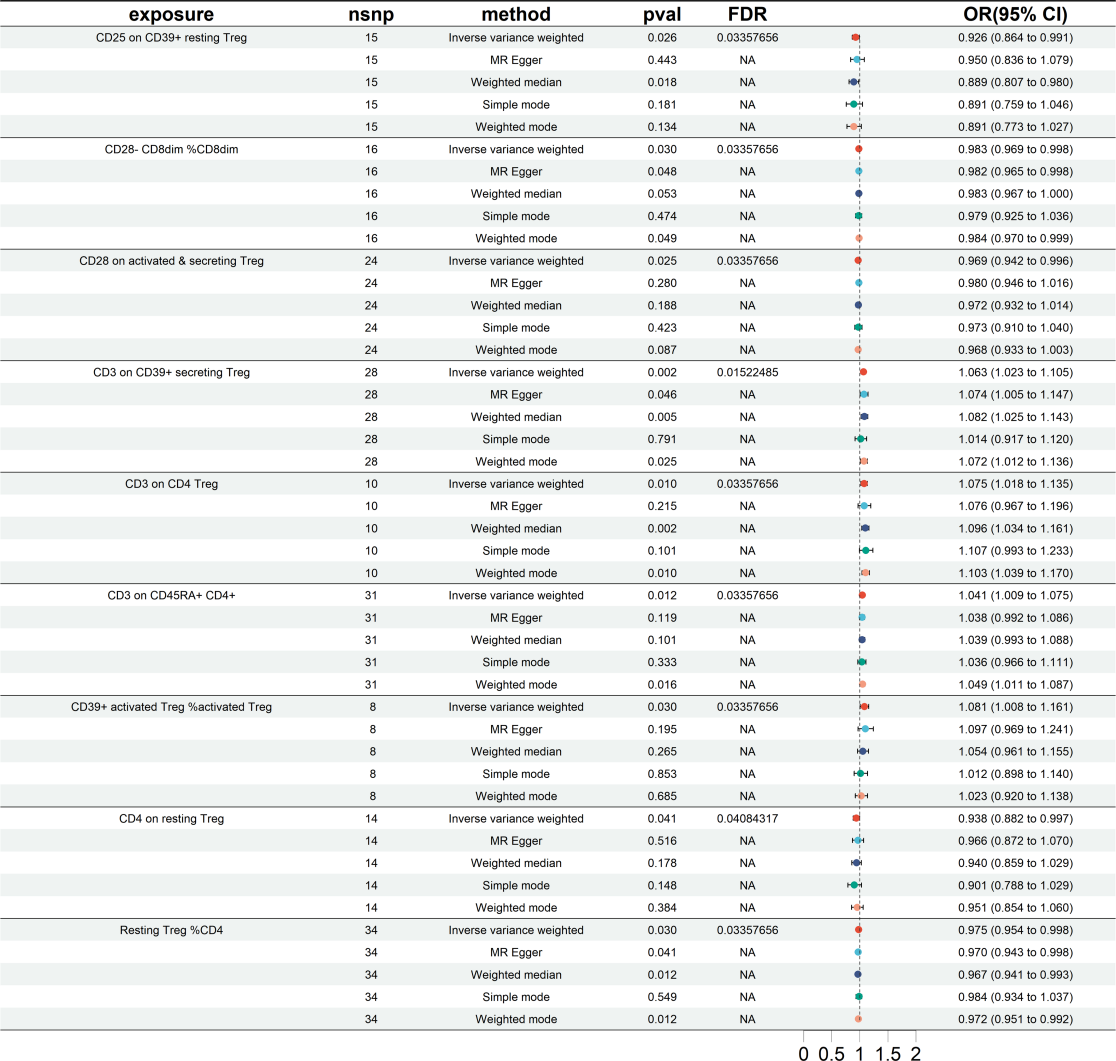
Forest plots showing significantly causal relationships between 9 Treg cell traits and HT (European cohorts). nsnp, number of single-nucleotide polymorphism; OR, odds ratio; CI, confidence interval.

**Figure 3:**
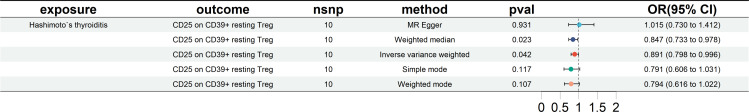
Forest plots showing reverse MR analysis results (European cohorts). nsnp, number of single-nucleotide polymorphism; OR, odds ratio; CI, confidence interval.

The results of the European cohorts study revealed nine Treg cell phenotypes significantly linked with HT risk. **Figure 2** presents these findings. Of the identified Treg cell features, we found five that were protective against HT risk: 1) CD25 on CD39+ resting Treg (IVW OR = 0.926, 95% CI = 0.864~0.991, *P* = 0.026); 2) CD28- CD8dim %CD8dim (IVW OR = 0.983, 95% CI = 0.969~0.998, *P* = 0.030); 3)CD28 on activated & secreting Treg (IVW OR = 0.969, 95% CI = 0.942~0.996, *P* = 0.025); 4)CD4 on resting Treg (IVW OR = 0.938, 95% CI = 0.882~0.997, *P* = 0.041); 5) Resting Treg %CD4 (IVW OR = 0.975, 95% CI = 0.954~0.998, *P* = 0.030), as well as four other Treg cell subtypes (CD3 on CD39+ secreting Treg, CD3 on CD4 Treg, CD3 on CD45RA+ CD4+, CD39+ activated Treg %activated Treg). Reverse MR analysis revealed a negative causal relationship between HT and CD25 on CD39+ resting Treg (IVW OR = 0.891, 95% CI = 0.798~0.996, *P* = 0.042) (see **Figure 3**). The sensitivity test results are displayed in **Table 1**. Additionally, the leave-one-out analysis corroborated this finding (see **Supplementary Figure 1**). Causal effect estimates were depicted using scatter plots (see **Supplementary Figure 2**). Moreover, funnel plots, providing a visual appraisal, revealed no heterogeneity in the causal effect (see **Supplementary Figure 3**).

**Table 1:** Result of sensitivity analysis (European cohorts).

Exposure	Outcome	Method	Cochran’s Q			Pleiotropy		
			Q	Q_df	Q_pval	Egger intercept	SE	pval
CD25 on CD39+ resting Treg	Hashimoto`s thyroiditis	MR Egger	14.26	13	0.356	-0.0067	0.014	0.641
		IVW	14.51	14	0.412			
		MR-PRESSO						0.457
CD28- CD8dim %CD8dim	Hashimoto`s thyroiditis	MR Egger	18.33	14	0.192	0.0044	0.008	0.607
		IVW	18.70	15	0.228			
		MR-PRESSO						0.425
CD28 on activated & secreting Treg	Hashimoto`s thyroiditis	MR Egger	17.89	22	0.713	-0.0081	0.007	0.298
		IVW	19.02	23	0.700			
		MR-PRESSO						0.758
CD3 on CD39+ secreting Treg	Hashimoto`s thyroiditis	MR Egger	31.89	26	0.197	-0.0035	0.010	0.734
		IVW	32.04	27	0.231			
		MR-PRESSO						0.226
CD3 on CD4 Treg	Hashimoto`s thyroiditis	MR Egger	11.95	8	0.153	-0.0004	0.019	0.985
		IVW	11.95	9	0.216			
		MR-PRESSO						0.311
CD3 on CD45RA+ CD4+	Hashimoto`s thyroiditis	MR Egger	39.70	29	0.089	0.0016	0.008	0.835
		IVW	39.77	30	0.110			
		MR-PRESSO						0.104
CD39+ activated Treg %activated Treg	Hashimoto`s thyroiditis	MR Egger	7.28	6	0.296	-0.0056	0.020	0.790
		IVW	7.37	7	0.391			
		MR-PRESSO						0.452
CD4 on resting Treg	Hashimoto`s thyroiditis	MR Egger	11.91	12	0.453	-0.0081	0.012	0.499
		IVW	12.39	13	0.496			
		MR-PRESSO						0.527
Resting Treg %CD4	Hashimoto`s thyroiditis	MR Egger	47.07	32	0.042	0.0065	0.010	0.508
		IVW	47.73	33	0.047			
		MR-PRESSO						0.070
Hashimoto`s thyroiditis	CD25 on CD39+ resting Treg	MR Egger	4.74	8	0.785	-0.0221	0.027	0.435
		IVW	5.41	9	0.797			
		MR-PRESSO						0.708

IVW, Inverse variance weighted; MR-PRESSO, MR-Pleiotropy Residual Sum and Outlier.

### 3.2 The MR analysis of results from East Asian cohorts

**Figure 4:**
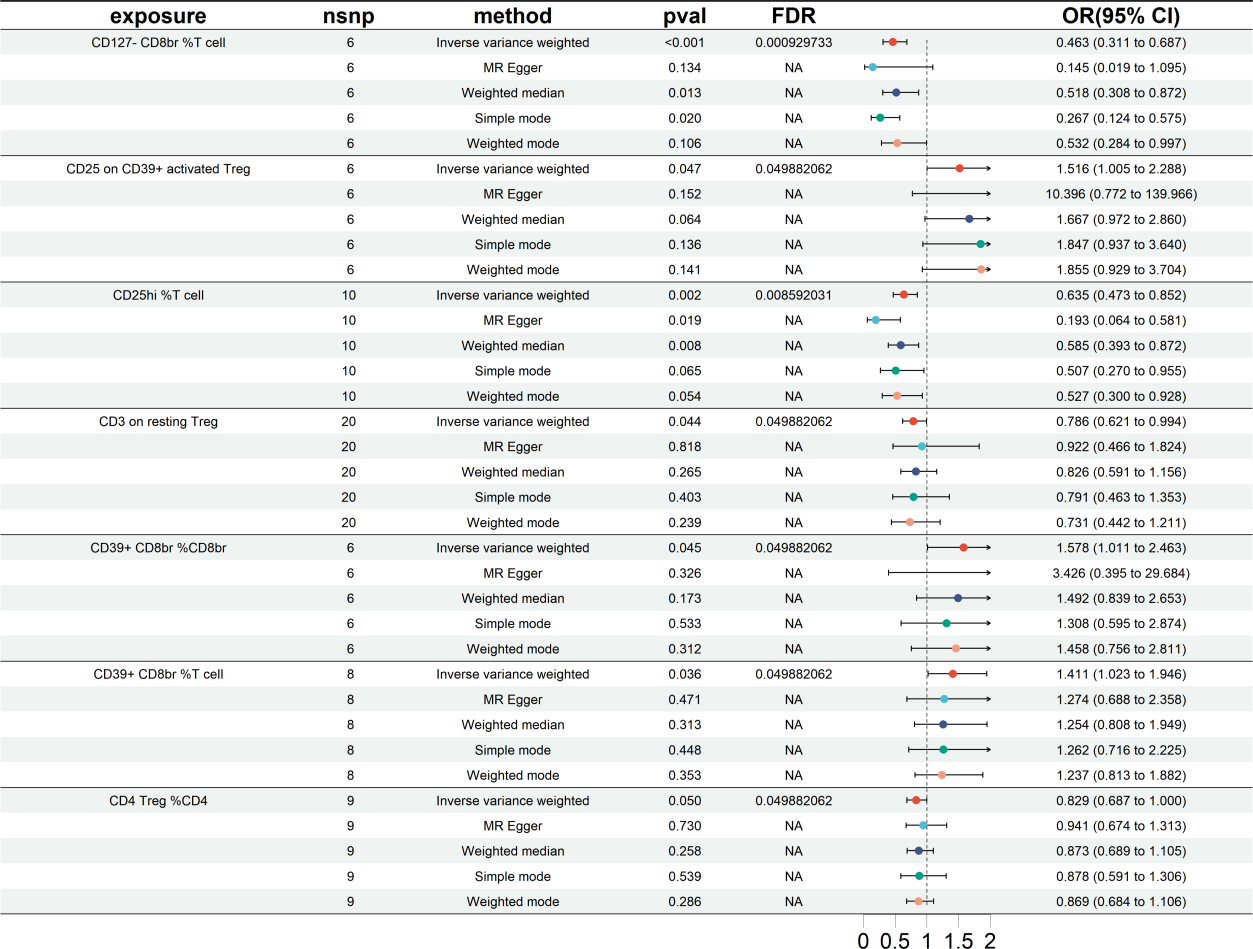
Forest plots showing significantly causal relationships between 7 Treg cell traits and HT (East Asian cohorts). nsnp, number of single-nucleotide polymorphism; OR, odds ratio; CI, confidence interval.

The study’s findings in the East Asian cohorts indicate that seven Treg cell phenotypes are significantly linked to HT risk. **Figure 4** demonstrates these associations. Four Treg cell features were identified as beneficial in mitigating HT risk: 1) CD127-CD8br %T cell (OR = 0.463, 95% CI =0.311~0.687, *P*< 0.001); 2) CD25hi %T cell (OR = 0.635, 95% CI = 0.473~852, *P* = 0.002); 3) CD3 on resting Treg (OR = 0.786, 95% CI = 0.621~0.994, *P* = 0.044); 4) CD4 Treg %CD4 (OR = 0.829, 95% CI = 0.687~1.000, *P* = 0.050). And 3 other Treg cell subtypes (CD25 on CD39+activated Treg, CD39+CD8br %CD8br, CD39+CD8br %T cell).

Reverse MR analysis revealed no significant causal relationship between HT and Treg cell phenotype. The results of the sensitivity test are shown in **Table 2**. In the same vein, the leave-one-out test also supported this observation (**Supplementary Figure 4**). The scatter plots see **Supplementary Figure 5**, funnel plots see **Supplementary Figure 6**.

**Table 2:** Result of sensitivity analysis (East Asian cohorts).

Exposure	Outcome	Method	Cochran’s Q			Pleiotropy		
			Q	Q_df	Q_pval	Egger intercept	SE	pval
CD127- CD8br %T cell	Hashimoto`s thyroiditis	MR Egger	4.17	4	0.38	0.19	0.165	0.32
		IVW	5.53	5	0.35			
		MR-PRESSO						0.45
CD25 on CD39+ activated Treg	Hashimoto`s thyroiditis	MR Egger	1.74	4	0.78	-0.28	0.19	0.22
		IVW	3.90	5	0.56			
		MR-PRESSO						0.6
CD25hi %T cell	Hashimoto`s thyroiditis	MR Egger	3.59	8	0.89	0.20	0.093	0.06
		IVW	8.42	9	0.49			
		MR-PRESSO						0.54
CD3 on resting Treg	Hashimoto`s thyroiditis	MR Egger	13.95	18	0.73	-0.03	0.061	0.63
		IVW	14.19	19	0.77			
		MR-PRESSO						0.78
CD39+ CD8br %T cell	Hashimoto`s thyroiditis	MR Egger	4.85	6	0.56	0.03	0.066	0.72
		IVW	5.00	7	0.66			
		MR-PRESSO						0.71
CD39+ CD8br %CD8br	Hashimoto`s thyroiditis	MR Egger	0.50	4	0.97	0.01	0.023	0.72
		IVW	1.01	5	0.96			
		MR-PRESSO						0.95
CD4 Treg %CD4	Hashimoto`s thyroiditis	MR Egger	3.71	7	0.81	-0.04	0.046	0.4
		IVW	4.51	8	0.81			
		MR-PRESSO						0.79

IVW, Inverse variance weighted; MR-PRESSO, MR-Pleiotropy Residual Sum and Outlier.

### 3.3 Cross-validation results comparing the European and East Asian cohorts

**Figure 5:**
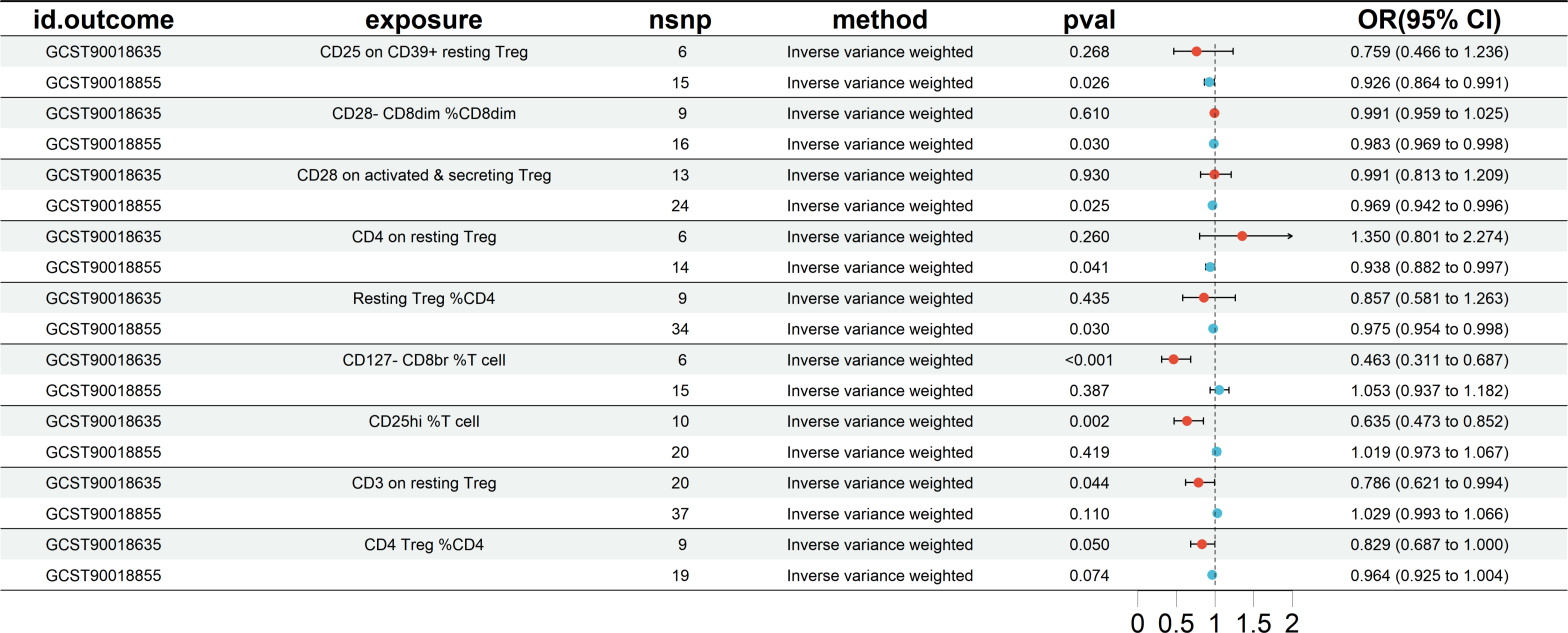
The MR analysis results of mutual validation was performed between European groups and East Asian groups. nsnp, number of SNP. CI, confidence interval.

Mutual validation was performed between European groups and East Asian groups. The MR results showed a total of five results for both datasets that showed a similar propensity to protect as the preliminary analysis (IVW OR<1), but the significance did not reach statistical significance, as shown in **Figure 5**. Specifically included were CD25 on CD39+ resting Treg (East Asian OR = 0.759, and European OR = 0.926), CD28- CD8dim %CD8dim (East Asian OR = 0.991, and European OR = 0.983), CD28 on activated & secreting Treg (East Asian OR = 0.991, and European OR = 0.969), Resting Treg %CD4 (East Asian OR = 0.857, and European OR = 0.975), and CD4 Treg %CD4 (East Asian OR = 0.829, and European OR = 0.964). And 4 other Treg cell subtypes (CD4 on resting Treg, CD127-CD8br %T cell, CD25hi %T cell, and CD3 on resting Treg).

In conclusion, through MR analysis, we identified five treg cell characteristics of interest that were protective against the risk of HT. They include Resting Treg %CD4, CD4 Treg %CD4, CD28 on activated & secreting Treg, CD28- CD8dim %CD8dim, and CD25 on CD39+ resting Treg. These features exhibit a negative correlation with the risk of HT, suggesting a potential protective role against HT.

## 4 Discussion

HT is an autoimmune disease in which Treg cells play a crucial role in preventing the onset and progression by suppressing the production of harmful autoantibodies targeting thyroglobulin (TG) and thyroid peroxidase (TPO) (35) by autoreactive B cells, thus preventing the destruction caused by these autoreactive B cells (36, 37). Furthermore, Treg cells protect the thyroid tissue from attack by the release of immunosuppressive molecules such as IL-10 and TGF- β (38). In addition to their role in immune homeostasis, Treg cells play indispensable roles in self-tolerance (39). Numerous animal experiments (12, 13, 40) have demonstrated that the absence of Tregs can trigger the spontaneous development of autoimmune thyroiditis. Meanwhile, clinical evidence (10) suggests a decrease in the frequency of circulating Tregs among individuals diagnosed with HT. However, there are also reports indicating that Treg cells may contribute to the progression of HT (16, 17). Such discrepancies may be attributed to different detection markers or subsets with distinct phenotypes. Considering the dissection of Tregs into subpopulations and limited data reported in autoimmune thyroiditis, in order to delve deeper into the potential Treg cell phenotypes associated with reduced risk of HT, we comprehensively examined the causal relationship between 165 Treg cell phenotypes and HT utilizing public genetic data and employing Mendelian randomization methods (41). MR employs genetic variants as instrumental variables, which are fixed at conception, to perform causal inferences regarding the effects of modifiable risk factors. This approach can effectively mitigate certain types of confounding (42). Notably, this study represented a pioneering analysis utilizing Mendelian randomization to scrutinize the causal association between multiple Treg cell traits and HT. In our study, five protective factors were ultimately identified.

The first trait is Resting Treg %CD4. Previous research has demonstrated that by utilizing FoxP3 and CD45RA, three distinct subpopulations can be differentiated based on both phenotypic and functional characteristics: the CD45RA + FoxP3lo resting Treg (rTreg), the CD45RA-FoxP3hi effector Treg (eTreg), and the CD45RA-FoxP3lo cells (Fraction III, FIII). Resting Treg cells have the ability to suppress autoimmune reactions (43), which prevents the immune system from launching attacks against its own tissues. Resting-phase Treg has been demonstrated to decrease in autoimmune disease, indicating a potential role for Resting Treg in mitigating autoimmune disease (44, 45). Research on resting Tregs in HT is limited; currently, only one clinical study has reported no significant differences in the proportion of resting Tregs between HT patients and healthy volunteers, and there is no direct research establishing a link between resting Treg %CD4 and HT. However, HT is considered an autoimmune disease, and our MR analysis results also indicate a negative correlation between the percentage of resting Treg %CD4 and the risk of HT, suggesting a potential protective role of resting Treg %CD4 in HT.

The second protective trait is CD4 Treg %CD4. CD4 is a membrane protein expressed extensively on the surface of T cells, which is indispensable for recognizing most antigens in the body. The CD4 co-receptor augments the T cell sensitivities to antigens by 30- to 100-fold (46), and its primary function is to bind with MHC-II (major histocompatibility complex class II) (47) molecules that are present on the surfaces of antigen-presenting cells, mainly associating with the β-chain binding. This association allows CD4+ T cells to closely associate with antigen-presenting cells, which initiates T cell activation and guides their immune response (48, 49). This process is crucial for regulating the immune system’s normal functionality and preventing excessive immune responses. Hence, the activation and execution of Treg cells’ biological functions are integrally linked to CD4 molecules. Experimental *in vivo* studies have indicated a significant reduction in the proportion of CD4CD25 Treg cells in patients with HT compared to healthy controls (HCs) (50). One meta-analysis showed that newly diagnosed HT patients have lower levels of Tregs compared to HCs (51). Another meta-analysis investigated the percentage of peripheral blood Tregs among CD4+ T cells in patients with AITDs. The results revealed that the proportions of Tregs among CD4+ T cells in untreated AITDs were significantly lower than those in HCs. However, unfortunately, no significant differences were observed in untreated HT patients (52). Although our findings suggest a negative correlation between CD4 Treg %CD4 and HT, further studies are warranted to establish and validate this relationship, considering the limited research on the association between CD4 Treg %CD4 and HT.

The third and fourth involve CD28 on activated & secreting Treg, and CD28- CD8dim %CD8dim. CD28 is a type I transmembrane glycoprotein receptor typically expressed on most CD4^+^ T cells (53). It serves as a co-stimulatory receptor expressed differently at specific stages of T cell differentiation and within specific T cell subgroups to regulate immune responses (54). T cell activation involves two signals. The first signal involves the T cell receptor (TCR) recognizing and binding specifically to the antigenic peptide-major histocompatibility complex (MHC) on antigen-presenting cells. The second signal results from the interaction between T cells and a range of co-stimulatory proteins expressed on the surface of antigen-presenting cells, generating co-stimulatory signals (55). It is worth noting that the most crucial interaction among these proteins is the binding of CD28 to CD80/CD86 (56). There is ongoing clinical development of a solitary CD28 agonist antibody, specifically TAB 08 (TGN 1412) (57, 58). However, the potential risks and benefits of this immunological agonist remain unclear and require further study (59). Additionally, CD28 superagonistic monoclonal antibodies (CD28SAB) have been found to preferentially activate and expand immuno-suppressive regulatory T (Treg) cells. In preclinical tests, CD28SABs have demonstrated the ability to alleviate the pathogenesis, progression, and clinical course of autoimmune diseases (60). Although our results revealed a negative association between CD28 on activated & secreting Treg and the risk of HT, which is consistent with previous findings, it is noteworthy that the CD28- CD8dim %CD8dim phenotype was also negatively associated with the risk of HT. Previous studies indicated that since CD28 was expressed not only on Treg but also on effector T cells, over-activation of effector T cells aggravated the progression of thyroid tissue injury or inflammation (61). Hyperreactivity of B7/CD28 signaling is closely linked with the development of autoimmune diseases (62). Cytotoxic T-Lymphocyte Antigen-4 (CTLA-4) is an inhibitory protein that competitively binds to B7 molecules (CD80 and CD86) in competition with CD28 (63), exerting immune co-suppressive responses (64), making it a highly regarded therapeutic target in the treatment of autoimmune diseases. It has been reported that using CTLA-4 fusion protein to block the interaction between CD28 and B7 can inhibit the autoimmune response in experimental rheumatoid arthritis (RA) (65). Additionally, inhibiting the CD80-CD28-mediated signaling pathway via specific RNAi and antibodies to block the interaction between CD28 and B7 reduced immune cell activation and reversed the damage in a pathological lupus nephritis model (66). Therefore, We believe that solely activating or inhibiting the CD28 molecule may not be ideal for improving HT.

The final parameter is CD25 on CD39+ resting Treg. The α-chain of the interleukin-2 receptor (IL-2R) is CD25, which is expressed mainly on immune-related cells’ surfaces, including activated T cells, B cells, and NK cells. It is another Treg surface marker that interacts with the ligand IL-2 and is involved in a variety of immune responses (67, 68). In 1995, Sakaguchi et al. identified a subset of CD4+ T lymphocytes expressing abundant CD25 in thymectomized mice aged 3 days and other autoimmune models, demonstrating their capacity to impede disease progression (69). Similarly, the elevated counts of CD4+ CD25 high HLA-DR+ cells in HT patients may suggest compensatory expansion of this Treg subpopulation to suppress the immune response (70). In an *in vivo* study (71), CD4+CD25+Foxp3+ Tregs were revealed to play a role in the pathogenesis of experimental autoimmune thyroiditis (EAT), a murine model of HT. CD25 expression is indispensable for Tregs’ differentiation and adaptive proliferation. Mice lacking IL2Rα or IL2Rβ chains manifest severe systemic autoimmunity, a condition ameliorated by transferring CD4 + CD25 + T cells from wild-type mice (72). Furthermore, Mice with deficiencies in IL-2 or the IL-2 receptor exhibit diminished Treg generation, precipitating autoimmune disorders (73). Our findings indicate a negative correlation between HT risk and CD25 on CD39+ resting Treg, consistent with previous research. However, the reverse MR analysis revealed a significant causal relationship between HT risk and CD25 on CD39+ resting Treg, which may be related to unknown confounding factors. Our MR analysis attempted to exclude confounding factors, but there may still be unidentified factors that could impact the results.

Our study has inherent limitations. First, we cannot entirely exclude the potential influence of other confounding factors on this association. Considering the possible presence of additional variables, further exploration of the precise role of these Treg cell phenotypes in the pathogenesis of HT is warranted based on our study findings. Second, despite leveraging GWAS data from European and Asian populations, the generalizability of our findings to other ethnic groups remains subject to constraints. Third, the absence of individual-specific data precluded a more nuanced, stratified analysis of the population, introducing potential inaccuracies into our speculative conclusions. Finally, Our study reported on the Treg cell phenotypes that may decrease the risk of HT in both European and Asian populations. We conducted mutual validation in two study cohorts and applied a more lenient FDR correction for p-values. Although this effectively reduces the occurrence of false-negative results, it inevitably leads to an increased probability of type I errors.

## 5 Conclusions

In summary, we have established a causal association between five distinct Treg cell phenotypes and HT through an extensive two-way Mendelian randomization analysis, elucidating the intricate interplay between the immune system and HT. Furthermore, our research has effectively mitigated the impact of inevitable confounding factors, reverse causality, and other variables. This may serve as a foundational basis for researchers to delve into Treg cells as potential immunotherapeutic targets for HT.

## Data availability statement

The original contributions presented in the study are included in the article/**Supplementary Material**. Further inquiries can be directed to the corresponding author.

## Author contributions

JG: Data curation, Methodology, Software, Visualization, Writing – original draft. GS: Investigation, Methodology, Software, Validation, Visualization, Writing – review & editing. FS: Conceptualization, Formal Analysis, Funding acquisition, Project administration, Supervision, Writing – review & editing.
