# Supplementary material for: Treg cells as a protective factor for Hashimoto`s thyroiditis: a Mendelian randomization study
Source: Front Endocrinol (Lausanne). 2024 Mar 8;15:1347695. doi: 10.3389/fendo.2024.1347695 (PMC10957564; doi:10.3389/fendo.2024.1347695)
Supplement: Supplementary file 1 [file Image_1.pdf]

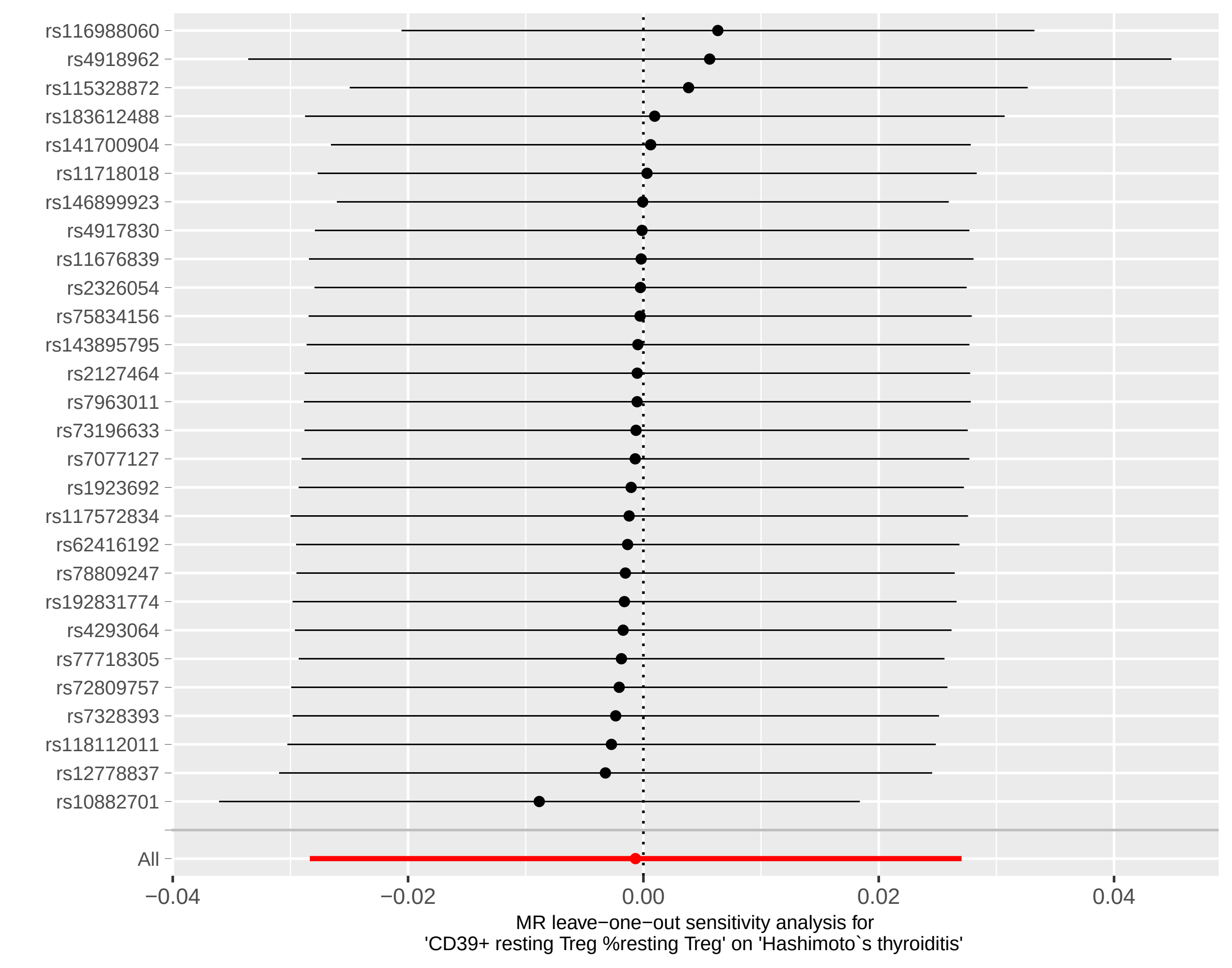
rs116988060
rs4918962
rs115328872
rs183612488
rs141700904
rs11718018
rs146899923
rs4917830
rs11676839
rs2326054
rs75834156
rs143895795
rs2127464
rs7963011
rs73196633
rs7077127
rs1923692
rs117572834
rs62416192
rs78809247
rs192831774
rs4293064
rs77718305
rs72809757
rs7328393
rs118112011
rs12778837
rs10882701
All
−0.04
−0.02
0.00
0.02
0.04
MR leave−one−out sensitivity analysis for
'CD39+ resting Treg %resting Treg' on 'Hashimoto`s thyroiditis'

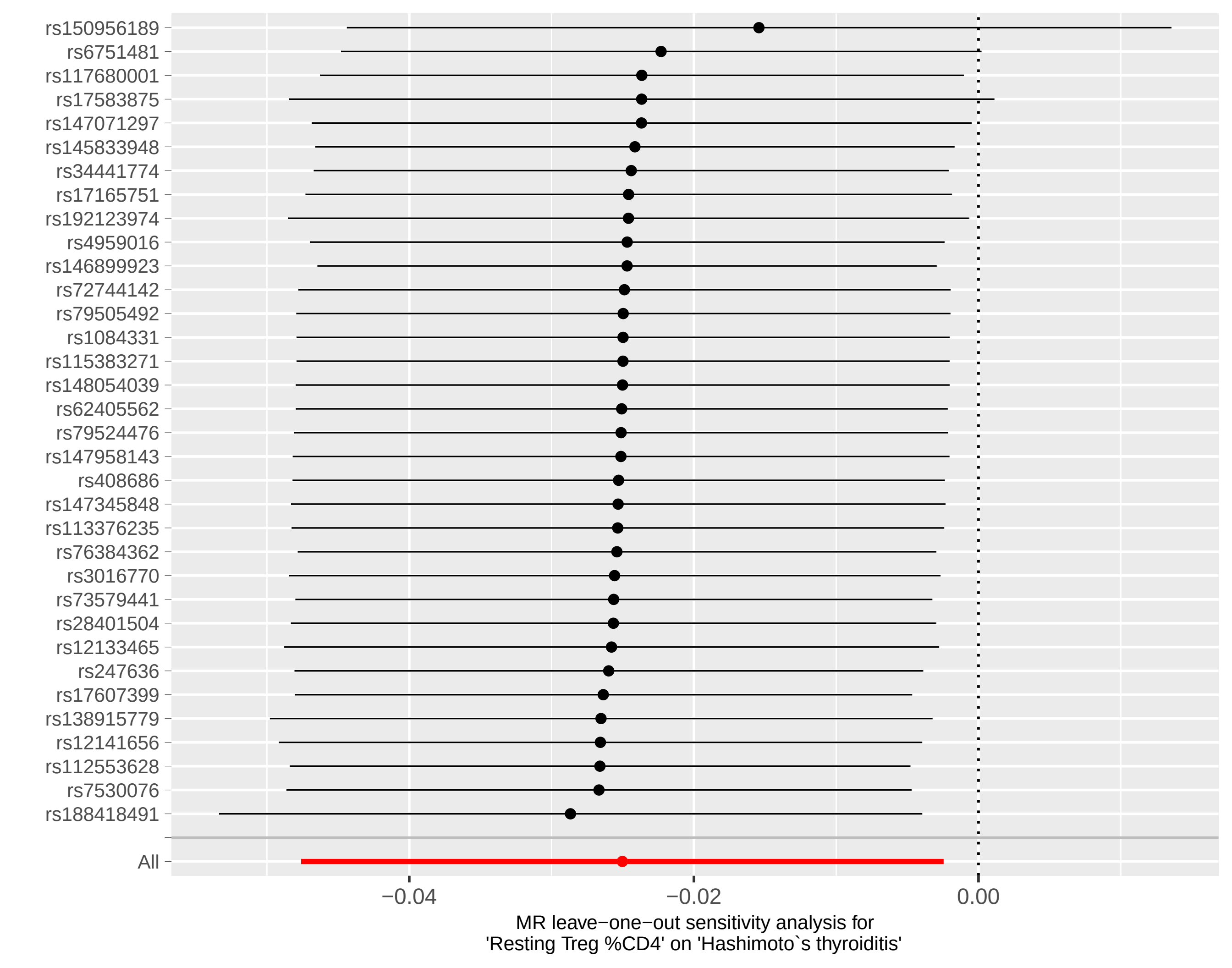
rs150956189
rs6751481
rs117680001
rs17583875
rs147071297
rs145833948
rs34441774
rs17165751
rs192123974
rs4959016
rs146899923
rs72744142
rs79505492
rs1084331
rs115383271
rs148054039
rs62405562
rs79524476
rs147958143
rs408686
rs147345848
rs113376235
rs76384362
rs3016770
rs73579441
rs28401504
rs12133465
rs247636
rs17607399
rs138915779
rs12141656
rs112553628
rs7530076
rs188418491
All
−0.04
−0.02
0.00
MR leave−one−out sensitivity analysis for
'Resting Treg %CD4' on 'Hashimoto`s thyroiditis'

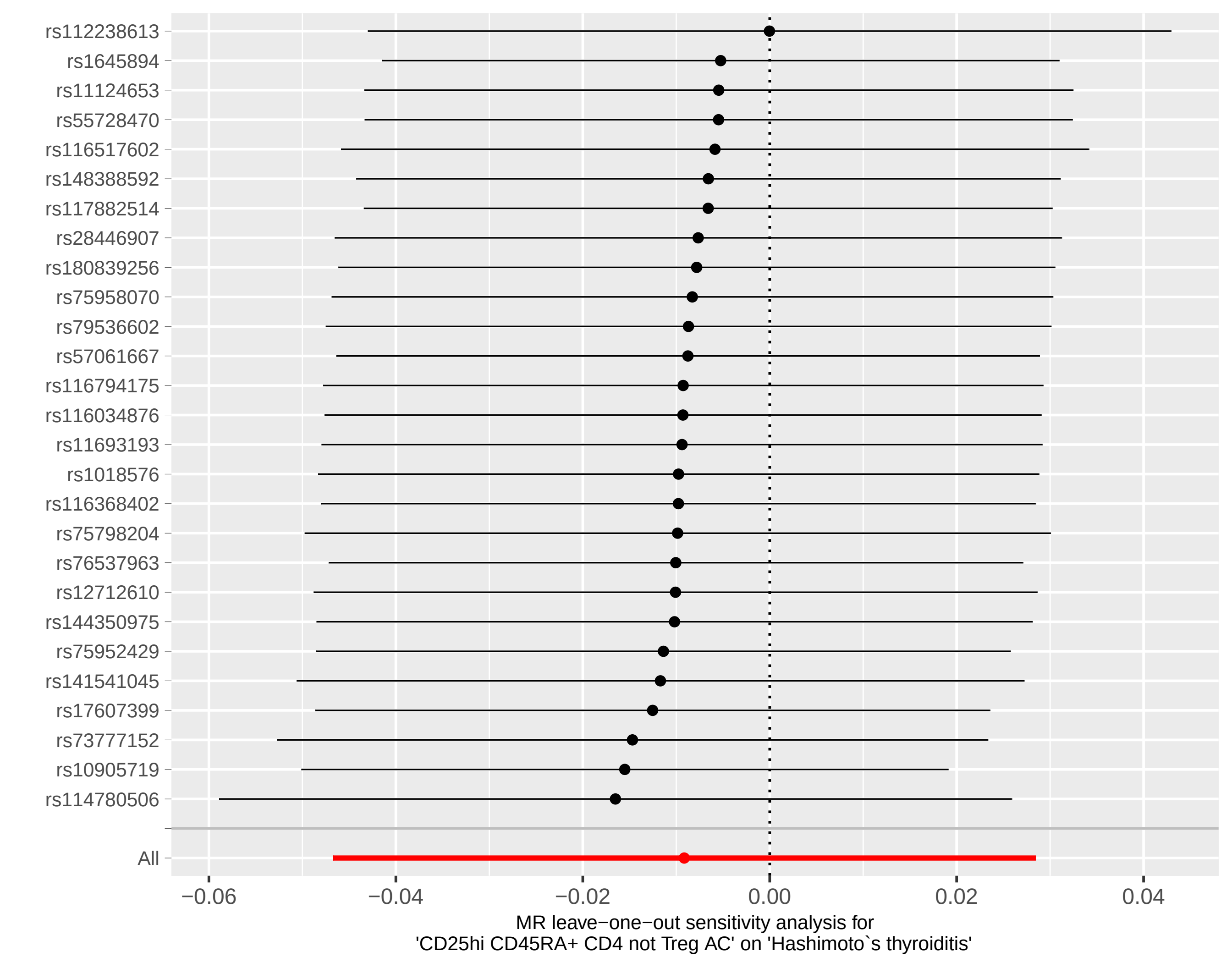
rs112238613
rs1645894
rs11124653
rs55728470
rs116517602
rs148388592
rs117882514
rs28446907
rs180839256
rs75958070
rs79536602
rs57061667
rs116794175
rs116034876
rs11693193
rs1018576
rs116368402
rs75798204
rs76537963
rs12712610
rs144350975
rs75952429
rs141541045
rs17607399
rs73777152
rs10905719
rs114780506
All
−0.06
−0.04
−0.02
0.00
0.02
0.04
MR leave−one−out sensitivity analysis for
'CD25hi CD45RA+ CD4 not Treg AC' on 'Hashimoto`s thyroiditis'

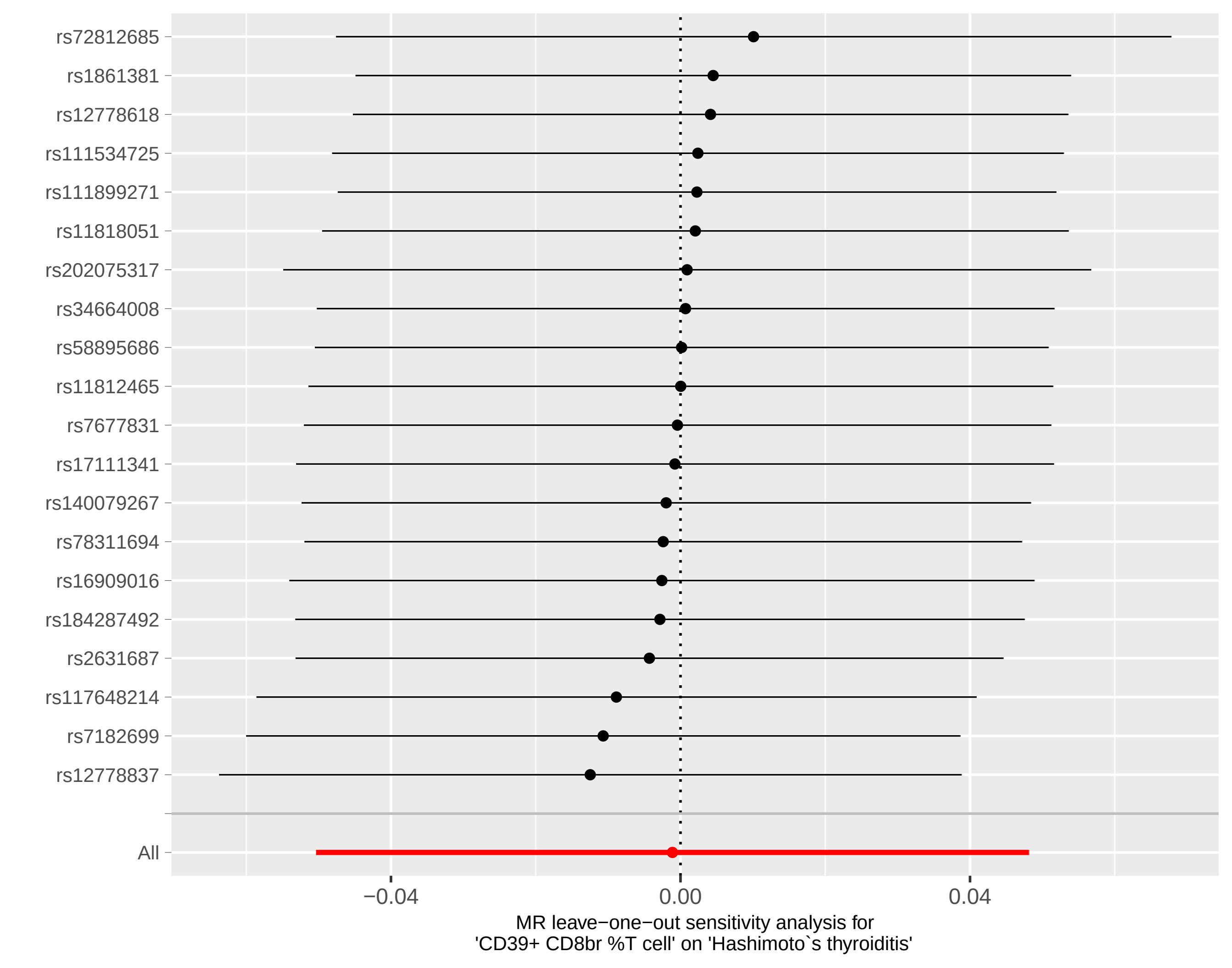
rs72812685
rs1861381
rs12778618
rs111534725
rs111899271
rs11818051
rs202075317
rs34664008
rs58895686
rs11812465
rs7677831
rs17111341
rs140079267
rs78311694
rs16909016
rs184287492
rs2631687
rs117648214
rs7182699
rs12778837
All
−0.04
0.00
0.04
MR leave−one−out sensitivity analysis for
'CD39+ CD8br %T cell' on 'Hashimoto`s thyroiditis'

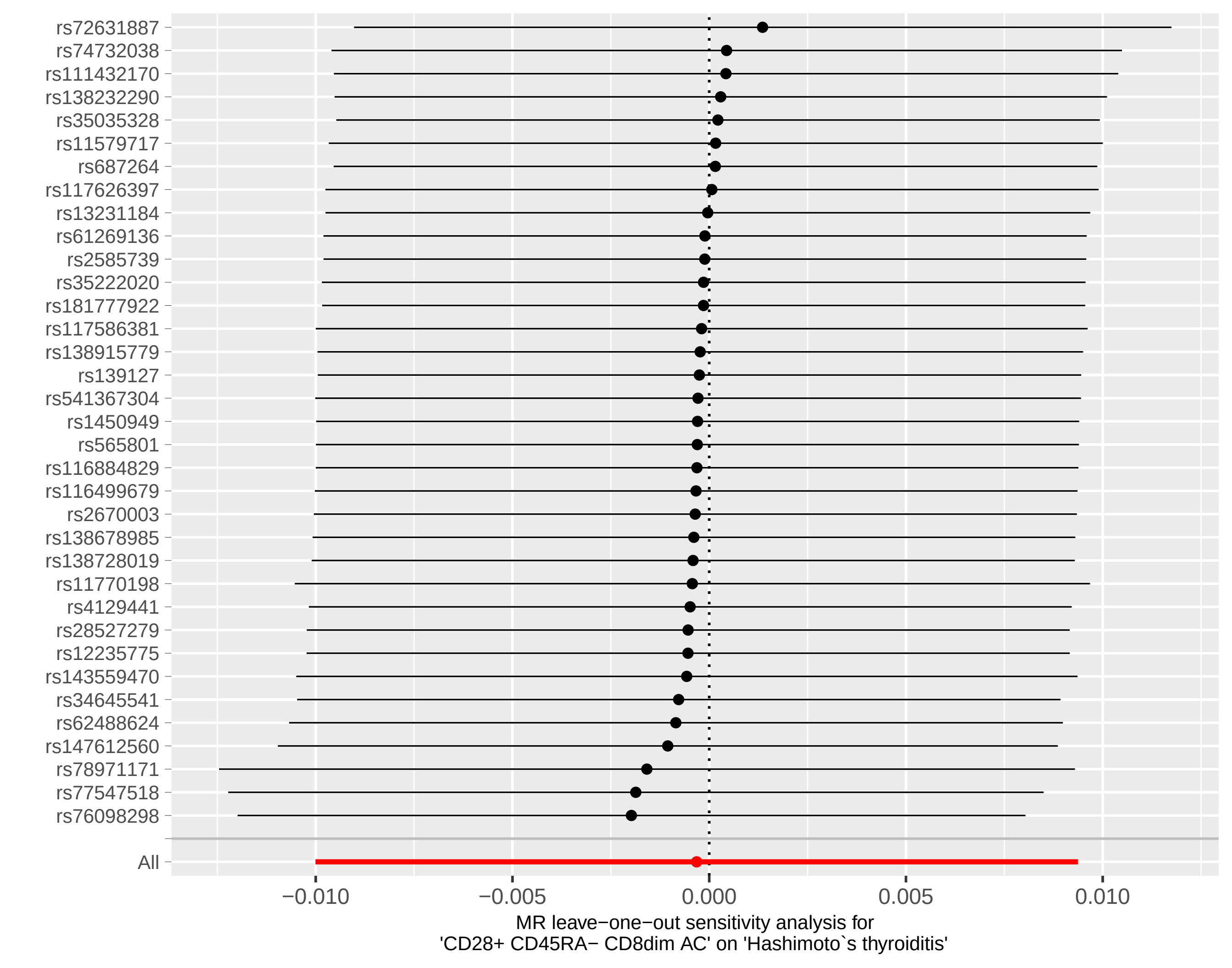
rs72631887
rs74732038
rs111432170
rs138232290
rs35035328
rs11579717
rs687264
rs117626397
rs13231184
rs61269136
rs2585739
rs35222020
rs181777922
rs117586381
rs138915779
rs139127
rs541367304
rs1450949
rs565801
rs116884829
rs116499679
rs2670003
rs138678985
rs138728019
rs11770198
rs4129441
rs28527279
rs12235775
rs143559470
rs34645541
rs62488624
rs147612560
rs78971171
rs77547518
rs76098298
All
−0.010
−0.005
0.000
0.005
0.010
MR leave−one−out sensitivity analysis for
'CD28+ CD45RA− CD8dim AC' on 'Hashimoto`s thyroiditis'

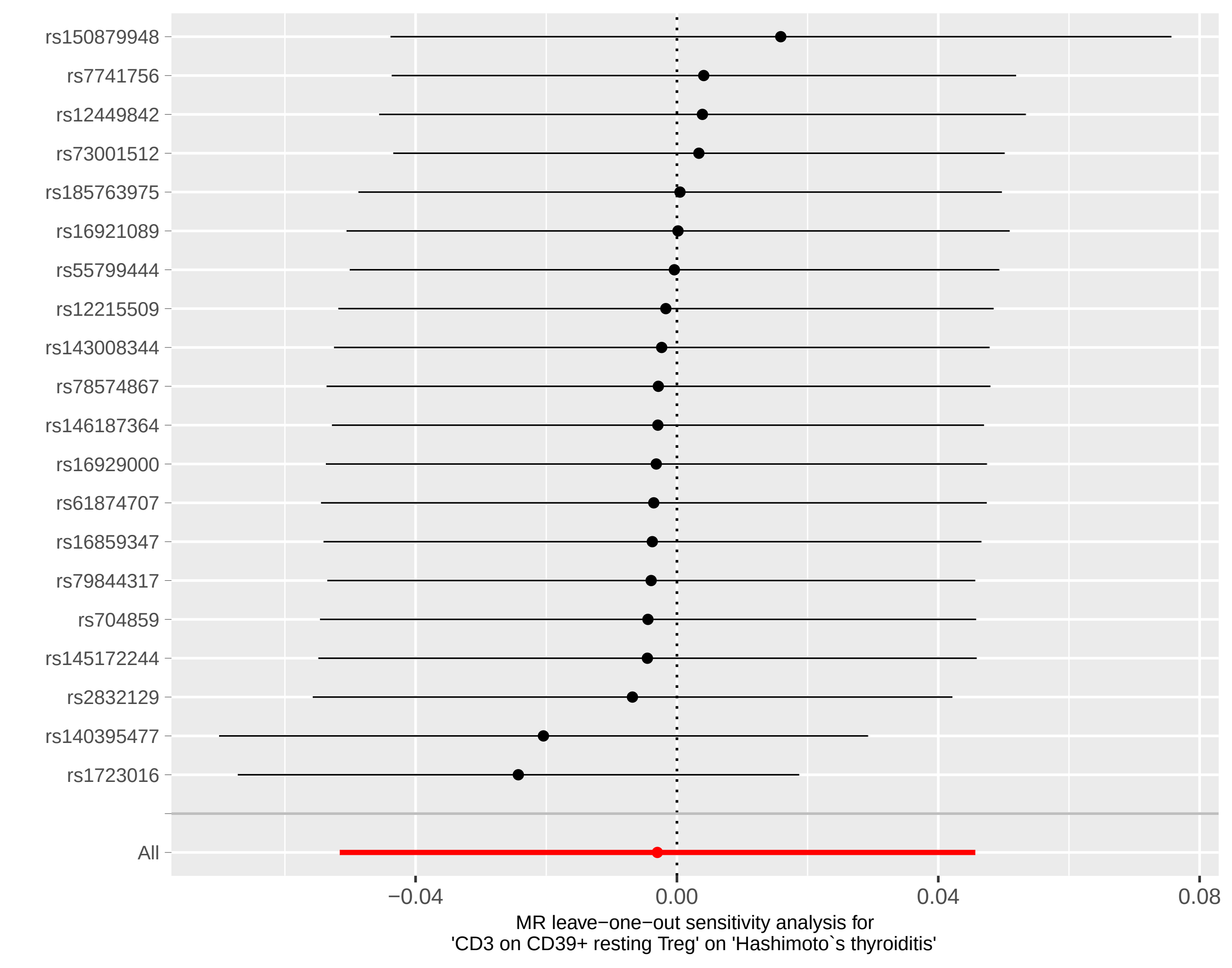
rs150879948
rs7741756
rs12449842
rs73001512
rs185763975
rs16921089
rs55799444
rs12215509
rs143008344
rs78574867
rs146187364
rs16929000
rs61874707
rs16859347
rs79844317
rs704859
rs145172244
rs2832129
rs140395477
rs1723016
All
−0.04
0.00
0.04
0.08
MR leave−one−out sensitivity analysis for
'CD3 on CD39+ resting Treg' on 'Hashimoto`s thyroiditis'

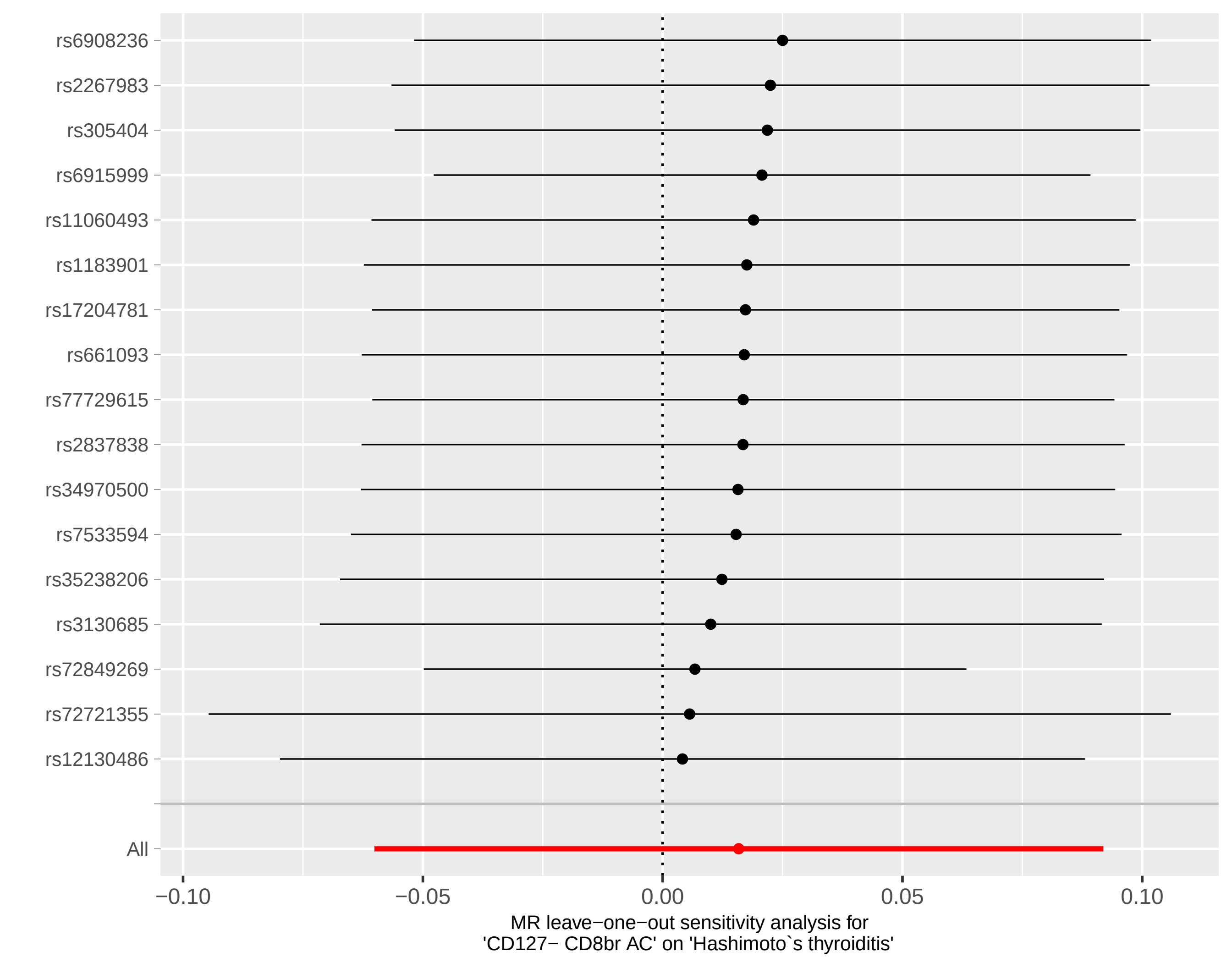

rs6908236
rs2267983
rs305404
rs6915999
rs11060493
rs1183901
rs17204781
rs661093
rs77729615
rs2837838
rs34970500
rs7533594
rs35238206
rs3130685
rs72849269
rs72721355
rs12130486
All
−0.10
−0.05
0.00
0.05
0.10
MR leave−one−out sensitivity analysis for
'CD127− CD8br AC' on 'Hashimoto`s thyroiditis'

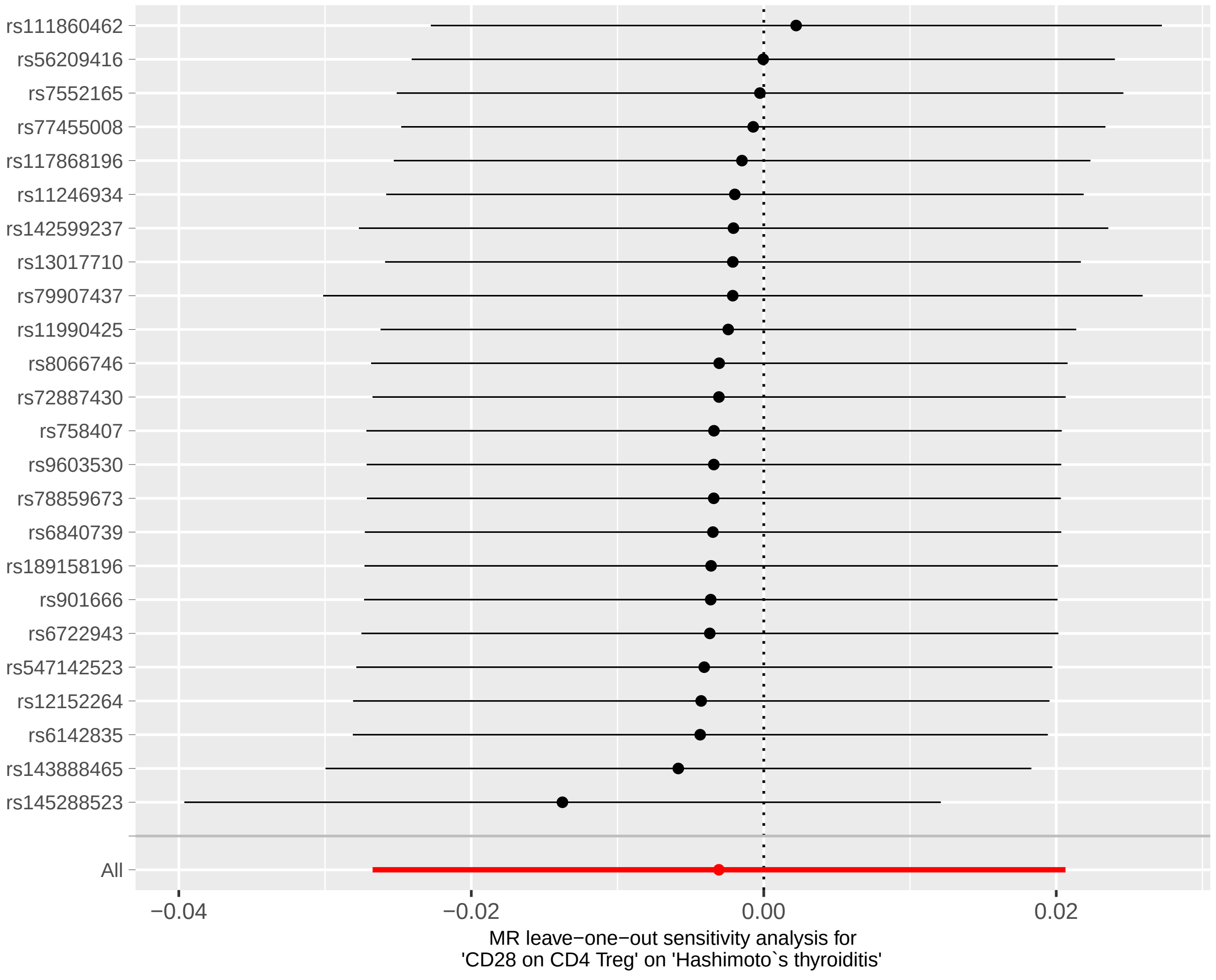

rs111860462
rs56209416
rs7552165
rs77455008
rs117868196
rs11246934
rs142599237
rs13017710
rs79907437
rs11990425
rs8066746
rs72887430
rs758407
rs9603530
rs78859673
rs6840739
rs189158196
rs901666
rs6722943
rs547142523
rs12152264
rs6142835
rs143888465
rs145288523
All
−0.04
−0.02
0.00
0.02
MR leave−one−out sensitivity analysis for
'CD28 on CD4 Treg' on 'Hashimoto`s thyroiditis'

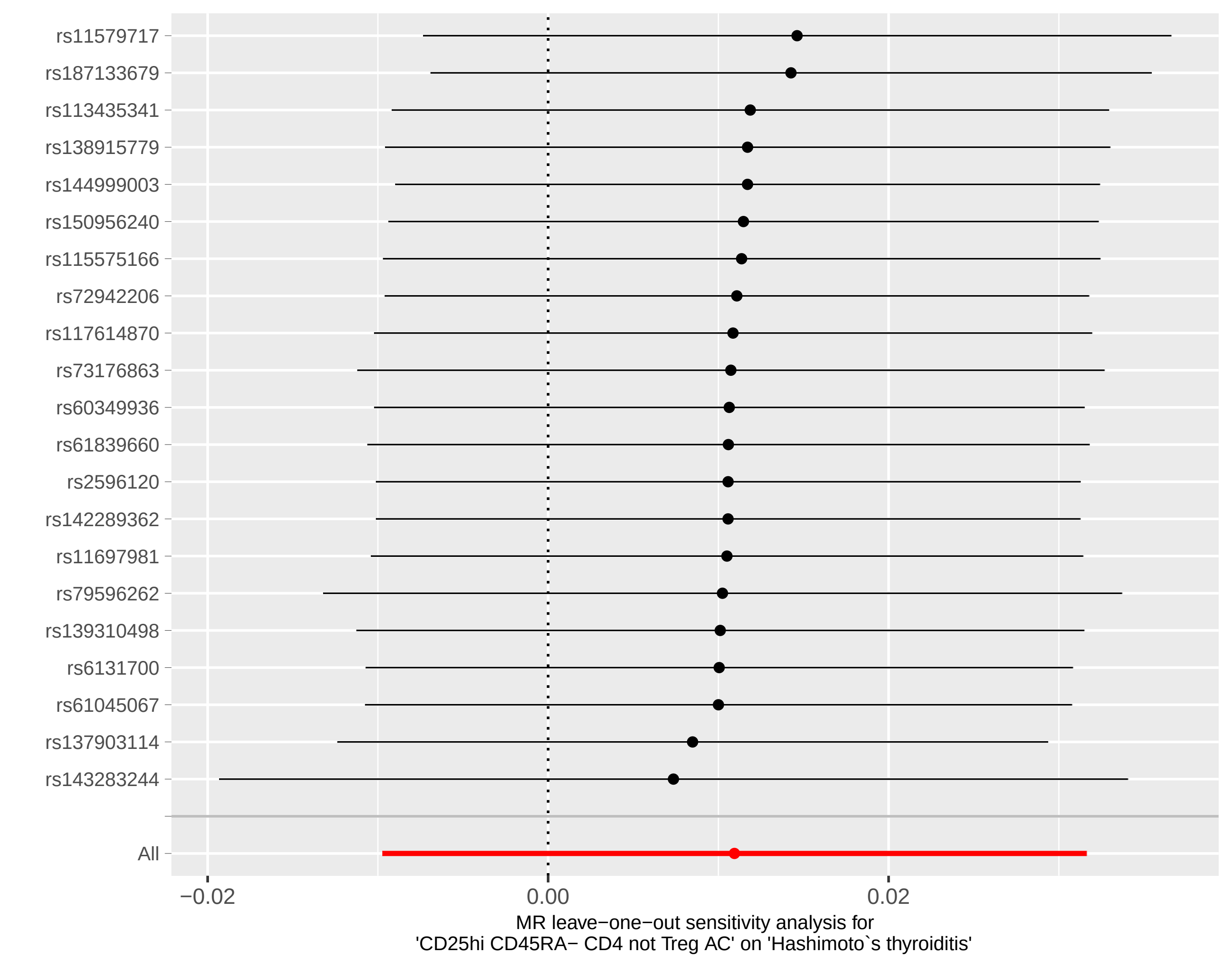

rs11579717
rs187133679
rs113435341
rs138915779
rs144999003
rs150956240
rs115575166
rs72942206
rs117614870
rs73176863
rs60349936
rs61839660
rs2596120
rs142289362
rs11697981
rs79596262
rs139310498
rs6131700
rs61045067
rs137903114
rs143283244
All
−0.02
0.00
0.02
MR leave−one−out sensitivity analysis for
'CD25hi CD45RA− CD4 not Treg AC' on 'Hashimoto`s thyroiditis'

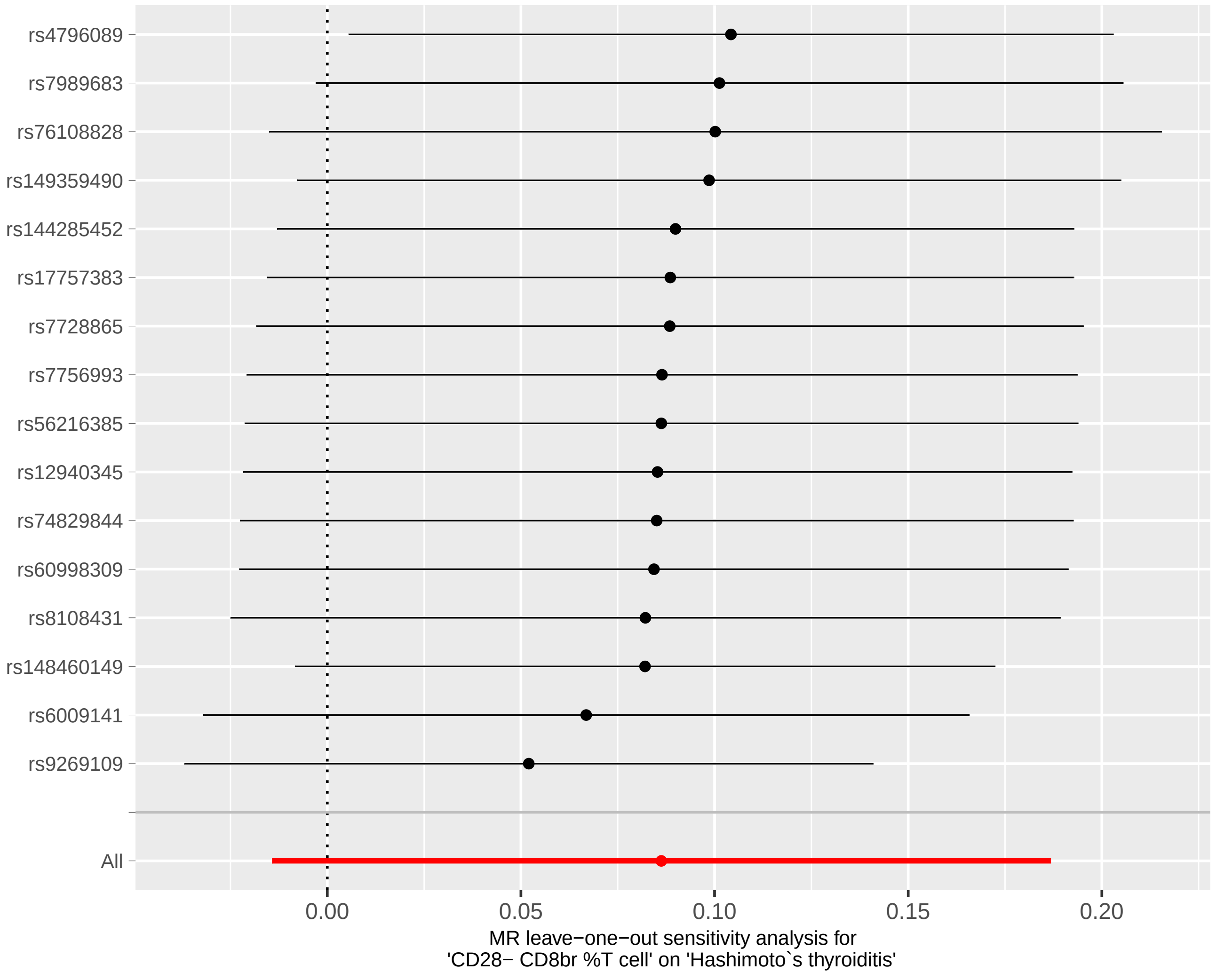

rs4796089
rs7989683
rs76108828
rs149359490
rs144285452
rs17757383
rs7728865
rs7756993
rs56216385
rs12940345
rs74829844
rs60998309
rs8108431
rs148460149
rs6009141
rs9269109
All
0.00
0.05
0.10
0.15
0.20
MR leave−one−out sensitivity analysis for
'CD28− CD8br %T cell' on 'Hashimoto`s thyroiditis'

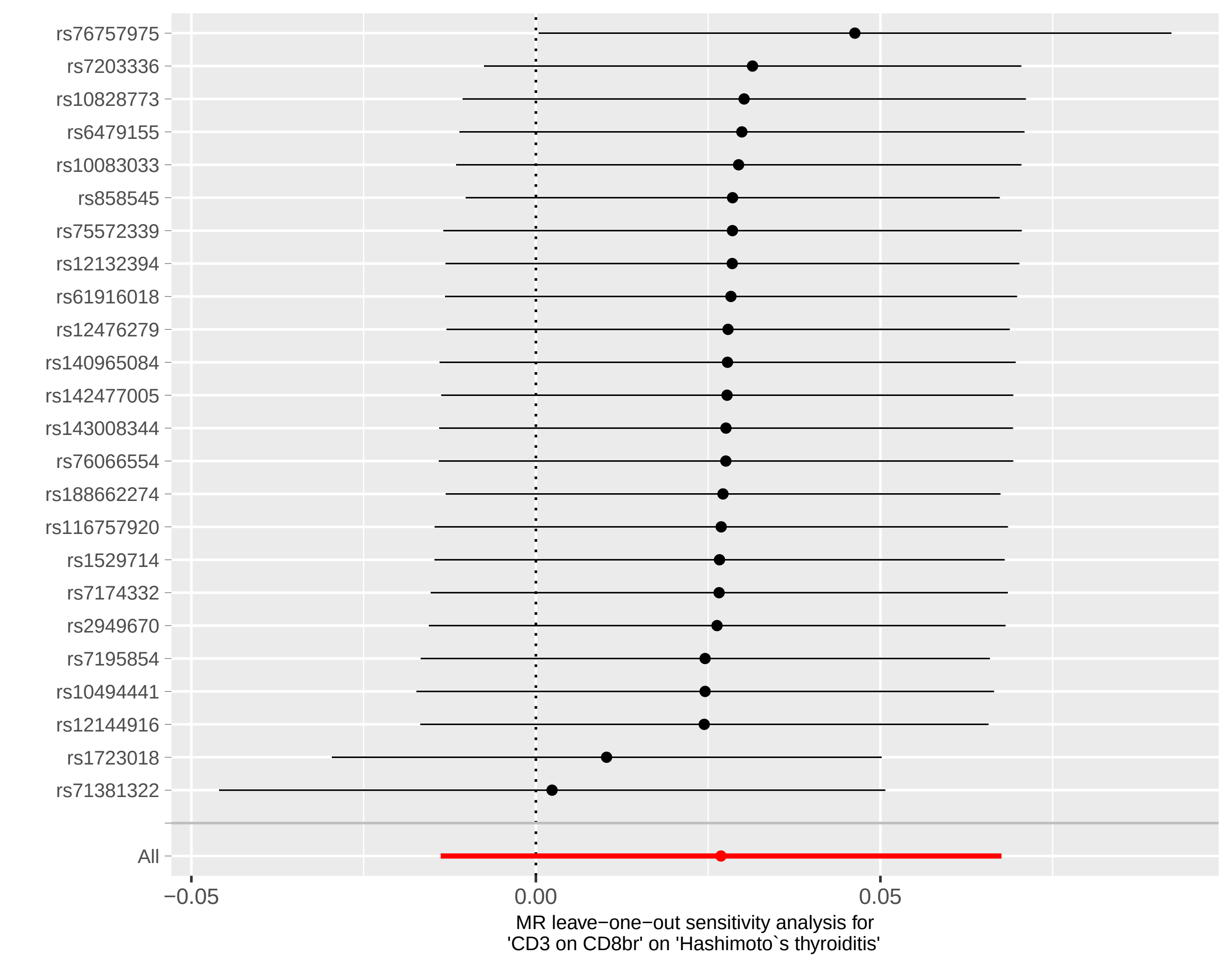
rs76757975
rs7203336
rs10828773
rs6479155
rs10083033
rs858545
rs75572339
rs12132394
rs61916018
rs12476279
rs140965084
rs142477005
rs143008344
rs76066554
rs188662274
rs116757920
rs1529714
rs7174332
rs2949670
rs7195854
rs10494441
rs12144916
rs1723018
rs71381322
All
−0.05
0.00
0.05
MR leave−one−out sensitivity analysis for
'CD3 on CD8br' on 'Hashimoto`s thyroiditis'

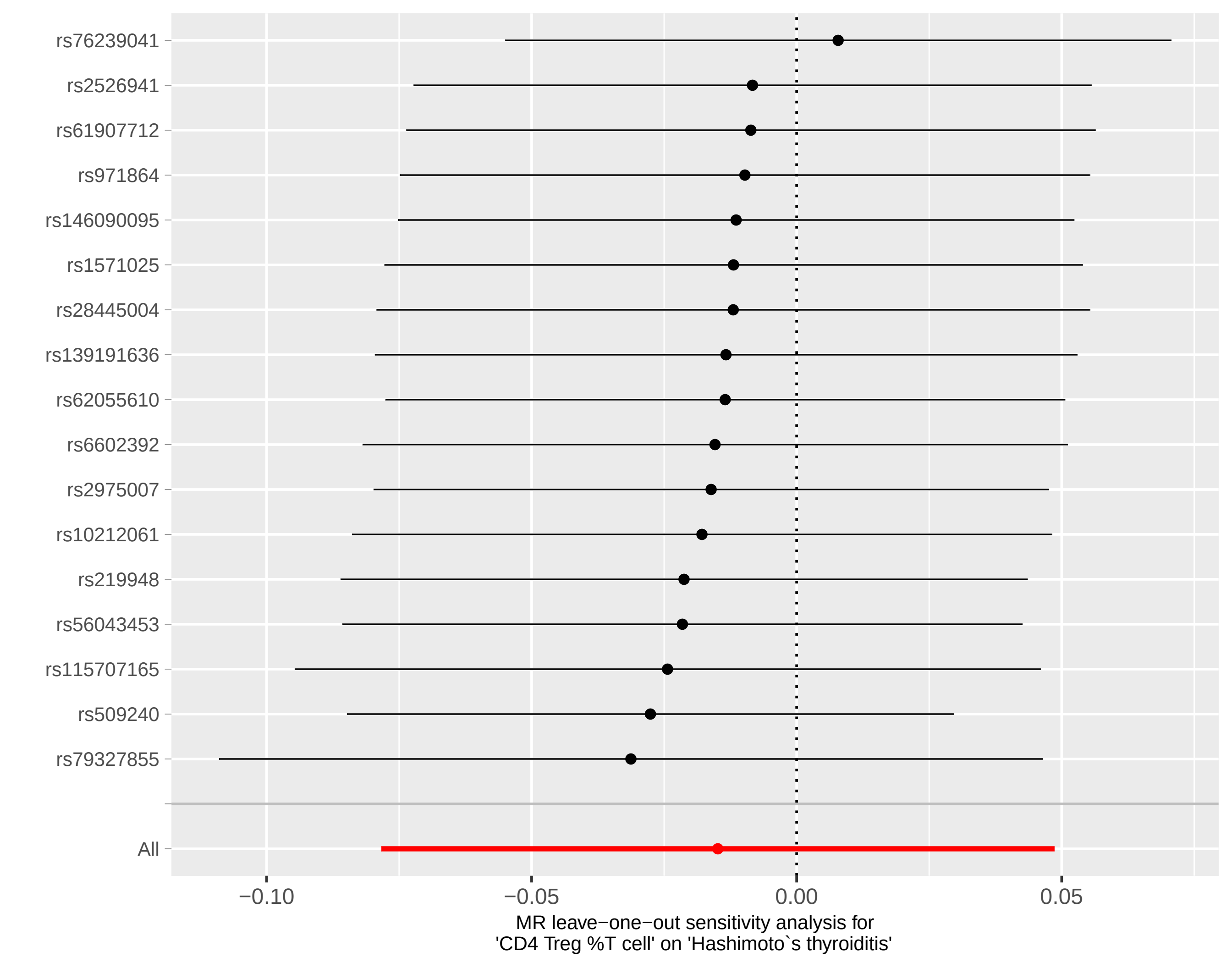
rs76239041
rs2526941
rs61907712
rs971864
rs146090095
rs1571025
rs28445004
rs139191636
rs62055610
rs6602392
rs2975007
rs10212061
rs219948
rs56043453
rs115707165
rs509240
rs79327855
All
−0.10
−0.05
0.00
0.05
MR leave−one−out sensitivity analysis for
'CD4 Treg %T cell' on 'Hashimoto`s thyroiditis'

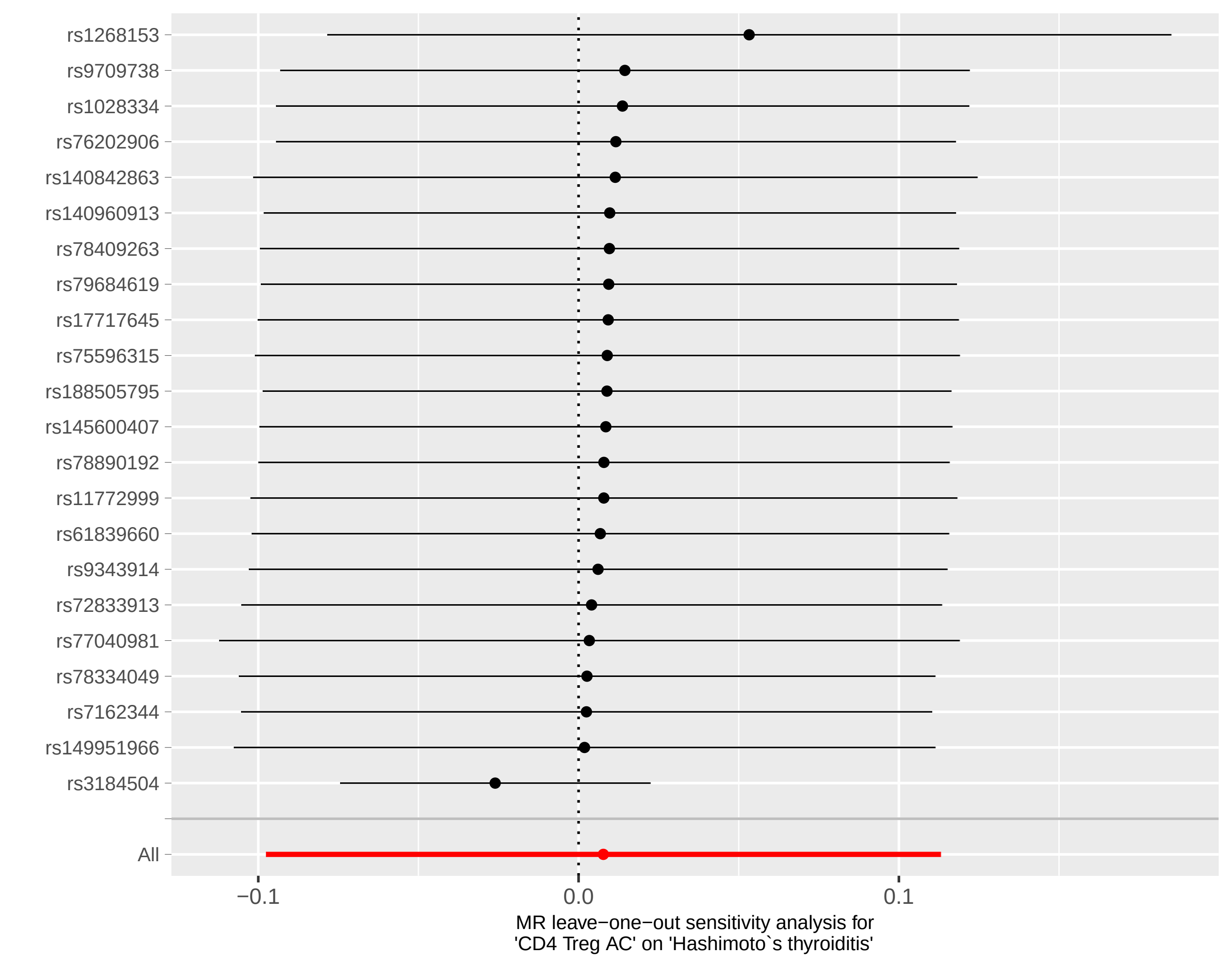
rs1268153
rs9709738
rs1028334
rs76202906
rs140842863
rs140960913
rs78409263
rs79684619
rs17717645
rs75596315
rs188505795
rs145600407
rs78890192
rs11772999
rs61839660
rs9343914
rs72833913
rs77040981
rs78334049
rs7162344
rs149951966
rs3184504
All
−0.1
0.0
0.1
MR leave−one−out sensitivity analysis for
'CD4 Treg AC' on 'Hashimoto`s thyroiditis'

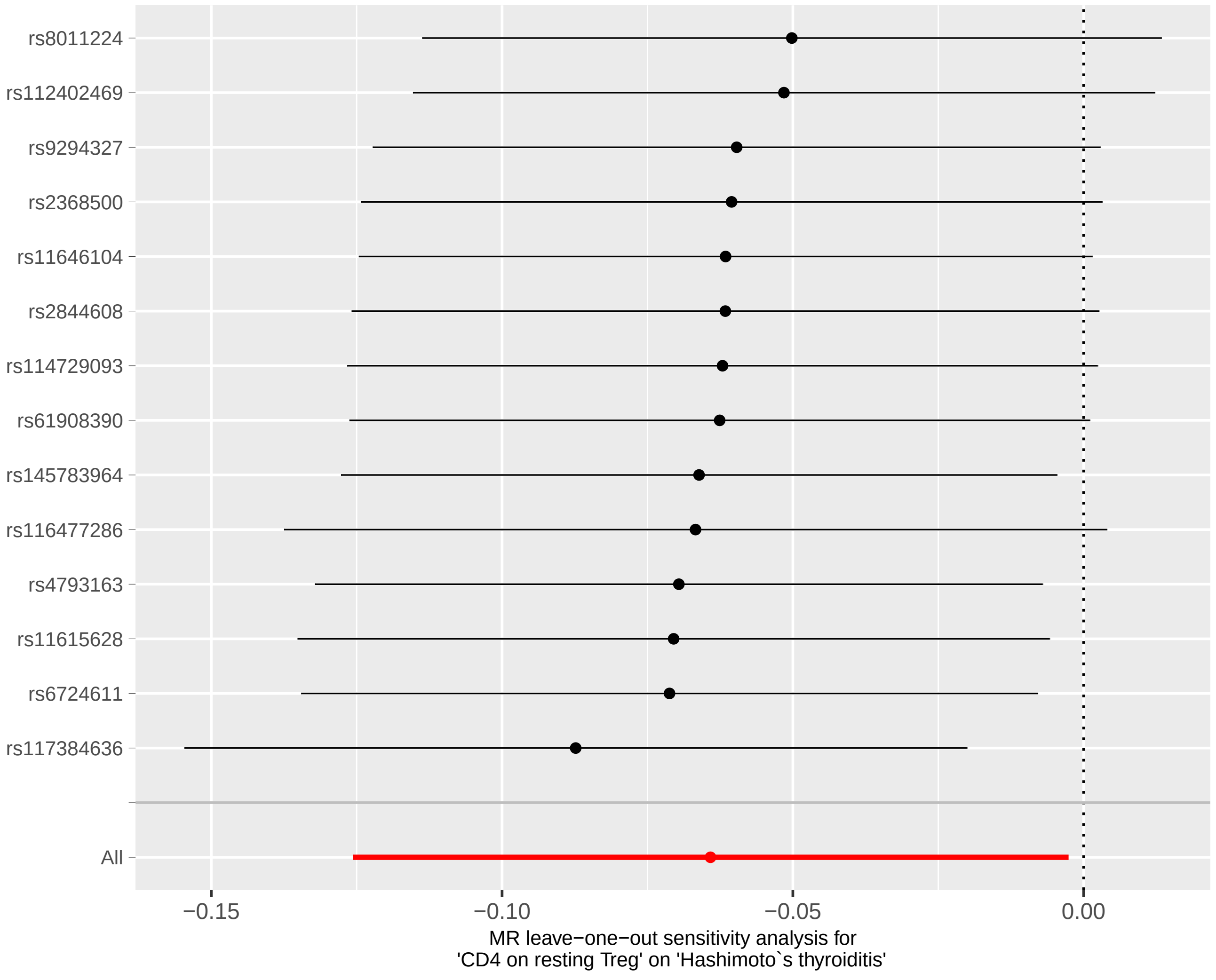
rs8011224
rs112402469
rs9294327
rs2368500
rs11646104
rs2844608
rs114729093
rs61908390
rs145783964
rs116477286
rs4793163
rs11615628
rs6724611
rs117384636
All
−0.15
−0.10
−0.05
0.00
MR leave−one−out sensitivity analysis for
'CD4 on resting Treg' on 'Hashimoto`s thyroiditis'

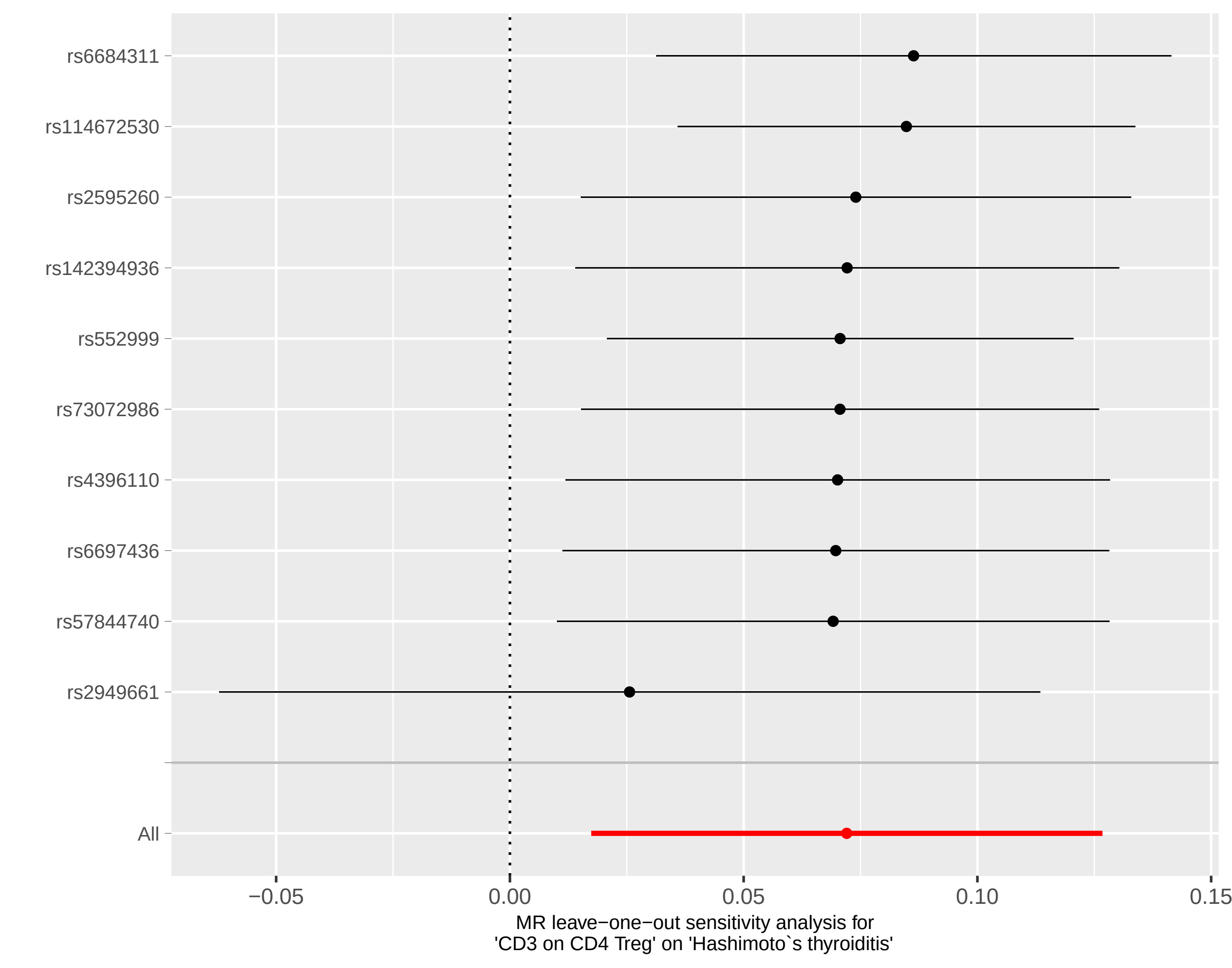
rs6684311
rs114672530
rs2595260
rs142394936
rs552999
rs73072986
rs4396110
rs6697436
rs57844740
rs2949661
All
−0.05
0.00
0.05
0.10
0.15
MR leave−one−out sensitivity analysis for
'CD3 on CD4 Treg' on 'Hashimoto`s thyroiditis'

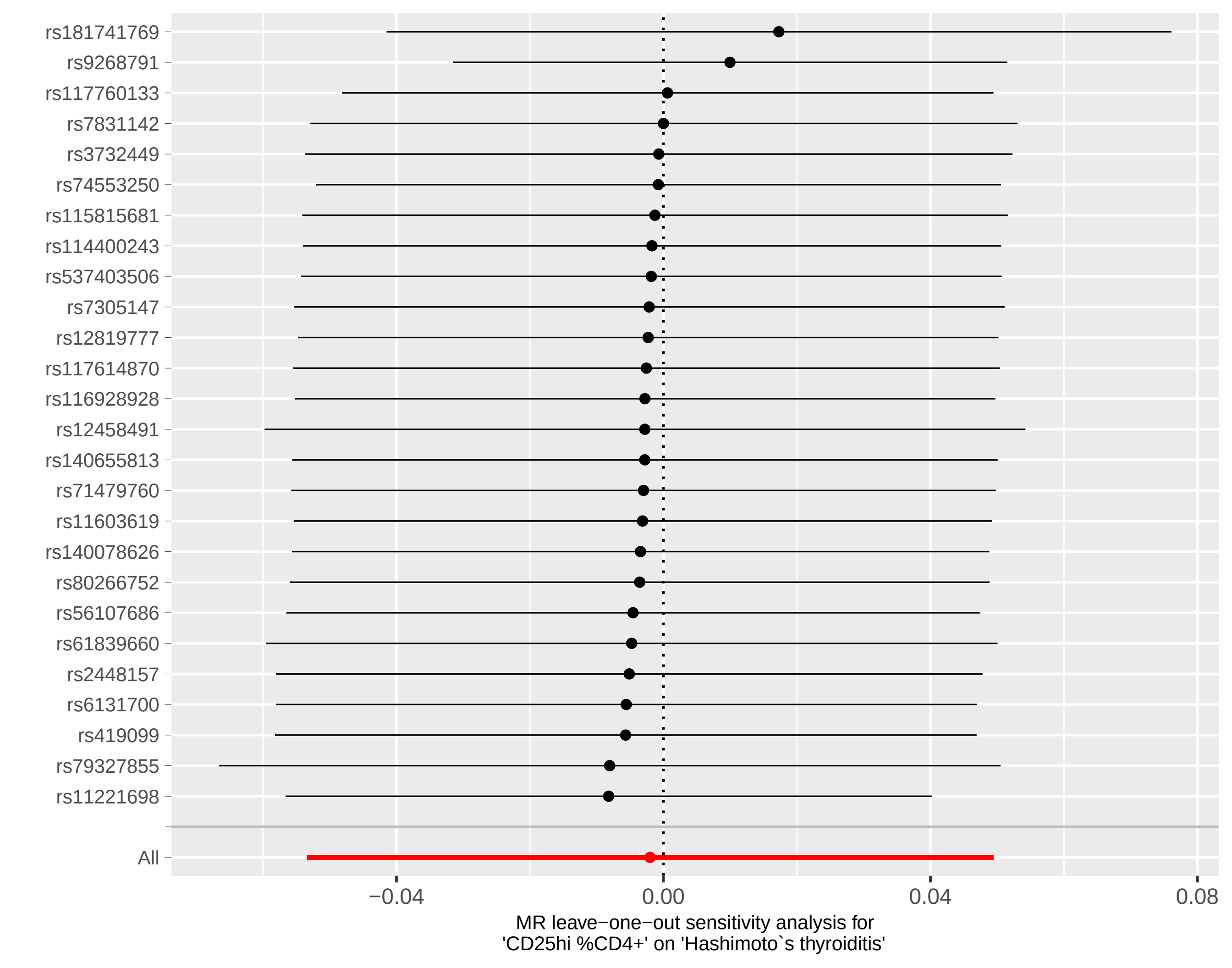
rs181741769
rs9268791
rs117760133
rs7831142
rs3732449
rs74553250
rs115815681
rs114400243
rs537403506
rs7305147
rs12819777
rs117614870
rs116928928
rs12458491
rs140655813
rs71479760
rs11603619
rs140078626
rs80266752
rs56107686
rs61839660
rs2448157
rs6131700
rs419099
rs79327855
rs11221698
All
−0.04
0.00
0.04
0.08
MR leave−one−out sensitivity analysis for
'CD25hi %CD4+' on 'Hashimoto`s thyroiditis'

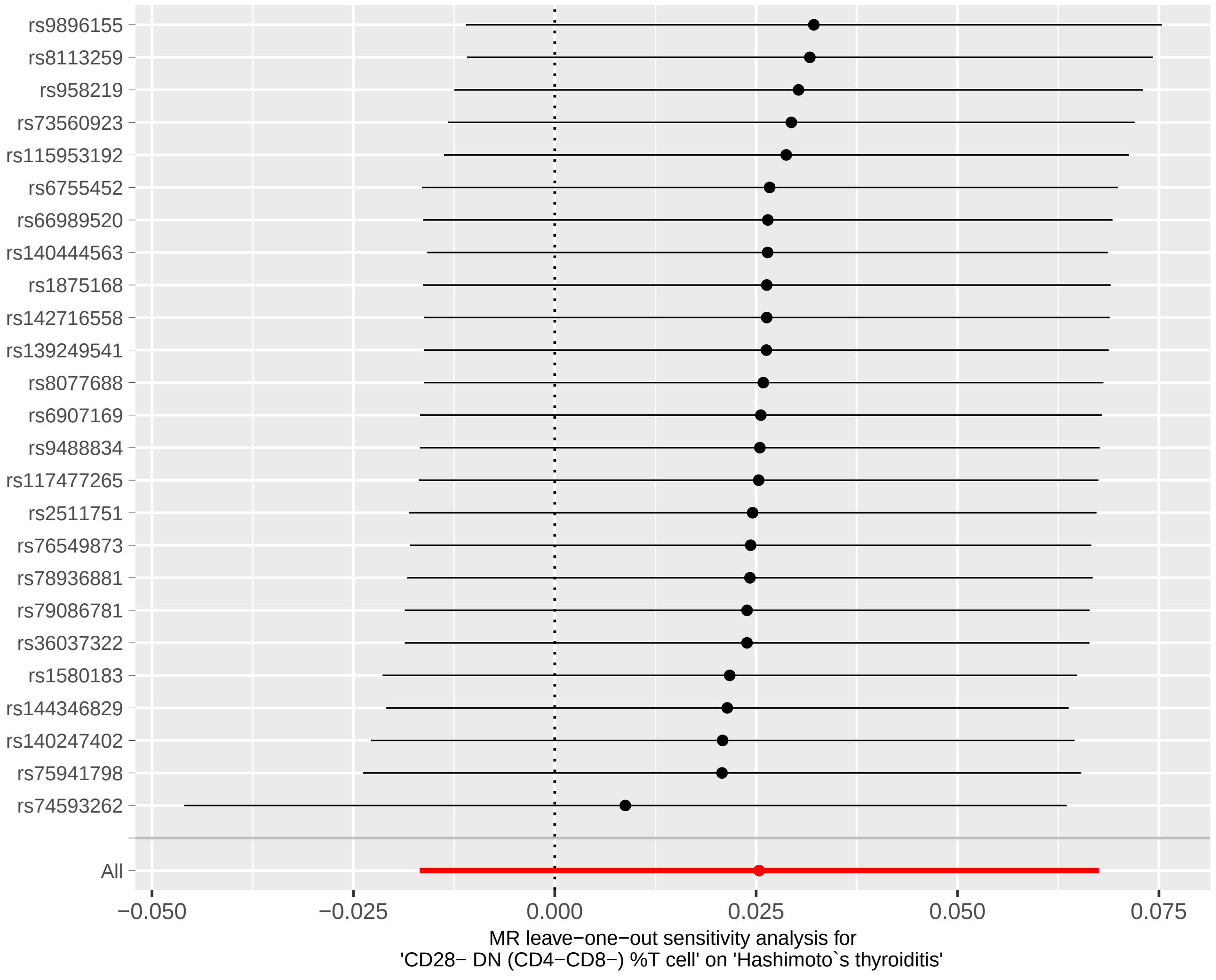

rs9896155
rs8113259
rs958219
rs73560923
rs115953192
rs6755452
rs66989520
rs140444563
rs1875168
rs142716558
rs139249541
rs8077688
rs6907169
rs9488834
rs117477265
rs2511751
rs76549873
rs78936881
rs79086781
rs36037322
rs1580183
rs144346829
rs140247402
rs75941798
rs74593262
All
−0.050
−0.025
0.000
0.025
0.050
0.075
MR leave−one−out sensitivity analysis for
'CD28− DN (CD4−CD8−) %T cell' on 'Hashimoto`s thyroiditis'

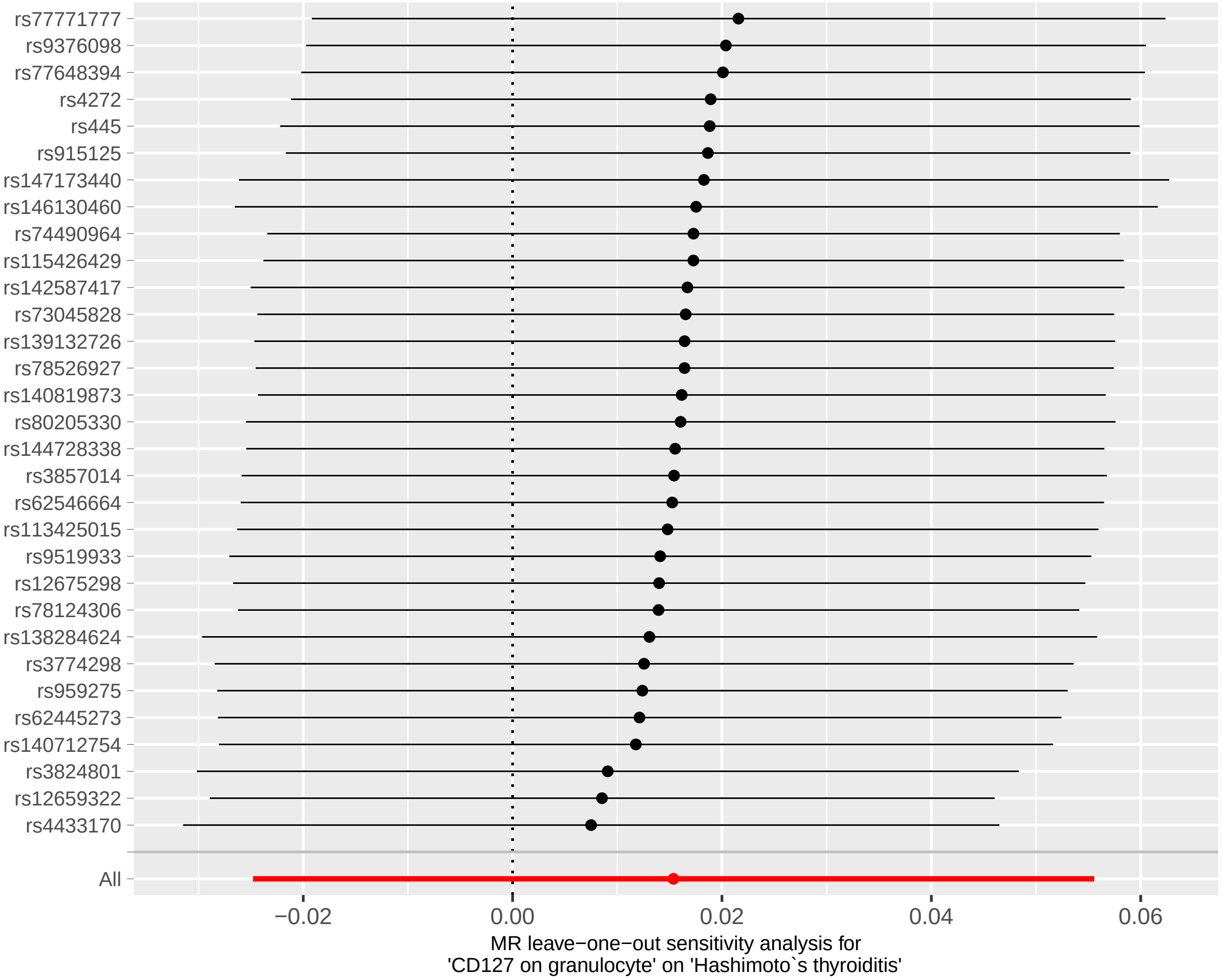

rs77771777
rs9376098
rs77648394
rs4272
rs445
rs915125
rs147173440
rs146130460
rs74490964
rs115426429
rs142587417
rs73045828
rs139132726
rs78526927
rs140819873
rs80205330
rs144728338
rs3857014
rs62546664
rs113425015
rs9519933
rs12675298
rs78124306
rs138284624
rs3774298
rs959275
rs62445273
rs140712754
rs3824801
rs12659322
rs4433170
All
−0.02
0.00
0.02
0.04
0.06
MR leave−one−out sensitivity analysis for
'CD127 on granulocyte' on 'Hashimoto`s thyroiditis'

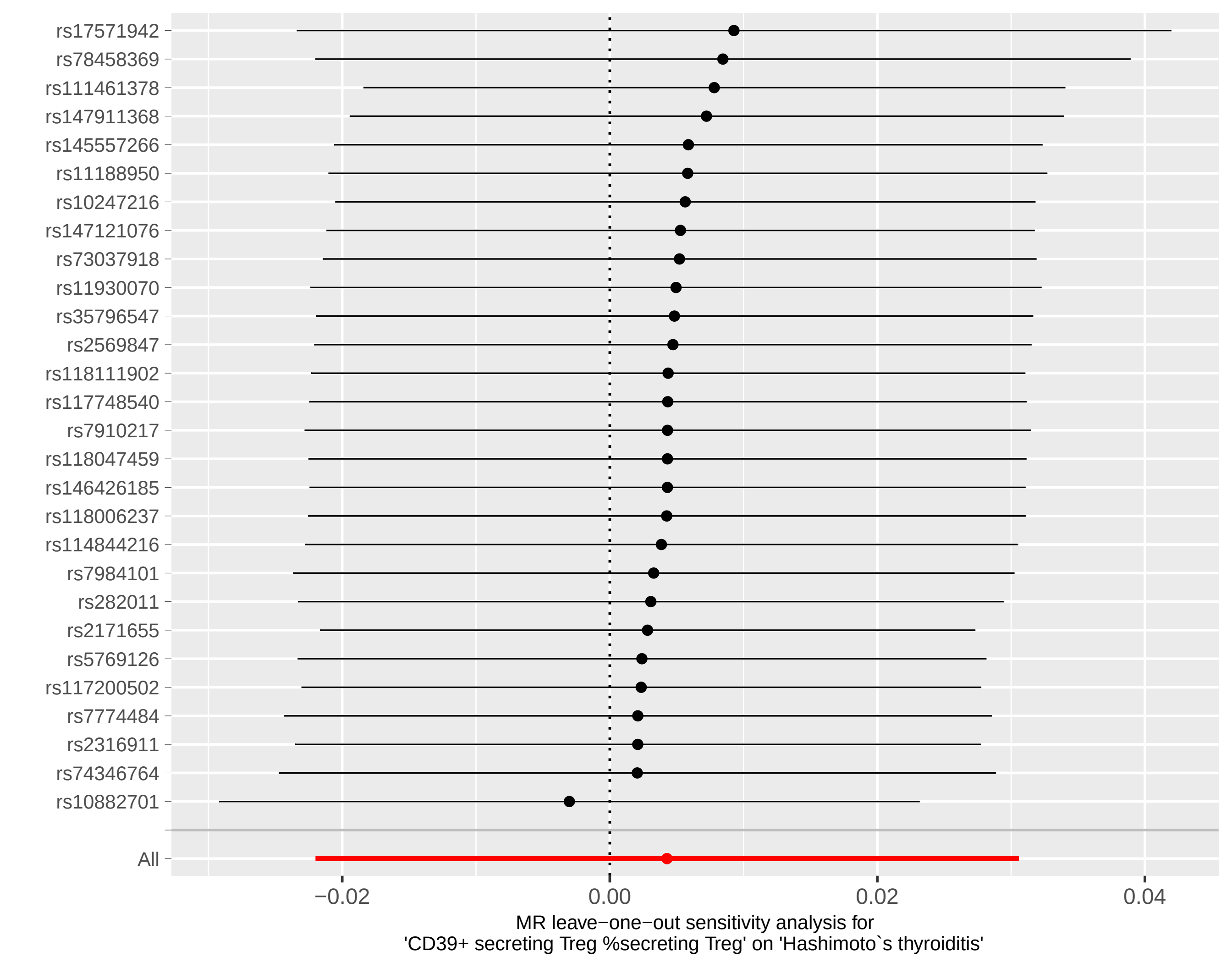
rs17571942
rs78458369
rs111461378
rs147911368
rs145557266
rs11188950
rs10247216
rs147121076
rs73037918
rs11930070
rs35796547
rs2569847
rs118111902
rs117748540
rs7910217
rs118047459
rs146426185
rs118006237
rs114844216
rs7984101
rs282011
rs2171655
rs5769126
rs117200502
rs7774484
rs2316911
rs74346764
rs10882701
All
−0.02
0.00
0.02
0.04
MR leave−one−out sensitivity analysis for
'CD39+ secreting Treg %secreting Treg' on 'Hashimoto`s thyroiditis'

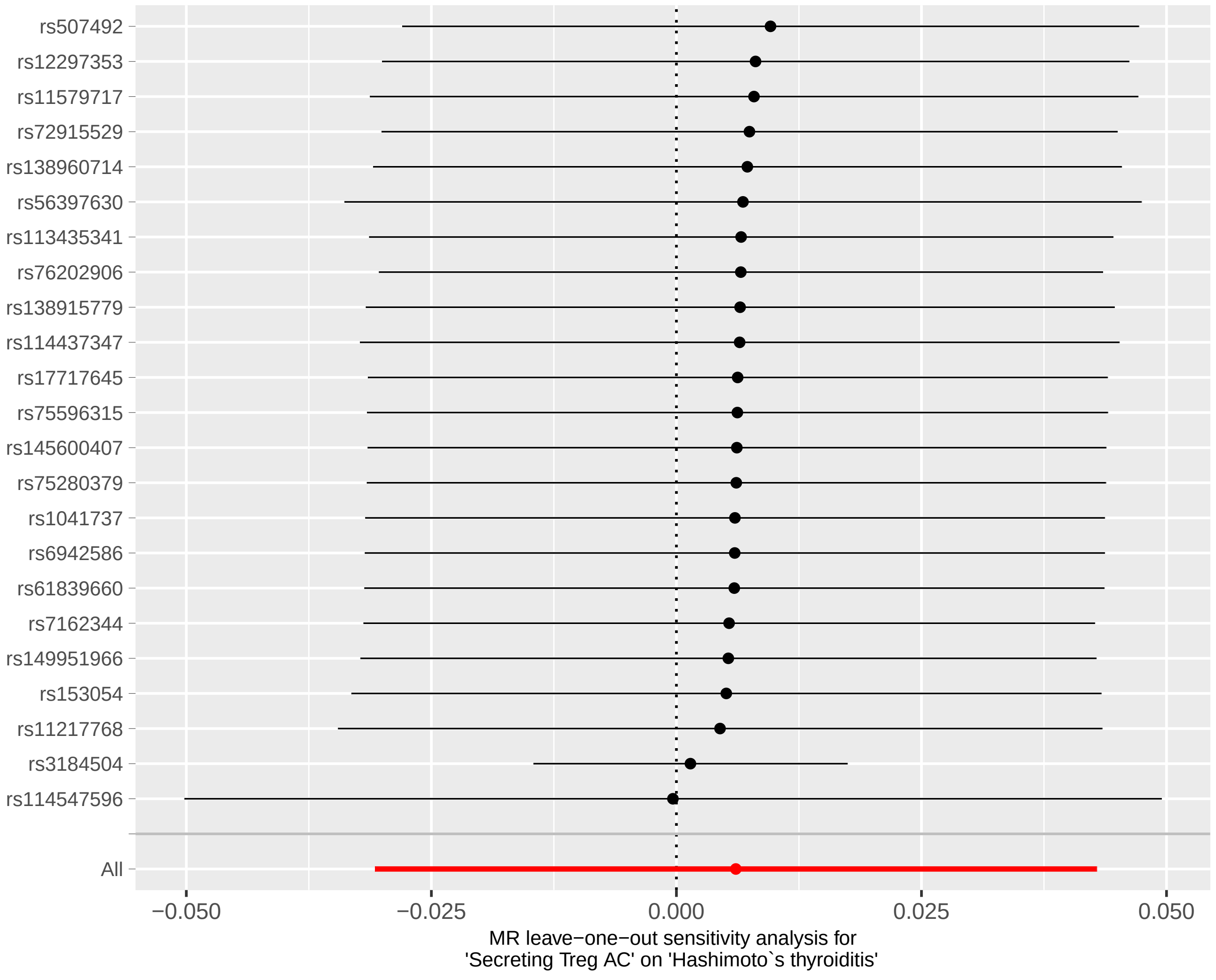
rs507492
rs12297353
rs11579717
rs72915529
rs138960714
rs56397630
rs113435341
rs76202906
rs138915779
rs114437347
rs17717645
rs75596315
rs145600407
rs75280379
rs1041737
rs6942586
rs61839660
rs7162344
rs149951966
rs153054
rs11217768
rs3184504
rs114547596
All
−0.050
−0.025
0.000
0.025
0.050
MR leave−one−out sensitivity analysis for
'Secreting Treg AC' on 'Hashimoto`s thyroiditis'

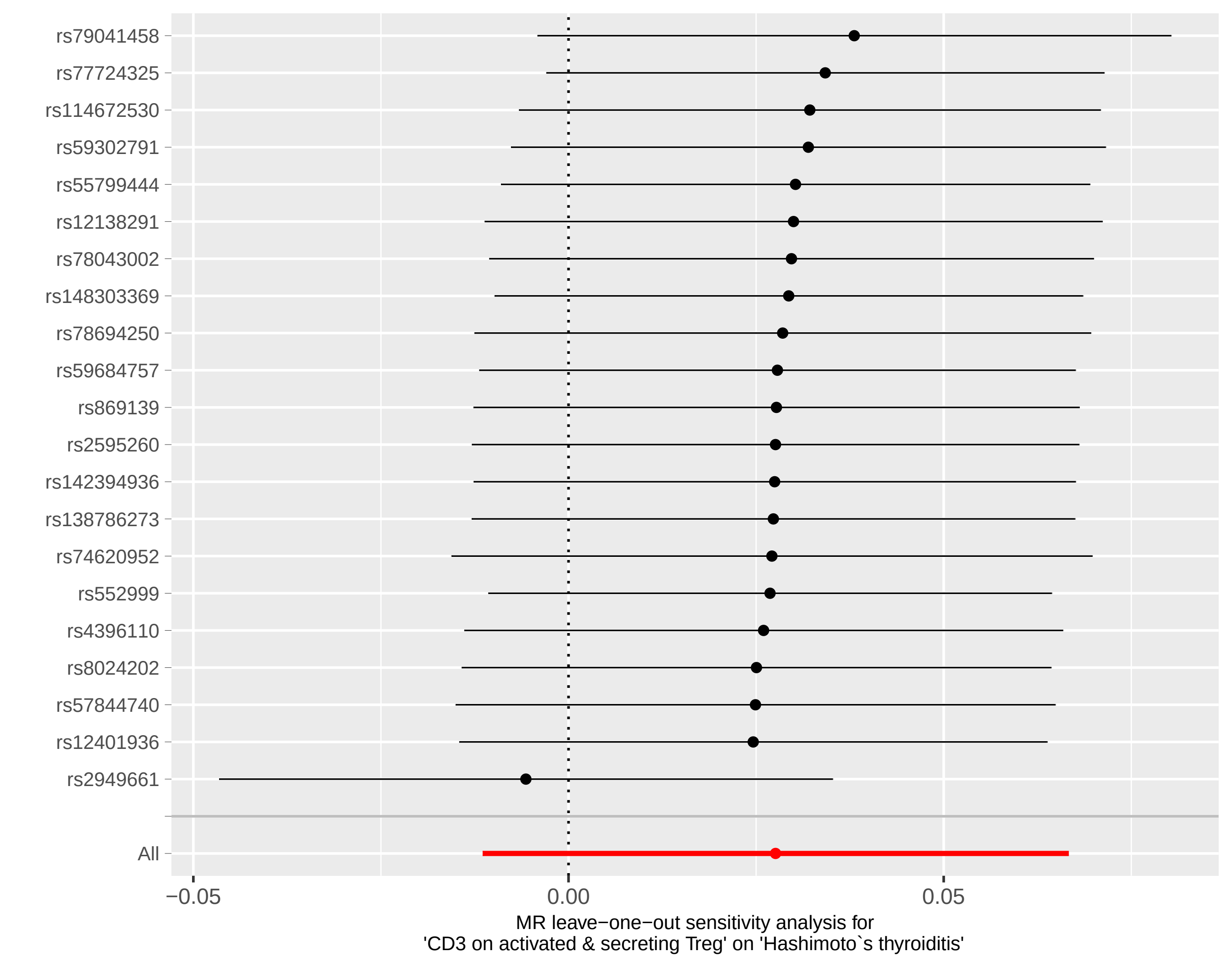

rs79041458
rs77724325
rs114672530
rs59302791
rs55799444
rs12138291
rs78043002
rs148303369
rs78694250
rs59684757
rs869139
rs2595260
rs142394936
rs138786273
rs74620952
rs552999
rs4396110
rs8024202
rs57844740
rs12401936
rs2949661
All
−0.05
0.00
0.05
MR leave−one−out sensitivity analysis for
'CD3 on activated & secreting Treg' on 'Hashimoto`s thyroiditis'

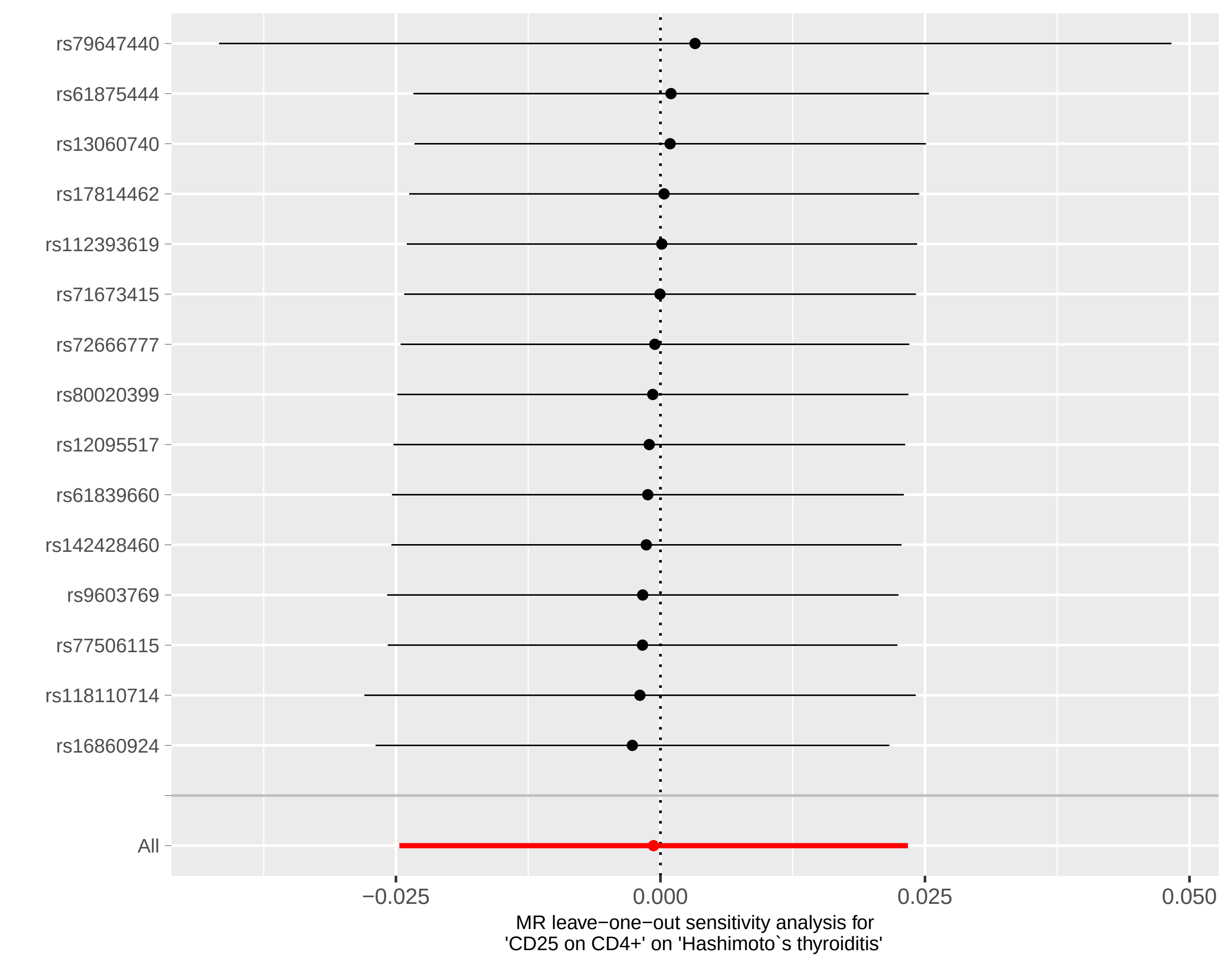
rs79647440
rs61875444
rs13060740
rs17814462
rs112393619
rs71673415
rs72666777
rs80020399
rs12095517
rs61839660
rs142428460
rs9603769
rs77506115
rs118110714
rs16860924
All
−0.025
0.000
0.025
0.050
MR leave−one−out sensitivity analysis for
'CD25 on CD4+' on 'Hashimoto`s thyroiditis'

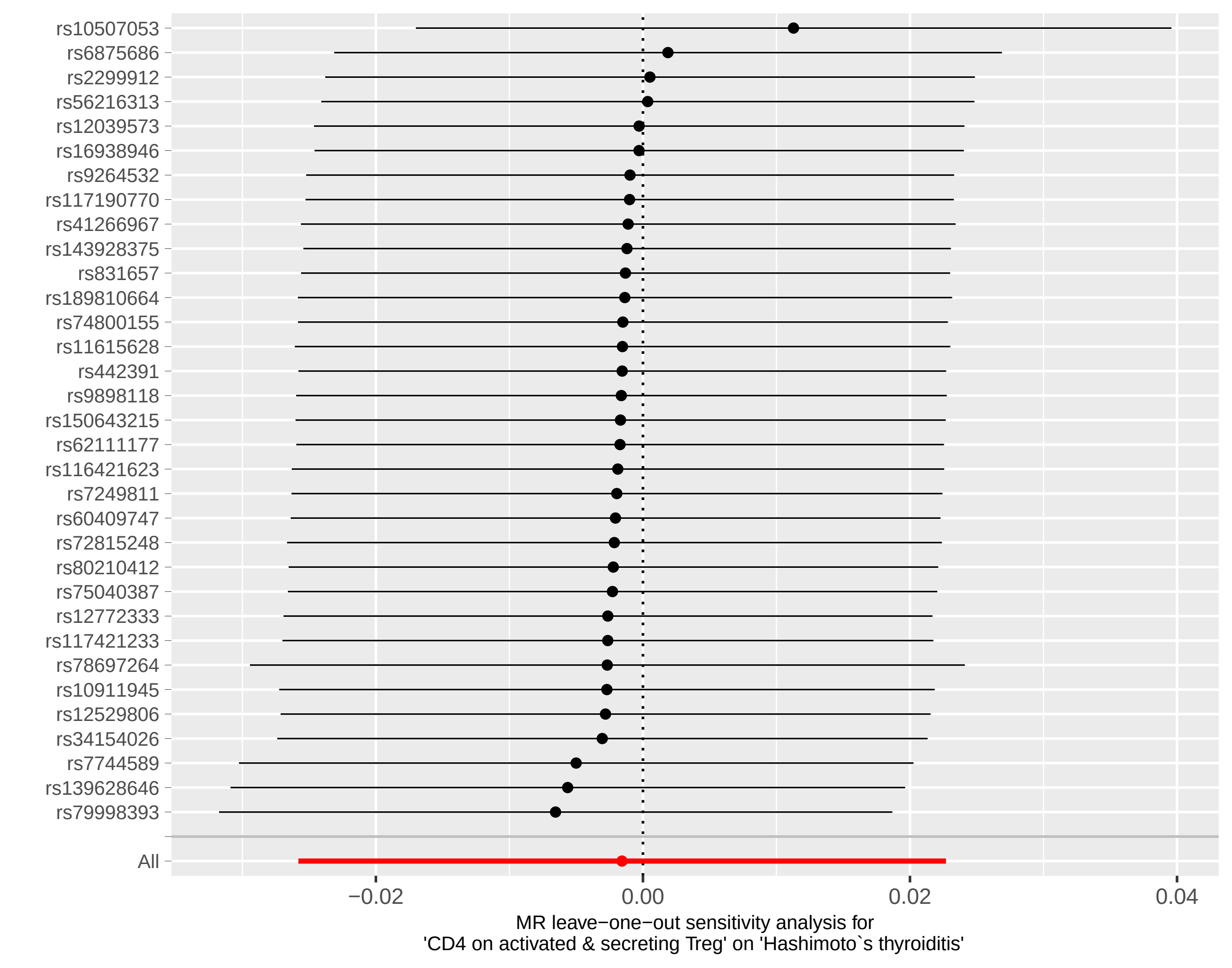

rs10507053
rs6875686
rs2299912
rs56216313
rs12039573
rs16938946
rs9264532
rs117190770
rs41266967
rs143928375
rs831657
rs189810664
rs74800155
rs11615628
rs442391
rs9898118
rs150643215
rs62111177
rs116421623
rs7249811
rs60409747
rs72815248
rs80210412
rs75040387
rs12772333
rs117421233
rs78697264
rs10911945
rs12529806
rs34154026
rs7744589
rs139628646
rs79998393
All
−0.02
0.00
0.02
0.04
MR leave−one−out sensitivity analysis for
'CD4 on activated & secreting Treg' on 'Hashimoto`s thyroiditis'

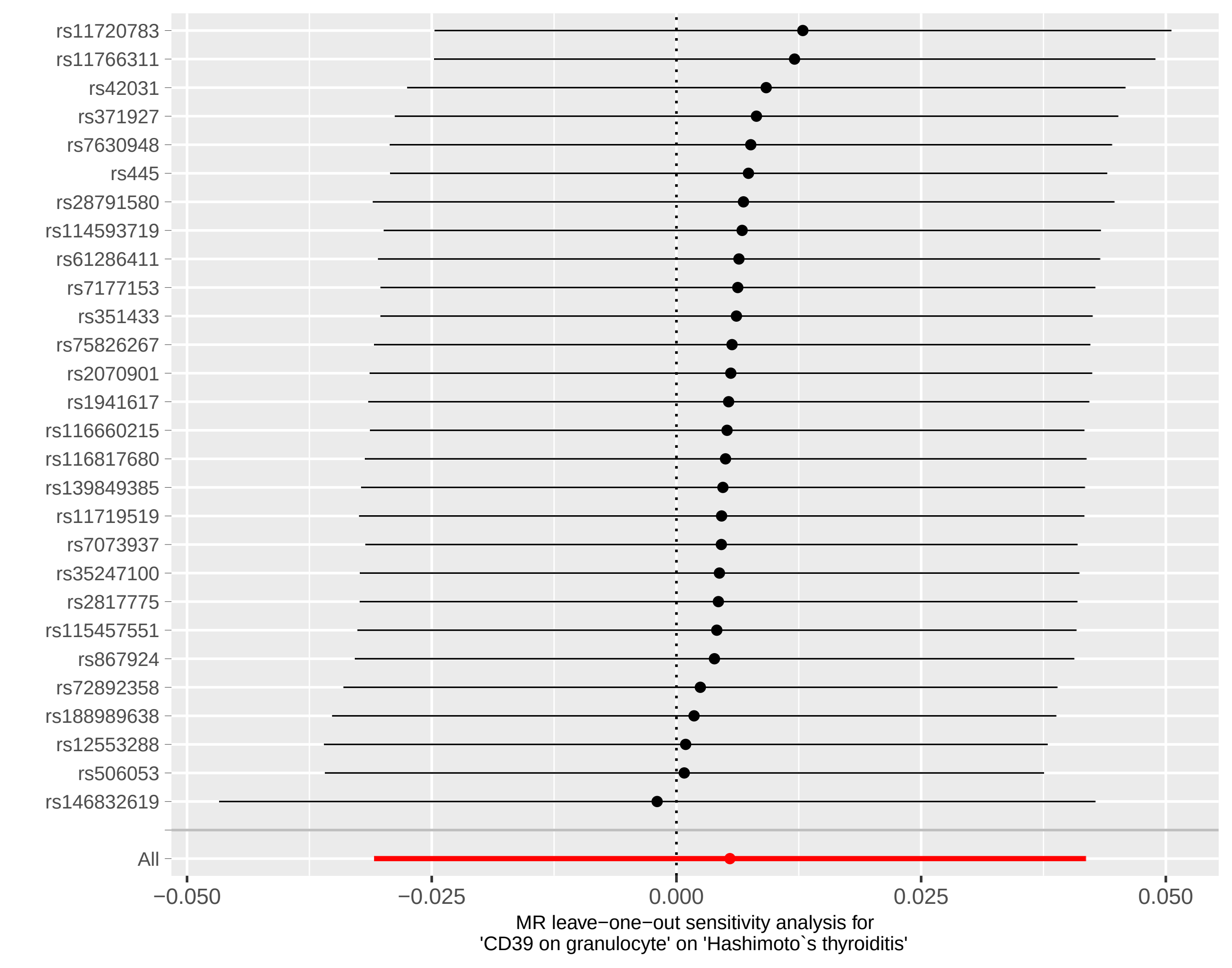
rs11720783
rs11766311
rs42031
rs371927
rs7630948
rs445
rs28791580
rs114593719
rs61286411
rs7177153
rs351433
rs75826267
rs2070901
rs1941617
rs116660215
rs116817680
rs139849385
rs11719519
rs7073937
rs35247100
rs2817775
rs115457551
rs867924
rs72892358
rs188989638
rs12553288
rs506053
rs146832619
All
−0.050
−0.025
0.000
0.025
0.050
MR leave−one−out sensitivity analysis for
'CD39 on granulocyte' on 'Hashimoto`s thyroiditis'

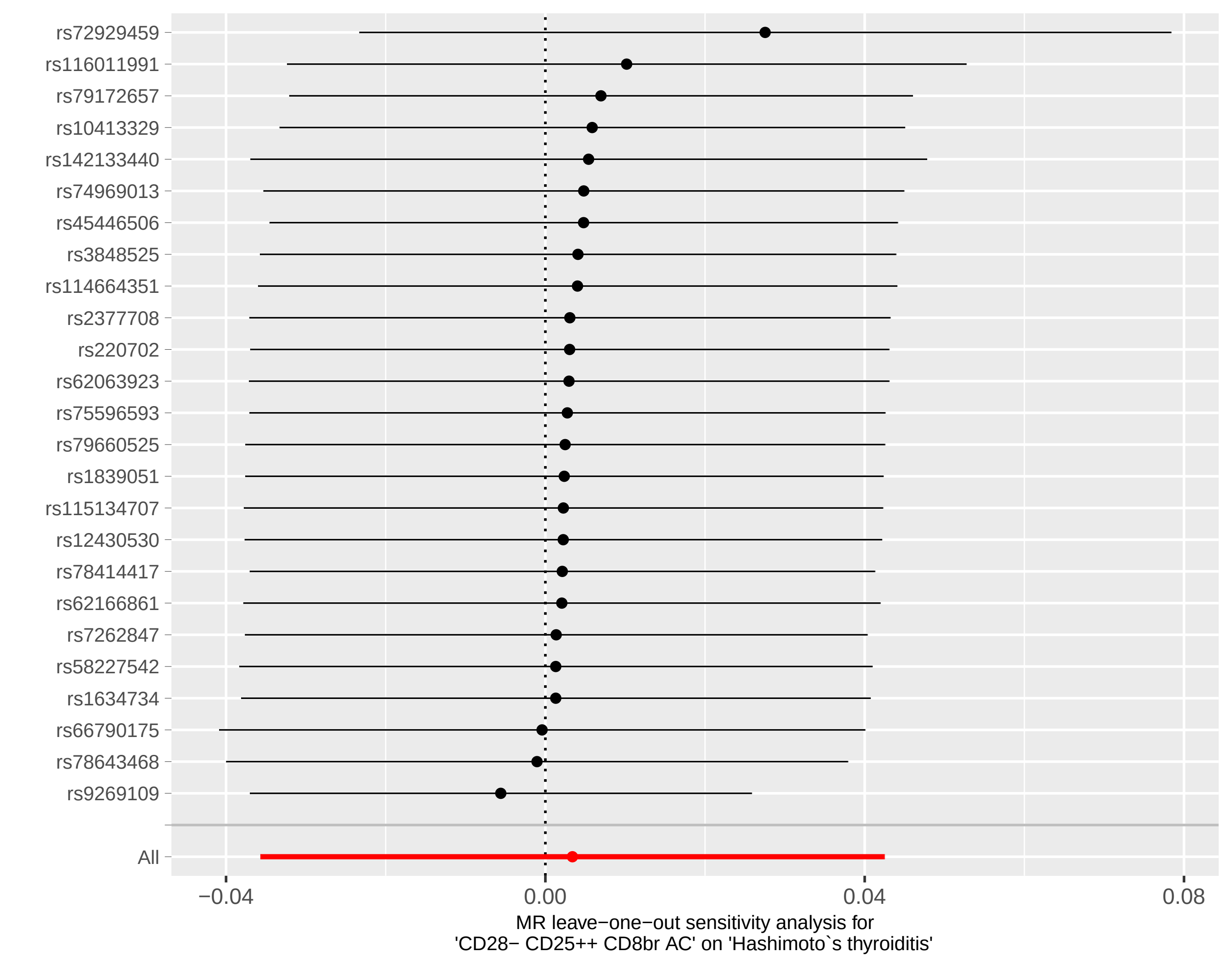
rs72929459
rs116011991
rs79172657
rs10413329
rs142133440
rs74969013
rs45446506
rs3848525
rs114664351
rs2377708
rs220702
rs62063923
rs75596593
rs79660525
rs1839051
rs115134707
rs12430530
rs78414417
rs62166861
rs7262847
rs58227542
rs1634734
rs66790175
rs78643468
rs9269109
All
−0.04
0.00
0.04
0.08
MR leave−one−out sensitivity analysis for
'CD28− CD25++ CD8br AC' on 'Hashimoto`s thyroiditis'

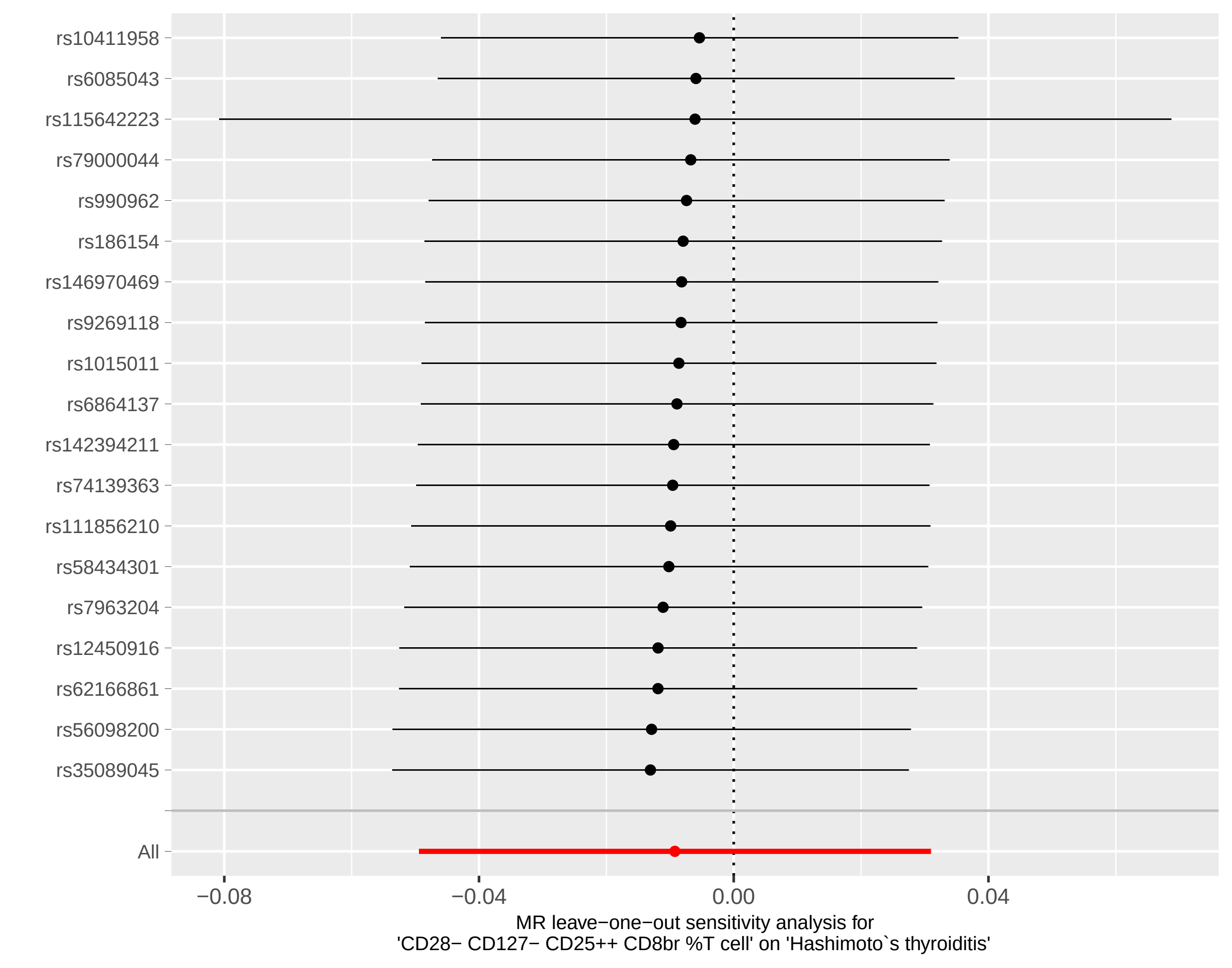
rs10411958
rs6085043
rs115642223
rs79000044
rs990962
rs186154
rs146970469
rs9269118
rs1015011
rs6864137
rs142394211
rs74139363
rs111856210
rs58434301
rs7963204
rs12450916
rs62166861
rs56098200
rs35089045
All
−0.08
−0.04
0.00
0.04
MR leave−one−out sensitivity analysis for
'CD28− CD127− CD25++ CD8br %T cell' on 'Hashimoto`s thyroiditis'

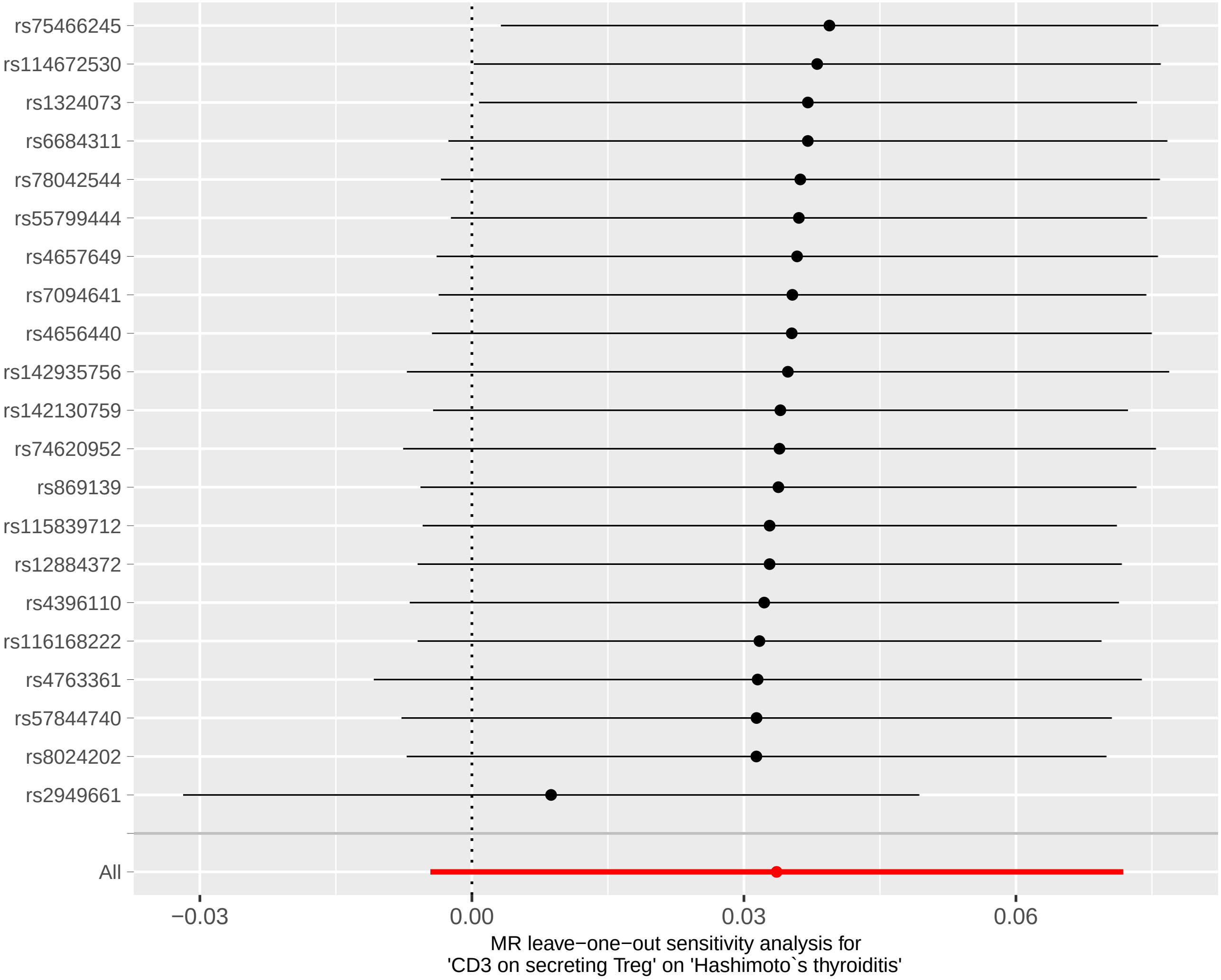
rs75466245
rs114672530
rs1324073
rs6684311
rs78042544
rs55799444
rs4657649
rs7094641
rs4656440
rs142935756
rs142130759
rs74620952
rs869139
rs115839712
rs12884372
rs4396110
rs116168222
rs4763361
rs57844740
rs8024202
rs2949661
All
−0.03
0.00
0.03
0.06
MR leave−one−out sensitivity analysis for
'CD3 on secreting Treg' on 'Hashimoto`s thyroiditis'

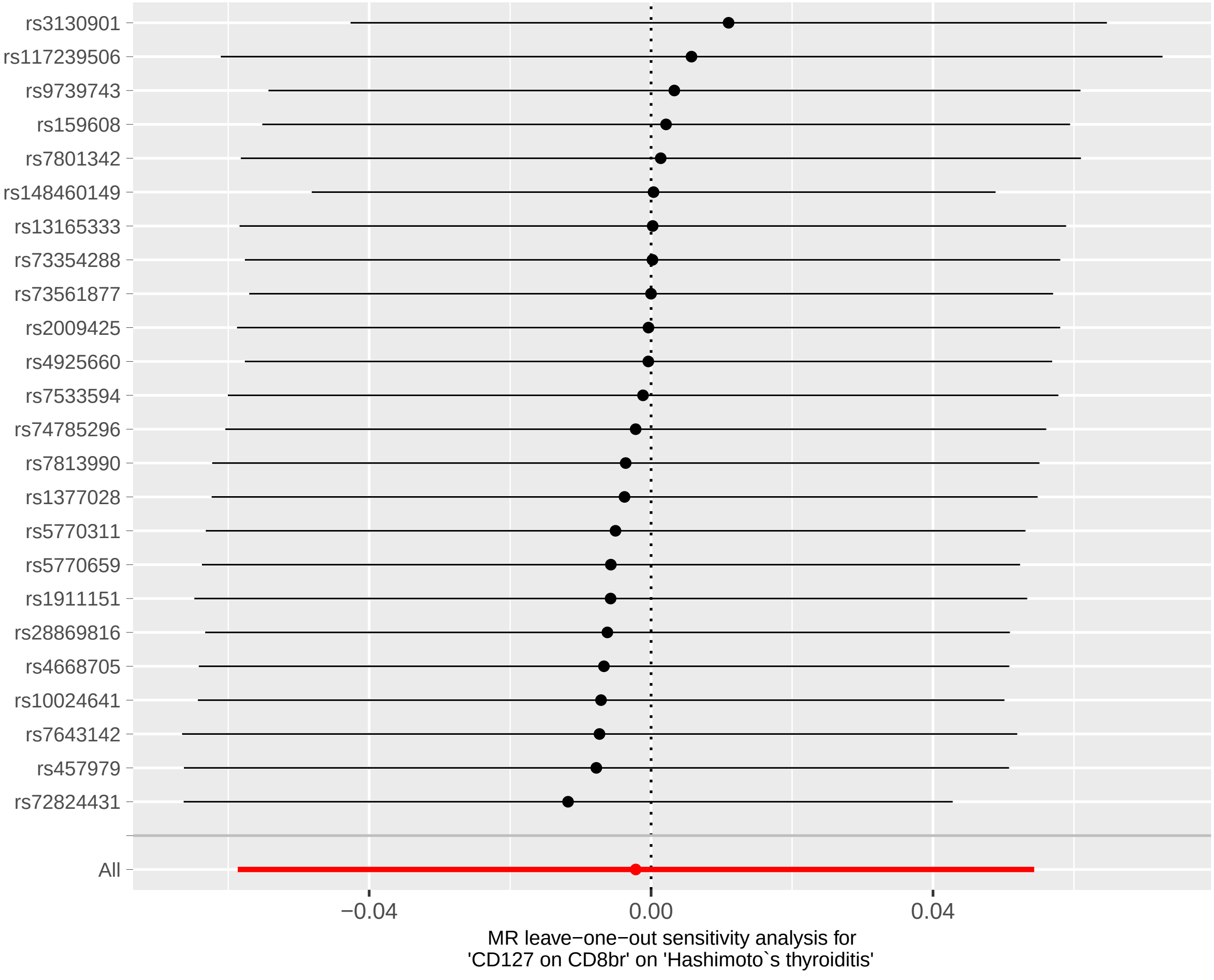
rs3130901
rs117239506
rs9739743
rs159608
rs7801342
rs148460149
rs13165333
rs73354288
rs73561877
rs2009425
rs4925660
rs7533594
rs74785296
rs7813990
rs1377028
rs5770311
rs5770659
rs1911151
rs28869816
rs4668705
rs10024641
rs7643142
rs457979
rs72824431
All
−0.04
0.00
0.04
MR leave−one−out sensitivity analysis for
'CD127 on CD8br' on 'Hashimoto`s thyroiditis'

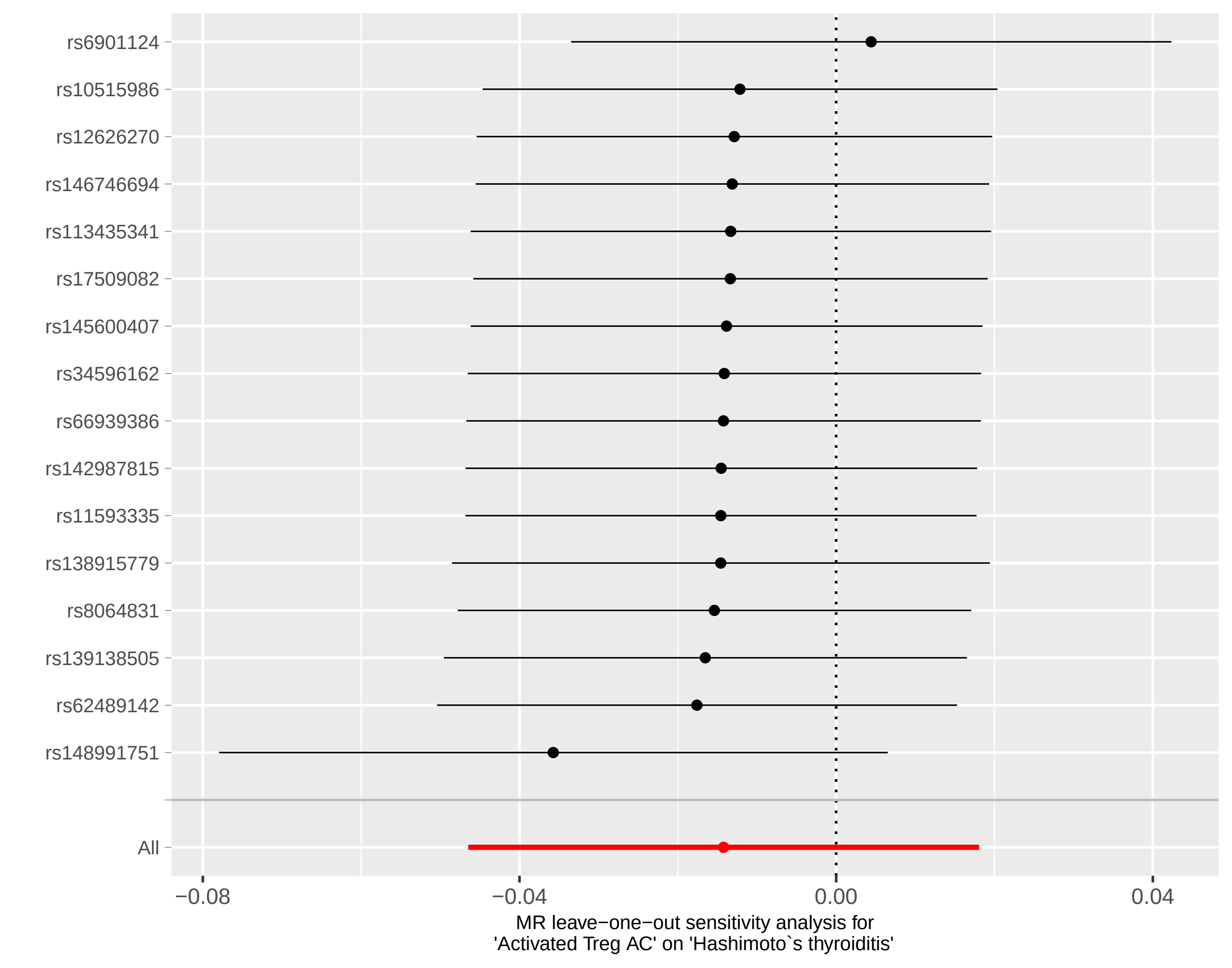
rs6901124
rs10515986
rs12626270
rs146746694
rs113435341
rs17509082
rs145600407
rs34596162
rs66939386
rs142987815
rs11593335
rs138915779
rs8064831
rs139138505
rs62489142
rs148991751
All
−0.08
−0.04
0.00
0.04
MR leave−one−out sensitivity analysis for
'Activated Treg AC' on 'Hashimoto`s thyroiditis'

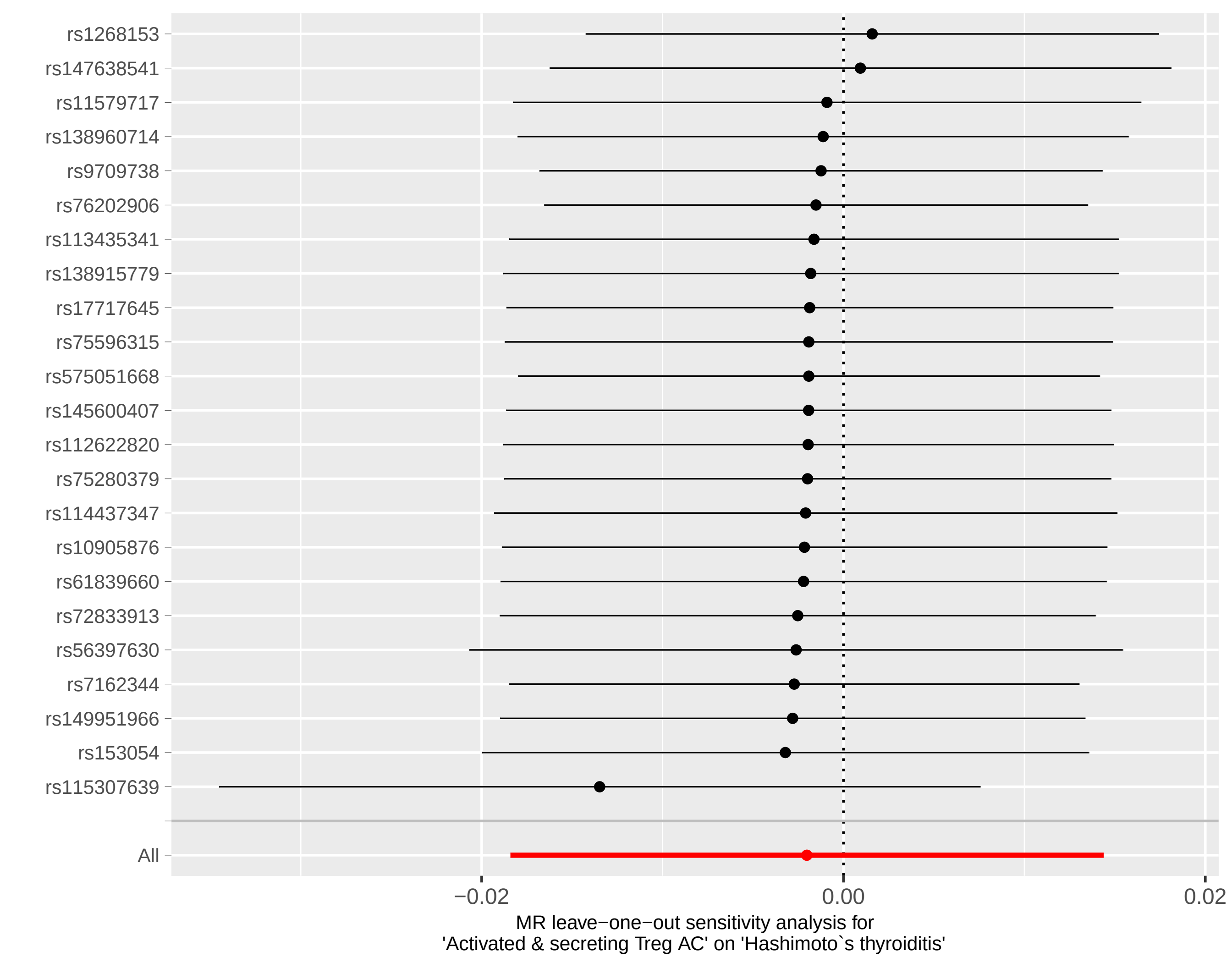
rs1268153
rs147638541
rs11579717
rs138960714
rs9709738
rs76202906
rs113435341
rs138915779
rs17717645
rs75596315
rs575051668
rs145600407
rs112622820
rs75280379
rs114437347
rs10905876
rs61839660
rs72833913
rs56397630
rs7162344
rs149951966
rs153054
rs115307639
All
−0.02
0.00
0.02
MR leave−one−out sensitivity analysis for
'Activated & secreting Treg AC' on 'Hashimoto`s thyroiditis'

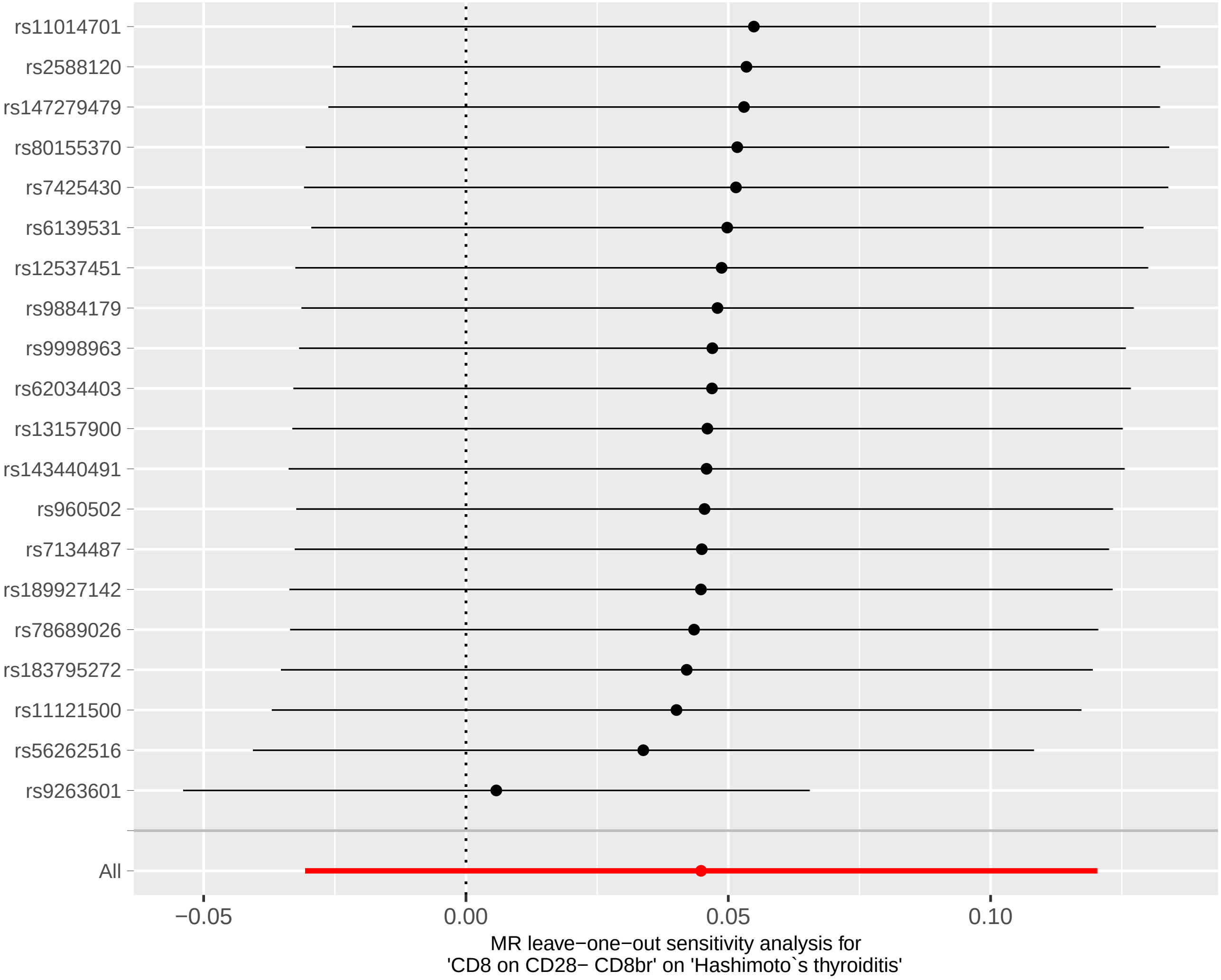

rs11014701
rs2588120
rs147279479
rs80155370
rs7425430
rs6139531
rs12537451
rs9884179
rs9998963
rs62034403
rs13157900
rs143440491
rs960502
rs7134487
rs189927142
rs78689026
rs183795272
rs11121500
rs56262516
rs9263601
All
−0.05
0.00
0.05
0.10
MR leave−one−out sensitivity analysis for
'CD8 on CD28− CD8br' on 'Hashimoto`s thyroiditis'

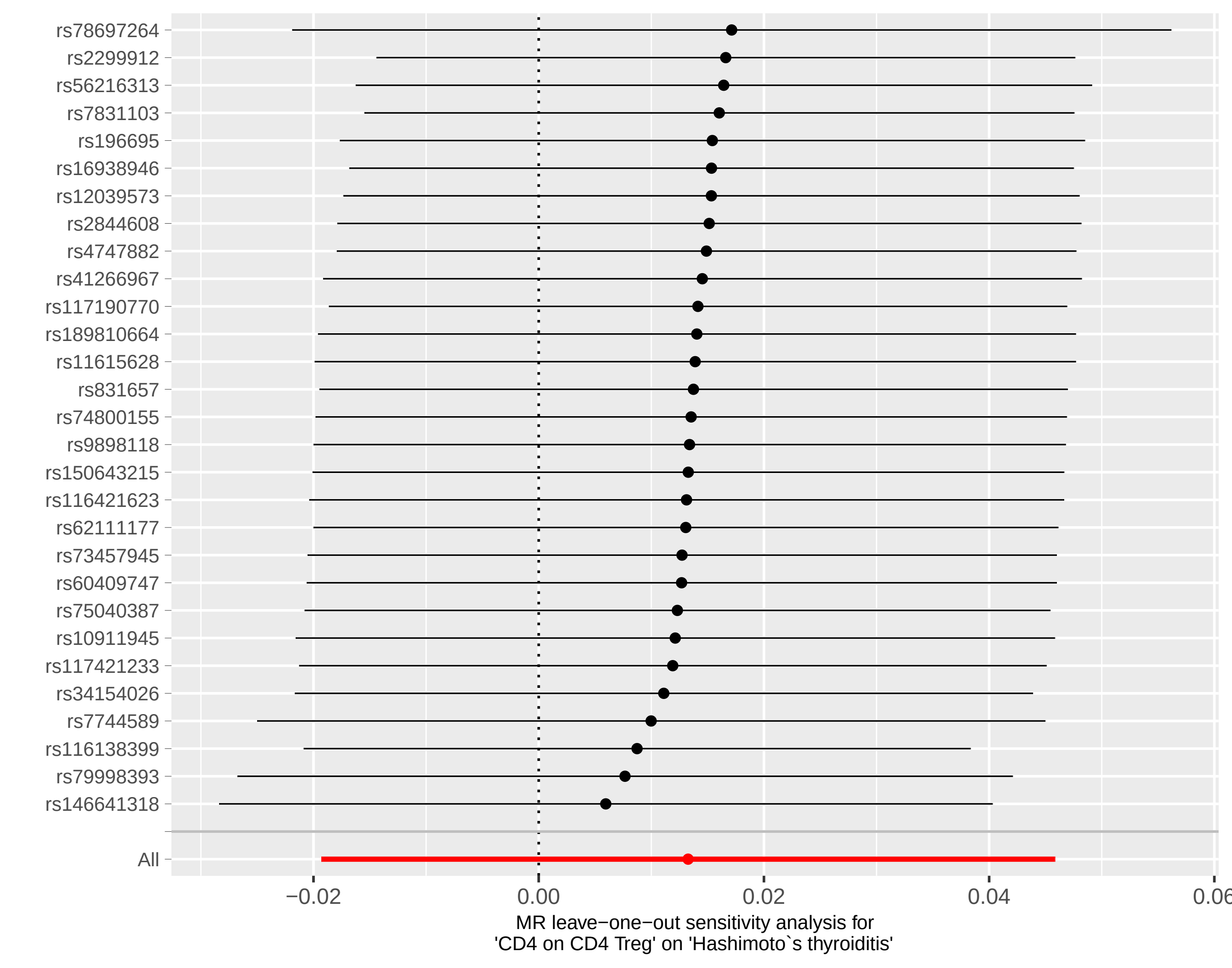
rs78697264
rs2299912
rs56216313
rs7831103
rs196695
rs16938946
rs12039573
rs2844608
rs4747882
rs41266967
rs117190770
rs189810664
rs11615628
rs831657
rs74800155
rs9898118
rs150643215
rs116421623
rs62111177
rs73457945
rs60409747
rs75040387
rs10911945
rs117421233
rs34154026
rs7744589
rs116138399
rs79998393
rs146641318
All
−0.02
0.00
0.02
0.04
0.06
MR leave−one−out sensitivity analysis for
'CD4 on CD4 Treg' on 'Hashimoto`s thyroiditis'

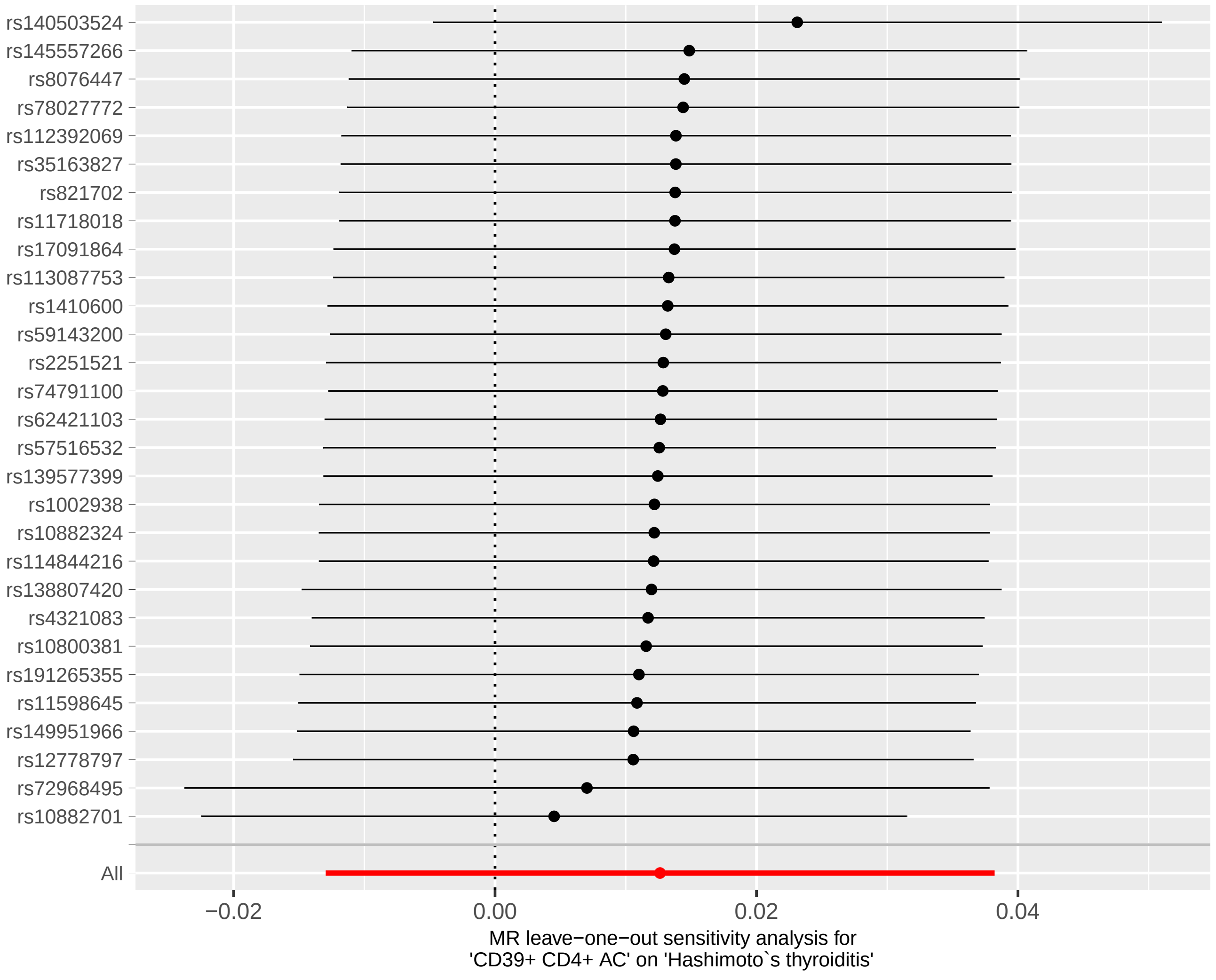
rs140503524
rs145557266
rs8076447
rs78027772
rs112392069
rs35163827
rs821702
rs11718018
rs17091864
rs113087753
rs1410600
rs59143200
rs2251521
rs74791100
rs62421103
rs57516532
rs139577399
rs1002938
rs10882324
rs114844216
rs138807420
rs4321083
rs10800381
rs191265355
rs11598645
rs149951966
rs12778797
rs72968495
rs10882701
All
−0.02
0.00
0.02
0.04
MR leave−one−out sensitivity analysis for
'CD39+ CD4+ AC' on 'Hashimoto`s thyroiditis'

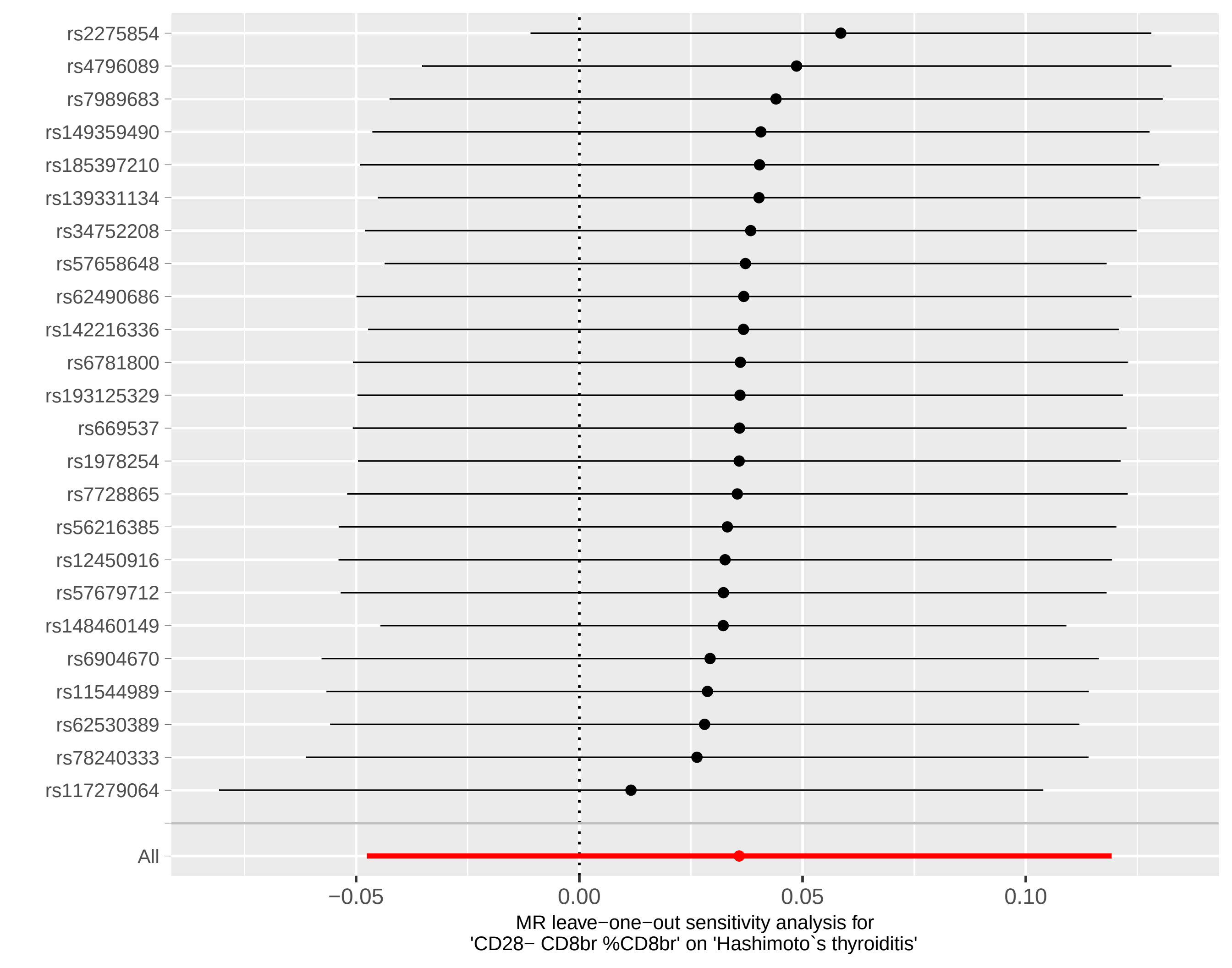

rs2275854
rs4796089
rs7989683
rs149359490
rs185397210
rs139331134
rs34752208
rs57658648
rs62490686
rs142216336
rs6781800
rs193125329
rs669537
rs1978254
rs7728865
rs56216385
rs12450916
rs57679712
rs148460149
rs6904670
rs11544989
rs62530389
rs78240333
rs117279064
All
−0.05
0.00
0.05
0.10
MR leave−one−out sensitivity analysis for
'CD28− CD8br %CD8br' on 'Hashimoto`s thyroiditis'

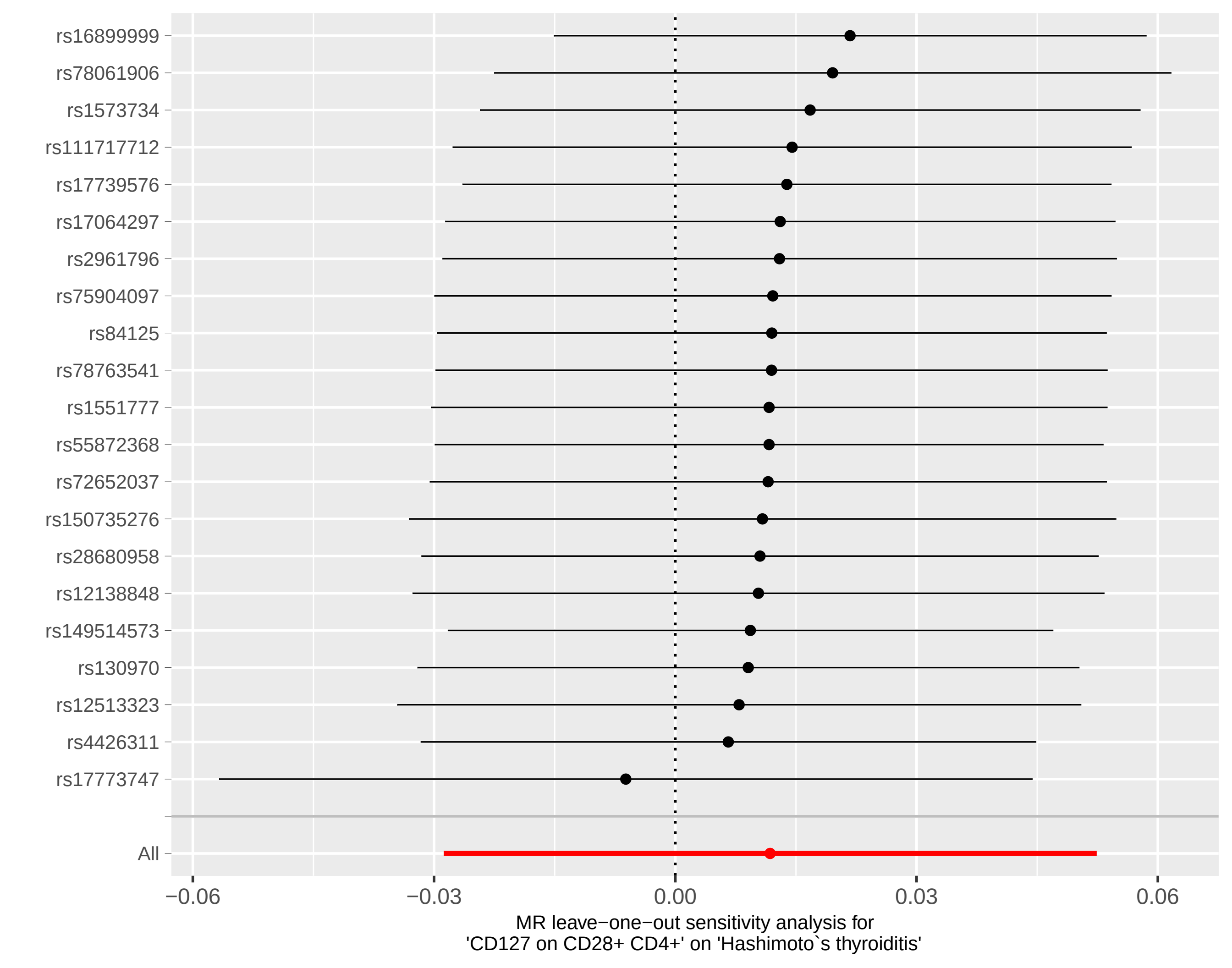
rs16899999
rs78061906
rs1573734
rs111717712
rs17739576
rs17064297
rs2961796
rs75904097
rs84125
rs78763541
rs1551777
rs55872368
rs72652037
rs150735276
rs28680958
rs12138848
rs149514573
rs130970
rs12513323
rs4426311
rs17773747
All
−0.06
−0.03
0.00
0.03
0.06
MR leave−one−out sensitivity analysis for
'CD127 on CD28+ CD4+' on 'Hashimoto`s thyroiditis'

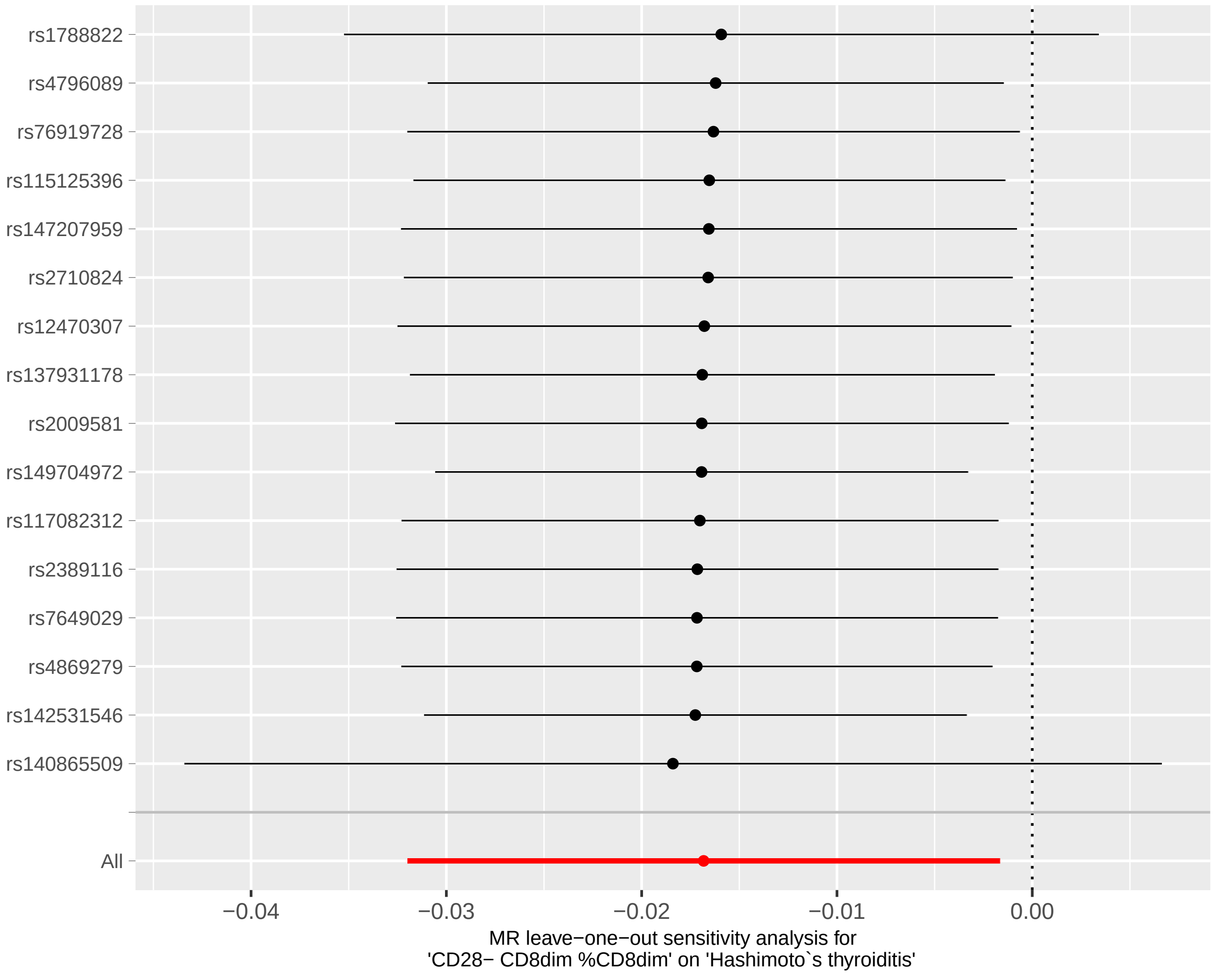

rs1788822
rs4796089
rs76919728
rs115125396
rs147207959
rs2710824
rs12470307
rs137931178
rs2009581
rs149704972
rs117082312
rs2389116
rs7649029
rs4869279
rs142531546
rs140865509
All
−0.04
−0.03
−0.02
−0.01
0.00
MR leave−one−out sensitivity analysis for
'CD28− CD8dim %CD8dim' on 'Hashimoto`s thyroiditis'

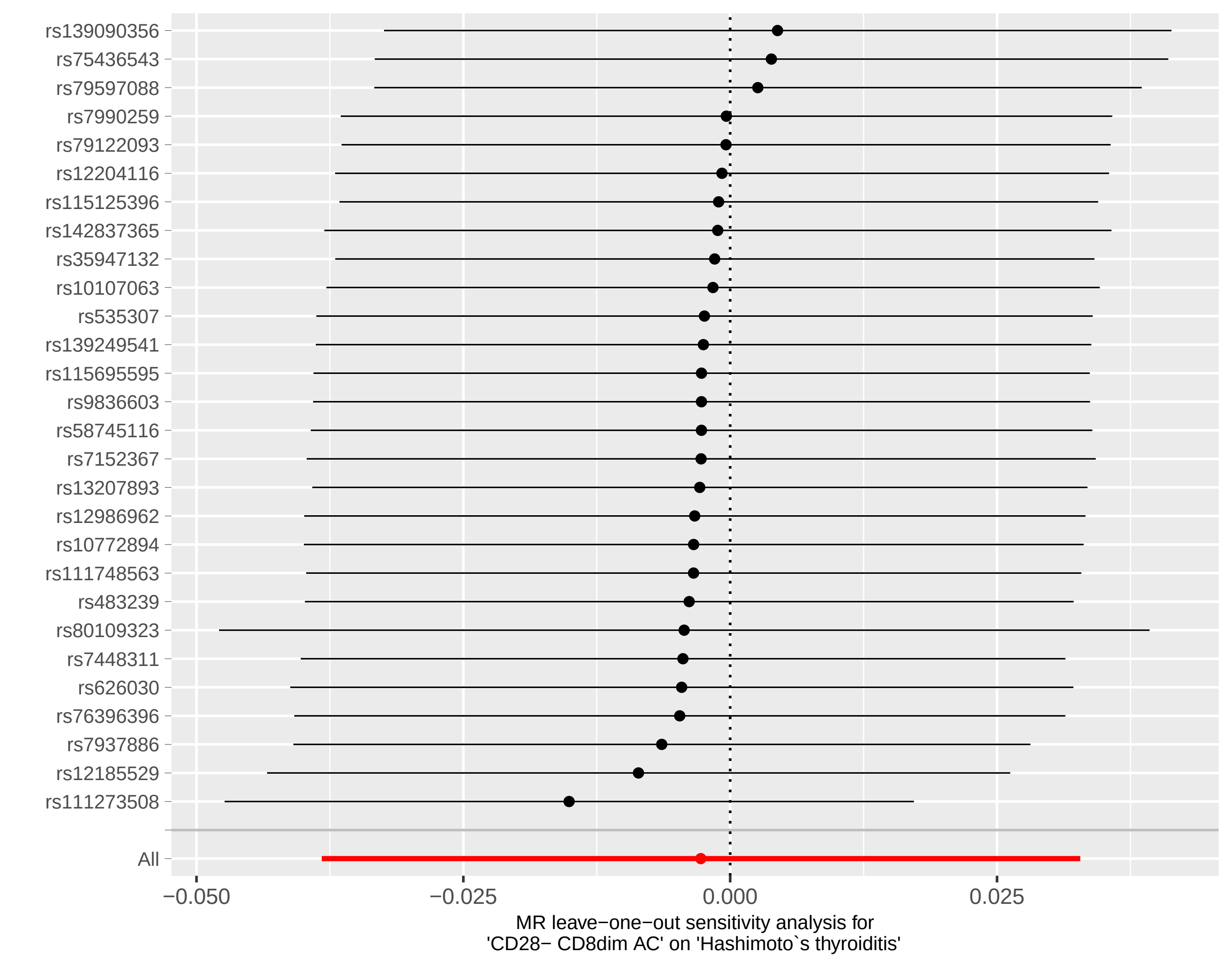
rs139090356
rs75436543
rs79597088
rs7990259
rs79122093
rs12204116
rs115125396
rs142837365
rs35947132
rs10107063
rs535307
rs139249541
rs115695595
rs9836603
rs58745116
rs7152367
rs13207893
rs12986962
rs10772894
rs111748563
rs483239
rs80109323
rs7448311
rs626030
rs76396396
rs7937886
rs12185529
rs111273508
All
−0.050
−0.025
0.000
0.025
MR leave−one−out sensitivity analysis for
'CD28− CD8dim AC' on 'Hashimoto`s thyroiditis'

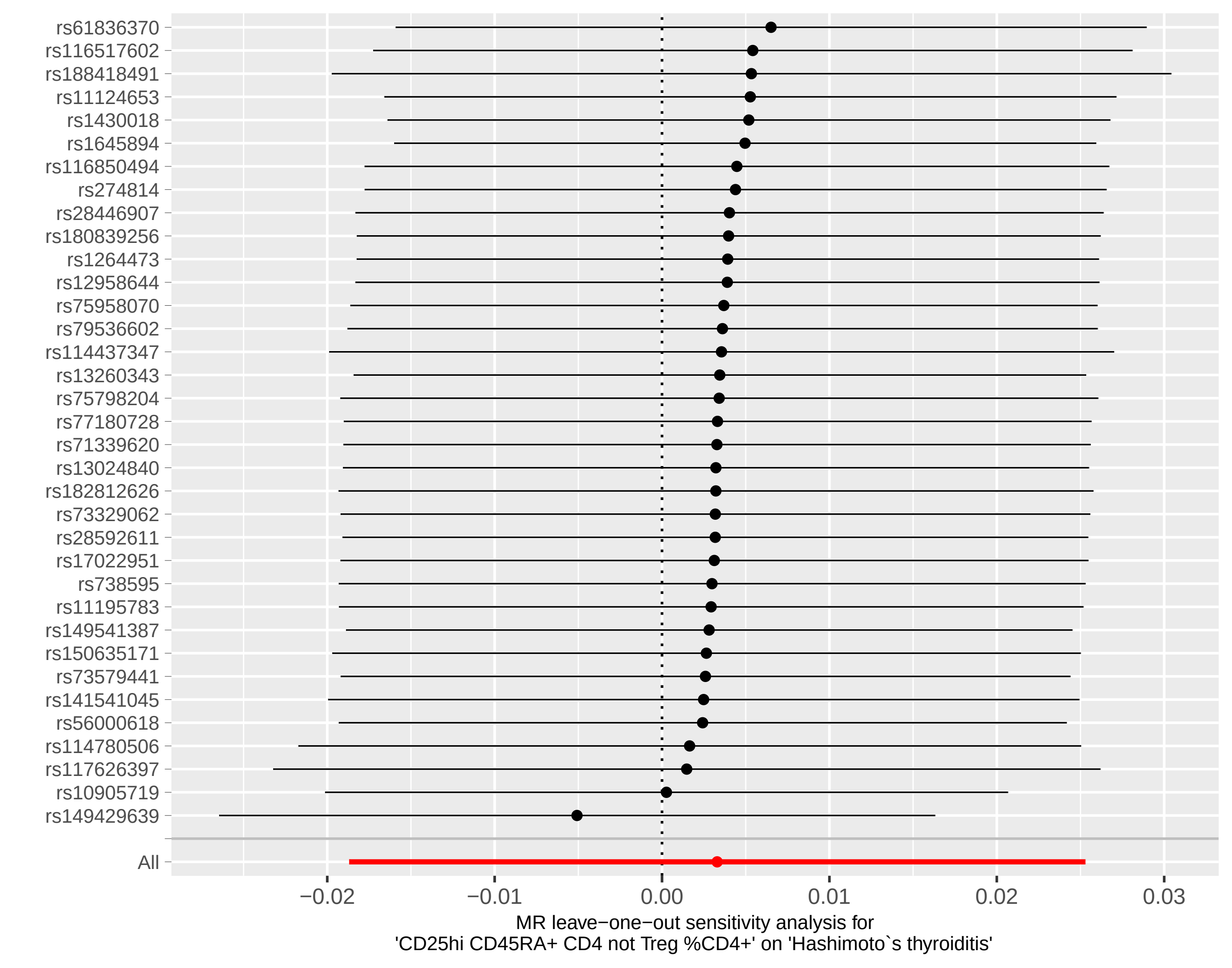
rs61836370
rs116517602
rs188418491
rs11124653
rs1430018
rs1645894
rs116850494
rs274814
rs28446907
rs180839256
rs1264473
rs12958644
rs75958070
rs79536602
rs114437347
rs13260343
rs75798204
rs77180728
rs71339620
rs13024840
rs182812626
rs73329062
rs28592611
rs17022951
rs738595
rs11195783
rs149541387
rs150635171
rs73579441
rs141541045
rs56000618
rs114780506
rs117626397
rs10905719
rs149429639
All
−0.02
−0.01
0.00
0.01
0.02
0.03
MR leave−one−out sensitivity analysis for
'CD25hi CD45RA+ CD4 not Treg %CD4+' on 'Hashimoto`s thyroiditis'

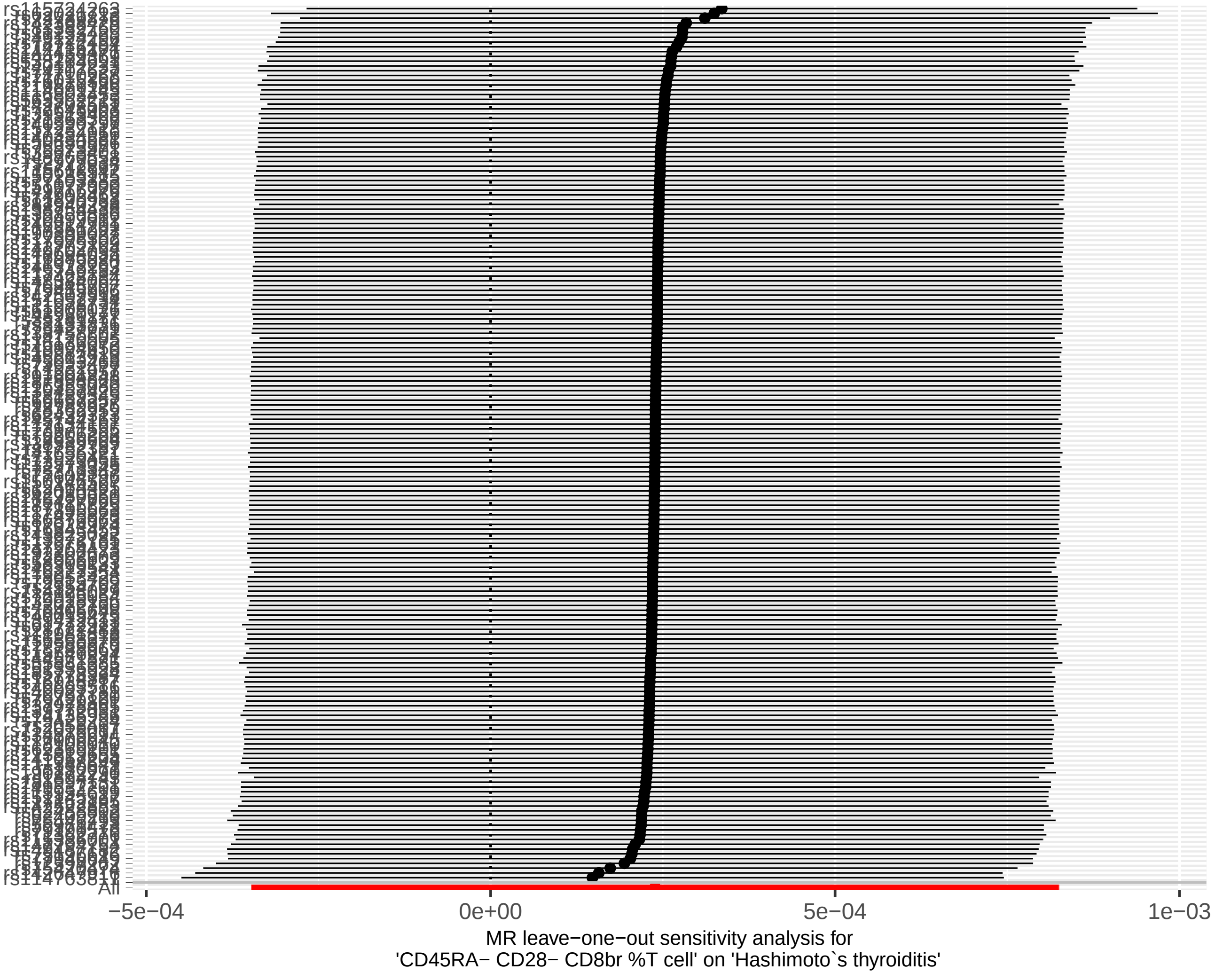

All
−5e−04
0e+00
5e−04
1e−03
MR leave−one−out sensitivity analysis for
'CD45RA− CD28− CD8br %T cell' on 'Hashimoto`s thyroiditis'

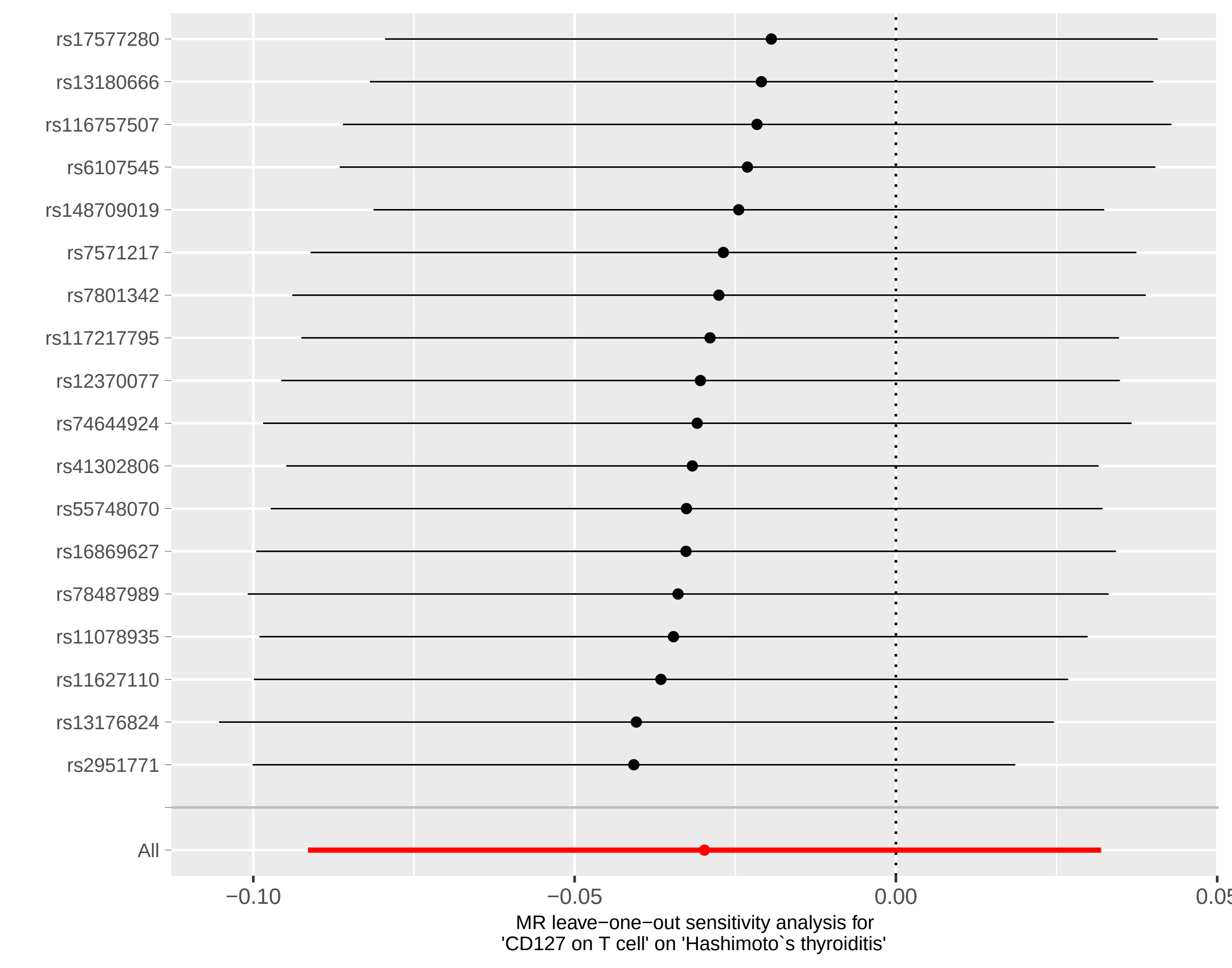
rs17577280
rs13180666
rs116757507
rs6107545
rs148709019
rs7571217
rs7801342
rs117217795
rs12370077
rs74644924
rs41302806
rs55748070
rs16869627
rs78487989
rs11078935
rs11627110
rs13176824
rs2951771
All
−0.10
−0.05
0.00
MR leave−one−out sensitivity analysis for
'CD127 on T cell' on 'Hashimoto`s thyroiditis'

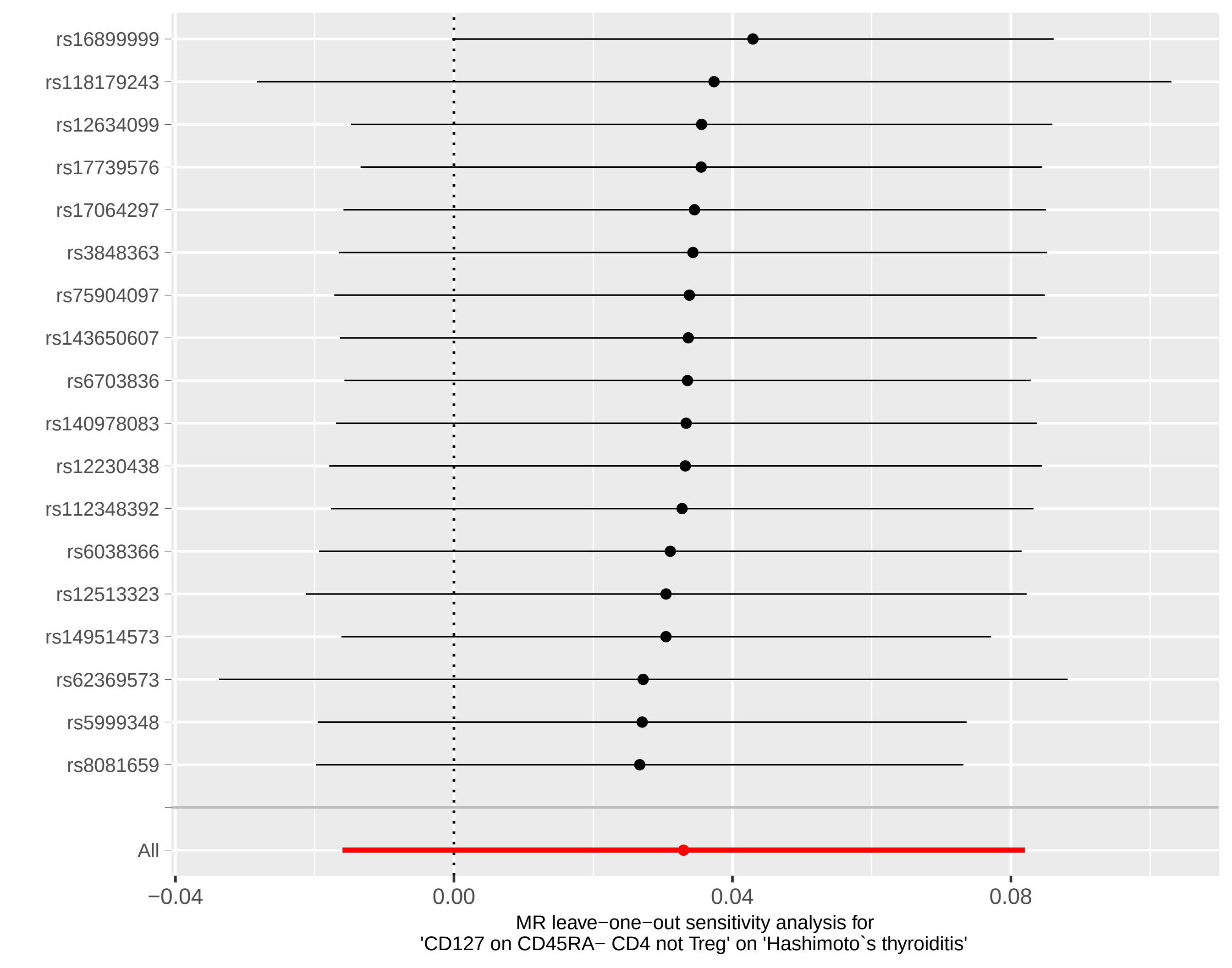
rs16899999
rs118179243
rs12634099
rs17739576
rs17064297
rs3848363
rs75904097
rs143650607
rs6703836
rs140978083
rs12230438
rs112348392
rs6038366
rs12513323
rs149514573
rs62369573
rs5999348
rs8081659
All
−0.04
0.00
0.04
0.08
MR leave−one−out sensitivity analysis for
'CD127 on CD45RA− CD4 not Treg' on 'Hashimoto`s thyroiditis'

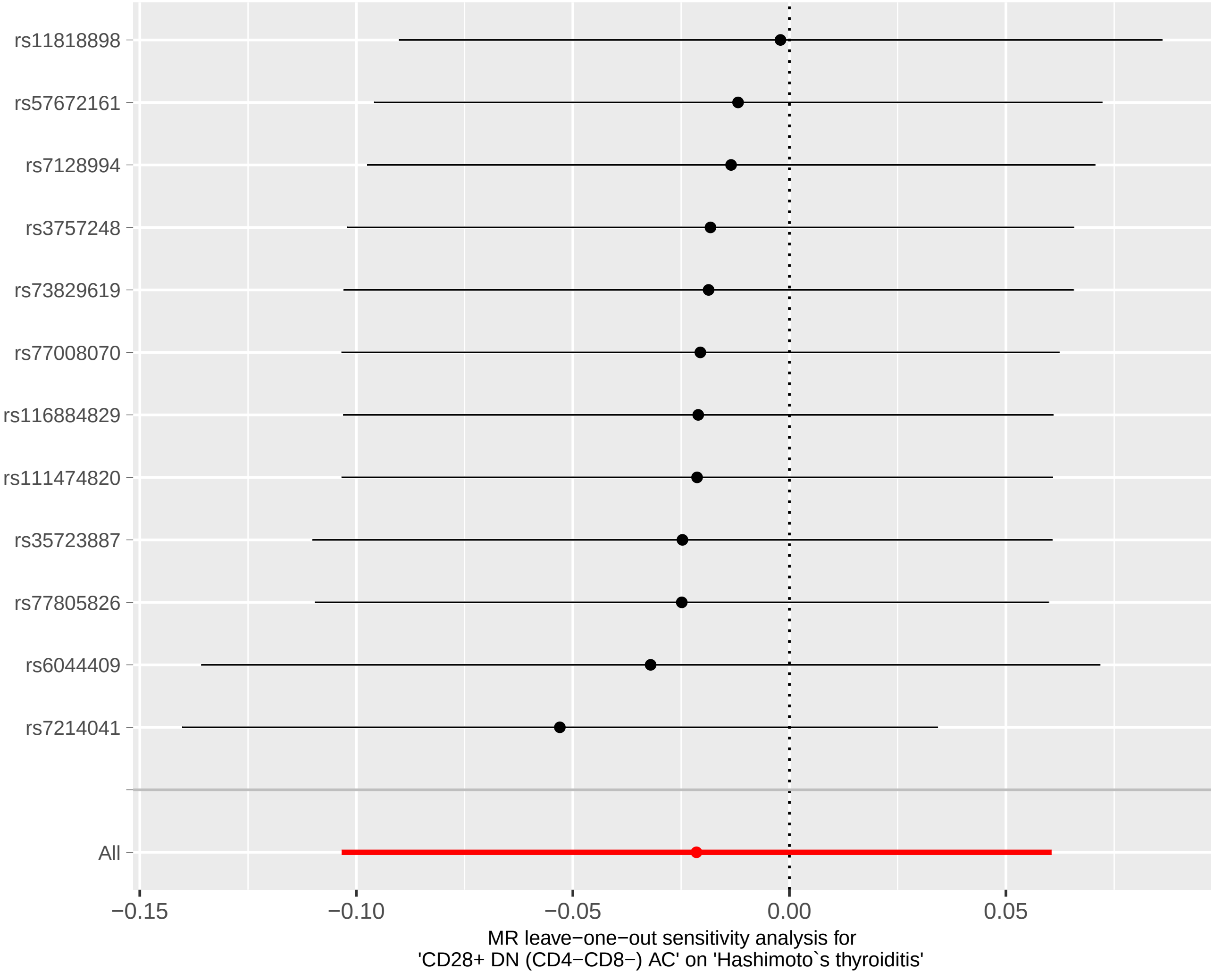
rs11818898
rs57672161
rs7128994
rs3757248
rs73829619
rs77008070
rs116884829
rs111474820
rs35723887
rs77805826
rs6044409
rs7214041
All
−0.15
−0.10
−0.05
0.00
0.05
MR leave−one−out sensitivity analysis for
'CD28+ DN (CD4−CD8−) AC' on 'Hashimoto`s thyroiditis'

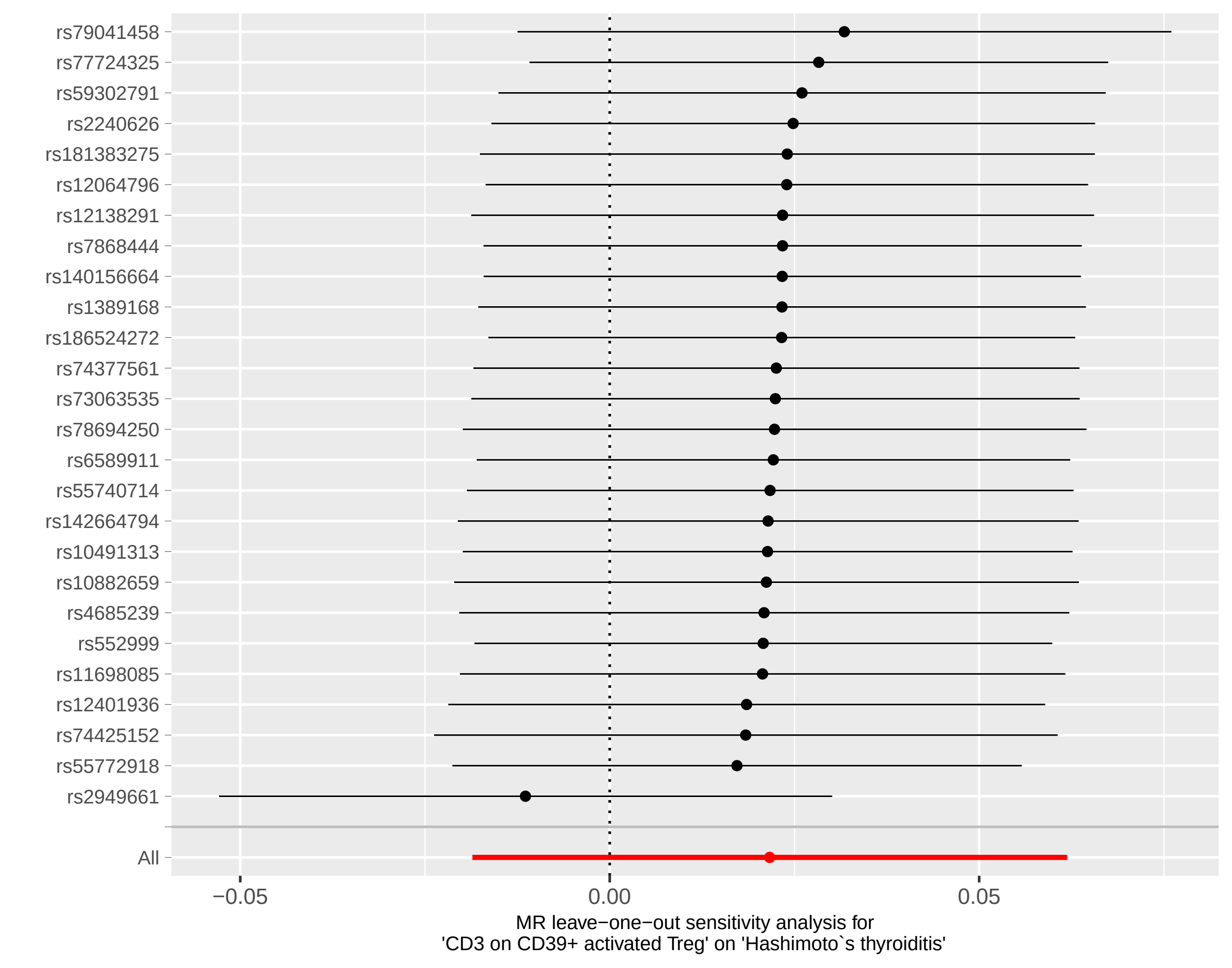
rs79041458
rs77724325
rs59302791
rs2240626
rs181383275
rs12064796
rs12138291
rs7868444
rs140156664
rs1389168
rs186524272
rs74377561
rs73063535
rs78694250
rs6589911
rs55740714
rs142664794
rs10491313
rs10882659
rs4685239
rs552999
rs11698085
rs12401936
rs74425152
rs55772918
rs2949661
All
−0.05
0.00
0.05
MR leave−one−out sensitivity analysis for
'CD3 on CD39+ activated Treg' on 'Hashimoto`s thyroiditis'

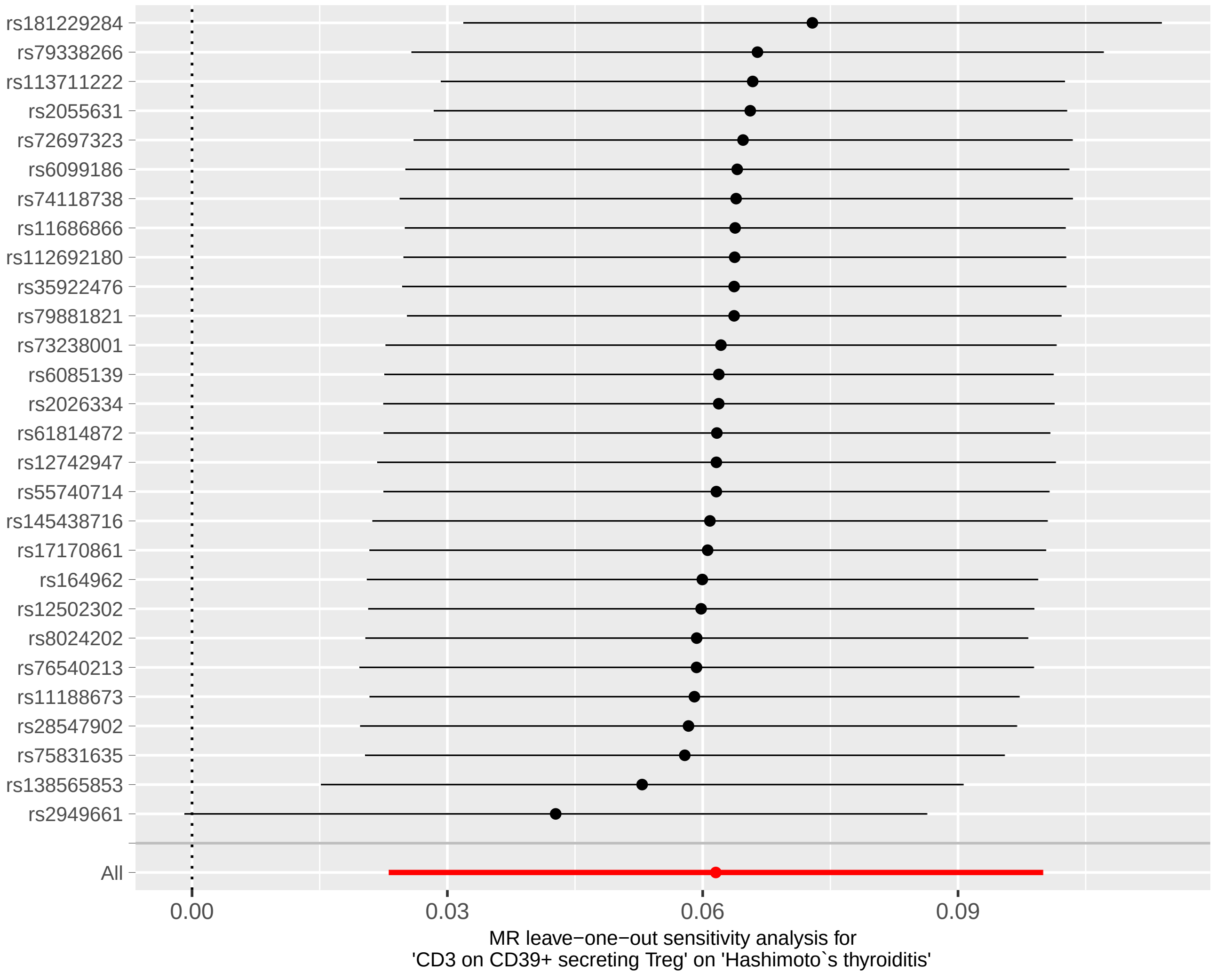
rs181229284
rs79338266
rs113711222
rs2055631
rs72697323
rs6099186
rs74118738
rs11686866
rs112692180
rs35922476
rs79881821
rs73238001
rs6085139
rs2026334
rs61814872
rs12742947
rs55740714
rs145438716
rs17170861
rs164962
rs12502302
rs8024202
rs76540213
rs11188673
rs28547902
rs75831635
rs138565853
rs2949661
All
0.00
0.03
0.06
0.09
MR leave−one−out sensitivity analysis for
'CD3 on CD39+ secreting Treg' on 'Hashimoto`s thyroiditis'

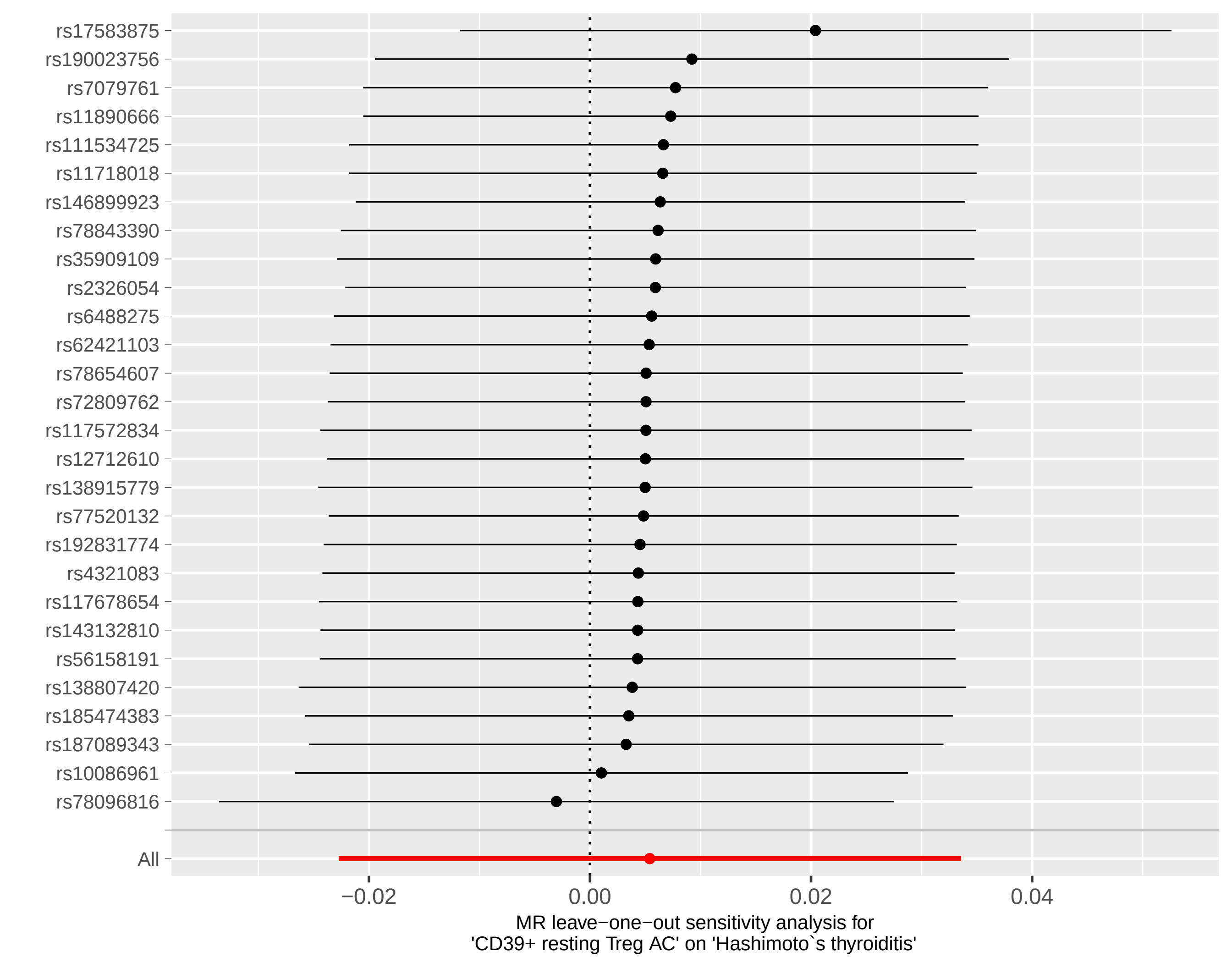
rs17583875
rs190023756
rs7079761
rs11890666
rs111534725
rs11718018
rs146899923
rs78843390
rs35909109
rs2326054
rs6488275
rs62421103
rs78654607
rs72809762
rs117572834
rs12712610
rs138915779
rs77520132
rs192831774
rs4321083
rs117678654
rs143132810
rs56158191
rs138807420
rs185474383
rs187089343
rs10086961
rs78096816
All
−0.02
0.00
0.02
0.04
MR leave−one−out sensitivity analysis for
'CD39+ resting Treg AC' on 'Hashimoto`s thyroiditis'

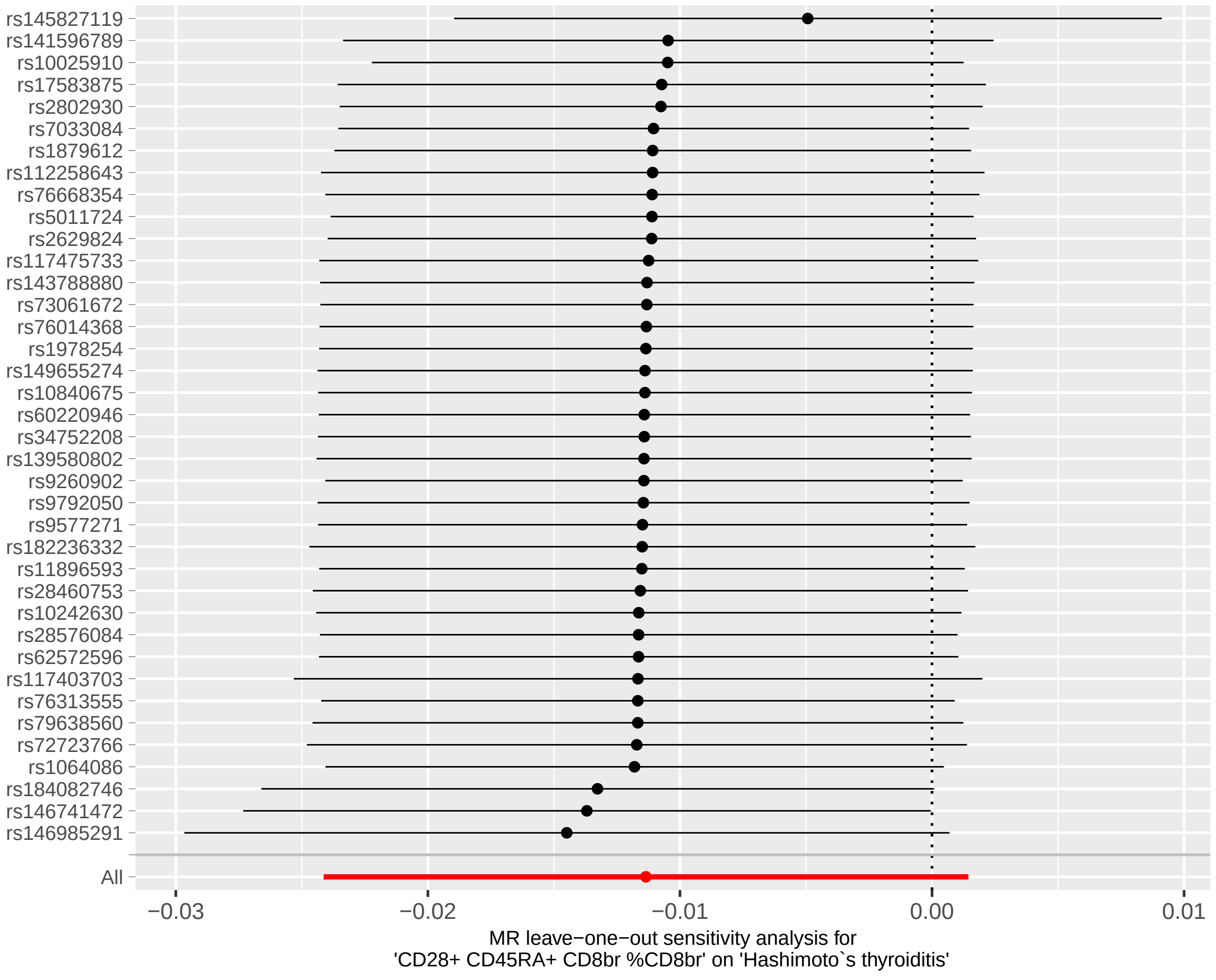
rs145827119
rs141596789
rs10025910
rs17583875
rs2802930
rs7033084
rs1879612
rs112258643
rs76668354
rs5011724
rs2629824
rs117475733
rs143788880
rs73061672
rs76014368
rs1978254
rs149655274
rs10840675
rs60220946
rs34752208
rs139580802
rs9260902
rs9792050
rs9577271
rs182236332
rs11896593
rs28460753
rs10242630
rs28576084
rs62572596
rs117403703
rs76313555
rs79638560
rs72723766
rs1064086
rs184082746
rs146741472
rs146985291
All
−0.03
−0.02
−0.01
0.00
0.01
MR leave−one−out sensitivity analysis for
'CD28+ CD45RA+ CD8br %CD8br' on 'Hashimoto`s thyroiditis'

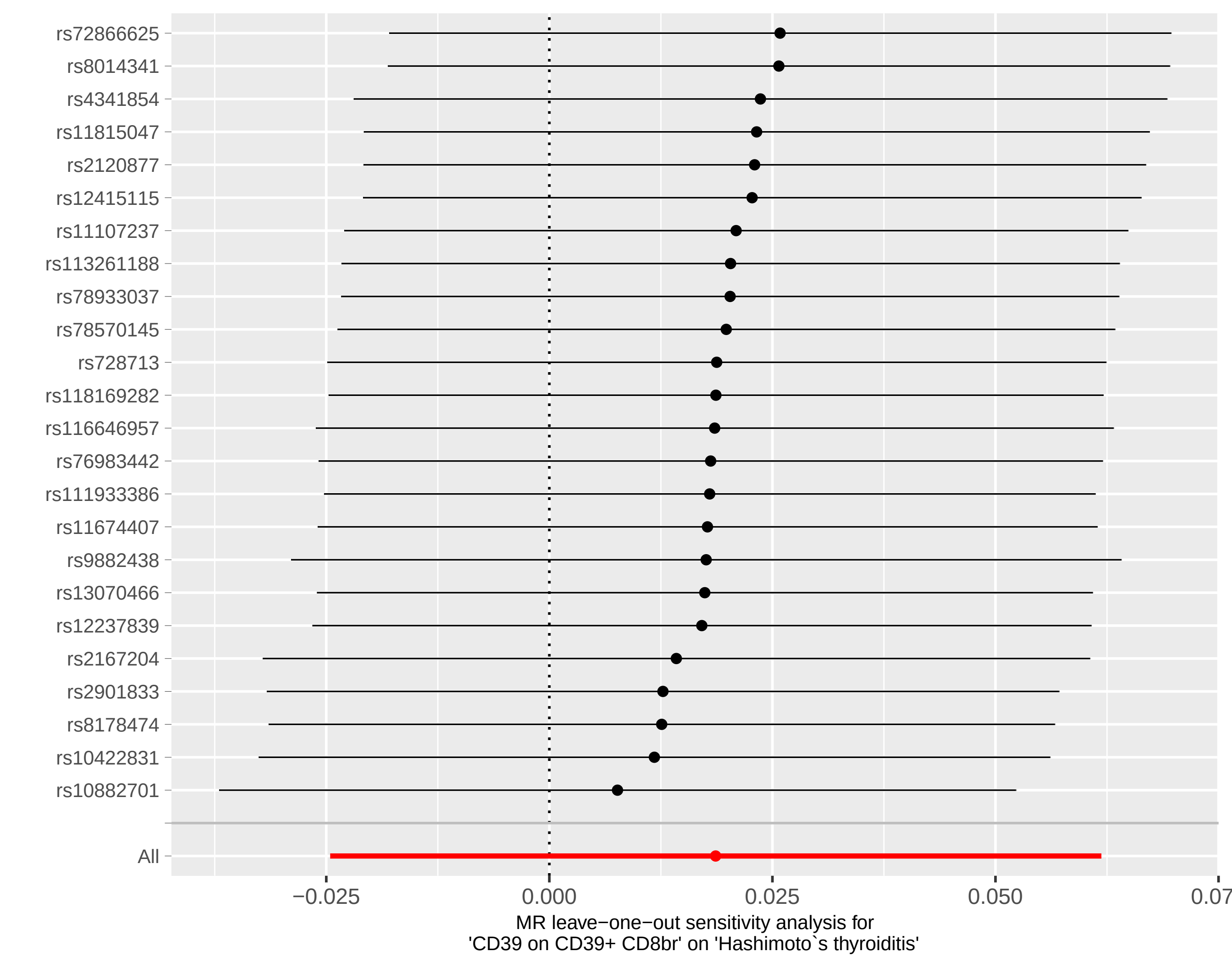
rs72866625
rs8014341
rs4341854
rs11815047
rs2120877
rs12415115
rs11107237
rs113261188
rs78933037
rs78570145
rs728713
rs118169282
rs116646957
rs76983442
rs111933386
rs11674407
rs9882438
rs13070466
rs12237839
rs2167204
rs2901833
rs8178474
rs10422831
rs10882701
All
−0.025
0.000
0.025
0.050
0.07
MR leave−one−out sensitivity analysis for
'CD39 on CD39+ CD8br' on 'Hashimoto`s thyroiditis'

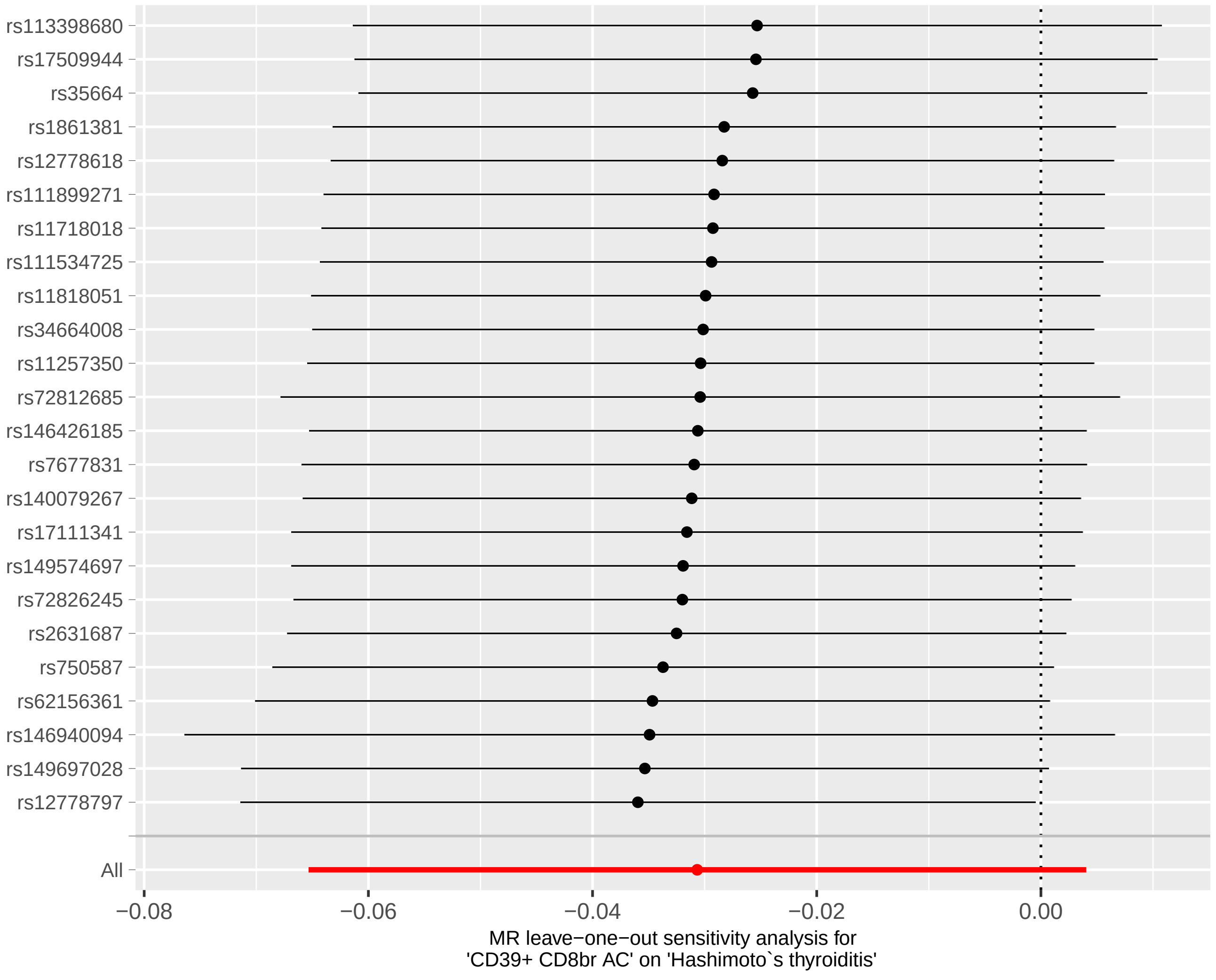
rs113398680
rs17509944
rs35664
rs1861381
rs12778618
rs111899271
rs11718018
rs111534725
rs11818051
rs34664008
rs11257350
rs72812685
rs146426185
rs7677831
rs140079267
rs17111341
rs149574697
rs72826245
rs2631687
rs750587
rs62156361
rs146940094
rs149697028
rs12778797
All
−0.08
−0.06
−0.04
−0.02
0.00
MR leave−one−out sensitivity analysis for
'CD39+ CD8br AC' on 'Hashimoto`s thyroiditis'

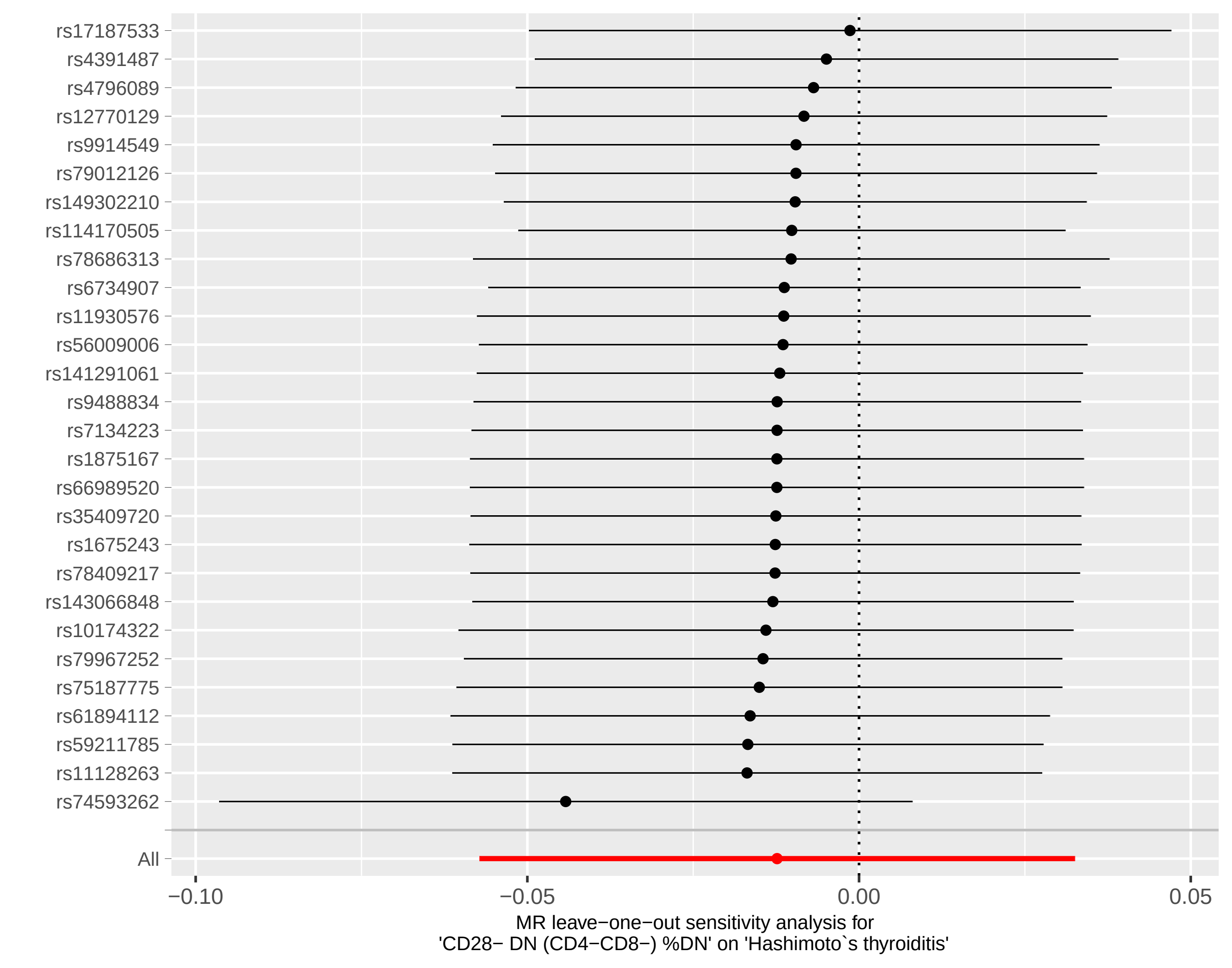
rs17187533
rs4391487
rs4796089
rs12770129
rs9914549
rs79012126
rs149302210
rs114170505
rs78686313
rs6734907
rs11930576
rs56009006
rs141291061
rs9488834
rs7134223
rs1875167
rs66989520
rs35409720
rs1675243
rs78409217
rs143066848
rs10174322
rs79967252
rs75187775
rs61894112
rs59211785
rs11128263
rs74593262
All
−0.10
−0.05
0.00
0.05
MR leave−one−out sensitivity analysis for
'CD28− DN (CD4−CD8−) %DN' on 'Hashimoto`s thyroiditis'

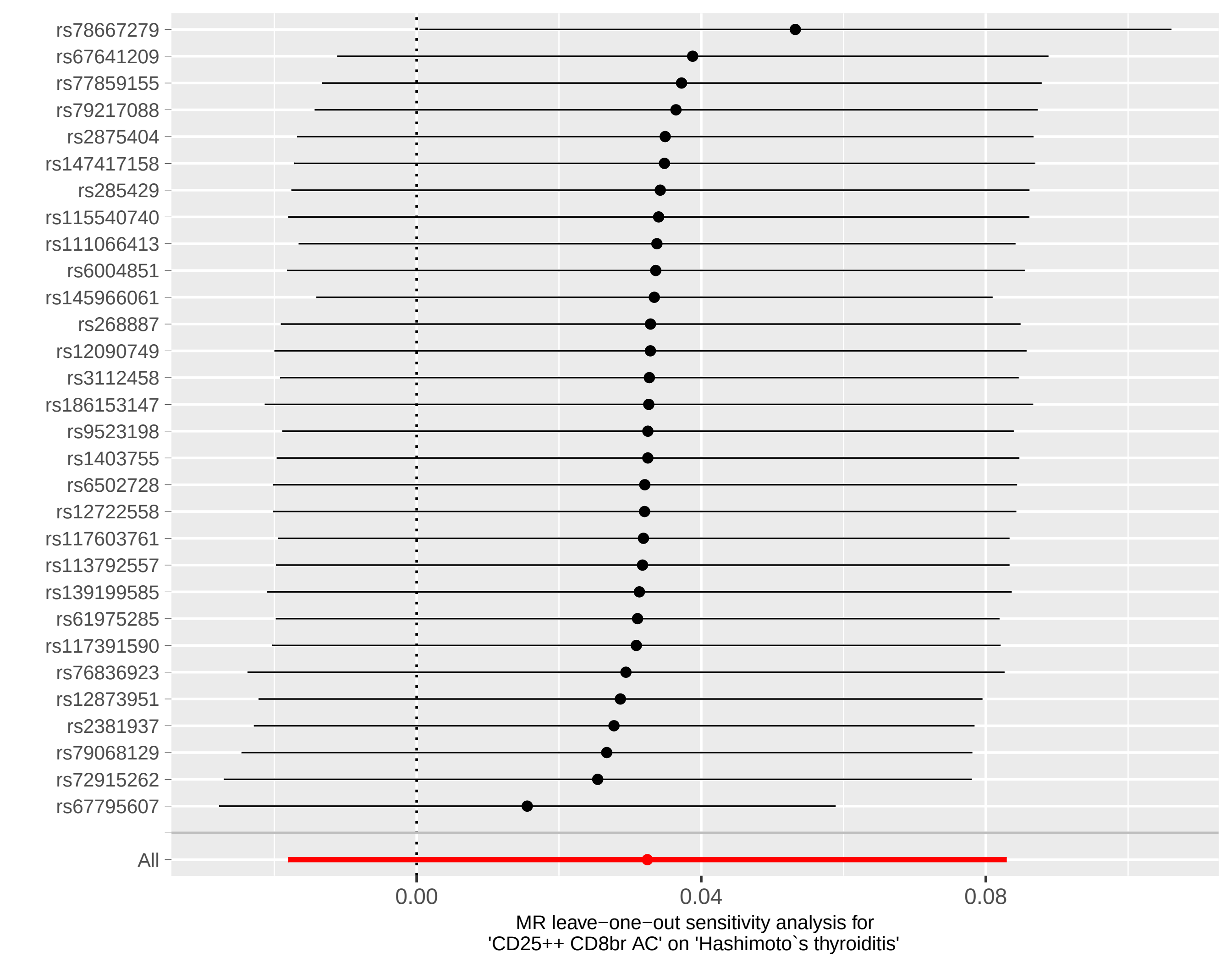
rs78667279
rs67641209
rs77859155
rs79217088
rs2875404
rs147417158
rs285429
rs115540740
rs111066413
rs6004851
rs145966061
rs268887
rs12090749
rs3112458
rs186153147
rs9523198
rs1403755
rs6502728
rs12722558
rs117603761
rs113792557
rs139199585
rs61975285
rs117391590
rs76836923
rs12873951
rs2381937
rs79068129
rs72915262
rs67795607
All
0.00
0.04
0.08
MR leave−one−out sensitivity analysis for
'CD25++ CD8br AC' on 'Hashimoto`s thyroiditis'

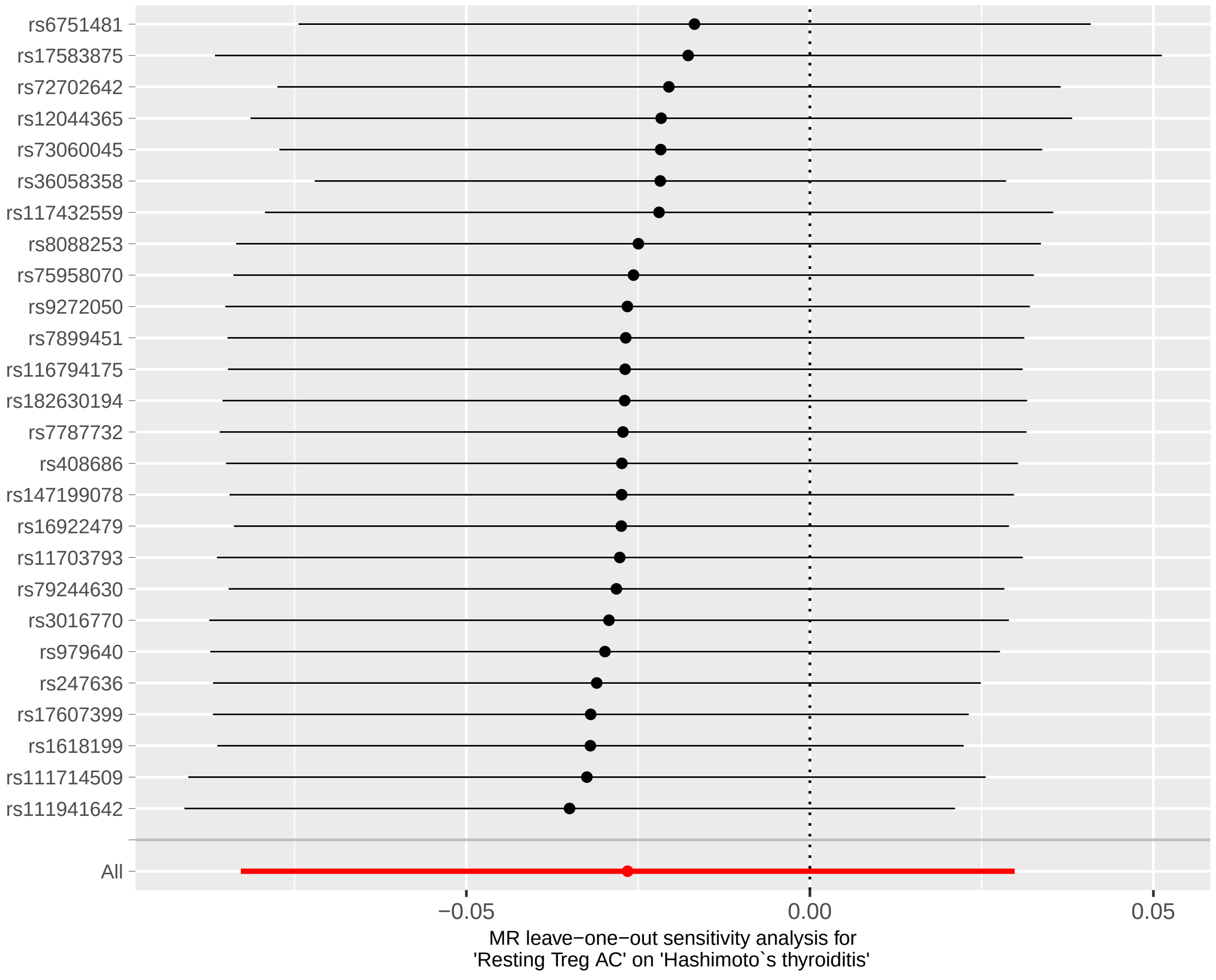
rs6751481
rs17583875
rs72702642
rs12044365
rs73060045
rs36058358
rs117432559
rs8088253
rs75958070
rs9272050
rs7899451
rs116794175
rs182630194
rs7787732
rs408686
rs147199078
rs16922479
rs11703793
rs79244630
rs3016770
rs979640
rs247636
rs17607399
rs1618199
rs111714509
rs111941642
All
−0.05
0.00
0.05
MR leave−one−out sensitivity analysis for
'Resting Treg AC' on 'Hashimoto`s thyroiditis'

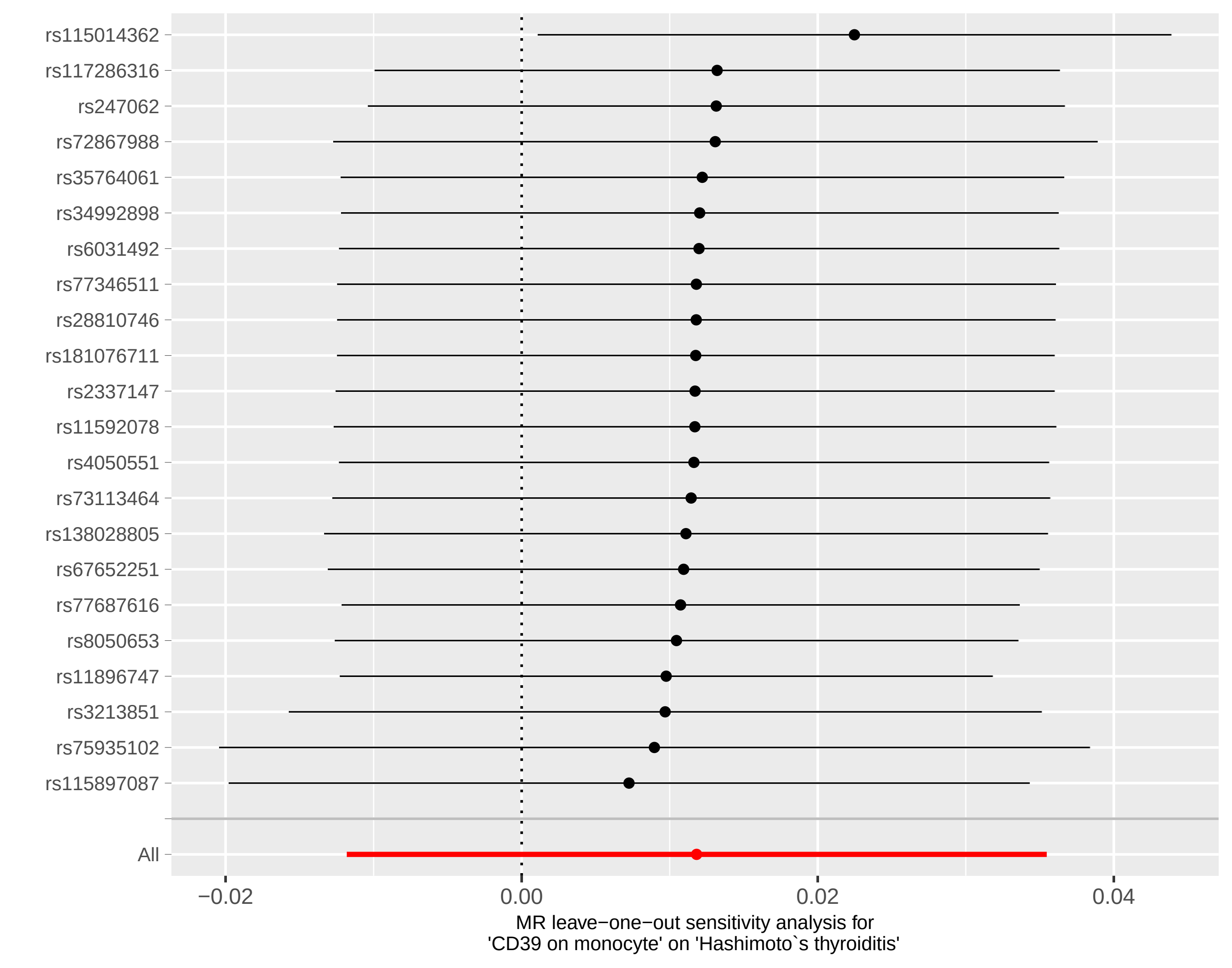
rs115014362
rs117286316
rs247062
rs72867988
rs35764061
rs34992898
rs6031492
rs77346511
rs28810746
rs181076711
rs2337147
rs11592078
rs4050551
rs73113464
rs138028805
rs67652251
rs77687616
rs8050653
rs11896747
rs3213851
rs75935102
rs115897087
All
−0.02
0.00
0.02
0.04
MR leave−one−out sensitivity analysis for
'CD39 on monocyte' on 'Hashimoto`s thyroiditis'

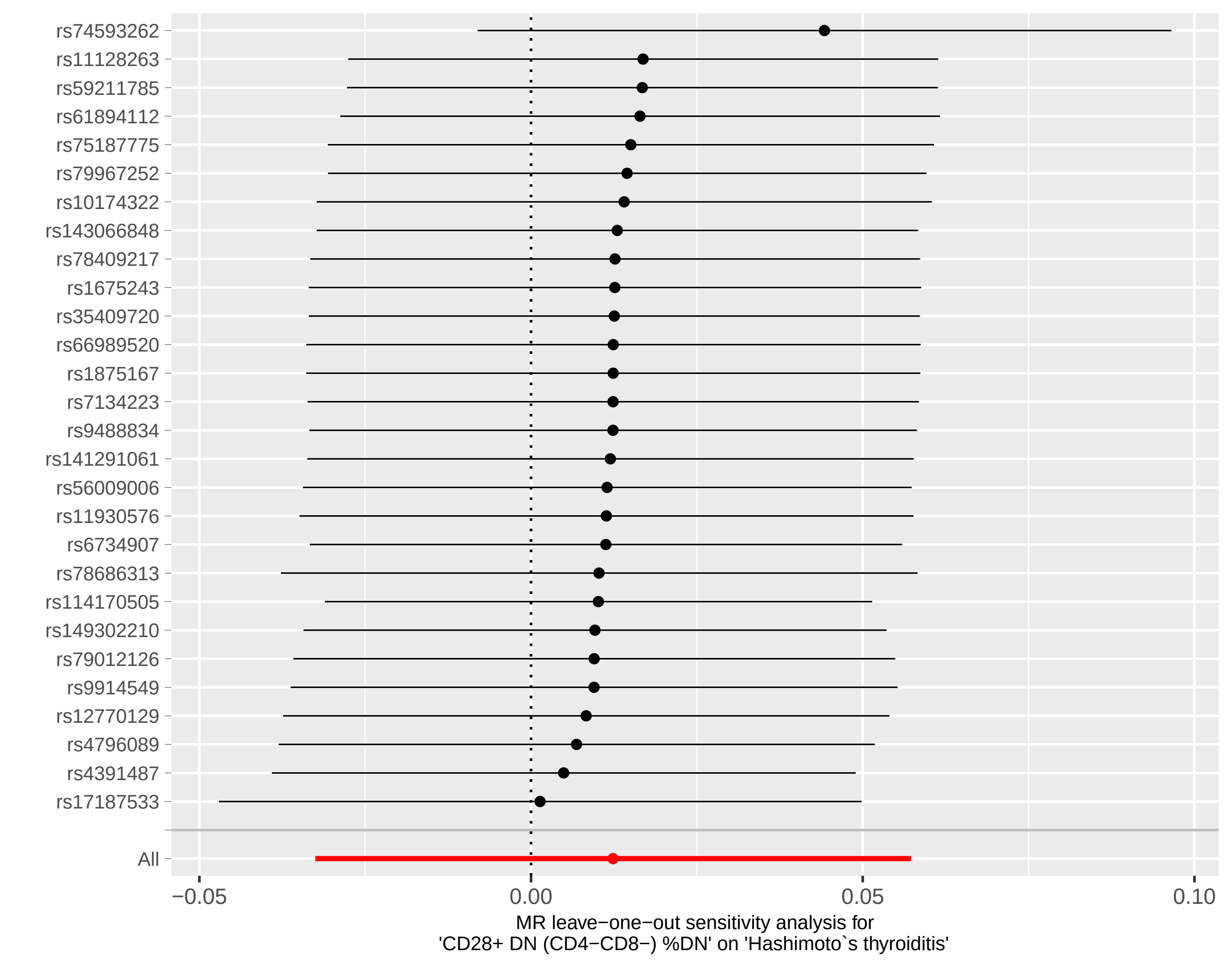
rs74593262
rs11128263
rs59211785
rs61894112
rs75187775
rs79967252
rs10174322
rs143066848
rs78409217
rs1675243
rs35409720
rs66989520
rs1875167
rs7134223
rs9488834
rs141291061
rs56009006
rs11930576
rs6734907
rs78686313
rs114170505
rs149302210
rs79012126
rs9914549
rs12770129
rs4796089
rs4391487
rs17187533
All
−0.05
0.00
0.05
0.10
MR leave−one−out sensitivity analysis for
'CD28+ DN (CD4−CD8−) %DN' on 'Hashimoto`s thyroiditis'

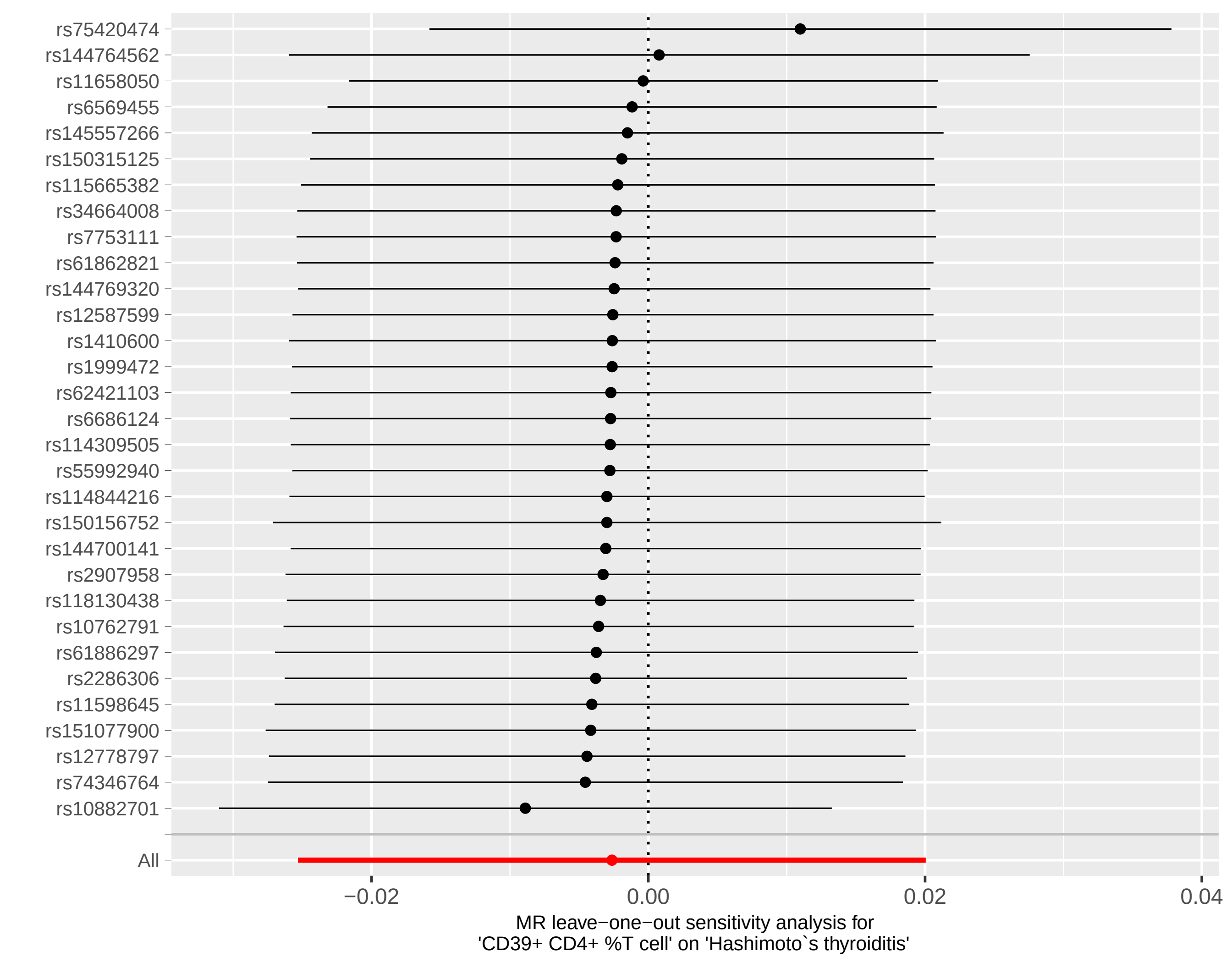
rs75420474
rs144764562
rs11658050
rs6569455
rs145557266
rs150315125
rs115665382
rs34664008
rs7753111
rs61862821
rs144769320
rs12587599
rs1410600
rs1999472
rs62421103
rs6686124
rs114309505
rs55992940
rs114844216
rs150156752
rs144700141
rs2907958
rs118130438
rs10762791
rs61886297
rs2286306
rs11598645
rs151077900
rs12778797
rs74346764
rs10882701
All
−0.02
0.00
0.02
0.04
MR leave−one−out sensitivity analysis for
'CD39+ CD4+ %T cell' on 'Hashimoto`s thyroiditis'

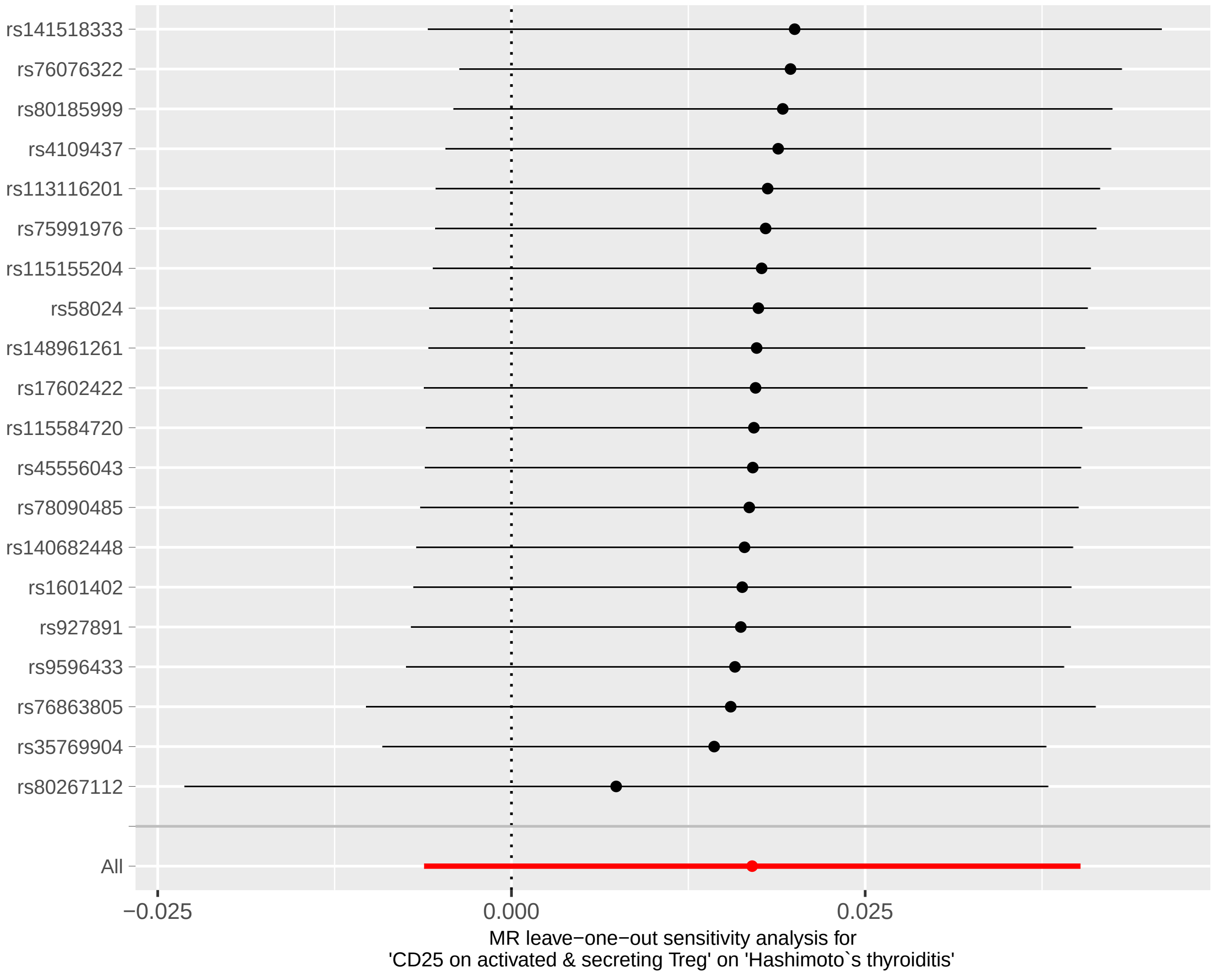
rs141518333
rs76076322
rs80185999
rs4109437
rs113116201
rs75991976
rs115155204
rs58024
rs148961261
rs17602422
rs115584720
rs45556043
rs78090485
rs140682448
rs1601402
rs927891
rs9596433
rs76863805
rs35769904
rs80267112
All
−0.025
0.000
0.025
MR leave−one−out sensitivity analysis for
'CD25 on activated & secreting Treg' on 'Hashimoto`s thyroiditis'

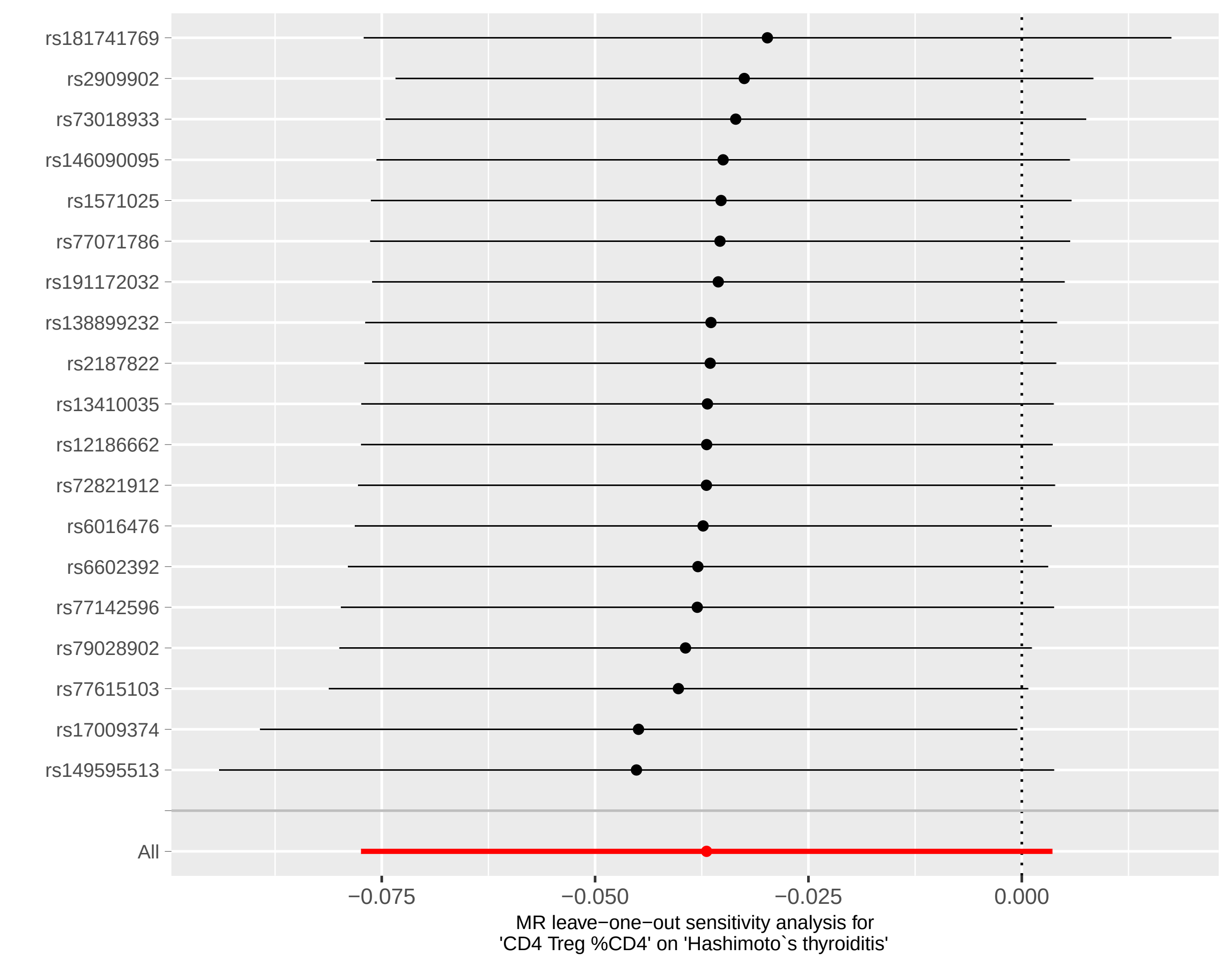
rs181741769
rs2909902
rs73018933
rs146090095
rs1571025
rs77071786
rs191172032
rs138899232
rs2187822
rs13410035
rs12186662
rs72821912
rs6016476
rs6602392
rs77142596
rs79028902
rs77615103
rs17009374
rs149595513
All
−0.075
−0.050
−0.025
0.000
MR leave−one−out sensitivity analysis for
'CD4 Treg %CD4' on 'Hashimoto`s thyroiditis'

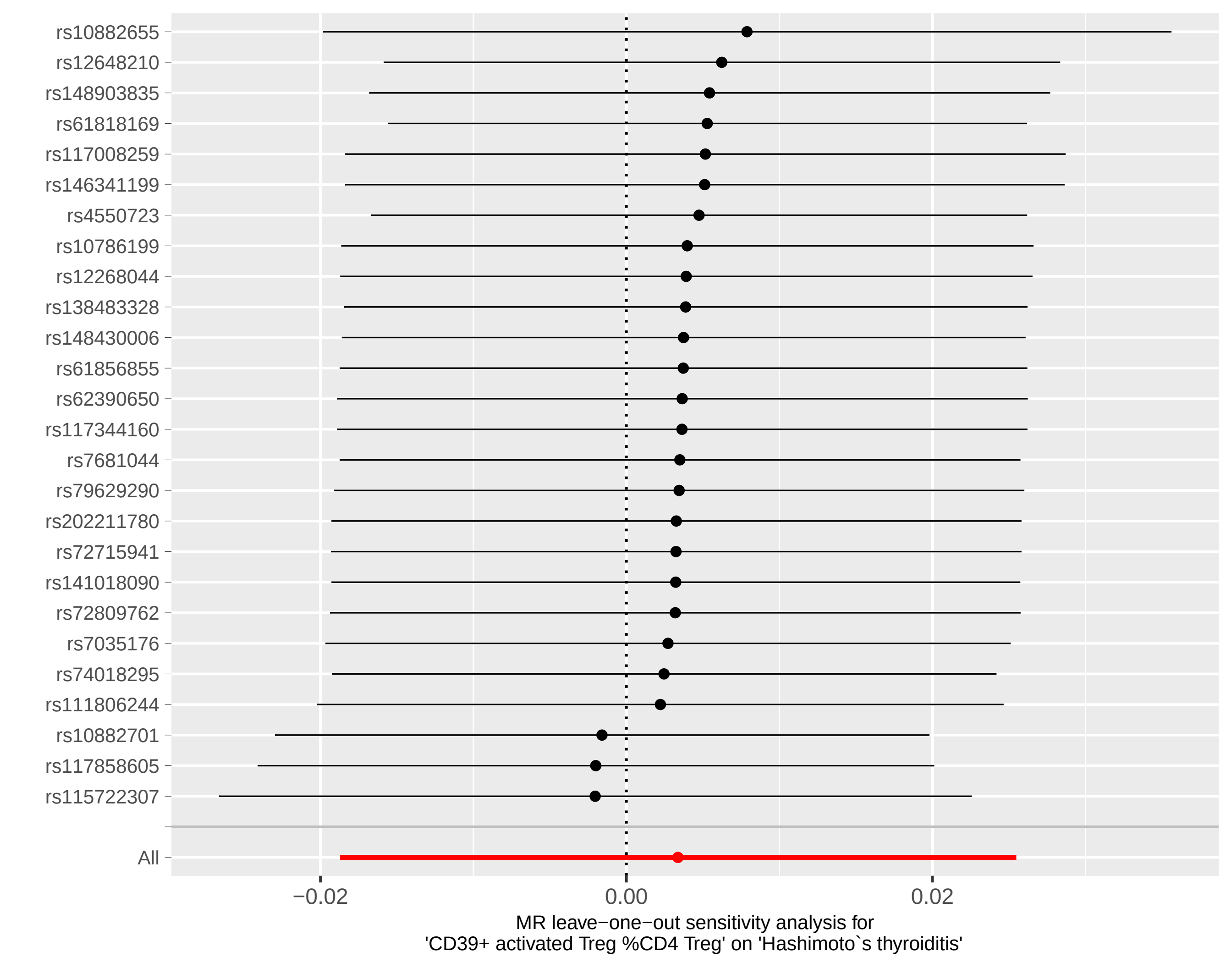

rs10882655
rs12648210
rs148903835
rs61818169
rs117008259
rs146341199
rs4550723
rs10786199
rs12268044
rs138483328
rs148430006
rs61856855
rs62390650
rs117344160
rs7681044
rs79629290
rs202211780
rs72715941
rs141018090
rs72809762
rs7035176
rs74018295
rs111806244
rs10882701
rs117858605
rs115722307
All
−0.02
0.00
0.02
MR leave−one−out sensitivity analysis for
'CD39+ activated Treg %CD4 Treg' on 'Hashimoto`s thyroiditis'

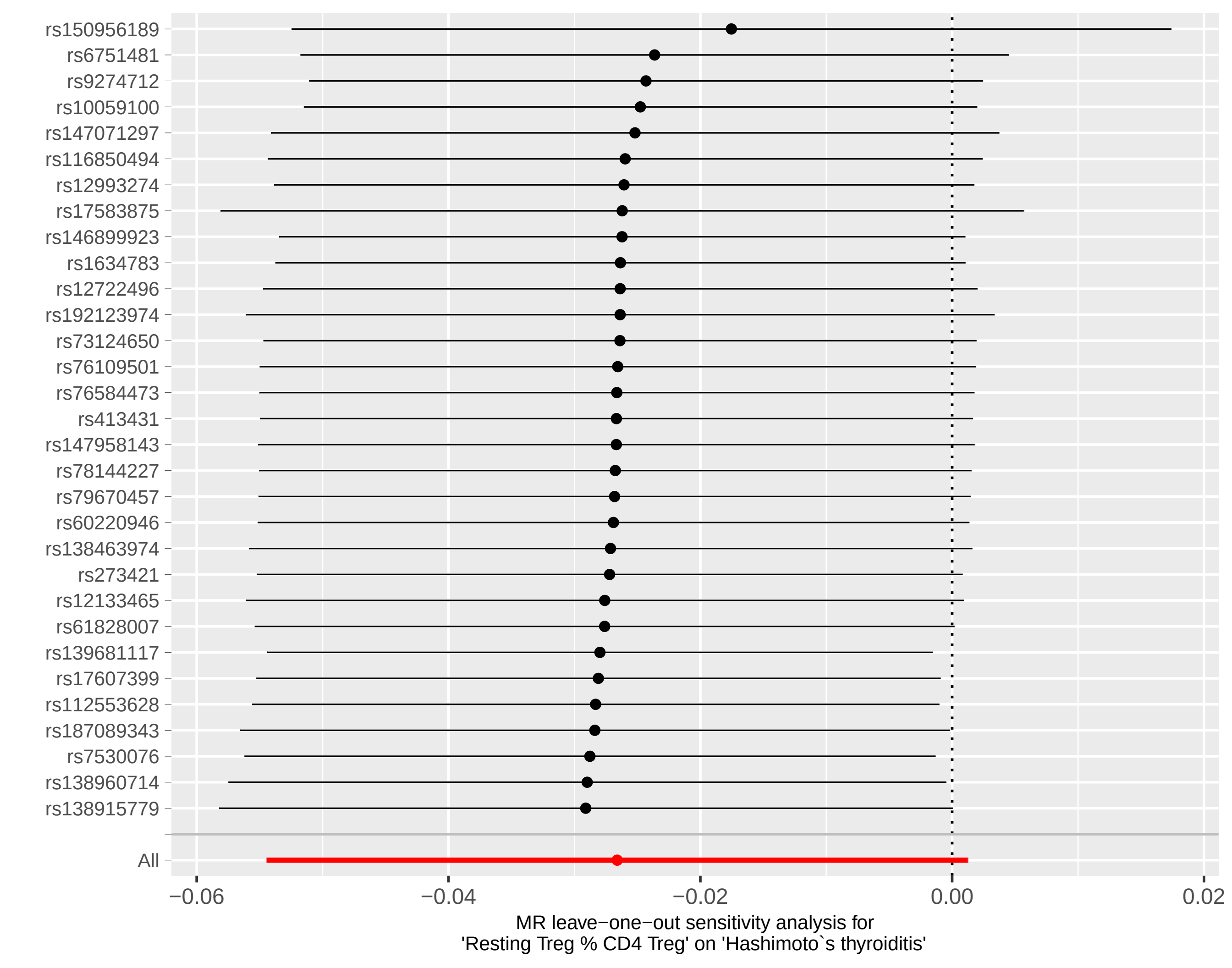
rs150956189
rs6751481
rs9274712
rs10059100
rs147071297
rs116850494
rs12993274
rs17583875
rs146899923
rs1634783
rs12722496
rs192123974
rs73124650
rs76109501
rs76584473
rs413431
rs147958143
rs78144227
rs79670457
rs60220946
rs138463974
rs273421
rs12133465
rs61828007
rs139681117
rs17607399
rs112553628
rs187089343
rs7530076
rs138960714
rs138915779
All
−0.06
−0.04
−0.02
0.00
0.02
MR leave−one−out sensitivity analysis for
'Resting Treg % CD4 Treg' on 'Hashimoto`s thyroiditis'

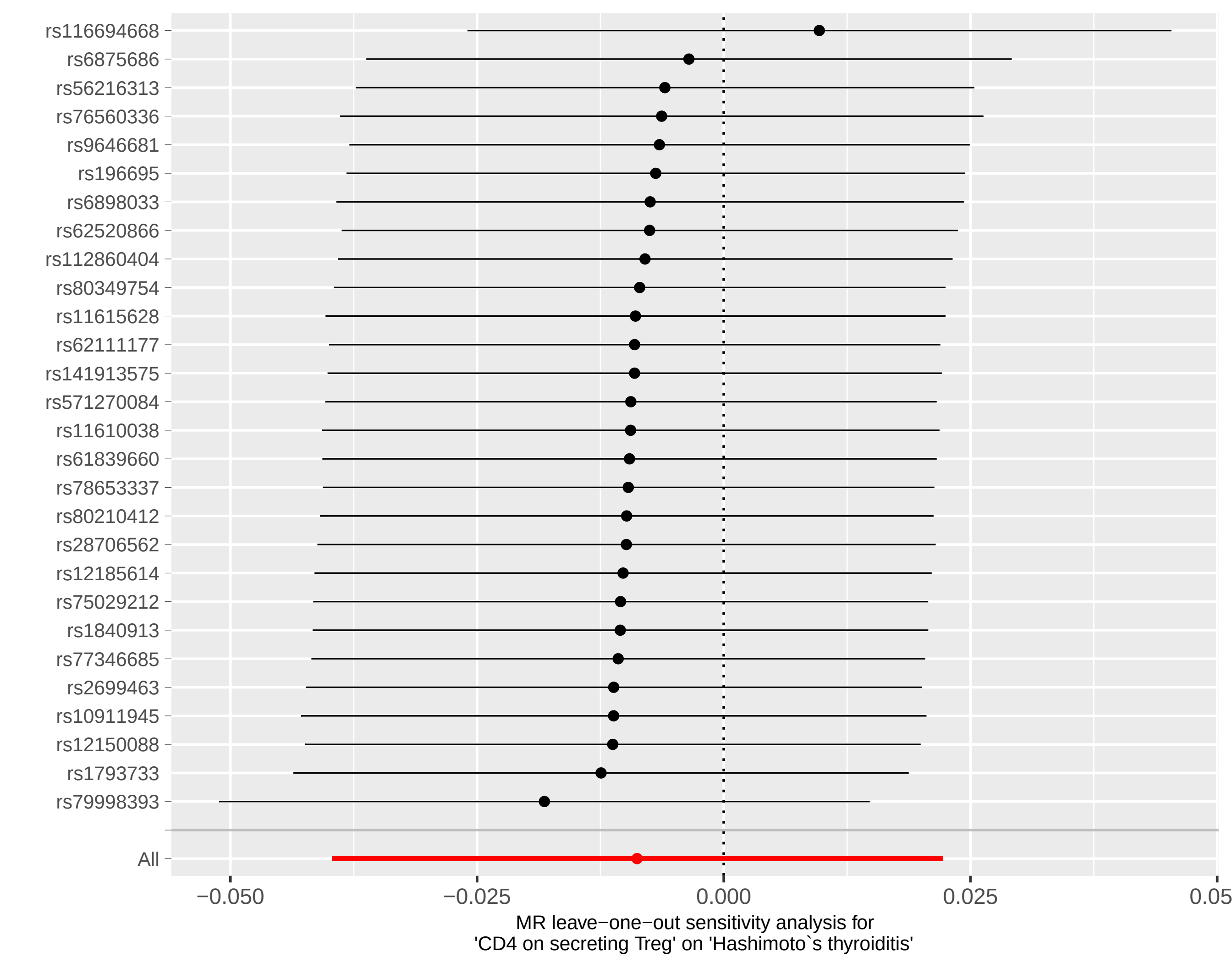

rs116694668
rs6875686
rs56216313
rs76560336
rs9646681
rs196695
rs6898033
rs62520866
rs112860404
rs80349754
rs11615628
rs62111177
rs141913575
rs571270084
rs11610038
rs61839660
rs78653337
rs80210412
rs28706562
rs12185614
rs75029212
rs1840913
rs77346685
rs2699463
rs10911945
rs12150088
rs1793733
rs79998393
All
−0.050
−0.025
0.000
0.025
0.05
MR leave−one−out sensitivity analysis for
'CD4 on secreting Treg' on 'Hashimoto`s thyroiditis'

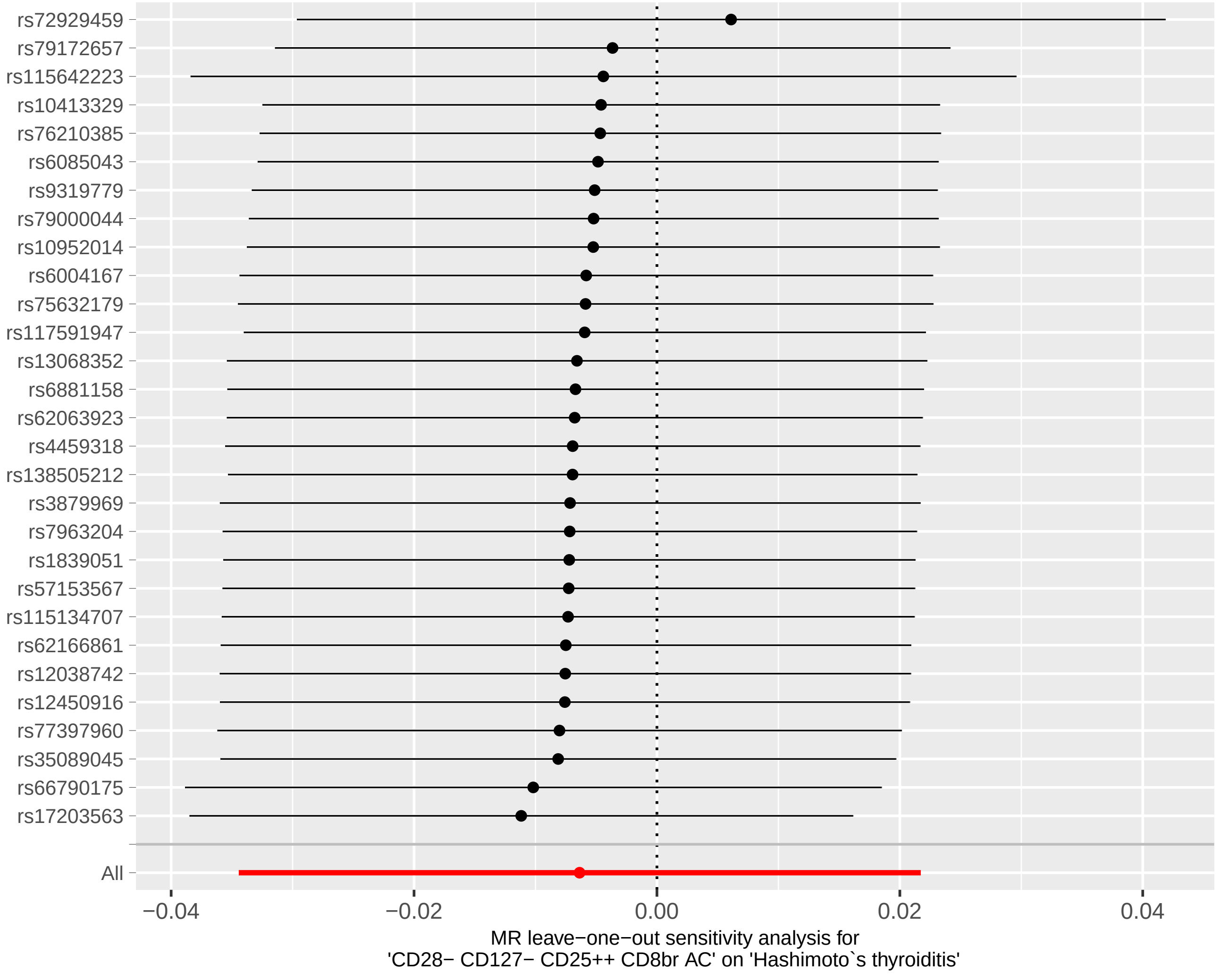
rs72929459
rs79172657
rs115642223
rs10413329
rs76210385
rs6085043
rs9319779
rs79000044
rs10952014
rs6004167
rs75632179
rs117591947
rs13068352
rs6881158
rs62063923
rs4459318
rs138505212
rs3879969
rs7963204
rs1839051
rs57153567
rs115134707
rs62166861
rs12038742
rs12450916
rs77397960
rs35089045
rs66790175
rs17203563
All
−0.04
−0.02
0.00
0.02
0.04
MR leave−one−out sensitivity analysis for
'CD28− CD127− CD25++ CD8br AC' on 'Hashimoto`s thyroiditis'

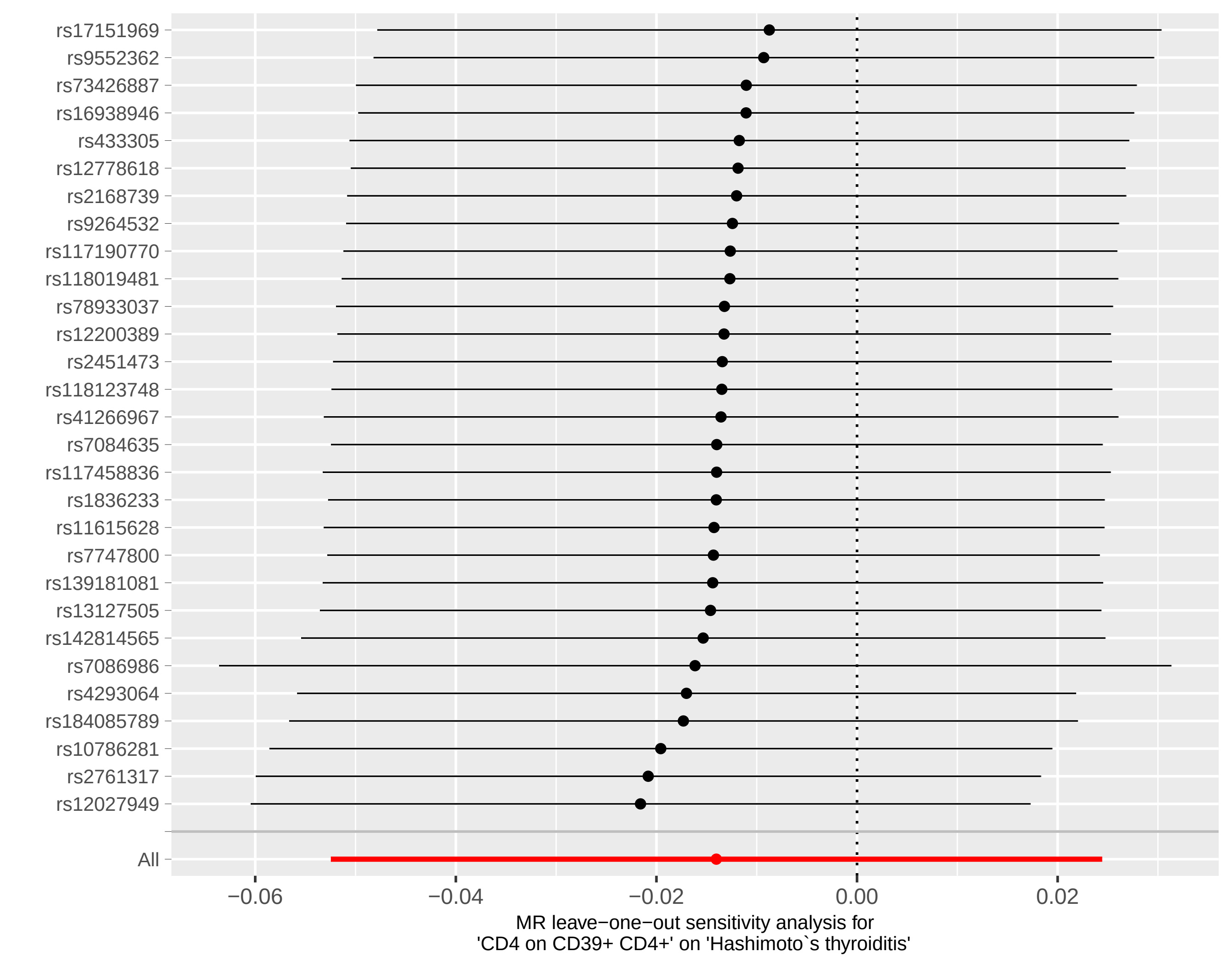

rs17151969
rs9552362
rs73426887
rs16938946
rs433305
rs12778618
rs2168739
rs9264532
rs117190770
rs118019481
rs78933037
rs12200389
rs2451473
rs118123748
rs41266967
rs7084635
rs117458836
rs1836233
rs11615628
rs7747800
rs139181081
rs13127505
rs142814565
rs7086986
rs4293064
rs184085789
rs10786281
rs2761317
rs12027949
All
−0.06
−0.04
−0.02
0.00
0.02
MR leave−one−out sensitivity analysis for
'CD4 on CD39+ CD4+' on 'Hashimoto`s thyroiditis'

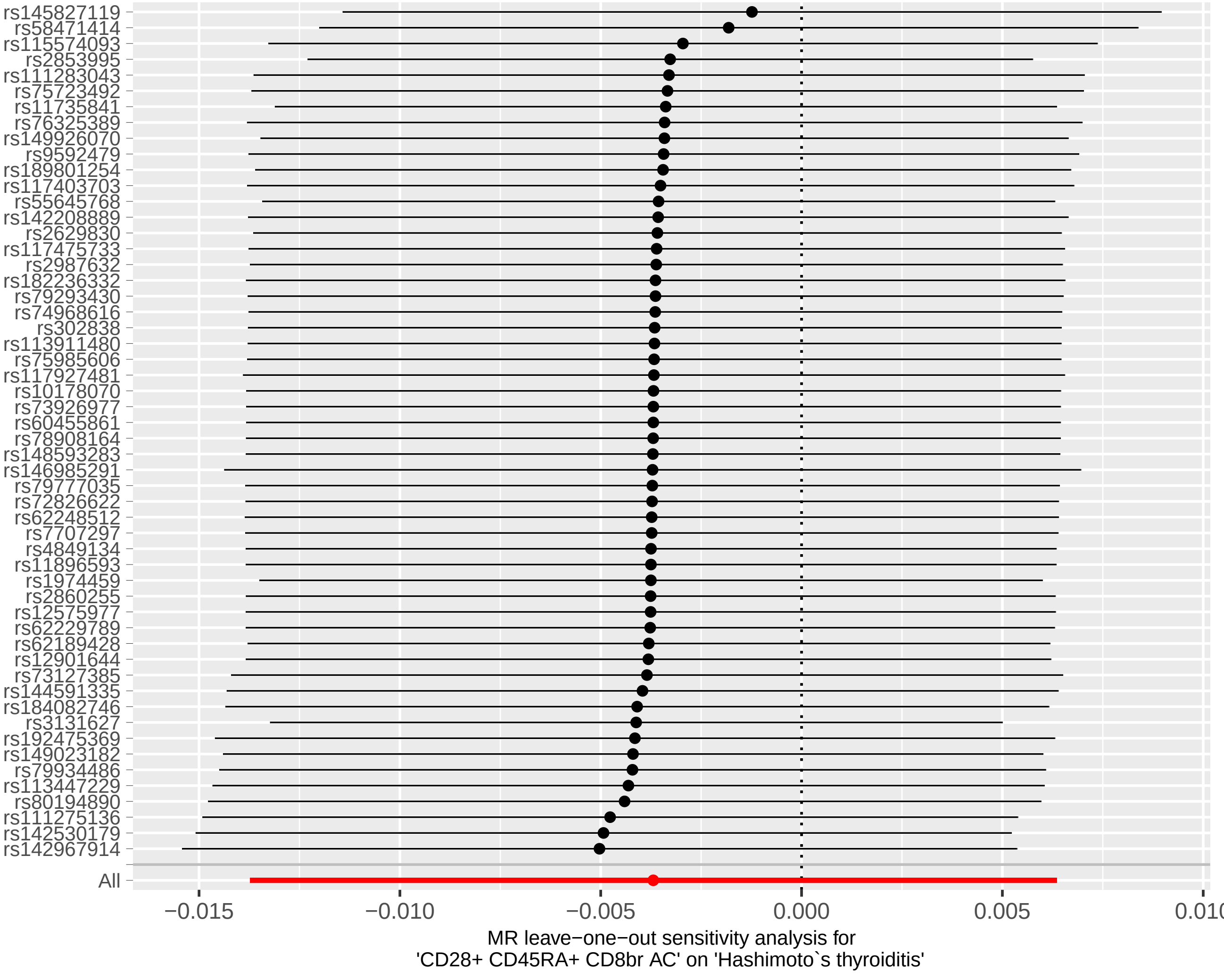
rs145827119
rs58471414
rs115574093
rs2853995
rs111283043
rs75723492
rs11735841
rs76325389
rs149926070
rs9592479
rs189801254
rs117403703
rs55645768
rs142208889
rs2629830
rs117475733
rs2987632
rs182236332
rs79293430
rs74968616
rs302838
rs113911480
rs75985606
rs117927481
rs10178070
rs73926977
rs60455861
rs78908164
rs148593283
rs146985291
rs79777035
rs72826622
rs62248512
rs7707297
rs4849134
rs11896593
rs1974459
rs2860255
rs12575977
rs62229789
rs62189428
rs12901644
rs73127385
rs144591335
rs184082746
rs3131627
rs192475369
rs149023182
rs79934486
rs113447229
rs80194890
rs111275136
rs142530179
rs142967914
All
−0.015
−0.010
−0.005
0.000
0.005
MR leave−one−out sensitivity analysis for
'CD28+ CD45RA+ CD8br AC' on 'Hashimoto`s thyroiditis'

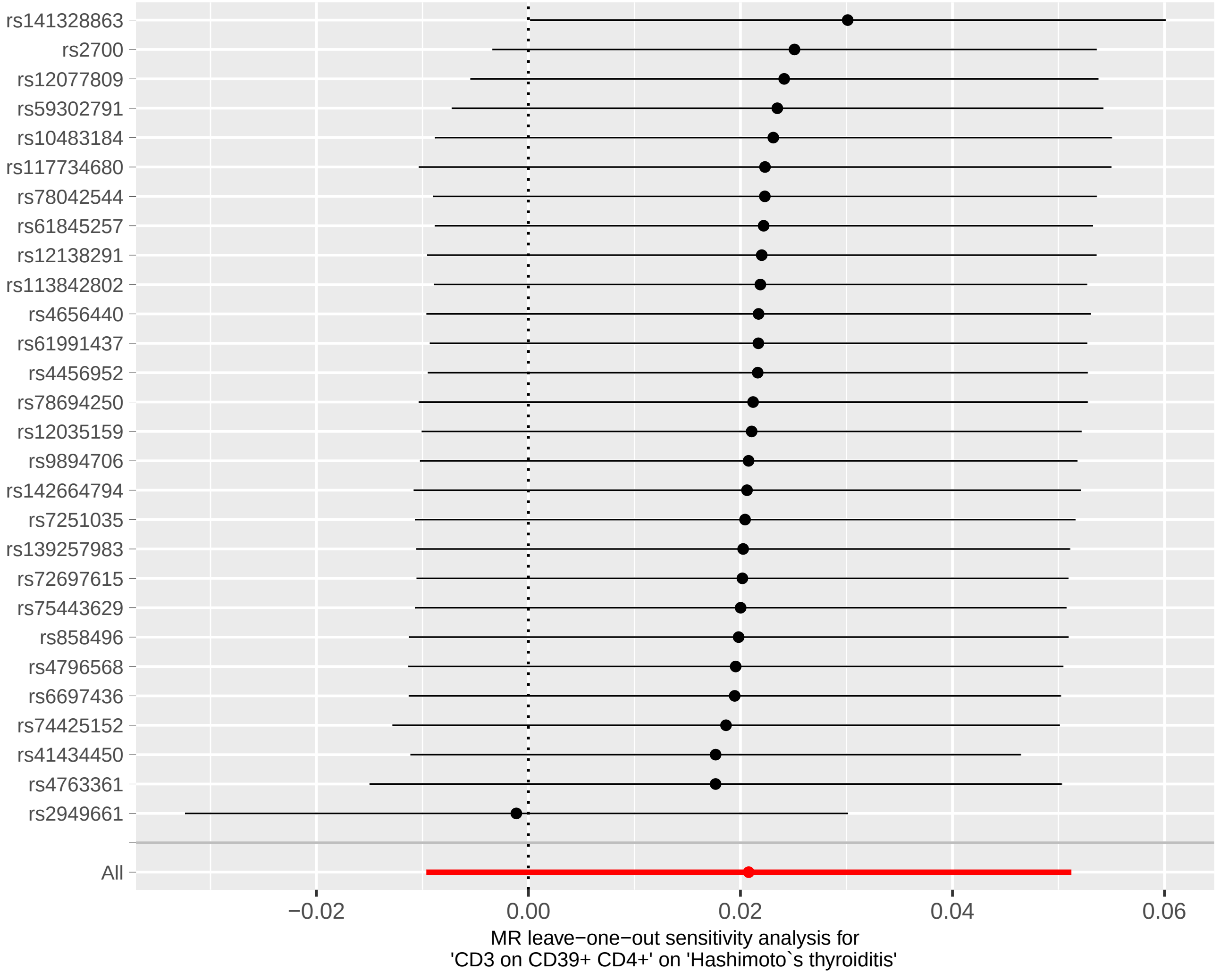

rs141328863
rs2700
rs12077809
rs59302791
rs10483184
rs117734680
rs78042544
rs61845257
rs12138291
rs113842802
rs4656440
rs61991437
rs4456952
rs78694250
rs12035159
rs9894706
rs142664794
rs7251035
rs139257983
rs72697615
rs75443629
rs858496
rs4796568
rs6697436
rs74425152
rs41434450
rs4763361
rs2949661
All
−0.02
0.00
0.02
0.04
0.06
MR leave−one−out sensitivity analysis for
'CD3 on CD39+ CD4+' on 'Hashimoto`s thyroiditis'

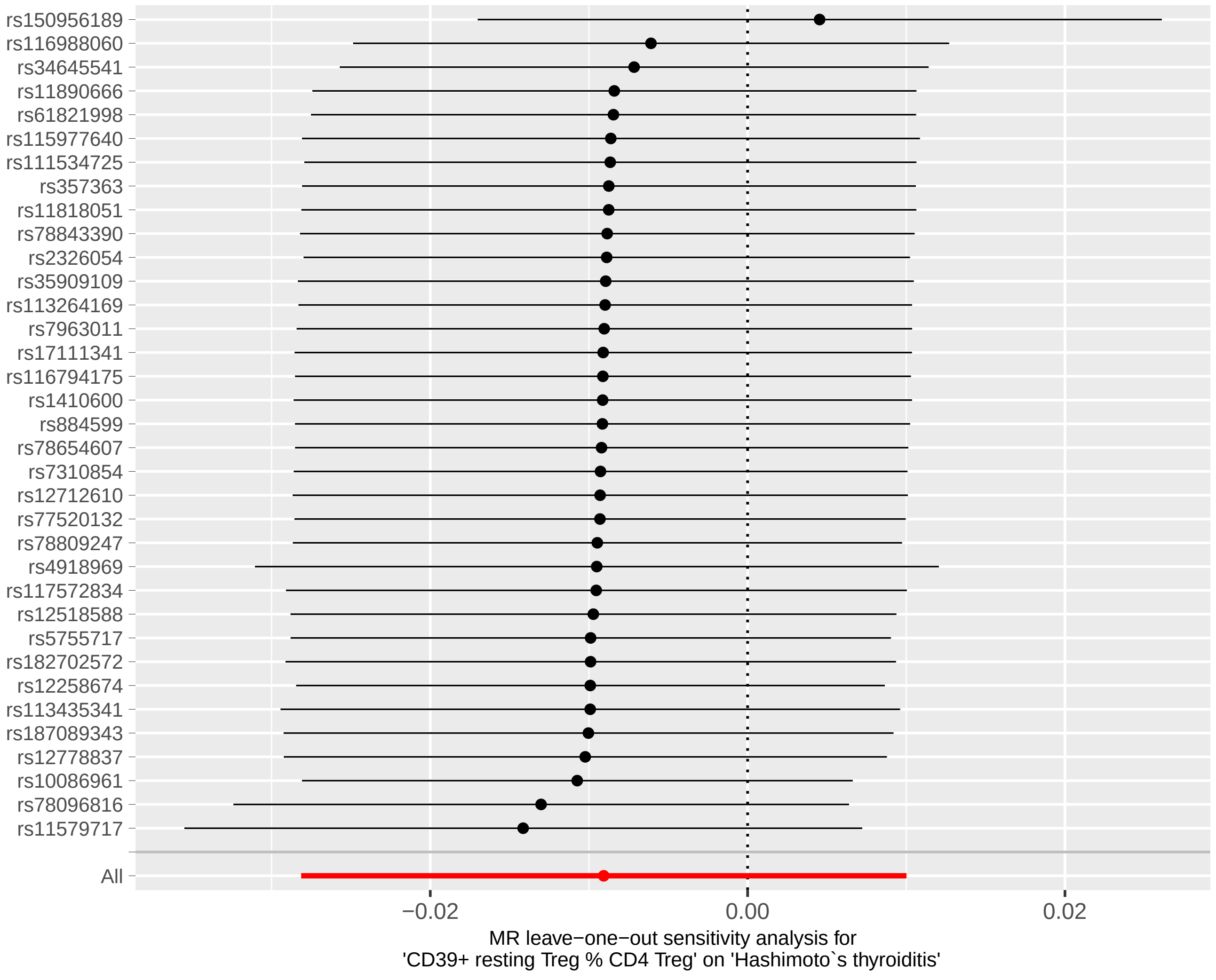

rs150956189
rs116988060
rs34645541
rs11890666
rs61821998
rs115977640
rs111534725
rs357363
rs11818051
rs78843390
rs2326054
rs35909109
rs113264169
rs7963011
rs17111341
rs116794175
rs1410600
rs884599
rs78654607
rs7310854
rs12712610
rs77520132
rs78809247
rs4918969
rs117572834
rs12518588
rs5755717
rs182702572
rs12258674
rs113435341
rs187089343
rs12778837
rs10086961
rs78096816
rs11579717
All
−0.02
0.00
0.02
MR leave−one−out sensitivity analysis for
'CD39+ resting Treg % CD4 Treg' on 'Hashimoto`s thyroiditis'

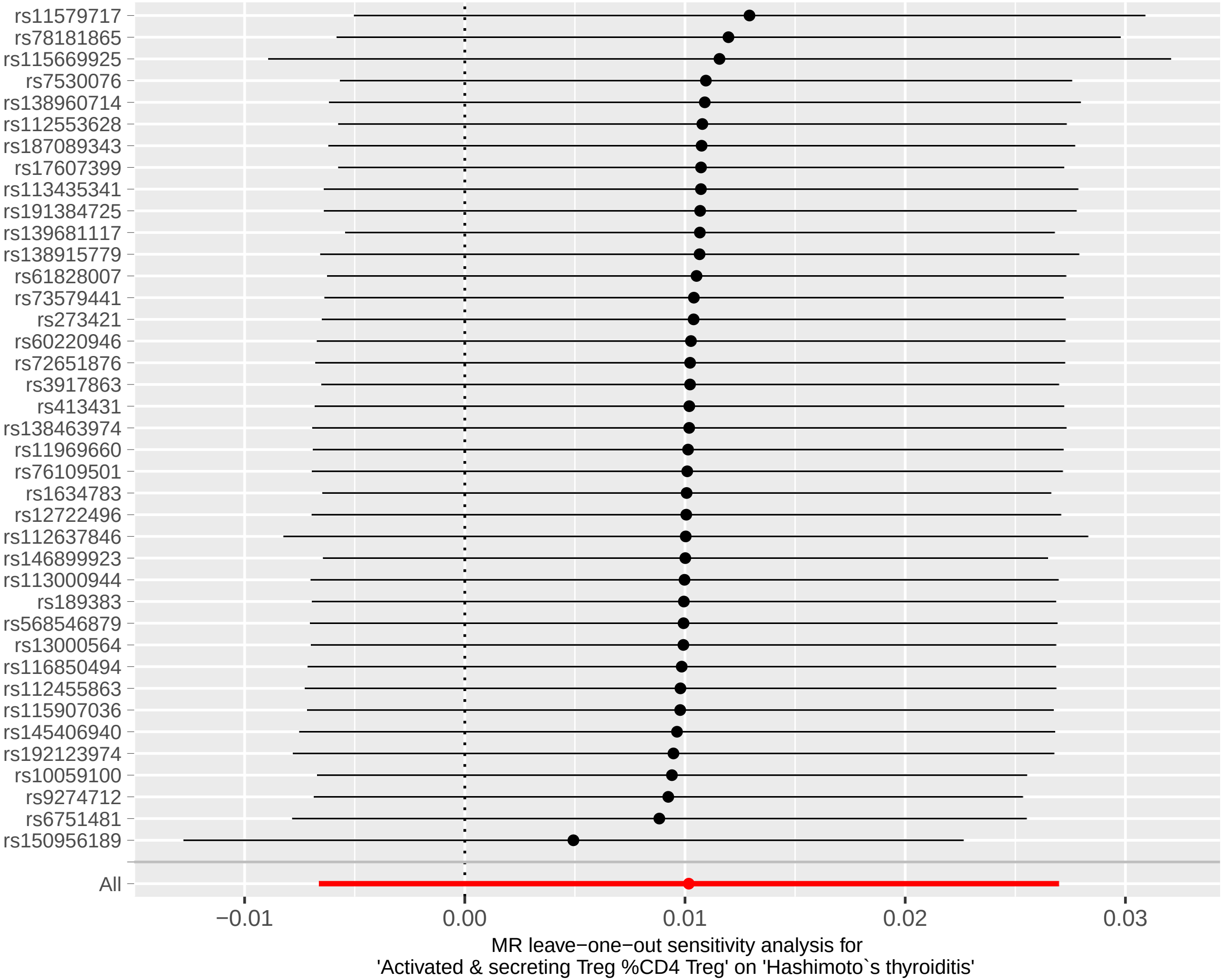

rs11579717
rs78181865
rs115669925
rs7530076
rs138960714
rs112553628
rs187089343
rs17607399
rs113435341
rs191384725
rs139681117
rs138915779
rs61828007
rs73579441
rs273421
rs60220946
rs72651876
rs3917863
rs413431
rs138463974
rs11969660
rs76109501
rs1634783
rs12722496
rs112637846
rs146899923
rs113000944
rs189383
rs568546879
rs13000564
rs116850494
rs112455863
rs115907036
rs145406940
rs192123974
rs10059100
rs9274712
rs6751481
rs150956189
All
−0.01
0.00
0.01
0.02
0.03
MR leave−one−out sensitivity analysis for
'Activated & secreting Treg %CD4 Treg' on 'Hashimoto`s thyroiditis'

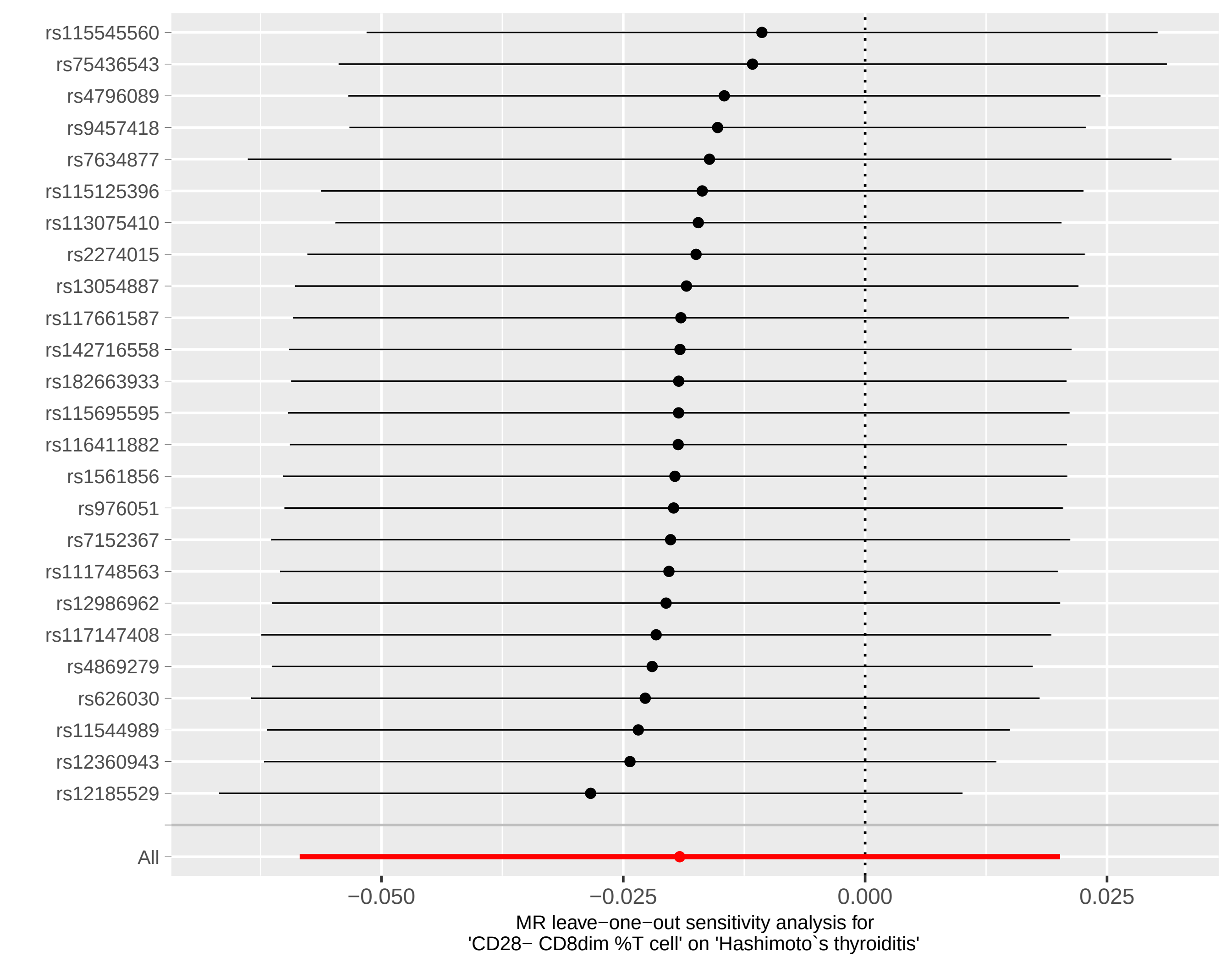
rs115545560
rs75436543
rs4796089
rs9457418
rs7634877
rs115125396
rs113075410
rs2274015
rs13054887
rs117661587
rs142716558
rs182663933
rs115695595
rs116411882
rs1561856
rs976051
rs7152367
rs111748563
rs12986962
rs117147408
rs4869279
rs626030
rs11544989
rs12360943
rs12185529
All
−0.050
−0.025
0.000
0.025
MR leave−one−out sensitivity analysis for
'CD28− CD8dim %T cell' on 'Hashimoto`s thyroiditis'

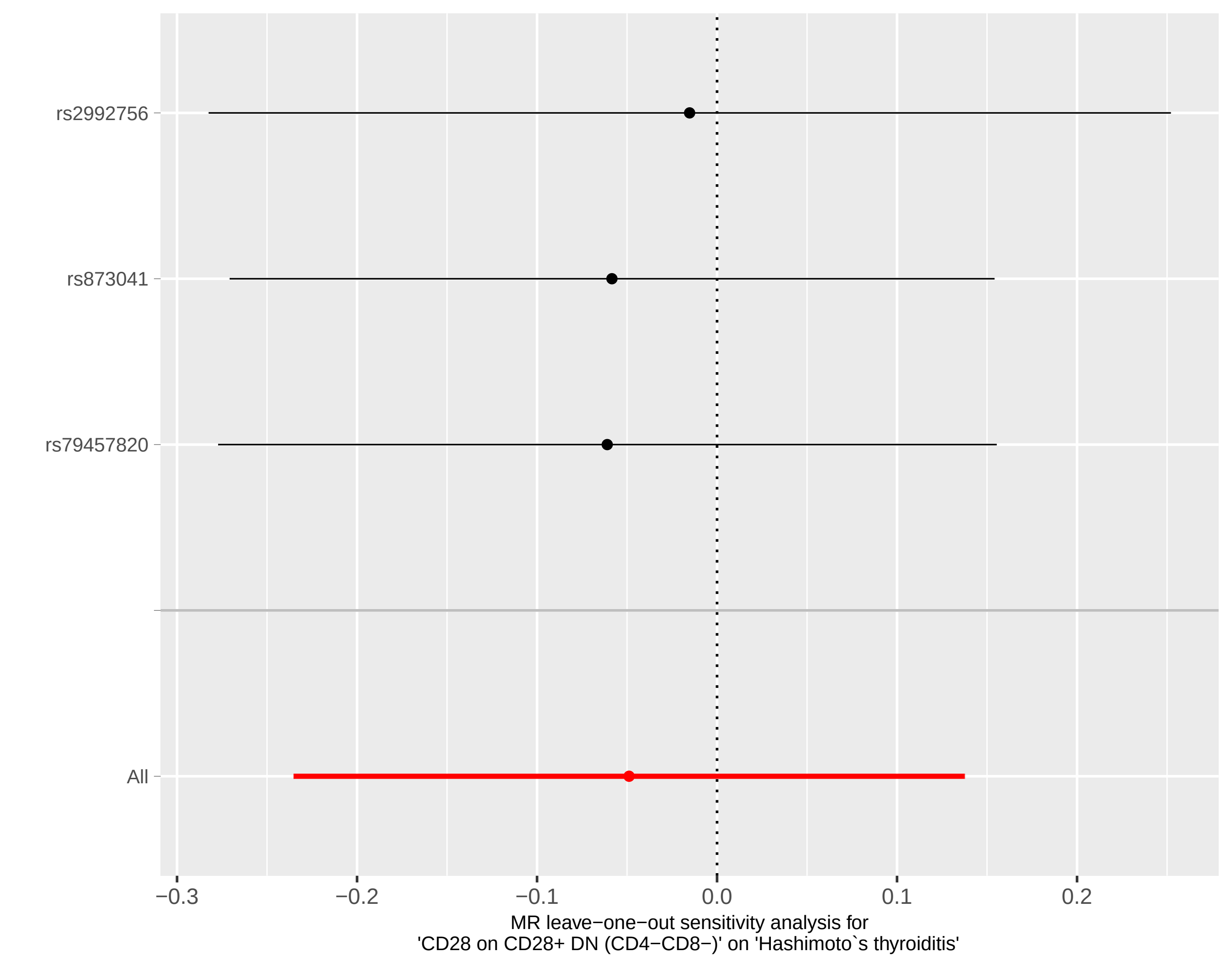
rs2992756
rs873041
rs79457820
All
−0.3
−0.2
−0.1
0.0
0.1
0.2
MR leave−one−out sensitivity analysis for
'CD28 on CD28+ DN (CD4−CD8−)' on 'Hashimoto`s thyroiditis'

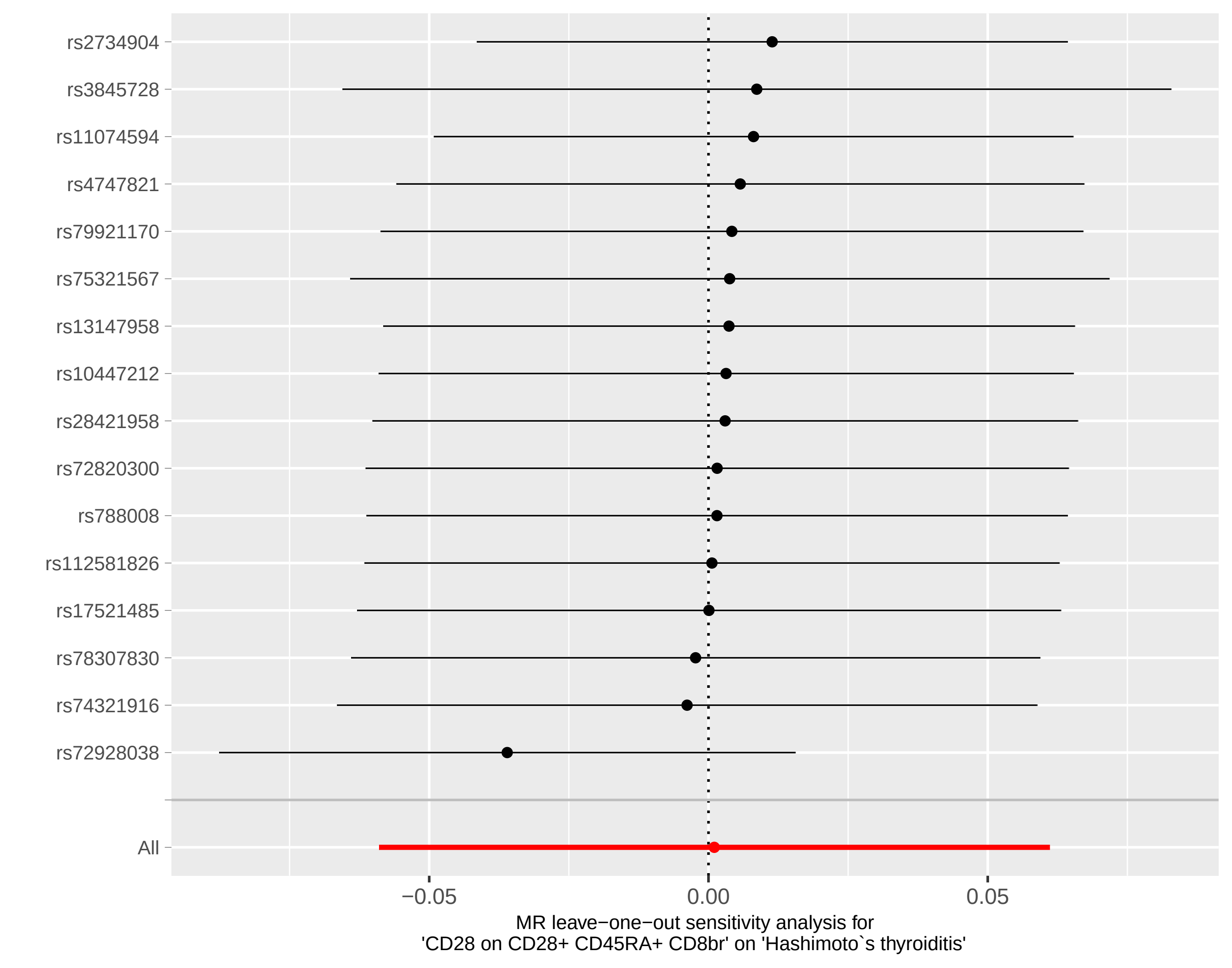

rs2734904
rs3845728
rs11074594
rs4747821
rs79921170
rs75321567
rs13147958
rs10447212
rs28421958
rs72820300
rs788008
rs112581826
rs17521485
rs78307830
rs74321916
rs72928038
All
−0.05
0.00
0.05
MR leave−one−out sensitivity analysis for
'CD28 on CD28+ CD45RA+ CD8br' on 'Hashimoto`s thyroiditis'

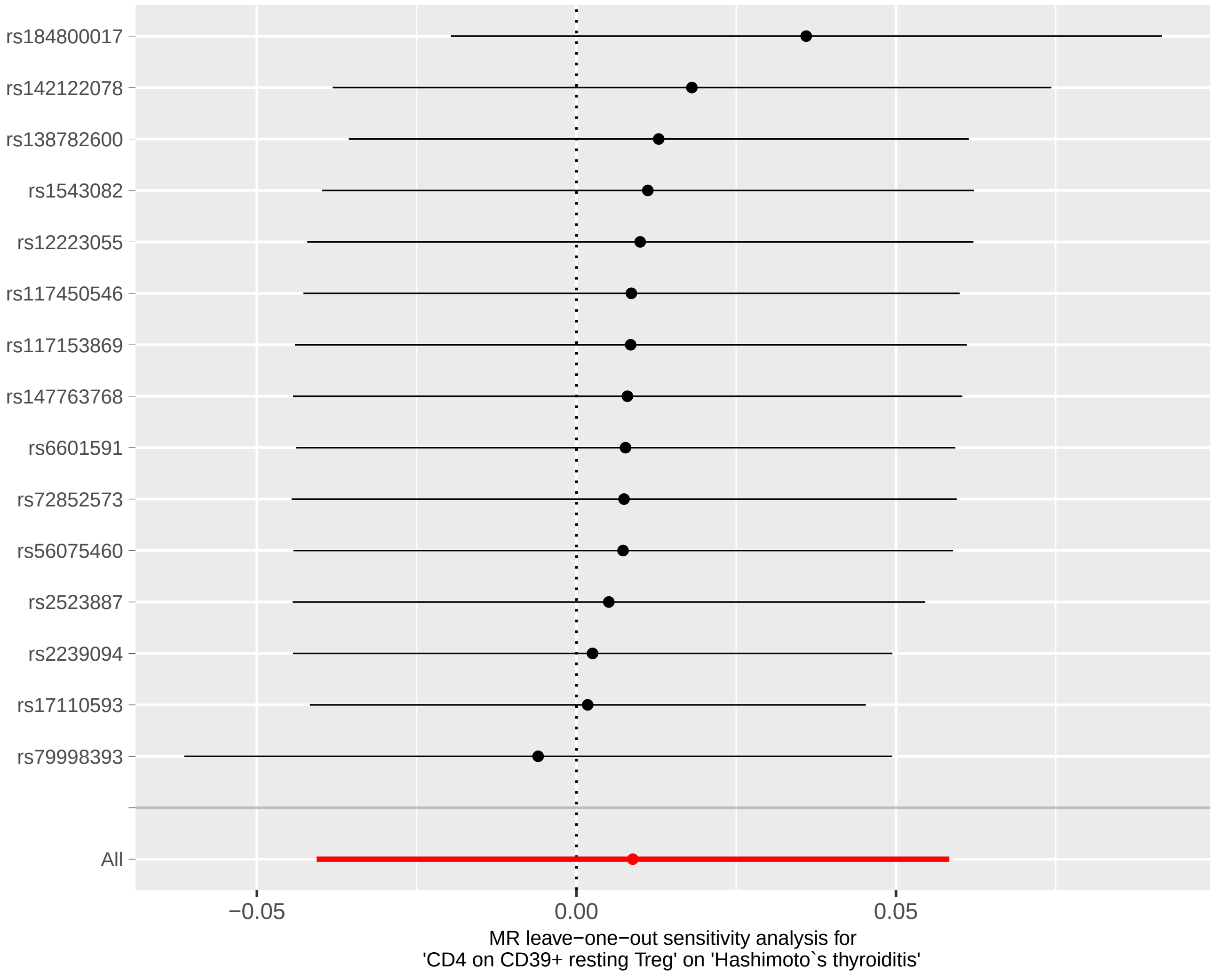

rs184800017
rs142122078
rs138782600
rs1543082
rs12223055
rs117450546
rs117153869
rs147763768
rs6601591
rs72852573
rs56075460
rs2523887
rs2239094
rs17110593
rs79998393
All
−0.05
0.00
0.05
MR leave−one−out sensitivity analysis for
'CD4 on CD39+ resting Treg' on 'Hashimoto`s thyroiditis'

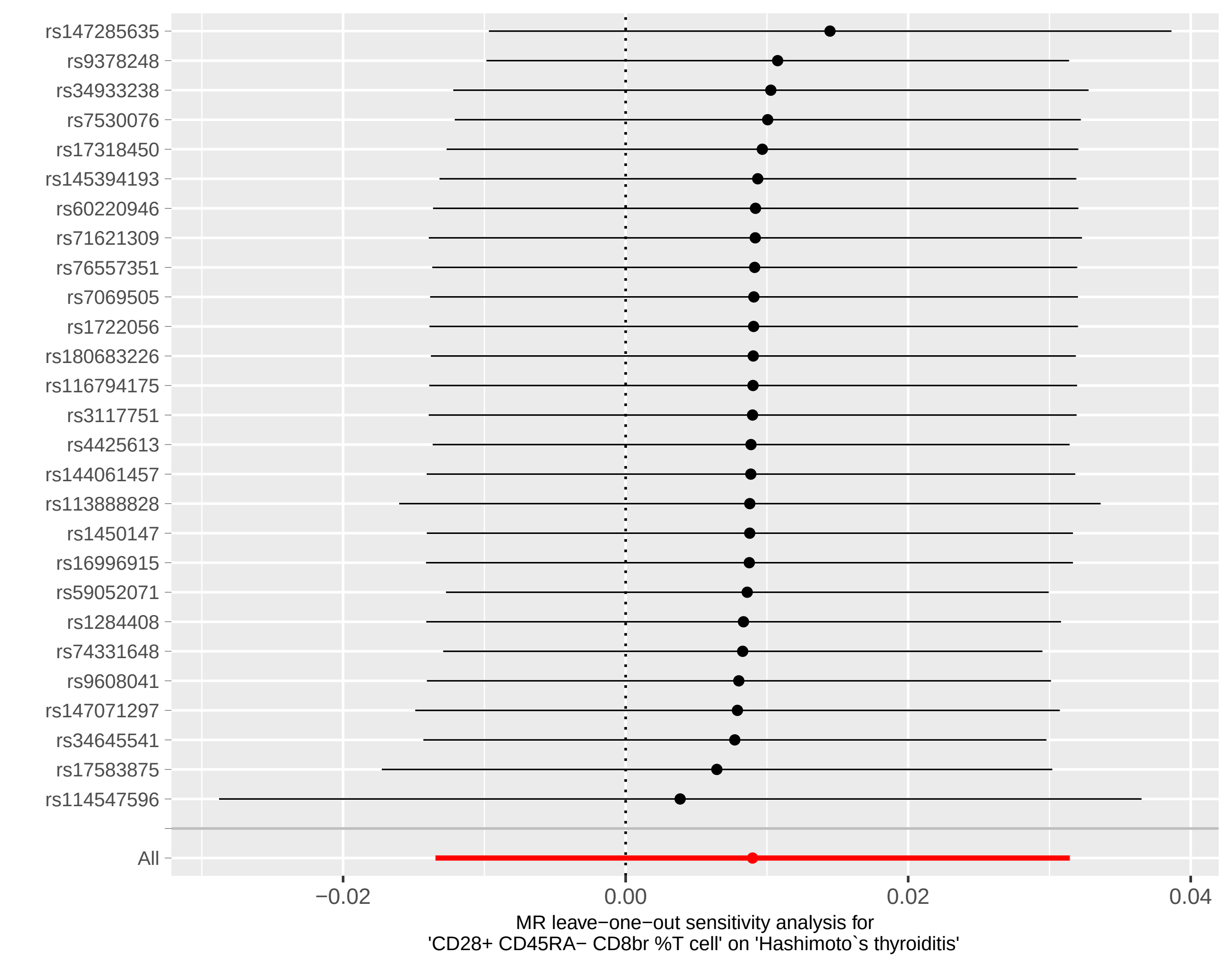
rs147285635
rs9378248
rs34933238
rs7530076
rs17318450
rs145394193
rs60220946
rs71621309
rs76557351
rs7069505
rs1722056
rs180683226
rs116794175
rs3117751
rs4425613
rs144061457
rs113888828
rs1450147
rs16996915
rs59052071
rs1284408
rs74331648
rs9608041
rs147071297
rs34645541
rs17583875
rs114547596
All
−0.02
0.00
0.02
0.04
MR leave−one−out sensitivity analysis for
'CD28+ CD45RA− CD8br %T cell' on 'Hashimoto`s thyroiditis'

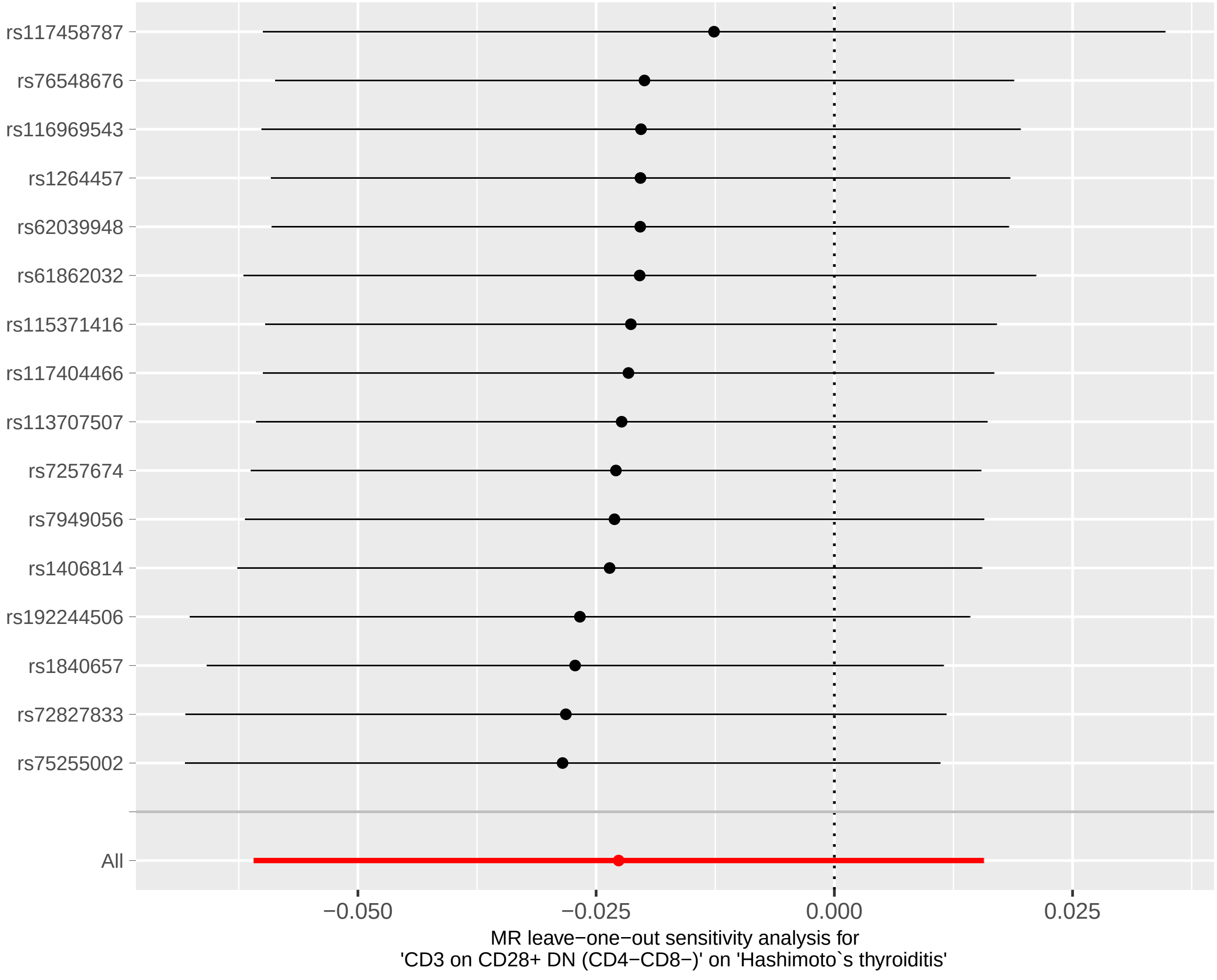
rs117458787
rs76548676
rs116969543
rs1264457
rs62039948
rs61862032
rs115371416
rs117404466
rs113707507
rs7257674
rs7949056
rs1406814
rs192244506
rs1840657
rs72827833
rs75255002
All
−0.050
−0.025
0.000
0.025
MR leave−one−out sensitivity analysis for
'CD3 on CD28+ DN (CD4−CD8−)' on 'Hashimoto`s thyroiditis'

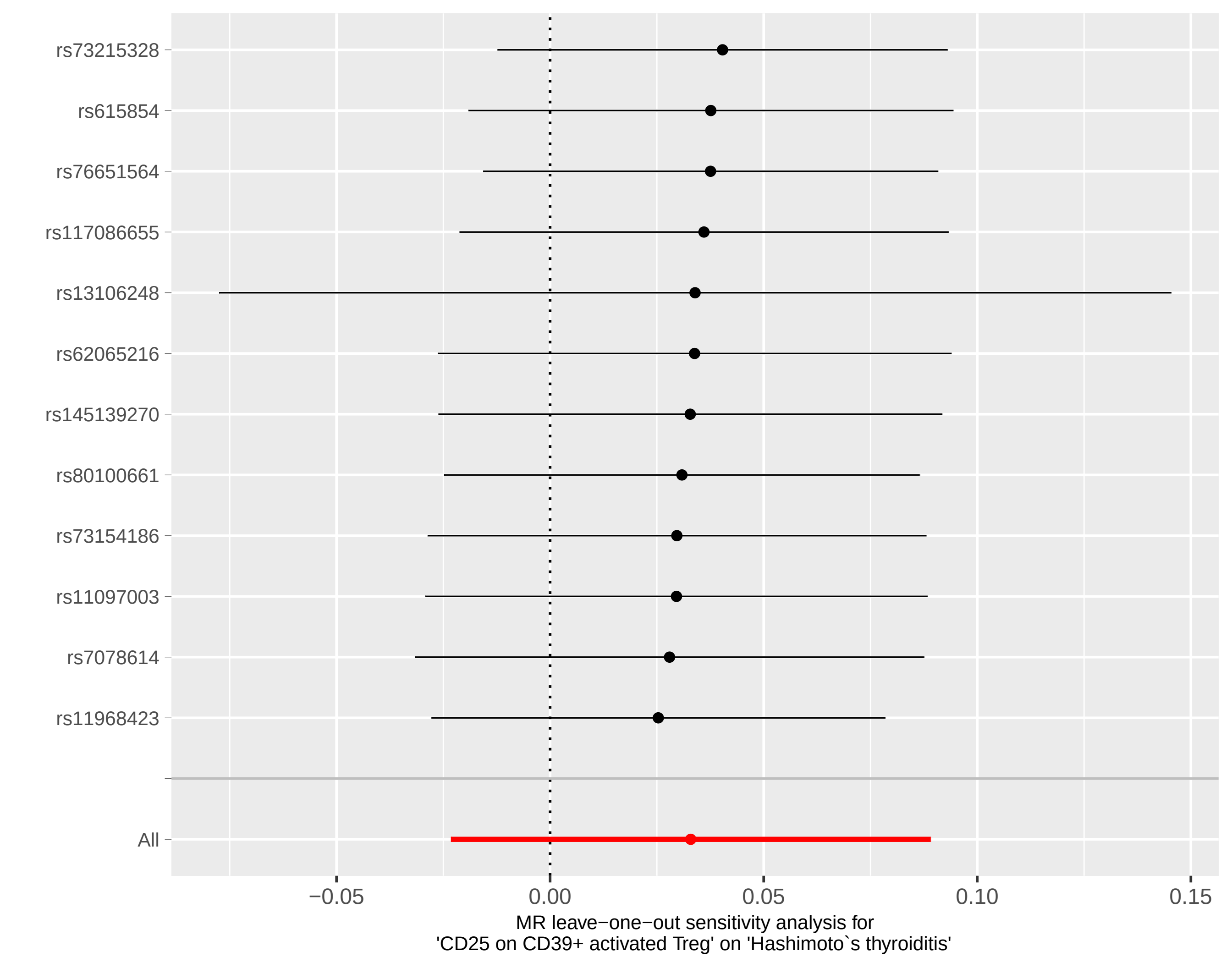
rs73215328
rs615854
rs76651564
rs117086655
rs13106248
rs62065216
rs145139270
rs80100661
rs73154186
rs11097003
rs7078614
rs11968423
All
−0.05
0.00
0.05
0.10
0.15
MR leave−one−out sensitivity analysis for
'CD25 on CD39+ activated Treg' on 'Hashimoto`s thyroiditis'

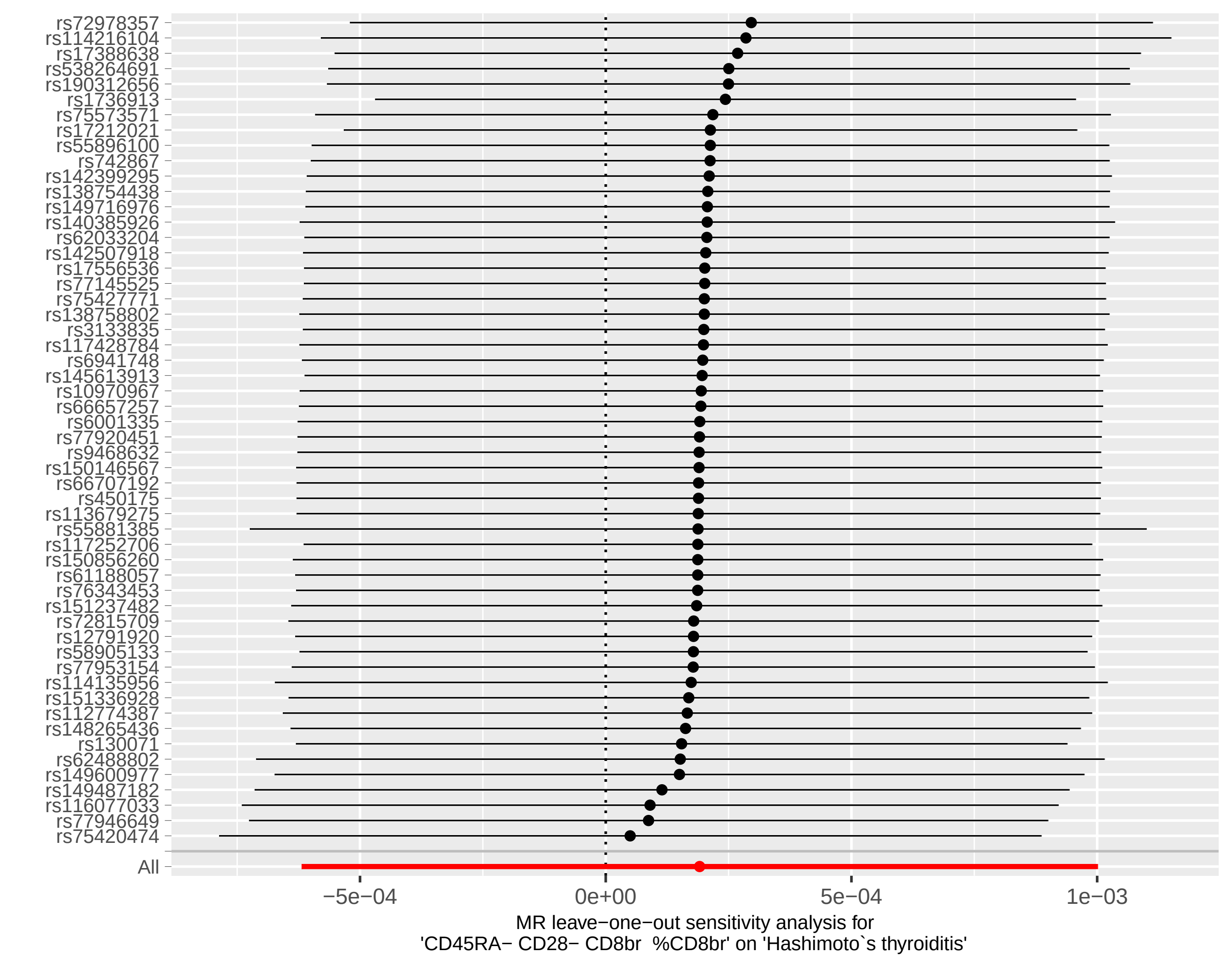

rs72978357
rs114216104
rs17388638
rs538264691
rs190312656
rs1736913
rs75573571
rs17212021
rs55896100
rs742867
rs142399295
rs138754438
rs149716976
rs140385926
rs62033204
rs142507918
rs17556536
rs77145525
rs75427771
rs138758802
rs3133835
rs117428784
rs6941748
rs145613913
rs10970967
rs66657257
rs6001335
rs77920451
rs9468632
rs150146567
rs66707192
rs450175
rs113679275
rs55881385
rs117252706
rs150856260
rs61188057
rs76343453
rs151237482
rs72815709
rs12791920
rs58905133
rs77953154
rs114135956
rs151336928
rs112774387
rs148265436
rs130071
rs62488802
rs149600977
rs149487182
rs116077033
rs77946649
rs75420474
All
−5e−04
0e+00
5e−04
1e−03
MR leave−one−out sensitivity analysis for
'CD45RA− CD28− CD8br %CD8br' on 'Hashimoto`s thyroiditis'

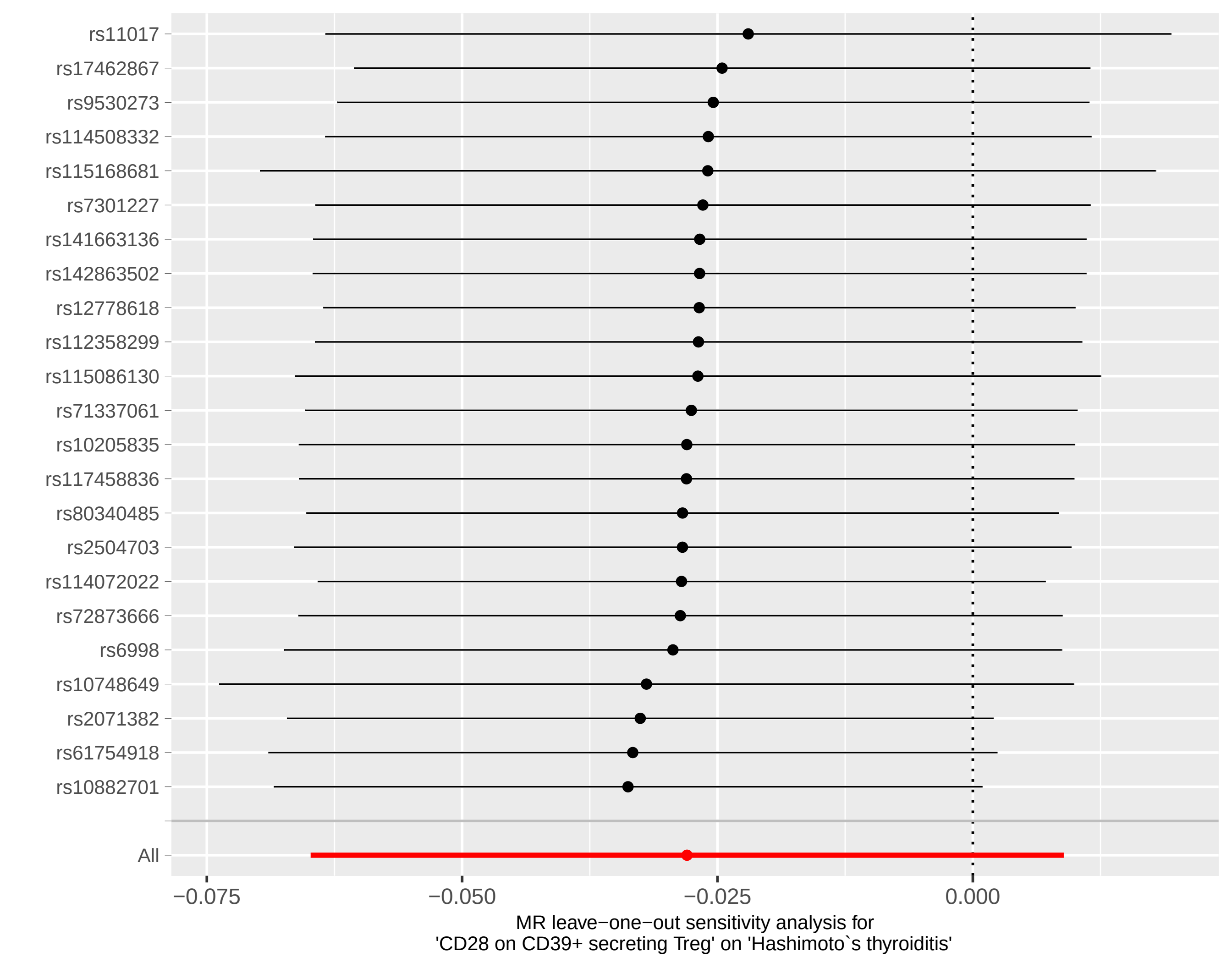
rs11017
rs17462867
rs9530273
rs114508332
rs115168681
rs7301227
rs141663136
rs142863502
rs12778618
rs112358299
rs115086130
rs71337061
rs10205835
rs117458836
rs80340485
rs2504703
rs114072022
rs72873666
rs6998
rs10748649
rs2071382
rs61754918
rs10882701
All
−0.075
−0.050
−0.025
0.000
MR leave−one−out sensitivity analysis for
'CD28 on CD39+ secreting Treg' on 'Hashimoto`s thyroiditis'

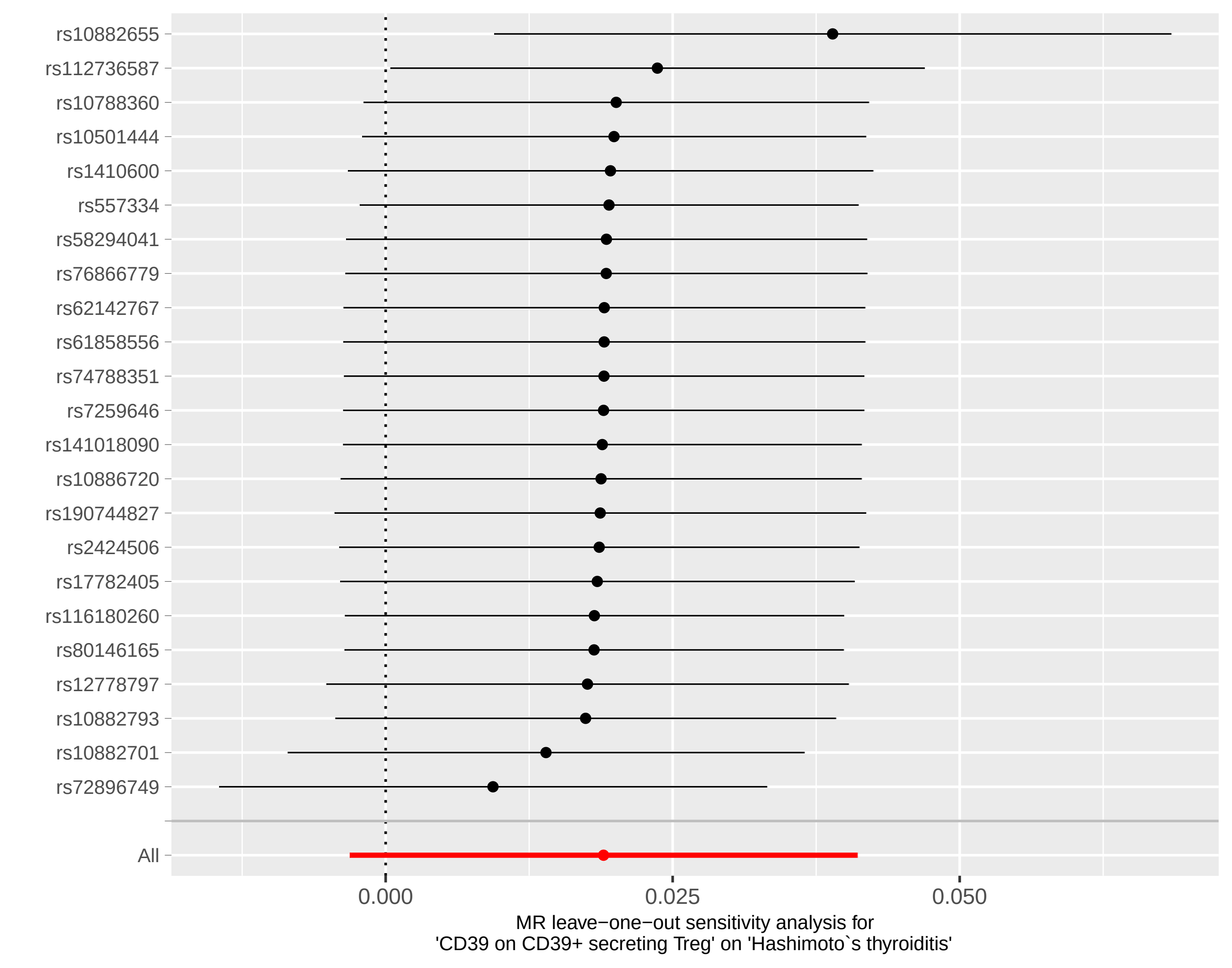

rs10882655
rs112736587
rs10788360
rs10501444
rs1410600
rs557334
rs58294041
rs76866779
rs62142767
rs61858556
rs74788351
rs7259646
rs141018090
rs10886720
rs190744827
rs2424506
rs17782405
rs116180260
rs80146165
rs12778797
rs10882793
rs10882701
rs72896749
All
0.000
0.025
0.050
MR leave−one−out sensitivity analysis for
'CD39 on CD39+ secreting Treg' on 'Hashimoto`s thyroiditis'

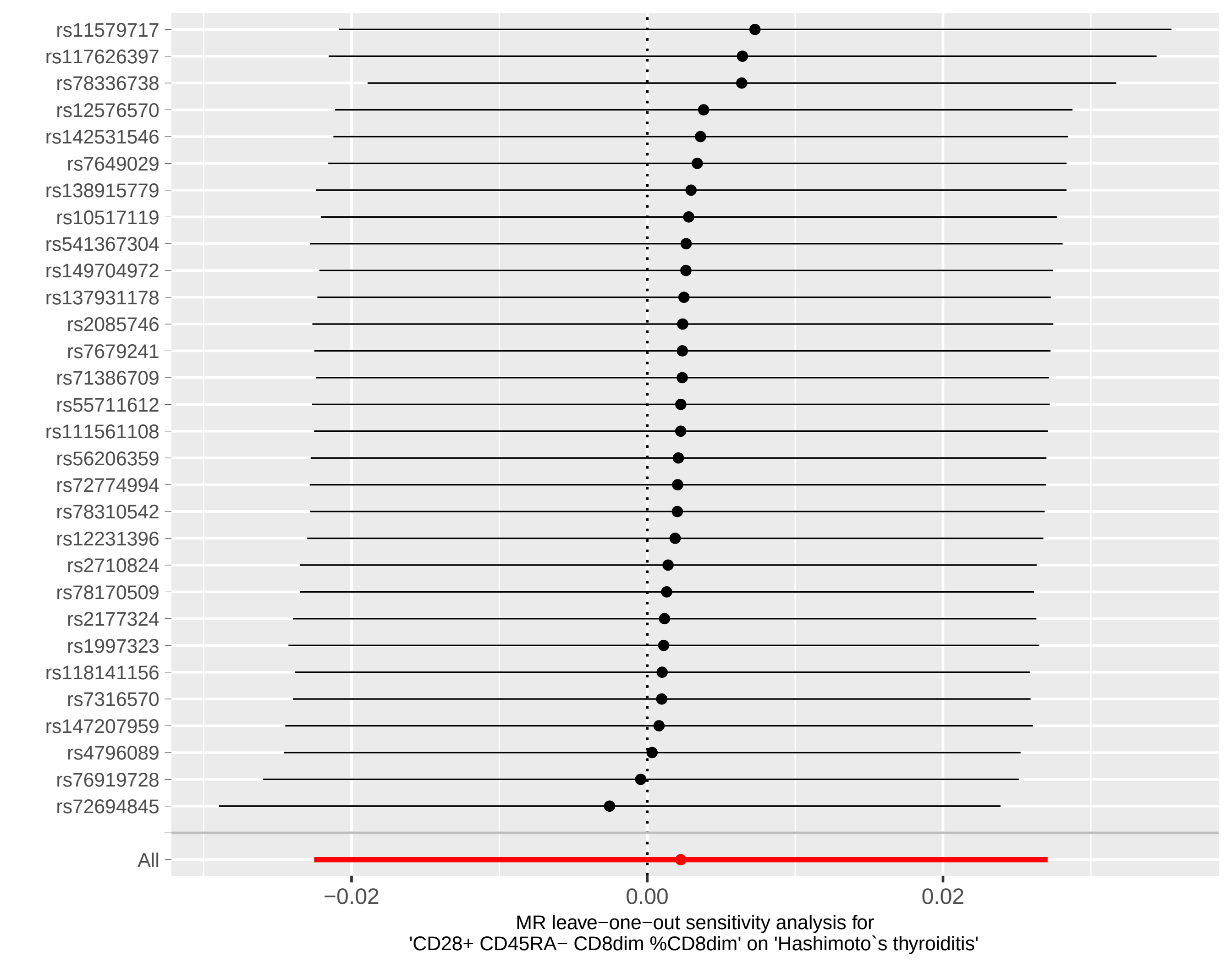
rs11579717
rs117626397
rs78336738
rs12576570
rs142531546
rs7649029
rs138915779
rs10517119
rs541367304
rs149704972
rs137931178
rs2085746
rs7679241
rs71386709
rs55711612
rs111561108
rs56206359
rs72774994
rs78310542
rs12231396
rs2710824
rs78170509
rs2177324
rs1997323
rs118141156
rs7316570
rs147207959
rs4796089
rs76919728
rs72694845
All
−0.02
0.00
0.02
MR leave−one−out sensitivity analysis for
'CD28+ CD45RA− CD8dim %CD8dim' on 'Hashimoto`s thyroiditis'

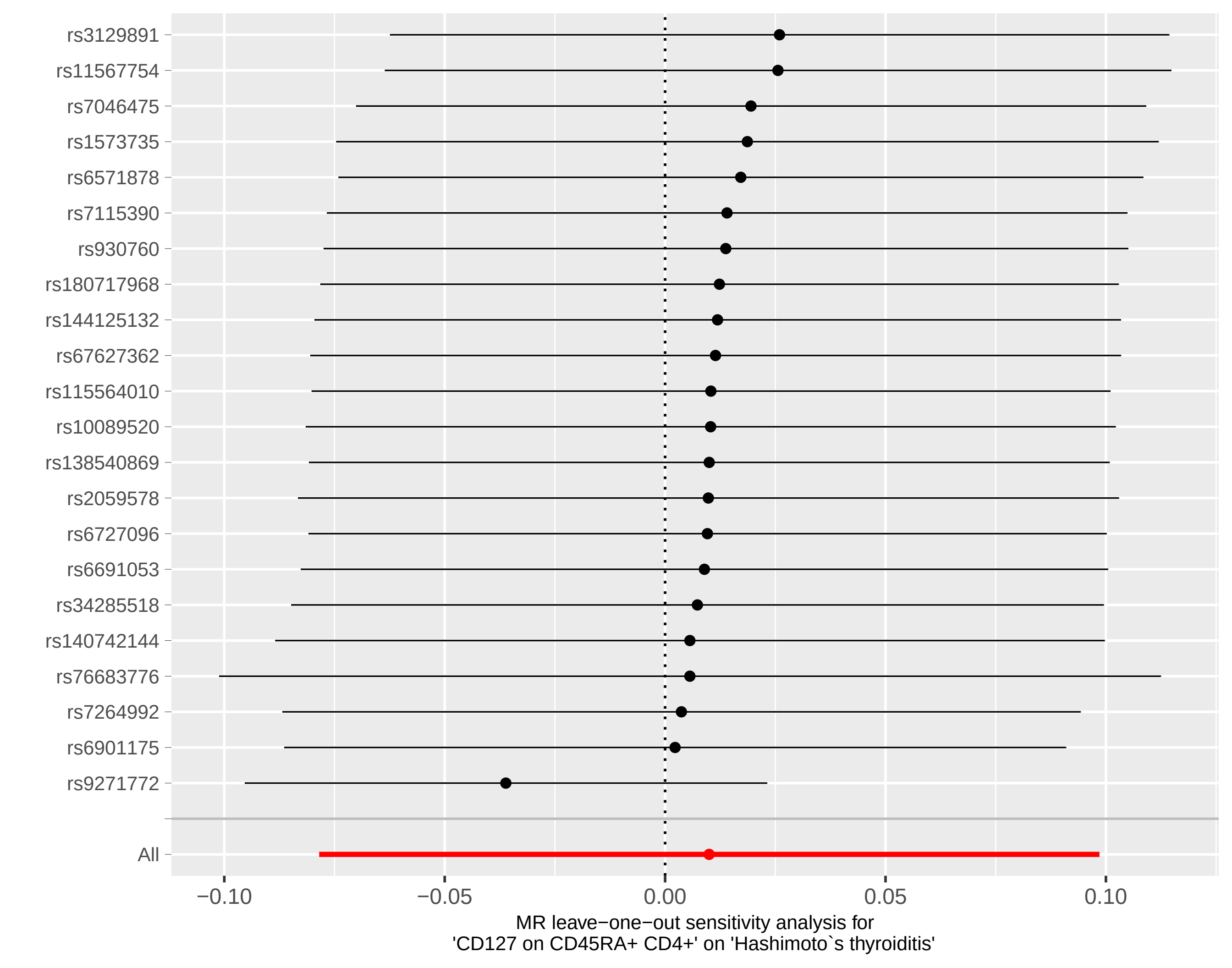
rs3129891
rs11567754
rs7046475
rs1573735
rs6571878
rs7115390
rs930760
rs180717968
rs144125132
rs67627362
rs115564010
rs10089520
rs138540869
rs2059578
rs6727096
rs6691053
rs34285518
rs140742144
rs76683776
rs7264992
rs6901175
rs9271772
All
−0.10
−0.05
0.00
0.05
0.10
MR leave−one−out sensitivity analysis for
'CD127 on CD45RA+ CD4+' on 'Hashimoto`s thyroiditis'

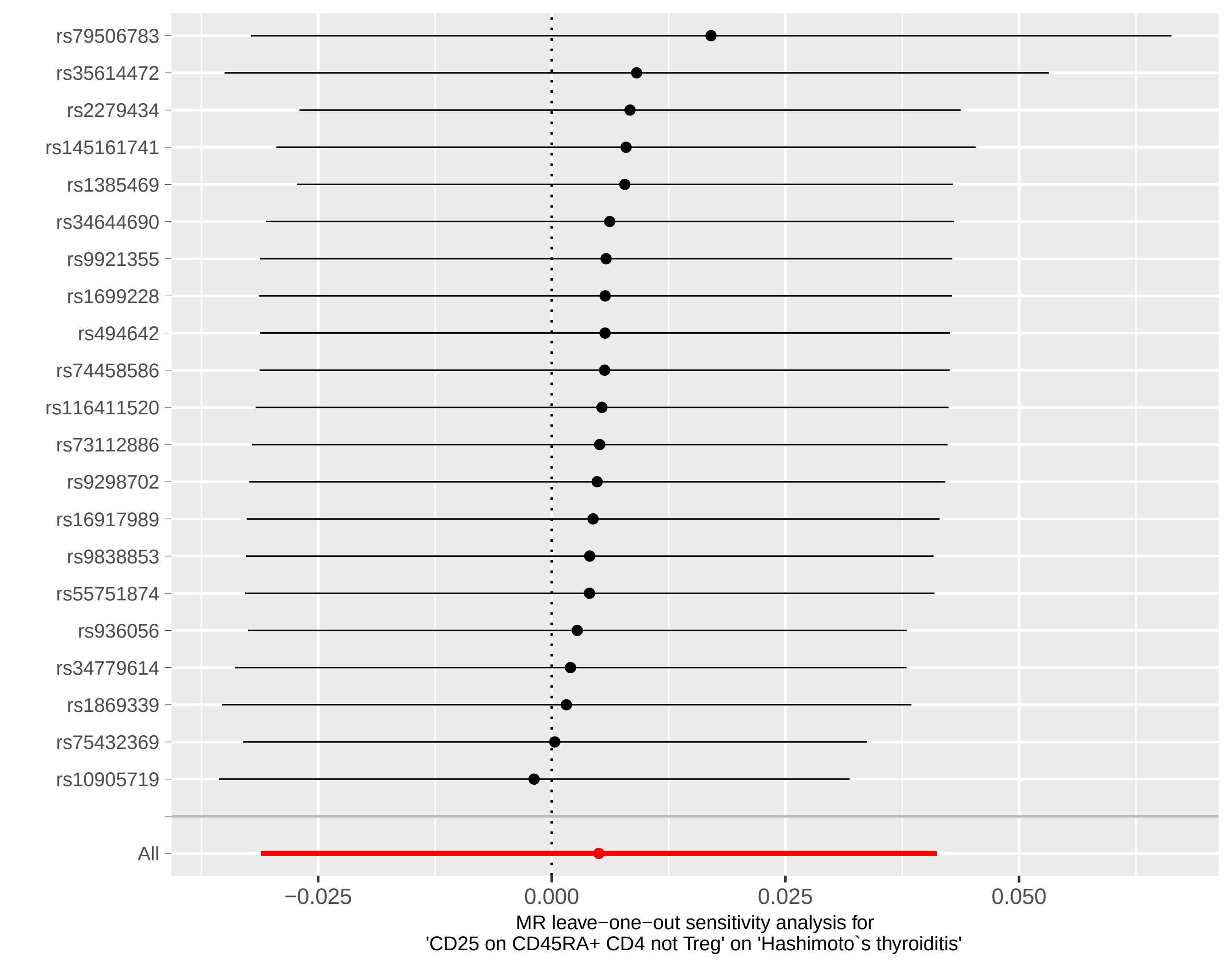

rs79506783
rs35614472
rs2279434
rs145161741
rs1385469
rs34644690
rs9921355
rs1699228
rs494642
rs74458586
rs116411520
rs73112886
rs9298702
rs16917989
rs9838853
rs55751874
rs936056
rs34779614
rs1869339
rs75432369
rs10905719
All
−0.025
0.000
0.025
0.050
MR leave−one−out sensitivity analysis for
'CD25 on CD45RA+ CD4 not Treg' on 'Hashimoto`s thyroiditis'

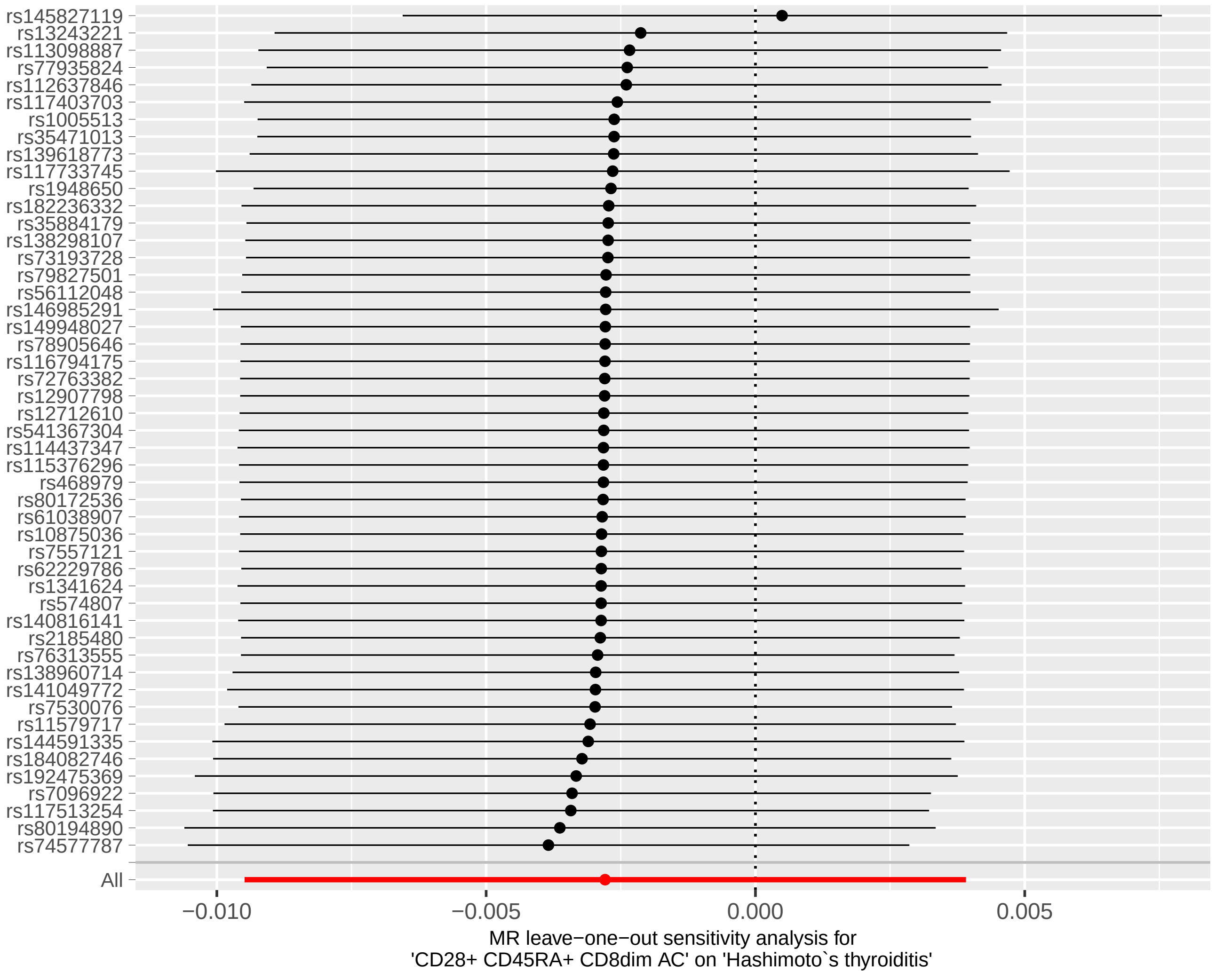

rs145827119
rs13243221
rs113098887
rs77935824
rs112637846
rs117403703
rs1005513
rs35471013
rs139618773
rs117733745
rs1948650
rs182236332
rs35884179
rs138298107
rs73193728
rs79827501
rs56112048
rs146985291
rs149948027
rs78905646
rs116794175
rs72763382
rs12907798
rs12712610
rs541367304
rs114437347
rs115376296
rs468979
rs80172536
rs61038907
rs10875036
rs7557121
rs62229786
rs1341624
rs574807
rs140816141
rs2185480
rs76313555
rs138960714
rs141049772
rs7530076
rs11579717
rs144591335
rs184082746
rs192475369
rs7096922
rs117513254
rs80194890
rs74577787
All
−0.010
−0.005
0.000
0.005
MR leave−one−out sensitivity analysis for
'CD28+ CD45RA+ CD8dim AC' on 'Hashimoto`s thyroiditis'

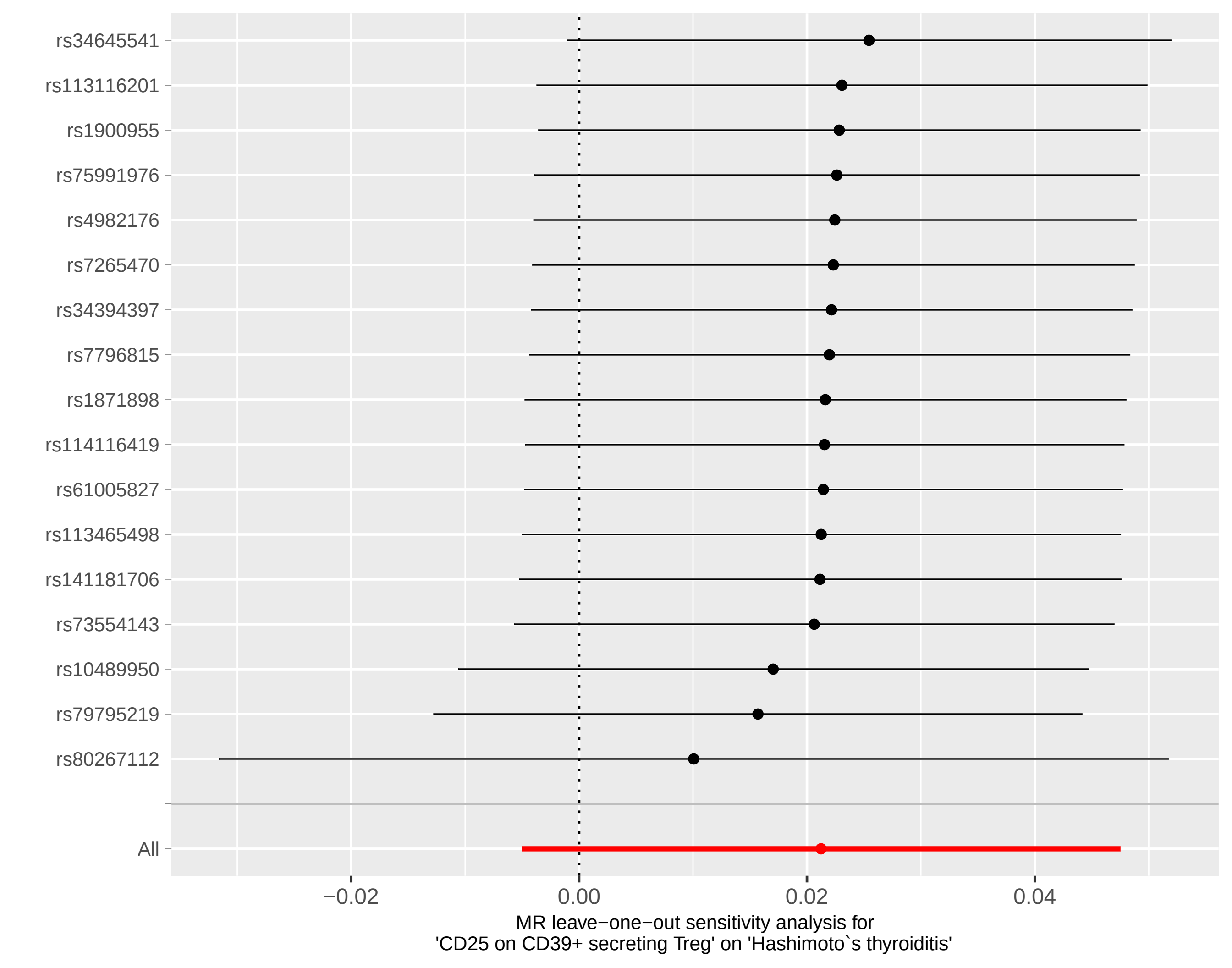
rs34645541
rs113116201
rs1900955
rs75991976
rs4982176
rs7265470
rs34394397
rs7796815
rs1871898
rs114116419
rs61005827
rs113465498
rs141181706
rs73554143
rs10489950
rs79795219
rs80267112
All
−0.02
0.00
0.02
0.04
MR leave−one−out sensitivity analysis for
'CD25 on CD39+ secreting Treg' on 'Hashimoto`s thyroiditis'

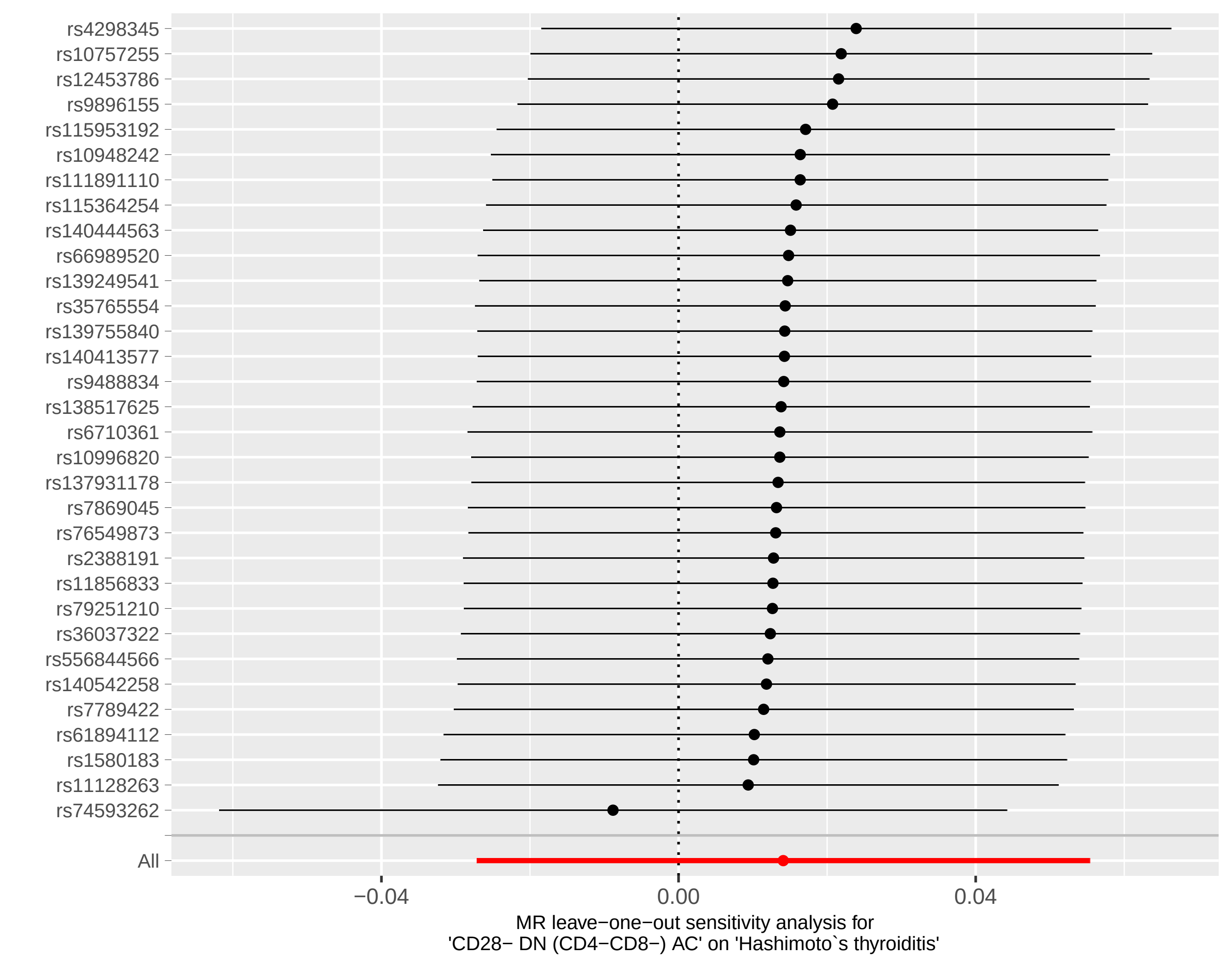

rs4298345
rs10757255
rs12453786
rs9896155
rs115953192
rs10948242
rs111891110
rs115364254
rs140444563
rs66989520
rs139249541
rs35765554
rs139755840
rs140413577
rs9488834
rs138517625
rs6710361
rs10996820
rs137931178
rs7869045
rs76549873
rs2388191
rs11856833
rs79251210
rs36037322
rs556844566
rs140542258
rs7789422
rs61894112
rs1580183
rs11128263
rs74593262
All
−0.04
0.00
0.04
MR leave−one−out sensitivity analysis for
'CD28− DN (CD4−CD8−) AC' on 'Hashimoto`s thyroiditis'

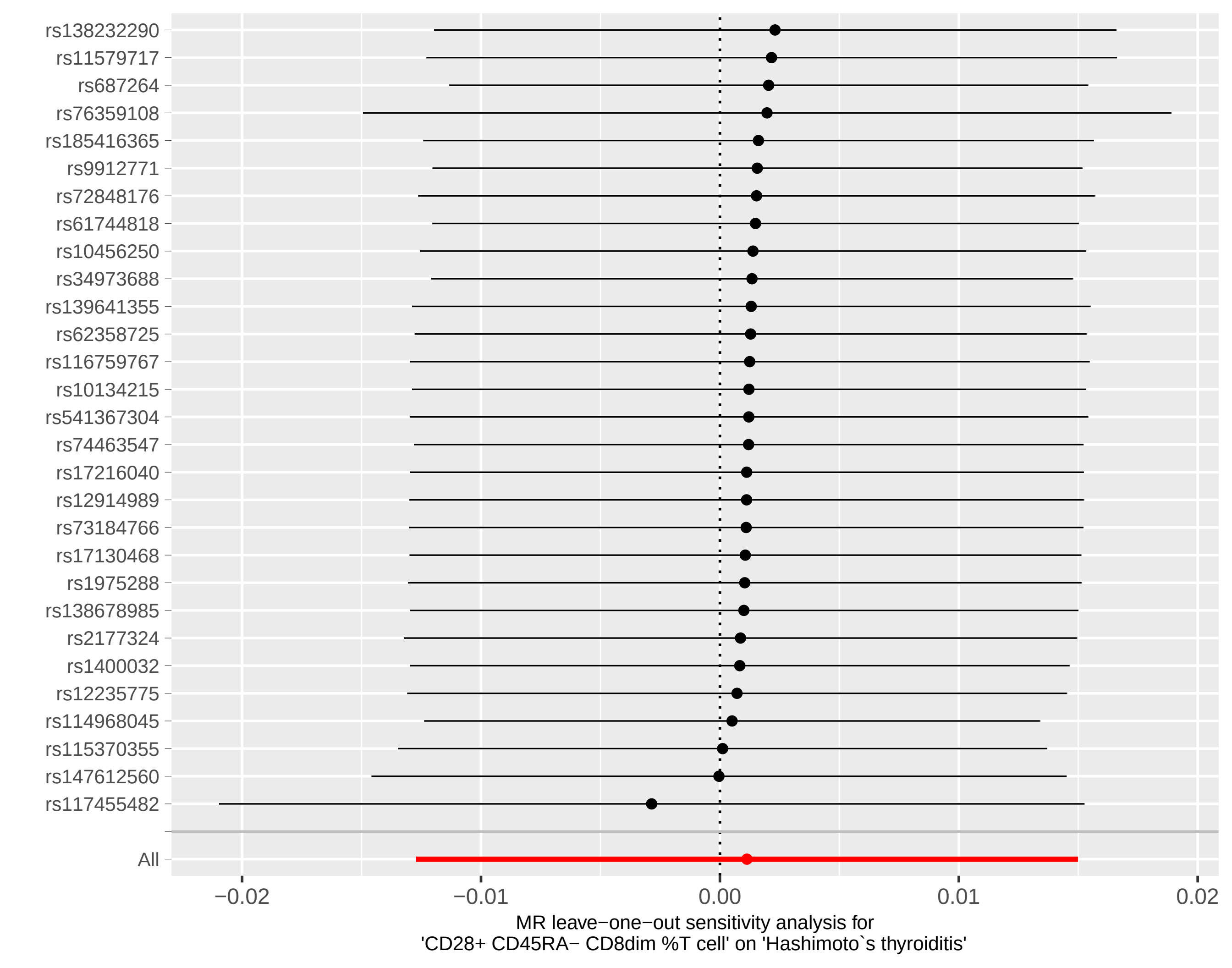
rs138232290
rs11579717
rs687264
rs76359108
rs185416365
rs9912771
rs72848176
rs61744818
rs10456250
rs34973688
rs139641355
rs62358725
rs116759767
rs10134215
rs541367304
rs74463547
rs17216040
rs12914989
rs73184766
rs17130468
rs1975288
rs138678985
rs2177324
rs1400032
rs12235775
rs114968045
rs115370355
rs147612560
rs117455482
All
−0.02
−0.01
0.00
0.01
0.02
MR leave−one−out sensitivity analysis for
'CD28+ CD45RA− CD8dim %T cell' on 'Hashimoto`s thyroiditis'

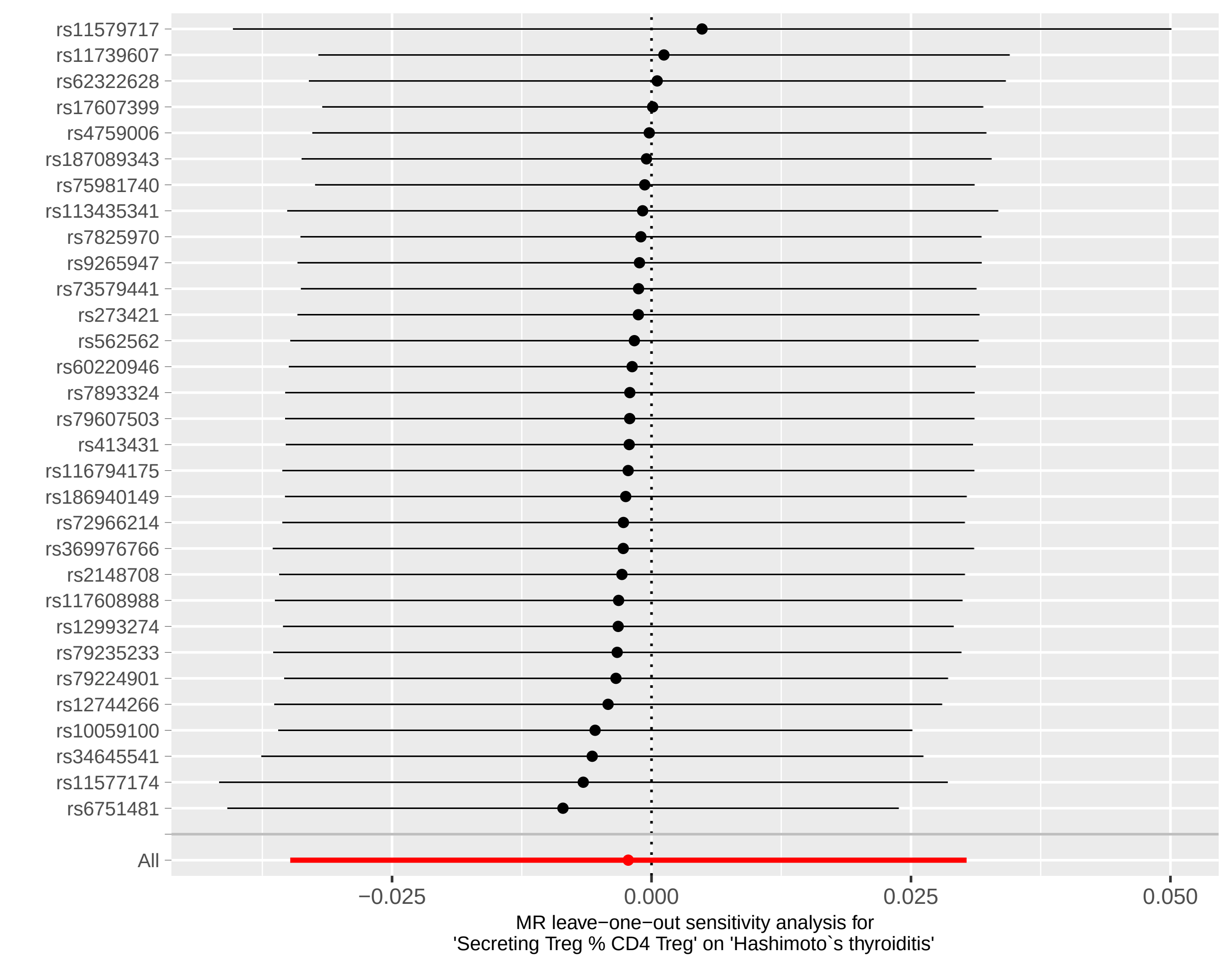
rs11579717
rs11739607
rs62322628
rs17607399
rs4759006
rs187089343
rs75981740
rs113435341
rs7825970
rs9265947
rs73579441
rs273421
rs562562
rs60220946
rs7893324
rs79607503
rs413431
rs116794175
rs186940149
rs72966214
rs369976766
rs2148708
rs117608988
rs12993274
rs79235233
rs79224901
rs12744266
rs10059100
rs34645541
rs11577174
rs6751481
All
−0.025
0.000
0.025
0.050
MR leave−one−out sensitivity analysis for
'Secreting Treg % CD4 Treg' on 'Hashimoto`s thyroiditis'

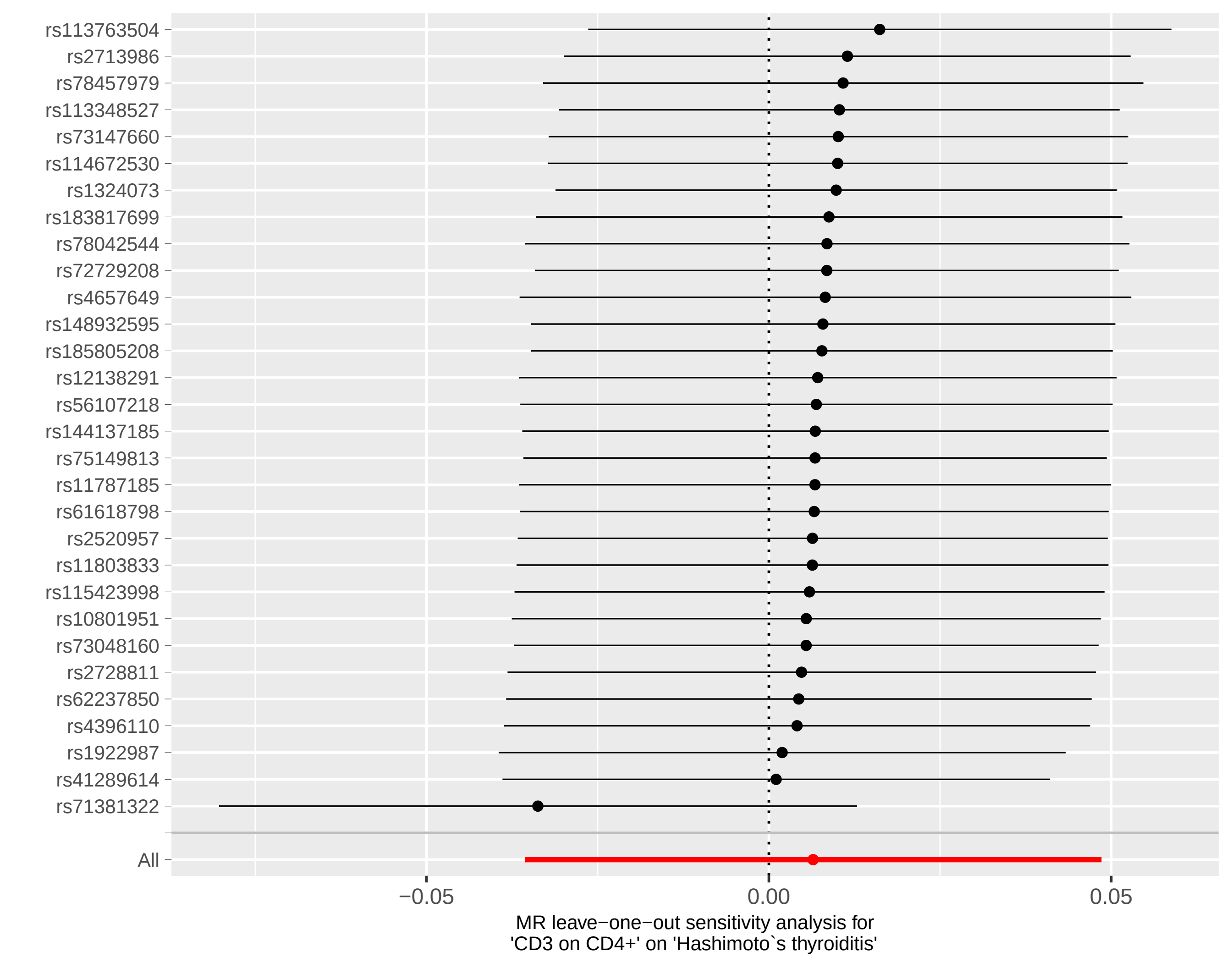
rs113763504
rs2713986
rs78457979
rs113348527
rs73147660
rs114672530
rs1324073
rs183817699
rs78042544
rs72729208
rs4657649
rs148932595
rs185805208
rs12138291
rs56107218
rs144137185
rs75149813
rs11787185
rs61618798
rs2520957
rs11803833
rs115423998
rs10801951
rs73048160
rs2728811
rs62237850
rs4396110
rs1922987
rs41289614
rs71381322
All
−0.05
0.00
0.05
MR leave−one−out sensitivity analysis for
'CD3 on CD4+' on 'Hashimoto`s thyroiditis'

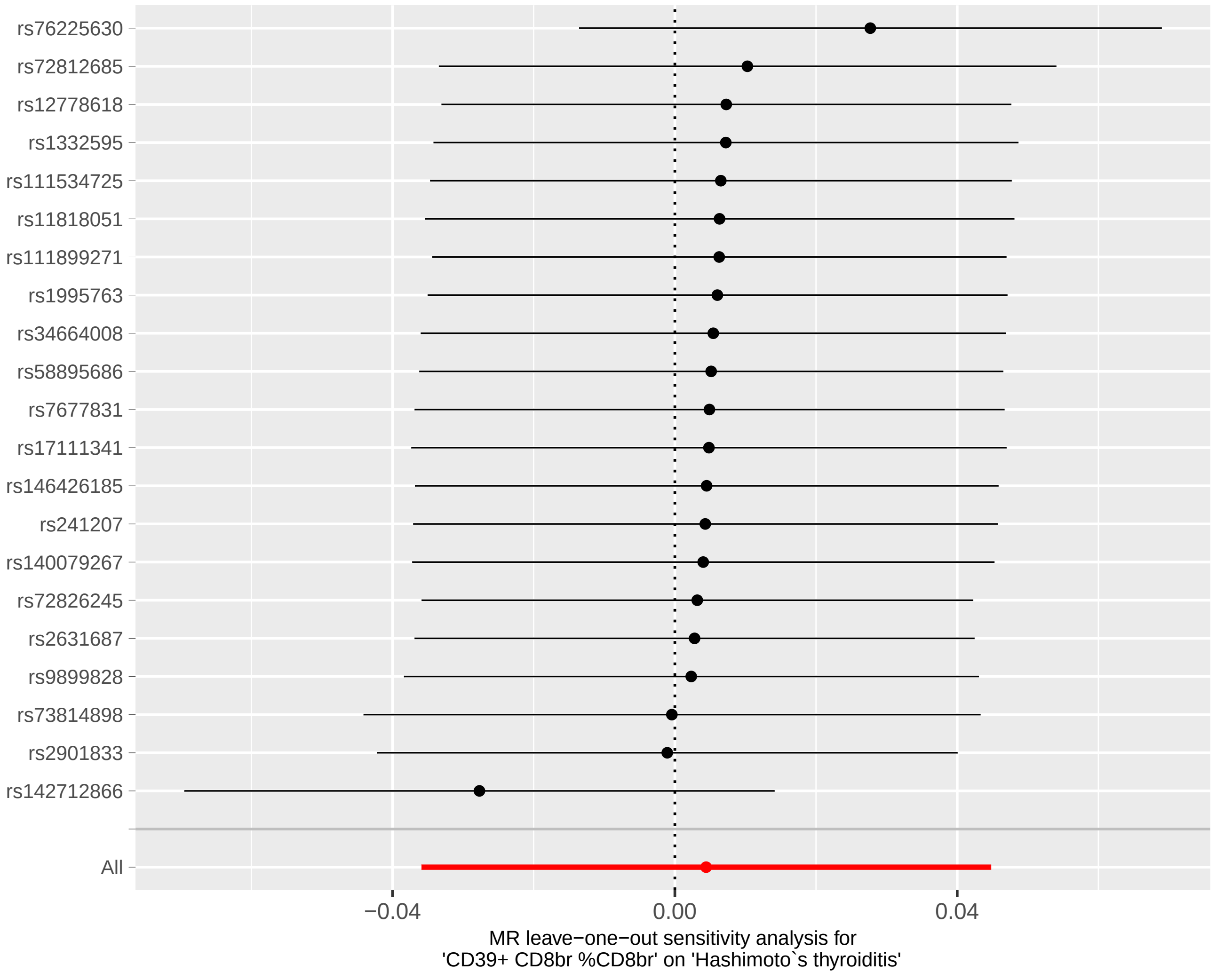

rs76225630
rs72812685
rs12778618
rs1332595
rs111534725
rs11818051
rs111899271
rs1995763
rs34664008
rs58895686
rs7677831
rs17111341
rs146426185
rs241207
rs140079267
rs72826245
rs2631687
rs9899828
rs73814898
rs2901833
rs142712866
All
−0.04
0.00
0.04
MR leave−one−out sensitivity analysis for
'CD39+ CD8br %CD8br' on 'Hashimoto`s thyroiditis'

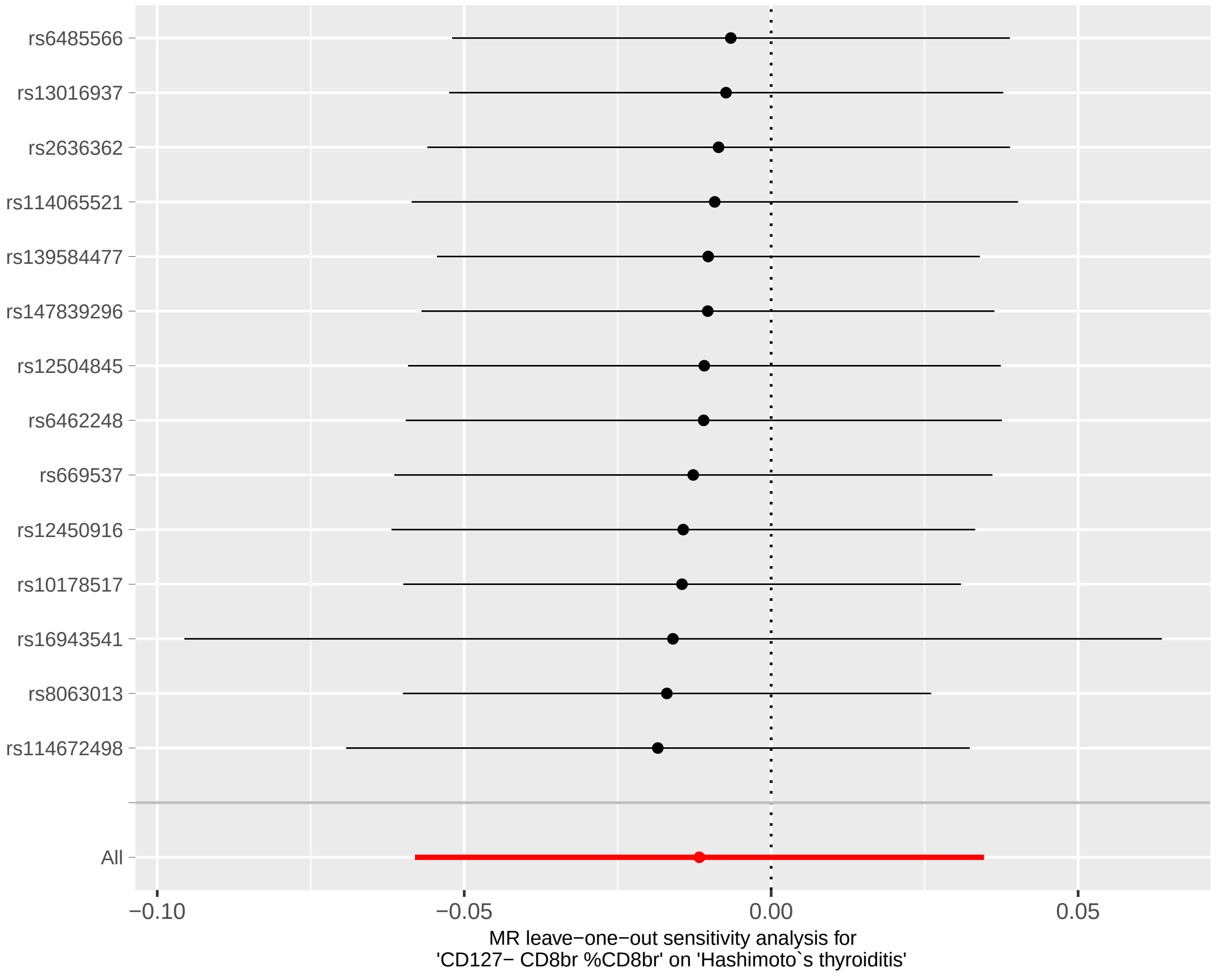

rs6485566
rs13016937
rs2636362
rs114065521
rs139584477
rs147839296
rs12504845
rs6462248
rs669537
rs12450916
rs10178517
rs16943541
rs8063013
rs114672498
All
−0.10
−0.05
0.00
0.05
MR leave−one−out sensitivity analysis for
'CD127− CD8br %CD8br' on 'Hashimoto`s thyroiditis'

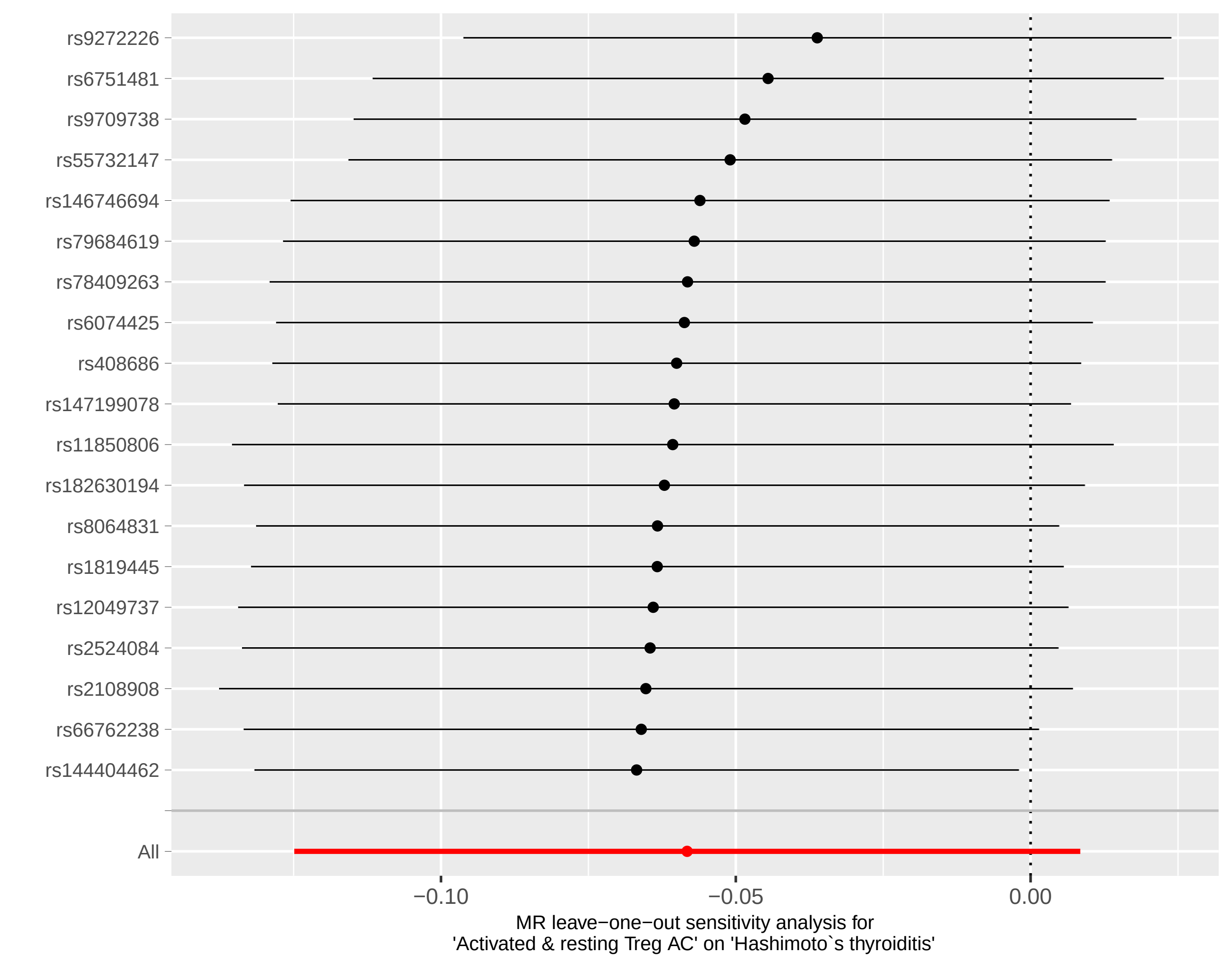
rs9272226
rs6751481
rs9709738
rs55732147
rs146746694
rs79684619
rs78409263
rs6074425
rs408686
rs147199078
rs11850806
rs182630194
rs8064831
rs1819445
rs12049737
rs2524084
rs2108908
rs66762238
rs144404462
All
−0.10
−0.05
0.00
MR leave−one−out sensitivity analysis for
'Activated & resting Treg AC' on 'Hashimoto`s thyroiditis'

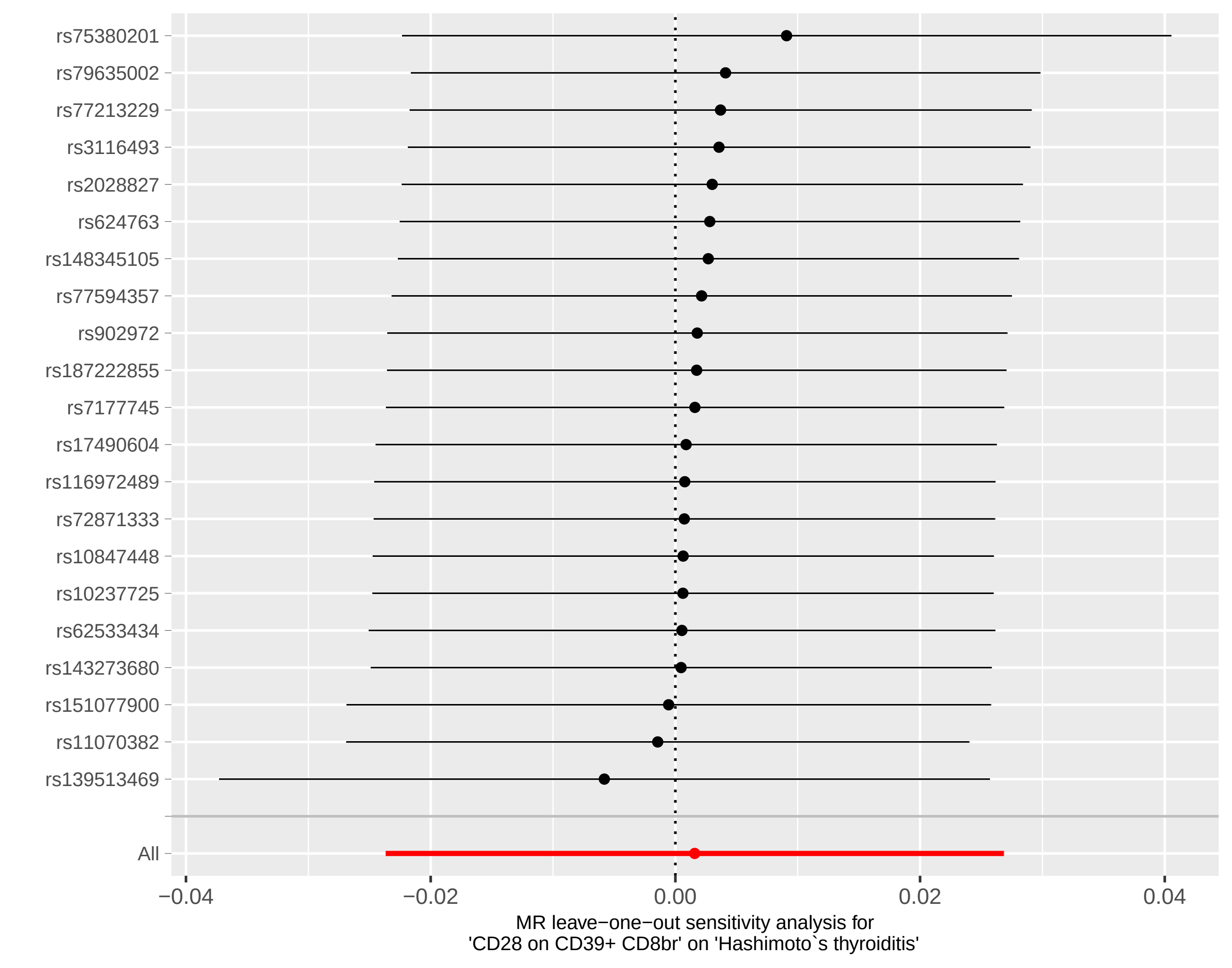
rs75380201
rs79635002
rs77213229
rs3116493
rs2028827
rs624763
rs148345105
rs77594357
rs902972
rs187222855
rs7177745
rs17490604
rs116972489
rs72871333
rs10847448
rs10237725
rs62533434
rs143273680
rs151077900
rs11070382
rs139513469
All
−0.04
−0.02
0.00
0.02
0.04
MR leave−one−out sensitivity analysis for
'CD28 on CD39+ CD8br' on 'Hashimoto`s thyroiditis'

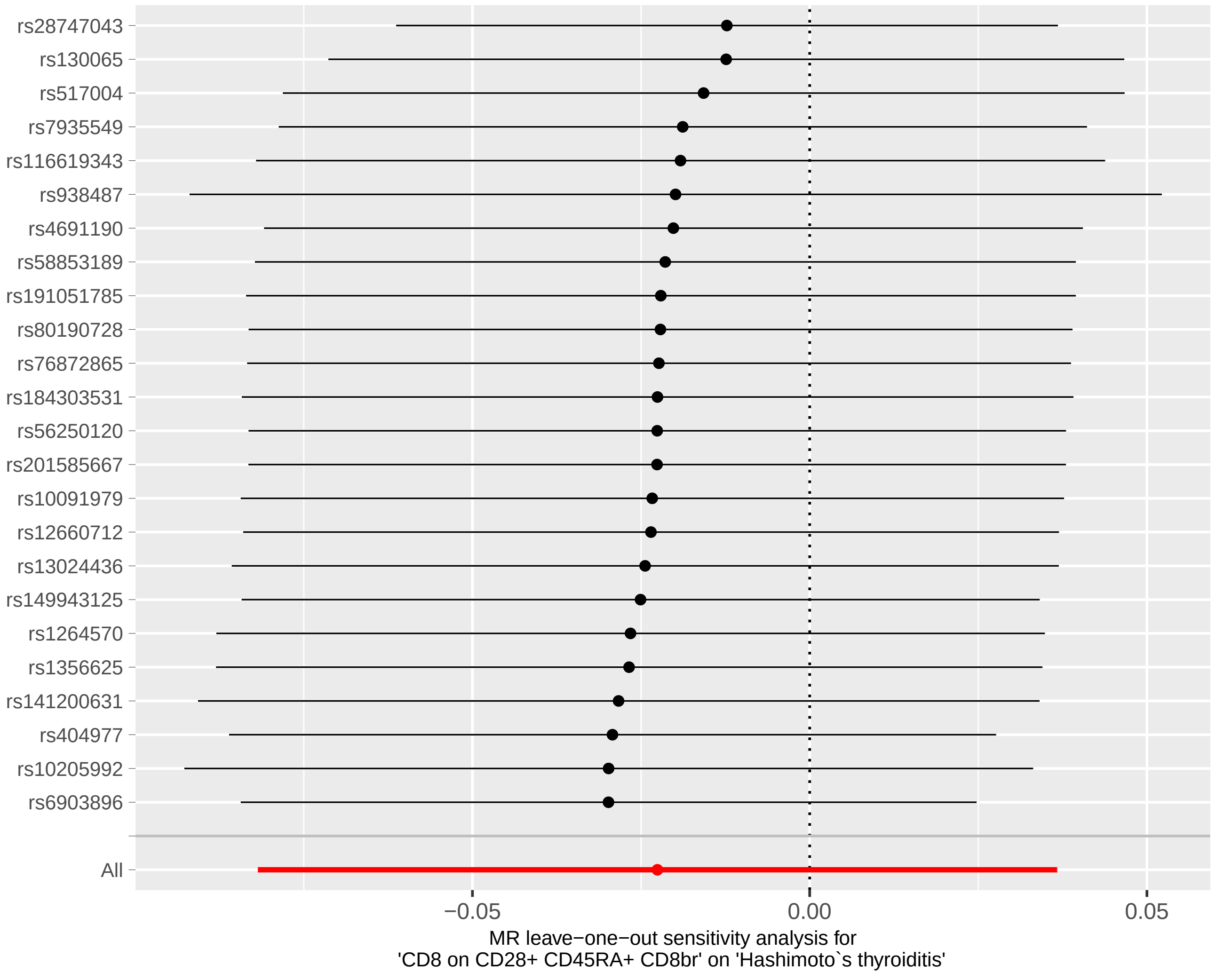

rs28747043
rs130065
rs517004
rs7935549
rs116619343
rs938487
rs4691190
rs58853189
rs191051785
rs80190728
rs76872865
rs184303531
rs56250120
rs201585667
rs10091979
rs12660712
rs13024436
rs149943125
rs1264570
rs1356625
rs141200631
rs404977
rs10205992
rs6903896
All
−0.05
0.00
0.05
MR leave−one−out sensitivity analysis for
'CD8 on CD28+ CD45RA+ CD8br' on 'Hashimoto`s thyroiditis'

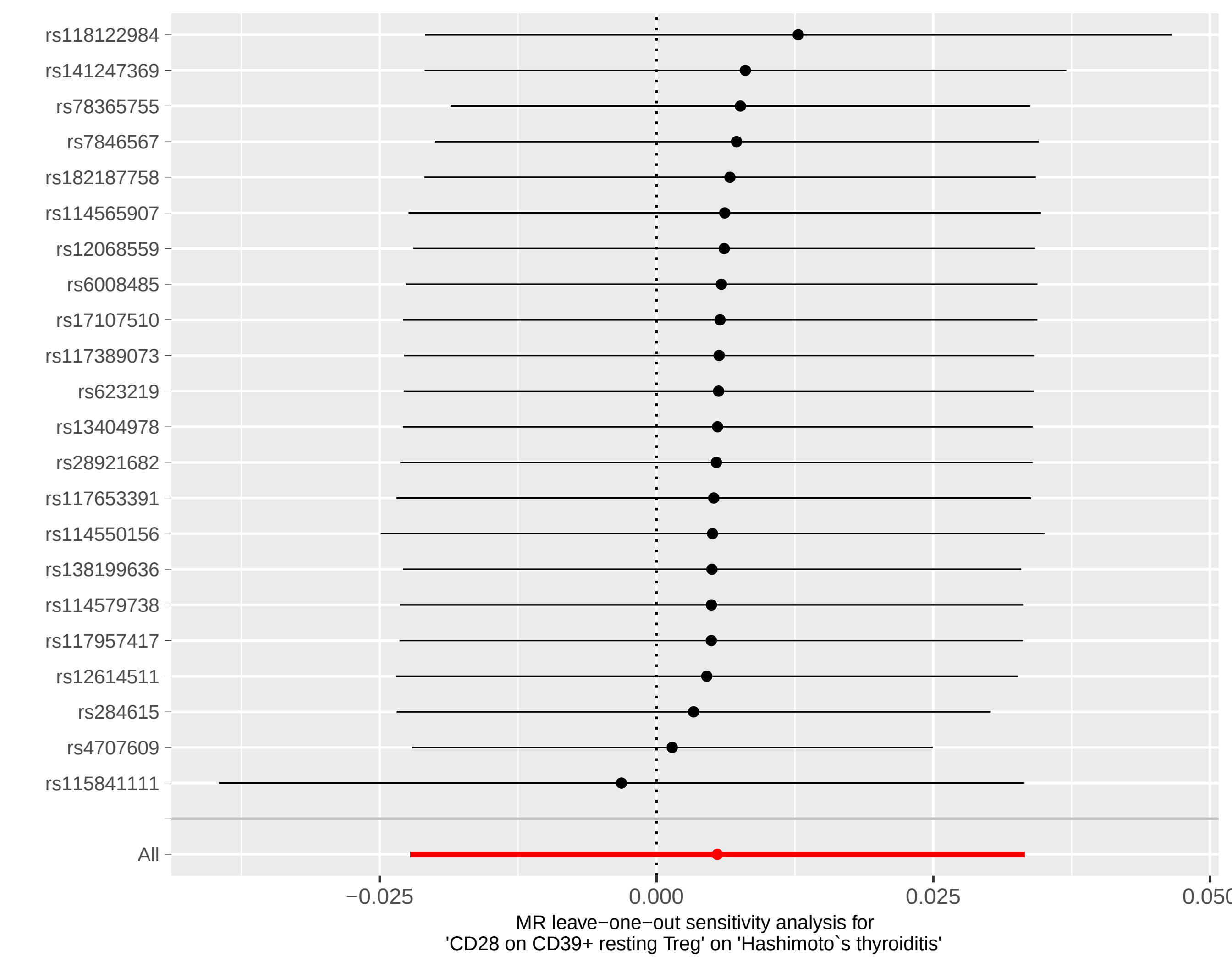
rs118122984
rs141247369
rs78365755
rs7846567
rs182187758
rs114565907
rs12068559
rs6008485
rs17107510
rs117389073
rs623219
rs13404978
rs28921682
rs117653391
rs114550156
rs138199636
rs114579738
rs117957417
rs12614511
rs284615
rs4707609
rs115841111
All
−0.025
0.000
0.025
0.050
MR leave−one−out sensitivity analysis for
'CD28 on CD39+ resting Treg' on 'Hashimoto`s thyroiditis'

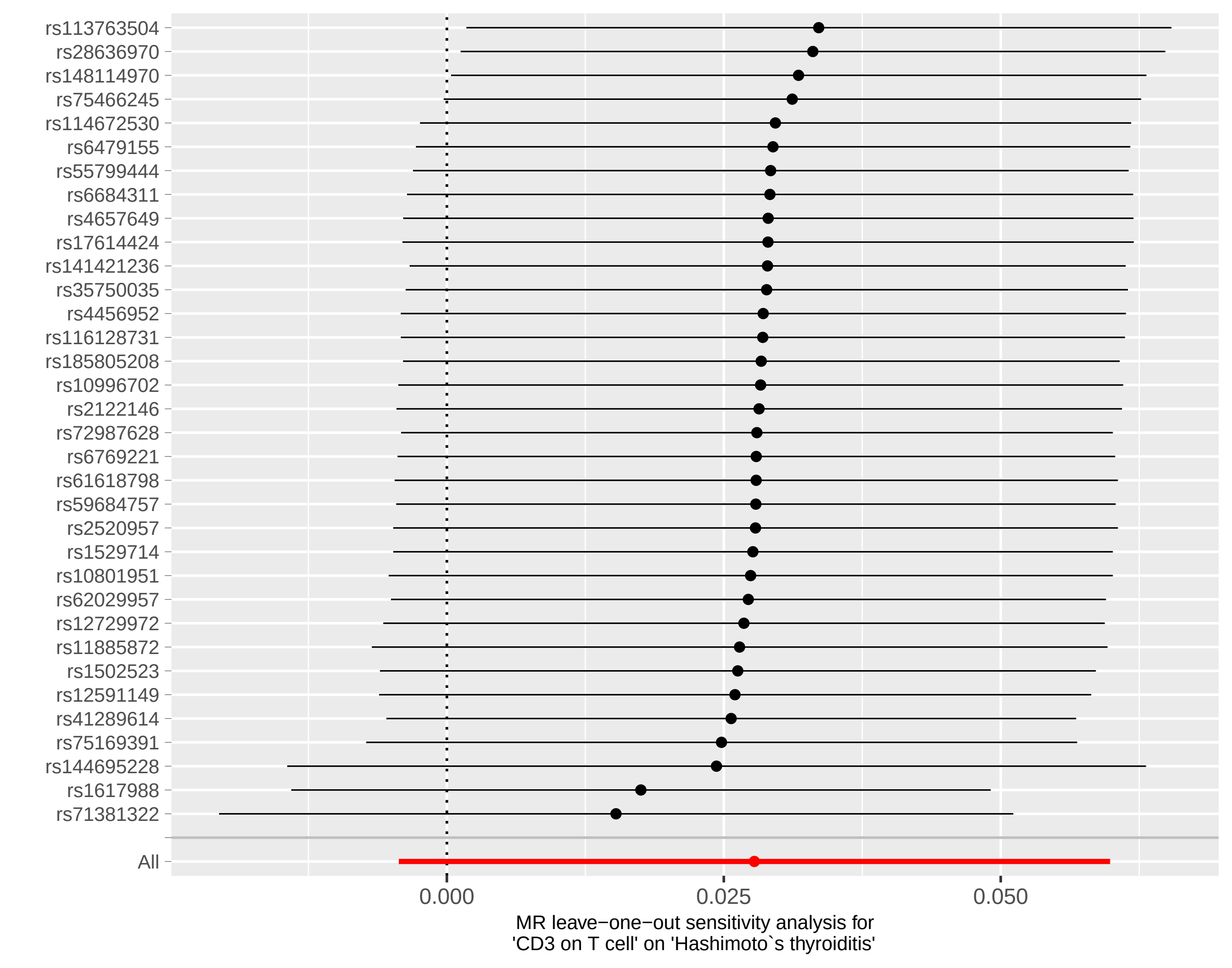

rs113763504
rs28636970
rs148114970
rs75466245
rs114672530
rs6479155
rs55799444
rs6684311
rs4657649
rs17614424
rs141421236
rs35750035
rs4456952
rs116128731
rs185805208
rs10996702
rs2122146
rs72987628
rs6769221
rs61618798
rs59684757
rs2520957
rs1529714
rs10801951
rs62029957
rs12729972
rs11885872
rs1502523
rs12591149
rs41289614
rs75169391
rs144695228
rs1617988
rs71381322
All
0.000
0.025
0.050
MR leave−one−out sensitivity analysis for
'CD3 on T cell' on 'Hashimoto`s thyroiditis'

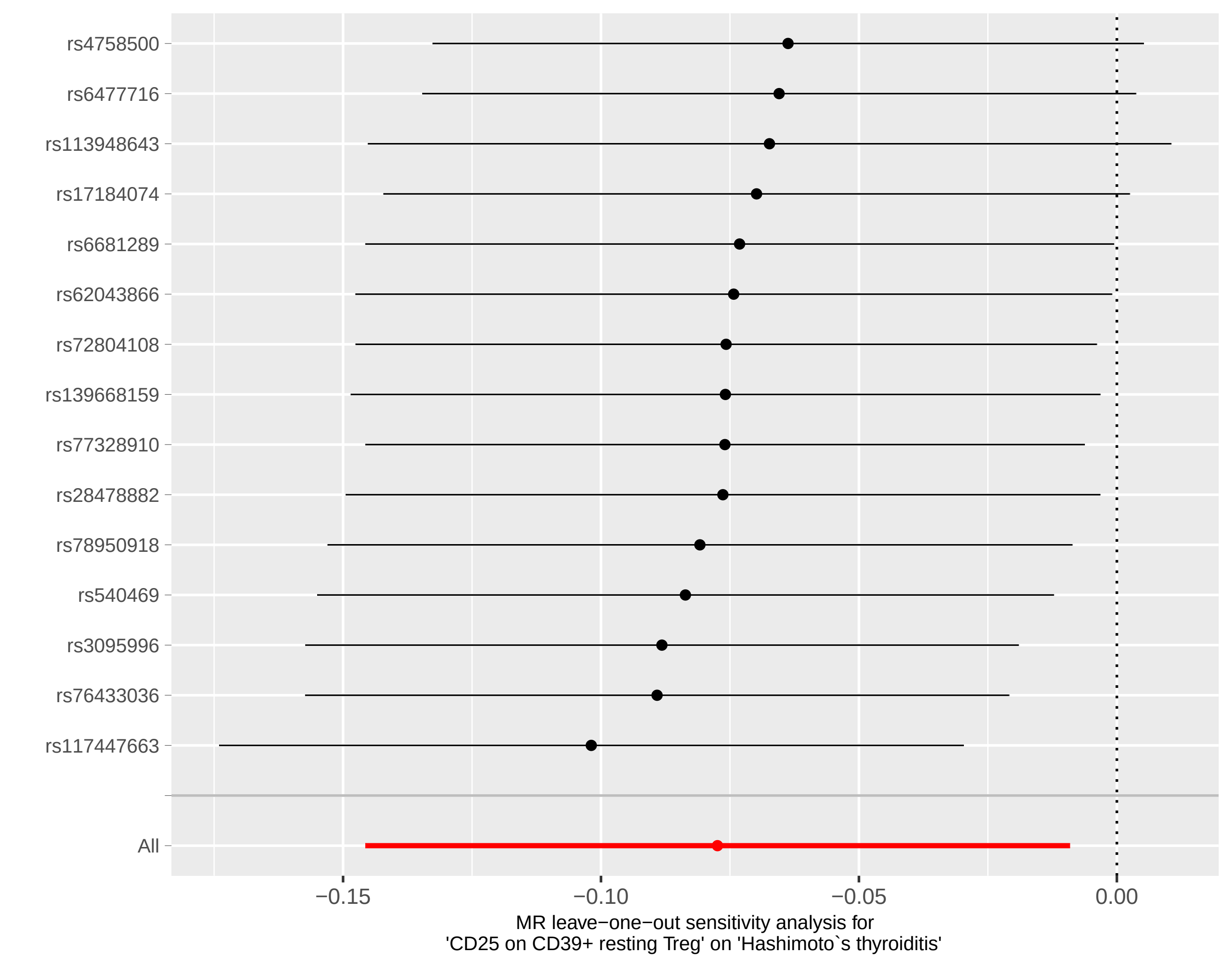

rs4758500
rs6477716
rs113948643
rs17184074
rs6681289
rs62043866
rs72804108
rs139668159
rs77328910
rs28478882
rs78950918
rs540469
rs3095996
rs76433036
rs117447663
All
−0.15
−0.10
−0.05
0.00
MR leave−one−out sensitivity analysis for
'CD25 on CD39+ resting Treg' on 'Hashimoto`s thyroiditis'

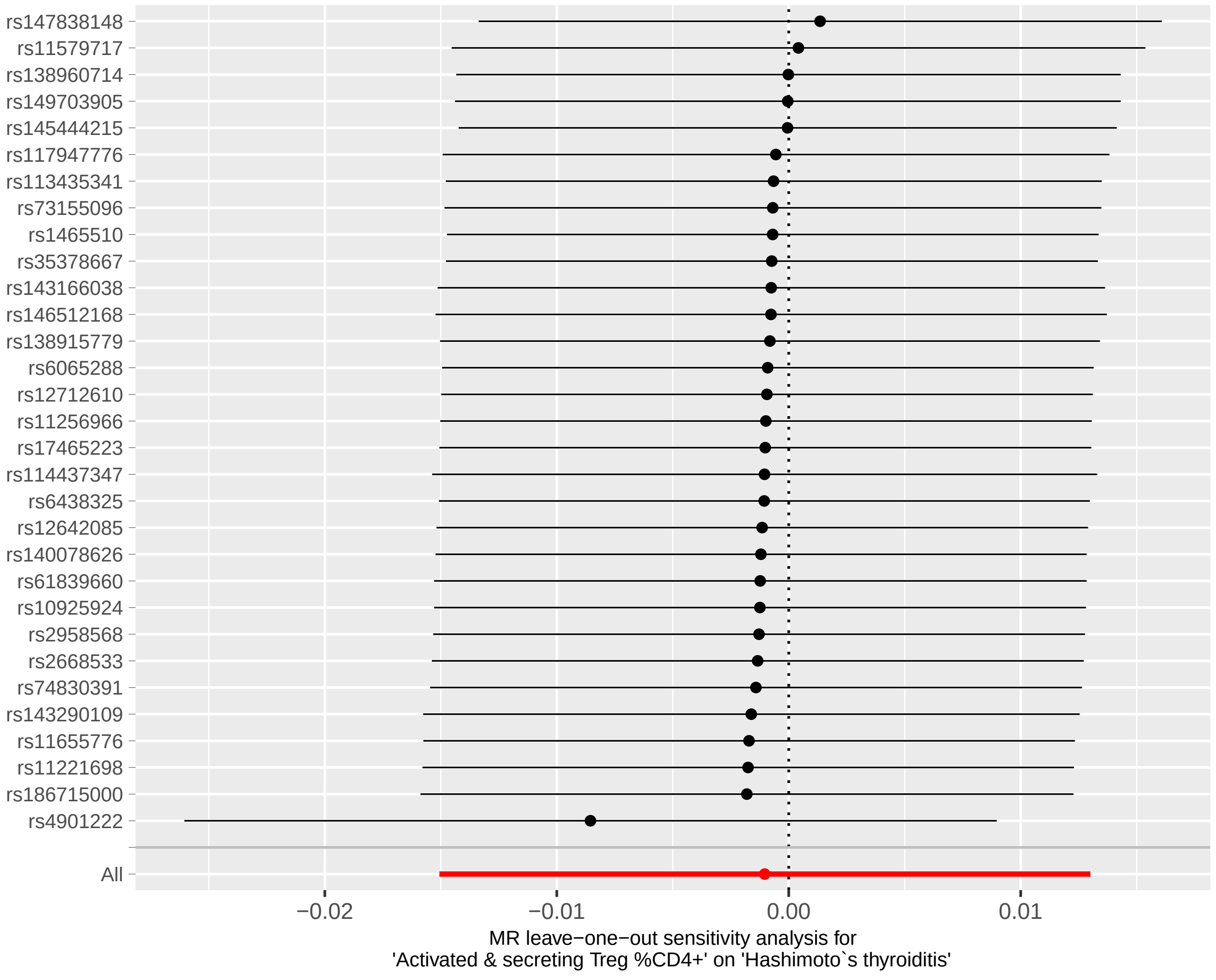

rs147838148
rs11579717
rs138960714
rs149703905
rs145444215
rs117947776
rs113435341
rs73155096
rs1465510
rs35378667
rs143166038
rs146512168
rs138915779
rs6065288
rs12712610
rs11256966
rs17465223
rs114437347
rs6438325
rs12642085
rs140078626
rs61839660
rs10925924
rs2958568
rs2668533
rs74830391
rs143290109
rs11655776
rs11221698
rs186715000
rs4901222
All
−0.02
−0.01
0.00
0.01
MR leave−one−out sensitivity analysis for
'Activated & secreting Treg %CD4+' on 'Hashimoto`s thyroiditis'

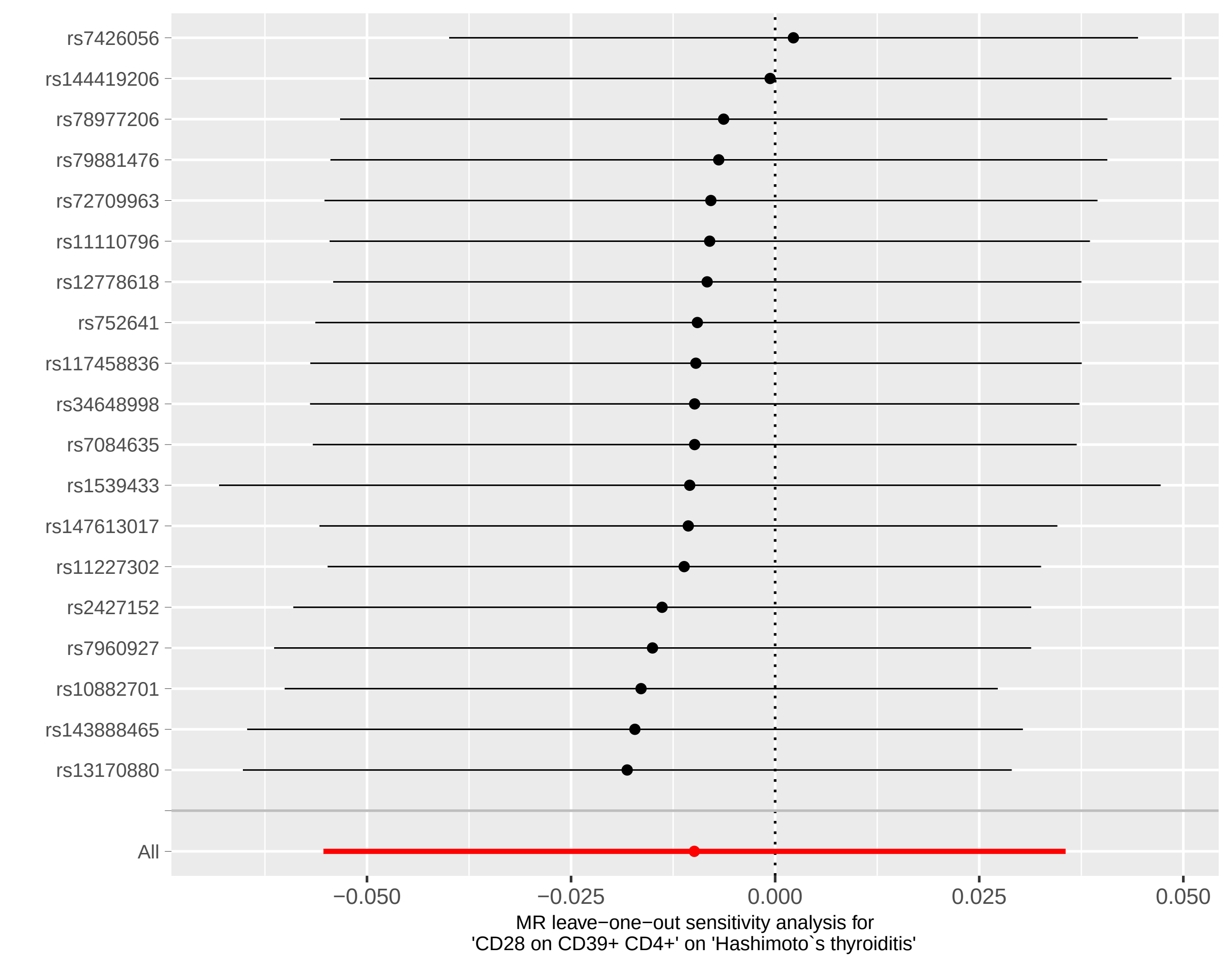

rs7426056
rs144419206
rs78977206
rs79881476
rs72709963
rs11110796
rs12778618
rs752641
rs117458836
rs34648998
rs7084635
rs1539433
rs147613017
rs11227302
rs2427152
rs7960927
rs10882701
rs143888465
rs13170880
All
−0.050
−0.025
0.000
0.025
0.050
MR leave−one−out sensitivity analysis for
'CD28 on CD39+ CD4+' on 'Hashimoto`s thyroiditis'

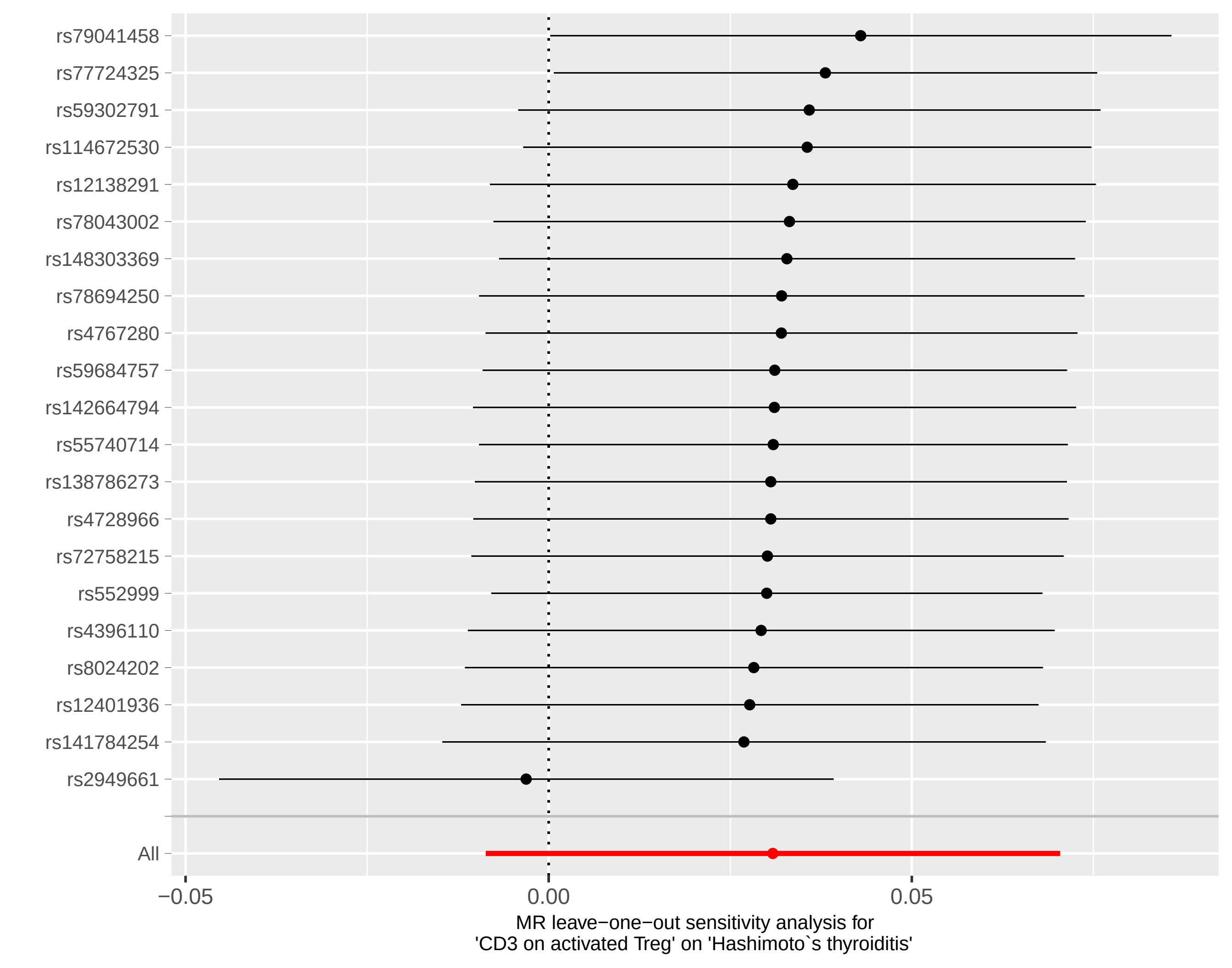

rs79041458
rs77724325
rs59302791
rs114672530
rs12138291
rs78043002
rs148303369
rs78694250
rs4767280
rs59684757
rs142664794
rs55740714
rs138786273
rs4728966
rs72758215
rs552999
rs4396110
rs8024202
rs12401936
rs141784254
rs2949661
All
−0.05
0.00
0.05
MR leave−one−out sensitivity analysis for
'CD3 on activated Treg' on 'Hashimoto`s thyroiditis'

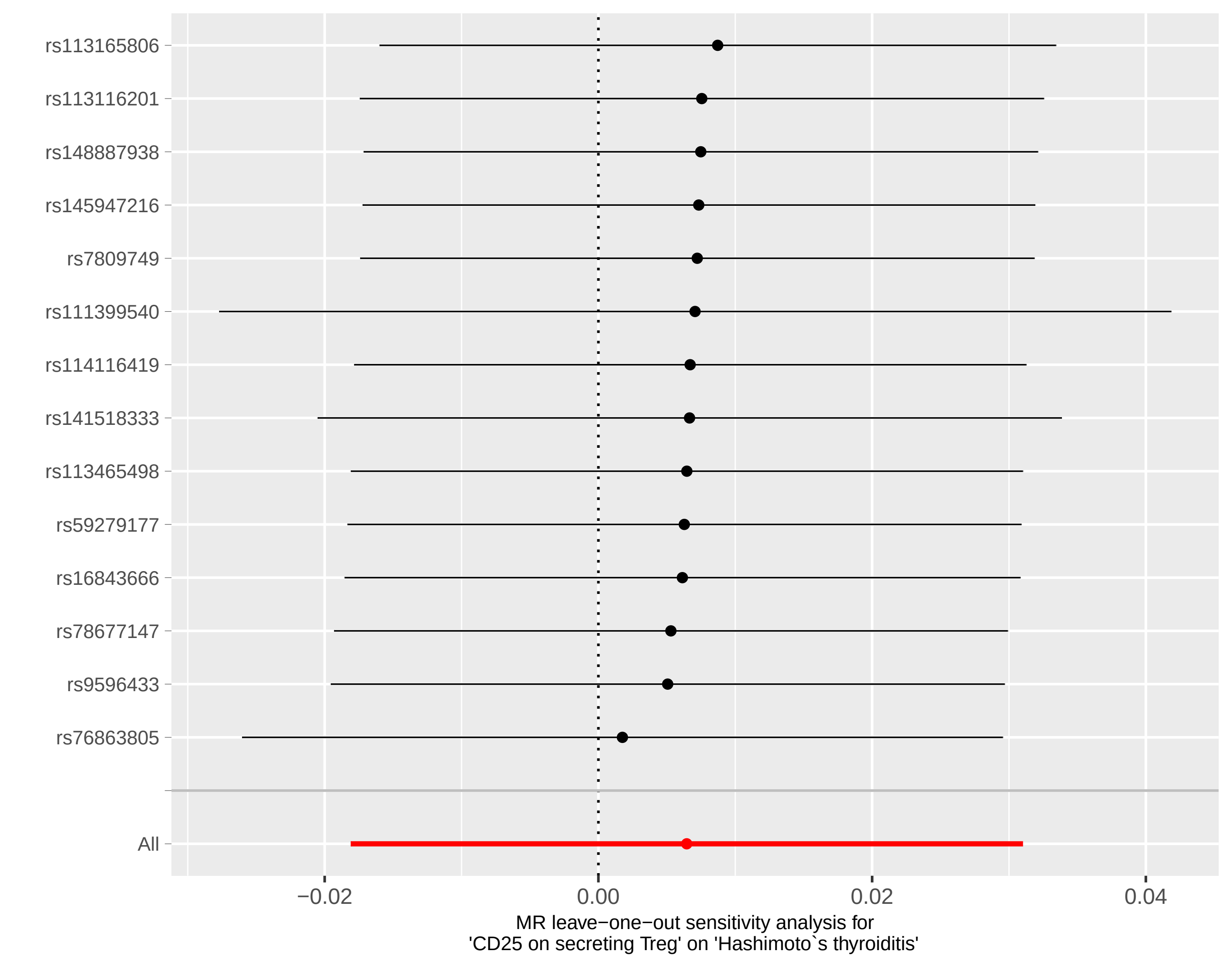

rs113165806
rs113116201
rs148887938
rs145947216
rs7809749
rs111399540
rs114116419
rs141518333
rs113465498
rs59279177
rs16843666
rs78677147
rs9596433
rs76863805
All
−0.02
0.00
0.02
0.04
MR leave−one−out sensitivity analysis for
'CD25 on secreting Treg' on 'Hashimoto`s thyroiditis'

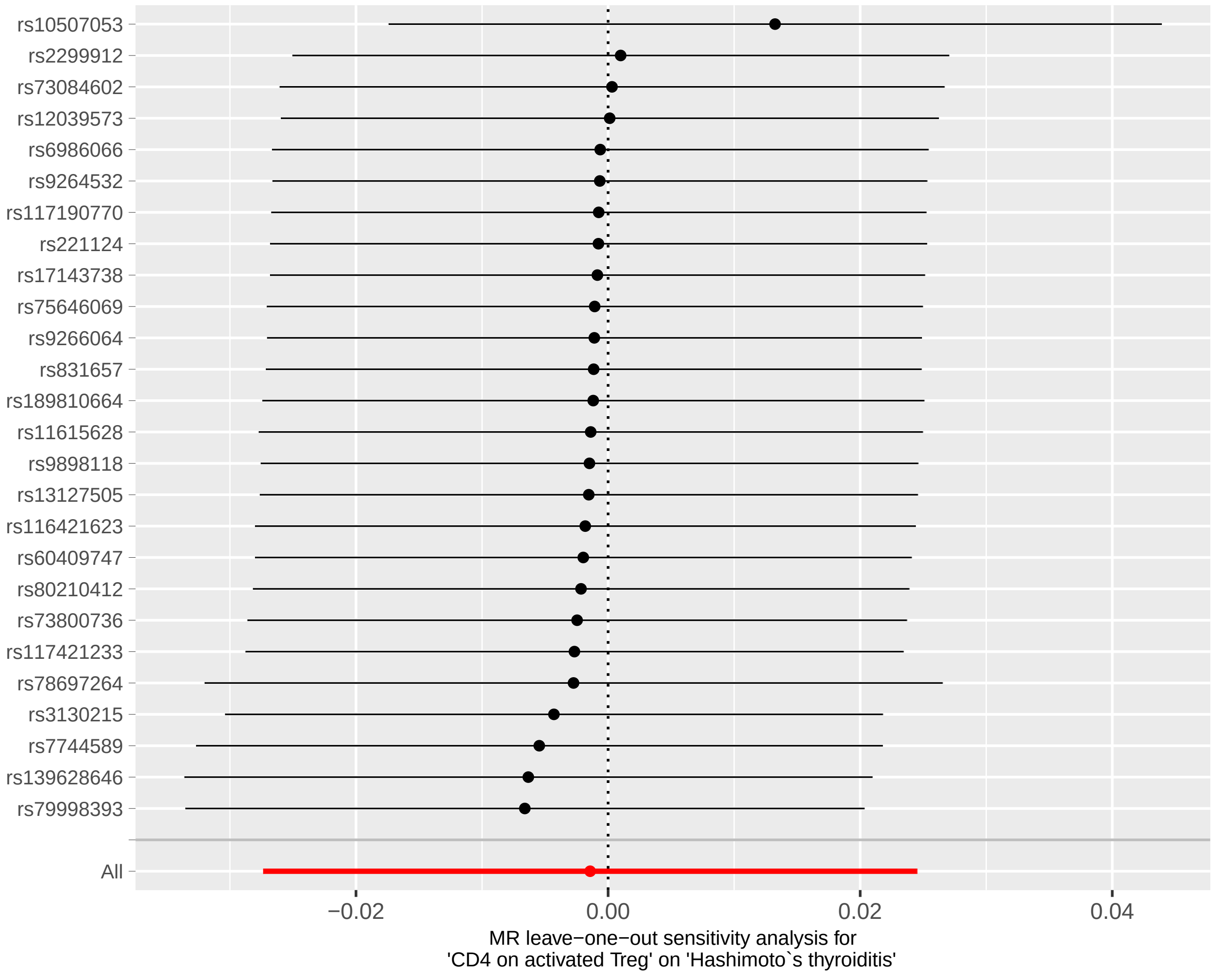
rs10507053
rs2299912
rs73084602
rs12039573
rs6986066
rs9264532
rs117190770
rs221124
rs17143738
rs75646069
rs9266064
rs831657
rs189810664
rs11615628
rs9898118
rs13127505
rs116421623
rs60409747
rs80210412
rs73800736
rs117421233
rs78697264
rs3130215
rs7744589
rs139628646
rs79998393
All
−0.02
0.00
0.02
0.04
MR leave−one−out sensitivity analysis for
'CD4 on activated Treg' on 'Hashimoto`s thyroiditis'

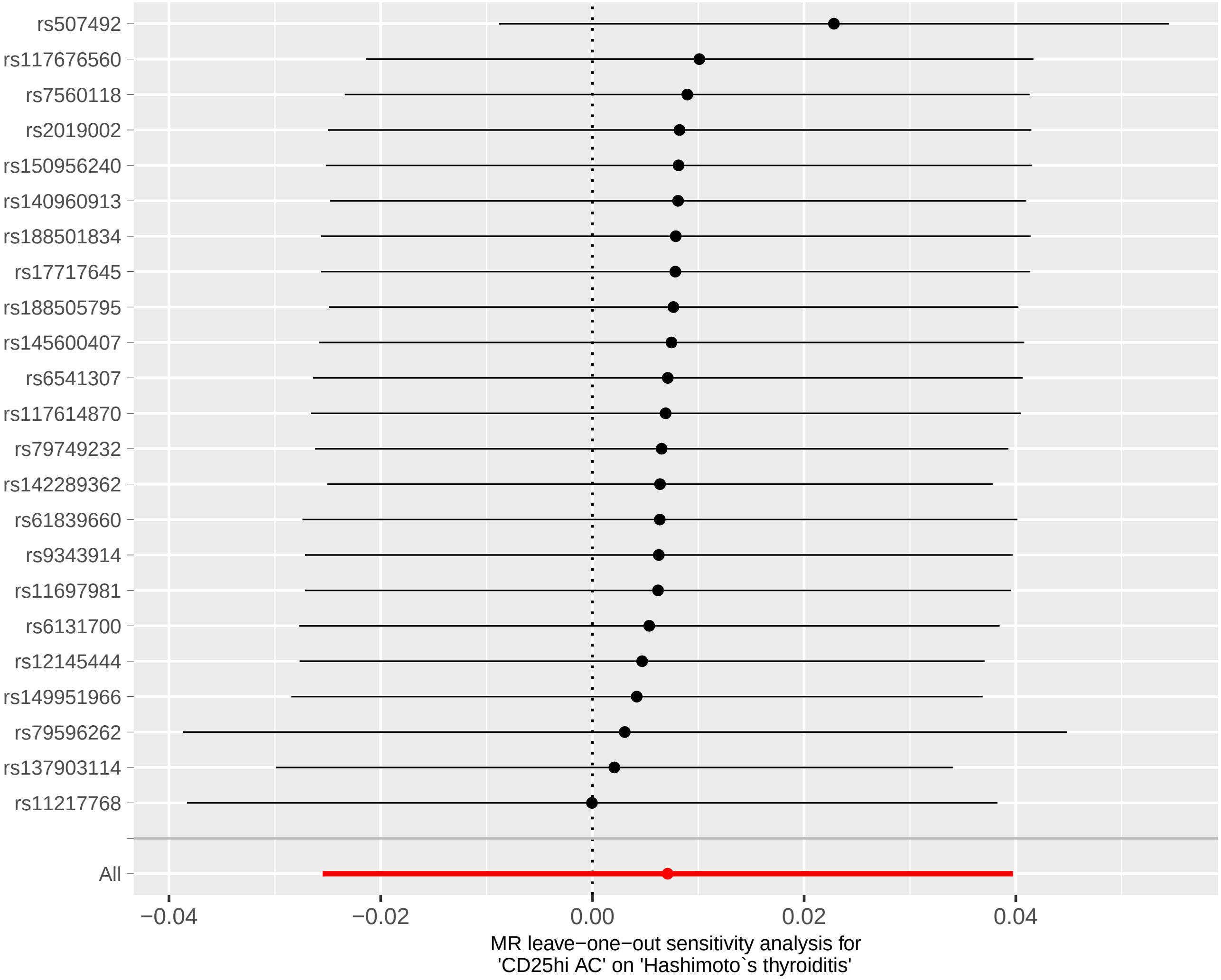
rs507492
rs117676560
rs7560118
rs2019002
rs150956240
rs140960913
rs188501834
rs17717645
rs188505795
rs145600407
rs6541307
rs117614870
rs79749232
rs142289362
rs61839660
rs9343914
rs11697981
rs6131700
rs12145444
rs149951966
rs79596262
rs137903114
rs11217768
All
−0.04
−0.02
0.00
0.02
0.04
MR leave−one−out sensitivity analysis for
'CD25hi AC' on 'Hashimoto`s thyroiditis'

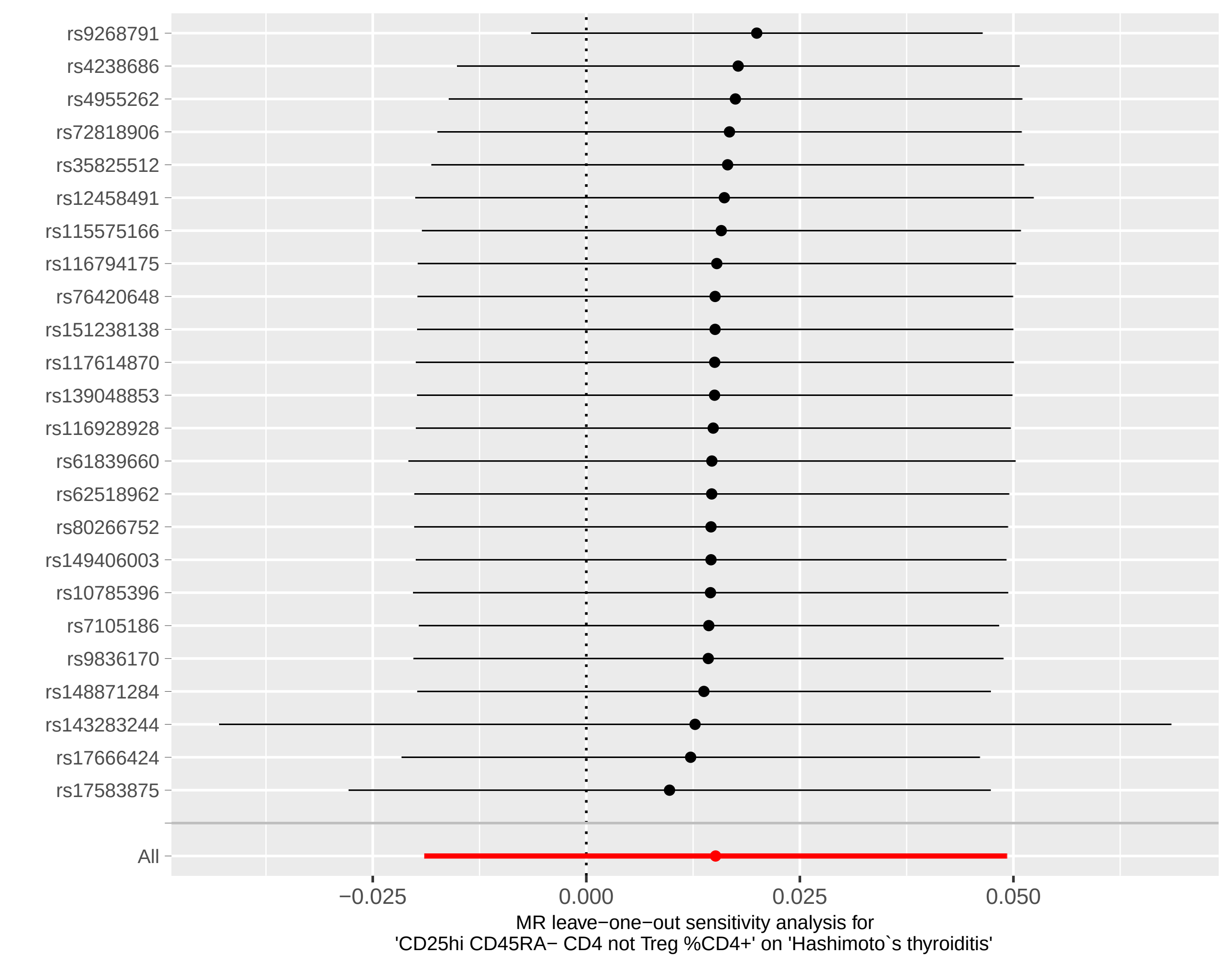
rs9268791
rs4238686
rs4955262
rs72818906
rs35825512
rs12458491
rs115575166
rs116794175
rs76420648
rs151238138
rs117614870
rs139048853
rs116928928
rs61839660
rs62518962
rs80266752
rs149406003
rs10785396
rs7105186
rs9836170
rs148871284
rs143283244
rs17666424
rs17583875
All
−0.025
0.000
0.025
0.050
MR leave−one−out sensitivity analysis for
'CD25hi CD45RA− CD4 not Treg %CD4+' on 'Hashimoto`s thyroiditis'

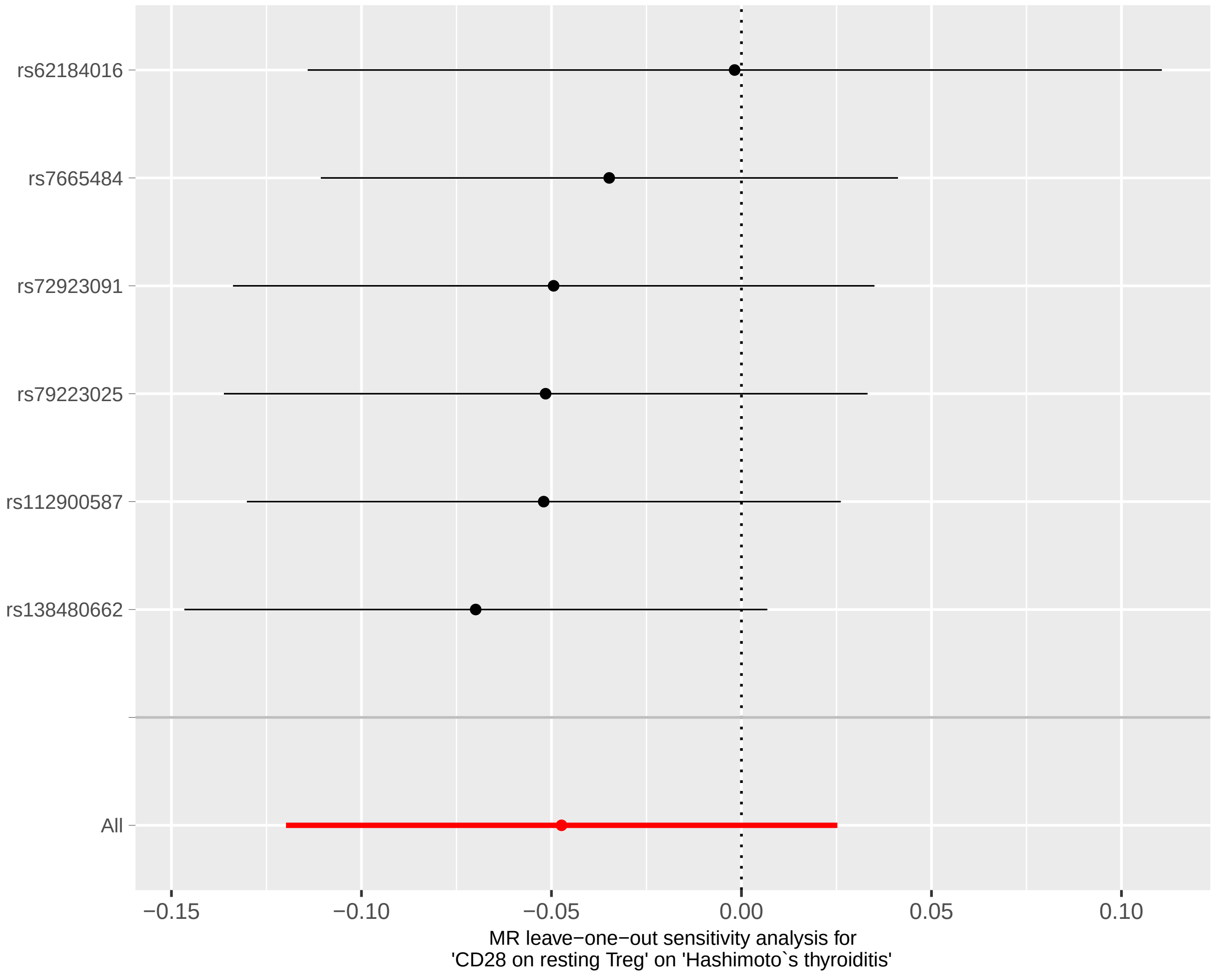
rs62184016
rs7665484
rs72923091
rs79223025
rs112900587
rs138480662
All
−0.15
−0.10
−0.05
0.00
0.05
0.10
MR leave−one−out sensitivity analysis for
'CD28 on resting Treg' on 'Hashimoto`s thyroiditis'

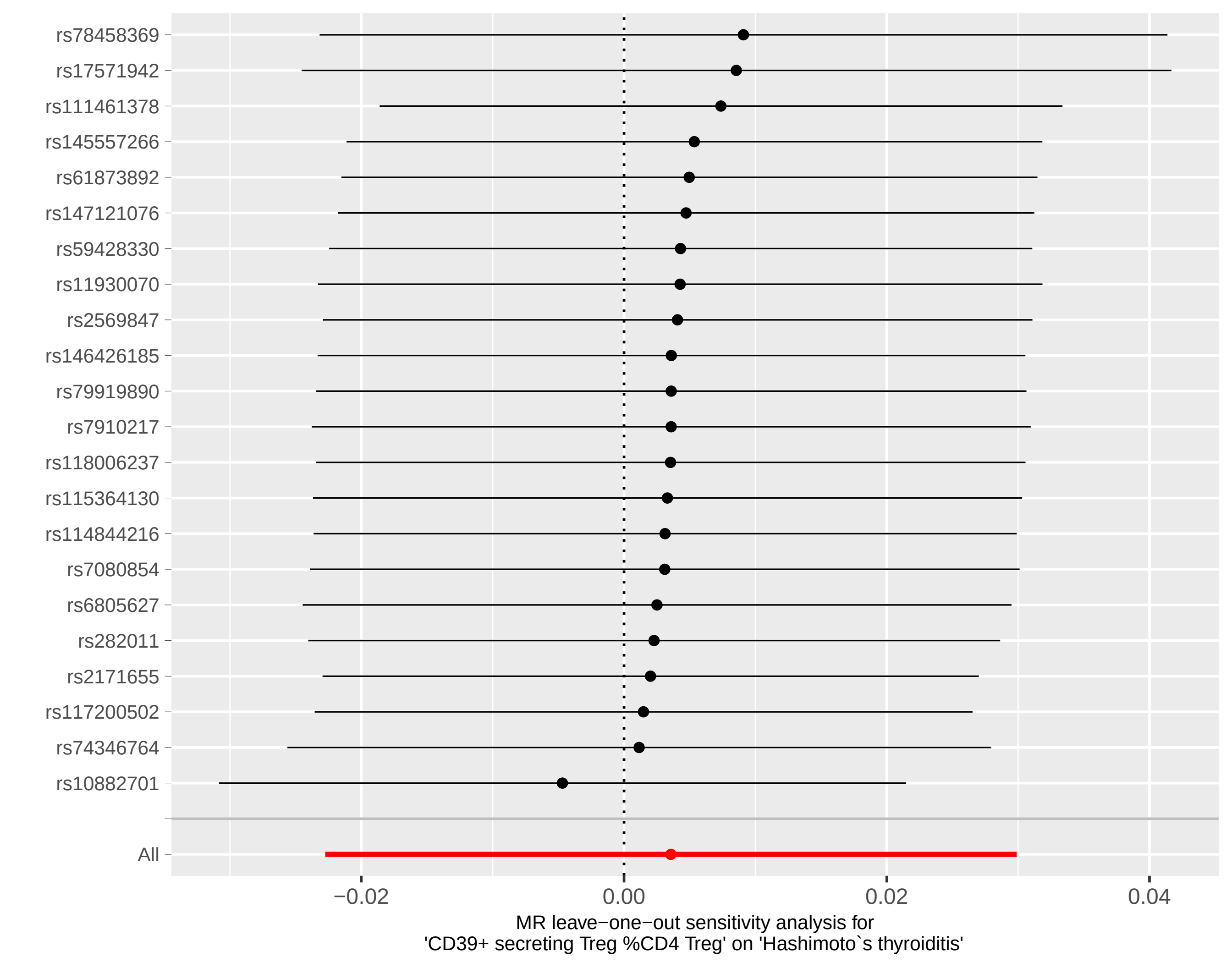
rs78458369
rs17571942
rs111461378
rs145557266
rs61873892
rs147121076
rs59428330
rs11930070
rs2569847
rs146426185
rs79919890
rs7910217
rs118006237
rs115364130
rs114844216
rs7080854
rs6805627
rs282011
rs2171655
rs117200502
rs74346764
rs10882701
All
−0.02
0.00
0.02
0.04
MR leave−one−out sensitivity analysis for
'CD39+ secreting Treg %CD4 Treg' on 'Hashimoto`s thyroiditis'

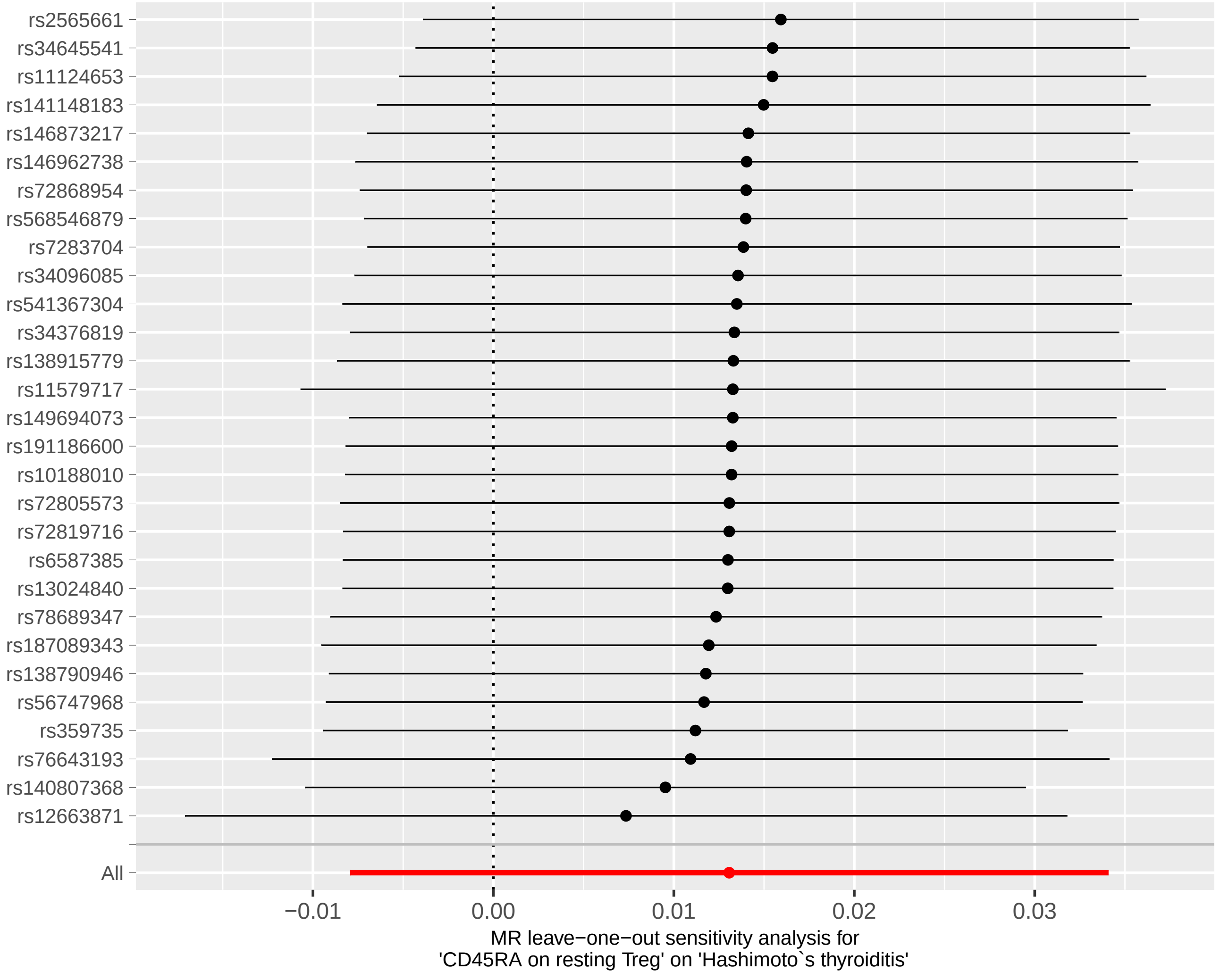
rs2565661
rs34645541
rs11124653
rs141148183
rs146873217
rs146962738
rs72868954
rs568546879
rs7283704
rs34096085
rs541367304
rs34376819
rs138915779
rs11579717
rs149694073
rs191186600
rs10188010
rs72805573
rs72819716
rs6587385
rs13024840
rs78689347
rs187089343
rs138790946
rs56747968
rs359735
rs76643193
rs140807368
rs12663871
All
−0.01
0.00
0.01
0.02
0.03
MR leave−one−out sensitivity analysis for
'CD45RA on resting Treg' on 'Hashimoto`s thyroiditis'

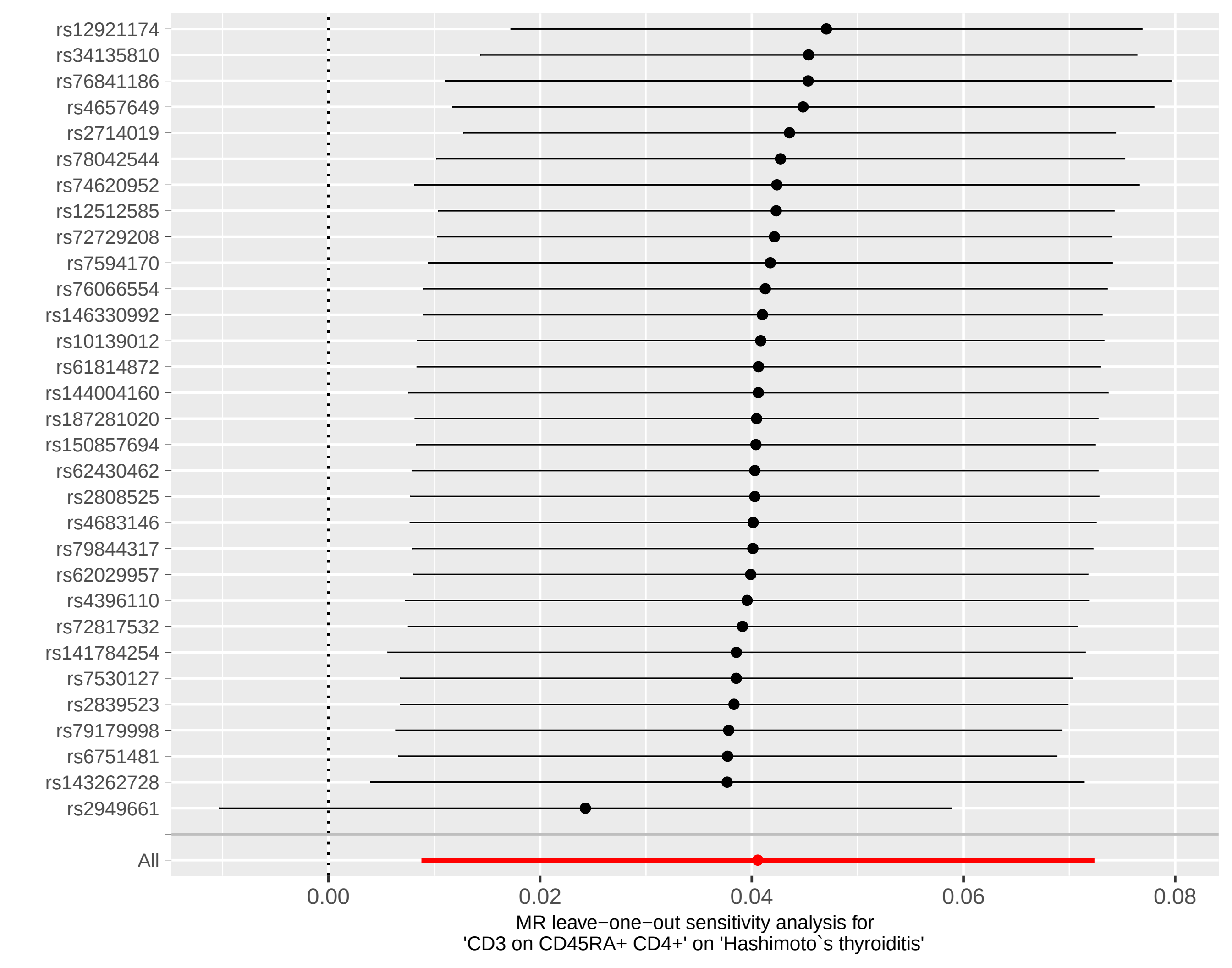
rs12921174
rs34135810
rs76841186
rs4657649
rs2714019
rs78042544
rs74620952
rs12512585
rs72729208
rs7594170
rs76066554
rs146330992
rs10139012
rs61814872
rs144004160
rs187281020
rs150857694
rs62430462
rs2808525
rs4683146
rs79844317
rs62029957
rs4396110
rs72817532
rs141784254
rs7530127
rs2839523
rs79179998
rs6751481
rs143262728
rs2949661
All
0.00
0.02
0.04
0.06
0.08
MR leave−one−out sensitivity analysis for
'CD3 on CD45RA+ CD4+' on 'Hashimoto`s thyroiditis'

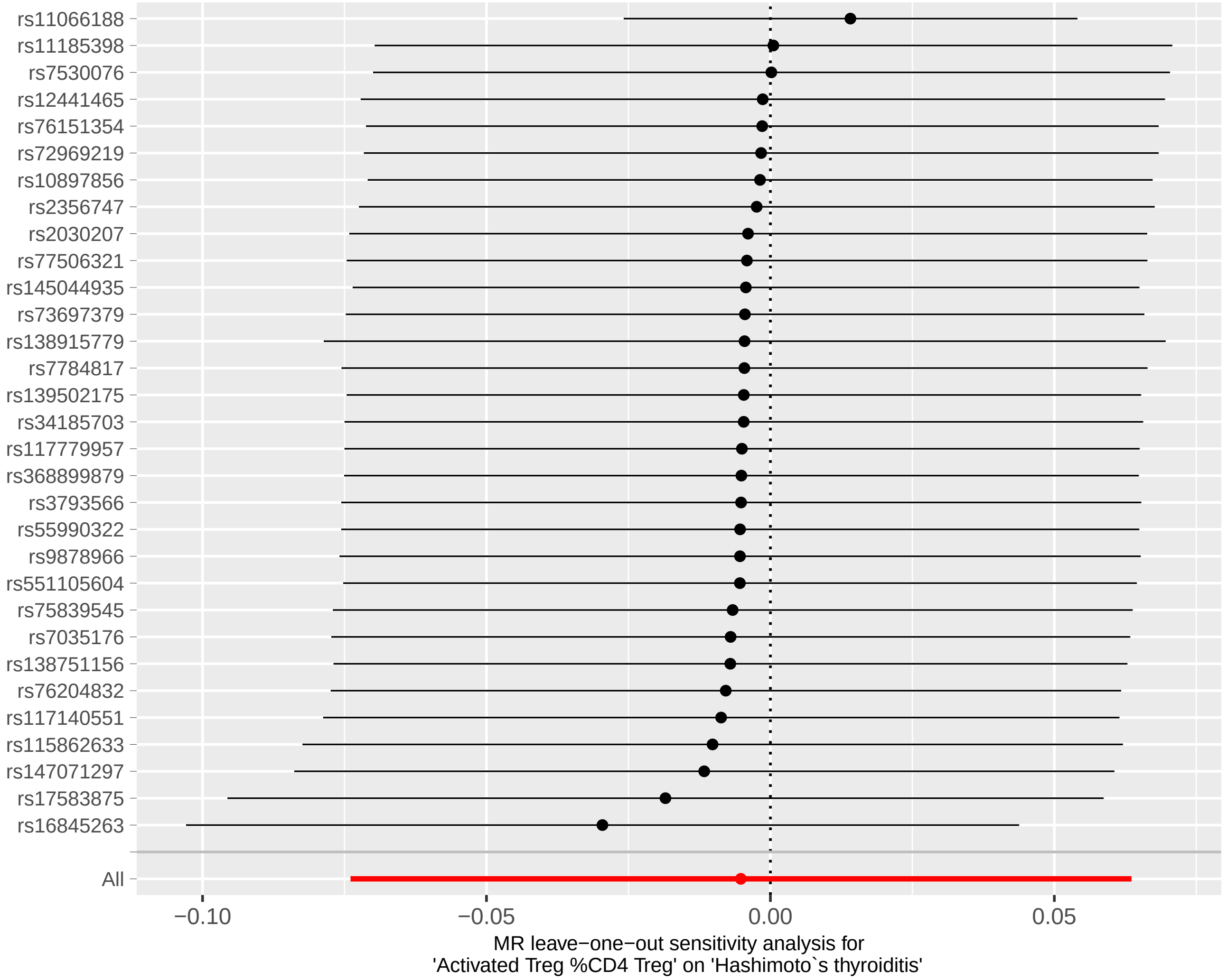

rs11066188
rs11185398
rs7530076
rs12441465
rs76151354
rs72969219
rs10897856
rs2356747
rs2030207
rs77506321
rs145044935
rs73697379
rs138915779
rs7784817
rs139502175
rs34185703
rs117779957
rs368899879
rs3793566
rs55990322
rs9878966
rs551105604
rs75839545
rs7035176
rs138751156
rs76204832
rs117140551
rs115862633
rs147071297
rs17583875
rs16845263
All
−0.10
−0.05
0.00
0.05
MR leave−one−out sensitivity analysis for
'Activated Treg %CD4 Treg' on 'Hashimoto`s thyroiditis'

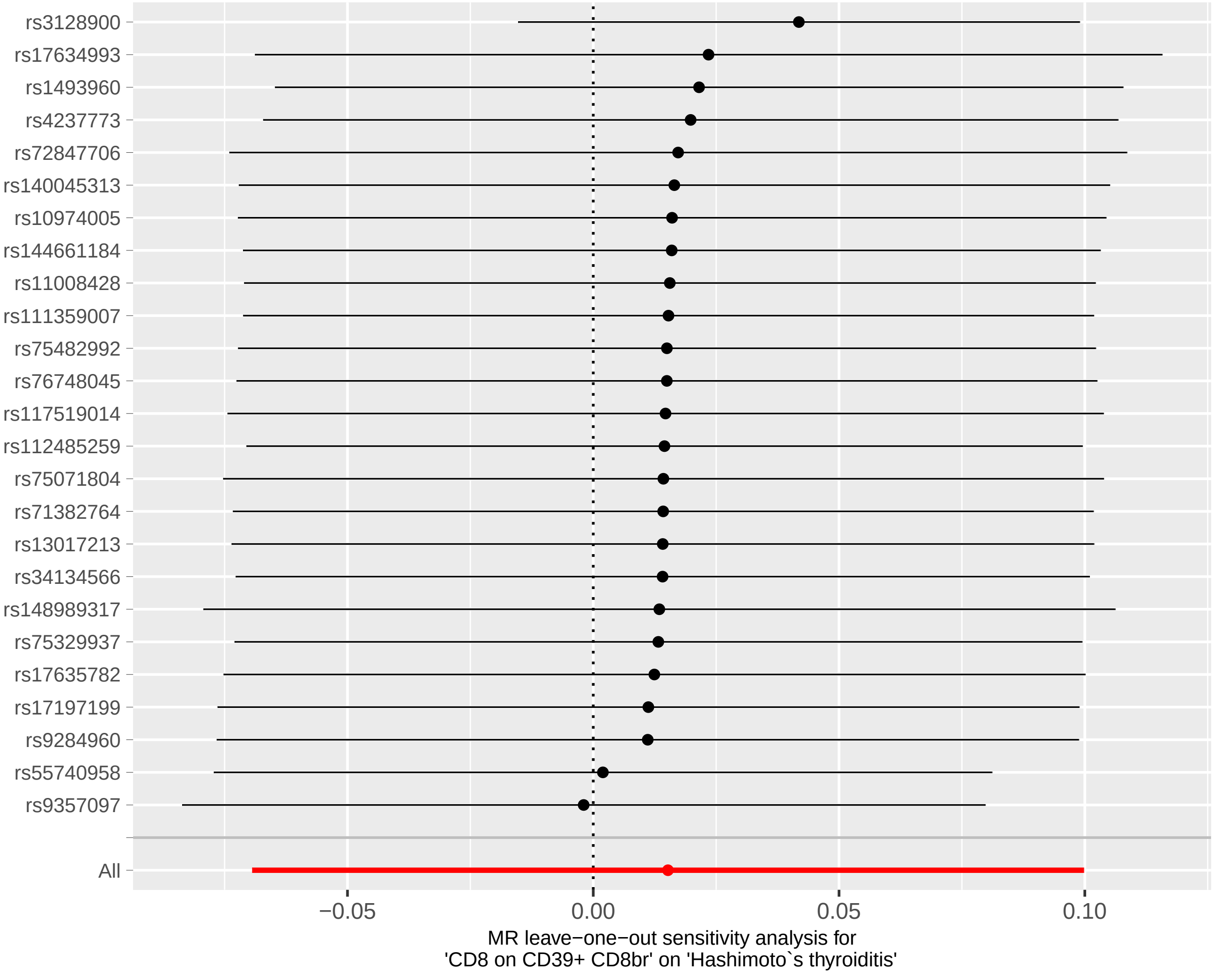
rs3128900
rs17634993
rs1493960
rs4237773
rs72847706
rs140045313
rs10974005
rs144661184
rs11008428
rs111359007
rs75482992
rs76748045
rs117519014
rs112485259
rs75071804
rs71382764
rs13017213
rs34134566
rs148989317
rs75329937
rs17635782
rs17197199
rs9284960
rs55740958
rs9357097
All
−0.05
0.00
0.05
0.10
MR leave−one−out sensitivity analysis for
'CD8 on CD39+ CD8br' on 'Hashimoto`s thyroiditis'

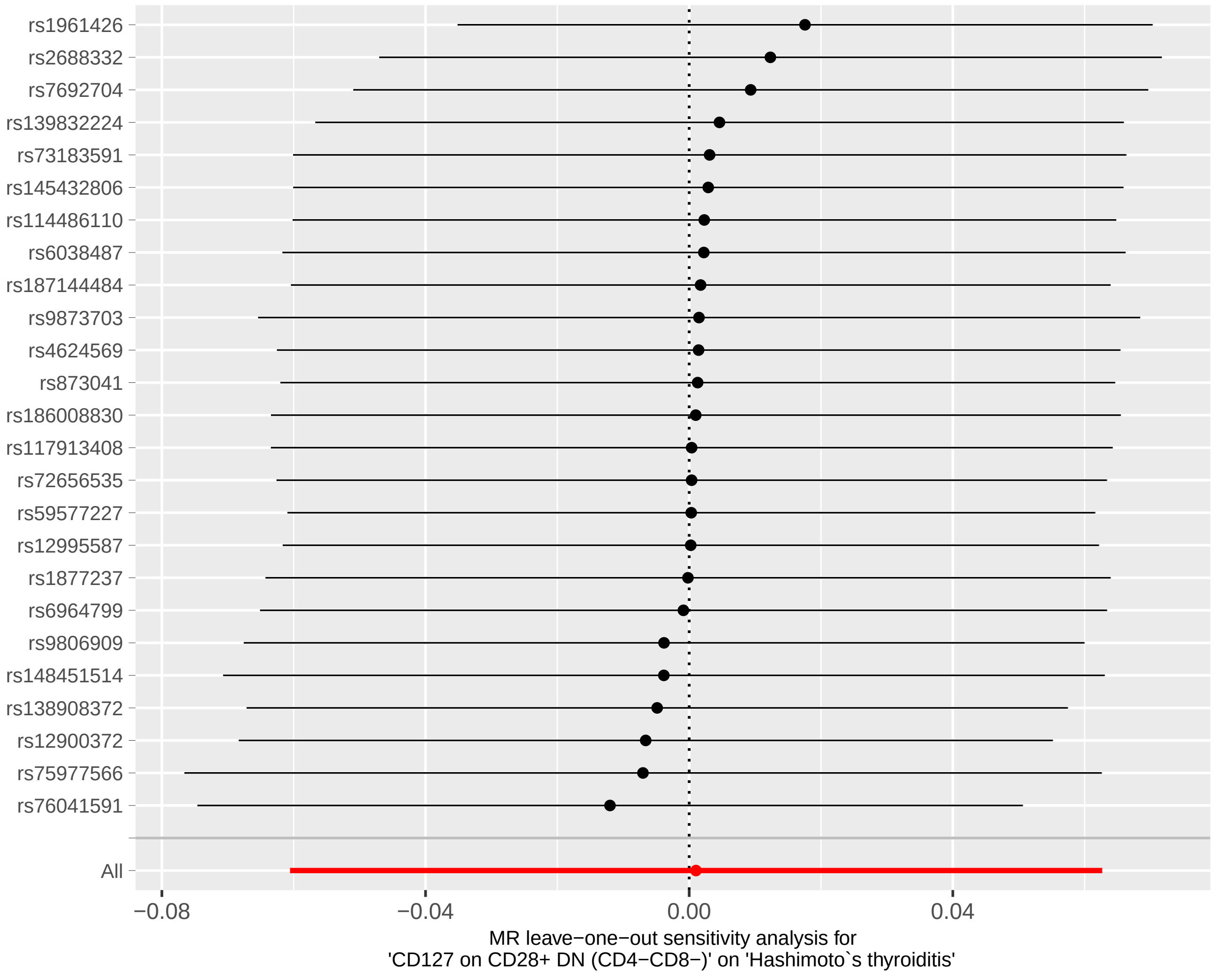

rs1961426
rs2688332
rs7692704
rs139832224
rs73183591
rs145432806
rs114486110
rs6038487
rs187144484
rs9873703
rs4624569
rs873041
rs186008830
rs117913408
rs72656535
rs59577227
rs12995587
rs1877237
rs6964799
rs9806909
rs148451514
rs138908372
rs12900372
rs75977566
rs76041591
All
−0.08
−0.04
0.00
0.04
MR leave−one−out sensitivity analysis for
'CD127 on CD28+ DN (CD4−CD8−)' on 'Hashimoto`s thyroiditis'

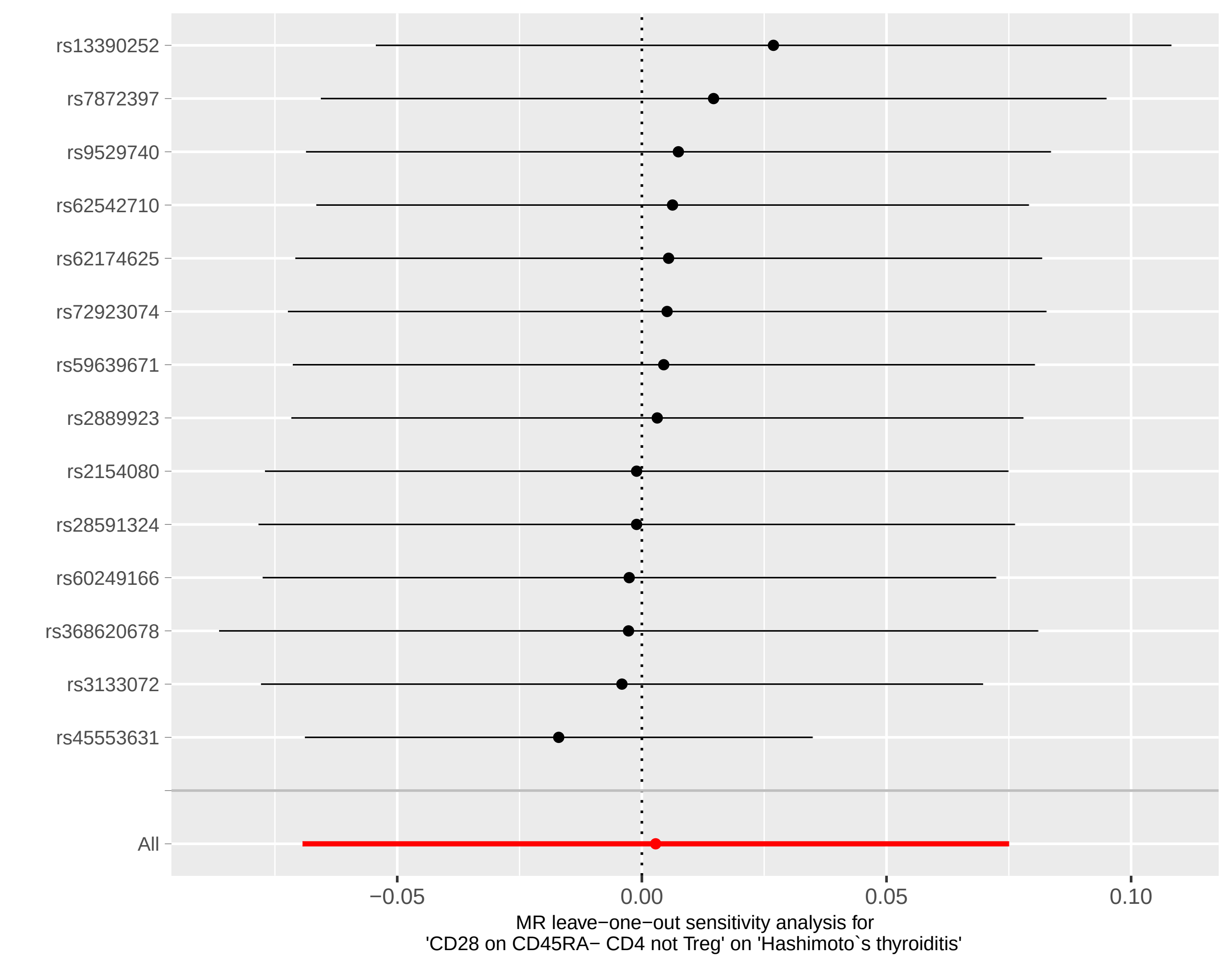
rs13390252
rs7872397
rs9529740
rs62542710
rs62174625
rs72923074
rs59639671
rs2889923
rs2154080
rs28591324
rs60249166
rs368620678
rs3133072
rs45553631
All
−0.05
0.00
0.05
0.10
MR leave−one−out sensitivity analysis for
'CD28 on CD45RA− CD4 not Treg' on 'Hashimoto`s thyroiditis'

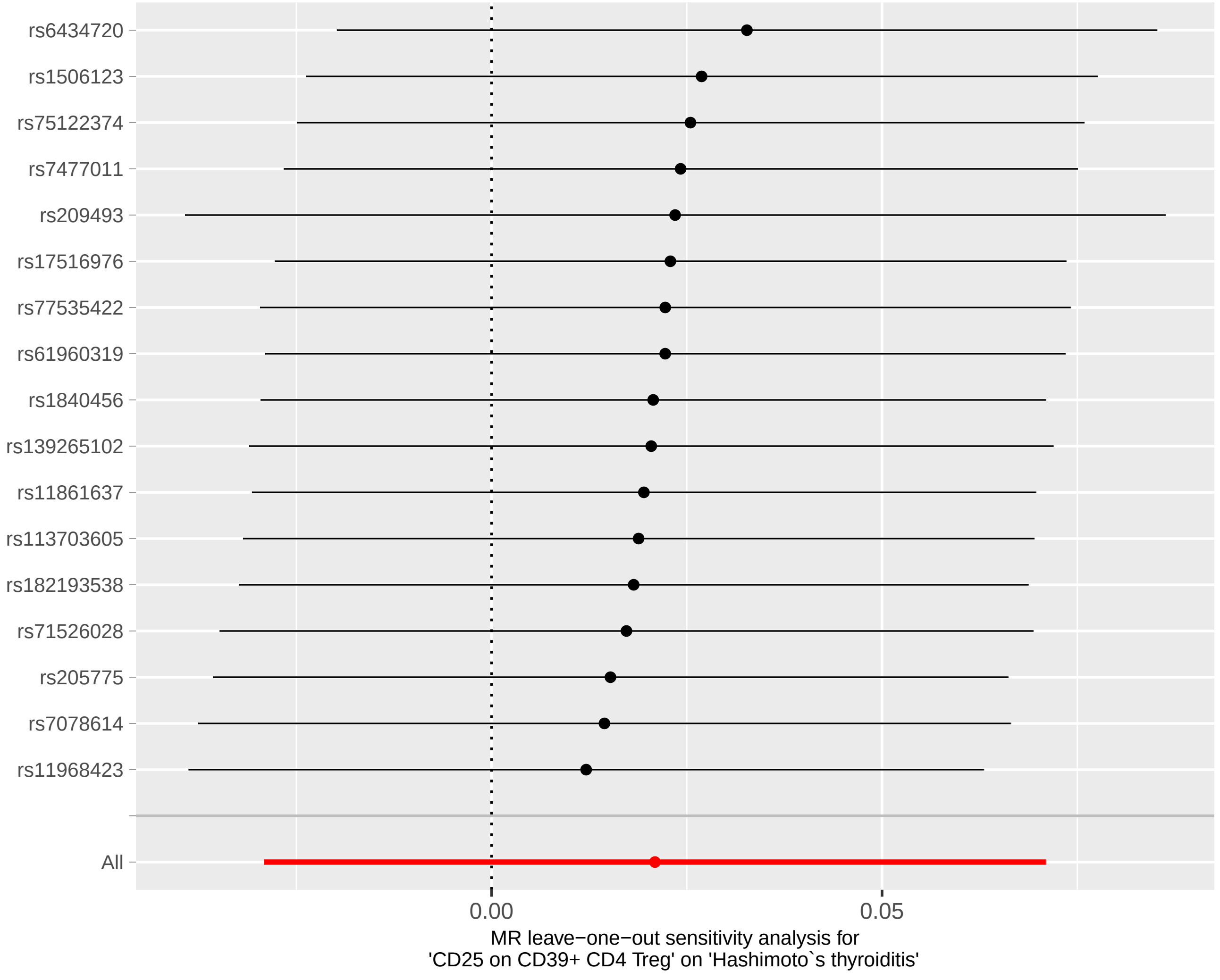
rs6434720
rs1506123
rs75122374
rs7477011
rs209493
rs17516976
rs77535422
rs61960319
rs1840456
rs139265102
rs11861637
rs113703605
rs182193538
rs71526028
rs205775
rs7078614
rs11968423
All
0.00
0.05
MR leave−one−out sensitivity analysis for
'CD25 on CD39+ CD4 Treg' on 'Hashimoto`s thyroiditis'

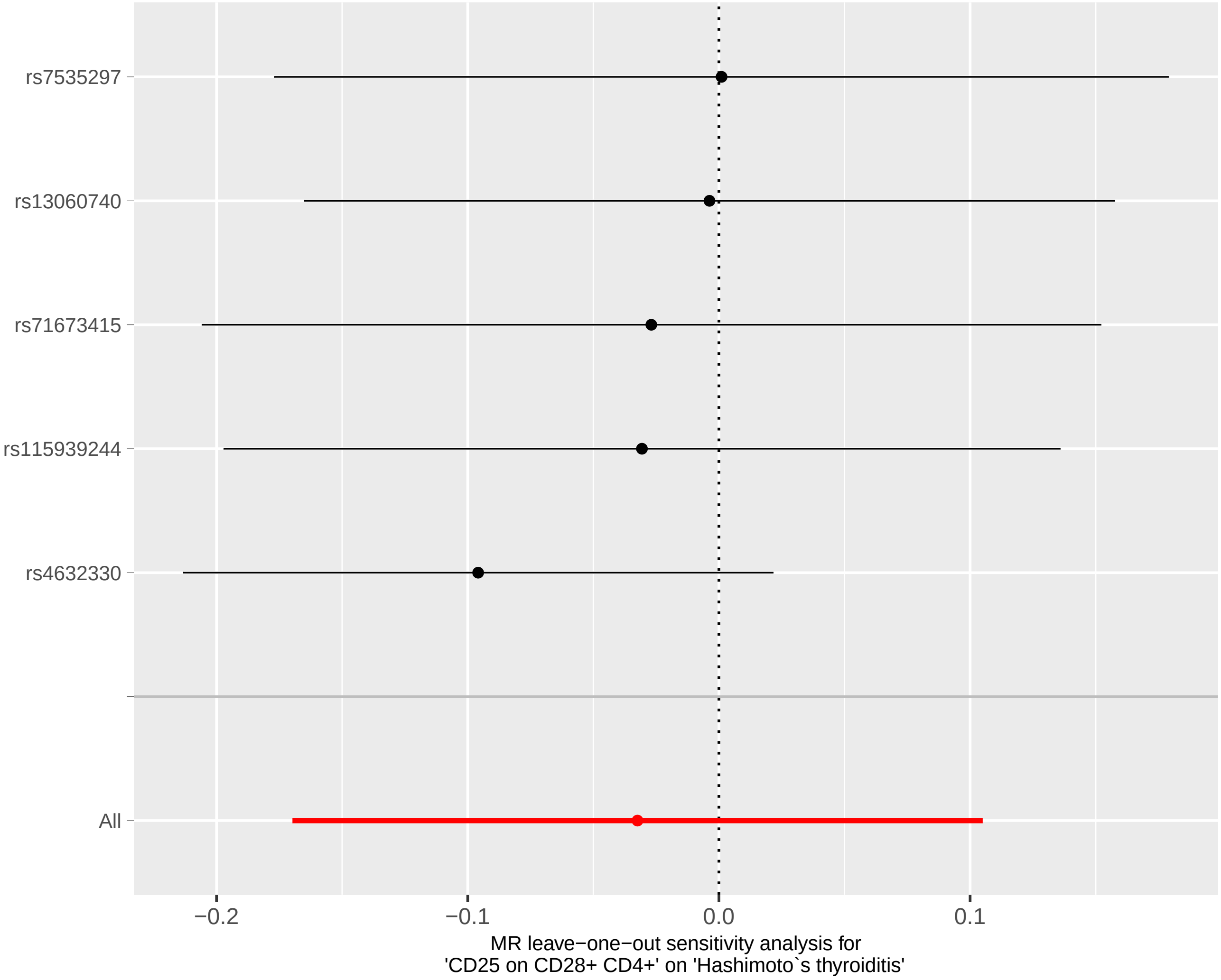

rs7535297
rs13060740
rs71673415
rs115939244
rs4632330
All
−0.2
−0.1
0.0
0.1
MR leave−one−out sensitivity analysis for
'CD25 on CD28+ CD4+' on 'Hashimoto`s thyroiditis'

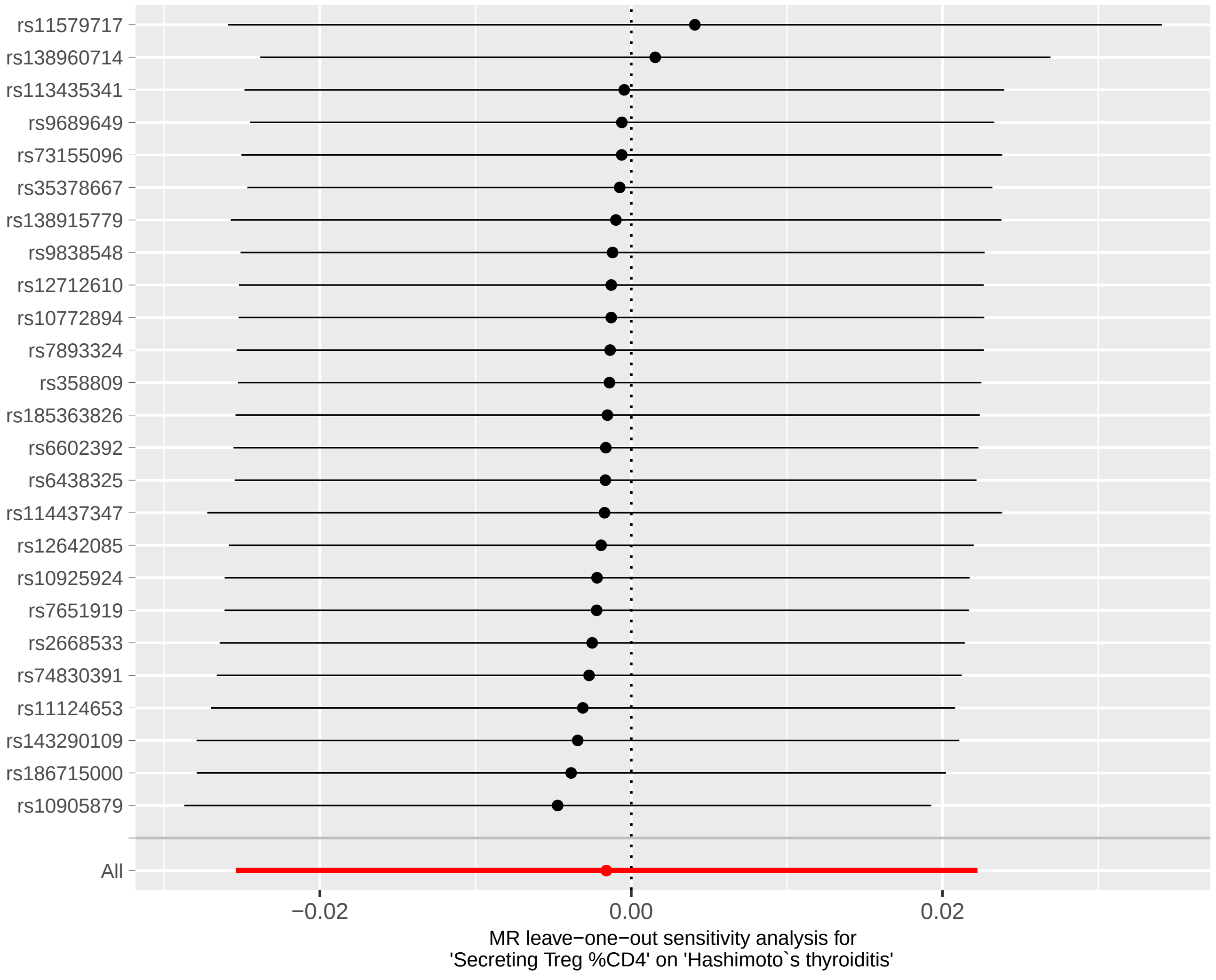
rs11579717
rs138960714
rs113435341
rs9689649
rs73155096
rs35378667
rs138915779
rs9838548
rs12712610
rs10772894
rs7893324
rs358809
rs185363826
rs6602392
rs6438325
rs114437347
rs12642085
rs10925924
rs7651919
rs2668533
rs74830391
rs11124653
rs143290109
rs186715000
rs10905879
All
−0.02
0.00
0.02
MR leave−one−out sensitivity analysis for
'Secreting Treg %CD4' on 'Hashimoto`s thyroiditis'

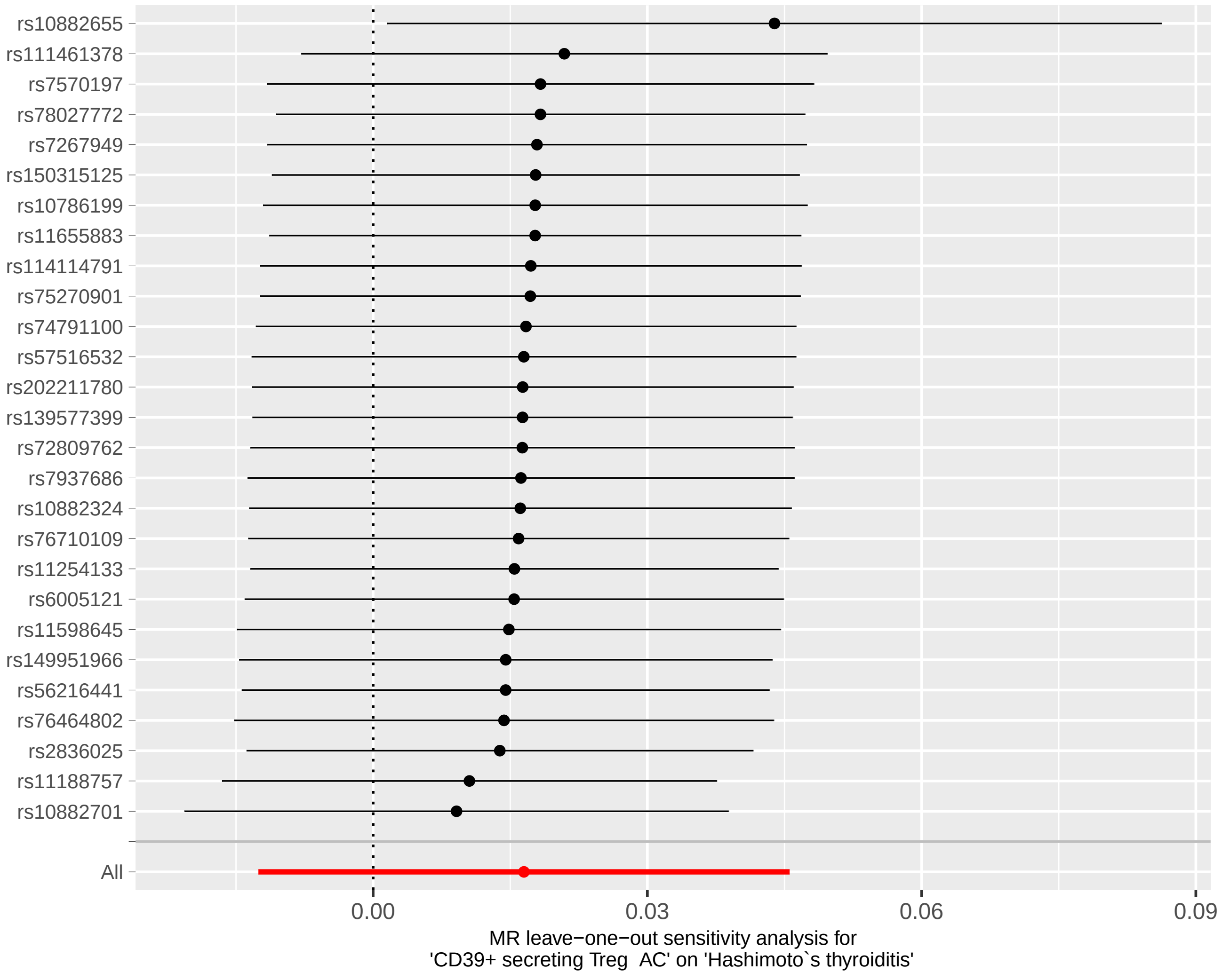

rs10882655
rs111461378
rs7570197
rs78027772
rs7267949
rs150315125
rs10786199
rs11655883
rs114114791
rs75270901
rs74791100
rs57516532
rs202211780
rs139577399
rs72809762
rs7937686
rs10882324
rs76710109
rs11254133
rs6005121
rs11598645
rs149951966
rs56216441
rs76464802
rs2836025
rs11188757
rs10882701
All
0.00
0.03
0.06
0.09
MR leave−one−out sensitivity analysis for
'CD39+ secreting Treg AC' on 'Hashimoto`s thyroiditis'

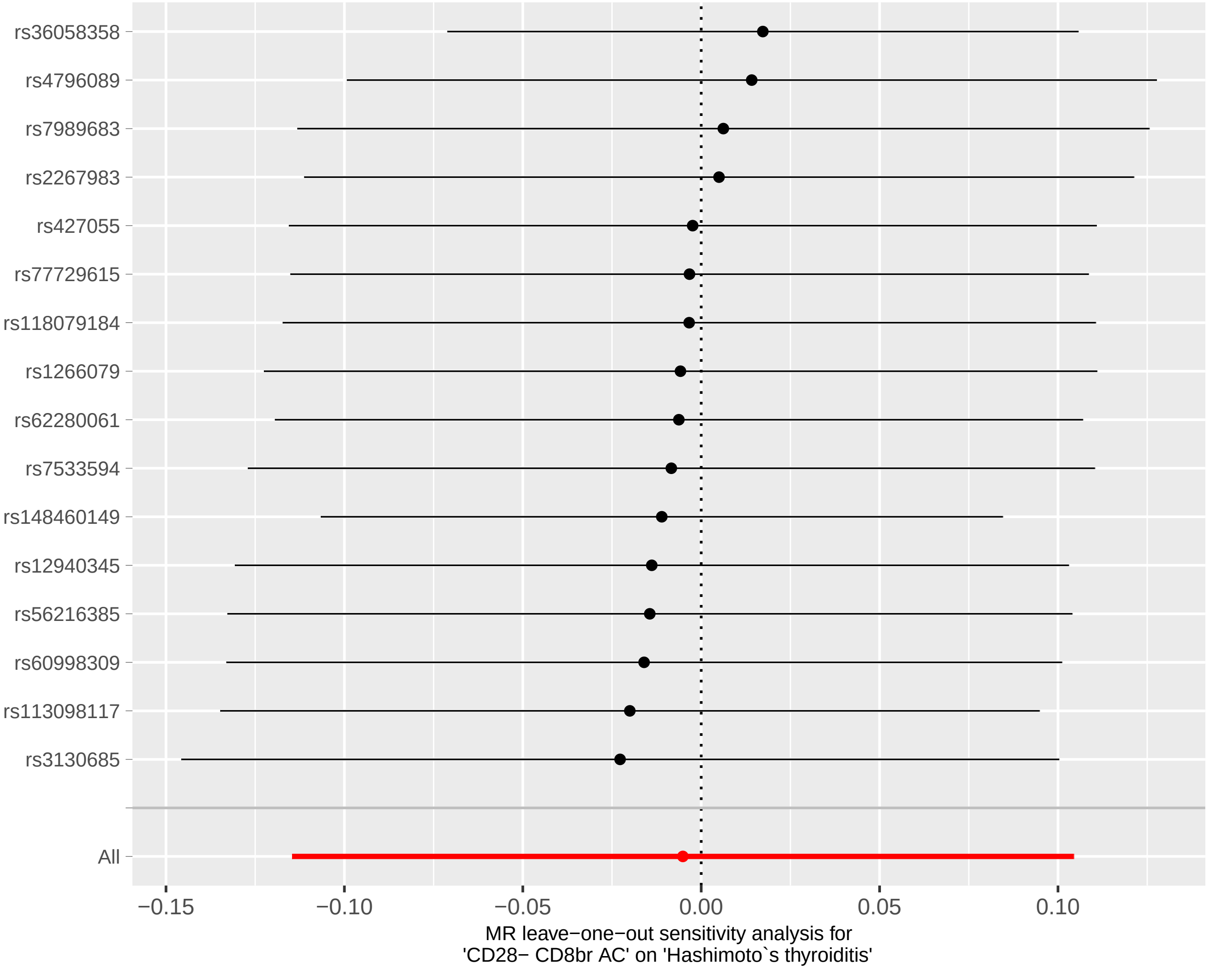
rs36058358
rs4796089
rs7989683
rs2267983
rs427055
rs77729615
rs118079184
rs1266079
rs62280061
rs7533594
rs148460149
rs12940345
rs56216385
rs60998309
rs113098117
rs3130685
All
−0.15
−0.10
−0.05
0.00
0.05
0.10
MR leave−one−out sensitivity analysis for
'CD28− CD8br AC' on 'Hashimoto`s thyroiditis'

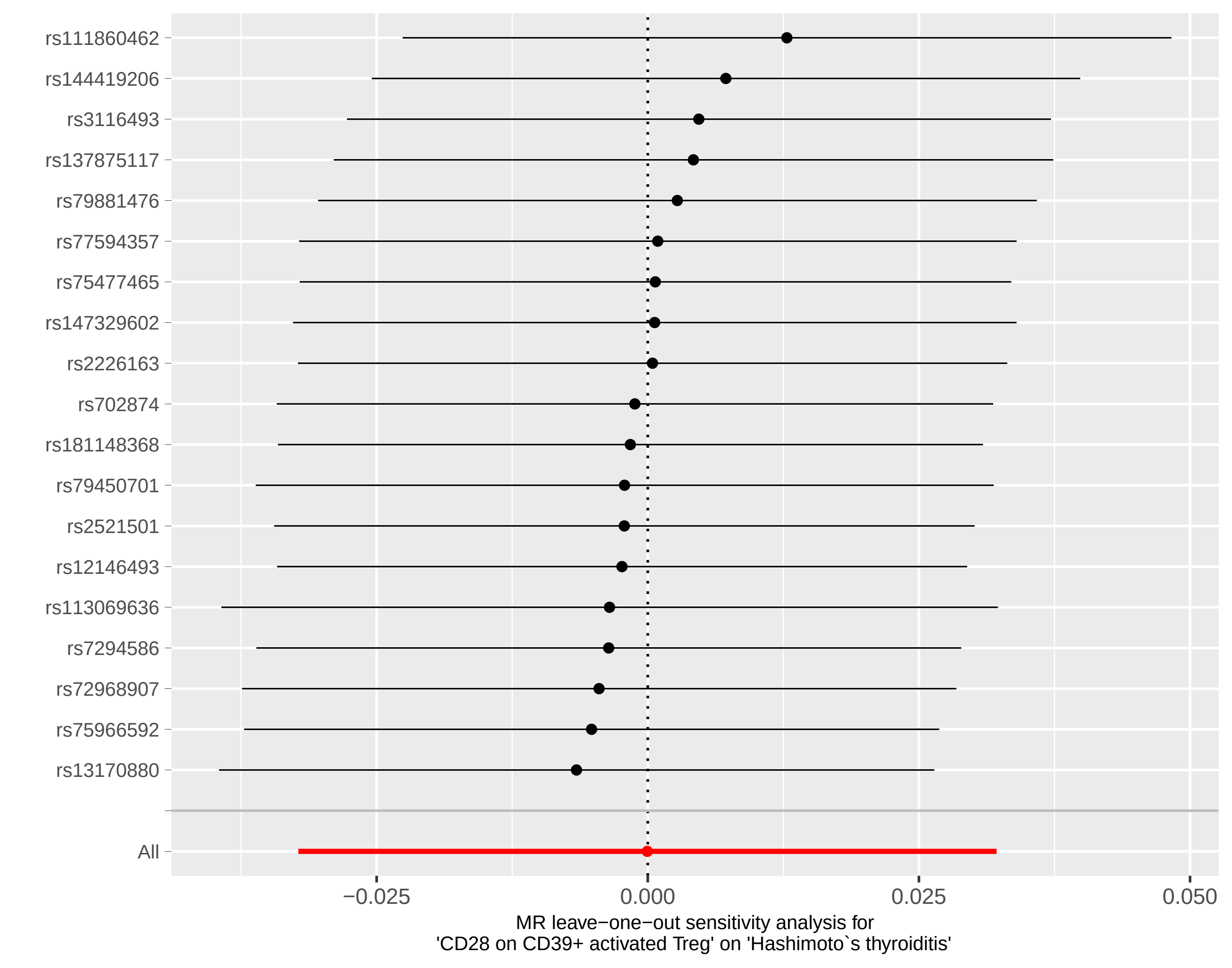

rs111860462
rs144419206
rs3116493
rs137875117
rs79881476
rs77594357
rs75477465
rs147329602
rs2226163
rs702874
rs181148368
rs79450701
rs2521501
rs12146493
rs113069636
rs7294586
rs72968907
rs75966592
rs13170880
All
−0.025
0.000
0.025
0.050
MR leave−one−out sensitivity analysis for
'CD28 on CD39+ activated Treg' on 'Hashimoto`s thyroiditis'

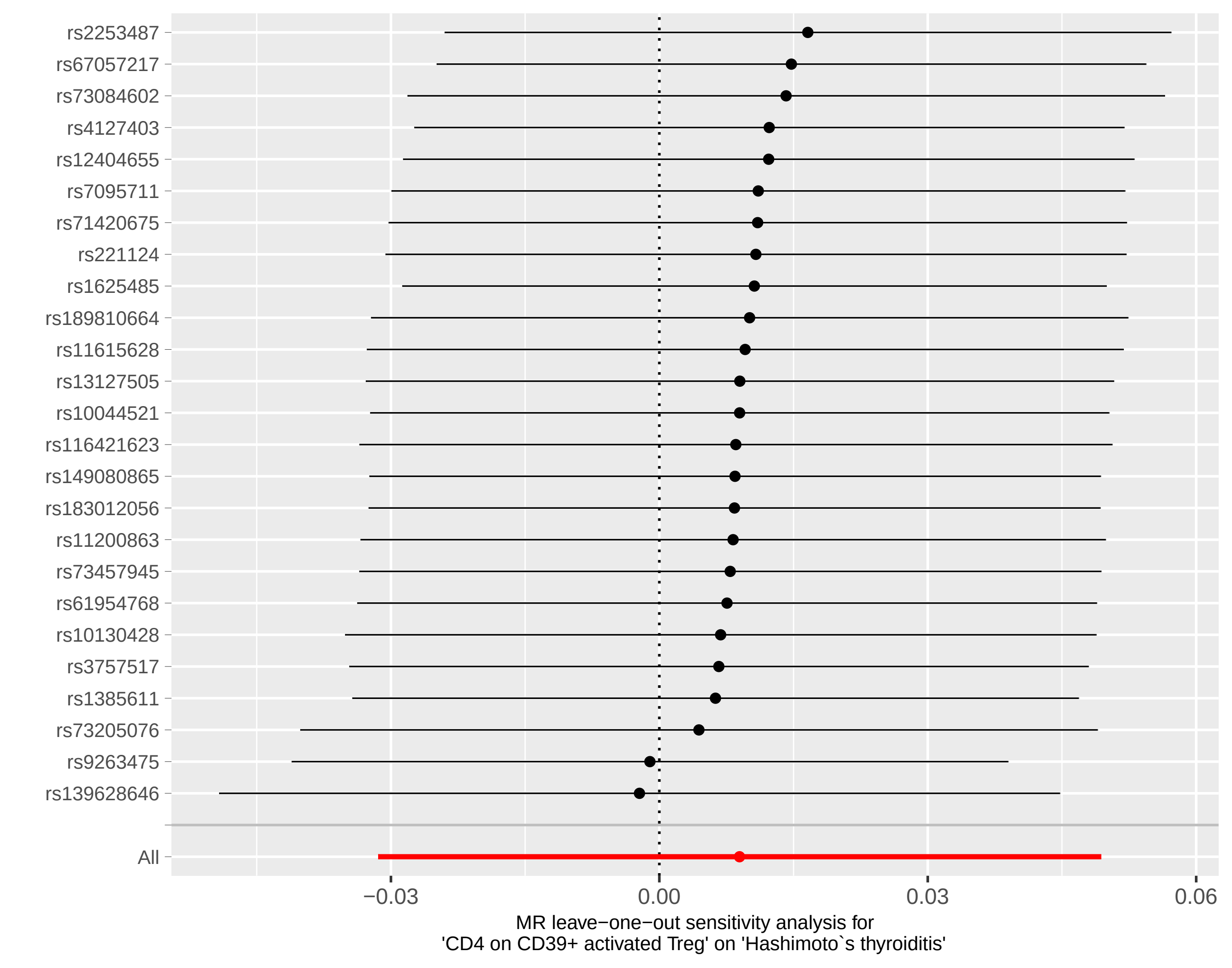

rs2253487
rs67057217
rs73084602
rs4127403
rs12404655
rs7095711
rs71420675
rs221124
rs1625485
rs189810664
rs11615628
rs13127505
rs10044521
rs116421623
rs149080865
rs183012056
rs11200863
rs73457945
rs61954768
rs10130428
rs3757517
rs1385611
rs73205076
rs9263475
rs139628646
All
−0.03
0.00
0.03
0.06
MR leave−one−out sensitivity analysis for
'CD4 on CD39+ activated Treg' on 'Hashimoto`s thyroiditis'

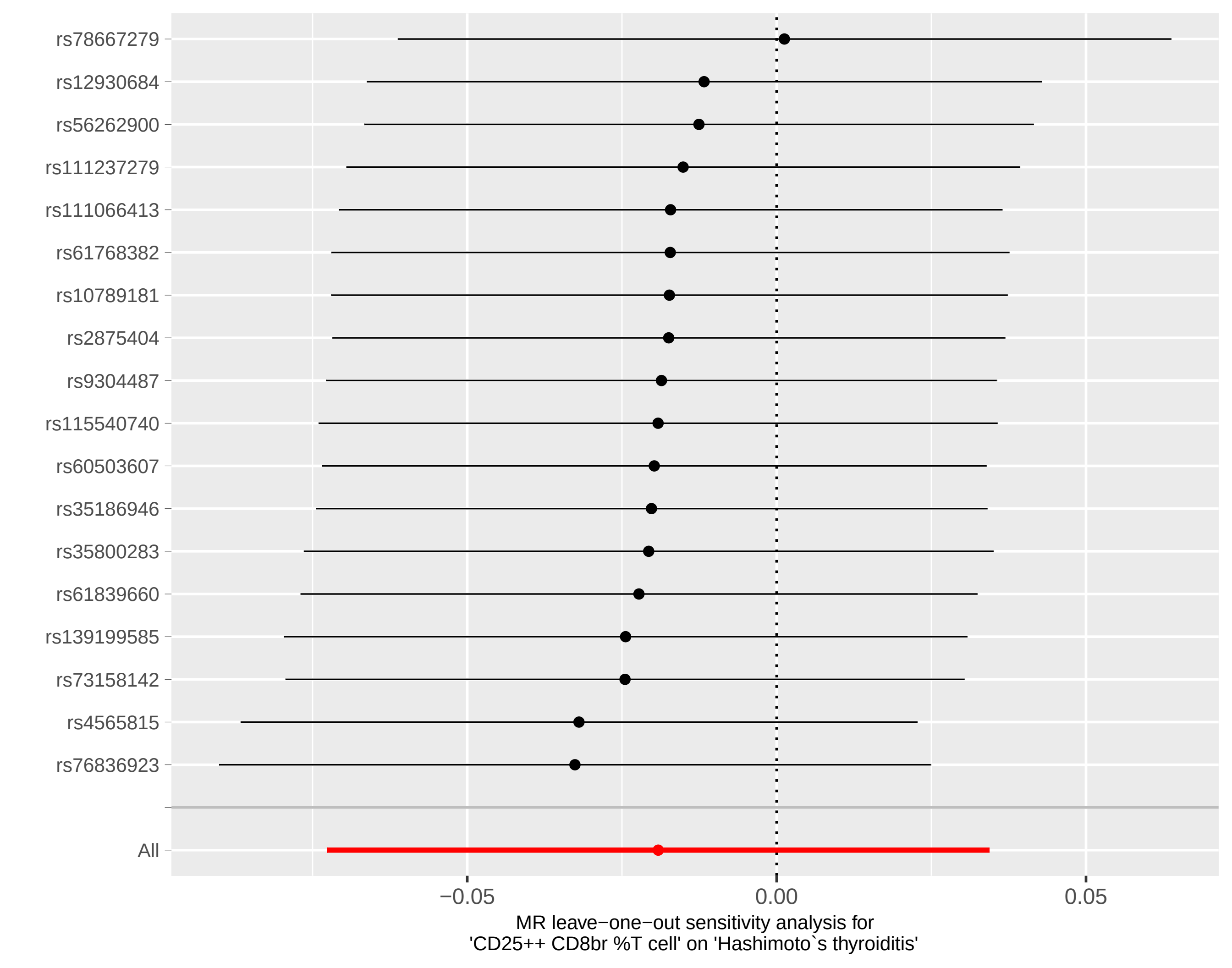
rs78667279
rs12930684
rs56262900
rs111237279
rs111066413
rs61768382
rs10789181
rs2875404
rs9304487
rs115540740
rs60503607
rs35186946
rs35800283
rs61839660
rs139199585
rs73158142
rs4565815
rs76836923
All
−0.05
0.00
0.05
MR leave−one−out sensitivity analysis for
'CD25++ CD8br %T cell' on 'Hashimoto`s thyroiditis'

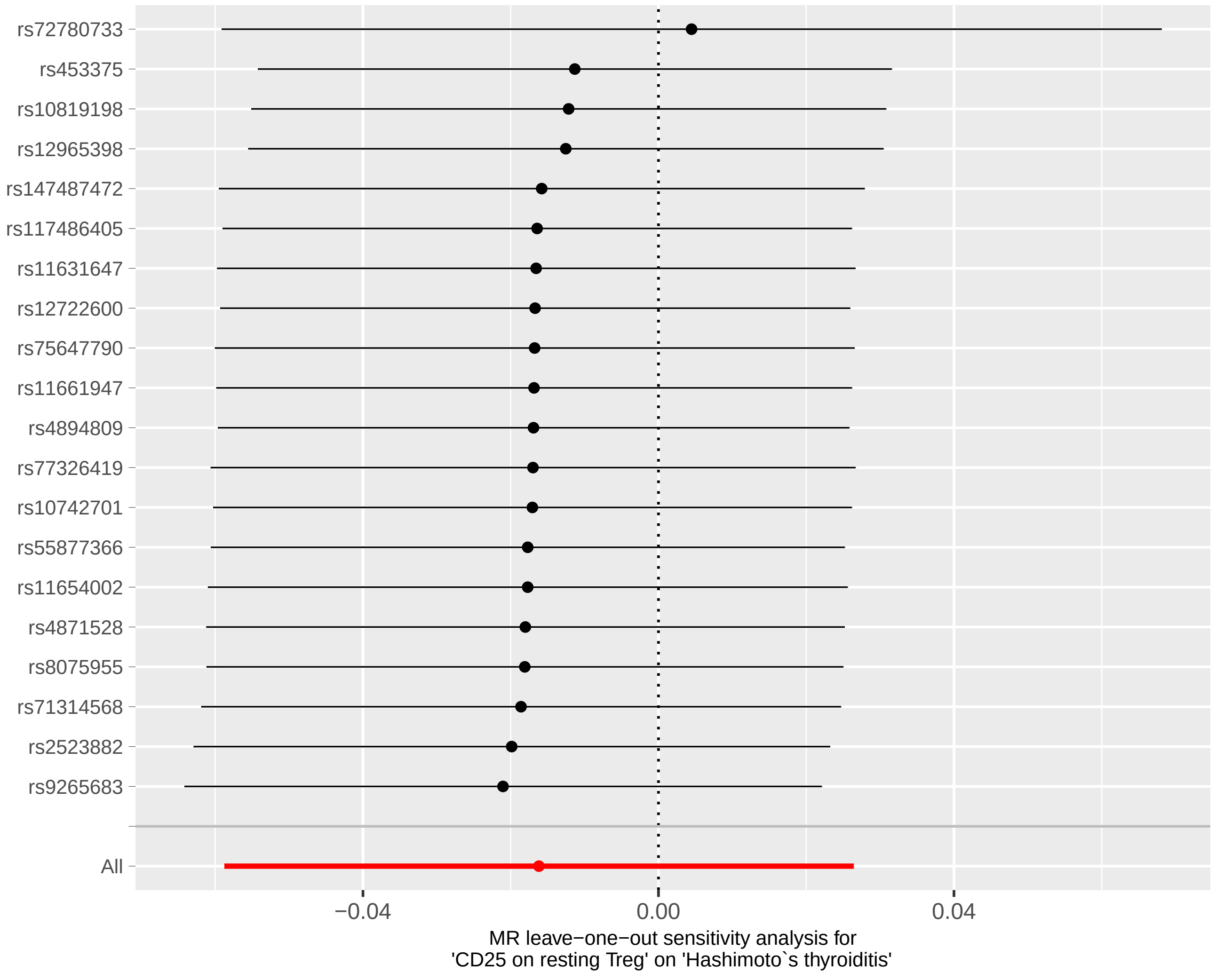
rs72780733
rs453375
rs10819198
rs12965398
rs147487472
rs117486405
rs11631647
rs12722600
rs75647790
rs11661947
rs4894809
rs77326419
rs10742701
rs55877366
rs11654002
rs4871528
rs8075955
rs71314568
rs2523882
rs9265683
All
−0.04
0.00
0.04
MR leave−one−out sensitivity analysis for
'CD25 on resting Treg' on 'Hashimoto`s thyroiditis'

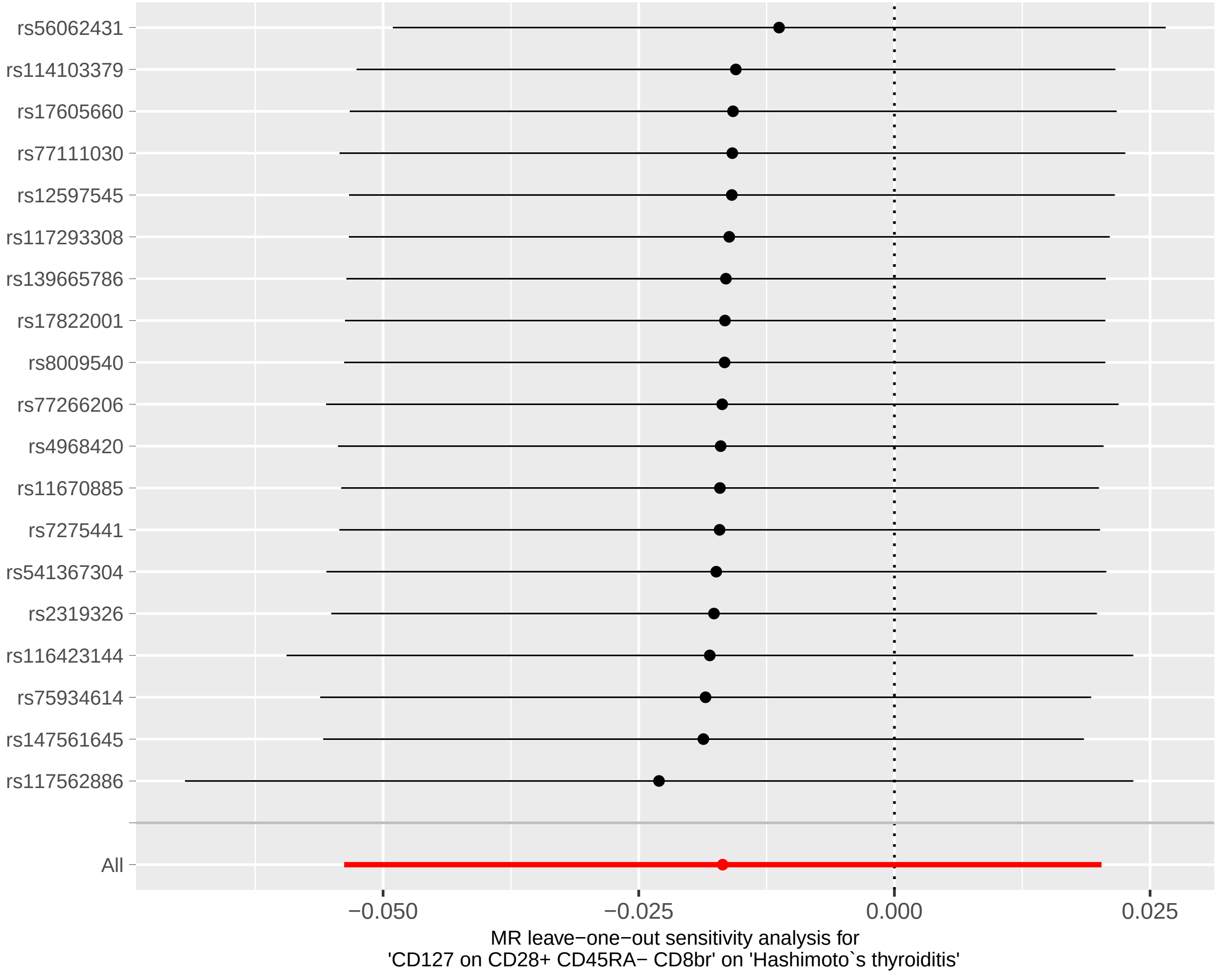

rs56062431
rs114103379
rs17605660
rs77111030
rs12597545
rs117293308
rs139665786
rs17822001
rs8009540
rs77266206
rs4968420
rs11670885
rs7275441
rs541367304
rs2319326
rs116423144
rs75934614
rs147561645
rs117562886
All
−0.050
−0.025
0.000
0.025
MR leave−one−out sensitivity analysis for
'CD127 on CD28+ CD45RA− CD8br' on 'Hashimoto`s thyroiditis'

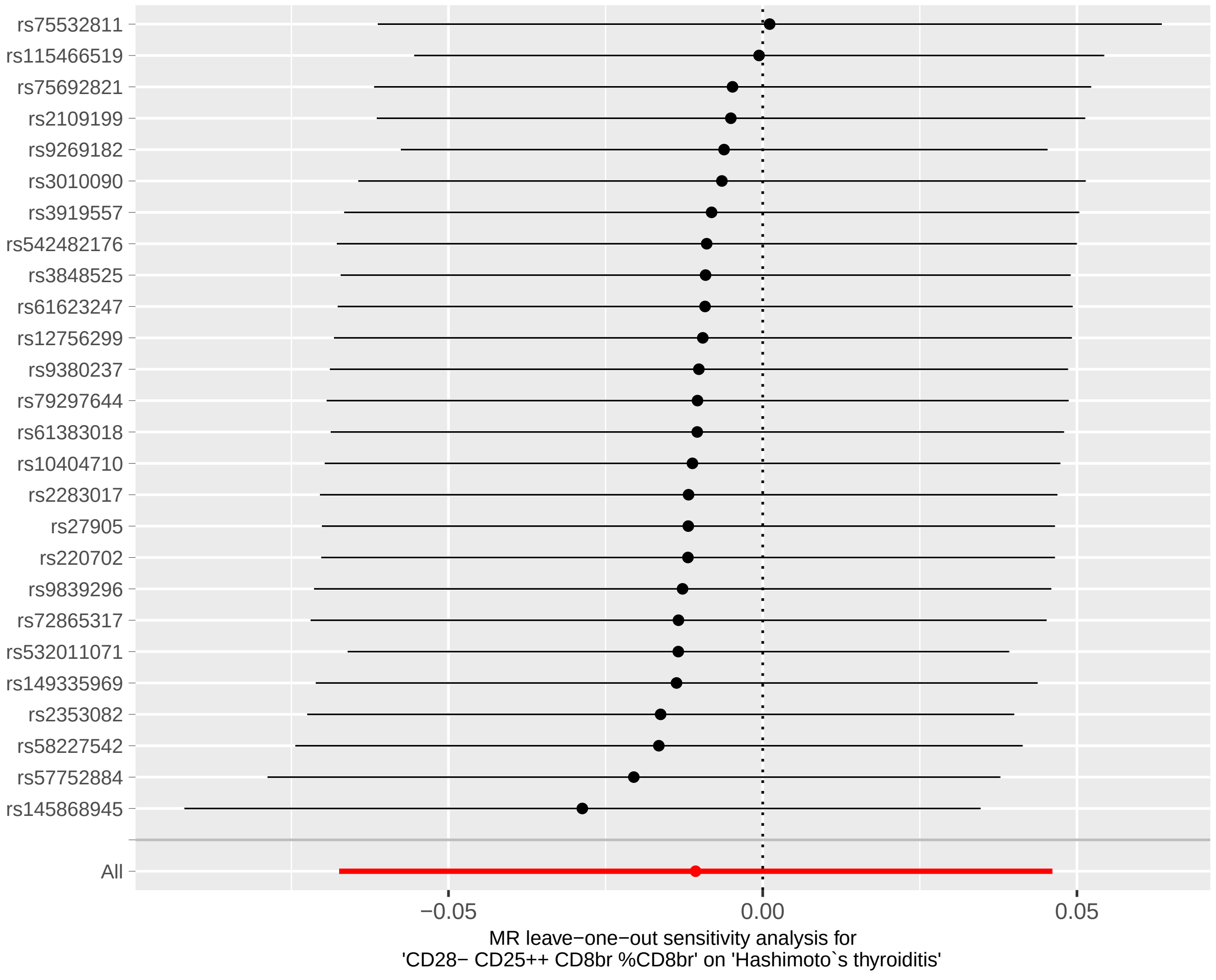

rs75532811
rs115466519
rs75692821
rs2109199
rs9269182
rs3010090
rs3919557
rs542482176
rs3848525
rs61623247
rs12756299
rs9380237
rs79297644
rs61383018
rs10404710
rs2283017
rs27905
rs220702
rs9839296
rs72865317
rs532011071
rs149335969
rs2353082
rs58227542
rs57752884
rs145868945
All
−0.05
0.00
0.05
MR leave−one−out sensitivity analysis for
'CD28− CD25++ CD8br %CD8br' on 'Hashimoto`s thyroiditis'

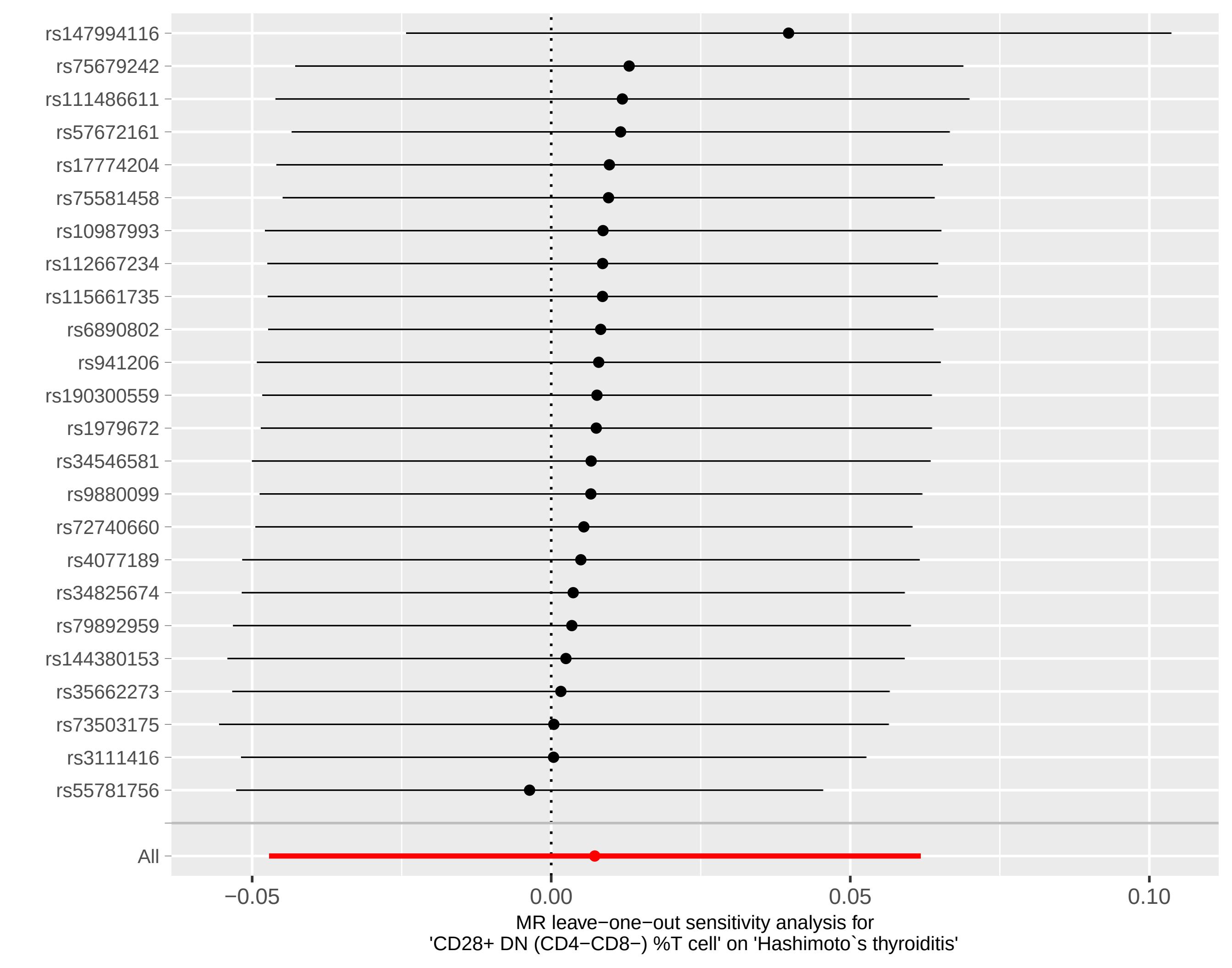

rs147994116
rs75679242
rs111486611
rs57672161
rs17774204
rs75581458
rs10987993
rs112667234
rs115661735
rs6890802
rs941206
rs190300559
rs1979672
rs34546581
rs9880099
rs72740660
rs4077189
rs34825674
rs79892959
rs144380153
rs35662273
rs73503175
rs3111416
rs55781756
All
−0.05
0.00
0.05
0.10
MR leave−one−out sensitivity analysis for
'CD28+ DN (CD4−CD8−) %T cell' on 'Hashimoto`s thyroiditis'

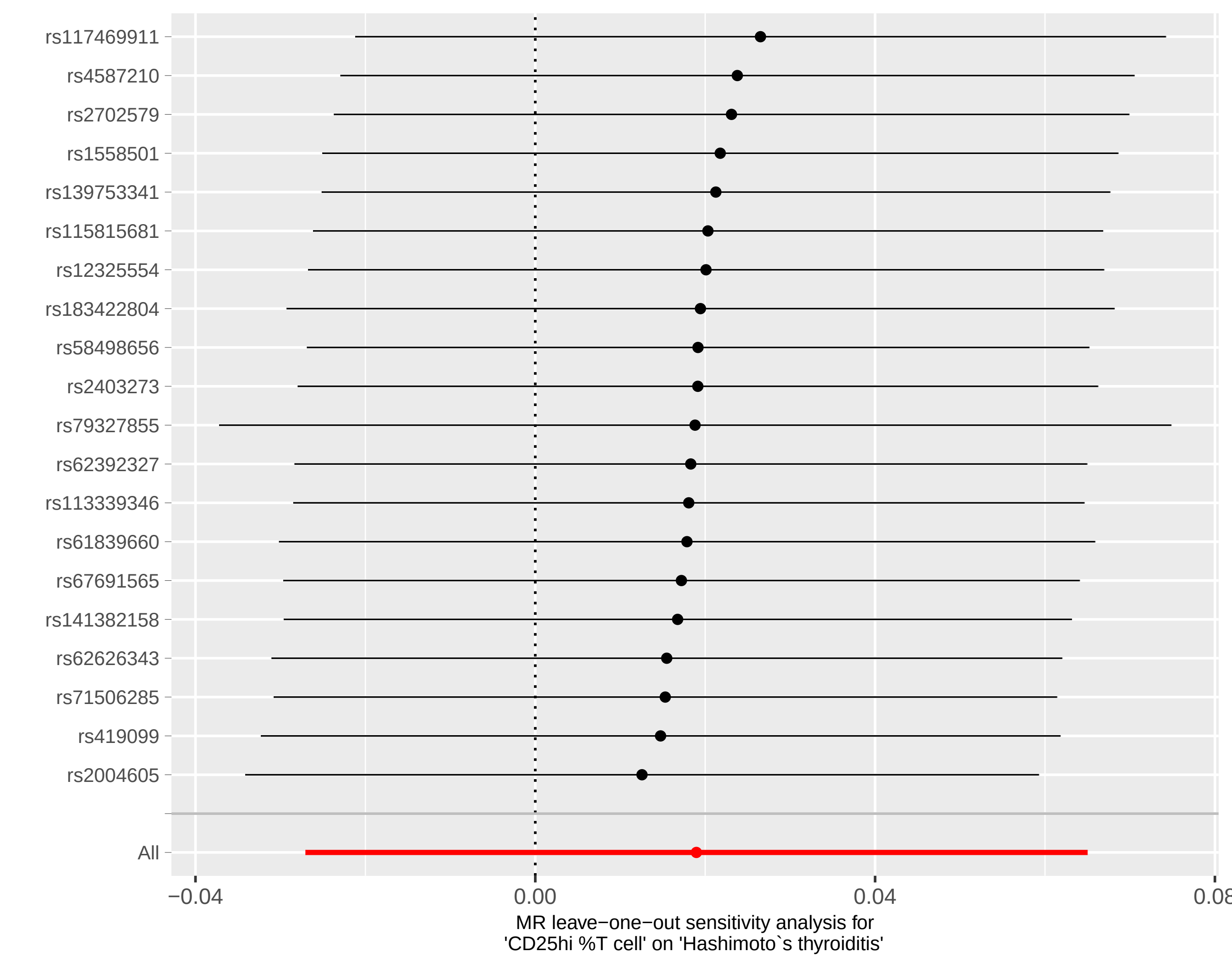
rs117469911
rs4587210
rs2702579
rs1558501
rs139753341
rs115815681
rs12325554
rs183422804
rs58498656
rs2403273
rs79327855
rs62392327
rs113339346
rs61839660
rs67691565
rs141382158
rs62626343
rs71506285
rs419099
rs2004605
All
−0.04
0.00
0.04
0.08
MR leave−one−out sensitivity analysis for
'CD25hi %T cell' on 'Hashimoto`s thyroiditis'

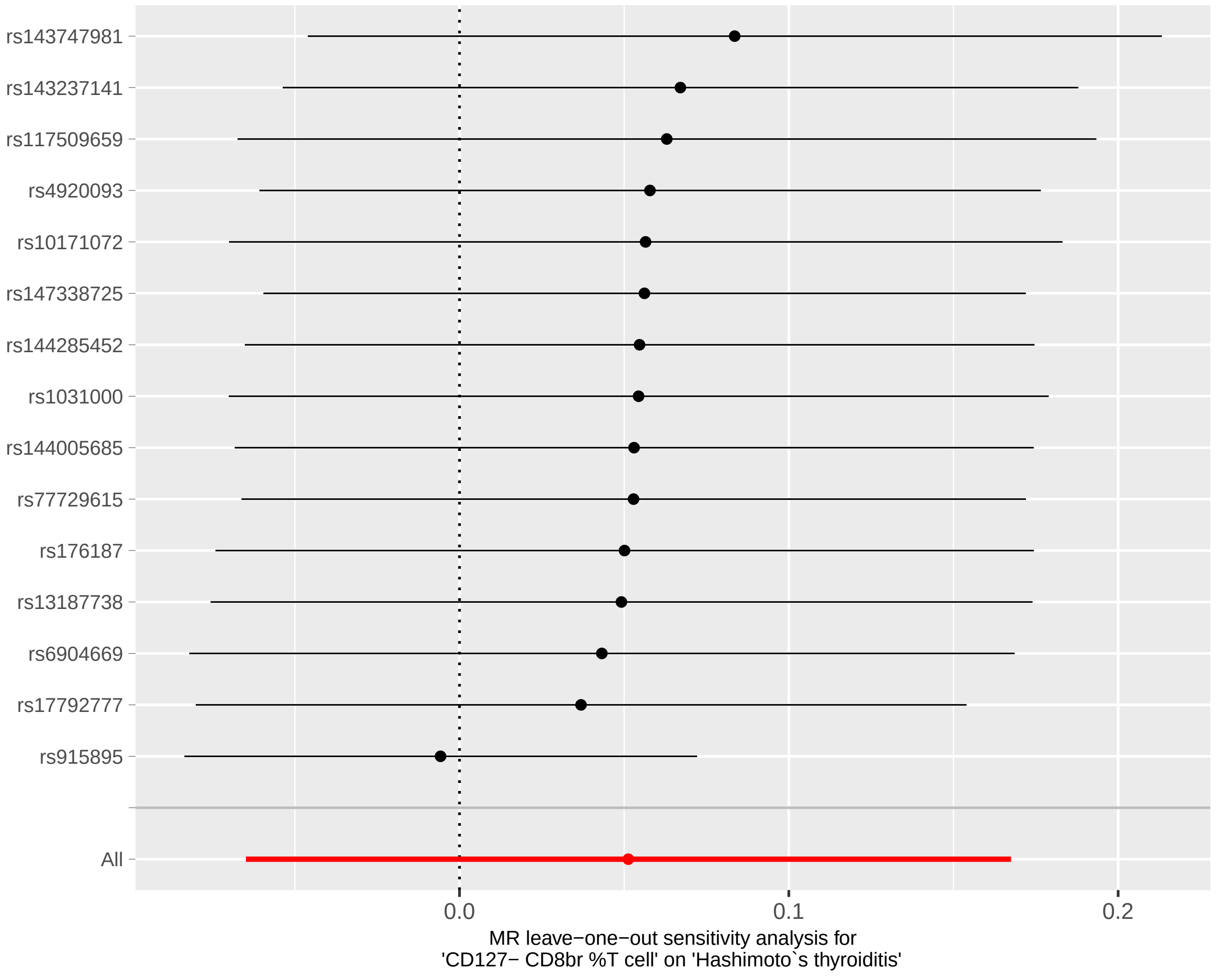
rs143747981
rs143237141
rs117509659
rs4920093
rs10171072
rs147338725
rs144285452
rs1031000
rs144005685
rs77729615
rs176187
rs13187738
rs6904669
rs17792777
rs915895
All
0.0
0.1
0.2
MR leave−one−out sensitivity analysis for
'CD127− CD8br %T cell' on 'Hashimoto`s thyroiditis'

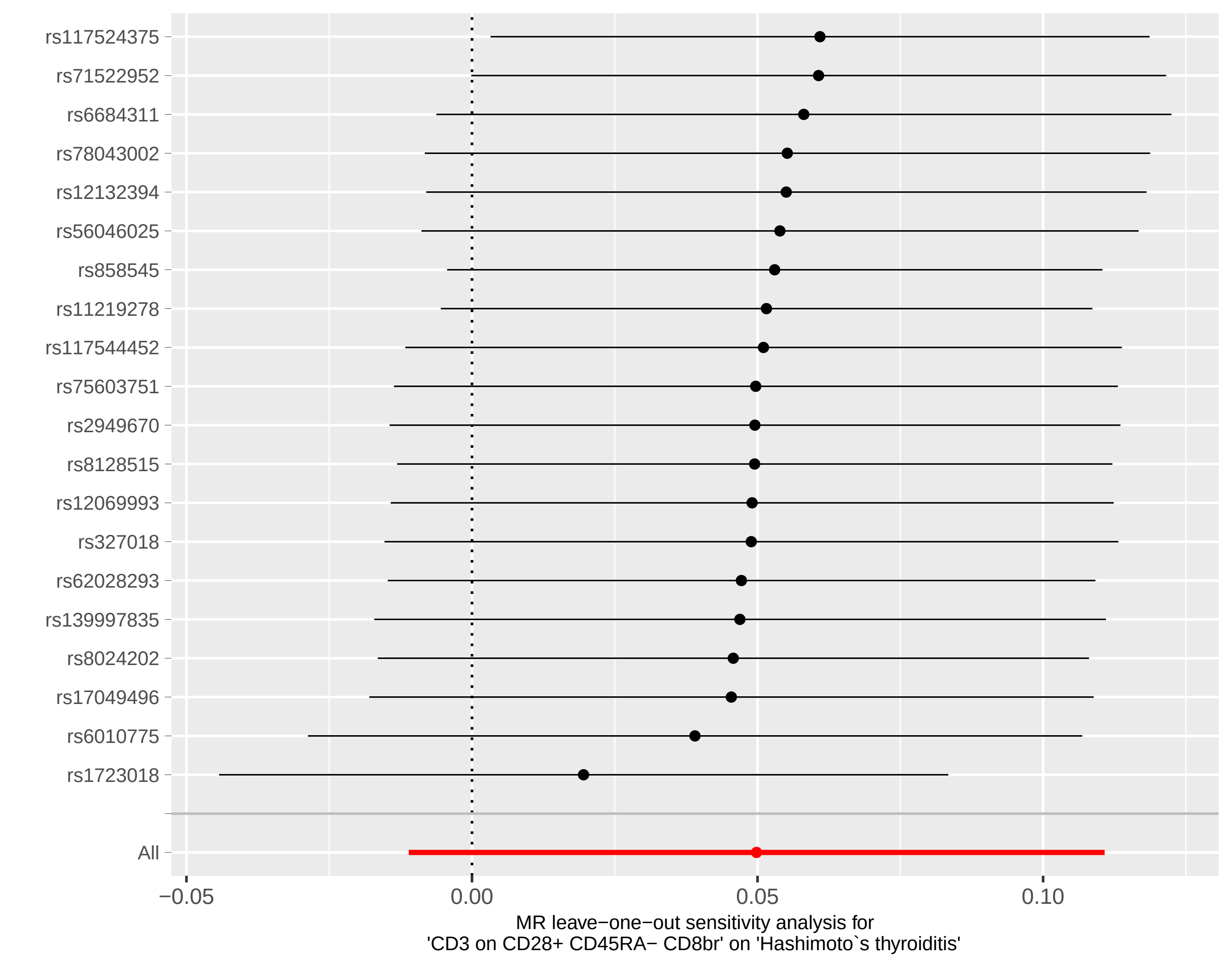
rs117524375
rs71522952
rs6684311
rs78043002
rs12132394
rs56046025
rs858545
rs11219278
rs117544452
rs75603751
rs2949670
rs8128515
rs12069993
rs327018
rs62028293
rs139997835
rs8024202
rs17049496
rs6010775
rs1723018
All
−0.05
0.00
0.05
0.10
MR leave−one−out sensitivity analysis for
'CD3 on CD28+ CD45RA− CD8br' on 'Hashimoto`s thyroiditis'

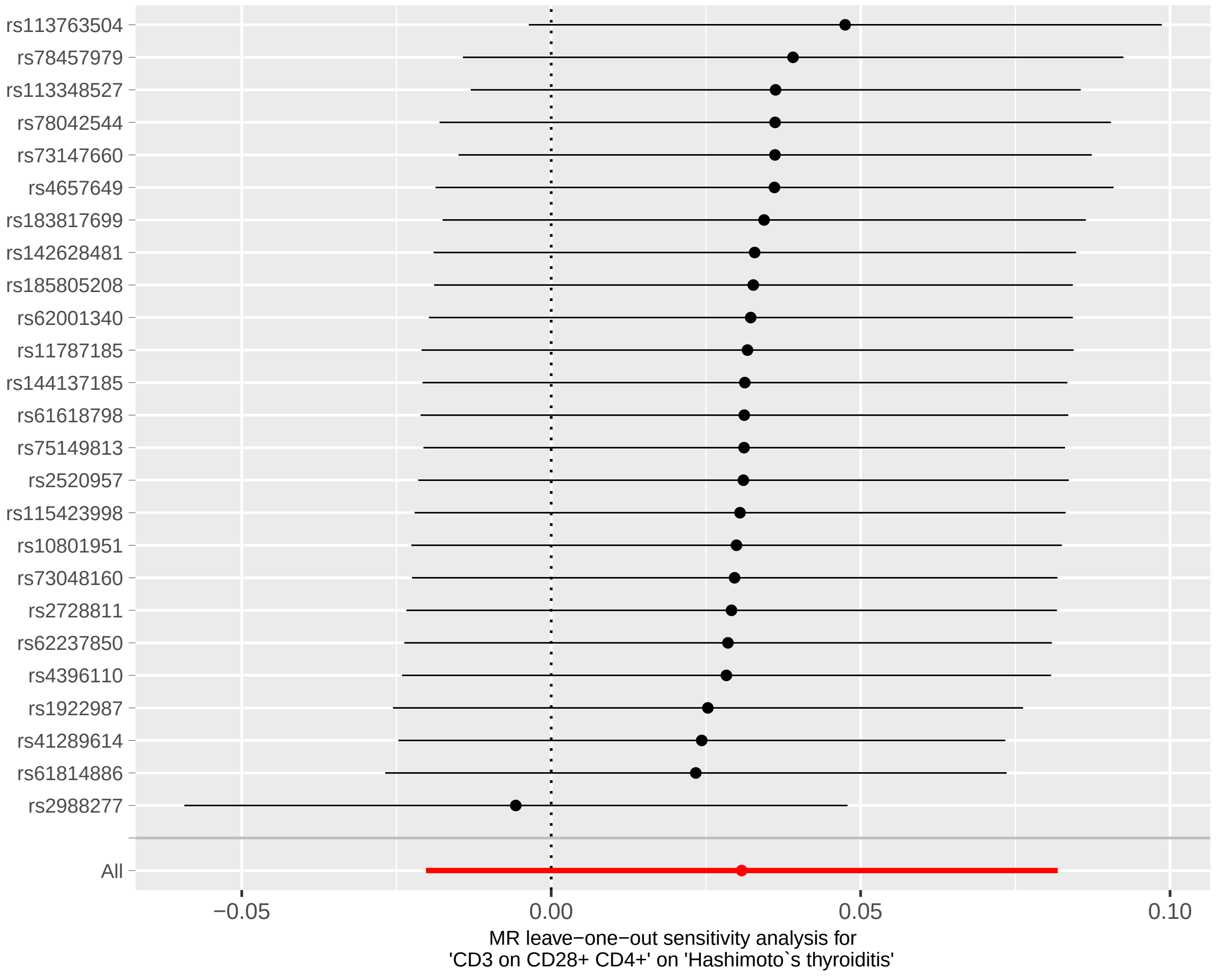
rs113763504
rs78457979
rs113348527
rs78042544
rs73147660
rs4657649
rs183817699
rs142628481
rs185805208
rs62001340
rs11787185
rs144137185
rs61618798
rs75149813
rs2520957
rs115423998
rs10801951
rs73048160
rs2728811
rs62237850
rs4396110
rs1922987
rs41289614
rs61814886
rs2988277
All
−0.05
0.00
0.05
0.10
MR leave−one−out sensitivity analysis for
'CD3 on CD28+ CD4+' on 'Hashimoto`s thyroiditis'

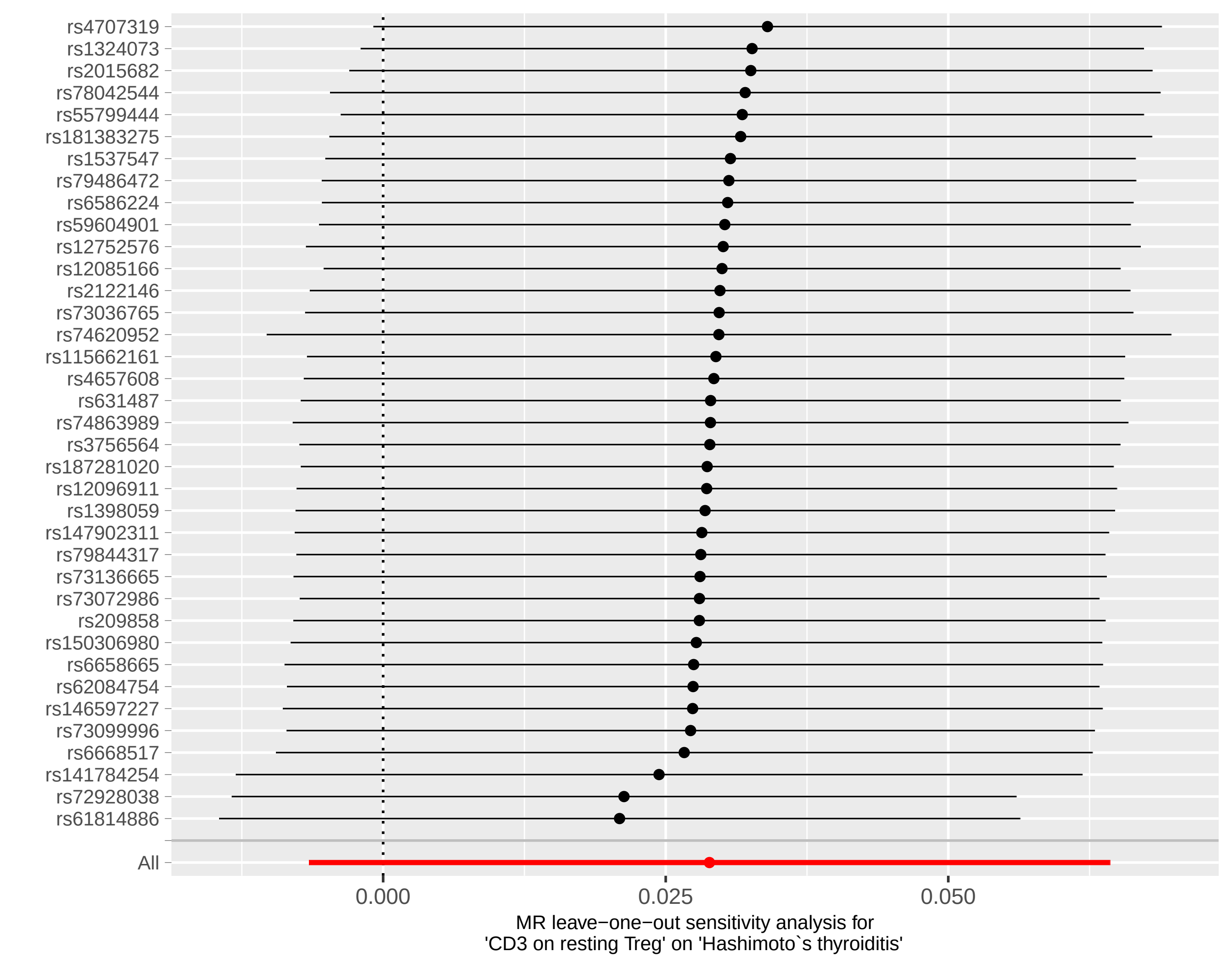

rs4707319
rs1324073
rs2015682
rs78042544
rs55799444
rs181383275
rs1537547
rs79486472
rs6586224
rs59604901
rs12752576
rs12085166
rs2122146
rs73036765
rs74620952
rs115662161
rs4657608
rs631487
rs74863989
rs3756564
rs187281020
rs12096911
rs1398059
rs147902311
rs79844317
rs73136665
rs73072986
rs209858
rs150306980
rs6658665
rs62084754
rs146597227
rs73099996
rs6668517
rs141784254
rs72928038
rs61814886
All
0.000
0.025
0.050
MR leave−one−out sensitivity analysis for
'CD3 on resting Treg' on 'Hashimoto`s thyroiditis'

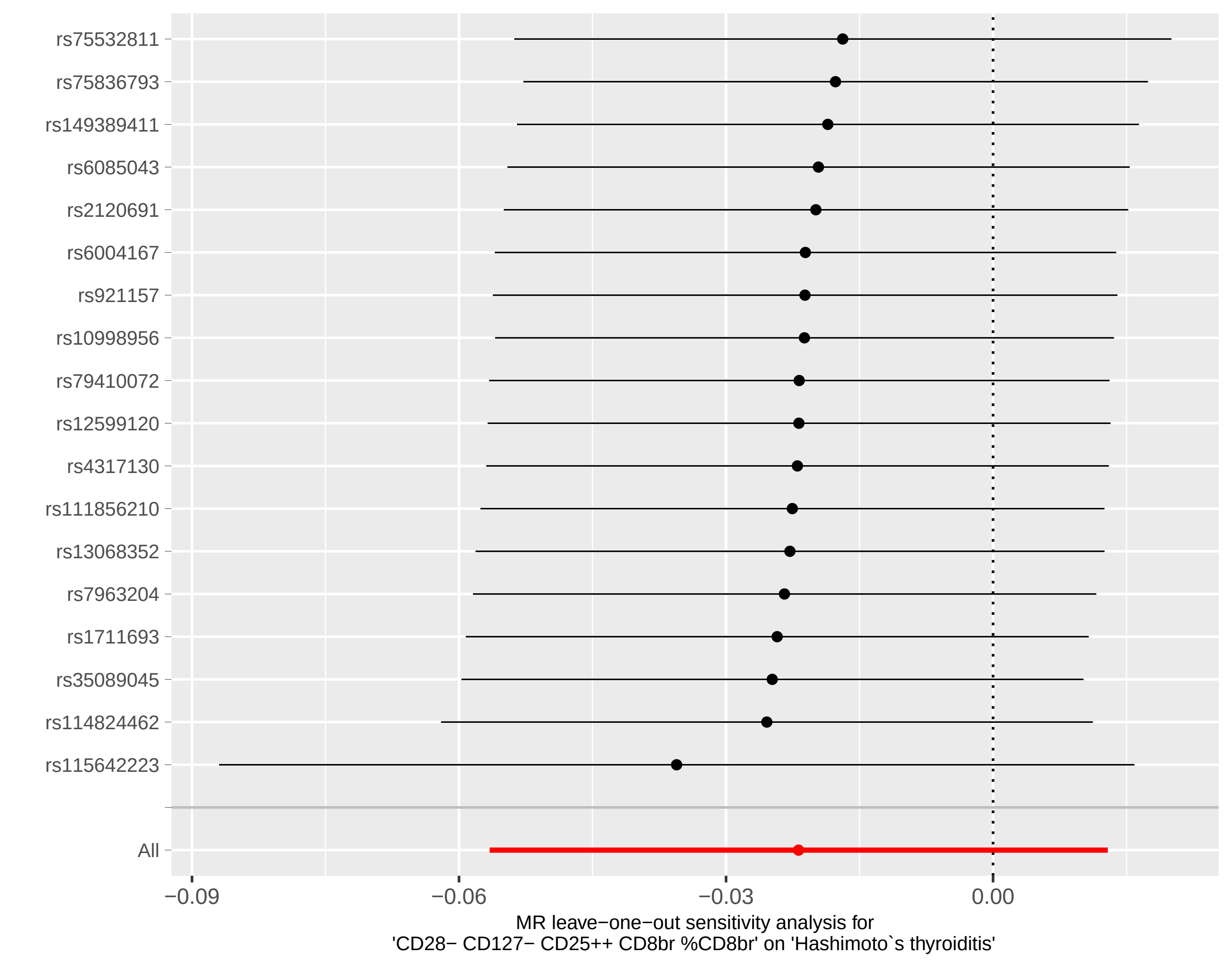
rs75532811
rs75836793
rs149389411
rs6085043
rs2120691
rs6004167
rs921157
rs10998956
rs79410072
rs12599120
rs4317130
rs111856210
rs13068352
rs7963204
rs1711693
rs35089045
rs114824462
rs115642223
All
−0.09
−0.06
−0.03
0.00
MR leave−one−out sensitivity analysis for
'CD28− CD127− CD25++ CD8br %CD8br' on 'Hashimoto`s thyroiditis'

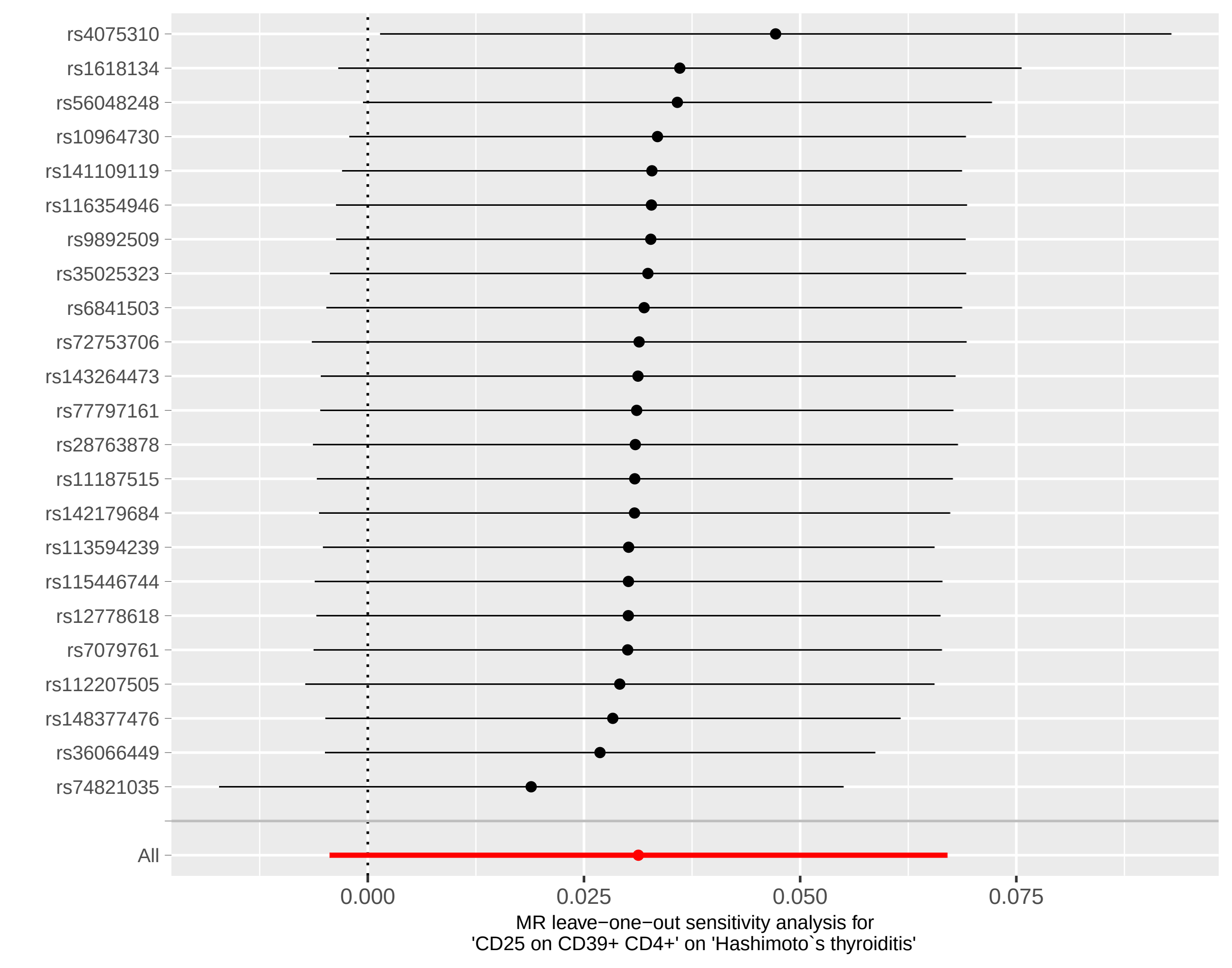
rs4075310
rs1618134
rs56048248
rs10964730
rs141109119
rs116354946
rs9892509
rs35025323
rs6841503
rs72753706
rs143264473
rs77797161
rs28763878
rs11187515
rs142179684
rs113594239
rs115446744
rs12778618
rs7079761
rs112207505
rs148377476
rs36066449
rs74821035
All
0.000
0.025
0.050
0.075
MR leave−one−out sensitivity analysis for
'CD25 on CD39+ CD4+' on 'Hashimoto`s thyroiditis'

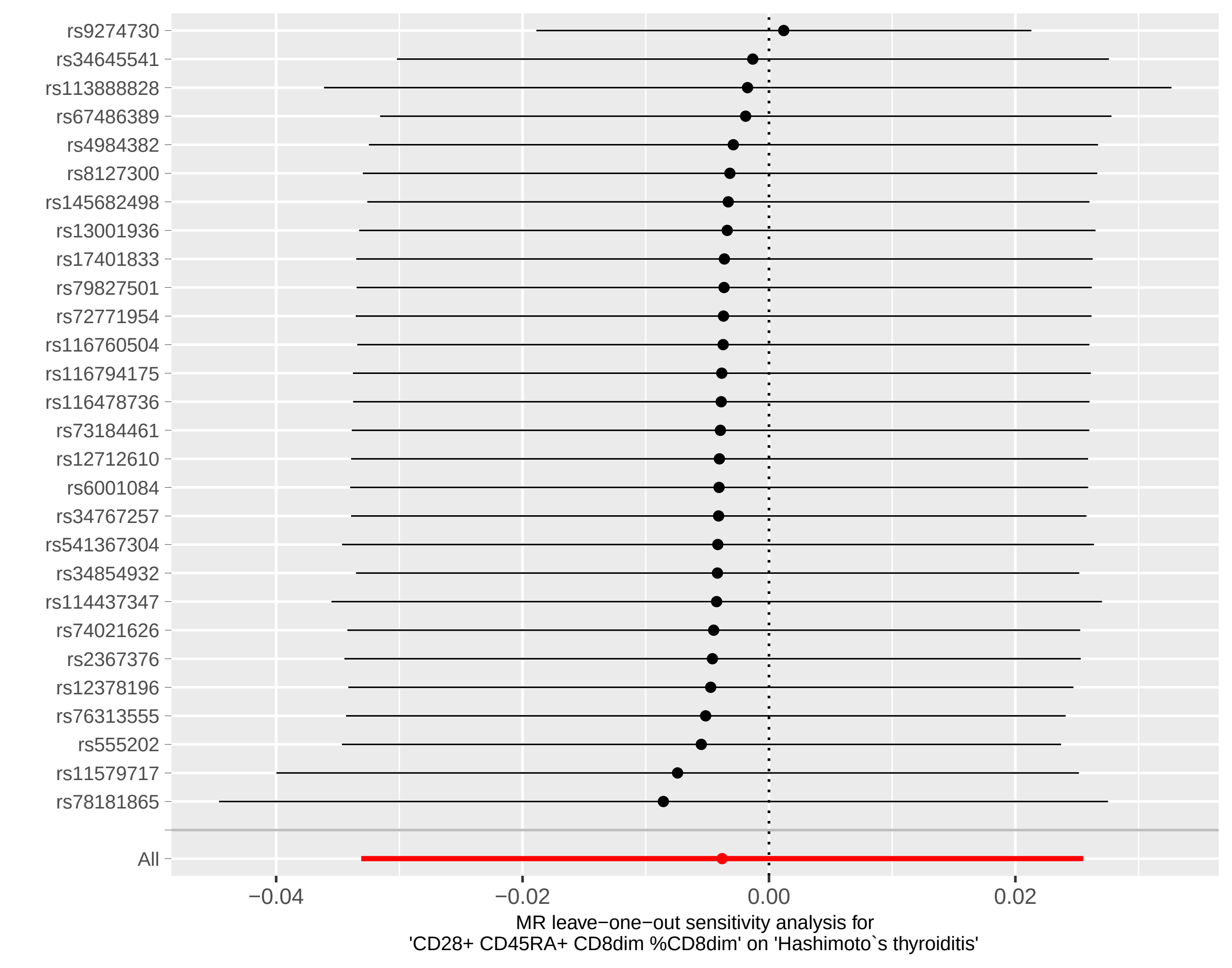
rs9274730
rs34645541
rs113888828
rs67486389
rs4984382
rs8127300
rs145682498
rs13001936
rs17401833
rs79827501
rs72771954
rs116760504
rs116794175
rs116478736
rs73184461
rs12712610
rs6001084
rs34767257
rs541367304
rs34854932
rs114437347
rs74021626
rs2367376
rs12378196
rs76313555
rs555202
rs11579717
rs78181865
All
−0.04
−0.02
0.00
0.02
MR leave−one−out sensitivity analysis for
'CD28+ CD45RA+ CD8dim %CD8dim' on 'Hashimoto`s thyroiditis'

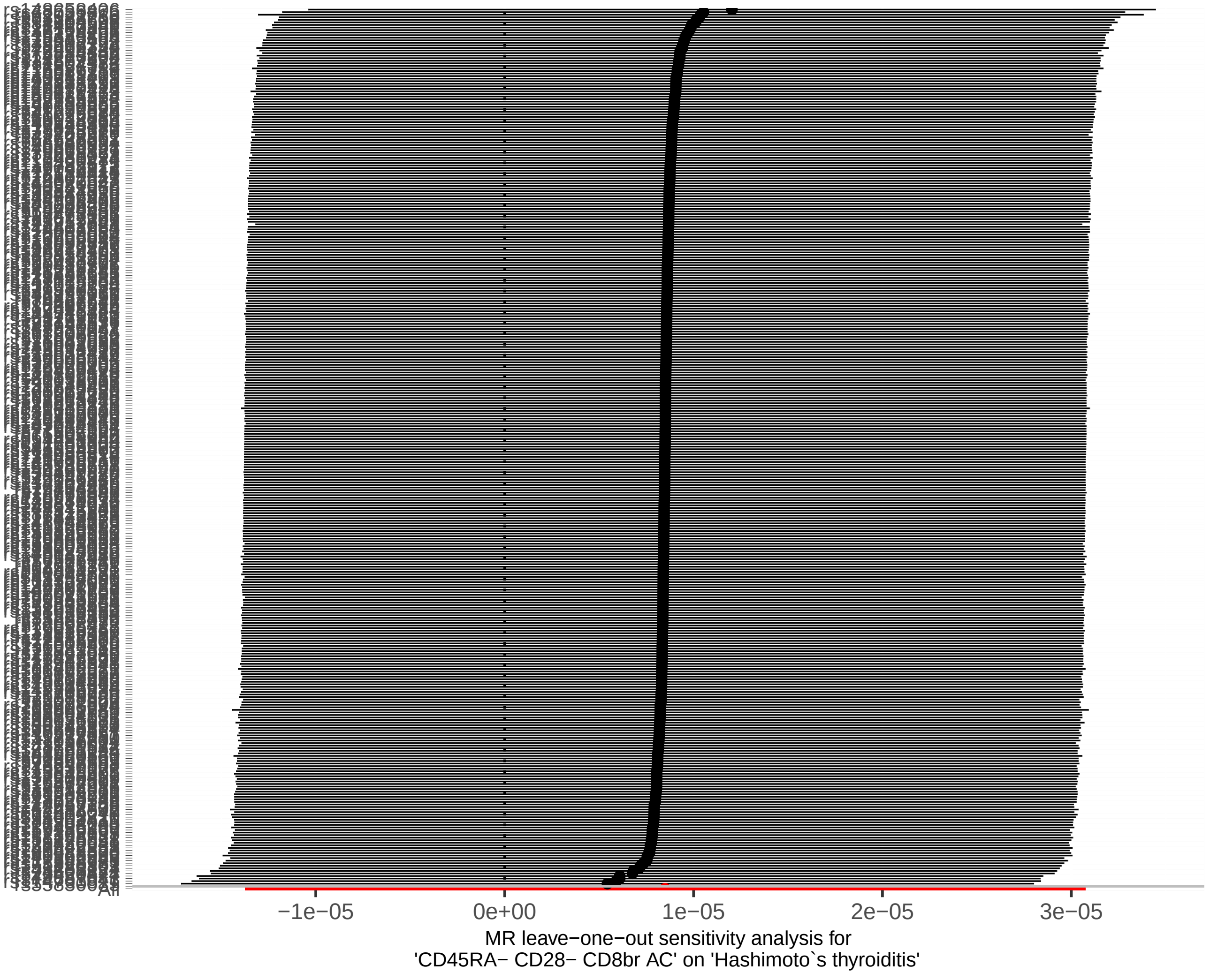

All
−1e−05
0e+00
1e−05
2e−05
3e−05
MR leave−one−out sensitivity analysis for
'CD45RA− CD28− CD8br AC' on 'Hashimoto`s thyroiditis'

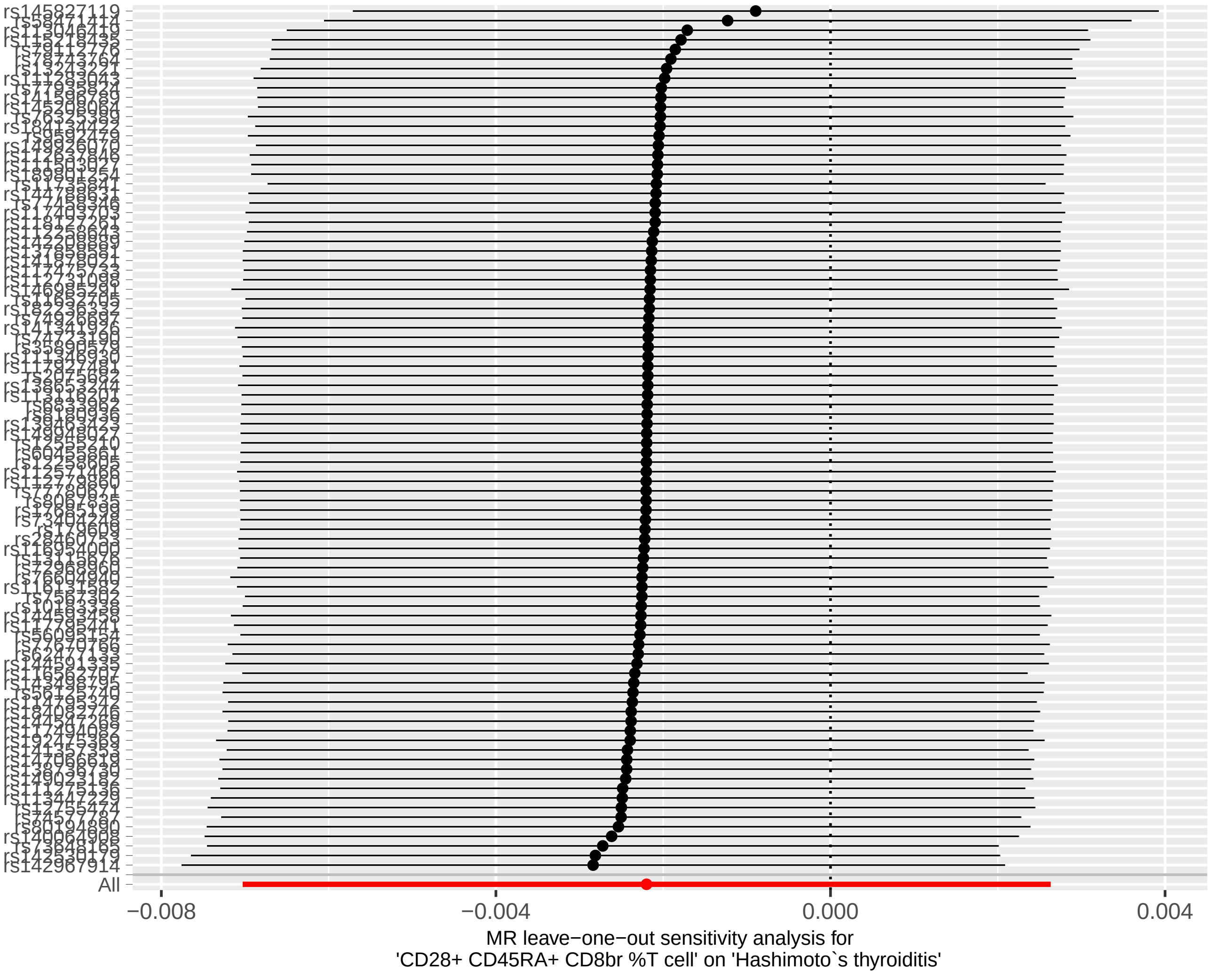
rs145827119
rs142967914
All
−0.008
−0.004
0.000
0.004
MR leave−one−out sensitivity analysis for
'CD28+ CD45RA+ CD8br %T cell' on 'Hashimoto`s thyroiditis'

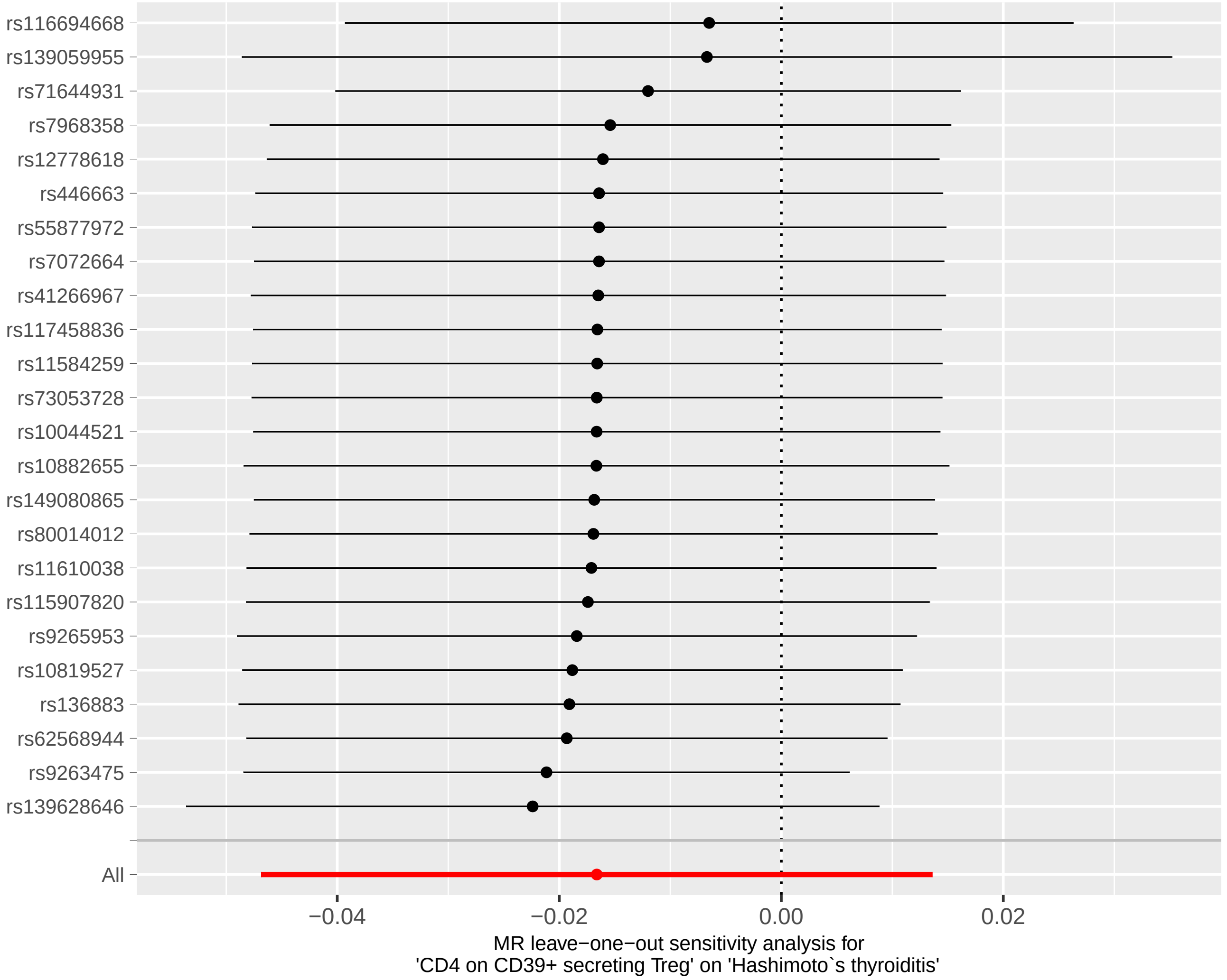
rs116694668
rs139059955
rs71644931
rs7968358
rs12778618
rs446663
rs55877972
rs7072664
rs41266967
rs117458836
rs11584259
rs73053728
rs10044521
rs10882655
rs149080865
rs80014012
rs11610038
rs115907820
rs9265953
rs10819527
rs136883
rs62568944
rs9263475
rs139628646
All
−0.04
−0.02
0.00
0.02
MR leave−one−out sensitivity analysis for
'CD4 on CD39+ secreting Treg' on 'Hashimoto`s thyroiditis'

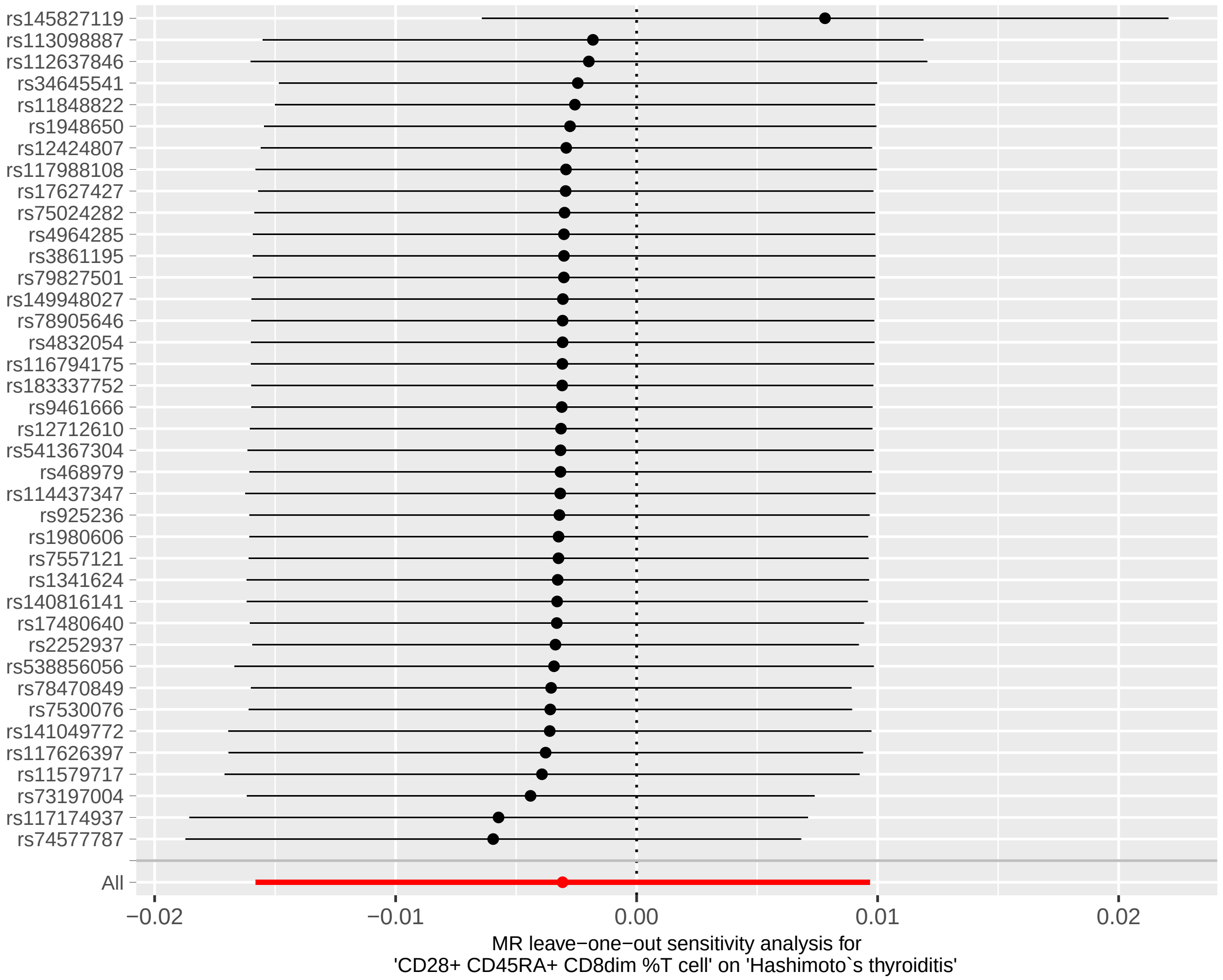
rs145827119
rs113098887
rs112637846
rs34645541
rs11848822
rs1948650
rs12424807
rs117988108
rs17627427
rs75024282
rs4964285
rs3861195
rs79827501
rs149948027
rs78905646
rs4832054
rs116794175
rs183337752
rs9461666
rs12712610
rs541367304
rs468979
rs114437347
rs925236
rs1980606
rs7557121
rs1341624
rs140816141
rs17480640
rs2252937
rs538856056
rs78470849
rs7530076
rs141049772
rs117626397
rs11579717
rs73197004
rs117174937
rs74577787
All
−0.02
−0.01
0.00
0.01
0.02
MR leave−one−out sensitivity analysis for
'CD28+ CD45RA+ CD8dim %T cell' on 'Hashimoto`s thyroiditis'

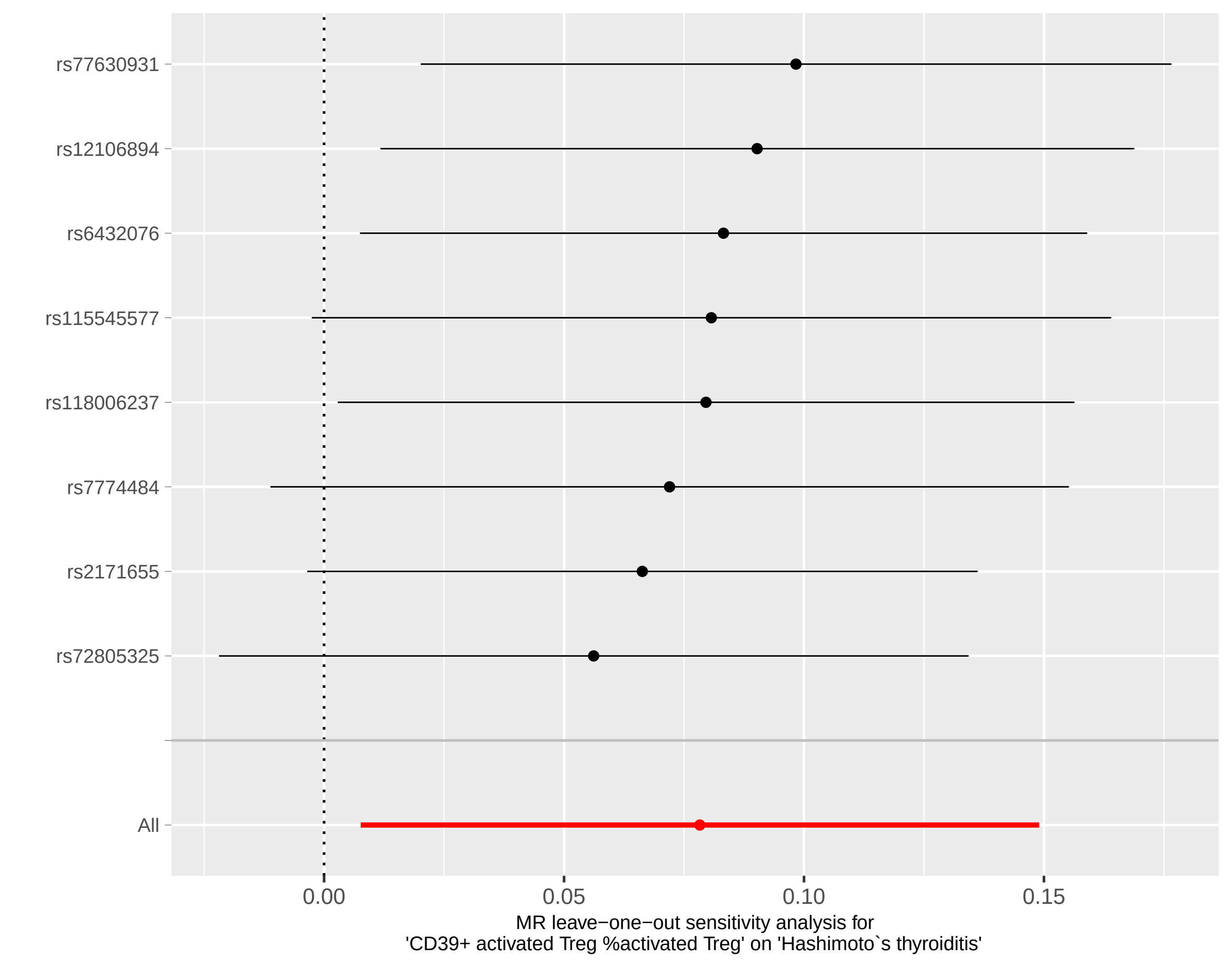
rs77630931
rs12106894
rs6432076
rs115545577
rs118006237
rs7774484
rs2171655
rs72805325
All
0.00
0.05
0.10
0.15
MR leave−one−out sensitivity analysis for
'CD39+ activated Treg %activated Treg' on 'Hashimoto`s thyroiditis'

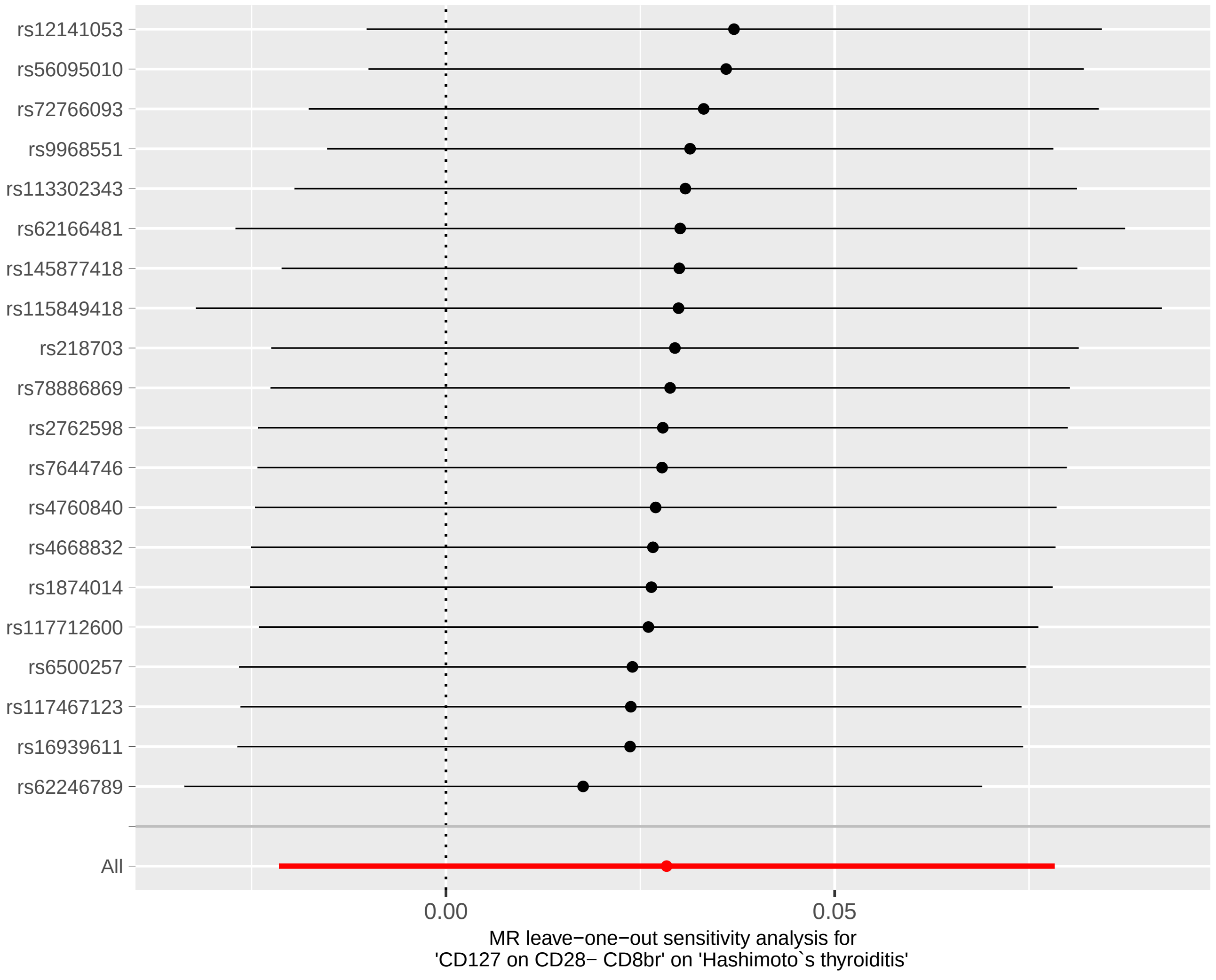
rs12141053
rs56095010
rs72766093
rs9968551
rs113302343
rs62166481
rs145877418
rs115849418
rs218703
rs78886869
rs2762598
rs7644746
rs4760840
rs4668832
rs1874014
rs117712600
rs6500257
rs117467123
rs16939611
rs62246789
All
0.00
0.05
MR leave−one−out sensitivity analysis for
'CD127 on CD28− CD8br' on 'Hashimoto`s thyroiditis'

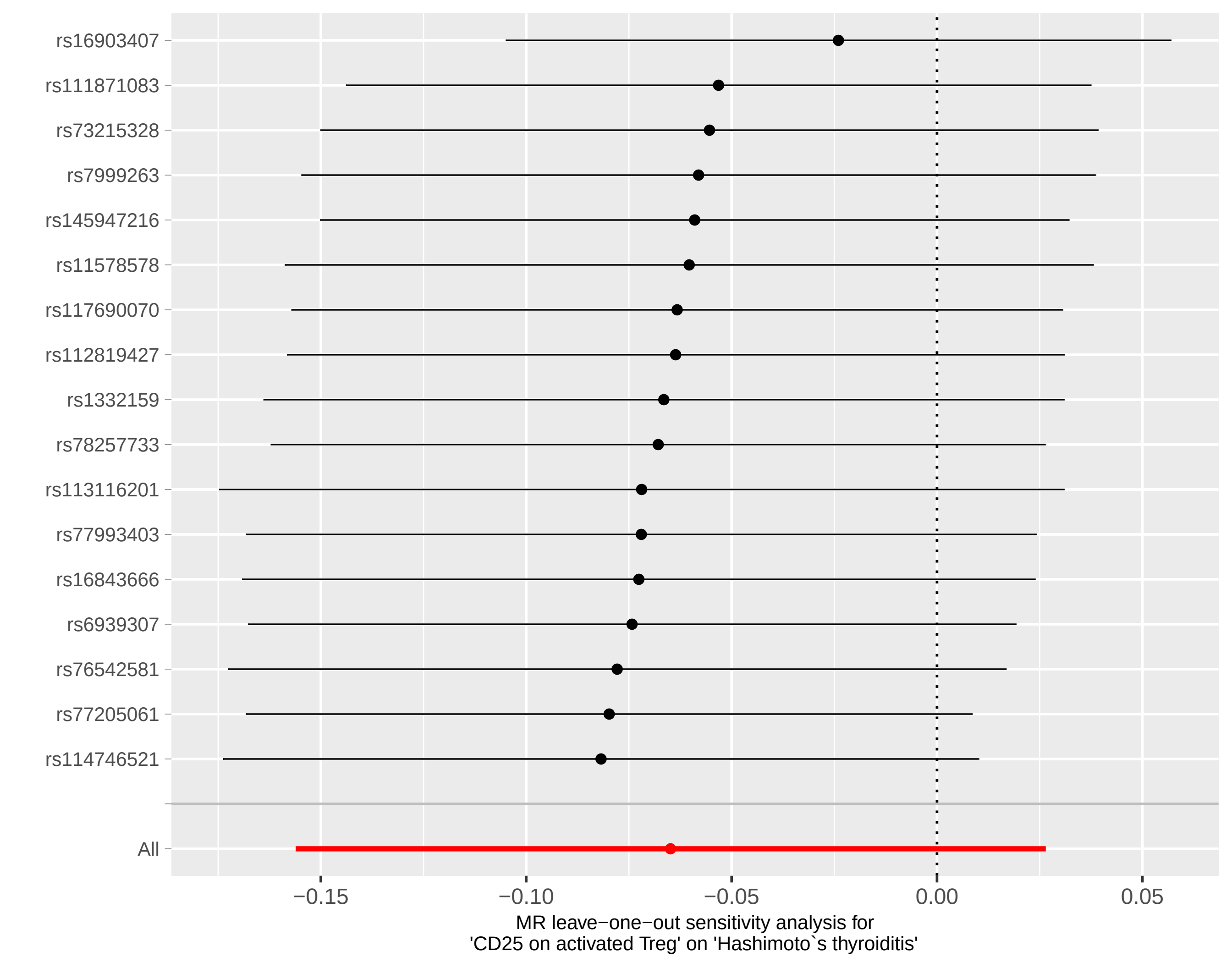
rs16903407
rs111871083
rs73215328
rs7999263
rs145947216
rs11578578
rs117690070
rs112819427
rs1332159
rs78257733
rs113116201
rs77993403
rs16843666
rs6939307
rs76542581
rs77205061
rs114746521
All
−0.15
−0.10
−0.05
0.00
0.05
MR leave−one−out sensitivity analysis for
'CD25 on activated Treg' on 'Hashimoto`s thyroiditis'

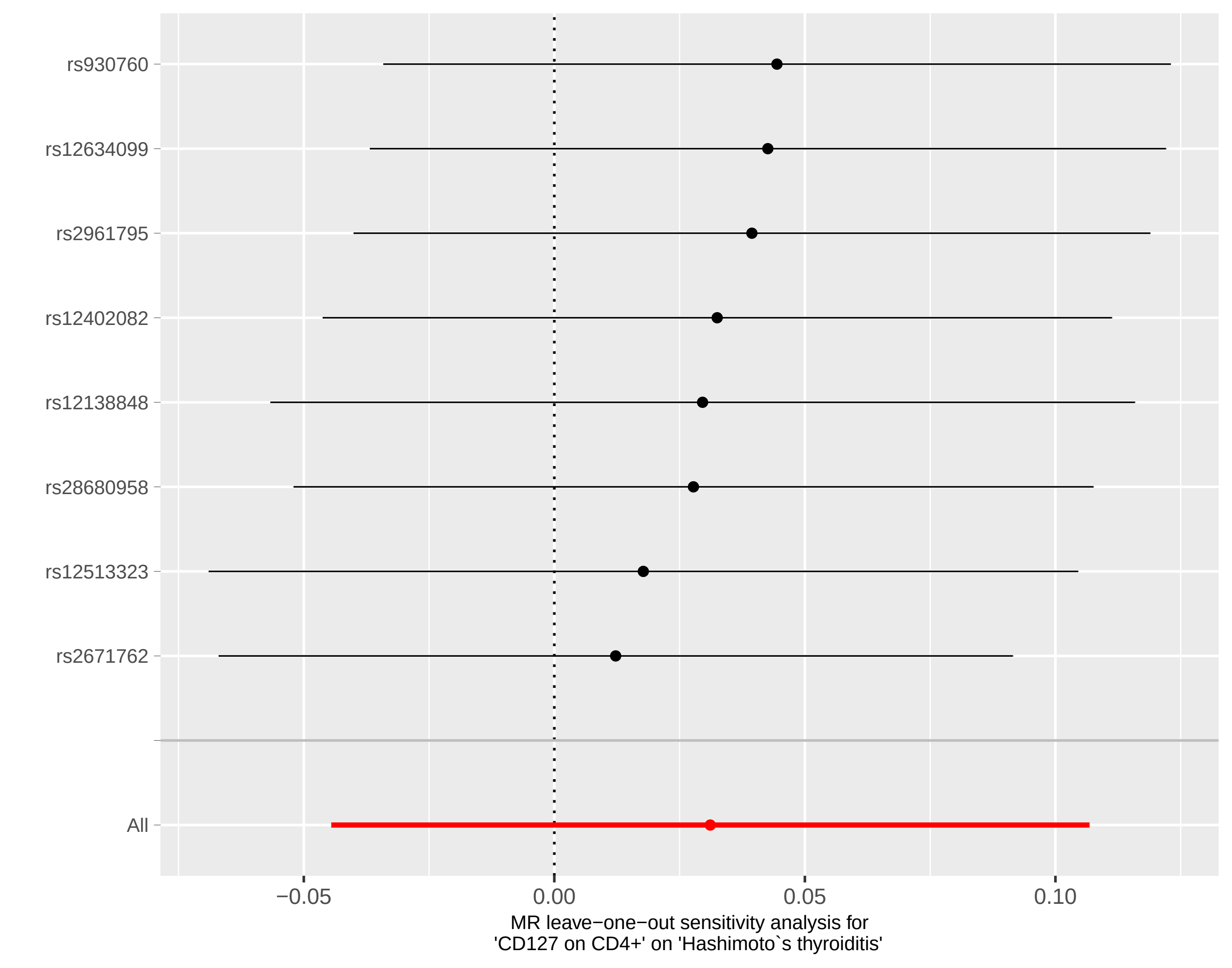
rs930760
rs12634099
rs2961795
rs12402082
rs12138848
rs28680958
rs12513323
rs2671762
All
−0.05
0.00
0.05
0.10
MR leave−one−out sensitivity analysis for
'CD127 on CD4+' on 'Hashimoto`s thyroiditis'

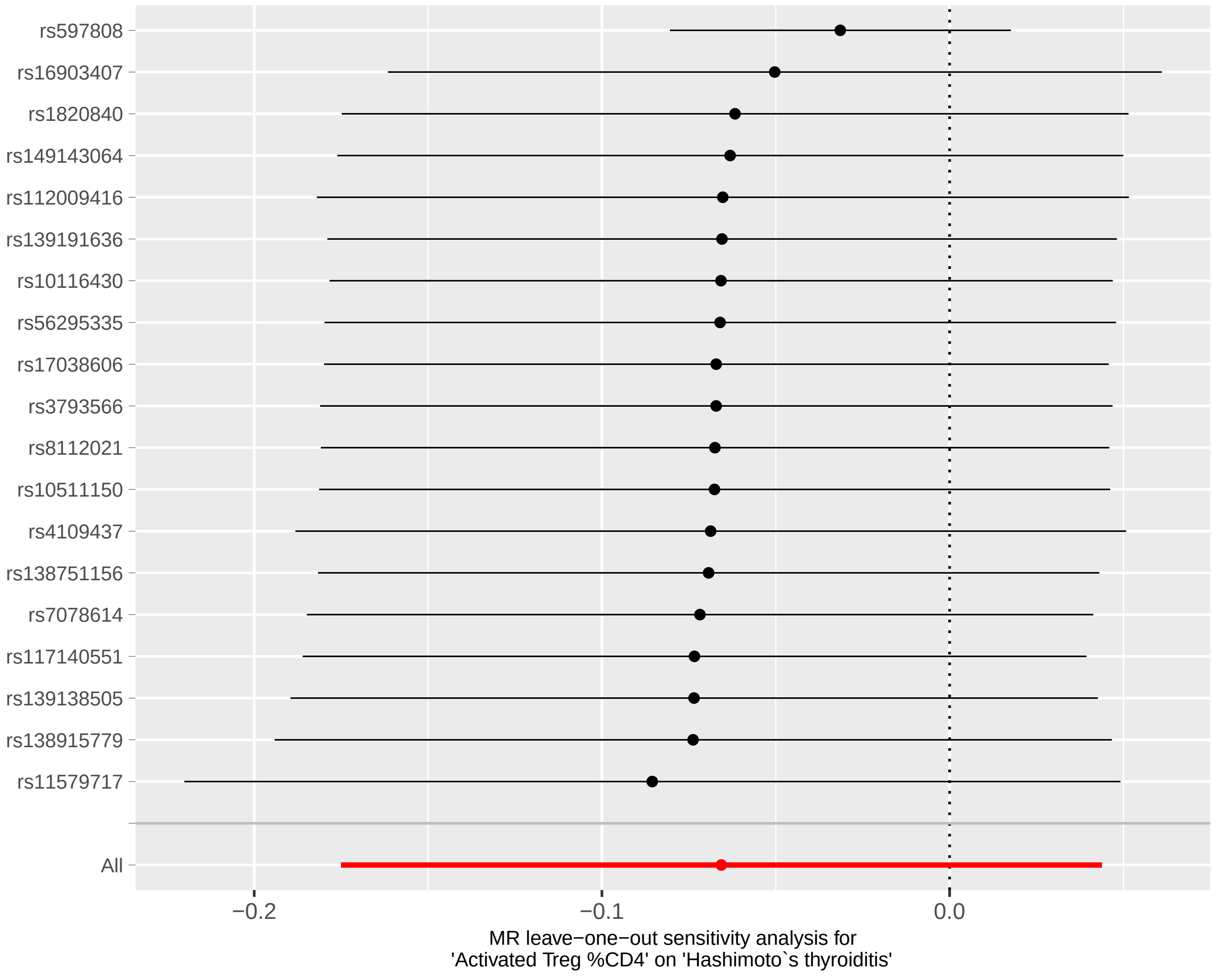
rs597808
rs16903407
rs1820840
rs149143064
rs112009416
rs139191636
rs10116430
rs56295335
rs17038606
rs3793566
rs8112021
rs10511150
rs4109437
rs138751156
rs7078614
rs117140551
rs139138505
rs138915779
rs11579717
All
−0.2
−0.1
0.0
MR leave−one−out sensitivity analysis for
'Activated Treg %CD4' on 'Hashimoto`s thyroiditis'

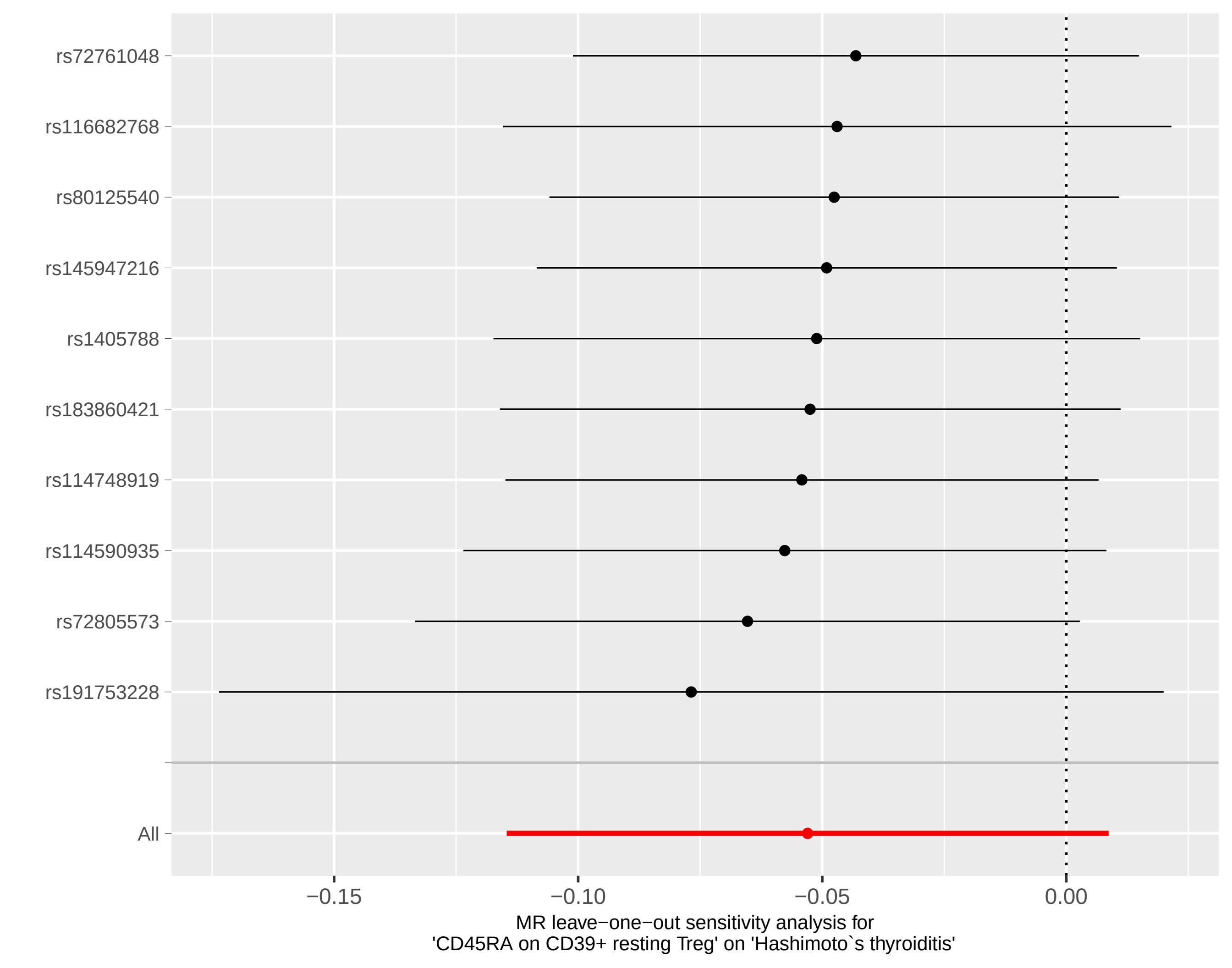
rs72761048
rs116682768
rs80125540
rs145947216
rs1405788
rs183860421
rs114748919
rs114590935
rs72805573
rs191753228
All
−0.15
−0.10
−0.05
0.00
MR leave−one−out sensitivity analysis for
'CD45RA on CD39+ resting Treg' on 'Hashimoto`s thyroiditis'

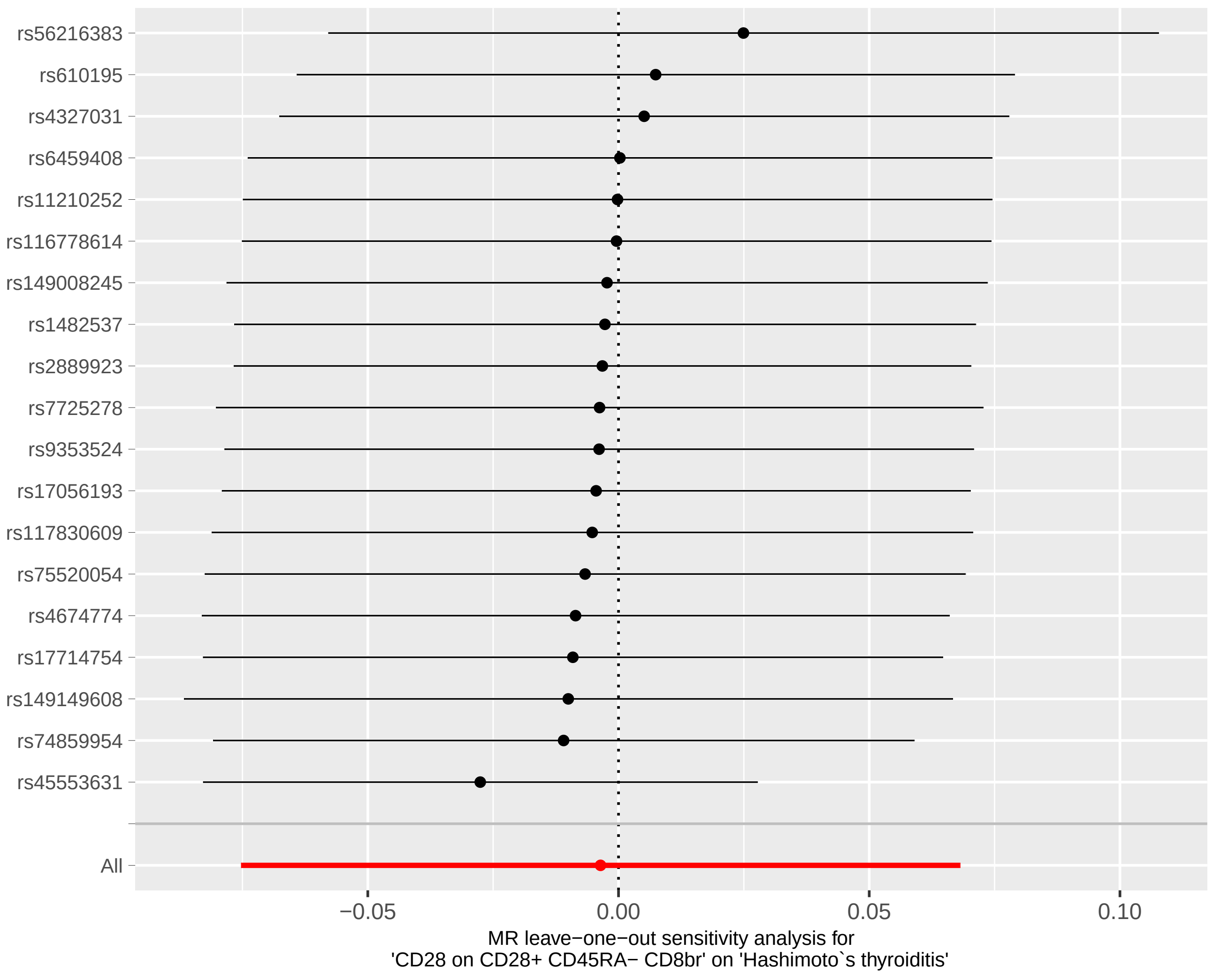
rs56216383
rs610195
rs4327031
rs6459408
rs11210252
rs116778614
rs149008245
rs1482537
rs2889923
rs7725278
rs9353524
rs17056193
rs117830609
rs75520054
rs4674774
rs17714754
rs149149608
rs74859954
rs45553631
All
−0.05
0.00
0.05
0.10
MR leave−one−out sensitivity analysis for
'CD28 on CD28+ CD45RA− CD8br' on 'Hashimoto`s thyroiditis'

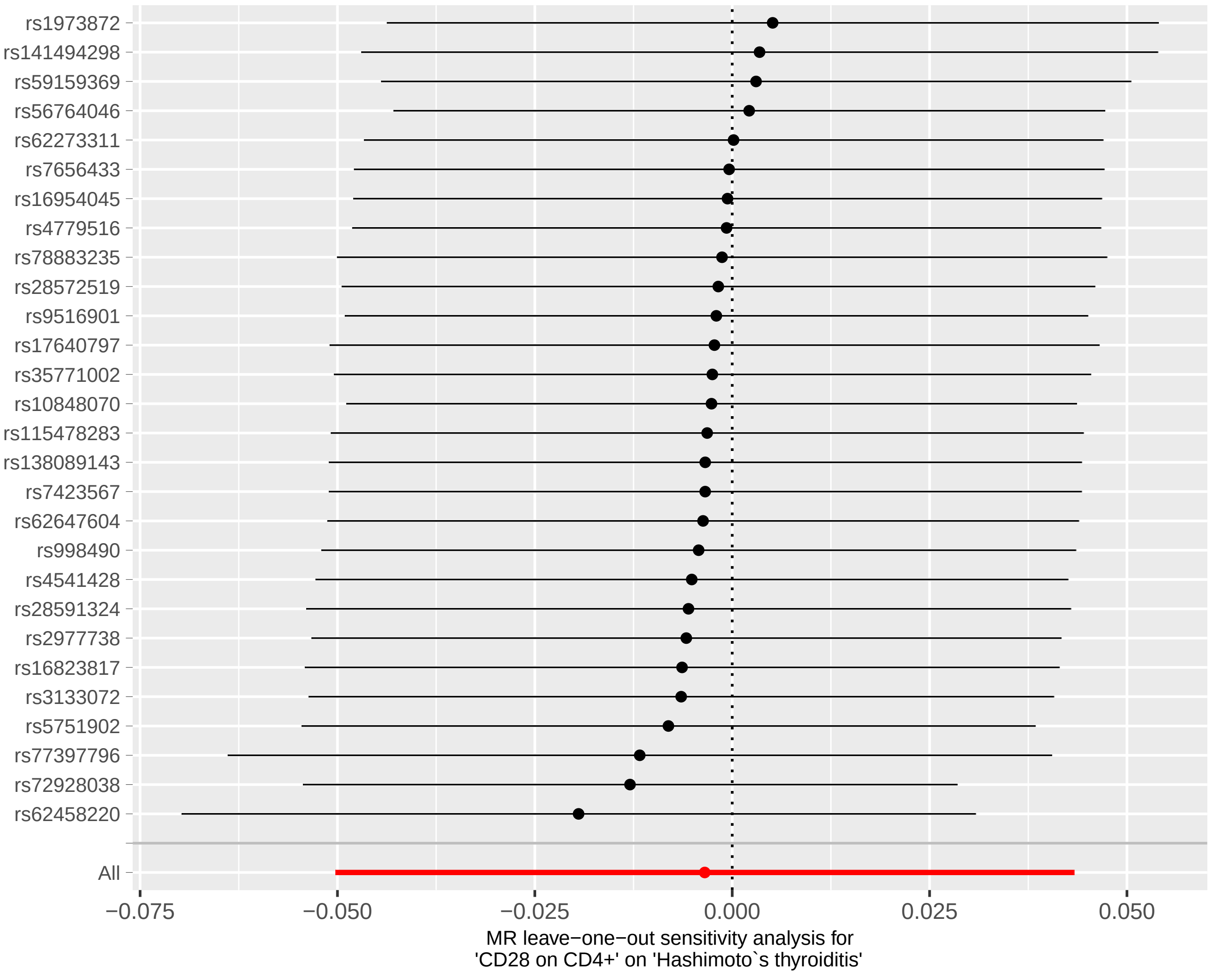
rs1973872
rs141494298
rs59159369
rs56764046
rs62273311
rs7656433
rs16954045
rs4779516
rs78883235
rs28572519
rs9516901
rs17640797
rs35771002
rs10848070
rs115478283
rs138089143
rs7423567
rs62647604
rs998490
rs4541428
rs28591324
rs2977738
rs16823817
rs3133072
rs5751902
rs77397796
rs72928038
rs62458220
All
−0.075
−0.050
−0.025
0.000
0.025
0.050
MR leave−one−out sensitivity analysis for
'CD28 on CD4+' on 'Hashimoto`s thyroiditis'

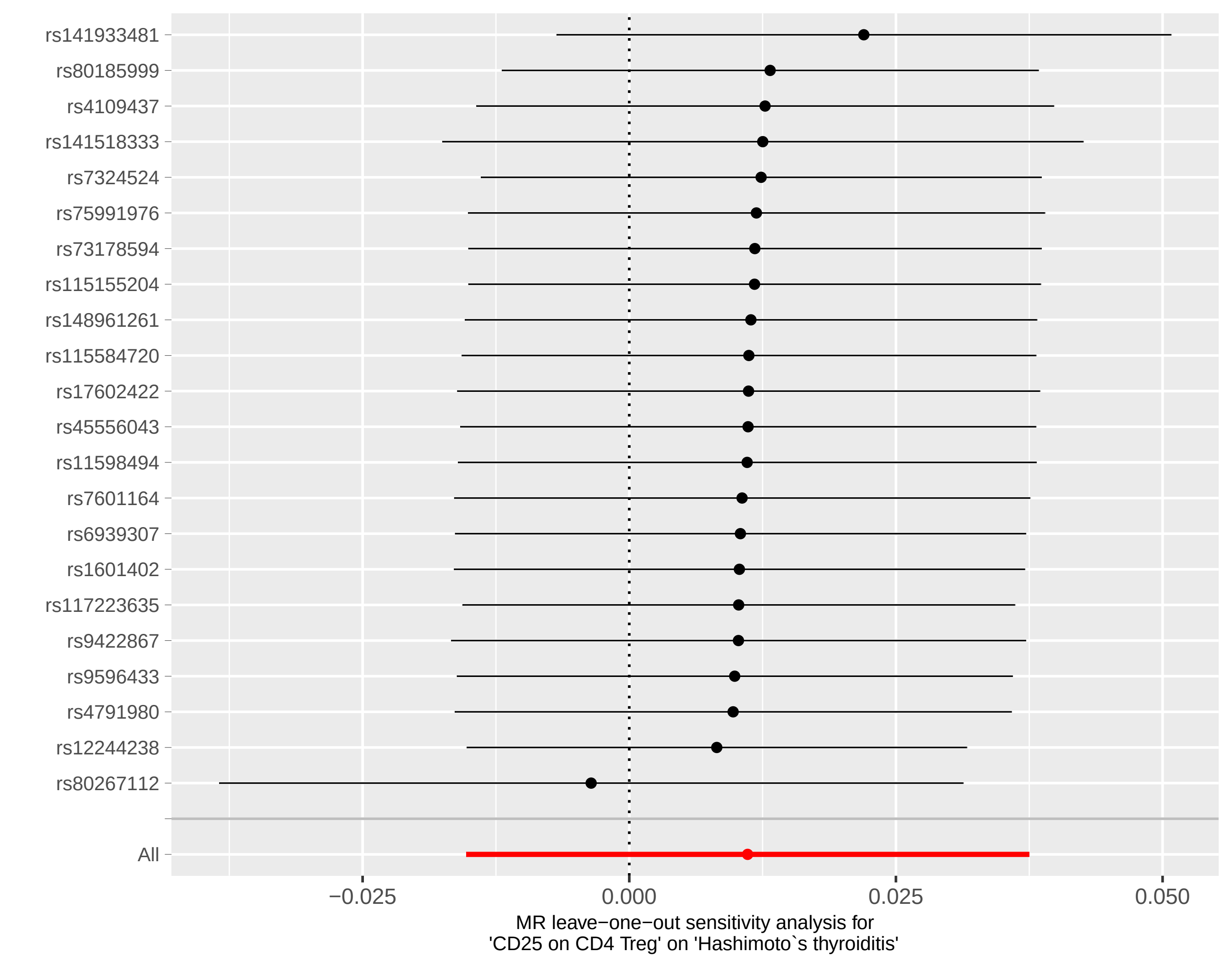
rs141933481
rs80185999
rs4109437
rs141518333
rs7324524
rs75991976
rs73178594
rs115155204
rs148961261
rs115584720
rs17602422
rs45556043
rs11598494
rs7601164
rs6939307
rs1601402
rs117223635
rs9422867
rs9596433
rs4791980
rs12244238
rs80267112
All
−0.025
0.000
0.025
0.050
MR leave−one−out sensitivity analysis for
'CD25 on CD4 Treg' on 'Hashimoto`s thyroiditis'

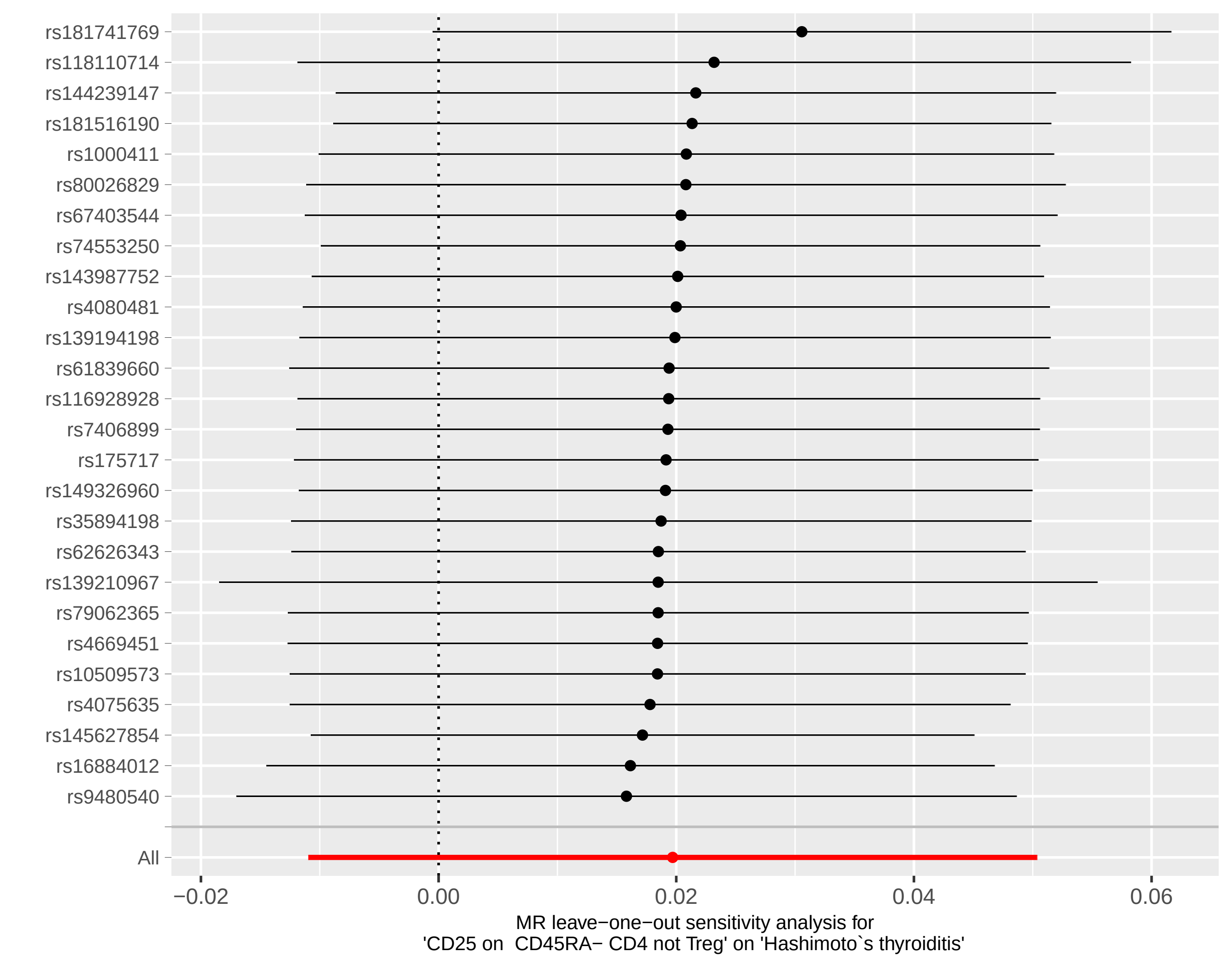

rs181741769
rs118110714
rs144239147
rs181516190
rs1000411
rs80026829
rs67403544
rs74553250
rs143987752
rs4080481
rs139194198
rs61839660
rs116928928
rs7406899
rs175717
rs149326960
rs35894198
rs62626343
rs139210967
rs79062365
rs4669451
rs10509573
rs4075635
rs145627854
rs16884012
rs9480540
All
−0.02
0.00
0.02
0.04
0.06
MR leave−one−out sensitivity analysis for
'CD25 on CD45RA− CD4 not Treg' on 'Hashimoto`s thyroiditis'

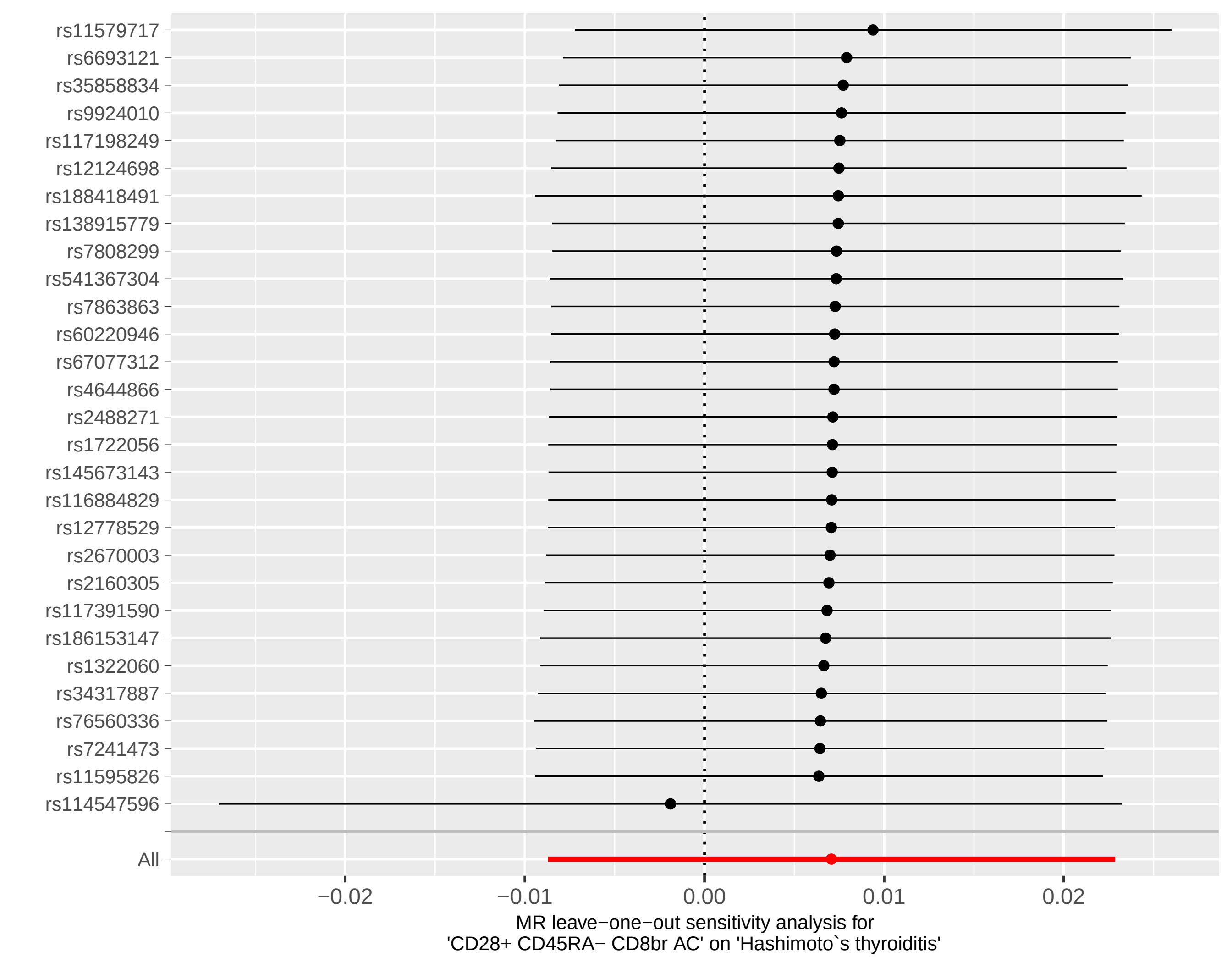
rs11579717
rs6693121
rs35858834
rs9924010
rs117198249
rs12124698
rs188418491
rs138915779
rs7808299
rs541367304
rs7863863
rs60220946
rs67077312
rs4644866
rs2488271
rs1722056
rs145673143
rs116884829
rs12778529
rs2670003
rs2160305
rs117391590
rs186153147
rs1322060
rs34317887
rs76560336
rs7241473
rs11595826
rs114547596
All
−0.02
−0.01
0.00
0.01
0.02
MR leave−one−out sensitivity analysis for
'CD28+ CD45RA− CD8br AC' on 'Hashimoto`s thyroiditis'

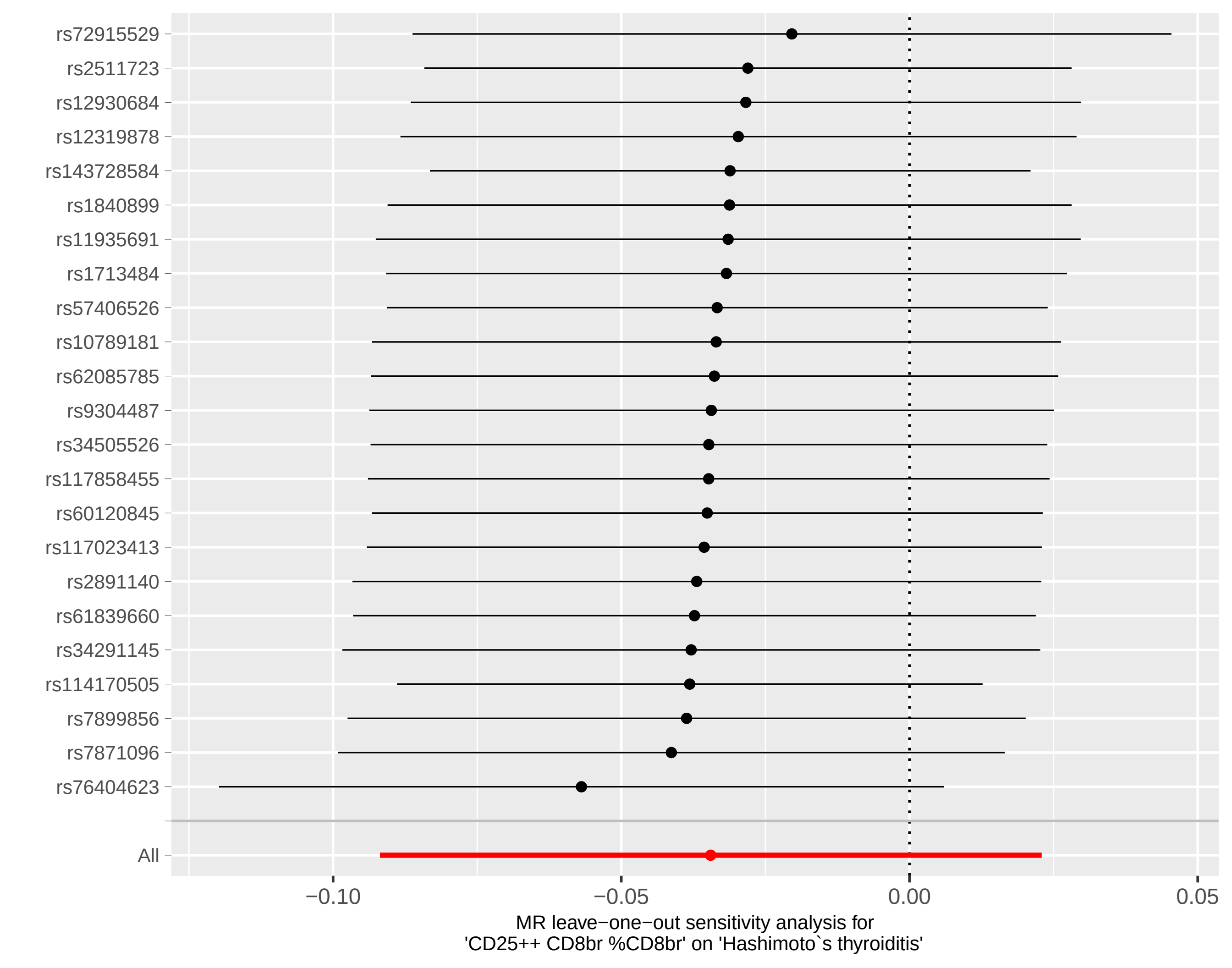

rs72915529
rs2511723
rs12930684
rs12319878
rs143728584
rs1840899
rs11935691
rs1713484
rs57406526
rs10789181
rs62085785
rs9304487
rs34505526
rs117858455
rs60120845
rs117023413
rs2891140
rs61839660
rs34291145
rs114170505
rs7899856
rs7871096
rs76404623
All
−0.10
−0.05
0.00
0.05
MR leave−one−out sensitivity analysis for
'CD25++ CD8br %CD8br' on 'Hashimoto`s thyroiditis'

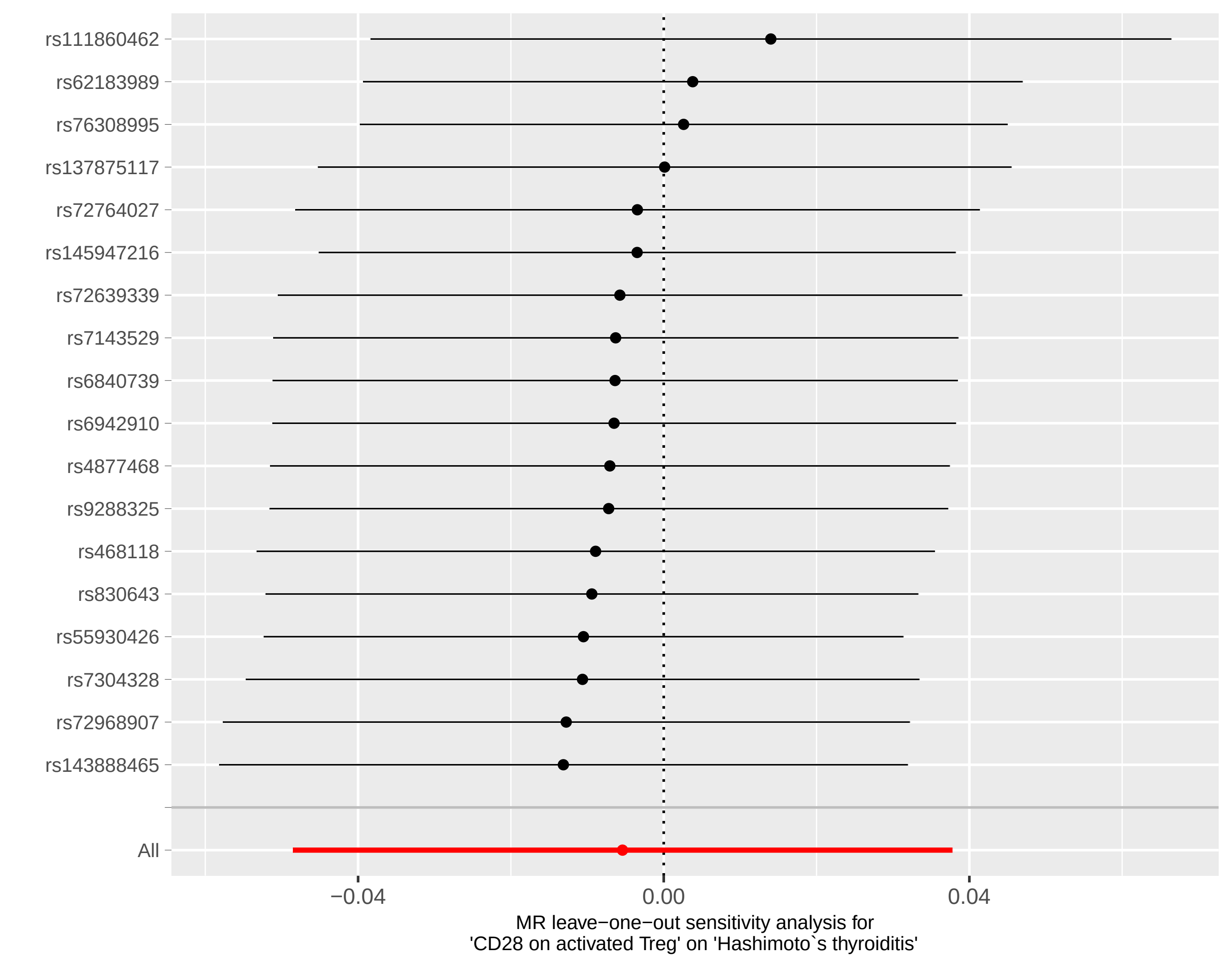
rs111860462
rs62183989
rs76308995
rs137875117
rs72764027
rs145947216
rs72639339
rs7143529
rs6840739
rs6942910
rs4877468
rs9288325
rs468118
rs830643
rs55930426
rs7304328
rs72968907
rs143888465
All
−0.04
0.00
0.04
MR leave−one−out sensitivity analysis for
'CD28 on activated Treg' on 'Hashimoto`s thyroiditis'

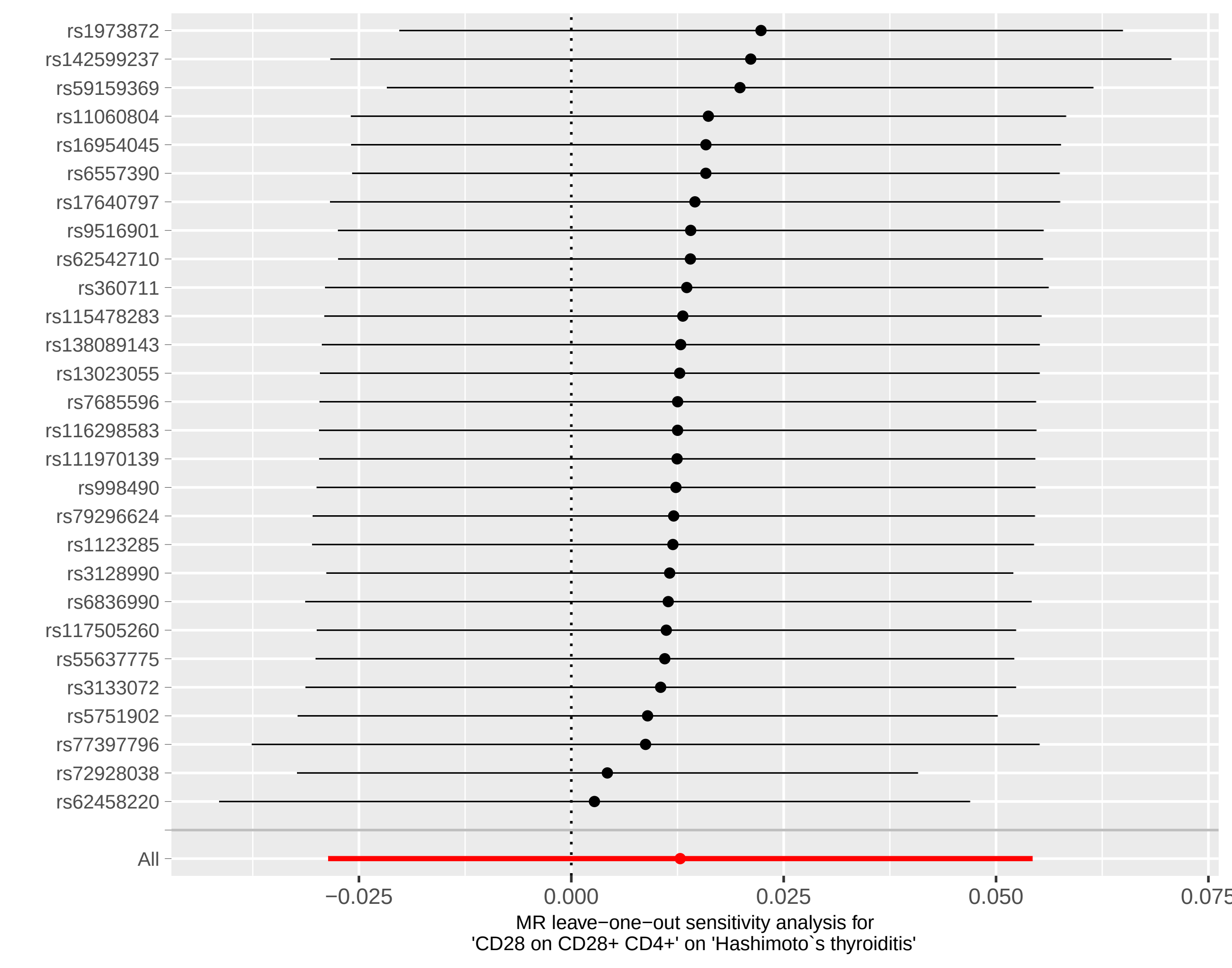
rs1973872
rs142599237
rs59159369
rs11060804
rs16954045
rs6557390
rs17640797
rs9516901
rs62542710
rs360711
rs115478283
rs138089143
rs13023055
rs7685596
rs116298583
rs111970139
rs998490
rs79296624
rs1123285
rs3128990
rs6836990
rs117505260
rs55637775
rs3133072
rs5751902
rs77397796
rs72928038
rs62458220
All
−0.025
0.000
0.025
0.050
0.075
MR leave−one−out sensitivity analysis for
'CD28 on CD28+ CD4+' on 'Hashimoto`s thyroiditis'

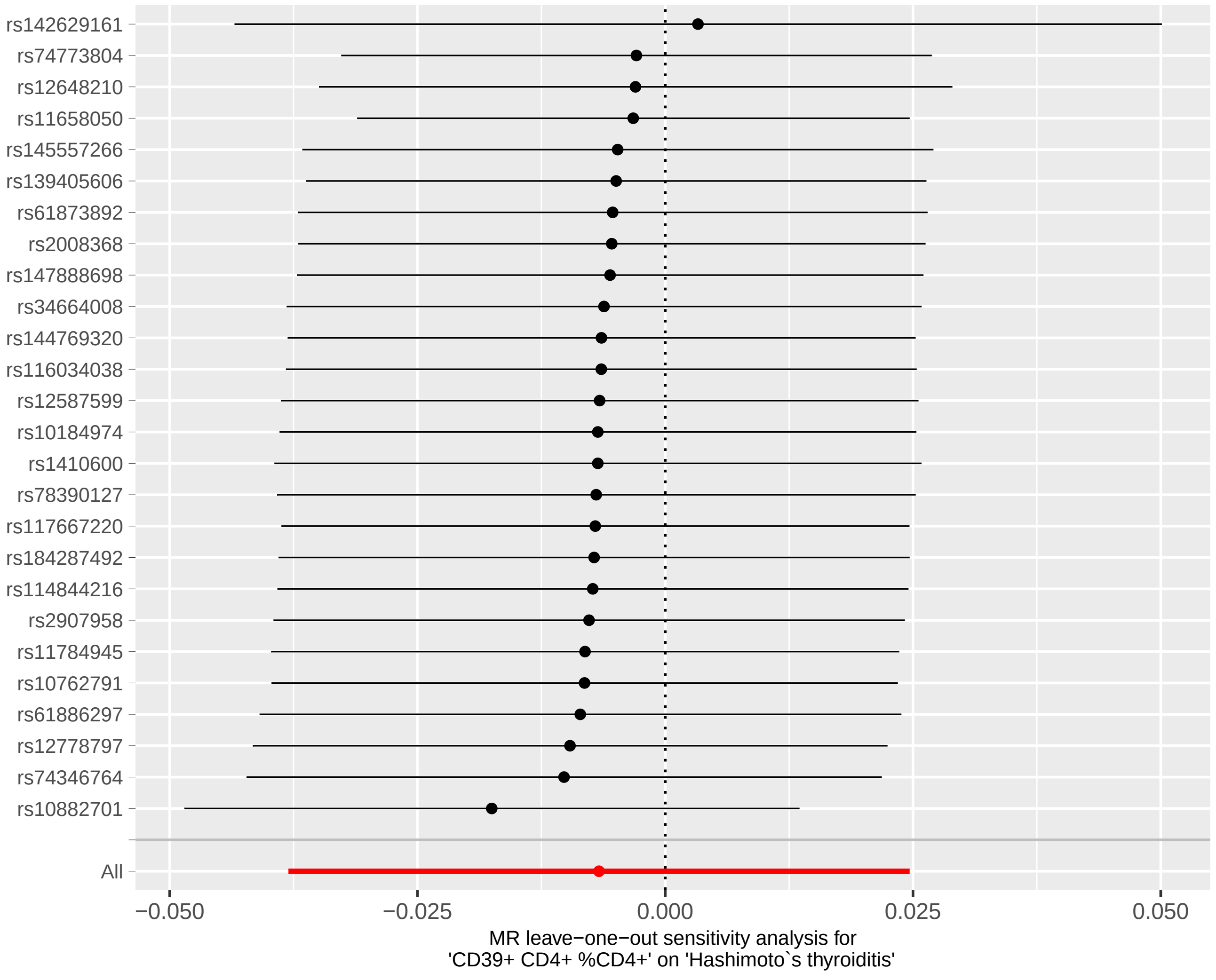
rs142629161
rs74773804
rs12648210
rs11658050
rs145557266
rs139405606
rs61873892
rs2008368
rs147888698
rs34664008
rs144769320
rs116034038
rs12587599
rs10184974
rs1410600
rs78390127
rs117667220
rs184287492
rs114844216
rs2907958
rs11784945
rs10762791
rs61886297
rs12778797
rs74346764
rs10882701
All
−0.050
−0.025
0.000
0.025
0.050
MR leave−one−out sensitivity analysis for
'CD39+ CD4+ %CD4+' on 'Hashimoto`s thyroiditis'

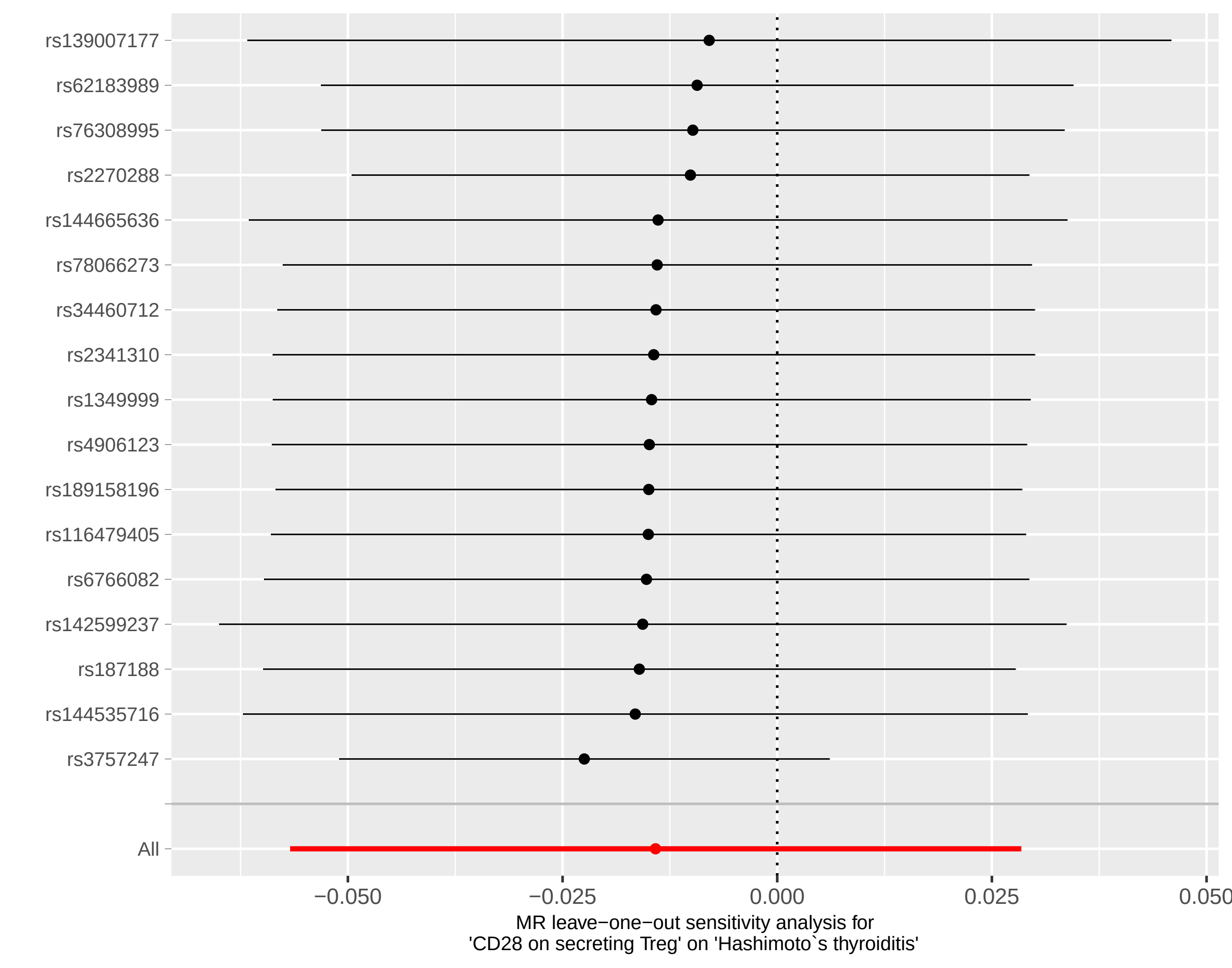
rs139007177
rs62183989
rs76308995
rs2270288
rs144665636
rs78066273
rs34460712
rs2341310
rs1349999
rs4906123
rs189158196
rs116479405
rs6766082
rs142599237
rs187188
rs144535716
rs3757247
All
−0.050
−0.025
0.000
0.025
0.050
MR leave−one−out sensitivity analysis for
'CD28 on secreting Treg' on 'Hashimoto`s thyroiditis'

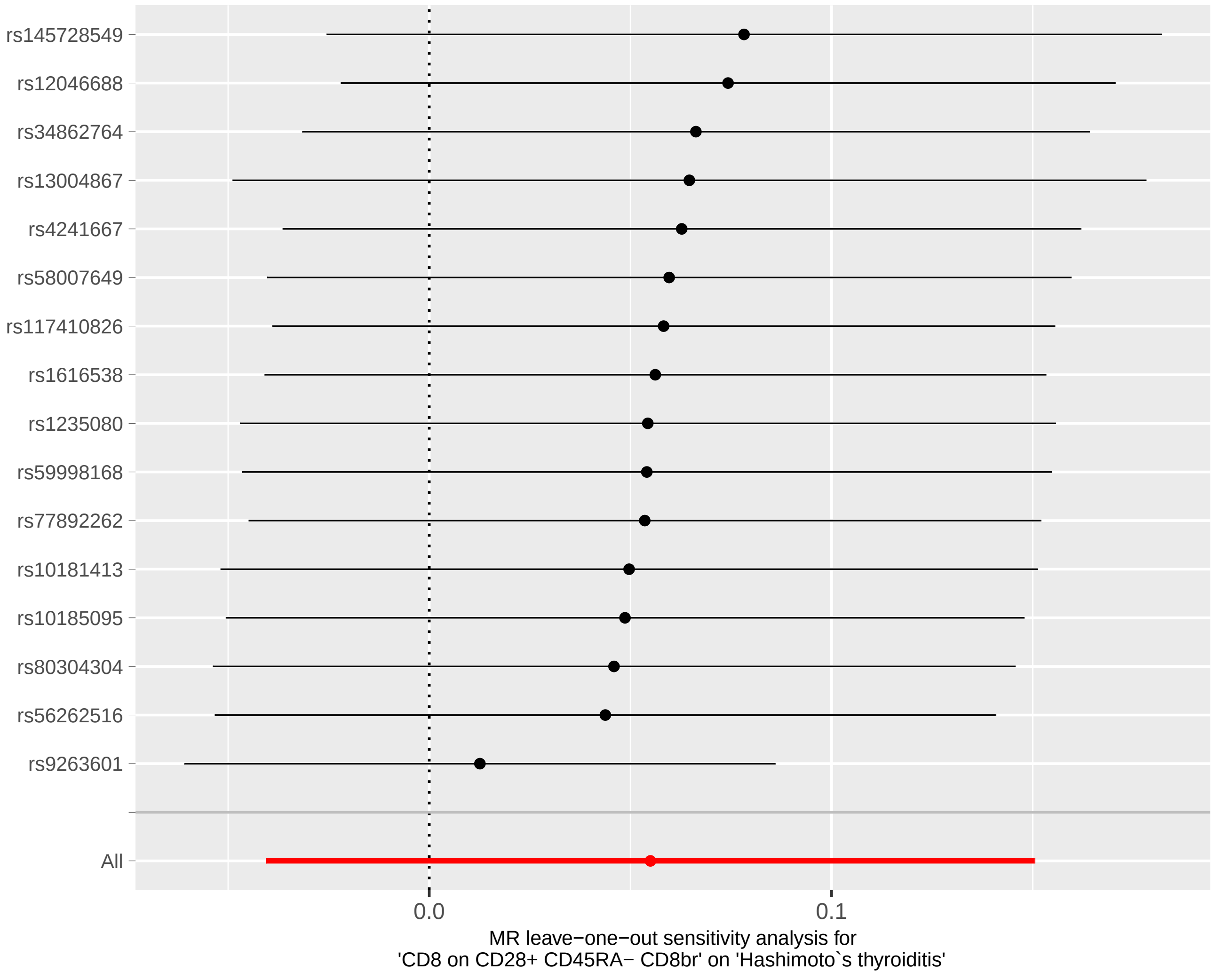
rs145728549
rs12046688
rs34862764
rs13004867
rs4241667
rs58007649
rs117410826
rs1616538
rs1235080
rs59998168
rs77892262
rs10181413
rs10185095
rs80304304
rs56262516
rs9263601
All
0.0
0.1
MR leave−one−out sensitivity analysis for
'CD8 on CD28+ CD45RA− CD8br' on 'Hashimoto`s thyroiditis'

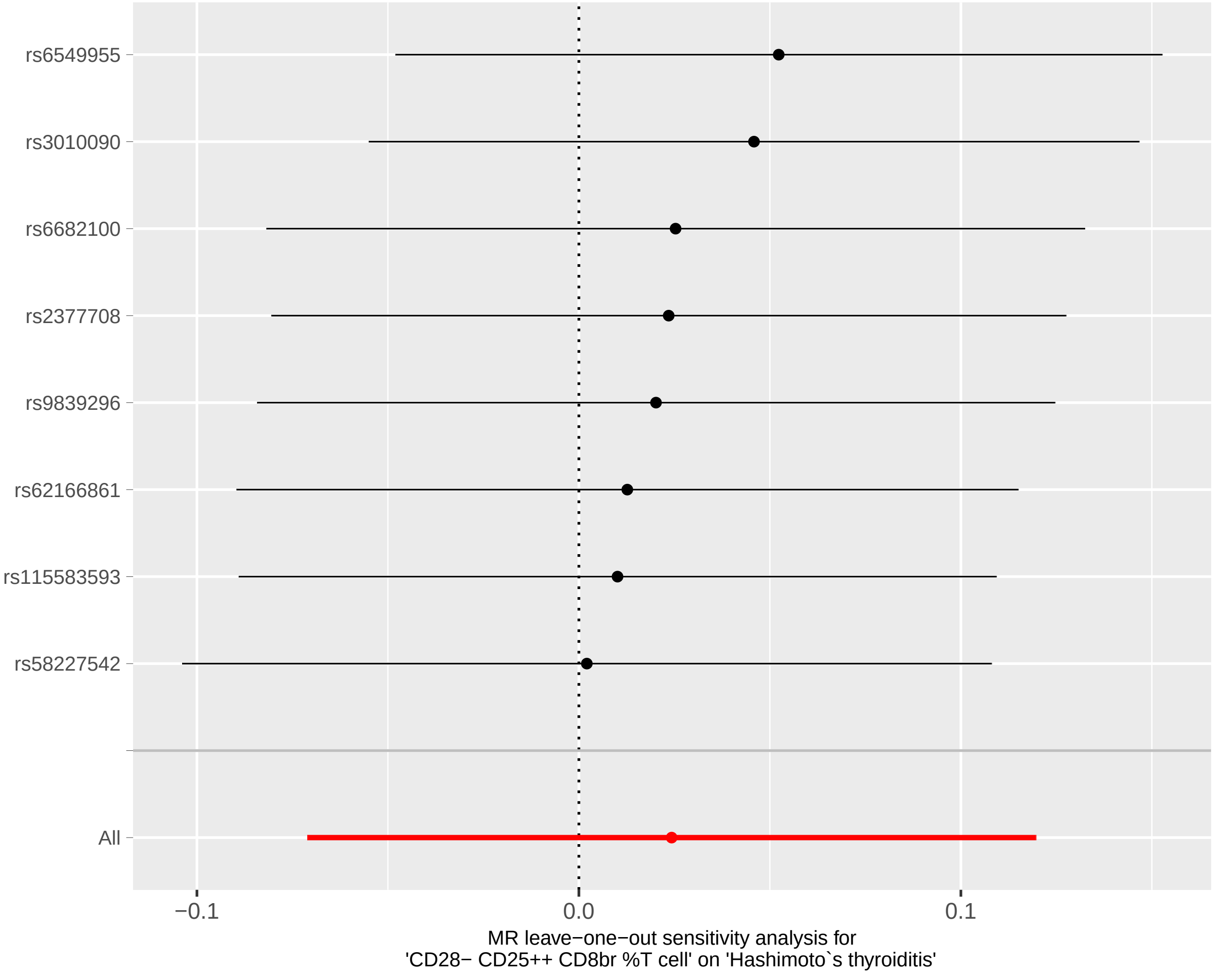
rs6549955
rs3010090
rs6682100
rs2377708
rs9839296
rs62166861
rs115583593
rs58227542
All
−0.1
0.0
0.1
MR leave−one−out sensitivity analysis for
'CD28− CD25++ CD8br %T cell' on 'Hashimoto`s thyroiditis'

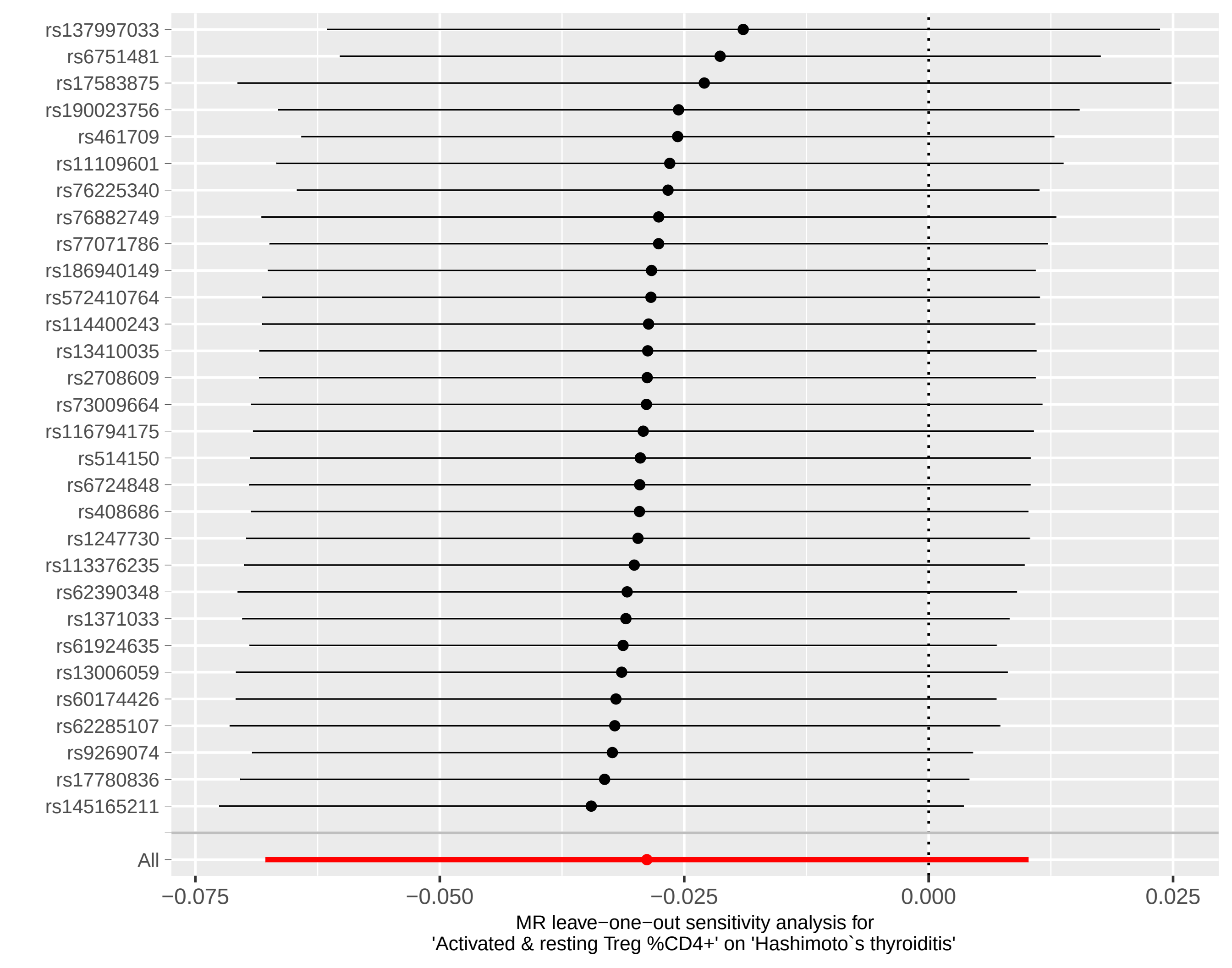

rs137997033
rs6751481
rs17583875
rs190023756
rs461709
rs11109601
rs76225340
rs76882749
rs77071786
rs186940149
rs572410764
rs114400243
rs13410035
rs2708609
rs73009664
rs116794175
rs514150
rs6724848
rs408686
rs1247730
rs113376235
rs62390348
rs1371033
rs61924635
rs13006059
rs60174426
rs62285107
rs9269074
rs17780836
rs145165211
All
−0.075
−0.050
−0.025
0.000
0.025
MR leave−one−out sensitivity analysis for
'Activated & resting Treg %CD4+' on 'Hashimoto`s thyroiditis'

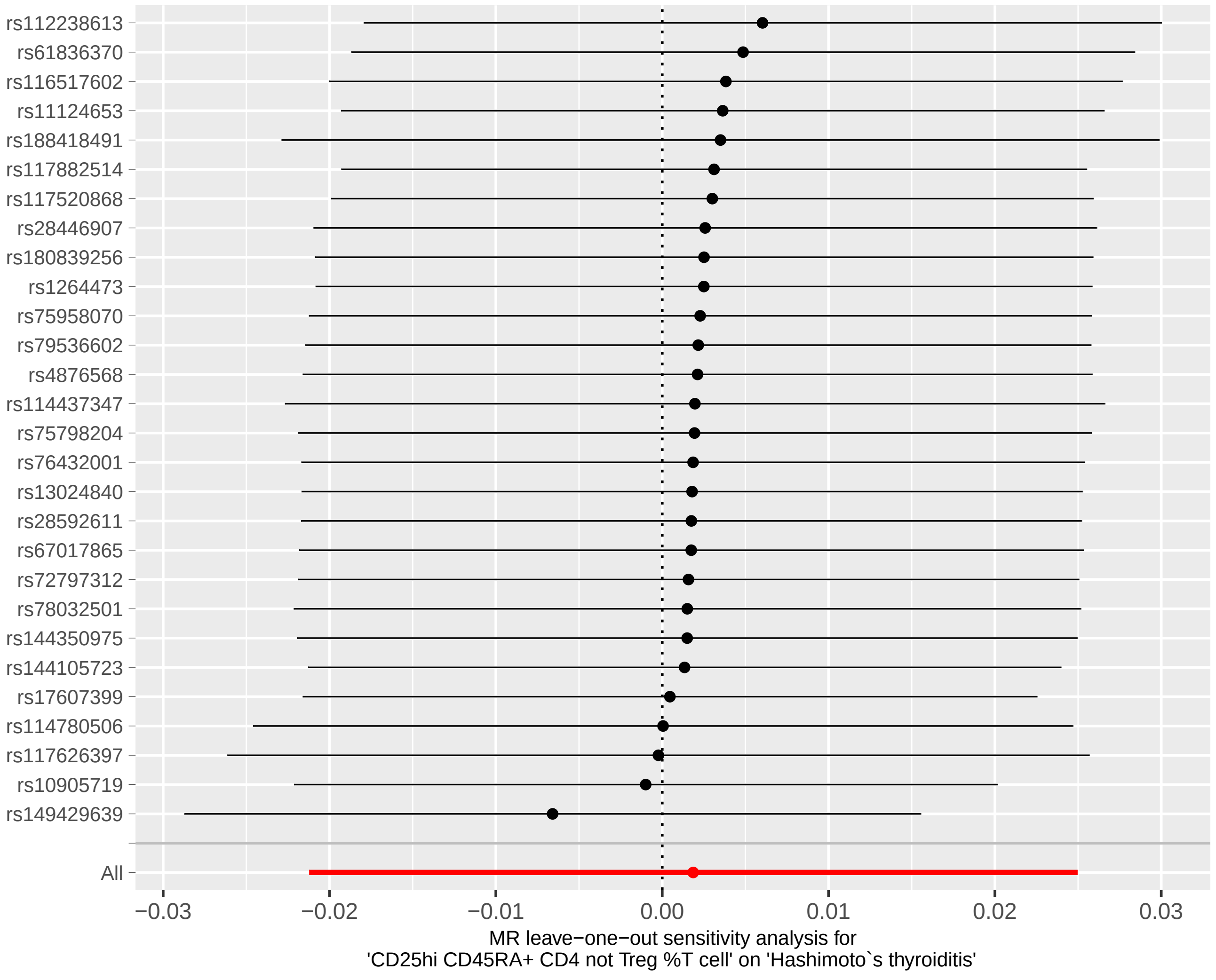

rs112238613
rs61836370
rs116517602
rs11124653
rs188418491
rs117882514
rs117520868
rs28446907
rs180839256
rs1264473
rs75958070
rs79536602
rs4876568
rs114437347
rs75798204
rs76432001
rs13024840
rs28592611
rs67017865
rs72797312
rs78032501
rs144350975
rs144105723
rs17607399
rs114780506
rs117626397
rs10905719
rs149429639
All
−0.03
−0.02
−0.01
0.00
0.01
0.02
0.03
MR leave−one−out sensitivity analysis for
'CD25hi CD45RA+ CD4 not Treg %T cell' on 'Hashimoto`s thyroiditis'

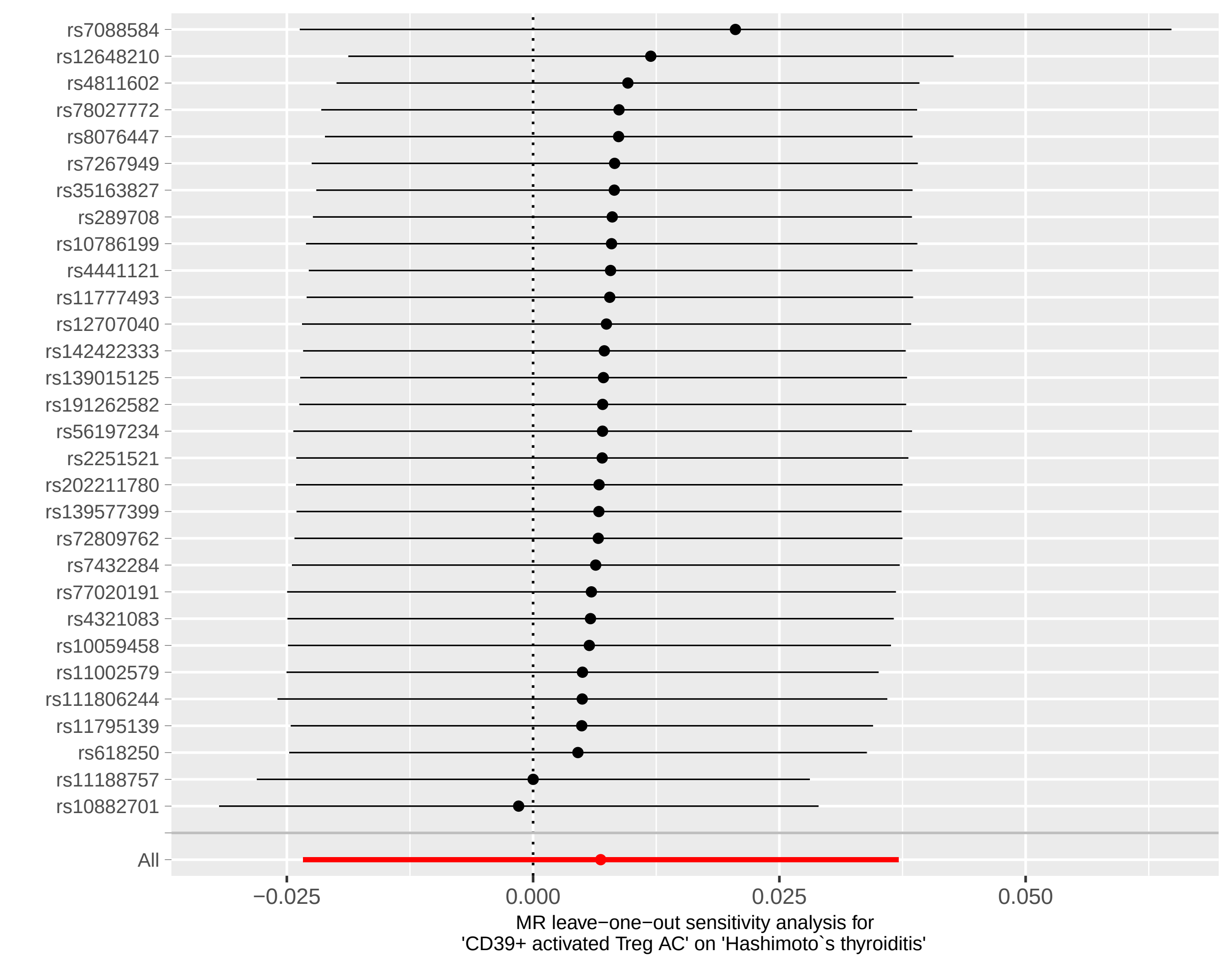

rs7088584
rs12648210
rs4811602
rs78027772
rs8076447
rs7267949
rs35163827
rs289708
rs10786199
rs4441121
rs11777493
rs12707040
rs142422333
rs139015125
rs191262582
rs56197234
rs2251521
rs202211780
rs139577399
rs72809762
rs7432284
rs77020191
rs4321083
rs10059458
rs11002579
rs111806244
rs11795139
rs618250
rs11188757
rs10882701
All
−0.025
0.000
0.025
0.050
MR leave−one−out sensitivity analysis for
'CD39+ activated Treg AC' on 'Hashimoto`s thyroiditis'

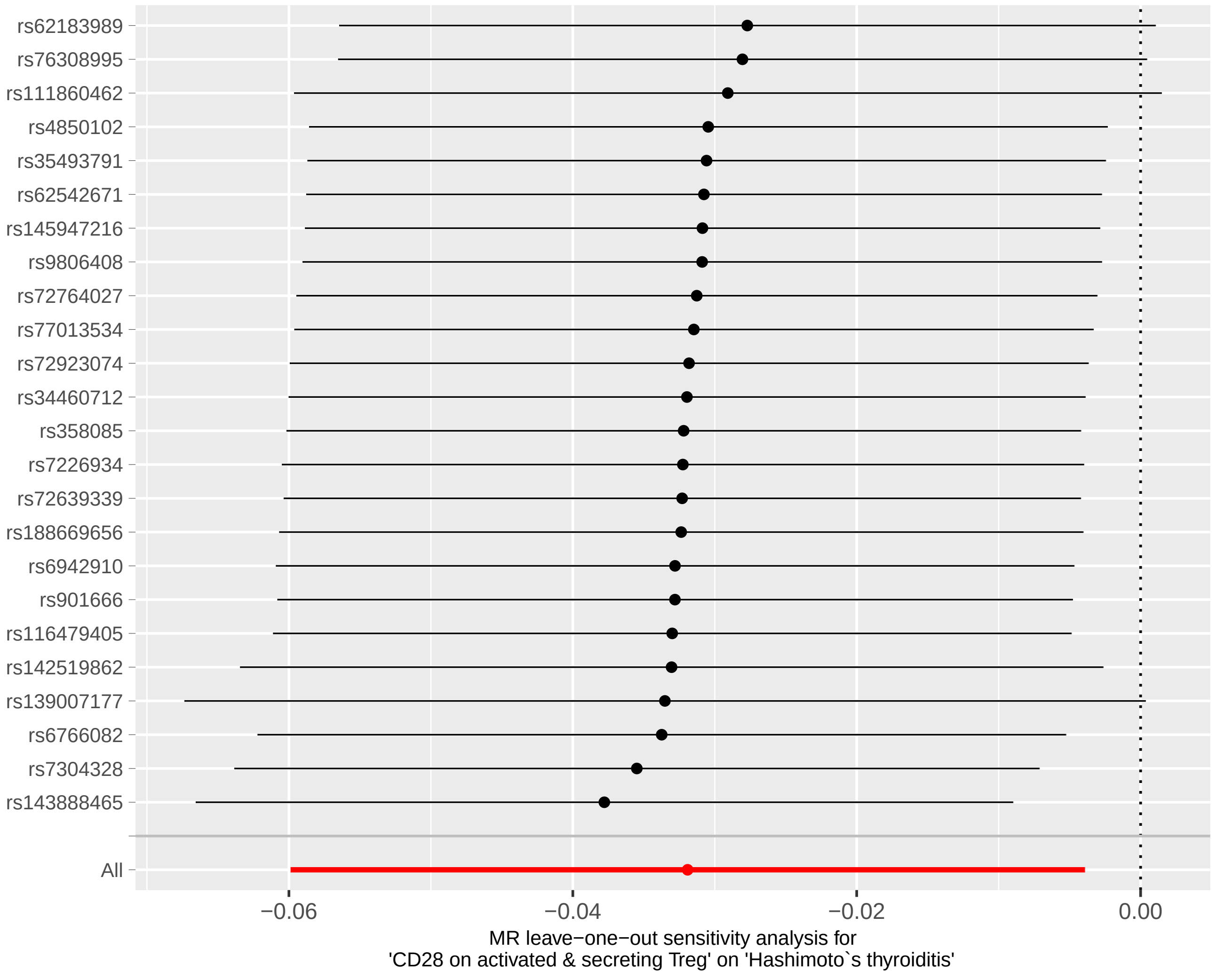
rs62183989
rs76308995
rs111860462
rs4850102
rs35493791
rs62542671
rs145947216
rs9806408
rs72764027
rs77013534
rs72923074
rs34460712
rs358085
rs7226934
rs72639339
rs188669656
rs6942910
rs901666
rs116479405
rs142519862
rs139007177
rs6766082
rs7304328
rs143888465
All
−0.06
−0.04
−0.02
0.00
MR leave−one−out sensitivity analysis for
'CD28 on activated & secreting Treg' on 'Hashimoto`s thyroiditis'

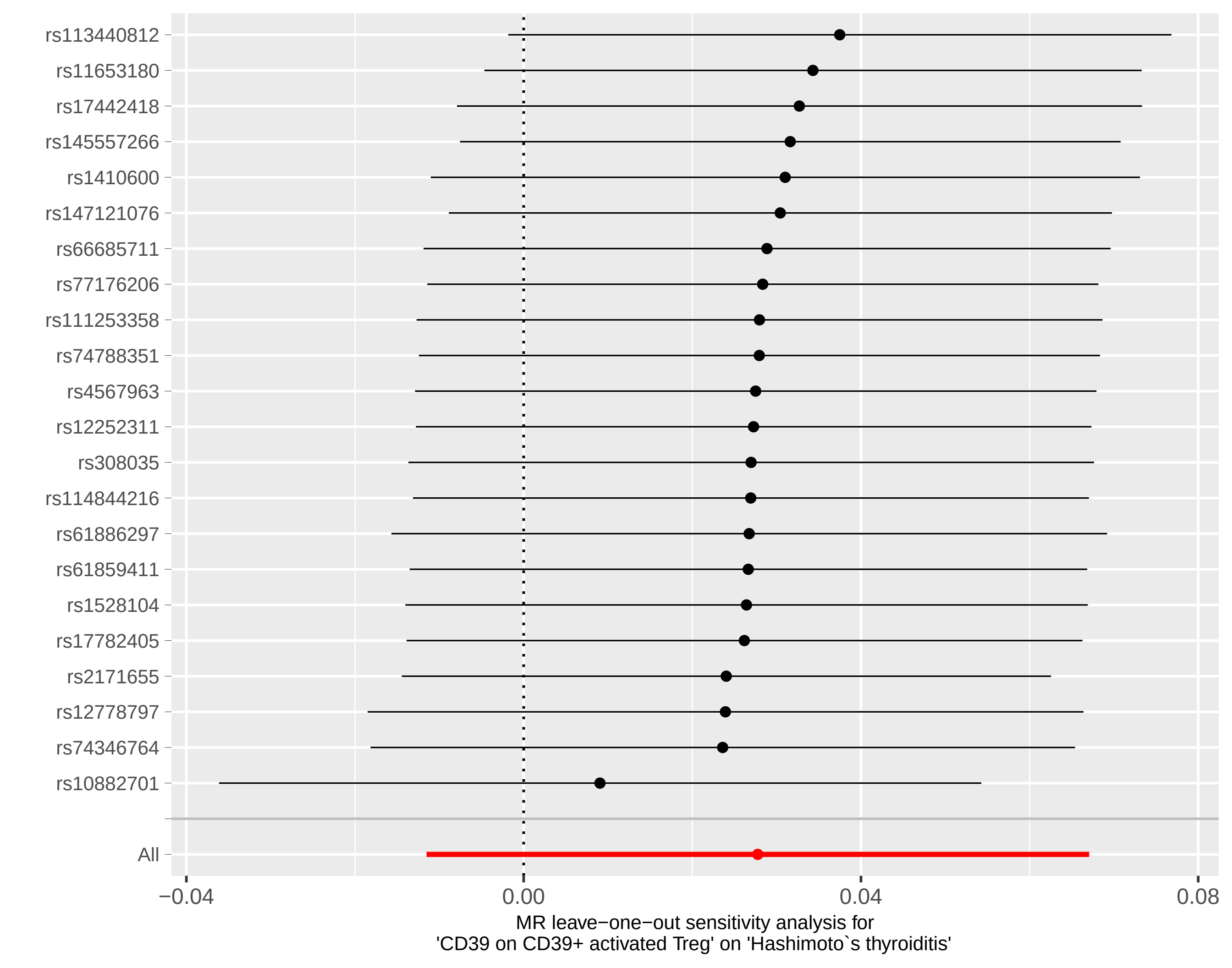
rs113440812
rs11653180
rs17442418
rs145557266
rs1410600
rs147121076
rs66685711
rs77176206
rs111253358
rs74788351
rs4567963
rs12252311
rs308035
rs114844216
rs61886297
rs61859411
rs1528104
rs17782405
rs2171655
rs12778797
rs74346764
rs10882701
All
−0.04
0.00
0.04
0.08
MR leave−one−out sensitivity analysis for
'CD39 on CD39+ activated Treg' on 'Hashimoto`s thyroiditis'

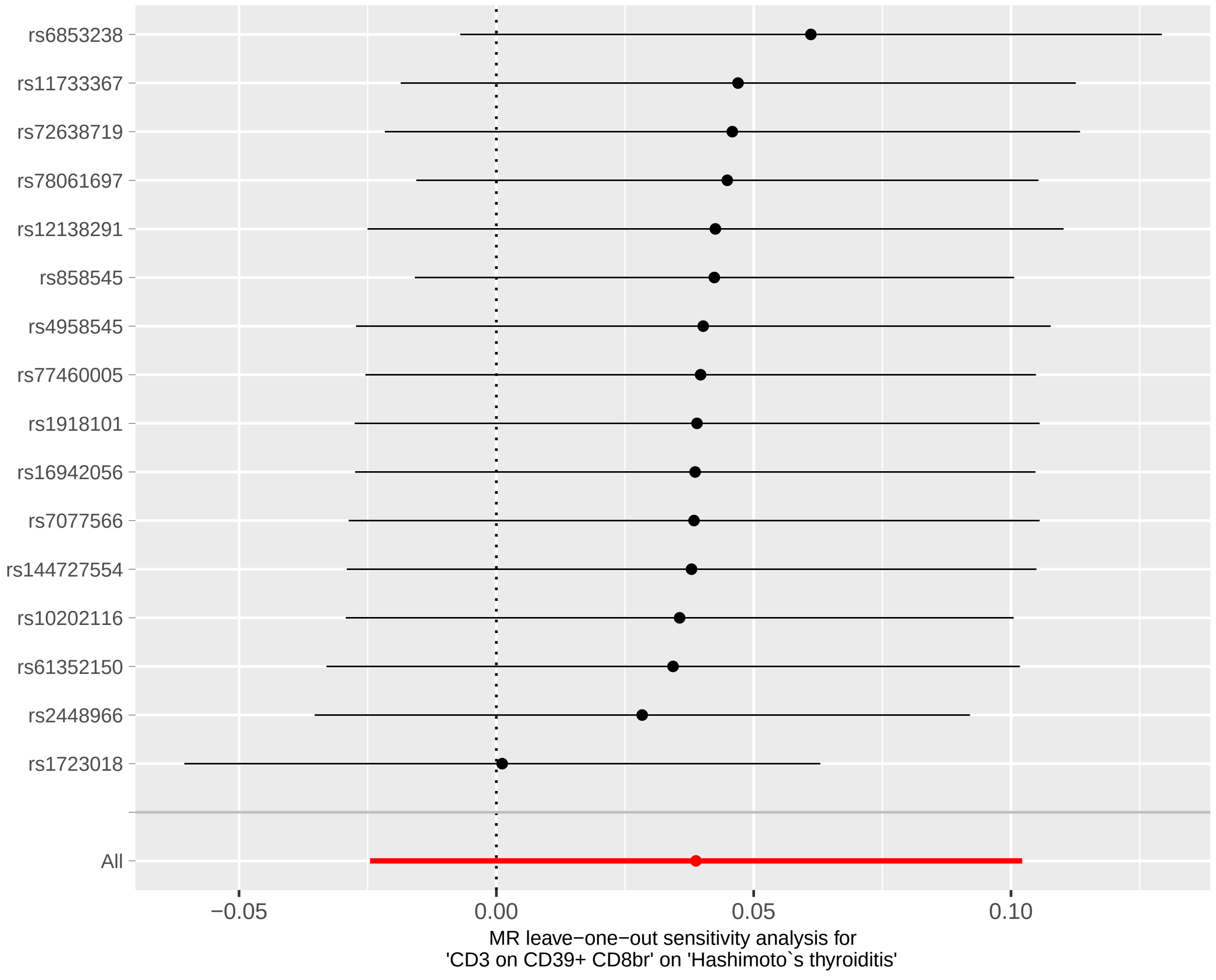
rs6853238
rs11733367
rs72638719
rs78061697
rs12138291
rs858545
rs4958545
rs77460005
rs1918101
rs16942056
rs7077566
rs144727554
rs10202116
rs61352150
rs2448966
rs1723018
All
−0.05
0.00
0.05
0.10
MR leave−one−out sensitivity analysis for
'CD3 on CD39+ CD8br' on 'Hashimoto`s thyroiditis'

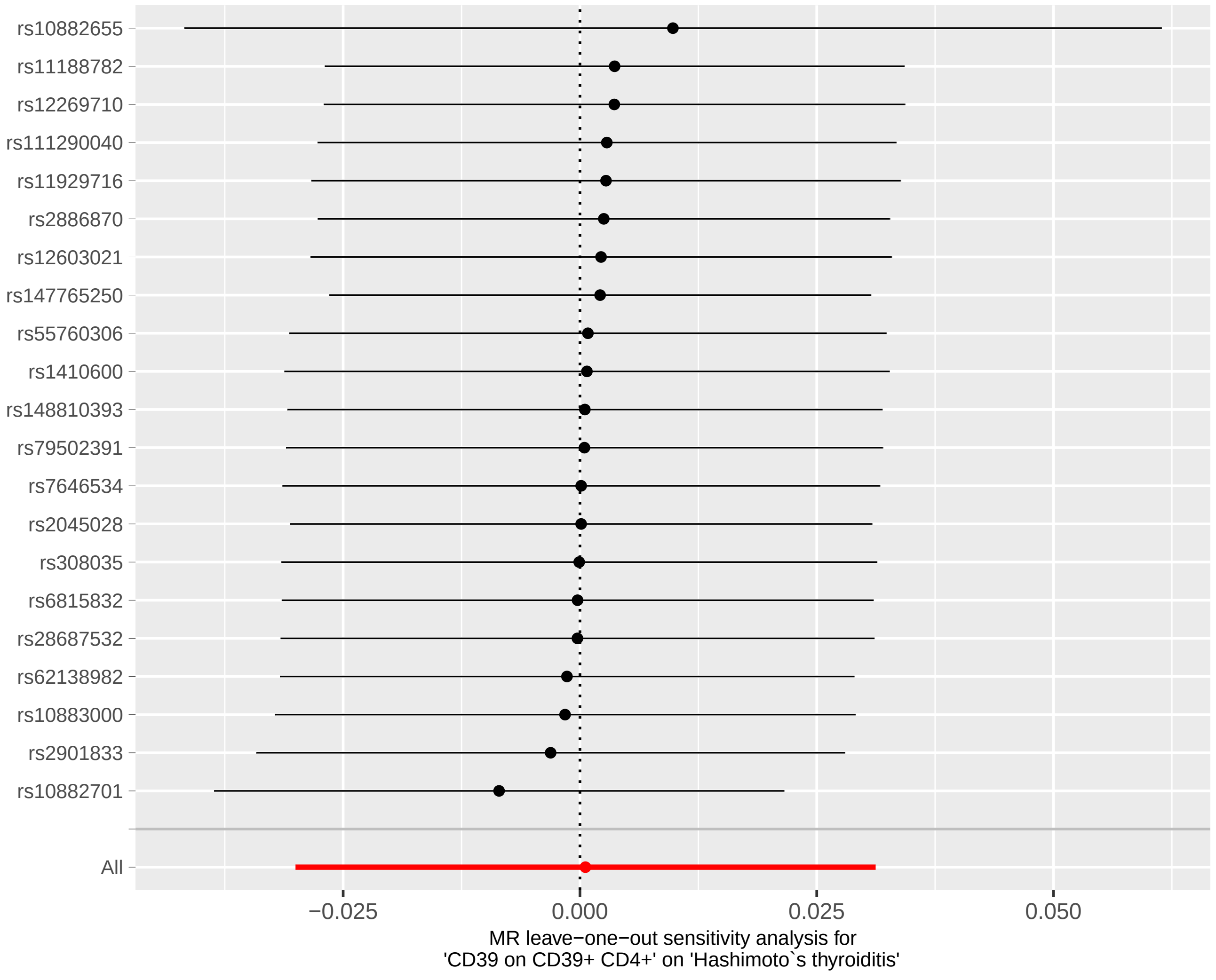

rs10882655
rs11188782
rs12269710
rs111290040
rs11929716
rs2886870
rs12603021
rs147765250
rs55760306
rs1410600
rs148810393
rs79502391
rs7646534
rs2045028
rs308035
rs6815832
rs28687532
rs62138982
rs10883000
rs2901833
rs10882701
All
−0.025
0.000
0.025
0.050
MR leave−one−out sensitivity analysis for
'CD39 on CD39+ CD4+' on 'Hashimoto`s thyroiditis'

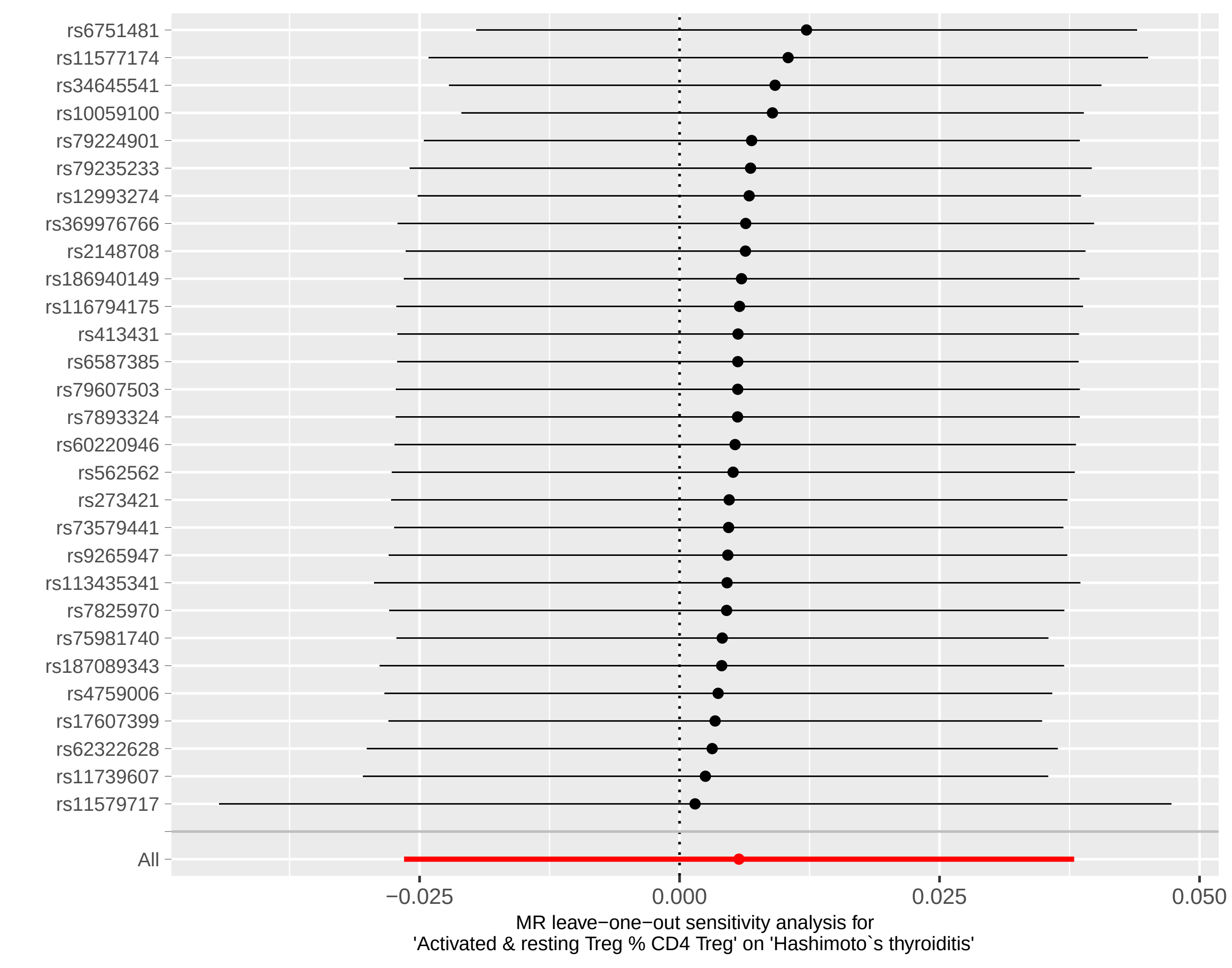

rs6751481
rs11577174
rs34645541
rs10059100
rs79224901
rs79235233
rs12993274
rs369976766
rs2148708
rs186940149
rs116794175
rs413431
rs6587385
rs79607503
rs7893324
rs60220946
rs562562
rs273421
rs73579441
rs9265947
rs113435341
rs7825970
rs75981740
rs187089343
rs4759006
rs17607399
rs62322628
rs11739607
rs11579717
All
−0.025
0.000
0.025
0.050
MR leave−one−out sensitivity analysis for
'Activated & resting Treg % CD4 Treg' on 'Hashimoto`s thyroiditis'

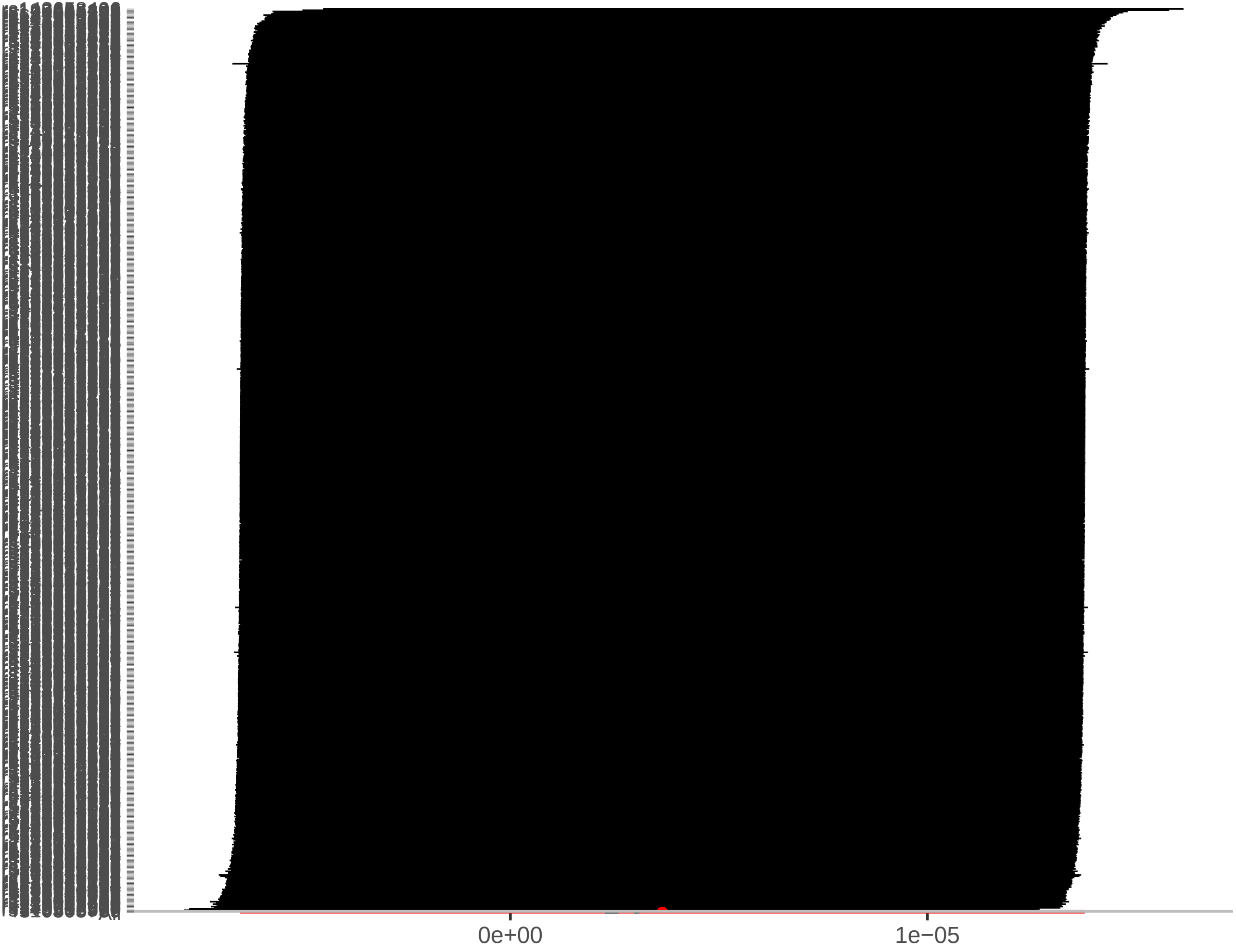

MR leave−one−out sensitivity analysis for
'CD45RA+ CD28− CD8br AC' on 'Hashimoto`s thyroiditis'

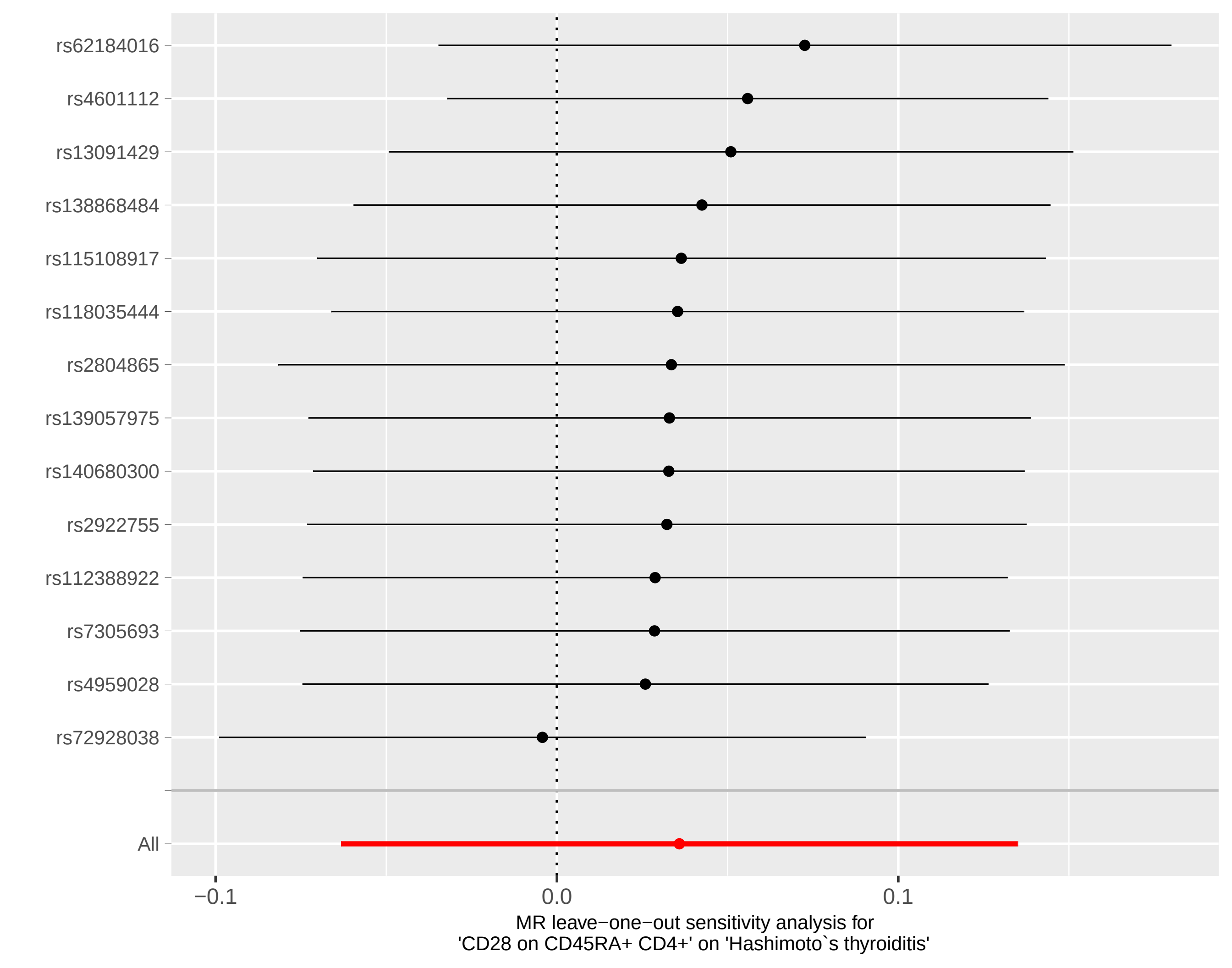
rs62184016
rs4601112
rs13091429
rs138868484
rs115108917
rs118035444
rs2804865
rs139057975
rs140680300
rs2922755
rs112388922
rs7305693
rs4959028
rs72928038
All
−0.1
0.0
0.1
MR leave−one−out sensitivity analysis for
'CD28 on CD45RA+ CD4+' on 'Hashimoto`s thyroiditis'

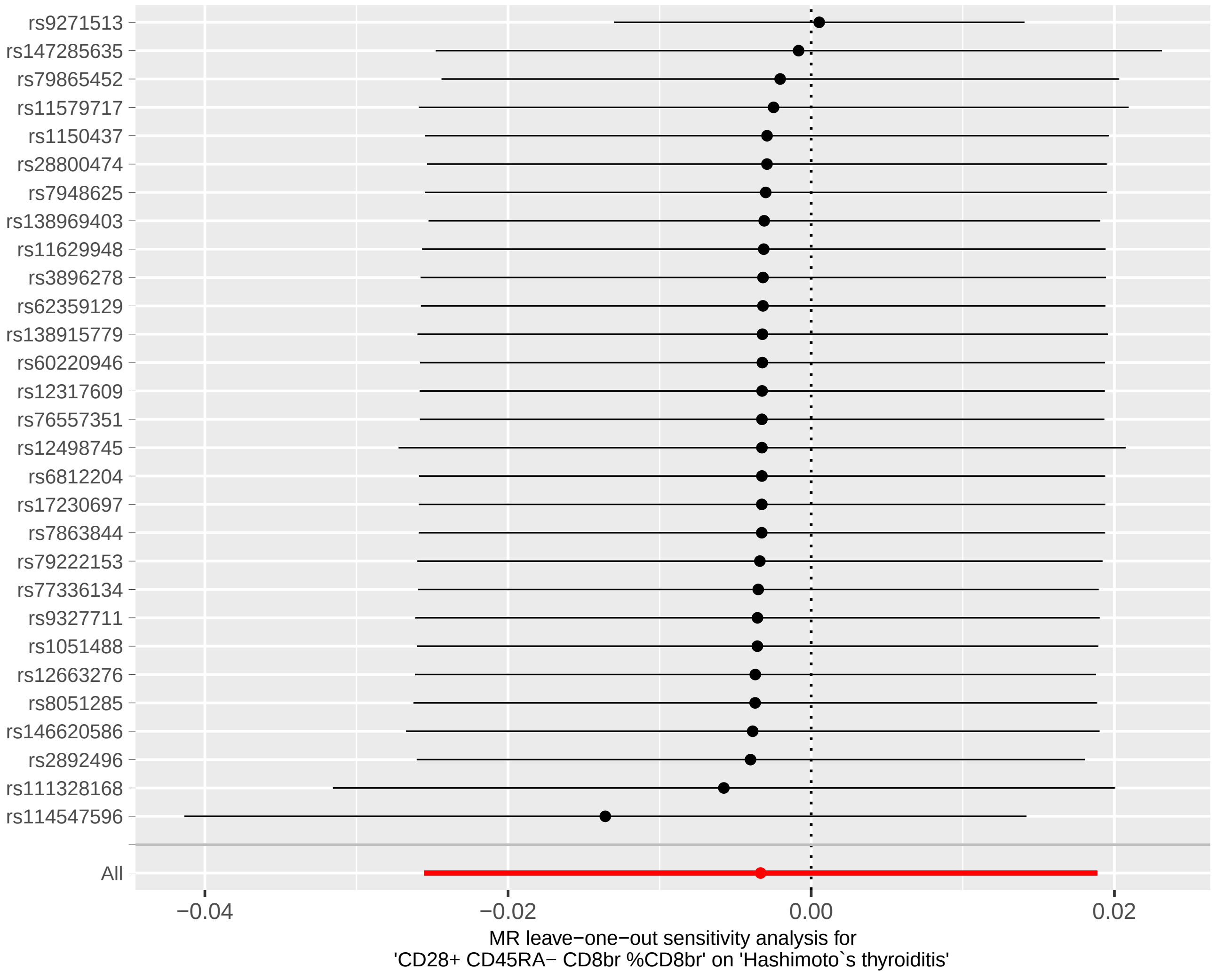
rs9271513
rs147285635
rs79865452
rs11579717
rs1150437
rs28800474
rs7948625
rs138969403
rs11629948
rs3896278
rs62359129
rs138915779
rs60220946
rs12317609
rs76557351
rs12498745
rs6812204
rs17230697
rs7863844
rs79222153
rs77336134
rs9327711
rs1051488
rs12663276
rs8051285
rs146620586
rs2892496
rs111328168
rs114547596
All
−0.04
−0.02
0.00
0.02
MR leave−one−out sensitivity analysis for
'CD28+ CD45RA− CD8br %CD8br' on 'Hashimoto`s thyroiditis'

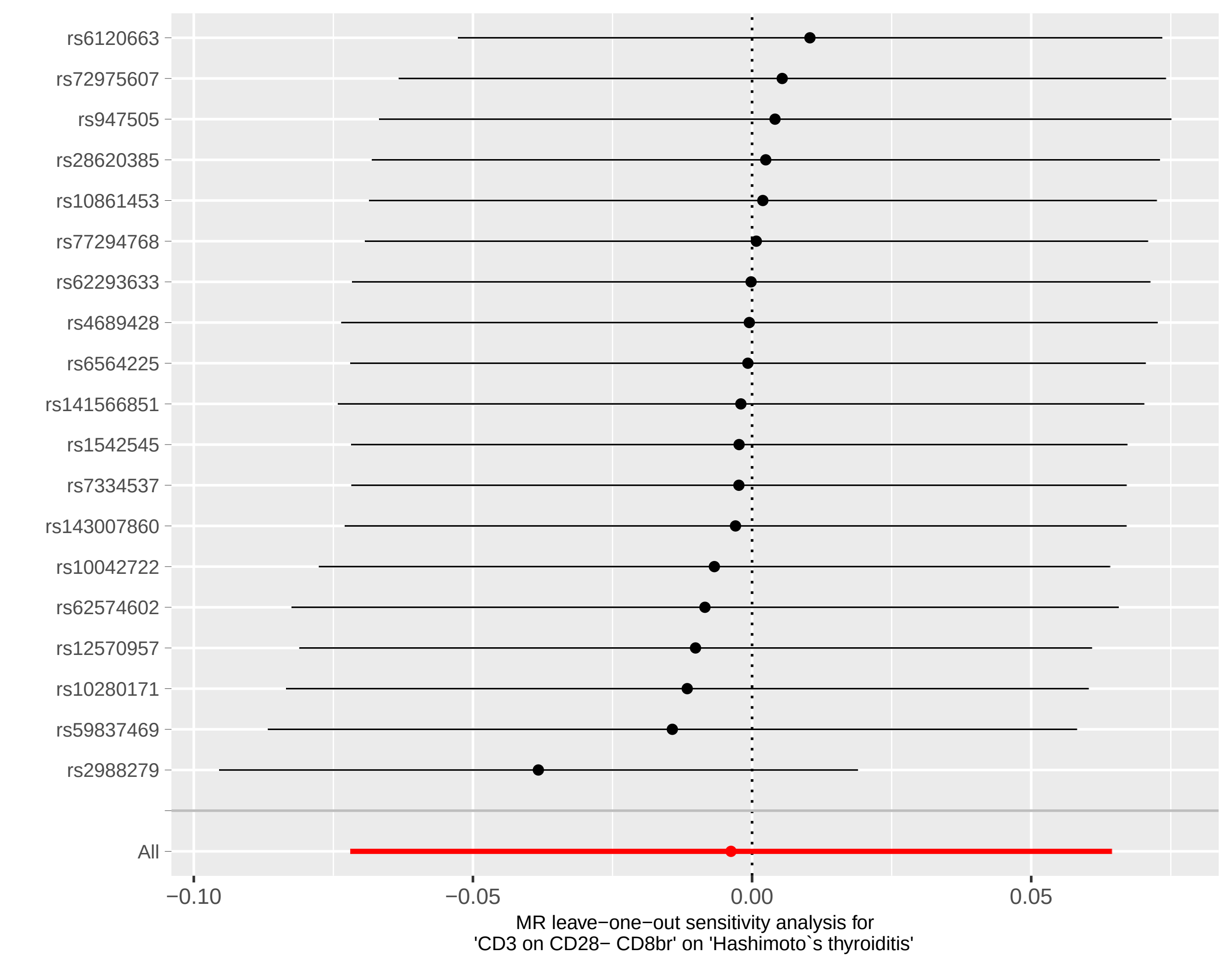
rs6120663
rs72975607
rs947505
rs28620385
rs10861453
rs77294768
rs62293633
rs4689428
rs6564225
rs141566851
rs1542545
rs7334537
rs143007860
rs10042722
rs62574602
rs12570957
rs10280171
rs59837469
rs2988279
All
−0.10
−0.05
0.00
0.05
MR leave−one−out sensitivity analysis for
'CD3 on CD28− CD8br' on 'Hashimoto`s thyroiditis'

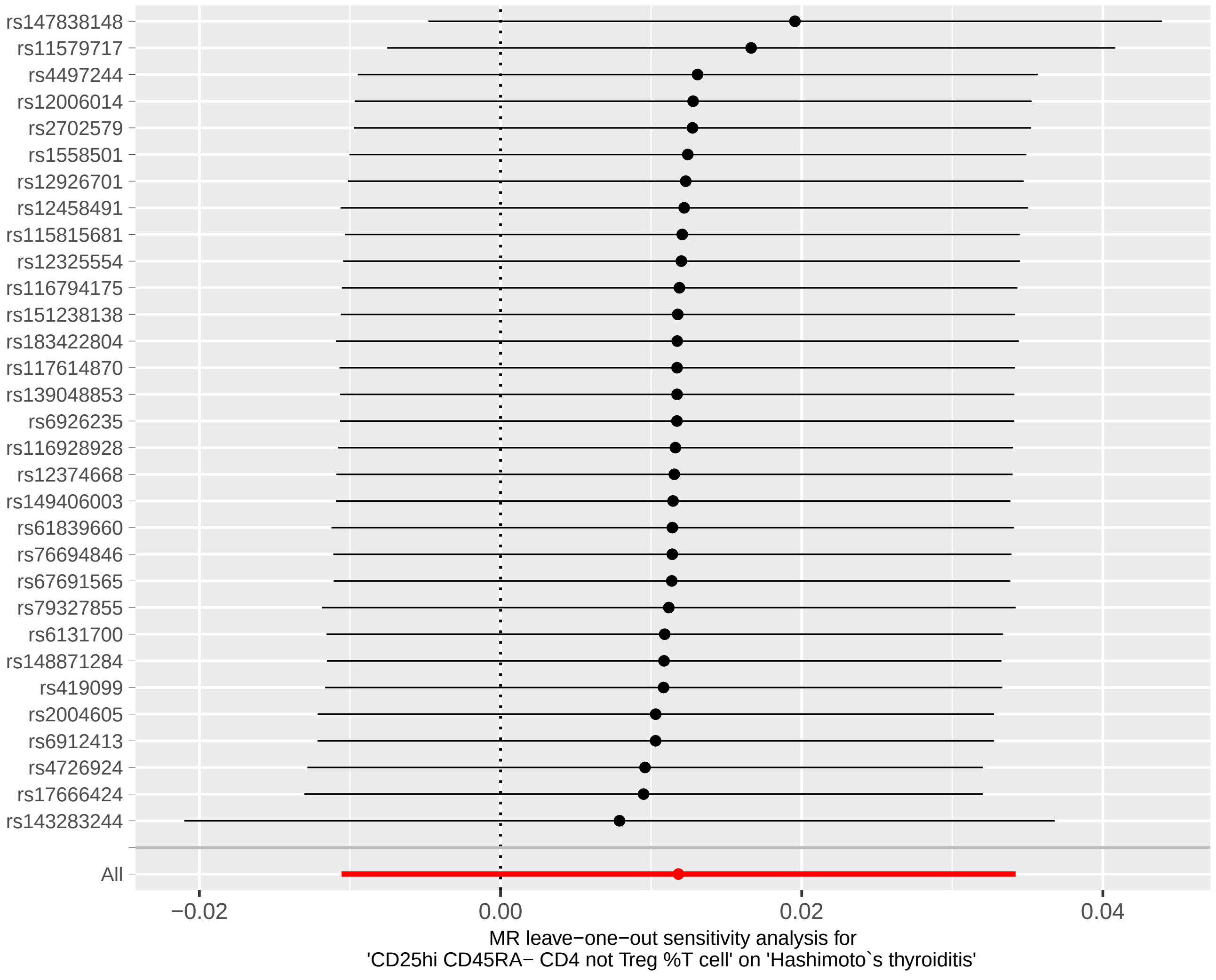
rs147838148
rs11579717
rs4497244
rs12006014
rs2702579
rs1558501
rs12926701
rs12458491
rs115815681
rs12325554
rs116794175
rs151238138
rs183422804
rs117614870
rs139048853
rs6926235
rs116928928
rs12374668
rs149406003
rs61839660
rs76694846
rs67691565
rs79327855
rs6131700
rs148871284
rs419099
rs2004605
rs6912413
rs4726924
rs17666424
rs143283244
All
−0.02
0.00
0.02
0.04
MR leave−one−out sensitivity analysis for
'CD25hi CD45RA− CD4 not Treg %T cell' on 'Hashimoto`s thyroiditis'

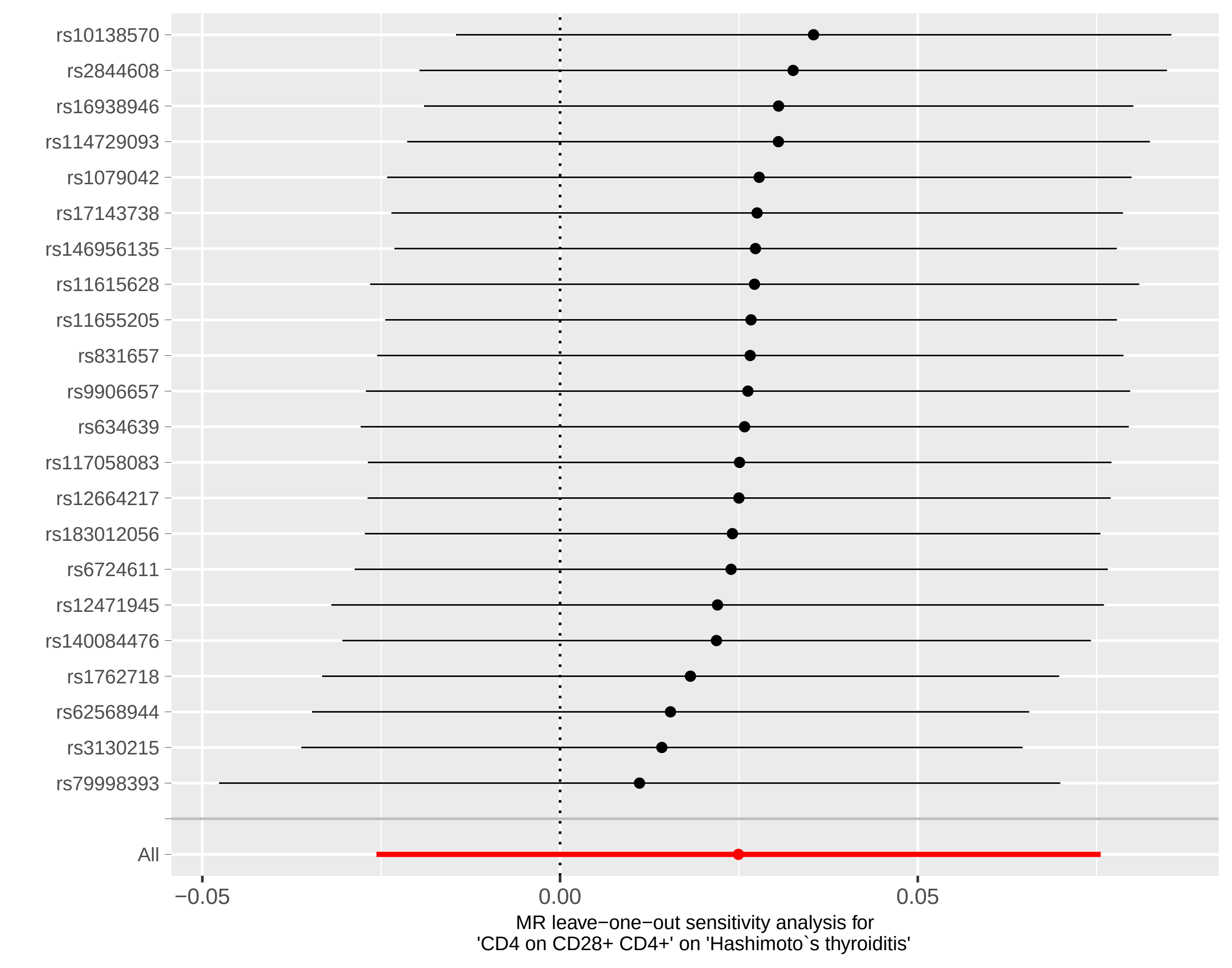
rs10138570
rs2844608
rs16938946
rs114729093
rs1079042
rs17143738
rs146956135
rs11615628
rs11655205
rs831657
rs9906657
rs634639
rs117058083
rs12664217
rs183012056
rs6724611
rs12471945
rs140084476
rs1762718
rs62568944
rs3130215
rs79998393
All
−0.05
0.00
0.05
MR leave−one−out sensitivity analysis for
'CD4 on CD28+ CD4+' on 'Hashimoto`s thyroiditis'

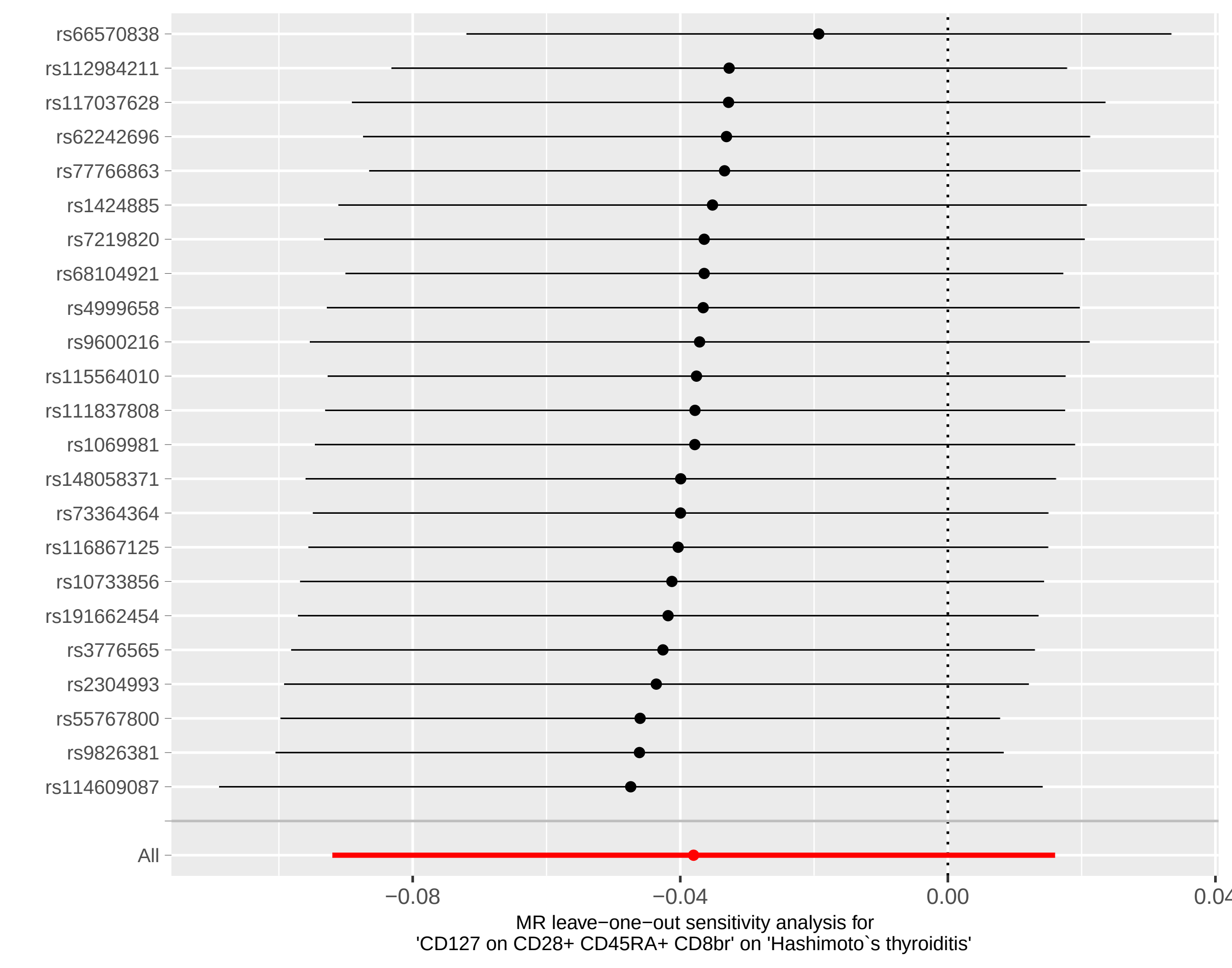

rs66570838
rs112984211
rs117037628
rs62242696
rs77766863
rs1424885
rs7219820
rs68104921
rs4999658
rs9600216
rs115564010
rs111837808
rs1069981
rs148058371
rs73364364
rs116867125
rs10733856
rs191662454
rs3776565
rs2304993
rs55767800
rs9826381
rs114609087
All
−0.08
−0.04
0.00
MR leave−one−out sensitivity analysis for
'CD127 on CD28+ CD45RA+ CD8br' on 'Hashimoto`s thyroiditis'

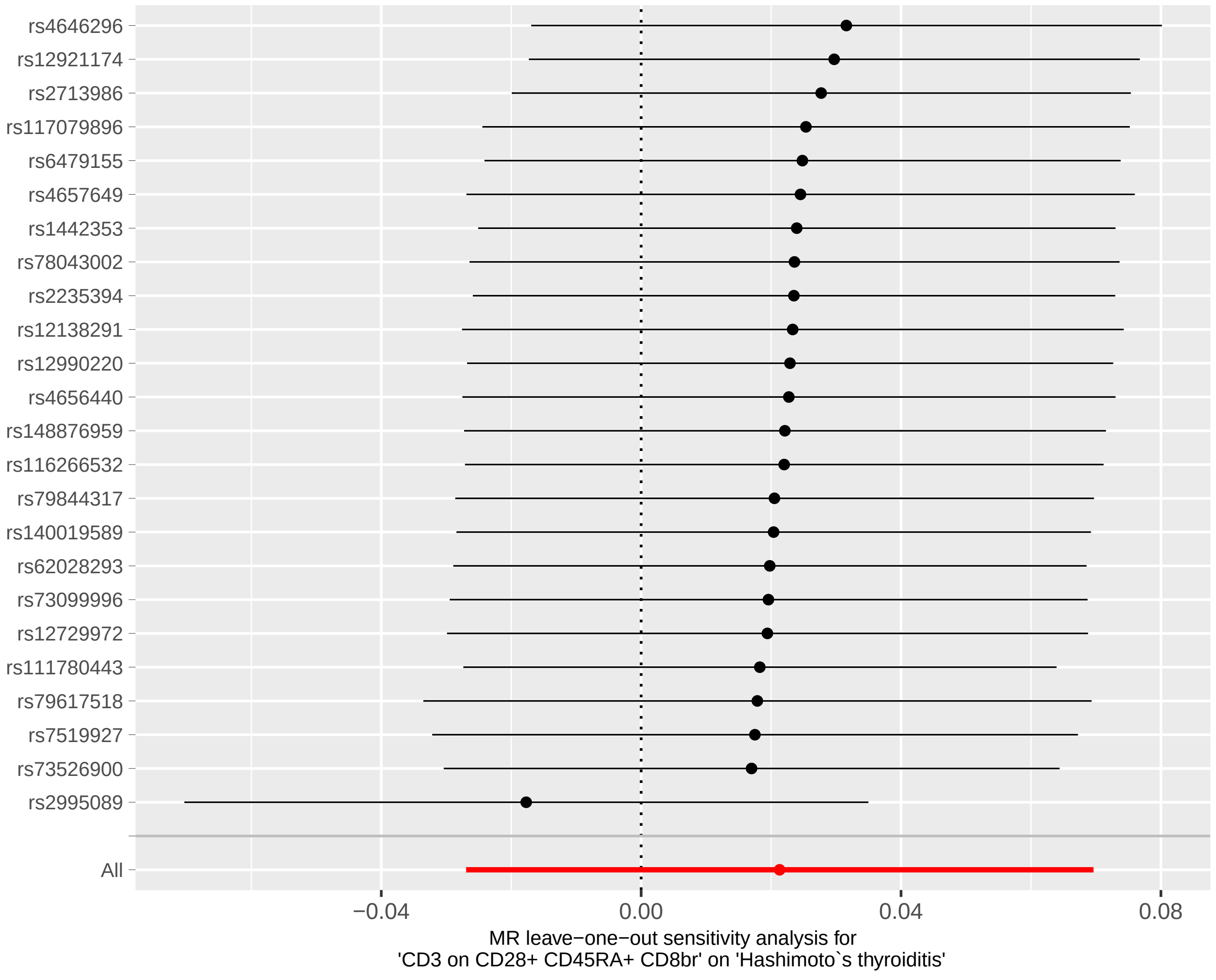
rs4646296
rs12921174
rs2713986
rs117079896
rs6479155
rs4657649
rs1442353
rs78043002
rs2235394
rs12138291
rs12990220
rs4656440
rs148876959
rs116266532
rs79844317
rs140019589
rs62028293
rs73099996
rs12729972
rs111780443
rs79617518
rs7519927
rs73526900
rs2995089
All
−0.04
0.00
0.04
0.08
MR leave−one−out sensitivity analysis for
'CD3 on CD28+ CD45RA+ CD8br' on 'Hashimoto`s thyroiditis'
