# Supplementary material for: Treg cells as a protective factor for Hashimoto`s thyroiditis: a Mendelian randomization study
Source: Front Endocrinol (Lausanne). 2024 Mar 8;15:1347695. doi: 10.3389/fendo.2024.1347695 (PMC10957564; doi:10.3389/fendo.2024.1347695)
Supplement: Supplementary file 2 [file Image_2.pdf]

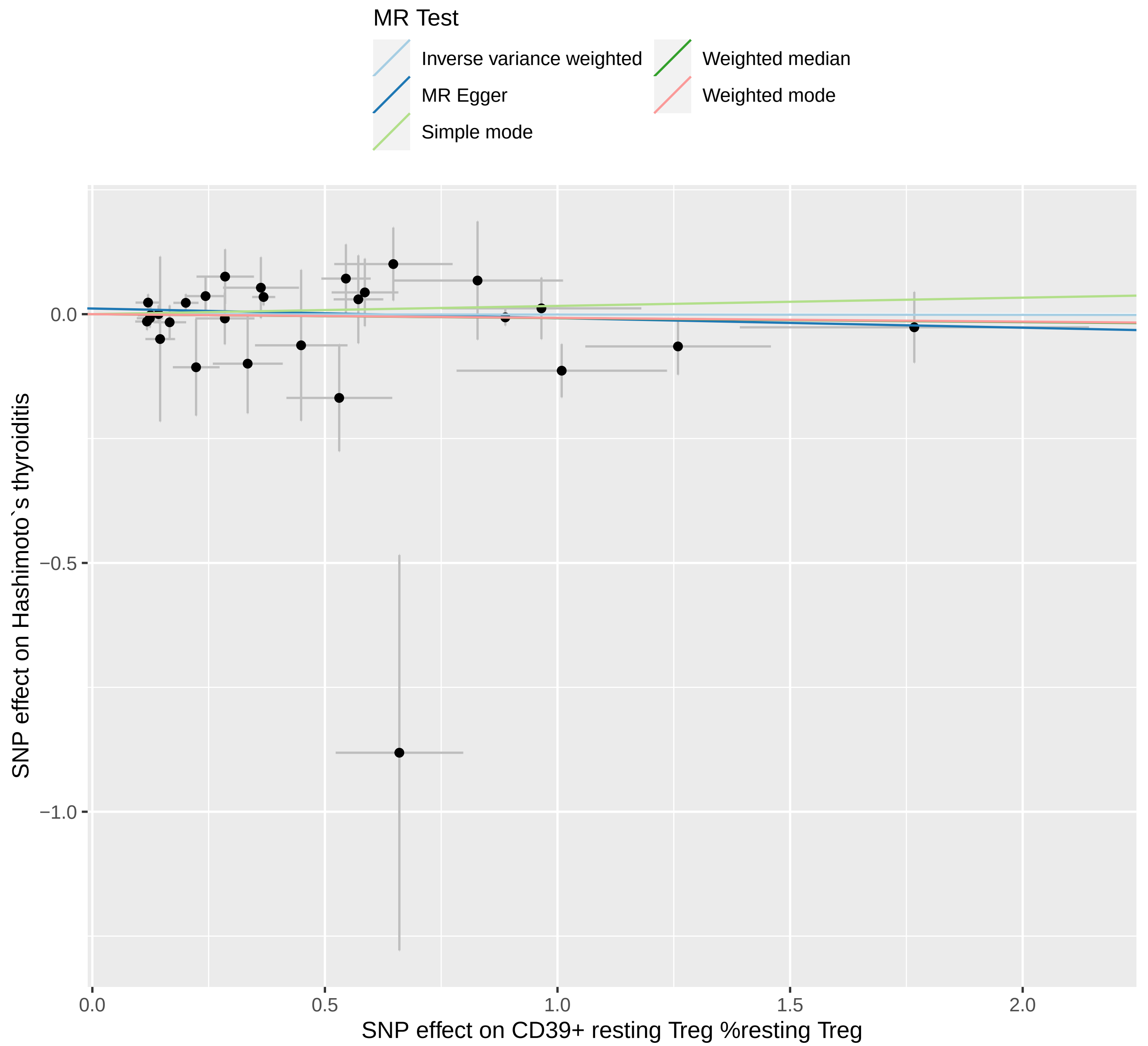
MR Test
Inverse variance weighted
MR Egger
Simple mode
Weighted median
Weighted mode
0.0
−0.5
−1.0
0.0
0.5
1.0
1.5
2.0
SNP effect on Hashimoto`s thyroiditis
SNP effect on CD39+ resting Treg %resting Treg

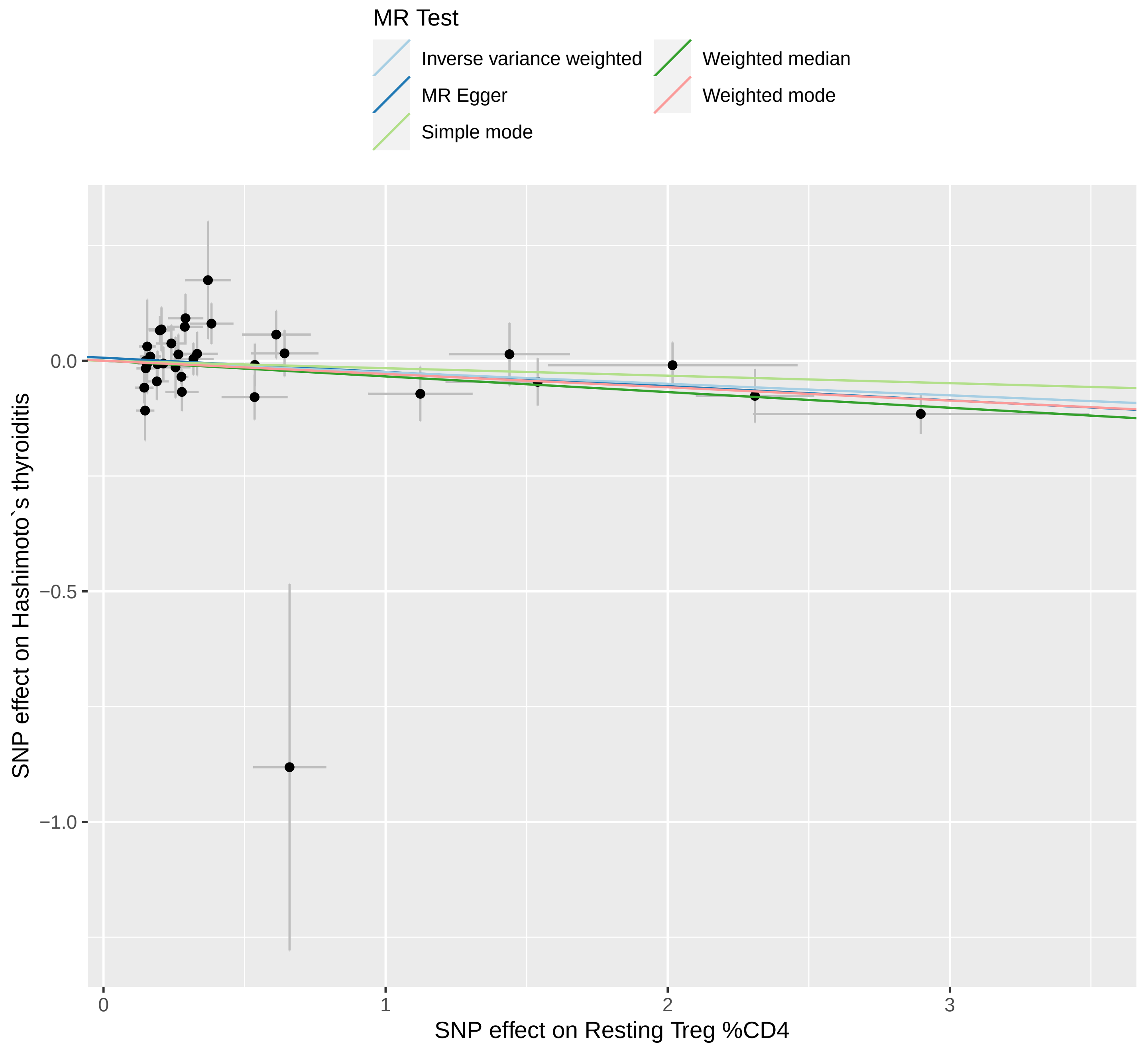

MR Test
Inverse variance weighted
MR Egger
Simple mode
Weighted median
Weighted mode
0.0
−0.5
−1.0
0
1
2
3
SNP effect on Hashimoto`s thyroiditis
SNP effect on Resting Treg %CD4

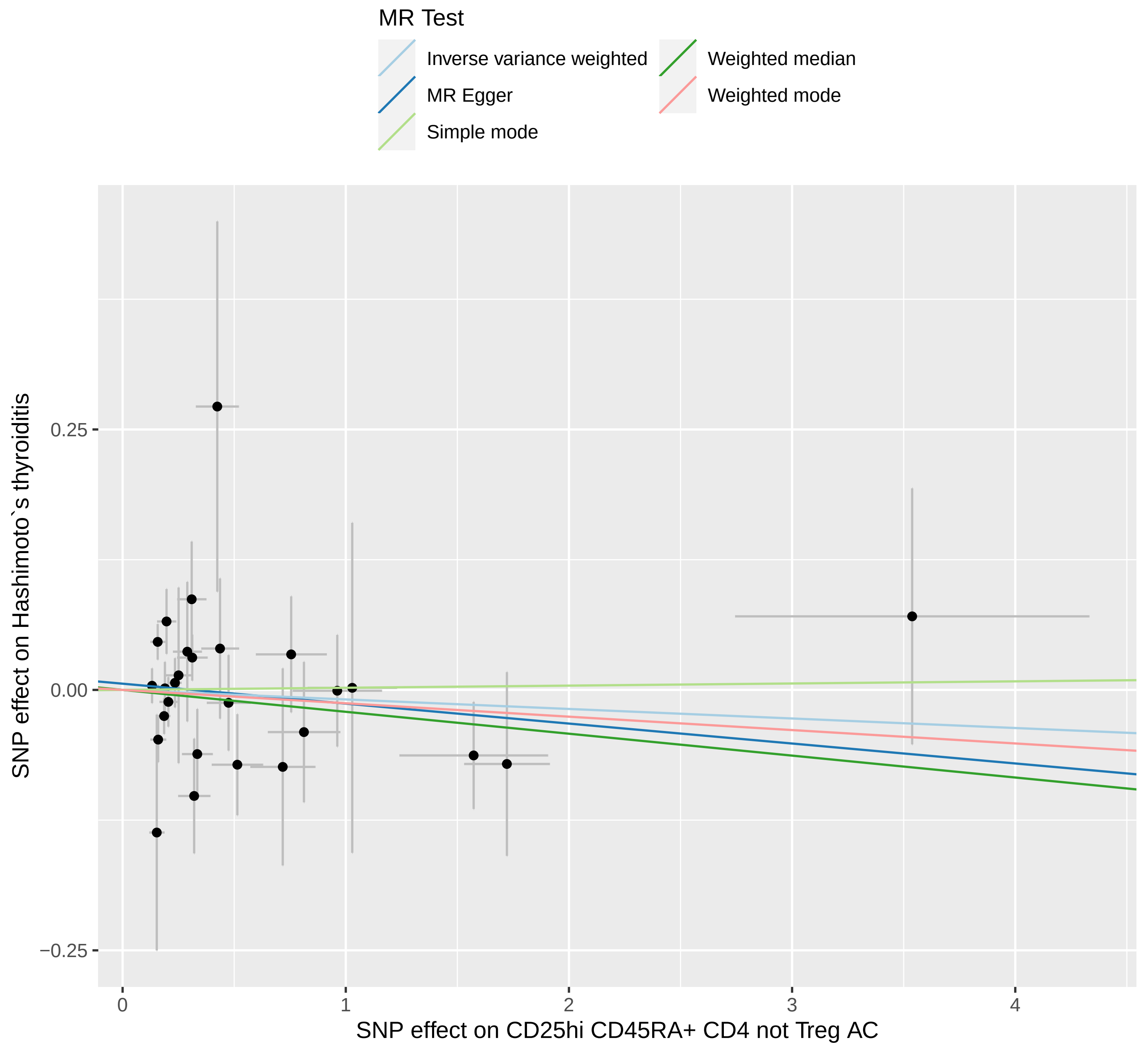

MR Test
Inverse variance weighted
MR Egger
Simple mode
Weighted median
Weighted mode
SNP effect on Hashimoto`s thyroiditis
0.25
0.00
−0.25
0
1
2
3
4
SNP effect on CD25hi CD45RA+ CD4 not Treg AC

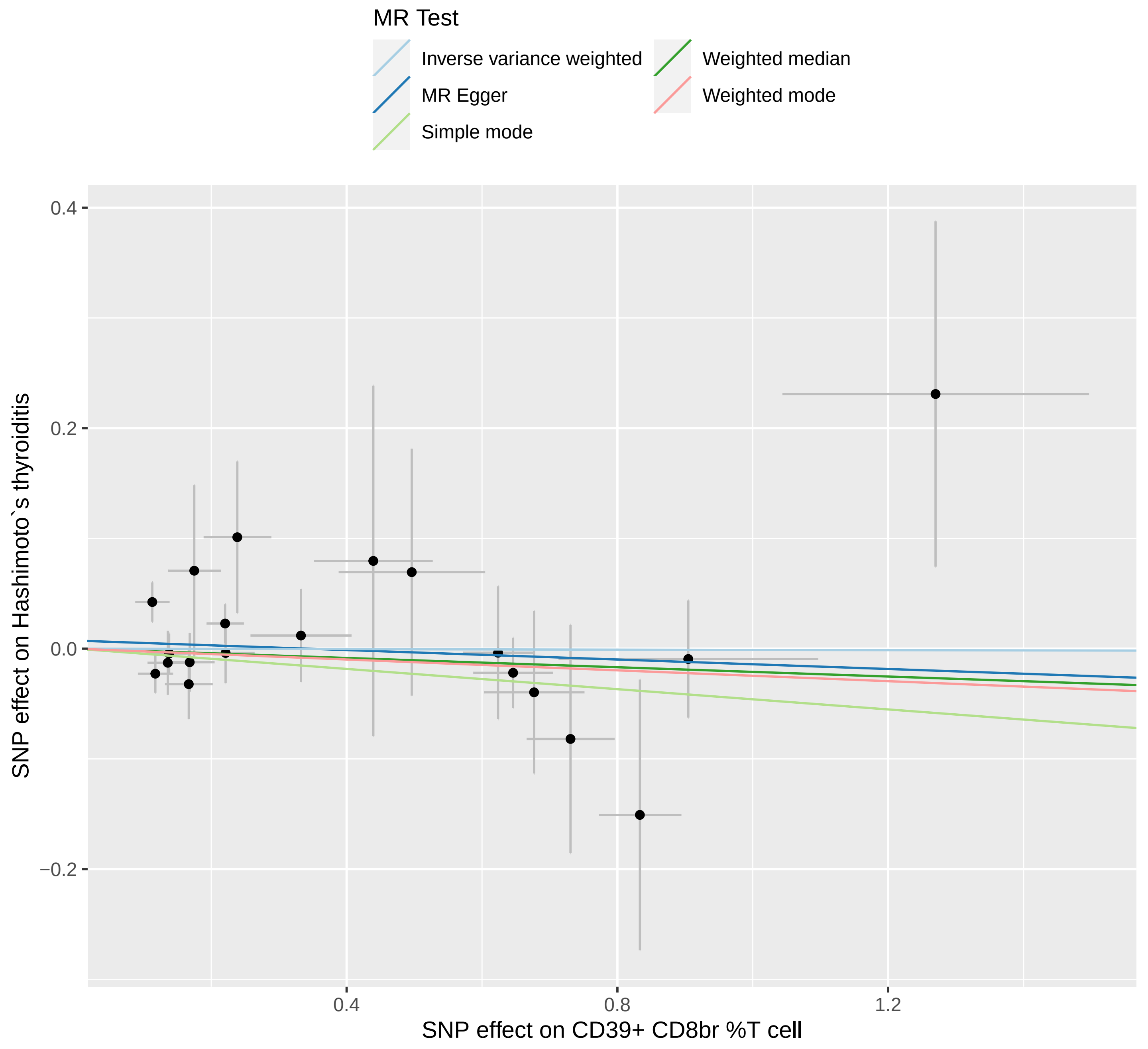

MR Test
Inverse variance weighted
MR Egger
Simple mode
Weighted median
Weighted mode
0.4
0.2
0.0
−0.2
SNP effect on Hashimoto`s thyroiditis
0.4
0.8
1.2
SNP effect on CD39+ CD8br %T cell

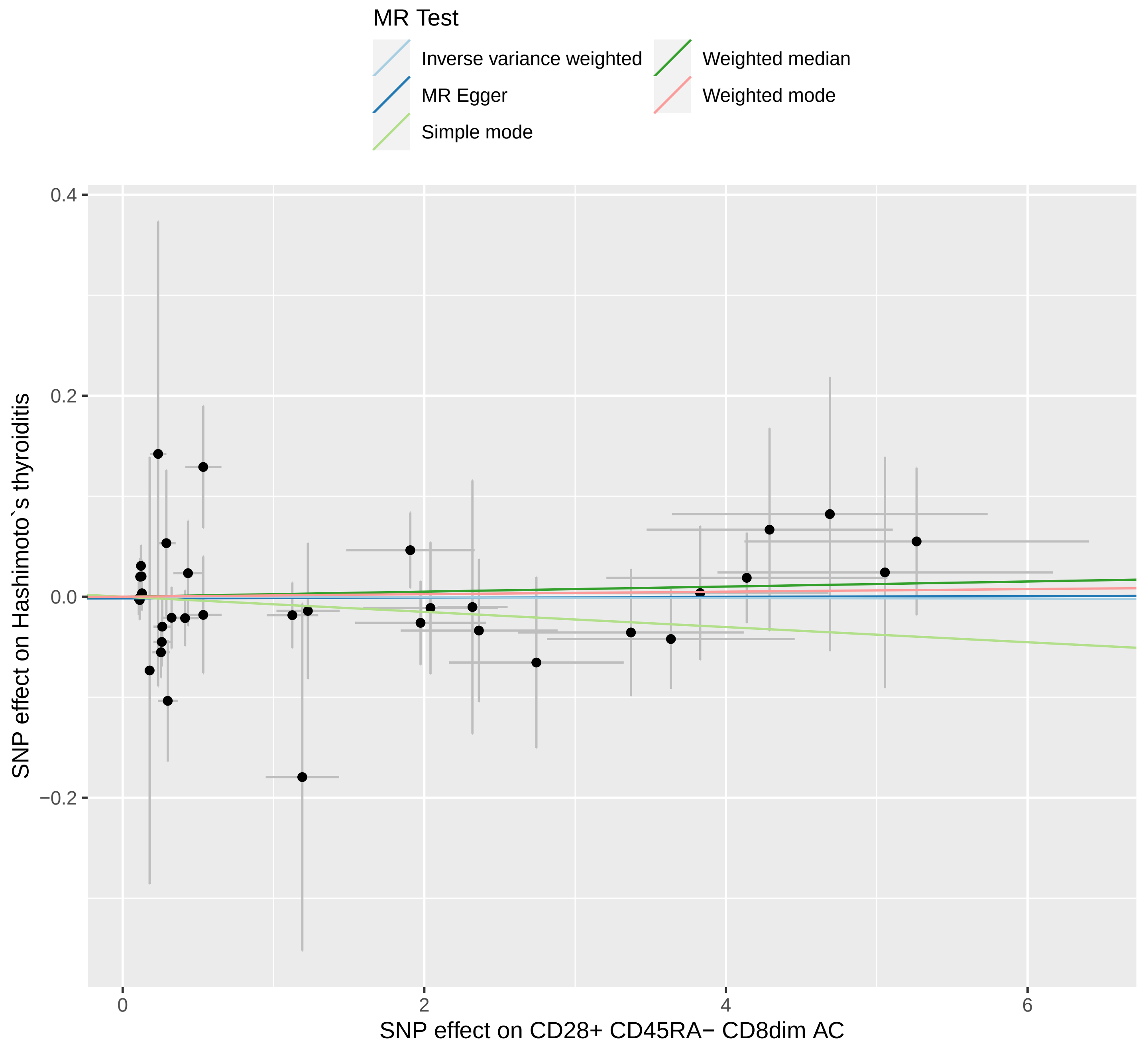
MR Test
Inverse variance weighted
MR Egger
Simple mode
Weighted median
Weighted mode
0.4
0.2
0.0
−0.2
0
2
4
6
SNP effect on Hashimoto`s thyroiditis
SNP effect on CD28+ CD45RA− CD8dim AC

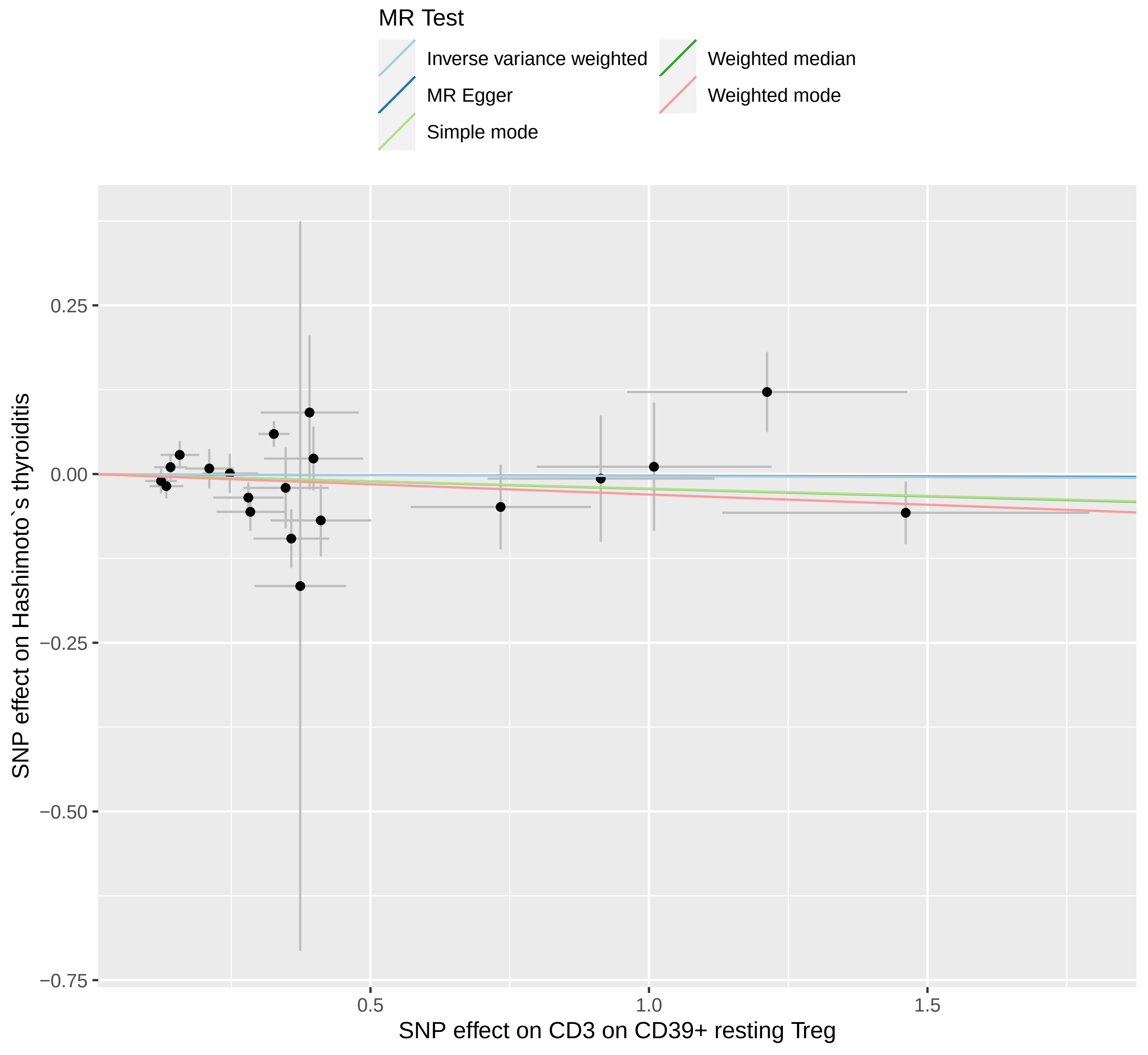

MR Test
Inverse variance weighted
MR Egger
Simple mode
Weighted median
Weighted mode
0.25
0.00
−0.25
−0.50
−0.75
SNP effect on Hashimoto`s thyroiditis
0.5
1.0
1.5
SNP effect on CD3 on CD39+ resting Treg

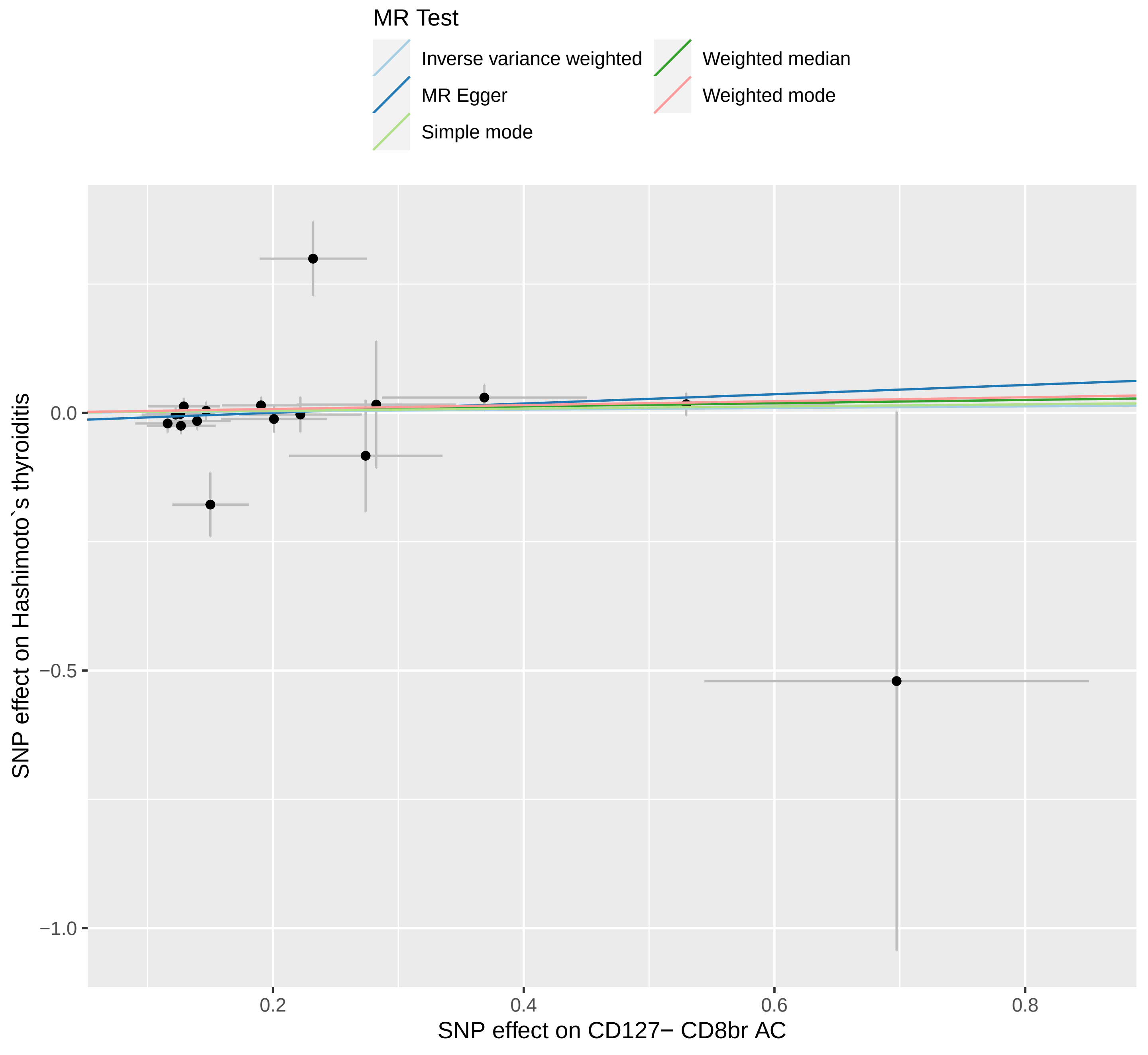
MR Test
Inverse variance weighted
MR Egger
Simple mode
Weighted median
Weighted mode
SNP effect on Hashimoto`s thyroiditis
0.0
−0.5
−1.0
0.2
0.4
0.6
0.8
SNP effect on CD127− CD8br AC

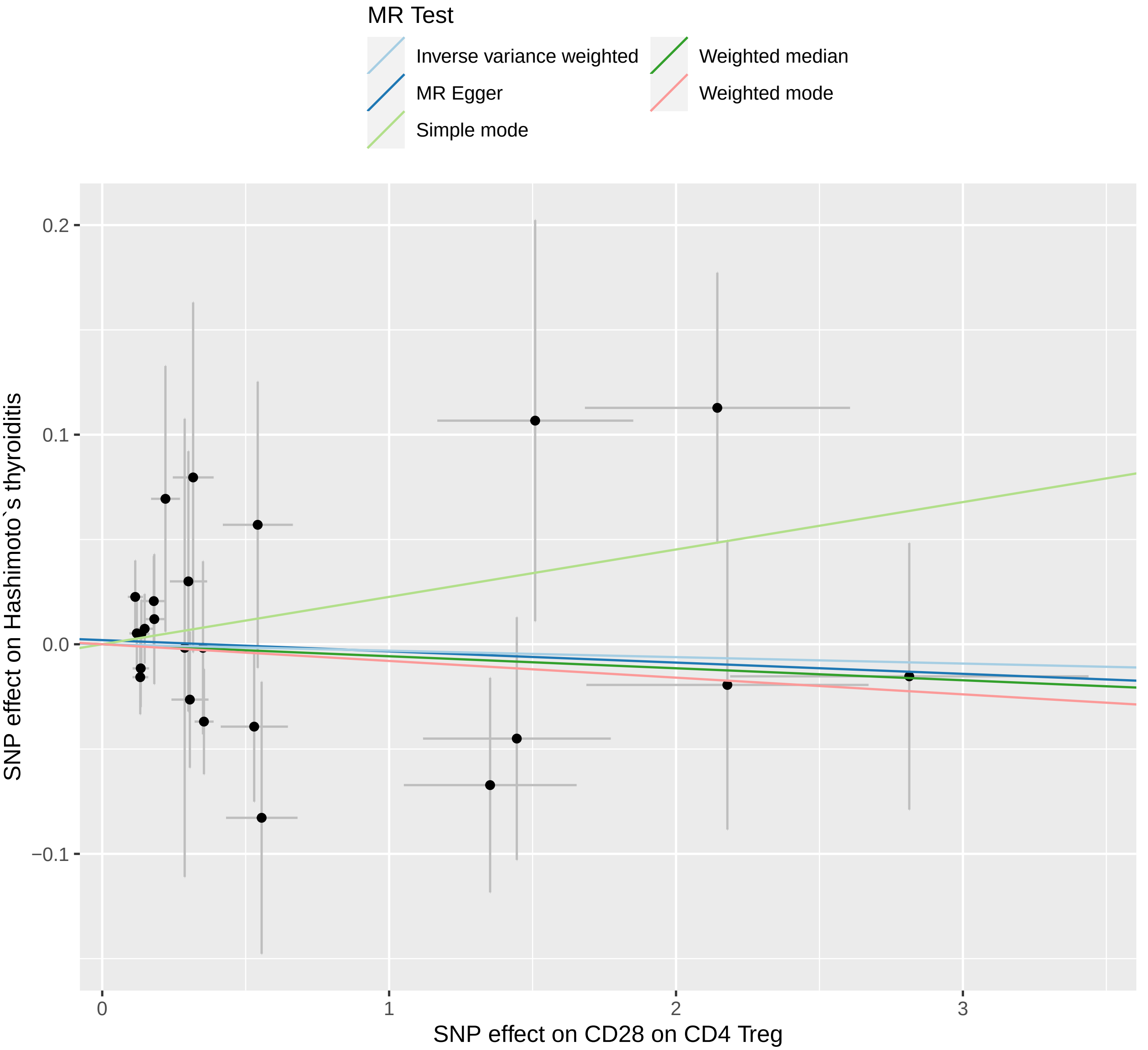

MR Test
Inverse variance weighted
MR Egger
Simple mode
Weighted median
Weighted mode
0.2
0.1
0.0
−0.1
0
1
2
3
SNP effect on Hashimoto`s thyroiditis
SNP effect on CD28 on CD4 Treg

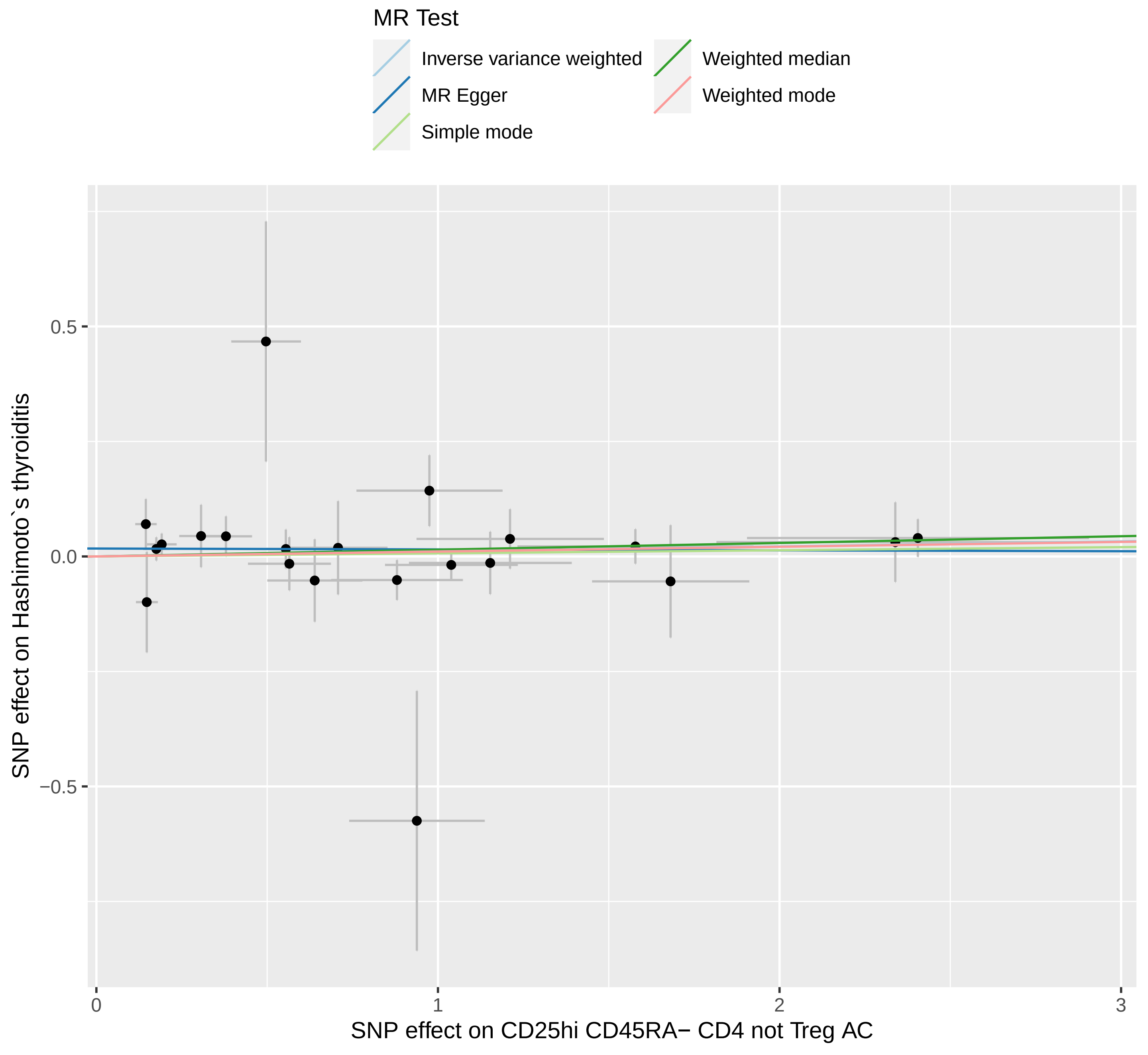

MR Test
Inverse variance weighted
MR Egger
Simple mode
Weighted median
Weighted mode
SNP effect on Hashimoto`s thyroiditis
SNP effect on CD25hi CD45RA− CD4 not Treg AC
0.5
0.0
−0.5
0
1
2
3

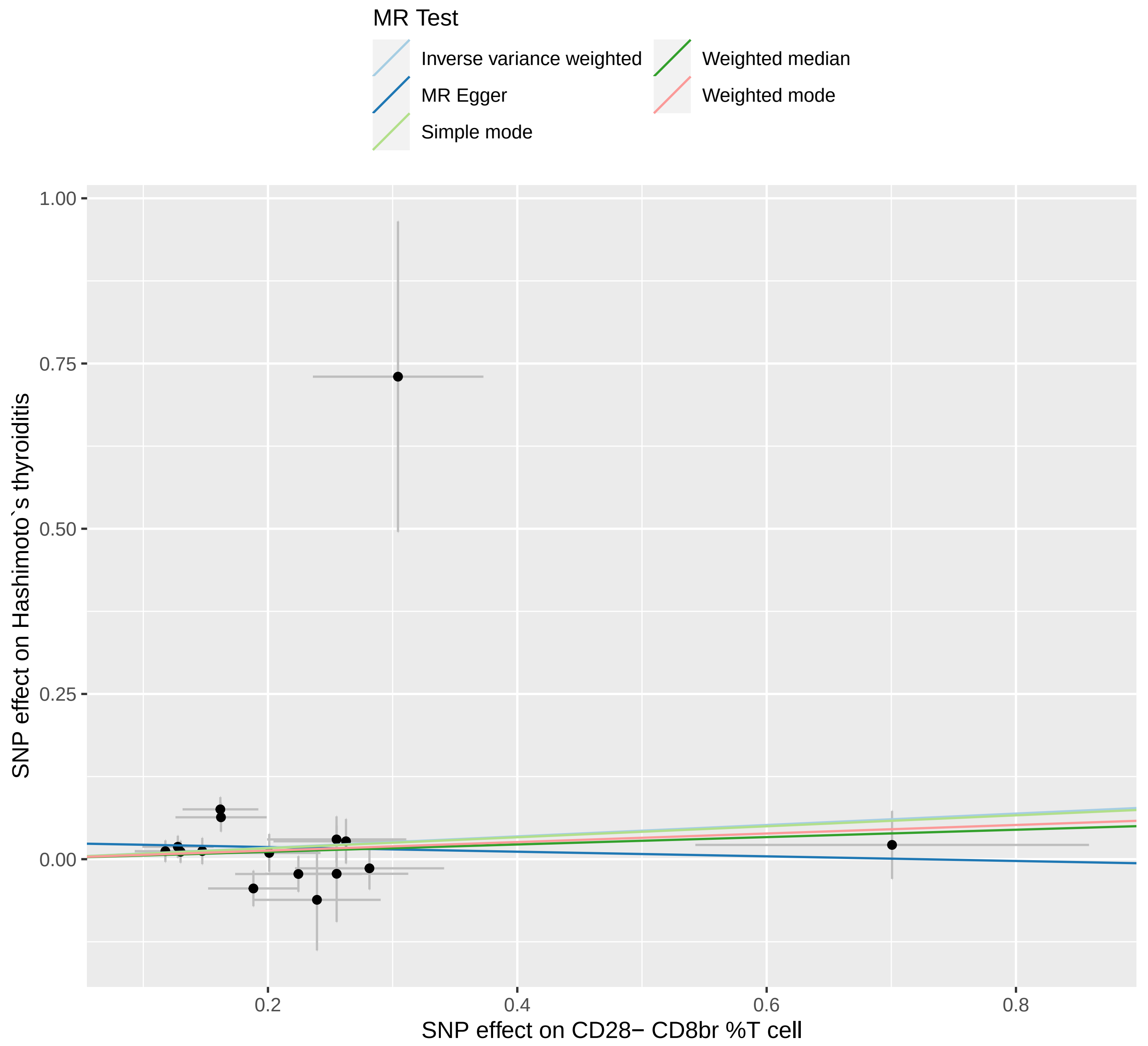

MR Test
Inverse variance weighted
MR Egger
Simple mode
Weighted median
Weighted mode
SNP effect on Hashimoto`s thyroiditis
1.00
0.75
0.50
0.25
0.00
0.2
0.4
0.6
0.8
SNP effect on CD28− CD8br %T cell

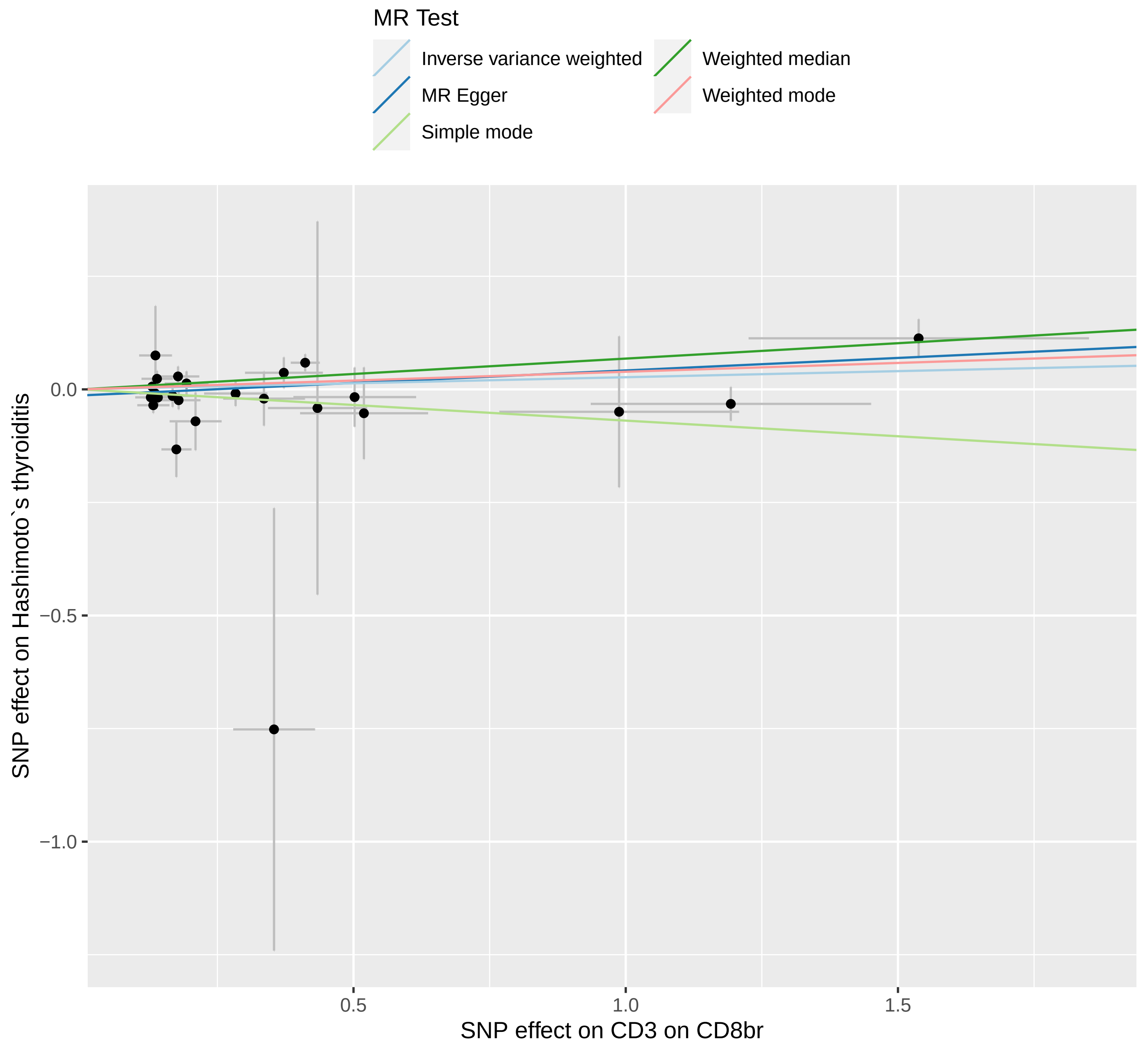
MR Test
Inverse variance weighted
MR Egger
Simple mode
Weighted median
Weighted mode
SNP effect on Hashimoto`s thyroiditis
0.0
−0.5
−1.0
0.5
1.0
1.5
SNP effect on CD3 on CD8br

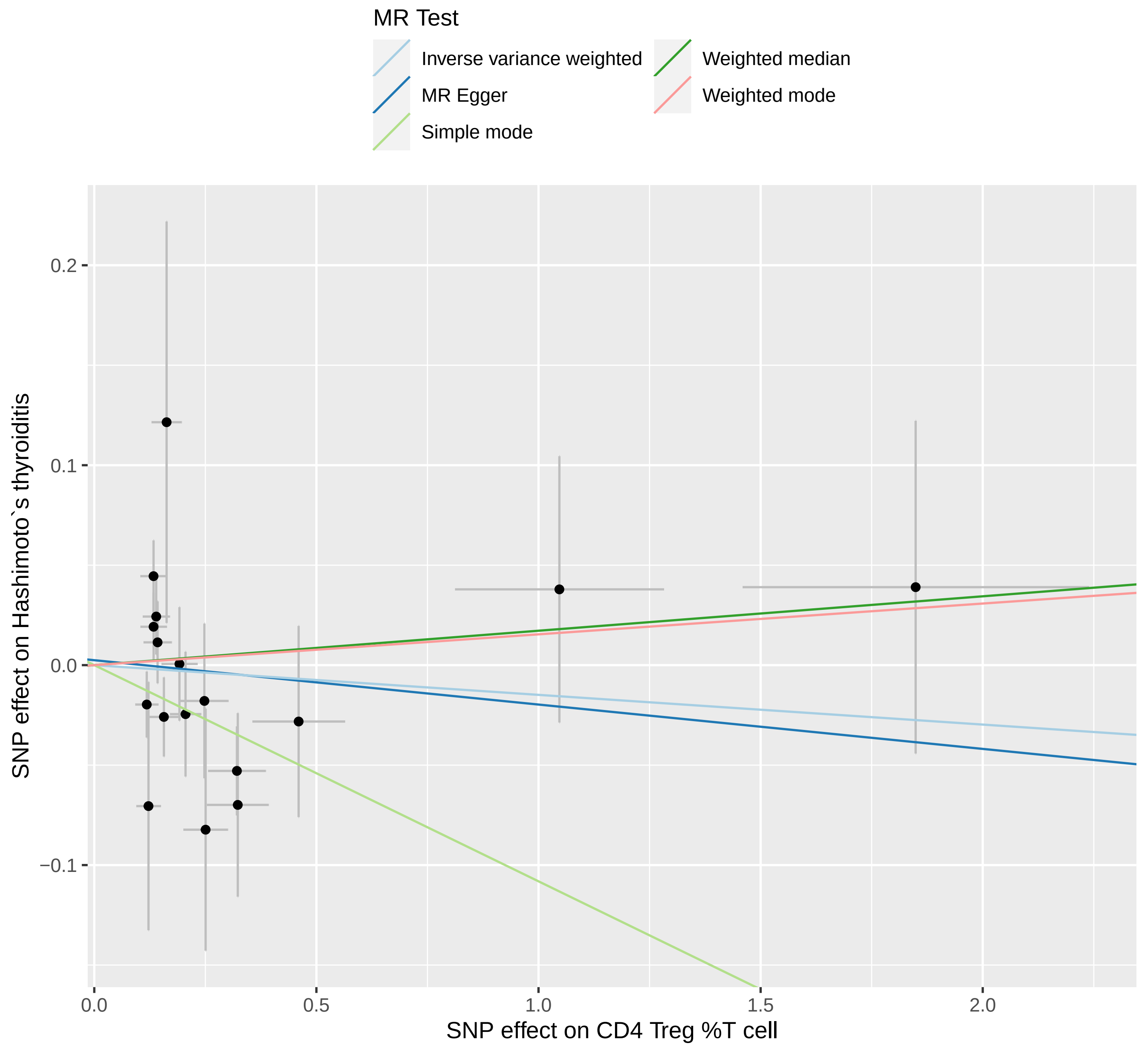
MR Test
Inverse variance weighted
MR Egger
Simple mode
Weighted median
Weighted mode
0.2
0.1
0.0
−0.1
0.0
0.5
1.0
1.5
2.0
SNP effect on Hashimoto`s thyroiditis
SNP effect on CD4 Treg %T cell

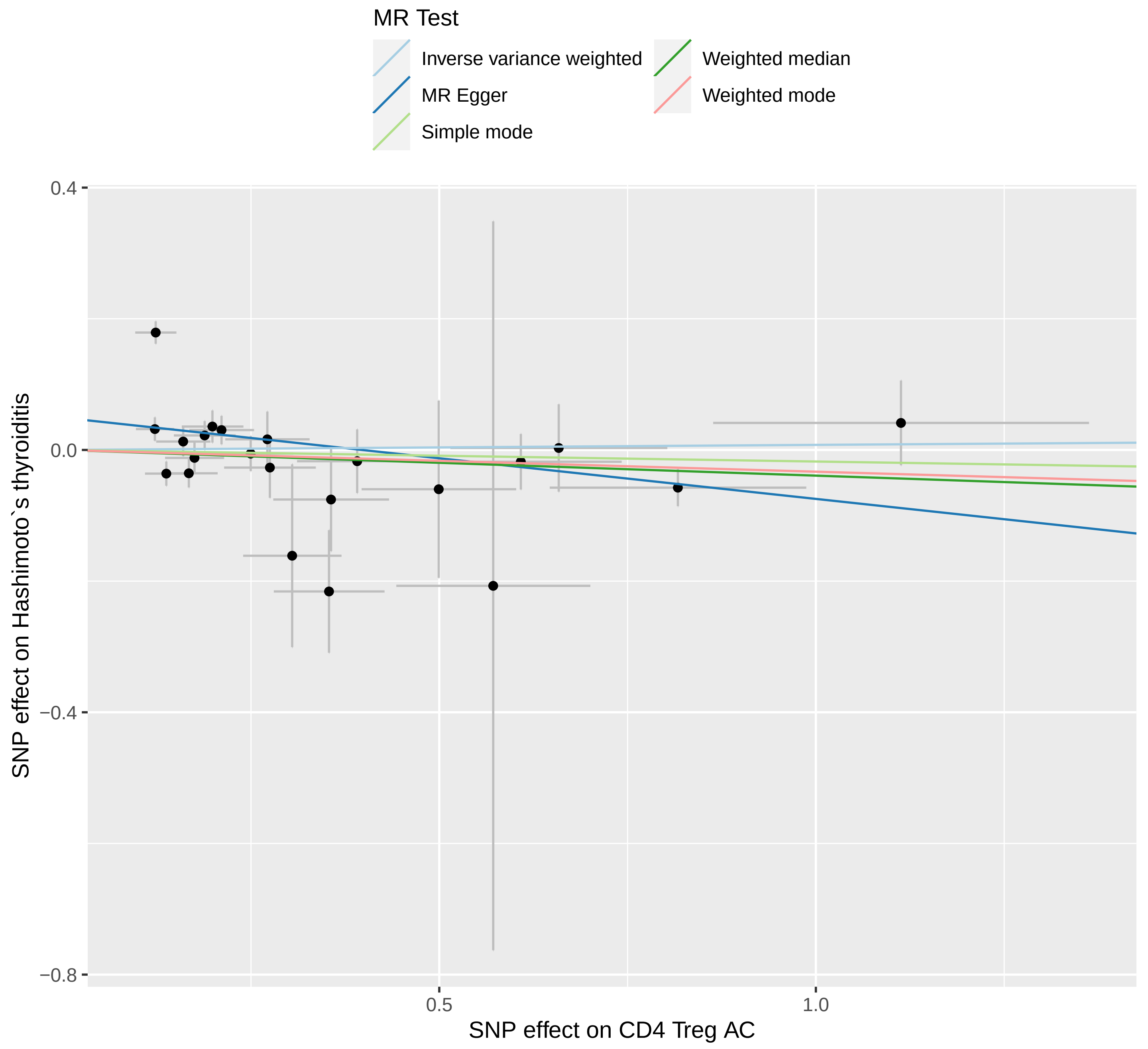
MR Test
Inverse variance weighted
MR Egger
Simple mode
Weighted median
Weighted mode
0.4
0.0
−0.4
−0.8
0.5
1.0
SNP effect on CD4 Treg AC
SNP effect on Hashimoto`s thyroiditis

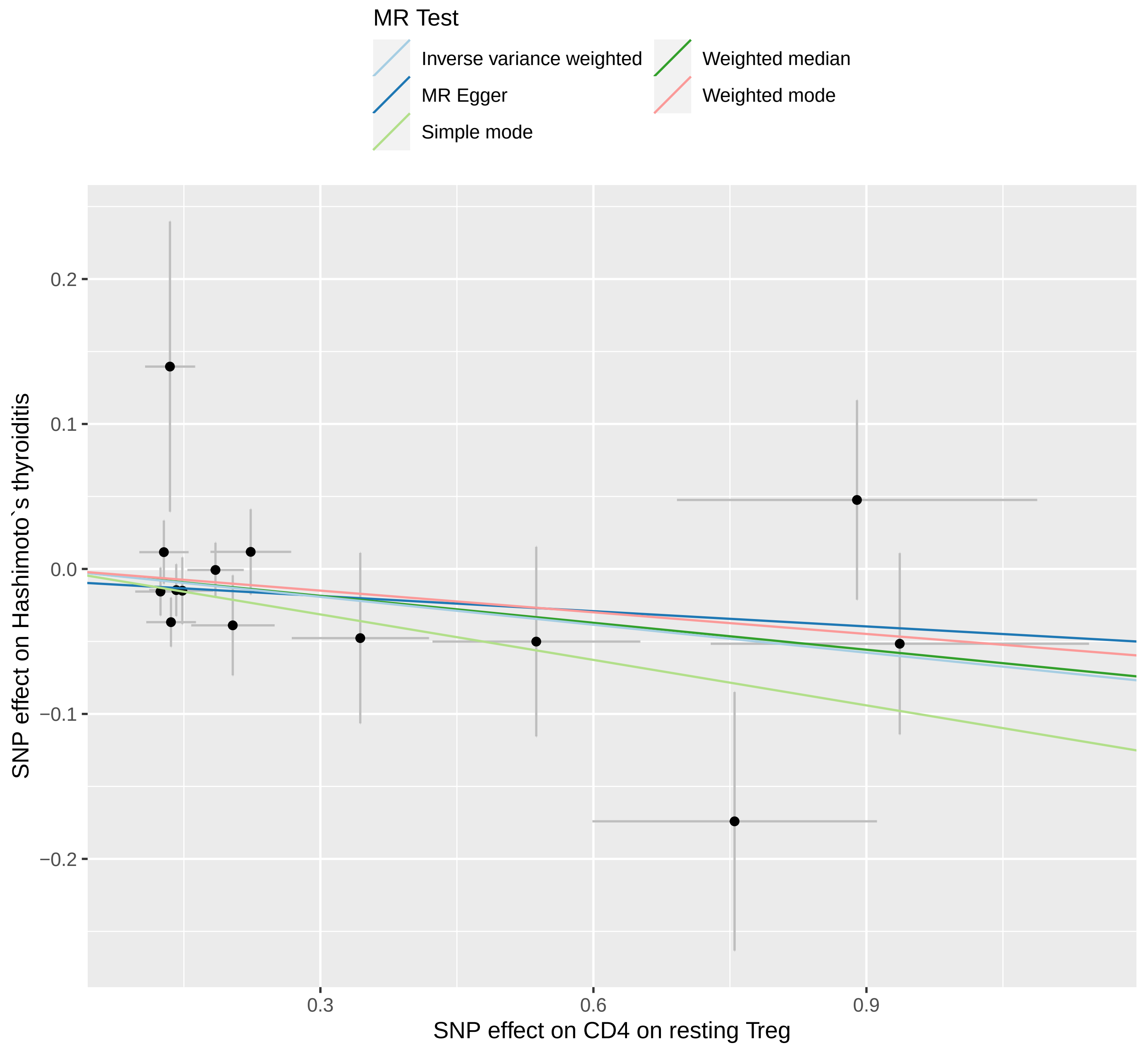

MR Test
Inverse variance weighted
MR Egger
Simple mode
Weighted median
Weighted mode
0.2
0.1
0.0
−0.1
−0.2
0.3
0.6
0.9
SNP effect on Hashimoto`s thyroiditis
SNP effect on CD4 on resting Treg

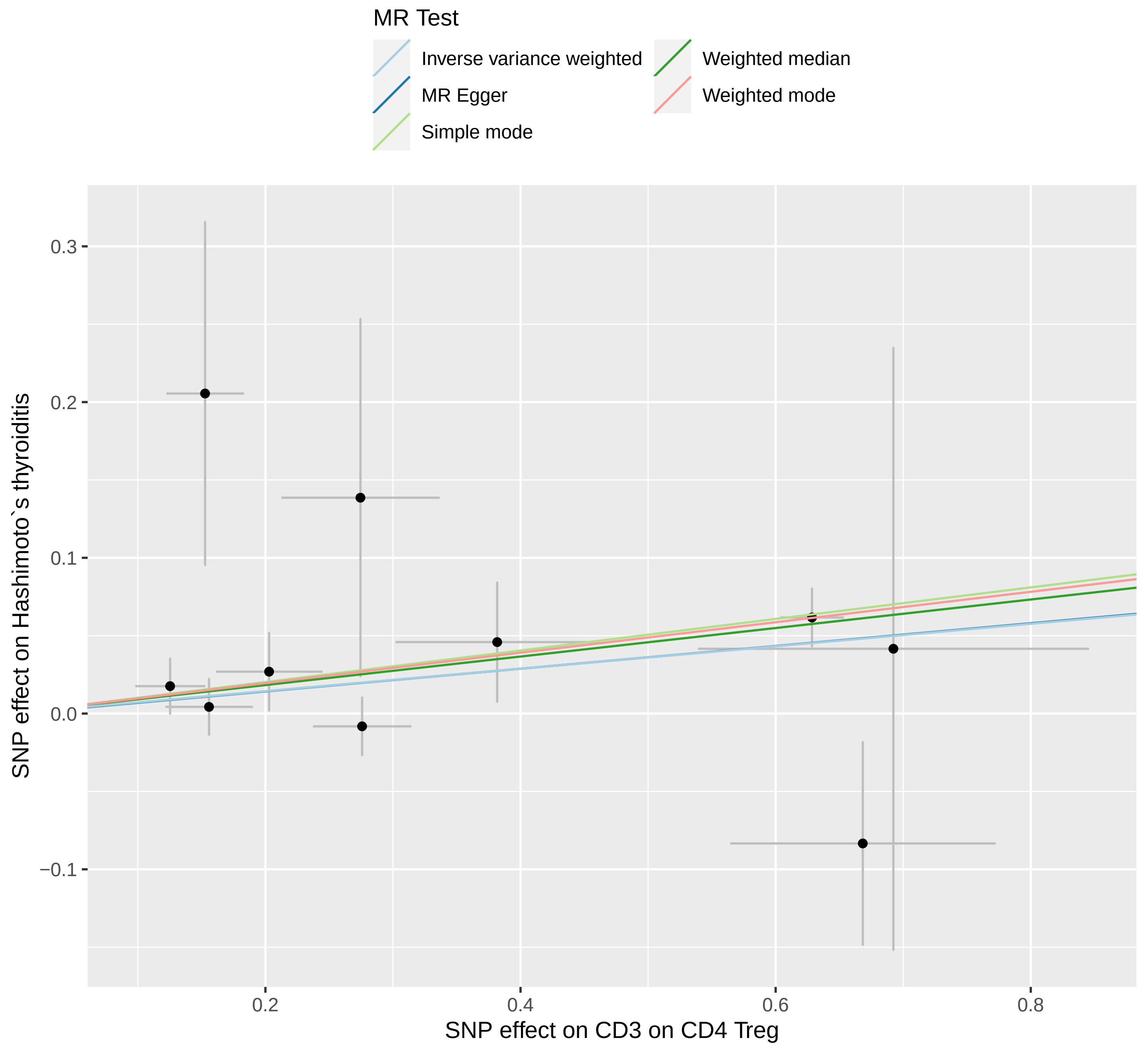
MR Test
Inverse variance weighted
MR Egger
Simple mode
Weighted median
Weighted mode
0.3
0.2
0.1
0.0
−0.1
SNP effect on Hashimoto`s thyroiditis
0.2
0.4
0.6
0.8
SNP effect on CD3 on CD4 Treg

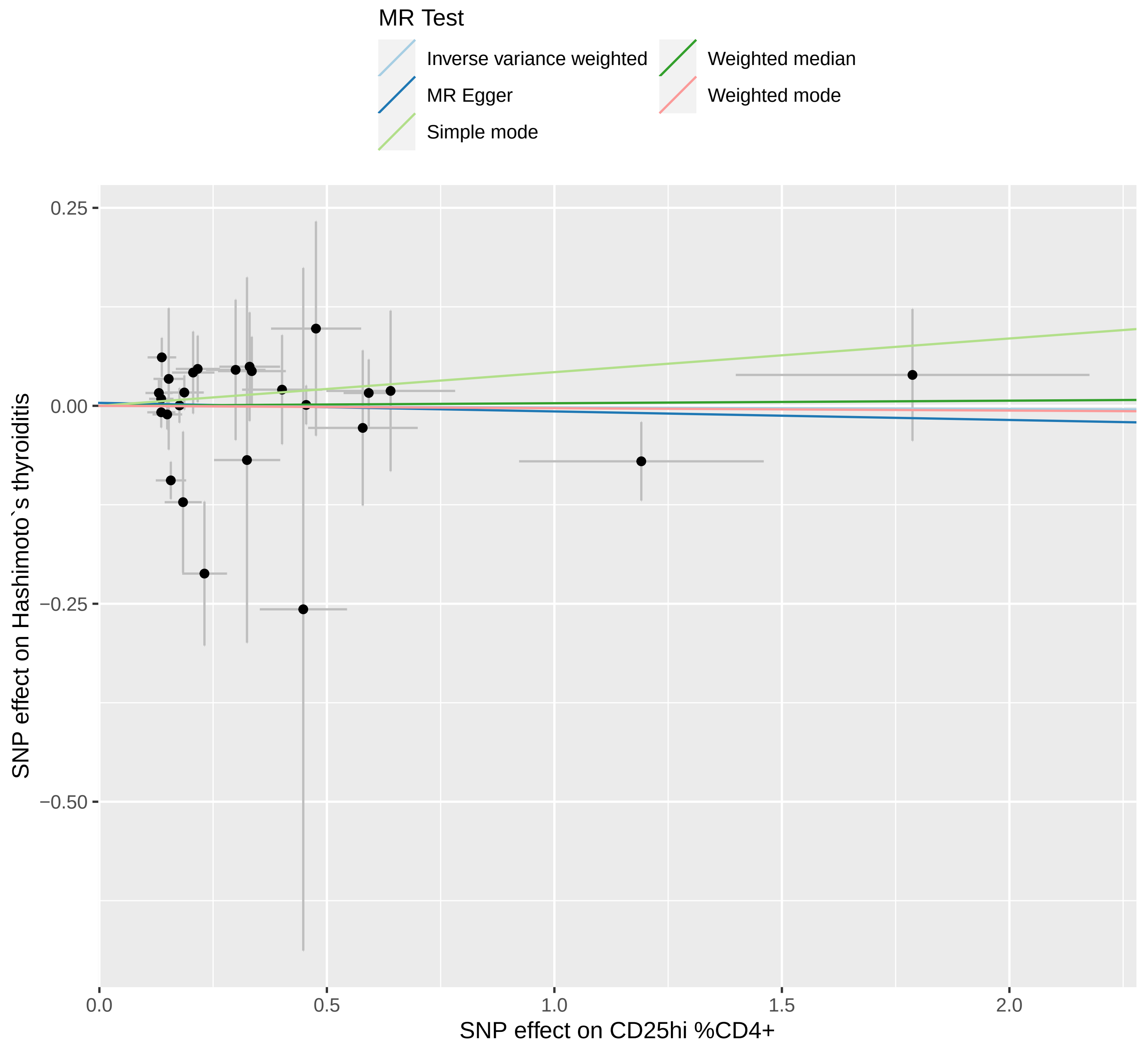
MR Test
Inverse variance weighted
MR Egger
Simple mode
Weighted median
Weighted mode
0.25
0.00
−0.25
−0.50
0.0
0.5
1.0
1.5
2.0
SNP effect on CD25hi %CD4+
SNP effect on Hashimoto`s thyroiditis

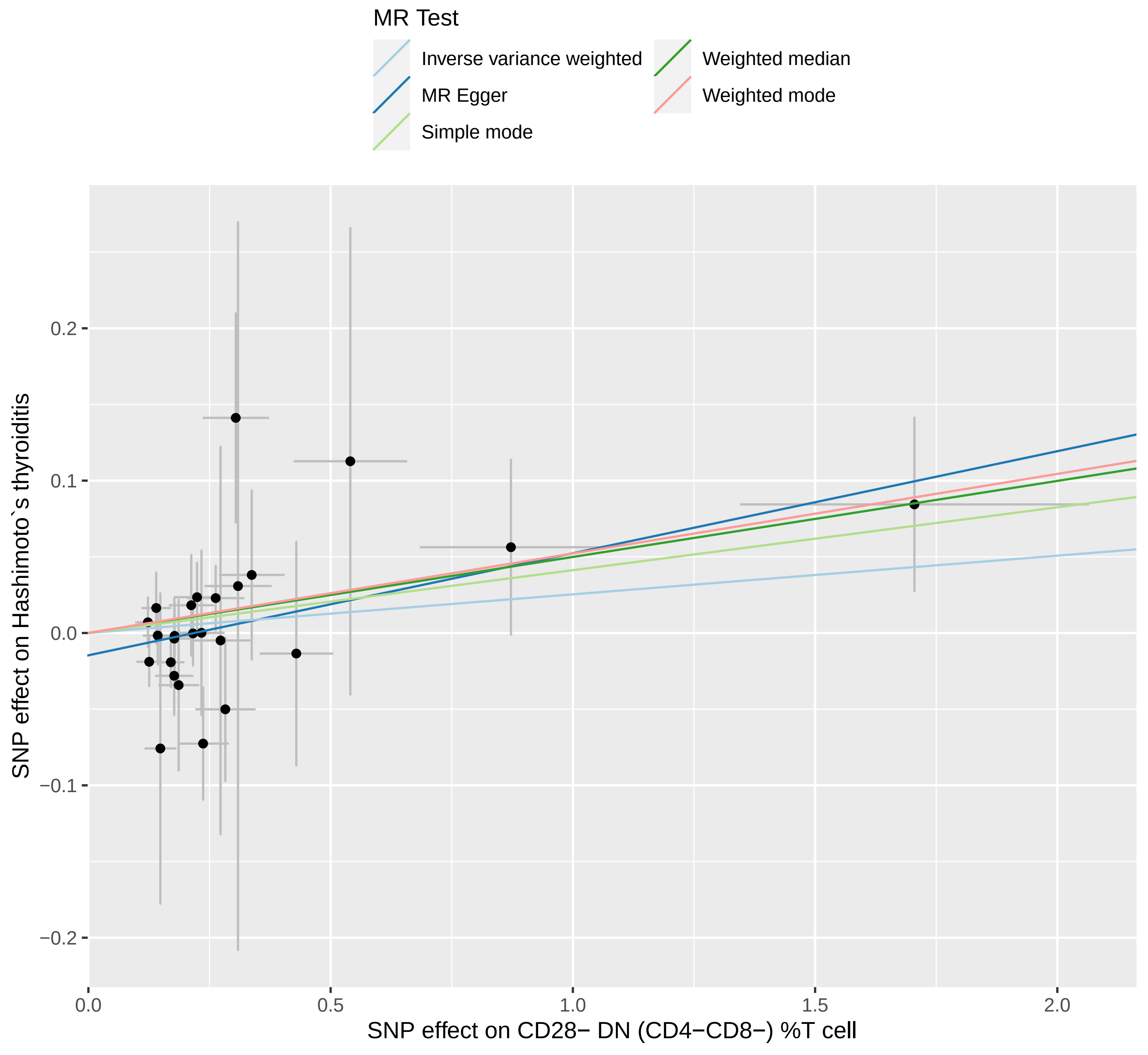
MR Test
Inverse variance weighted
MR Egger
Simple mode
Weighted median
Weighted mode
SNP effect on Hashimoto`s thyroiditis
0.2
0.1
0.0
−0.1
−0.2
0.0
0.5
1.0
1.5
2.0
SNP effect on CD28− DN (CD4−CD8−) %T cell

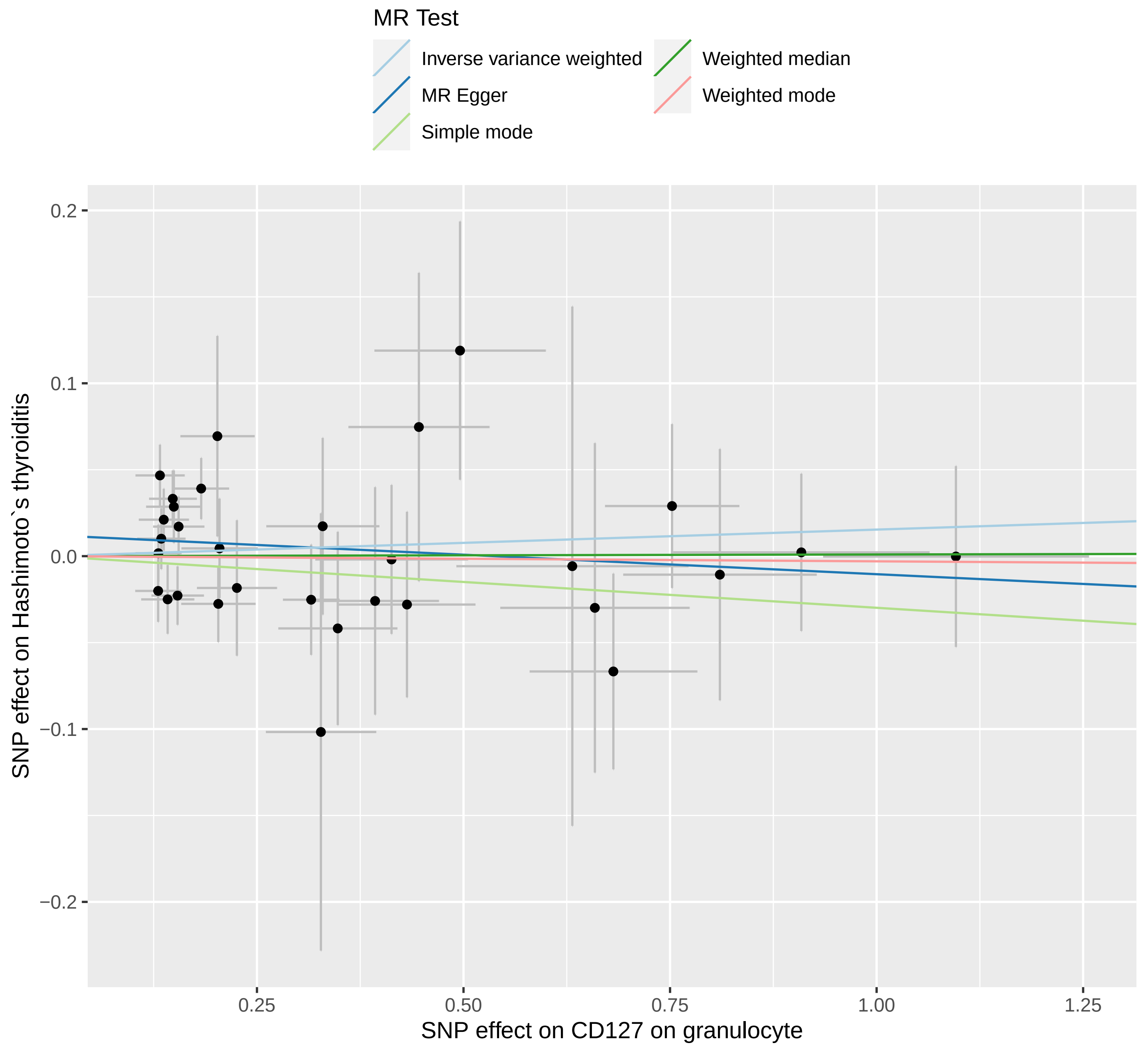
MR Test
Inverse variance weighted
MR Egger
Simple mode
Weighted median
Weighted mode
0.2
0.1
0.0
−0.1
−0.2
0.25
0.50
0.75
1.00
1.25
SNP effect on Hashimoto`s thyroiditis
SNP effect on CD127 on granulocyte

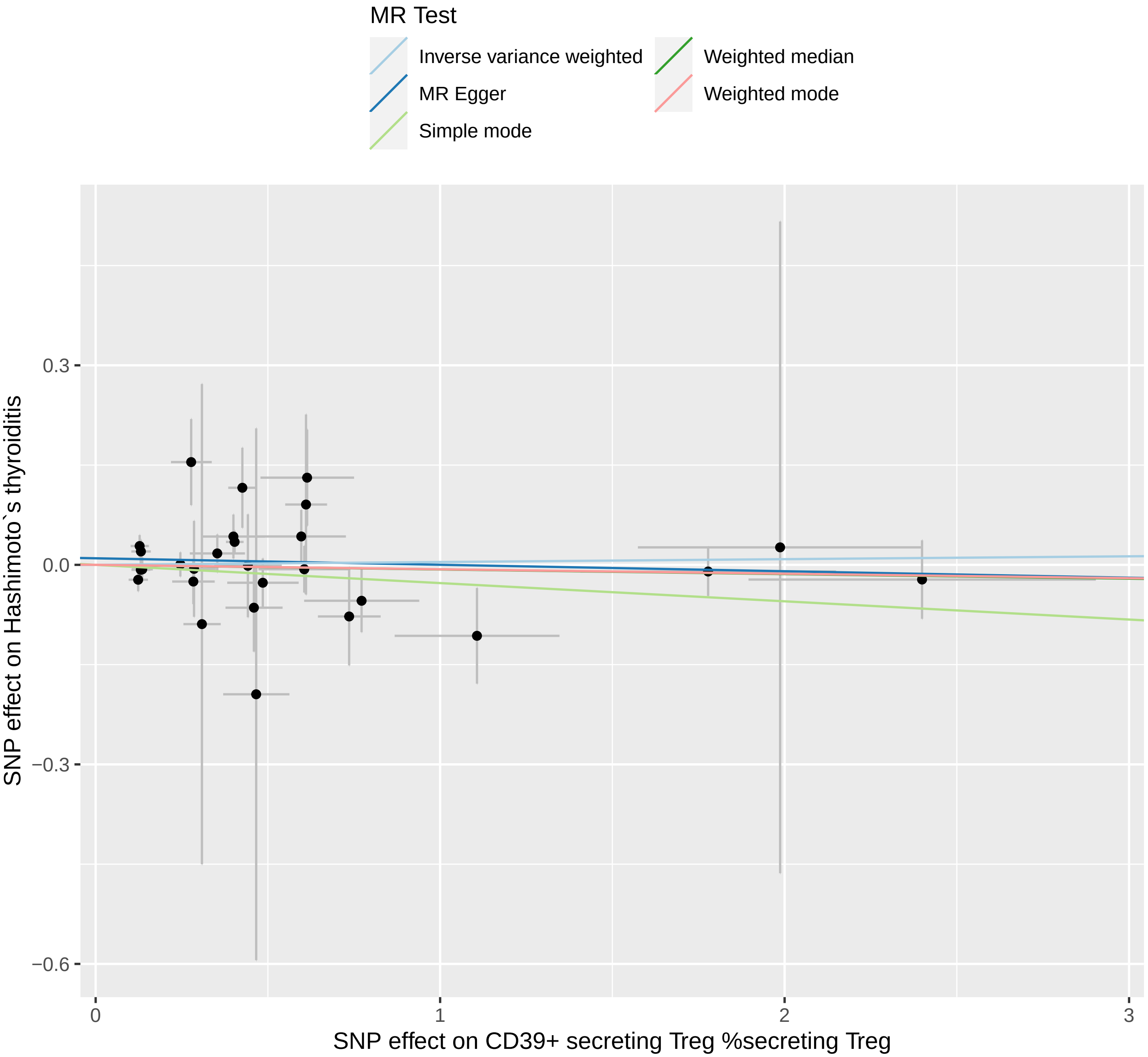

MR Test
Inverse variance weighted
MR Egger
Simple mode
Weighted median
Weighted mode
0.3
0.0
−0.3
−0.6
0
1
2
3
SNP effect on Hashimoto`s thyroiditis
SNP effect on CD39+ secreting Treg %secreting Treg

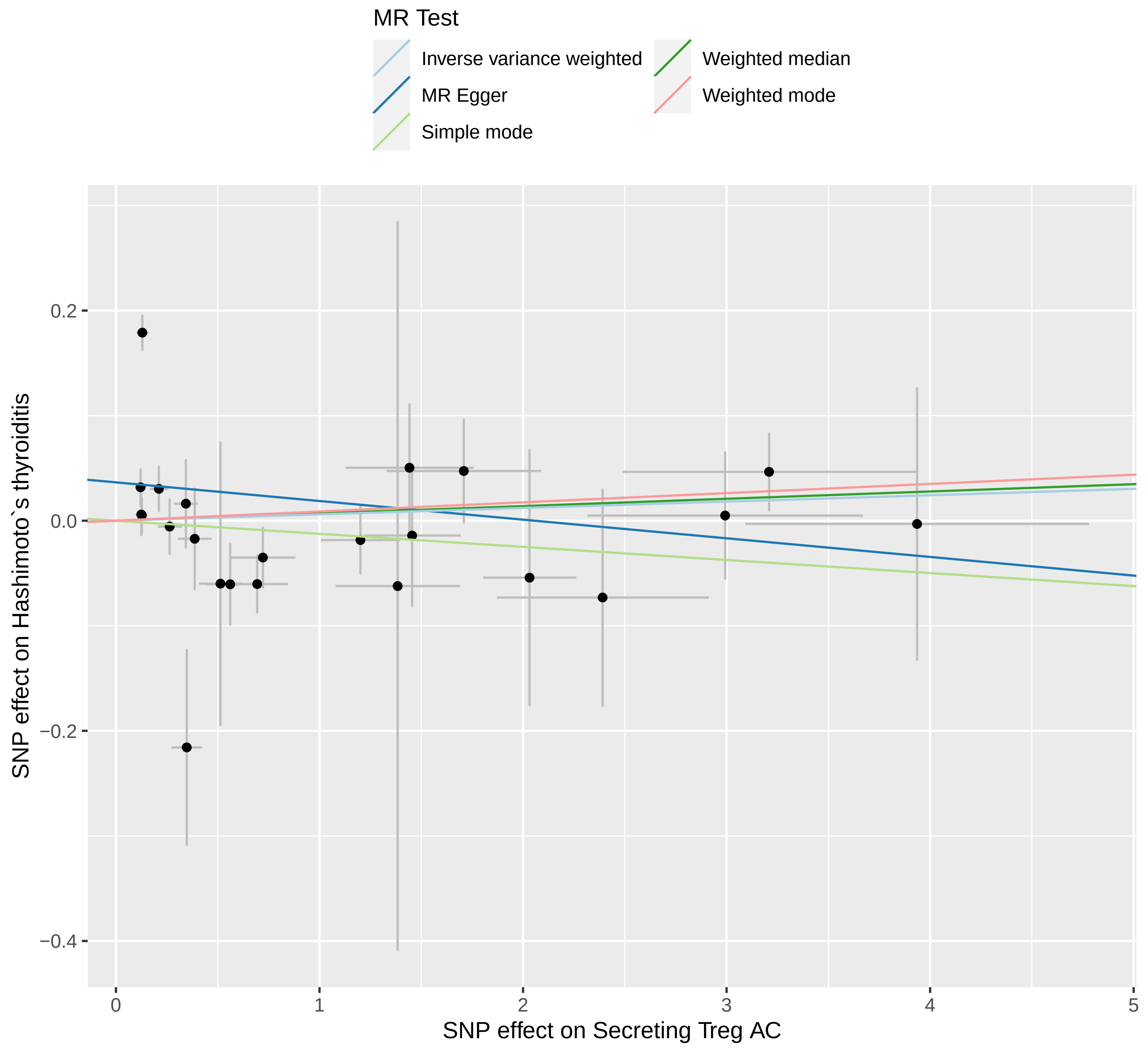

MR Test
Inverse variance weighted
MR Egger
Simple mode
Weighted median
Weighted mode
0.2
0.0
−0.2
−0.4
SNP effect on Hashimoto`s thyroiditis
0
1
2
3
4
5
SNP effect on Secreting Treg AC

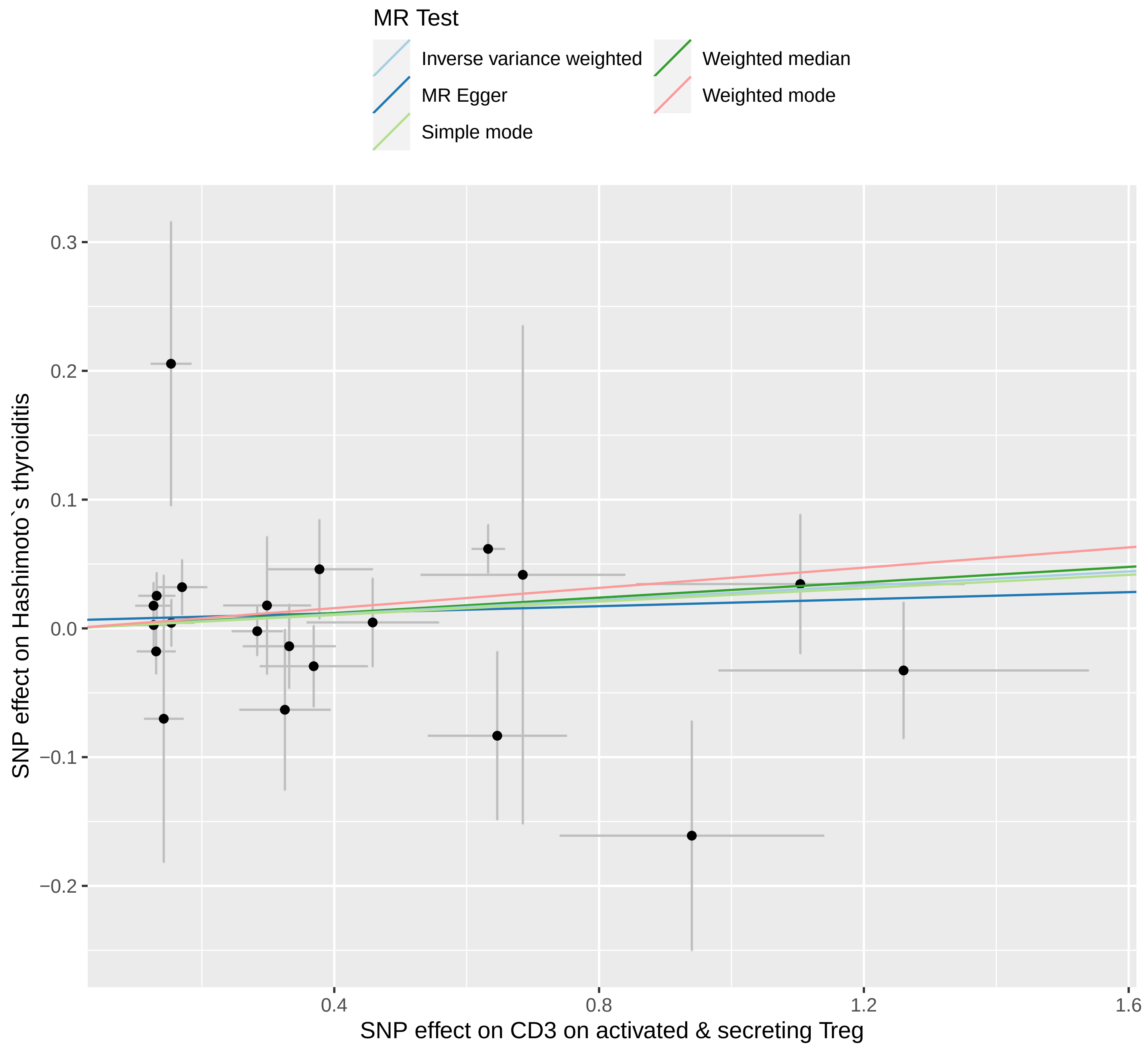
MR Test
Inverse variance weighted
MR Egger
Simple mode
Weighted median
Weighted mode
SNP effect on Hashimoto`s thyroiditis
SNP effect on CD3 on activated & secreting Treg
0.3
0.2
0.1
0.0
−0.1
−0.2
0.4
0.8
1.2
1.6

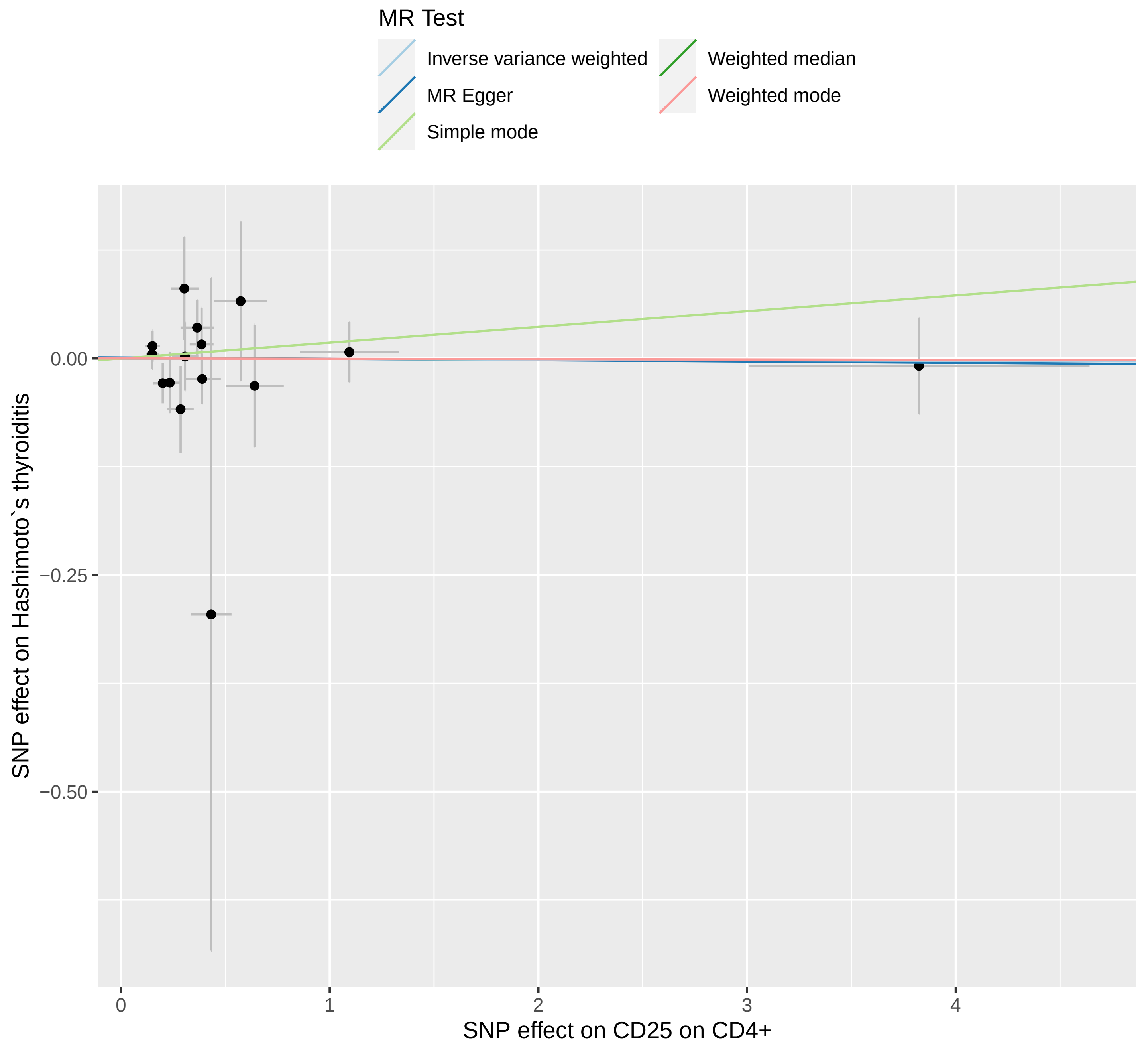
MR Test
Inverse variance weighted
MR Egger
Simple mode
Weighted median
Weighted mode
SNP effect on Hashimoto`s thyroiditis
0.00
−0.25
−0.50
0
1
2
3
4
SNP effect on CD25 on CD4+

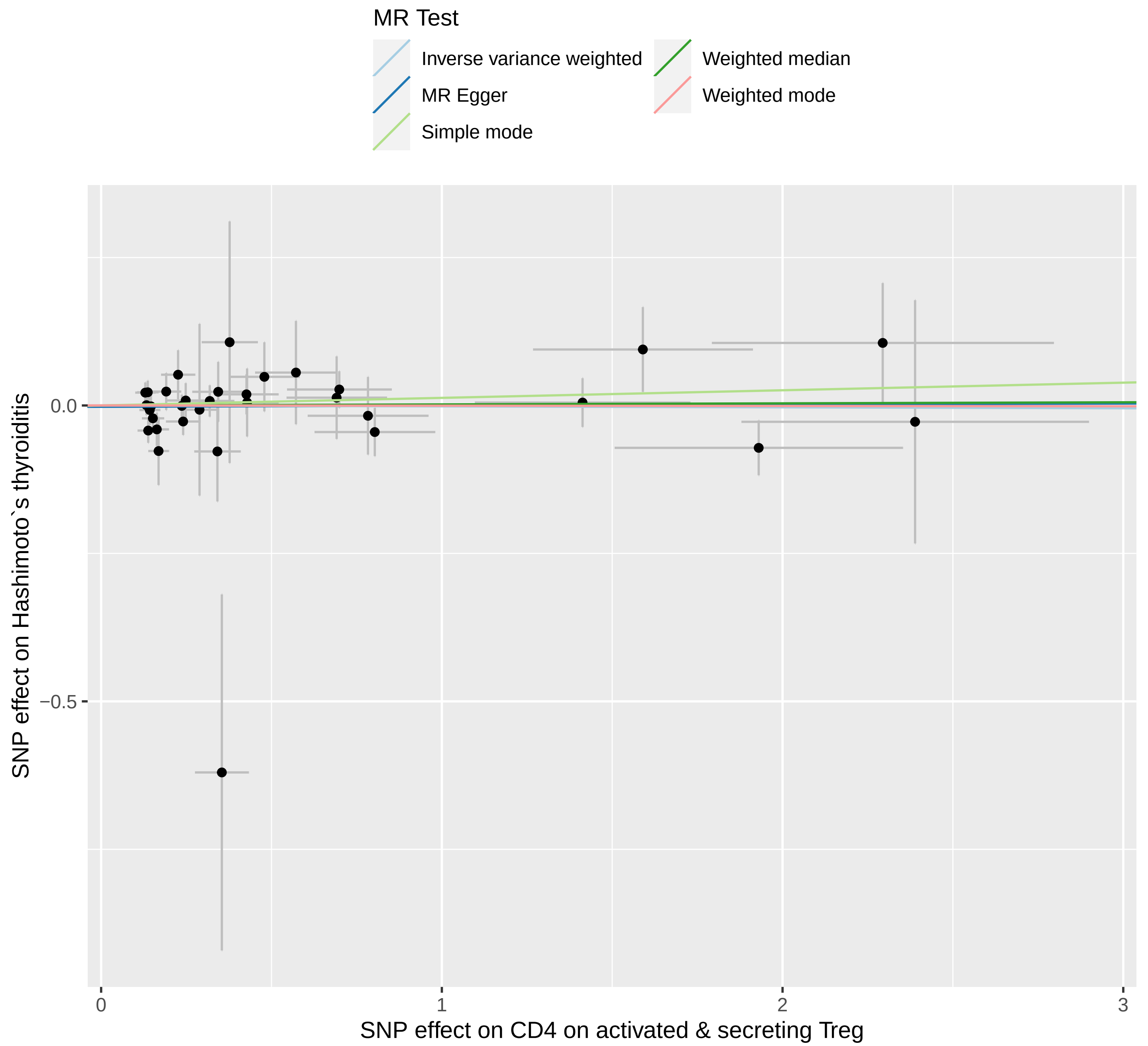

MR Test
Inverse variance weighted
MR Egger
Simple mode
Weighted median
Weighted mode
0.0
−0.5
0
1
2
3
SNP effect on Hashimoto`s thyroiditis
SNP effect on CD4 on activated & secreting Treg

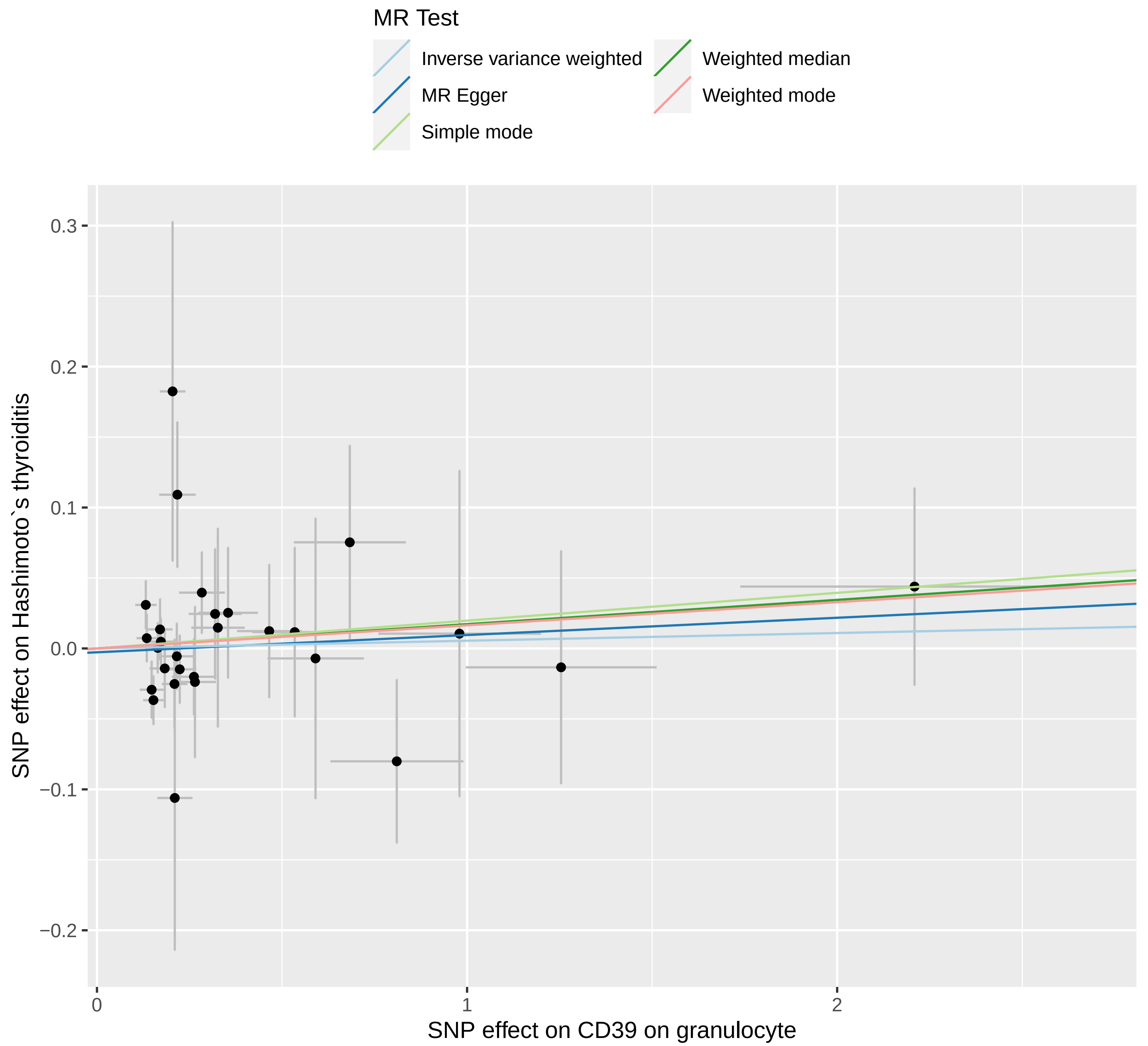

MR Test
Inverse variance weighted
MR Egger
Simple mode
Weighted median
Weighted mode
SNP effect on Hashimoto`s thyroiditis
0.3
0.2
0.1
0.0
−0.1
−0.2
0
1
2
SNP effect on CD39 on granulocyte

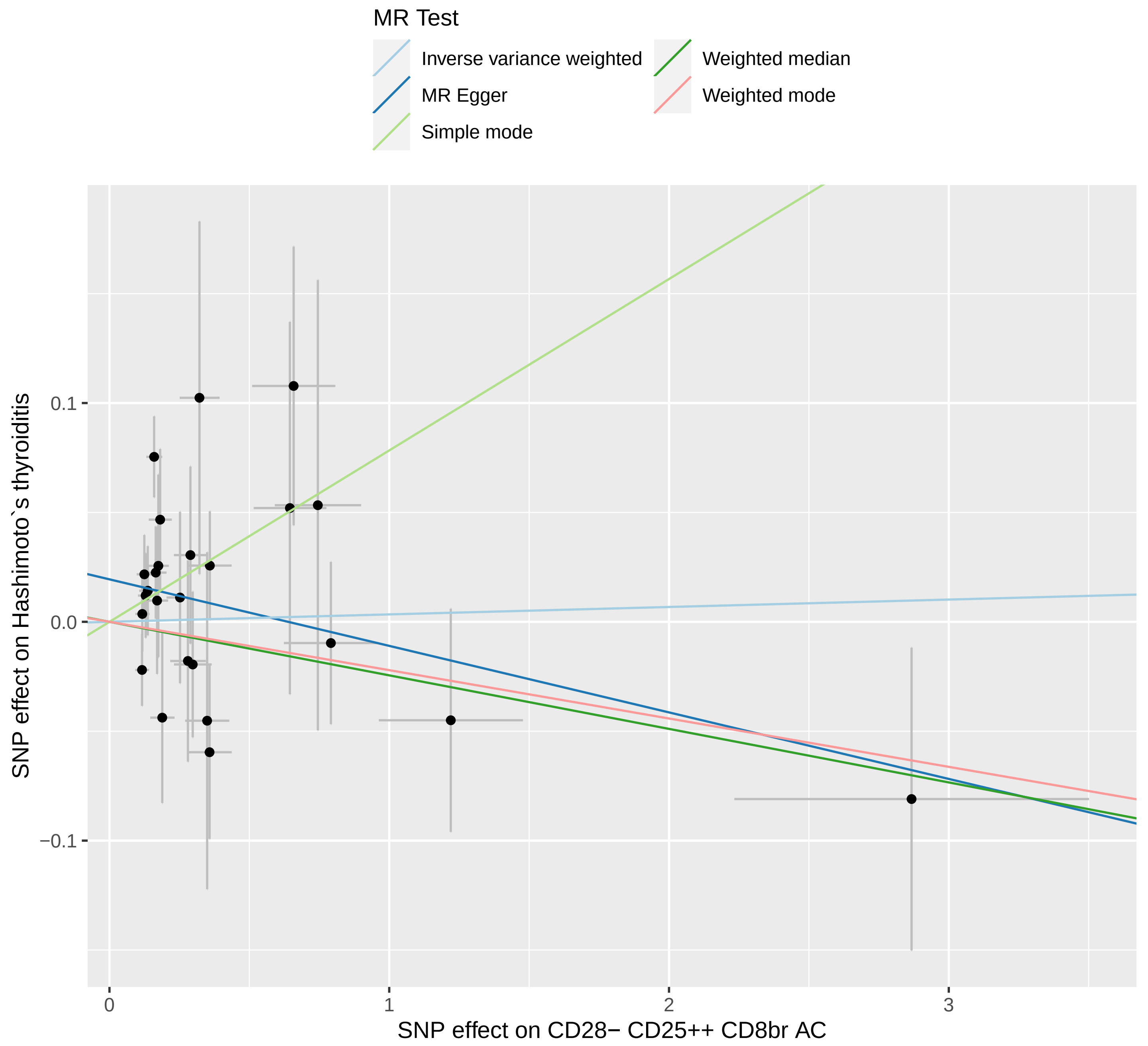
MR Test
Inverse variance weighted
MR Egger
Simple mode
Weighted median
Weighted mode
SNP effect on Hashimoto`s thyroiditis
0.1
0.0
−0.1
0
1
2
3
SNP effect on CD28− CD25++ CD8br AC

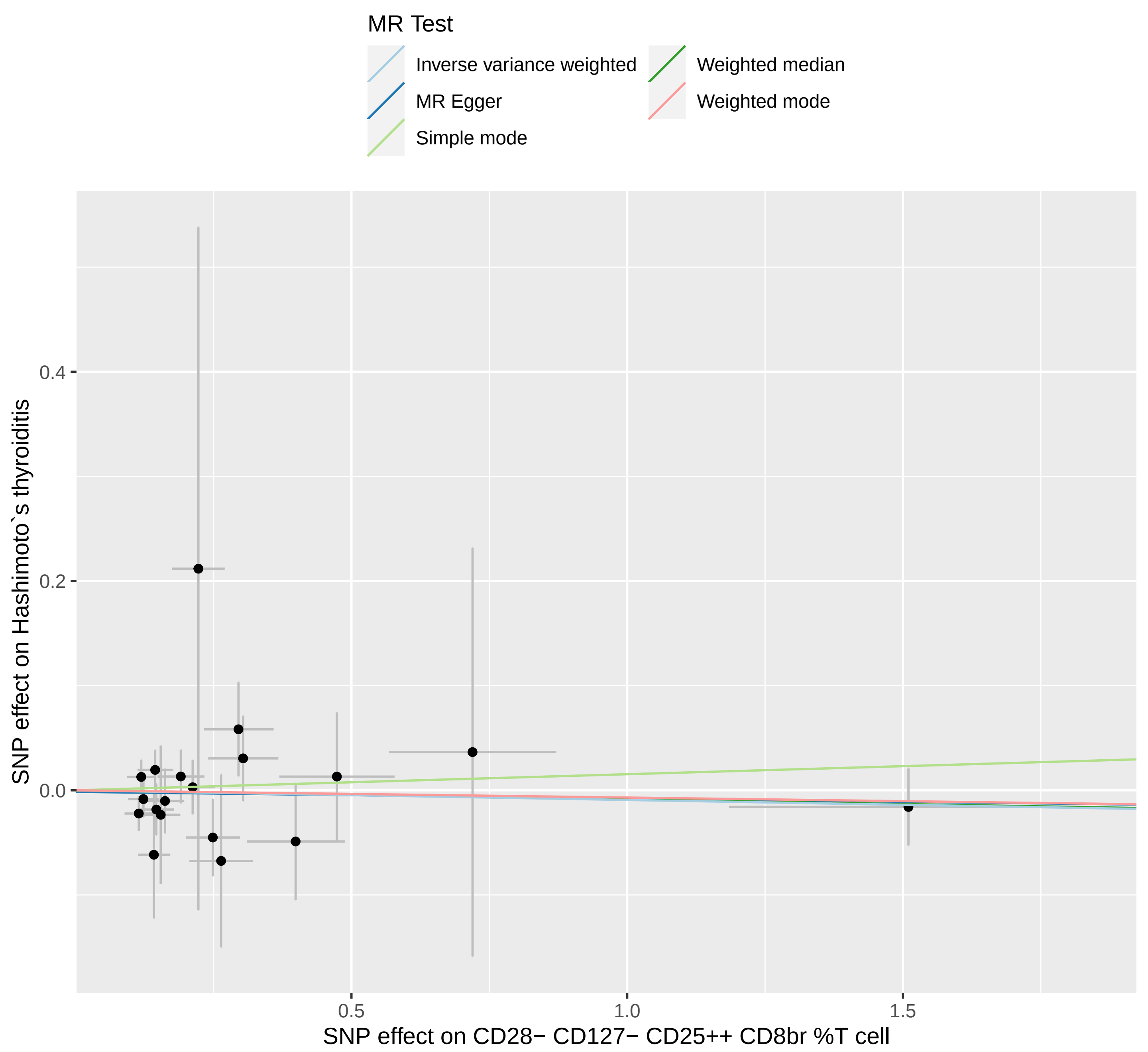
MR Test
Inverse variance weighted
MR Egger
Simple mode
Weighted median
Weighted mode
0.4
0.2
0.0
0.5
1.0
1.5
SNP effect on Hashimoto`s thyroiditis
SNP effect on CD28− CD127− CD25++ CD8br %T cell

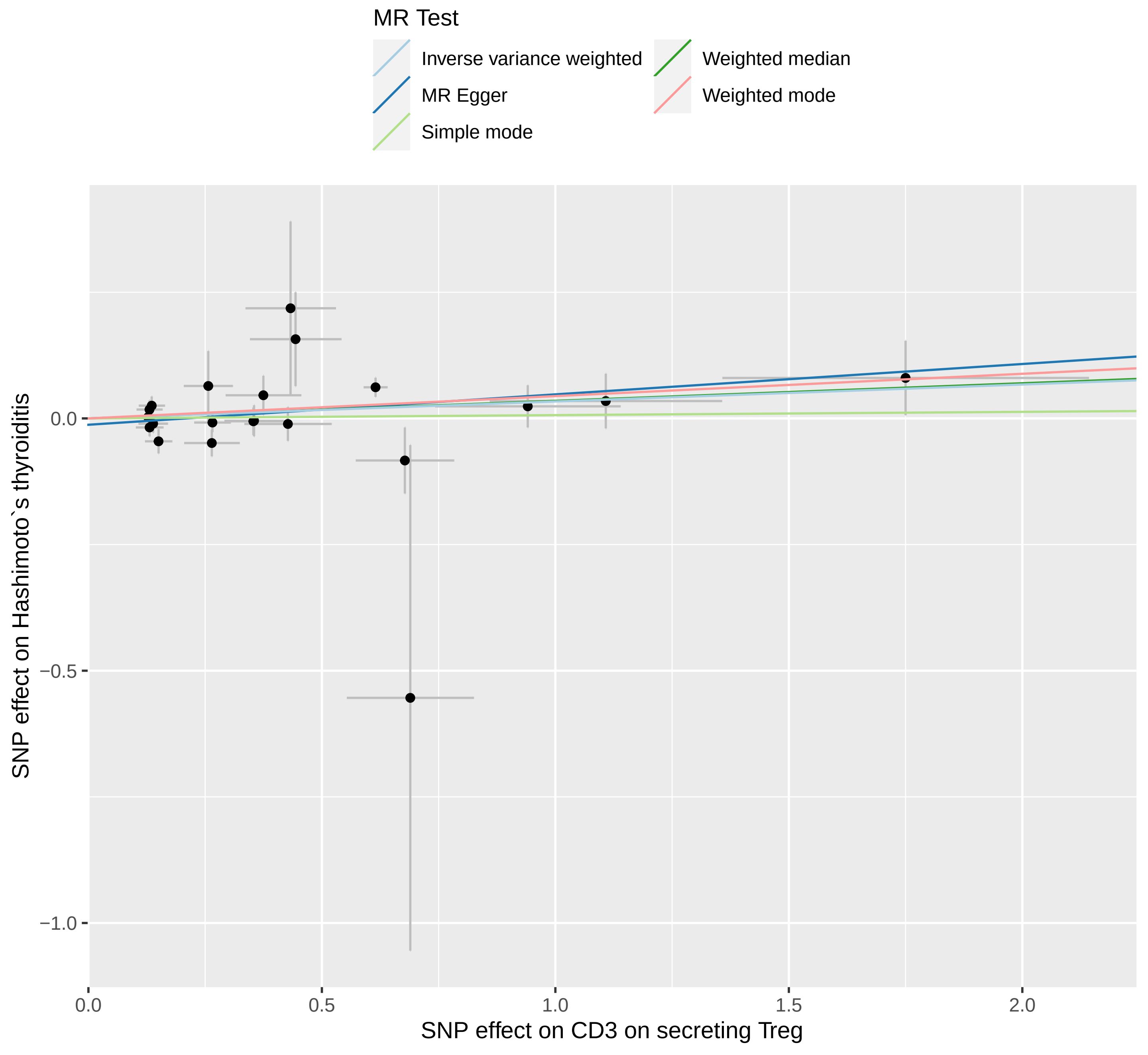
MR Test
Inverse variance weighted
MR Egger
Simple mode
Weighted median
Weighted mode
0.0
−0.5
−1.0
0.0
0.5
1.0
1.5
2.0
SNP effect on Hashimoto`s thyroiditis
SNP effect on CD3 on secreting Treg

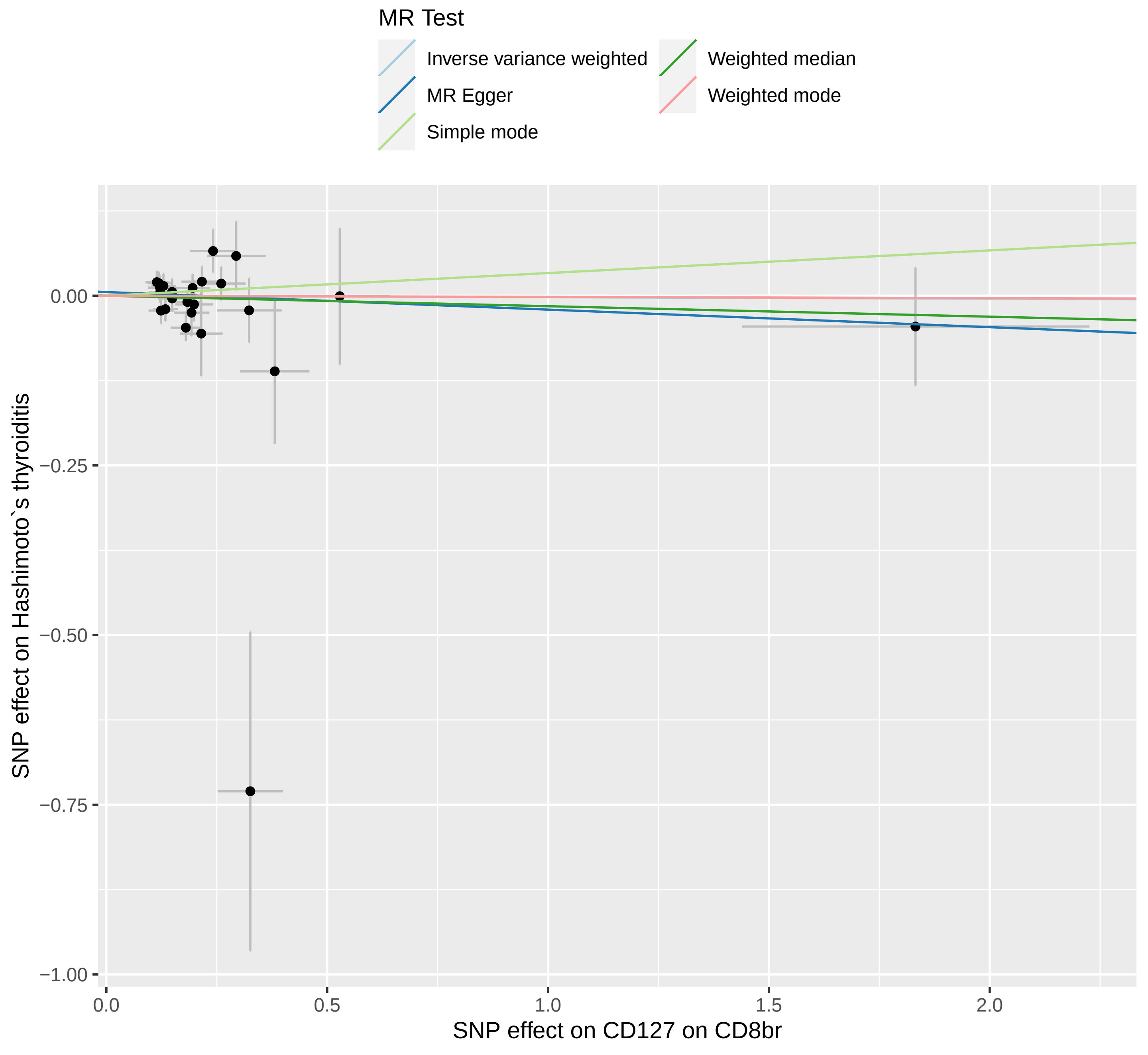

MR Test
Inverse variance weighted
MR Egger
Simple mode
Weighted median
Weighted mode
0.00
−0.25
−0.50
−0.75
−1.00
SNP effect on Hashimoto`s thyroiditis
0.0
0.5
1.0
1.5
2.0
SNP effect on CD127 on CD8br

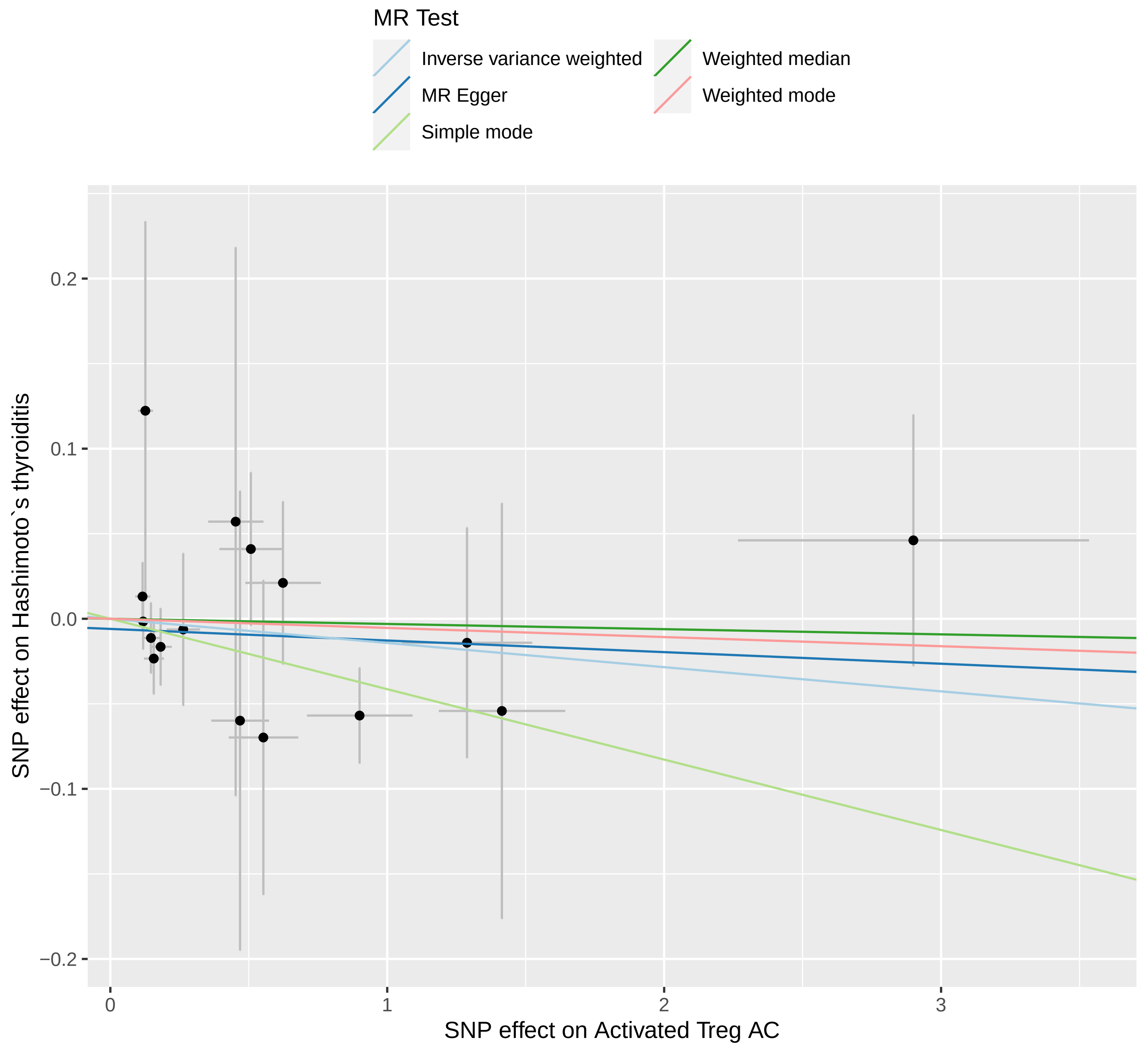
MR Test
Inverse variance weighted
MR Egger
Simple mode
Weighted median
Weighted mode
0.2
0.1
0.0
−0.1
−0.2
0
1
2
3
SNP effect on Hashimoto`s thyroiditis
SNP effect on Activated Treg AC

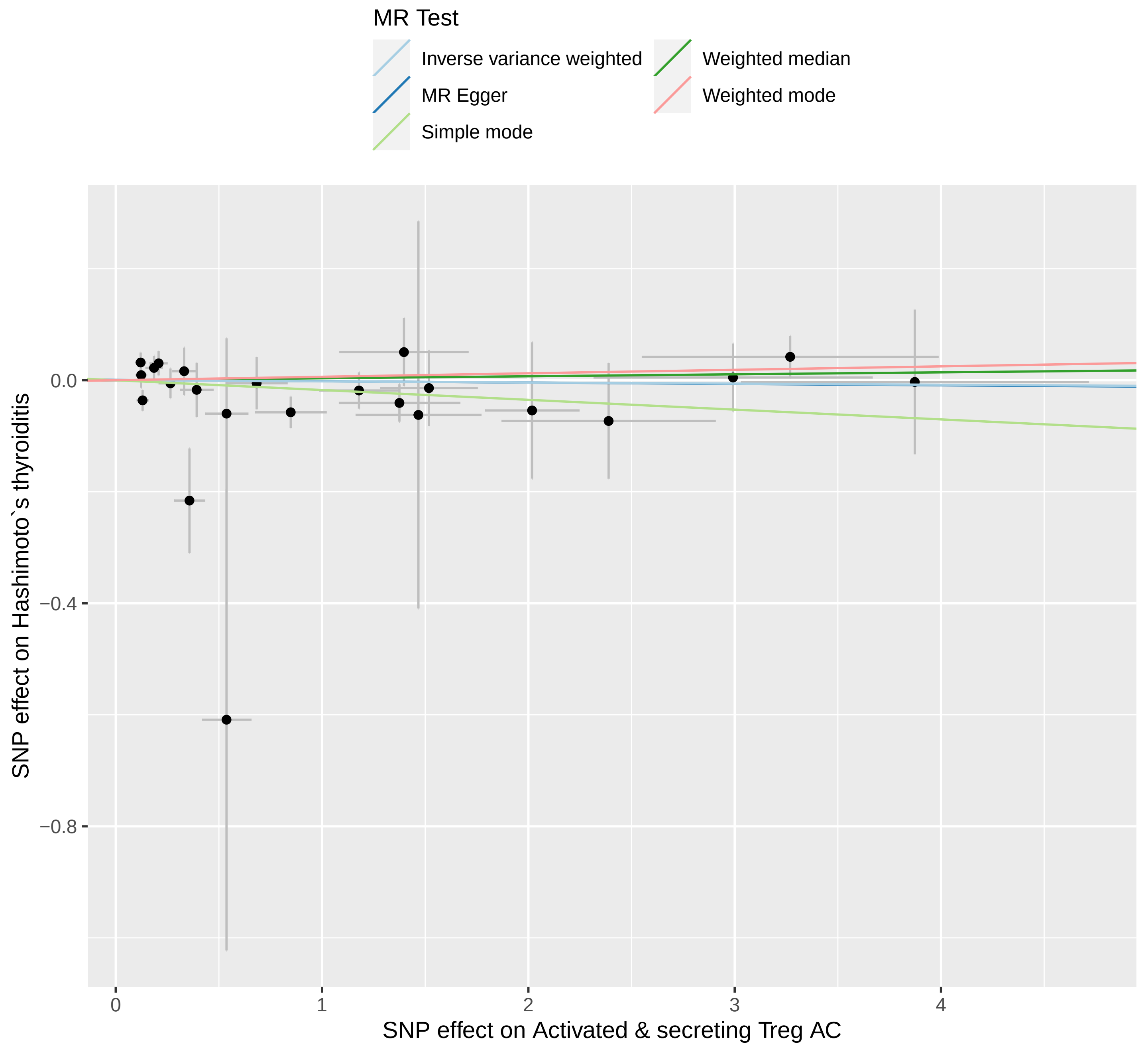

MR Test
Inverse variance weighted
MR Egger
Simple mode
Weighted median
Weighted mode
0.0
−0.4
−0.8
0
1
2
3
4
SNP effect on Hashimoto`s thyroiditis
SNP effect on Activated & secreting Treg AC

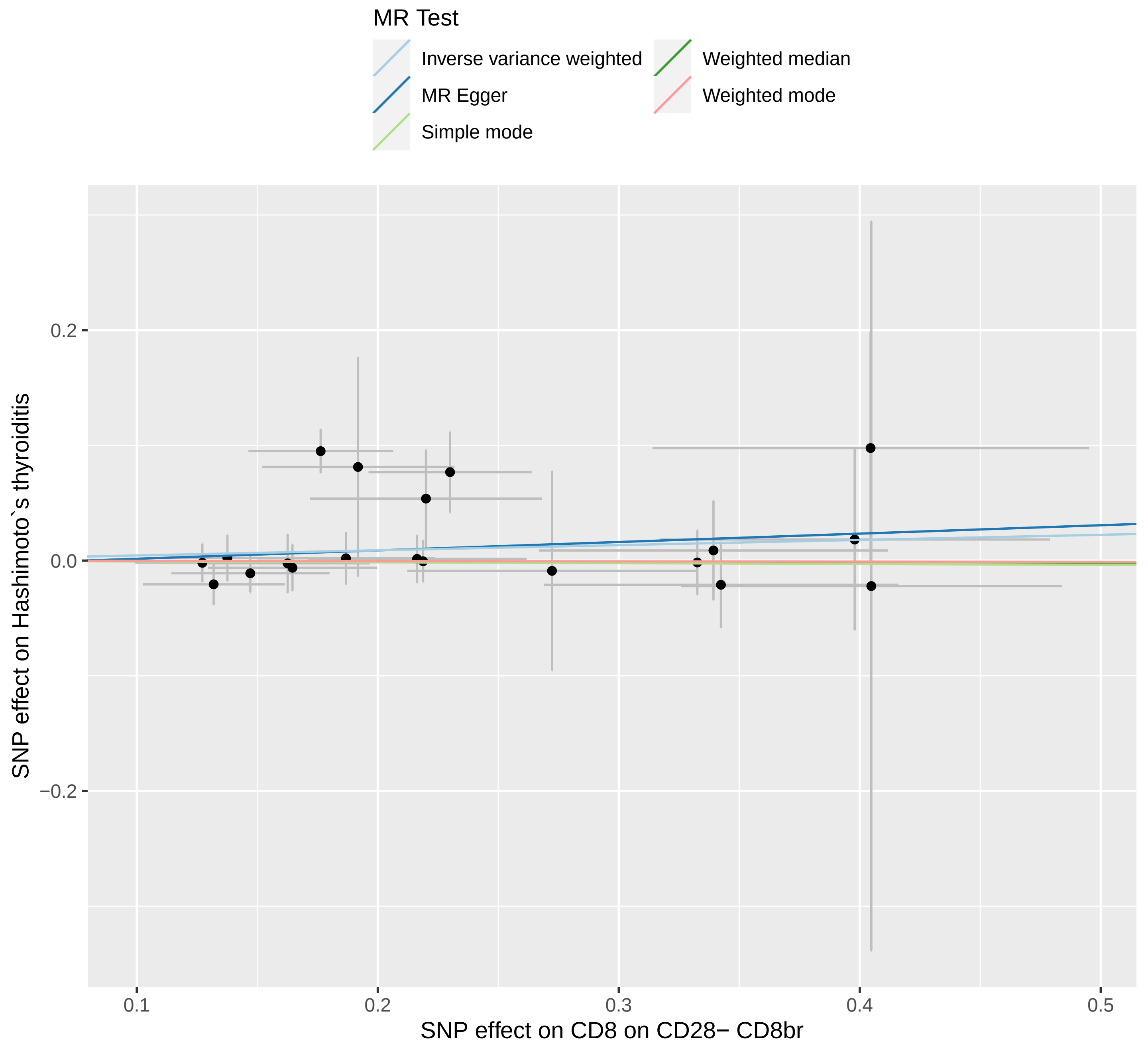
MR Test
Inverse variance weighted
MR Egger
Simple mode
Weighted median
Weighted mode
0.2
0.0
−0.2
0.1
0.2
0.3
0.4
0.5
SNP effect on Hashimoto`s thyroiditis
SNP effect on CD8 on CD28− CD8br

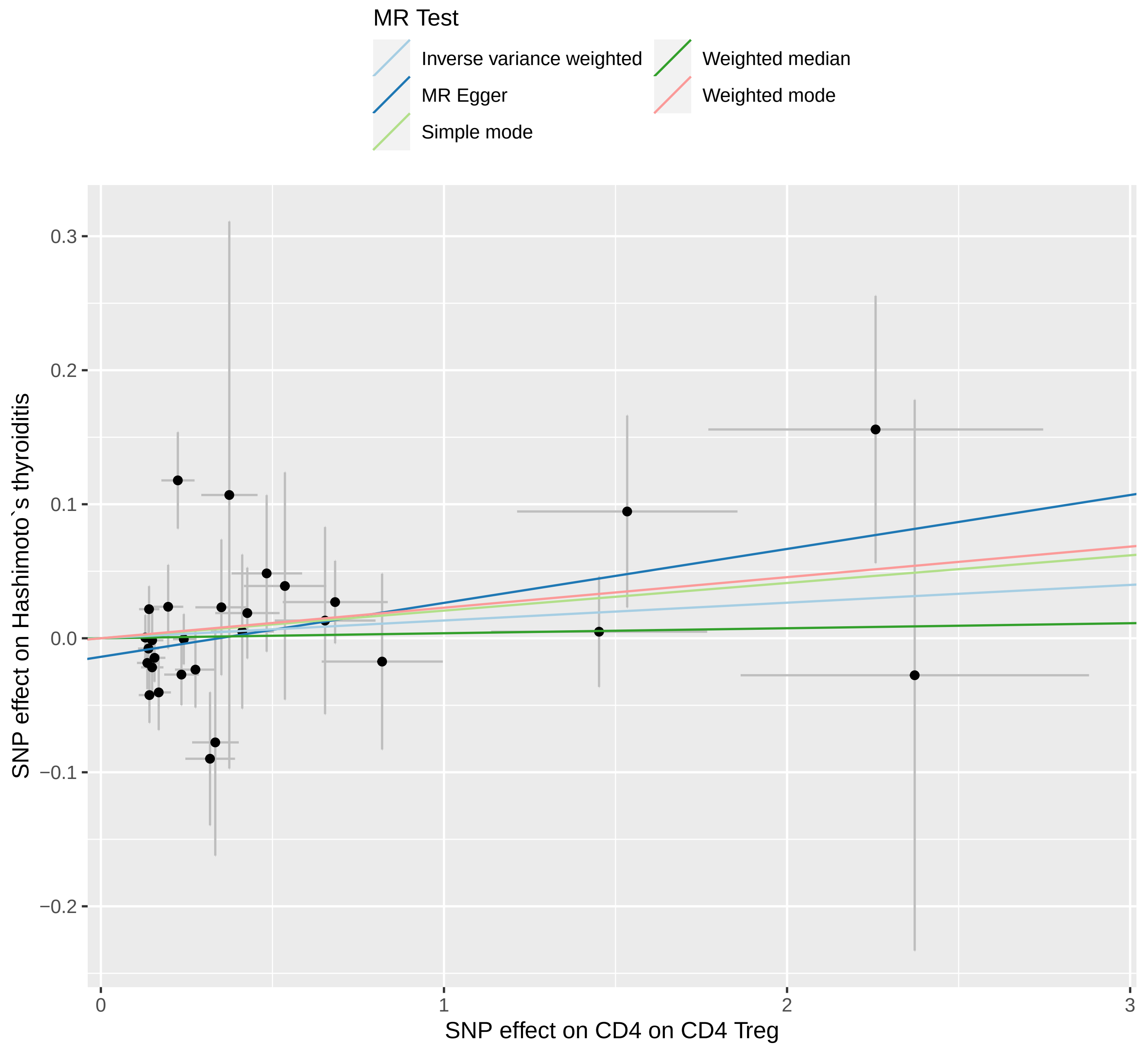

MR Test
Inverse variance weighted
MR Egger
Simple mode
Weighted median
Weighted mode
0.3
0.2
0.1
0.0
−0.1
−0.2
SNP effect on Hashimoto`s thyroiditis
0
1
2
3
SNP effect on CD4 on CD4 Treg

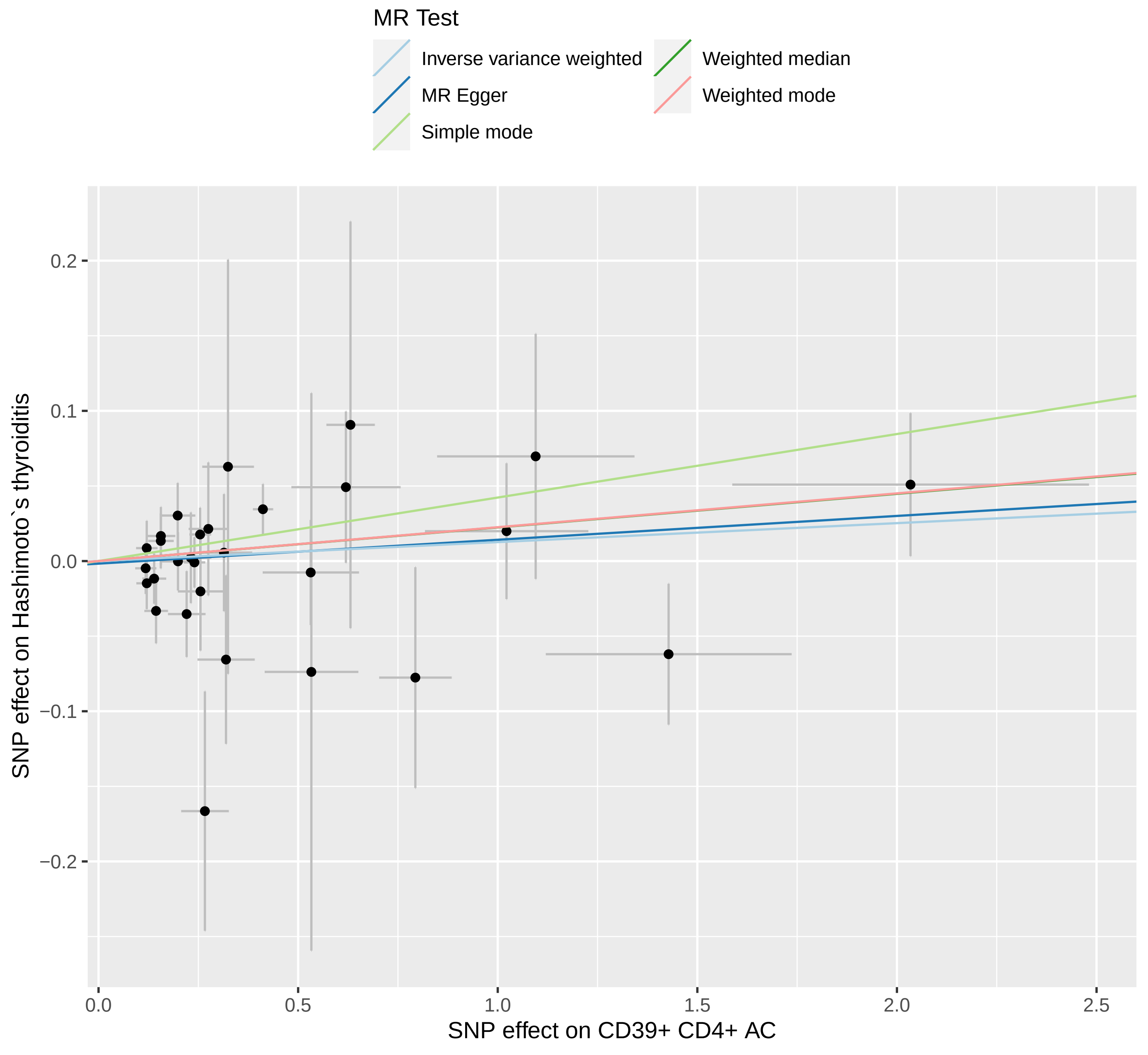

MR Test
Inverse variance weighted
MR Egger
Simple mode
Weighted median
Weighted mode
SNP effect on Hashimoto`s thyroiditis
SNP effect on CD39+ CD4+ AC
0.2
0.1
0.0
−0.1
−0.2
0.0
0.5
1.0
1.5
2.0
2.5

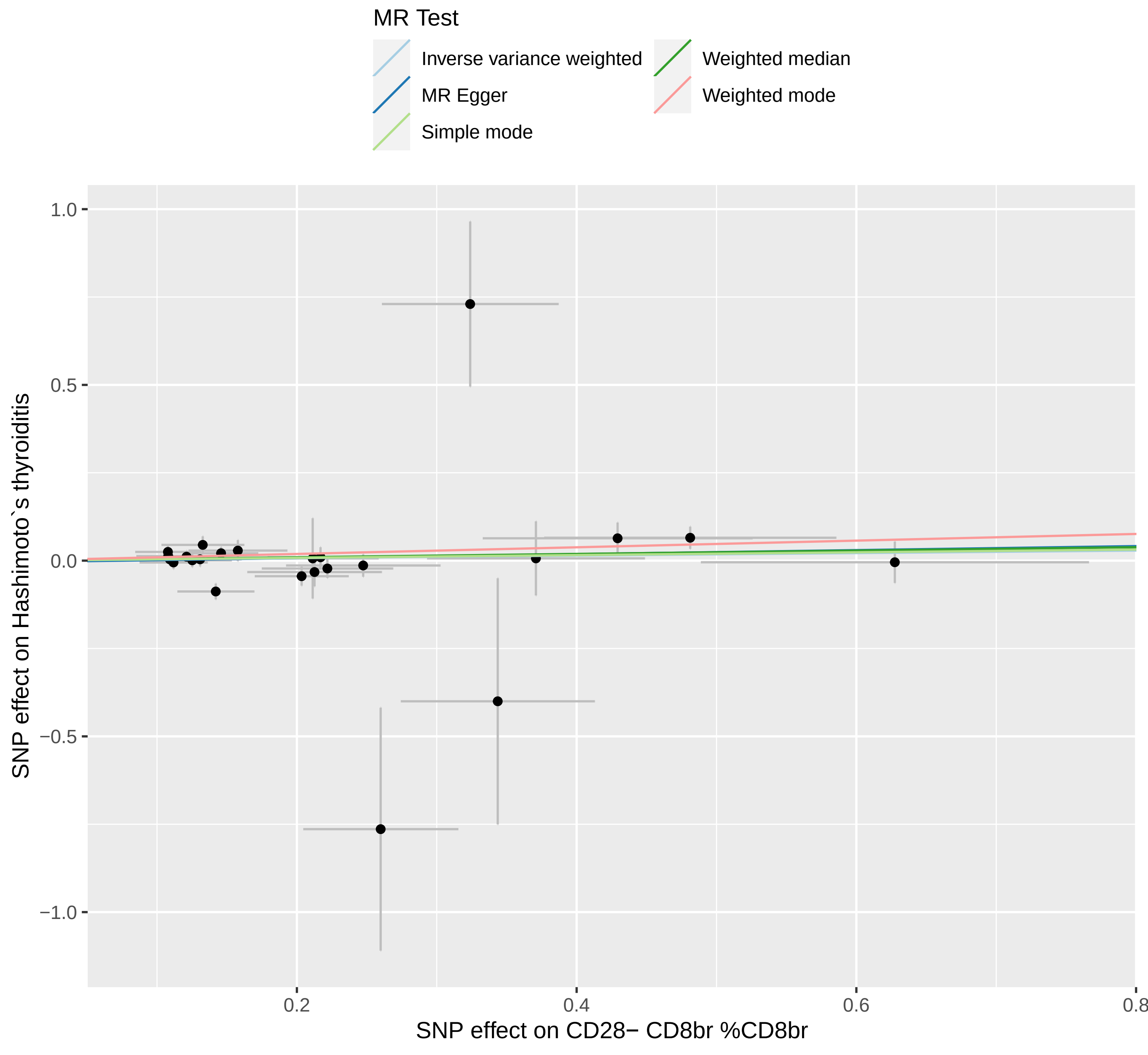
MR Test
Inverse variance weighted
MR Egger
Simple mode
Weighted median
Weighted mode
1.0
0.5
0.0
−0.5
−1.0
SNP effect on Hashimoto`s thyroiditis
0.2
0.4
0.6
0.8
SNP effect on CD28− CD8br %CD8br

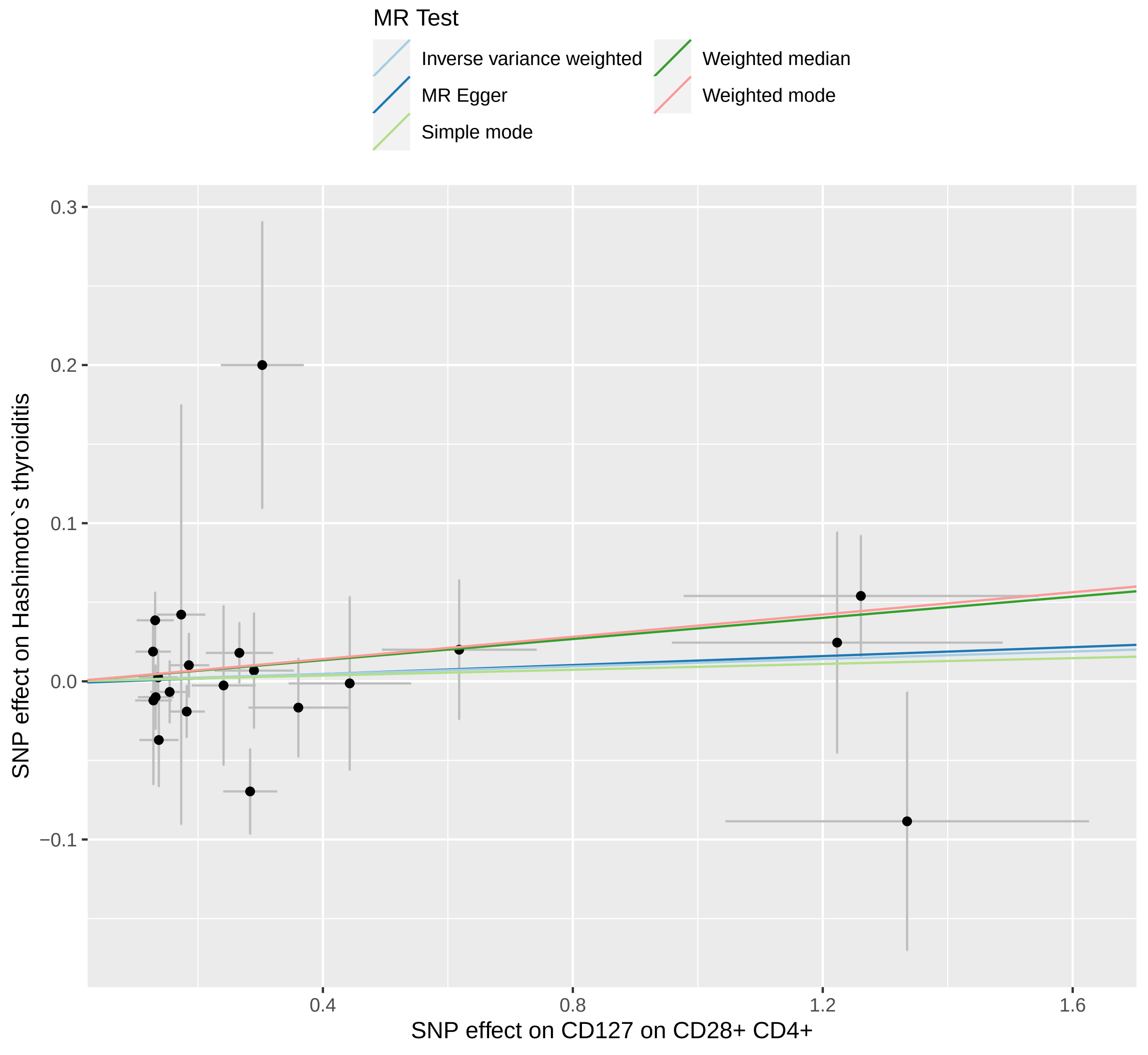
MR Test
Inverse variance weighted
MR Egger
Simple mode
Weighted median
Weighted mode
0.3
0.2
0.1
0.0
−0.1
0.4
0.8
1.2
1.6
SNP effect on Hashimoto`s thyroiditis
SNP effect on CD127 on CD28+ CD4+

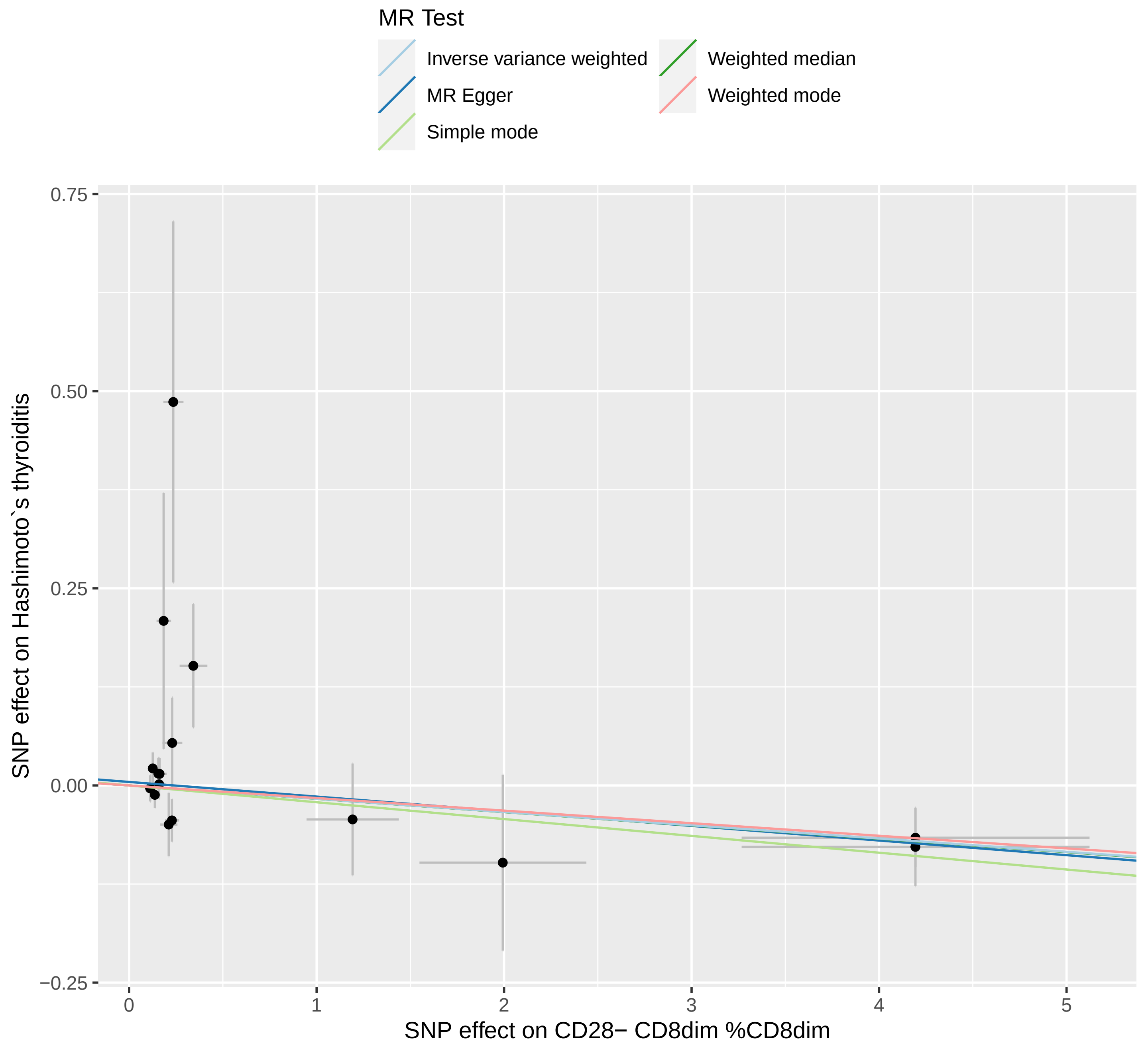
MR Test
Inverse variance weighted
MR Egger
Simple mode
Weighted median
Weighted mode
0.75
0.50
0.25
0.00
−0.25
0
1
2
3
4
5
SNP effect on Hashimoto`s thyroiditis
SNP effect on CD28− CD8dim %CD8dim

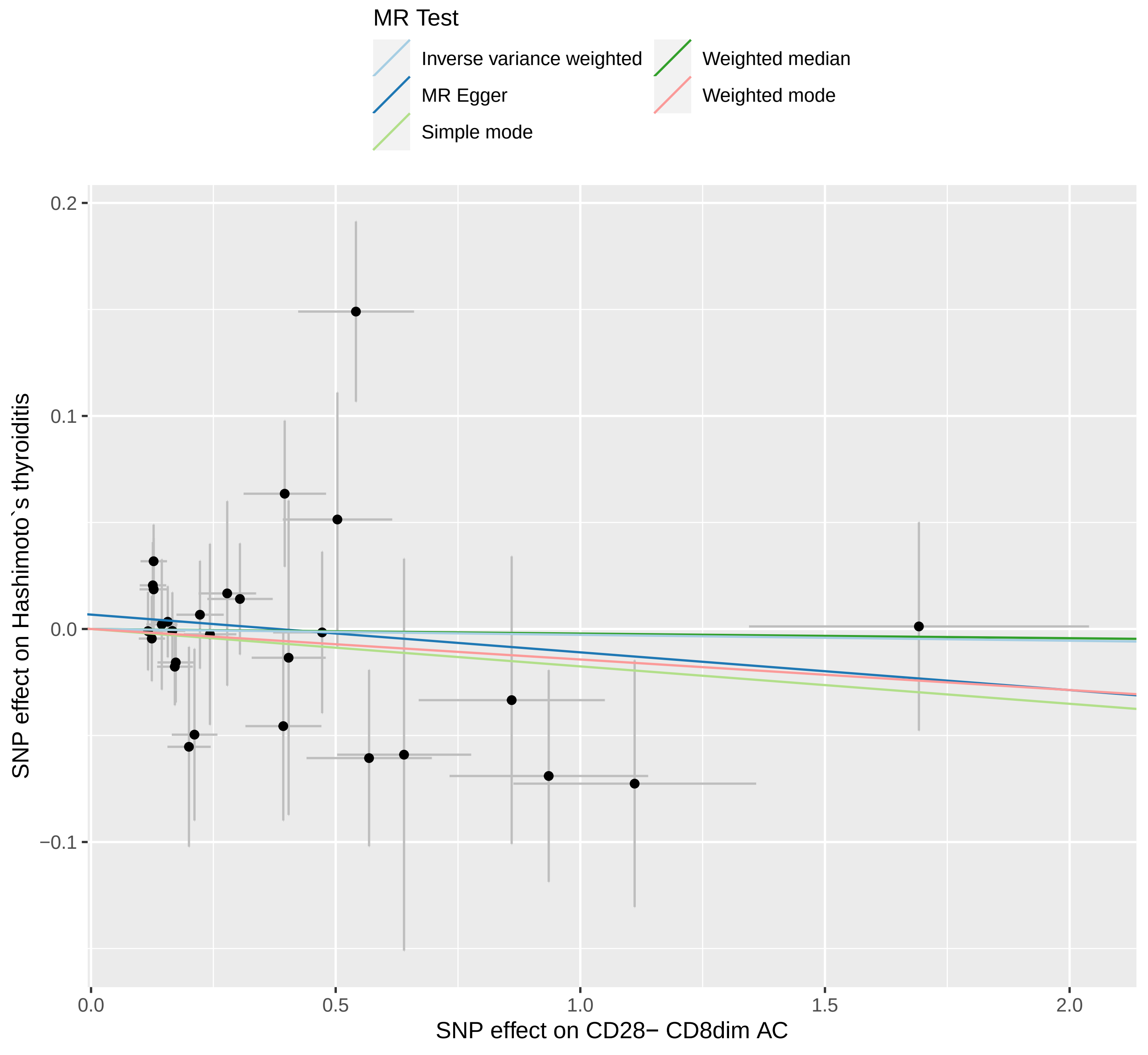

MR Test
Inverse variance weighted
MR Egger
Simple mode
Weighted median
Weighted mode
0.2
0.1
0.0
−0.1
0.0
0.5
1.0
1.5
2.0
SNP effect on Hashimoto`s thyroiditis
SNP effect on CD28− CD8dim AC

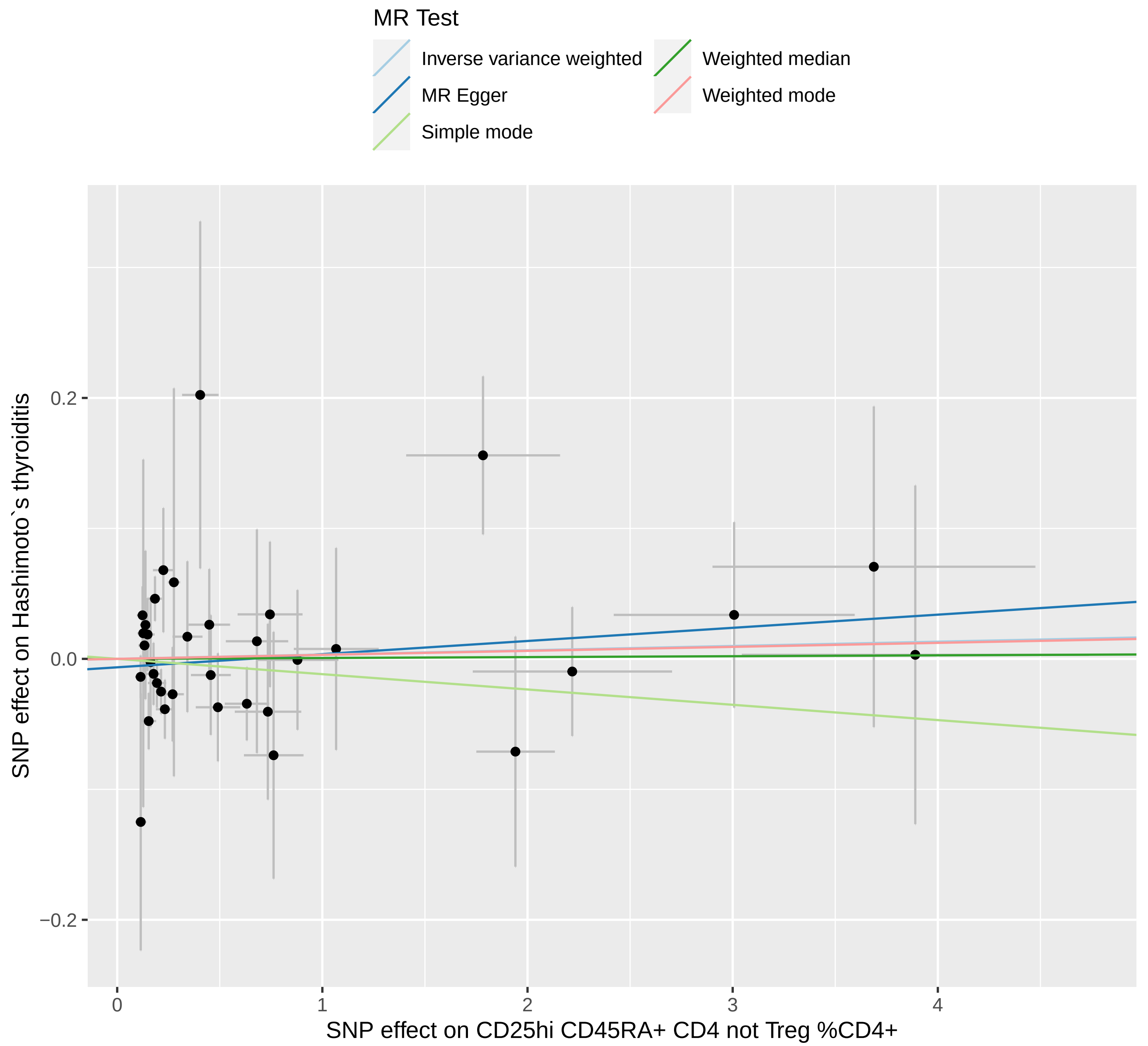
MR Test
Inverse variance weighted
MR Egger
Simple mode
Weighted median
Weighted mode
0.2
0.0
−0.2
0
1
2
3
4
SNP effect on Hashimoto`s thyroiditis
SNP effect on CD25hi CD45RA+ CD4 not Treg %CD4+

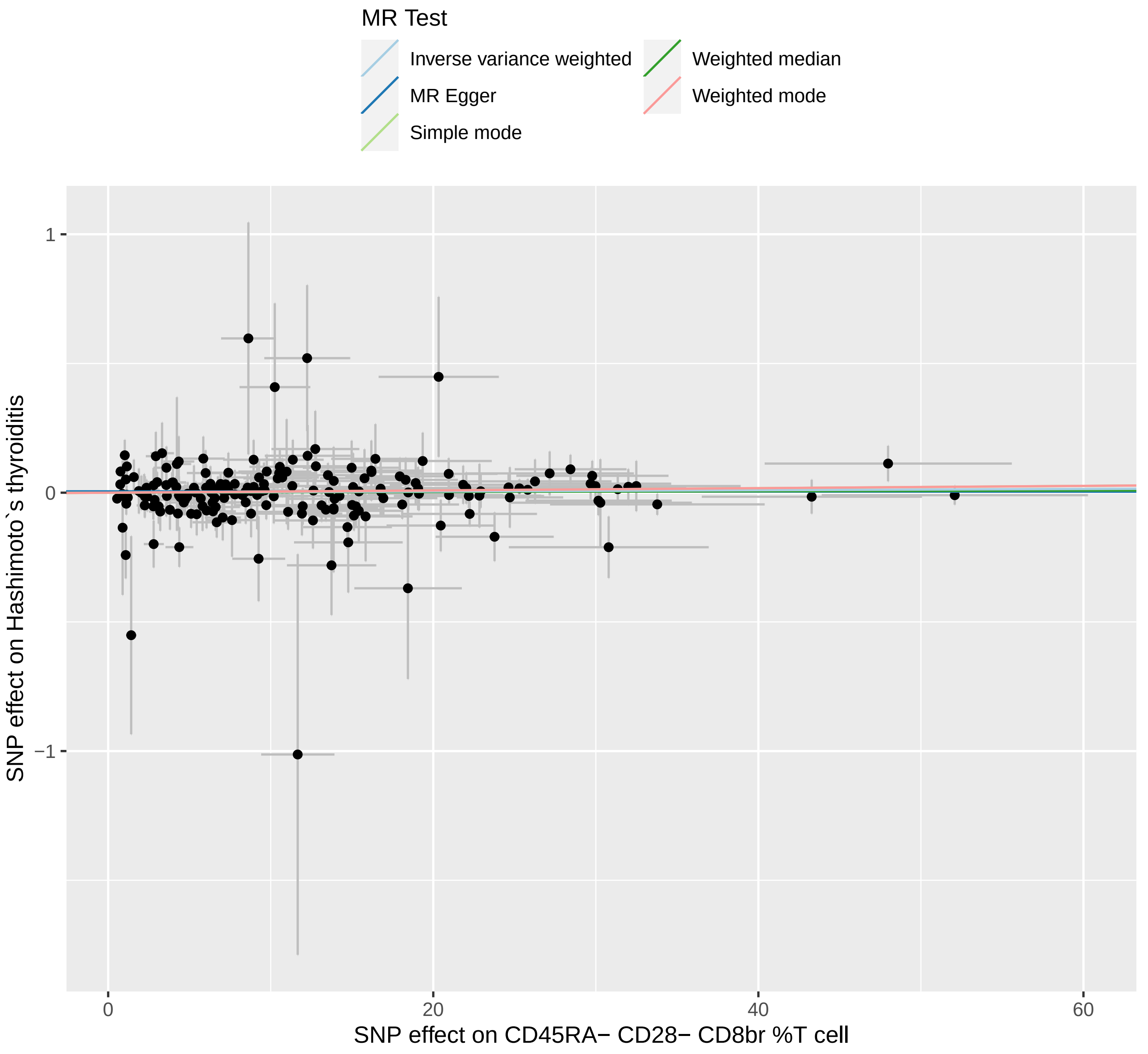
MR Test
Inverse variance weighted
MR Egger
Simple mode
Weighted median
Weighted mode
1
0
−1
0
20
40
60
SNP effect on Hashimoto`s thyroiditis
SNP effect on CD45RA− CD28− CD8br %T cell

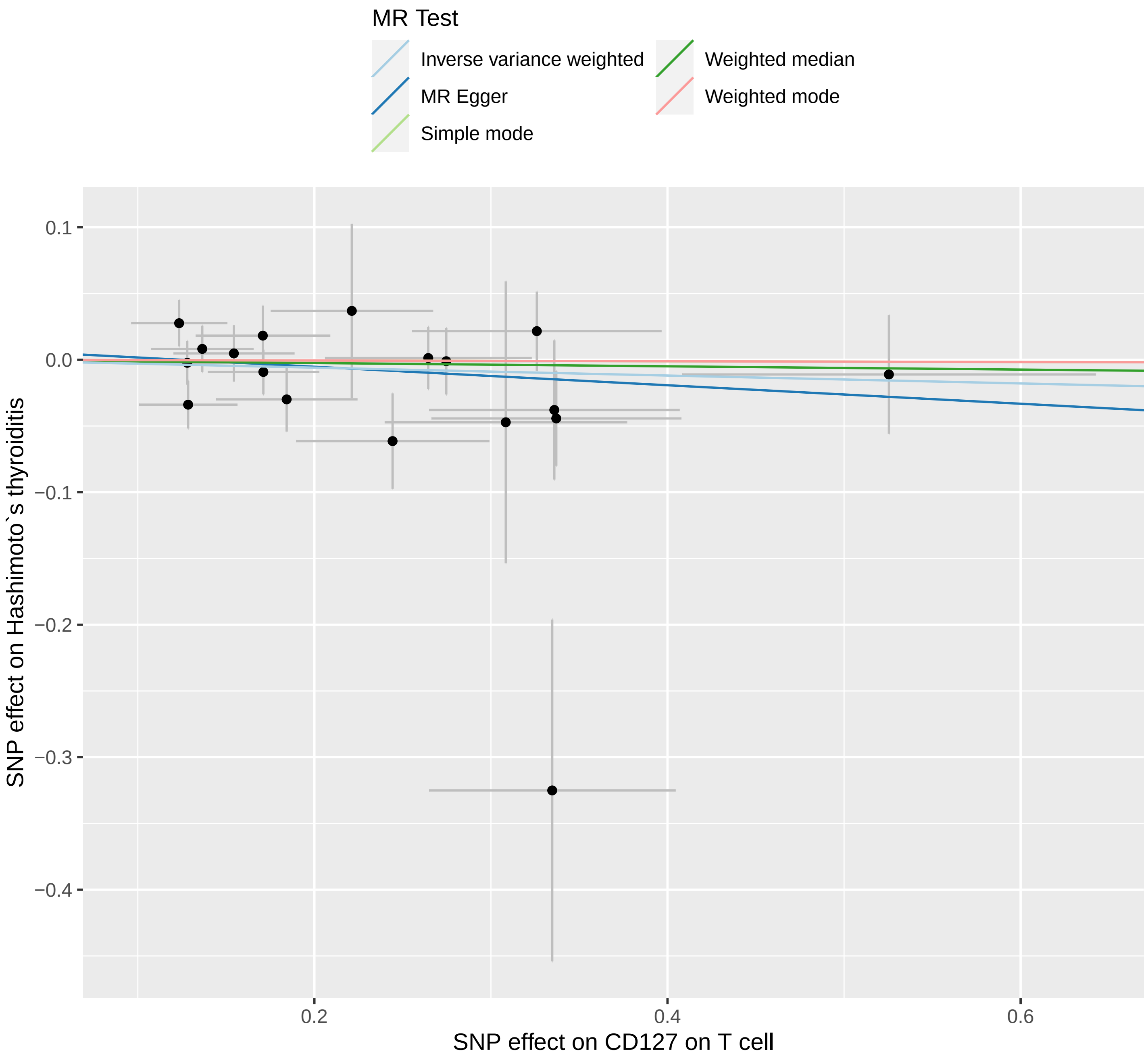
MR Test
Inverse variance weighted
MR Egger
Simple mode
Weighted median
Weighted mode
0.1
0.0
−0.1
−0.2
−0.3
−0.4
SNP effect on Hashimoto`s thyroiditis
0.2
0.4
0.6
SNP effect on CD127 on T cell

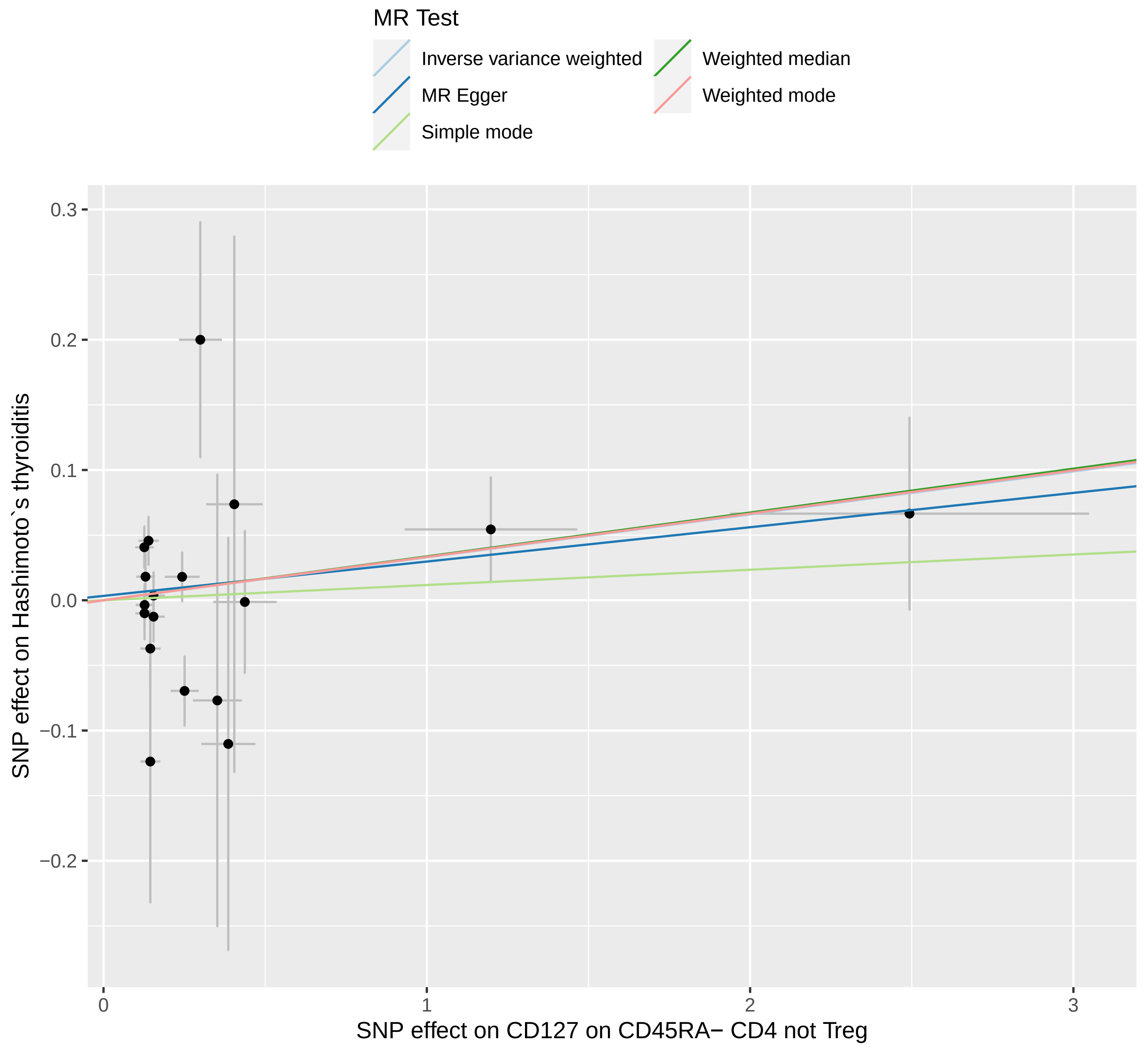
MR Test
Inverse variance weighted
MR Egger
Simple mode
Weighted median
Weighted mode
0.3
0.2
0.1
0.0
−0.1
−0.2
0
1
2
3
SNP effect on Hashimoto`s thyroiditis
SNP effect on CD127 on CD45RA− CD4 not Treg

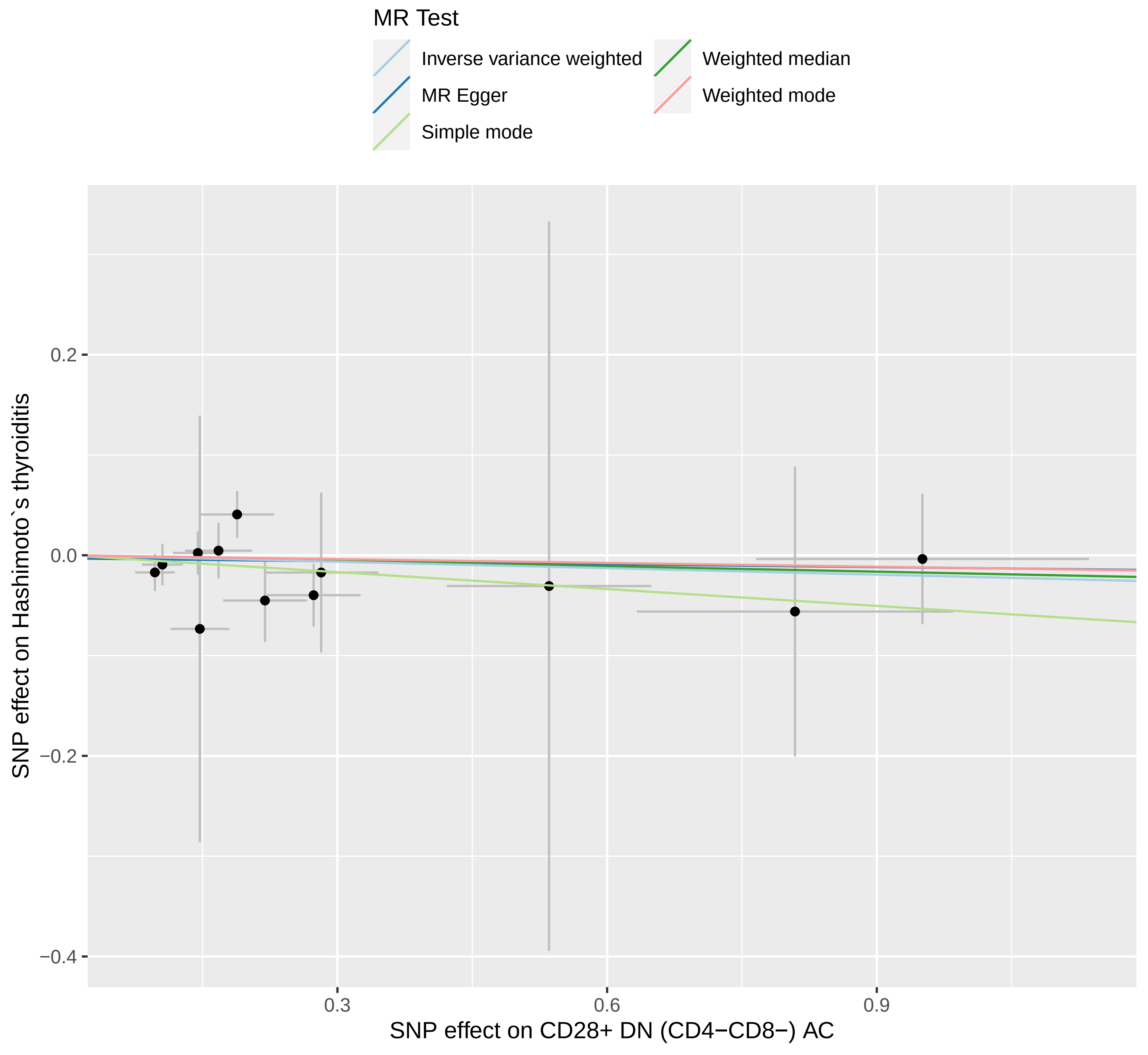
MR Test
Inverse variance weighted
MR Egger
Simple mode
Weighted median
Weighted mode
SNP effect on Hashimoto`s thyroiditis
0.2
0.0
−0.2
−0.4
0.3
0.6
0.9
SNP effect on CD28+ DN (CD4−CD8−) AC

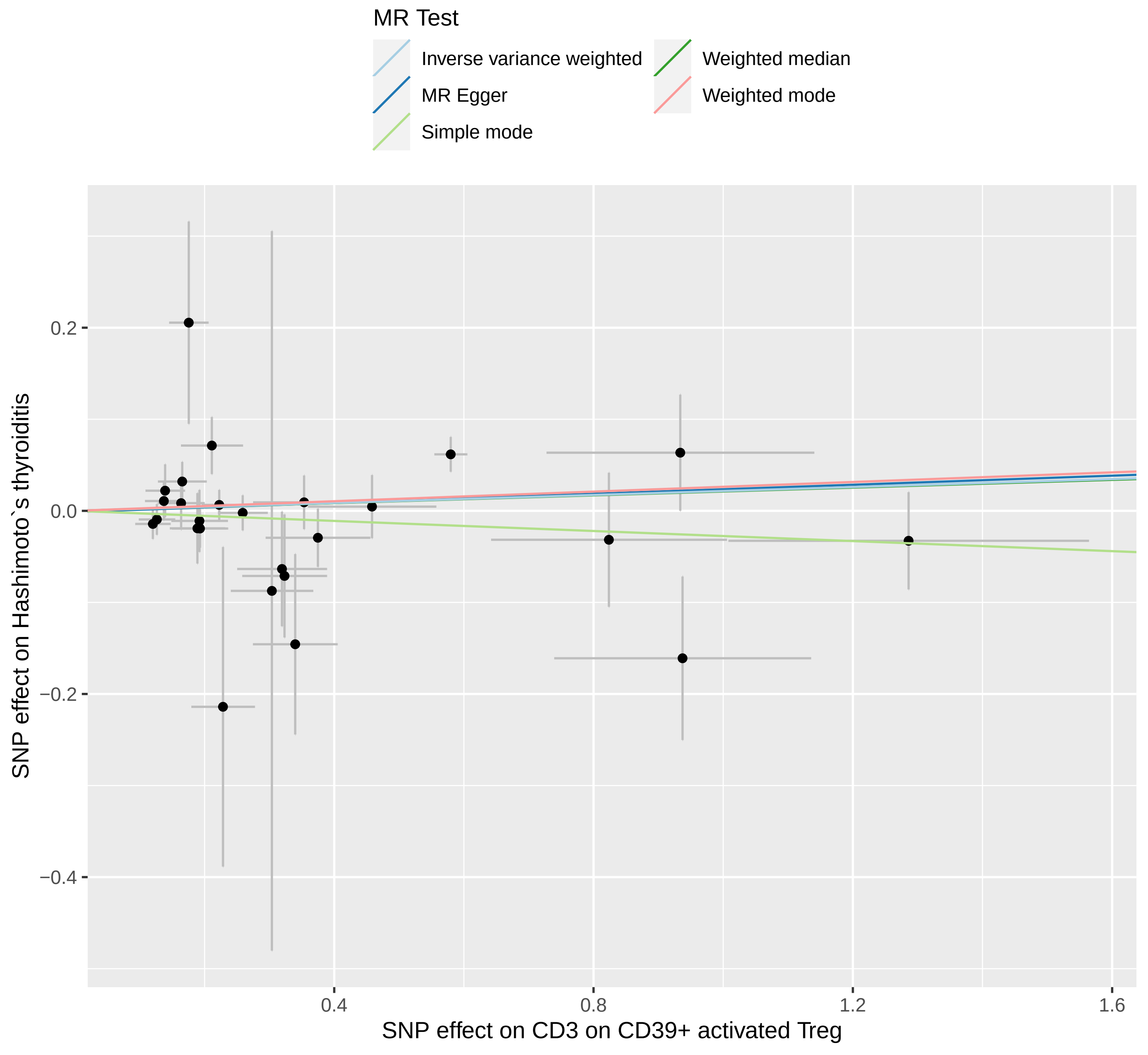

MR Test
Inverse variance weighted
MR Egger
Simple mode
Weighted median
Weighted mode
0.2
0.0
−0.2
−0.4
0.4
0.8
1.2
1.6
SNP effect on Hashimoto`s thyroiditis
SNP effect on CD3 on CD39+ activated Treg

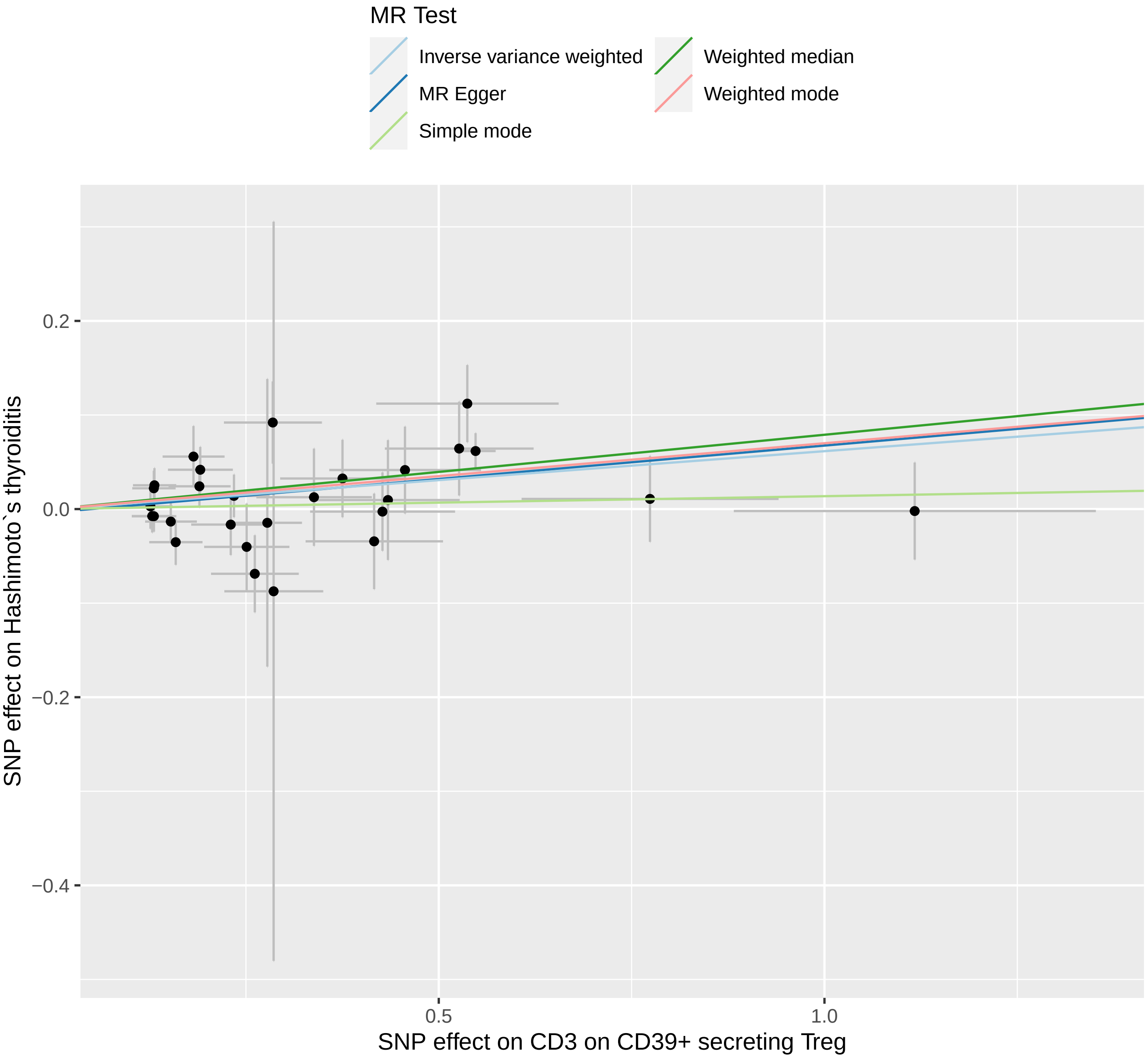
MR Test
Inverse variance weighted
MR Egger
Simple mode
Weighted median
Weighted mode
0.2
0.0
−0.2
−0.4
0.5
1.0
SNP effect on Hashimoto`s thyroiditis
SNP effect on CD3 on CD39+ secreting Treg

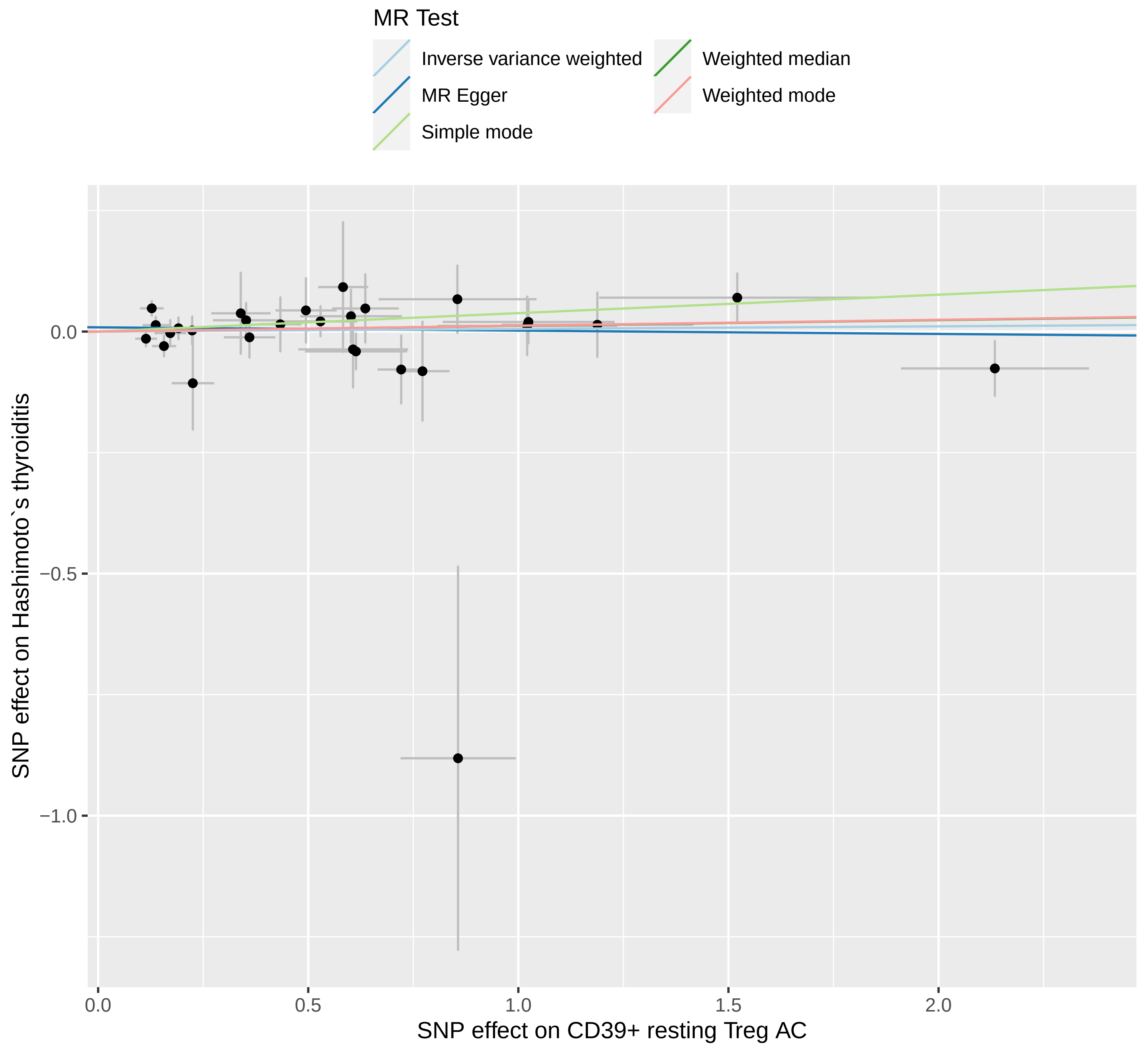
MR Test
Inverse variance weighted
MR Egger
Simple mode
Weighted median
Weighted mode
0.0
−0.5
−1.0
0.0
0.5
1.0
1.5
2.0
SNP effect on CD39+ resting Treg AC
SNP effect on Hashimoto`s thyroiditis

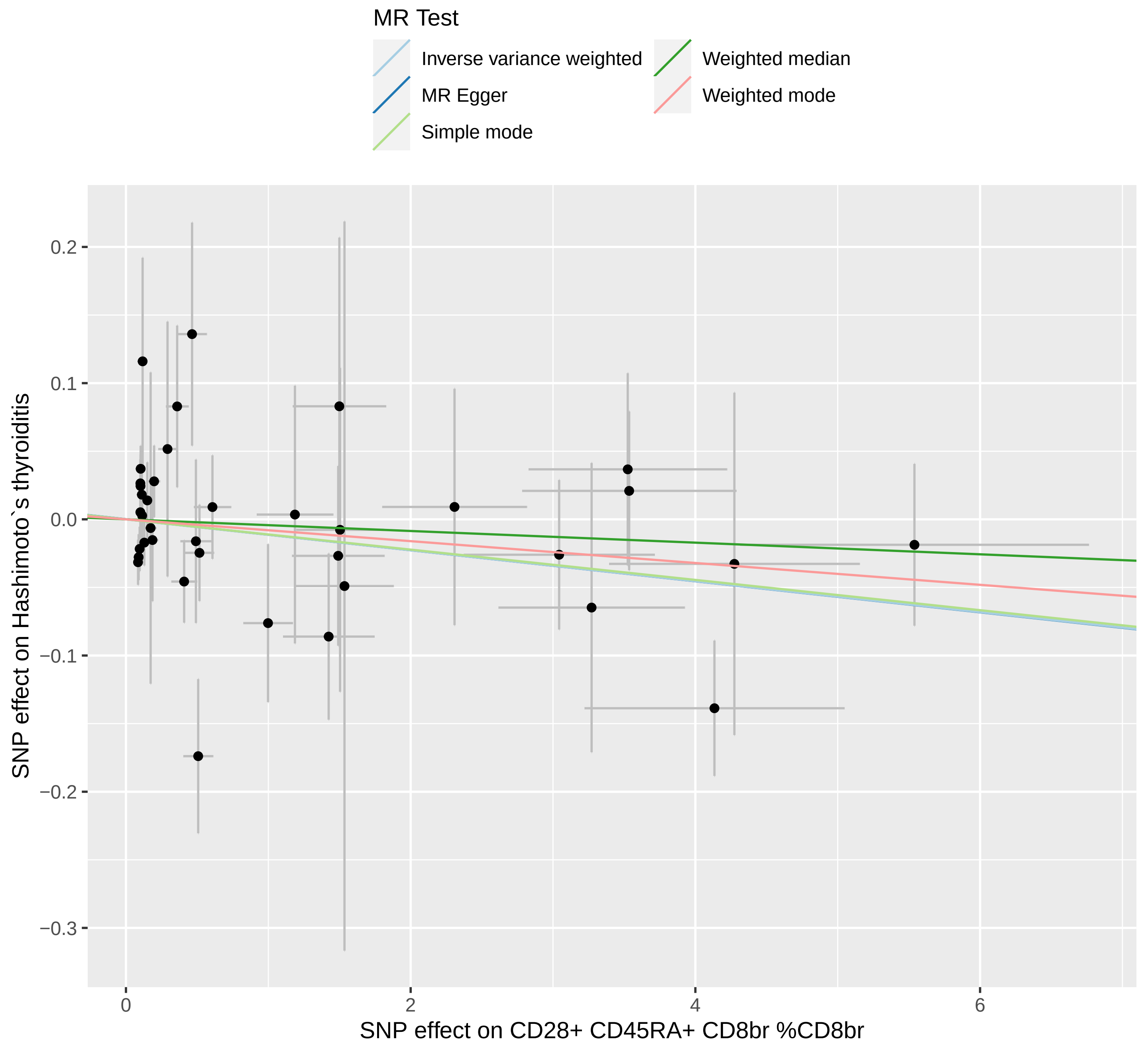
MR Test
Inverse variance weighted
MR Egger
Simple mode
Weighted median
Weighted mode
SNP effect on Hashimoto`s thyroiditis
0.2
0.1
0.0
−0.1
−0.2
−0.3
0
2
4
6
SNP effect on CD28+ CD45RA+ CD8br %CD8br

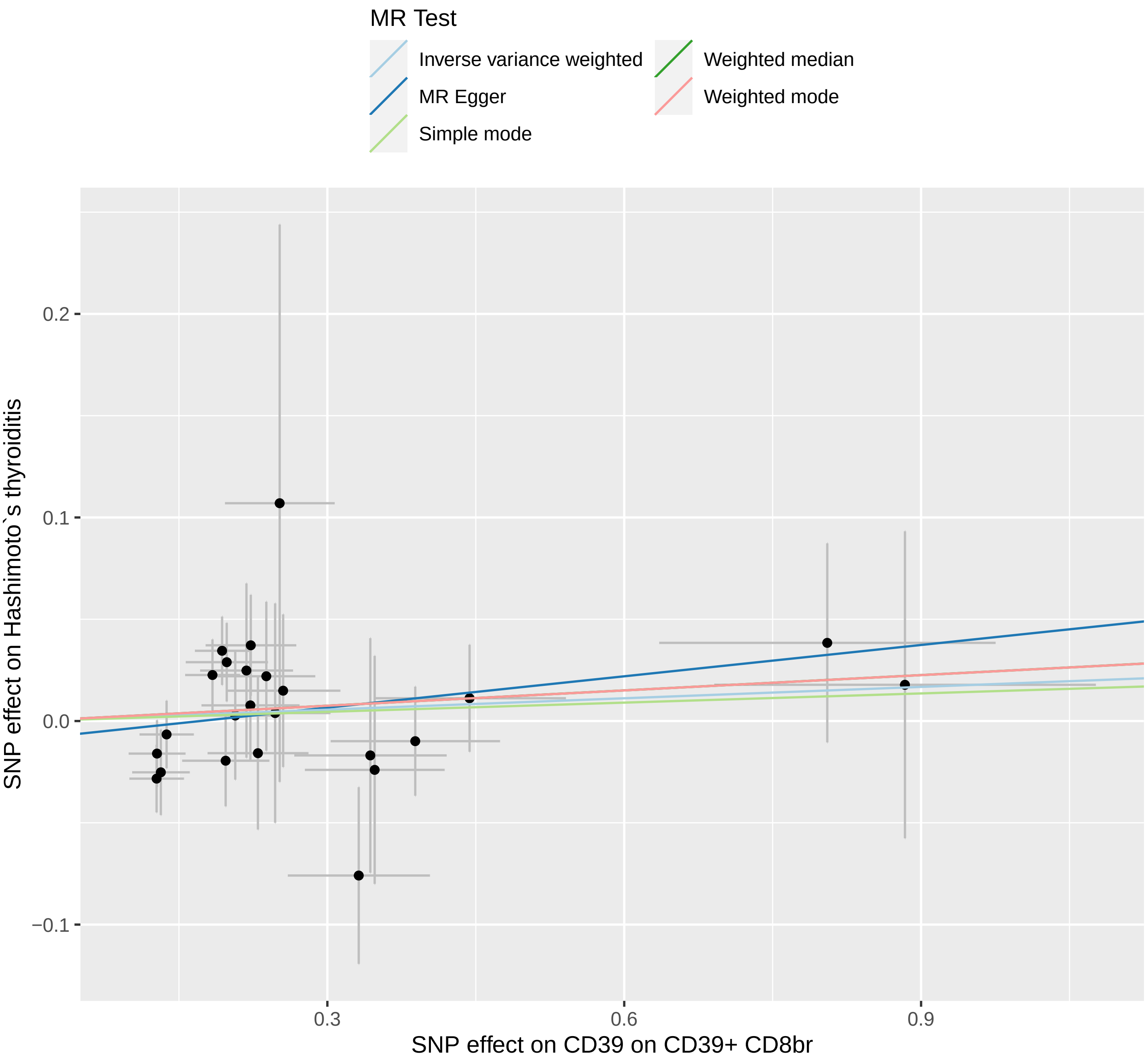

MR Test
Inverse variance weighted
MR Egger
Simple mode
Weighted median
Weighted mode
SNP effect on Hashimoto`s thyroiditis
0.2
0.1
0.0
−0.1
0.3
0.6
0.9
SNP effect on CD39 on CD39+ CD8br

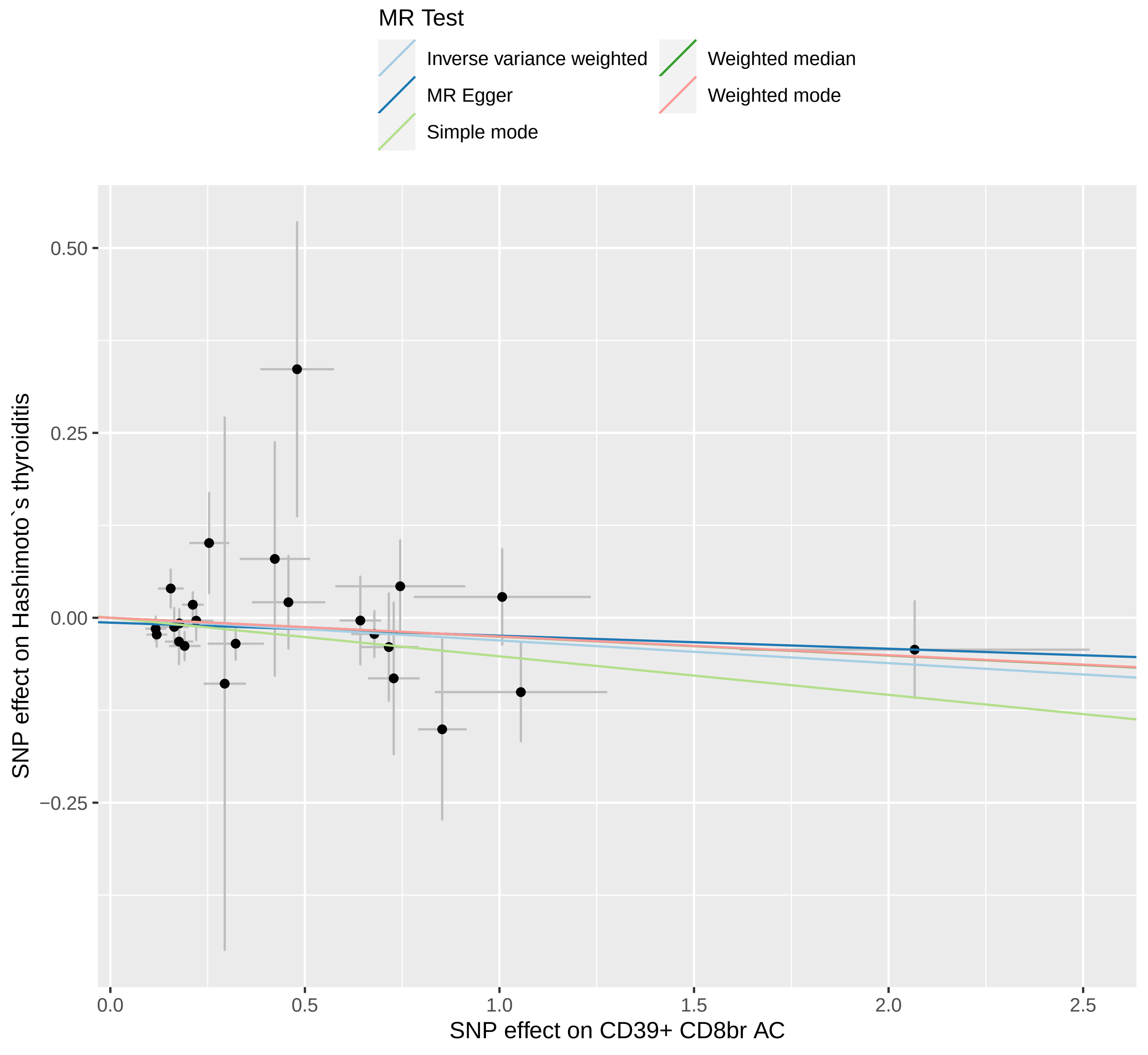

MR Test
Inverse variance weighted
MR Egger
Simple mode
Weighted median
Weighted mode
0.50
0.25
0.00
−0.25
0.0
0.5
1.0
1.5
2.0
2.5
SNP effect on Hashimoto`s thyroiditis
SNP effect on CD39+ CD8br AC

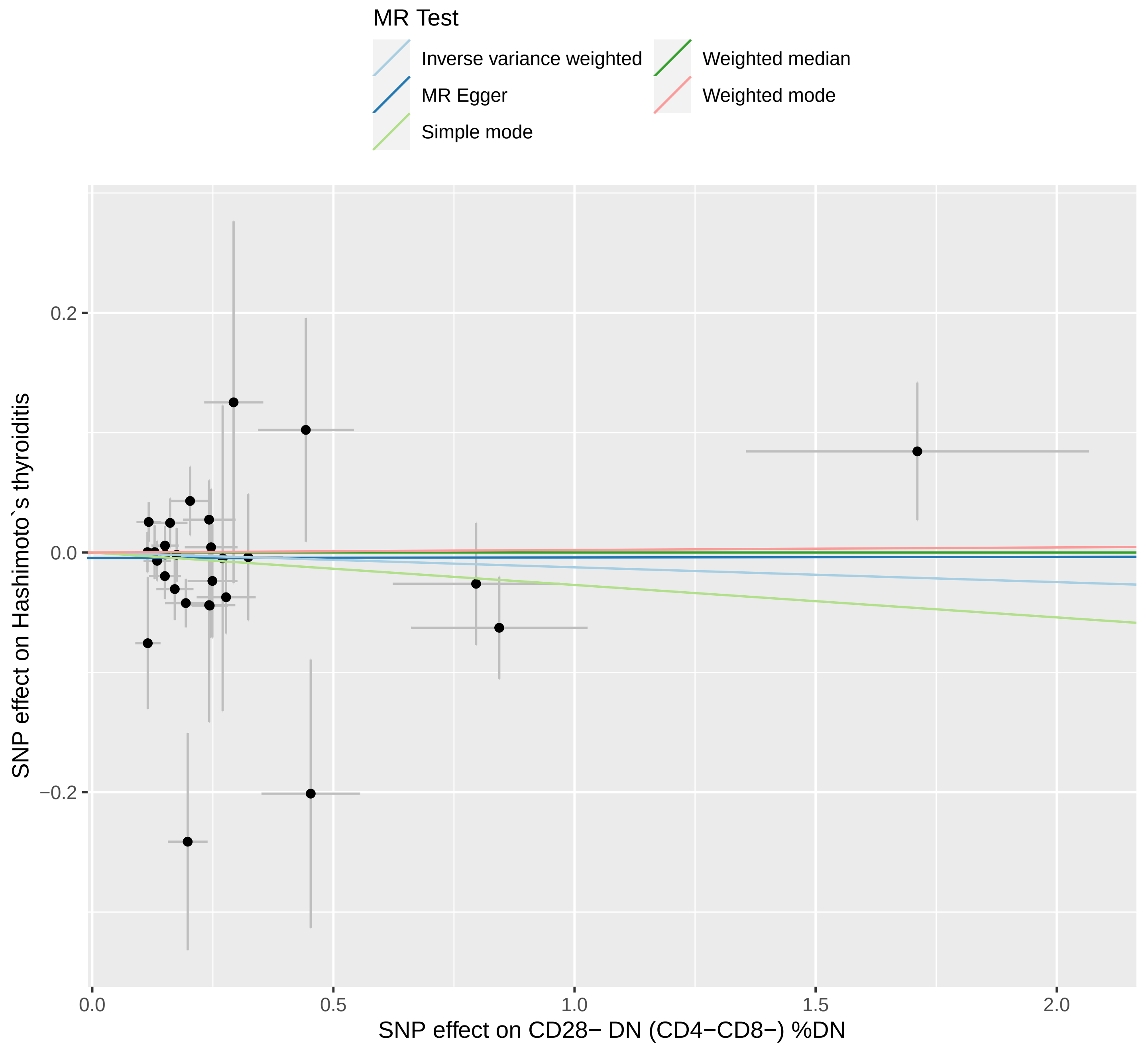

MR Test
Inverse variance weighted
MR Egger
Simple mode
Weighted median
Weighted mode
0.2
0.0
−0.2
0.0
0.5
1.0
1.5
2.0
SNP effect on Hashimoto`s thyroiditis
SNP effect on CD28− DN (CD4−CD8−) %DN

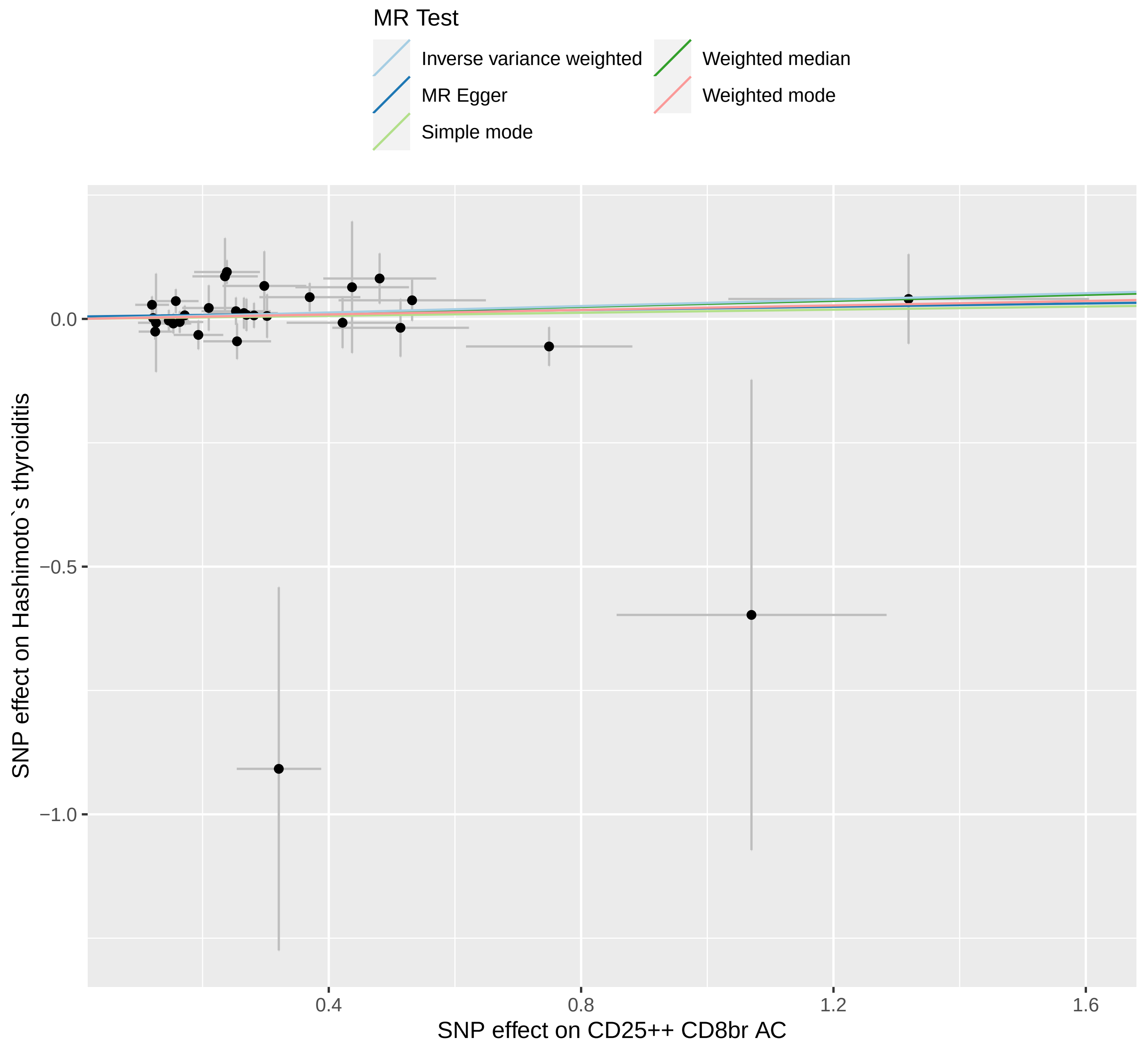

MR Test
Inverse variance weighted
MR Egger
Simple mode
Weighted median
Weighted mode
0.0
−0.5
−1.0
0.4
0.8
1.2
1.6
SNP effect on Hashimoto`s thyroiditis
SNP effect on CD25++ CD8br AC

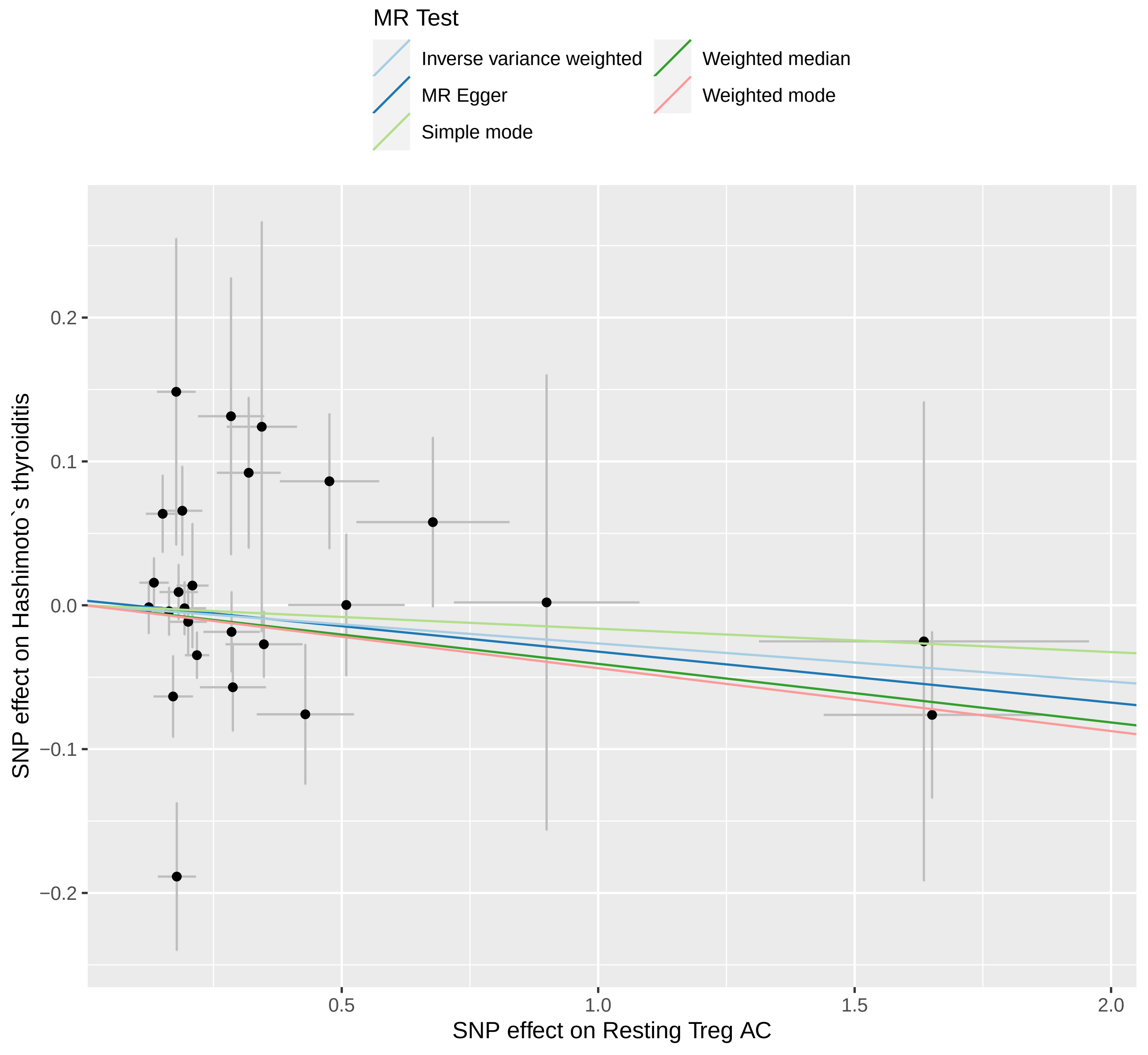

MR Test
Inverse variance weighted
MR Egger
Simple mode
Weighted median
Weighted mode
SNP effect on Hashimoto`s thyroiditis
SNP effect on Resting Treg AC
0.2
0.1
0.0
−0.1
−0.2
0.5
1.0
1.5
2.0

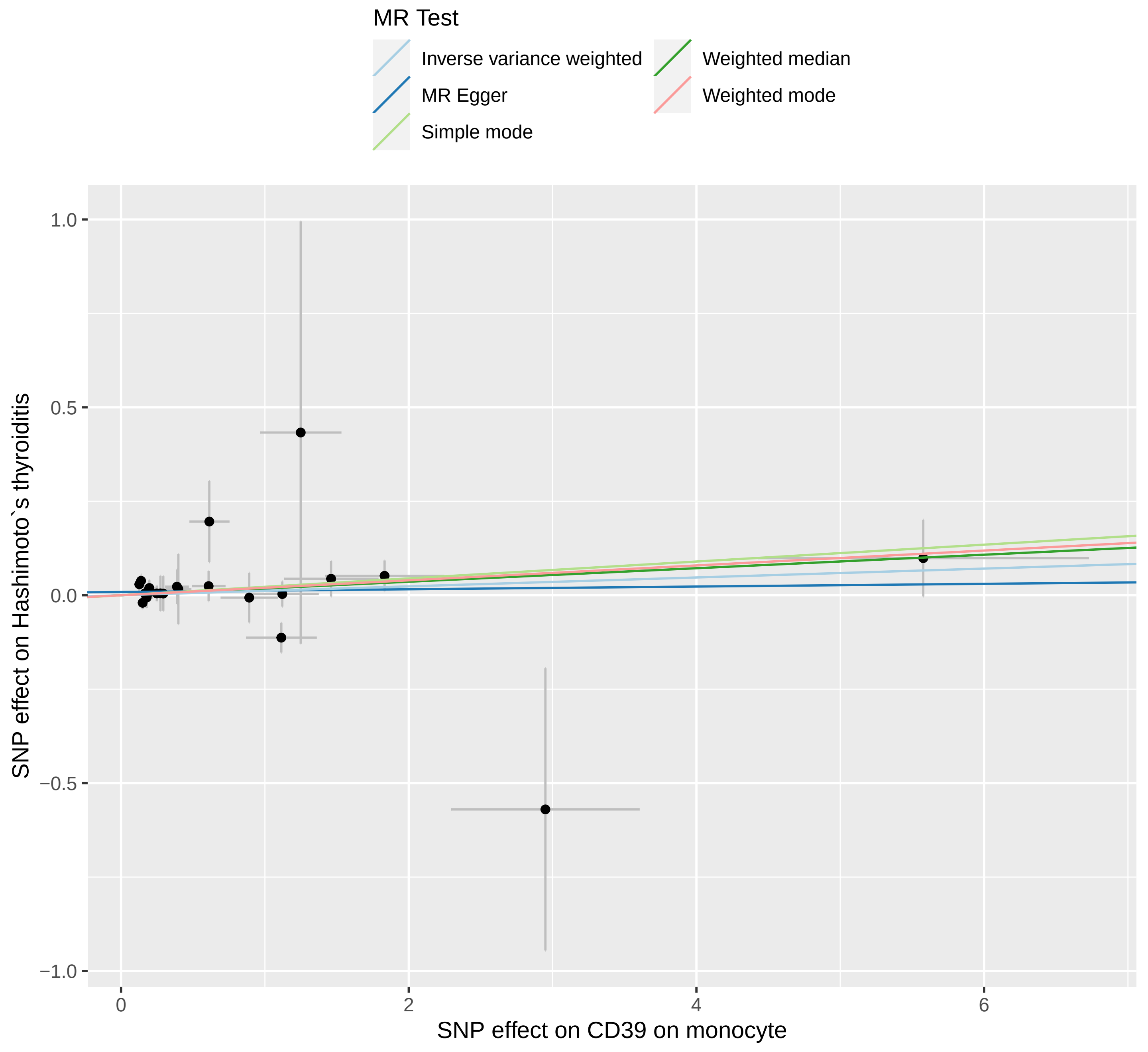
MR Test
Inverse variance weighted
MR Egger
Simple mode
Weighted median
Weighted mode
1.0
0.5
0.0
−0.5
−1.0
SNP effect on Hashimoto`s thyroiditis
0
2
4
6
SNP effect on CD39 on monocyte

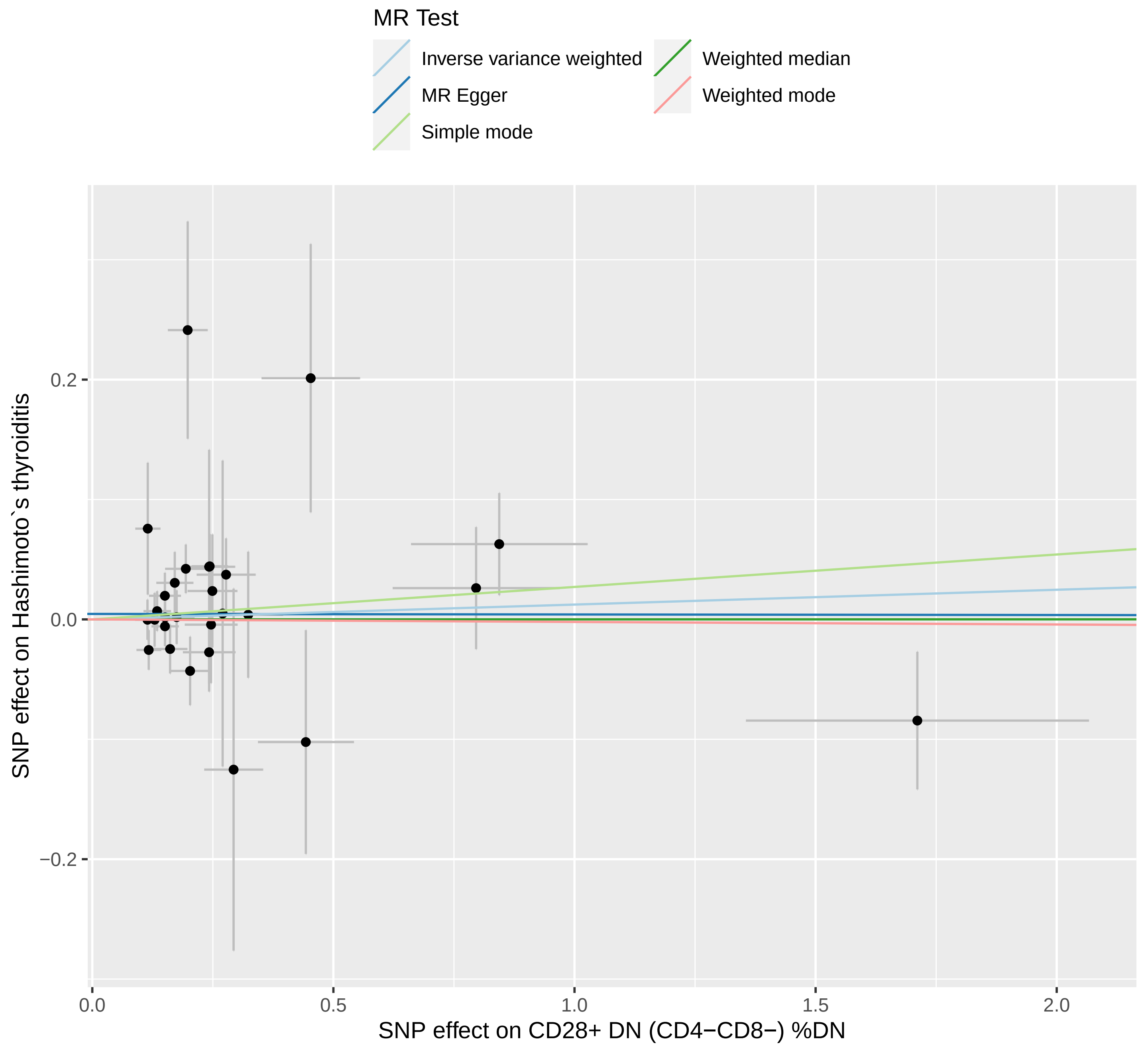

MR Test
Inverse variance weighted
MR Egger
Simple mode
Weighted median
Weighted mode
0.2
0.0
−0.2
0.0
0.5
1.0
1.5
2.0
SNP effect on Hashimoto`s thyroiditis
SNP effect on CD28+ DN (CD4−CD8−) %DN

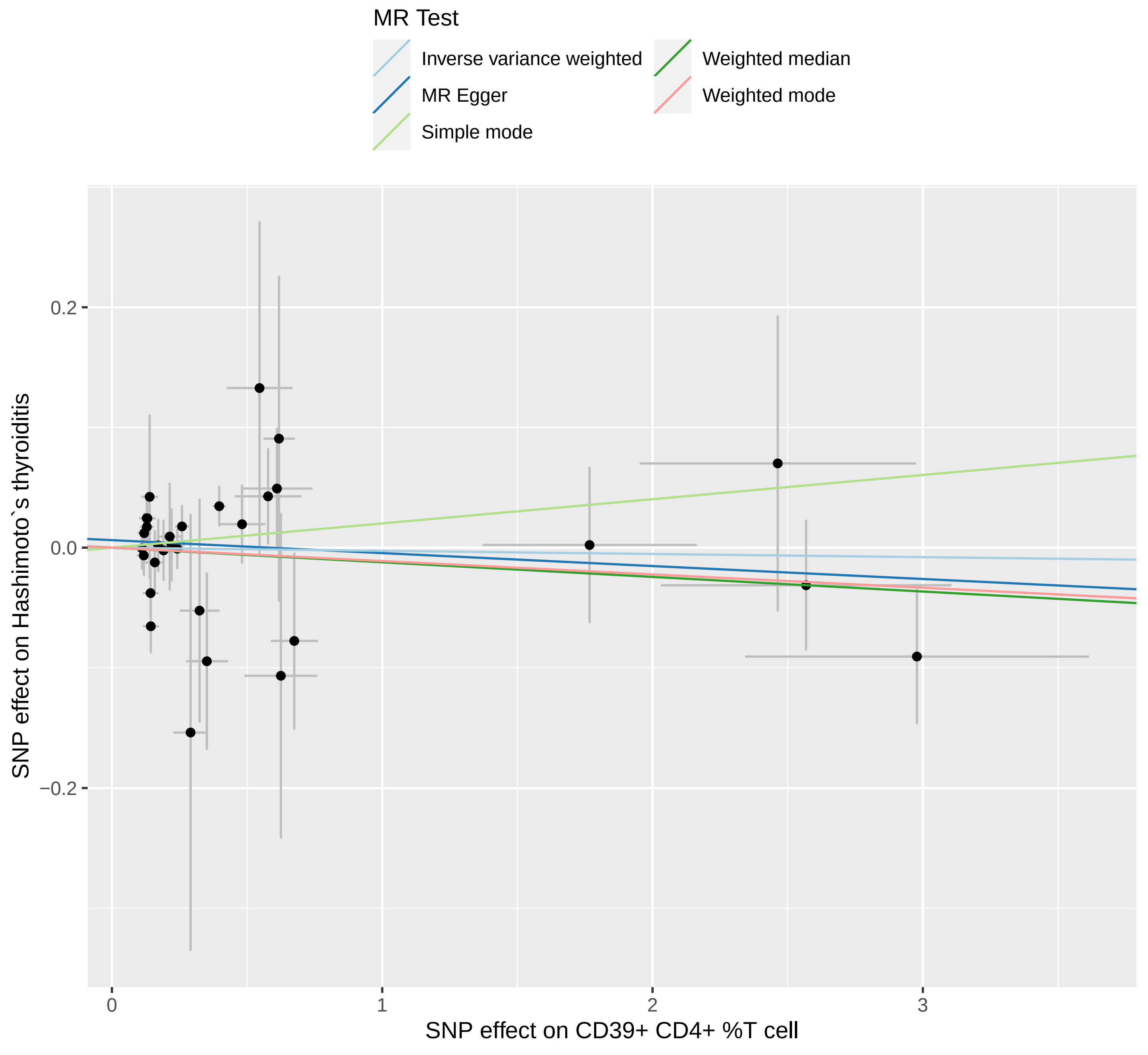

MR Test
Inverse variance weighted
MR Egger
Simple mode
Weighted median
Weighted mode
0.2
0.0
−0.2
0
1
2
3
SNP effect on Hashimoto`s thyroiditis
SNP effect on CD39+ CD4+ %T cell

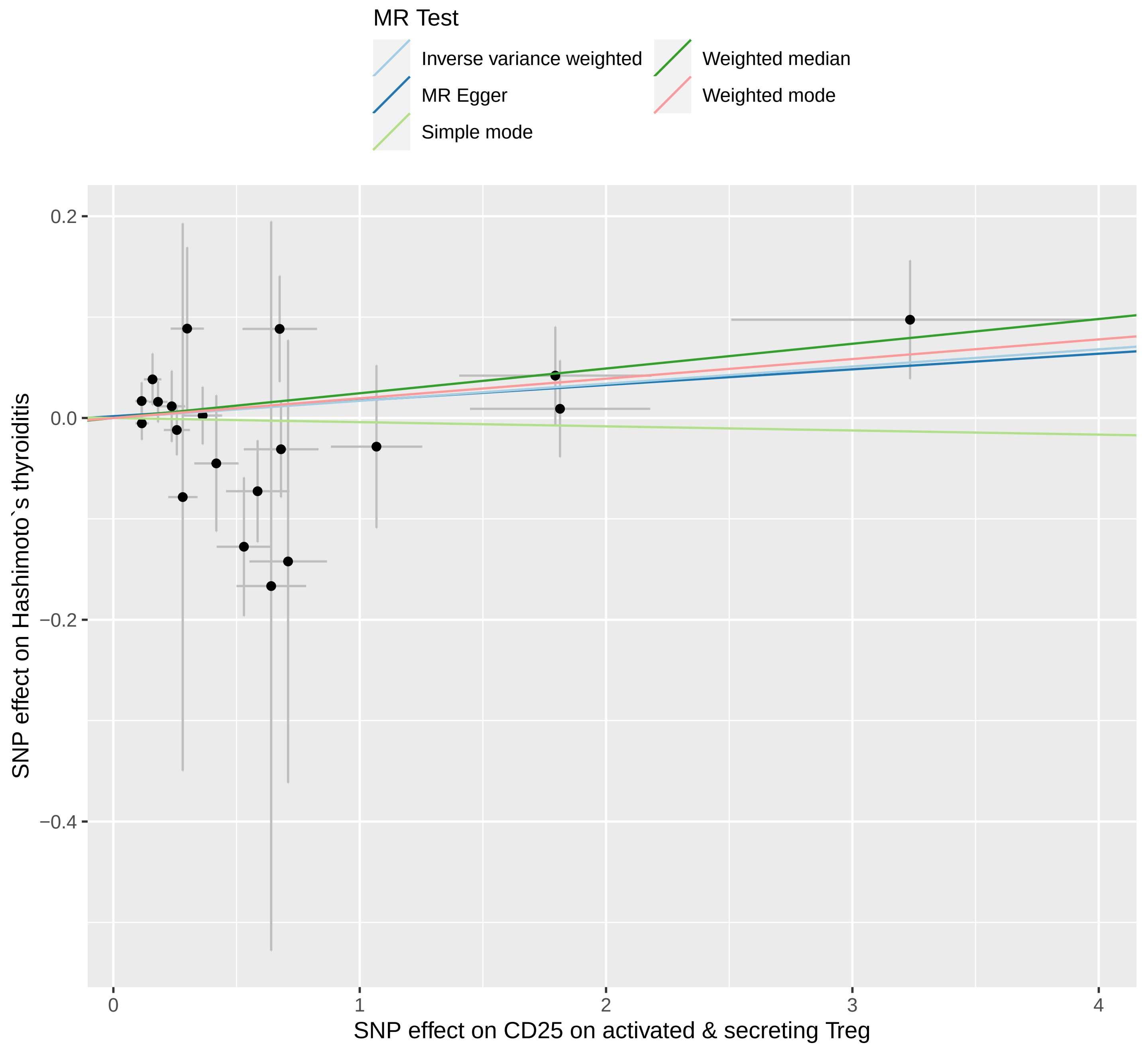
MR Test
Inverse variance weighted
MR Egger
Simple mode
Weighted median
Weighted mode
0.2
0.0
−0.2
−0.4
0
1
2
3
4
SNP effect on Hashimoto`s thyroiditis
SNP effect on CD25 on activated & secreting Treg

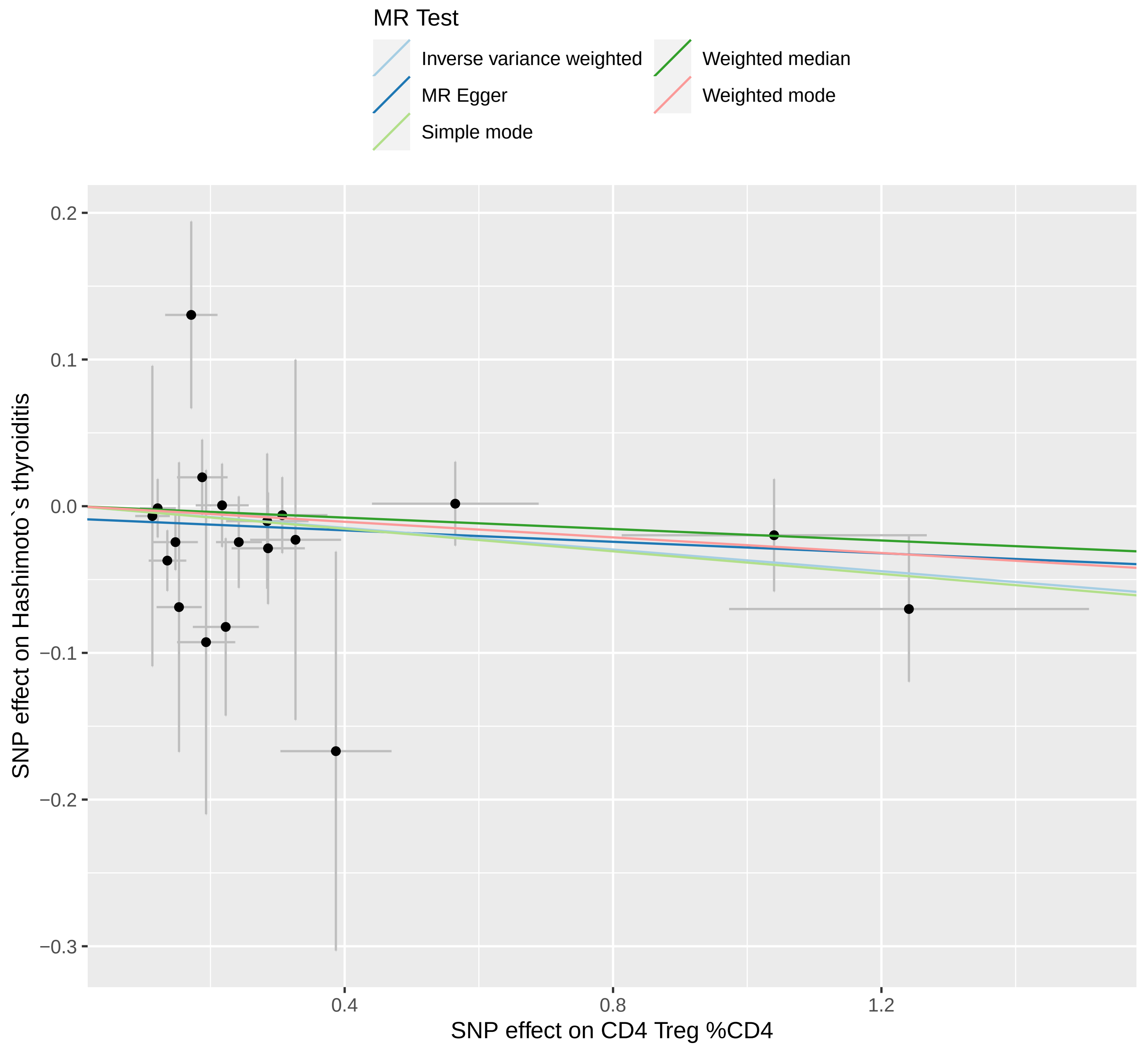
MR Test
Inverse variance weighted
MR Egger
Simple mode
Weighted median
Weighted mode
SNP effect on Hashimoto`s thyroiditis
SNP effect on CD4 Treg %CD4
0.2
0.1
0.0
−0.1
−0.2
−0.3
0.4
0.8
1.2

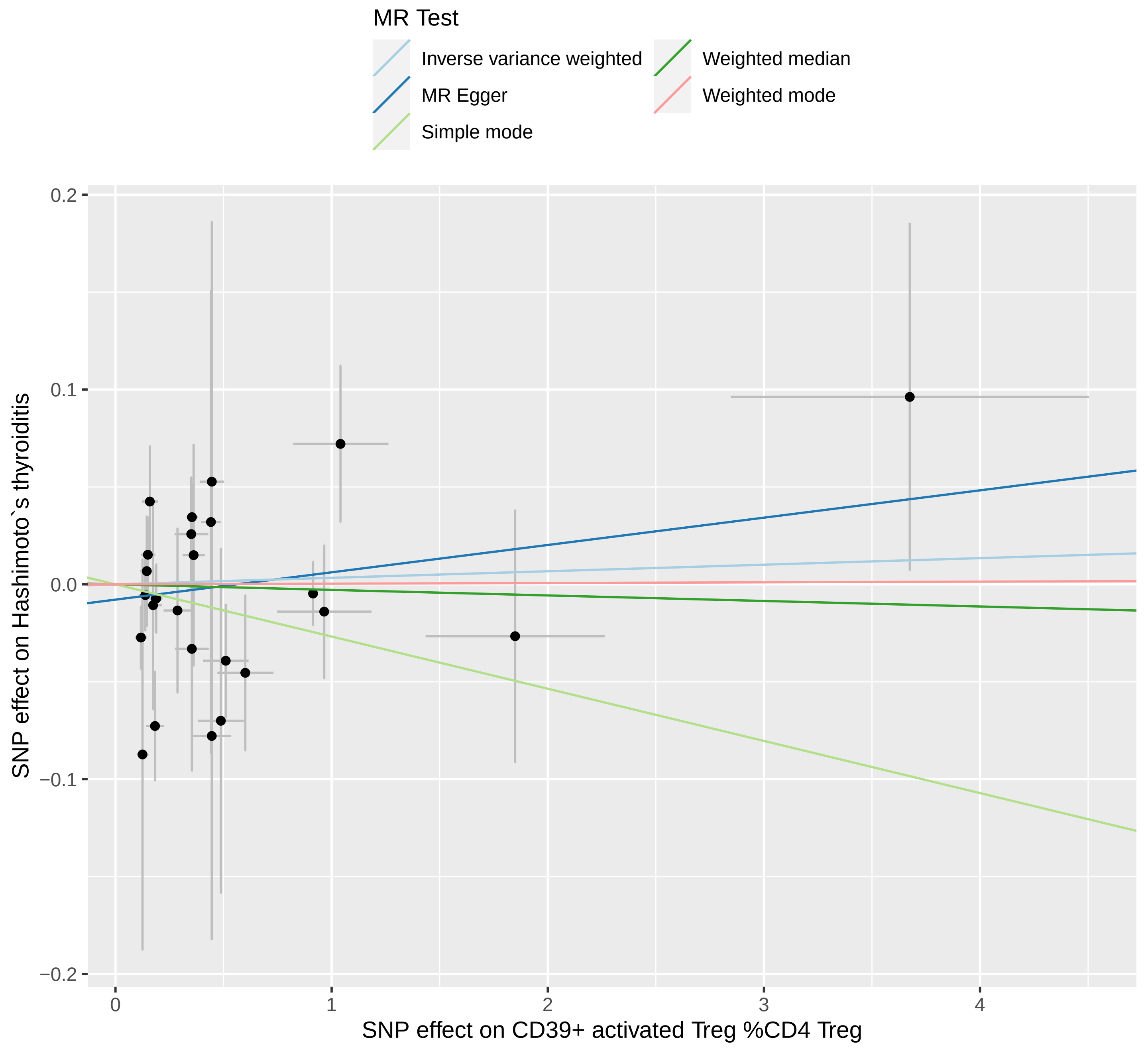

MR Test
Inverse variance weighted
MR Egger
Simple mode
Weighted median
Weighted mode
SNP effect on Hashimoto`s thyroiditis
SNP effect on CD39+ activated Treg %CD4 Treg
0.2
0.1
0.0
−0.1
−0.2
0
1
2
3
4

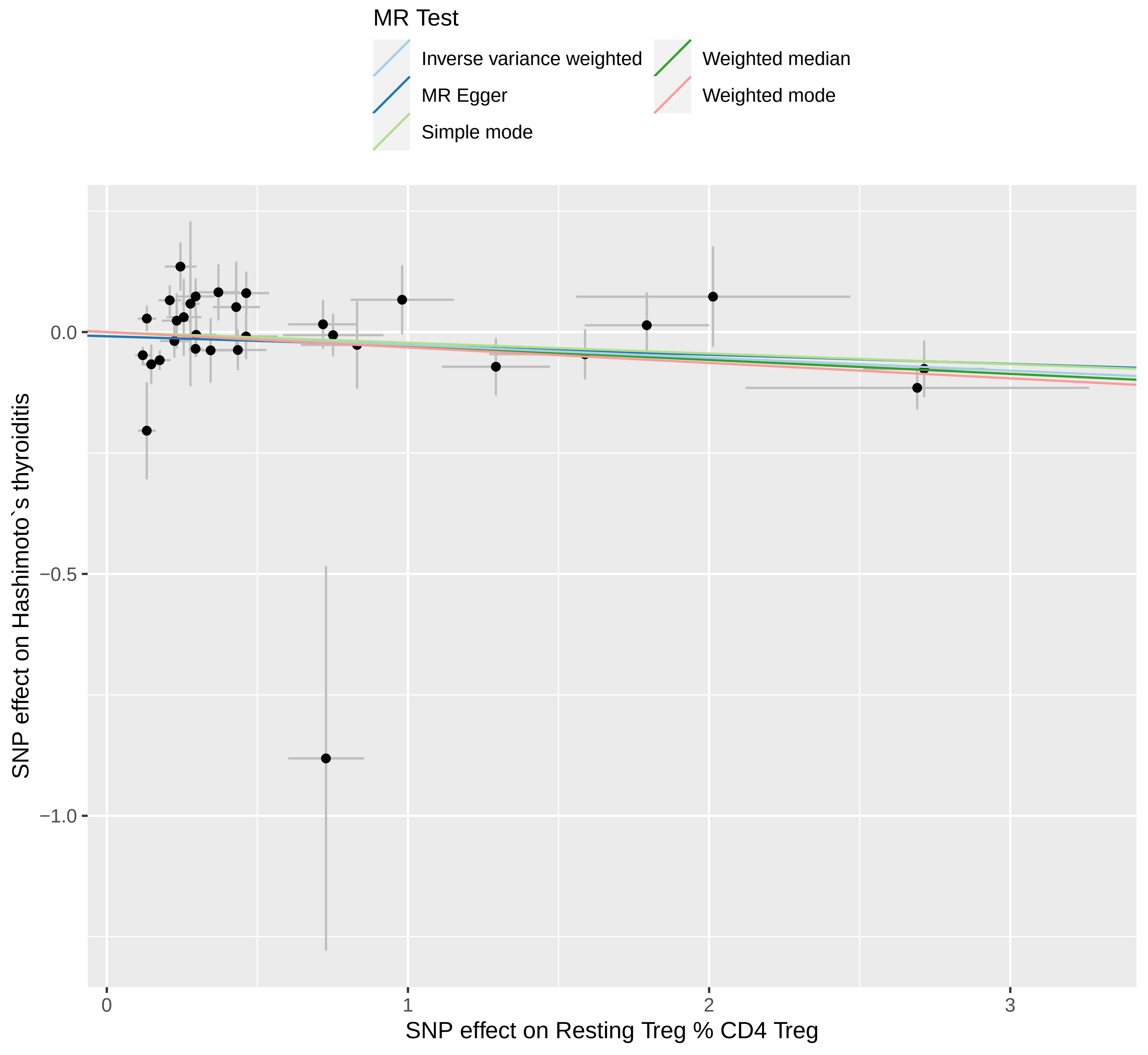

MR Test
Inverse variance weighted
MR Egger
Simple mode
Weighted median
Weighted mode
0.0
−0.5
−1.0
0
1
2
3
SNP effect on Hashimoto`s thyroiditis
SNP effect on Resting Treg % CD4 Treg

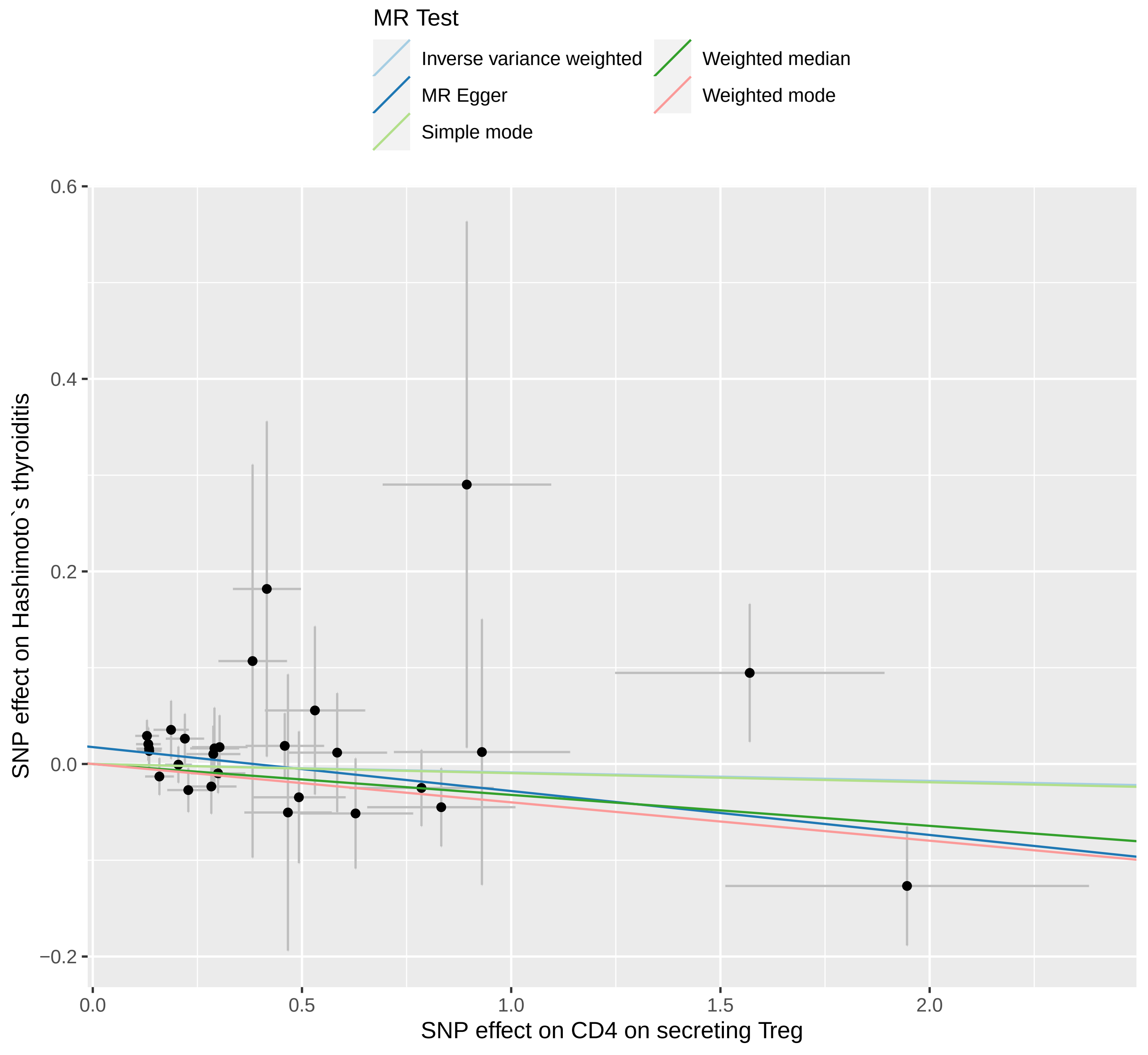
MR Test
Inverse variance weighted
MR Egger
Simple mode
Weighted median
Weighted mode
0.6
0.4
0.2
0.0
−0.2
0.0
0.5
1.0
1.5
2.0
SNP effect on Hashimoto`s thyroiditis
SNP effect on CD4 on secreting Treg

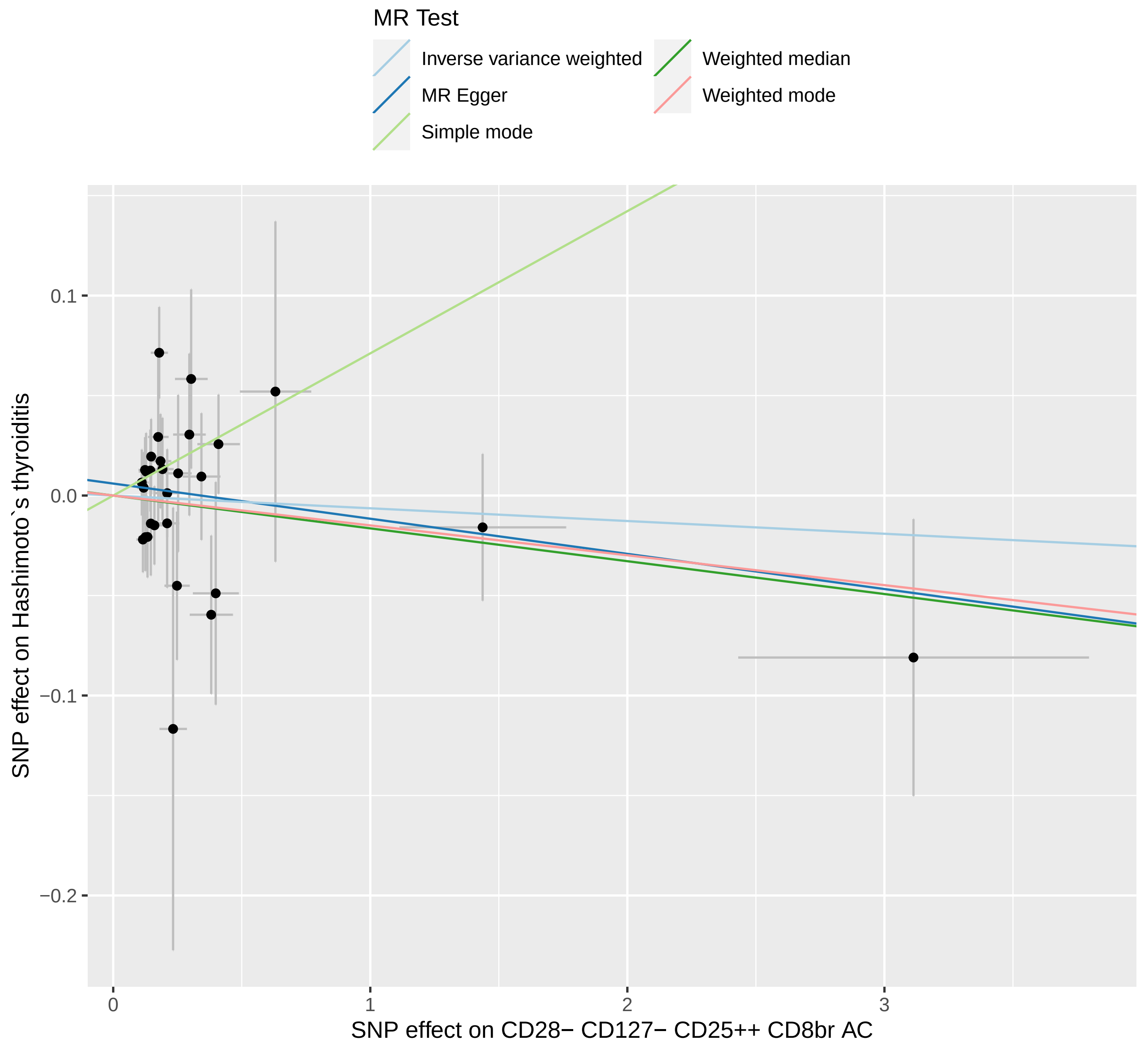

MR Test
Inverse variance weighted
MR Egger
Simple mode
Weighted median
Weighted mode
0.1
0.0
−0.1
−0.2
0
1
2
3
SNP effect on Hashimoto`s thyroiditis
SNP effect on CD28− CD127− CD25++ CD8br AC

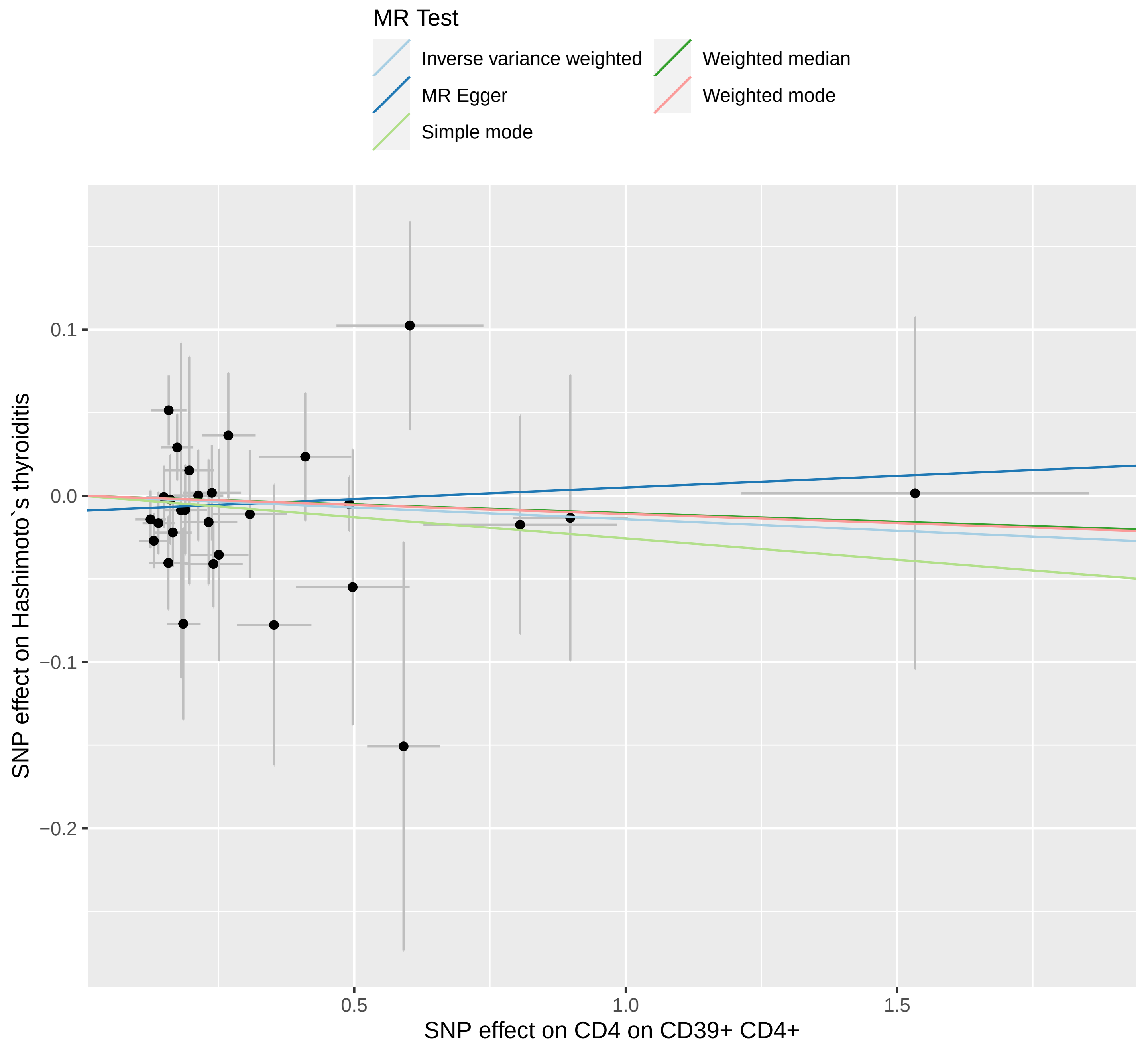

MR Test
Inverse variance weighted
MR Egger
Simple mode
Weighted median
Weighted mode
0.1
0.0
−0.1
−0.2
SNP effect on Hashimoto`s thyroiditis
0.5
1.0
1.5
SNP effect on CD4 on CD39+ CD4+

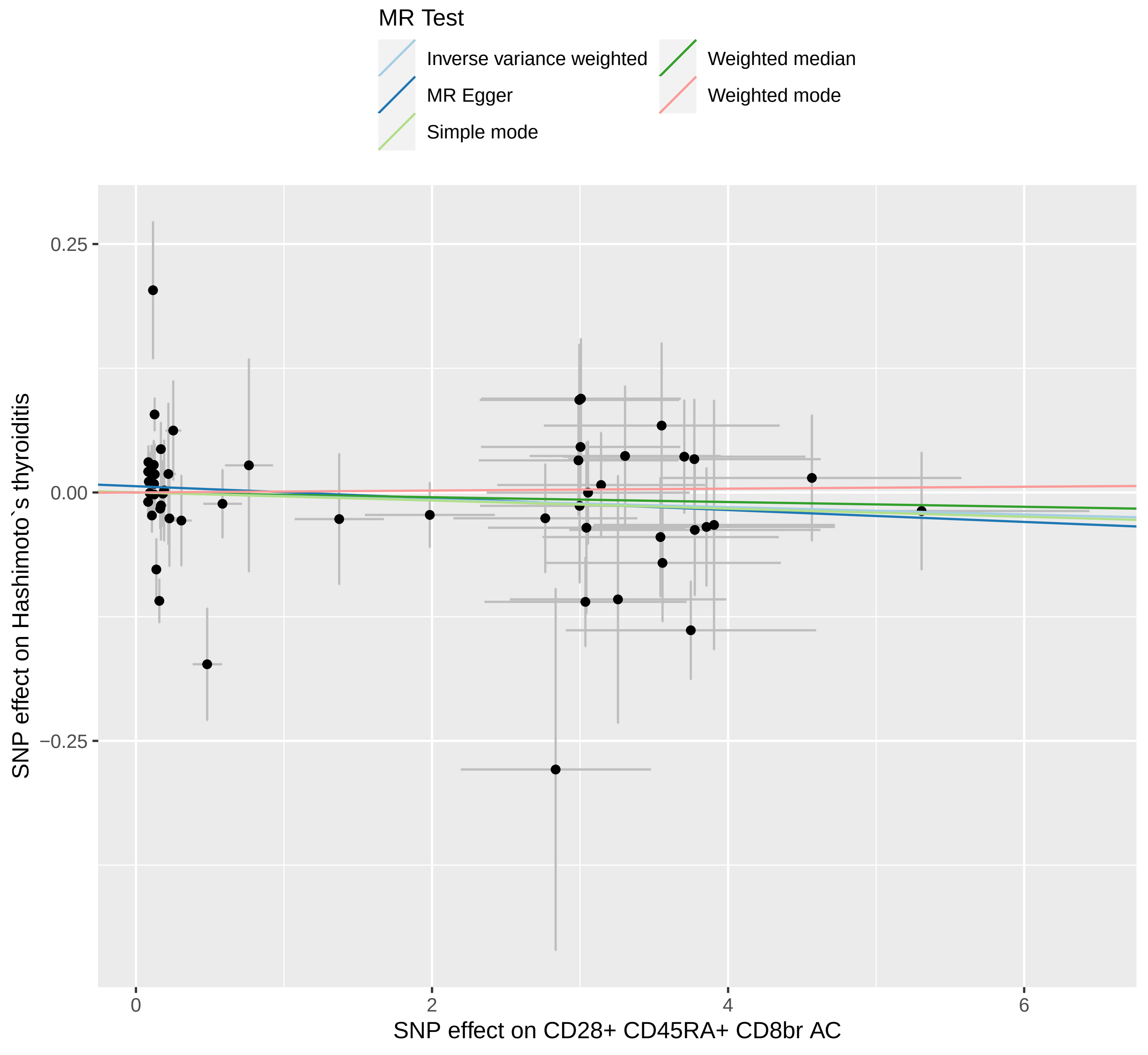
MR Test
Inverse variance weighted
MR Egger
Simple mode
Weighted median
Weighted mode
0.25
0.00
−0.25
0
2
4
6
SNP effect on Hashimoto`s thyroiditis
SNP effect on CD28+ CD45RA+ CD8br AC

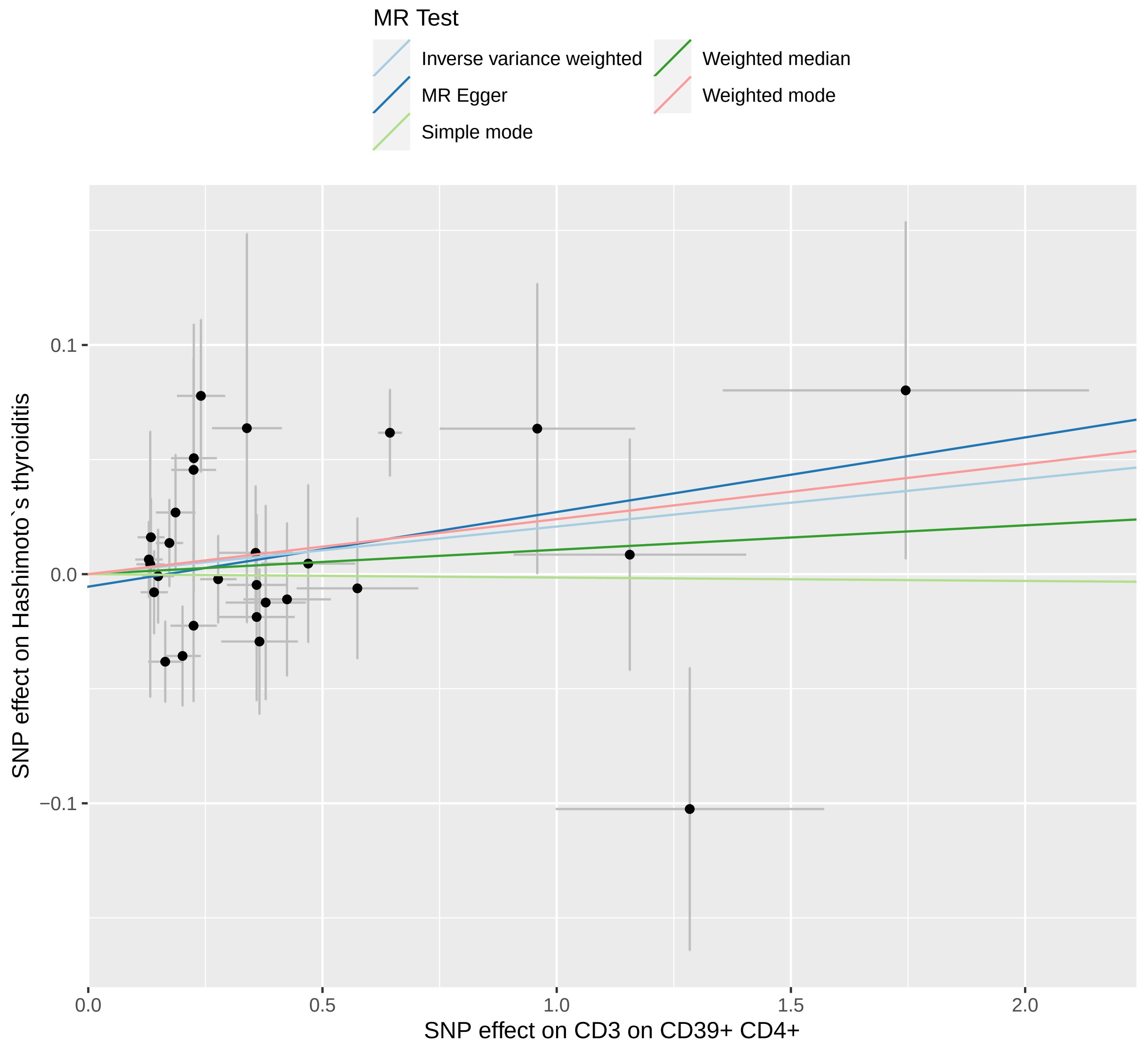
MR Test
Inverse variance weighted
MR Egger
Simple mode
Weighted median
Weighted mode
0.1
0.0
−0.1
0.0
0.5
1.0
1.5
2.0
SNP effect on Hashimoto`s thyroiditis
SNP effect on CD3 on CD39+ CD4+

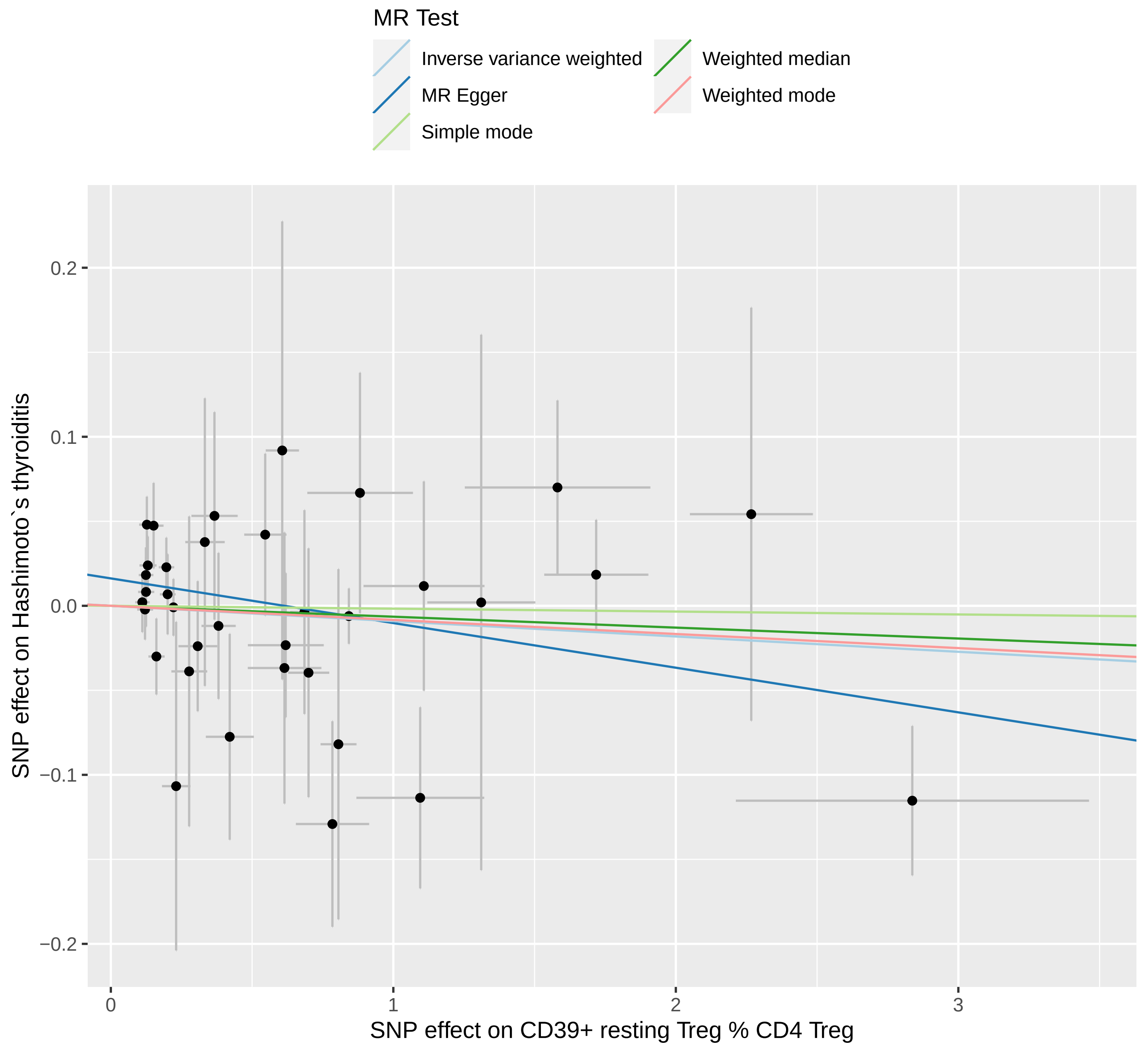
MR Test
Inverse variance weighted
Weighted median
MR Egger
Weighted mode
Simple mode
0.2
0.1
0.0
−0.1
−0.2
SNP effect on Hashimoto`s thyroiditis
0
1
2
3
SNP effect on CD39+ resting Treg % CD4 Treg

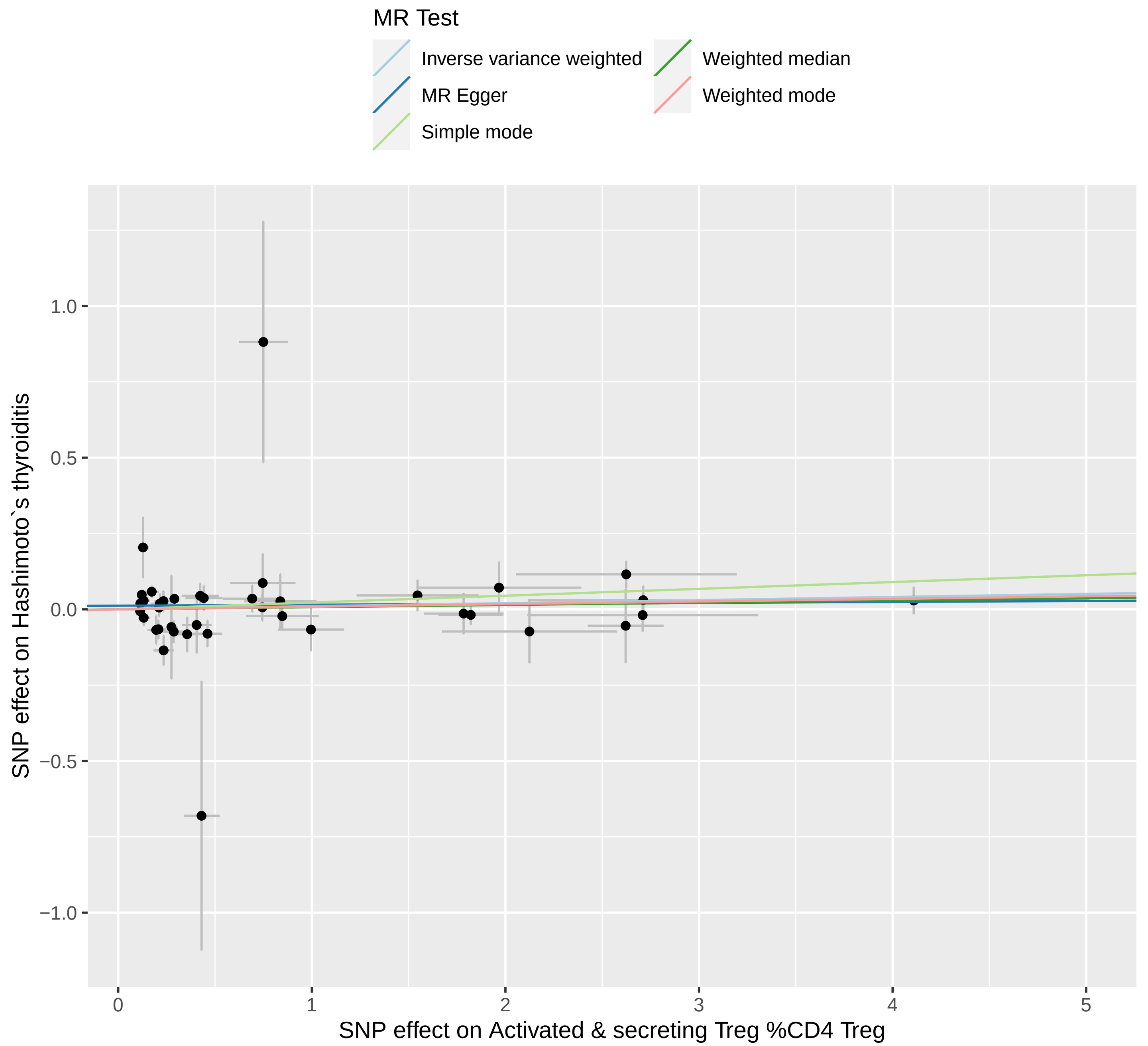
MR Test
Inverse variance weighted
MR Egger
Simple mode
Weighted median
Weighted mode
SNP effect on Hashimoto`s thyroiditis
1.0
0.5
0.0
−0.5
−1.0
0
1
2
3
4
5
SNP effect on Activated & secreting Treg %CD4 Treg

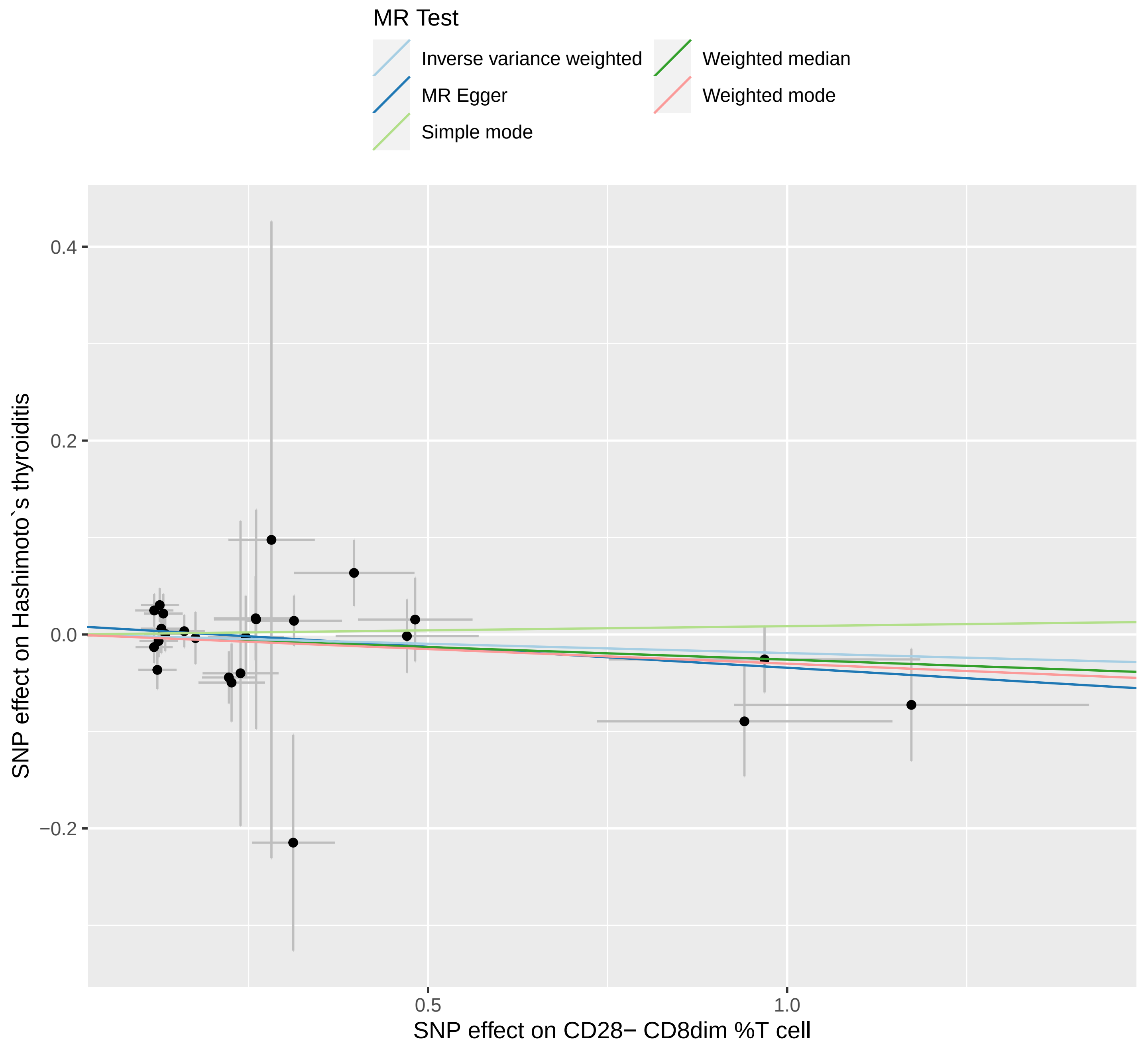
MR Test
Inverse variance weighted
MR Egger
Simple mode
Weighted median
Weighted mode
SNP effect on Hashimoto`s thyroiditis
0.4
0.2
0.0
−0.2
0.5
1.0
SNP effect on CD28− CD8dim %T cell

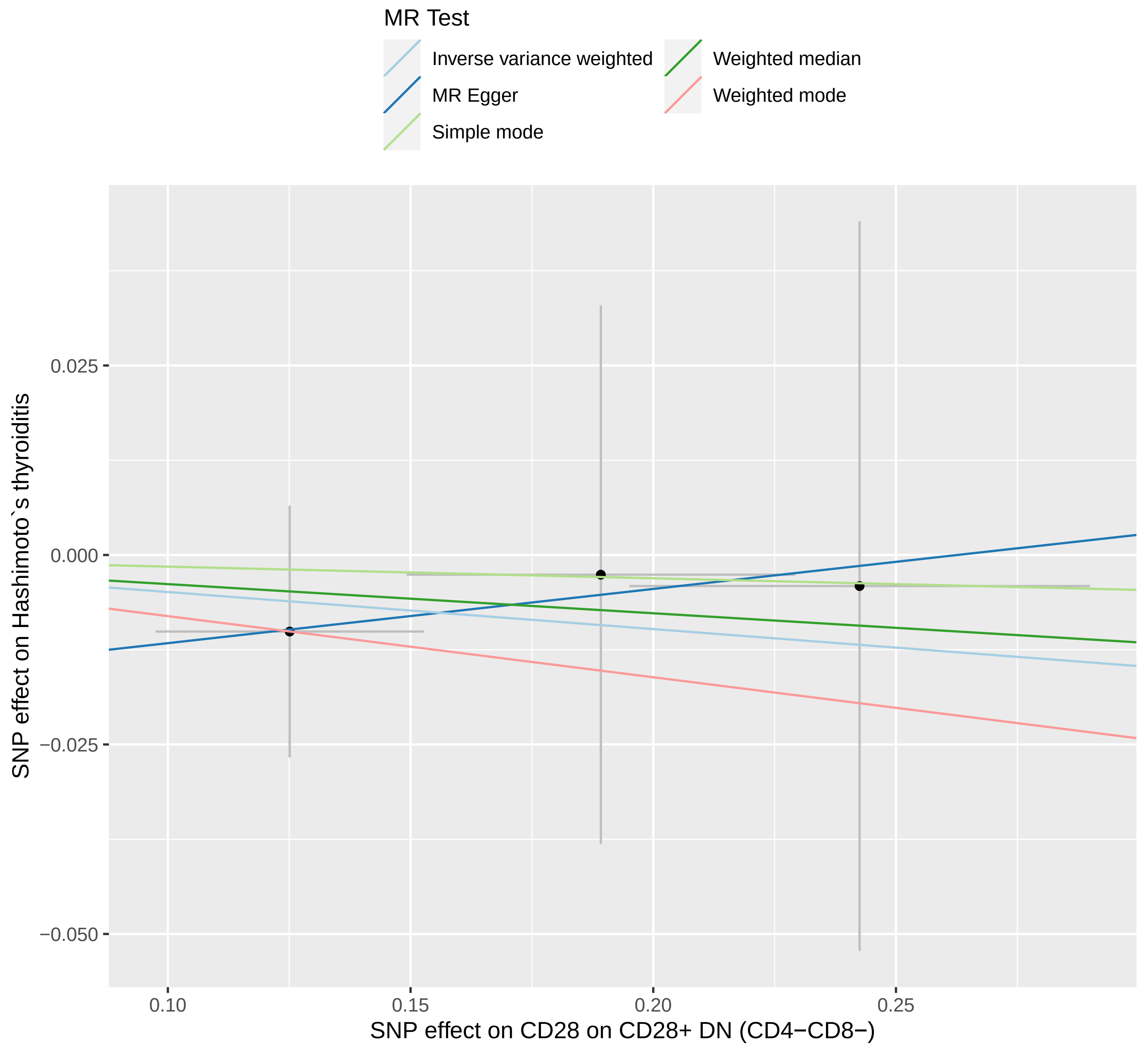

MR Test
Inverse variance weighted
MR Egger
Simple mode
Weighted median
Weighted mode
0.025
0.000
−0.025
−0.050
0.10
0.15
0.20
0.25
SNP effect on Hashimoto`s thyroiditis
SNP effect on CD28 on CD28+ DN (CD4−CD8−)

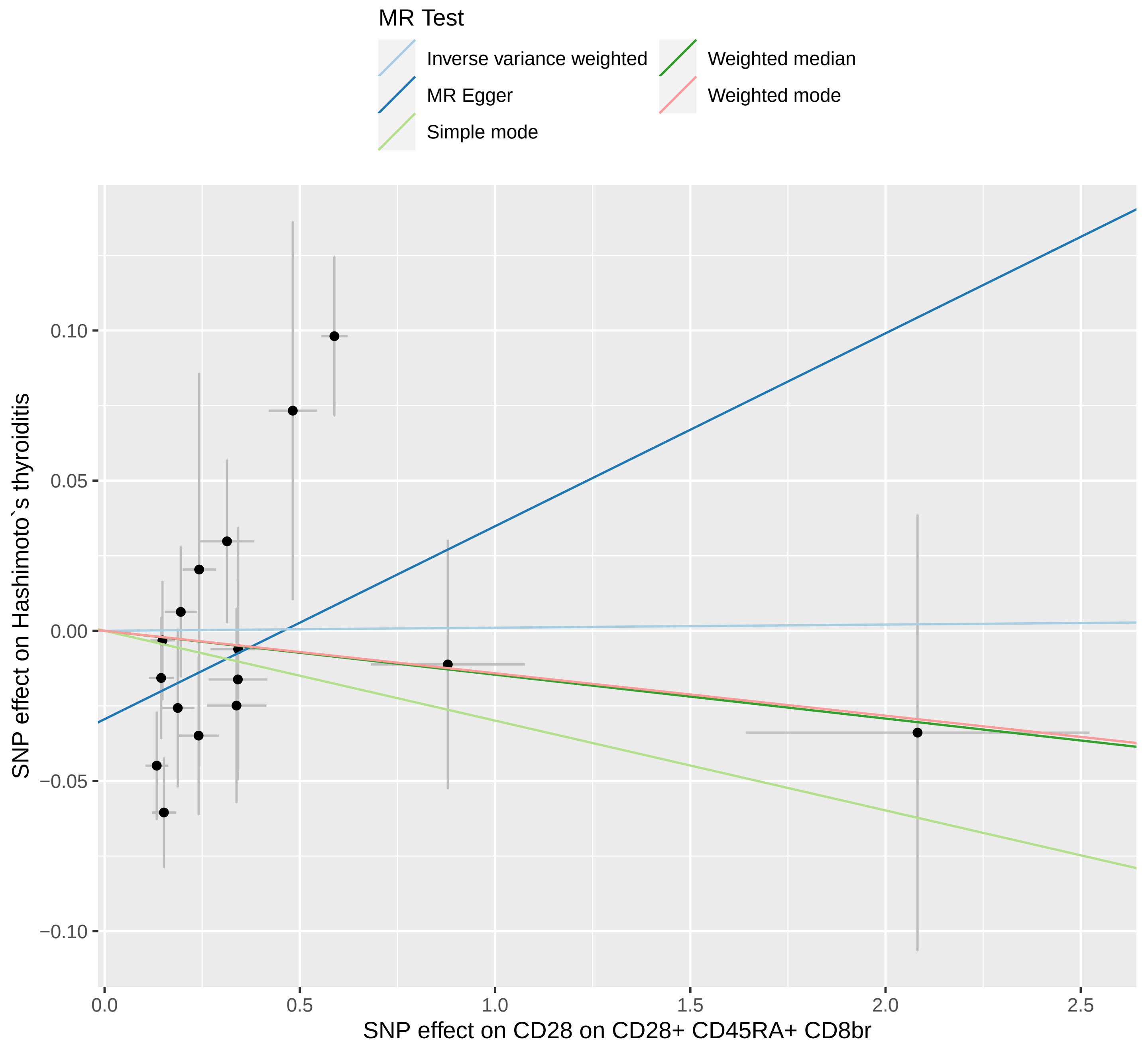
MR Test
Inverse variance weighted
MR Egger
Simple mode
Weighted median
Weighted mode
0.10
0.05
0.00
−0.05
−0.10
SNP effect on Hashimoto`s thyroiditis
0.0
0.5
1.0
1.5
2.0
2.5
SNP effect on CD28 on CD28+ CD45RA+ CD8br

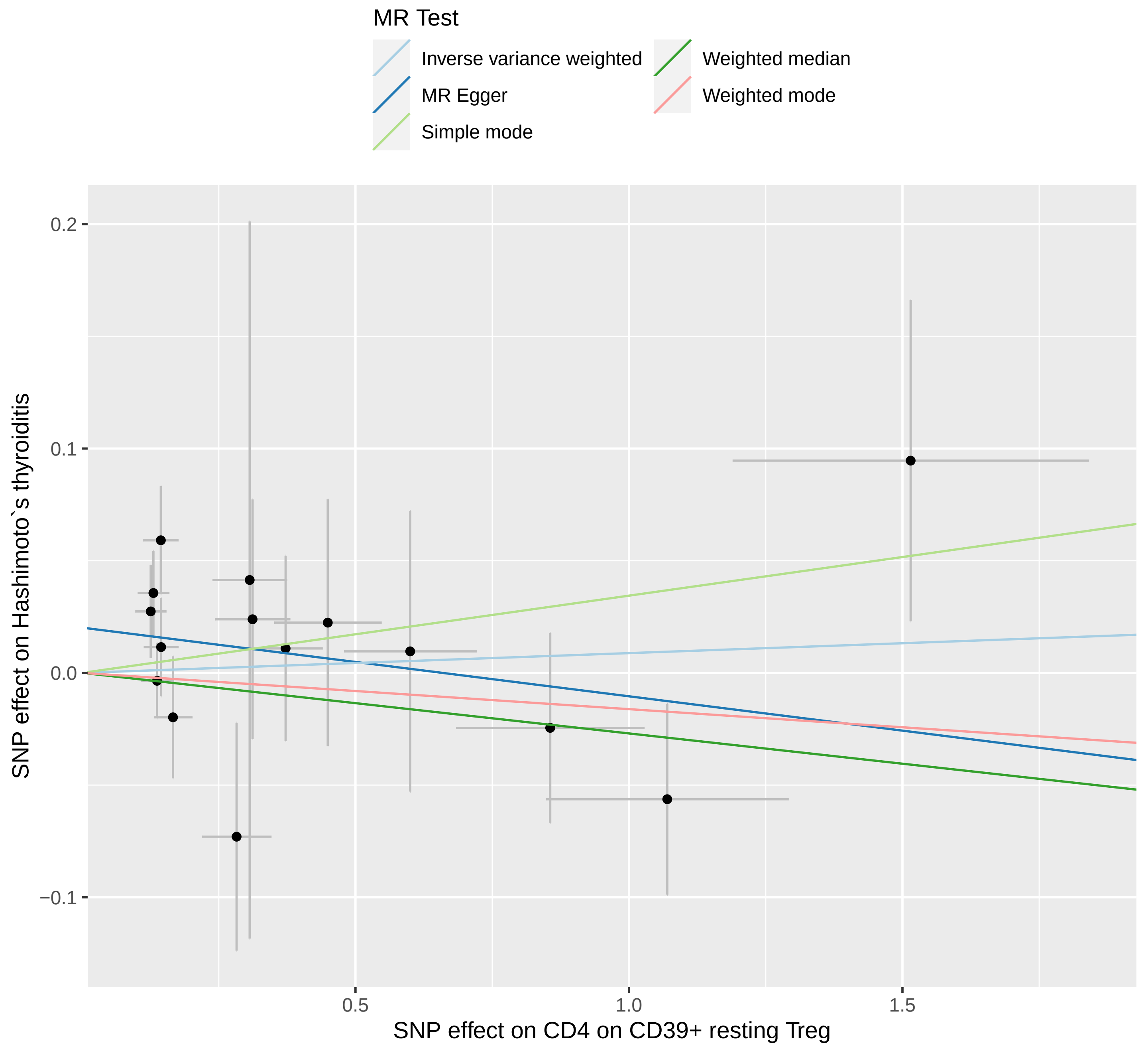

MR Test
Inverse variance weighted
MR Egger
Simple mode
Weighted median
Weighted mode
0.2
0.1
0.0
−0.1
0.5
1.0
1.5
SNP effect on Hashimoto`s thyroiditis
SNP effect on CD4 on CD39+ resting Treg

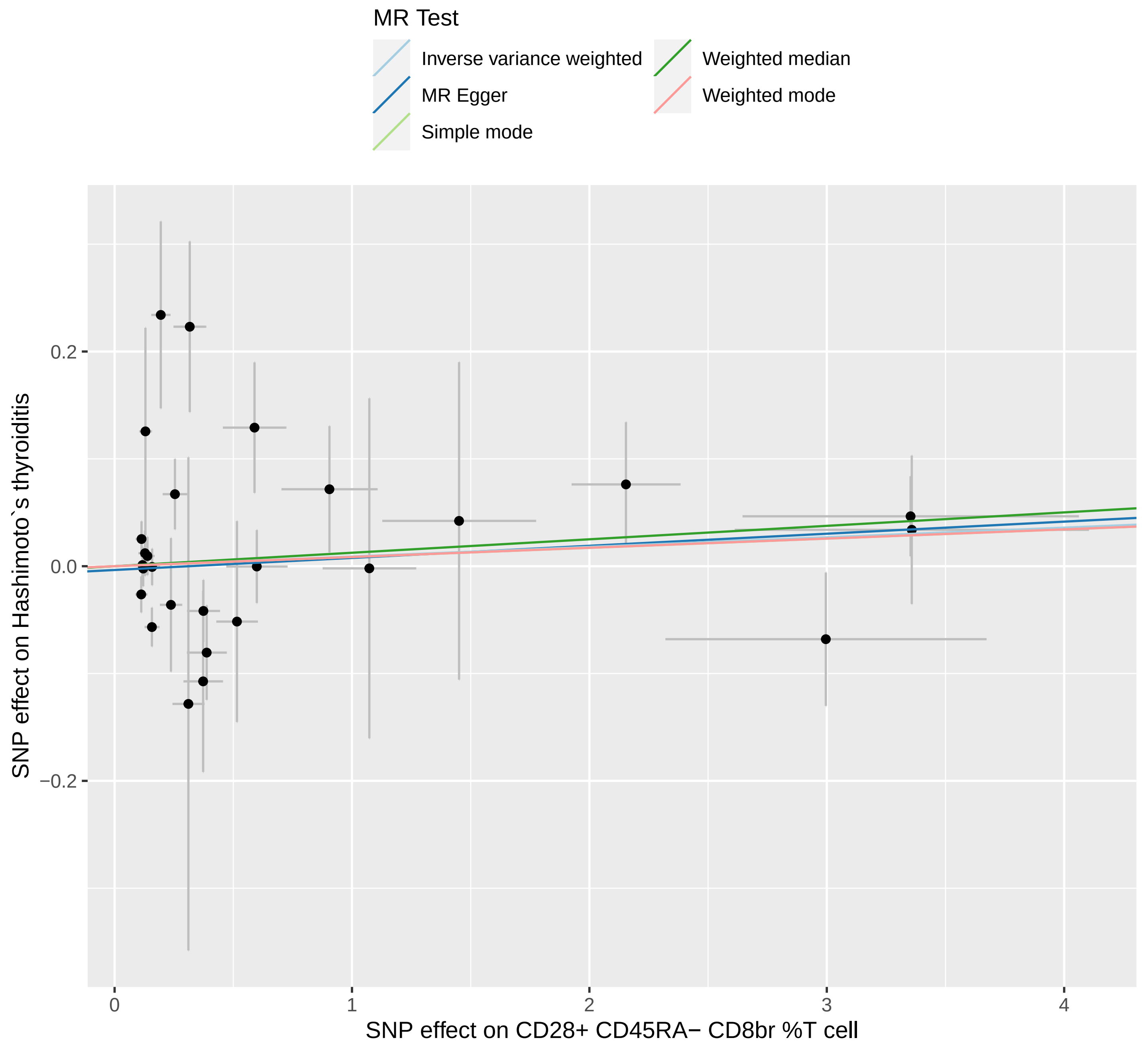

MR Test
Inverse variance weighted
MR Egger
Simple mode
Weighted median
Weighted mode
0.2
0.0
−0.2
0
1
2
3
4
SNP effect on Hashimoto`s thyroiditis
SNP effect on CD28+ CD45RA− CD8br %T cell

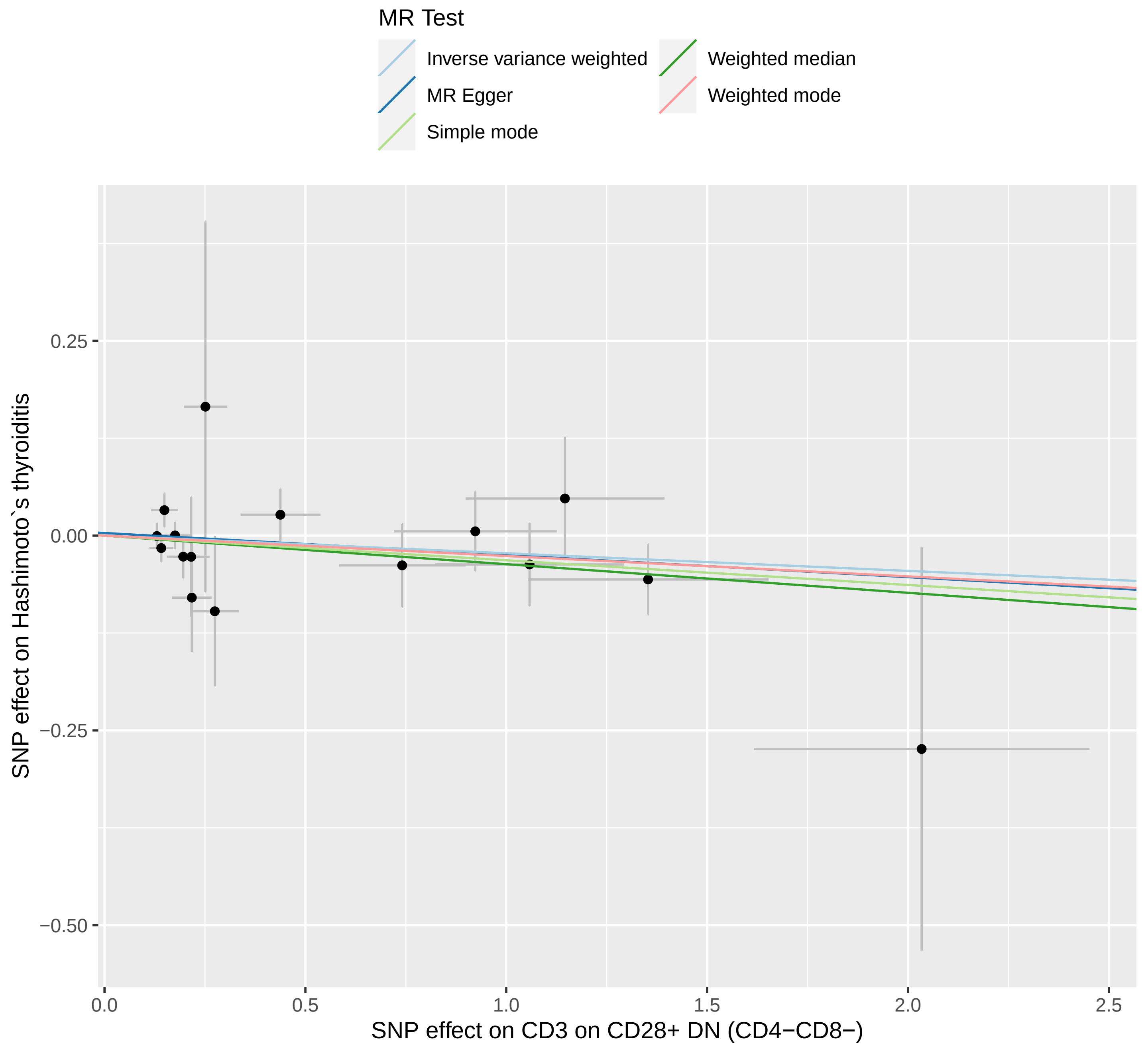
MR Test
Inverse variance weighted
MR Egger
Simple mode
Weighted median
Weighted mode
SNP effect on Hashimoto`s thyroiditis
0.25
0.00
−0.25
−0.50
0.0
0.5
1.0
1.5
2.0
2.5
SNP effect on CD3 on CD28+ DN (CD4−CD8−)

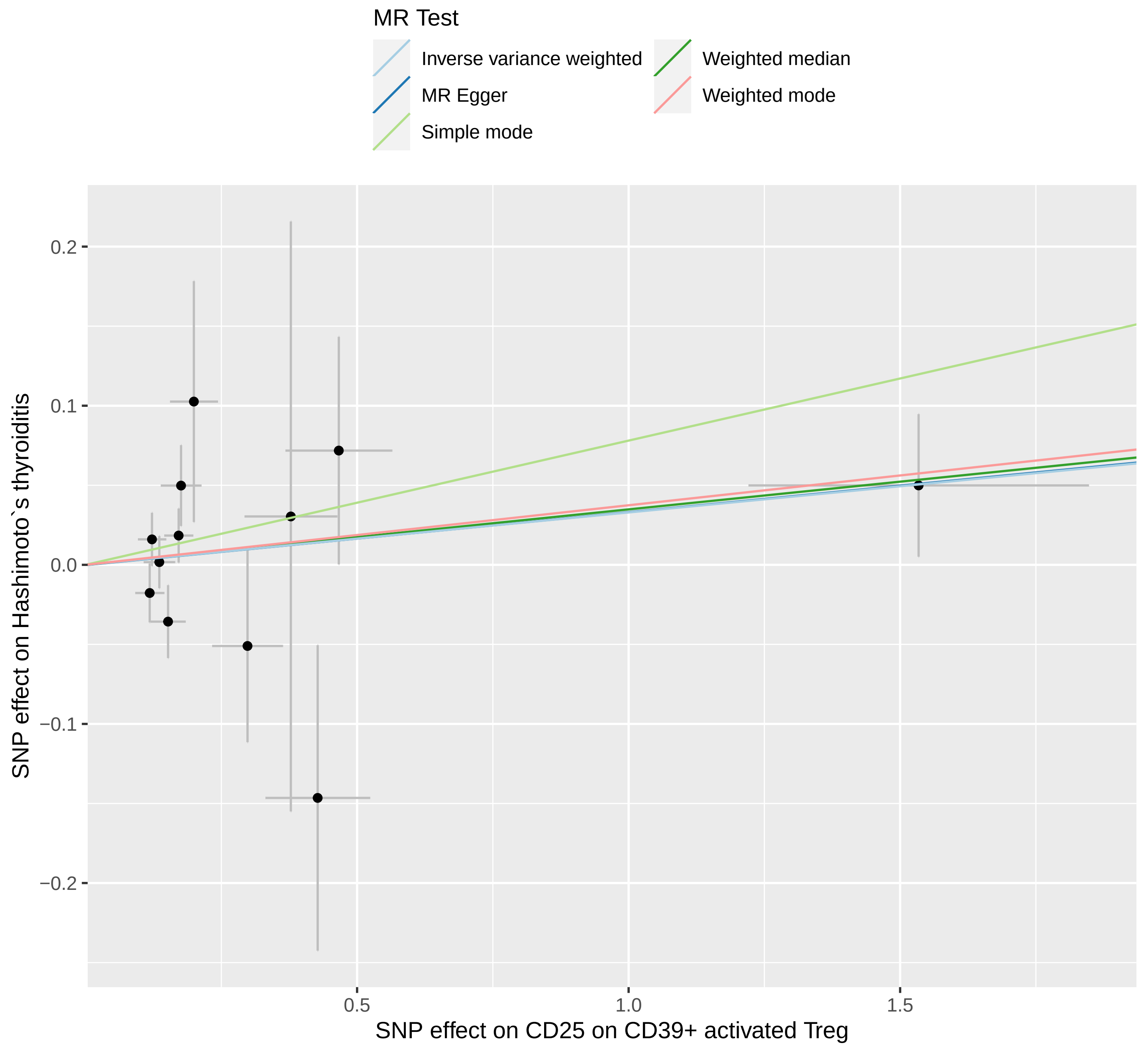

MR Test
Inverse variance weighted
MR Egger
Simple mode
Weighted median
Weighted mode
0.2
0.1
0.0
−0.1
−0.2
0.5
1.0
1.5
SNP effect on Hashimoto`s thyroiditis
SNP effect on CD25 on CD39+ activated Treg

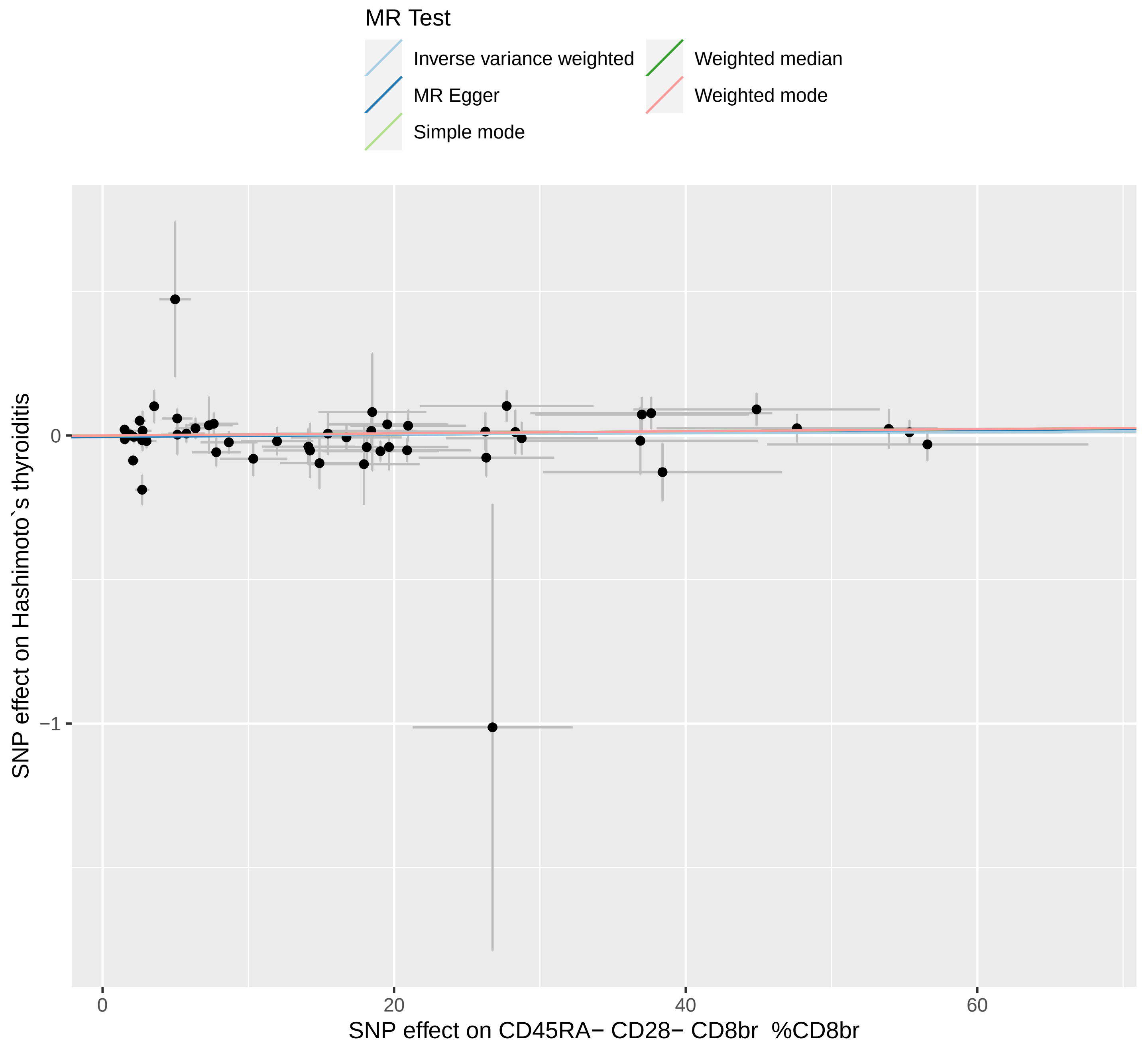
MR Test
Inverse variance weighted
MR Egger
Simple mode
Weighted median
Weighted mode
SNP effect on Hashimoto`s thyroiditis
0
−1
0
20
40
60
SNP effect on CD45RA− CD28− CD8br %CD8br

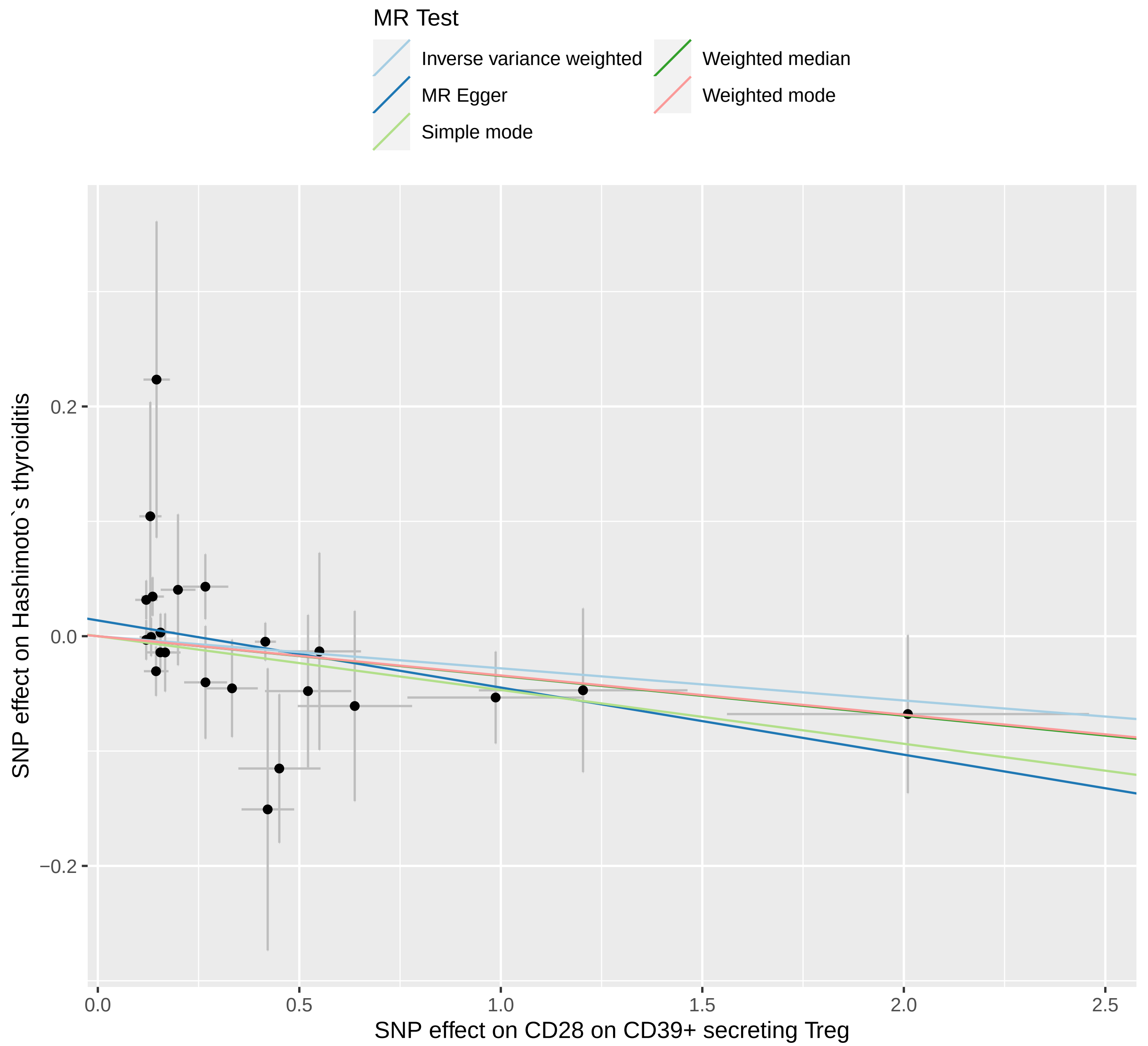

MR Test
Inverse variance weighted
MR Egger
Simple mode
Weighted median
Weighted mode
SNP effect on Hashimoto`s thyroiditis
0.2
0.0
−0.2
0.0
0.5
1.0
1.5
2.0
2.5
SNP effect on CD28 on CD39+ secreting Treg

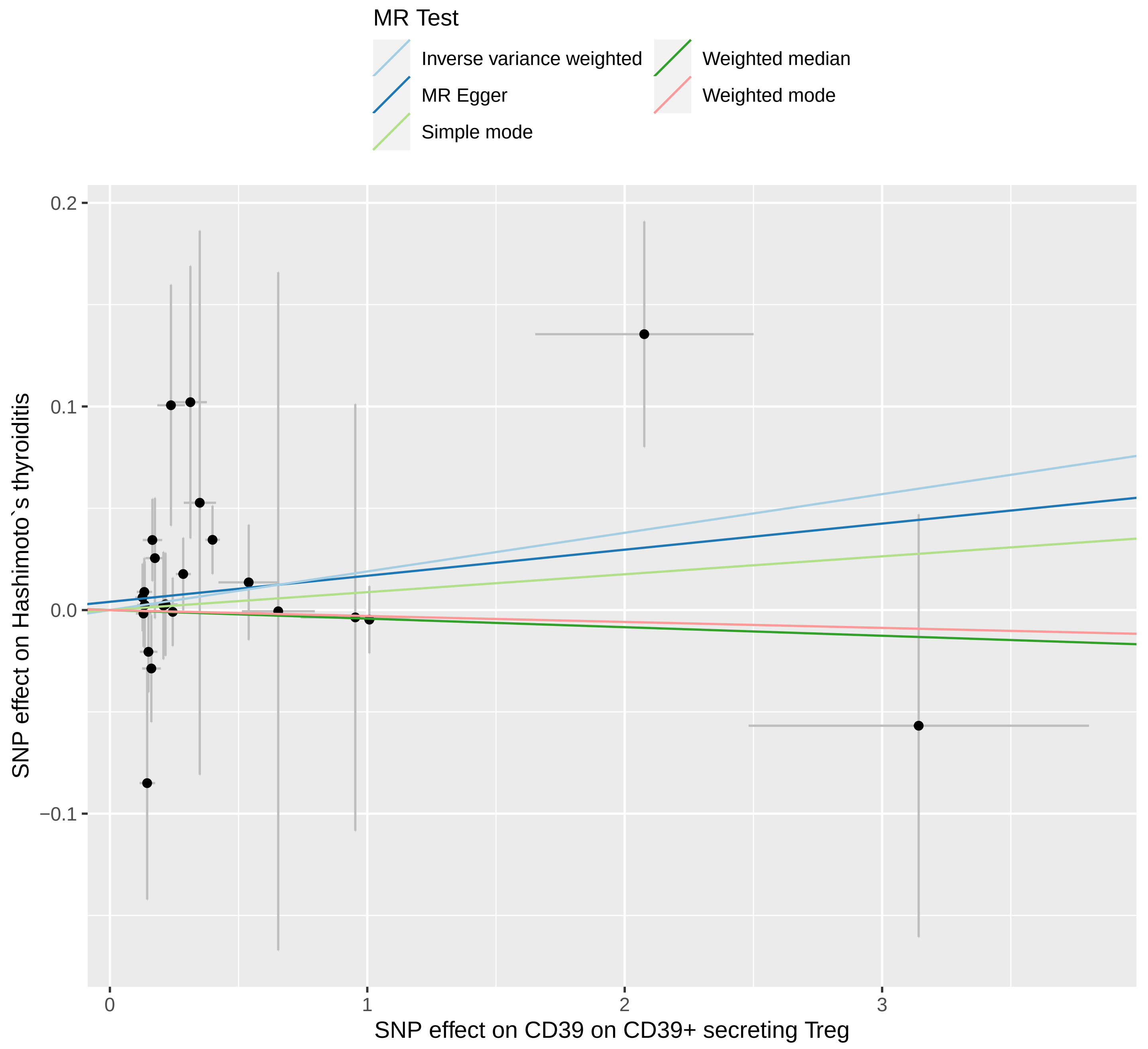

MR Test
Inverse variance weighted
MR Egger
Simple mode
Weighted median
Weighted mode
0.2
0.1
0.0
−0.1
0
1
2
3
SNP effect on Hashimoto`s thyroiditis
SNP effect on CD39 on CD39+ secreting Treg

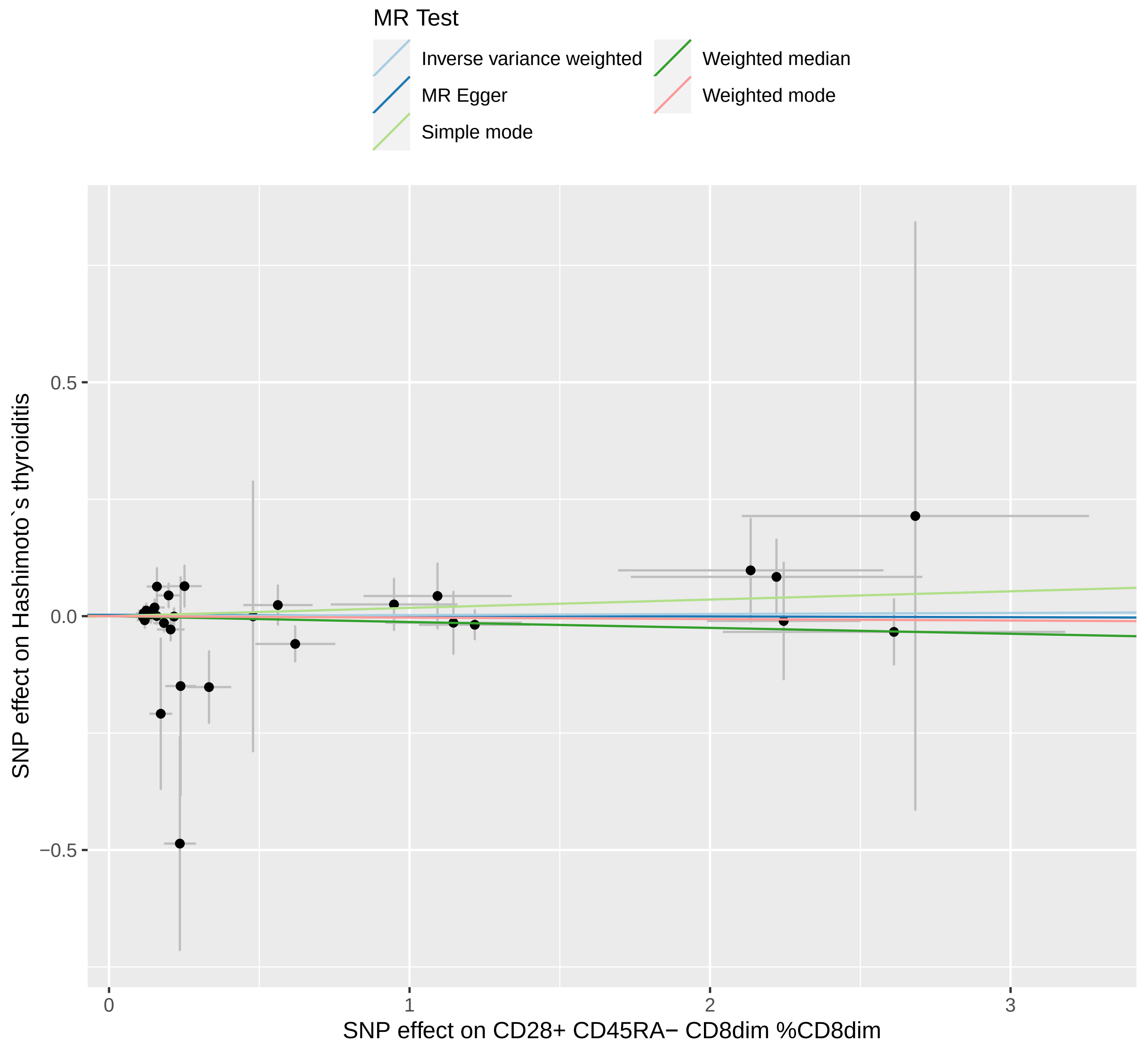
MR Test
Inverse variance weighted
MR Egger
Simple mode
Weighted median
Weighted mode
0.5
0.0
−0.5
0
1
2
3
SNP effect on Hashimoto`s thyroiditis
SNP effect on CD28+ CD45RA− CD8dim %CD8dim

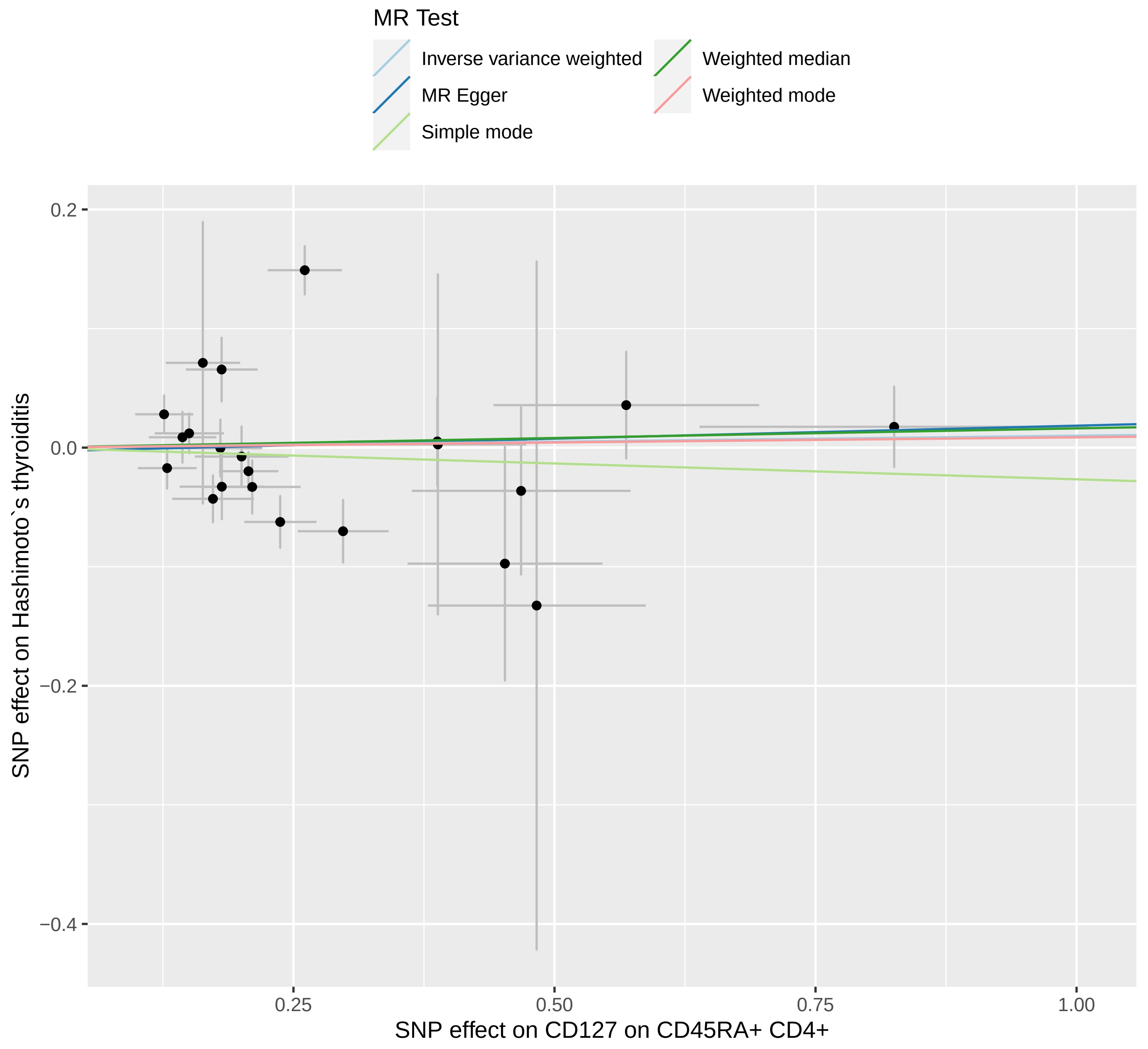

MR Test
Inverse variance weighted
MR Egger
Simple mode
Weighted median
Weighted mode
0.2
0.0
−0.2
−0.4
0.25
0.50
0.75
1.00
SNP effect on Hashimoto`s thyroiditis
SNP effect on CD127 on CD45RA+ CD4+

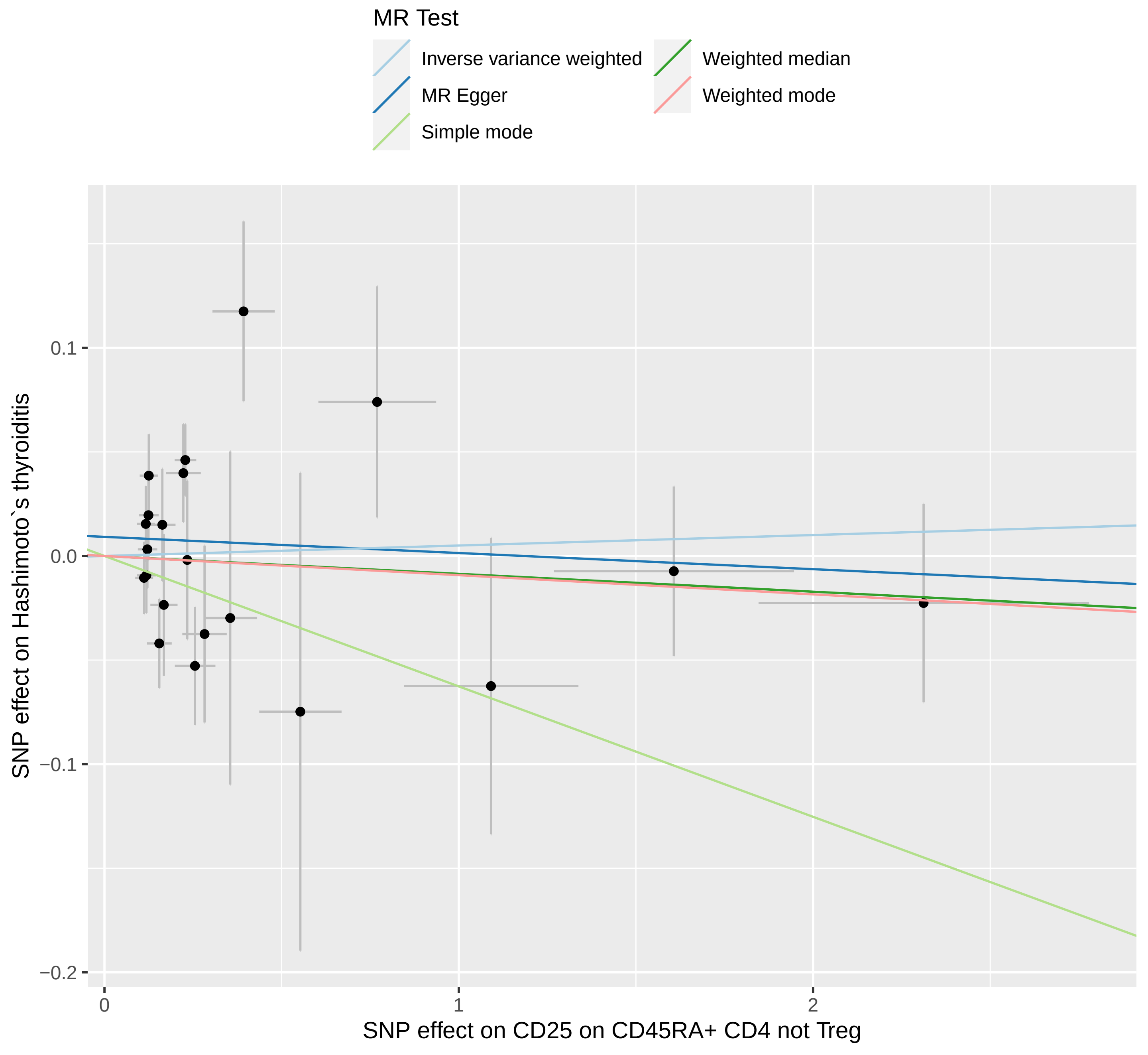
MR Test
Inverse variance weighted
MR Egger
Simple mode
Weighted median
Weighted mode
SNP effect on Hashimoto`s thyroiditis
0.1
0.0
−0.1
−0.2
0
1
2
SNP effect on CD25 on CD45RA+ CD4 not Treg

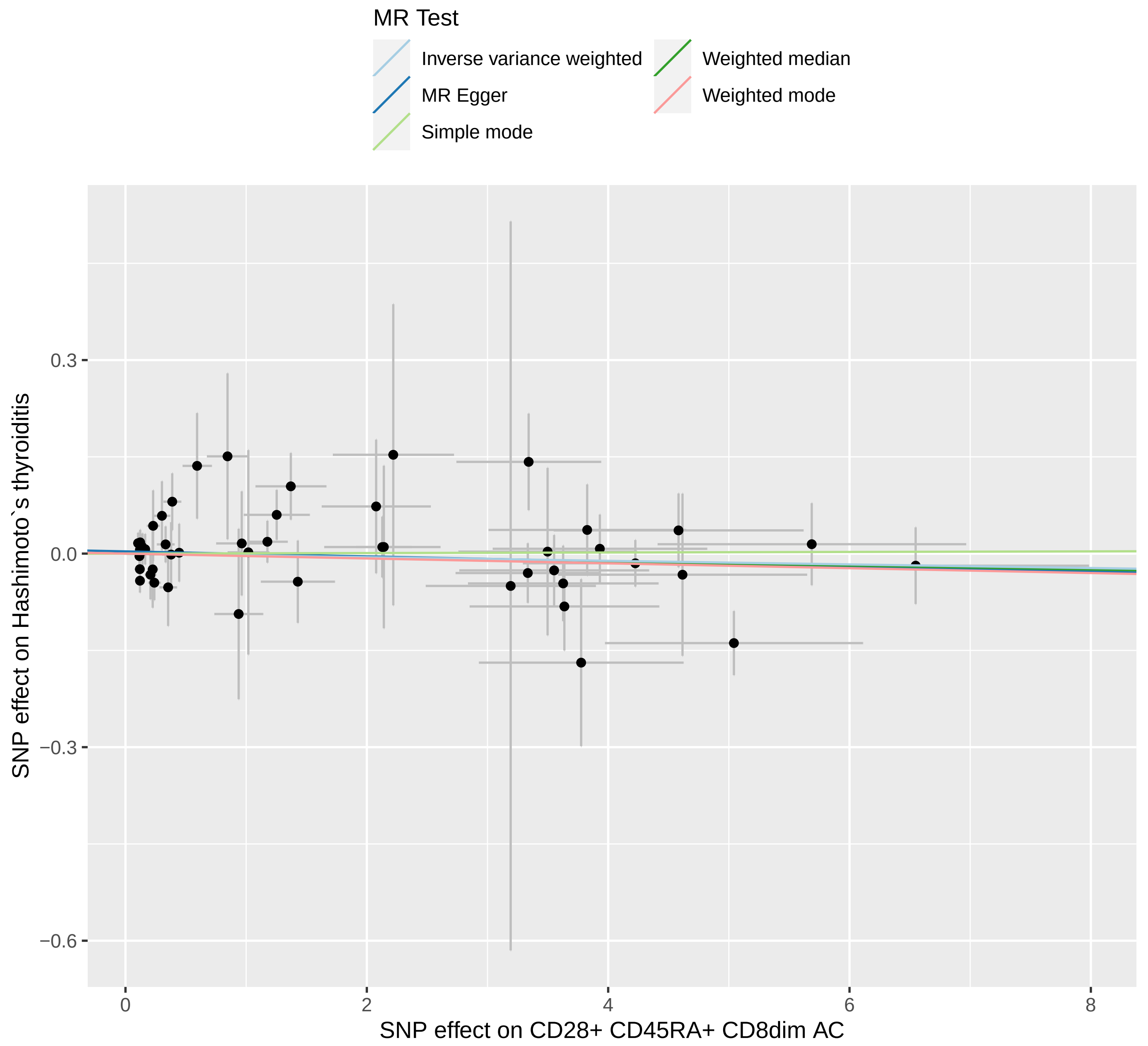
MR Test
Inverse variance weighted
MR Egger
Simple mode
Weighted median
Weighted mode
SNP effect on Hashimoto`s thyroiditis
0.3
0.0
−0.3
−0.6
0
2
4
6
8
SNP effect on CD28+ CD45RA+ CD8dim AC

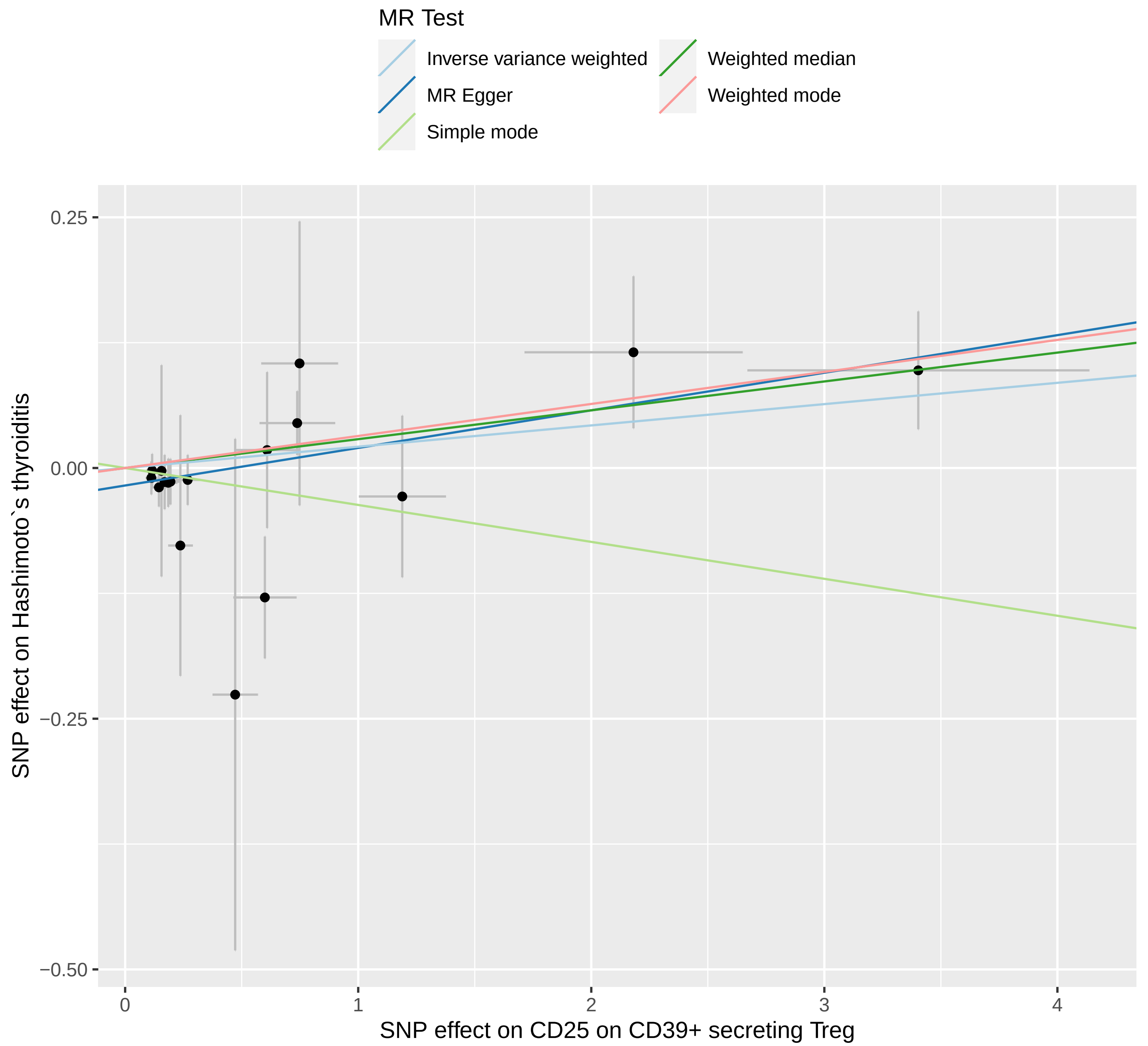

MR Test
Inverse variance weighted
MR Egger
Simple mode
Weighted median
Weighted mode
0.25
0.00
−0.25
−0.50
0
1
2
3
4
SNP effect on Hashimoto`s thyroiditis
SNP effect on CD25 on CD39+ secreting Treg

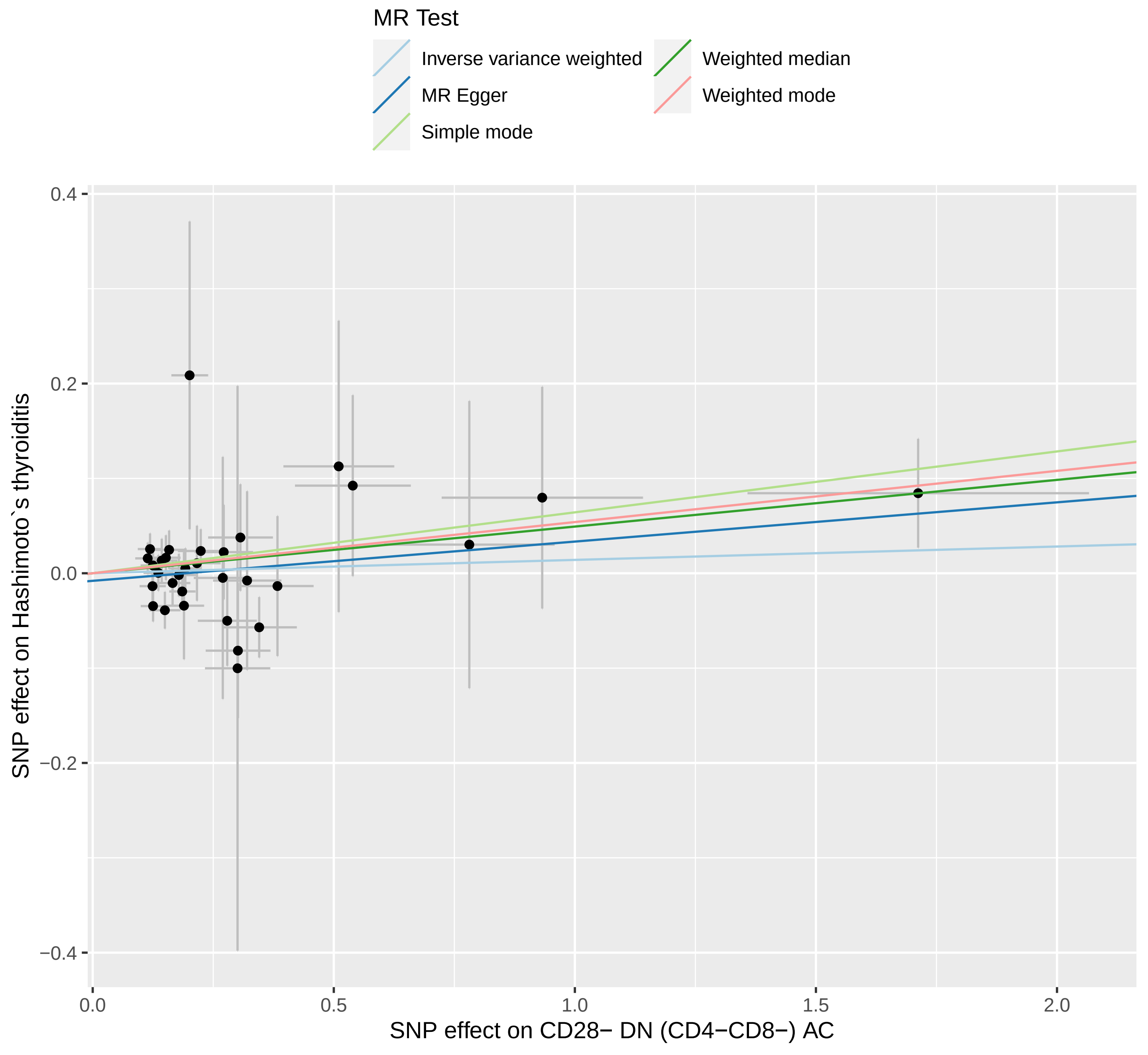
MR Test
Inverse variance weighted
MR Egger
Simple mode
Weighted median
Weighted mode
SNP effect on Hashimoto`s thyroiditis
SNP effect on CD28− DN (CD4−CD8−) AC
0.4
0.2
0.0
−0.2
−0.4
0.0
0.5
1.0
1.5
2.0

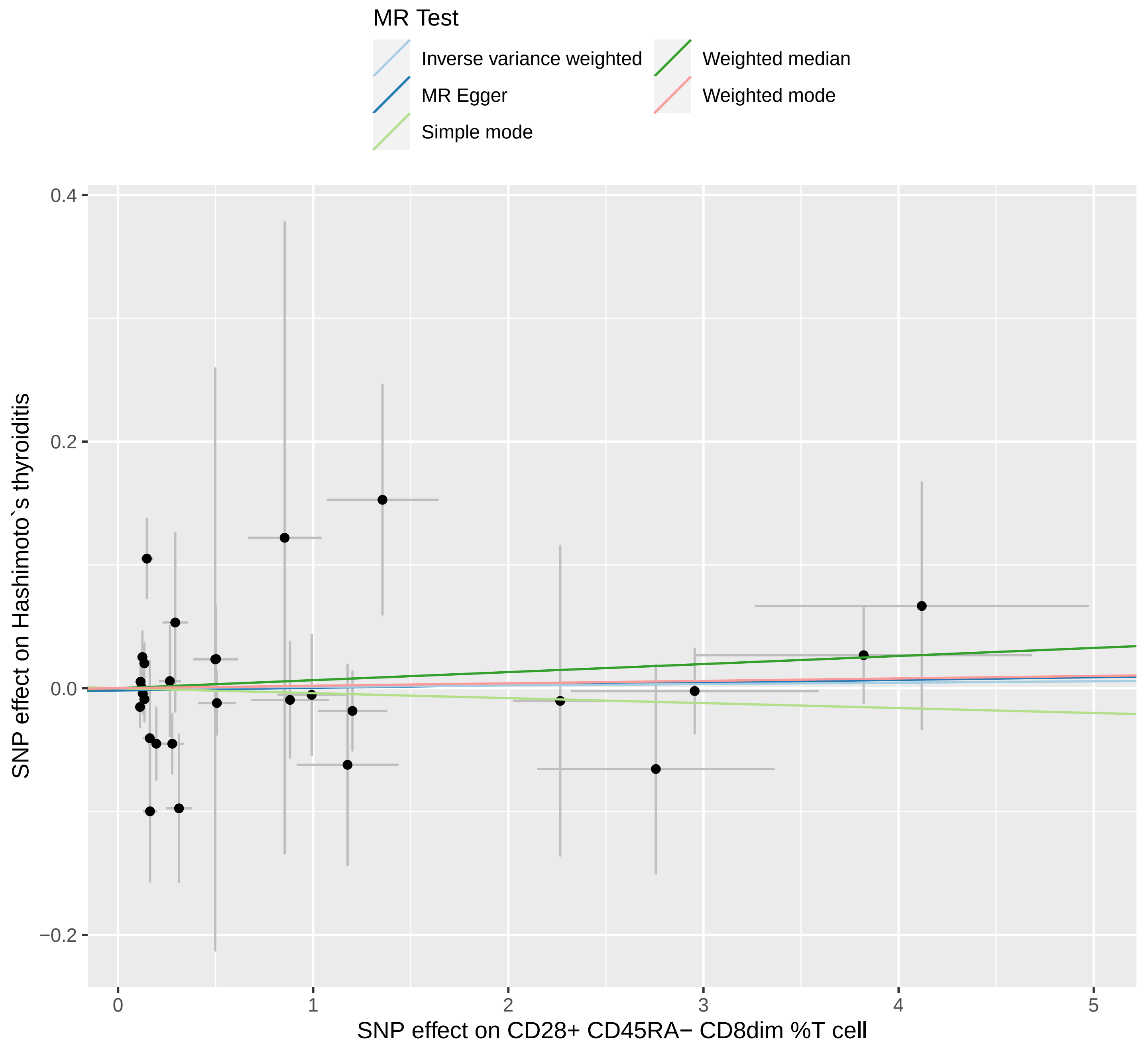
MR Test
Inverse variance weighted
MR Egger
Simple mode
Weighted median
Weighted mode
SNP effect on Hashimoto`s thyroiditis
0.4
0.2
0.0
−0.2
0
1
2
3
4
5
SNP effect on CD28+ CD45RA− CD8dim %T cell

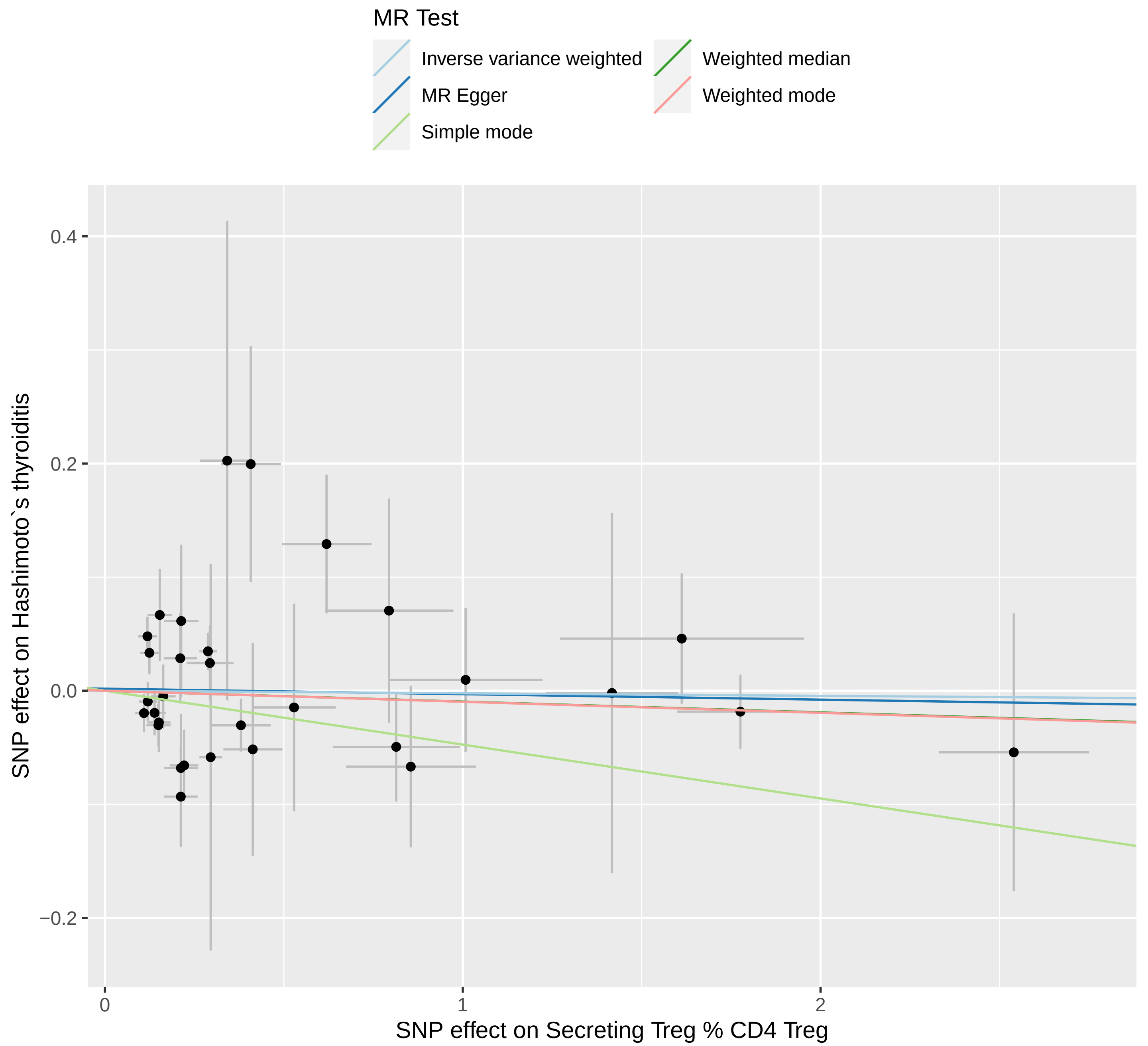

MR Test
Inverse variance weighted
MR Egger
Simple mode
Weighted median
Weighted mode
0.4
0.2
0.0
−0.2
0
1
2
SNP effect on Hashimoto`s thyroiditis
SNP effect on Secreting Treg % CD4 Treg

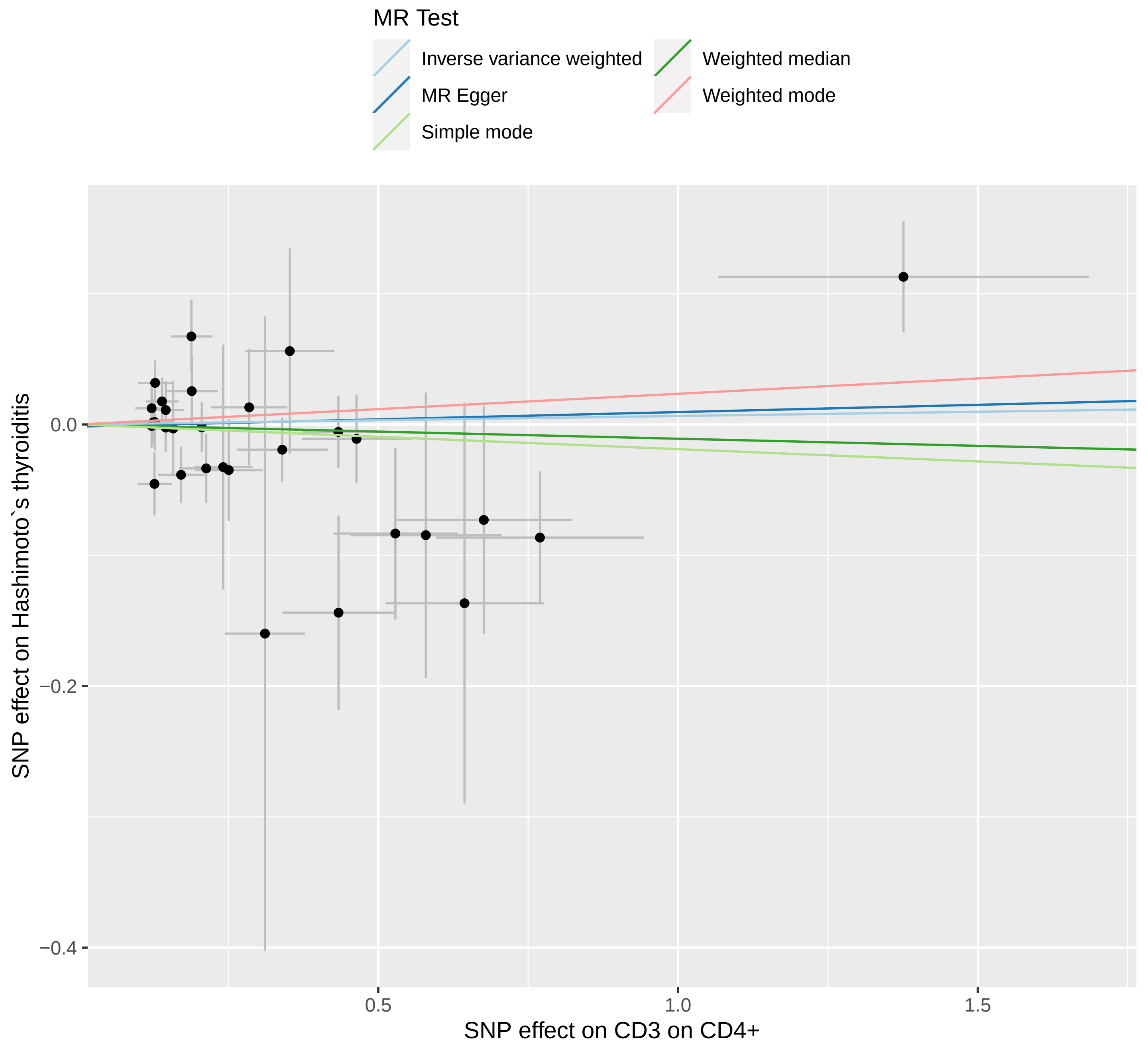

MR Test
Inverse variance weighted
MR Egger
Simple mode
Weighted median
Weighted mode
SNP effect on Hashimoto`s thyroiditis
0.0
−0.2
−0.4
0.5
1.0
1.5
SNP effect on CD3 on CD4+

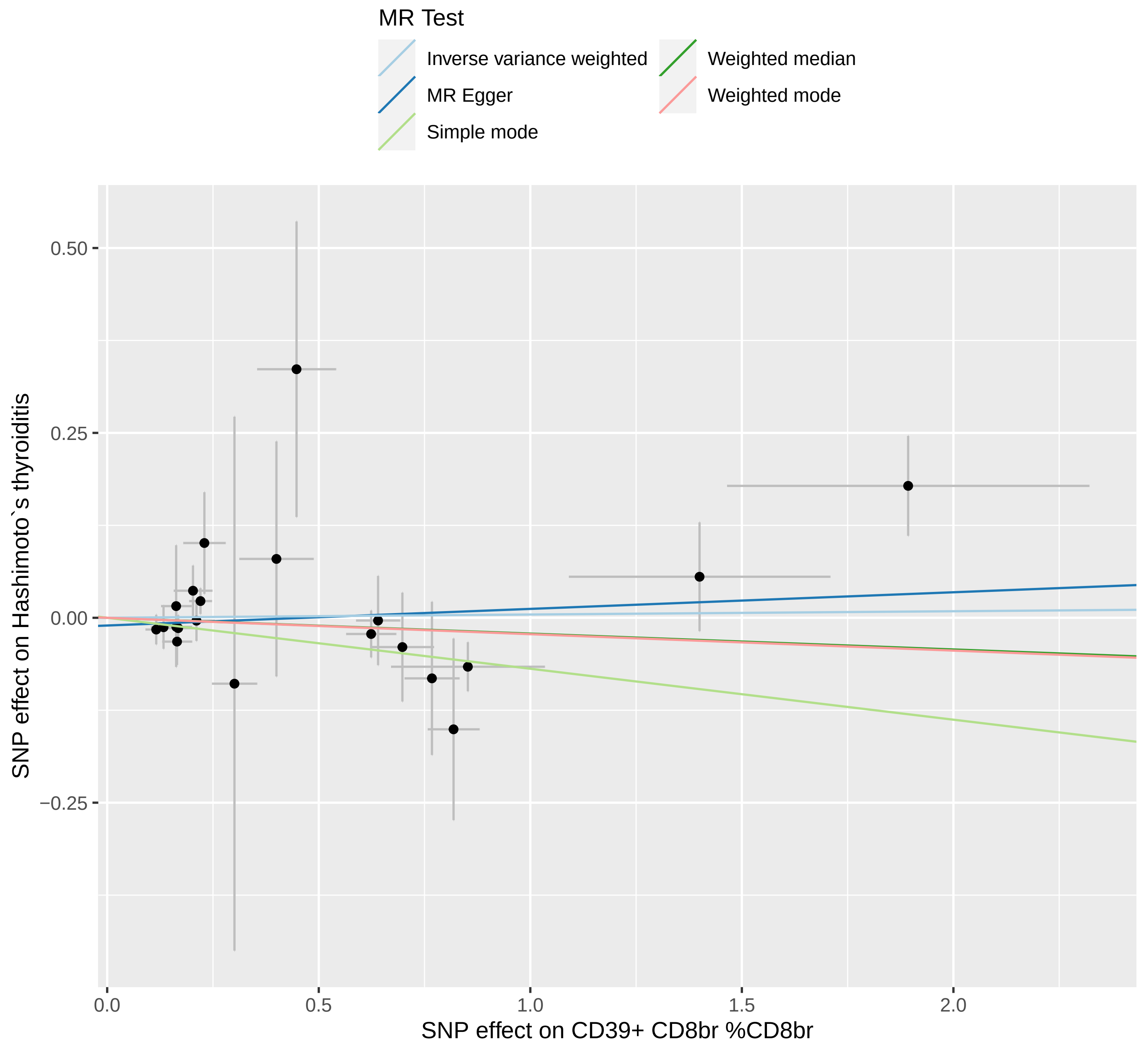

MR Test
Inverse variance weighted
MR Egger
Simple mode
Weighted median
Weighted mode
0.50
0.25
0.00
−0.25
0.0
0.5
1.0
1.5
2.0
SNP effect on CD39+ CD8br %CD8br
SNP effect on Hashimoto`s thyroiditis

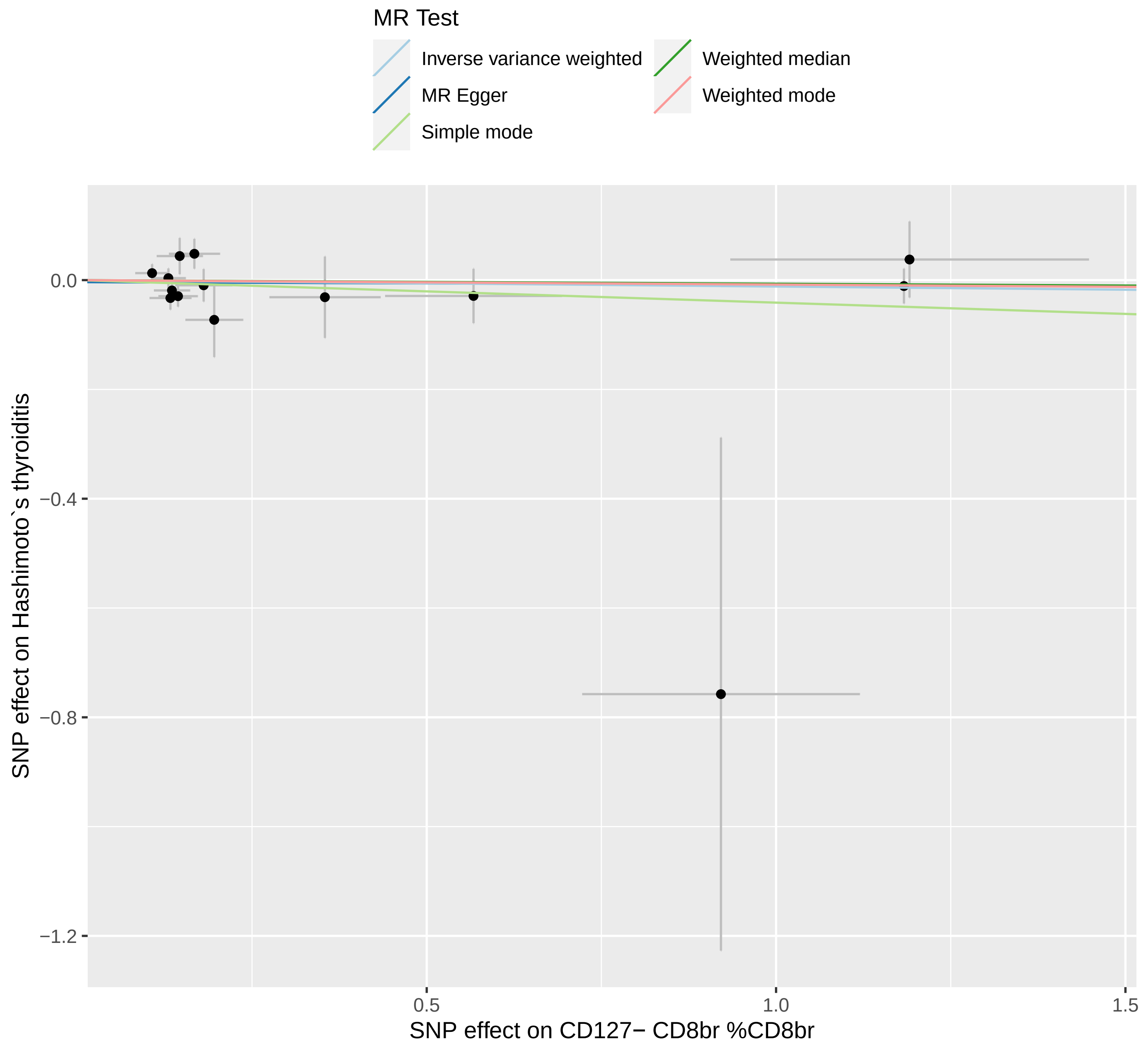

MR Test
Inverse variance weighted
MR Egger
Simple mode
Weighted median
Weighted mode
0.0
−0.4
−0.8
−1.2
0.5
1.0
1.5
SNP effect on Hashimoto`s thyroiditis
SNP effect on CD127− CD8br %CD8br

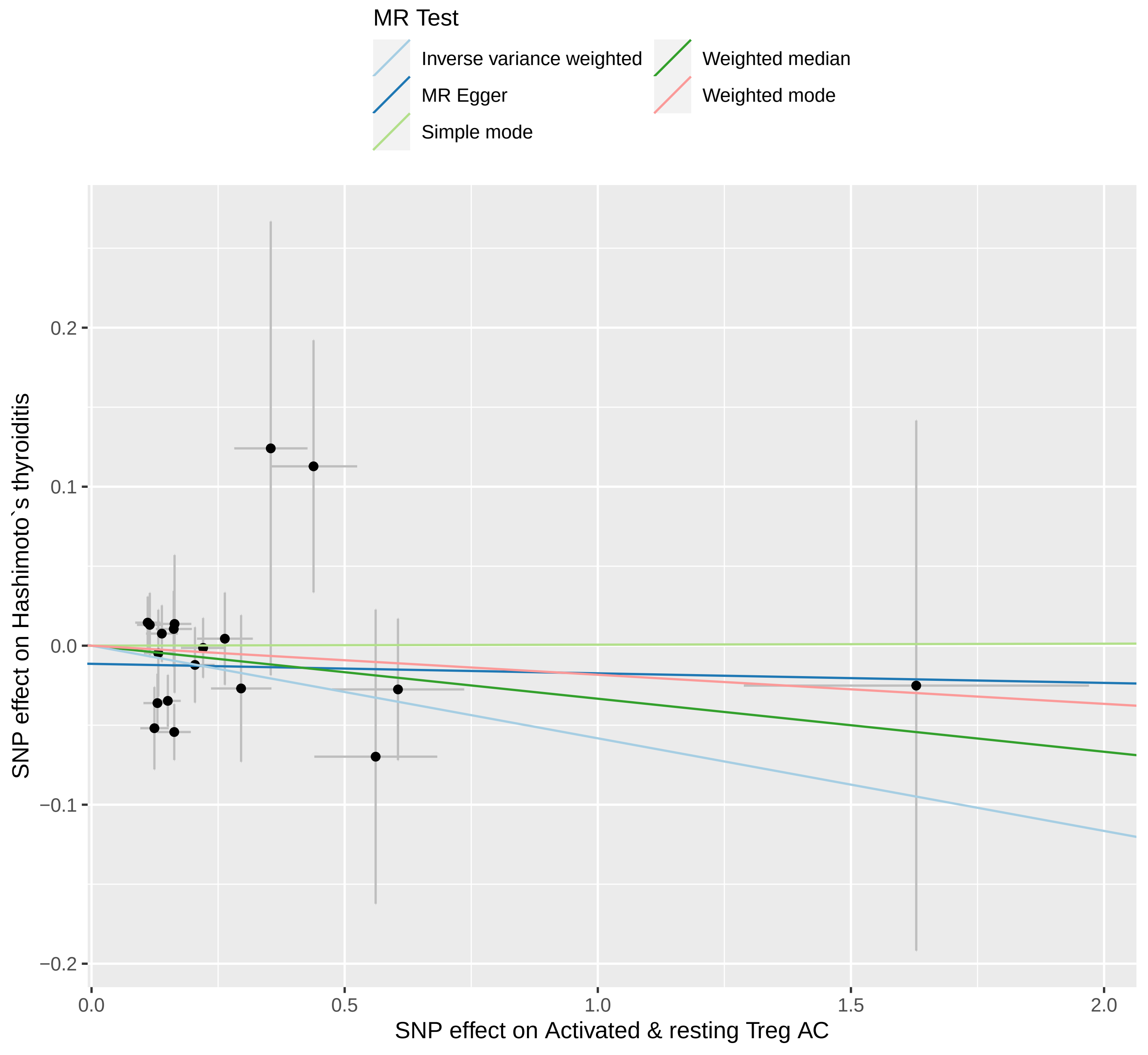

MR Test
Inverse variance weighted
MR Egger
Simple mode
Weighted median
Weighted mode
SNP effect on Hashimoto`s thyroiditis
0.2
0.1
0.0
−0.1
−0.2
0.0
0.5
1.0
1.5
2.0
SNP effect on Activated & resting Treg AC

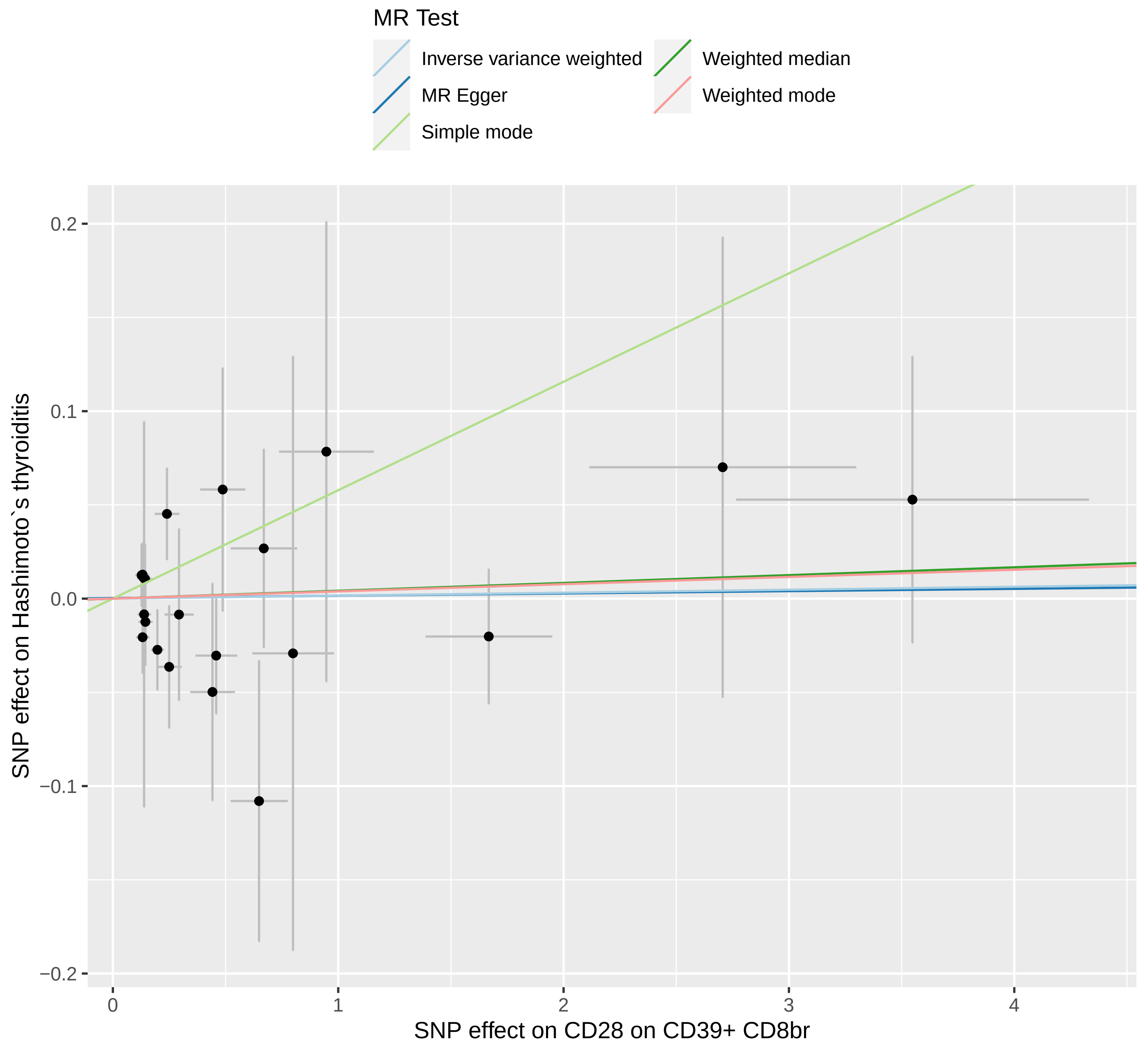
MR Test
Inverse variance weighted
MR Egger
Simple mode
Weighted median
Weighted mode
0.2
0.1
0.0
−0.1
−0.2
0
1
2
3
4
SNP effect on Hashimoto`s thyroiditis
SNP effect on CD28 on CD39+ CD8br

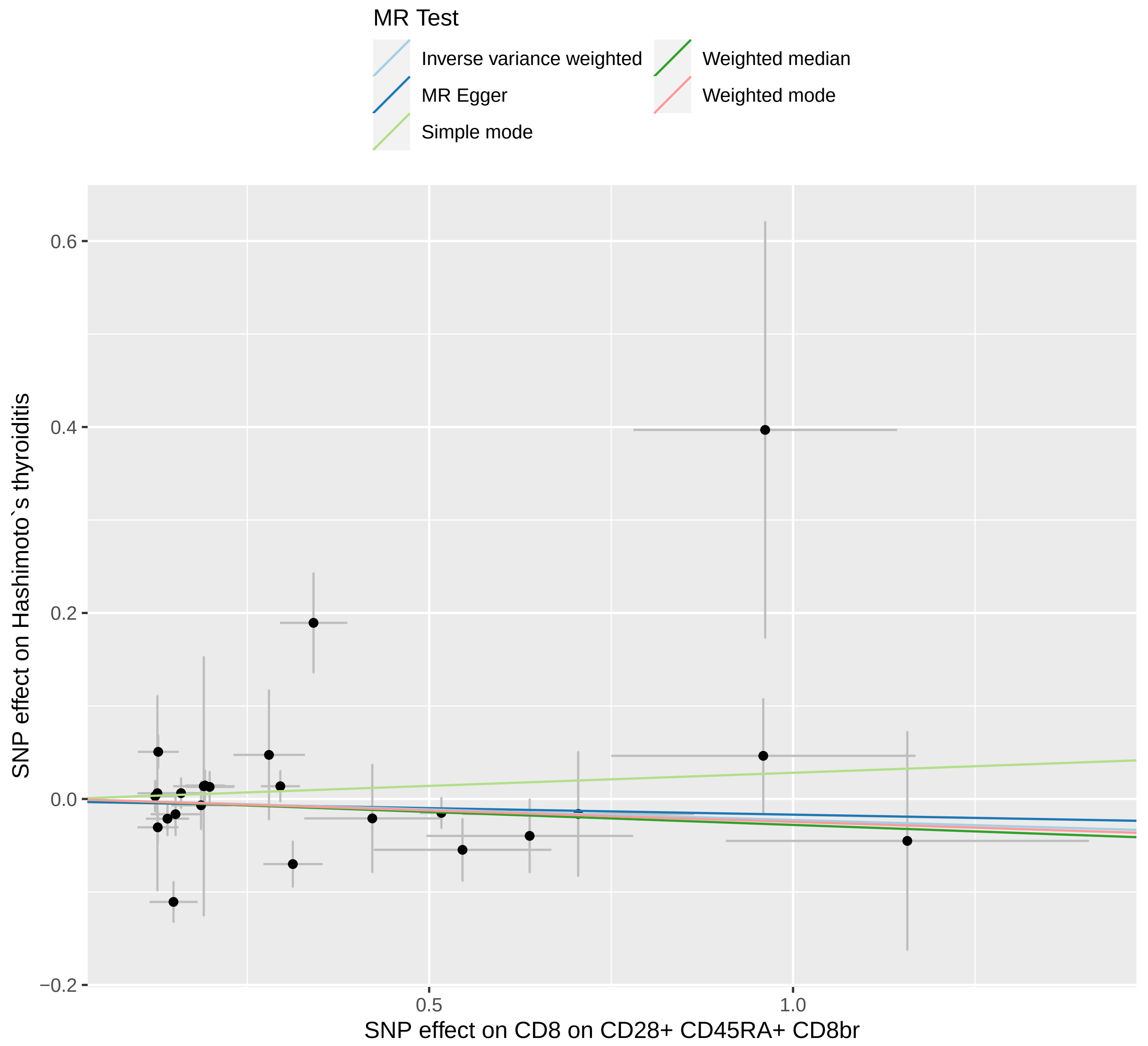

MR Test
Inverse variance weighted
MR Egger
Simple mode
Weighted median
Weighted mode
0.6
0.4
0.2
0.0
−0.2
0.5
1.0
SNP effect on Hashimoto`s thyroiditis
SNP effect on CD8 on CD28+ CD45RA+ CD8br

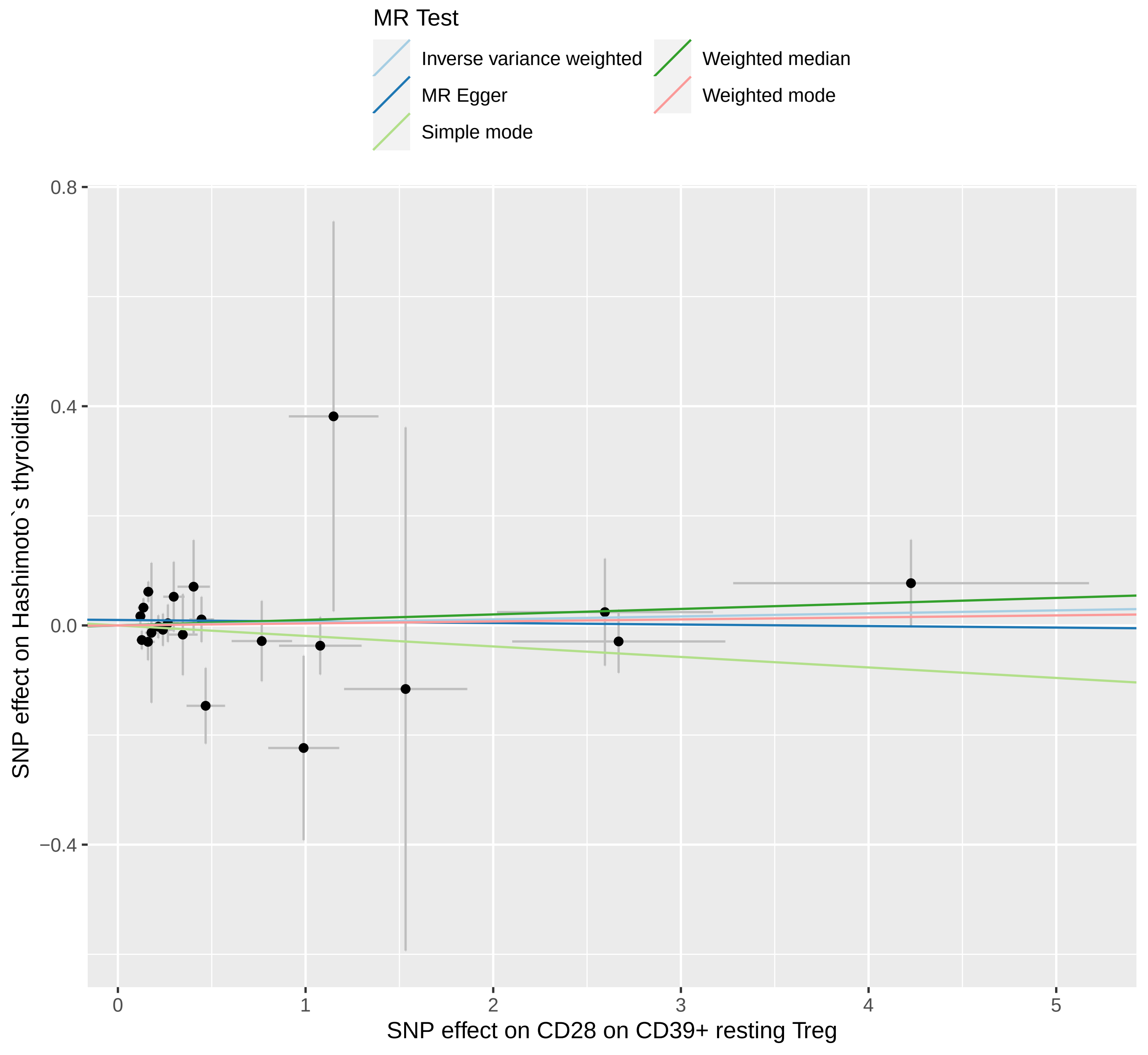

MR Test
Inverse variance weighted
MR Egger
Simple mode
Weighted median
Weighted mode
0.8
0.4
0.0
−0.4
0
1
2
3
4
5
SNP effect on Hashimoto`s thyroiditis
SNP effect on CD28 on CD39+ resting Treg

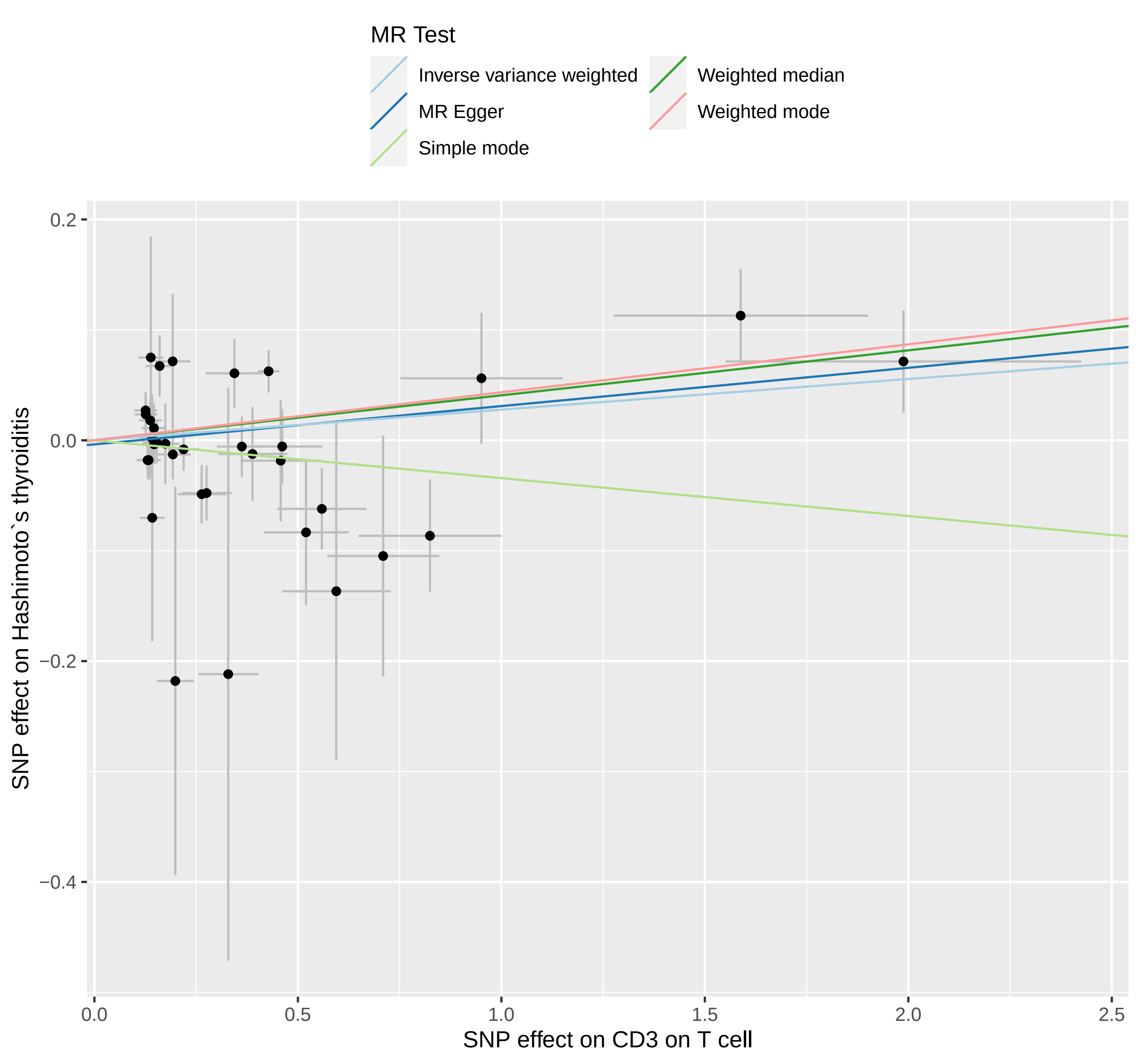

MR Test
Inverse variance weighted
MR Egger
Simple mode
Weighted median
Weighted mode
0.2
0.0
−0.2
−0.4
0.0
0.5
1.0
1.5
2.0
2.5
SNP effect on Hashimoto`s thyroiditis
SNP effect on CD3 on T cell

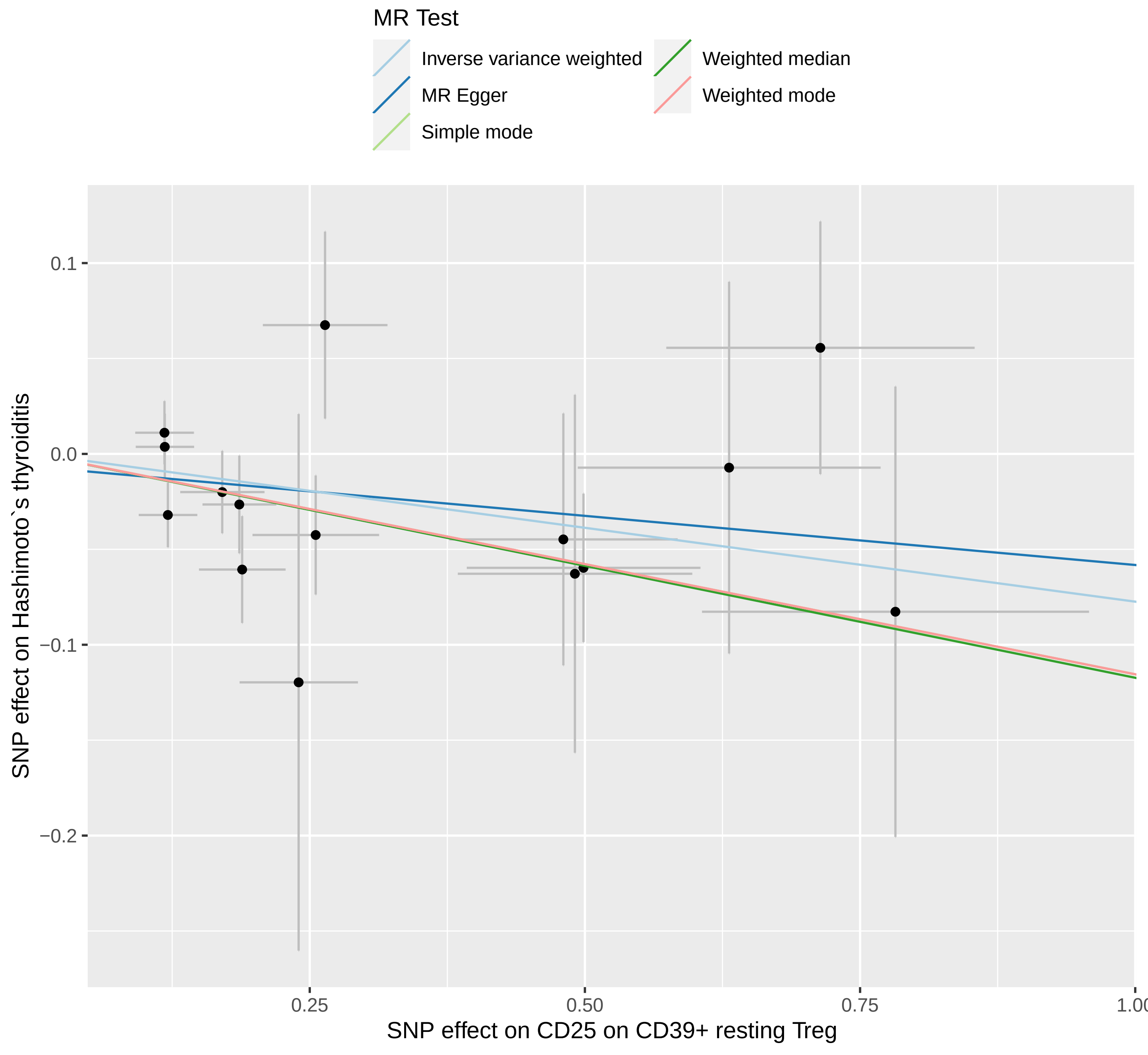
MR Test
Inverse variance weighted
MR Egger
Simple mode
Weighted median
Weighted mode
0.1
0.0
−0.1
−0.2
0.25
0.50
0.75
1.00
SNP effect on CD25 on CD39+ resting Treg
SNP effect on Hashimoto`s thyroiditis

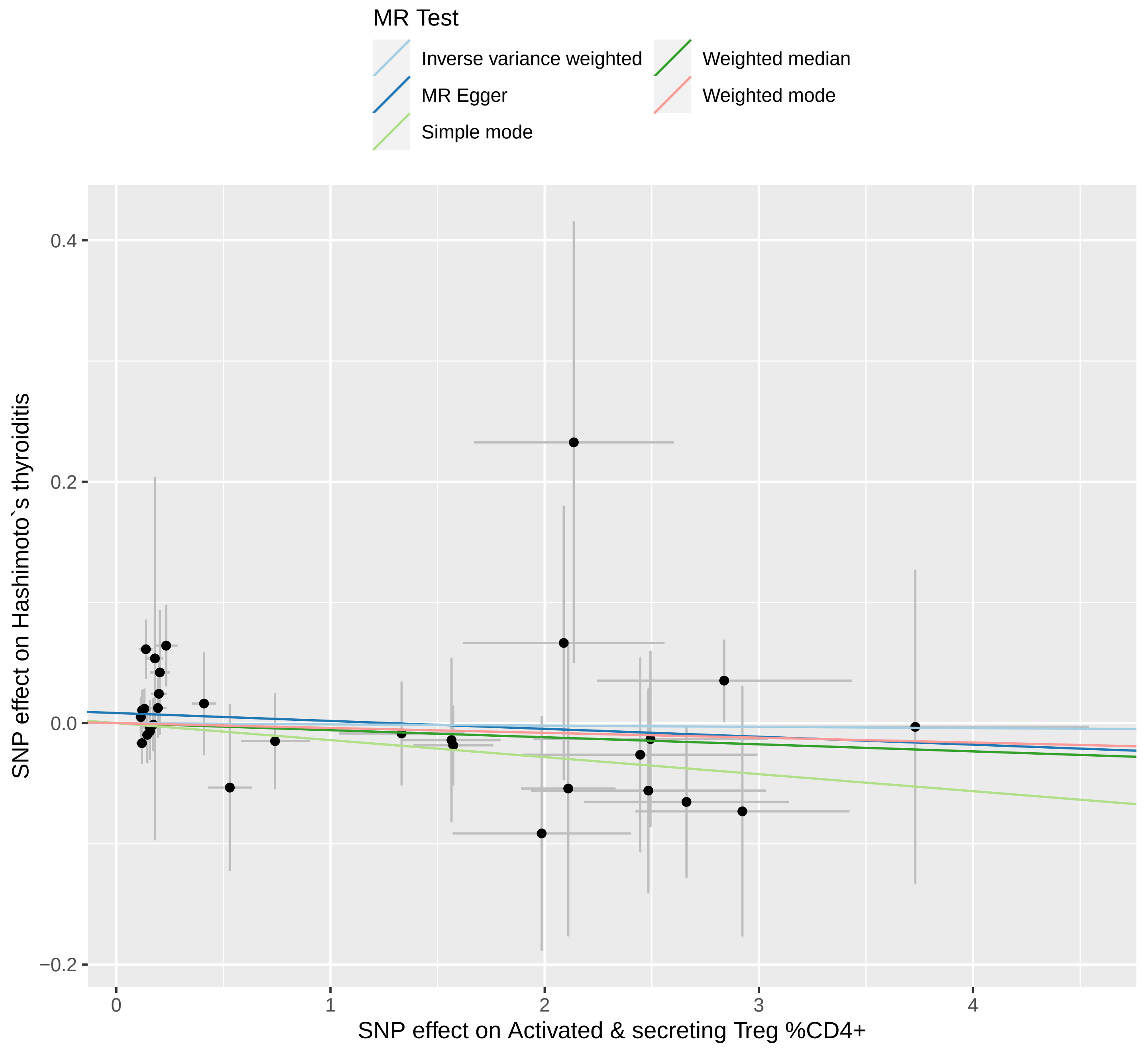
MR Test
Inverse variance weighted
MR Egger
Simple mode
Weighted median
Weighted mode
0.4
0.2
0.0
−0.2
0
1
2
3
4
SNP effect on Hashimoto`s thyroiditis
SNP effect on Activated & secreting Treg %CD4+

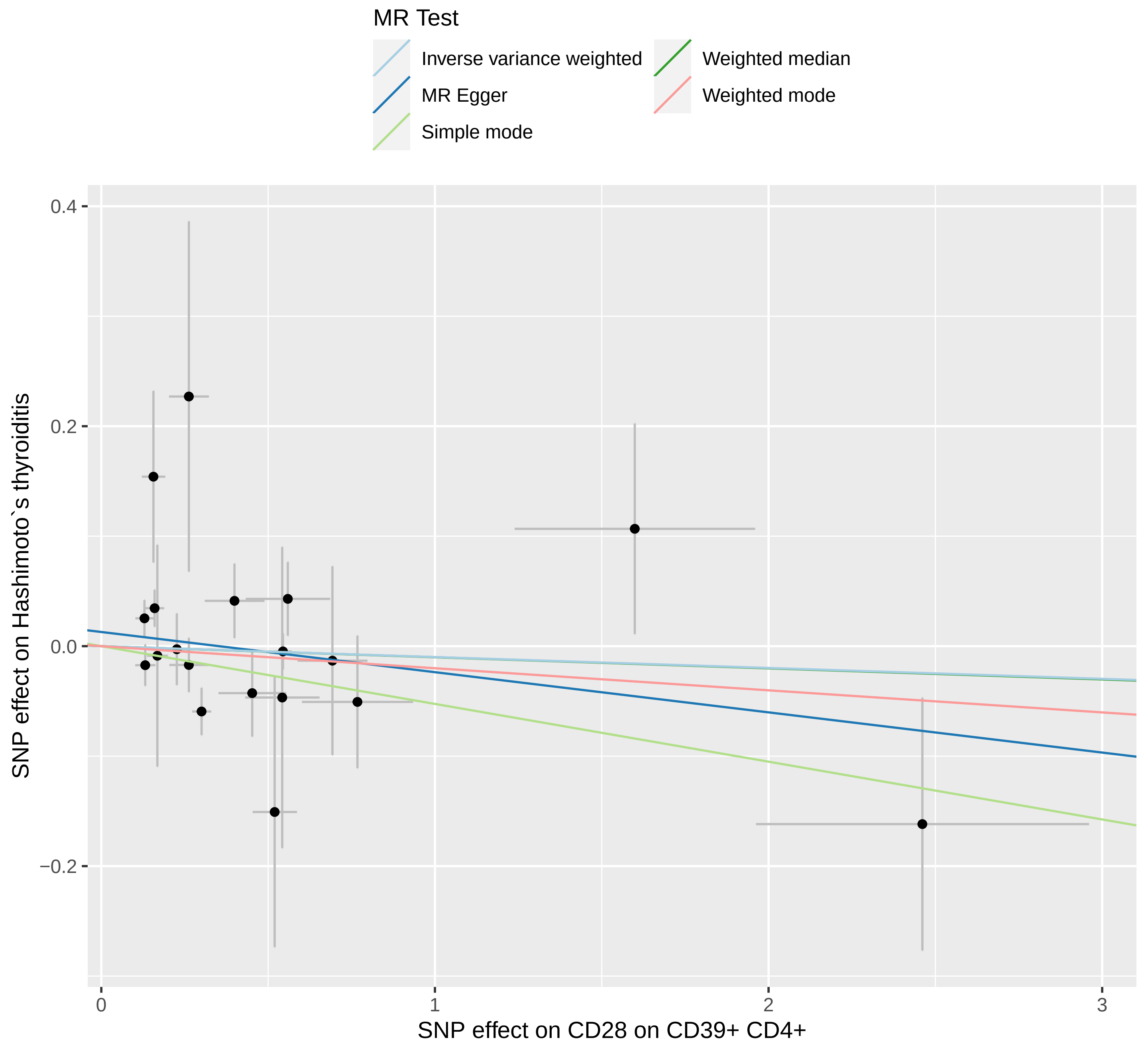
MR Test
Inverse variance weighted
MR Egger
Simple mode
Weighted median
Weighted mode
0.4
0.2
0.0
−0.2
0
1
2
3
SNP effect on Hashimoto`s thyroiditis
SNP effect on CD28 on CD39+ CD4+

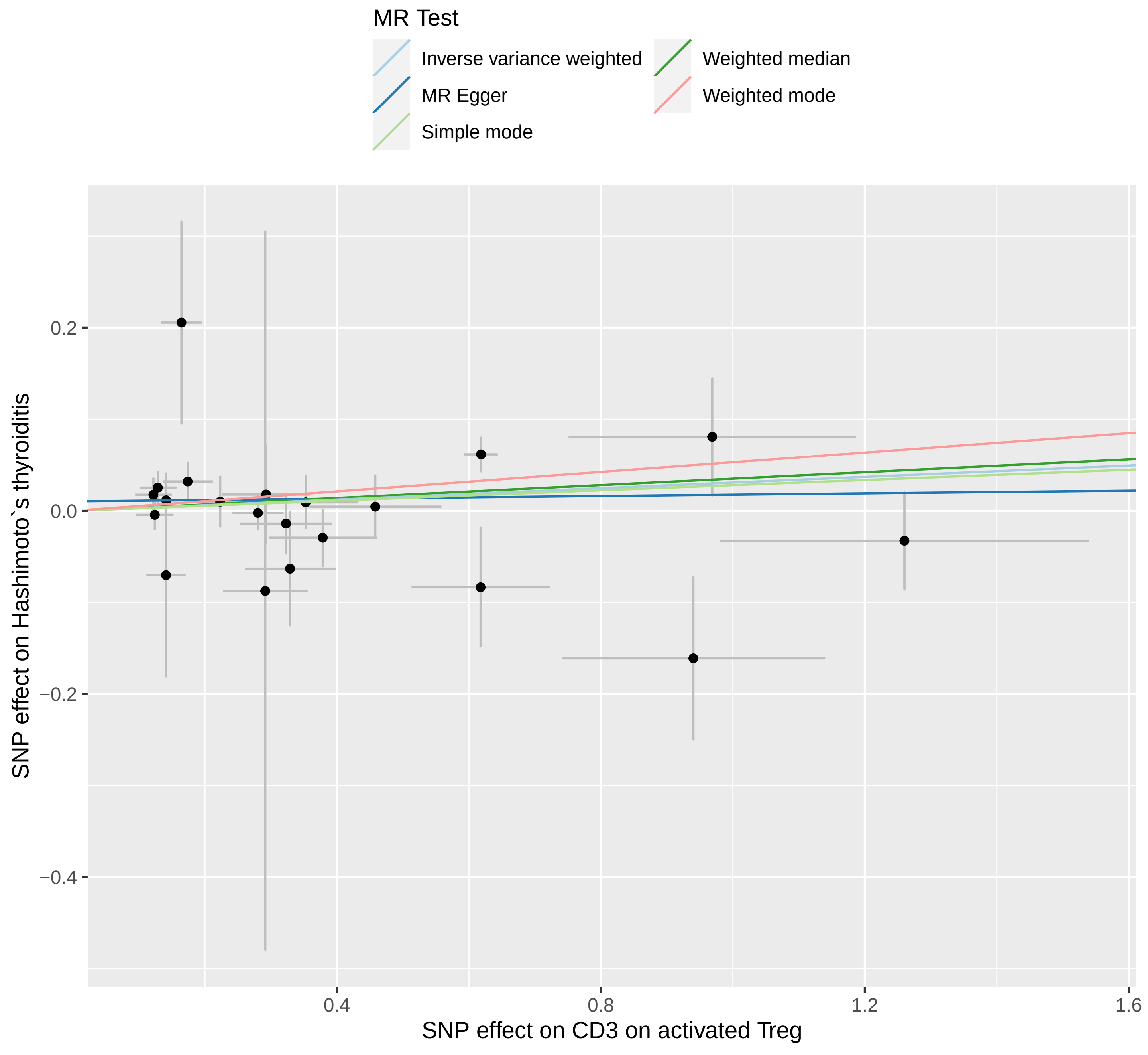
MR Test
Inverse variance weighted
MR Egger
Simple mode
Weighted median
Weighted mode
0.2
0.0
−0.2
−0.4
0.4
0.8
1.2
1.6
SNP effect on Hashimoto`s thyroiditis
SNP effect on CD3 on activated Treg

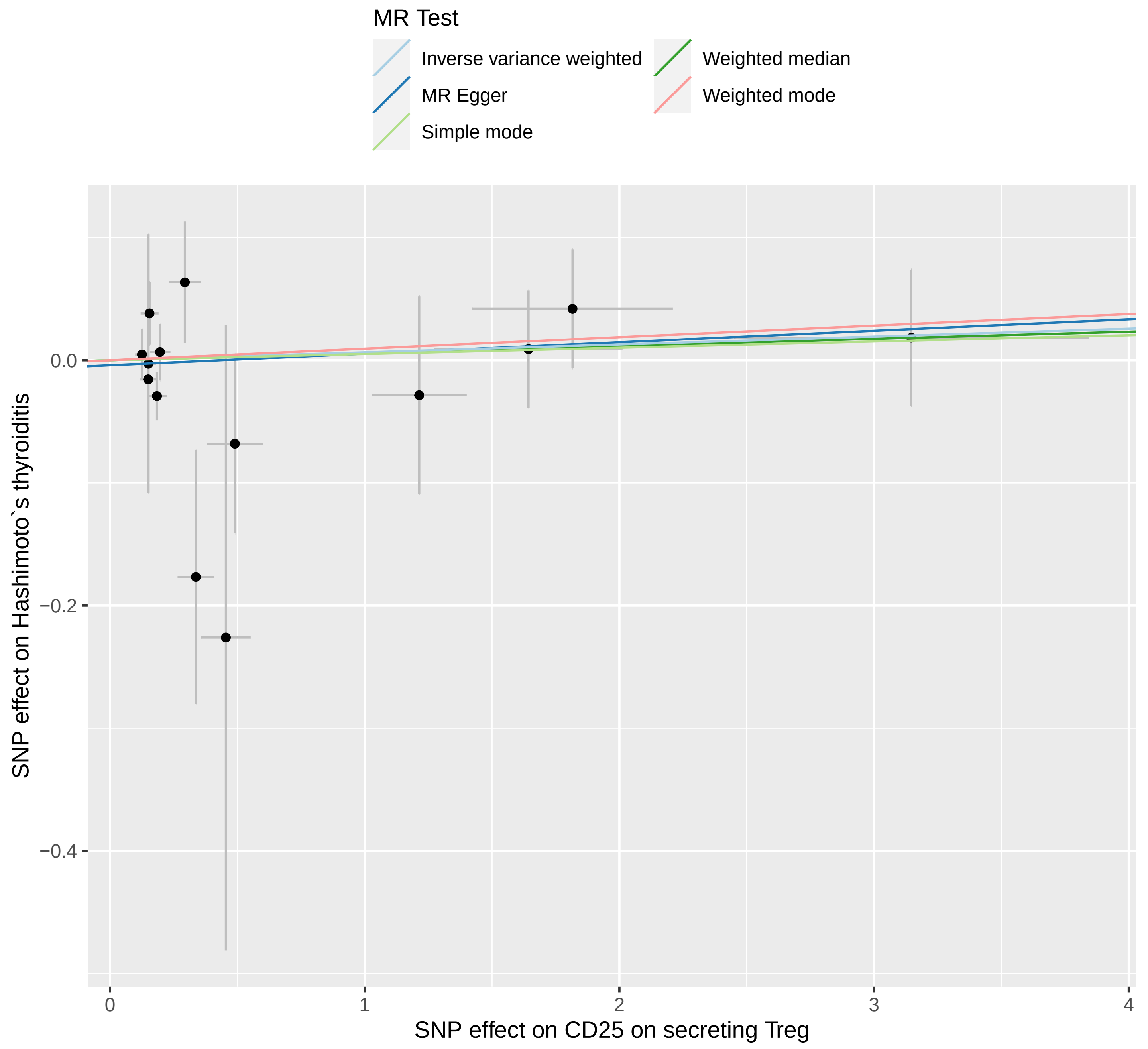

MR Test
Inverse variance weighted
MR Egger
Simple mode
Weighted median
Weighted mode
0.0
−0.2
−0.4
SNP effect on Hashimoto`s thyroiditis
0
1
2
3
4
SNP effect on CD25 on secreting Treg

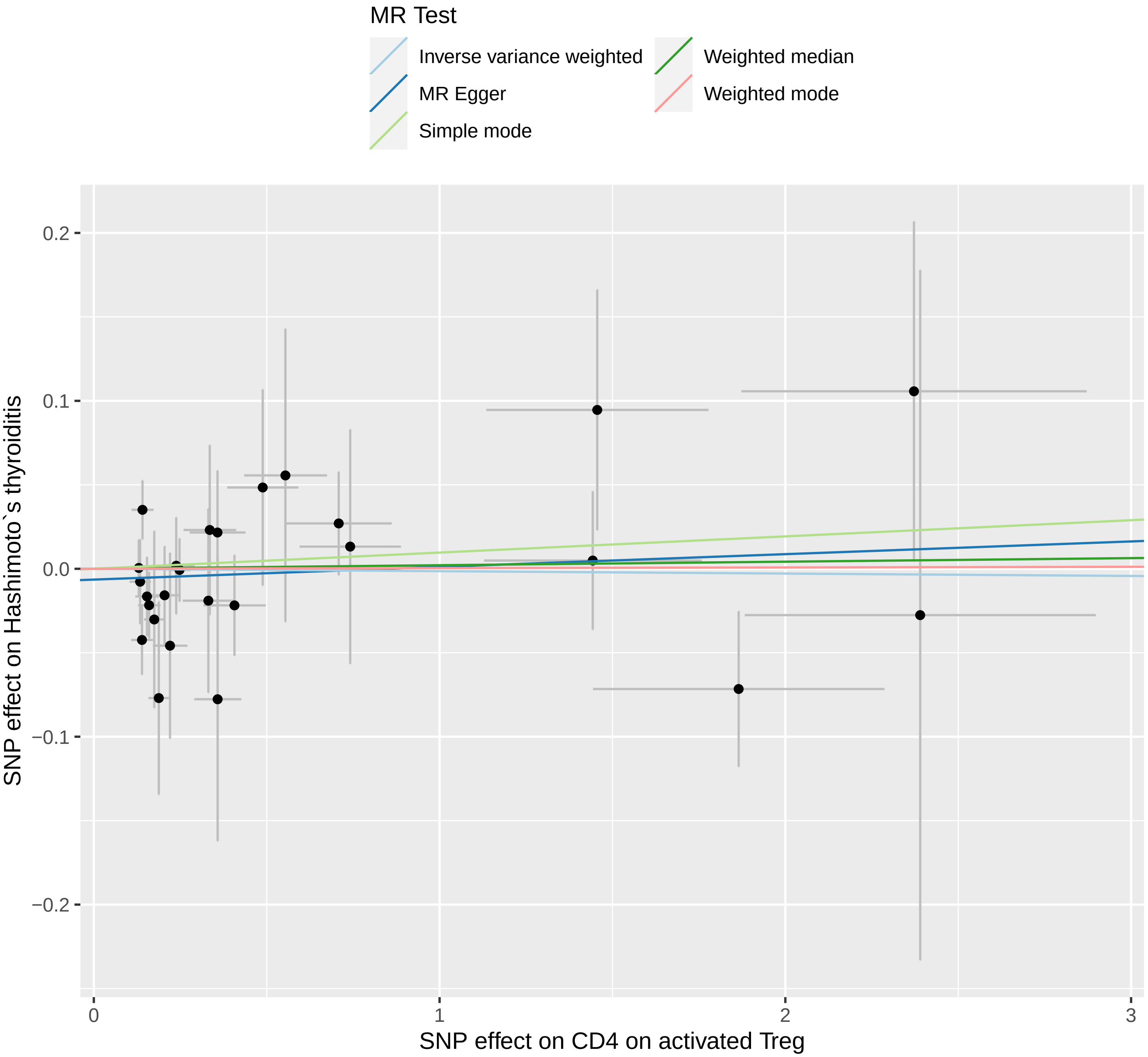

MR Test
Inverse variance weighted
MR Egger
Simple mode
Weighted median
Weighted mode
SNP effect on Hashimoto`s thyroiditis
SNP effect on CD4 on activated Treg
0.2
0.1
0.0
−0.1
−0.2
0
1
2
3

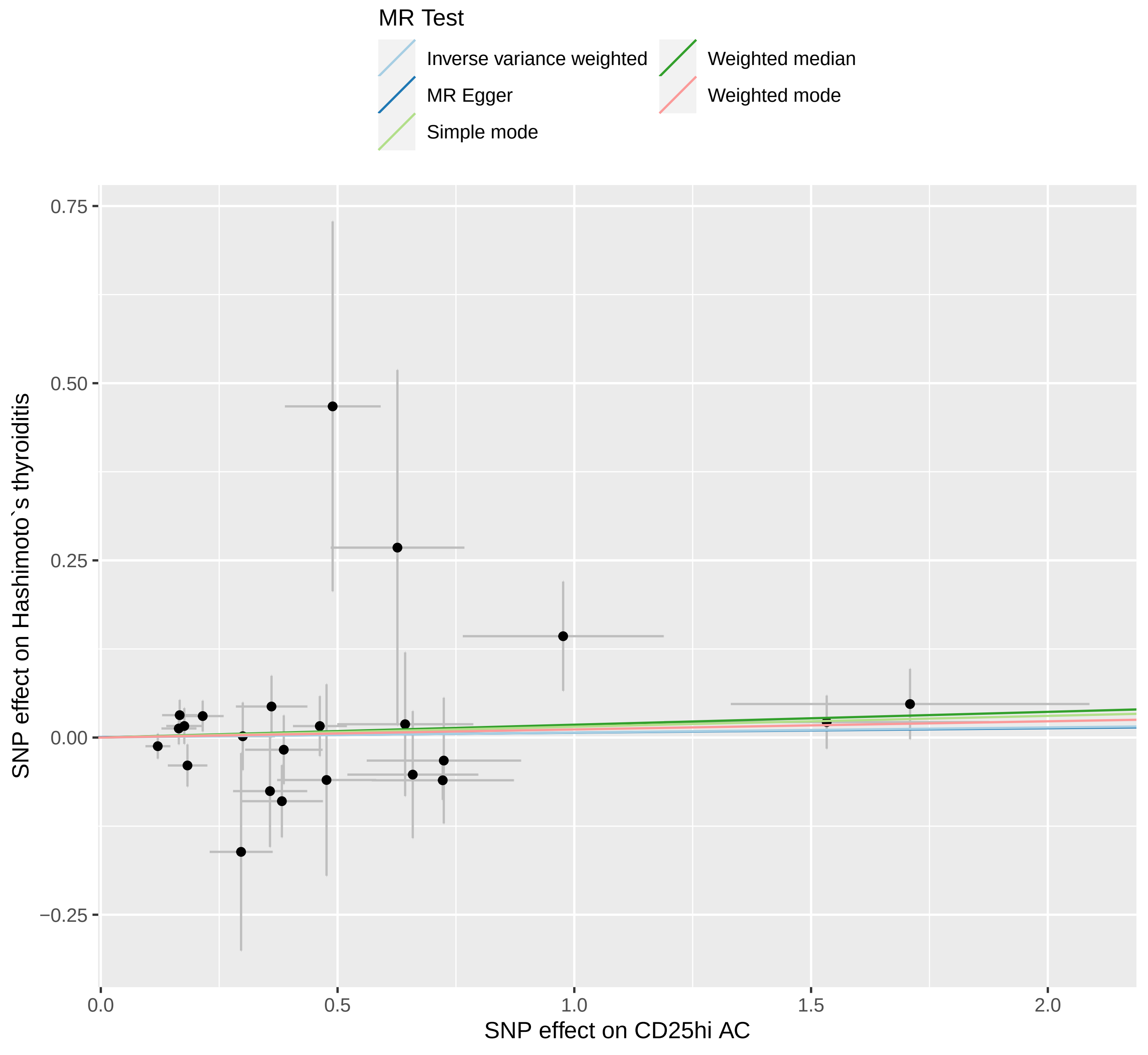

MR Test
Inverse variance weighted
MR Egger
Simple mode
Weighted median
Weighted mode
0.75
0.50
0.25
0.00
−0.25
SNP effect on Hashimoto`s thyroiditis
0.0
0.5
1.0
1.5
2.0
SNP effect on CD25hi AC

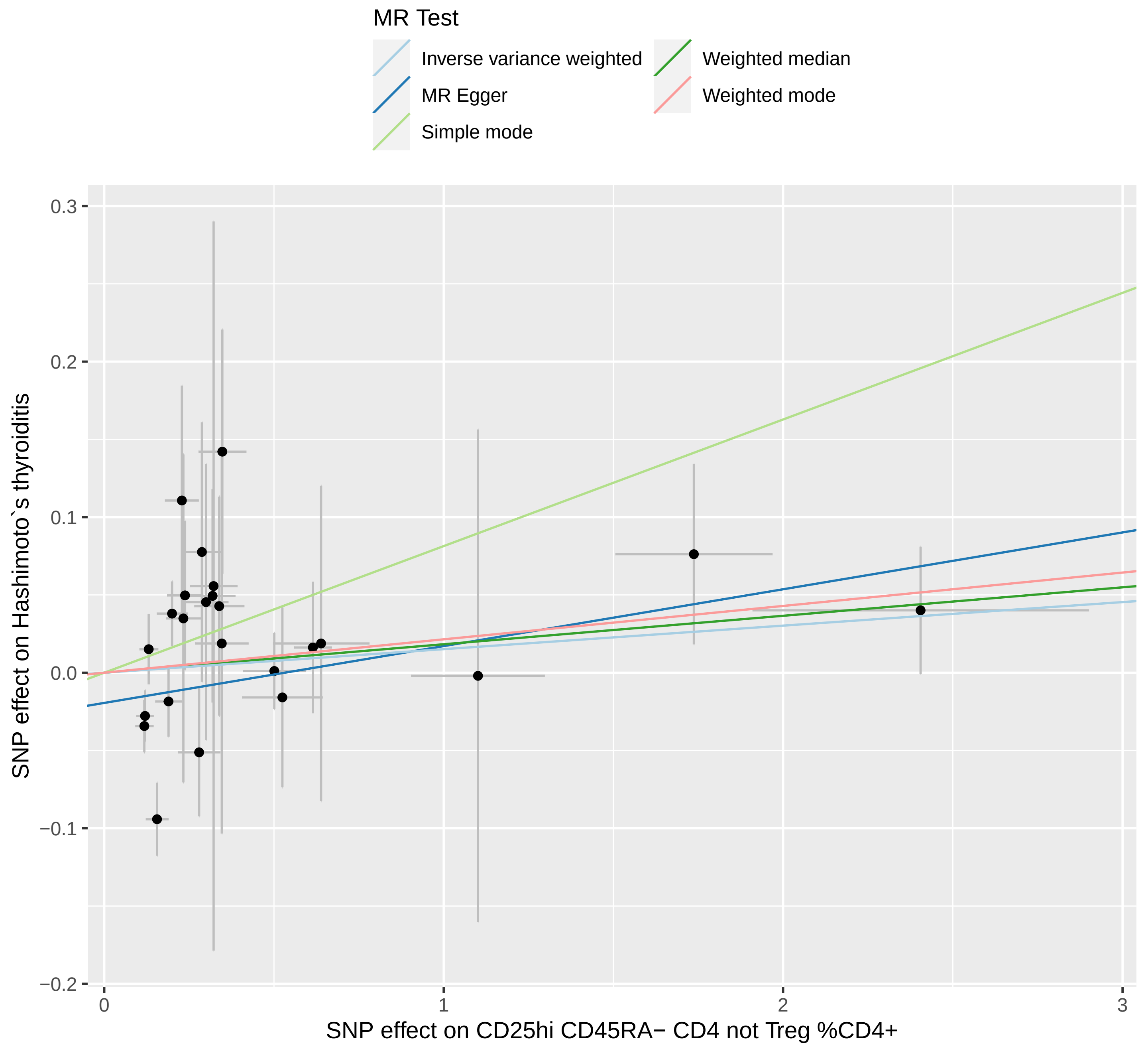

MR Test
Inverse variance weighted
MR Egger
Simple mode
Weighted median
Weighted mode
0.3
0.2
0.1
0.0
−0.1
−0.2
0
1
2
3
SNP effect on Hashimoto`s thyroiditis
SNP effect on CD25hi CD45RA− CD4 not Treg %CD4+

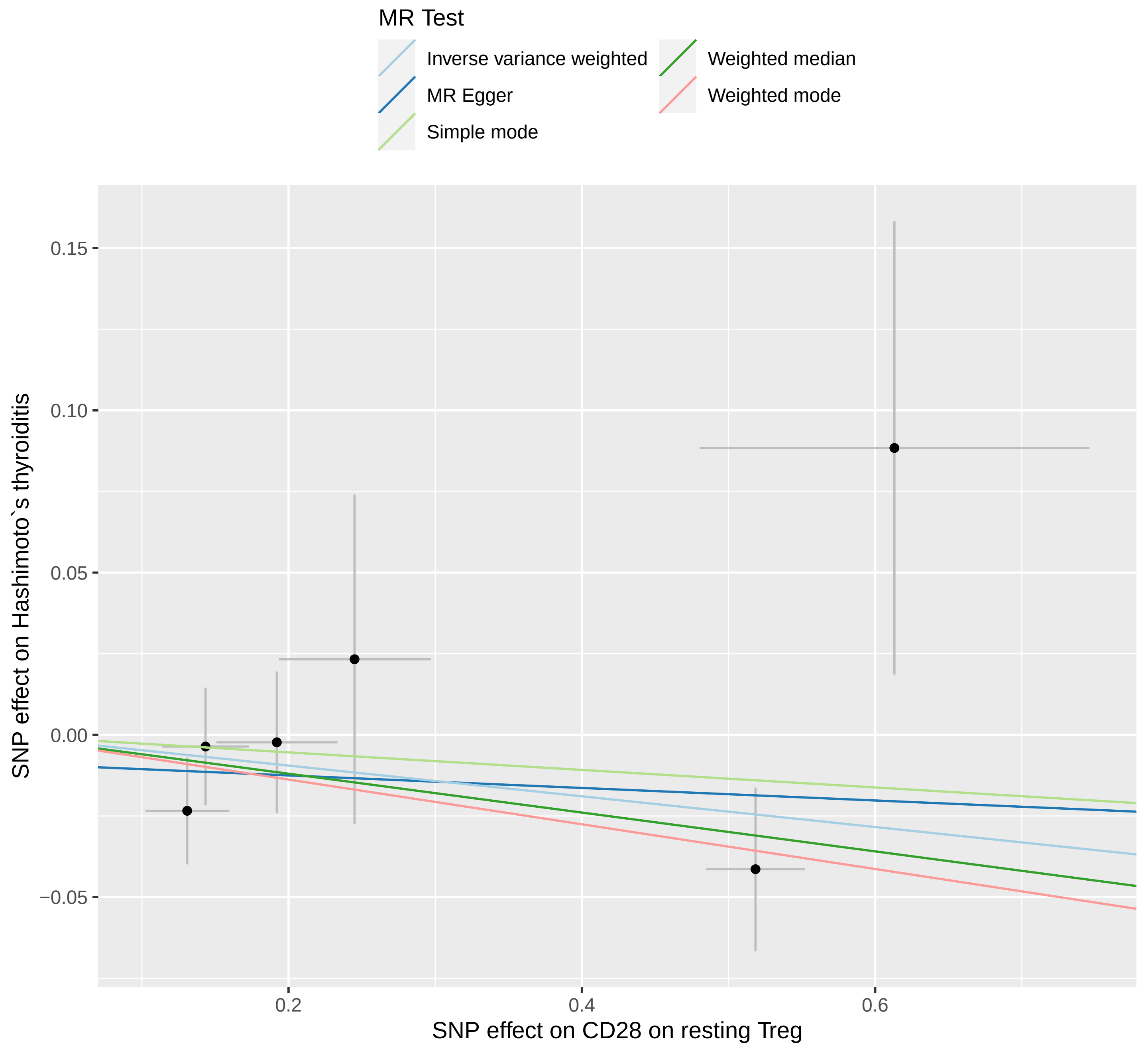
MR Test
Inverse variance weighted
MR Egger
Simple mode
Weighted median
Weighted mode
0.15
0.10
0.05
0.00
−0.05
0.2
0.4
0.6
SNP effect on CD28 on resting Treg
SNP effect on Hashimoto`s thyroiditis

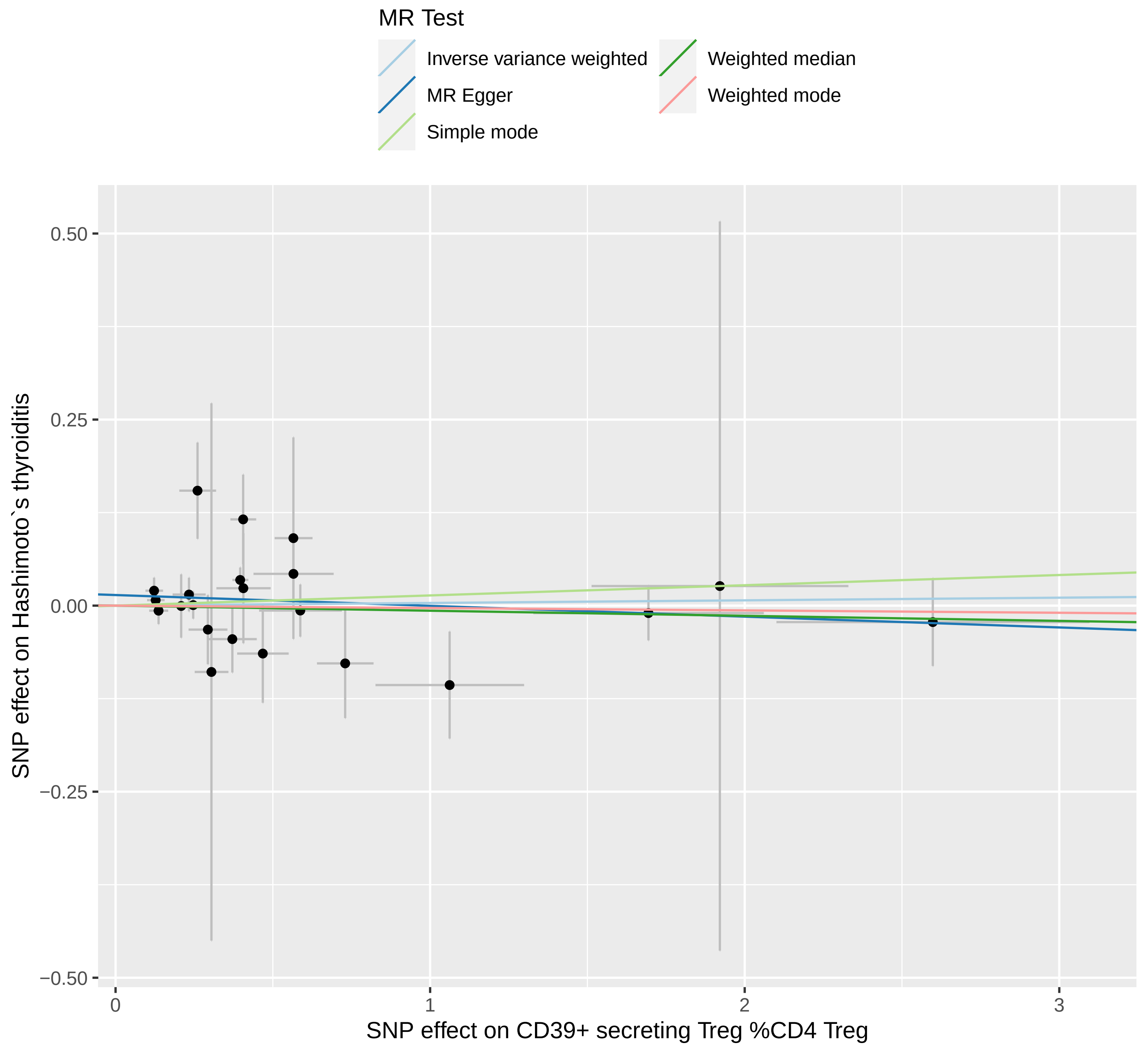

MR Test
Inverse variance weighted
MR Egger
Simple mode
Weighted median
Weighted mode
0.50
0.25
0.00
−0.25
−0.50
SNP effect on Hashimoto`s thyroiditis
0
1
2
3
SNP effect on CD39+ secreting Treg %CD4 Treg

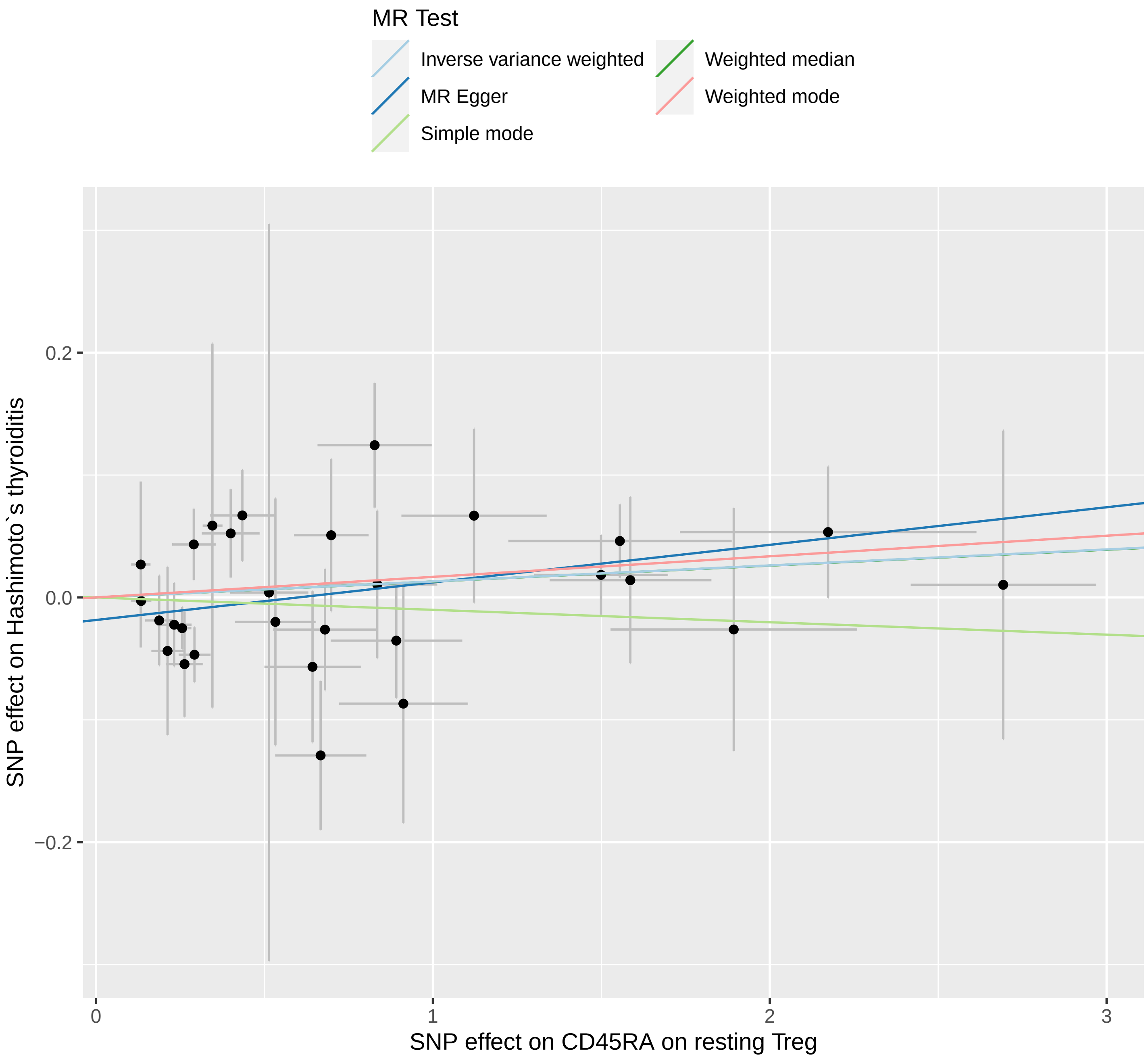

MR Test
Inverse variance weighted
MR Egger
Simple mode
Weighted median
Weighted mode
SNP effect on Hashimoto`s thyroiditis
0.2
0.0
−0.2
0
1
2
3
SNP effect on CD45RA on resting Treg

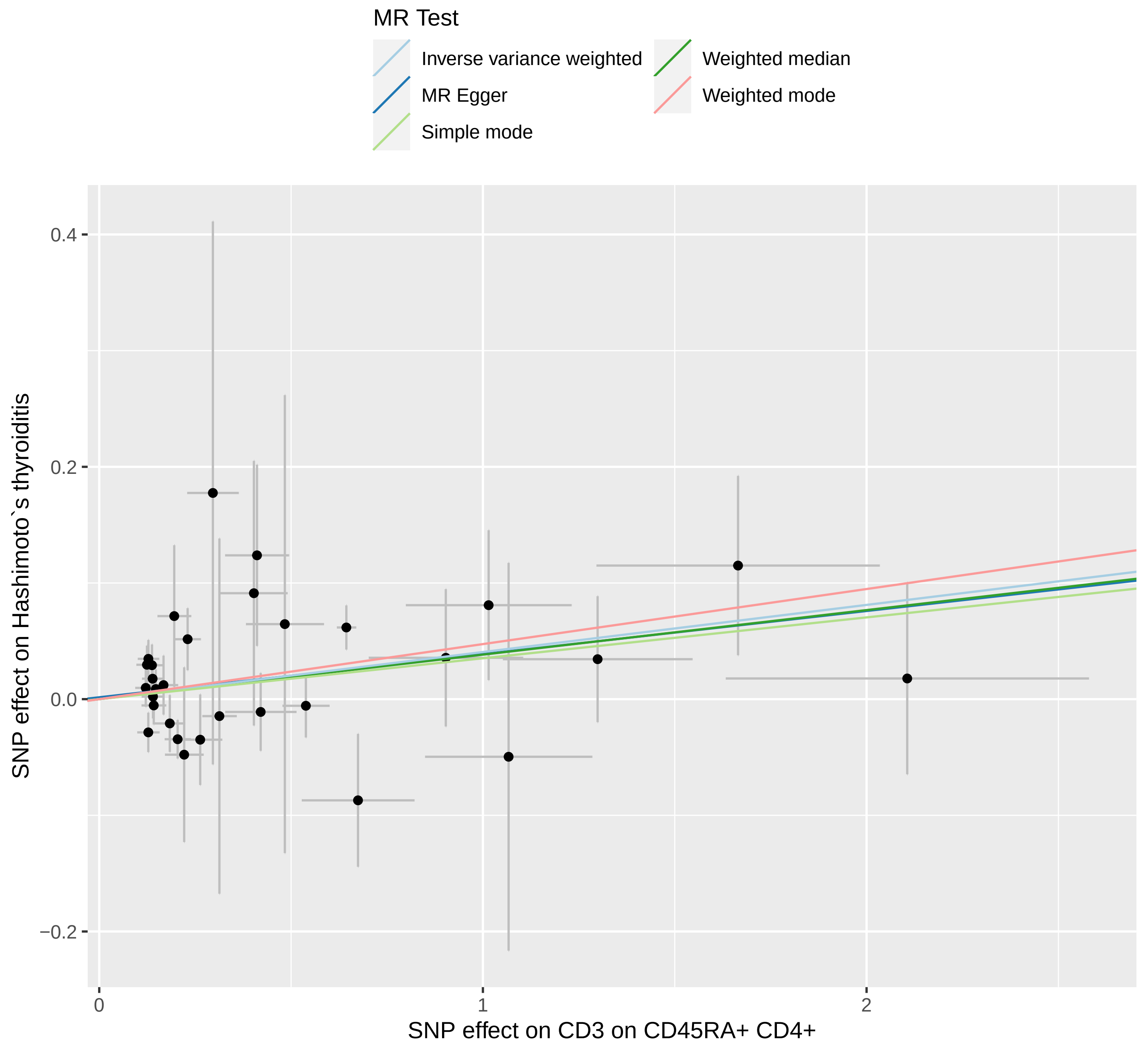

MR Test
Inverse variance weighted
MR Egger
Simple mode
Weighted median
Weighted mode
0.4
0.2
0.0
−0.2
0
1
2
SNP effect on Hashimoto`s thyroiditis
SNP effect on CD3 on CD45RA+ CD4+

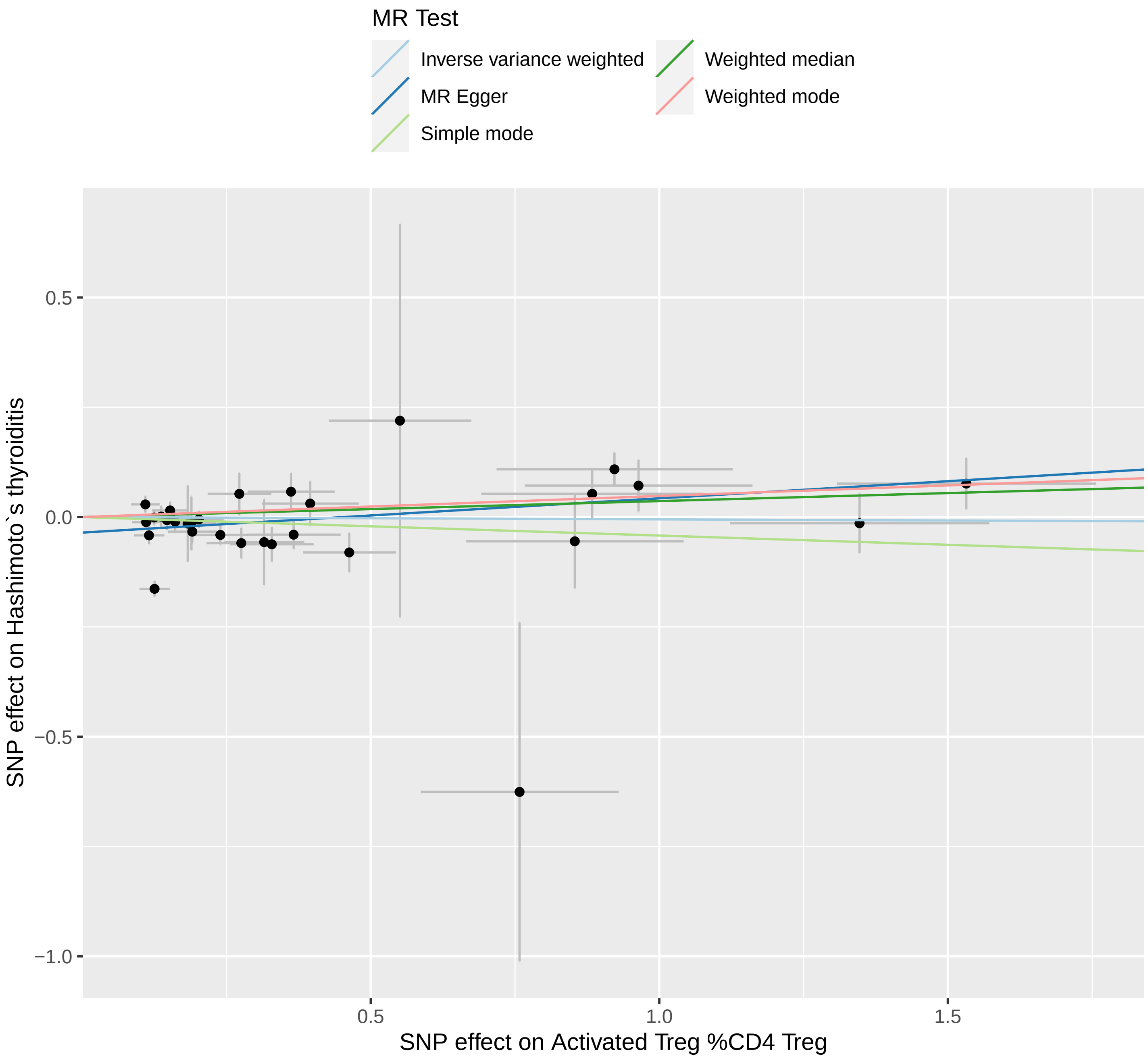
MR Test
Inverse variance weighted
MR Egger
Simple mode
Weighted median
Weighted mode
SNP effect on Hashimoto`s thyroiditis
SNP effect on Activated Treg %CD4 Treg
0.5
0.0
−0.5
−1.0
0.5
1.0
1.5

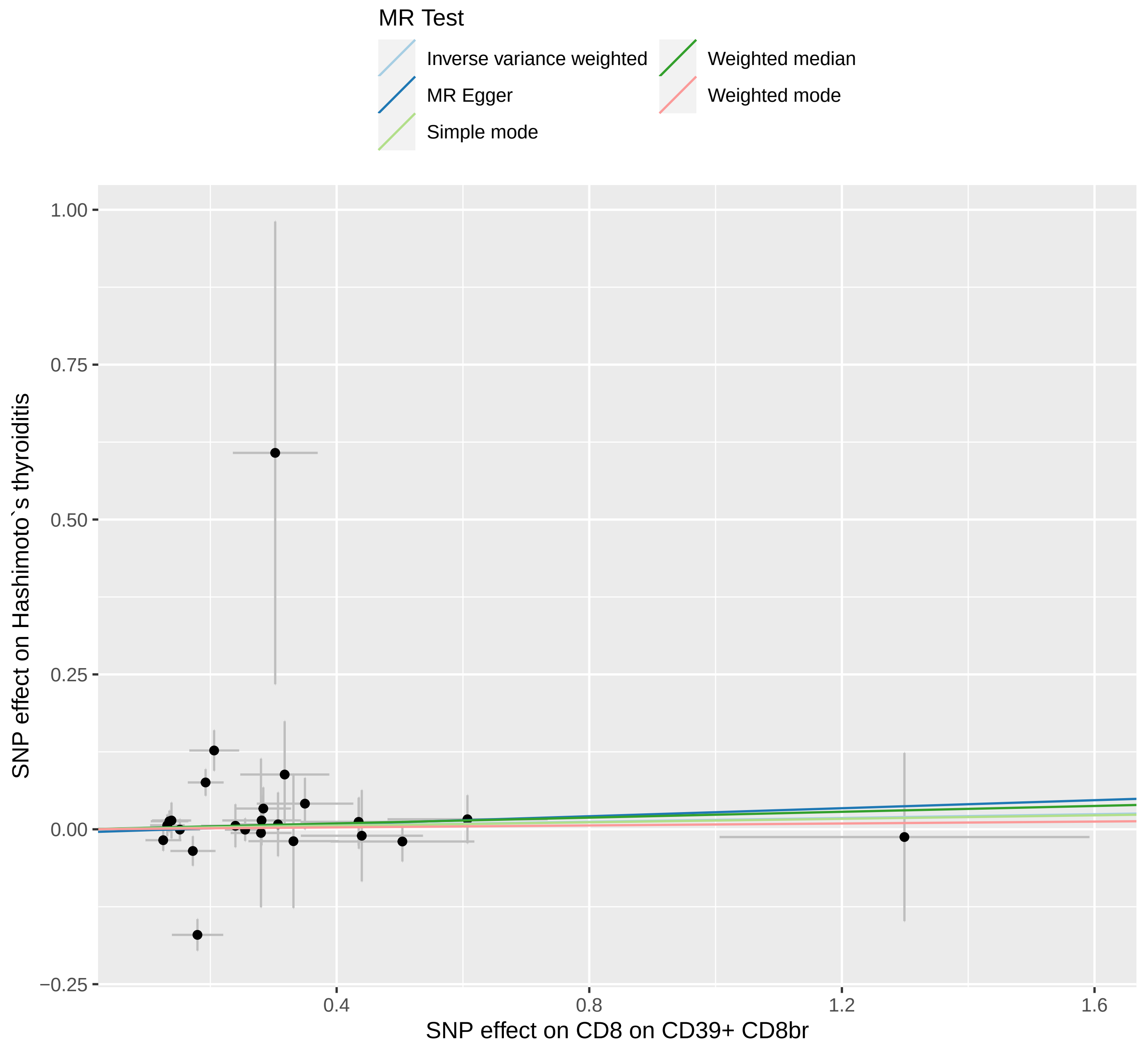
MR Test
Inverse variance weighted
MR Egger
Simple mode
Weighted median
Weighted mode
1.00
0.75
0.50
0.25
0.00
−0.25
0.4
0.8
1.2
1.6
SNP effect on Hashimoto`s thyroiditis
SNP effect on CD8 on CD39+ CD8br

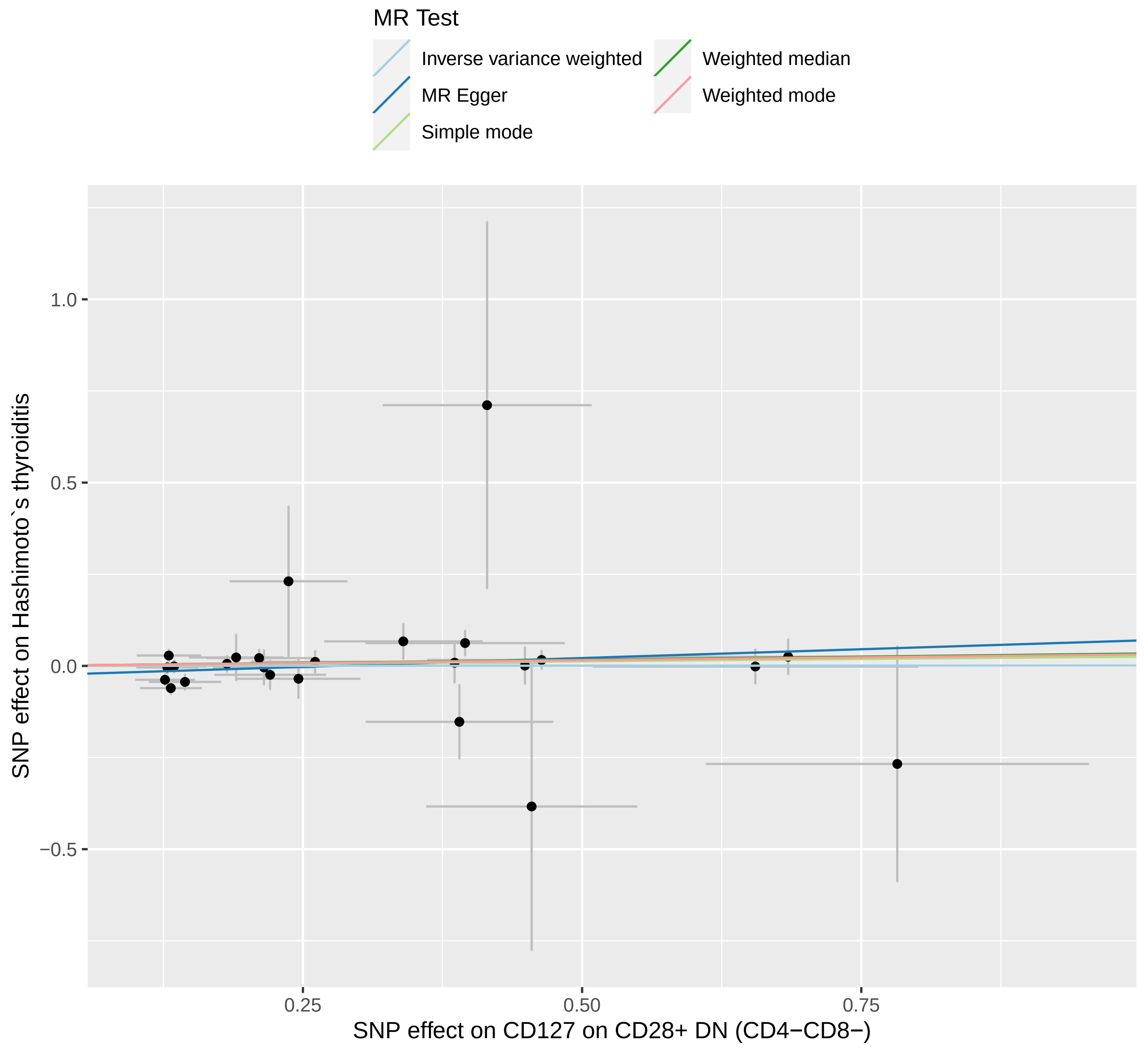

MR Test
Inverse variance weighted
MR Egger
Simple mode
Weighted median
Weighted mode
1.0
0.5
0.0
−0.5
0.25
0.50
0.75
SNP effect on CD127 on CD28+ DN (CD4−CD8−)
SNP effect on Hashimoto`s thyroiditis

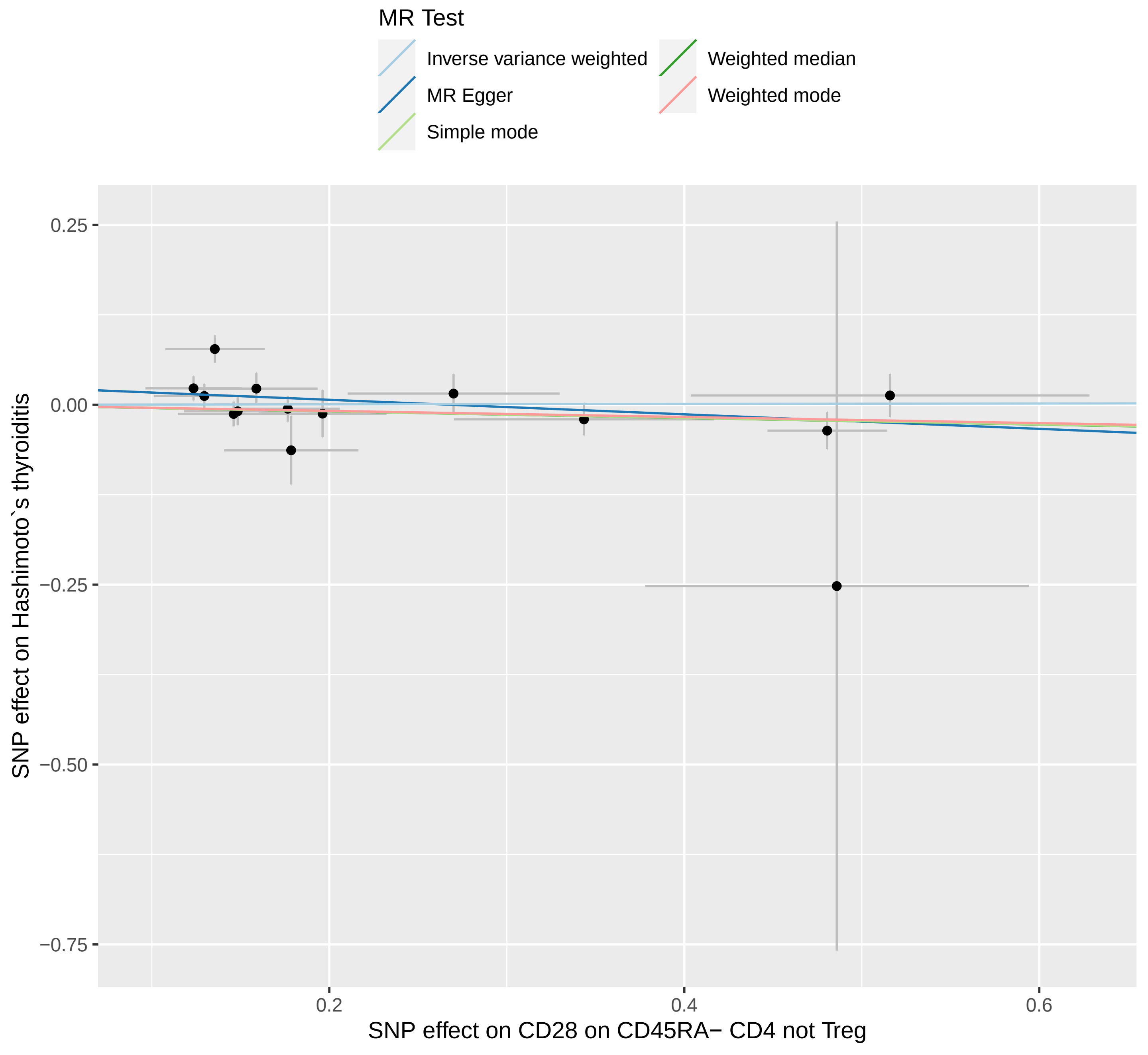

MR Test
Inverse variance weighted
MR Egger
Simple mode
Weighted median
Weighted mode
0.25
0.00
−0.25
−0.50
−0.75
SNP effect on Hashimoto`s thyroiditis
0.2
0.4
0.6
SNP effect on CD28 on CD45RA− CD4 not Treg

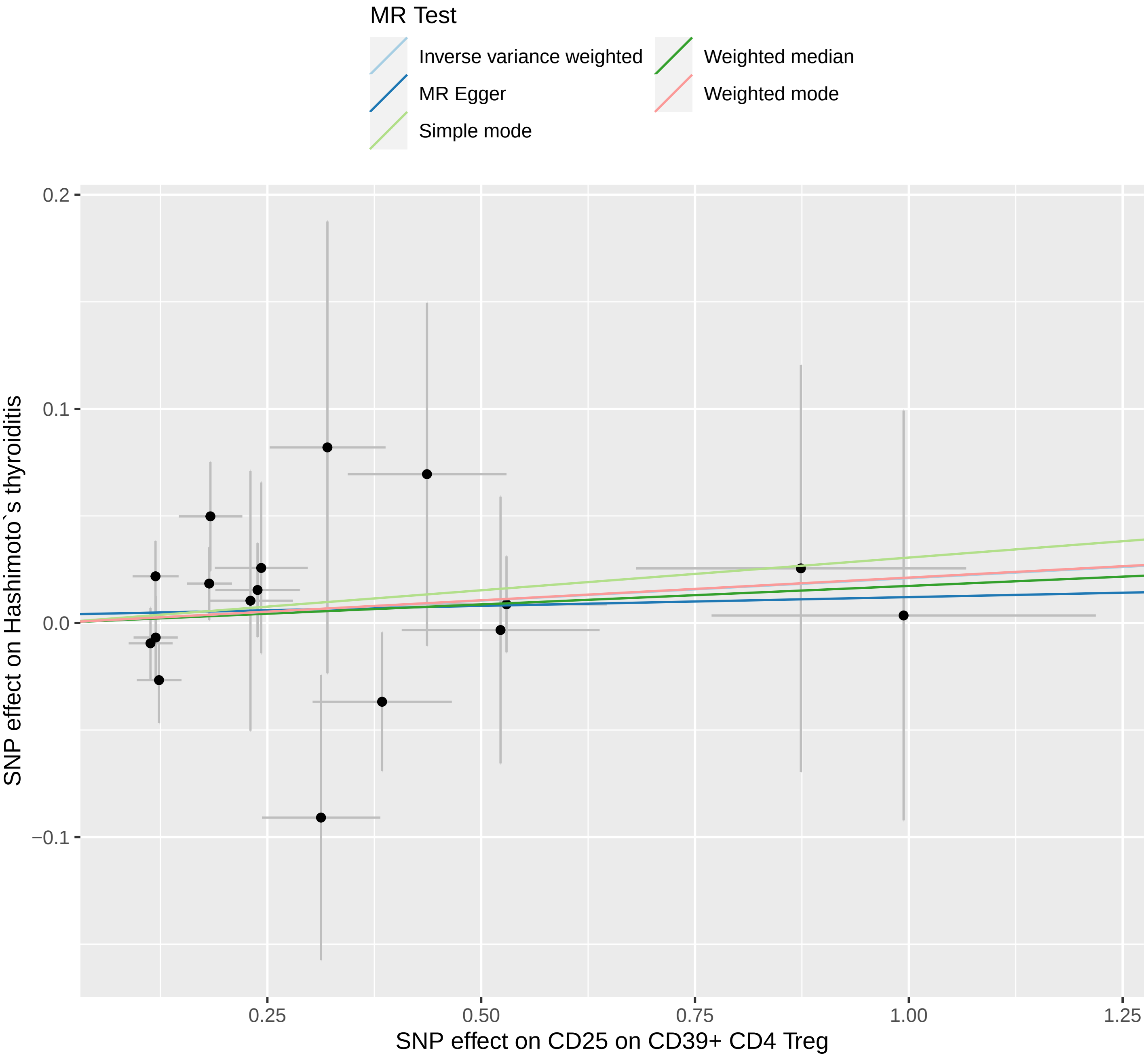
MR Test
Inverse variance weighted
MR Egger
Simple mode
Weighted median
Weighted mode
SNP effect on Hashimoto`s thyroiditis
0.2
0.1
0.0
−0.1
0.25
0.50
0.75
1.00
1.25
SNP effect on CD25 on CD39+ CD4 Treg

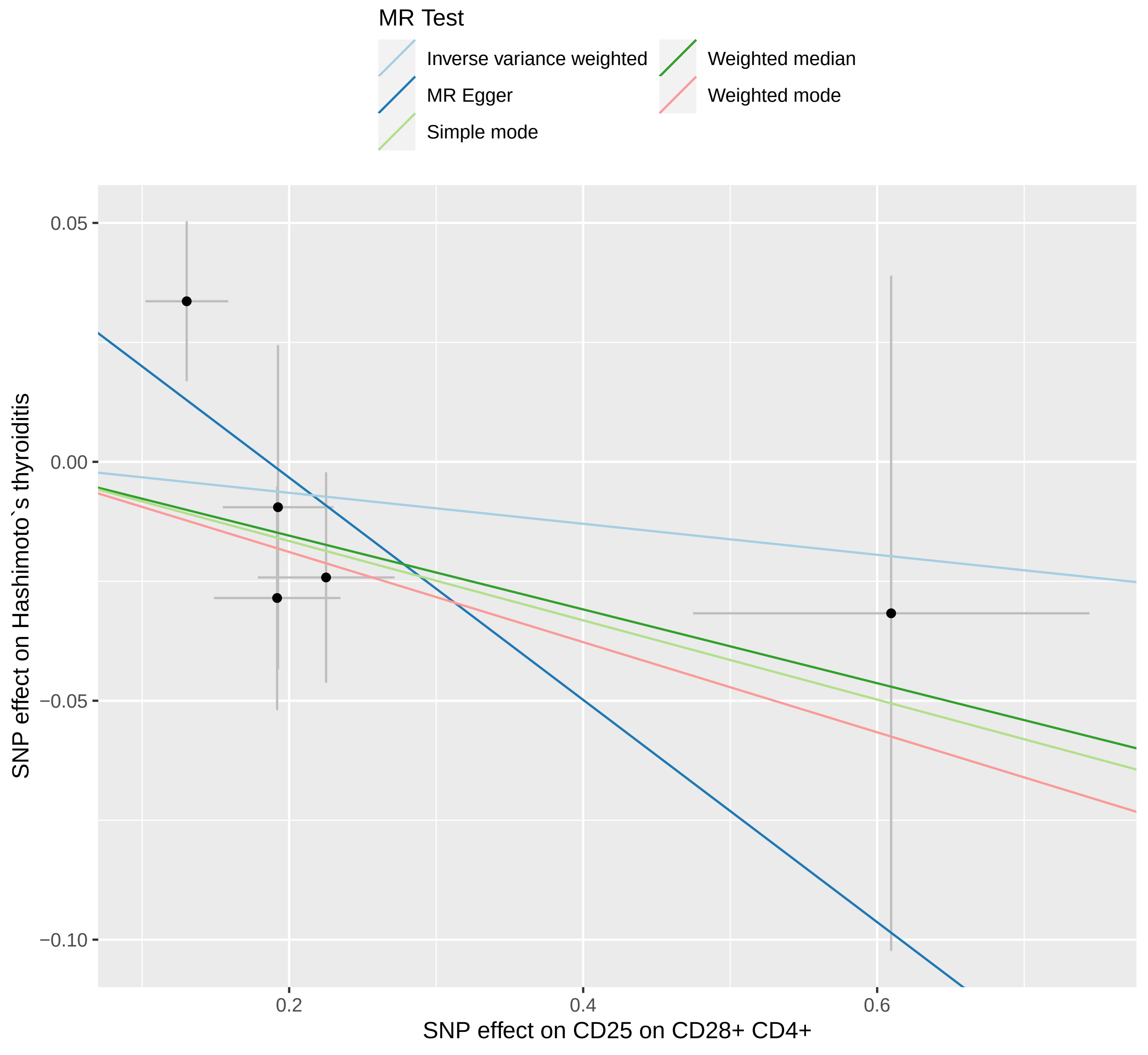
MR Test
Inverse variance weighted
MR Egger
Simple mode
Weighted median
Weighted mode
0.05
0.00
−0.05
−0.10
SNP effect on Hashimoto`s thyroiditis
0.2
0.4
0.6
SNP effect on CD25 on CD28+ CD4+

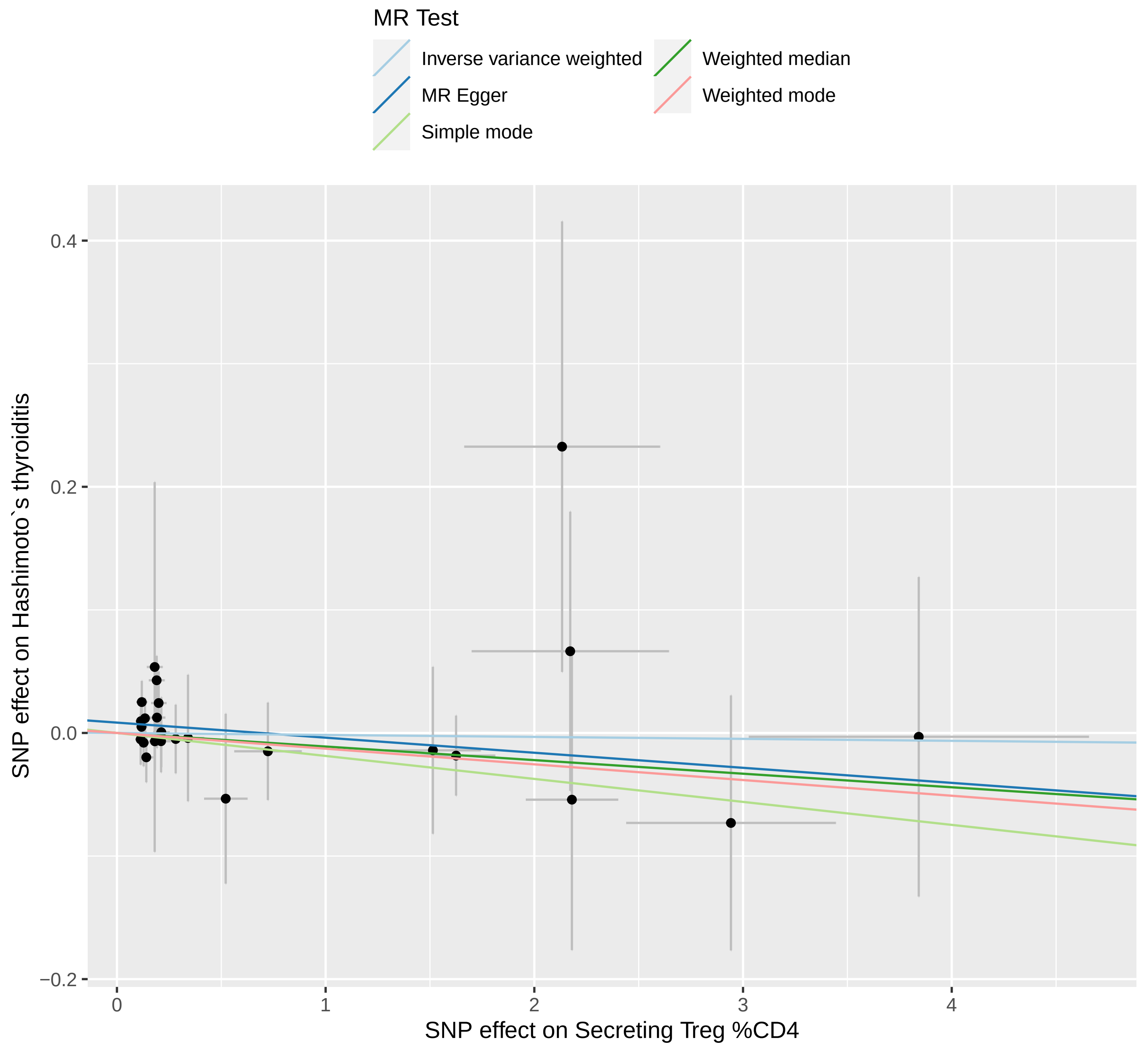

MR Test
Inverse variance weighted
MR Egger
Simple mode
Weighted median
Weighted mode
0.4
0.2
0.0
−0.2
0
1
2
3
4
SNP effect on Hashimoto`s thyroiditis
SNP effect on Secreting Treg %CD4

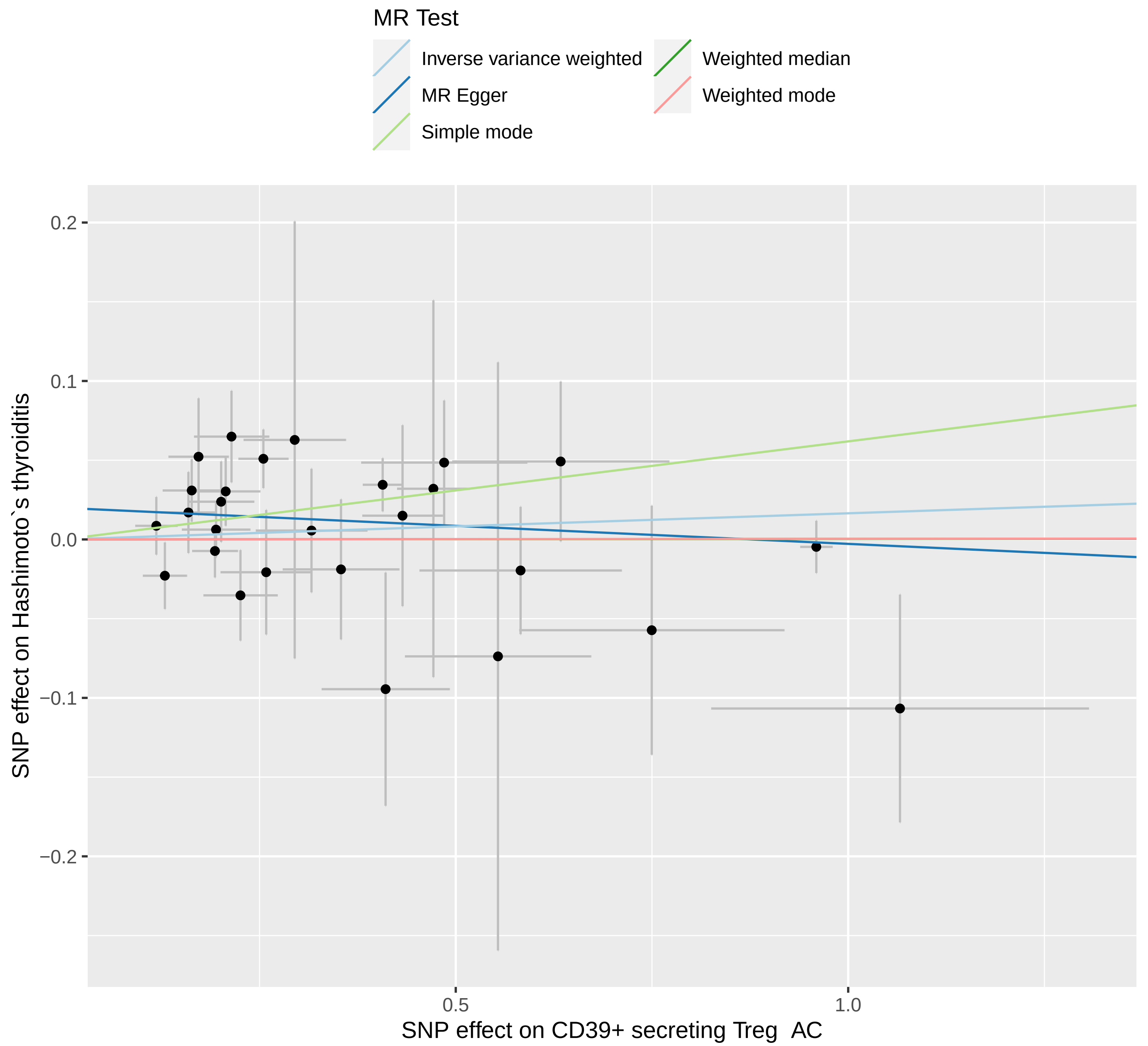
MR Test
Inverse variance weighted
MR Egger
Simple mode
Weighted median
Weighted mode
0.2
0.1
0.0
−0.1
−0.2
0.5
1.0
SNP effect on Hashimoto`s thyroiditis
SNP effect on CD39+ secreting Treg AC

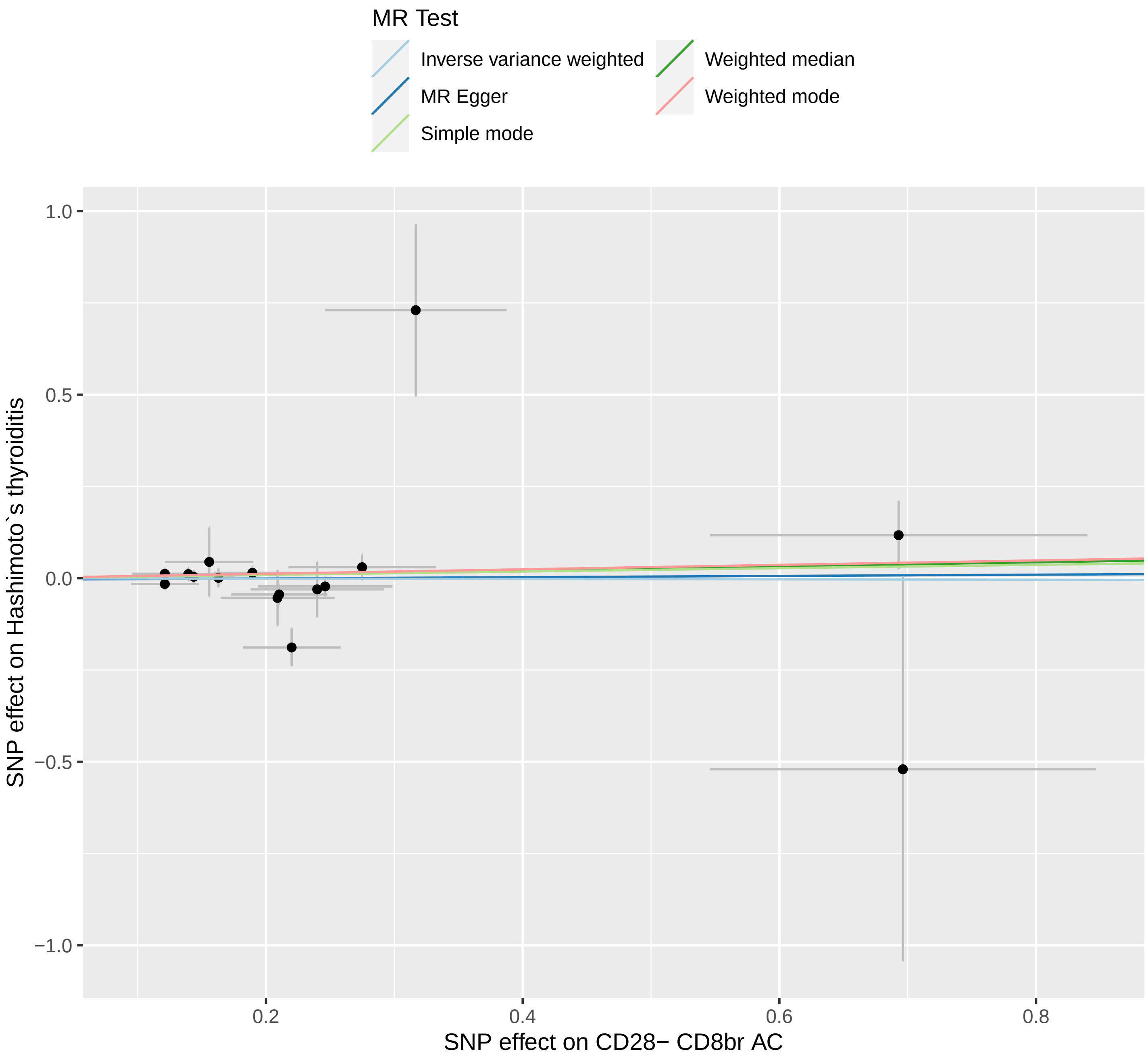

MR Test
Inverse variance weighted
MR Egger
Simple mode
Weighted median
Weighted mode
1.0
0.5
0.0
−0.5
−1.0
SNP effect on Hashimoto`s thyroiditis
0.2
0.4
0.6
0.8
SNP effect on CD28− CD8br AC

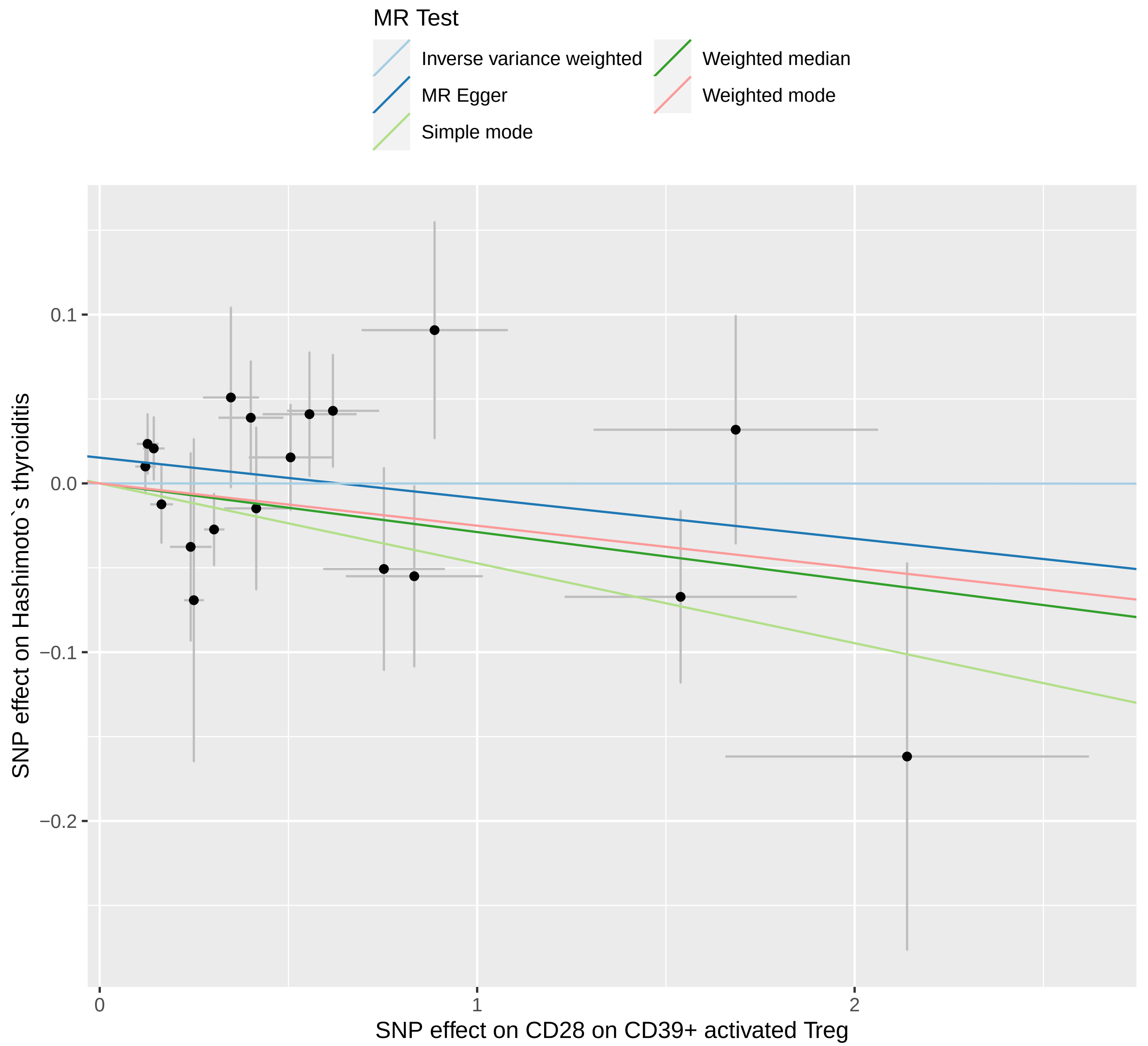

MR Test
Inverse variance weighted
MR Egger
Simple mode
Weighted median
Weighted mode
0.1
0.0
−0.1
−0.2
SNP effect on Hashimoto`s thyroiditis
0
1
2
SNP effect on CD28 on CD39+ activated Treg

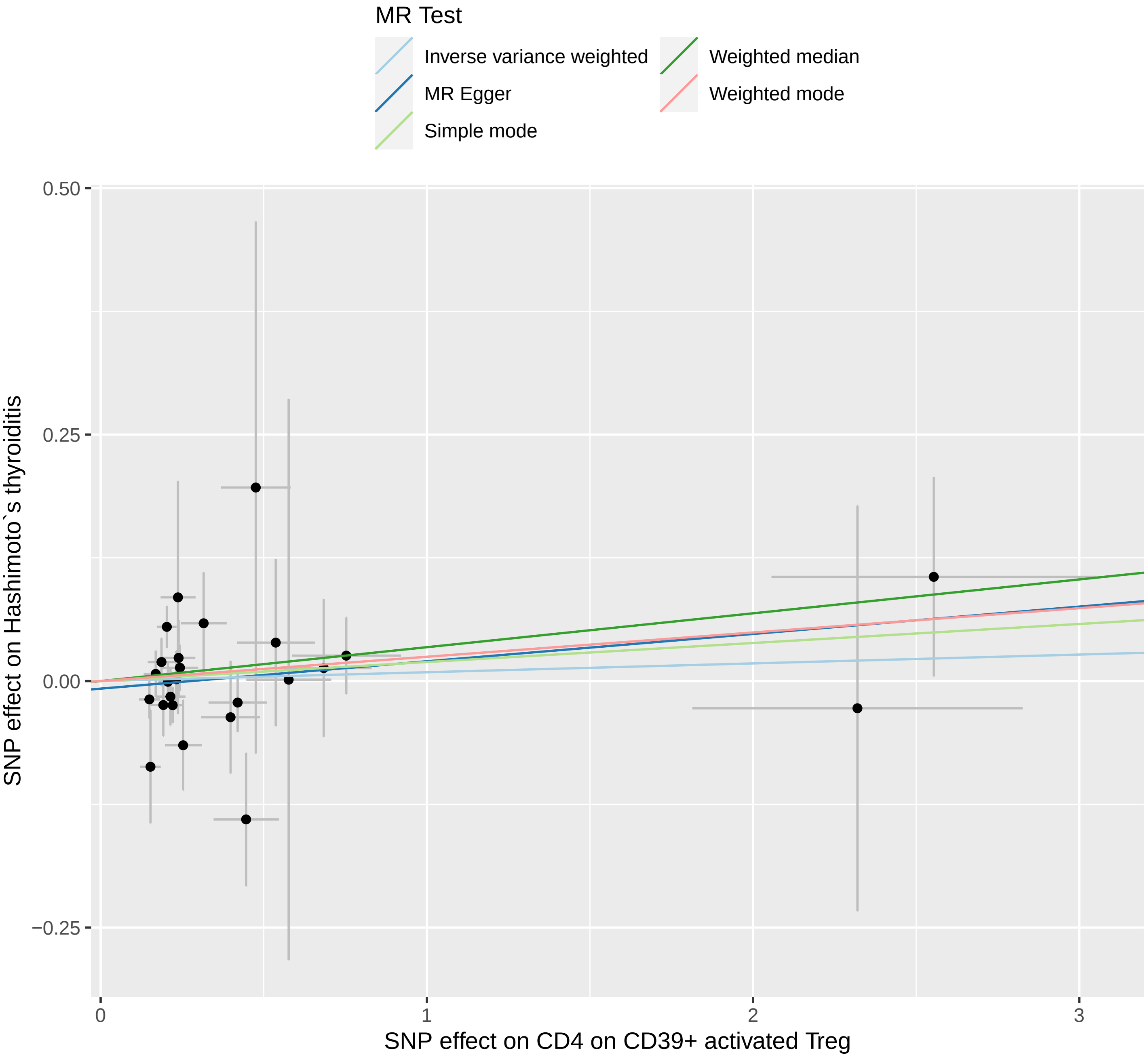
MR Test
Inverse variance weighted
MR Egger
Simple mode
Weighted median
Weighted mode
0.50
0.25
0.00
−0.25
0
1
2
3
SNP effect on Hashimoto`s thyroiditis
SNP effect on CD4 on CD39+ activated Treg

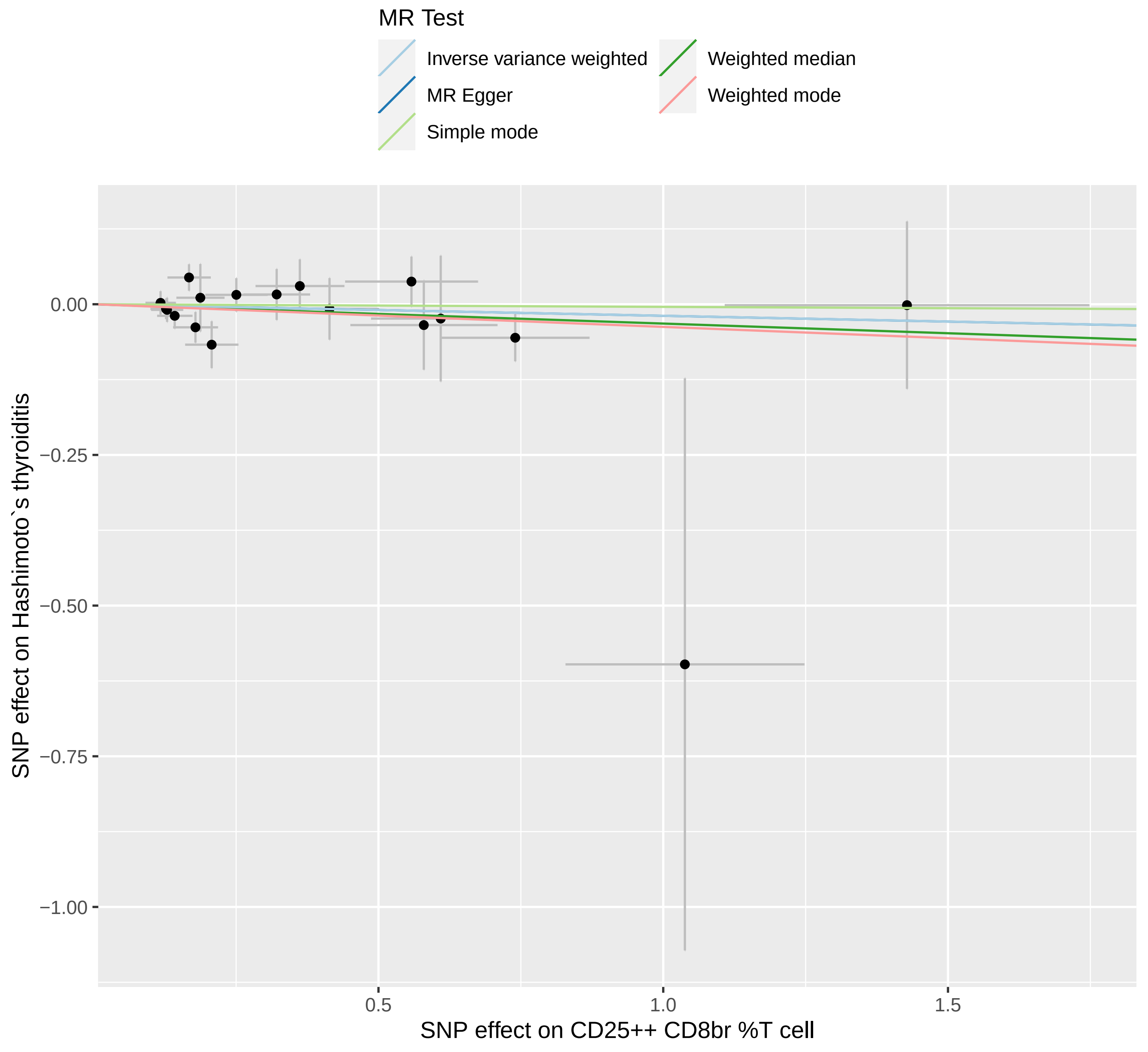

MR Test
Inverse variance weighted
MR Egger
Simple mode
Weighted median
Weighted mode
SNP effect on Hashimoto`s thyroiditis
0.00
−0.25
−0.50
−0.75
−1.00
0.5
1.0
1.5
SNP effect on CD25++ CD8br %T cell

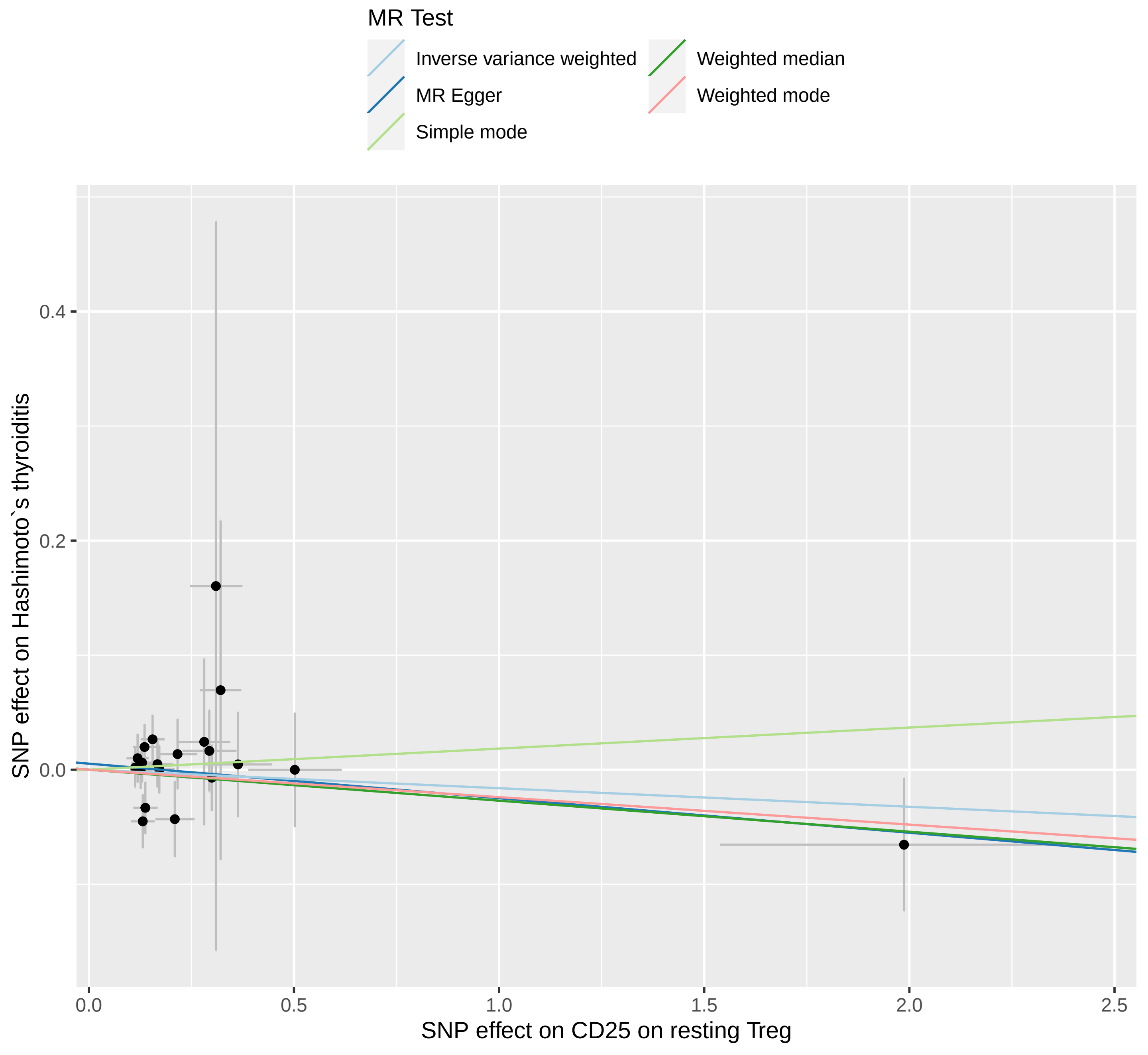
MR Test
Inverse variance weighted
MR Egger
Simple mode
Weighted median
Weighted mode
SNP effect on Hashimoto`s thyroiditis
0.4
0.2
0.0
SNP effect on CD25 on resting Treg
0.0
0.5
1.0
1.5
2.0
2.5

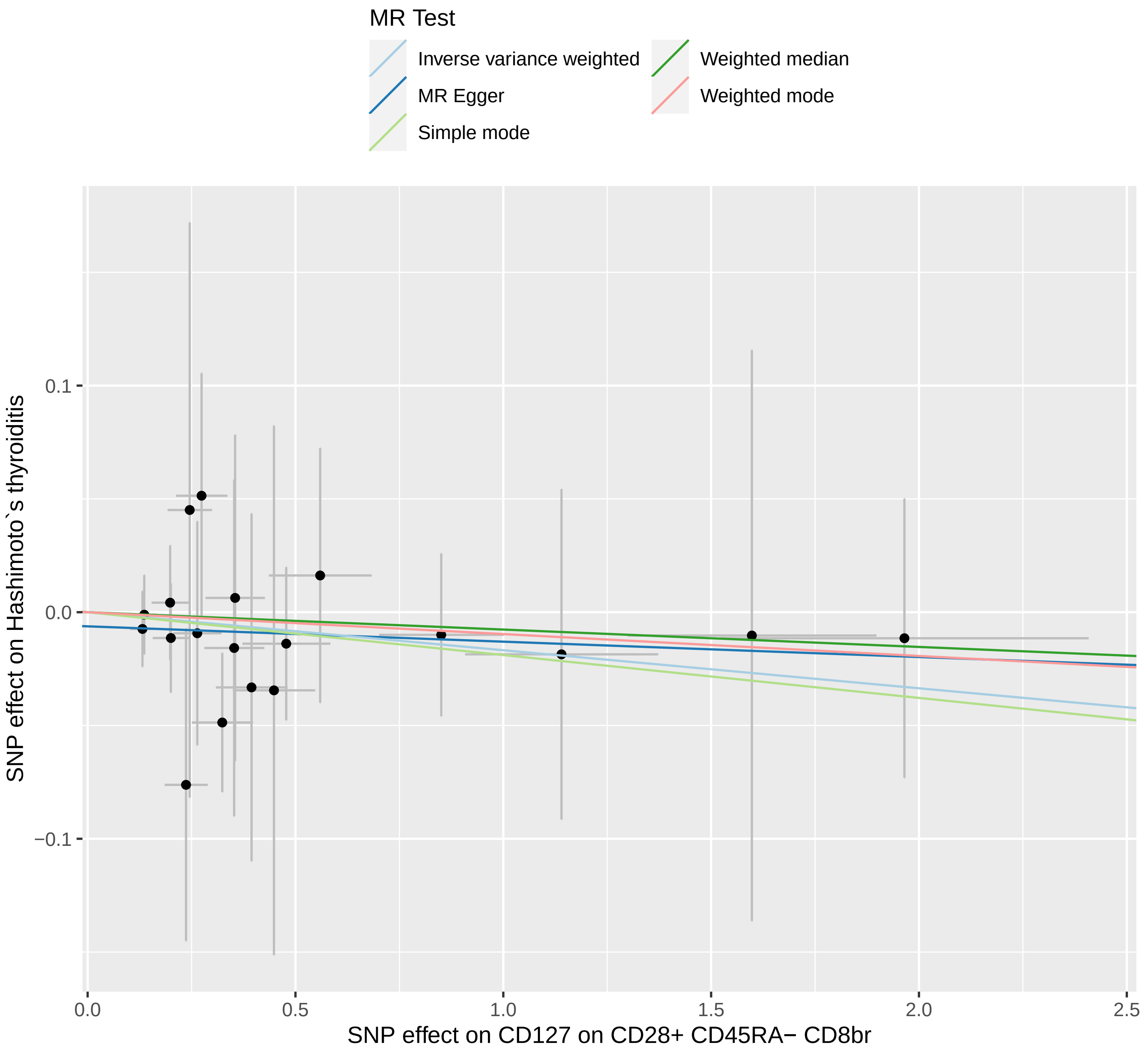
MR Test
Inverse variance weighted
MR Egger
Simple mode
Weighted median
Weighted mode
SNP effect on Hashimoto`s thyroiditis
0.1
0.0
−0.1
0.0
0.5
1.0
1.5
2.0
2.5
SNP effect on CD127 on CD28+ CD45RA− CD8br

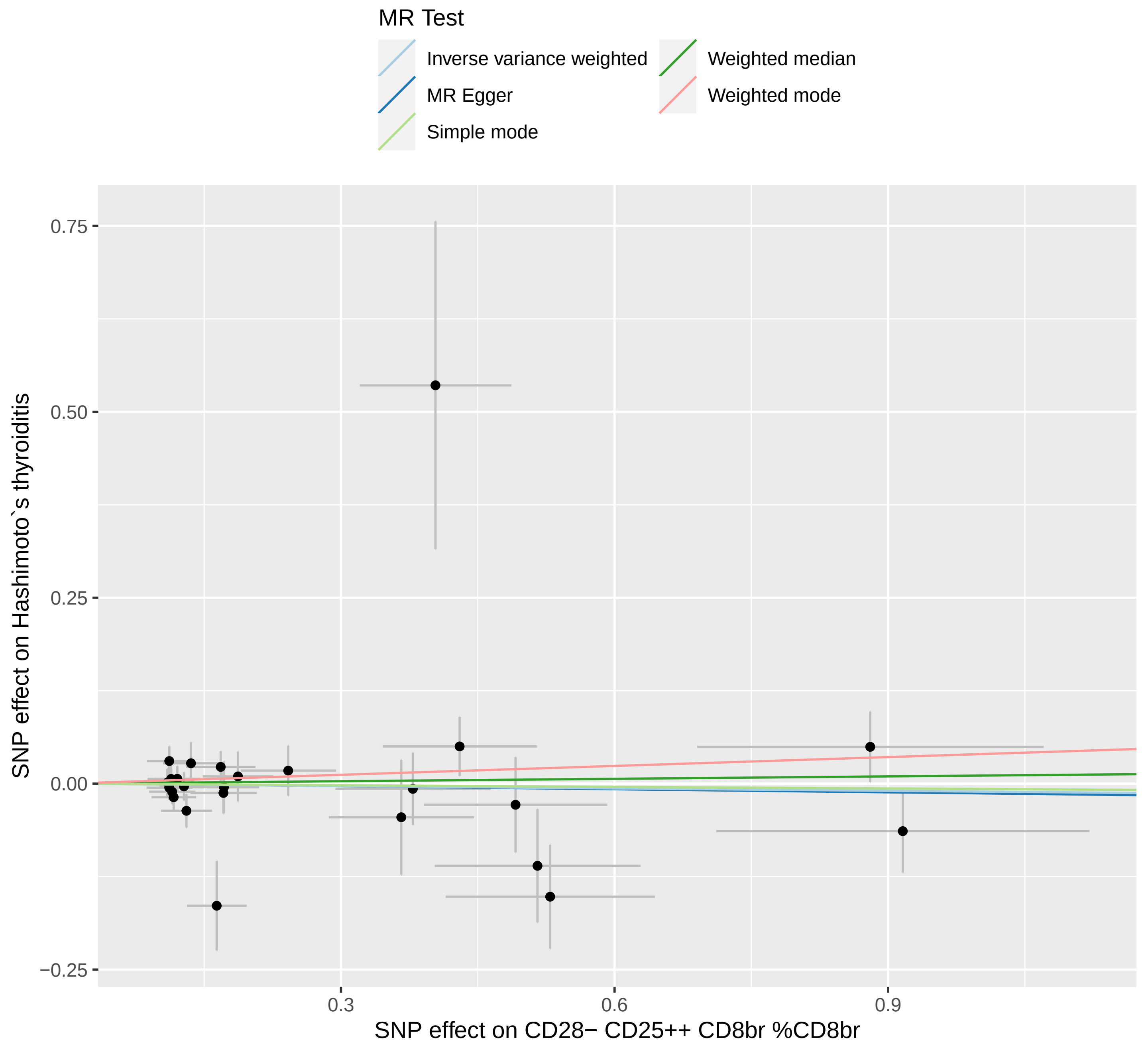
MR Test
Inverse variance weighted
MR Egger
Simple mode
Weighted median
Weighted mode
0.75
0.50
0.25
0.00
−0.25
0.3
0.6
0.9
SNP effect on Hashimoto`s thyroiditis
SNP effect on CD28− CD25++ CD8br %CD8br

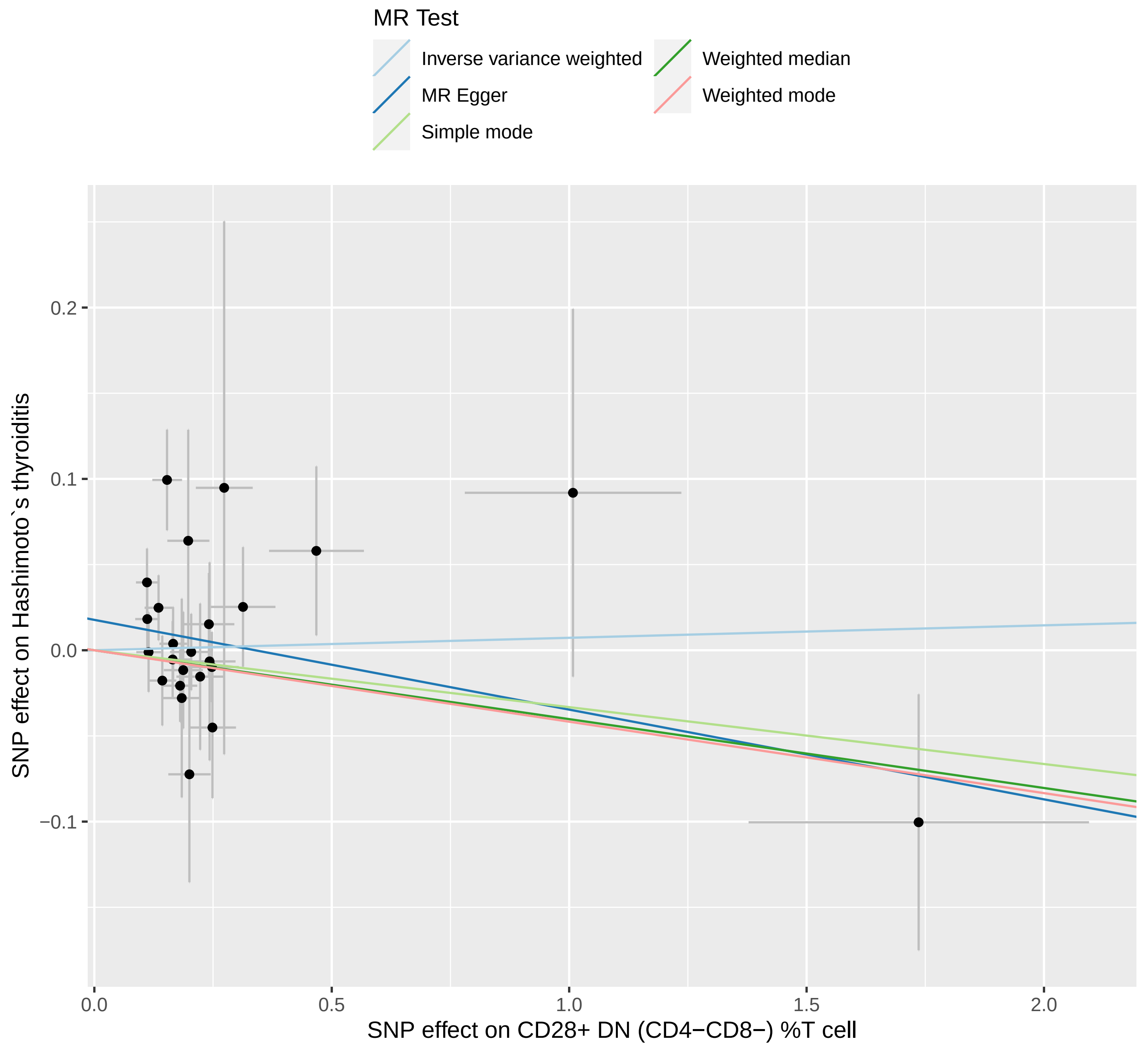

MR Test
Inverse variance weighted
MR Egger
Simple mode
Weighted median
Weighted mode
0.2
0.1
0.0
−0.1
SNP effect on Hashimoto`s thyroiditis
0.0
0.5
1.0
1.5
2.0
SNP effect on CD28+ DN (CD4−CD8−) %T cell

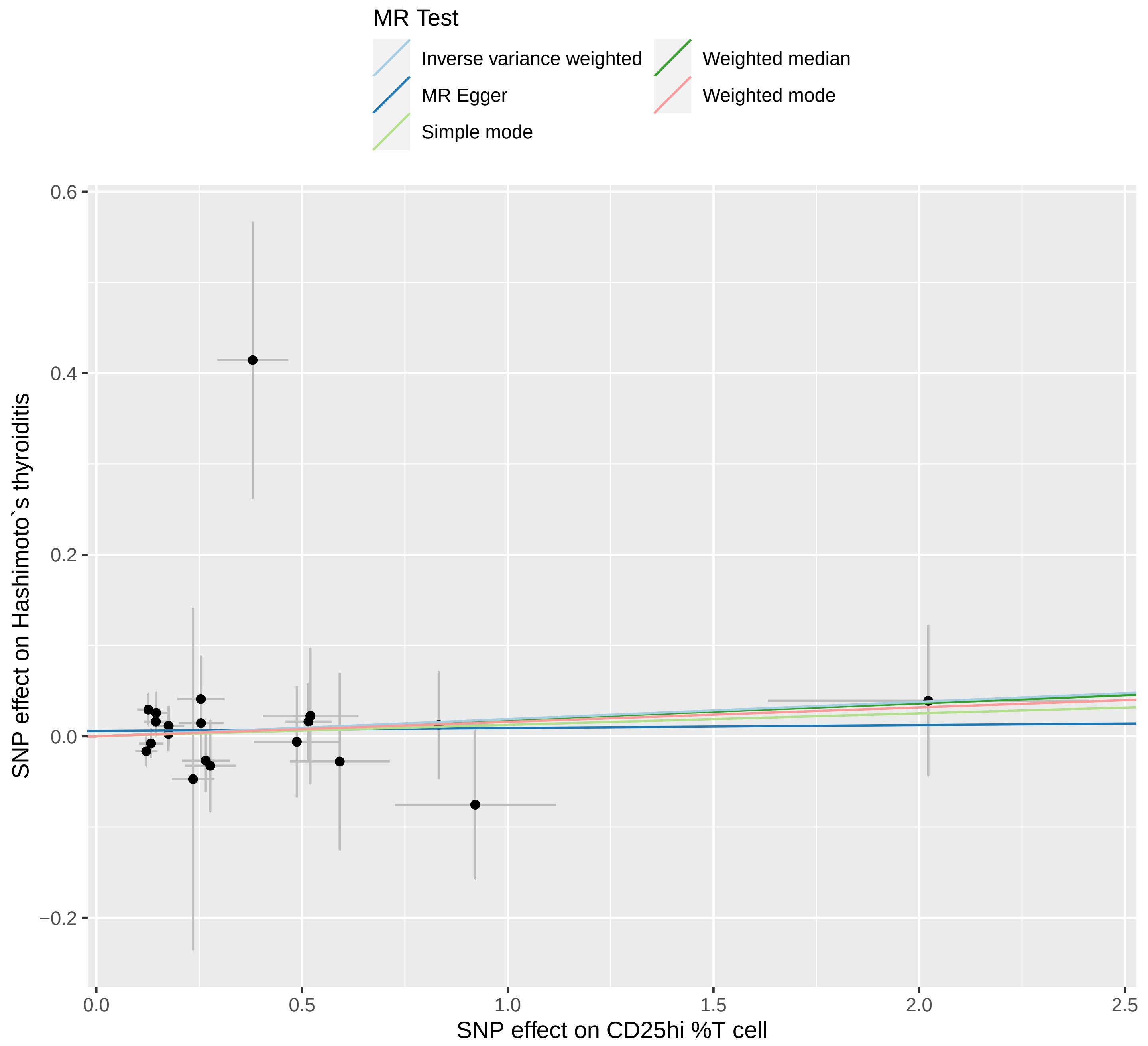
MR Test
Inverse variance weighted
Weighted median
MR Egger
Weighted mode
Simple mode
0.6
0.4
0.2
0.0
−0.2
0.0
0.5
1.0
1.5
2.0
2.5
SNP effect on Hashimoto`s thyroiditis
SNP effect on CD25hi %T cell

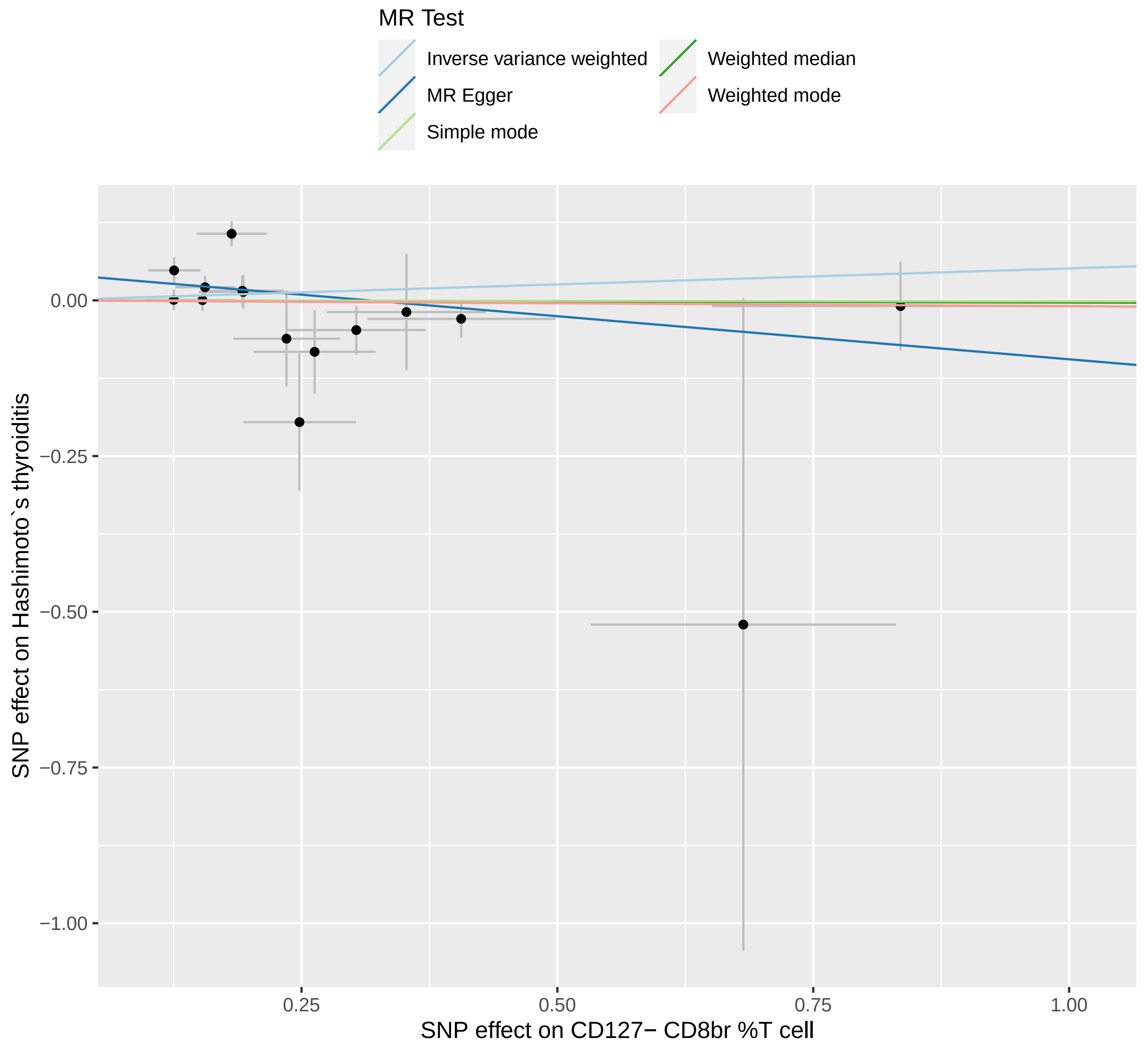
MR Test
Inverse variance weighted
MR Egger
Simple mode
Weighted median
Weighted mode
SNP effect on Hashimoto`s thyroiditis
0.00
−0.25
−0.50
−0.75
−1.00
0.25
0.50
0.75
1.00
SNP effect on CD127− CD8br %T cell

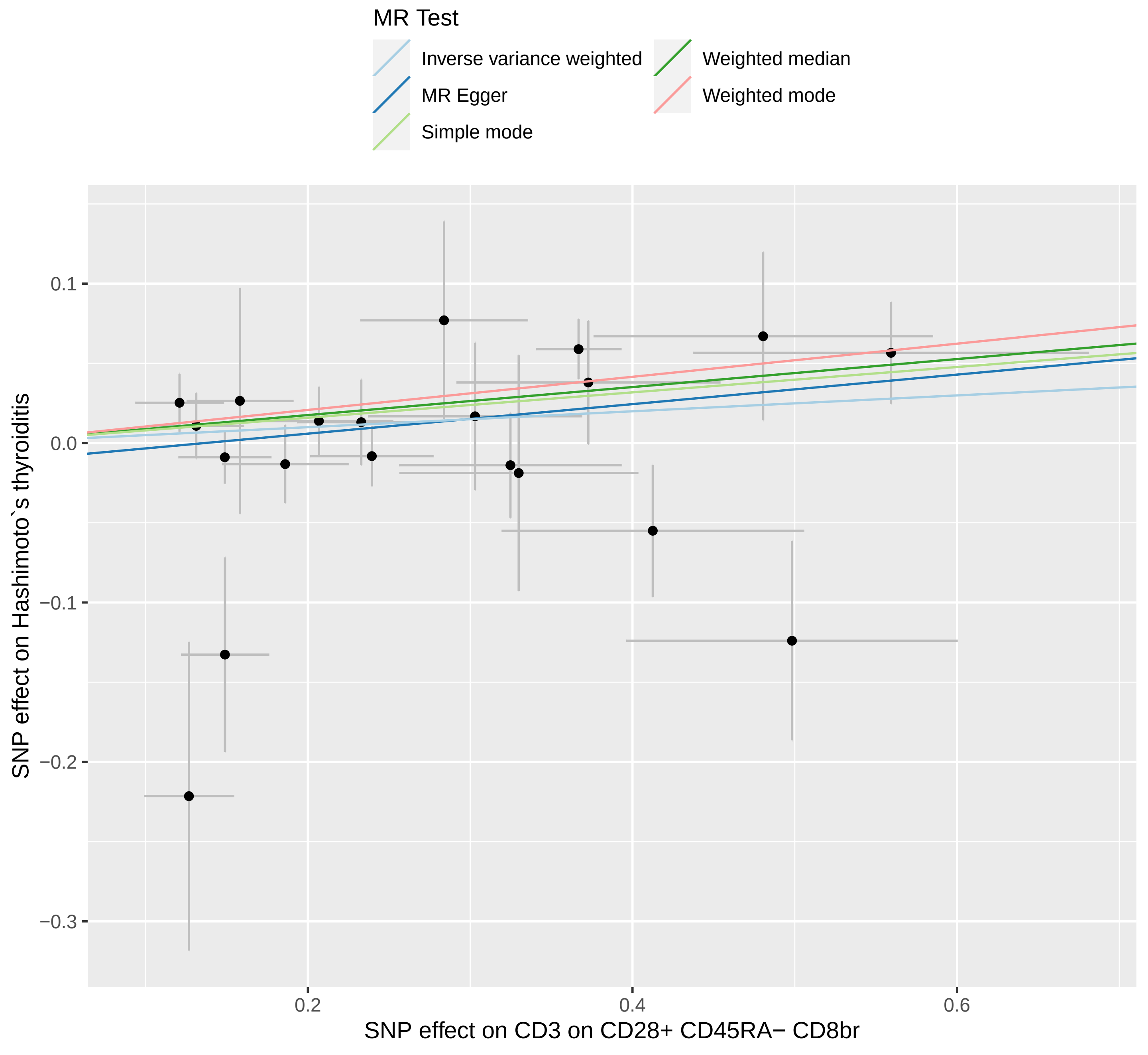

MR Test
Inverse variance weighted
MR Egger
Simple mode
Weighted median
Weighted mode
SNP effect on Hashimoto`s thyroiditis
0.1
0.0
−0.1
−0.2
−0.3
0.2
0.4
0.6
SNP effect on CD3 on CD28+ CD45RA− CD8br

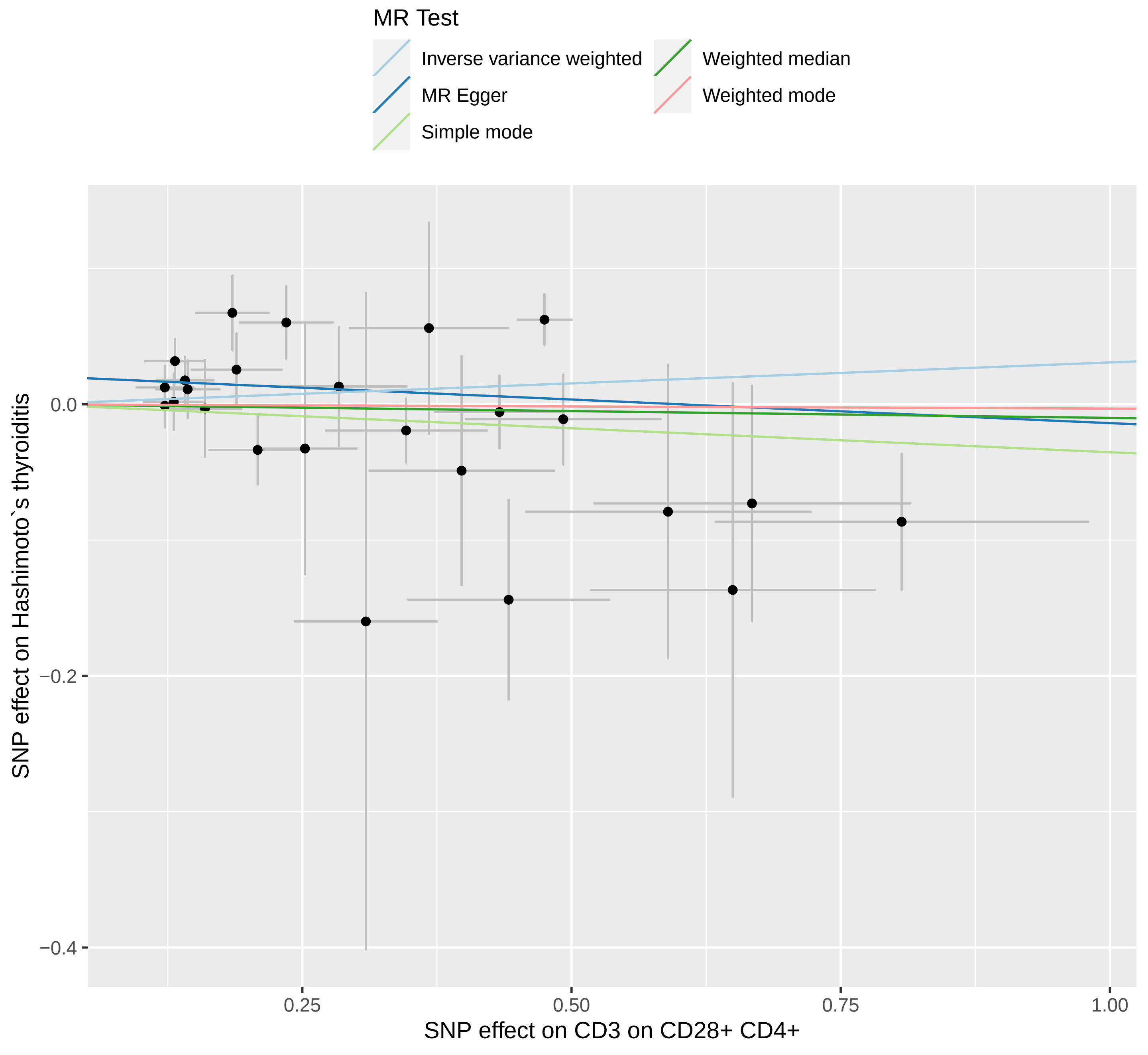

MR Test
Inverse variance weighted
MR Egger
Simple mode
Weighted median
Weighted mode
SNP effect on Hashimoto`s thyroiditis
0.0
−0.2
−0.4
0.25
0.50
0.75
1.00
SNP effect on CD3 on CD28+ CD4+

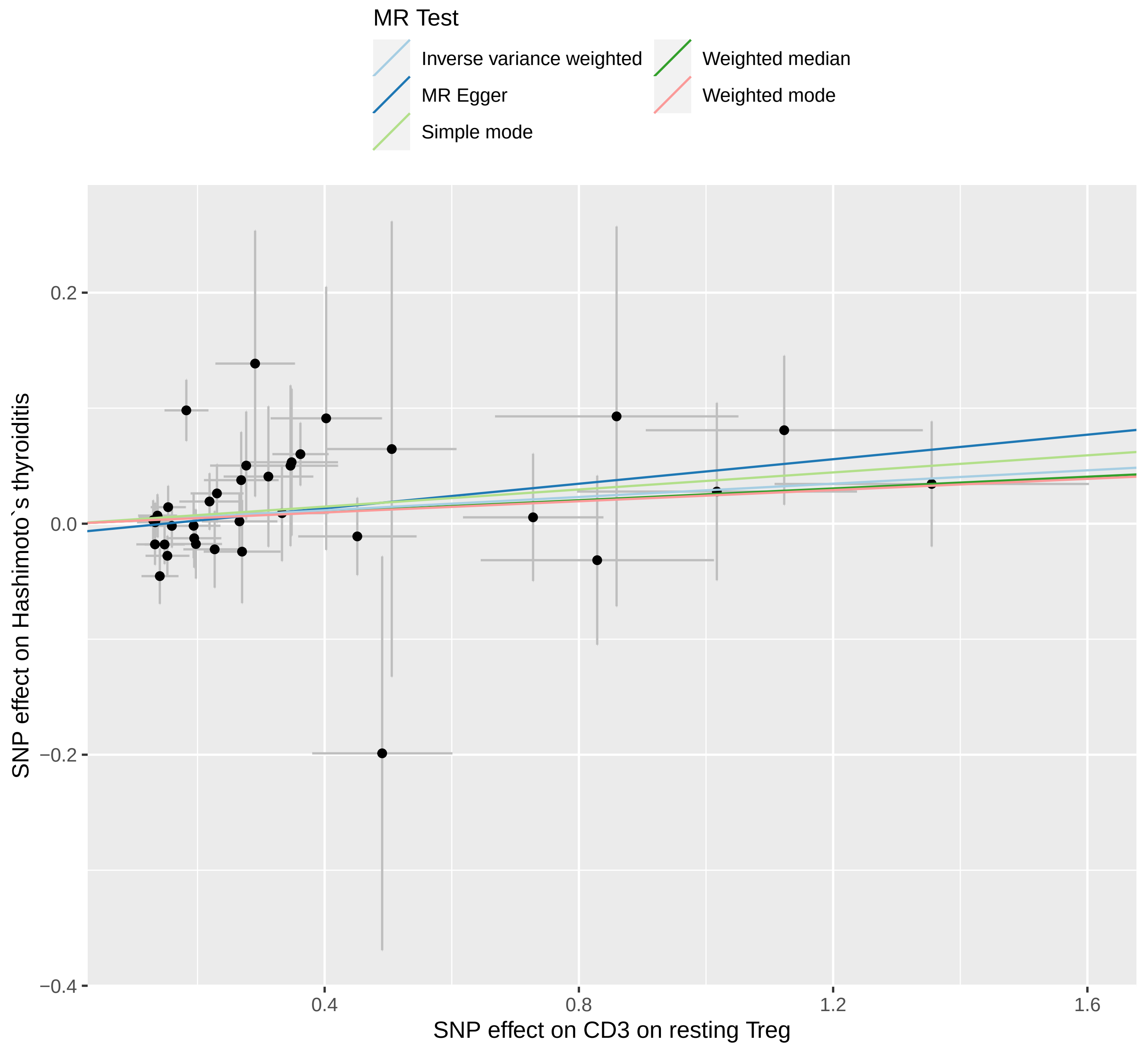
MR Test
Inverse variance weighted
MR Egger
Simple mode
Weighted median
Weighted mode
0.2
0.0
−0.2
−0.4
0.4
0.8
1.2
1.6
SNP effect on Hashimoto`s thyroiditis
SNP effect on CD3 on resting Treg

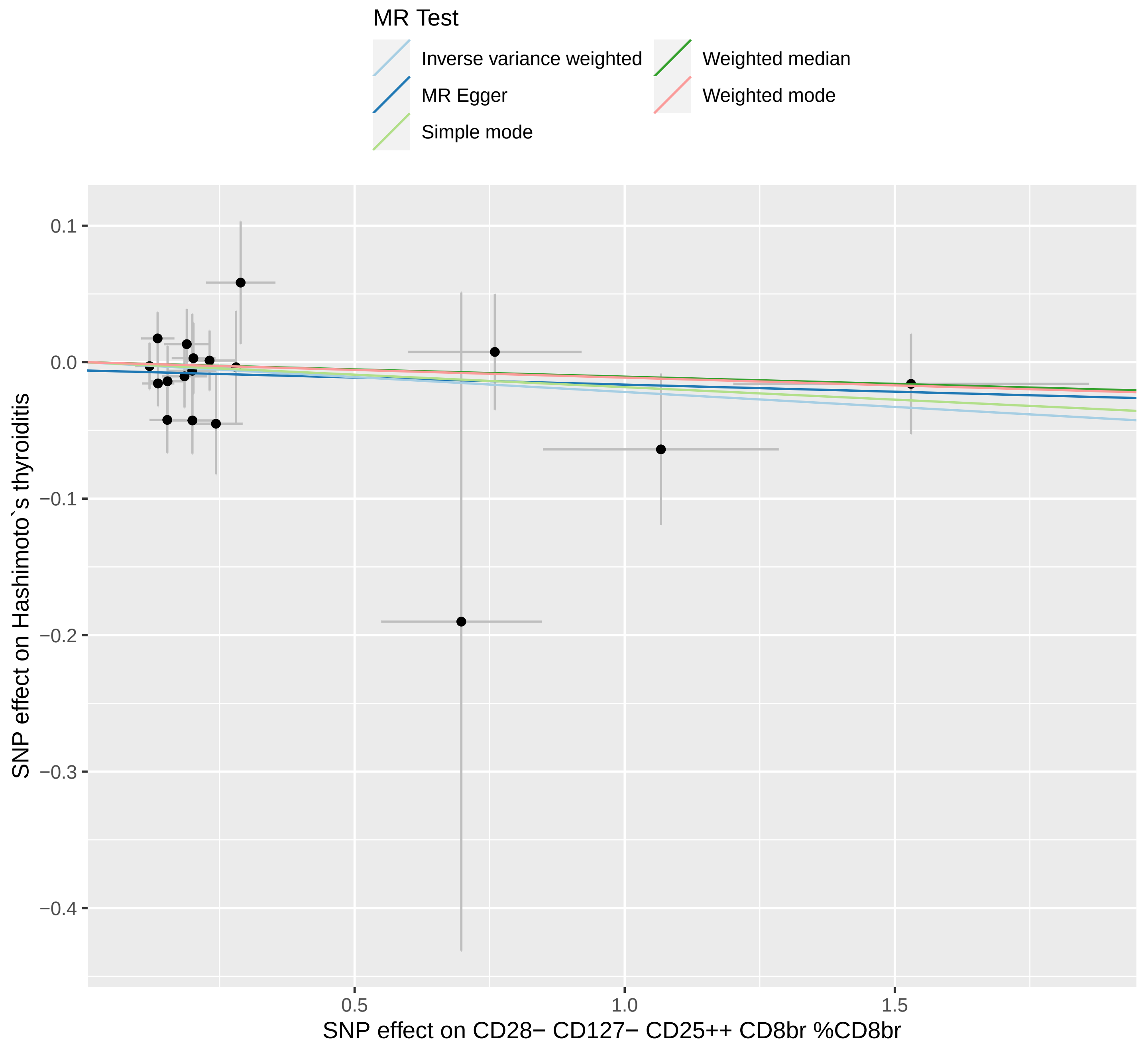

MR Test
Inverse variance weighted
MR Egger
Simple mode
Weighted median
Weighted mode
0.1
0.0
−0.1
−0.2
−0.3
−0.4
SNP effect on Hashimoto`s thyroiditis
0.5
1.0
1.5
SNP effect on CD28− CD127− CD25++ CD8br %CD8br

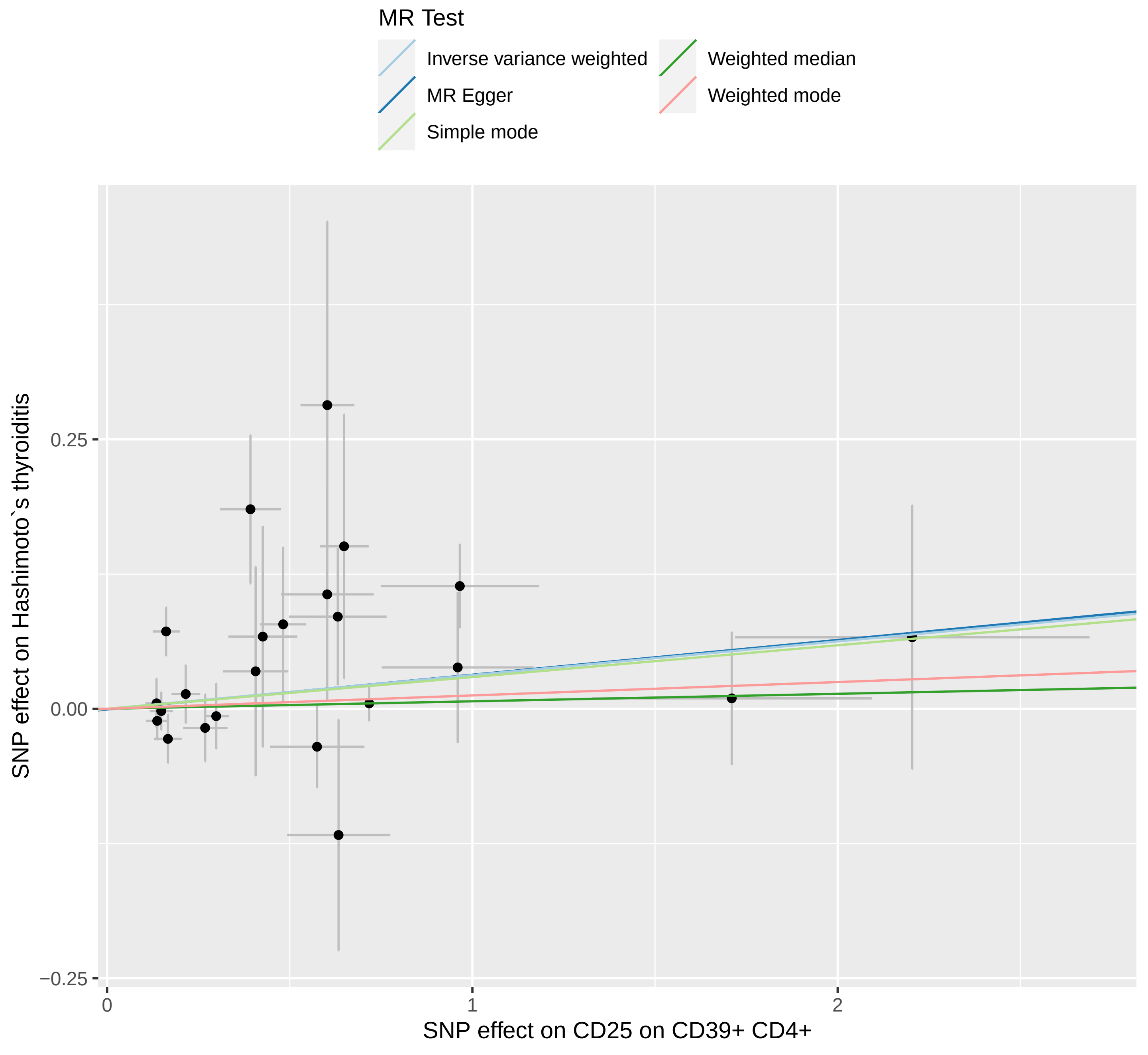

MR Test
Inverse variance weighted
MR Egger
Simple mode
Weighted median
Weighted mode
SNP effect on Hashimoto`s thyroiditis
0.25
0.00
−0.25
0
1
2
SNP effect on CD25 on CD39+ CD4+

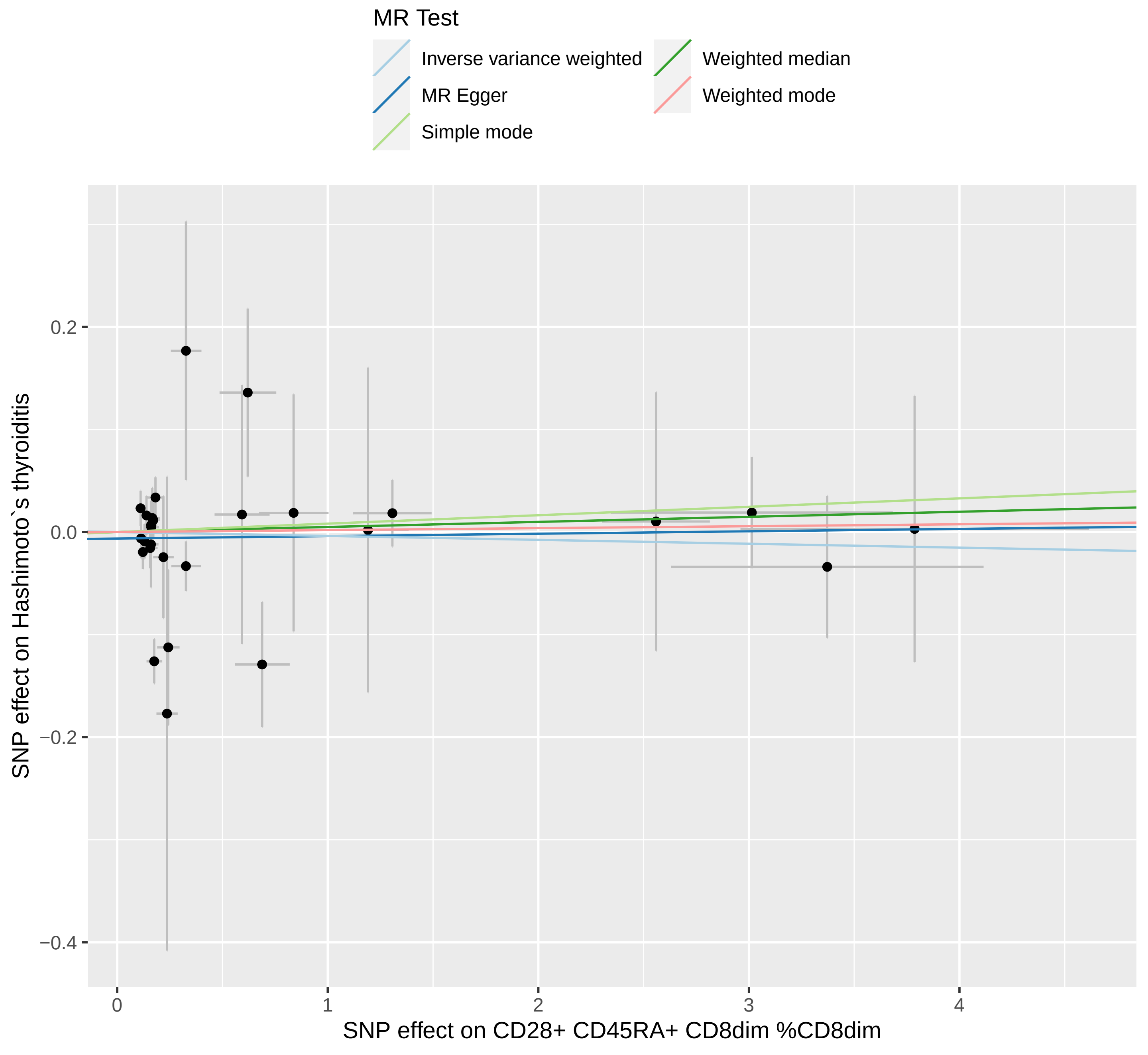

MR Test
Inverse variance weighted
MR Egger
Simple mode
Weighted median
Weighted mode
SNP effect on Hashimoto`s thyroiditis
0.2
0.0
−0.2
−0.4
0
1
2
3
4
SNP effect on CD28+ CD45RA+ CD8dim %CD8dim

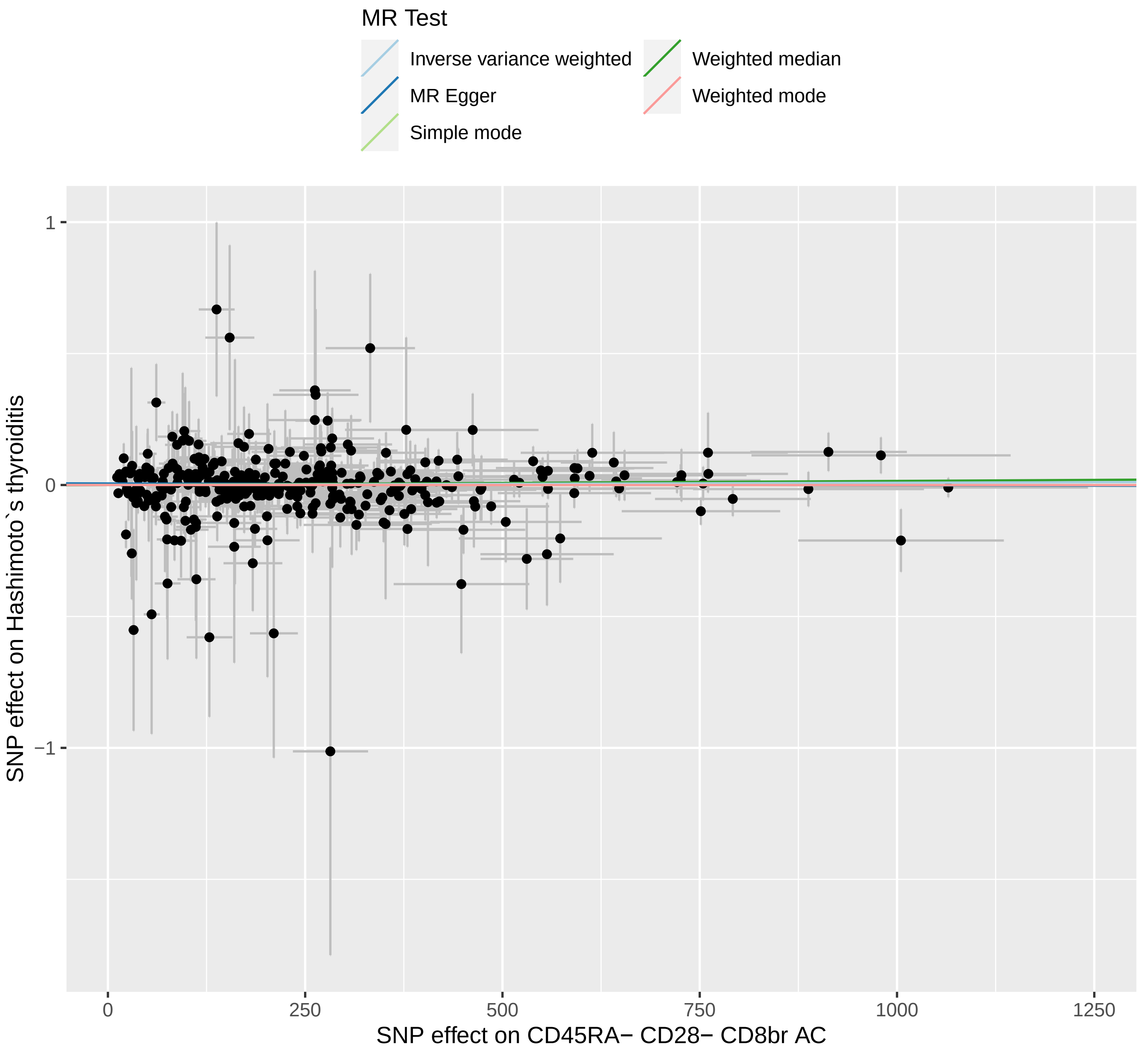

MR Test
Inverse variance weighted
MR Egger
Simple mode
Weighted median
Weighted mode
1
0
−1
0
250
500
750
1000
1250
SNP effect on Hashimoto`s thyroiditis
SNP effect on CD45RA− CD28− CD8br AC

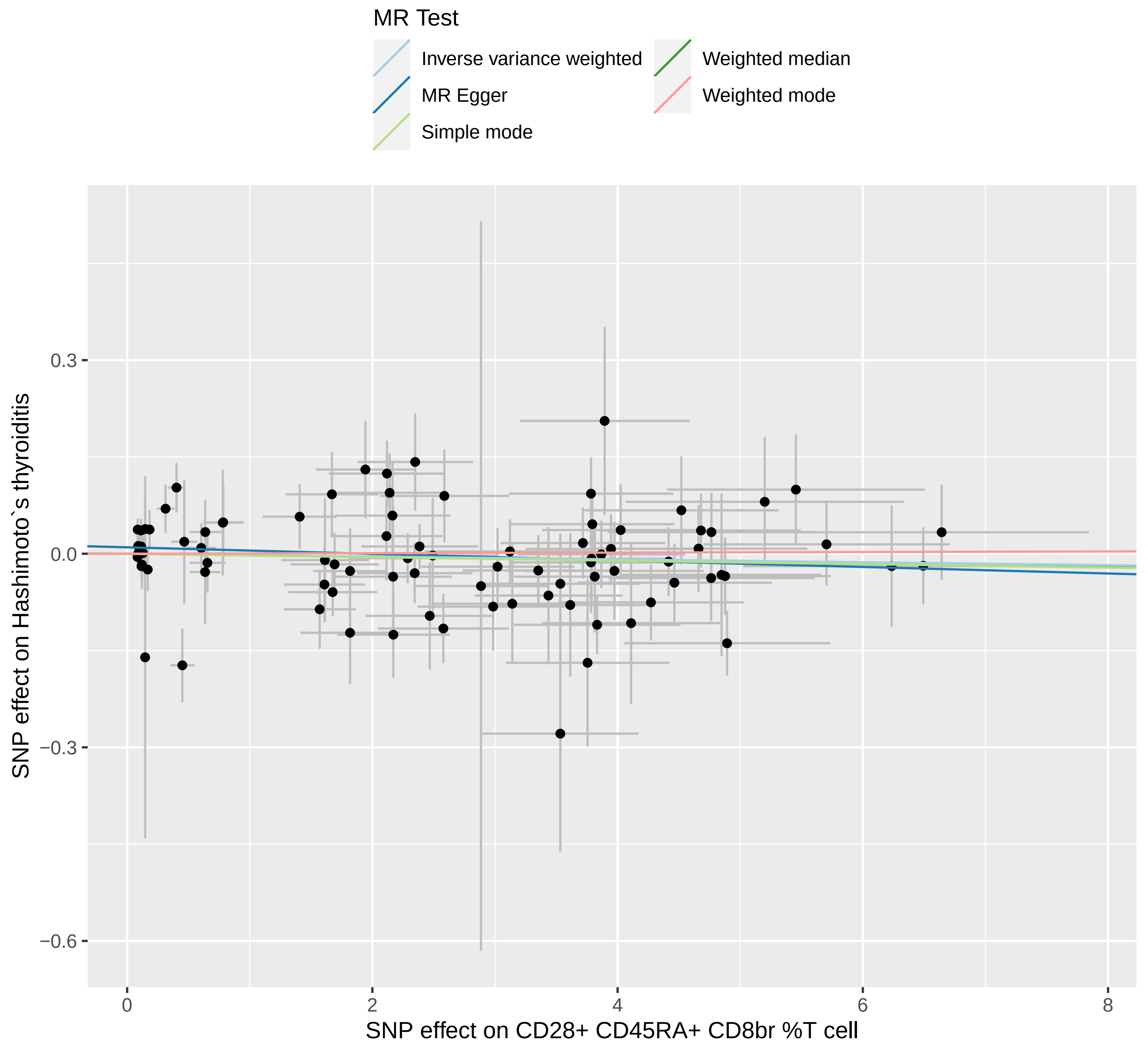
MR Test
Inverse variance weighted
MR Egger
Simple mode
Weighted median
Weighted mode
0.3
0.0
−0.3
−0.6
0
2
4
6
8
SNP effect on Hashimoto`s thyroiditis
SNP effect on CD28+ CD45RA+ CD8br %T cell

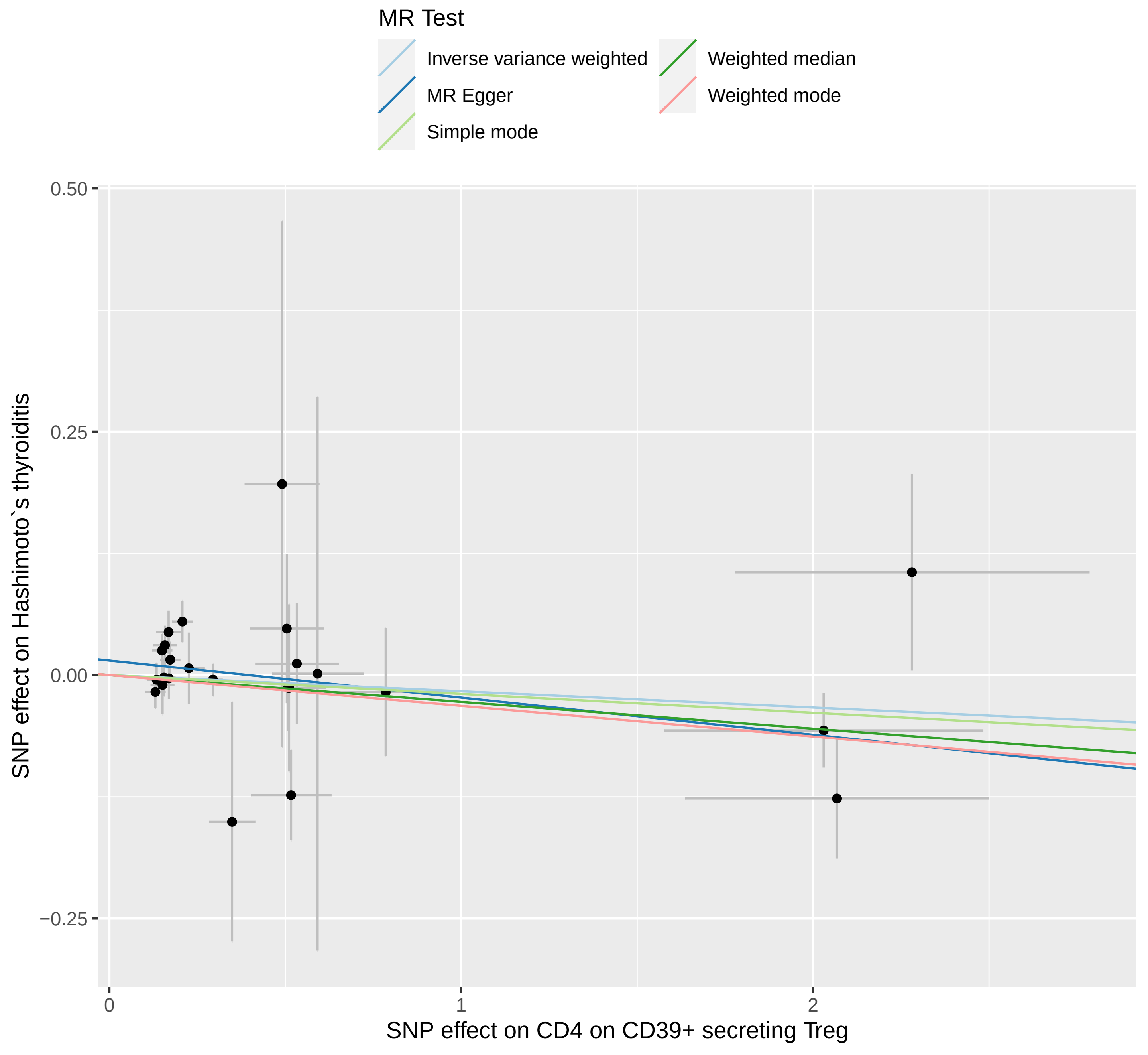

MR Test
Inverse variance weighted
MR Egger
Simple mode
Weighted median
Weighted mode
0.50
0.25
0.00
−0.25
0
1
2
SNP effect on Hashimoto`s thyroiditis
SNP effect on CD4 on CD39+ secreting Treg

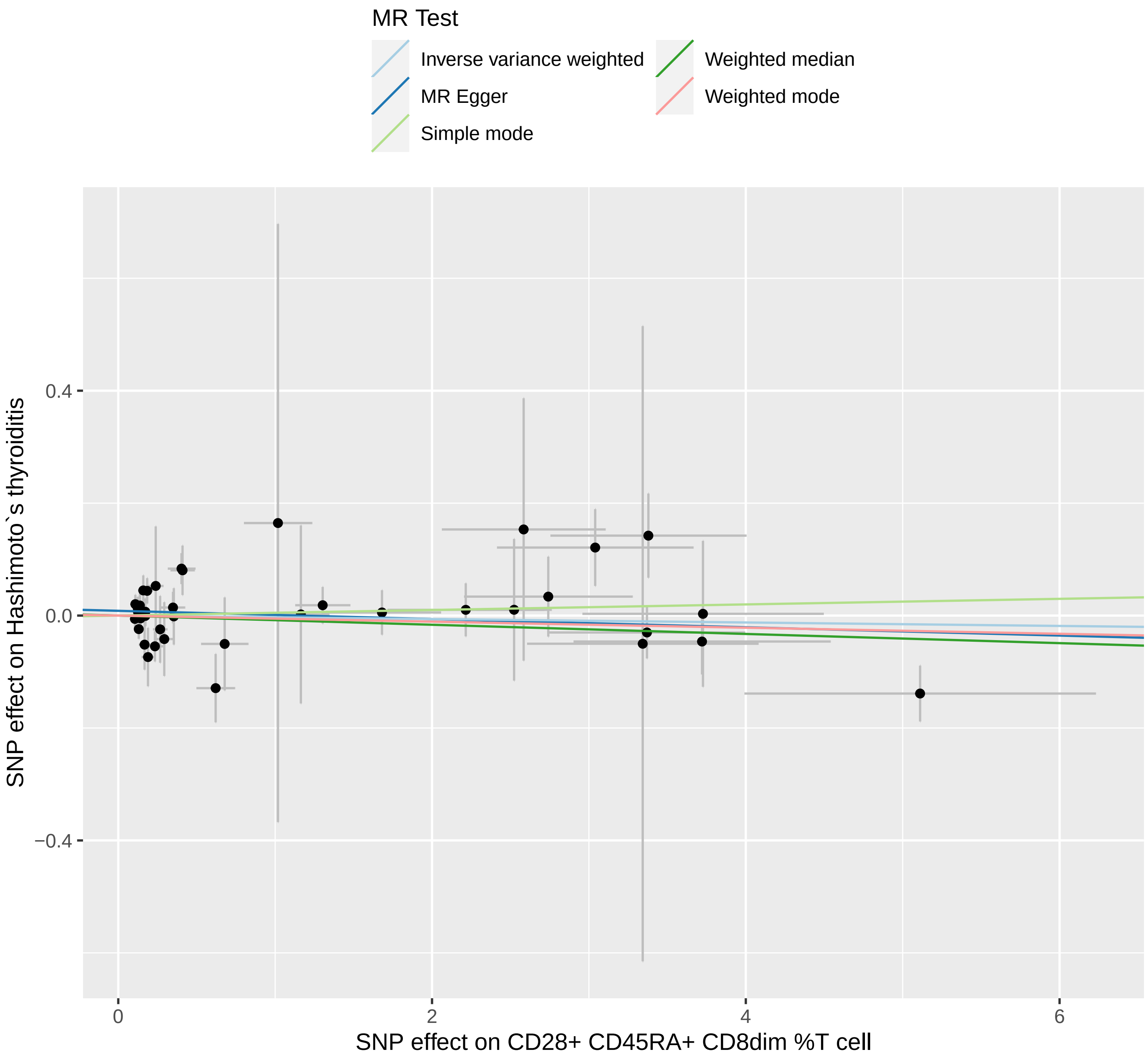
MR Test
Inverse variance weighted
MR Egger
Simple mode
Weighted median
Weighted mode
SNP effect on Hashimoto`s thyroiditis
0.4
0.0
−0.4
0
2
4
6
SNP effect on CD28+ CD45RA+ CD8dim %T cell

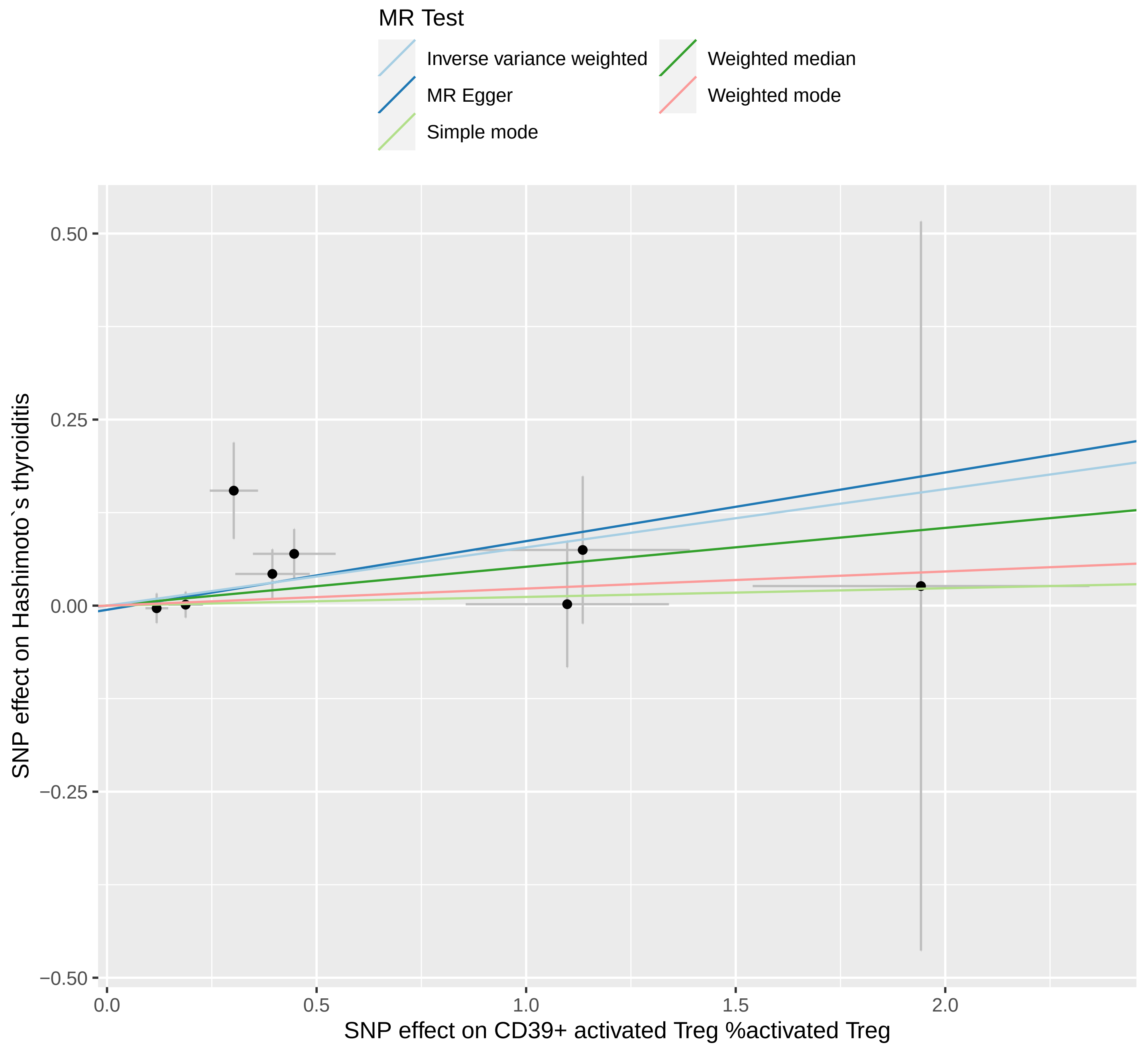
MR Test
Inverse variance weighted
MR Egger
Simple mode
Weighted median
Weighted mode
0.50
0.25
0.00
−0.25
−0.50
0.0
0.5
1.0
1.5
2.0
SNP effect on CD39+ activated Treg %activated Treg
SNP effect on Hashimoto`s thyroiditis

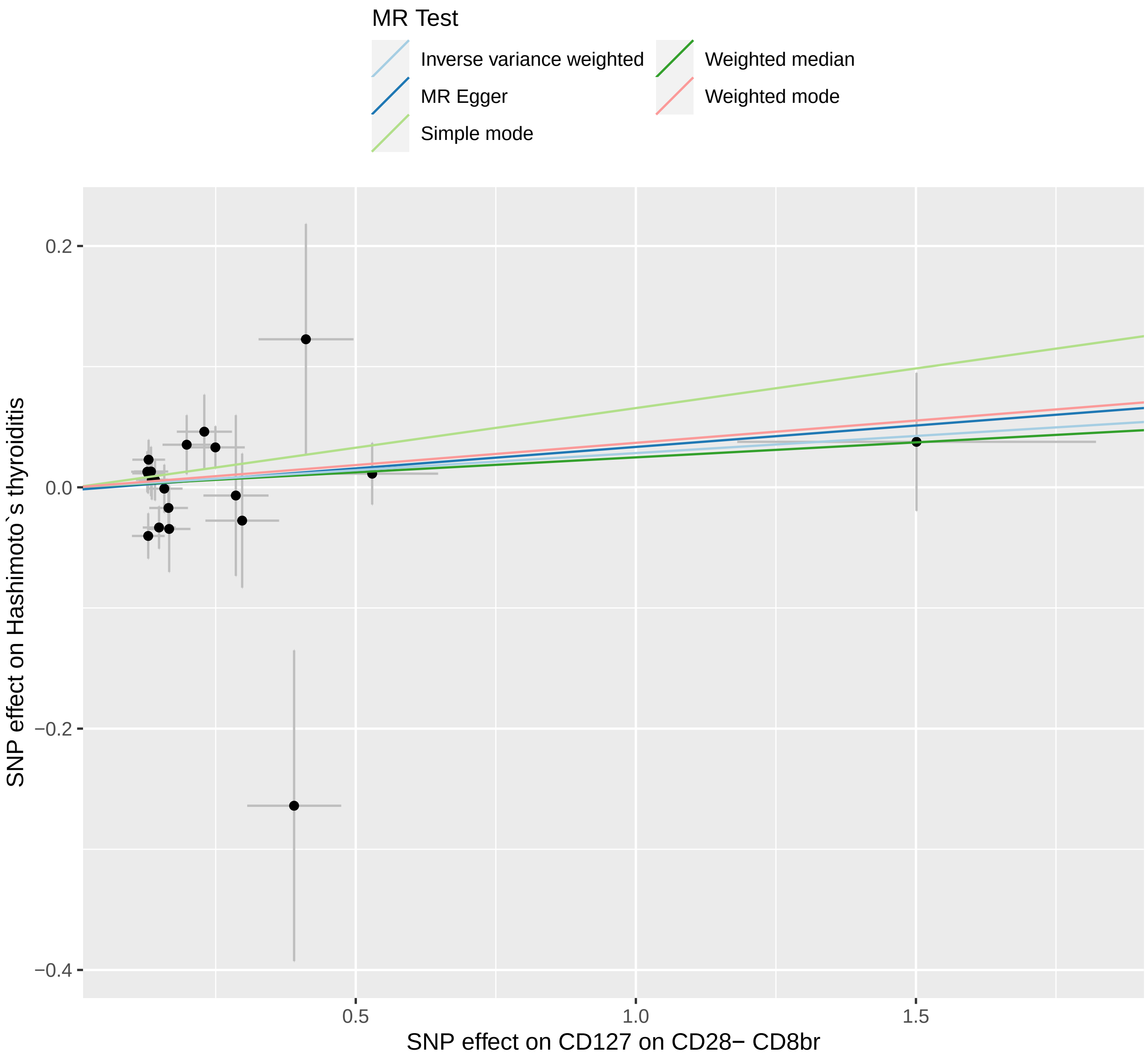

MR Test
Inverse variance weighted
MR Egger
Simple mode
Weighted median
Weighted mode
0.2
0.0
−0.2
−0.4
0.5
1.0
1.5
SNP effect on Hashimoto`s thyroiditis
SNP effect on CD127 on CD28− CD8br

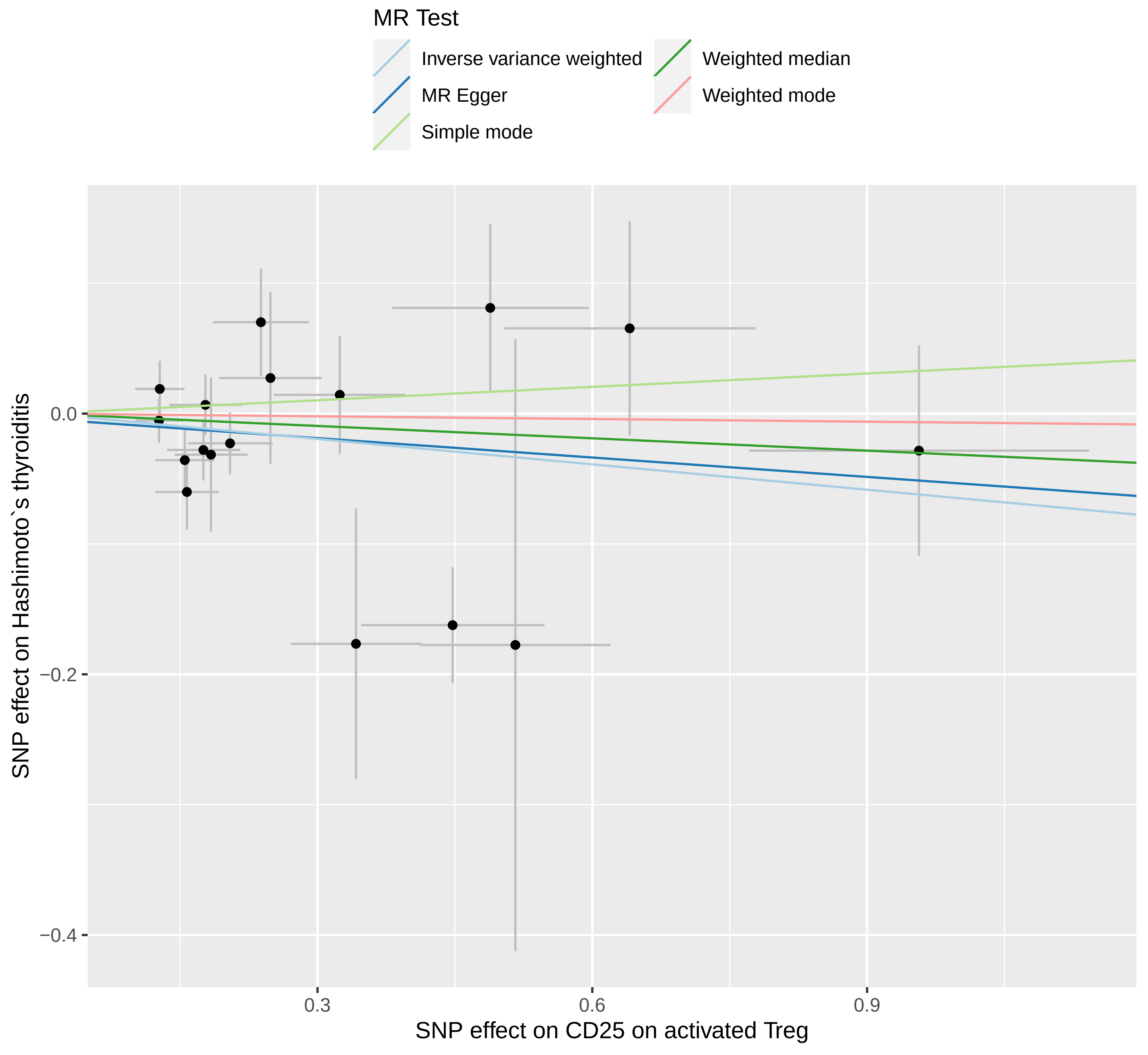

MR Test
Inverse variance weighted
MR Egger
Simple mode
Weighted median
Weighted mode
0.0
−0.2
−0.4
SNP effect on Hashimoto`s thyroiditis
0.3
0.6
0.9
SNP effect on CD25 on activated Treg

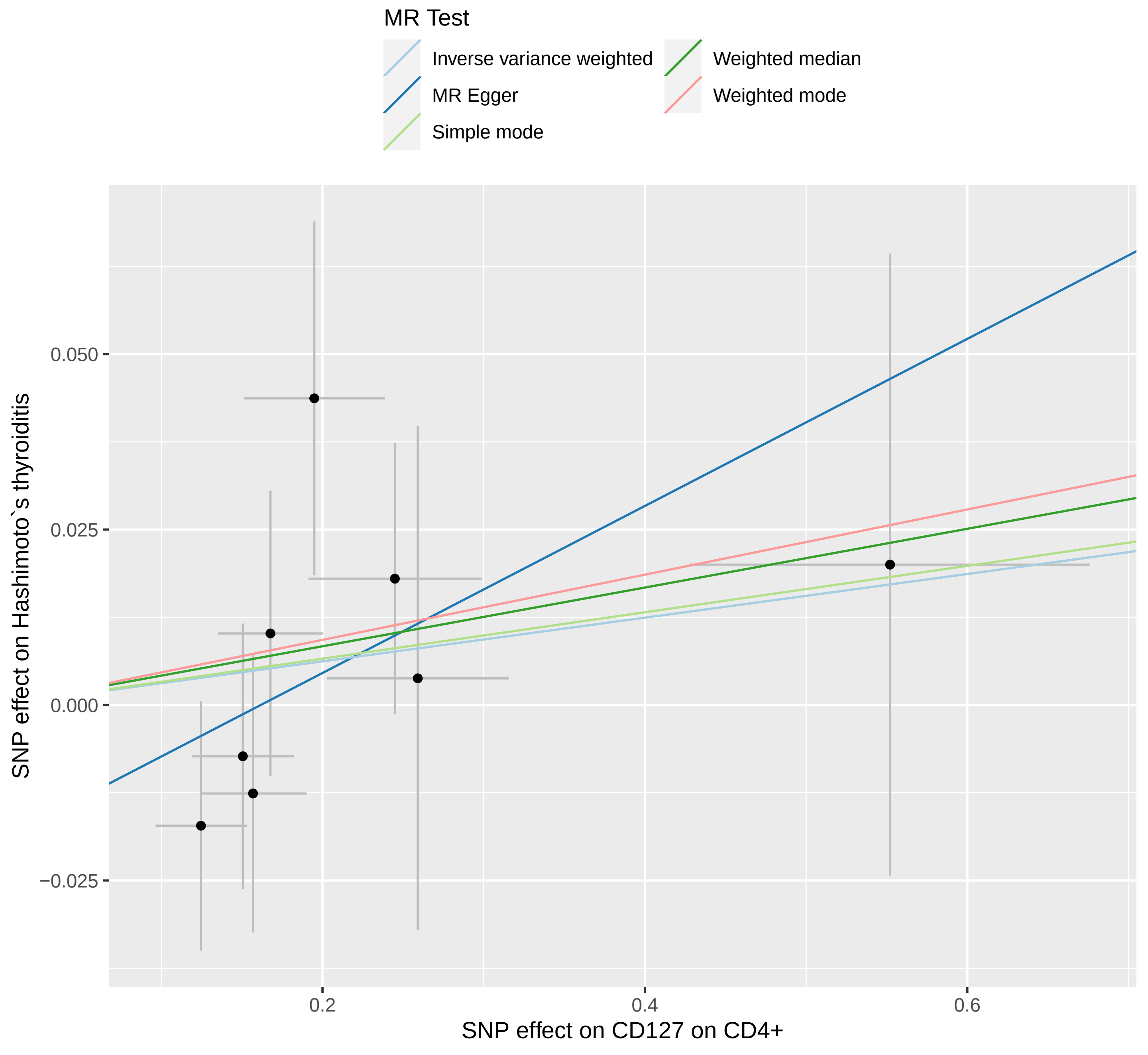

MR Test
Inverse variance weighted
MR Egger
Simple mode
Weighted median
Weighted mode
0.050
0.025
0.000
−0.025
SNP effect on Hashimoto`s thyroiditis
0.2
0.4
0.6
SNP effect on CD127 on CD4+

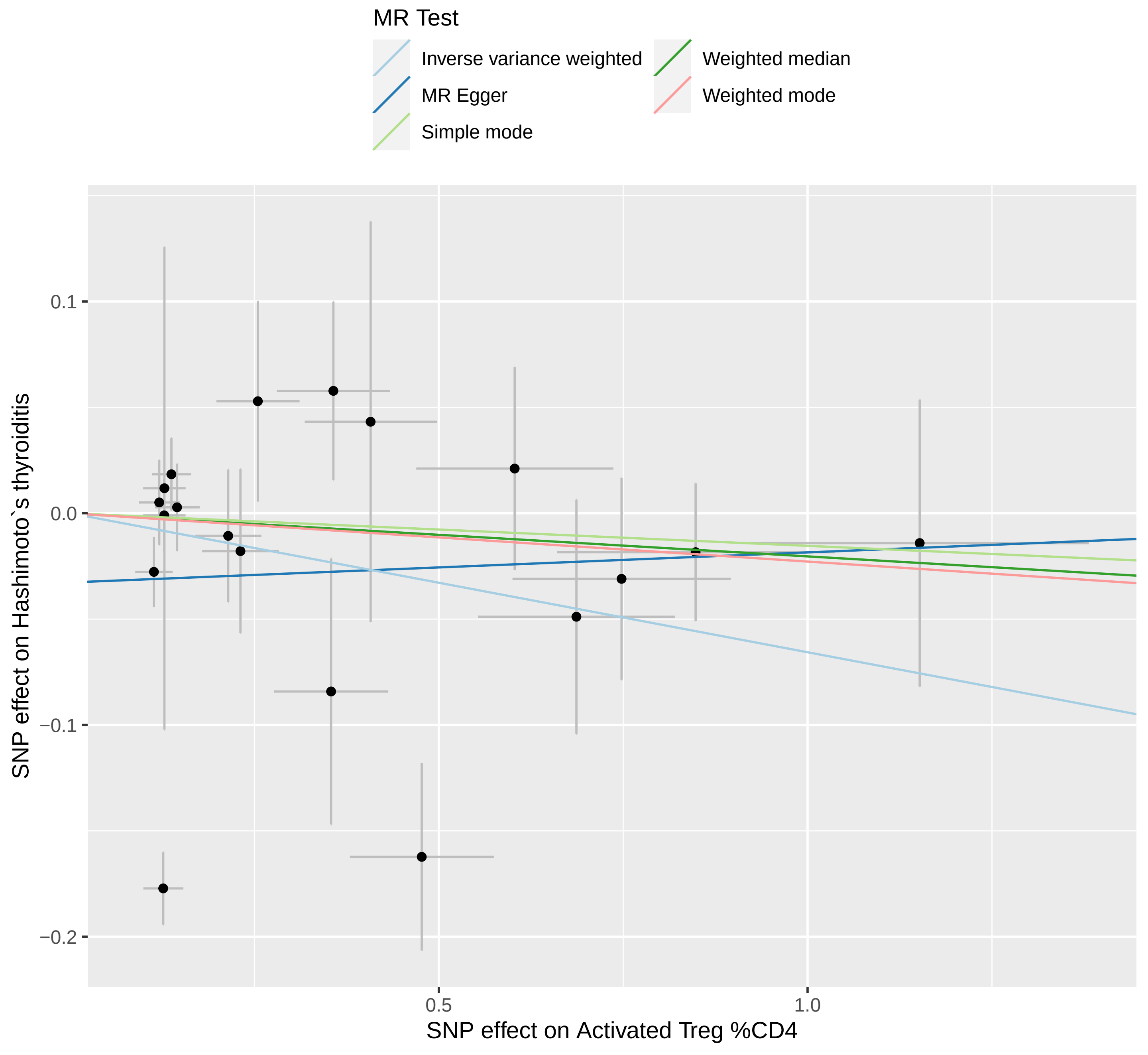

MR Test
Inverse variance weighted
MR Egger
Simple mode
Weighted median
Weighted mode
0.1
0.0
−0.1
−0.2
0.5
1.0
SNP effect on Activated Treg %CD4
SNP effect on Hashimoto`s thyroiditis

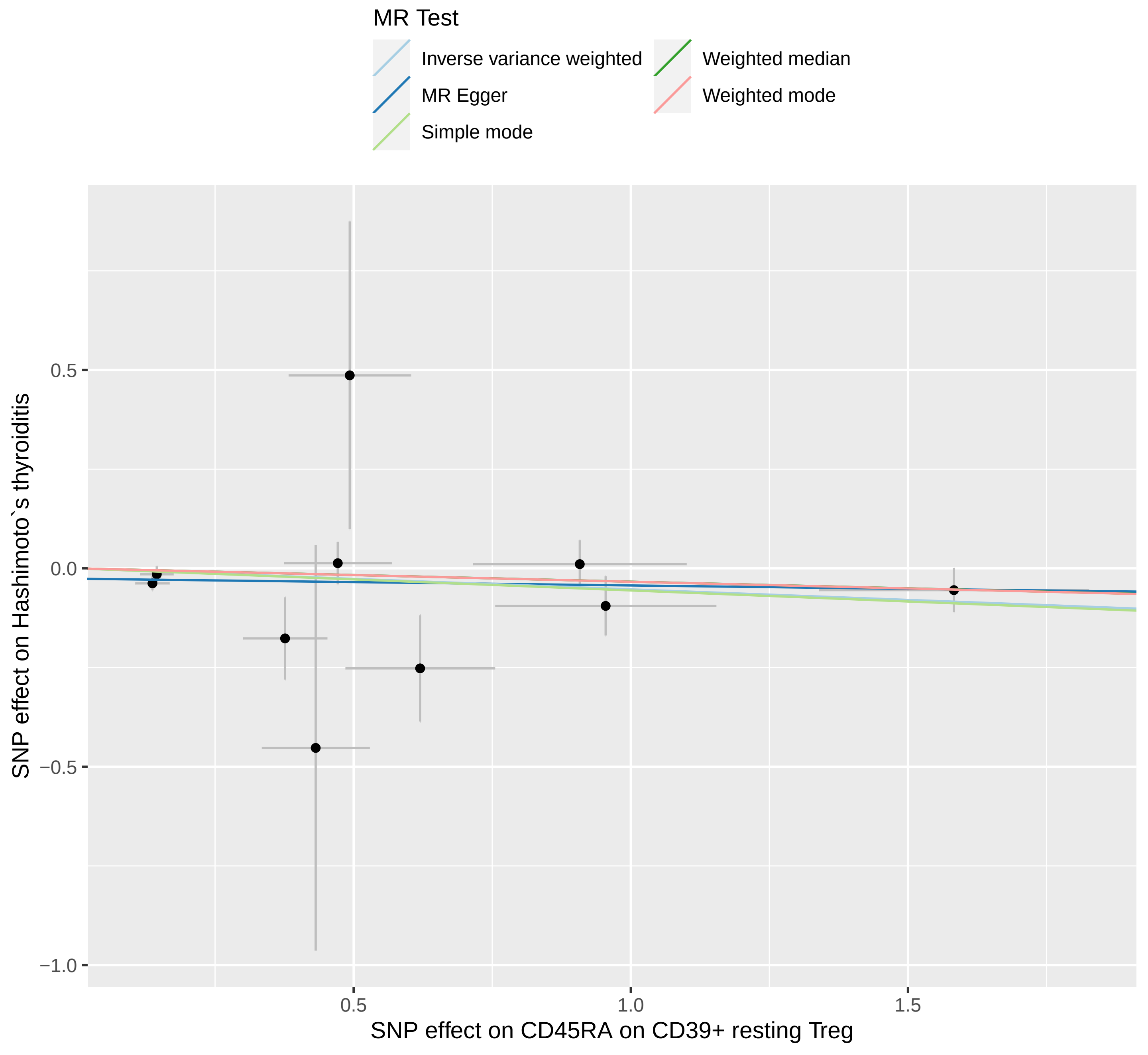
MR Test
Inverse variance weighted
MR Egger
Simple mode
Weighted median
Weighted mode
0.5
0.0
−0.5
−1.0
SNP effect on Hashimoto`s thyroiditis
0.5
1.0
1.5
SNP effect on CD45RA on CD39+ resting Treg

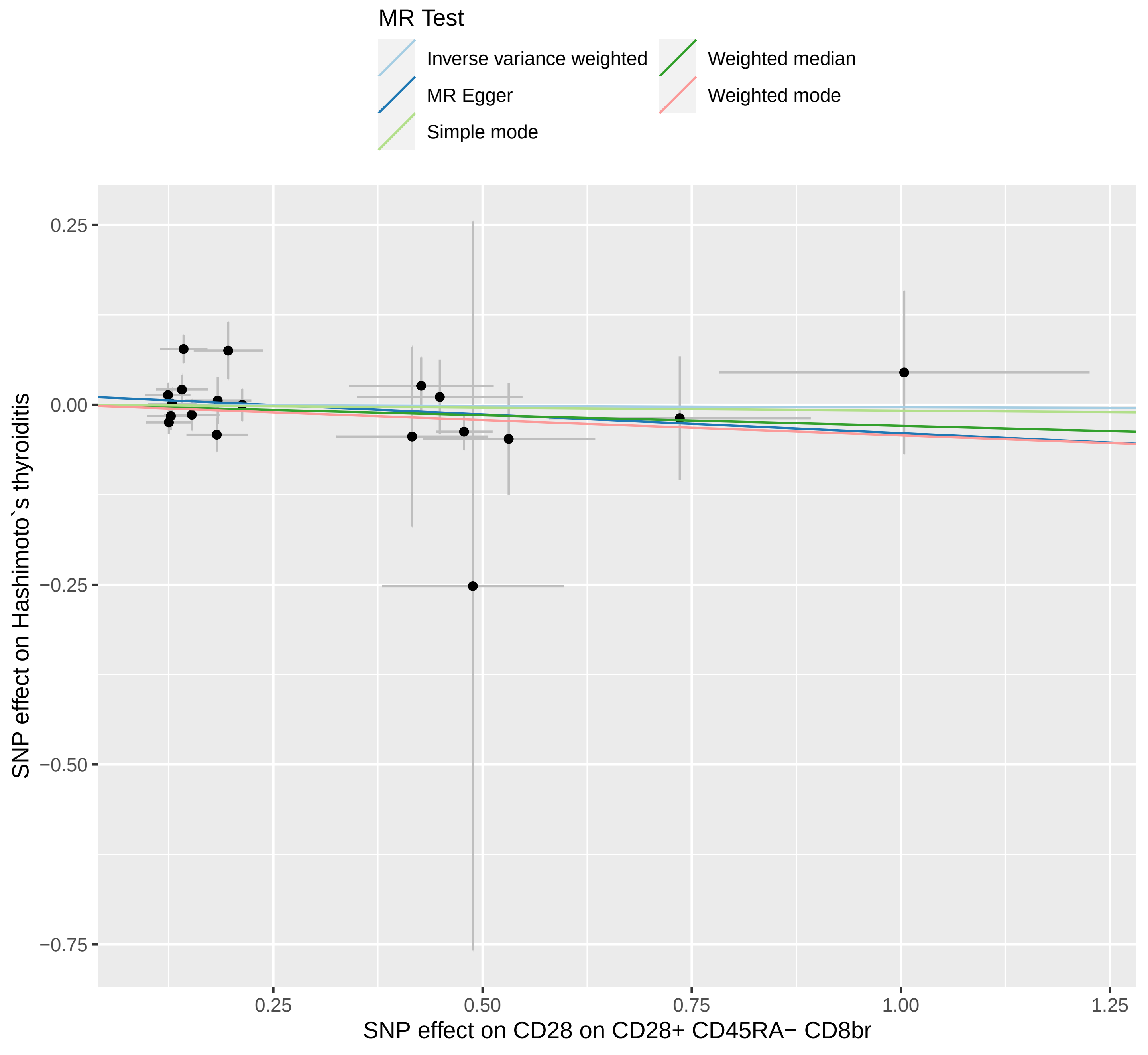
MR Test
Inverse variance weighted
MR Egger
Simple mode
Weighted median
Weighted mode
0.25
0.00
−0.25
−0.50
−0.75
SNP effect on Hashimoto`s thyroiditis
0.25
0.50
0.75
1.00
1.25
SNP effect on CD28 on CD28+ CD45RA− CD8br

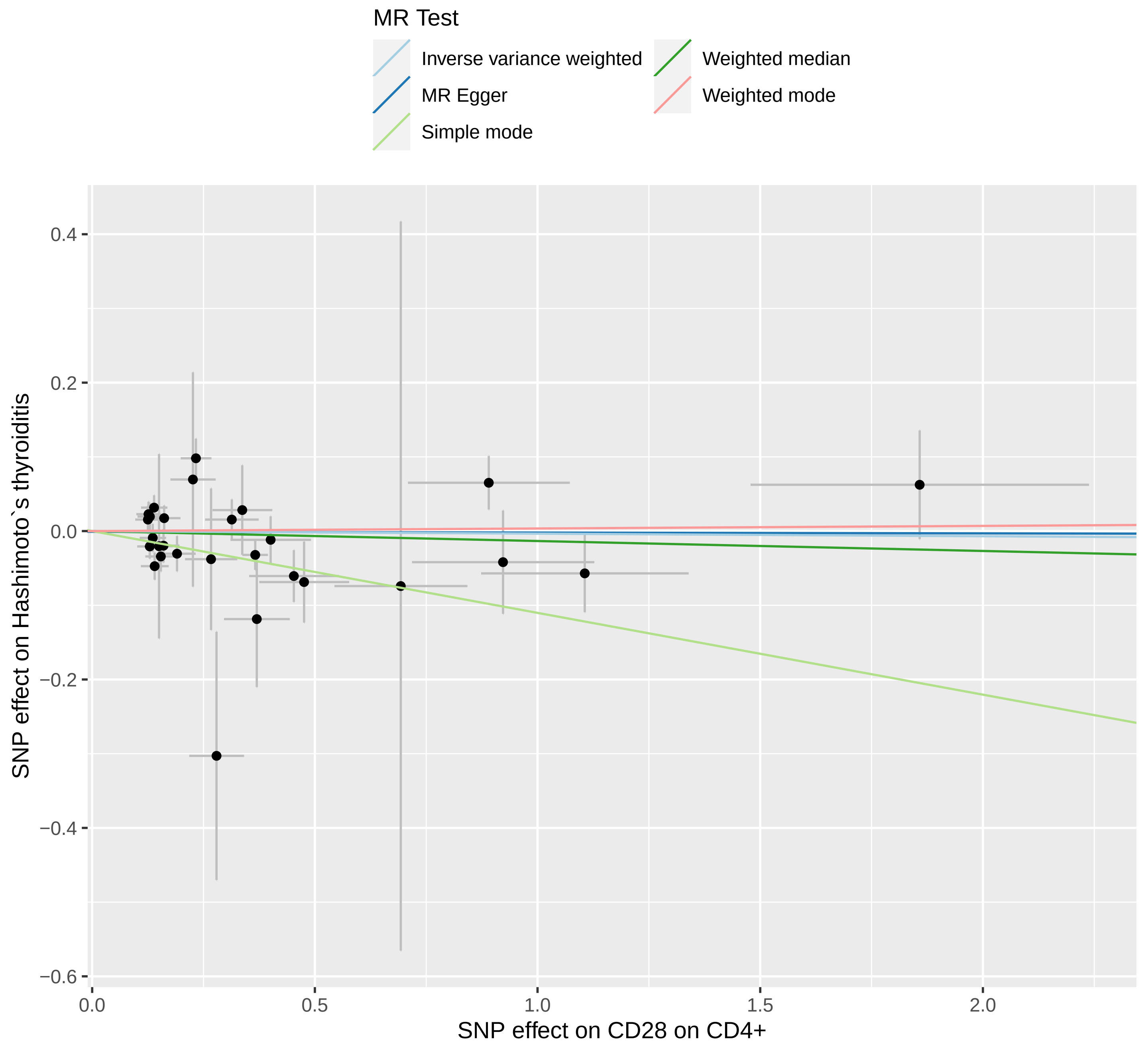
MR Test
Inverse variance weighted
MR Egger
Simple mode
Weighted median
Weighted mode
0.4
0.2
0.0
−0.2
−0.4
−0.6
0.0
0.5
1.0
1.5
2.0
SNP effect on Hashimoto`s thyroiditis
SNP effect on CD28 on CD4+

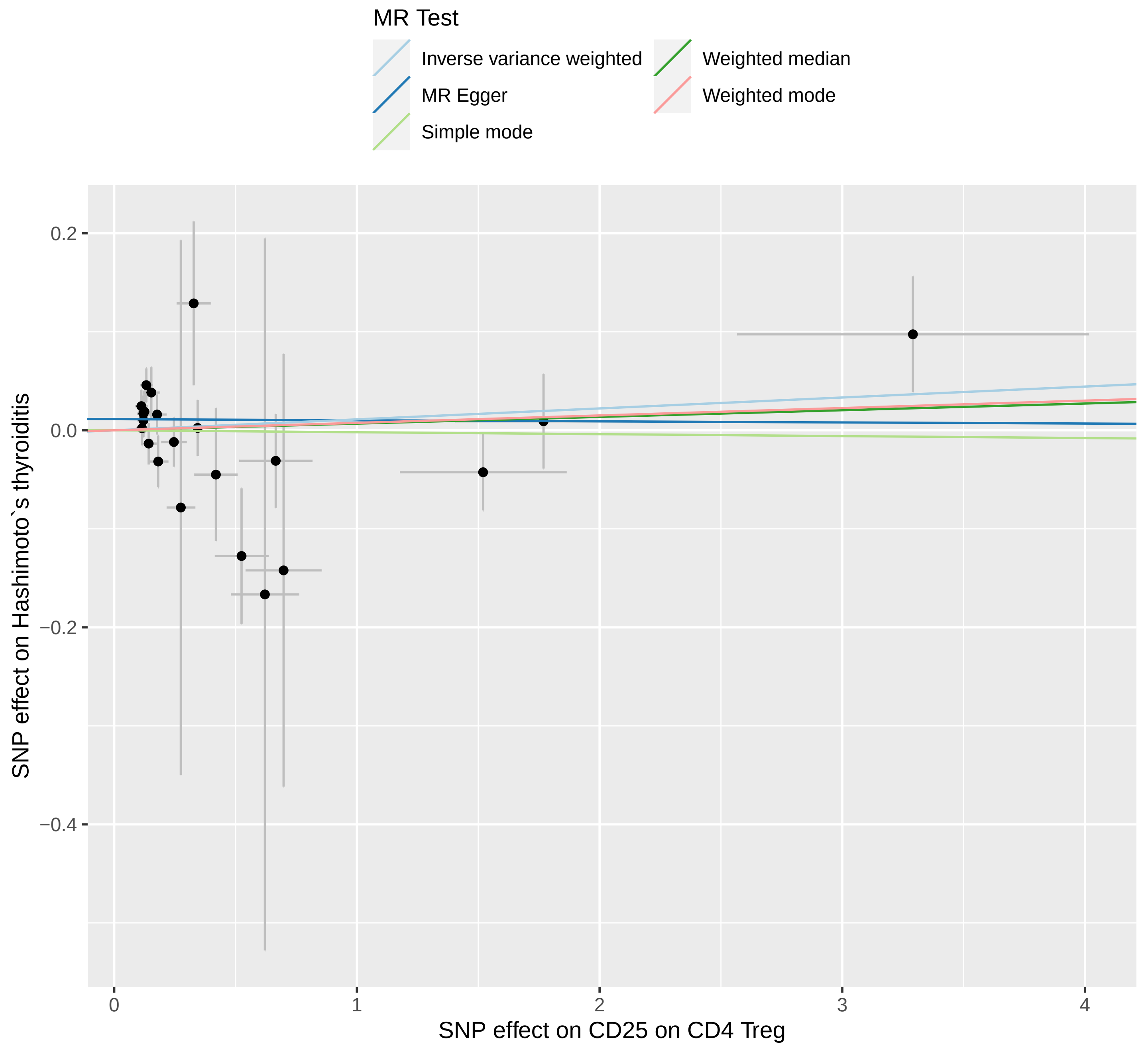

MR Test
Inverse variance weighted
MR Egger
Simple mode
Weighted median
Weighted mode
0.2
0.0
−0.2
−0.4
0
1
2
3
4
SNP effect on Hashimoto`s thyroiditis
SNP effect on CD25 on CD4 Treg

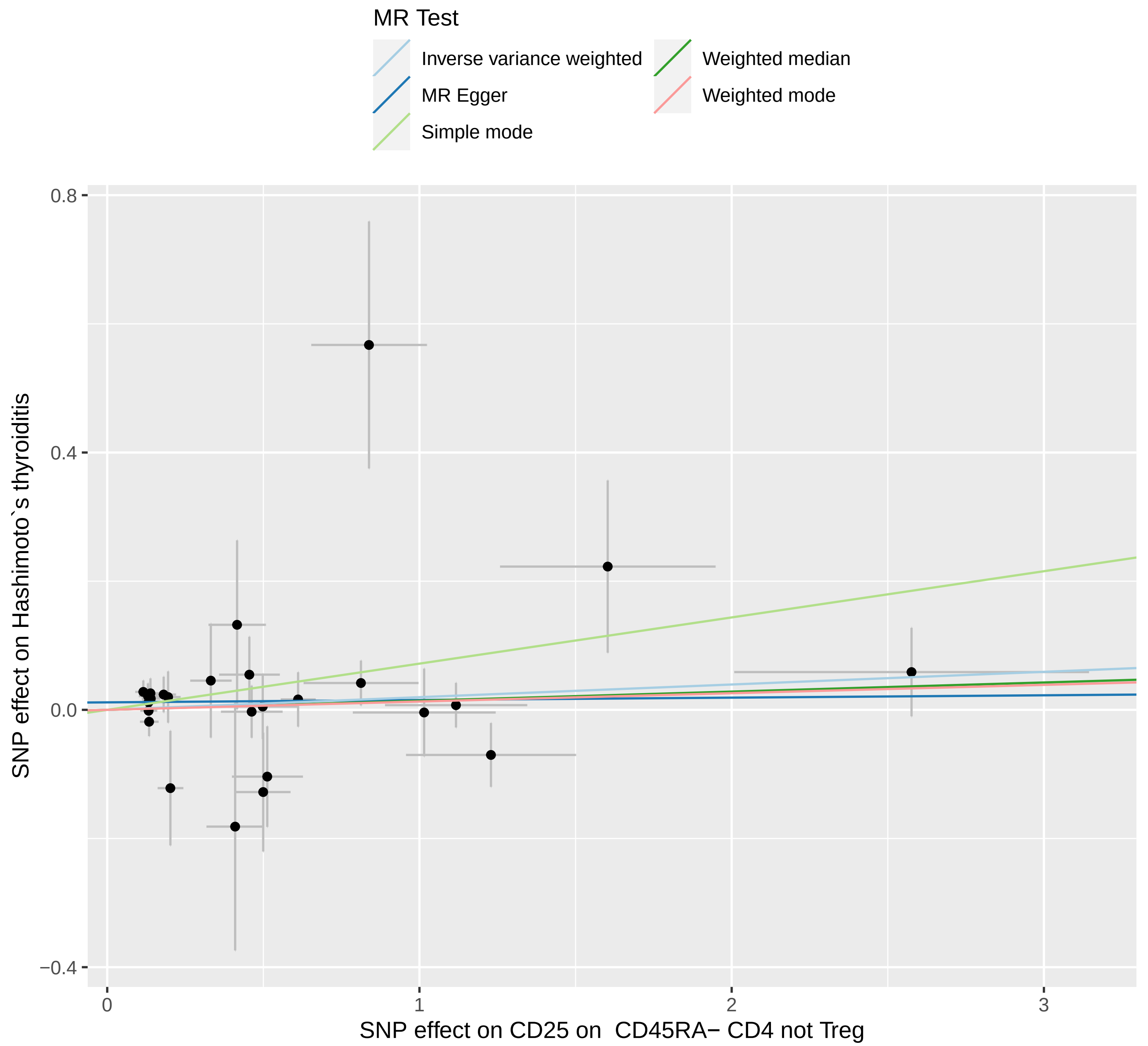
MR Test
Inverse variance weighted
MR Egger
Simple mode
Weighted median
Weighted mode
0.8
0.4
0.0
−0.4
0
1
2
3
SNP effect on Hashimoto`s thyroiditis
SNP effect on CD25 on CD45RA− CD4 not Treg

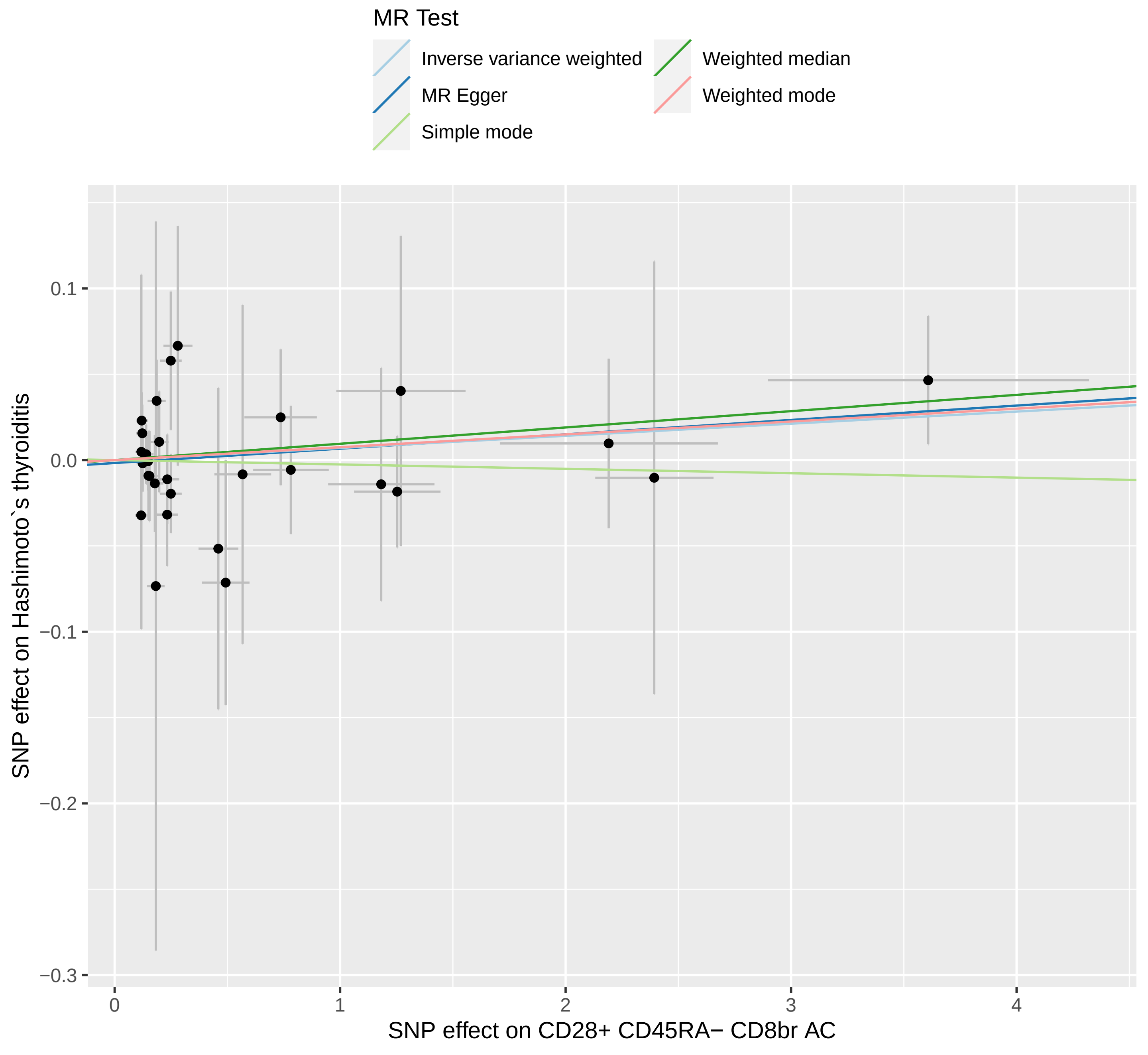
MR Test
Inverse variance weighted
Weighted median
MR Egger
Weighted mode
Simple mode
0.1
0.0
−0.1
−0.2
−0.3
0
1
2
3
4
SNP effect on Hashimoto`s thyroiditis
SNP effect on CD28+ CD45RA− CD8br AC

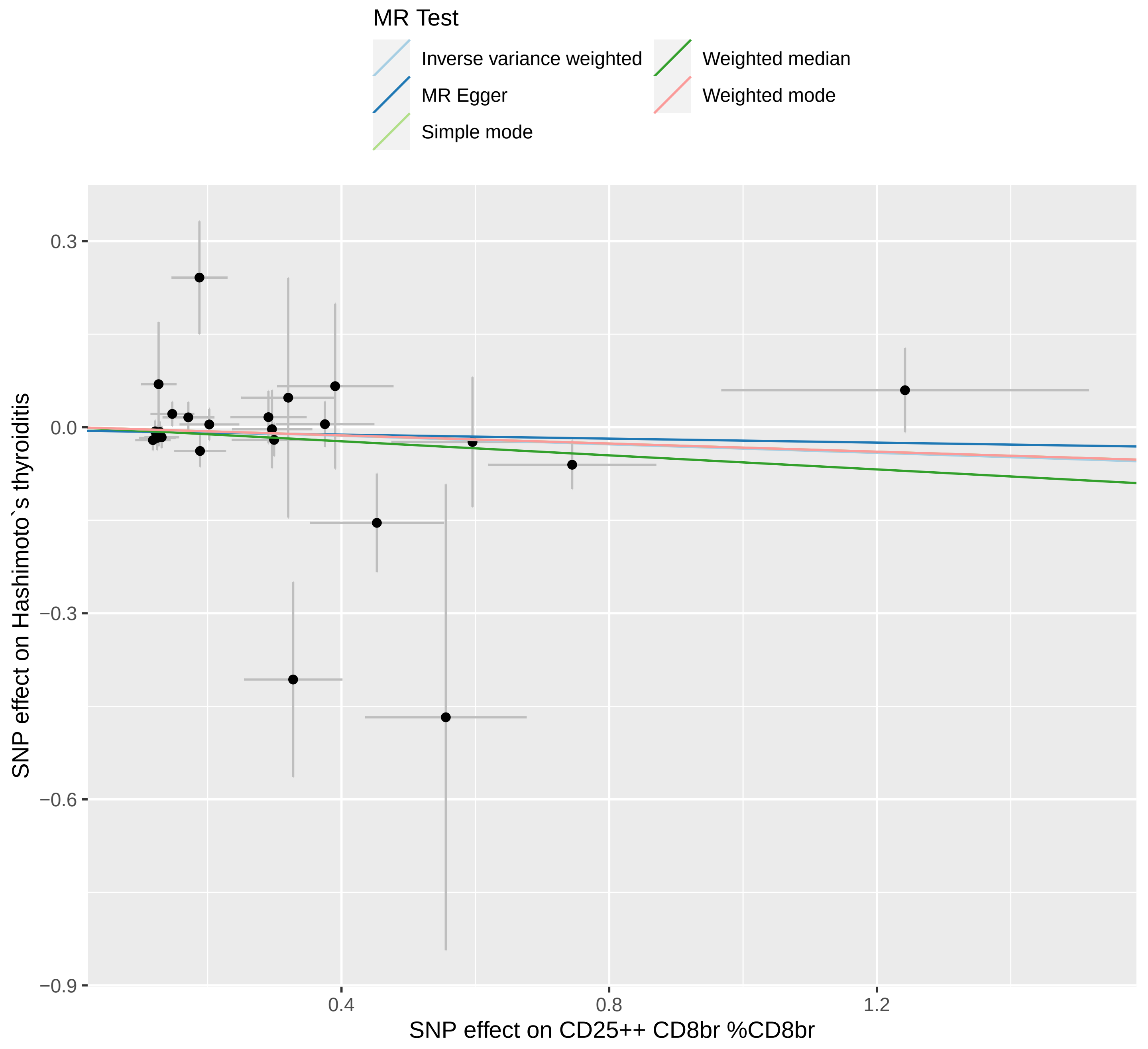
MR Test
Inverse variance weighted
MR Egger
Simple mode
Weighted median
Weighted mode
0.3
0.0
−0.3
−0.6
−0.9
0.4
0.8
1.2
SNP effect on Hashimoto`s thyroiditis
SNP effect on CD25++ CD8br %CD8br

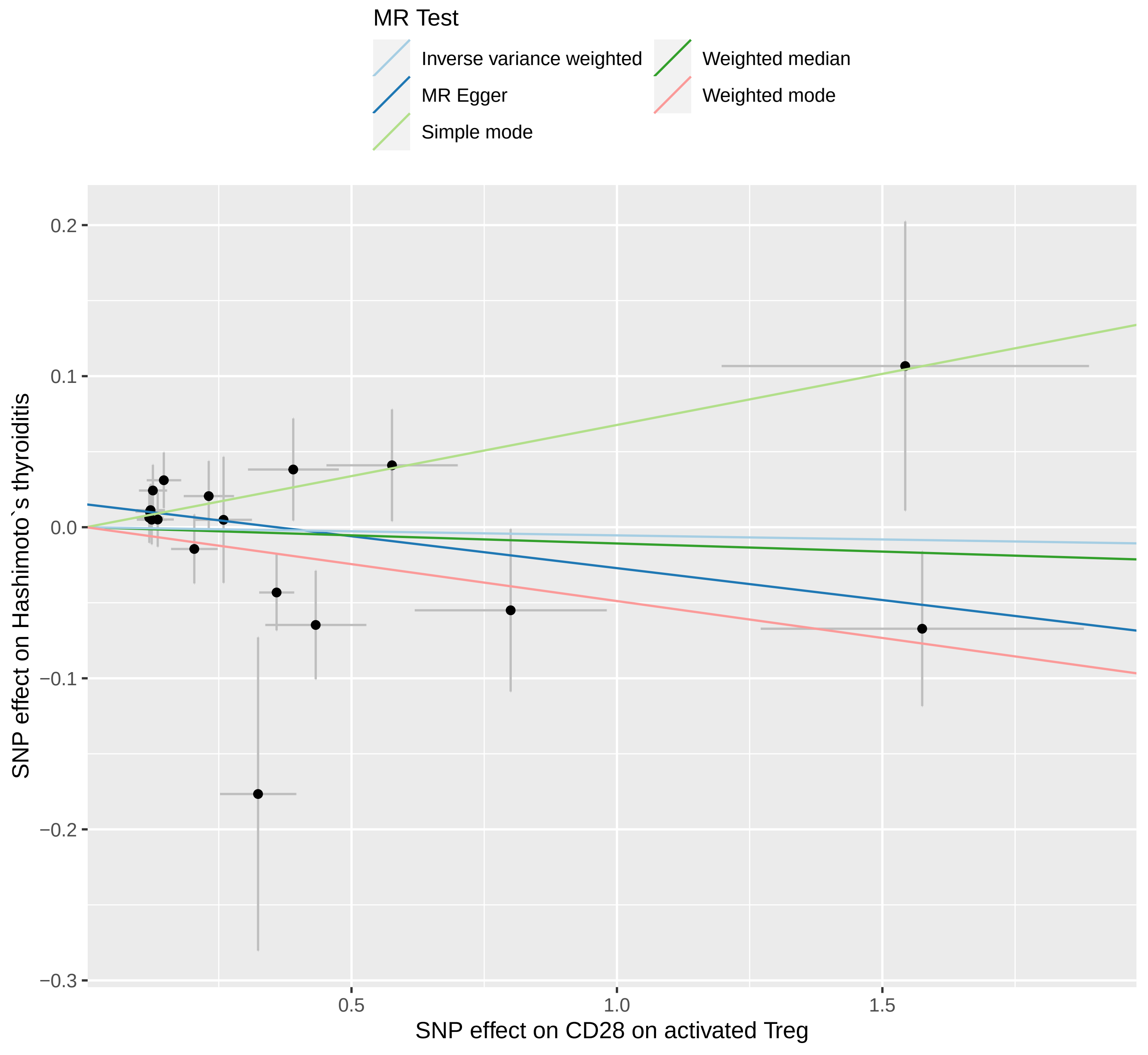
MR Test
Inverse variance weighted
MR Egger
Simple mode
Weighted median
Weighted mode
0.2
0.1
0.0
−0.1
−0.2
−0.3
0.5
1.0
1.5
SNP effect on Hashimoto`s thyroiditis
SNP effect on CD28 on activated Treg

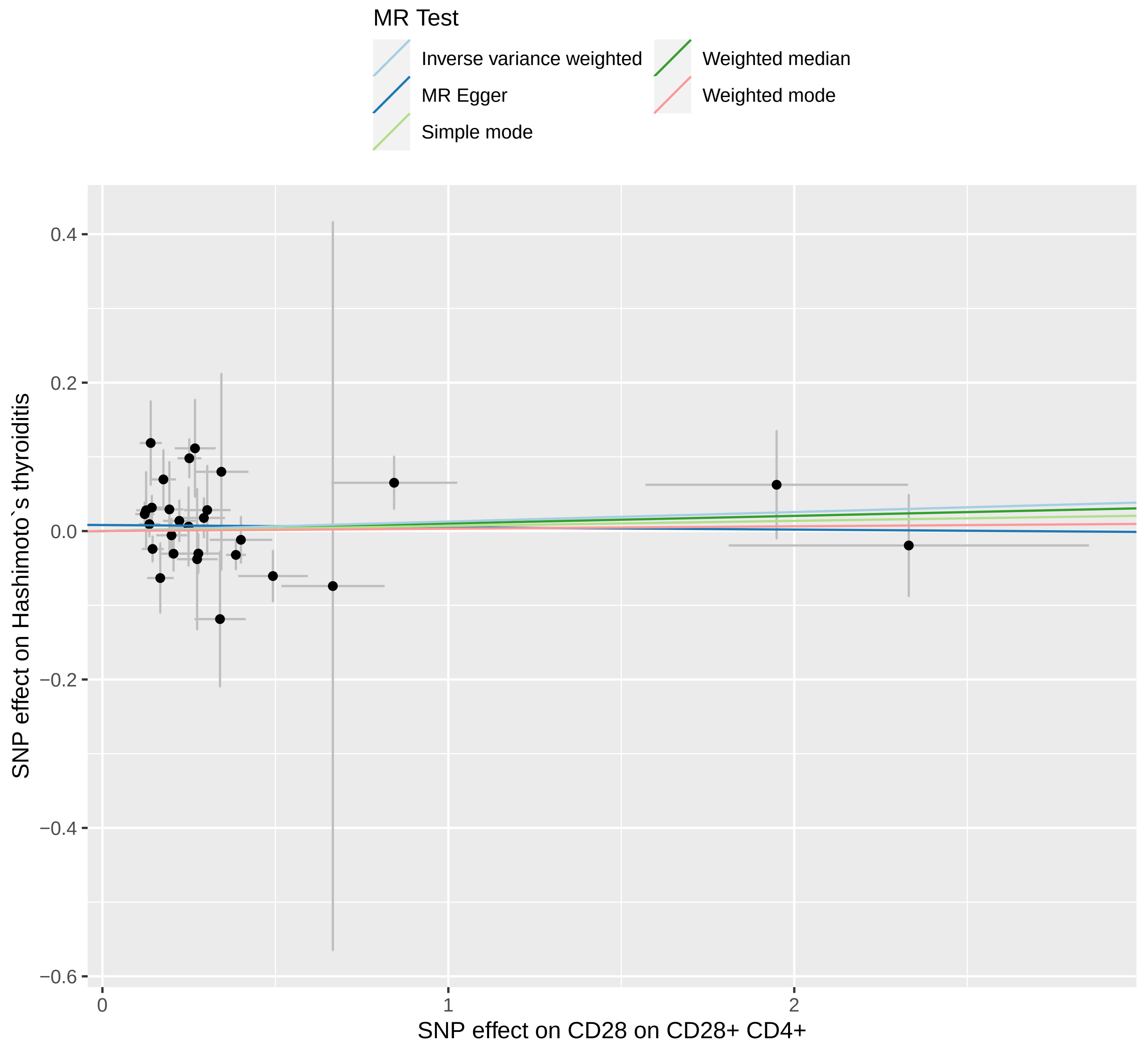
MR Test
Inverse variance weighted
MR Egger
Simple mode
Weighted median
Weighted mode
SNP effect on Hashimoto`s thyroiditis
0.4
0.2
0.0
−0.2
−0.4
−0.6
0
1
2
SNP effect on CD28 on CD28+ CD4+

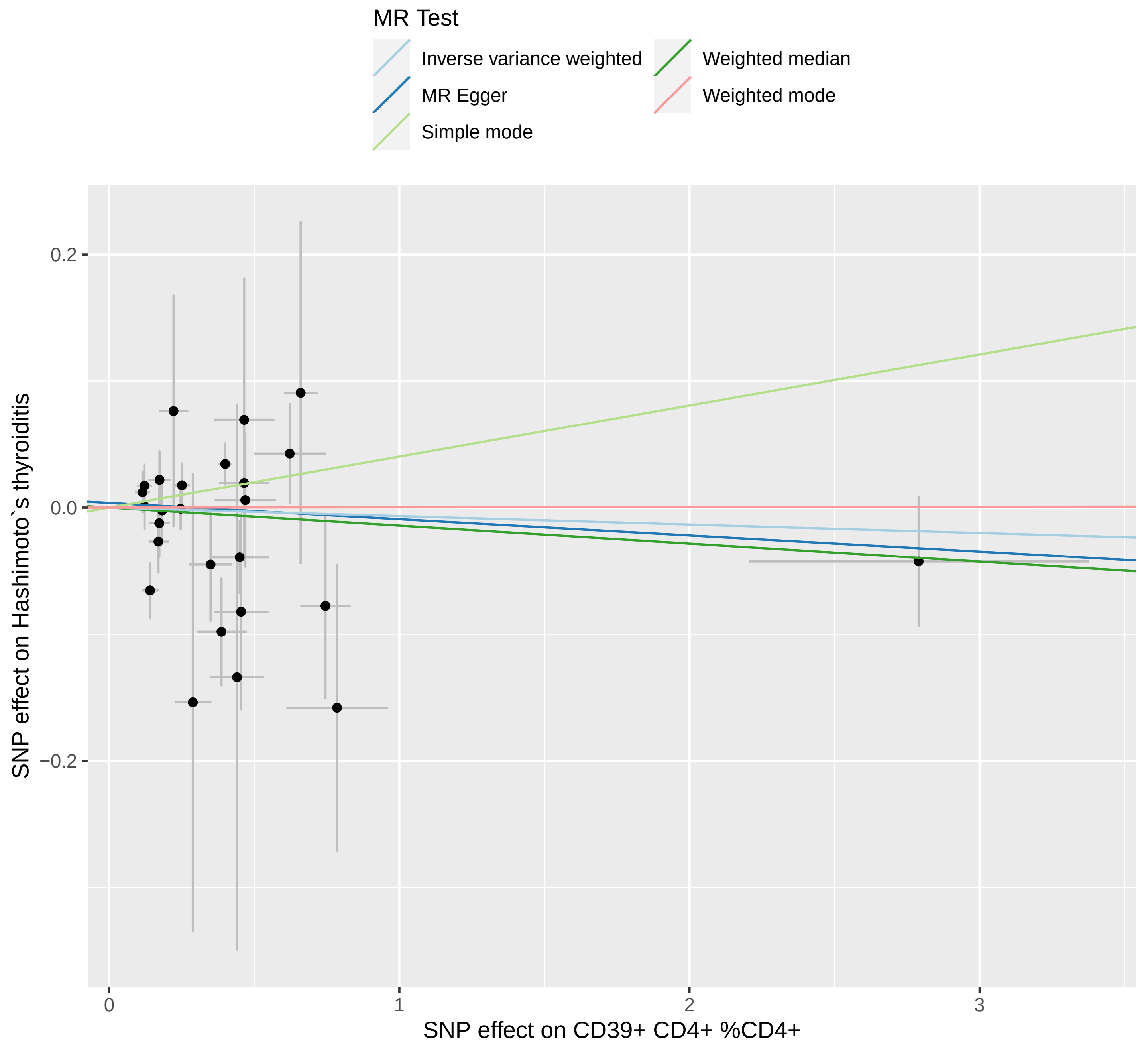

MR Test
Inverse variance weighted
MR Egger
Simple mode
Weighted median
Weighted mode
0.2
0.0
−0.2
0
1
2
3
SNP effect on Hashimoto`s thyroiditis
SNP effect on CD39+ CD4+ %CD4+

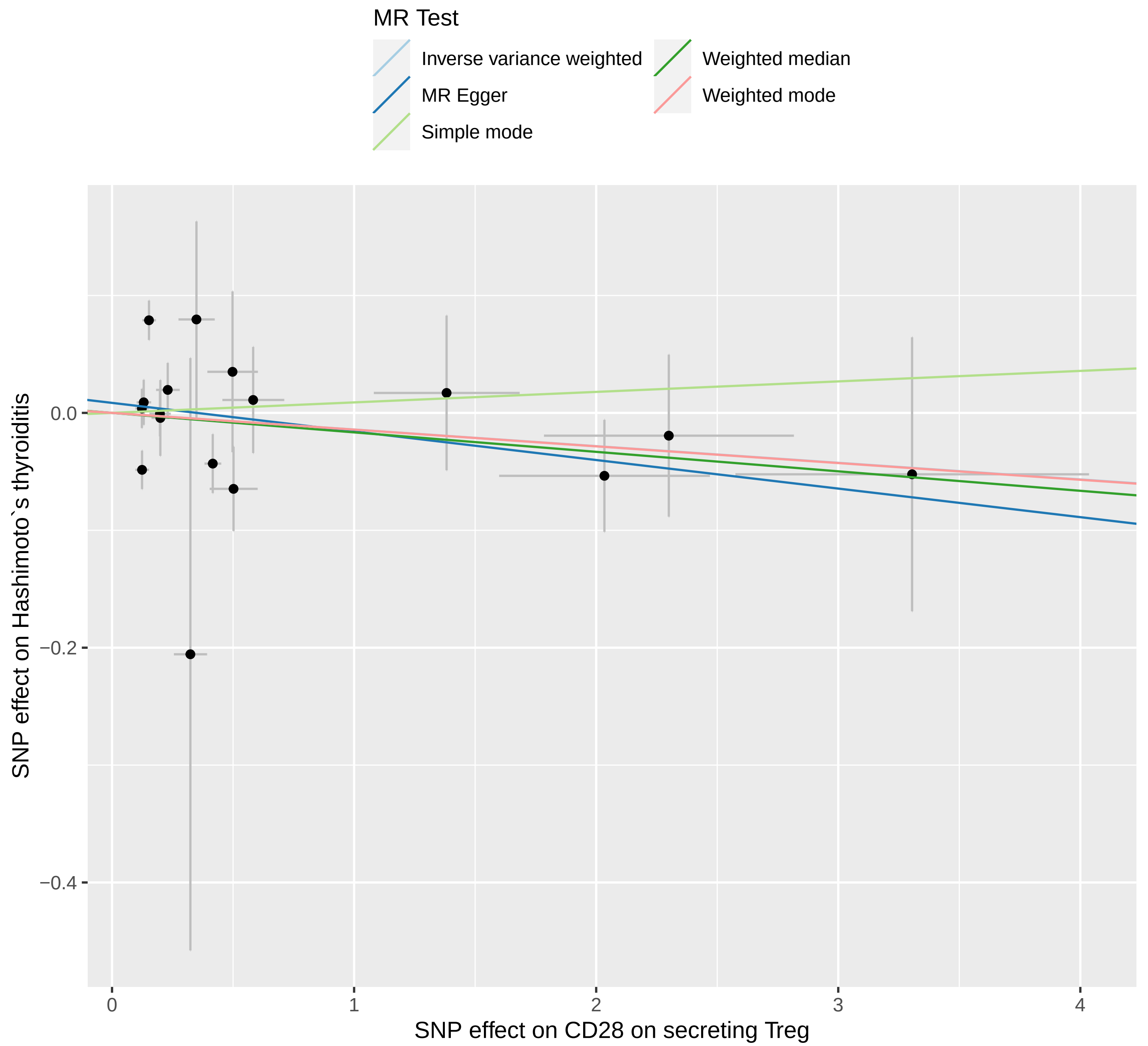
MR Test
Inverse variance weighted
MR Egger
Simple mode
Weighted median
Weighted mode
SNP effect on Hashimoto`s thyroiditis
0.0
−0.2
−0.4
0
1
2
3
4
SNP effect on CD28 on secreting Treg

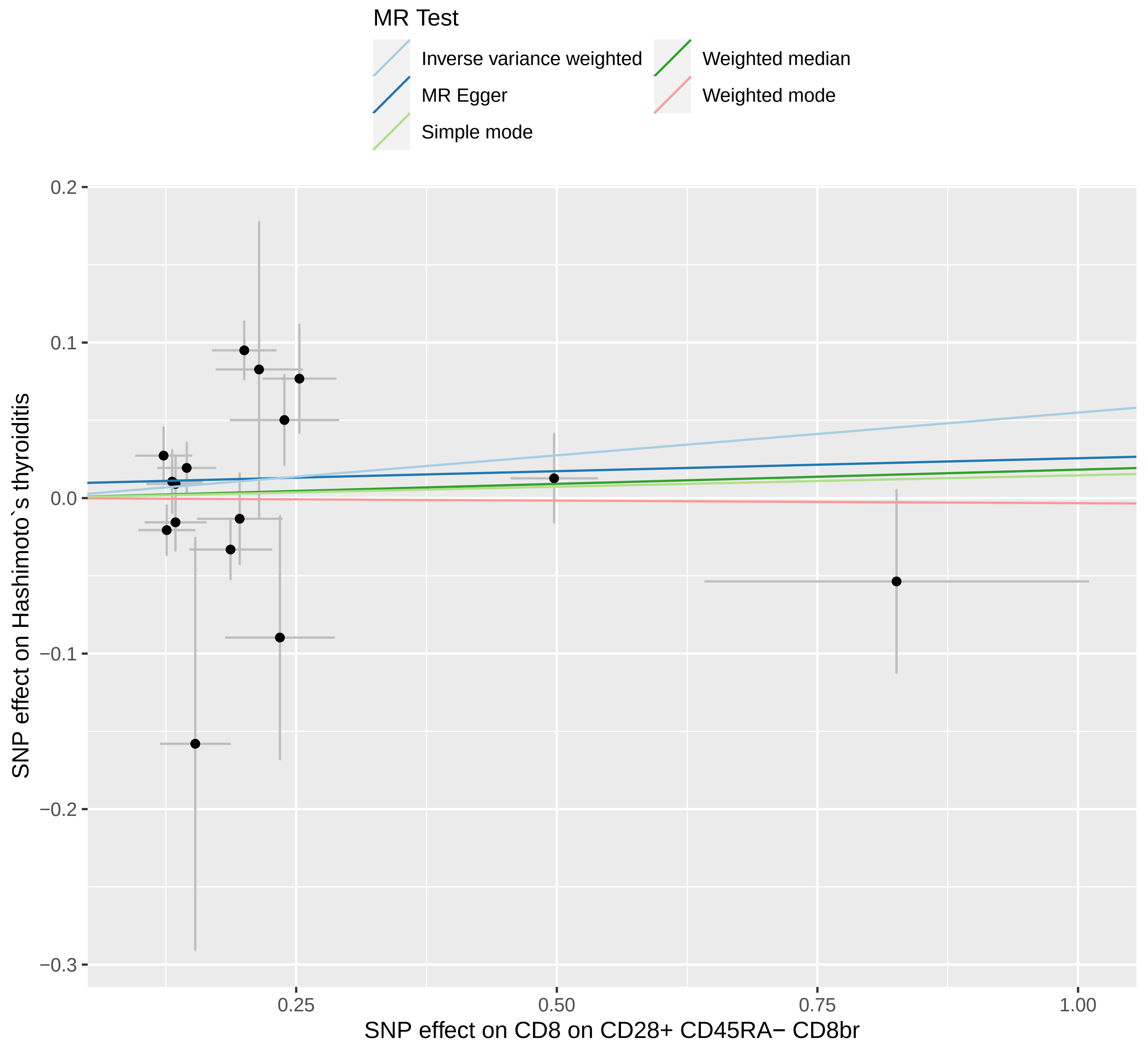

MR Test
Inverse variance weighted
MR Egger
Simple mode
Weighted median
Weighted mode
0.2
0.1
0.0
−0.1
−0.2
−0.3
0.25
0.50
0.75
1.00
SNP effect on Hashimoto`s thyroiditis
SNP effect on CD8 on CD28+ CD45RA− CD8br

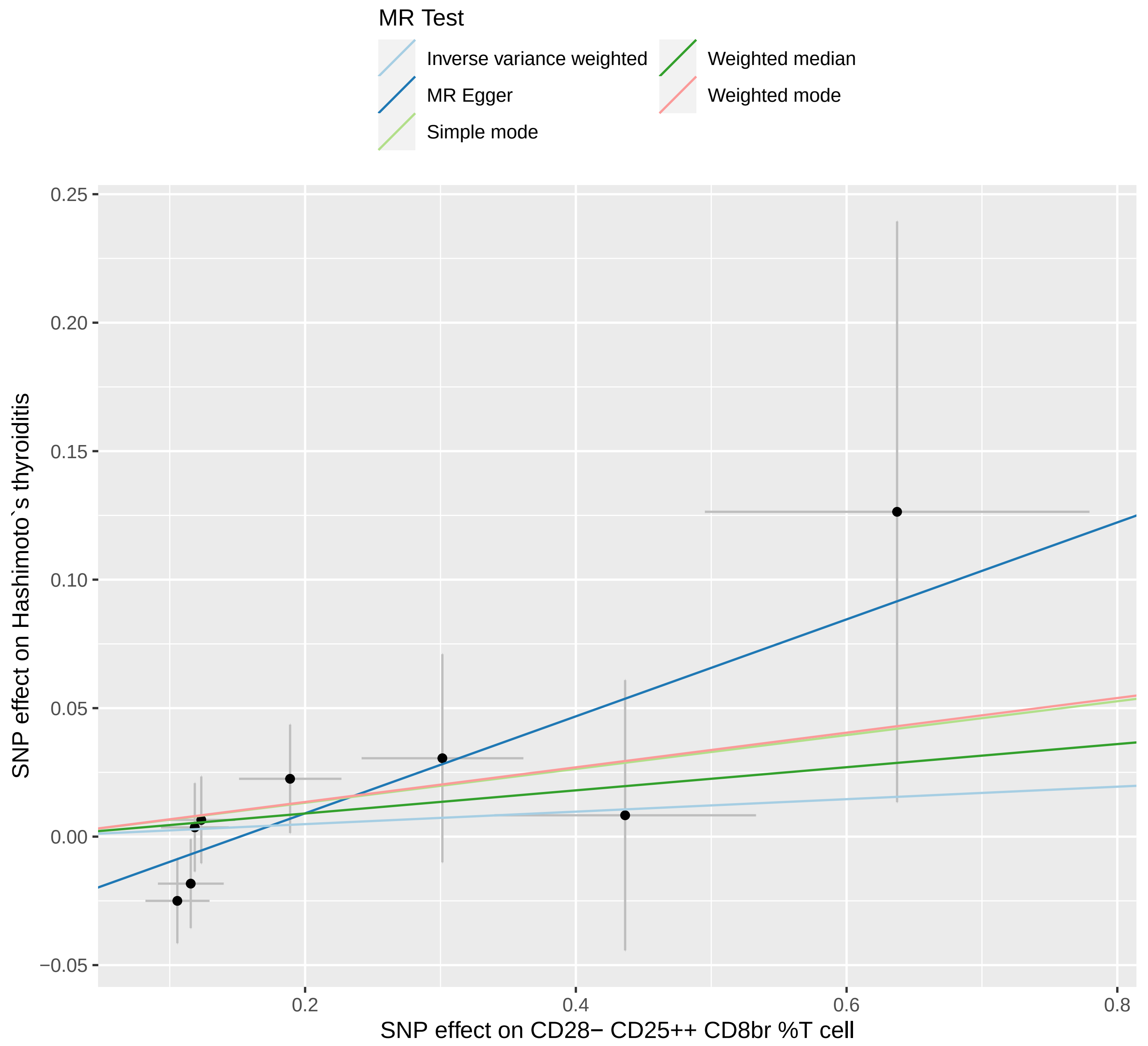

MR Test
Inverse variance weighted
MR Egger
Simple mode
Weighted median
Weighted mode
0.25
0.20
0.15
0.10
0.05
0.00
−0.05
0.2
0.4
0.6
0.8
SNP effect on Hashimoto`s thyroiditis
SNP effect on CD28− CD25++ CD8br %T cell

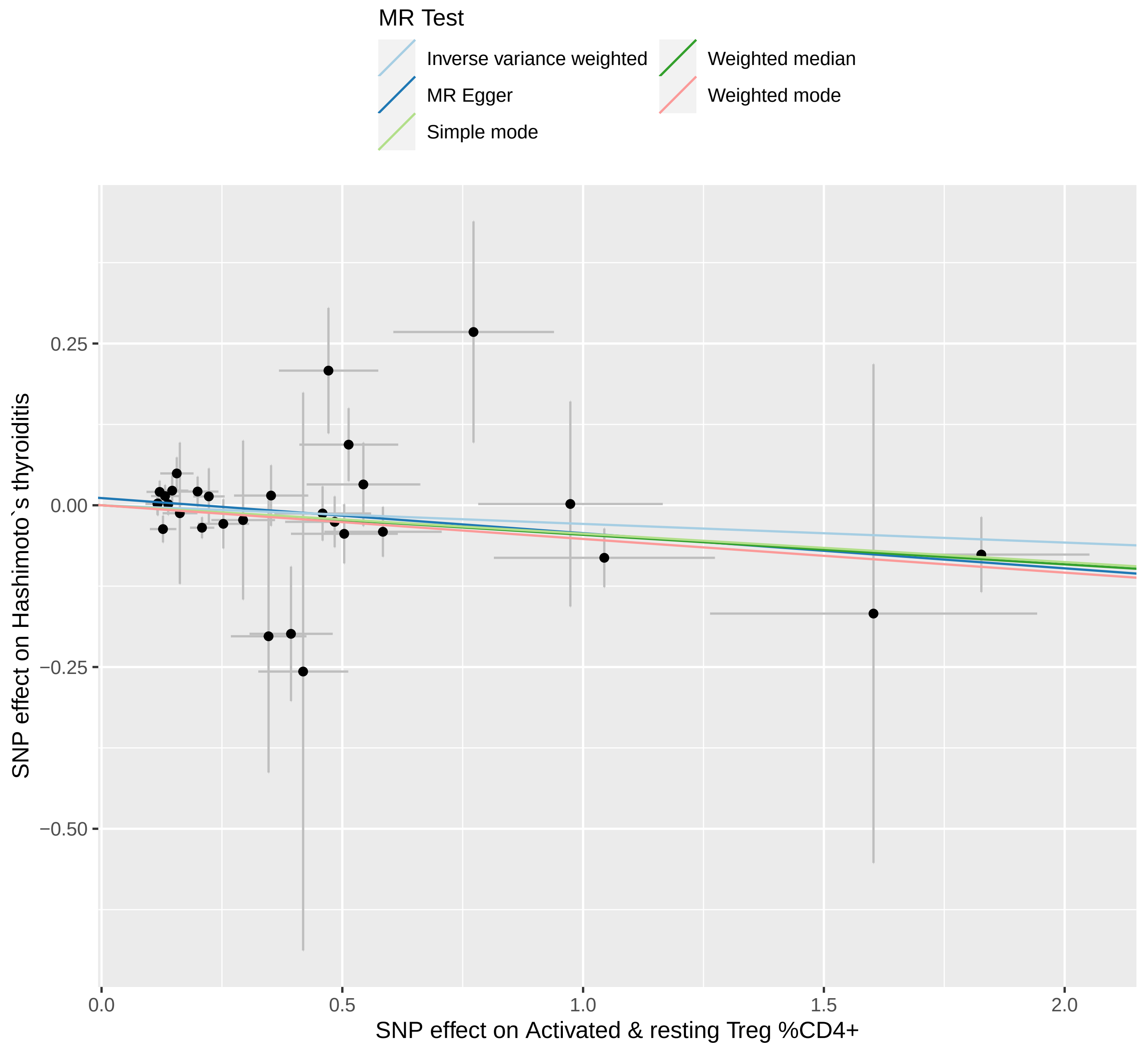

MR Test
Inverse variance weighted
MR Egger
Simple mode
Weighted median
Weighted mode
0.25
0.00
−0.25
−0.50
0.0
0.5
1.0
1.5
2.0
SNP effect on Hashimoto`s thyroiditis
SNP effect on Activated & resting Treg %CD4+

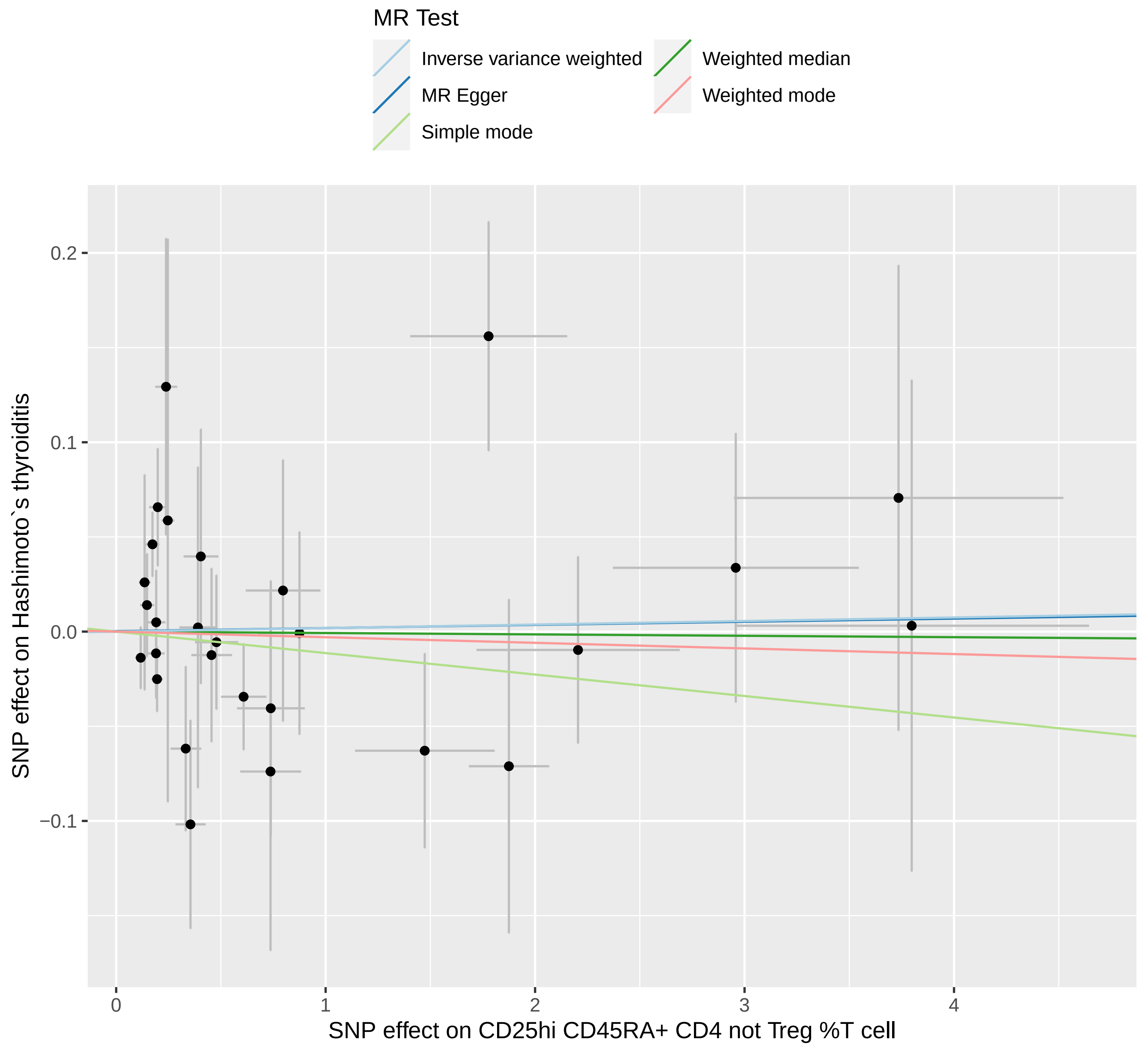

MR Test
Inverse variance weighted
Weighted median
MR Egger
Weighted mode
Simple mode
0.2
0.1
0.0
−0.1
0
1
2
3
4
SNP effect on Hashimoto`s thyroiditis
SNP effect on CD25hi CD45RA+ CD4 not Treg %T cell

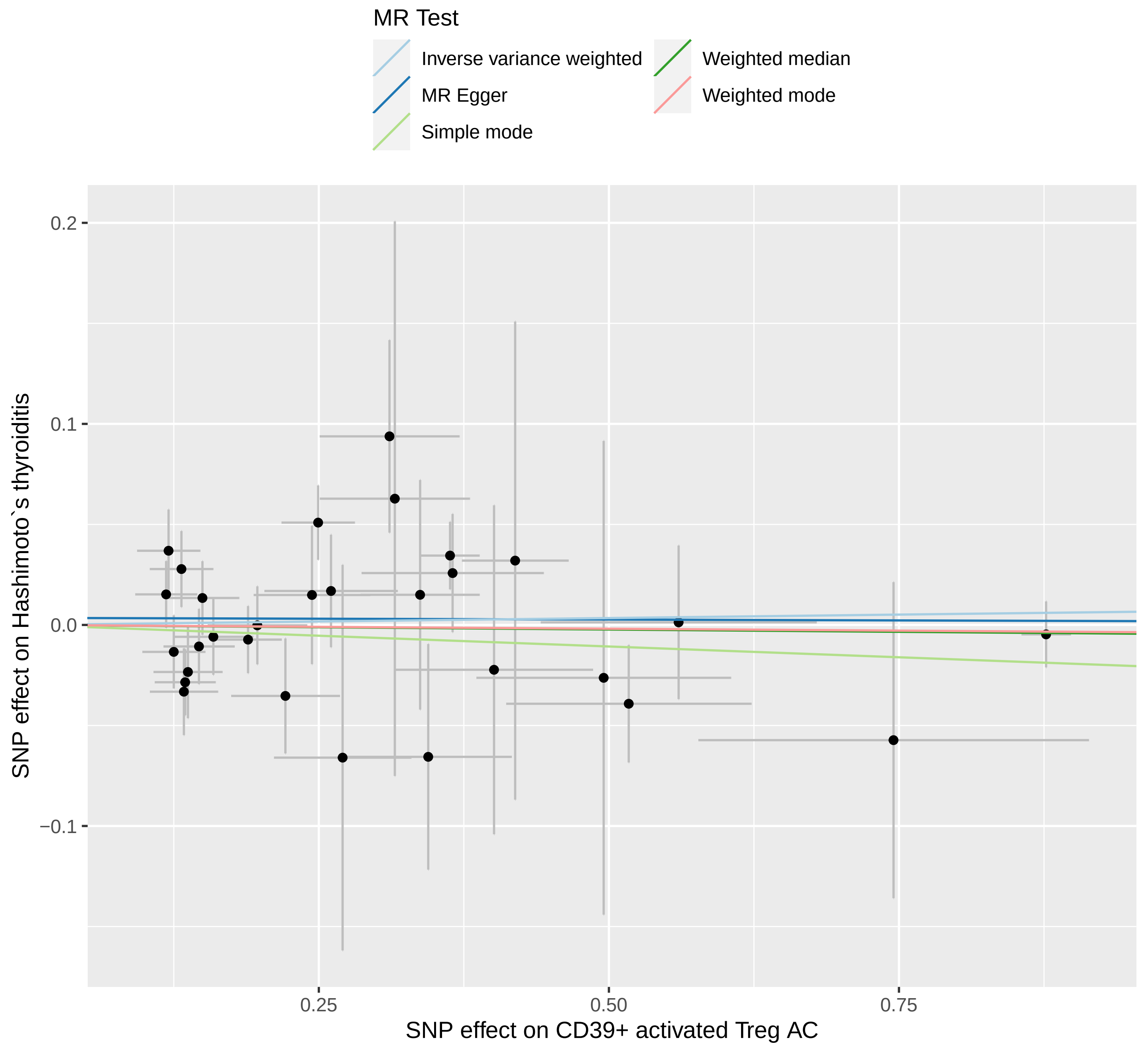
MR Test
Inverse variance weighted
MR Egger
Simple mode
Weighted median
Weighted mode
0.2
0.1
0.0
−0.1
0.25
0.50
0.75
SNP effect on Hashimoto`s thyroiditis
SNP effect on CD39+ activated Treg AC

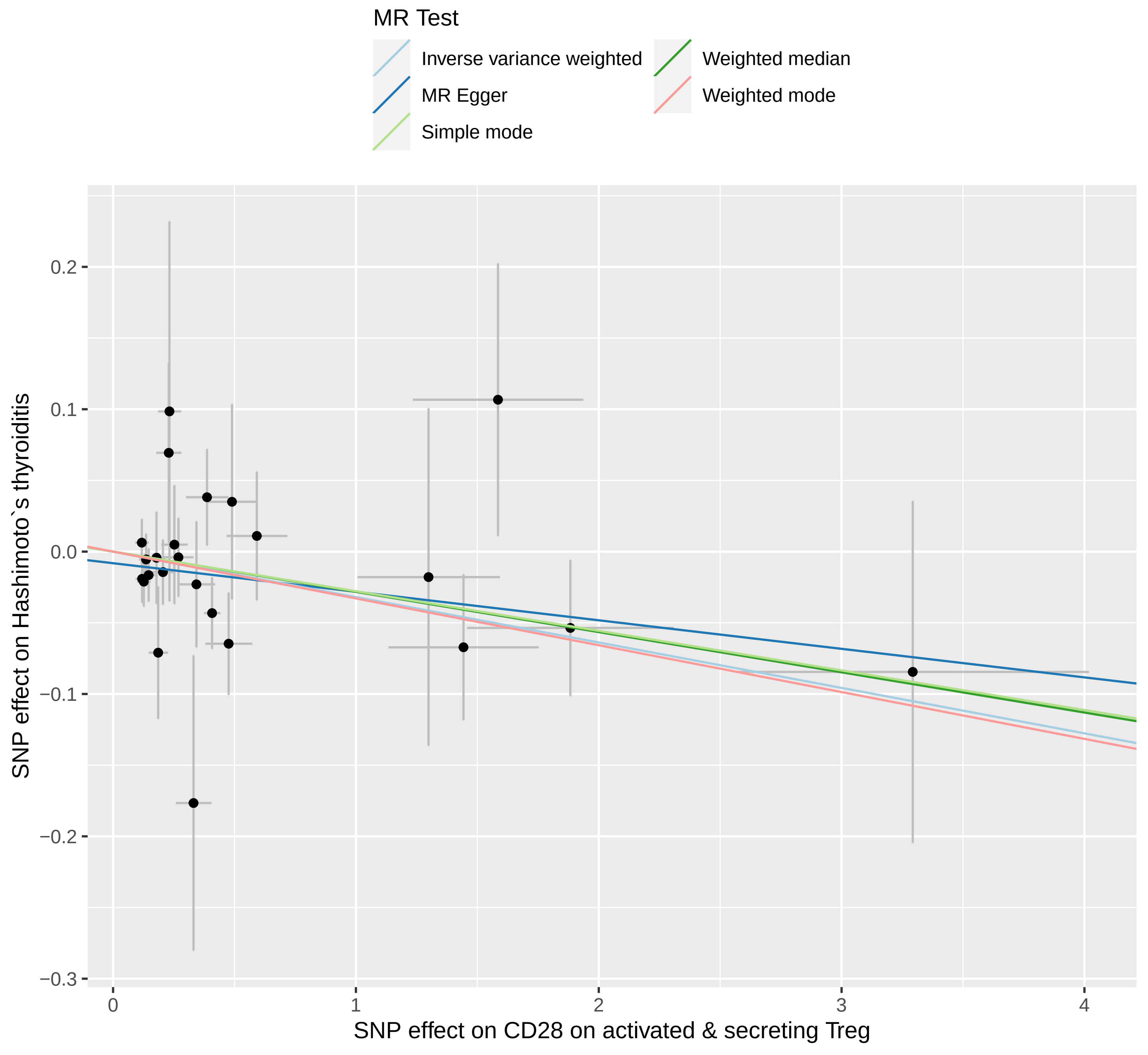
MR Test
Inverse variance weighted
MR Egger
Simple mode
Weighted median
Weighted mode
0.2
0.1
0.0
−0.1
−0.2
−0.3
0
1
2
3
4
SNP effect on Hashimoto`s thyroiditis
SNP effect on CD28 on activated & secreting Treg

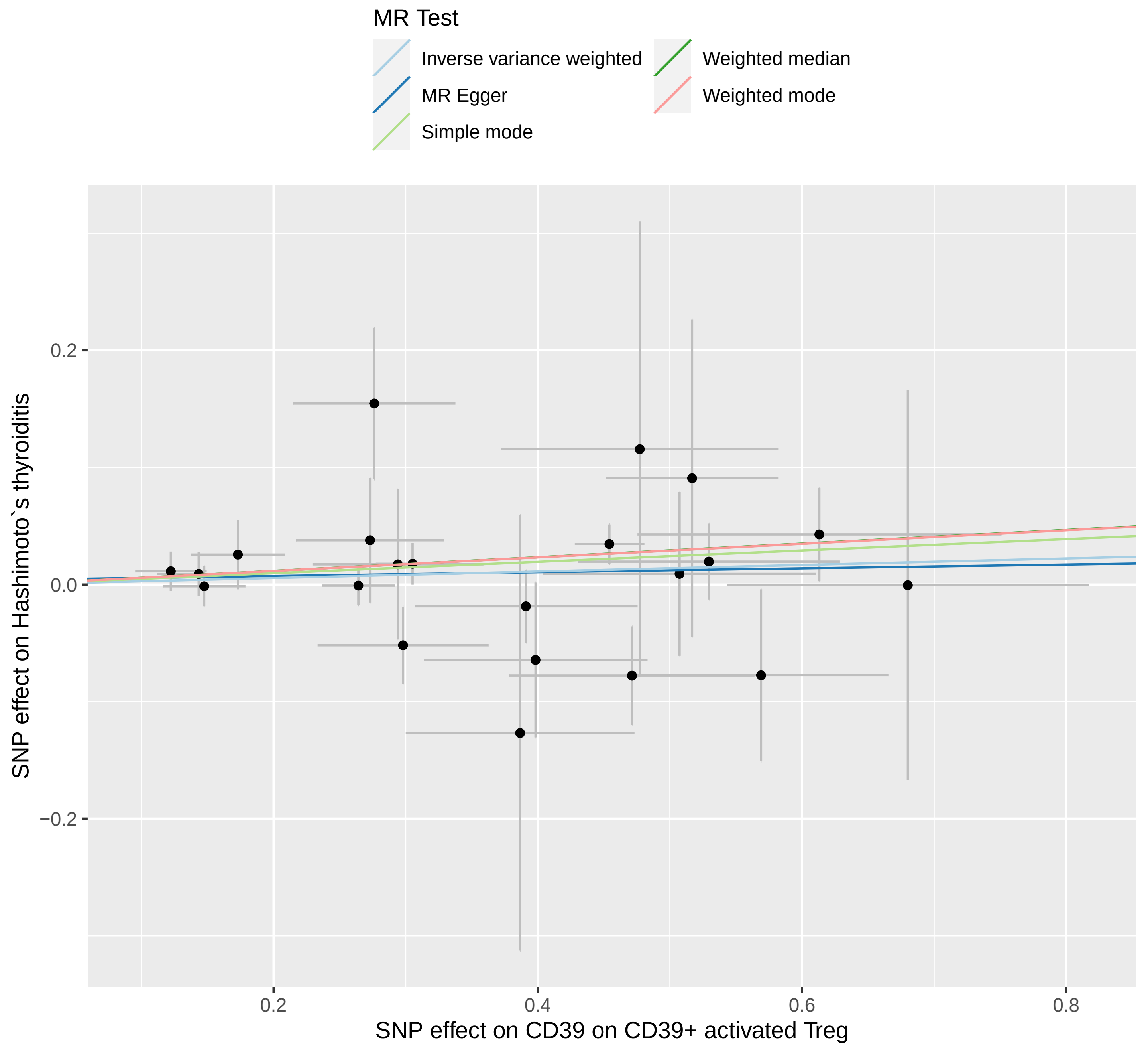
MR Test
Inverse variance weighted
MR Egger
Simple mode
Weighted median
Weighted mode
SNP effect on Hashimoto`s thyroiditis
0.2
0.0
−0.2
0.2
0.4
0.6
0.8
SNP effect on CD39 on CD39+ activated Treg

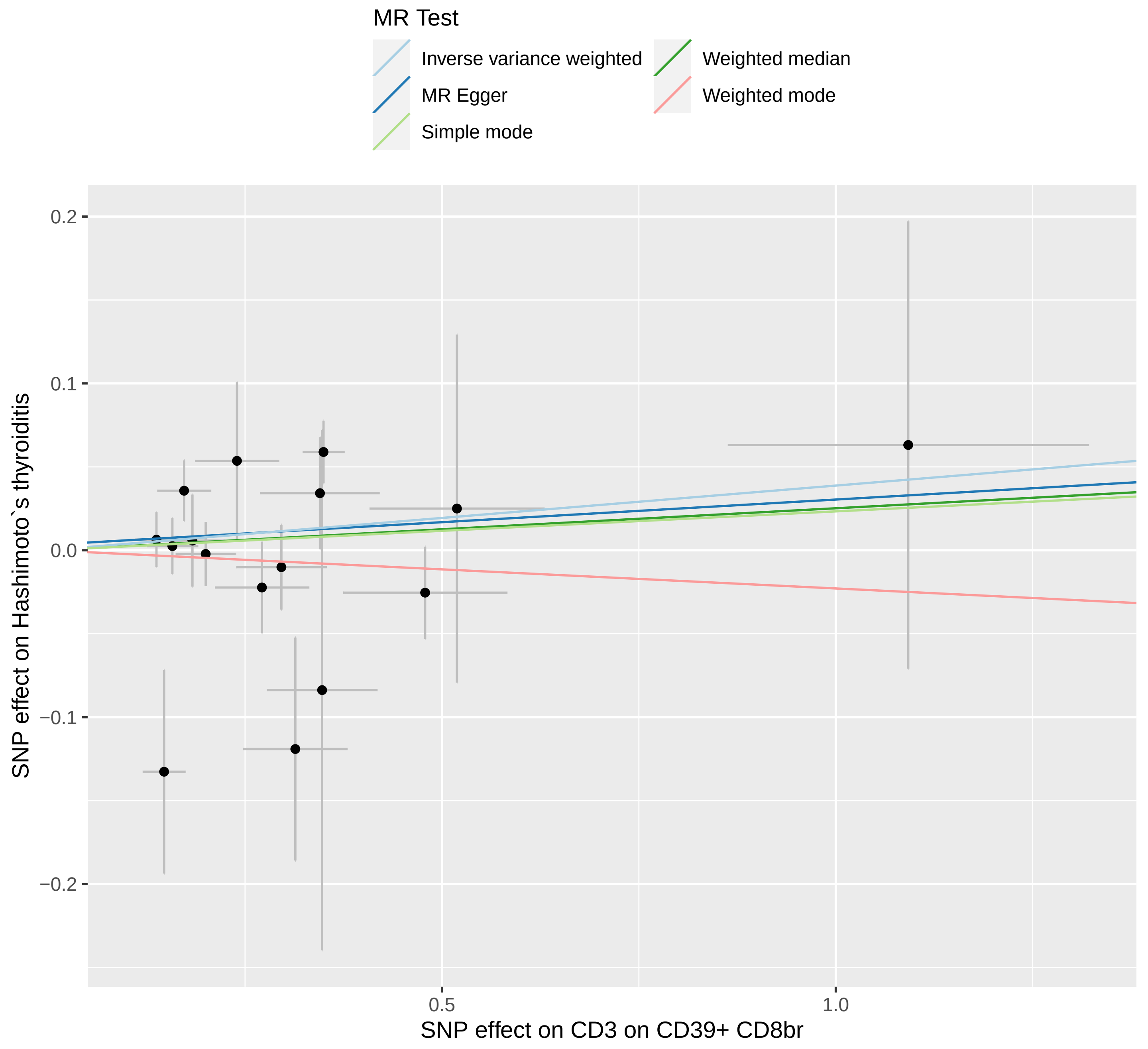
MR Test
Inverse variance weighted
MR Egger
Simple mode
Weighted median
Weighted mode
0.2
0.1
0.0
−0.1
−0.2
0.5
1.0
SNP effect on Hashimoto`s thyroiditis
SNP effect on CD3 on CD39+ CD8br

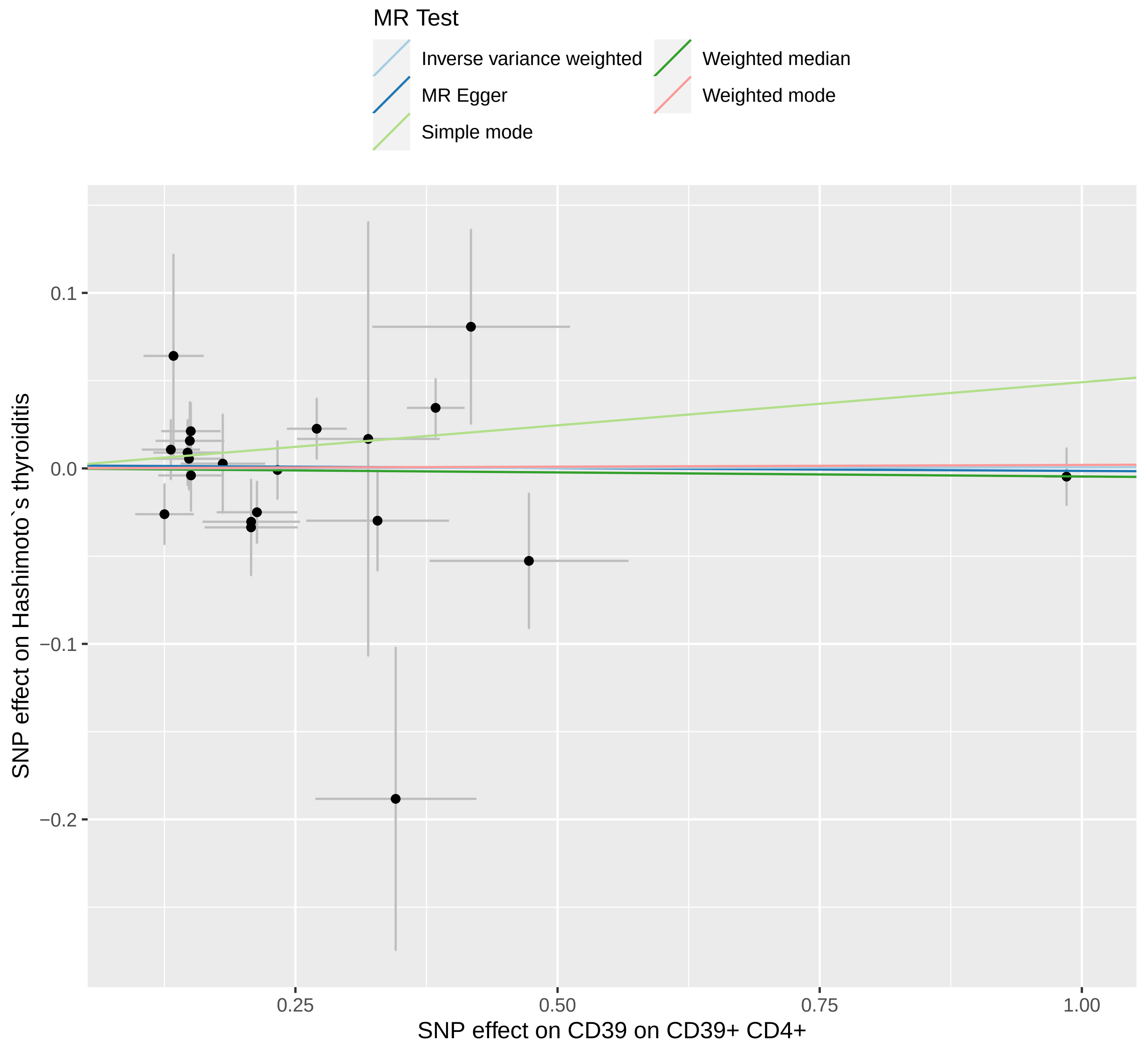
MR Test
Inverse variance weighted
MR Egger
Simple mode
Weighted median
Weighted mode
0.1
0.0
−0.1
−0.2
SNP effect on Hashimoto`s thyroiditis
0.25
0.50
0.75
1.00
SNP effect on CD39 on CD39+ CD4+

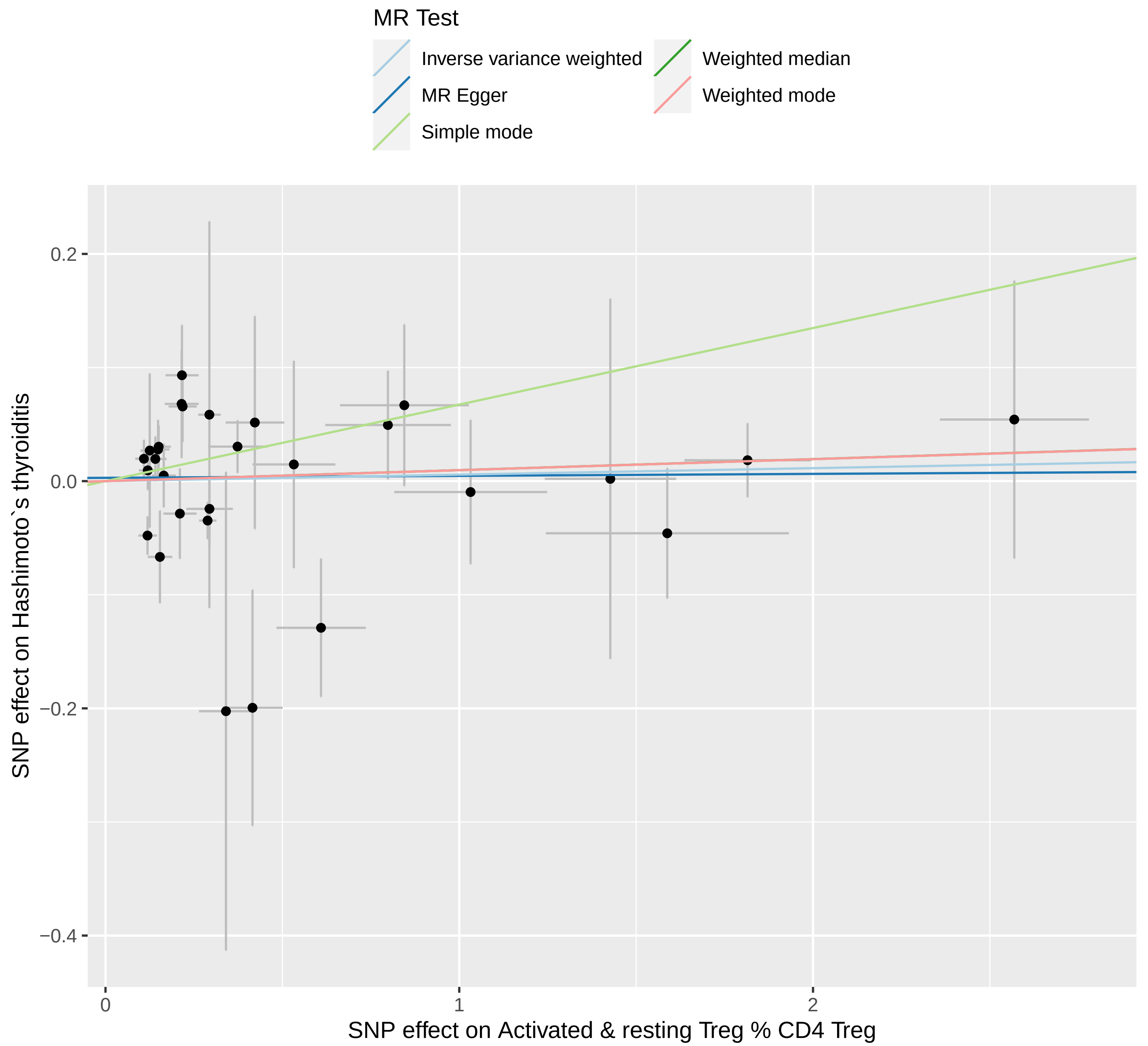
MR Test
Inverse variance weighted
MR Egger
Simple mode
Weighted median
Weighted mode
0.2
0.0
−0.2
−0.4
0
1
2
SNP effect on Hashimoto`s thyroiditis
SNP effect on Activated & resting Treg % CD4 Treg

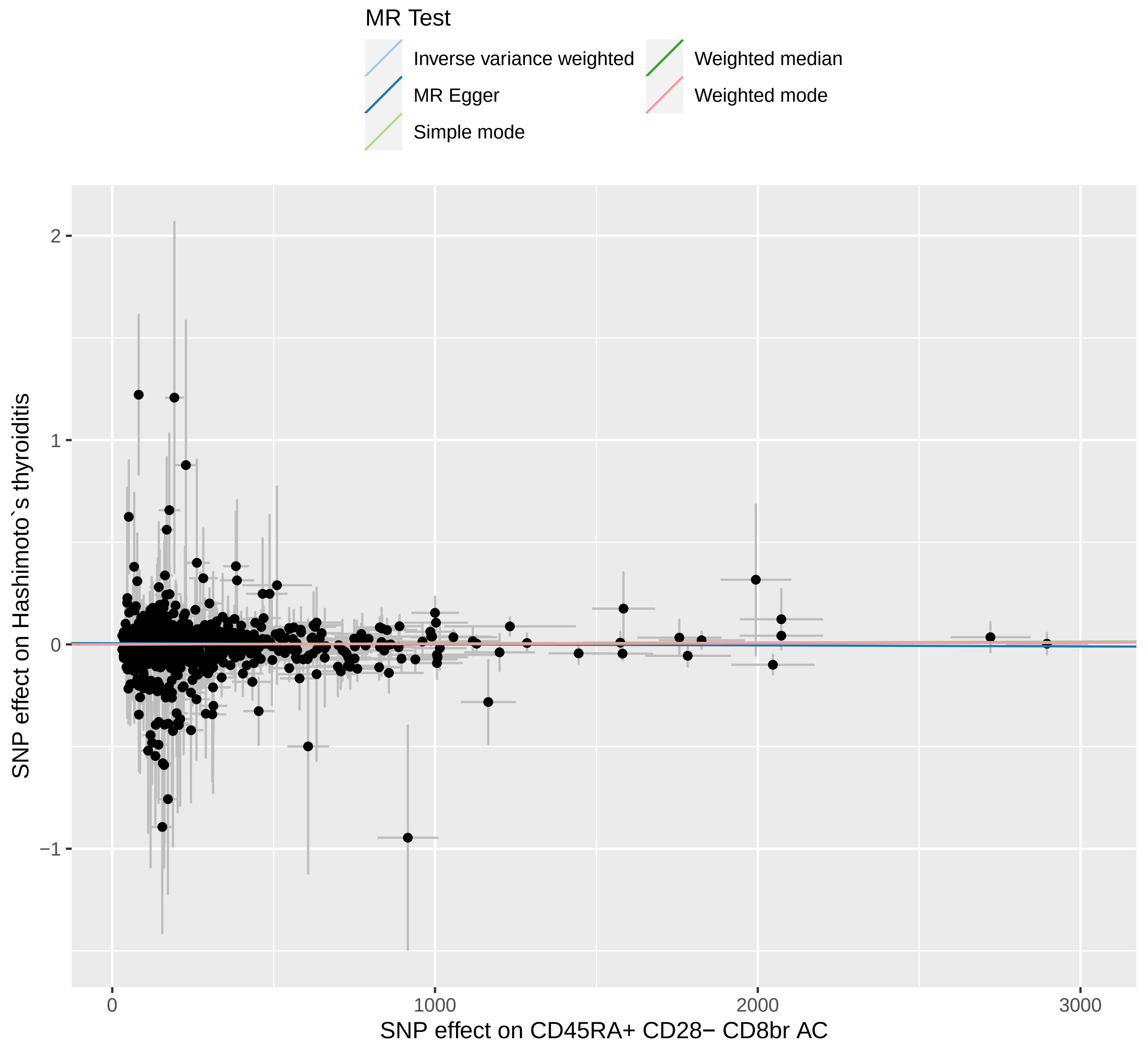
MR Test
Inverse variance weighted
MR Egger
Simple mode
Weighted median
Weighted mode
2
1
0
−1
0
1000
2000
3000
SNP effect on Hashimoto`s thyroiditis
SNP effect on CD45RA+ CD28− CD8br AC

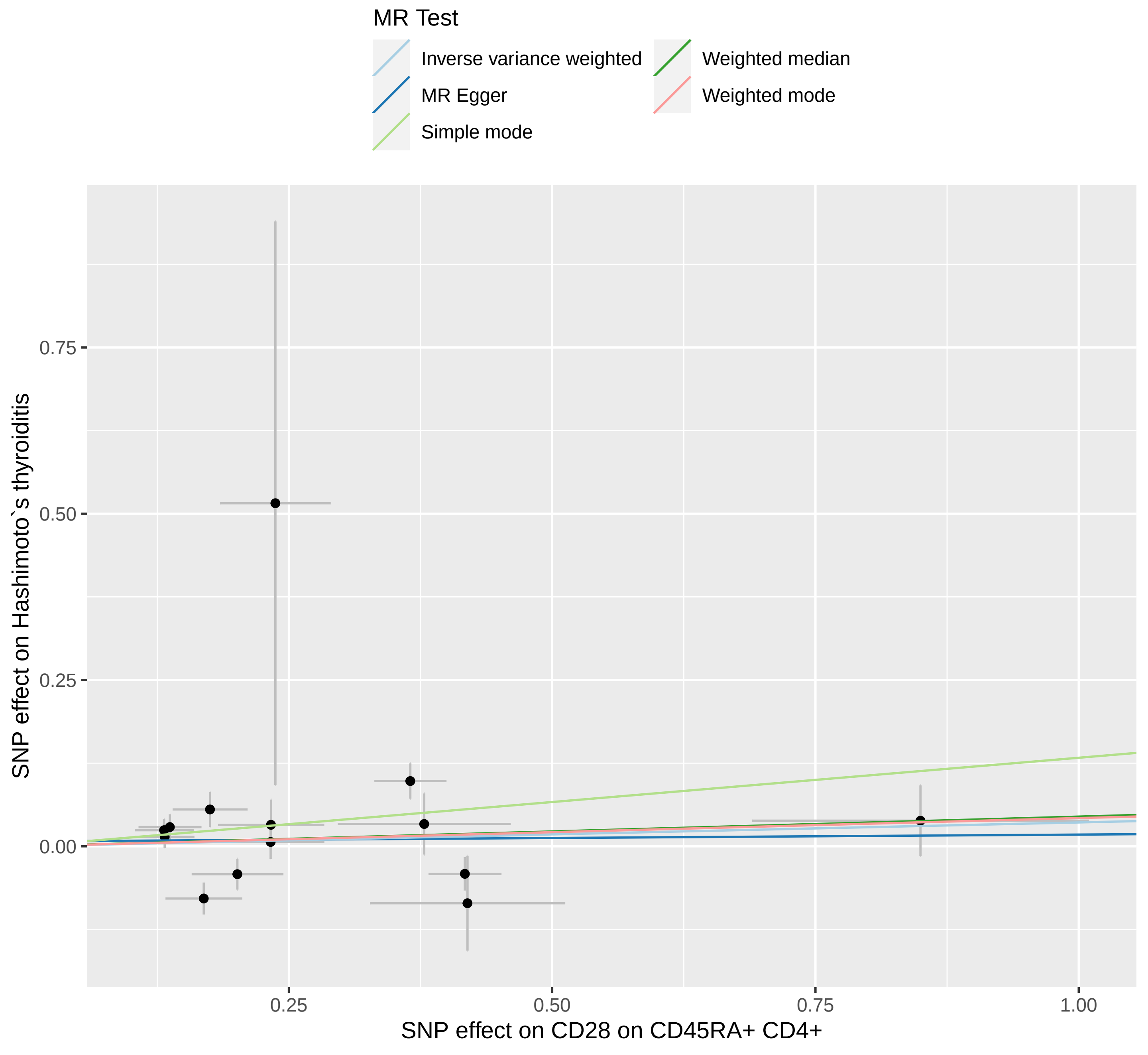
MR Test
Inverse variance weighted
MR Egger
Simple mode
Weighted median
Weighted mode
SNP effect on Hashimoto`s thyroiditis
0.75
0.50
0.25
0.00
0.25
0.50
0.75
1.00
SNP effect on CD28 on CD45RA+ CD4+

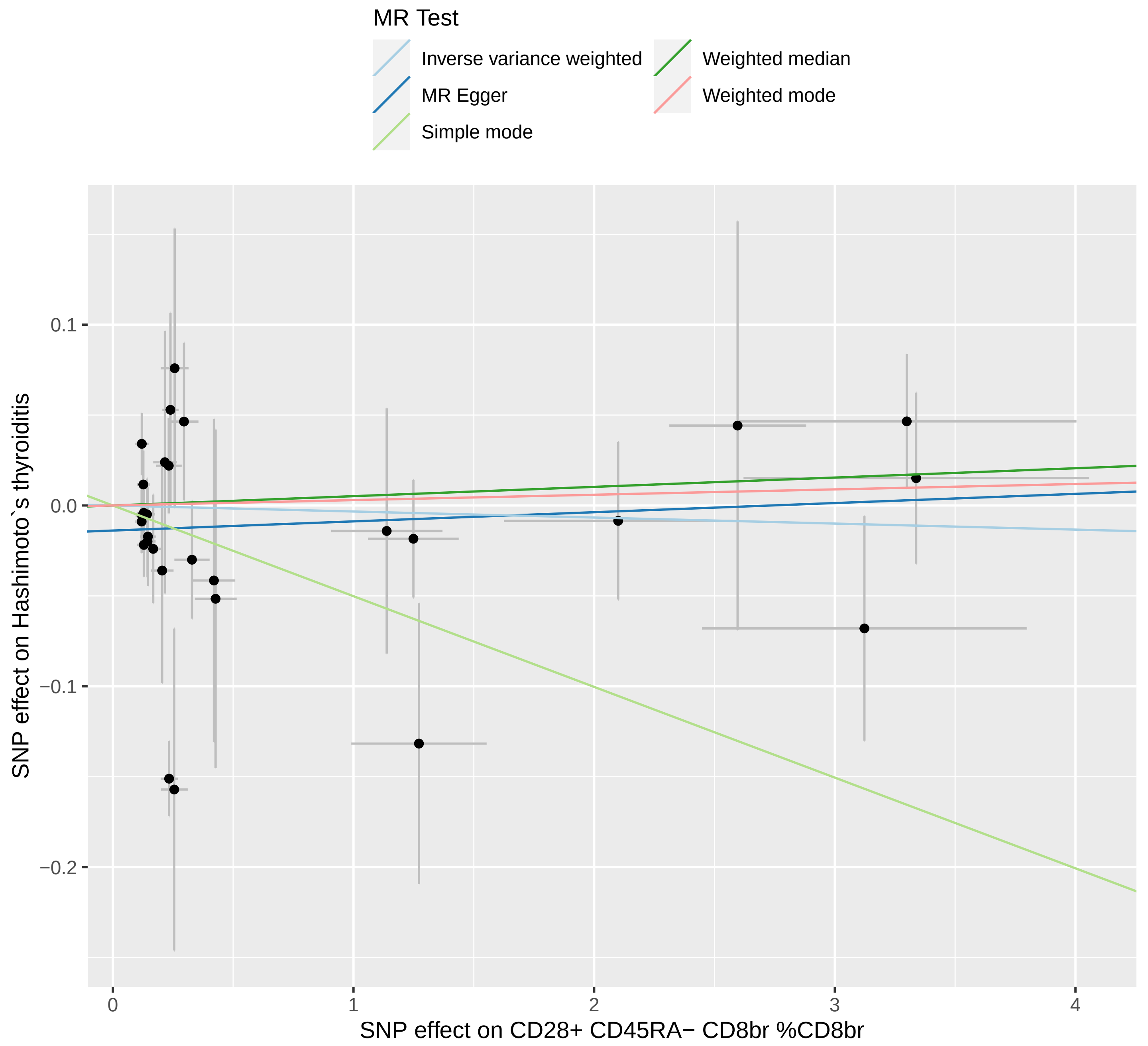
MR Test
Inverse variance weighted
MR Egger
Simple mode
Weighted median
Weighted mode
SNP effect on Hashimoto`s thyroiditis
0.1
0.0
−0.1
−0.2
0
1
2
3
4
SNP effect on CD28+ CD45RA− CD8br %CD8br

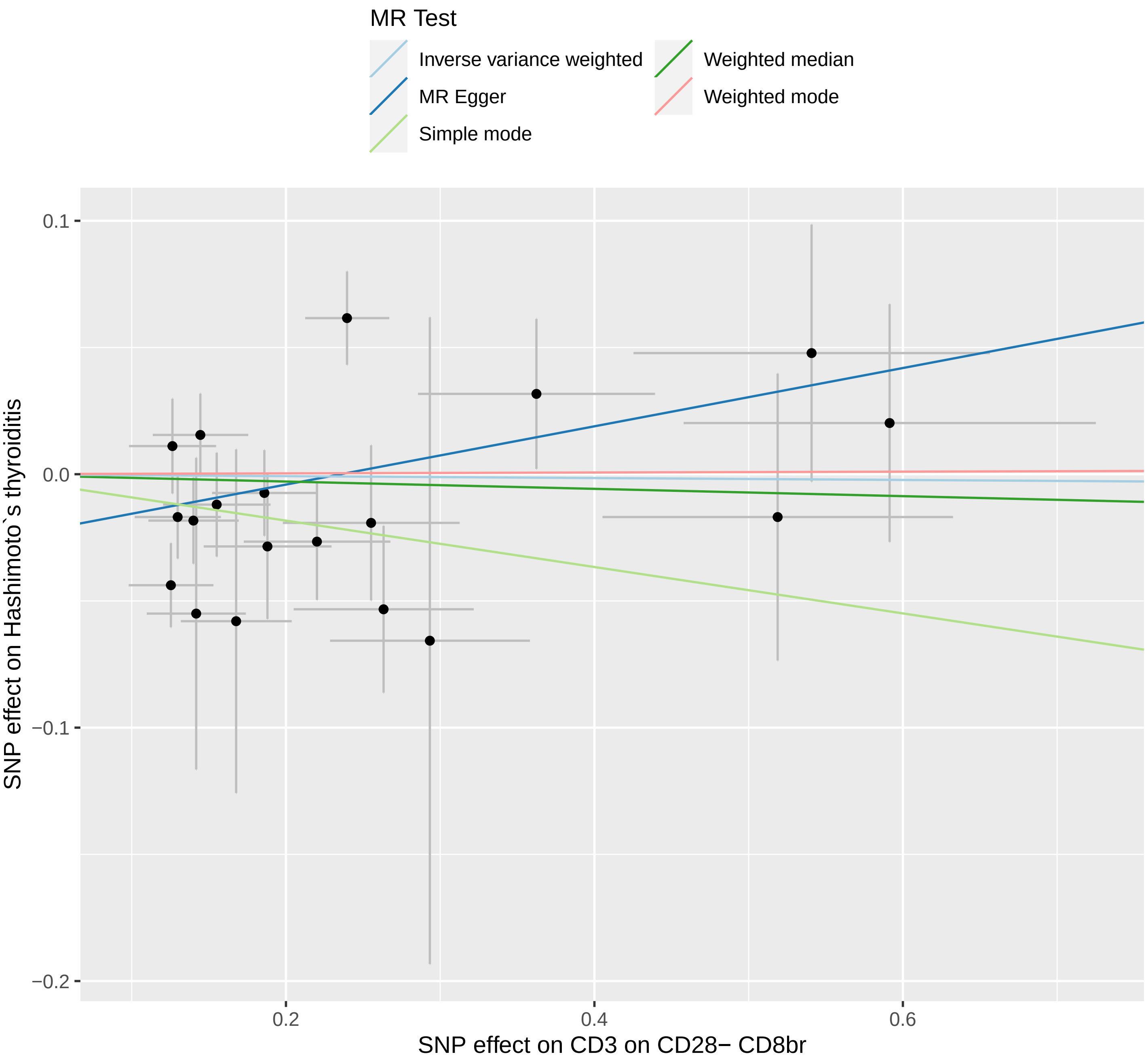
MR Test
Inverse variance weighted
MR Egger
Simple mode
Weighted median
Weighted mode
0.1
0.0
−0.1
−0.2
0.2
0.4
0.6
SNP effect on Hashimoto`s thyroiditis
SNP effect on CD3 on CD28− CD8br

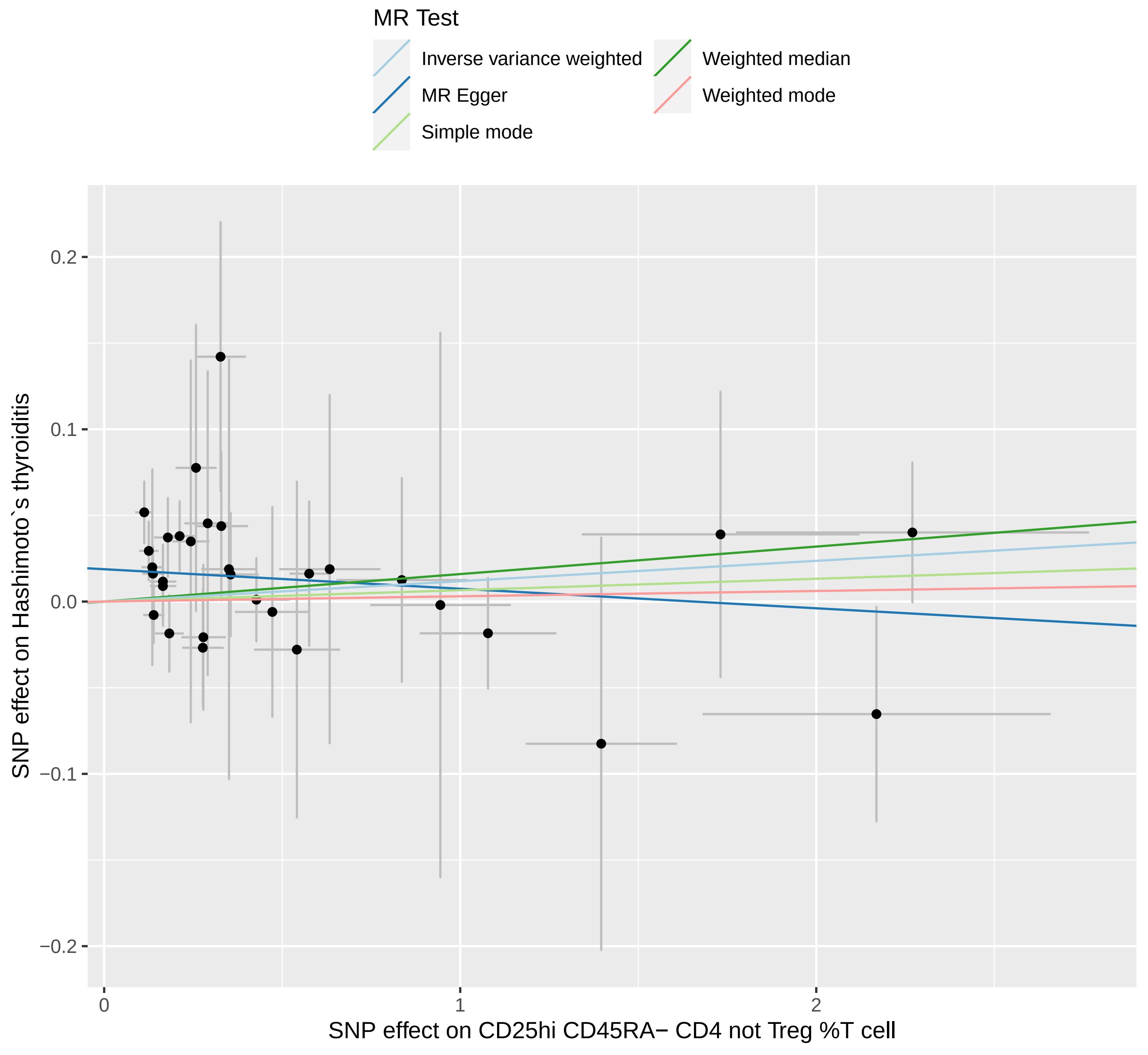
MR Test
Inverse variance weighted
MR Egger
Simple mode
Weighted median
Weighted mode
SNP effect on Hashimoto`s thyroiditis
SNP effect on CD25hi CD45RA− CD4 not Treg %T cell
0.2
0.1
0.0
−0.1
−0.2
0
1
2

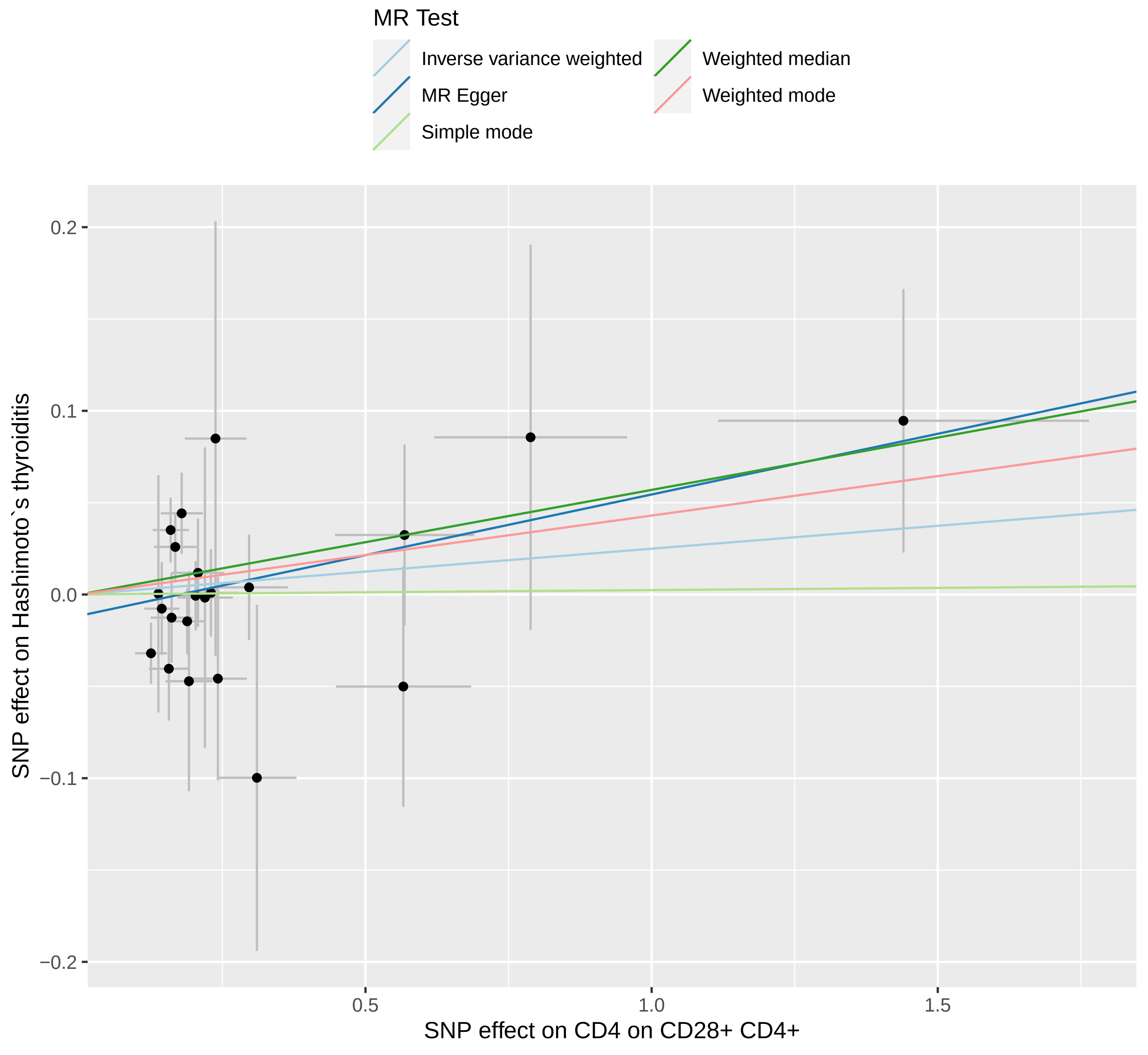

MR Test
Inverse variance weighted
MR Egger
Simple mode
Weighted median
Weighted mode
0.2
0.1
0.0
−0.1
−0.2
SNP effect on Hashimoto`s thyroiditis
0.5
1.0
1.5
SNP effect on CD4 on CD28+ CD4+

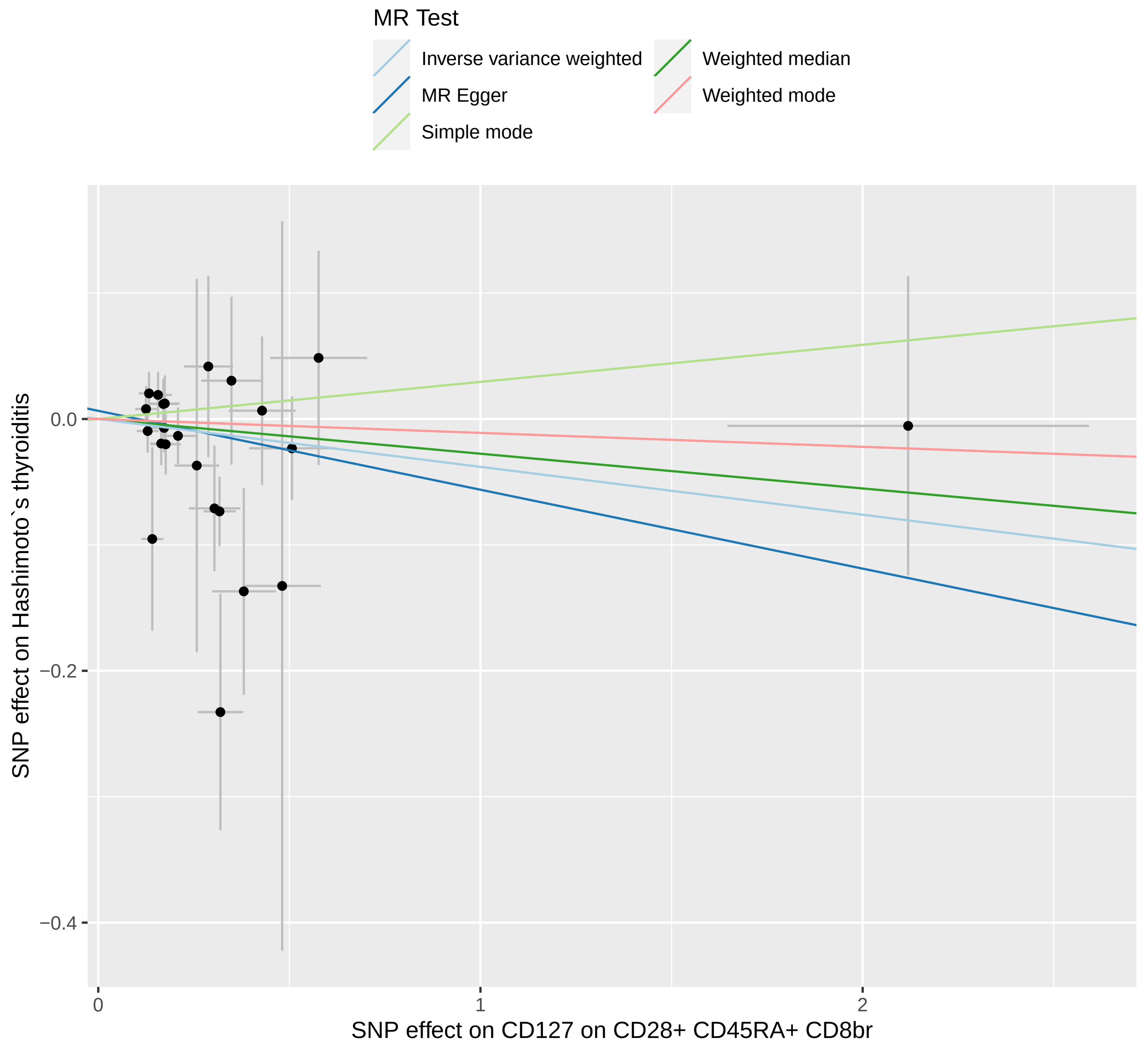

MR Test
Inverse variance weighted
MR Egger
Simple mode
Weighted median
Weighted mode
SNP effect on Hashimoto`s thyroiditis
0.0
−0.2
−0.4
0
1
2
SNP effect on CD127 on CD28+ CD45RA+ CD8br

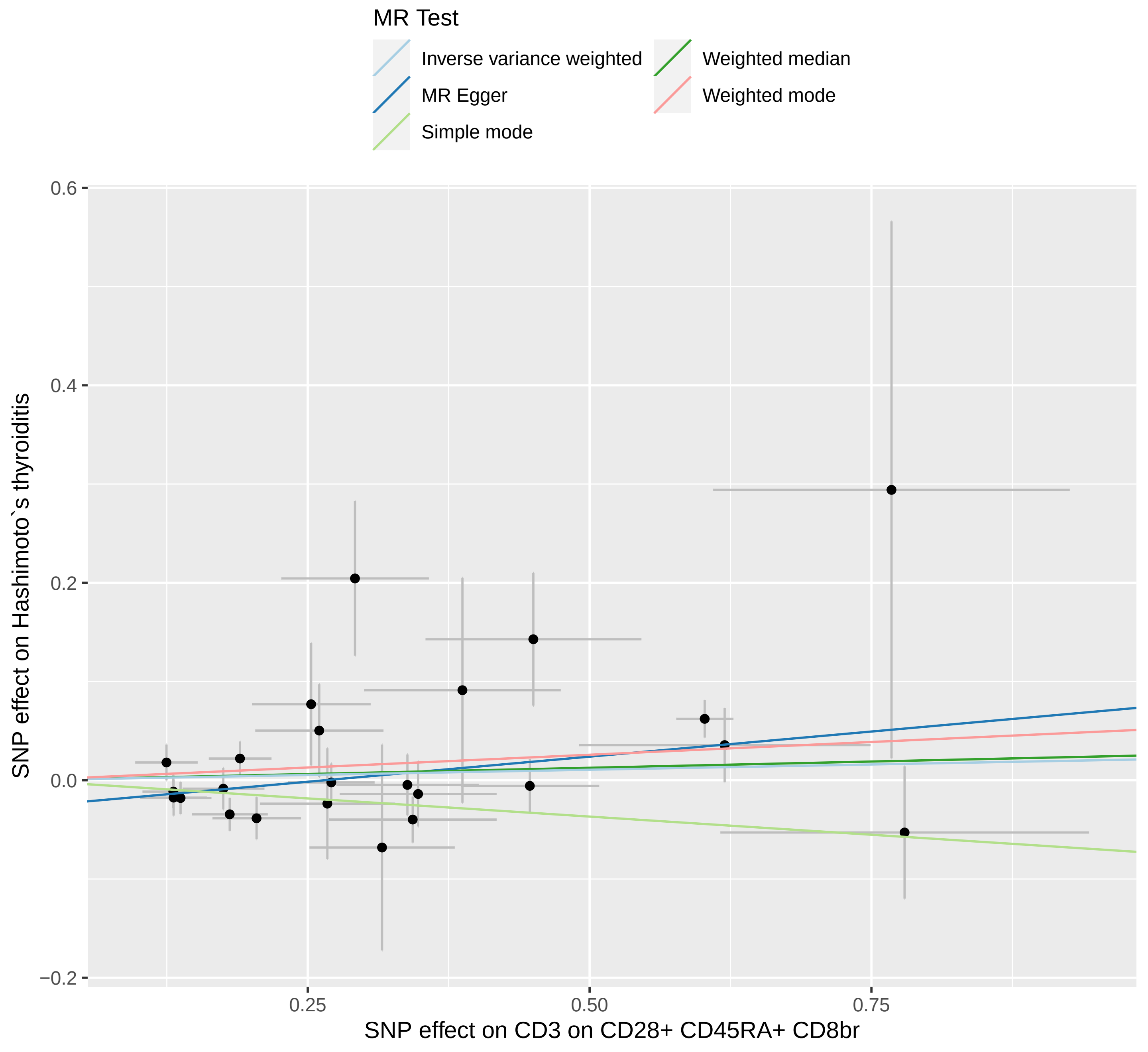
MR Test
Inverse variance weighted
MR Egger
Simple mode
Weighted median
Weighted mode
0.6
0.4
0.2
0.0
−0.2
0.25
0.50
0.75
SNP effect on Hashimoto`s thyroiditis
SNP effect on CD3 on CD28+ CD45RA+ CD8br
