# Supplementary material for: Treg cells as a protective factor for Hashimoto`s thyroiditis: a Mendelian randomization study
Source: Front Endocrinol (Lausanne). 2024 Mar 8;15:1347695. doi: 10.3389/fendo.2024.1347695 (PMC10957564; doi:10.3389/fendo.2024.1347695)
Supplement: Supplementary file 3 [file Image_3.pdf]

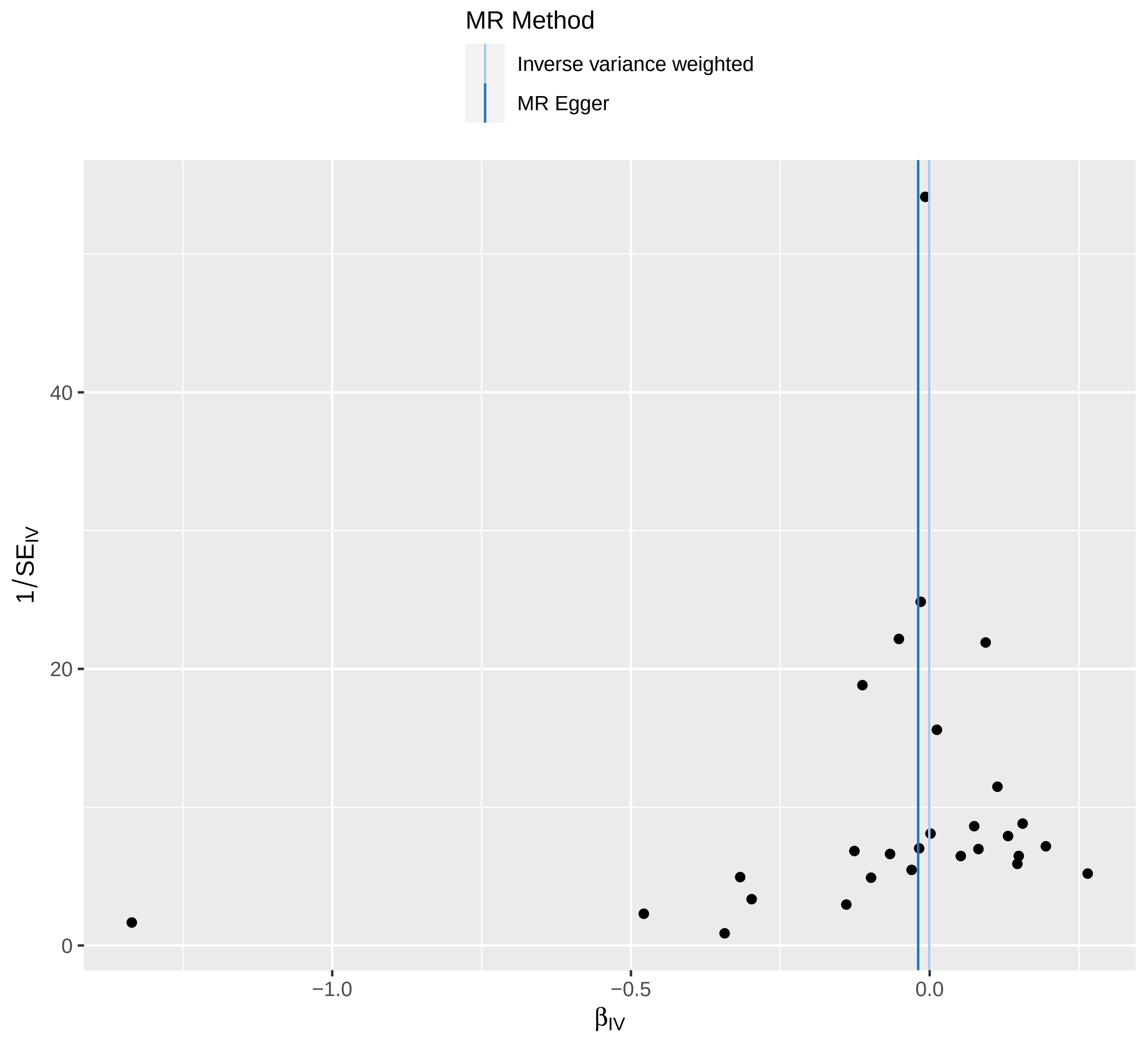
MR Method
Inverse variance weighted
MR Egger
$1/SE_{IV}$
0
20
40
−1.0
−0.5
0.0
β_{IV}

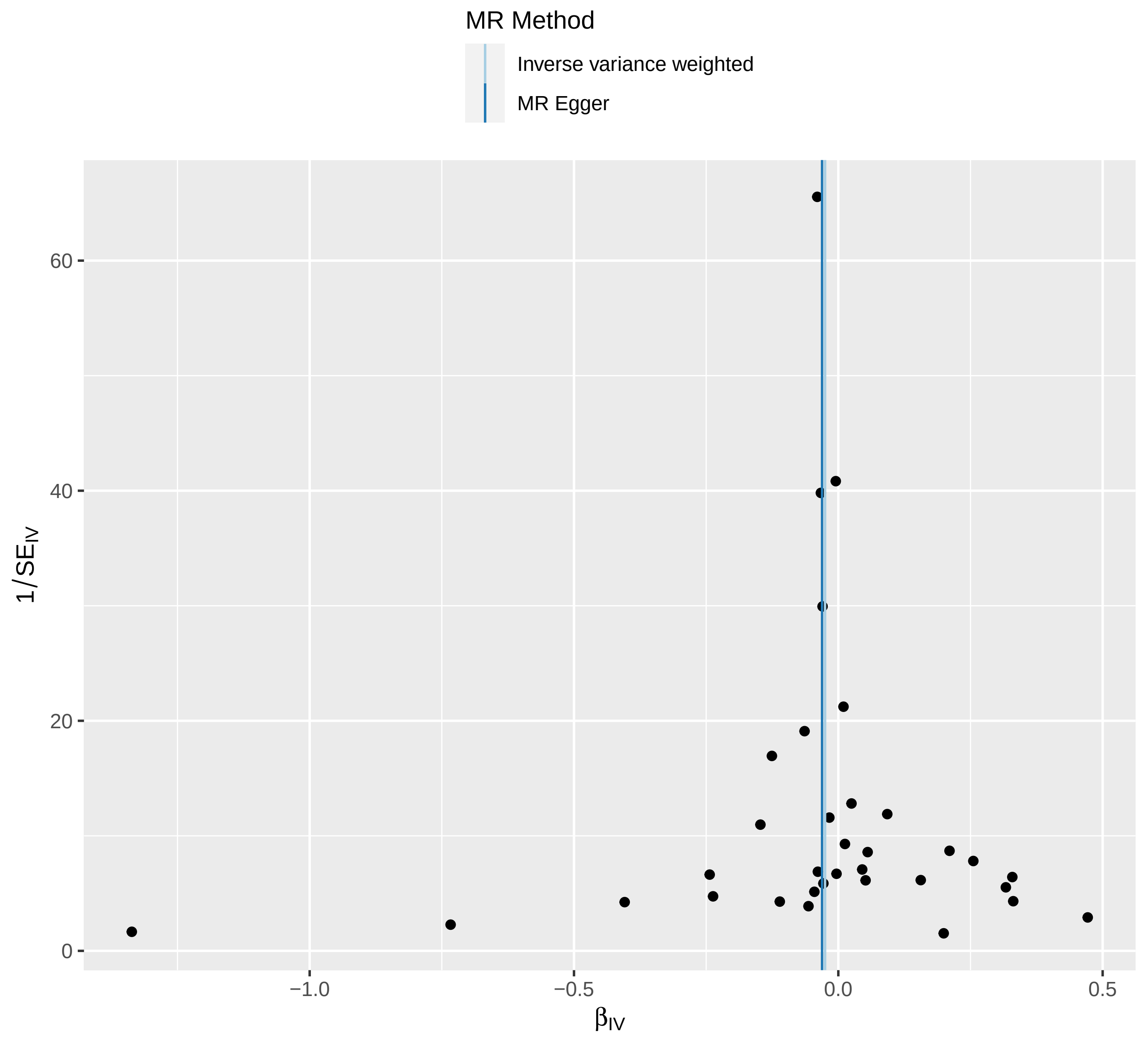
MR Method
Inverse variance weighted
MR Egger
60
40
20
0
1/SE_IV
−1.0
−0.5
0.0
0.5
β_IV

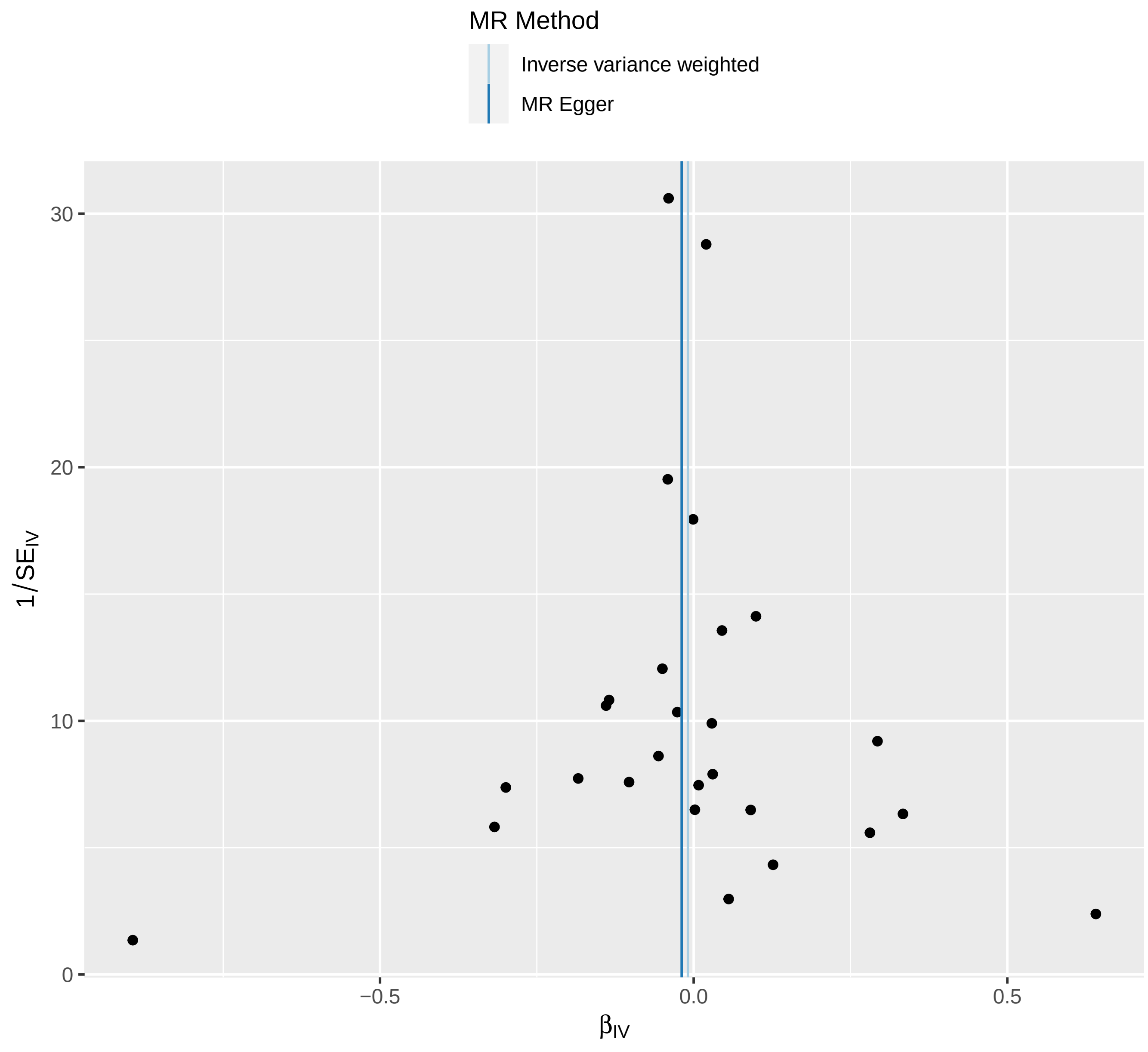
MR Method
Inverse variance weighted
MR Egger
$1/SE_{IV}$
30
20
10
0
−0.5
0.0
0.5
β_{IV}

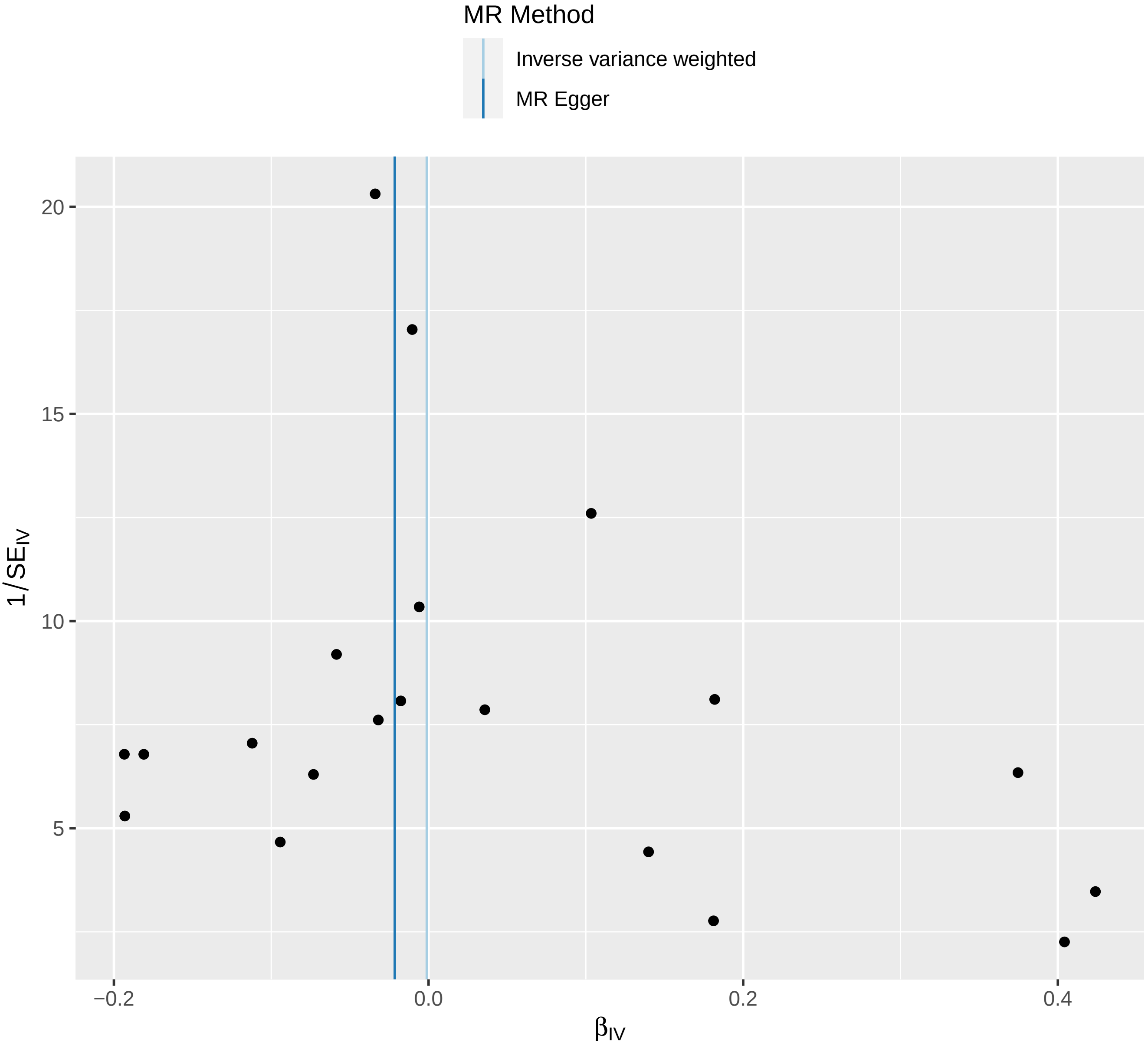

MR Method
Inverse variance weighted
MR Egger
20
15
10
5
−0.2
0.0
0.2
0.4
1/SE$_{IV}$
β_{IV}

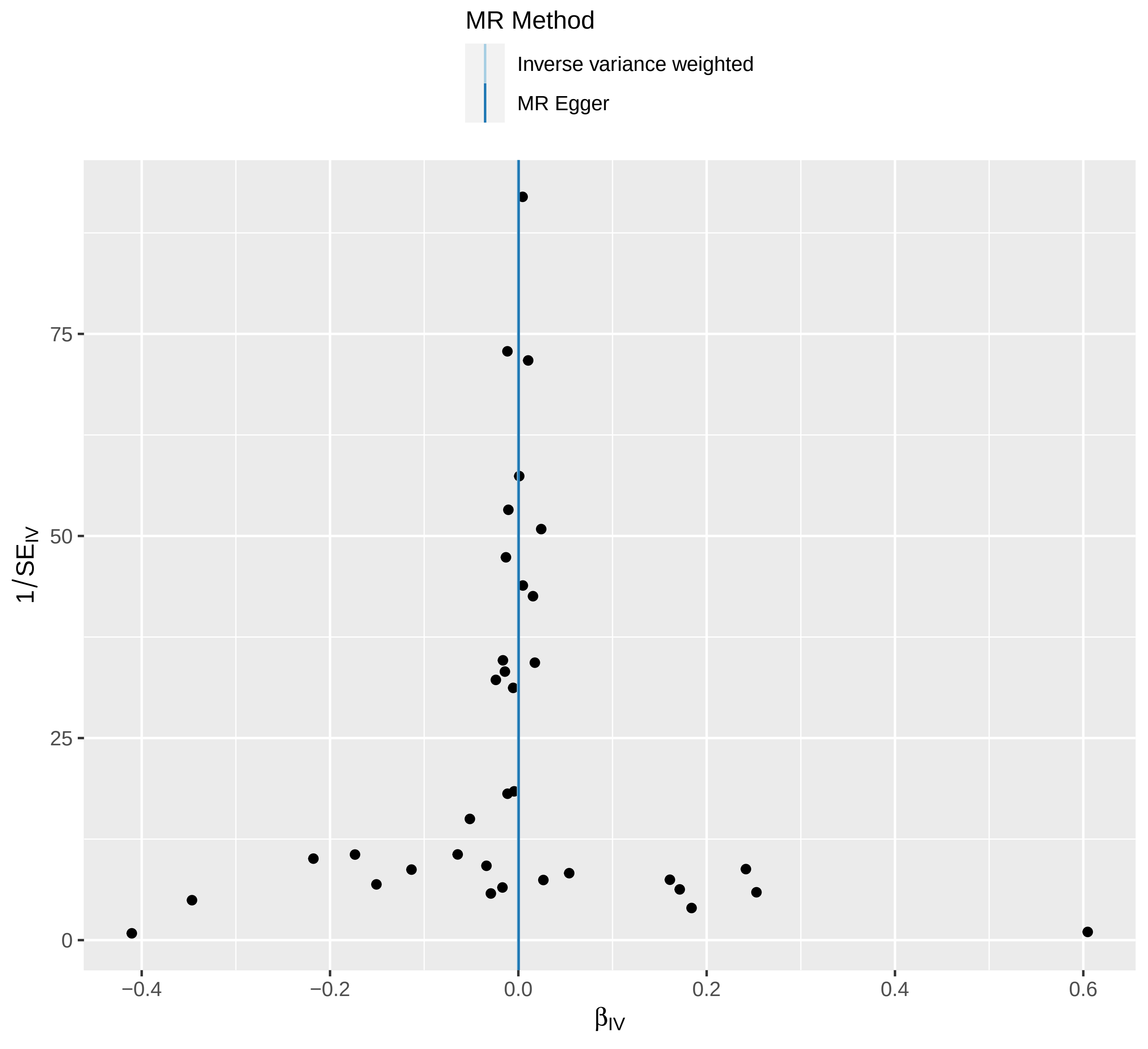

MR Method
Inverse variance weighted
MR Egger
75
50
25
0
1/SE_IV
−0.4
−0.2
0.0
0.2
0.4
0.6
β_IV

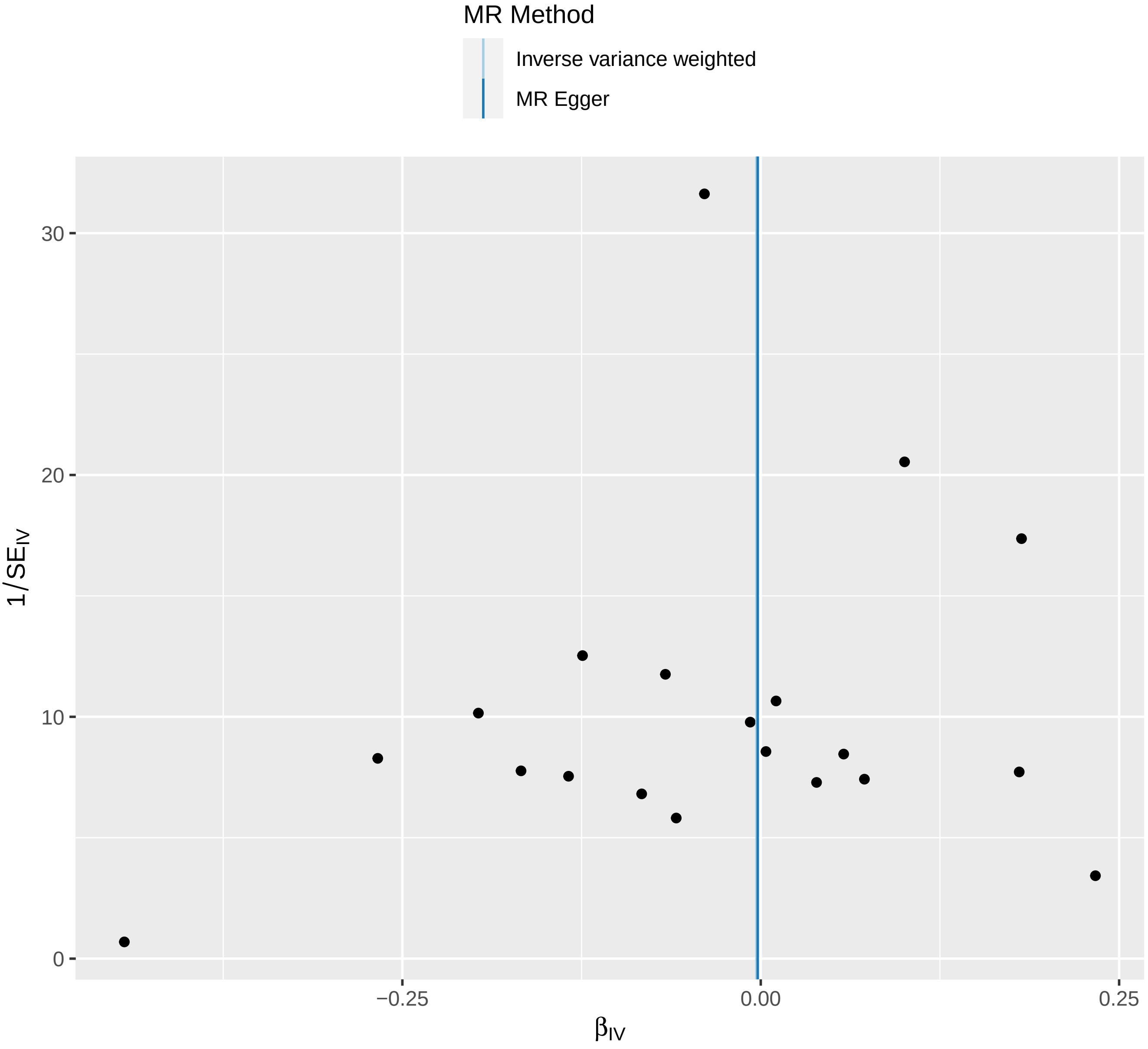
MR Method
Inverse variance weighted
MR Egger
30
20
10
0
1/SE_IV
−0.25
0.00
0.25
β_IV

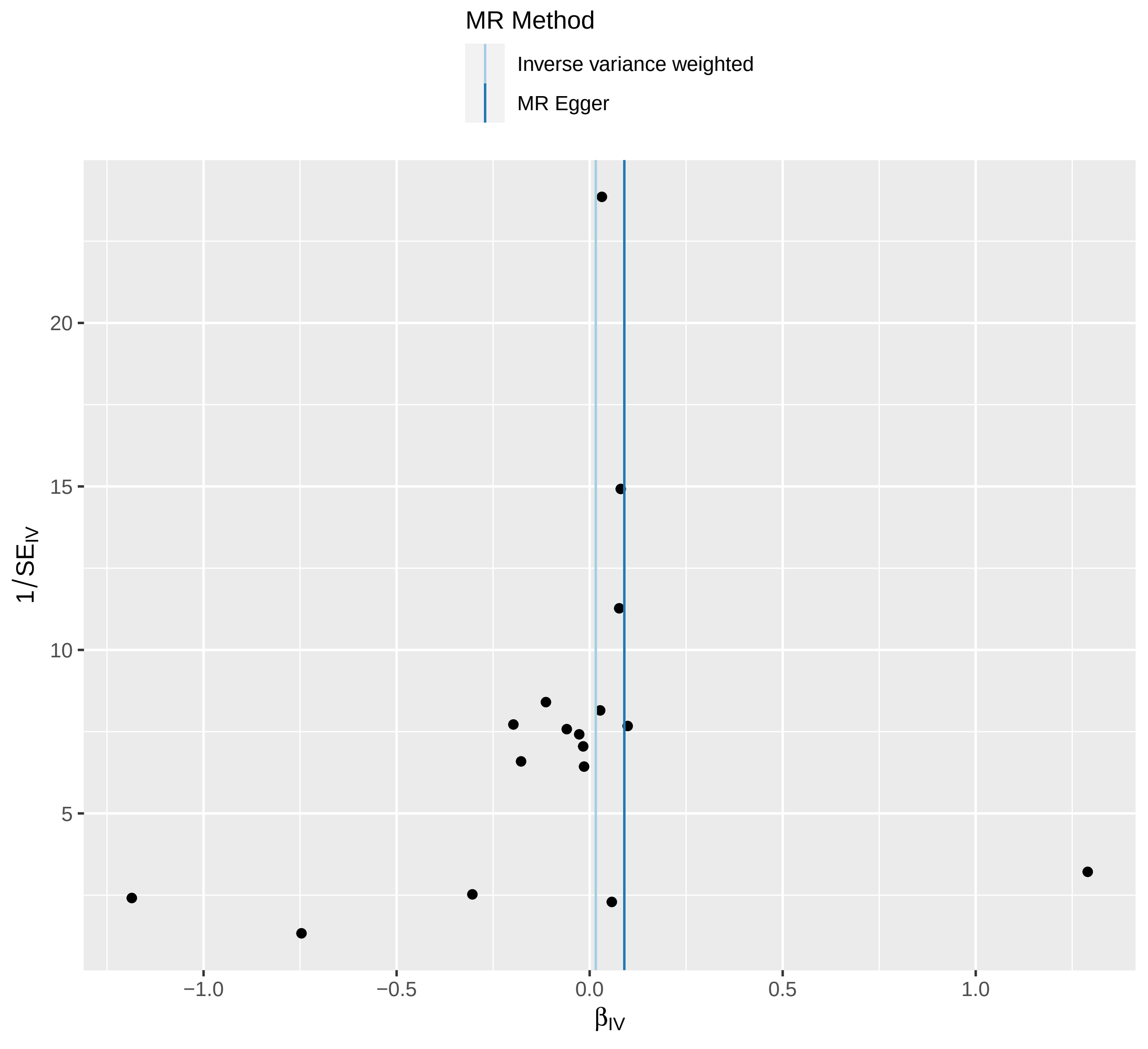
MR Method
Inverse variance weighted
MR Egger
1/SE$_{IV}$
20
15
10
5
−1.0
−0.5
0.0
0.5
1.0
β$_{IV}$

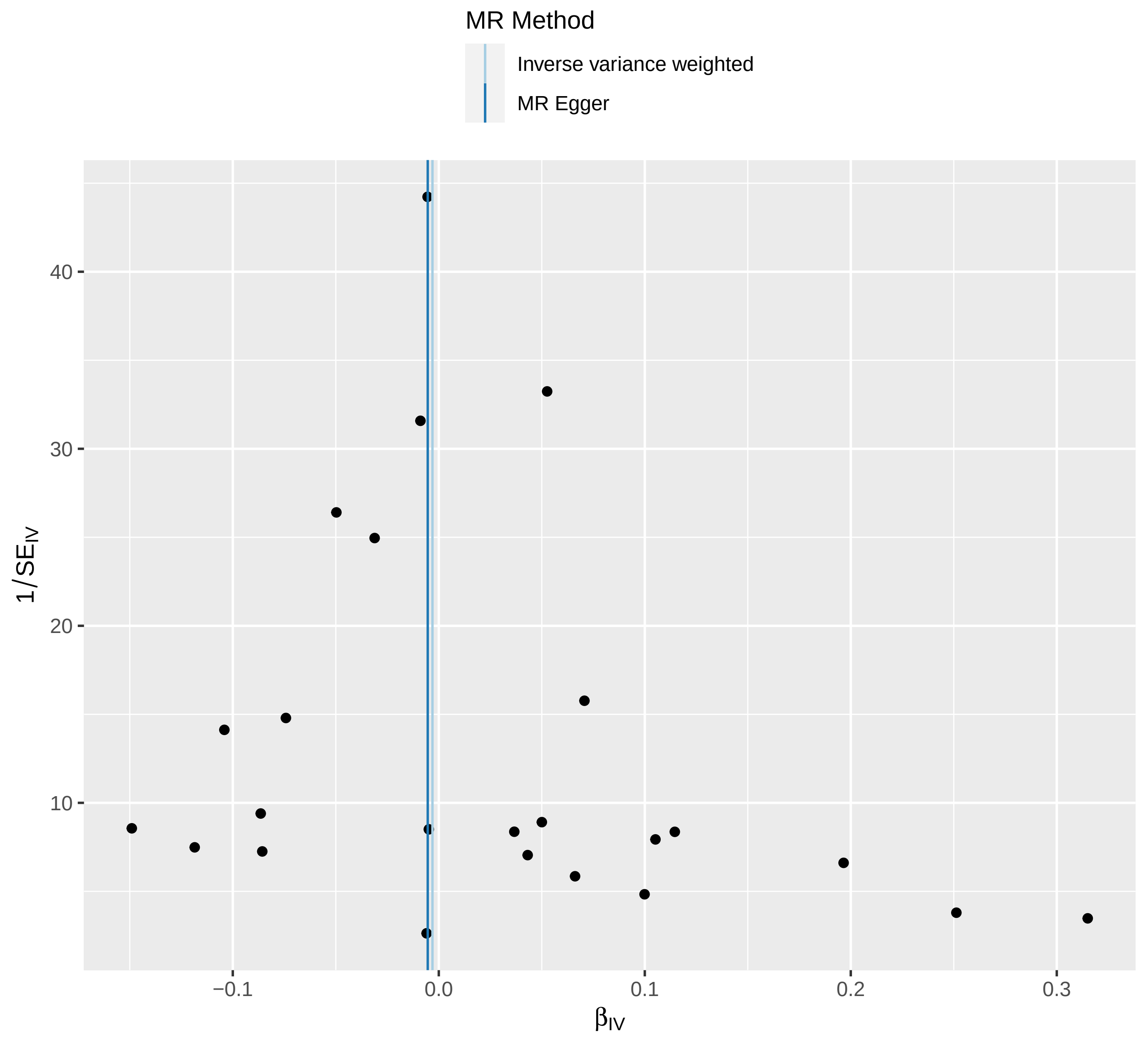

MR Method
Inverse variance weighted
MR Egger
1/SE_IV
β_IV
40
30
20
10
−0.1
0.0
0.1
0.2
0.3

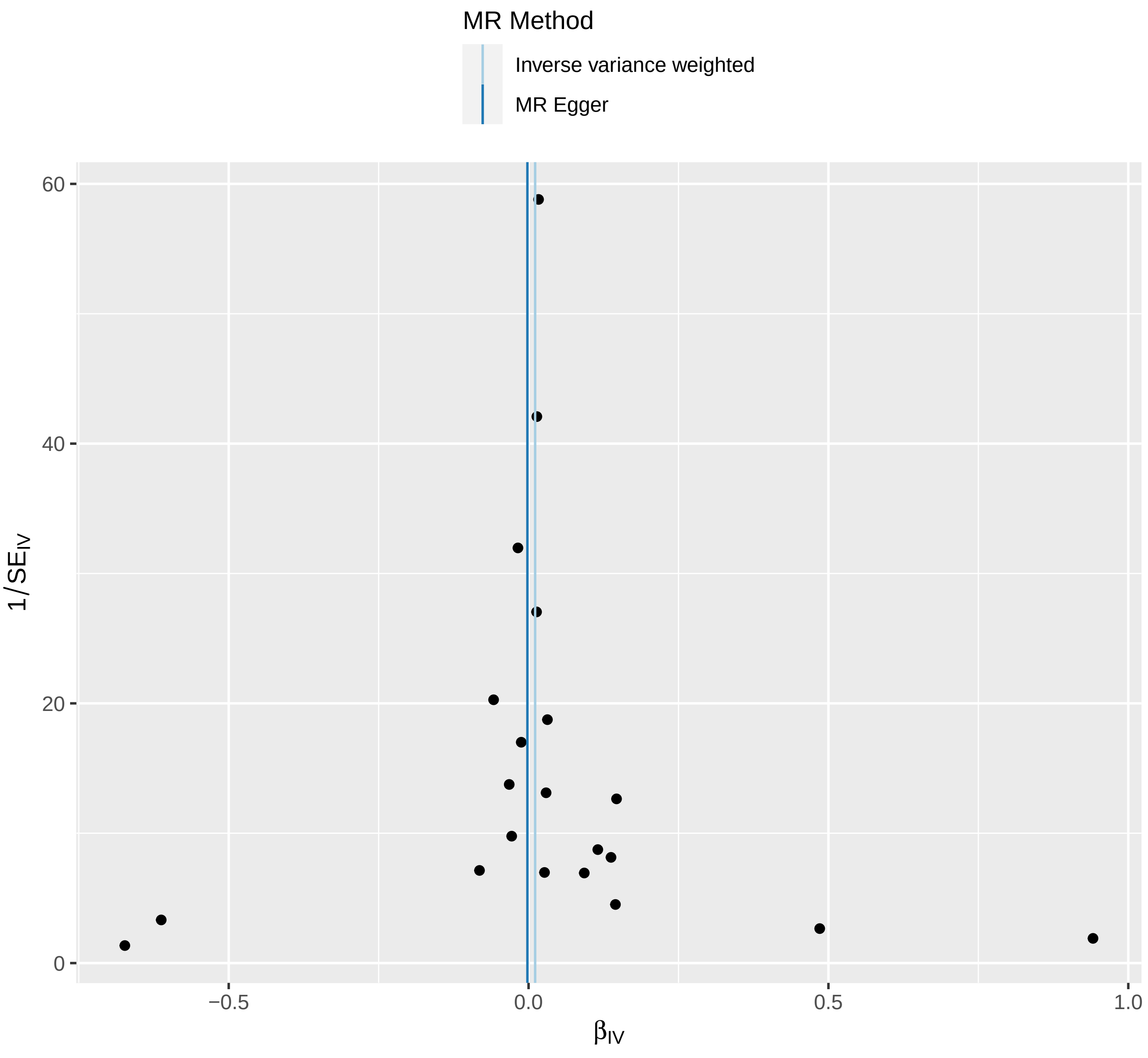

MR Method
Inverse variance weighted
MR Egger
60
40
20
0
1/SE_IV
−0.5
0.0
0.5
1.0
β_IV

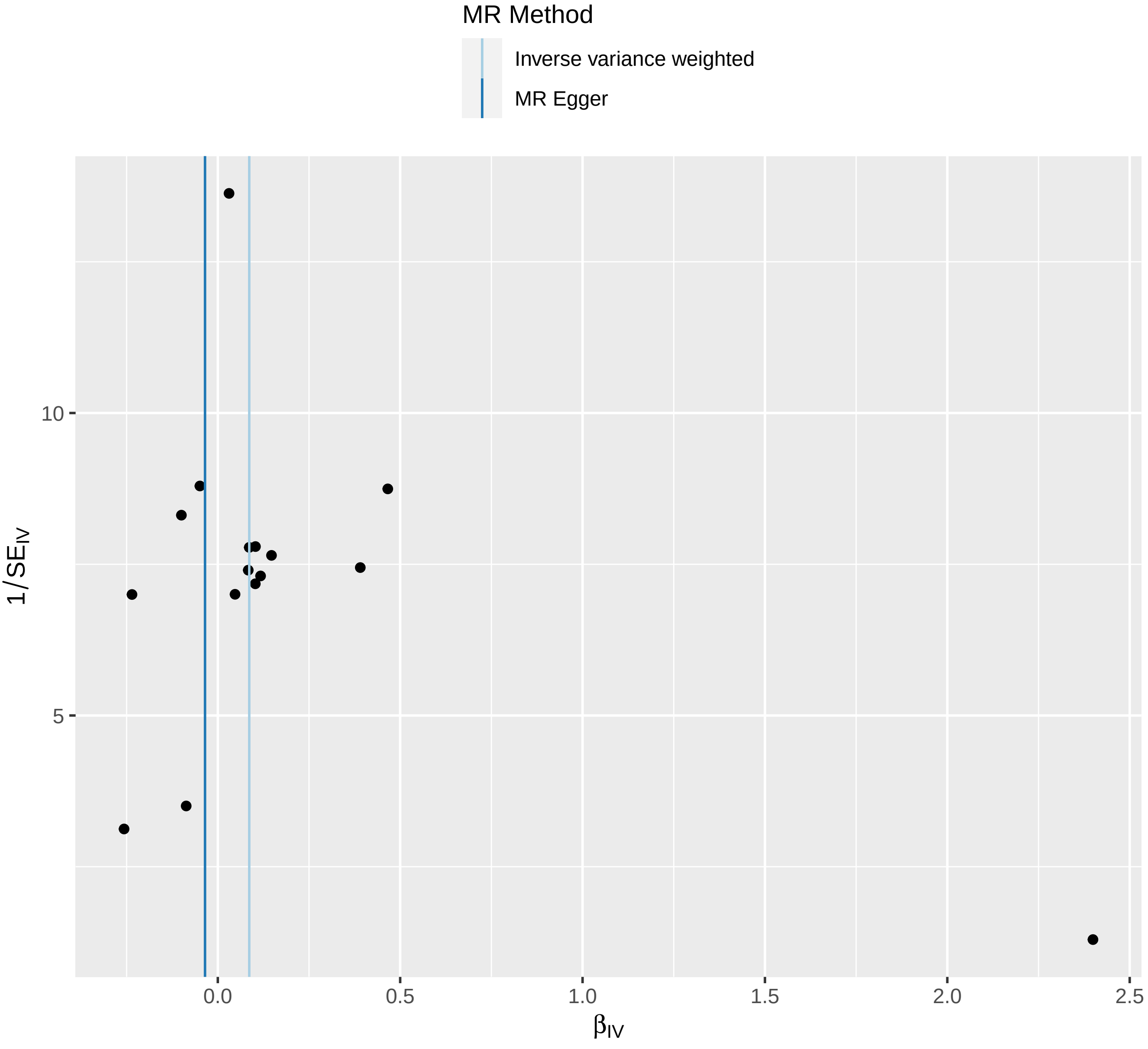

MR Method
Inverse variance weighted
MR Egger
10
5
1/SE$_{IV}$
0.0
0.5
1.0
1.5
2.0
2.5
β$_{IV}$

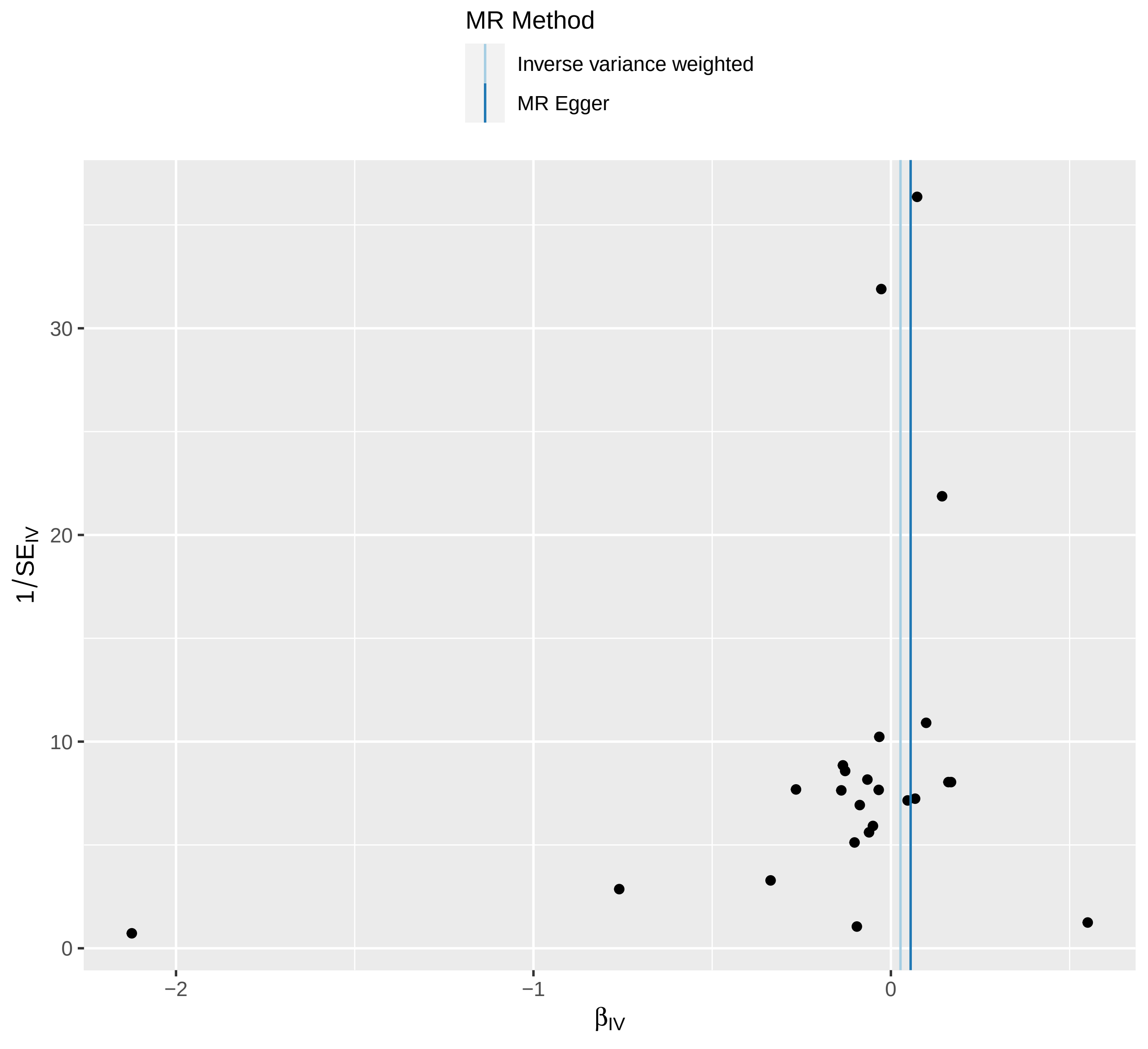

MR Method
Inverse variance weighted
MR Egger
30
20
10
0
1/SE_IV
−2
−1
0
β_IV

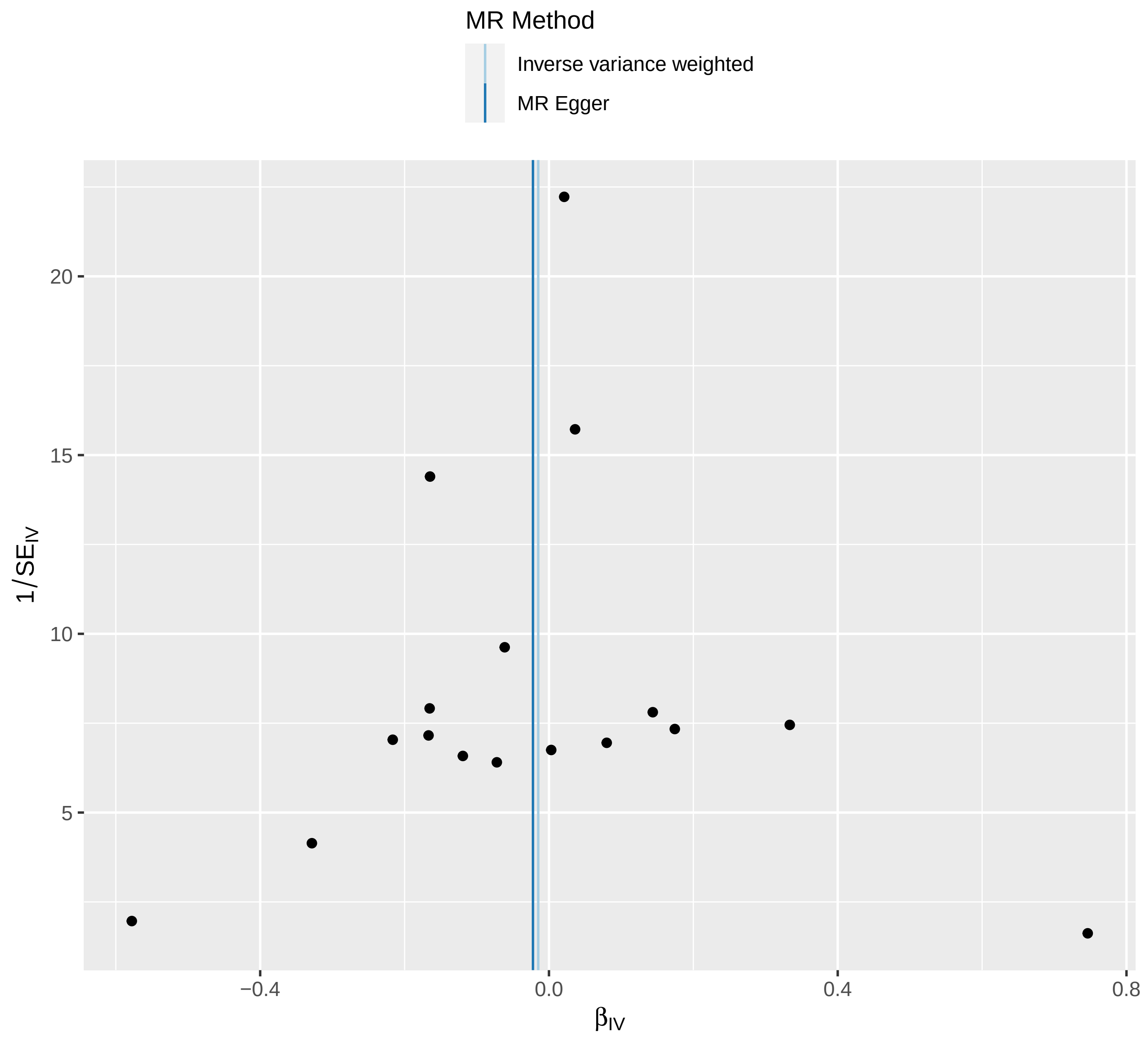

MR Method
Inverse variance weighted
MR Egger
20
15
10
5
1/SE_IV
−0.4
0.0
0.4
0.8
β_IV

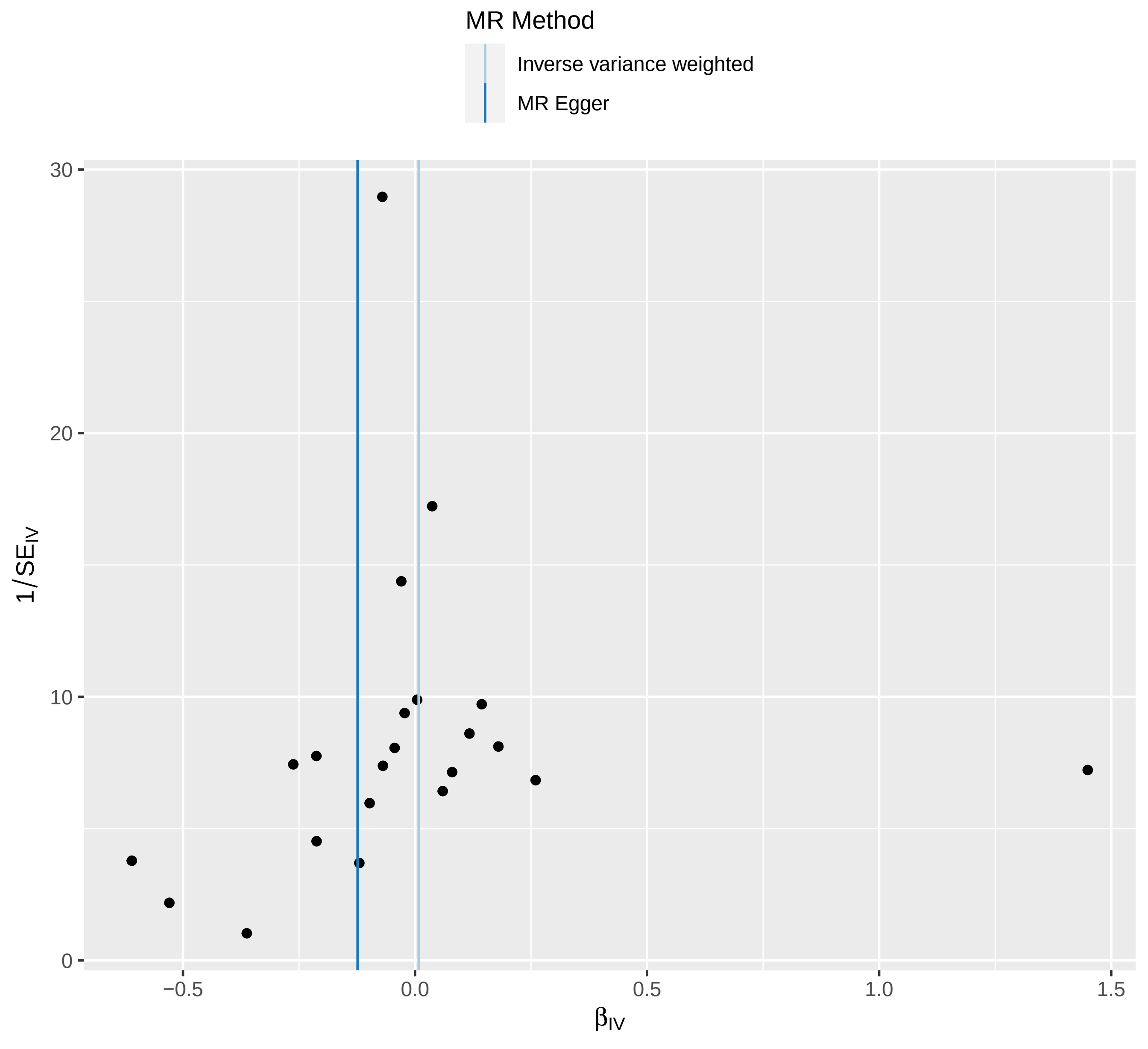
MR Method
Inverse variance weighted
MR Egger
30
20
10
0
1/SE_IV
−0.5
0.0
0.5
1.0
1.5
β_IV

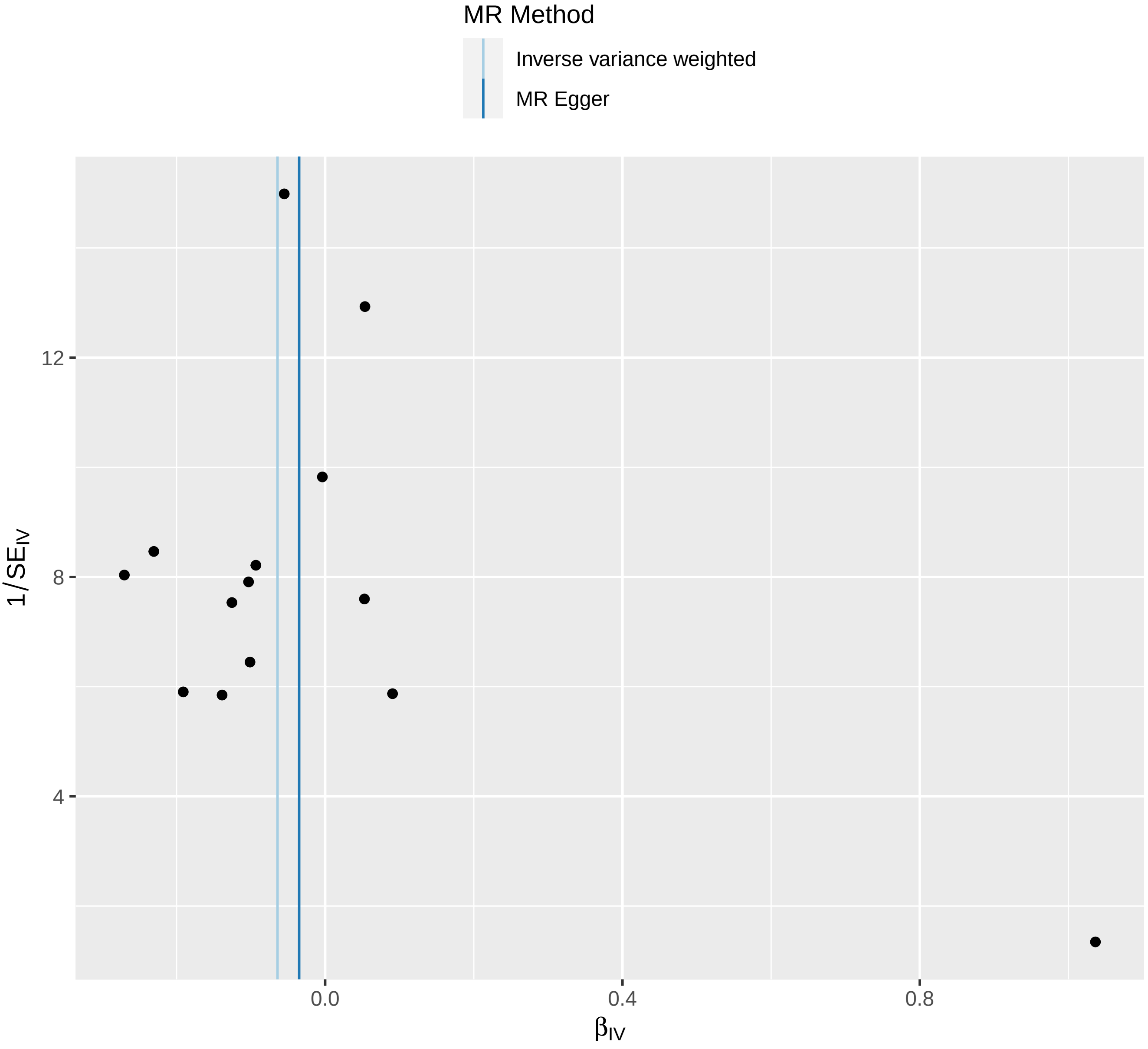
MR Method
Inverse variance weighted
MR Egger
12
8
4
$1/SE_{IV}$
0.0
0.4
0.8
β_{IV}

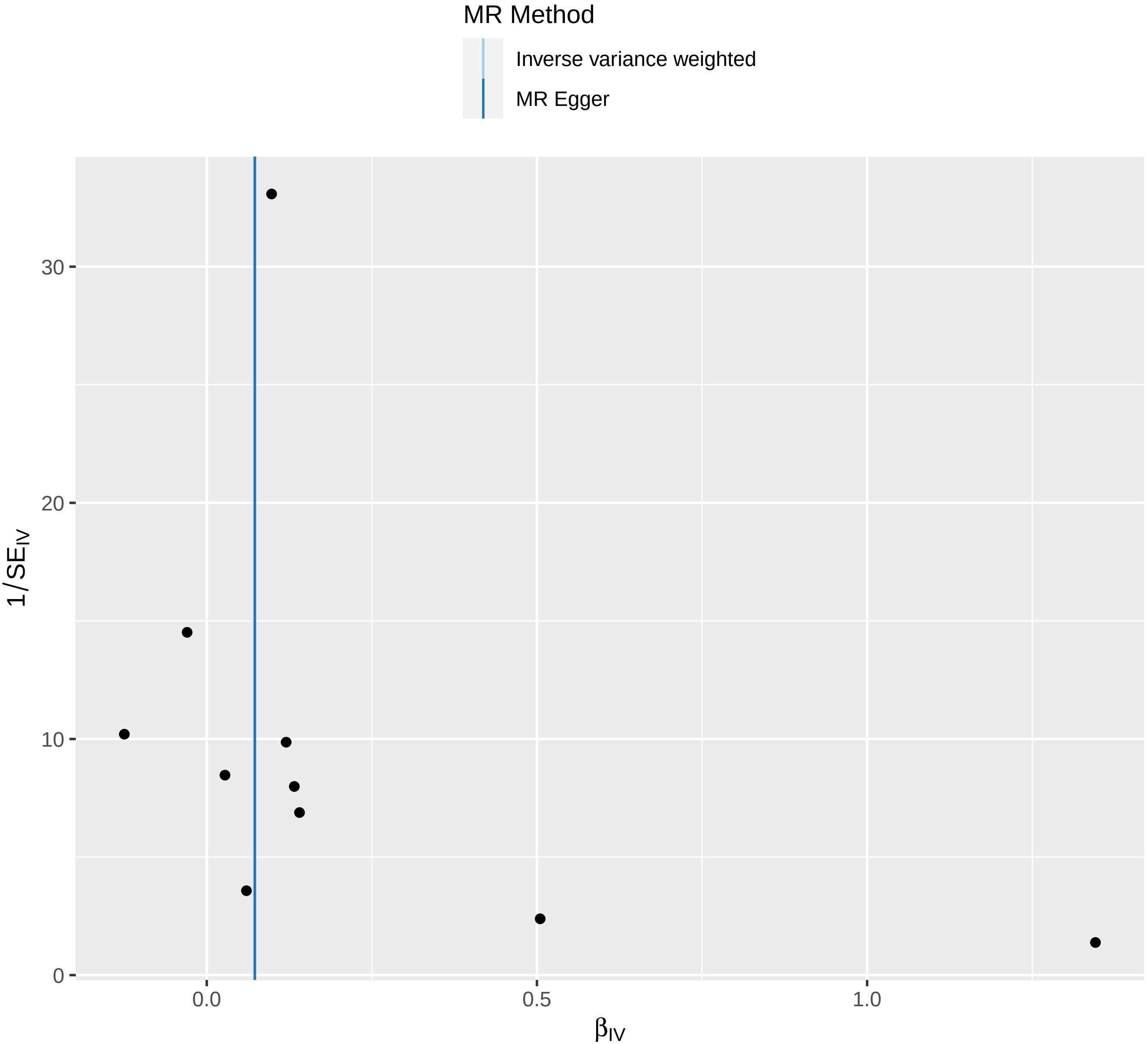
MR Method
Inverse variance weighted
MR Egger
30
20
10
0
0.0
0.5
1.0
$1/SE_{IV}$
β_{IV}

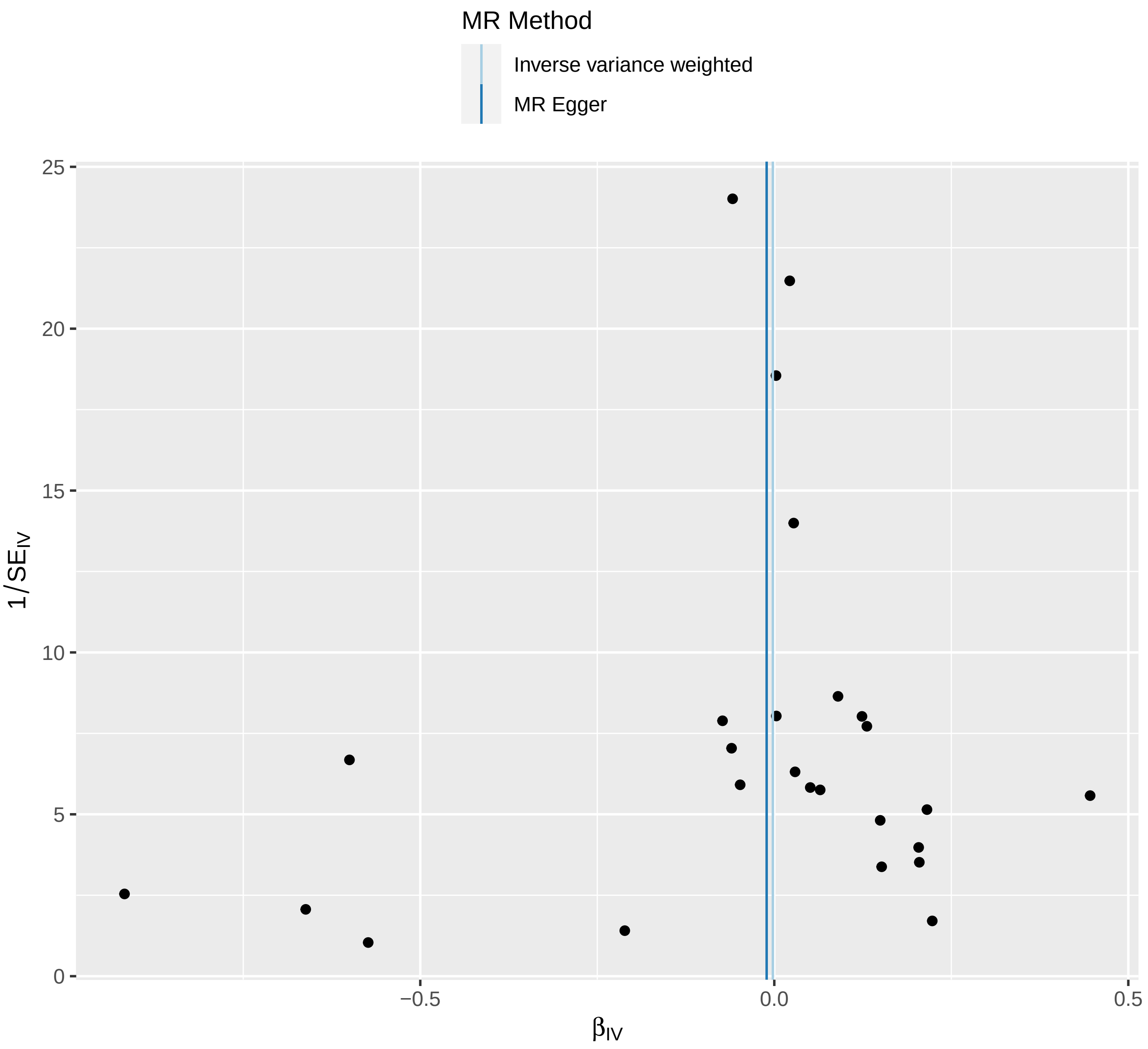

MR Method
Inverse variance weighted
MR Egger
25
20
15
10
5
0
$1/SE_{IV}$
−0.5
0.0
0.5
β_{IV}

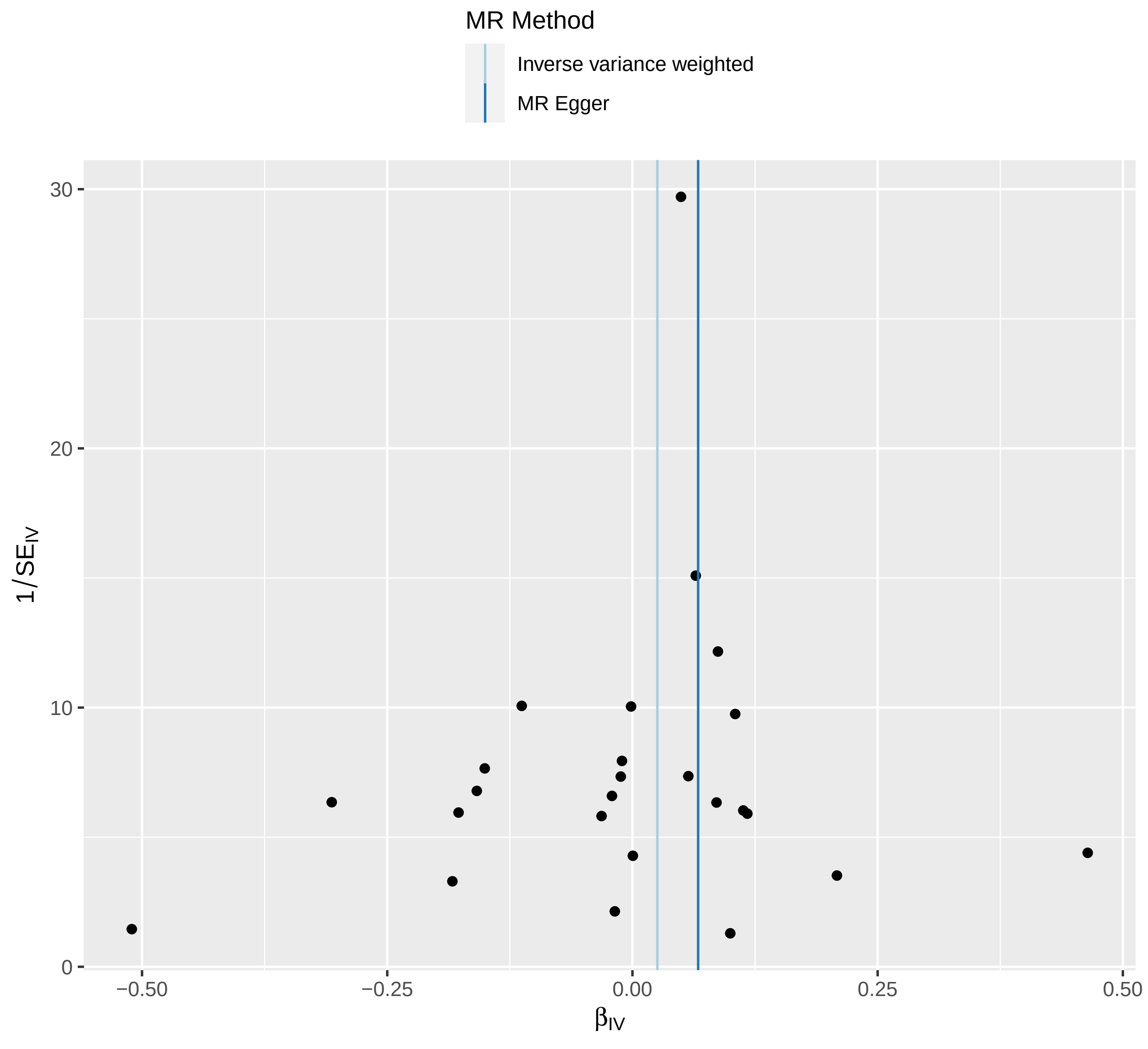
MR Method
Inverse variance weighted
MR Egger
30
20
10
0
1/SE$_{IV}$
−0.50
−0.25
0.00
0.25
0.50
β$_{IV}$

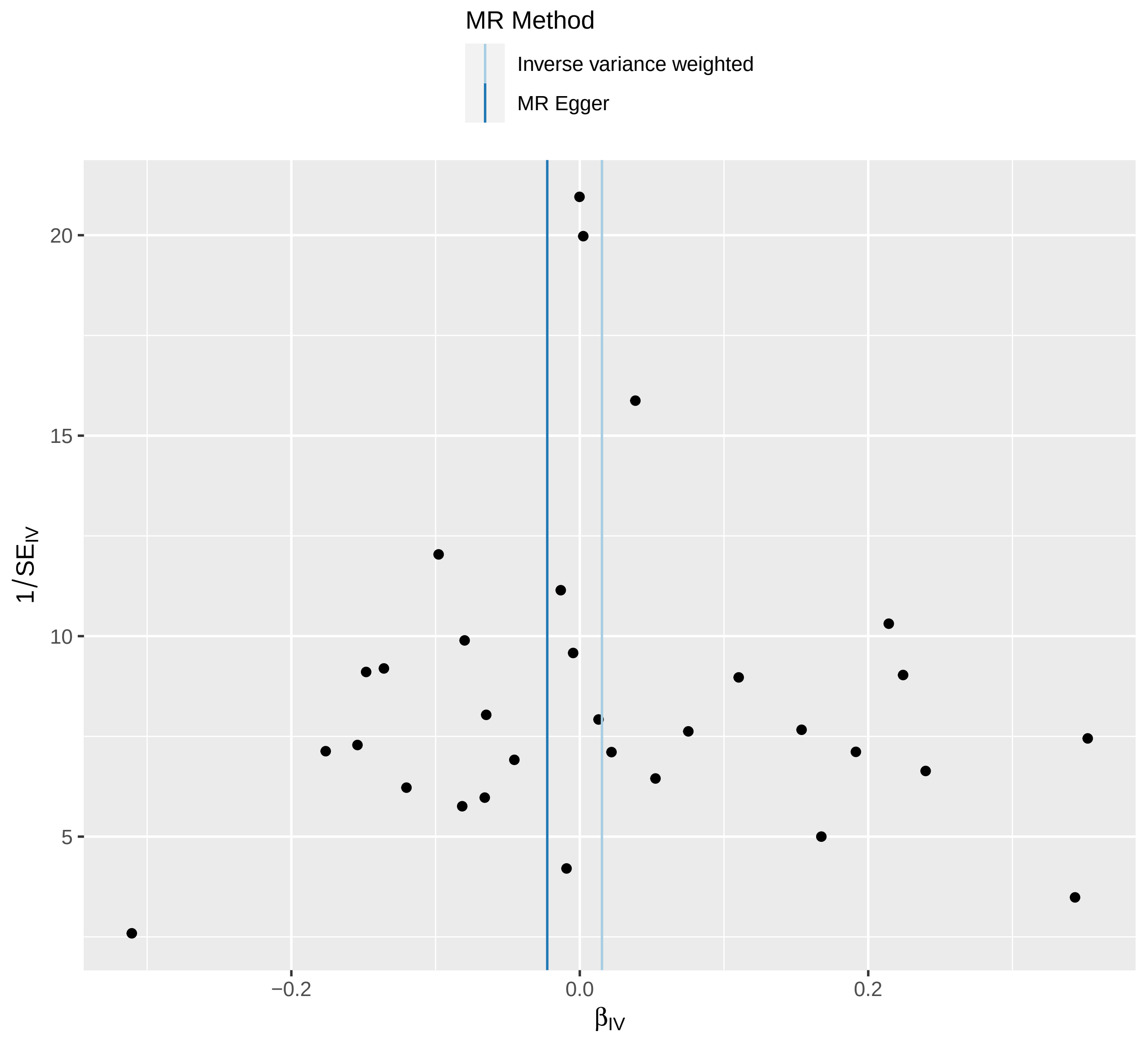
MR Method
Inverse variance weighted
MR Egger
1/SE_IV
20
15
10
5
−0.2
0.0
0.2
β_IV

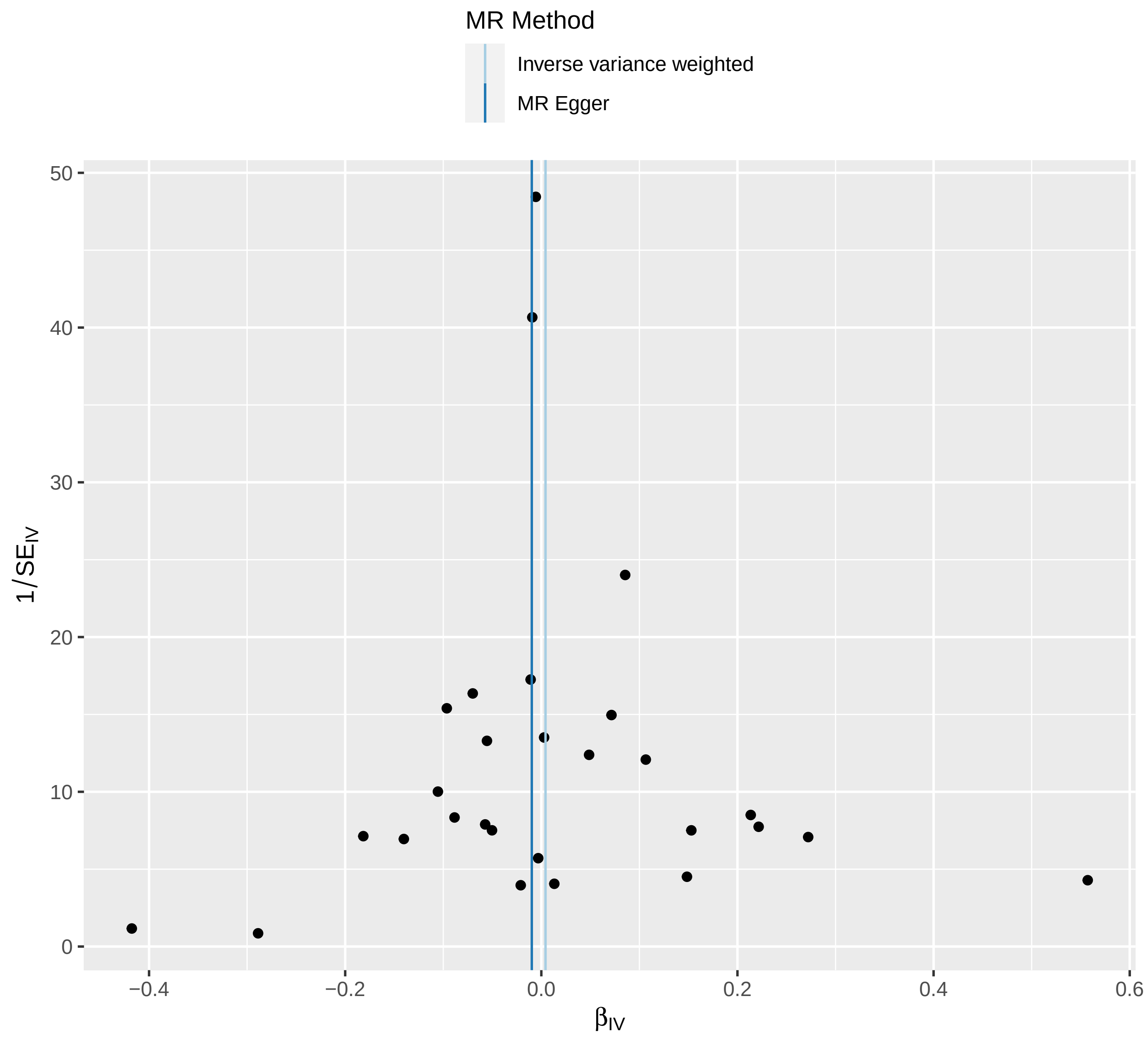
MR Method
Inverse variance weighted
MR Egger
1/SE_IV
β_IV
50
40
30
20
10
0
−0.4
−0.2
0.0
0.2
0.4
0.6

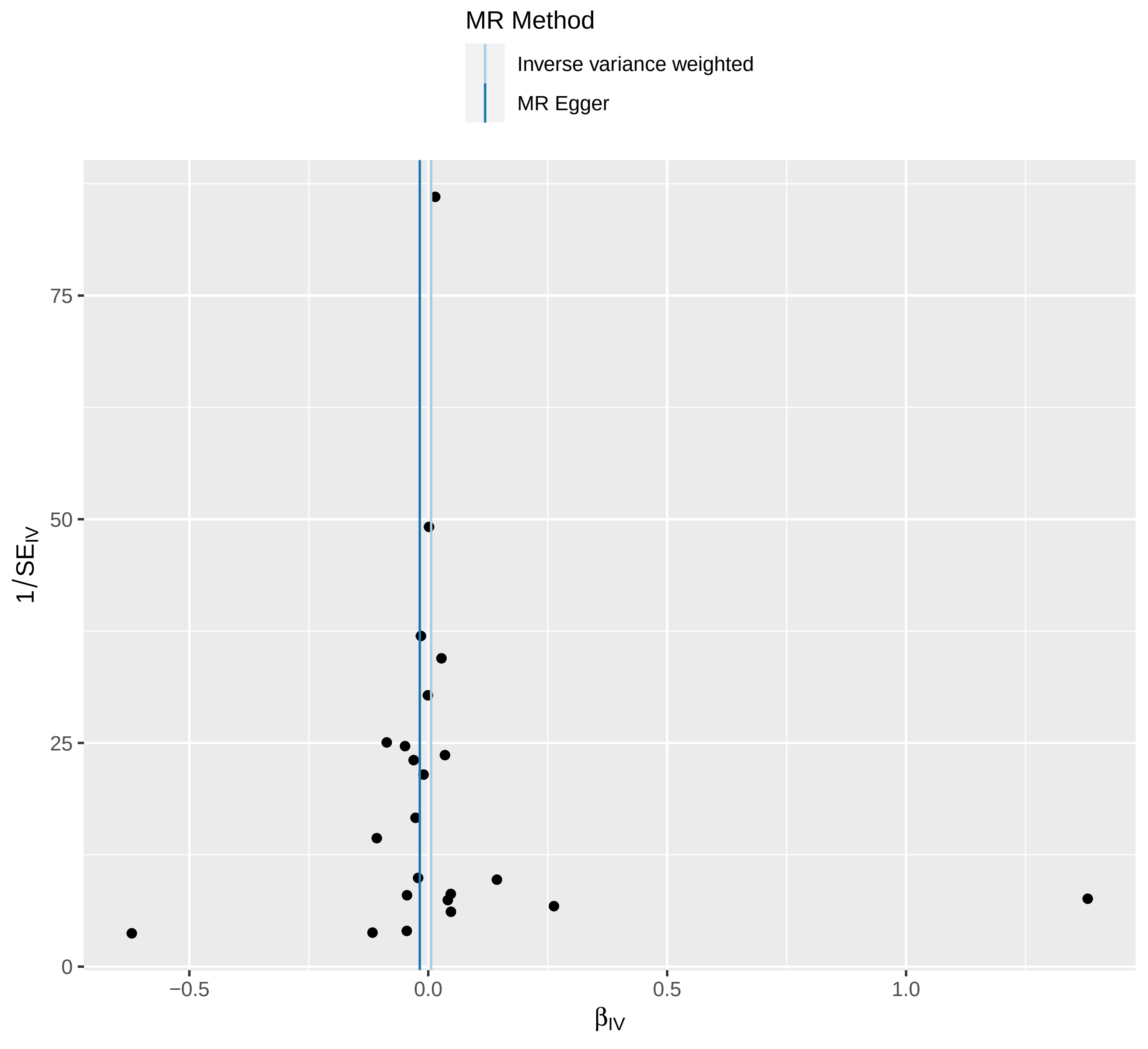

MR Method
Inverse variance weighted
MR Egger
75
50
25
0
1/SE$_{IV}$
−0.5
0.0
0.5
1.0
β$_{IV}$

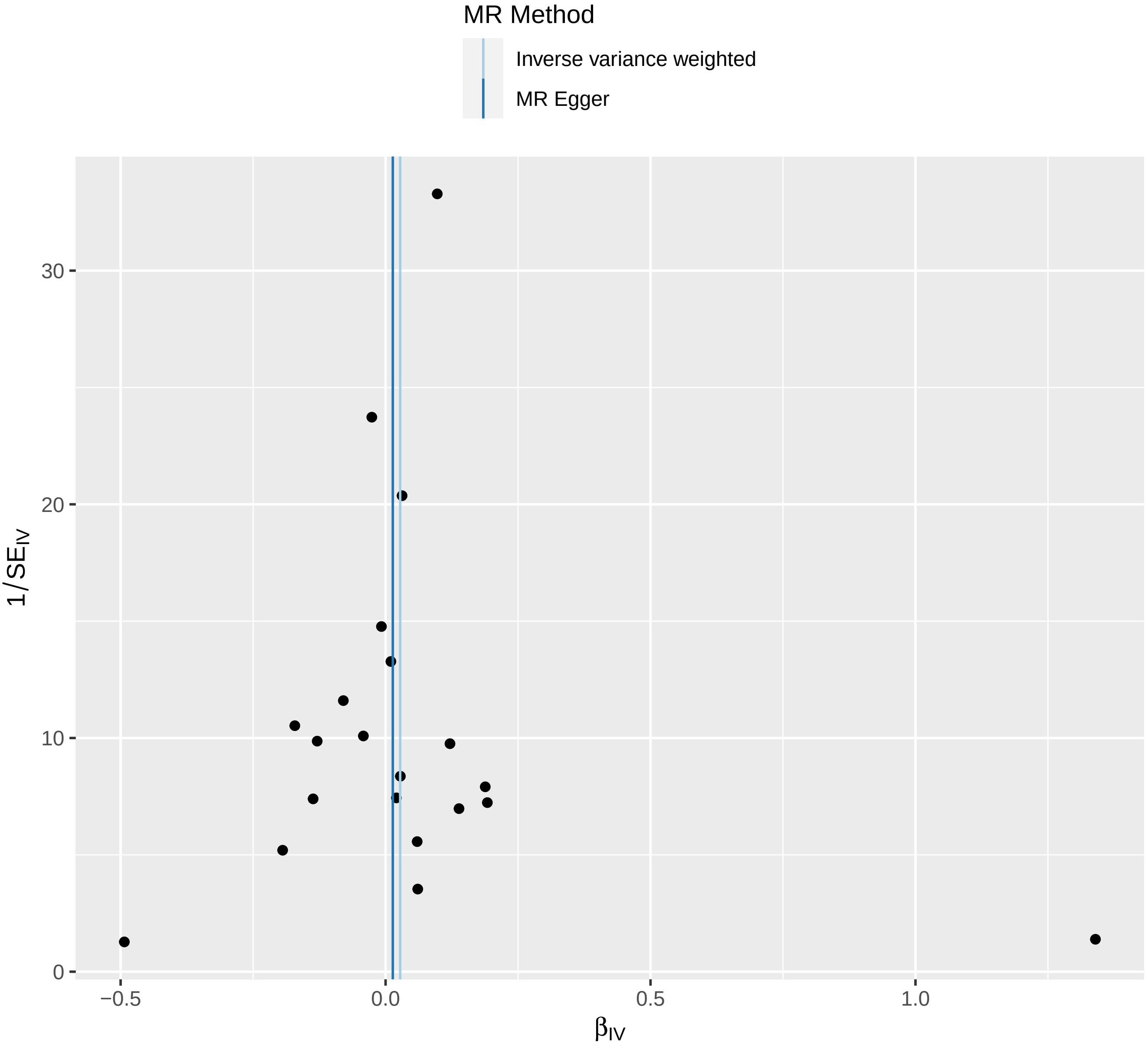

MR Method
Inverse variance weighted
MR Egger
30
20
10
0
1/SE$_{IV}$
−0.5
0.0
0.5
1.0
β$_{IV}$

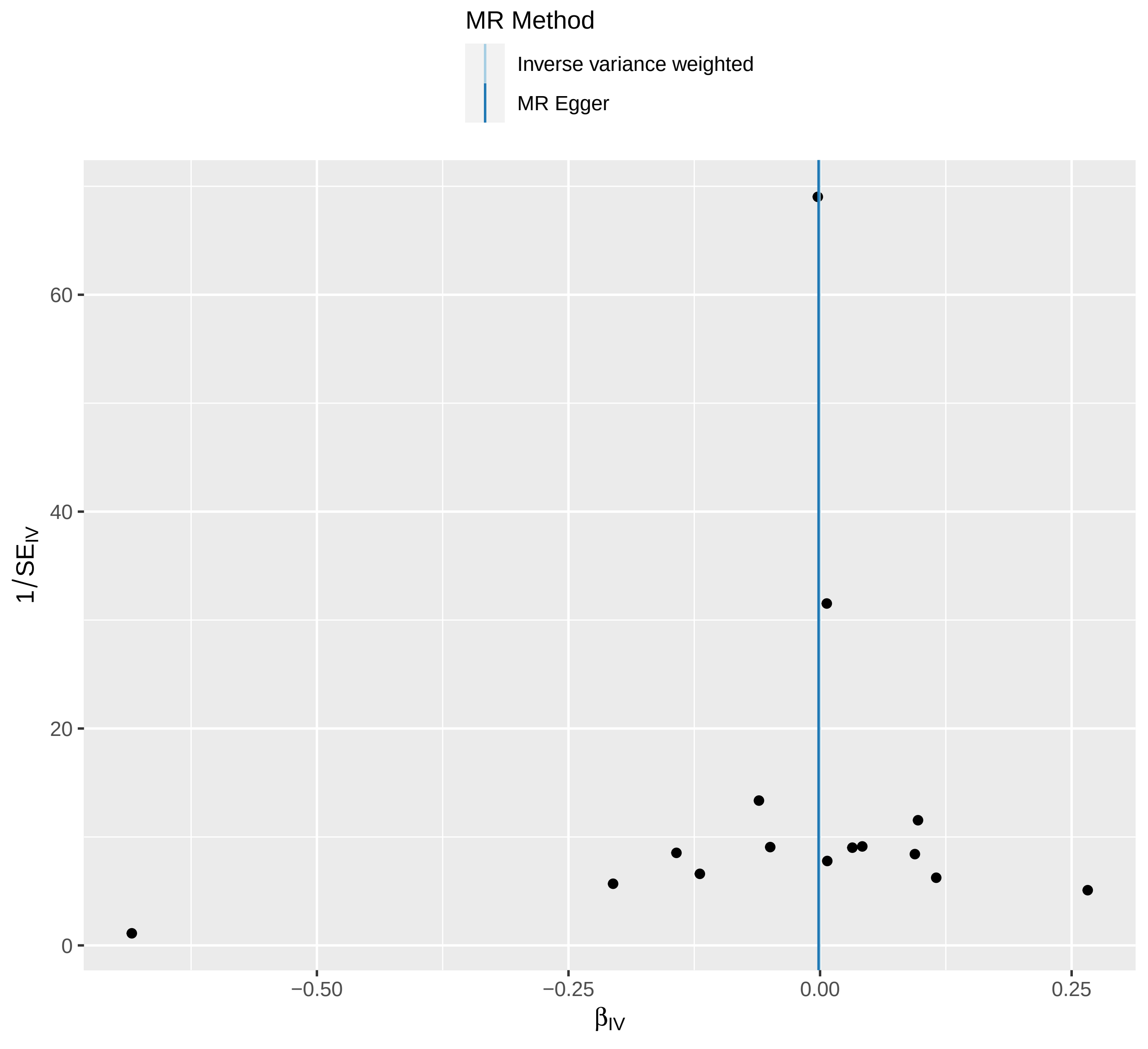
MR Method
Inverse variance weighted
MR Egger
60
40
20
0
1/SE_IV
−0.50
−0.25
0.00
0.25
β_IV

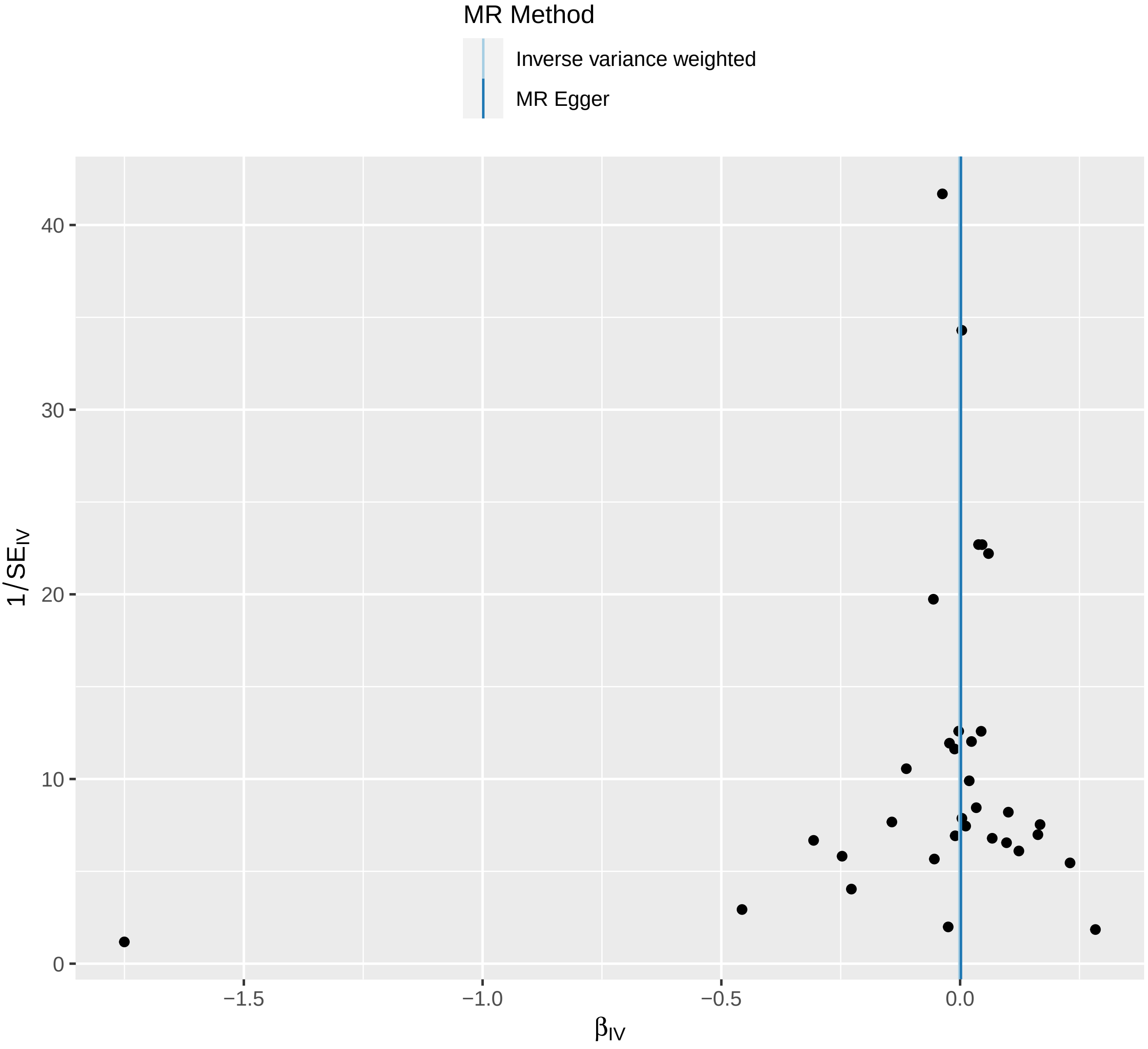

MR Method
Inverse variance weighted
MR Egger
40
30
20
10
0
1/SE$_{IV}$
−1.5
−1.0
−0.5
0.0
β$_{IV}$

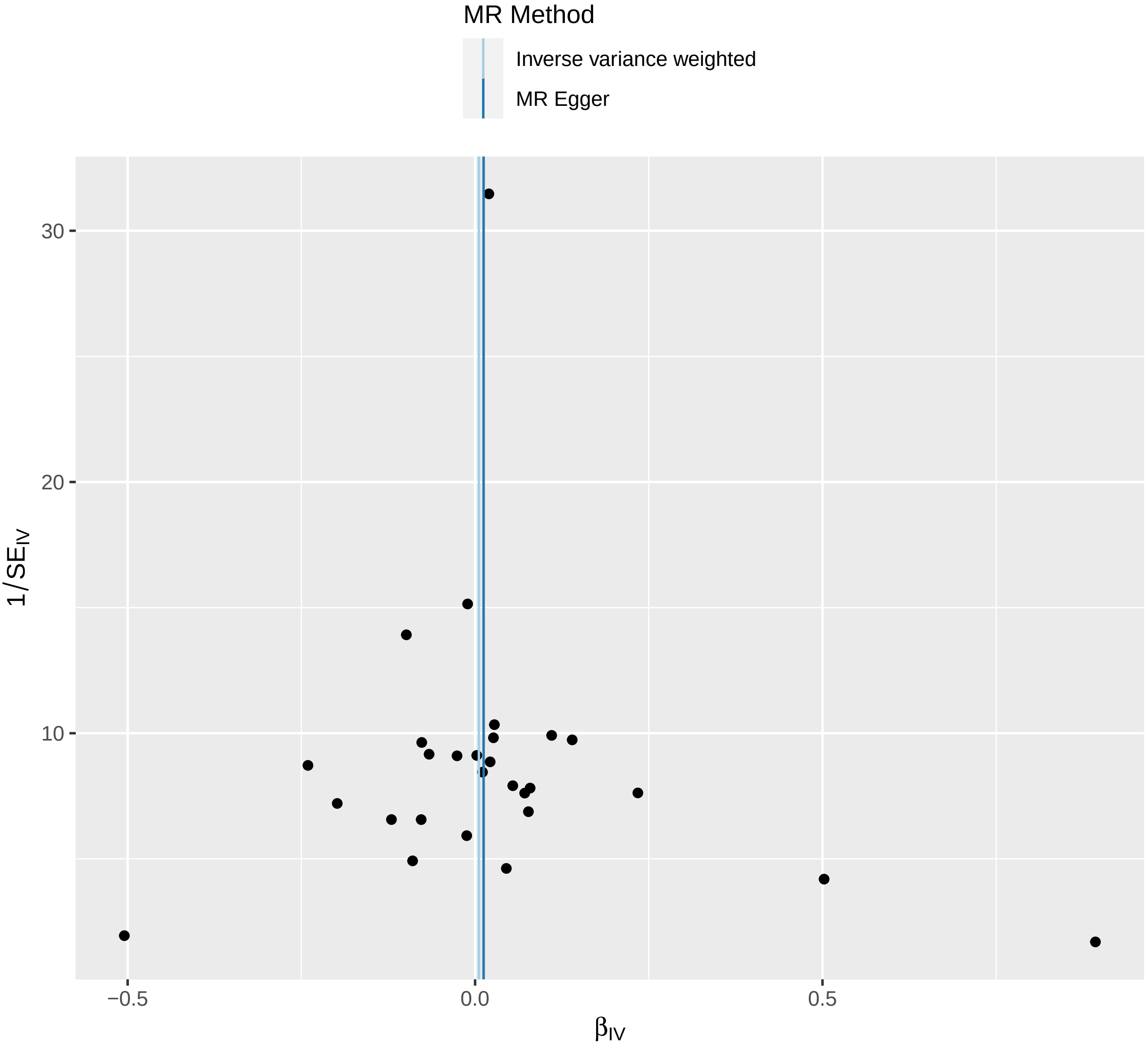

MR Method
Inverse variance weighted
MR Egger
30
20
10
1/SE_IV
−0.5
0.0
0.5
β_IV

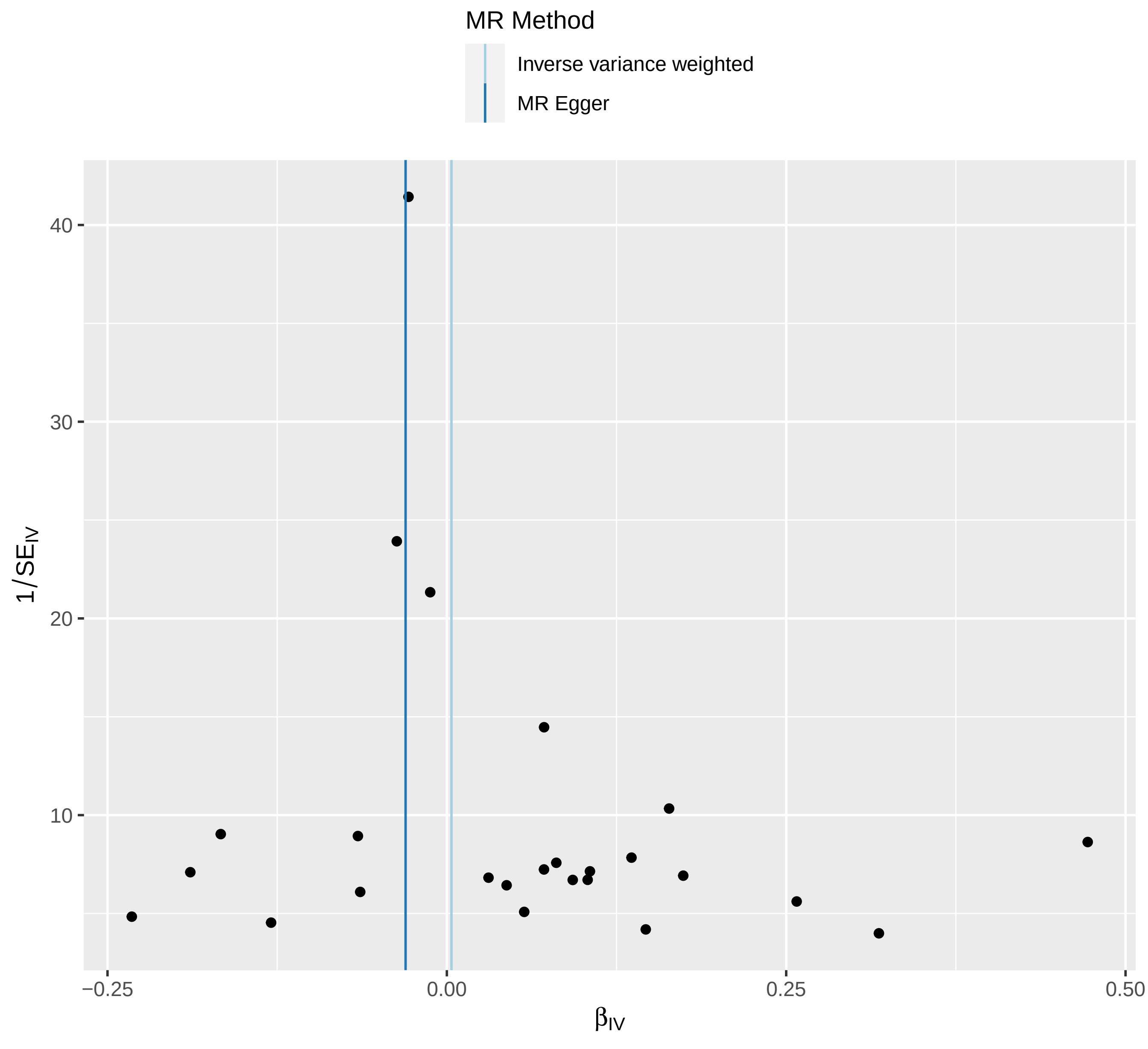

MR Method
Inverse variance weighted
MR Egger
40
30
20
10
$1/SE_{IV}$
−0.25
0.00
0.25
0.50
β_{IV}

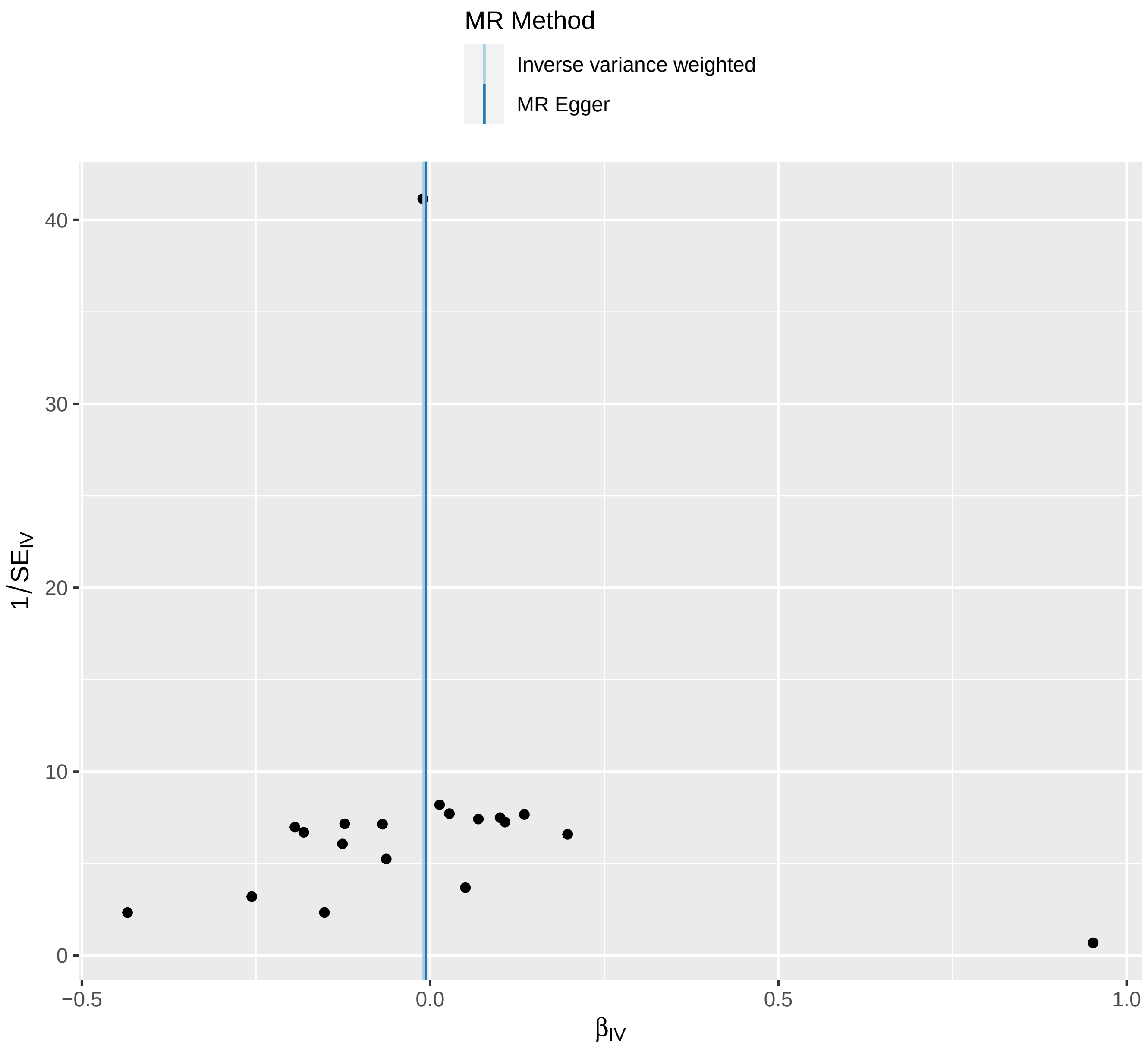
MR Method
Inverse variance weighted
MR Egger
1/SE_IV
β_IV
40
30
20
10
0
−0.5
0.0
0.5
1.0

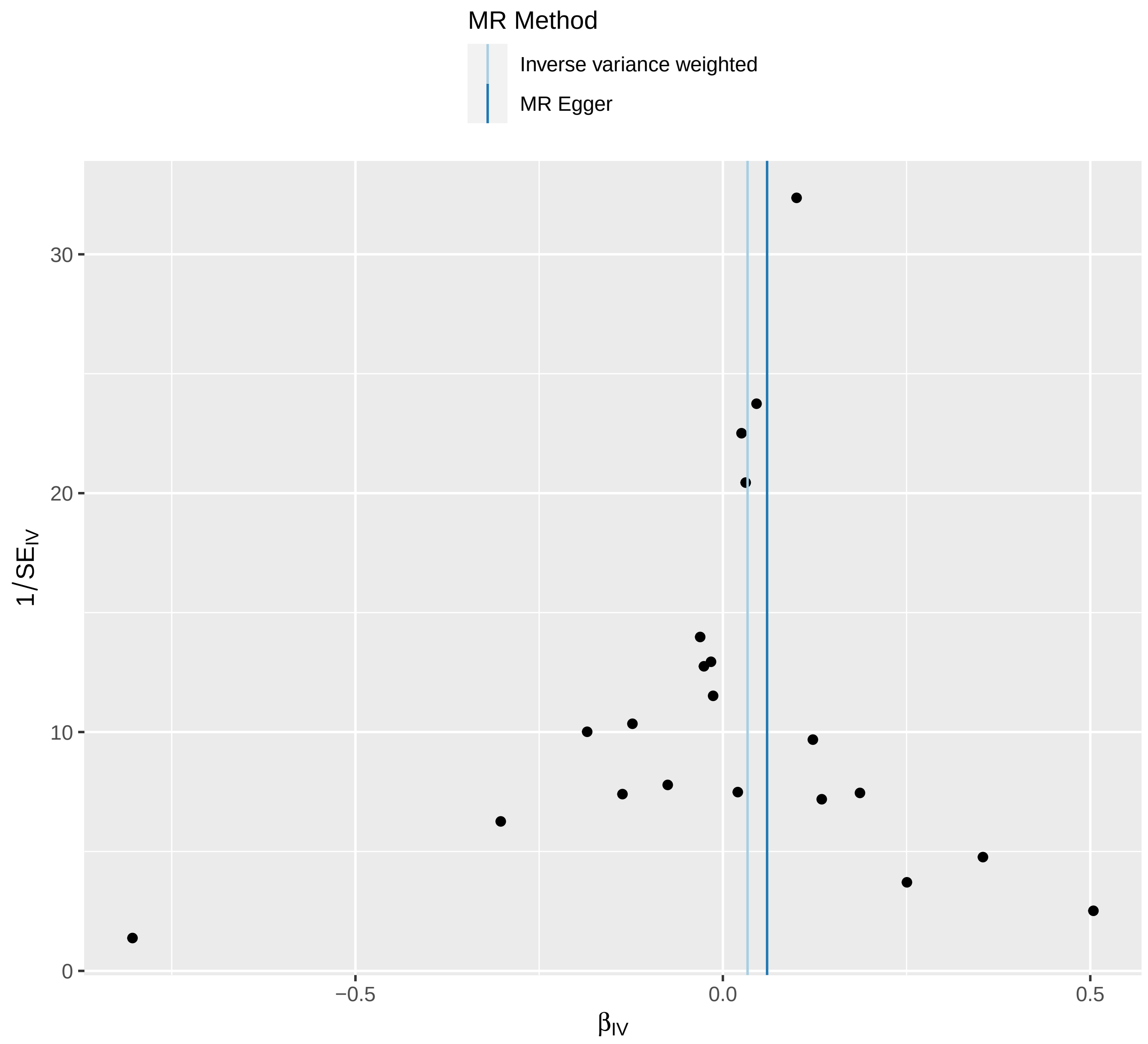

MR Method
Inverse variance weighted
MR Egger
30
20
10
0
−0.5
0.0
0.5
$1/SE_{IV}$
β_{IV}

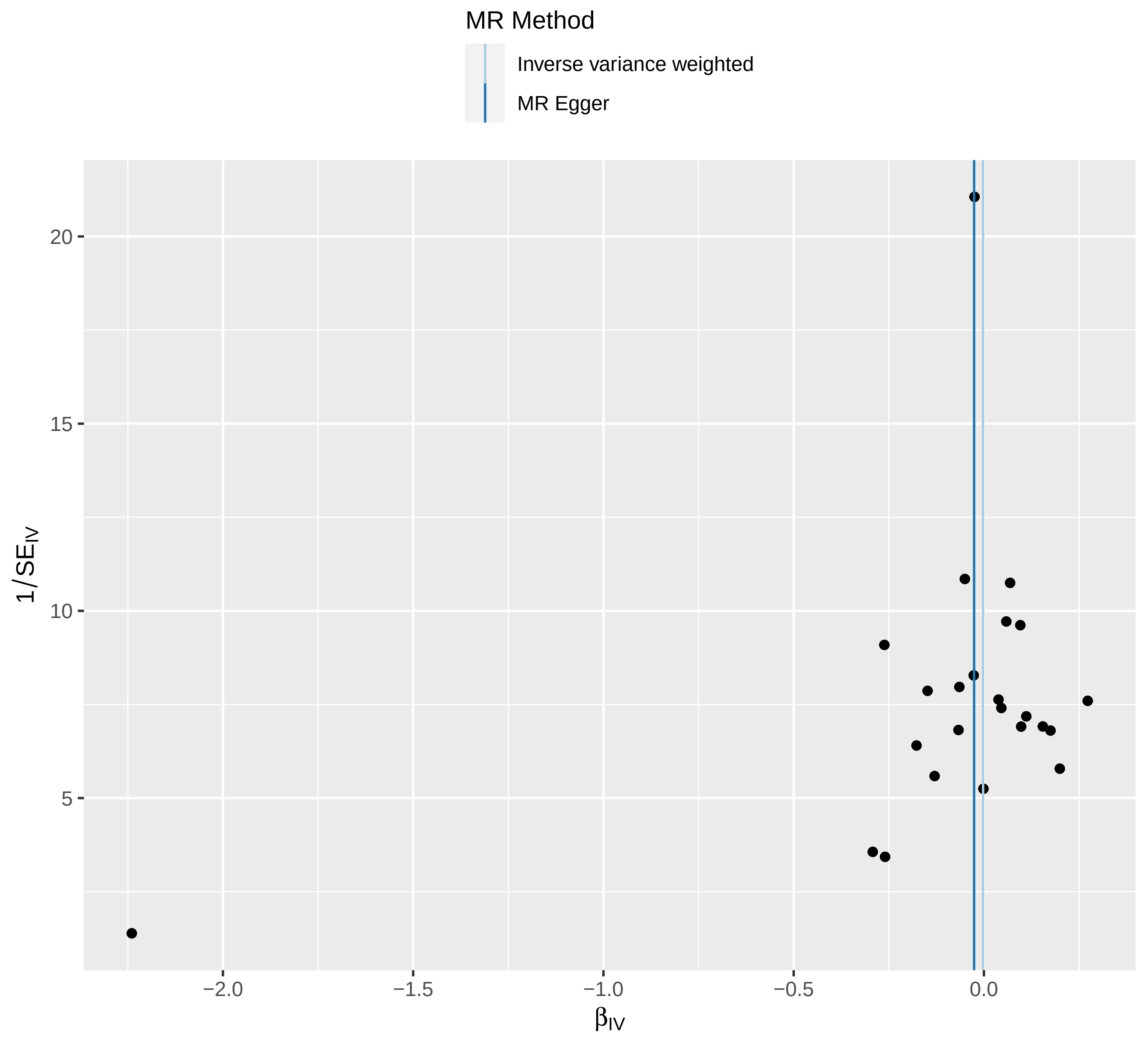
MR Method
Inverse variance weighted
MR Egger
20
15
10
5
1/SE_IV
−2.0
−1.5
−1.0
−0.5
0.0
β_IV

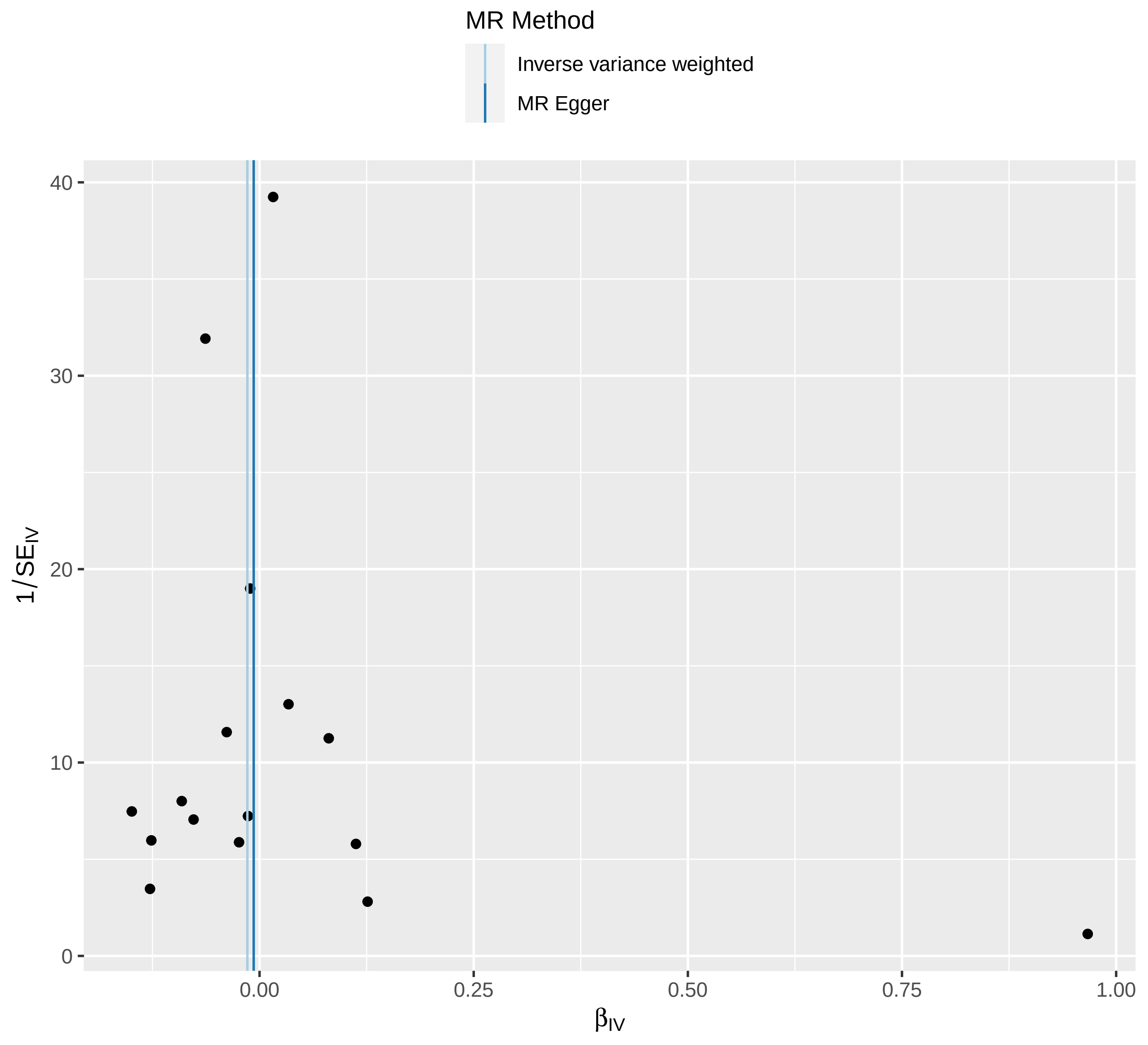

MR Method
Inverse variance weighted
MR Egger
40
30
20
10
0
1/SE$_{IV}$
0.00
0.25
0.50
0.75
1.00
β$_{IV}$

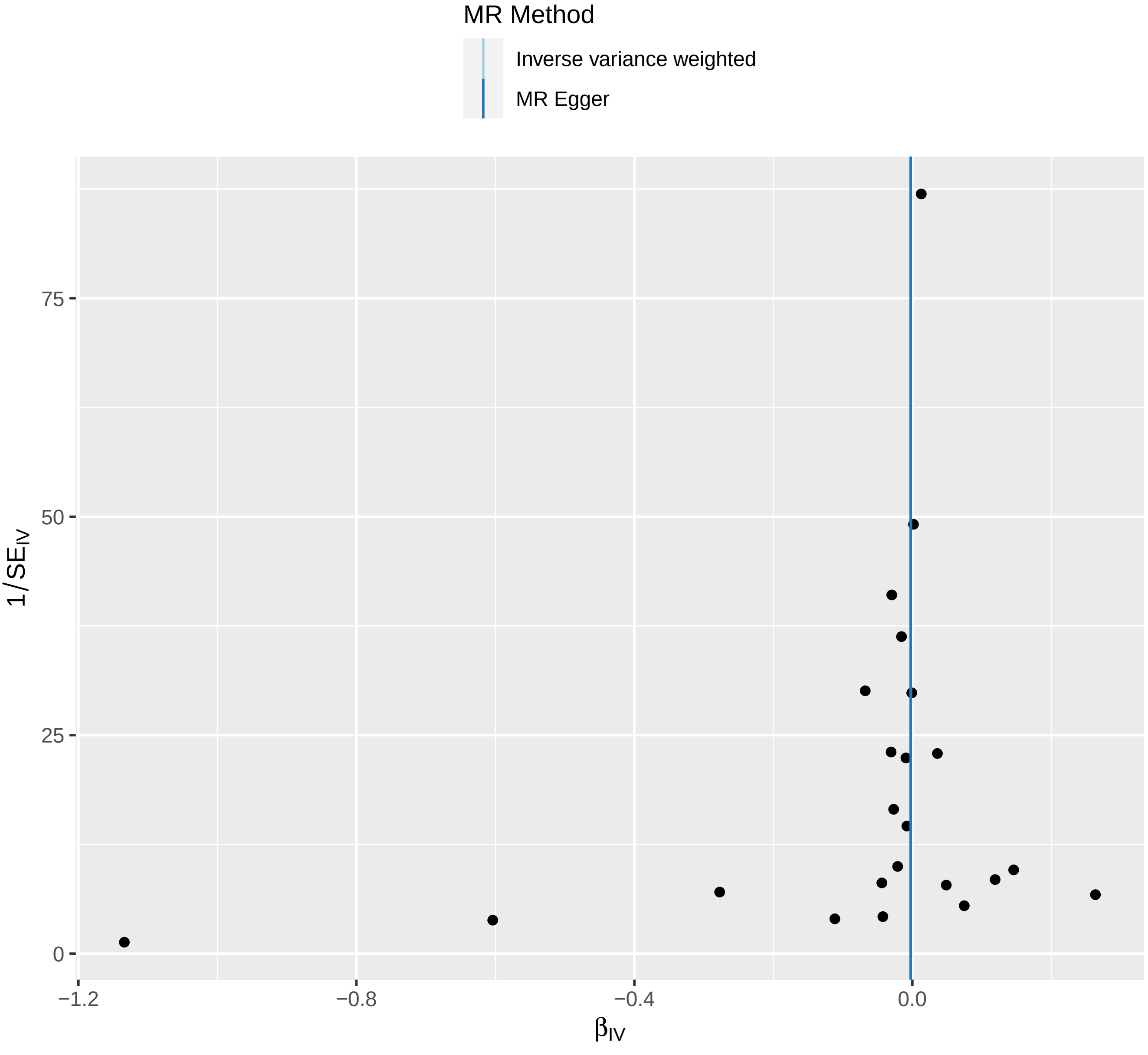

MR Method
Inverse variance weighted
MR Egger
1/SE$_{IV}$
0
25
50
75
−1.2
−0.8
−0.4
0.0
β$_{IV}$

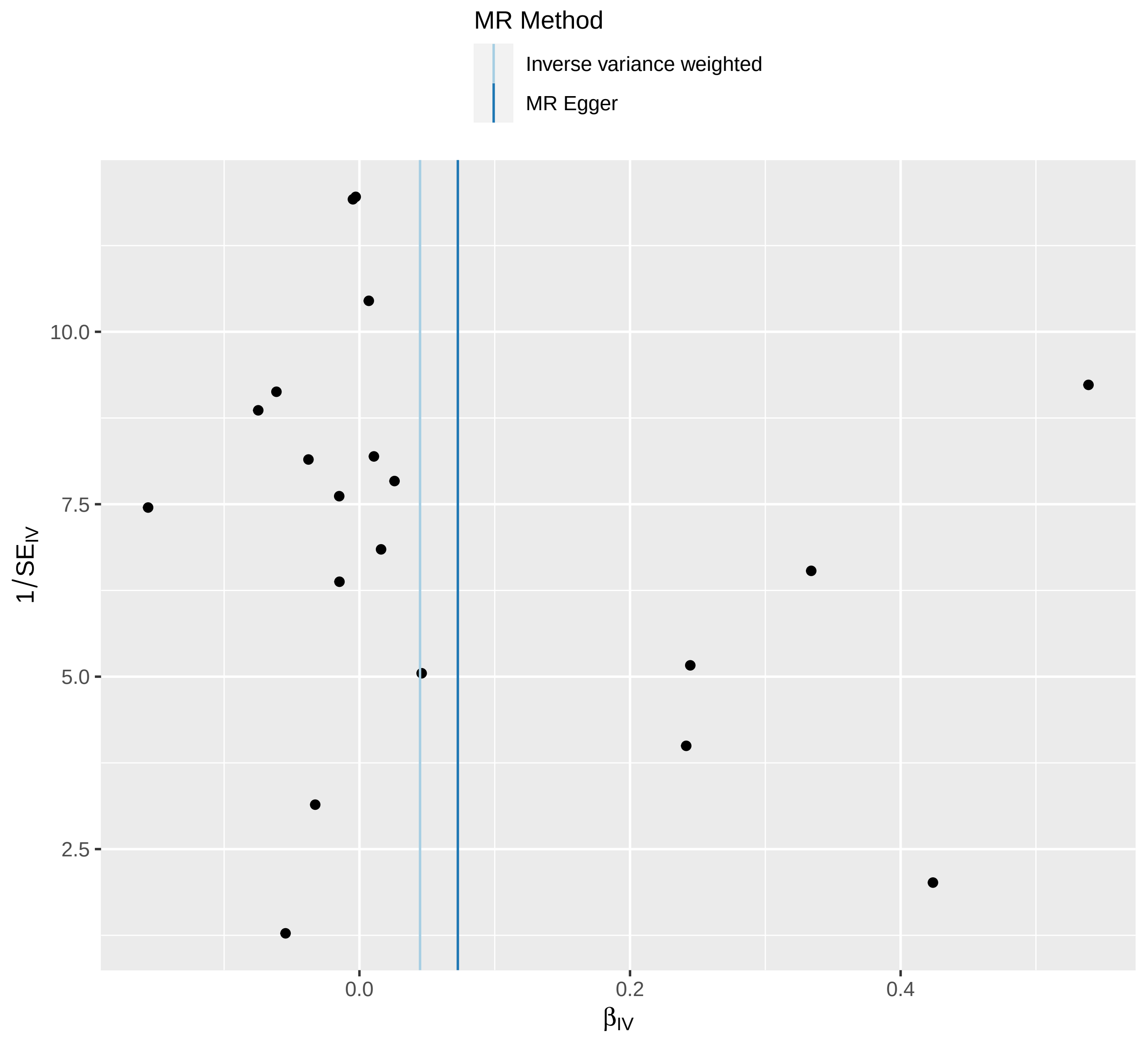
MR Method
Inverse variance weighted
MR Egger
10.0
7.5
5.0
2.5
$1/SE_{IV}$
0.0
0.2
0.4
β_{IV}

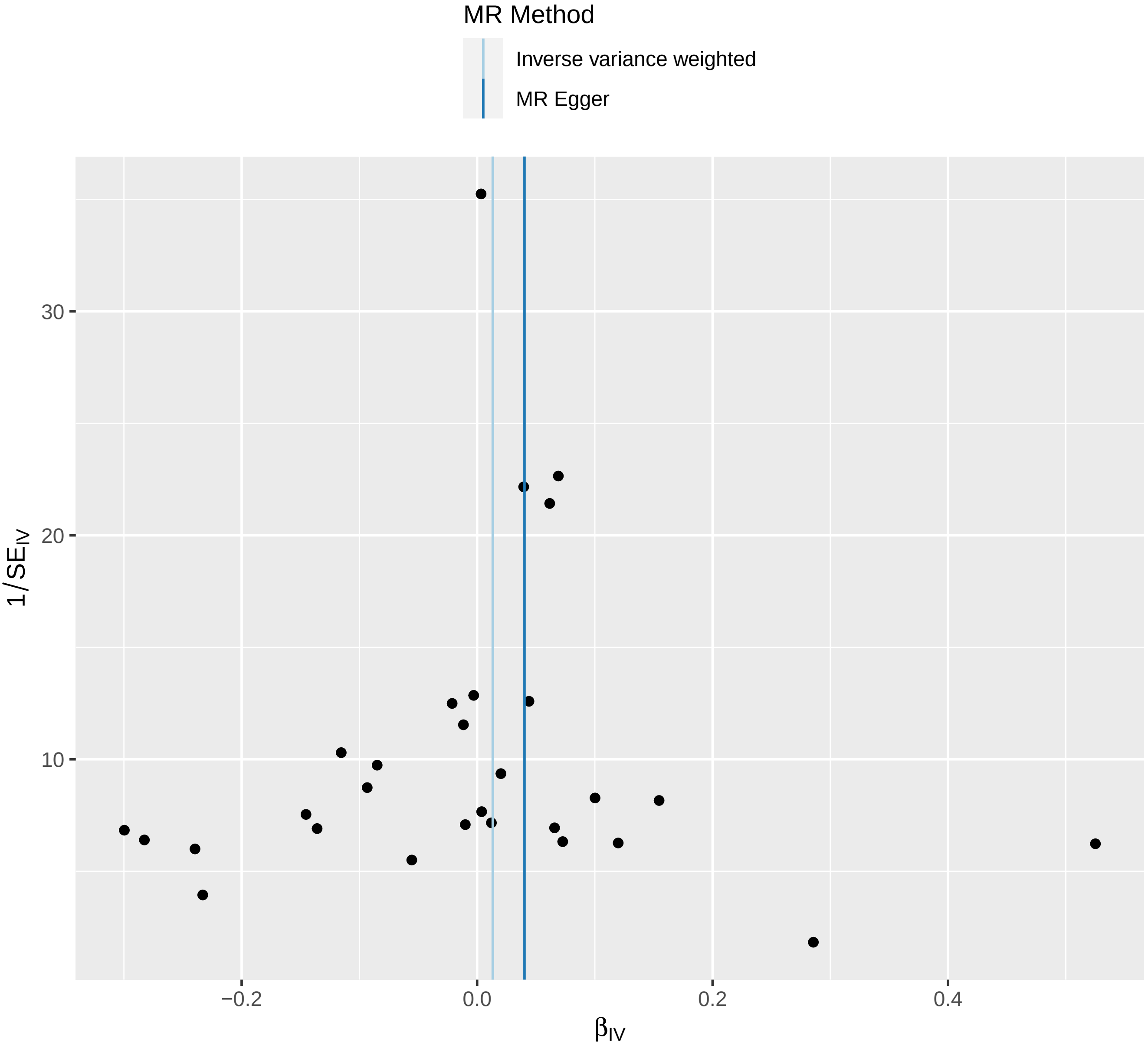

MR Method
Inverse variance weighted
MR Egger
30
20
10
1/SE$_{IV}$
−0.2
0.0
0.2
0.4
β$_{IV}$

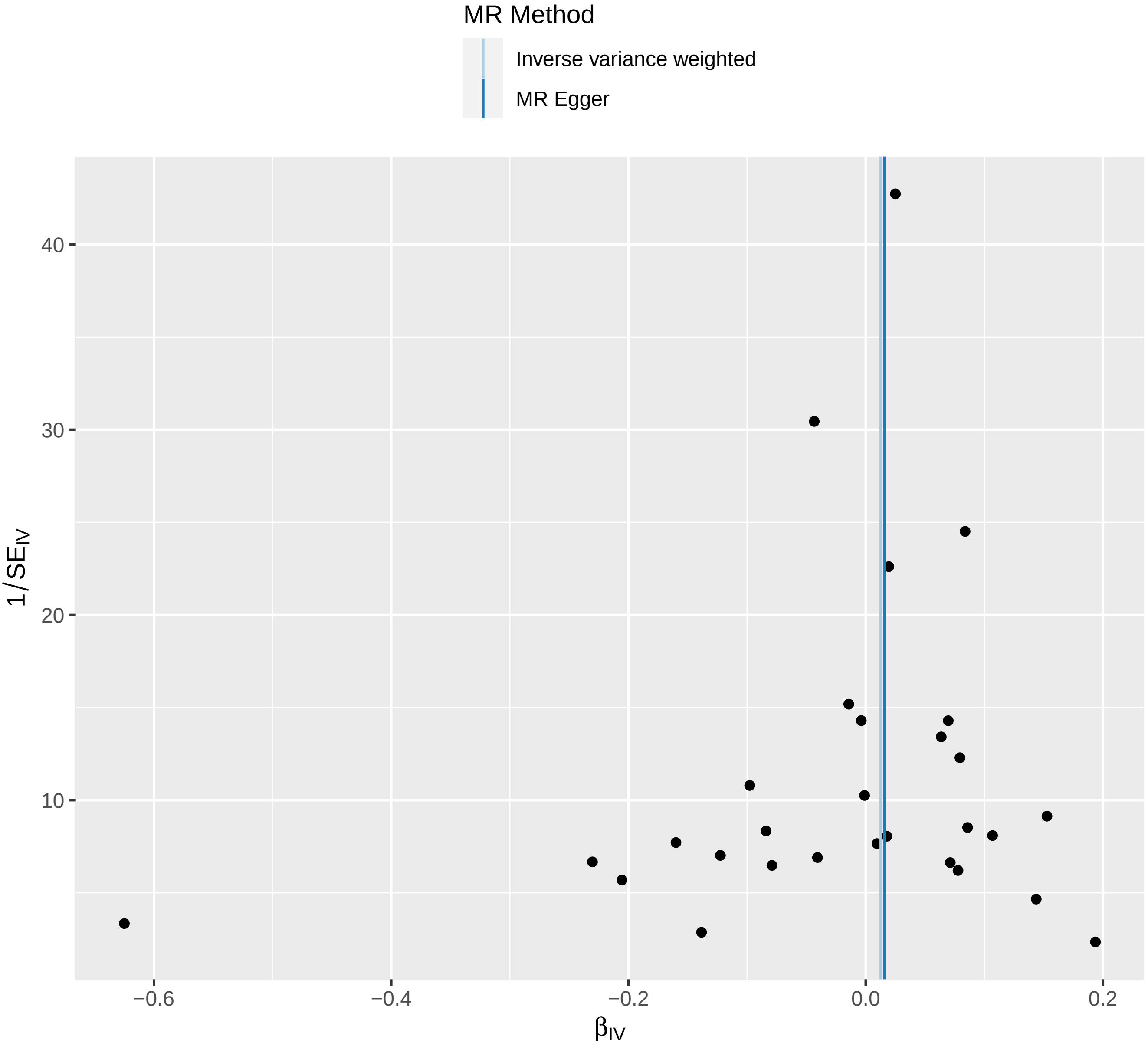

MR Method
Inverse variance weighted
MR Egger
40
30
20
10
1/SE$_{IV}$
−0.6
−0.4
−0.2
0.0
0.2
β$_{IV}$

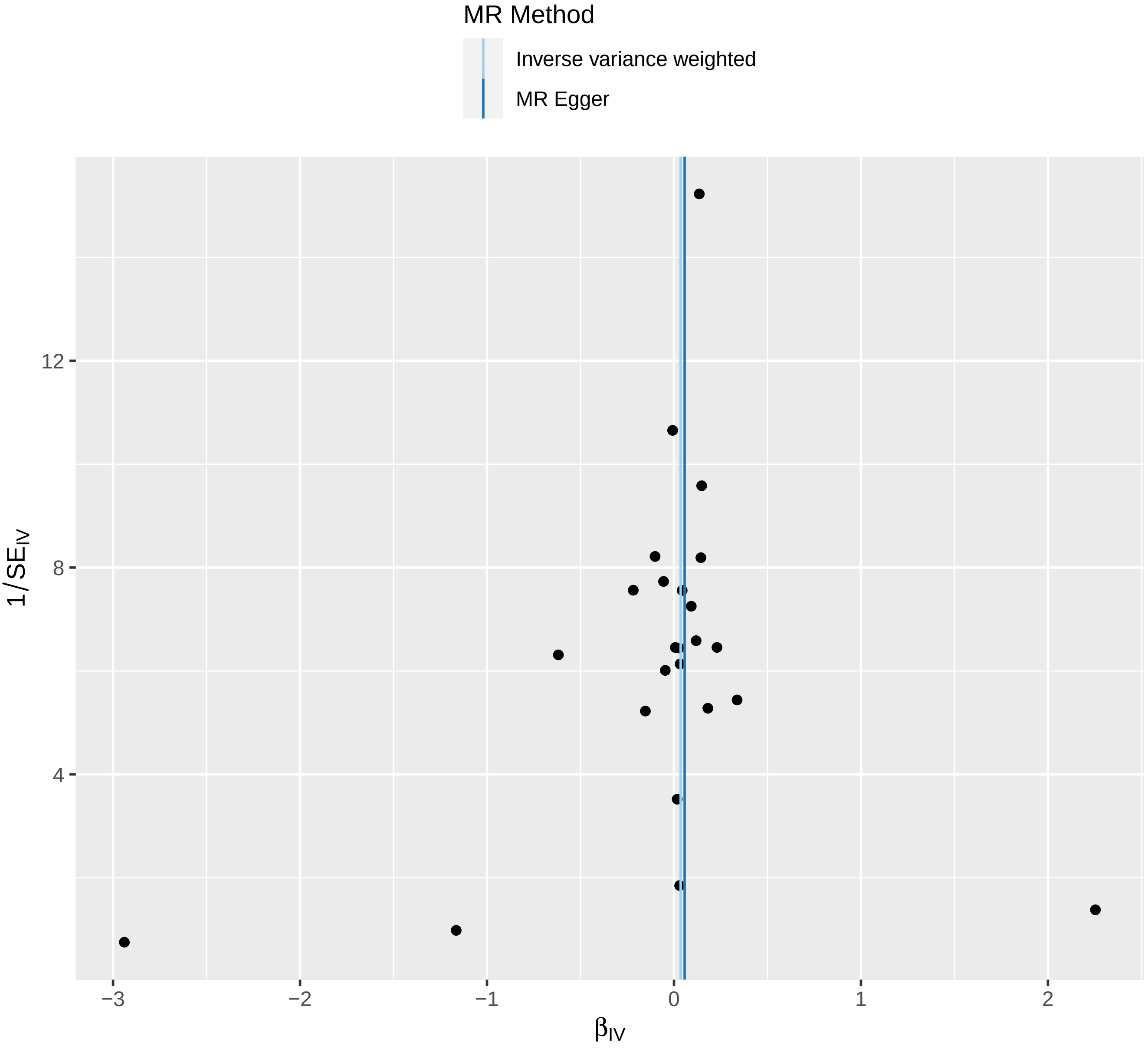

MR Method
Inverse variance weighted
MR Egger
1/SE$_{IV}$
12
8
4
−3
−2
−1
0
1
2
β$_{IV}$

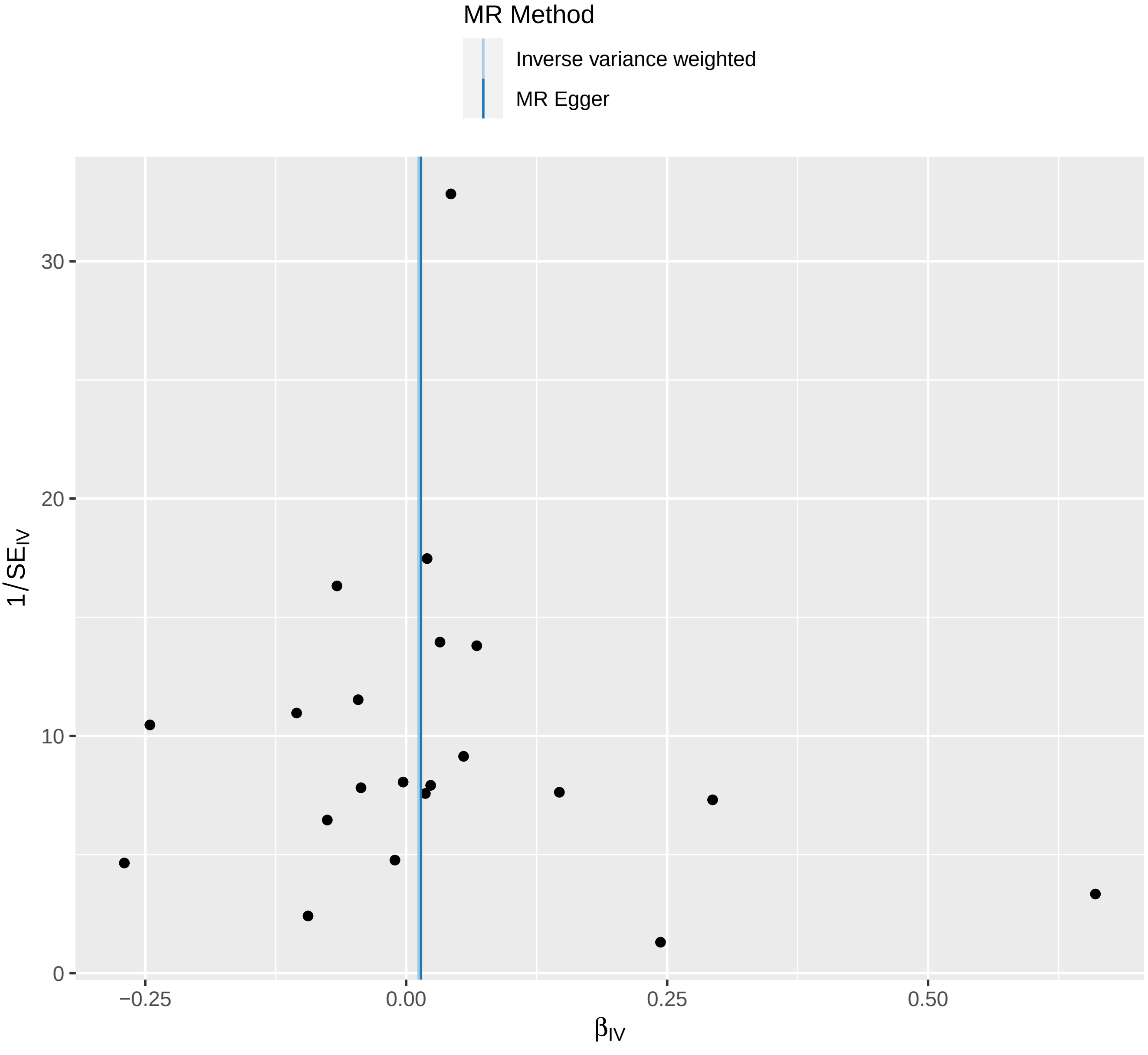

MR Method
Inverse variance weighted
MR Egger
30
20
10
0
1/SE_IV
−0.25
0.00
0.25
0.50
β_IV

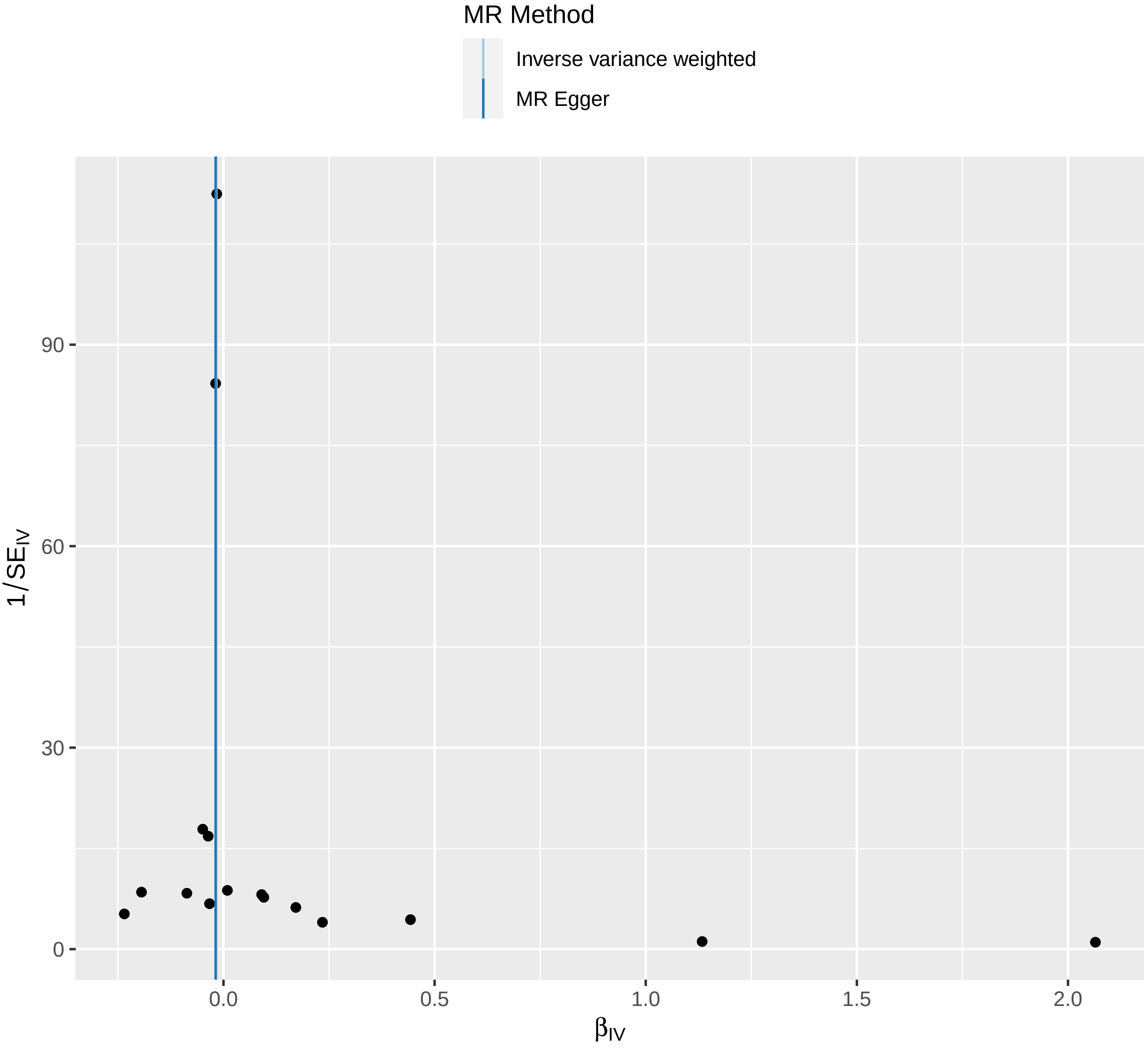
MR Method
Inverse variance weighted
MR Egger
1/SE_IV
90
60
30
0
0.0
0.5
1.0
1.5
2.0
β_IV

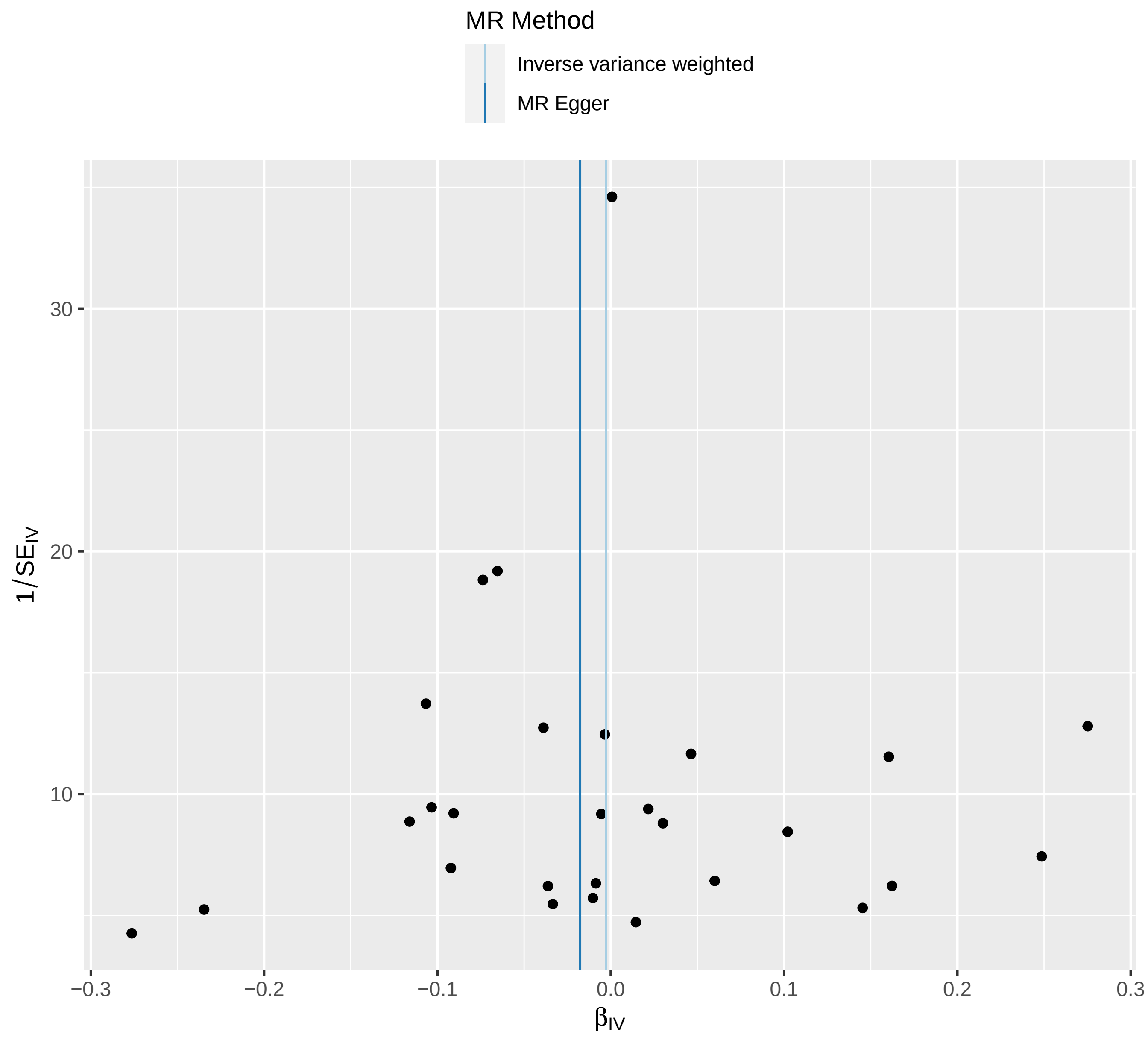
MR Method
Inverse variance weighted
MR Egger
1/SE$_{IV}$
30
20
10
−0.3
−0.2
−0.1
0.0
0.1
0.2
0.3
β$_{IV}$

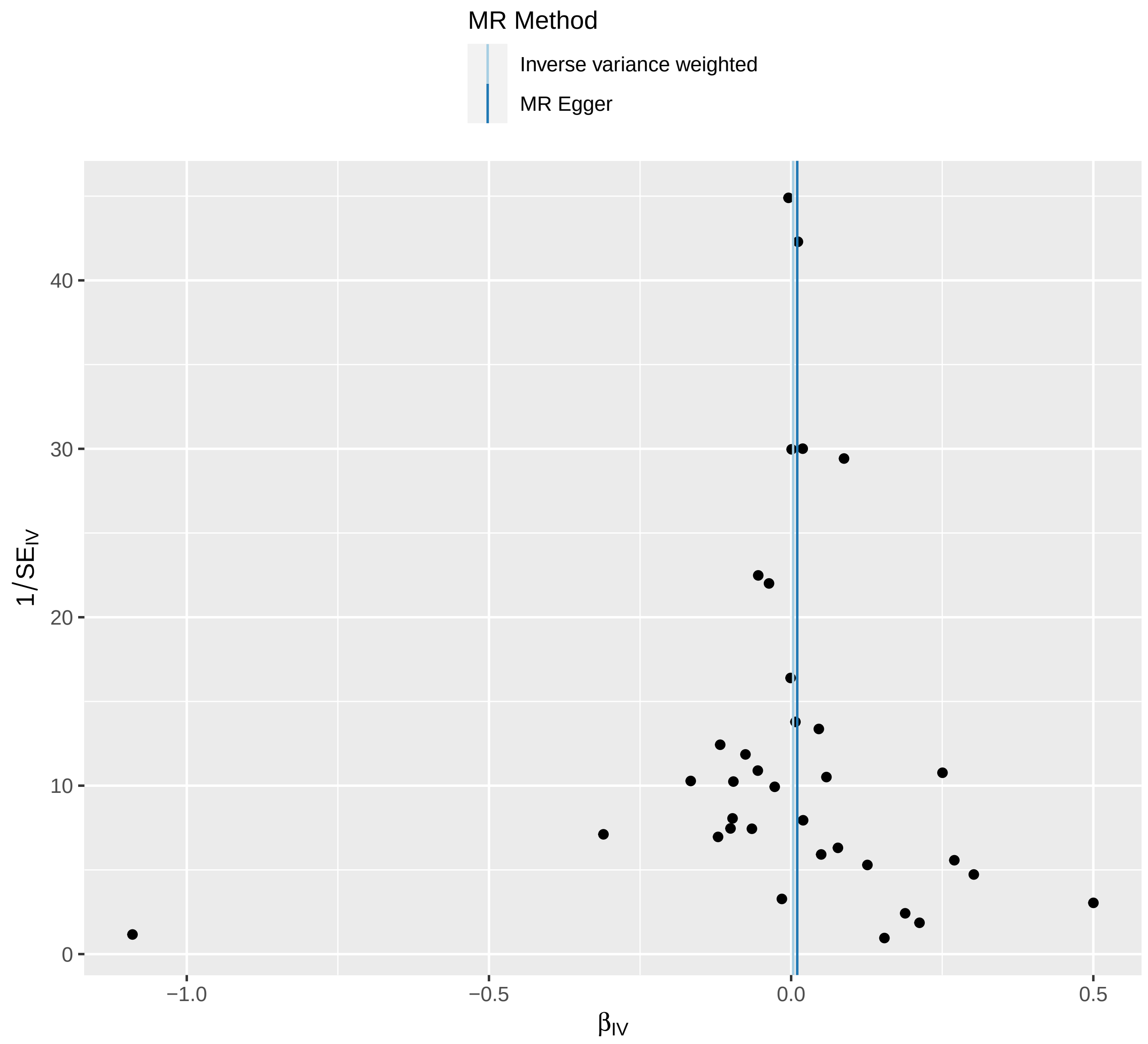

MR Method
Inverse variance weighted
MR Egger
1/SE_{IV}
0
10
20
30
40
−1.0
−0.5
0.0
0.5
β_{IV}

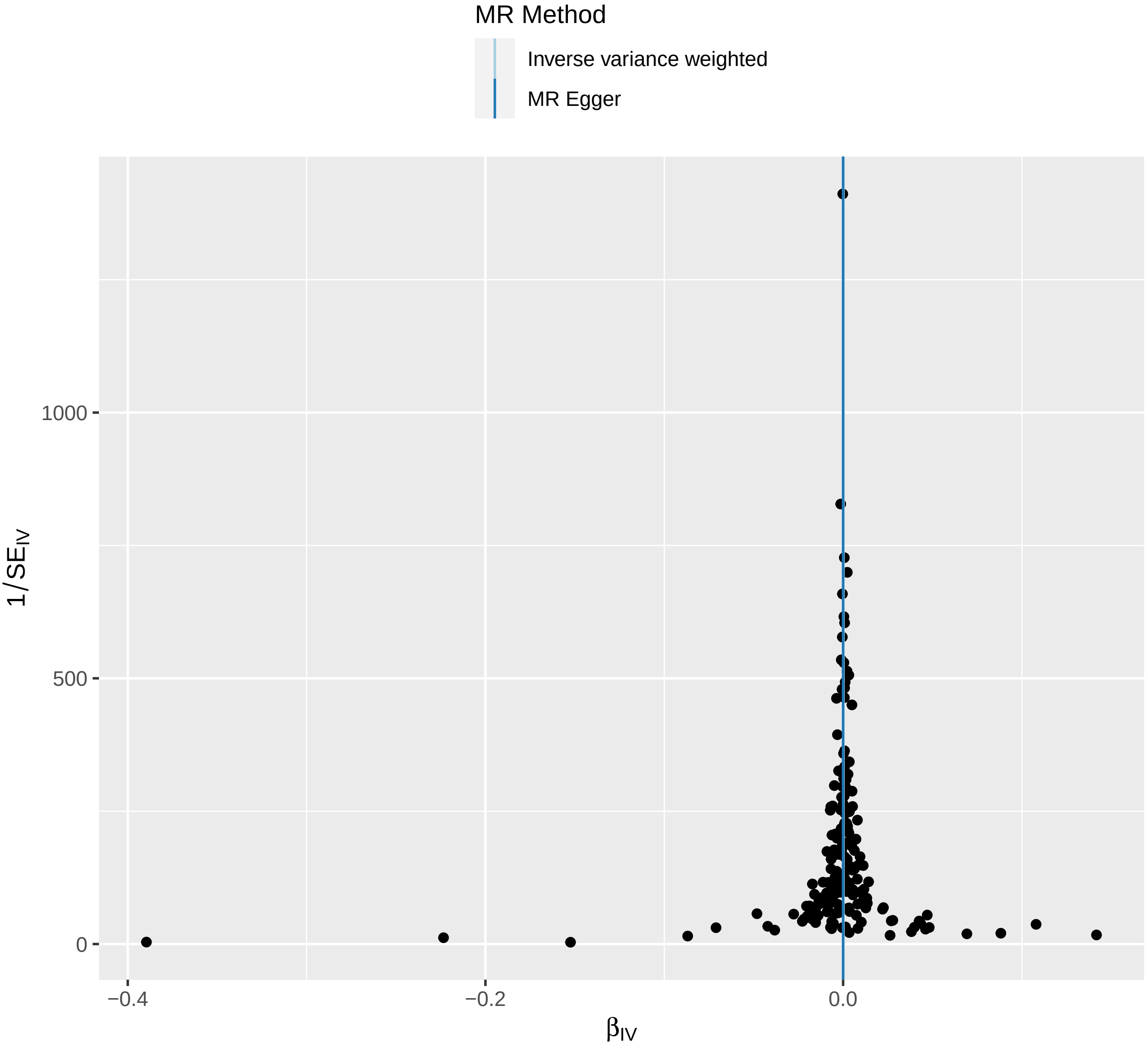

MR Method
Inverse variance weighted
MR Egger
1000
500
0
1/SE$_{IV}$
−0.4
−0.2
0.0
β_{IV}

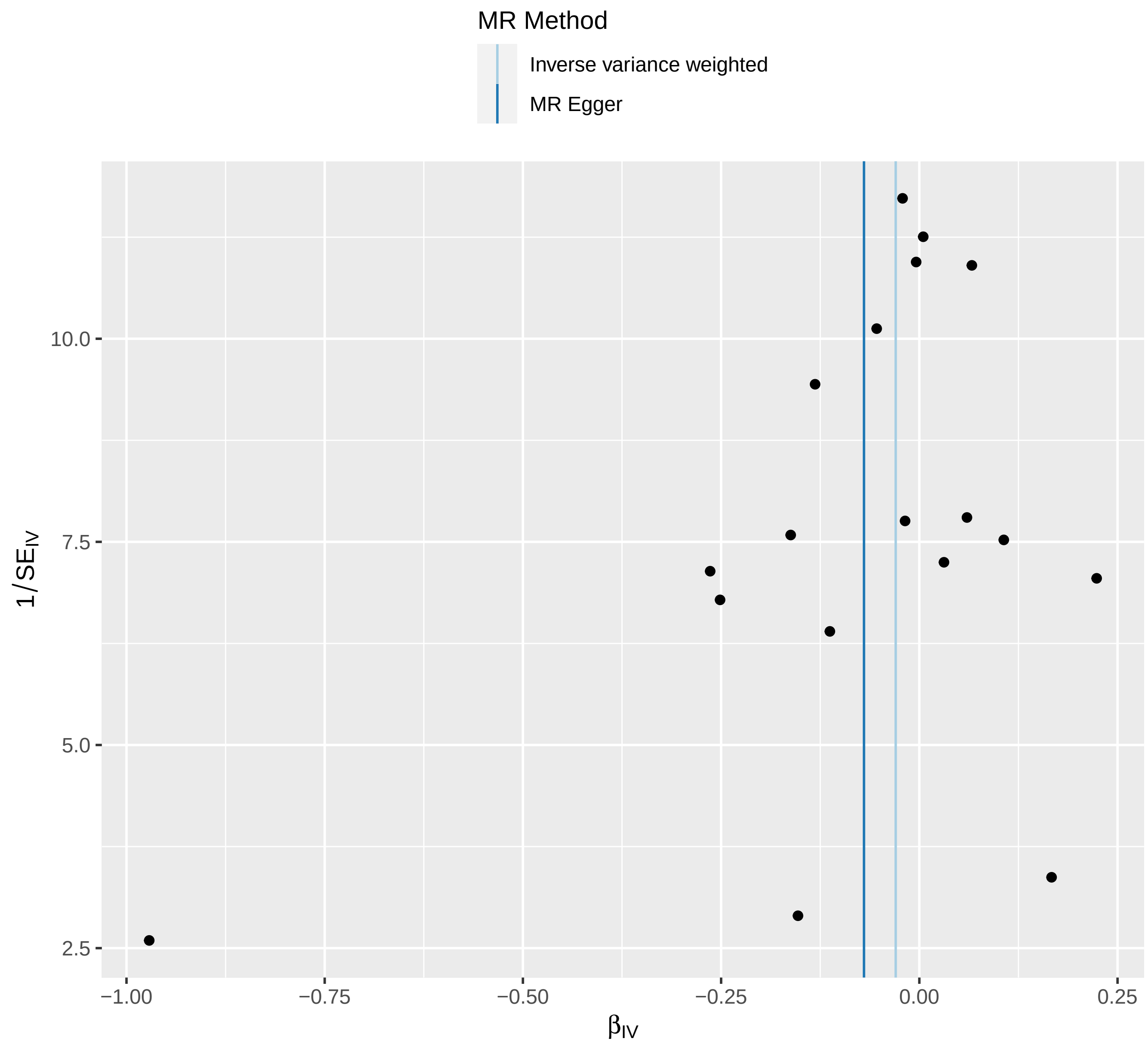
MR Method
Inverse variance weighted
MR Egger
10.0
7.5
5.0
2.5
1/SE$_{IV}$
−1.00
−0.75
−0.50
−0.25
0.00
0.25
β$_{IV}$

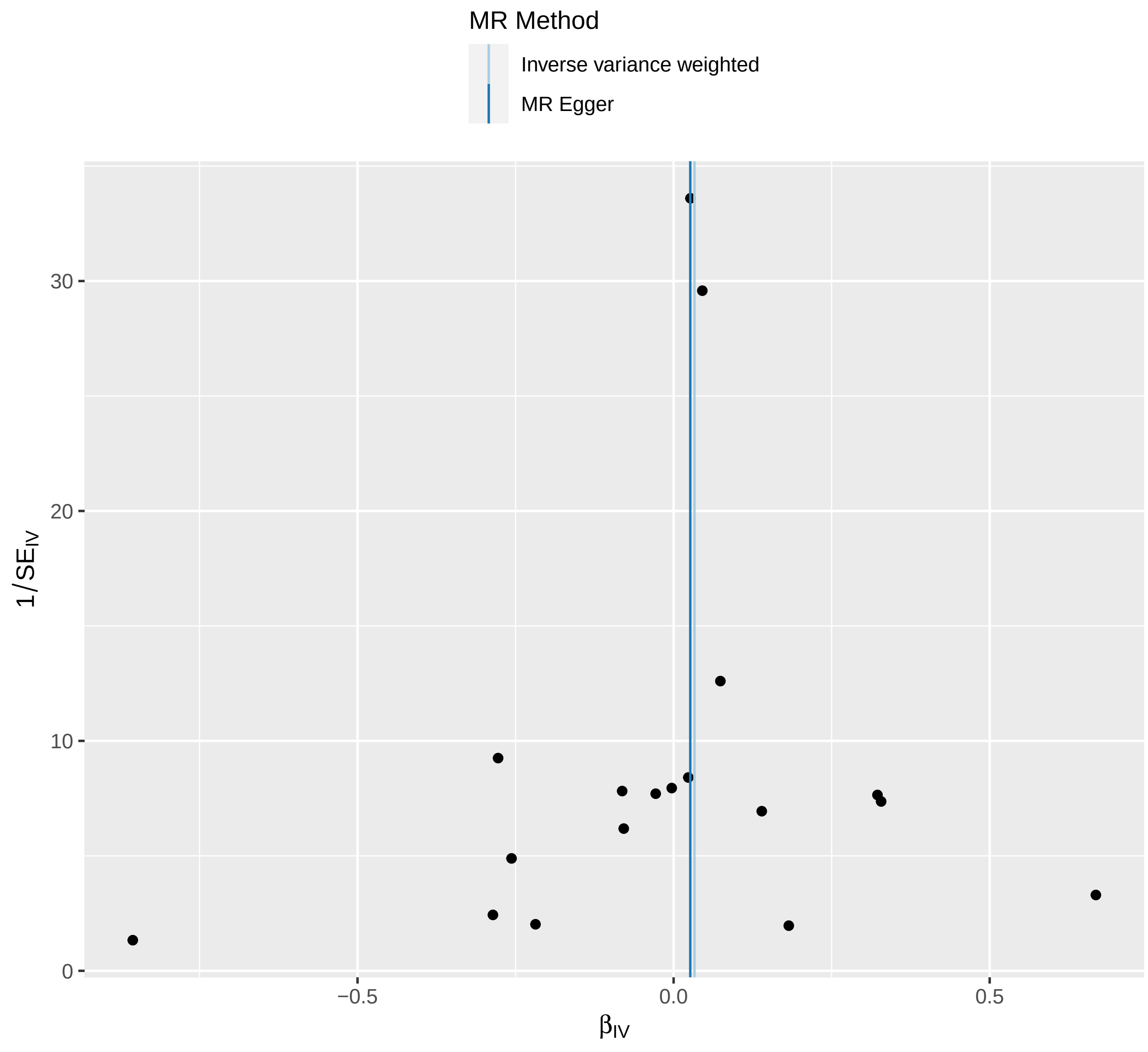
MR Method
Inverse variance weighted
MR Egger
30
20
10
0
1/SE_IV
−0.5
0.0
0.5
β_IV

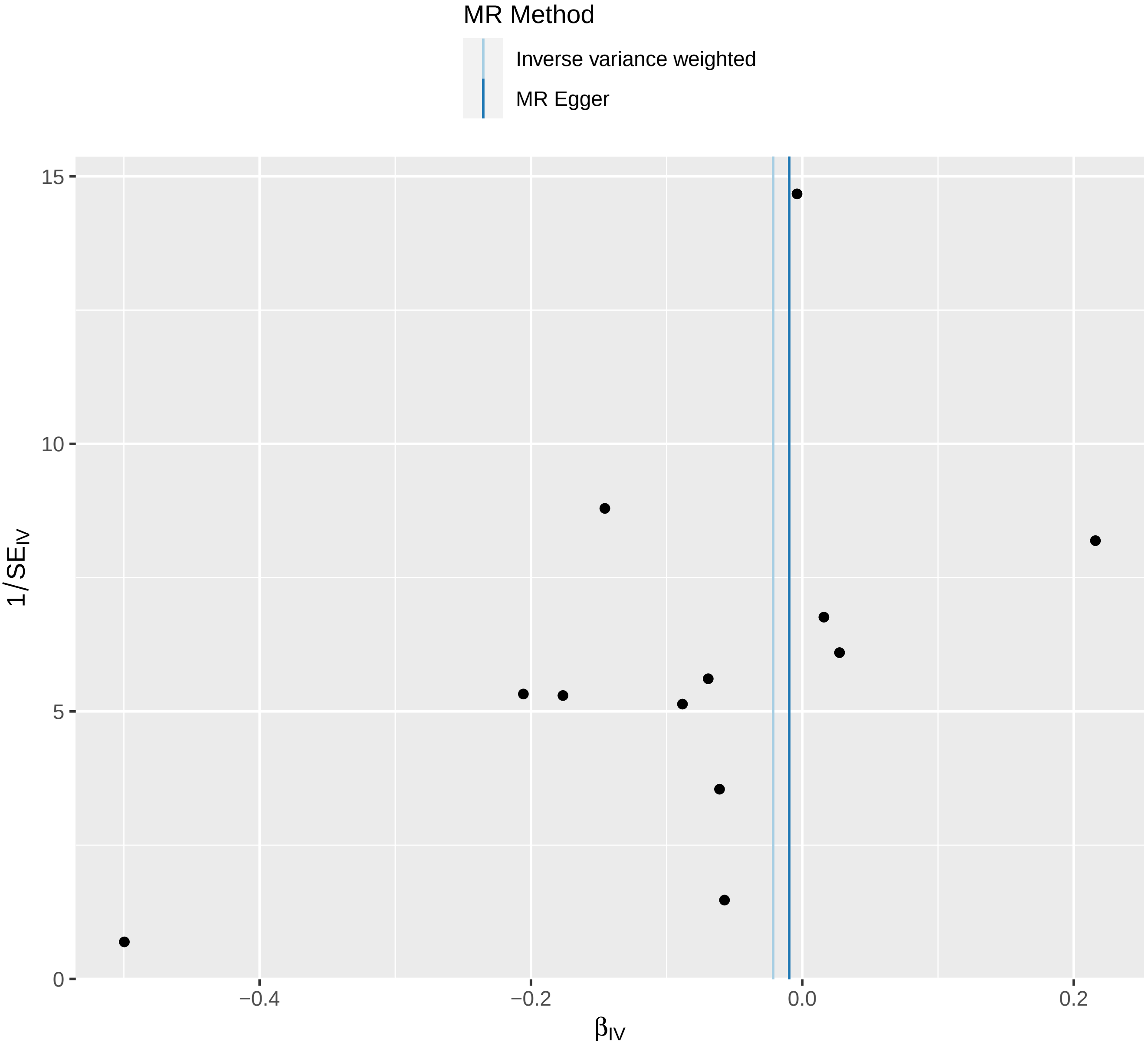

MR Method
Inverse variance weighted
MR Egger
15
10
5
0
−0.4
−0.2
0.0
0.2
$1/SE_{IV}$
β_{IV}

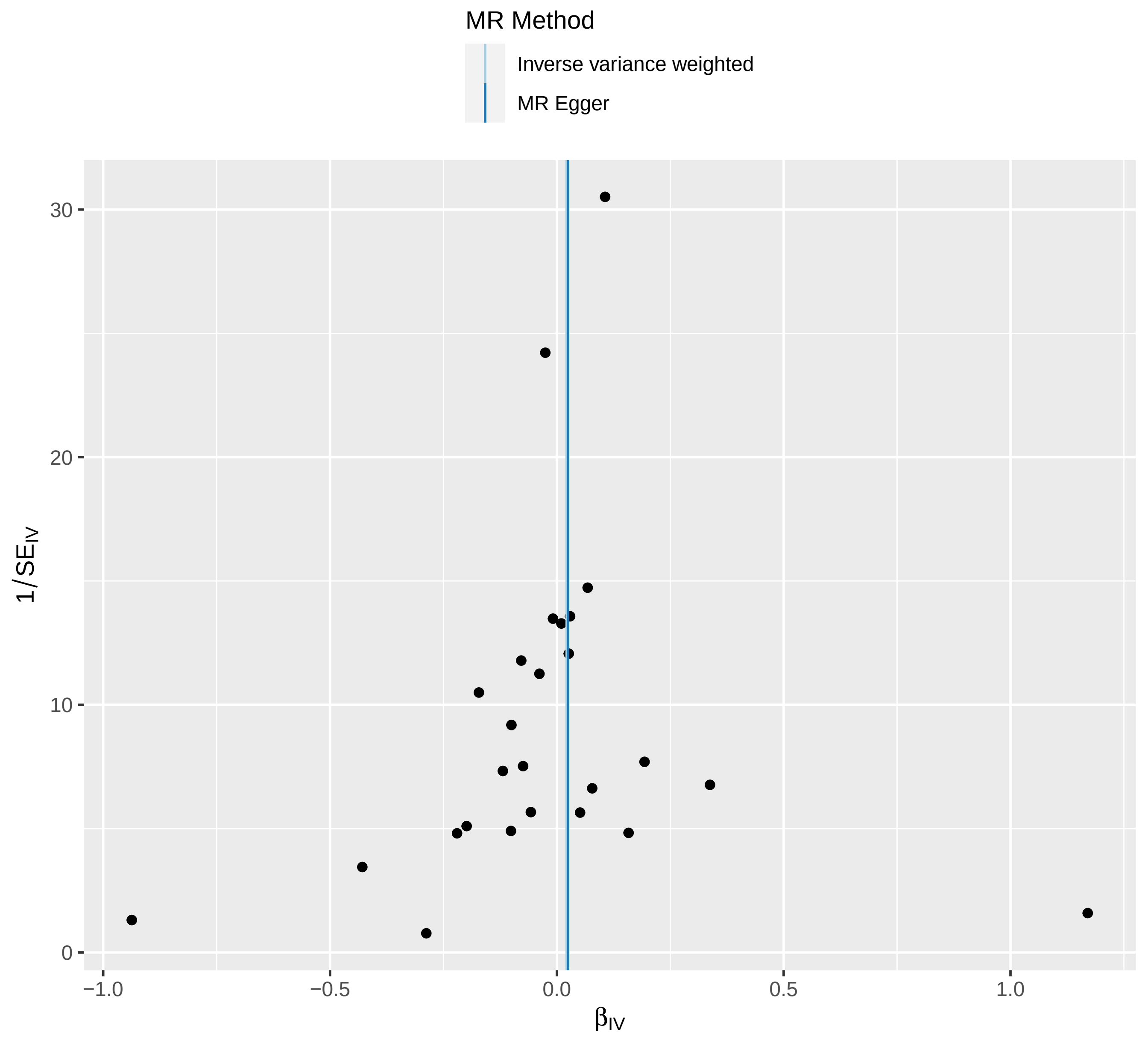

MR Method
Inverse variance weighted
MR Egger
30
20
10
0
−1.0
−0.5
0.0
0.5
1.0
$1/SE_{IV}$
β_{IV}

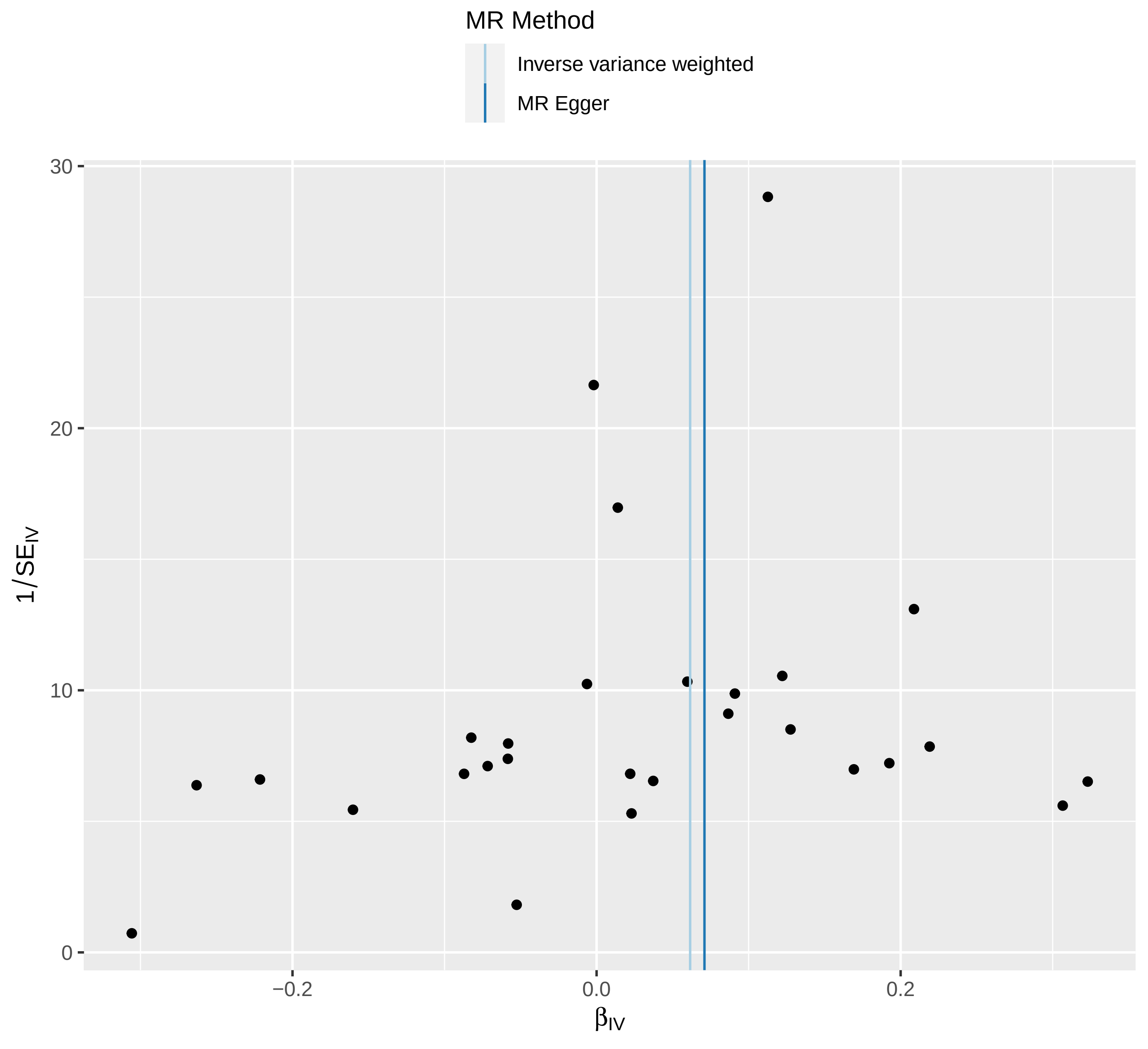

MR Method
Inverse variance weighted
MR Egger
30
20
10
0
1/SE$_{IV}$
−0.2
0.0
0.2
β$_{IV}$

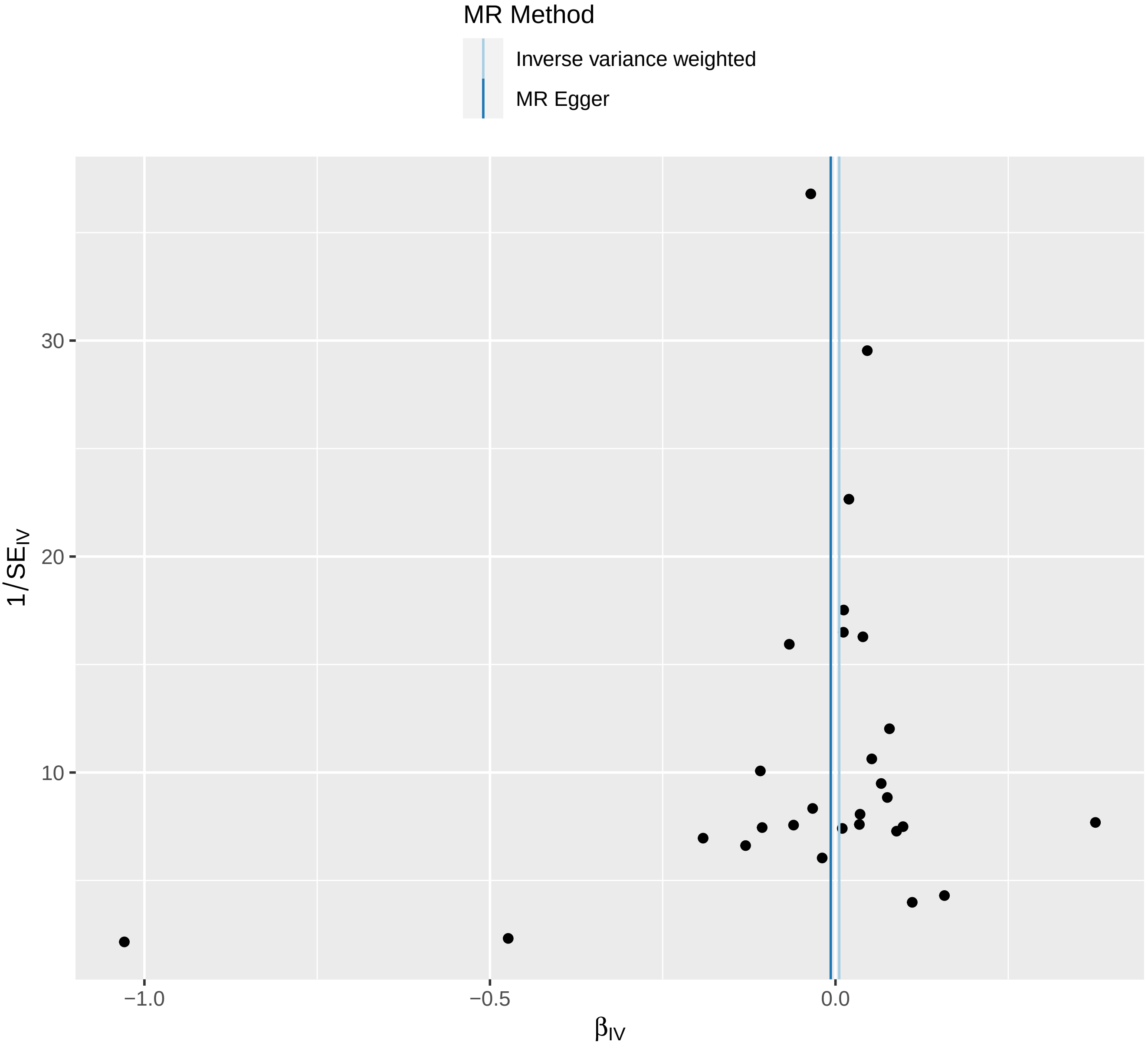

MR Method
Inverse variance weighted
MR Egger
30
20
10
$1/SE_{IV}$
−1.0
−0.5
0.0
β_{IV}

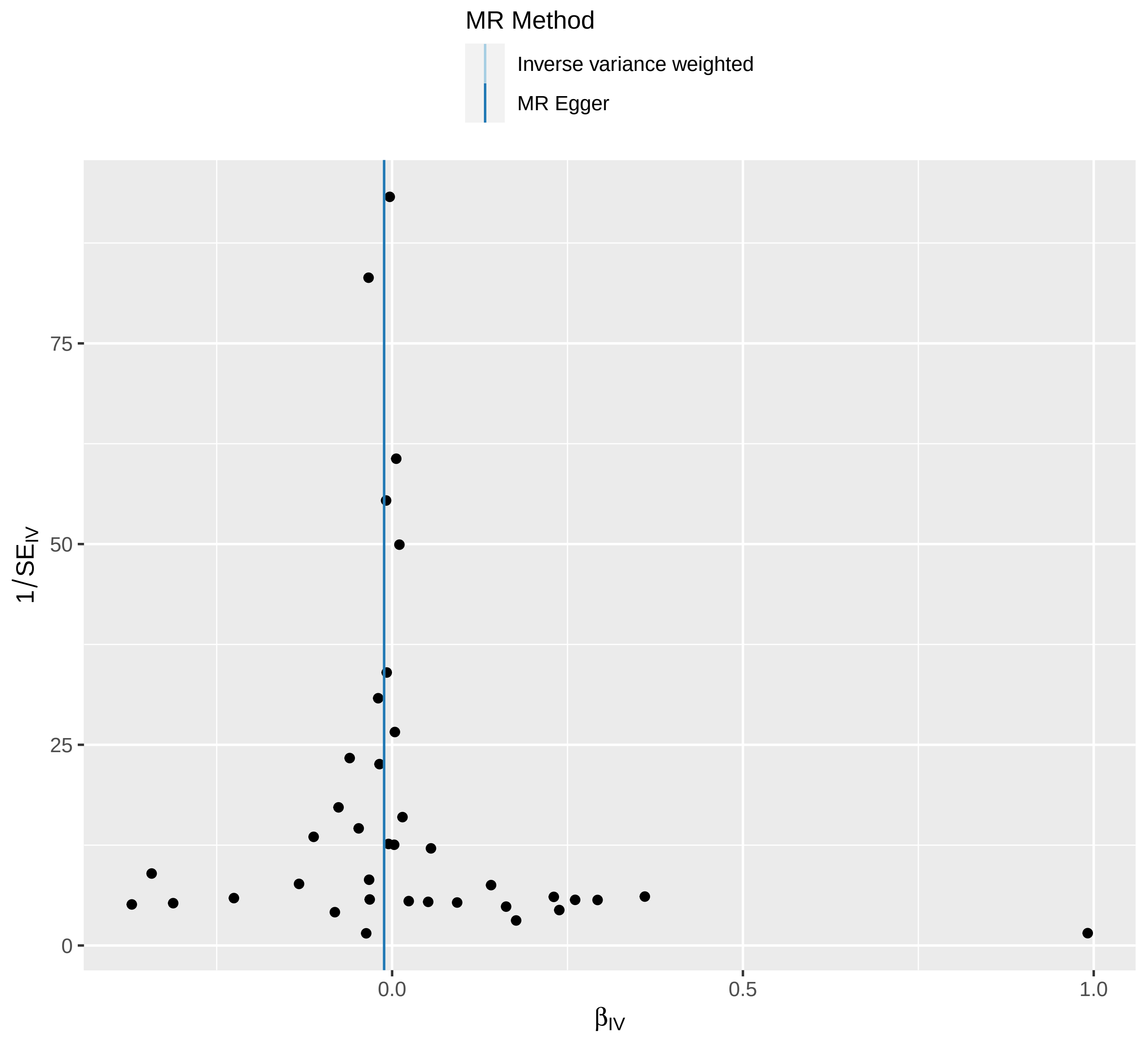

MR Method
Inverse variance weighted
MR Egger
75
50
25
0
0.0
0.5
1.0
$1/SE_{IV}$
β_{IV}

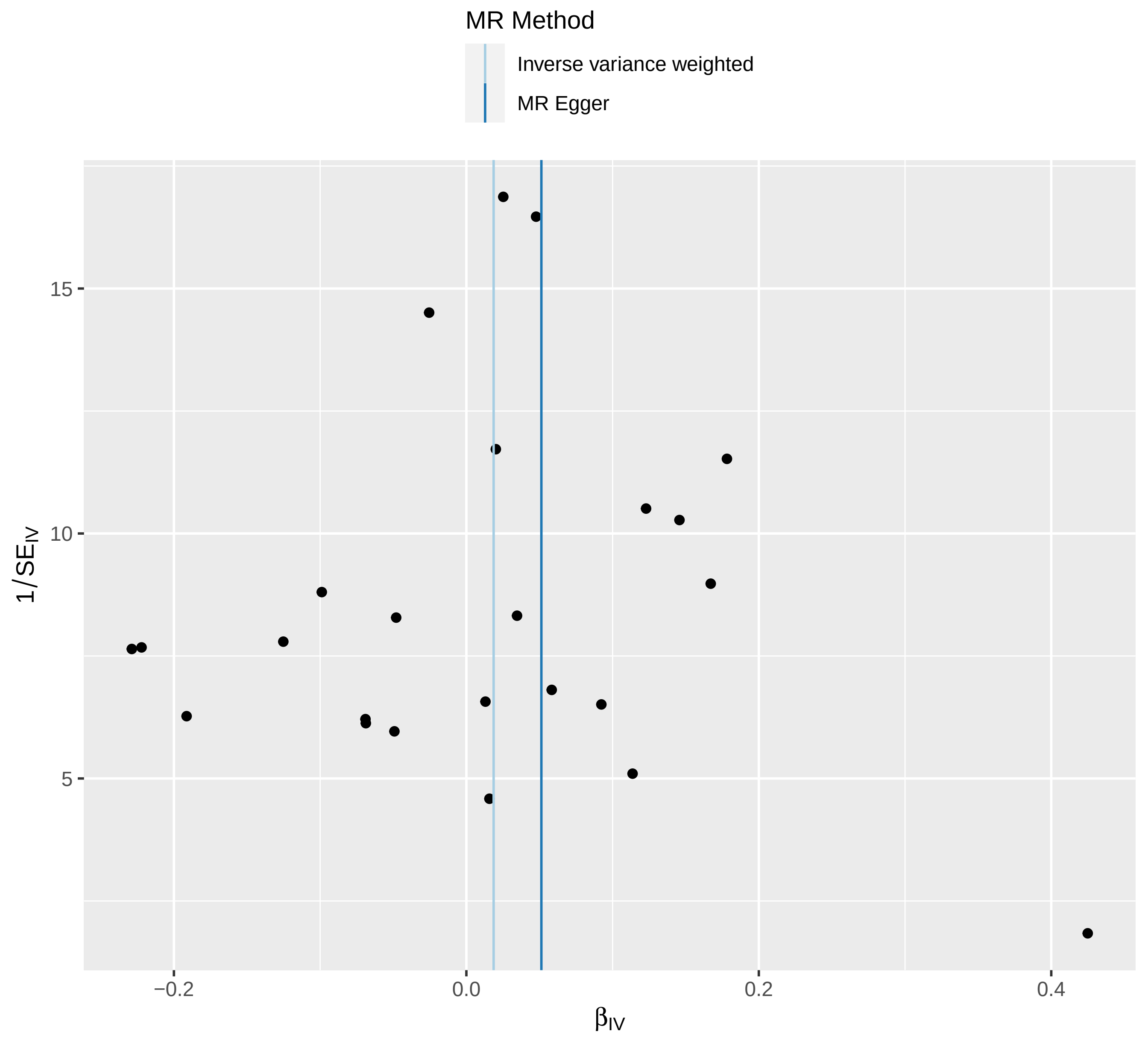

MR Method
Inverse variance weighted
MR Egger
15
10
5
1/SE_IV
−0.2
0.0
0.2
0.4
β_IV

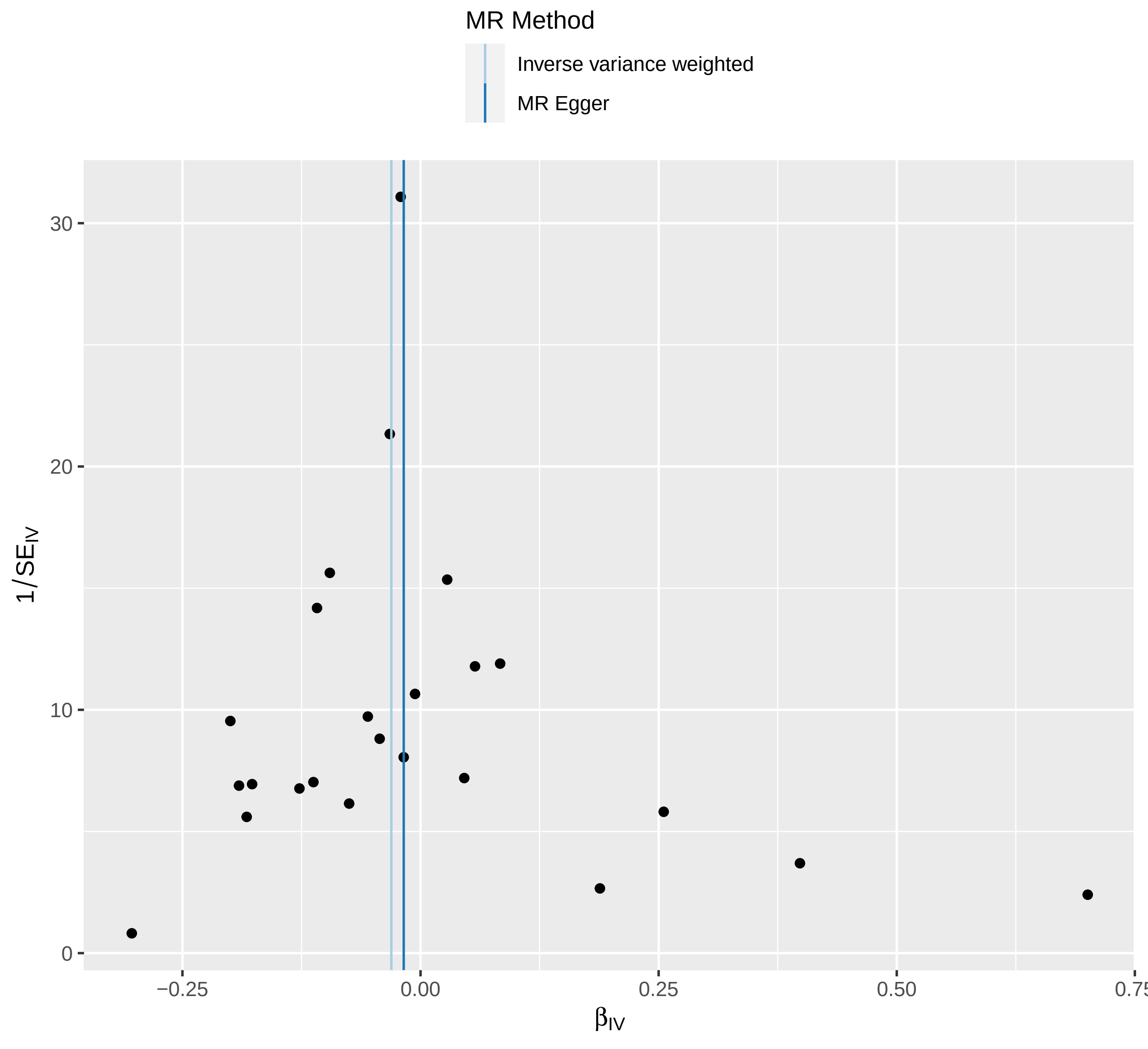
MR Method
Inverse variance weighted
MR Egger
1/SE$_{IV}$
β$_{IV}$
30
20
10
0
−0.25
0.00
0.25
0.50
0.75

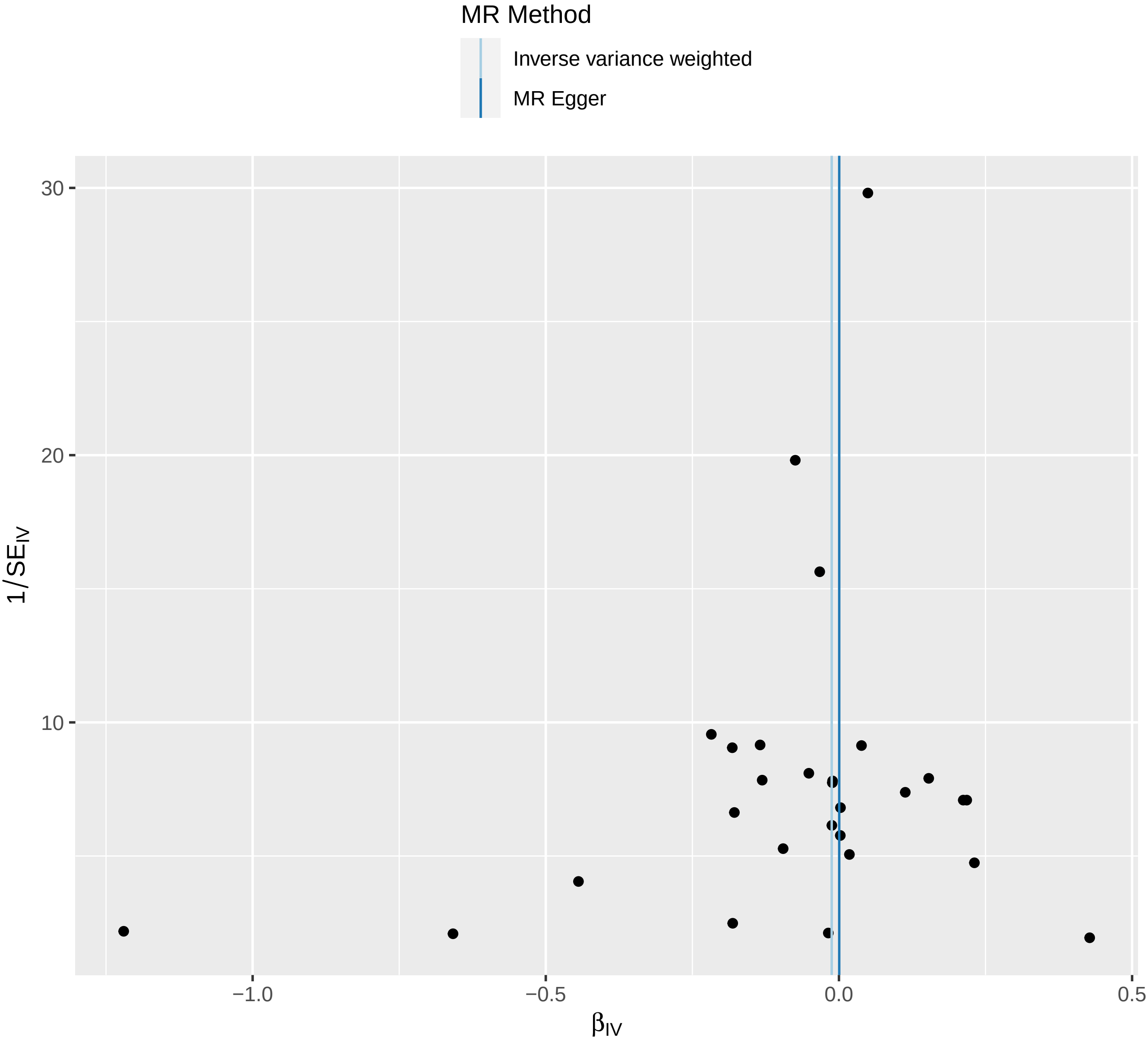

MR Method
Inverse variance weighted
MR Egger
30
20
10
1/SE$_{IV}$
−1.0
−0.5
0.0
0.5
β$_{IV}$

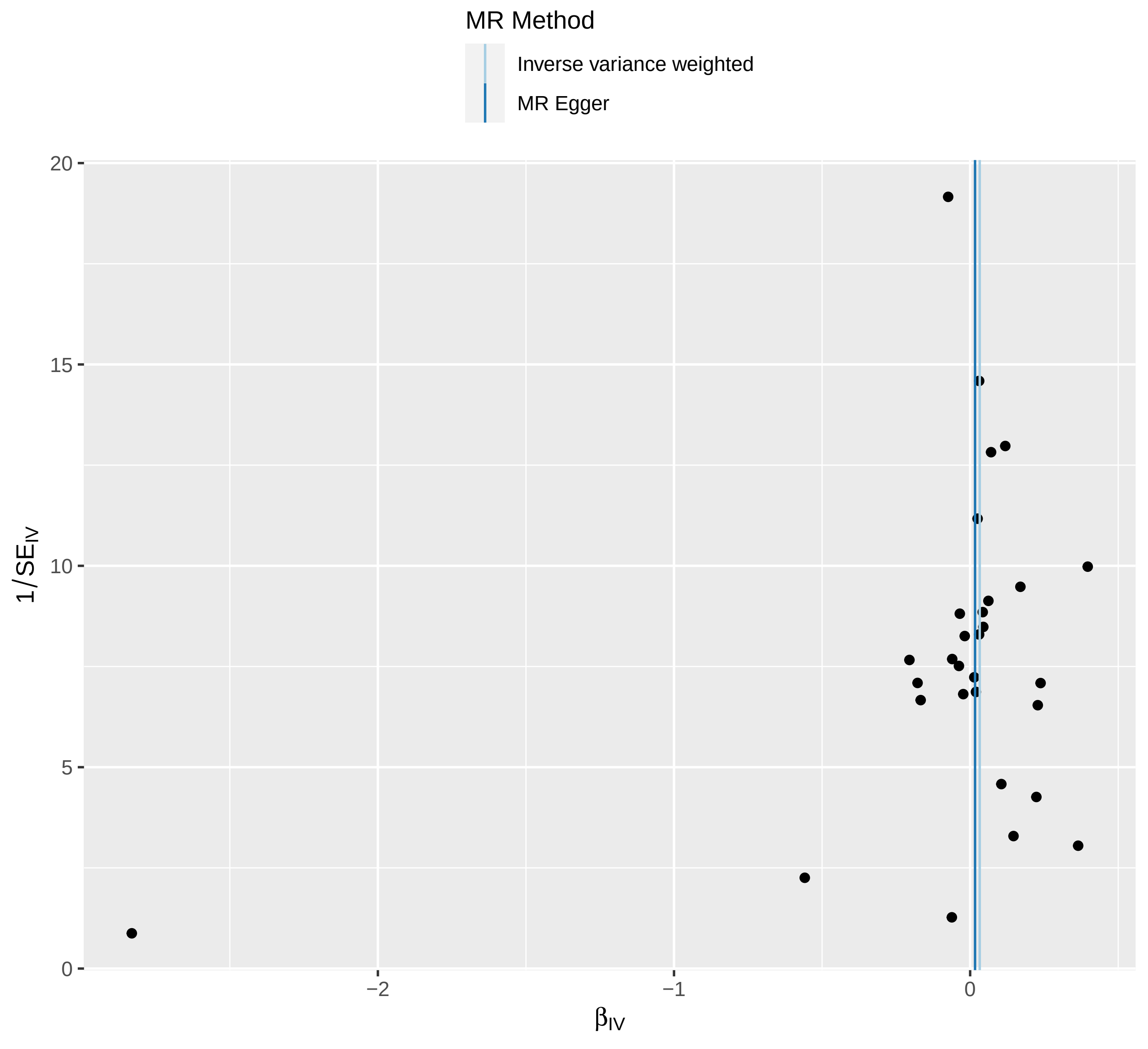
MR Method
Inverse variance weighted
MR Egger
20
15
10
5
0
1/SE$_{IV}$
−2
−1
0
β$_{IV}$

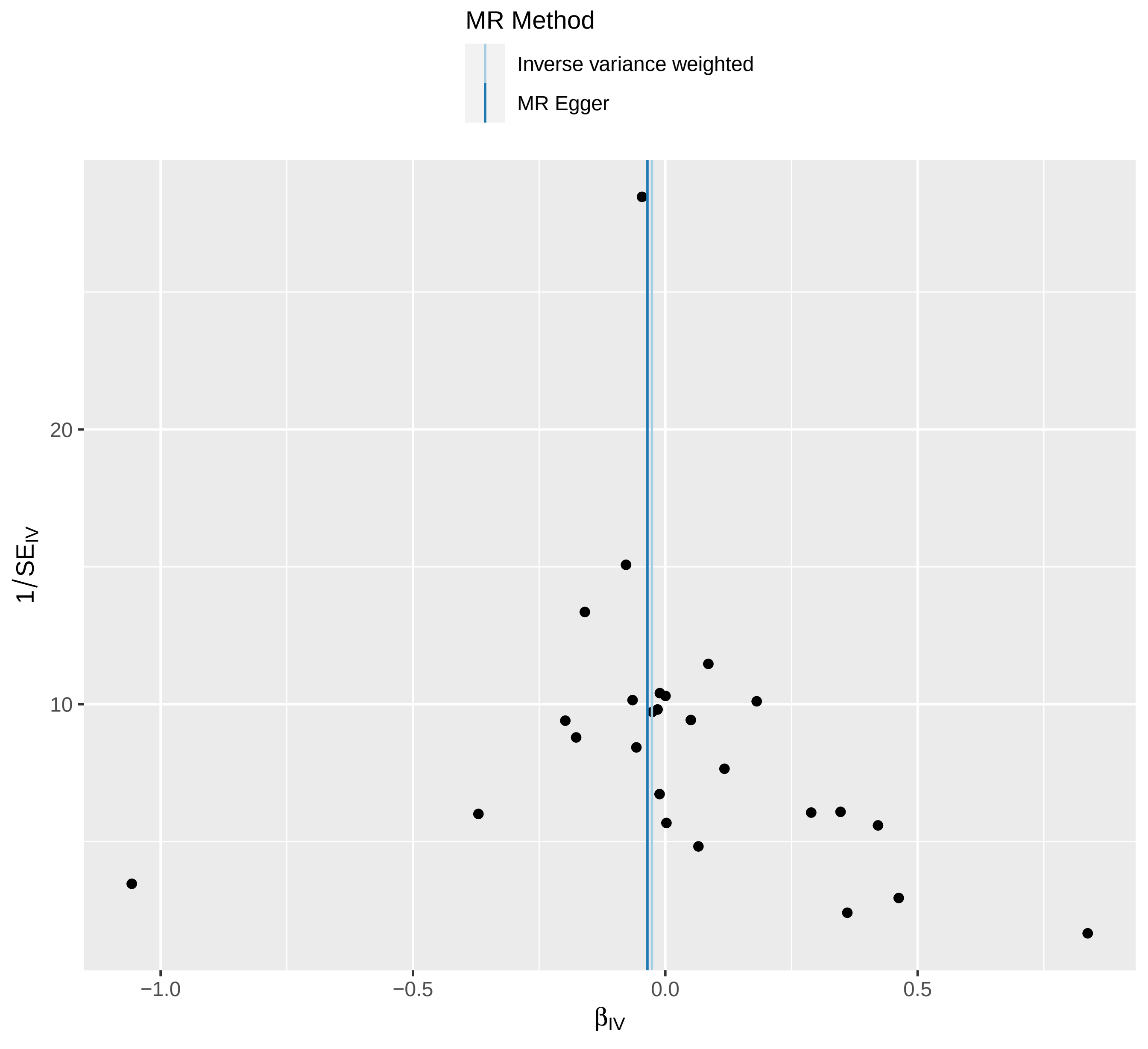

MR Method
Inverse variance weighted
MR Egger
20
10
1/SE_IV
−1.0
−0.5
0.0
0.5
β_IV

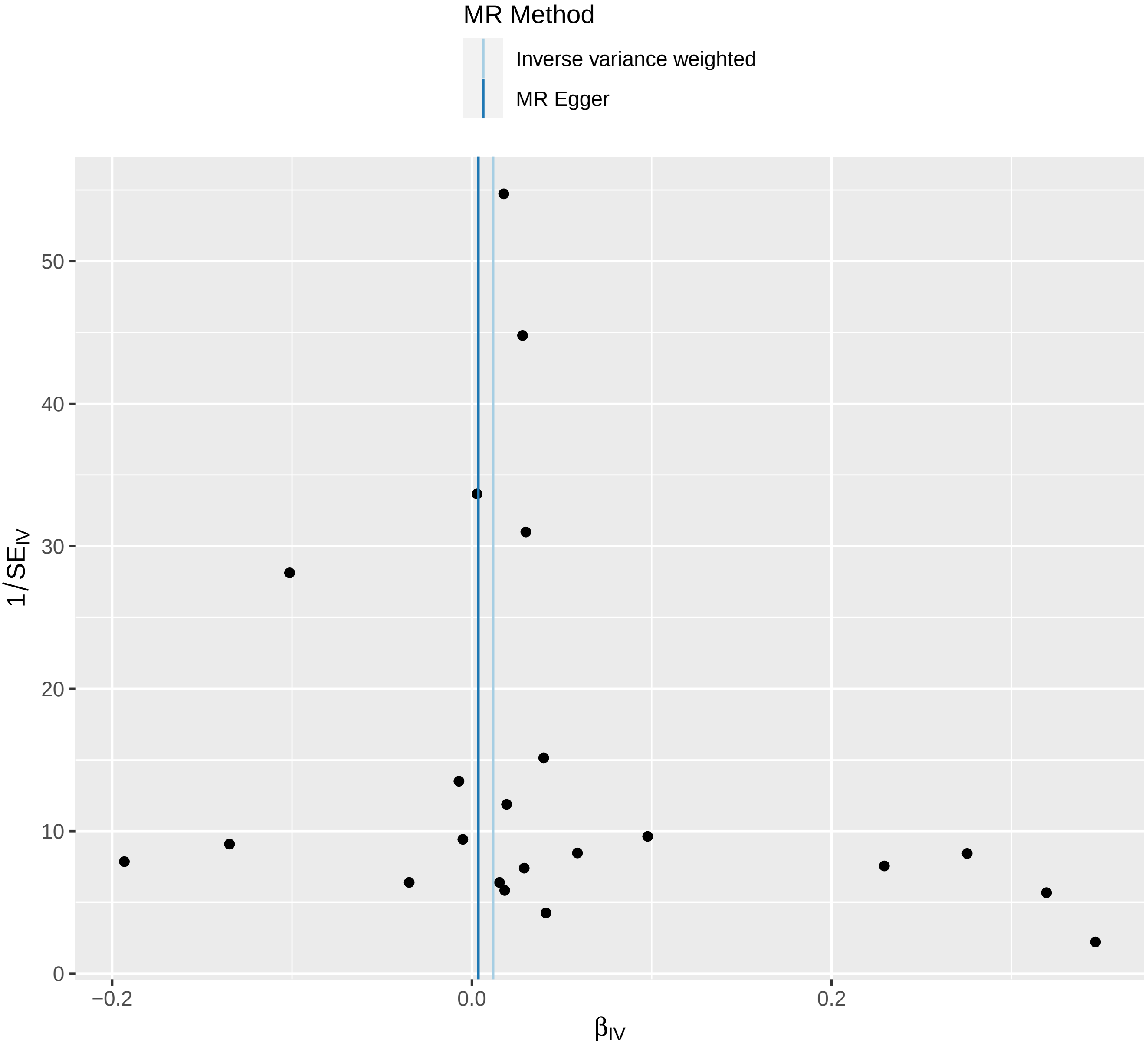

MR Method
Inverse variance weighted
MR Egger
50
40
30
20
10
0
1/SE$_{IV}$
−0.2
0.0
0.2
β$_{IV}$

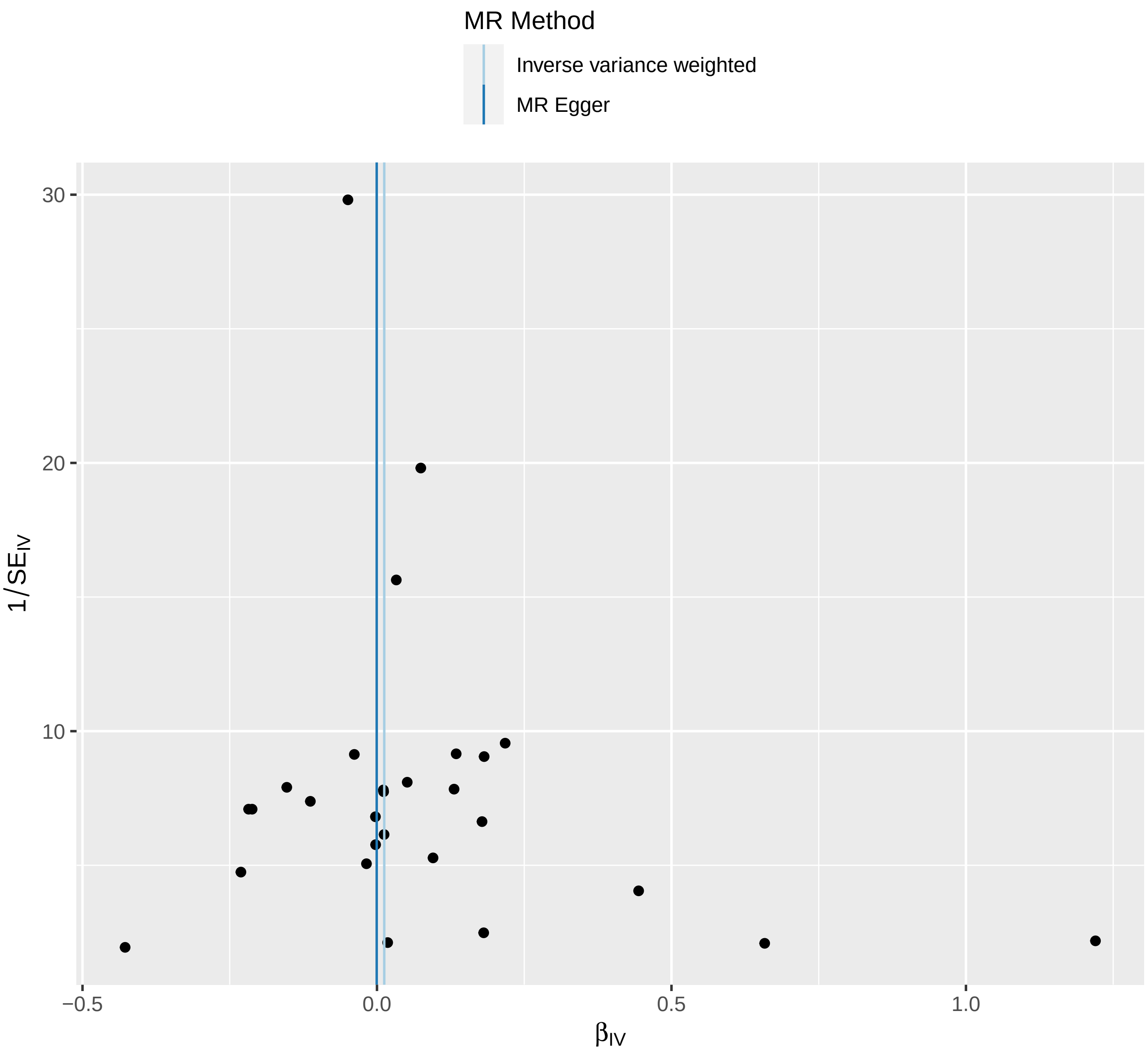

MR Method
Inverse variance weighted
MR Egger
30
20
10
1/SE$_{IV}$
−0.5
0.0
0.5
1.0
β$_{IV}$

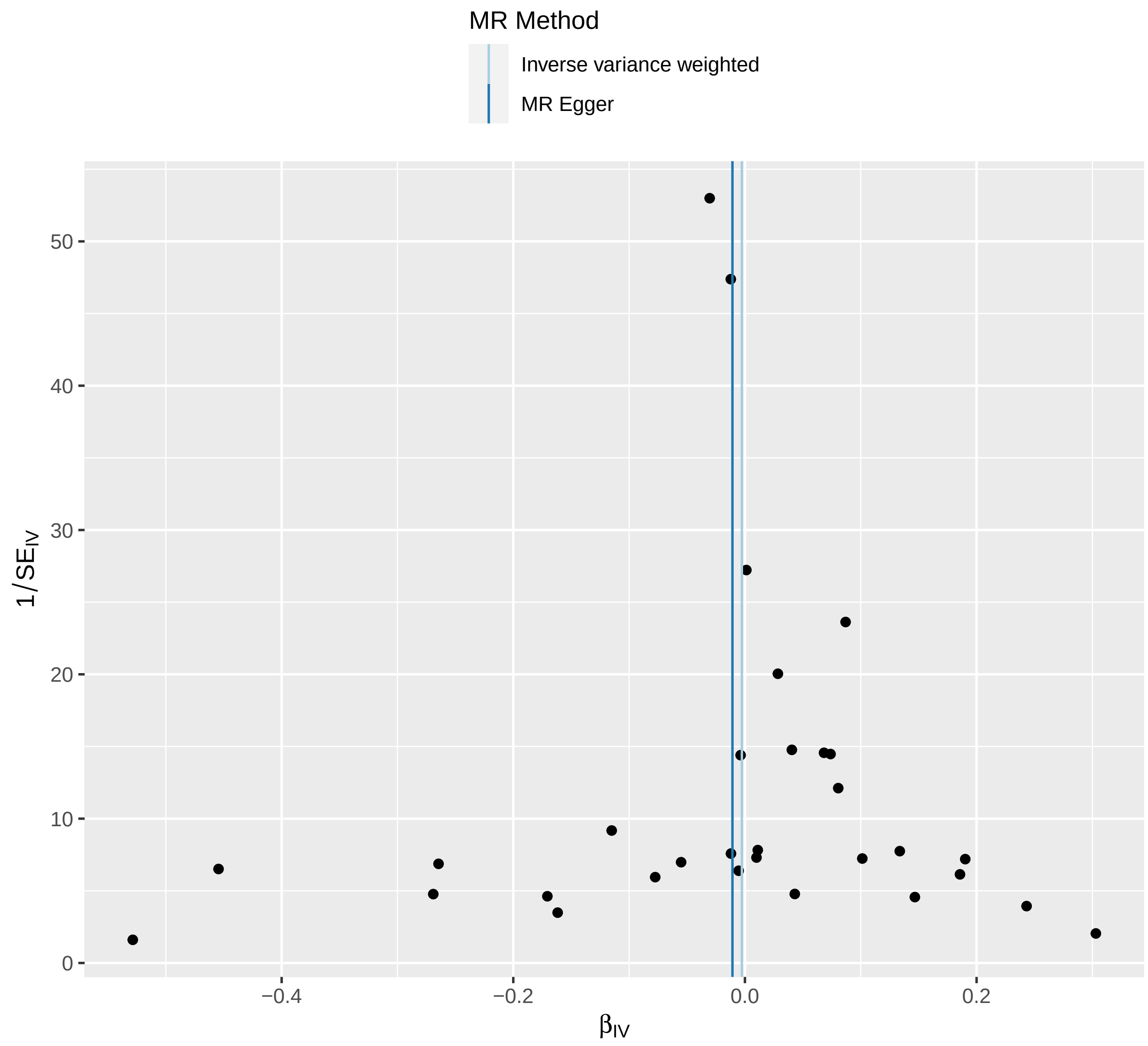

MR Method
Inverse variance weighted
MR Egger
$1/SE_{IV}$
β_{IV}
0
10
20
30
40
50
−0.4
−0.2
0.0
0.2

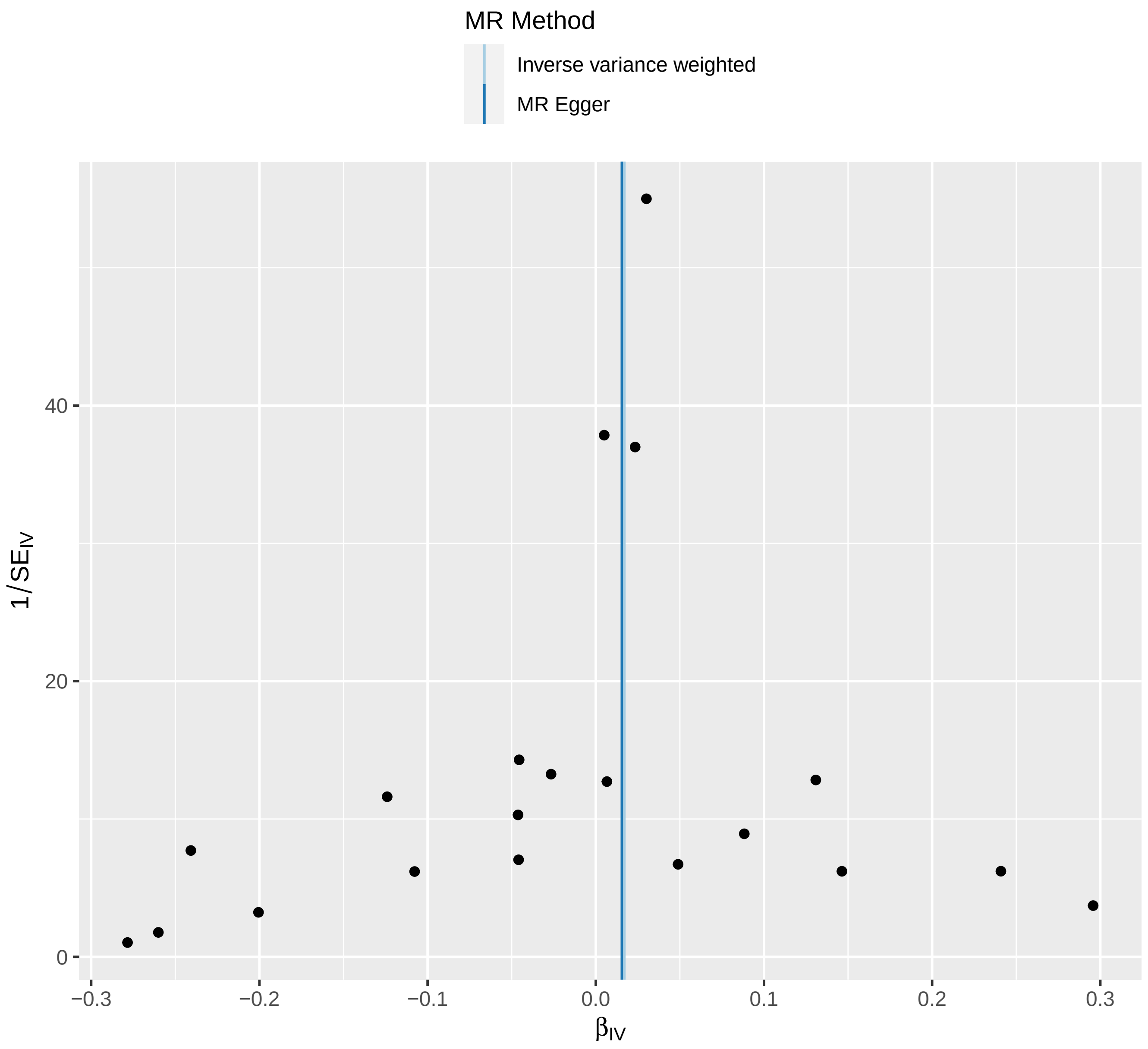
MR Method
Inverse variance weighted
MR Egger
$1/SE_{IV}$
40
20
0
−0.3
−0.2
−0.1
0.0
0.1
0.2
0.3
β_{IV}

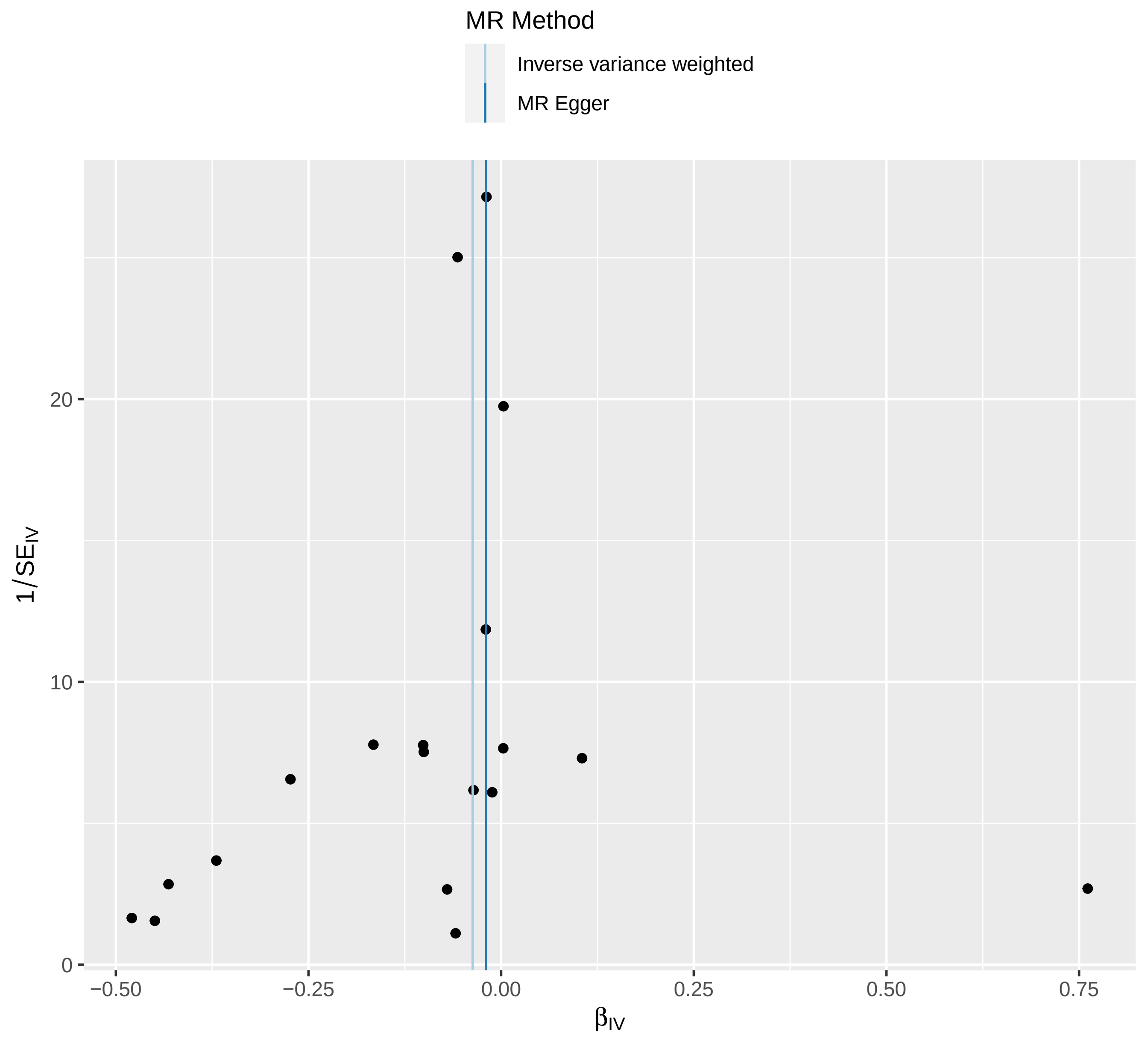

MR Method
Inverse variance weighted
MR Egger
20
10
0
1/SE_IV
−0.50
−0.25
0.00
0.25
0.50
0.75
β_IV

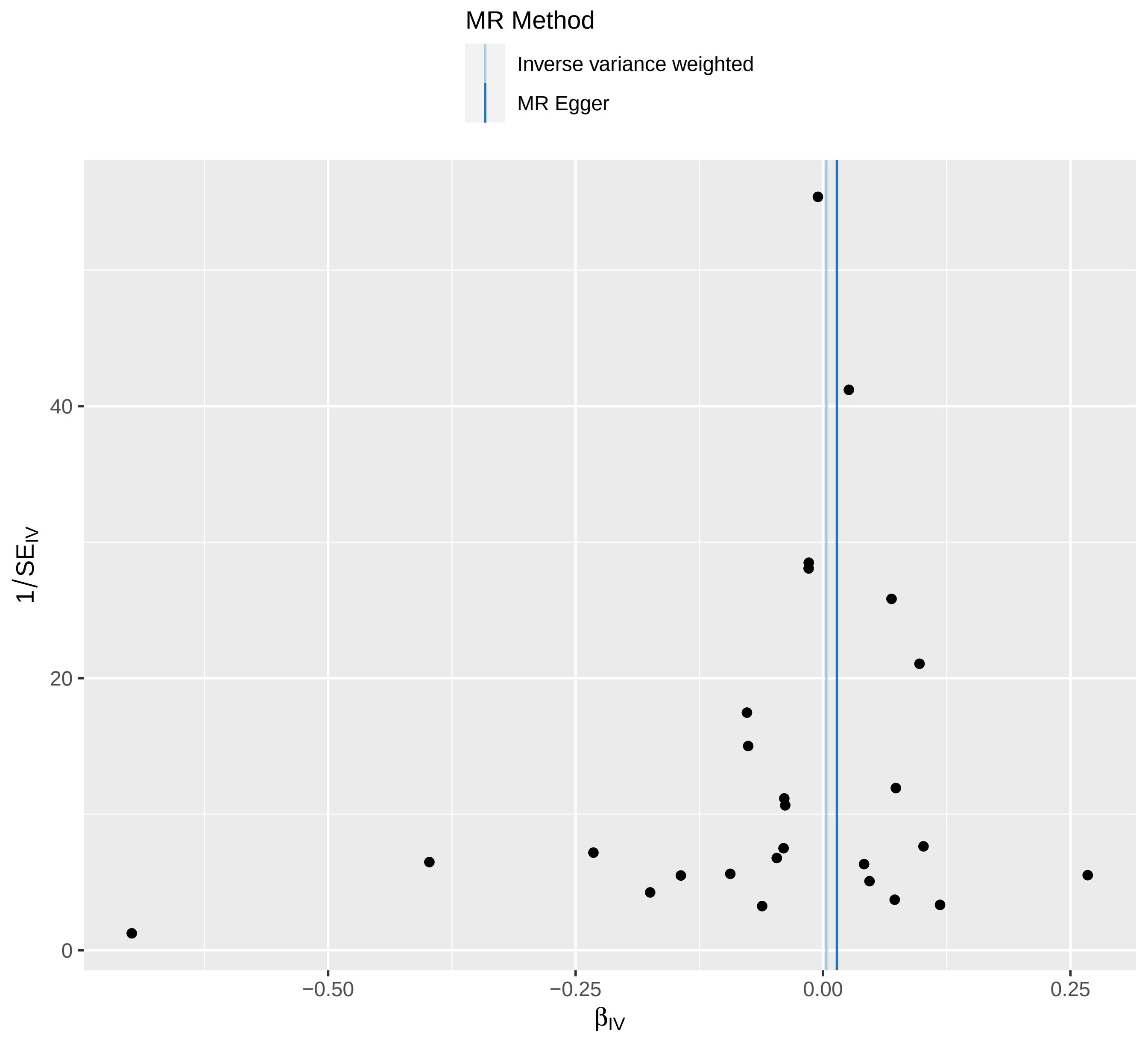

MR Method
Inverse variance weighted
MR Egger
40
20
0
−0.50
−0.25
0.00
0.25
$1/SE_{IV}$
β_{IV}

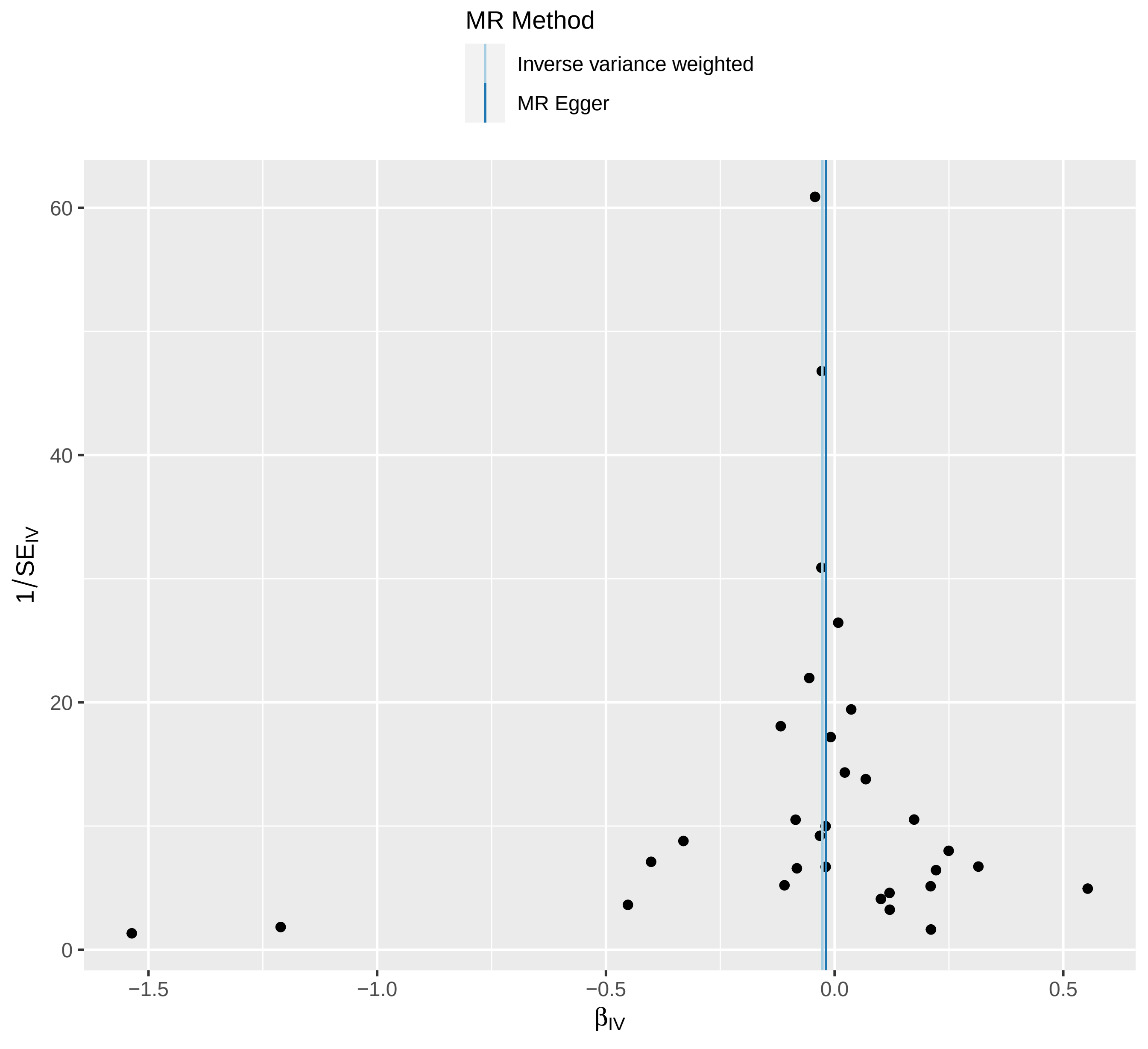

MR Method
Inverse variance weighted
MR Egger
60
40
20
0
$1/SE_{IV}$
−1.5
−1.0
−0.5
0.0
0.5
β_{IV}

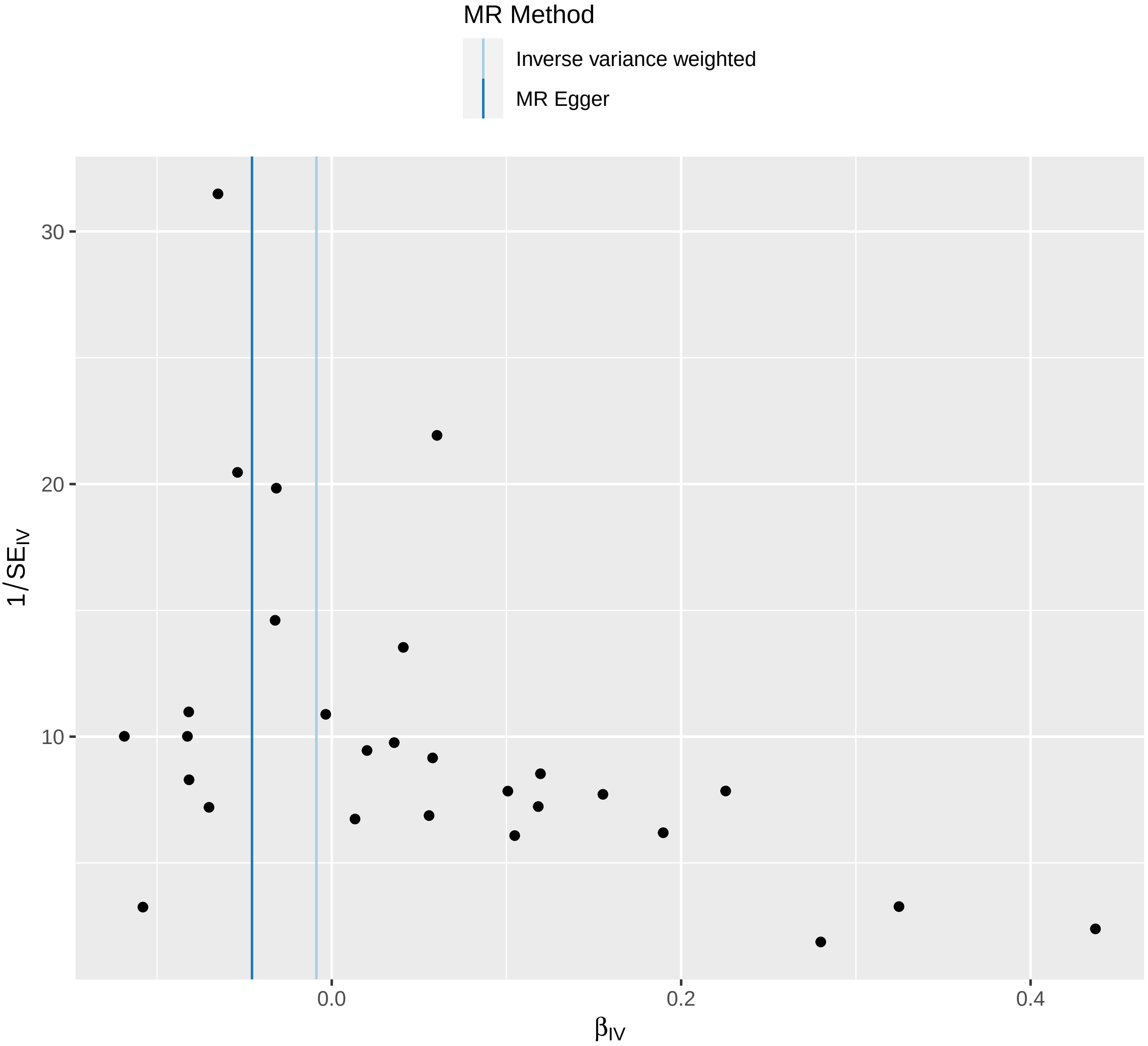

MR Method
Inverse variance weighted
MR Egger
30
20
10
1/SE$_{IV}$
0.0
0.2
0.4
β$_{IV}$

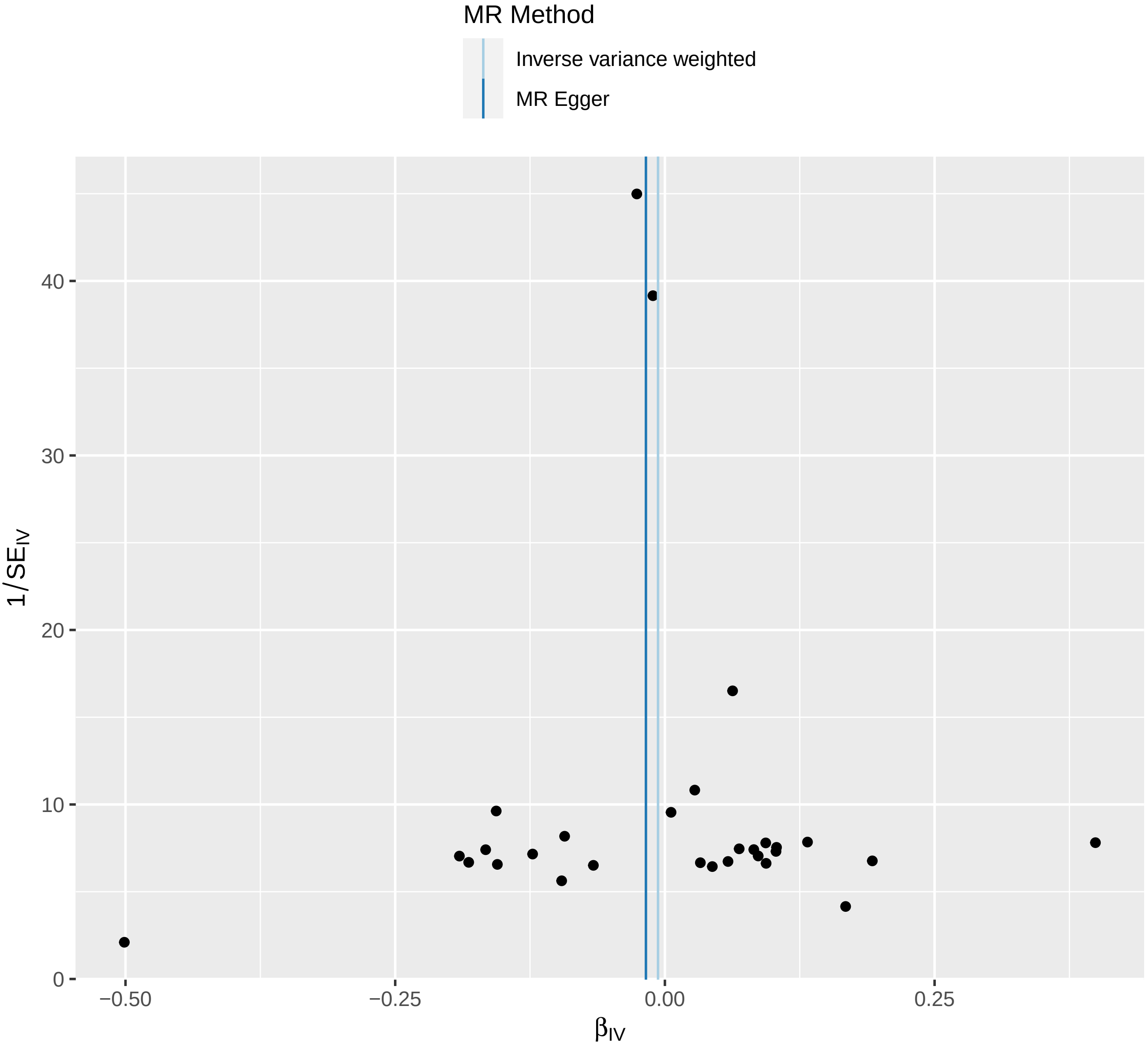

MR Method
Inverse variance weighted
MR Egger
40
30
20
10
0
1/SE_{IV}
−0.50
−0.25
0.00
0.25
β_{IV}

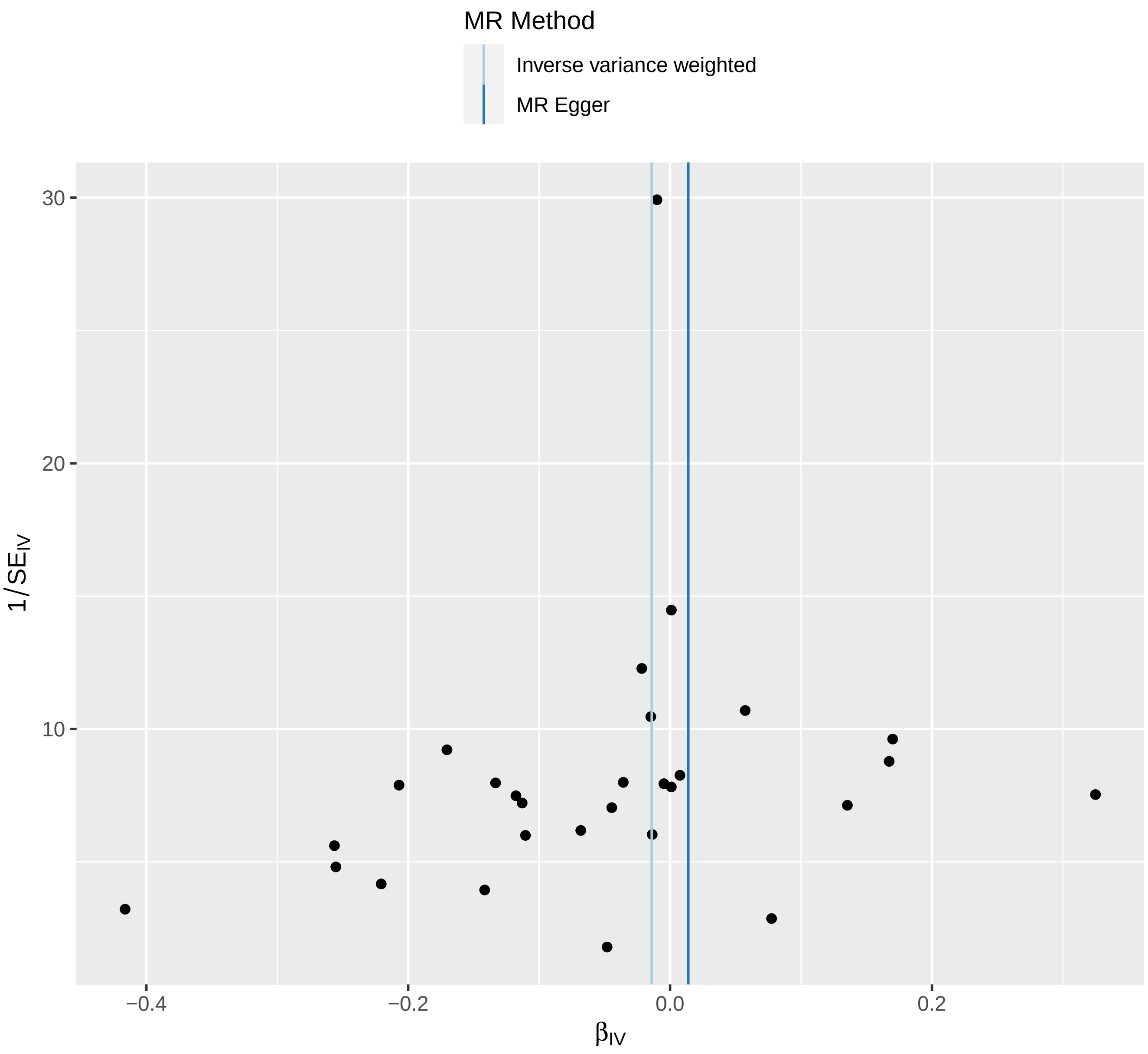
MR Method
Inverse variance weighted
MR Egger
30
20
10
−0.4
−0.2
0.0
0.2
$1/SE_{IV}$
β_{IV}

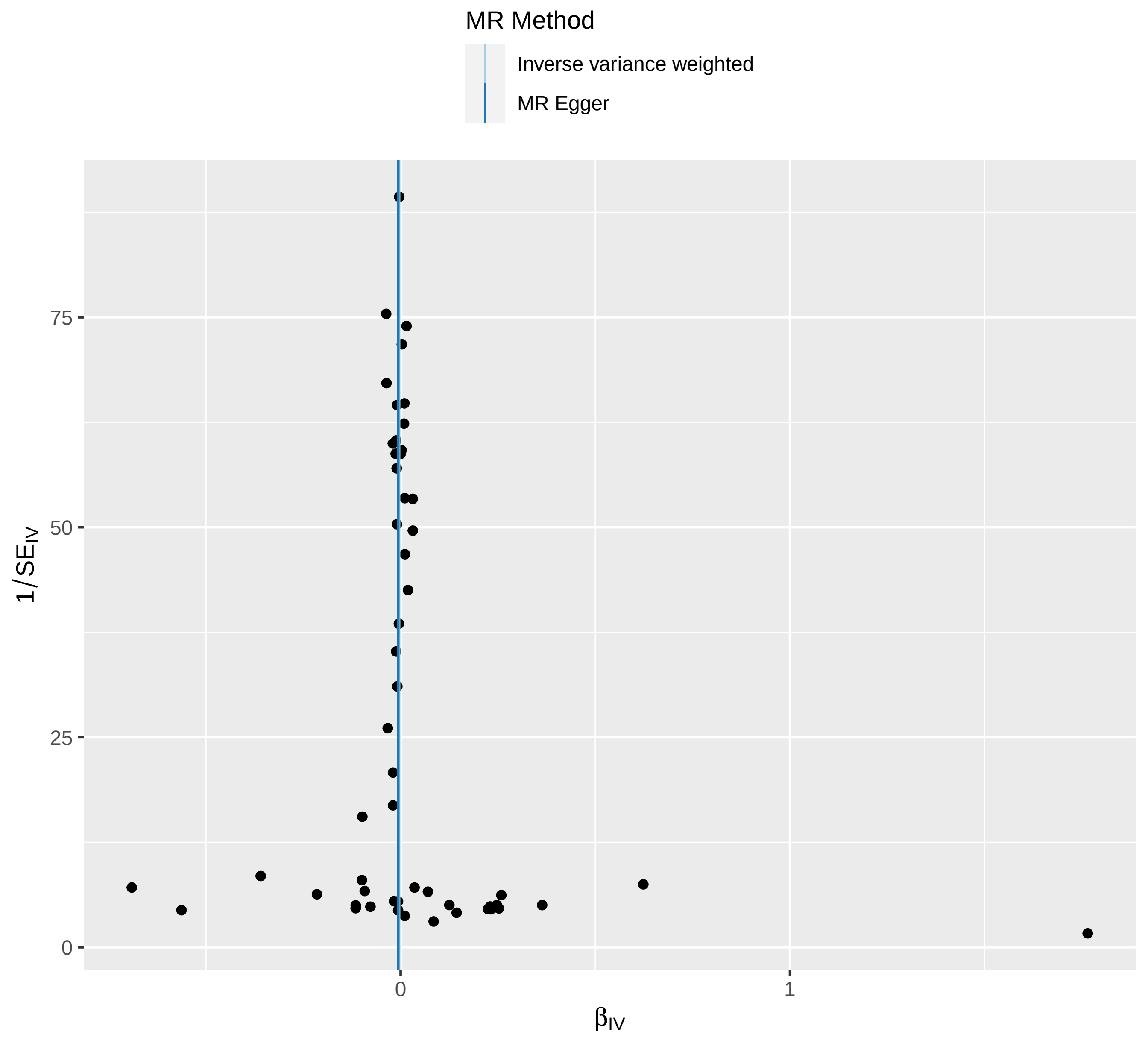

MR Method
Inverse variance weighted
MR Egger
75
50
25
0
1/SE$_{IV}$
0
1
β$_{IV}$

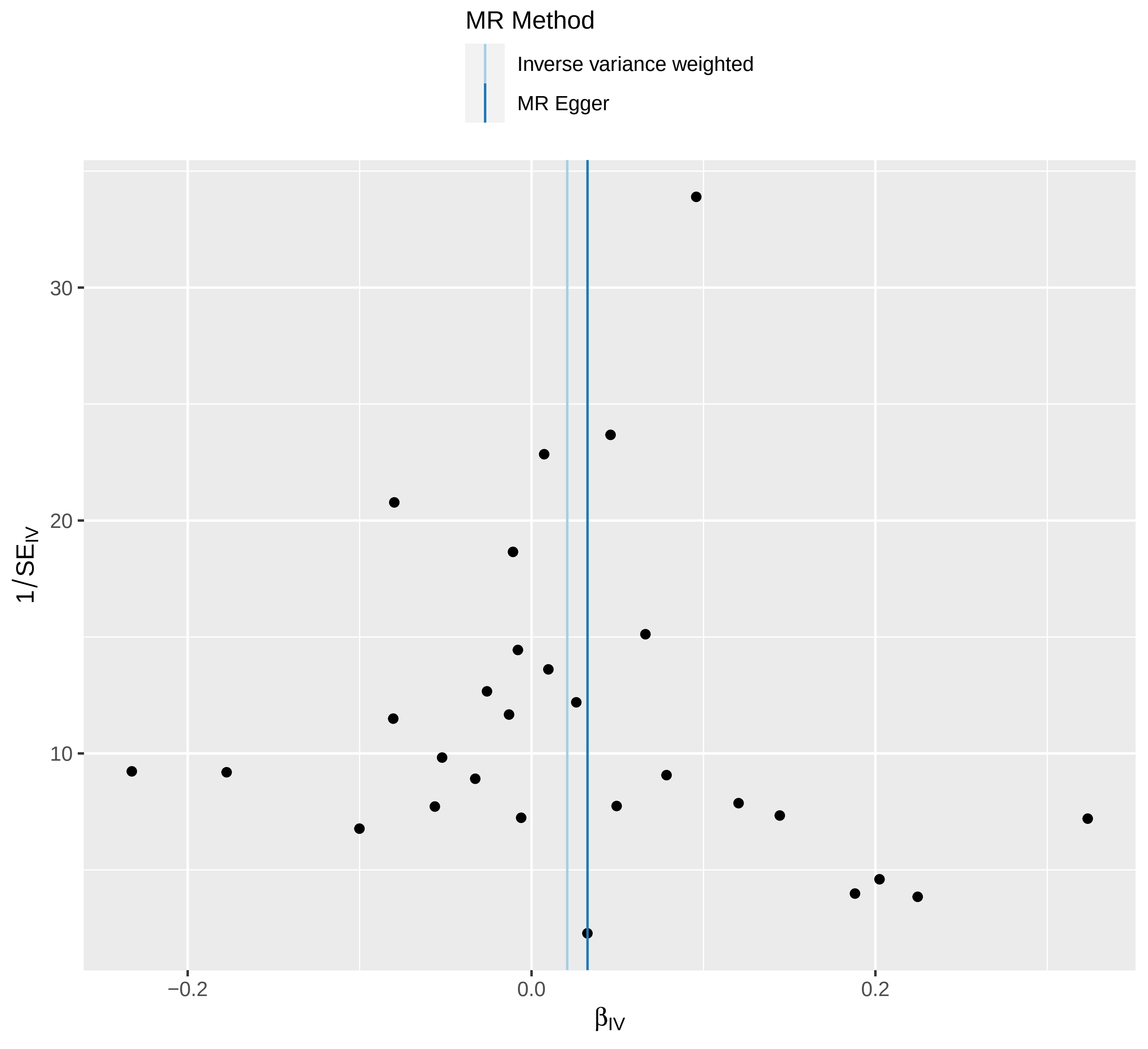

MR Method
Inverse variance weighted
MR Egger
30
20
10
1/SE$_{IV}$
−0.2
0.0
0.2
β_{IV}

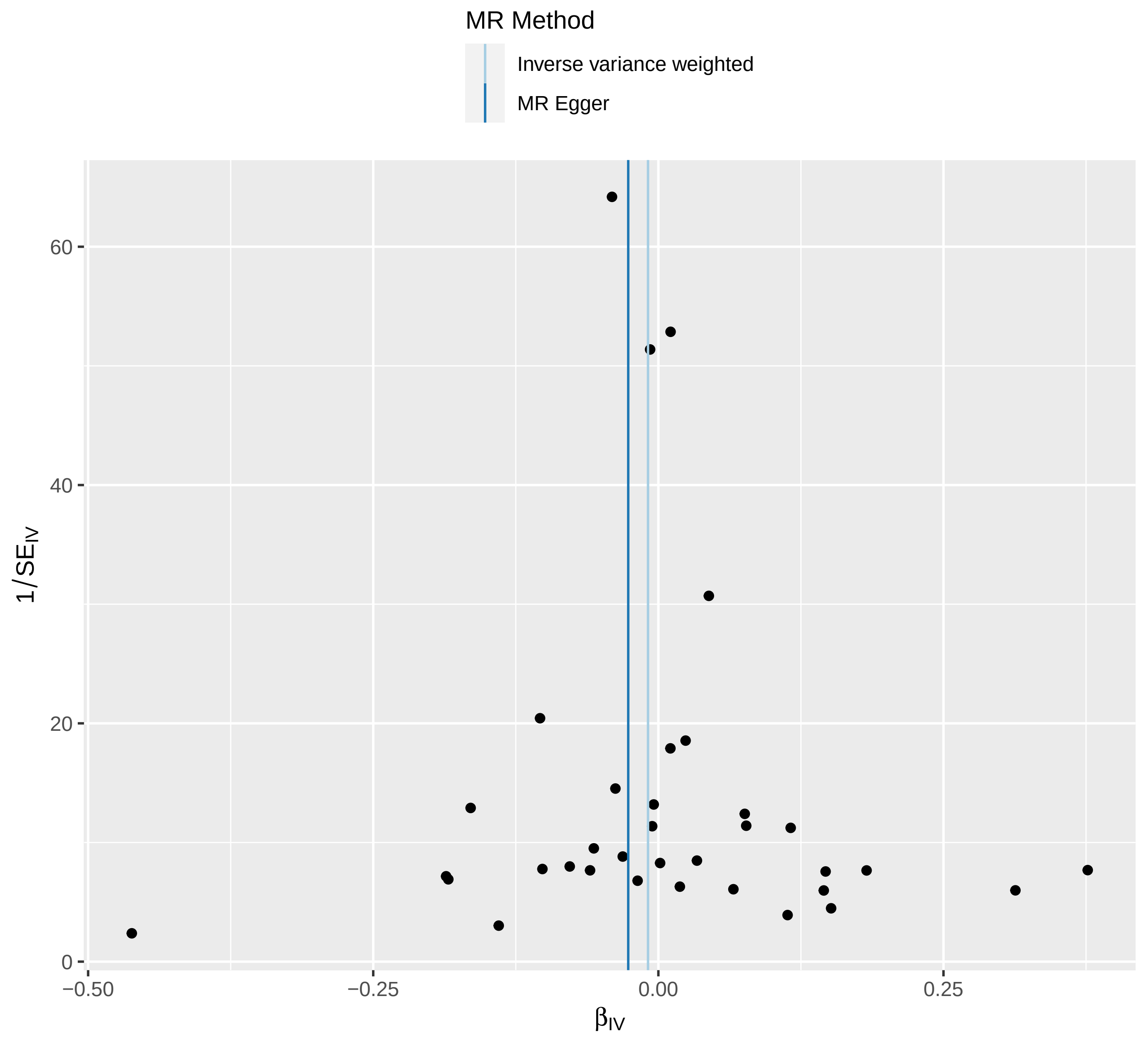

MR Method
Inverse variance weighted
MR Egger
60
40
20
0
1/SE$_{IV}$
−0.50
−0.25
0.00
0.25
β$_{IV}$

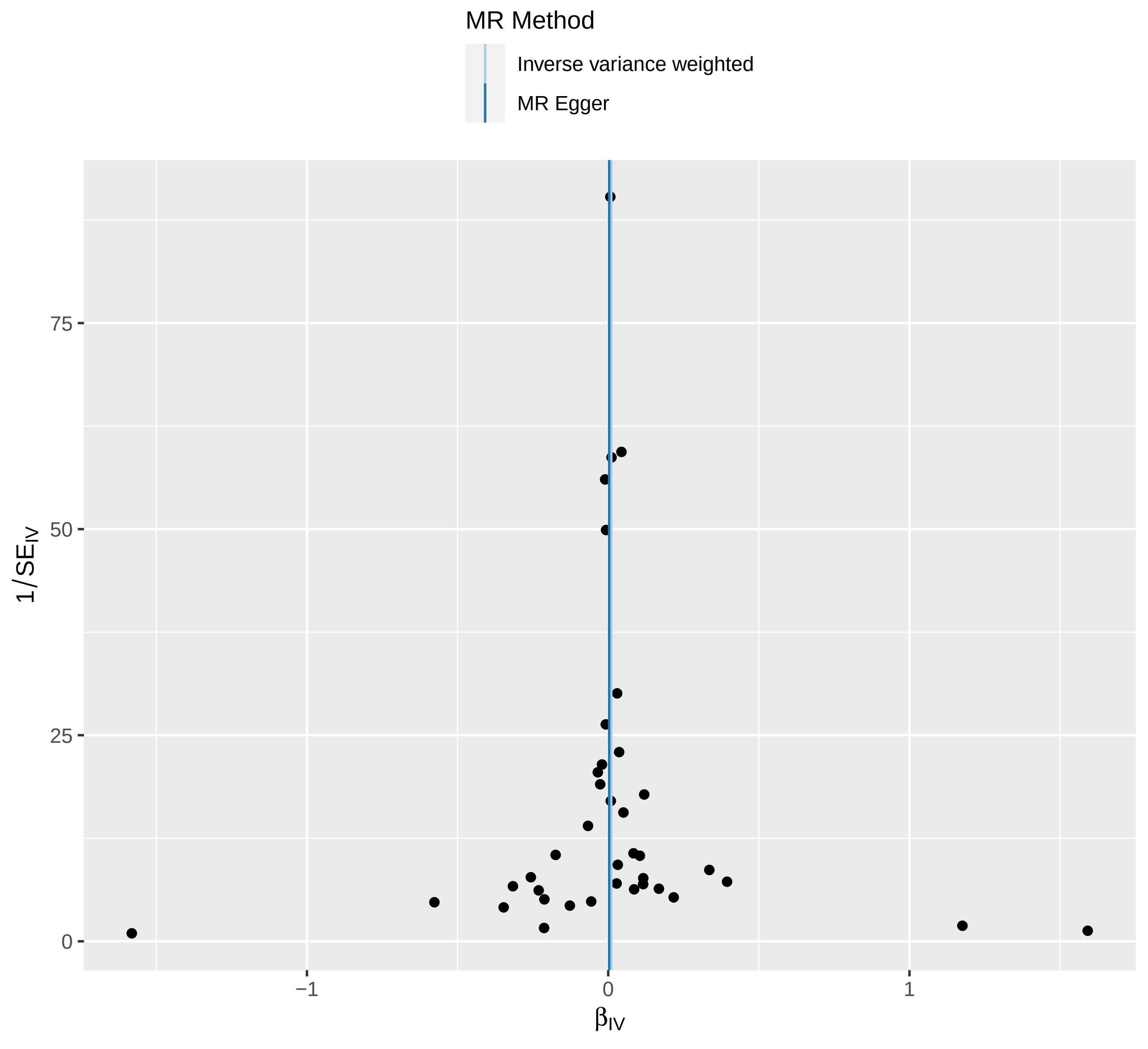

MR Method
Inverse variance weighted
MR Egger
1/SE_IV
75
50
25
0
−1
0
1
β_IV

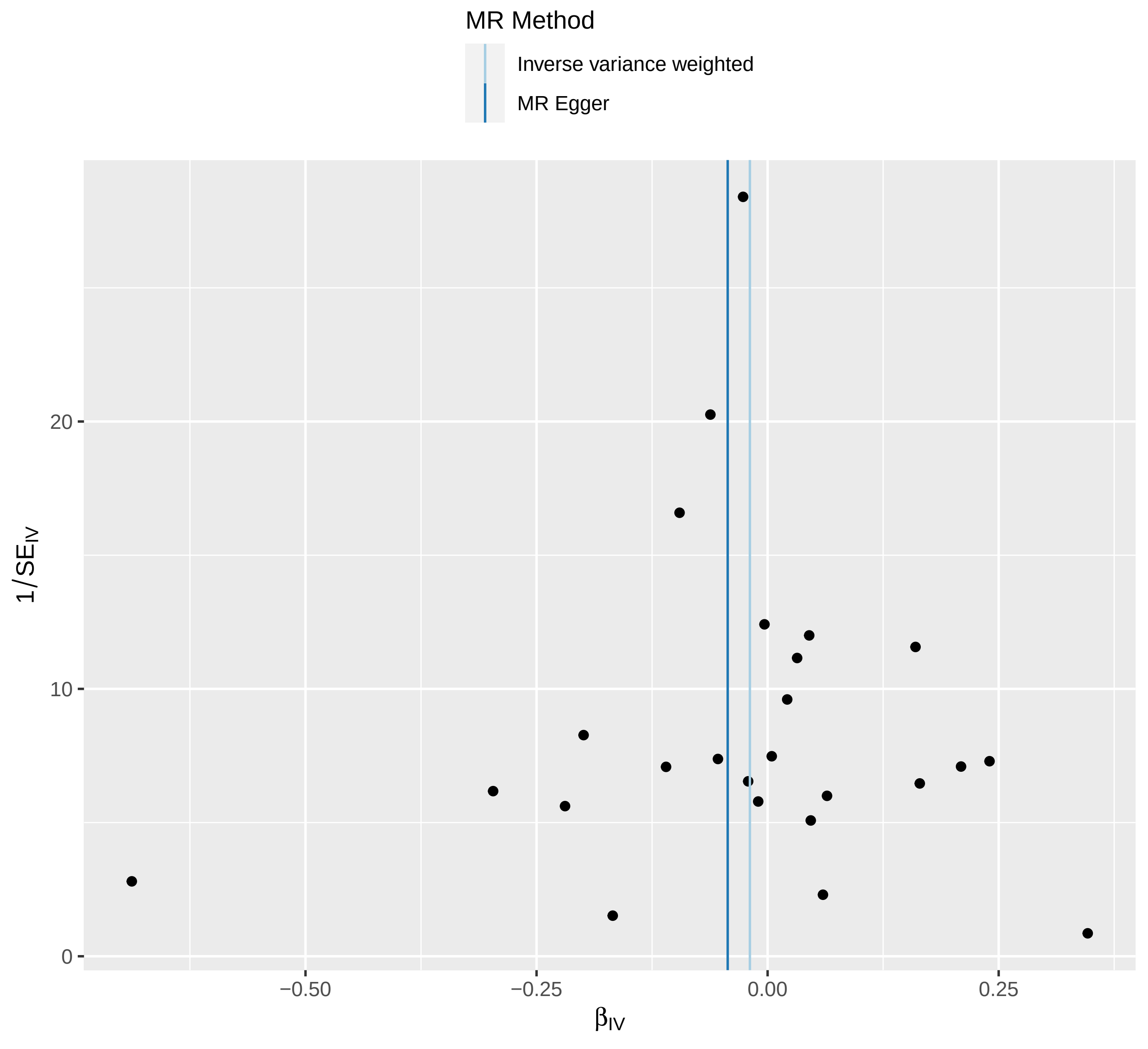

MR Method
Inverse variance weighted
MR Egger
1/SE_IV
20
10
0
−0.50
−0.25
0.00
0.25
β_IV

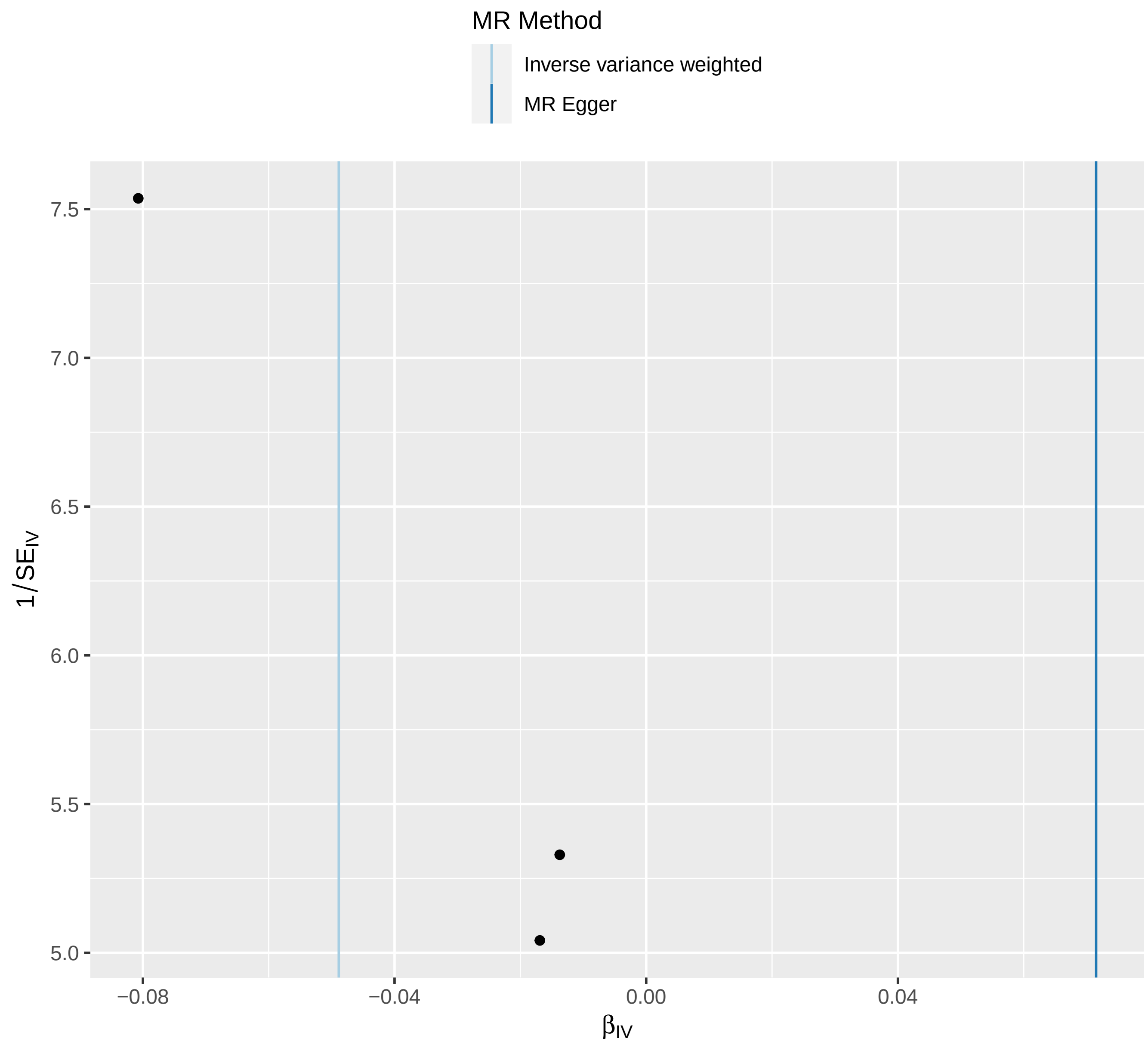

MR Method
Inverse variance weighted
MR Egger
7.5
7.0
6.5
6.0
5.5
5.0
1/SE_IV
−0.08
−0.04
0.00
0.04
β_IV

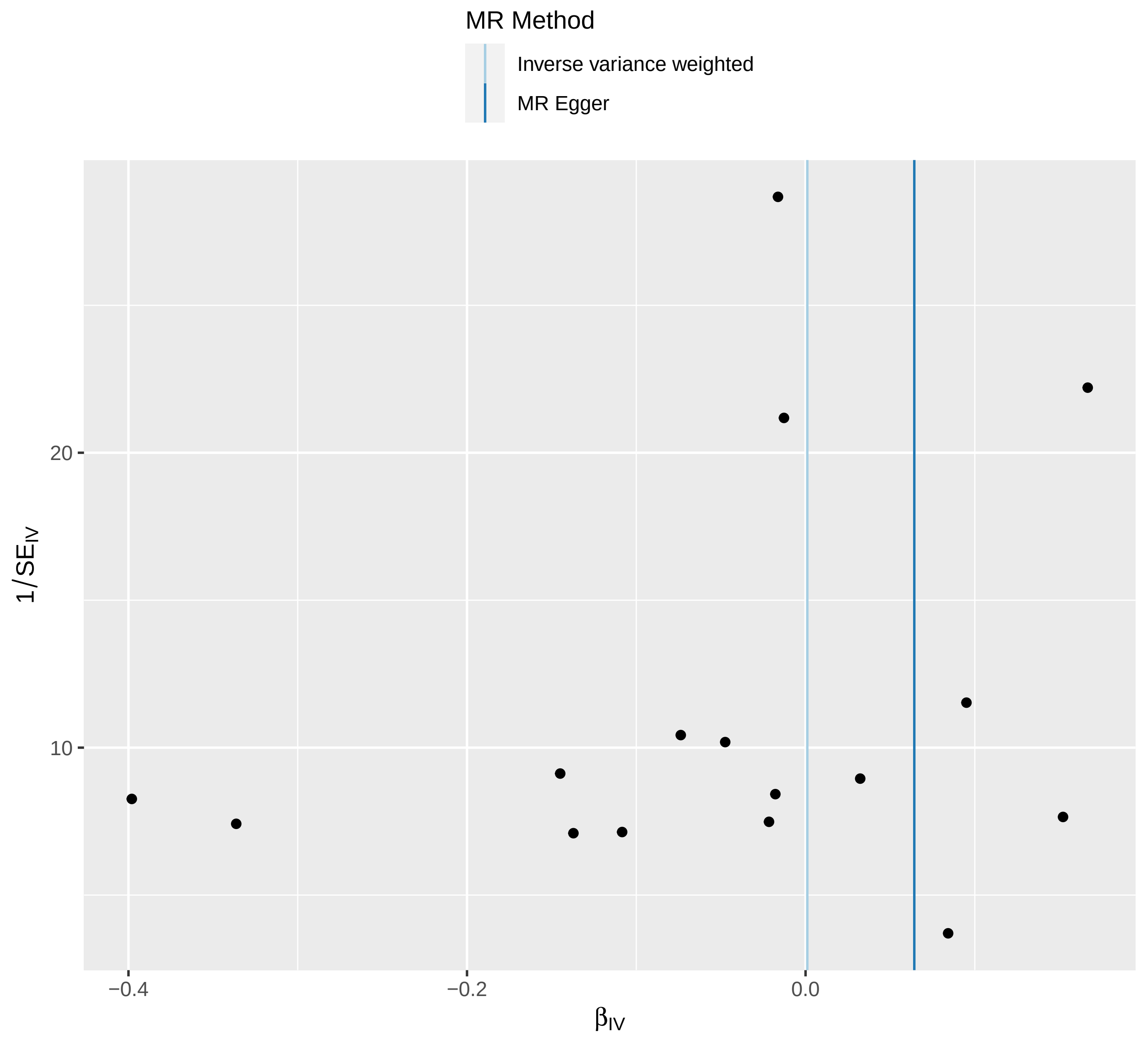

MR Method
Inverse variance weighted
MR Egger
20
10
1/SE$_{IV}$
−0.4
−0.2
0.0
β$_{IV}$

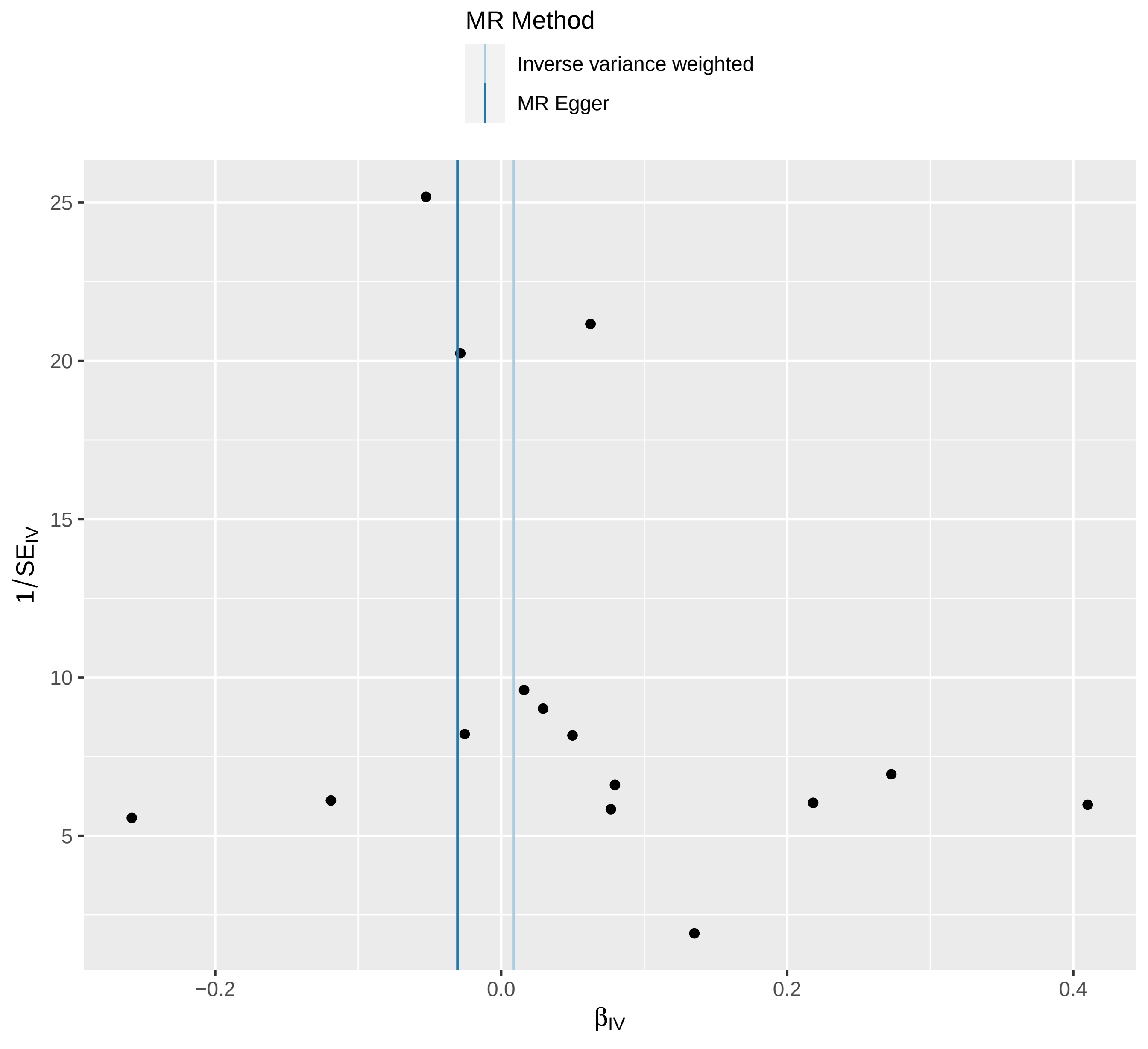
MR Method
Inverse variance weighted
MR Egger
25
20
15
10
5
1/SE_IV
−0.2
0.0
0.2
0.4
β_IV

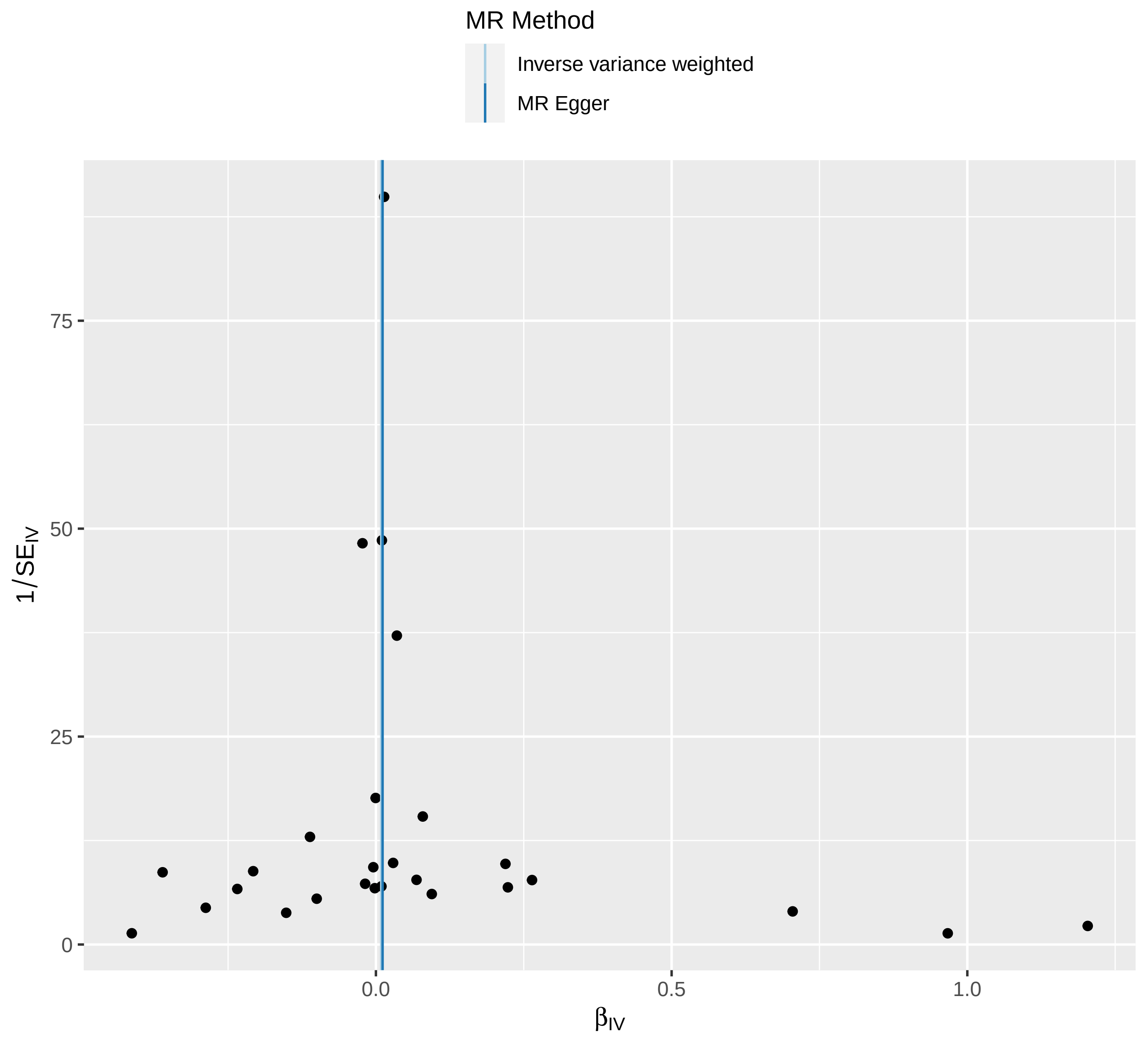

MR Method
Inverse variance weighted
MR Egger
1/SE_IV
0
25
50
75
β_IV
0.0
0.5
1.0

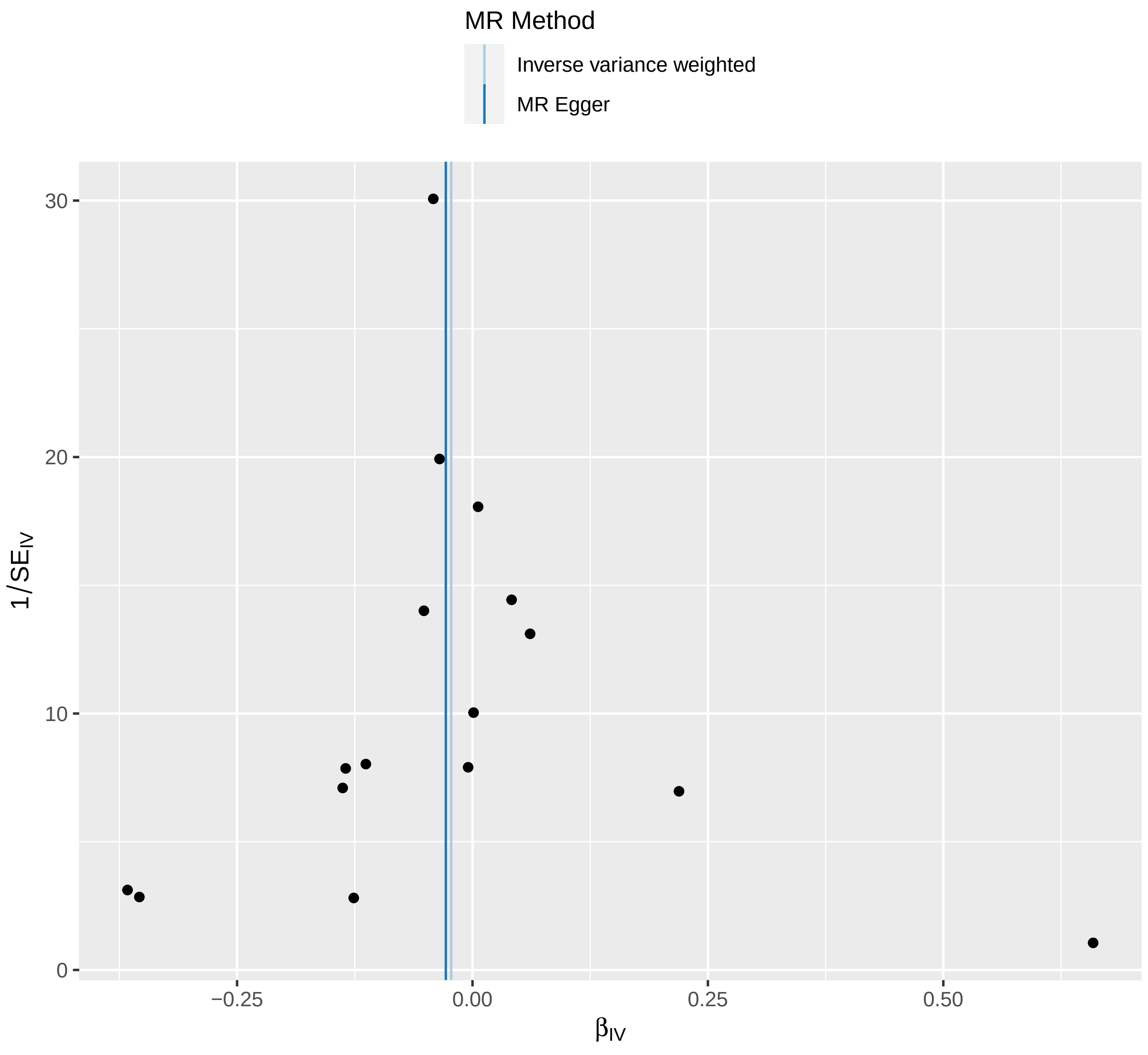

MR Method
Inverse variance weighted
MR Egger
30
20
10
0
1/SE_IV
−0.25
0.00
0.25
0.50
β_IV

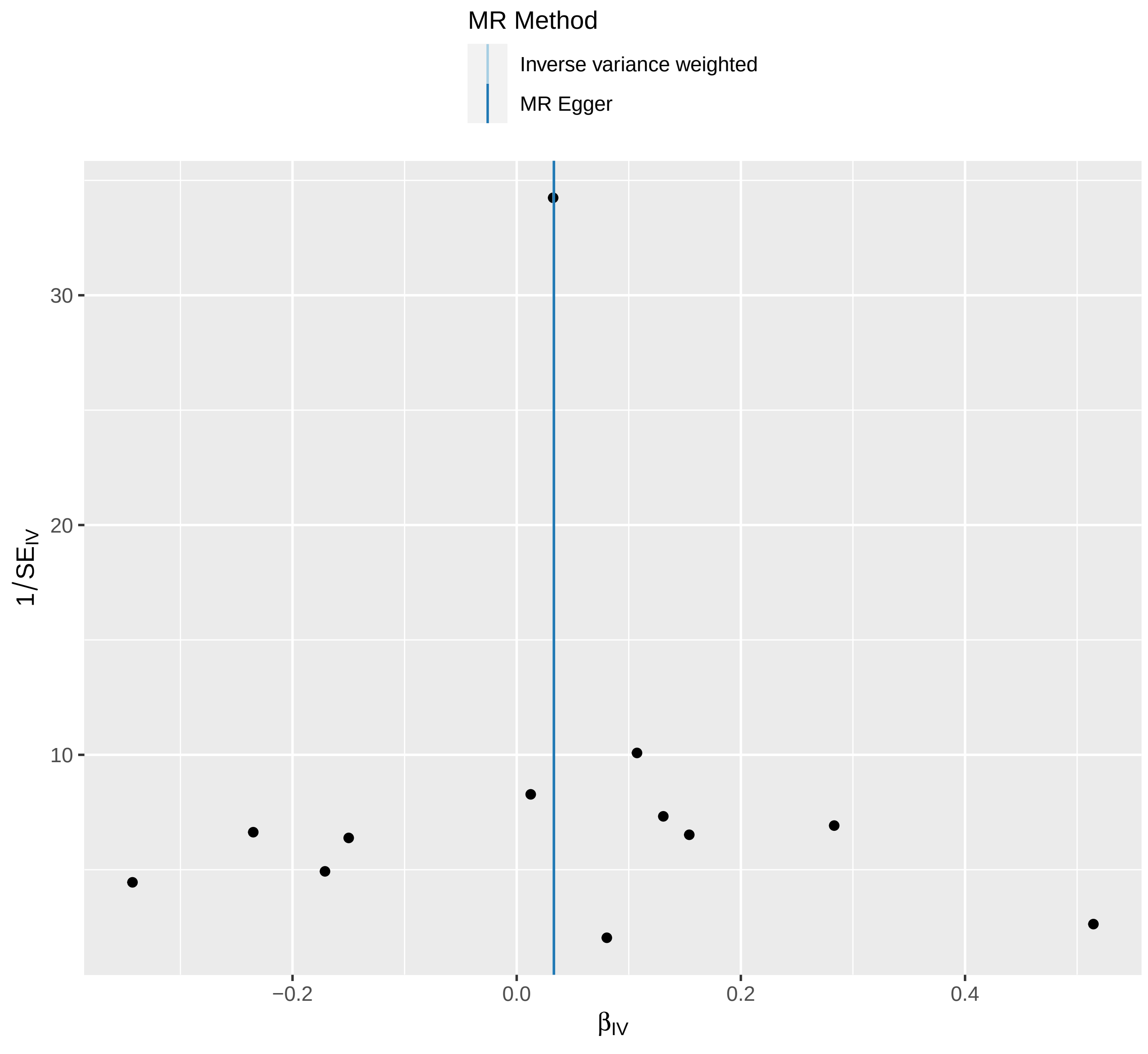
MR Method
Inverse variance weighted
MR Egger
30
20
10
1/SE$_{IV}$
−0.2
0.0
0.2
0.4
β$_{IV}$

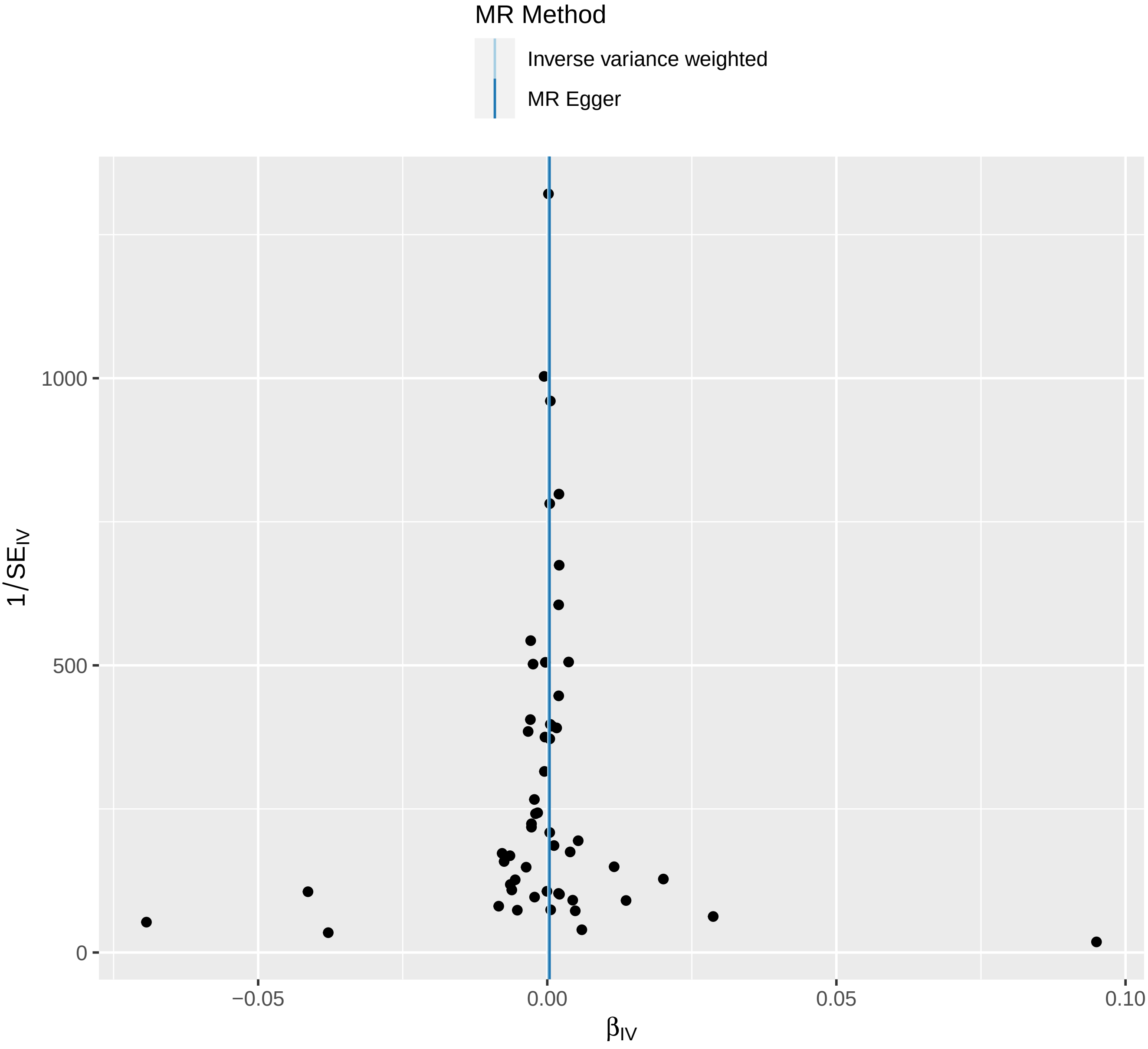
MR Method
Inverse variance weighted
MR Egger
1000
500
0
−0.05
0.00
0.05
0.10
β_{IV}
$1/SE_{IV}$

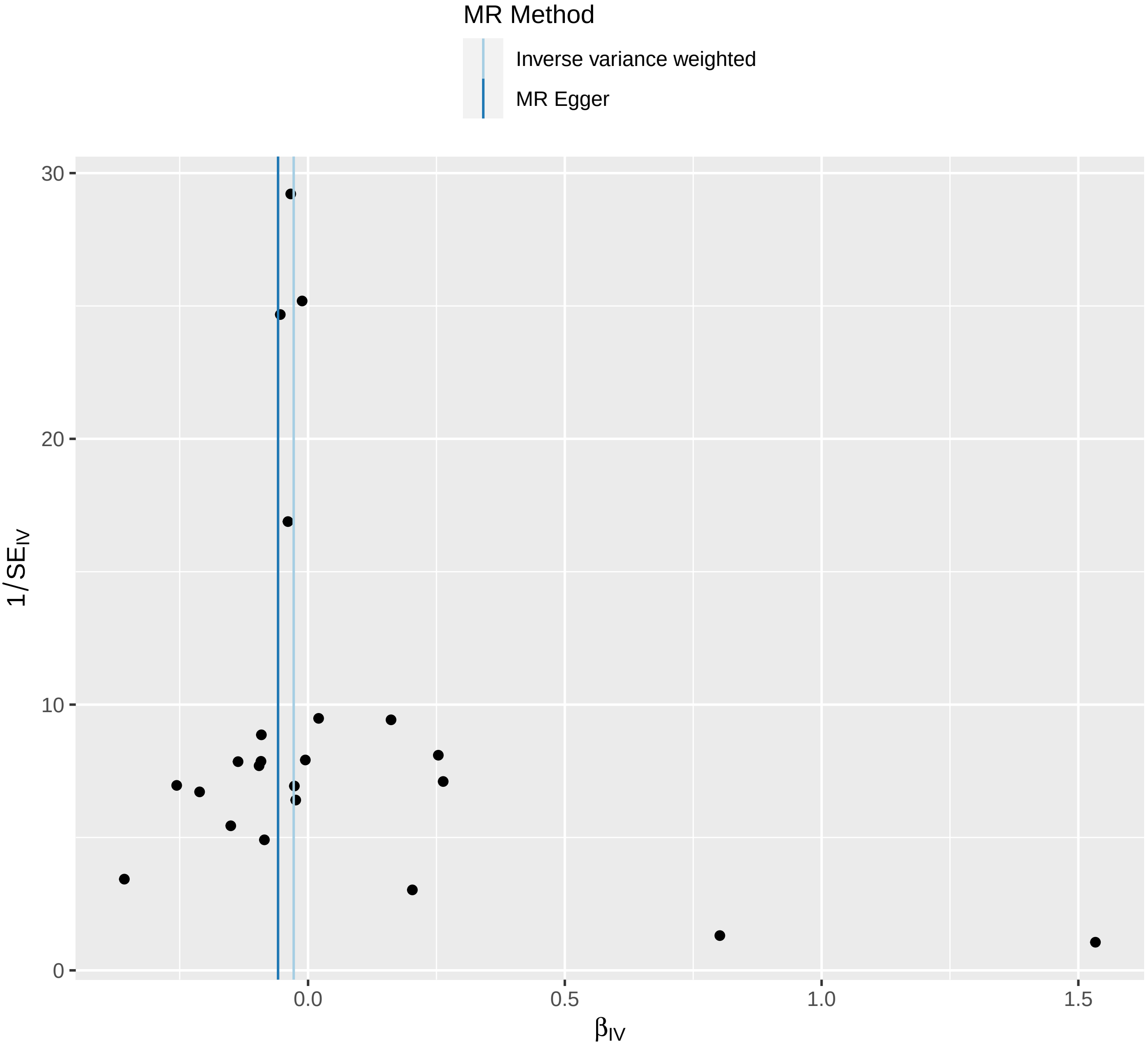
MR Method
Inverse variance weighted
MR Egger
30
20
10
0
0.0
0.5
1.0
1.5
1/SE_IV
β_IV

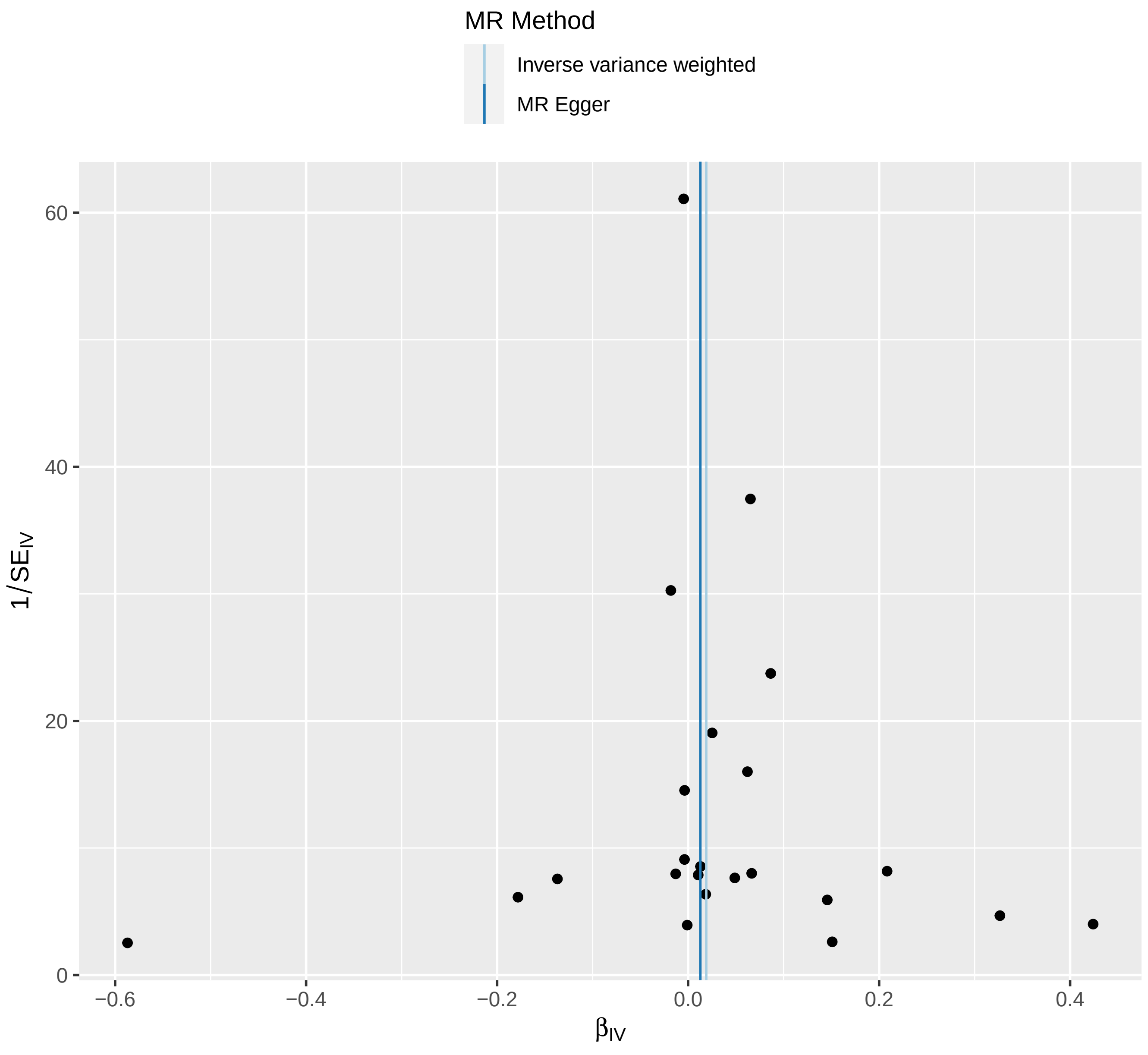

MR Method
Inverse variance weighted
MR Egger
60
40
20
0
1/SE_IV
−0.6
−0.4
−0.2
0.0
0.2
0.4
β_IV

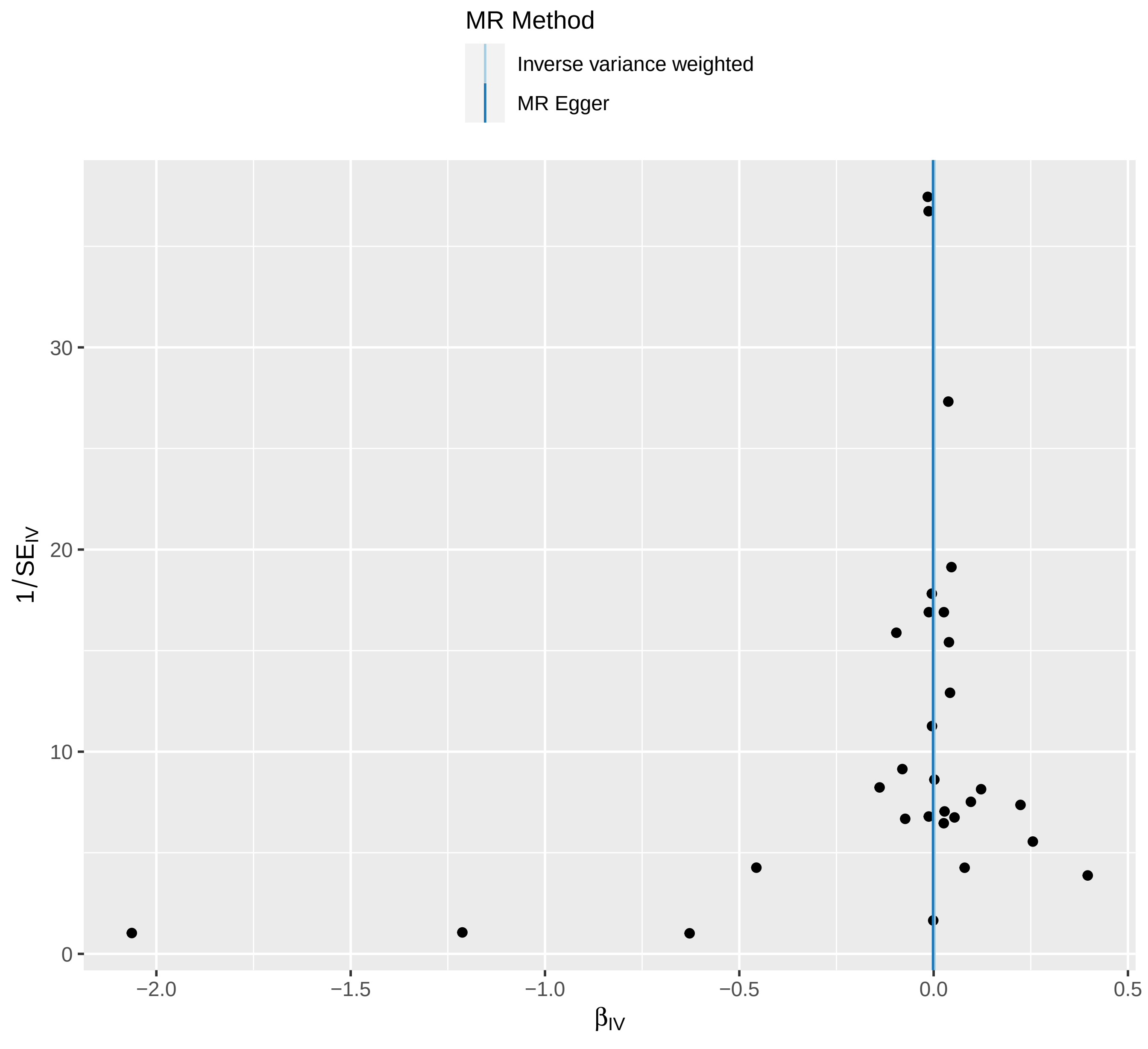

MR Method
Inverse variance weighted
MR Egger
1/SE_IV
β_IV
30
20
10
0
−2.0
−1.5
−1.0
−0.5
0.0
0.5

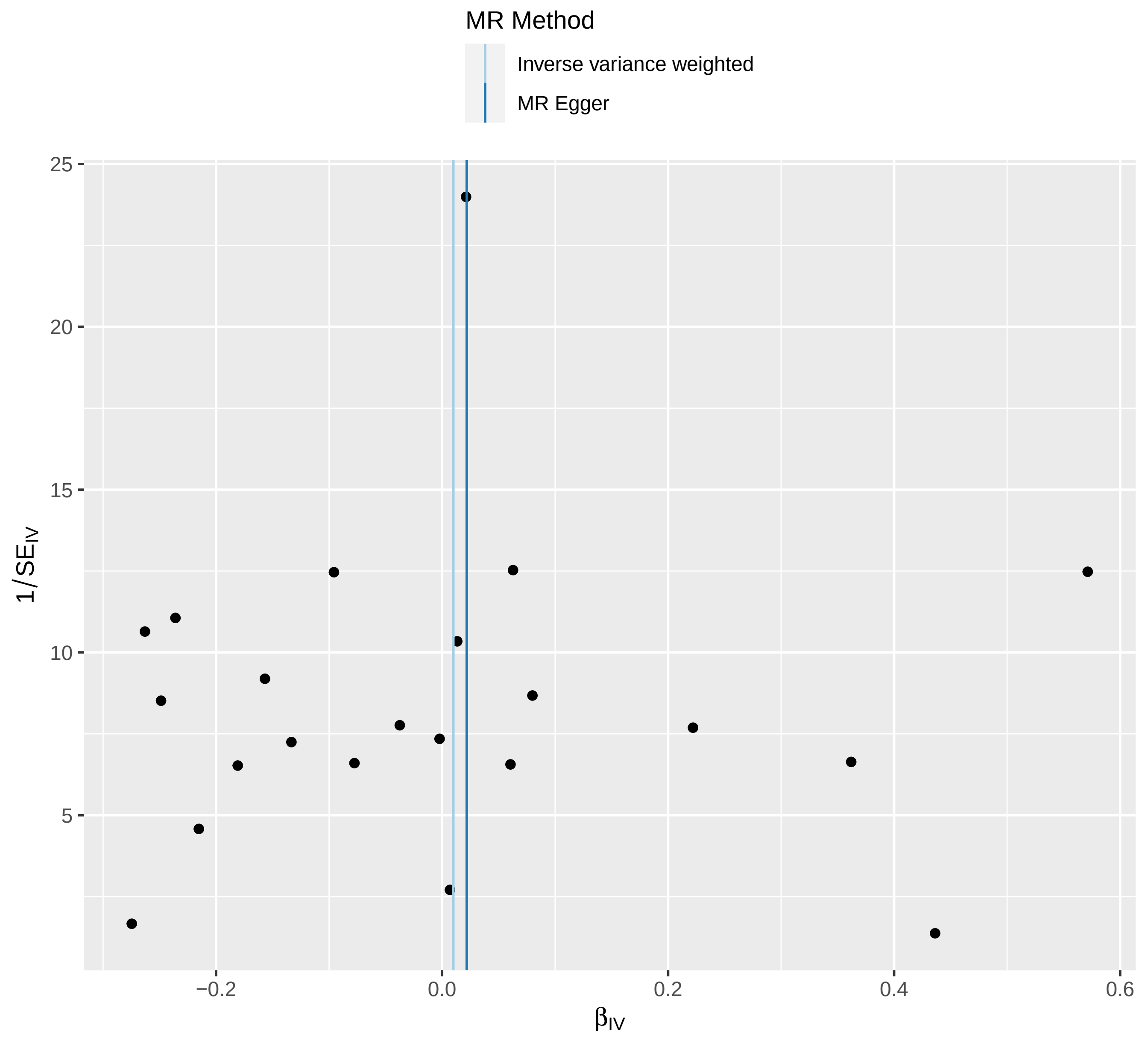

MR Method
Inverse variance weighted
MR Egger
25
20
15
10
5
1/SE_IV
−0.2
0.0
0.2
0.4
0.6
β_IV

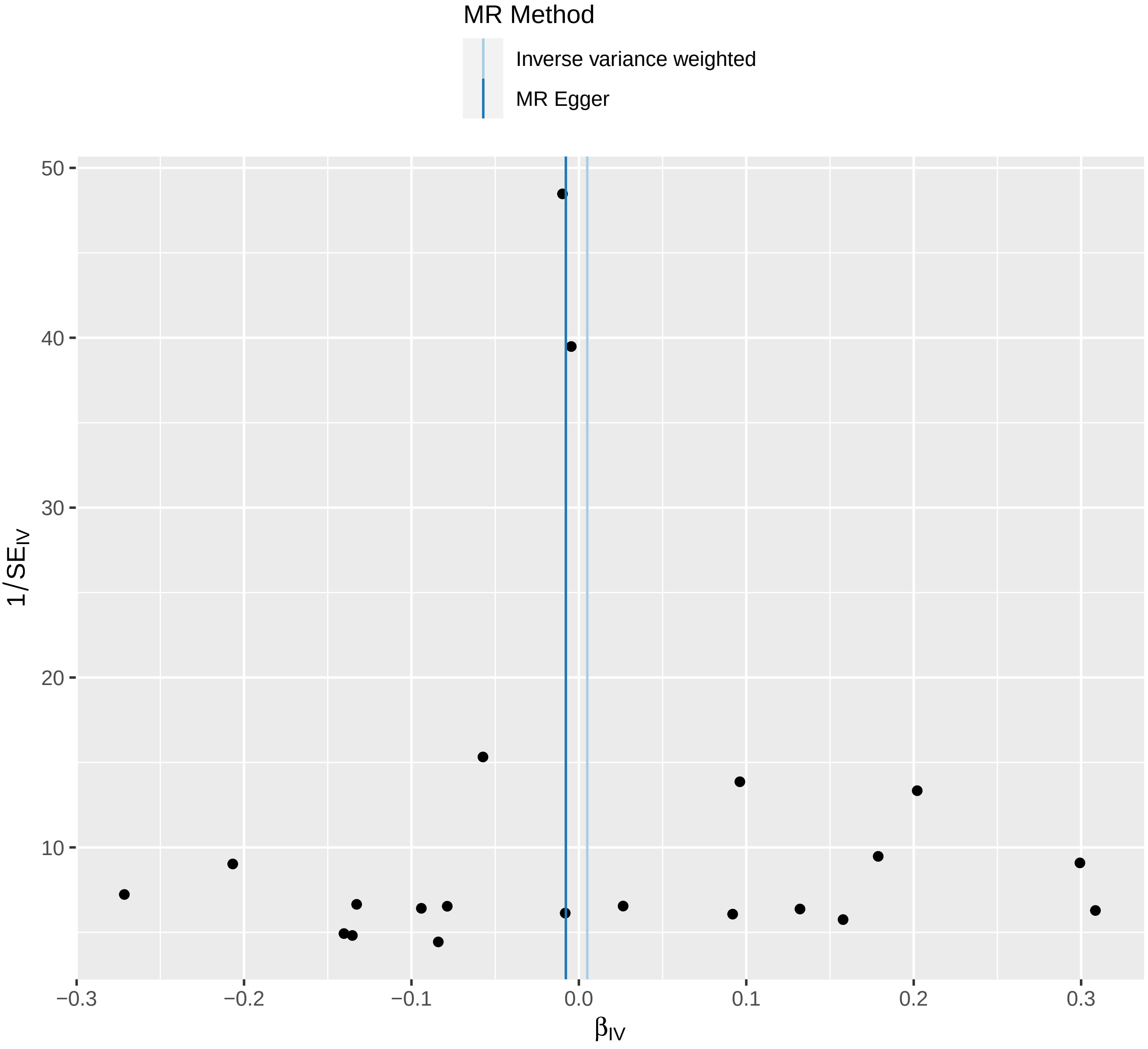

MR Method
Inverse variance weighted
MR Egger
50
40
30
20
10
1/SE_IV
−0.3
−0.2
−0.1
0.0
0.1
0.2
0.3
β_IV

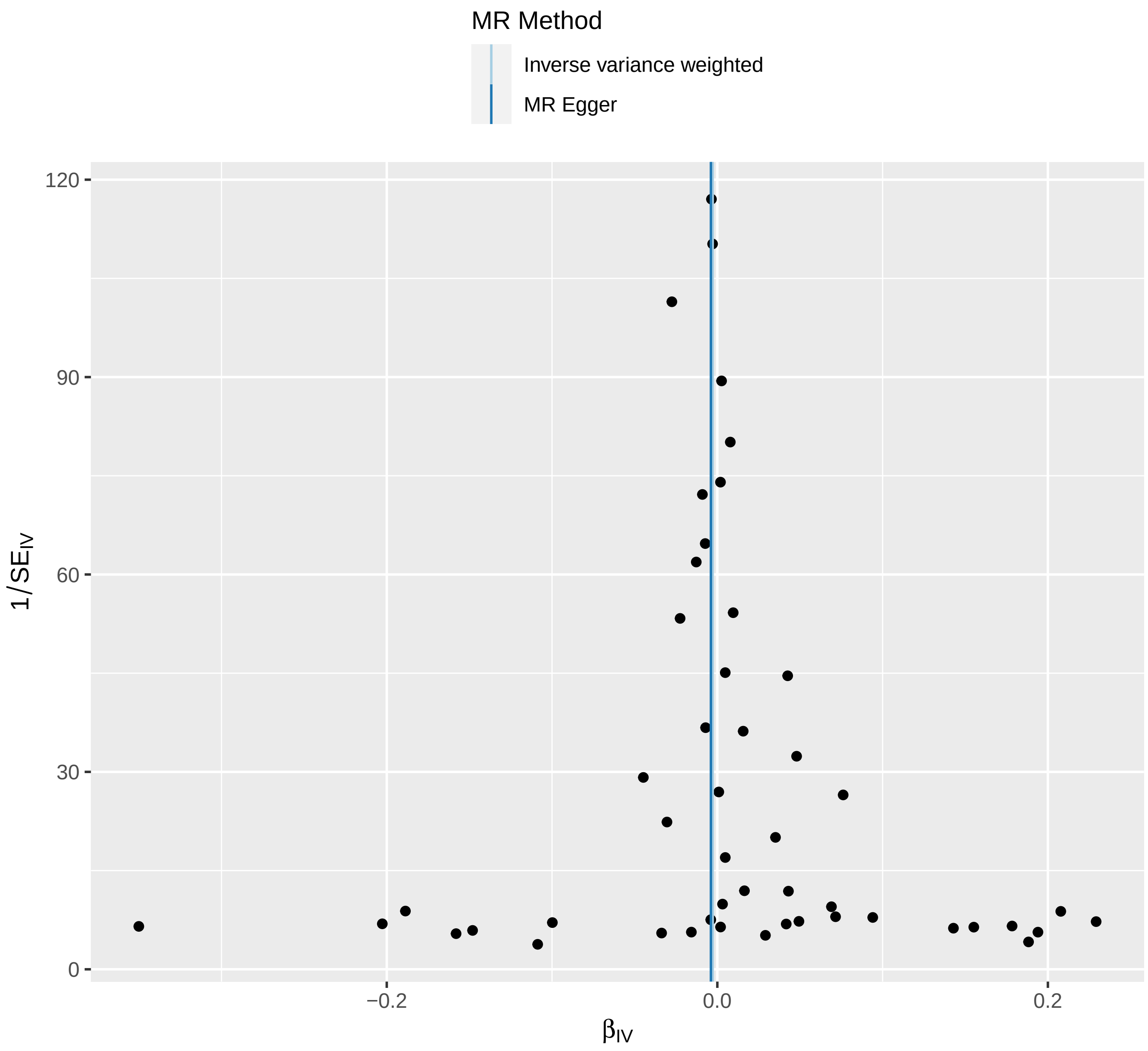

MR Method
Inverse variance weighted
MR Egger
1/SE_IV
120
90
60
30
0
−0.2
0.0
0.2
β_IV

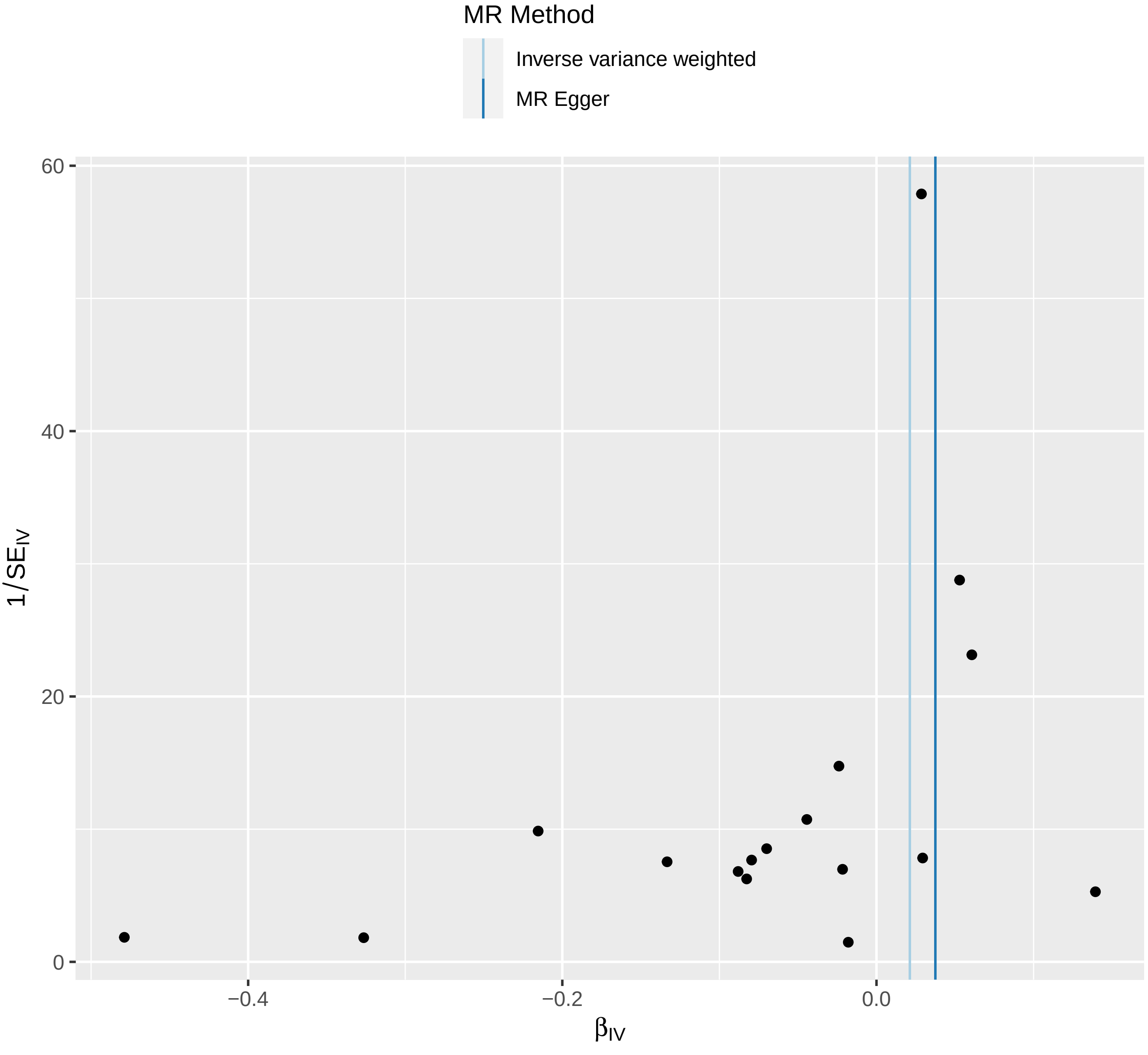
MR Method
Inverse variance weighted
MR Egger
60
40
20
0
1/SE_IV
−0.4
−0.2
0.0
β_IV

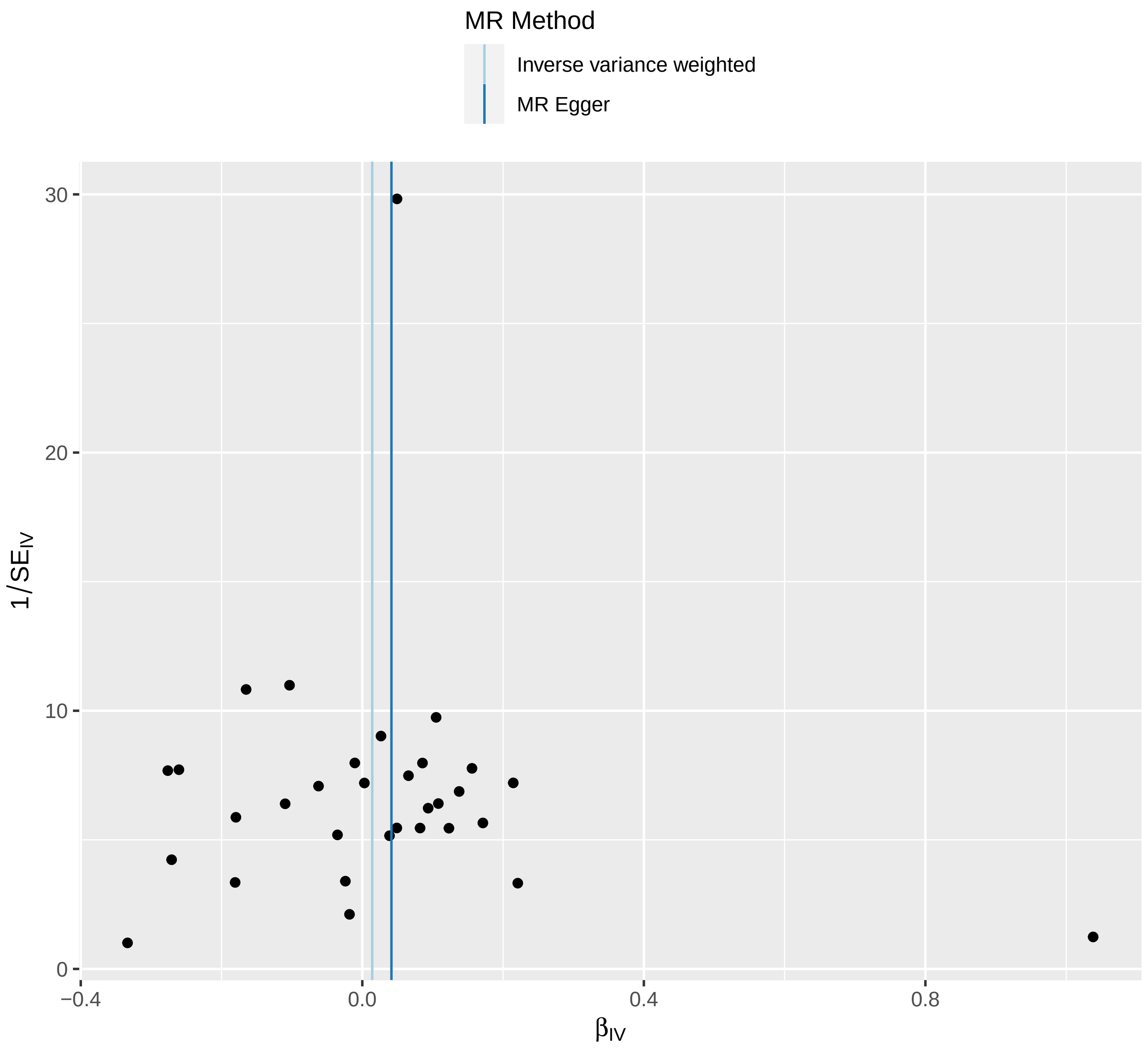

MR Method
Inverse variance weighted
MR Egger
$1/SE_{IV}$
0
10
20
30
−0.4
0.0
0.4
0.8
β_{IV}

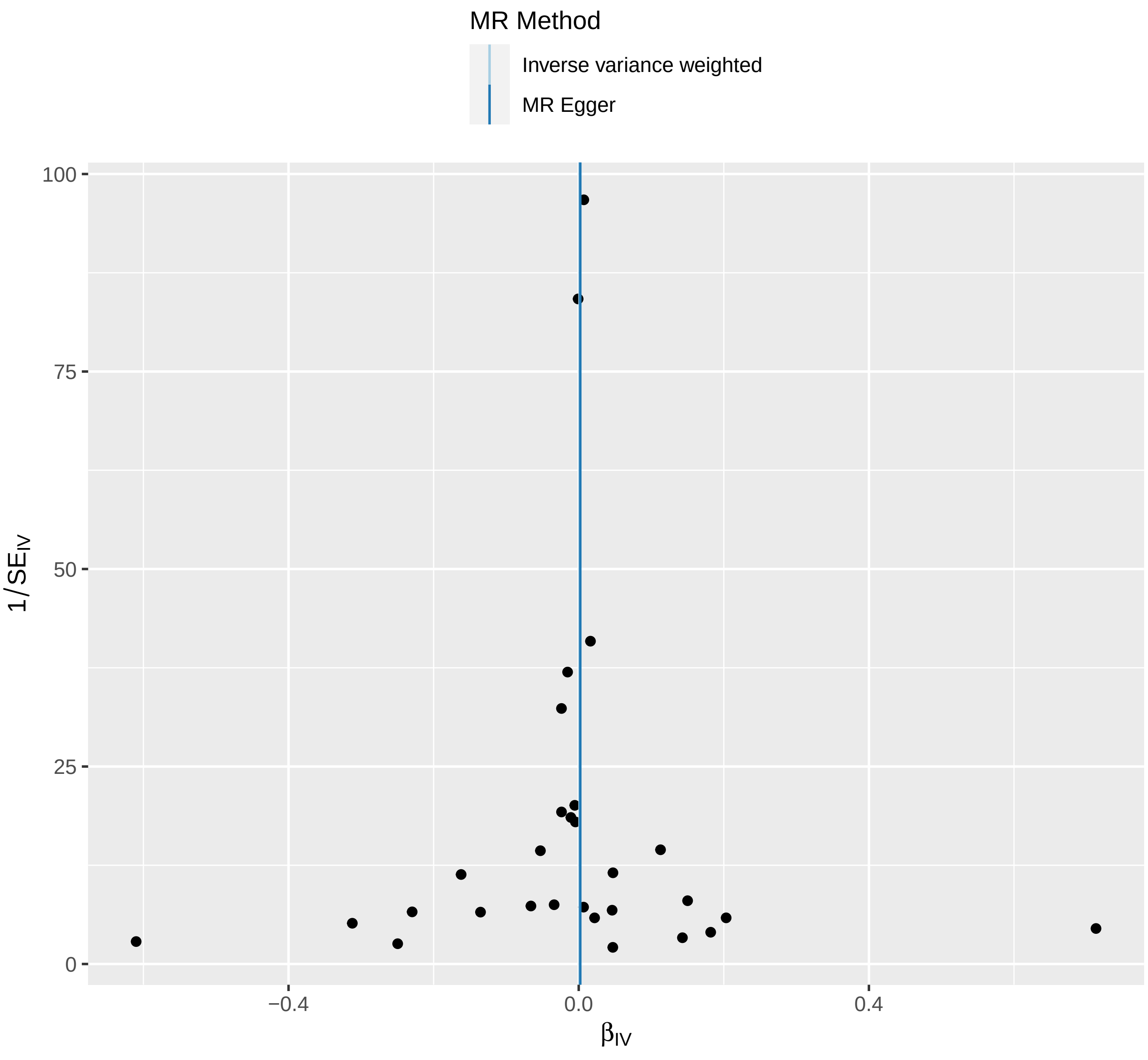
MR Method
Inverse variance weighted
MR Egger
100
75
50
25
0
1/SE_IV
−0.4
0.0
0.4
β_IV

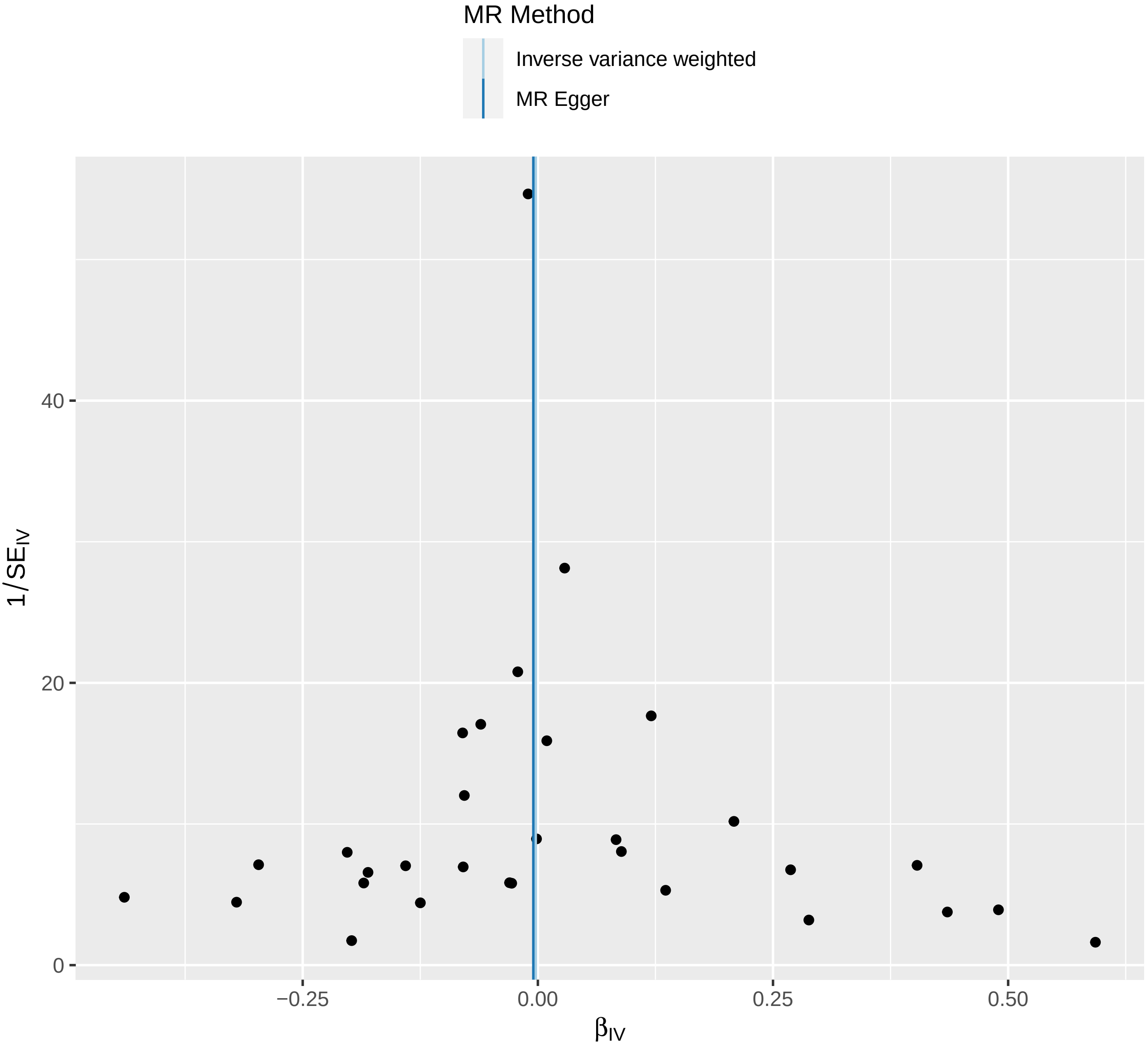
MR Method
Inverse variance weighted
MR Egger
1/SE$_{IV}$
β_{IV}
0
20
40
−0.25
0.00
0.25
0.50

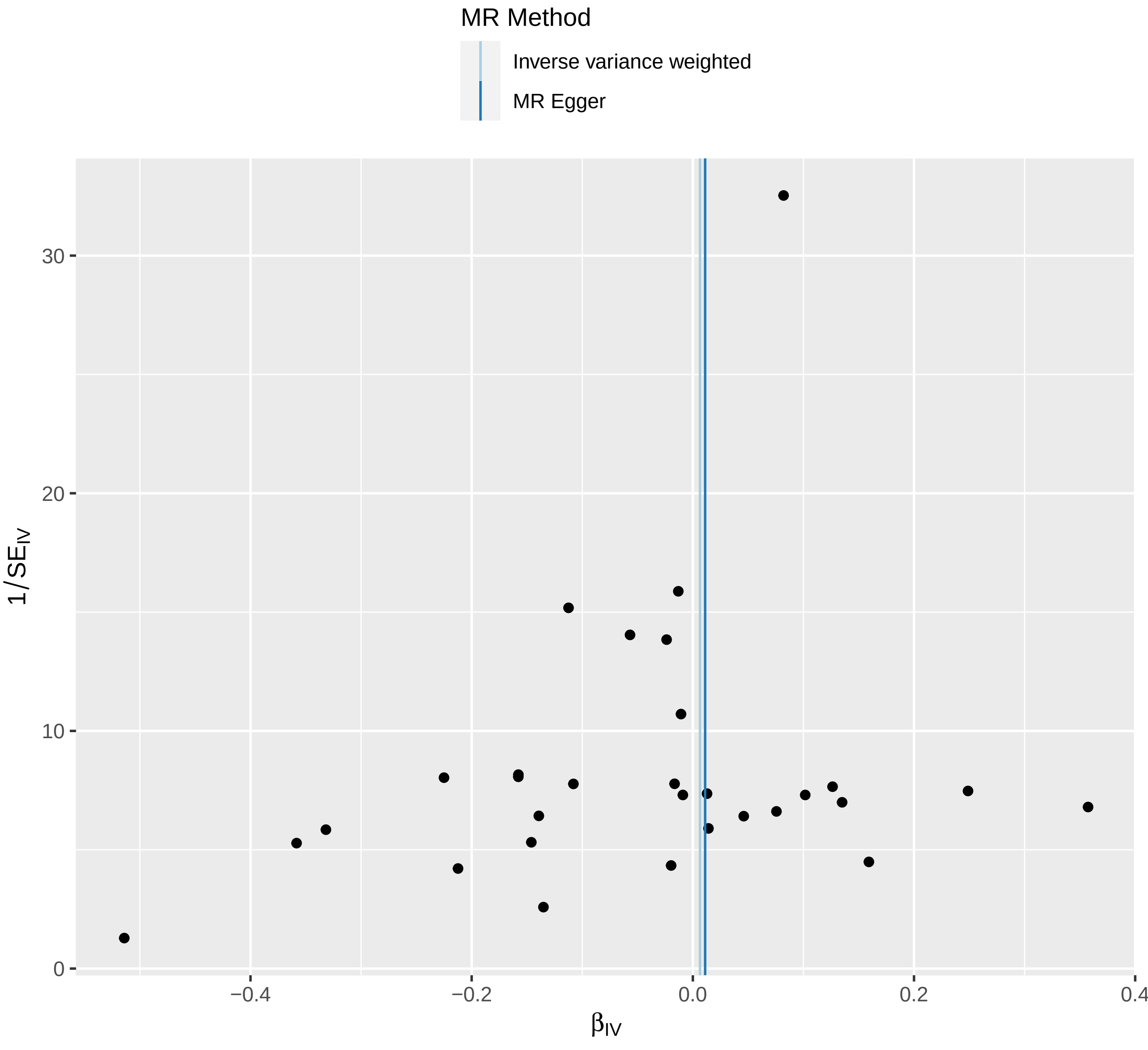
MR Method
Inverse variance weighted
MR Egger
30
20
10
0
−0.4
−0.2
0.0
0.2
0.4
$1/SE_{IV}$
β_{IV}

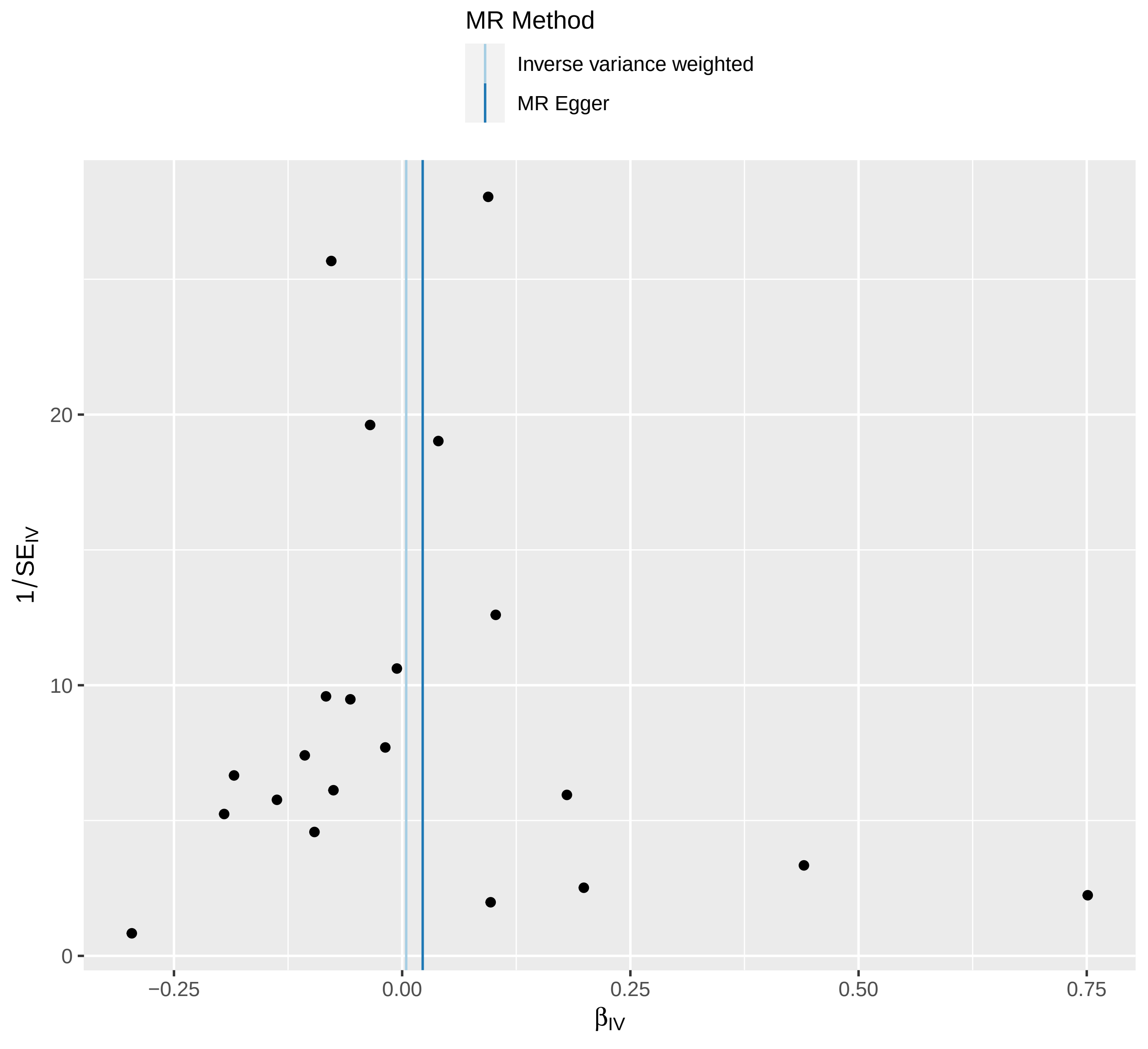
MR Method
Inverse variance weighted
MR Egger
20
10
0
1/SE$_{IV}$
−0.25
0.00
0.25
0.50
0.75
β$_{IV}$

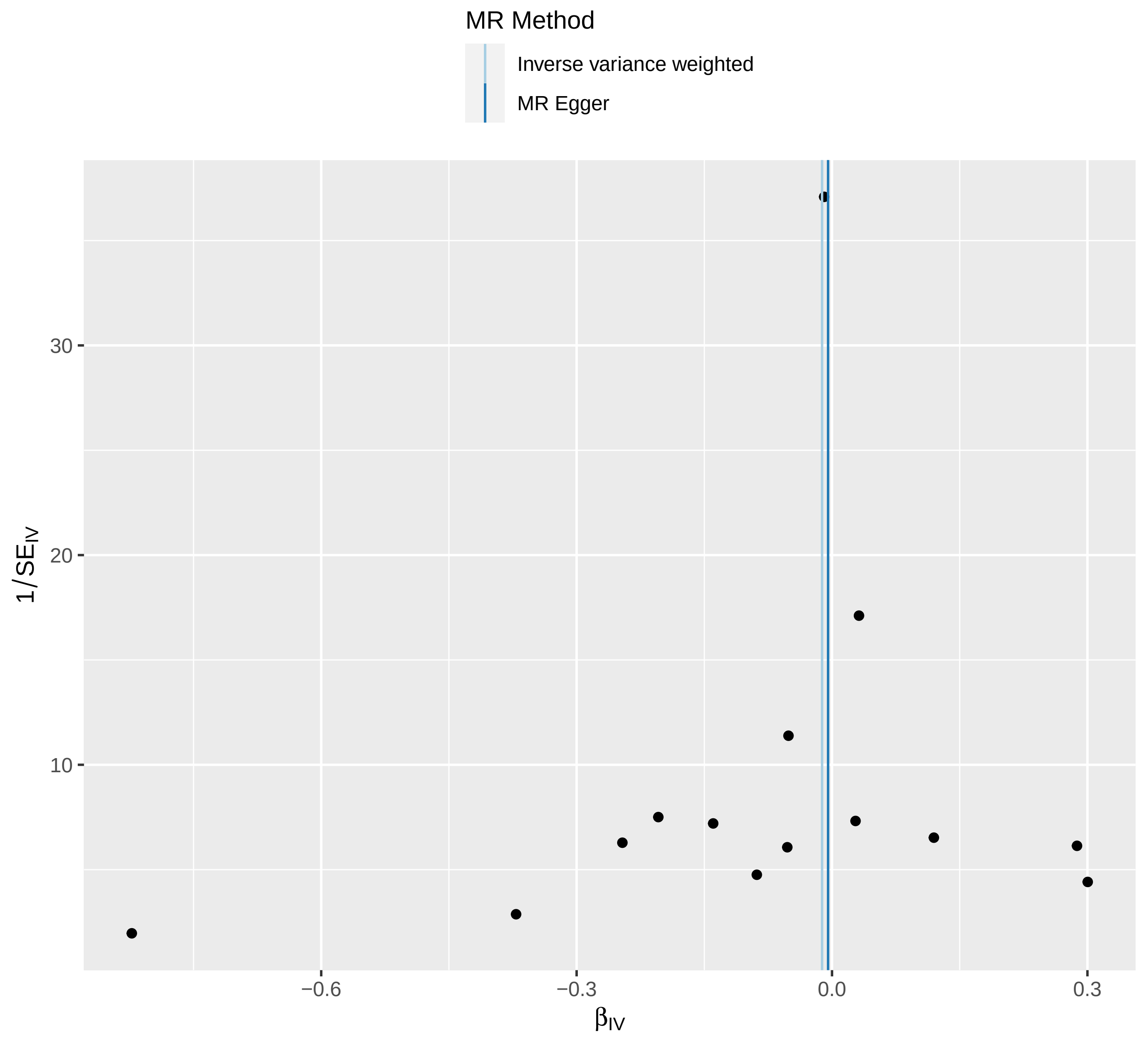
MR Method
Inverse variance weighted
MR Egger
30
20
10
$1/SE_{IV}$
−0.6
−0.3
0.0
0.3
β_{IV}

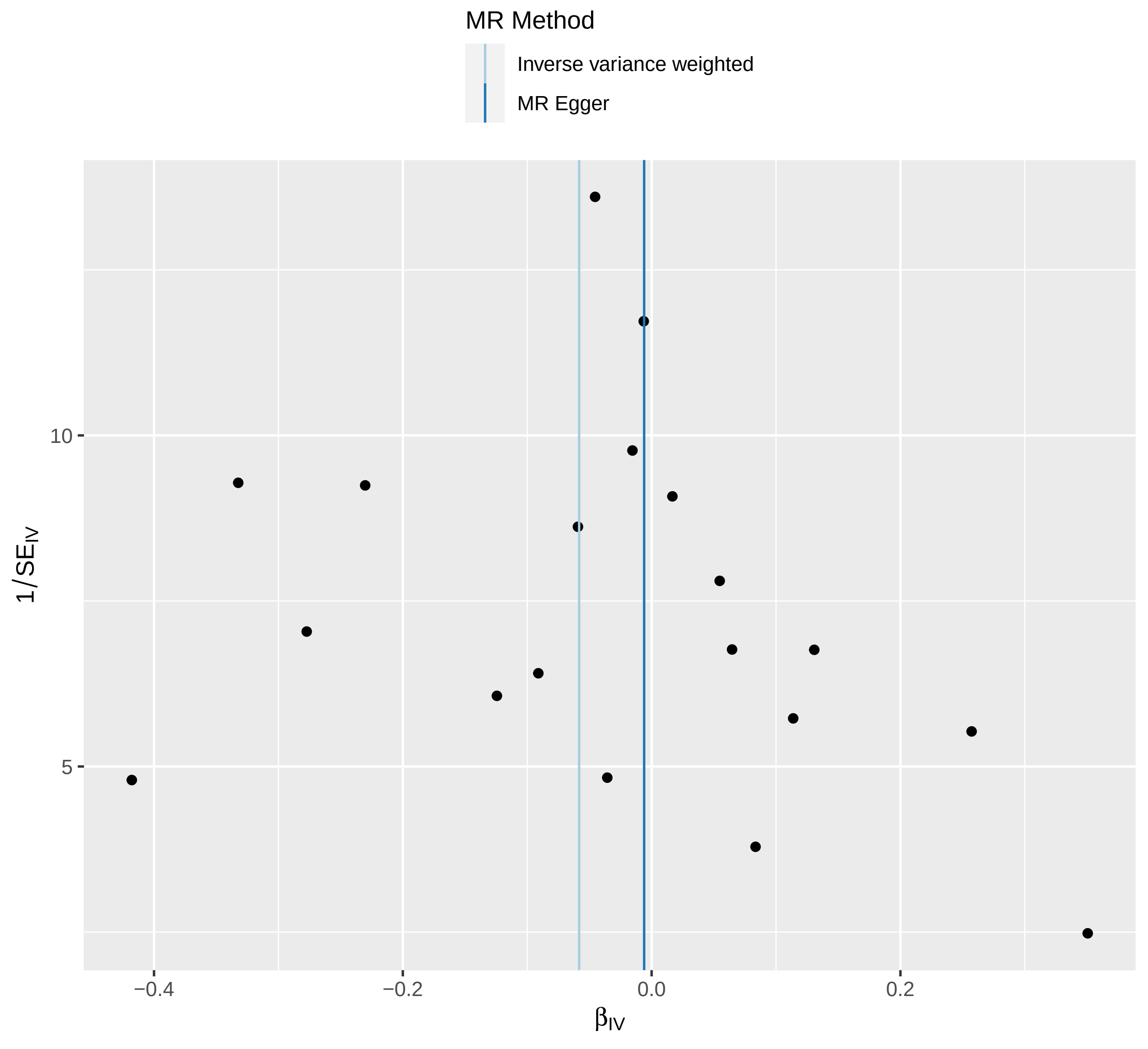
MR Method
Inverse variance weighted
MR Egger
10
5
$1/SE_{IV}$
−0.4
−0.2
0.0
0.2
β_{IV}

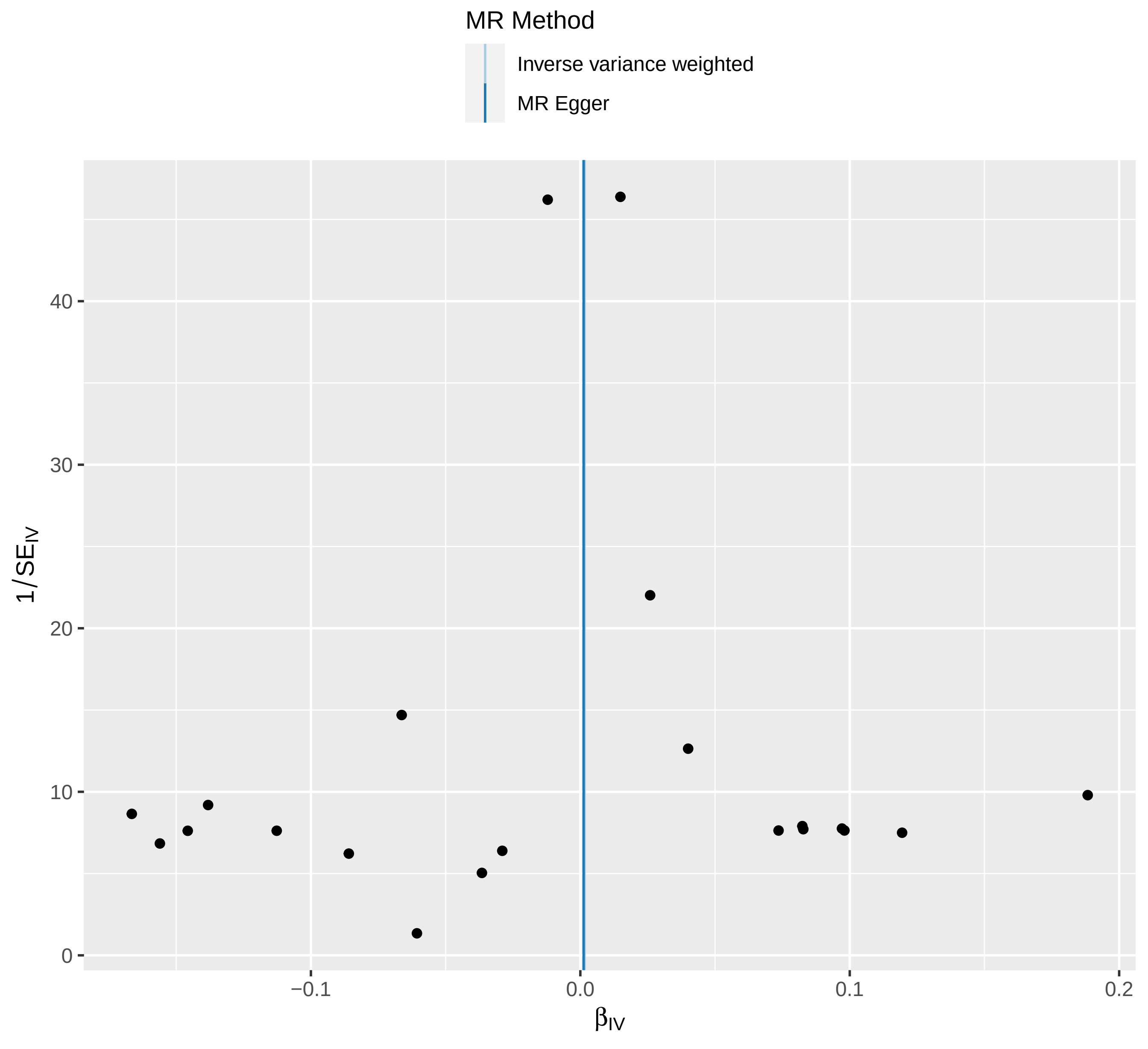
MR Method
Inverse variance weighted
MR Egger
1/SE$_{IV}$
β$_{IV}$
40
30
20
10
0
−0.1
0.0
0.1
0.2

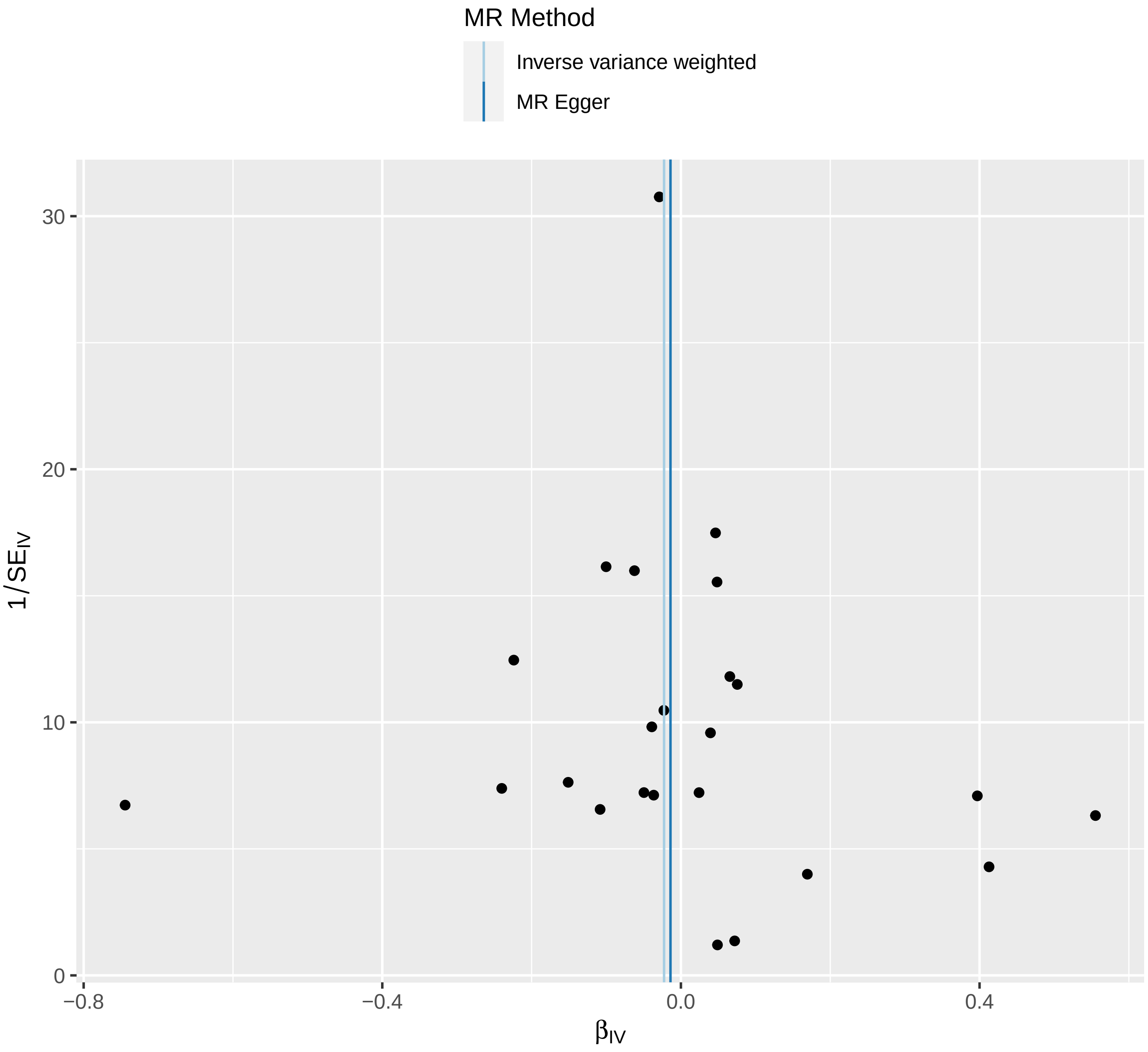
MR Method
Inverse variance weighted
MR Egger
1/SE$_{IV}$
30
20
10
0
−0.8
−0.4
0.0
0.4
β$_{IV}$

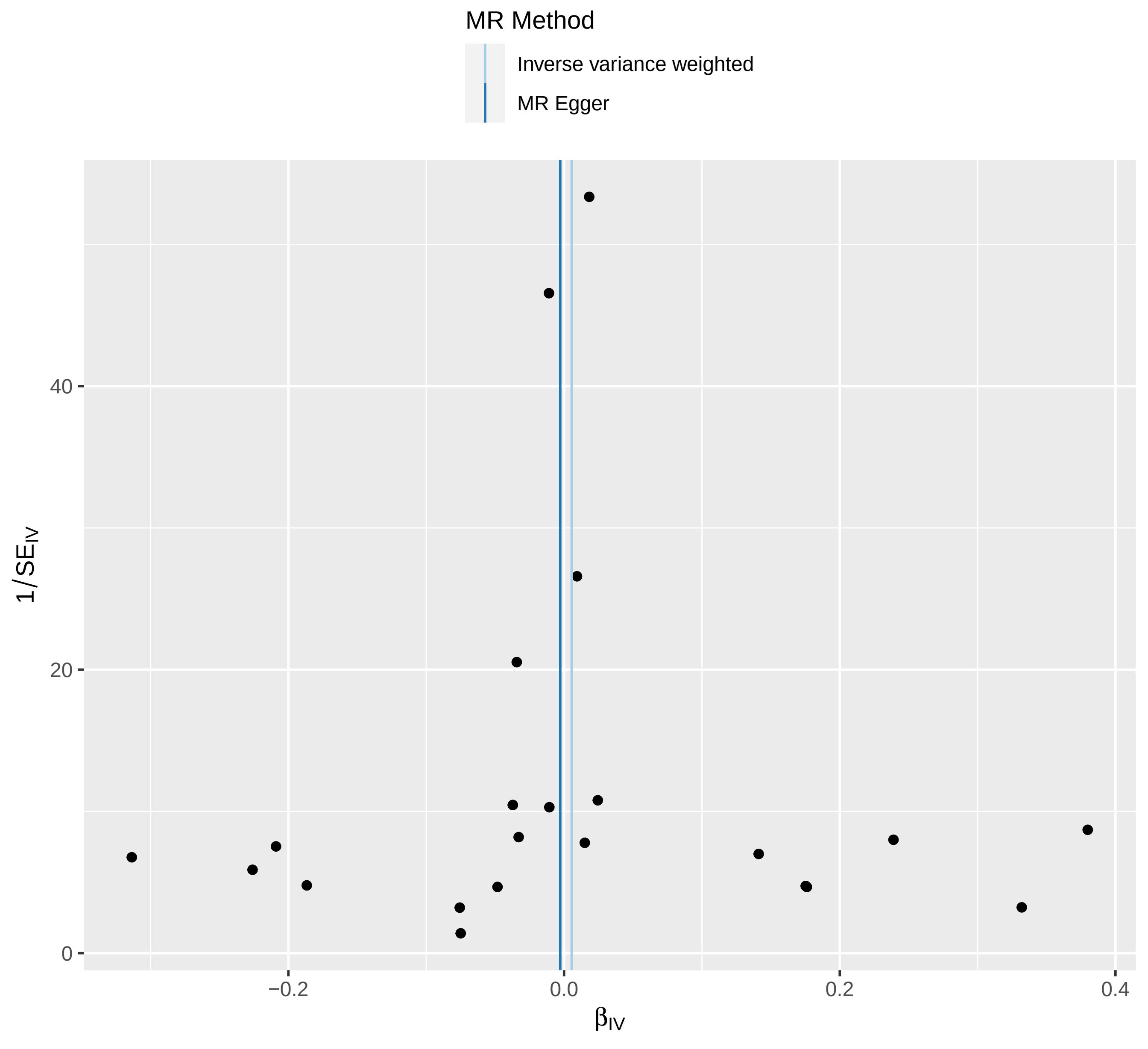
MR Method
Inverse variance weighted
MR Egger
$1/SE_{IV}$
0
20
40
−0.2
0.0
0.2
0.4
β_{IV}

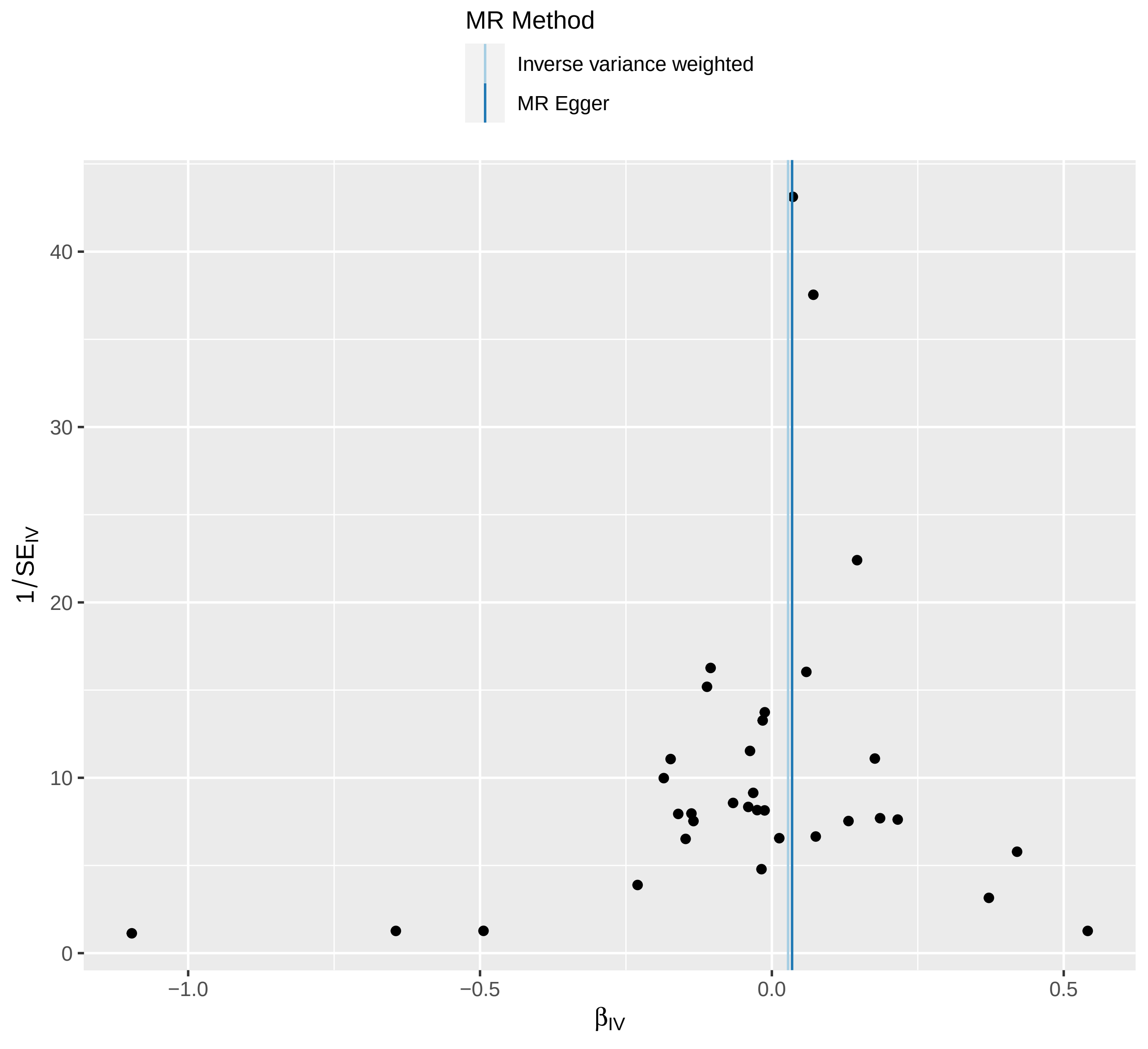

MR Method
Inverse variance weighted
MR Egger
40
30
20
10
0
1/SE$_{IV}$
−1.0
−0.5
0.0
0.5
β$_{IV}$

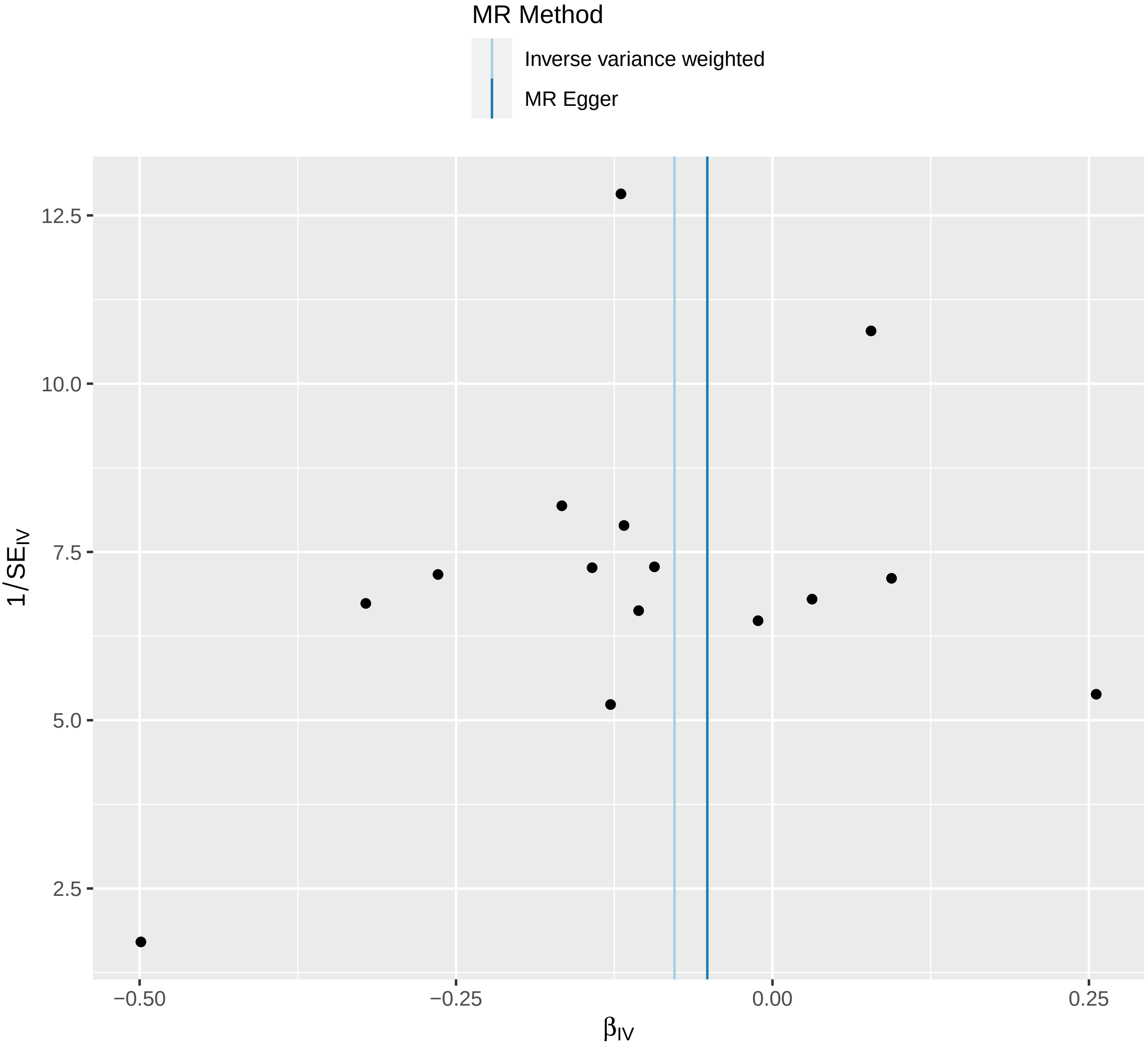

MR Method
Inverse variance weighted
MR Egger
12.5
10.0
7.5
5.0
2.5
1/SE$_{IV}$
−0.50
−0.25
0.00
0.25
β$_{IV}$

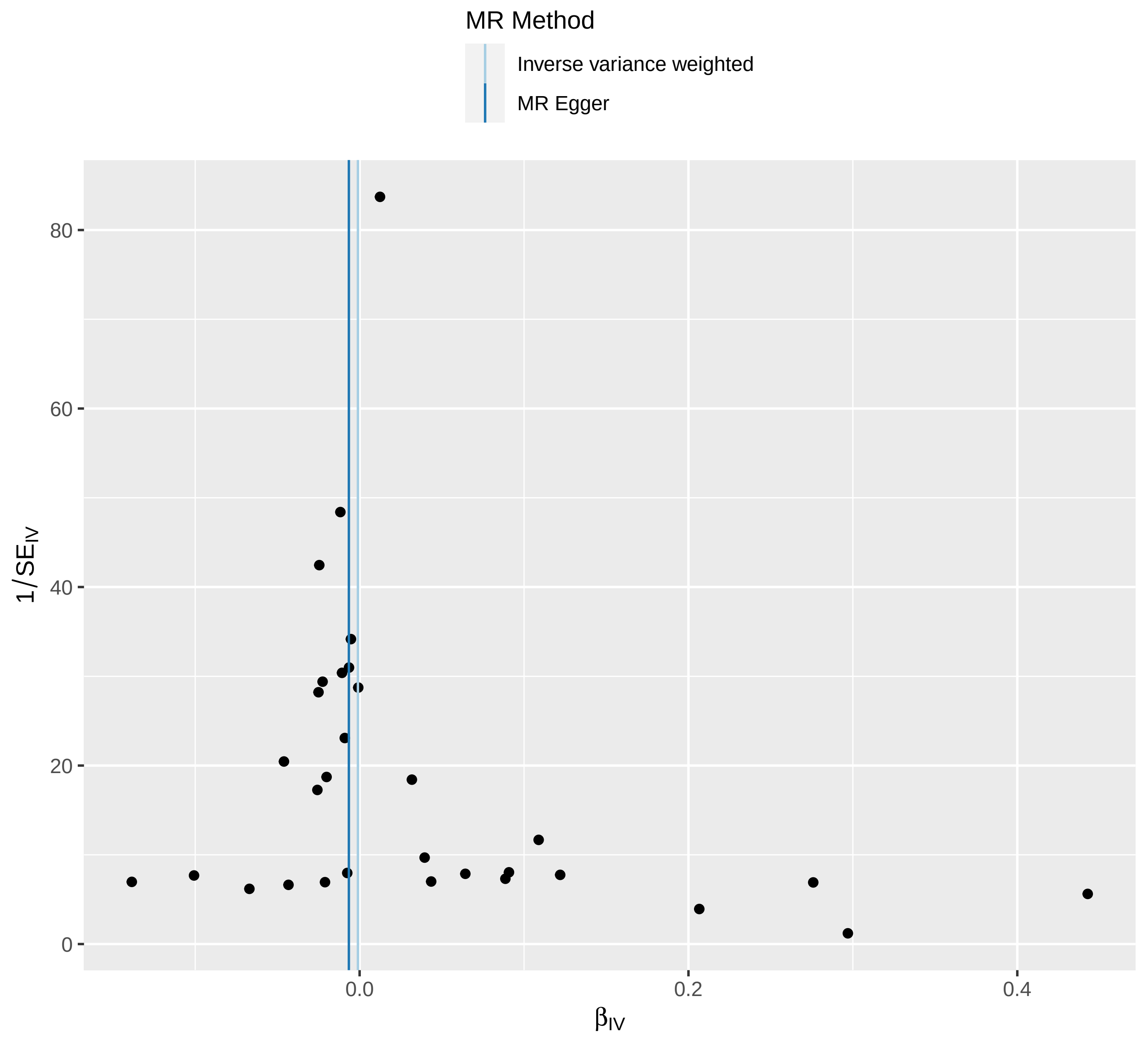

MR Method
Inverse variance weighted
MR Egger
80
60
40
20
0
0.0
0.2
0.4
$1/SE_{IV}$
β_{IV}

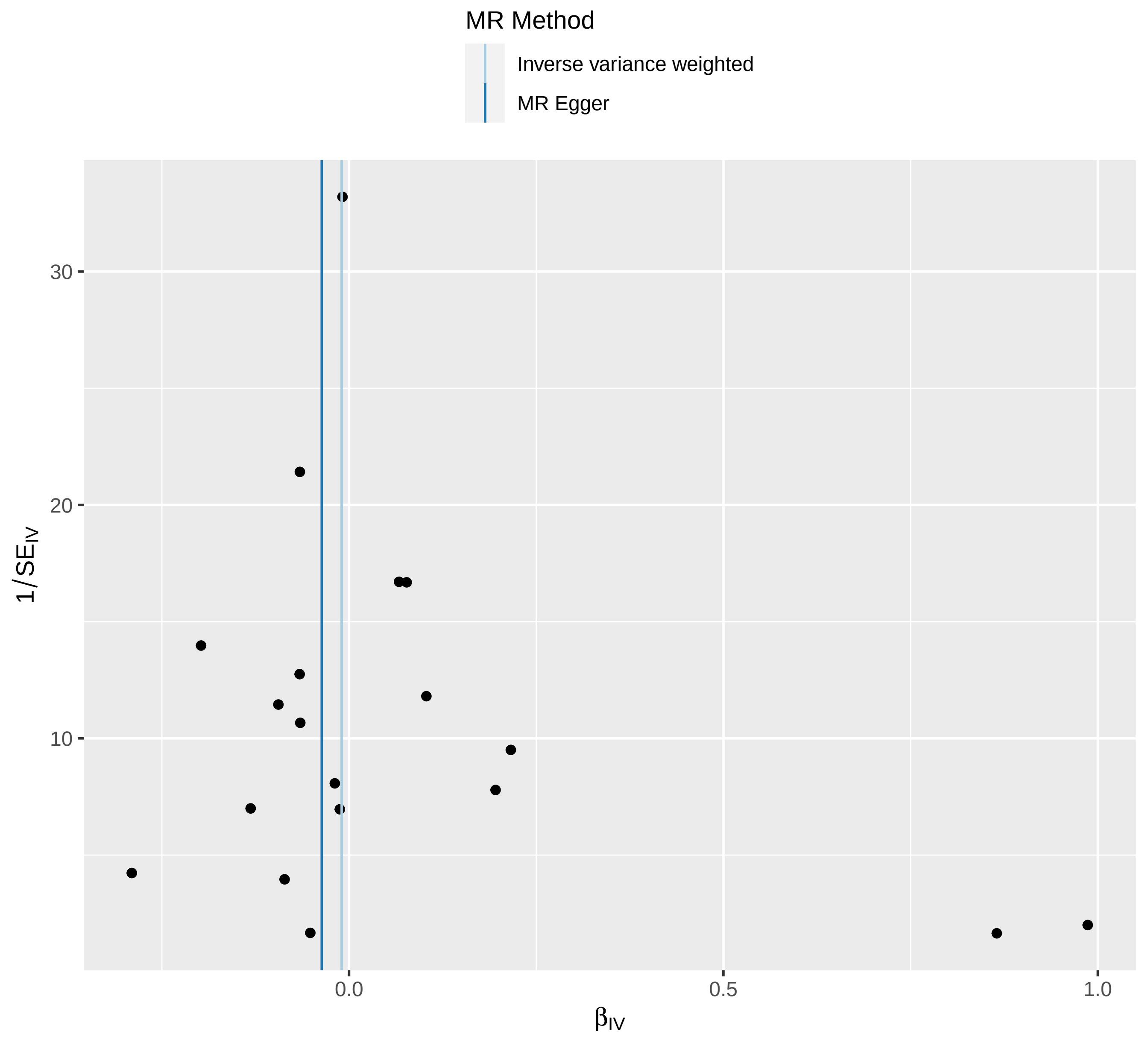
MR Method
Inverse variance weighted
MR Egger
30
20
10
0.0
0.5
1.0
$1/SE_{IV}$
β_{IV}

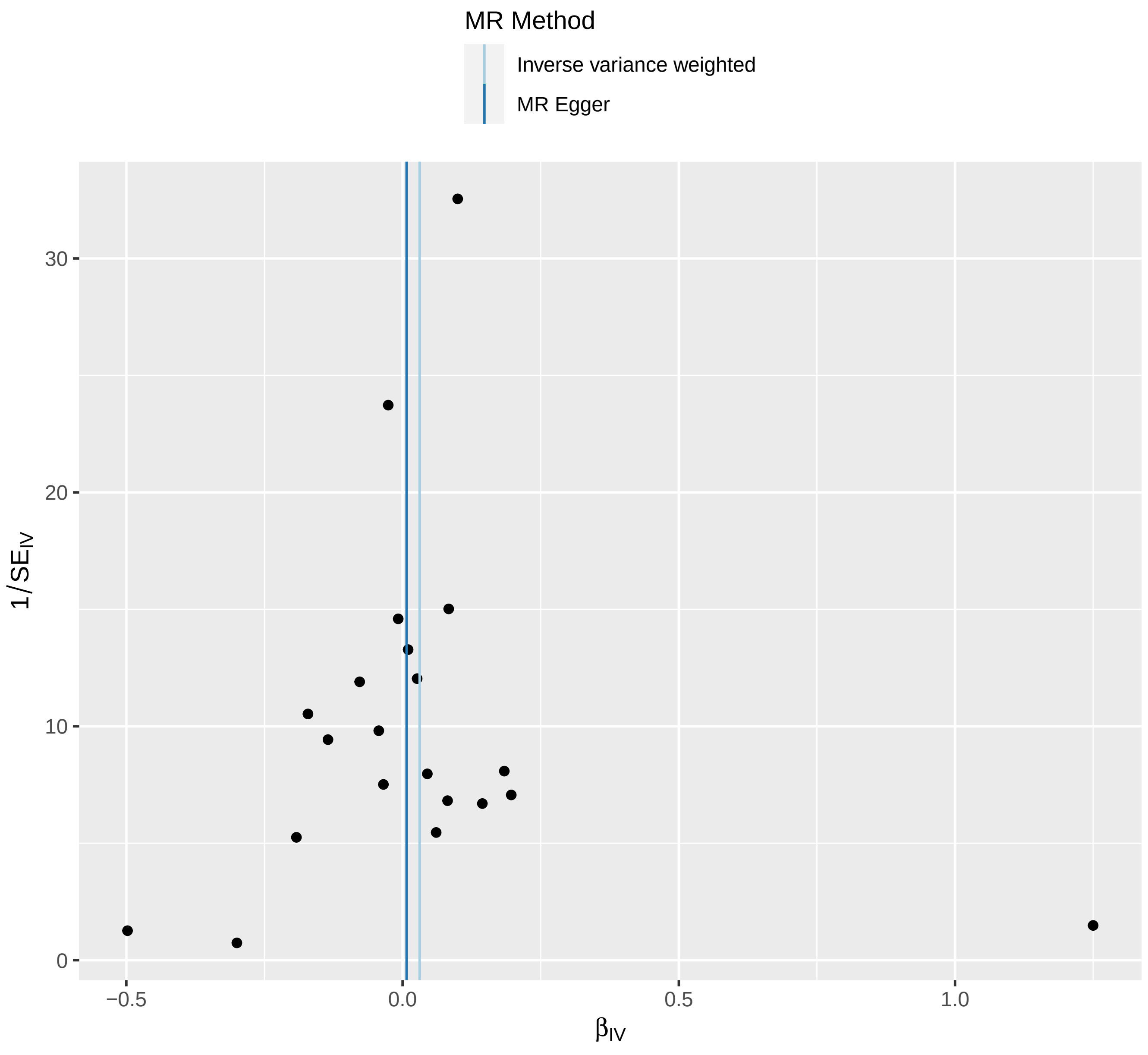
MR Method
Inverse variance weighted
MR Egger
30
20
10
0
$1/SE_{IV}$
−0.5
0.0
0.5
1.0
β_{IV}

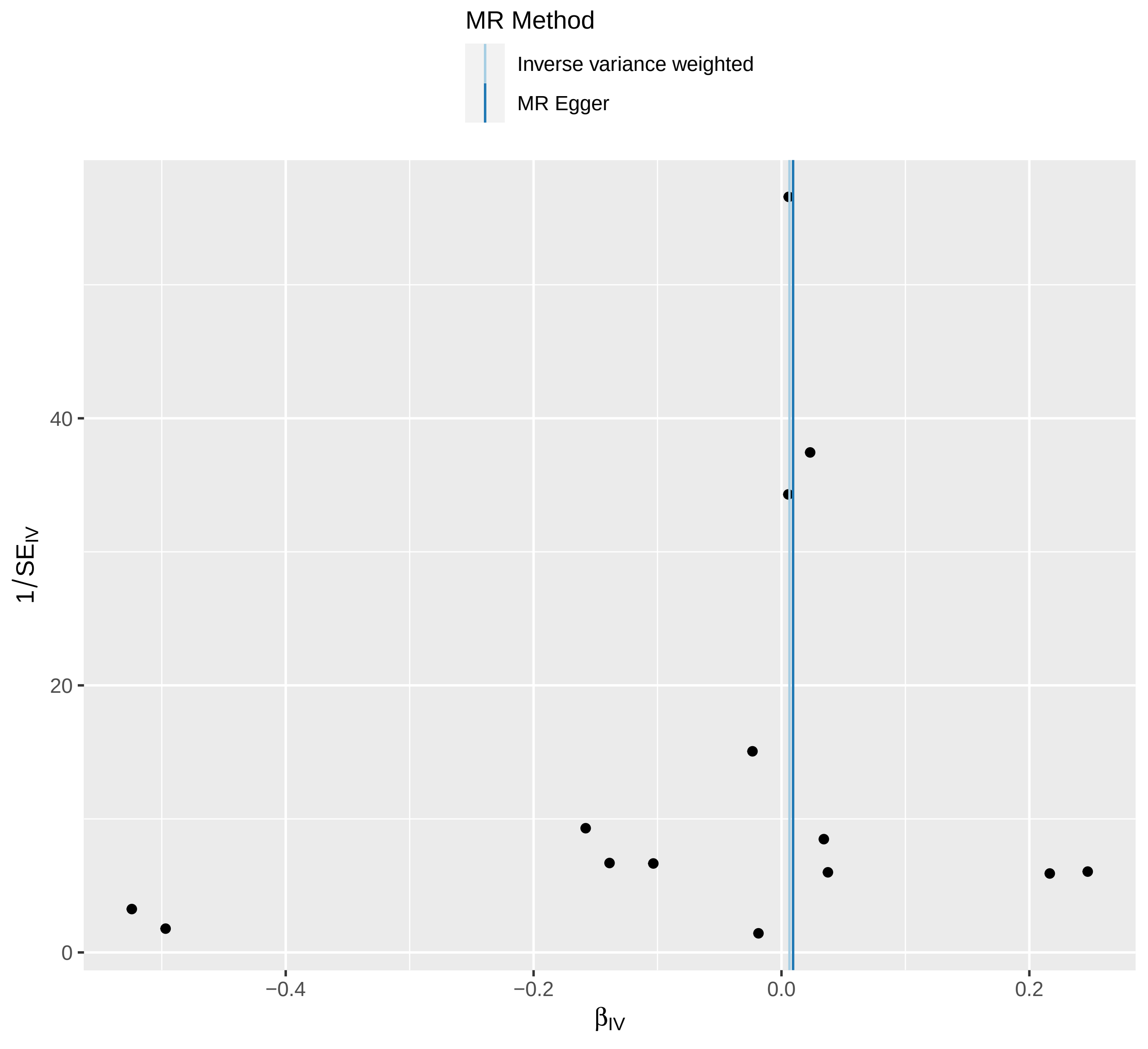
MR Method
Inverse variance weighted
MR Egger
40
20
0
1/SE$_{IV}$
−0.4
−0.2
0.0
0.2
β$_{IV}$

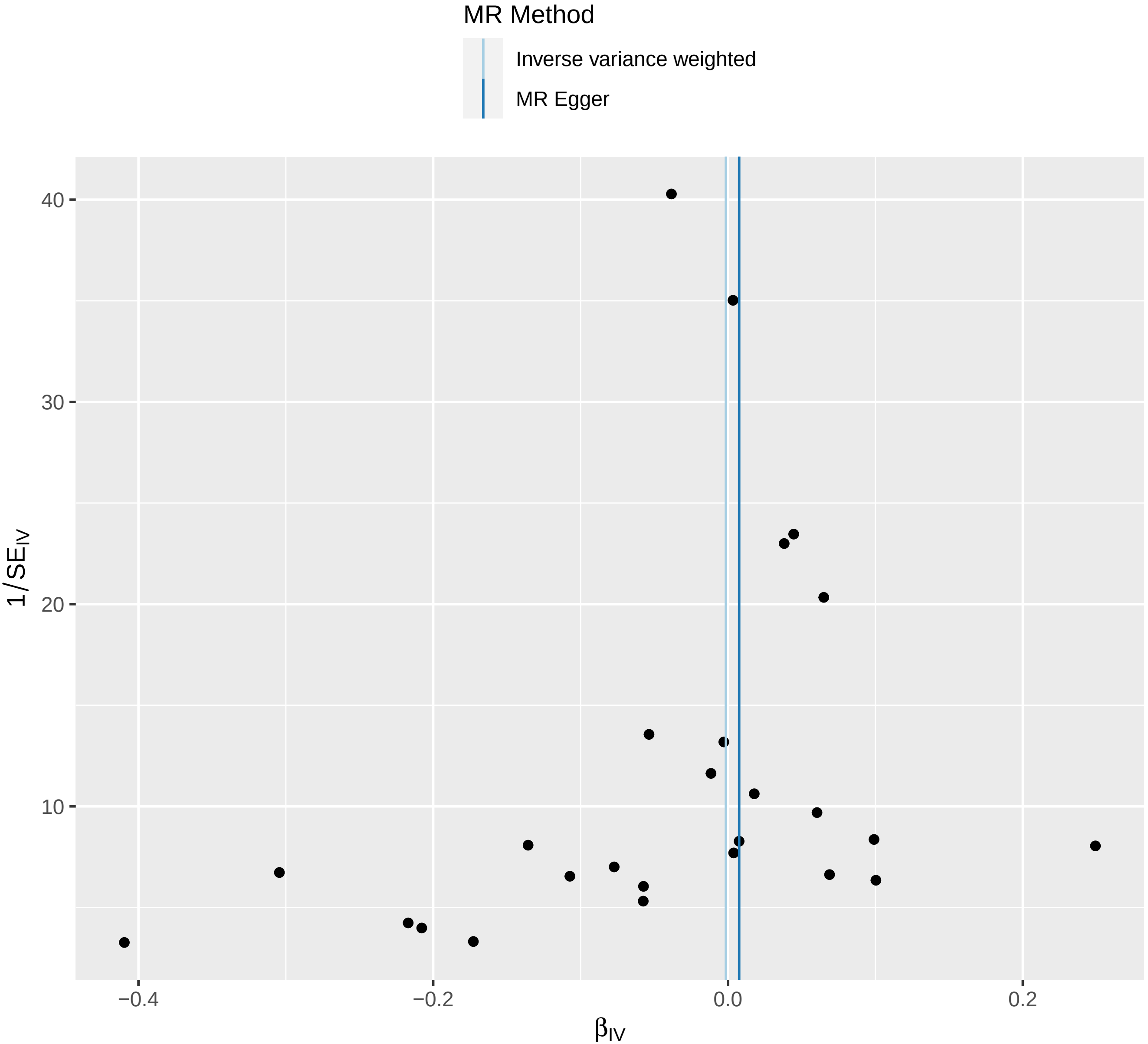

MR Method
Inverse variance weighted
MR Egger
1/SE$_{IV}$
β$_{IV}$
40
30
20
10
−0.4
−0.2
0.0
0.2

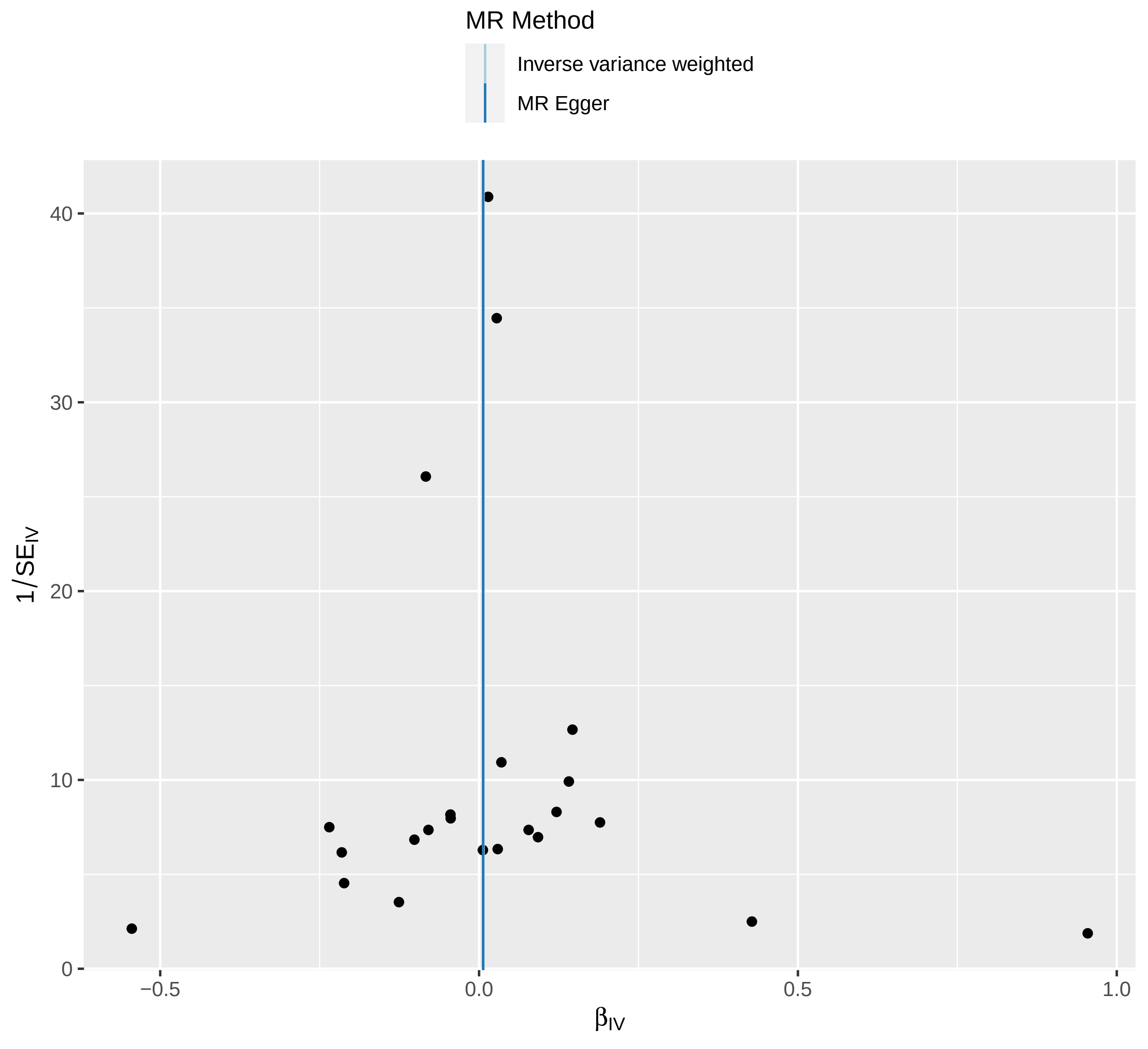

MR Method
Inverse variance weighted
MR Egger
1/SE_IV
β_IV
40
30
20
10
0
−0.5
0.0
0.5
1.0

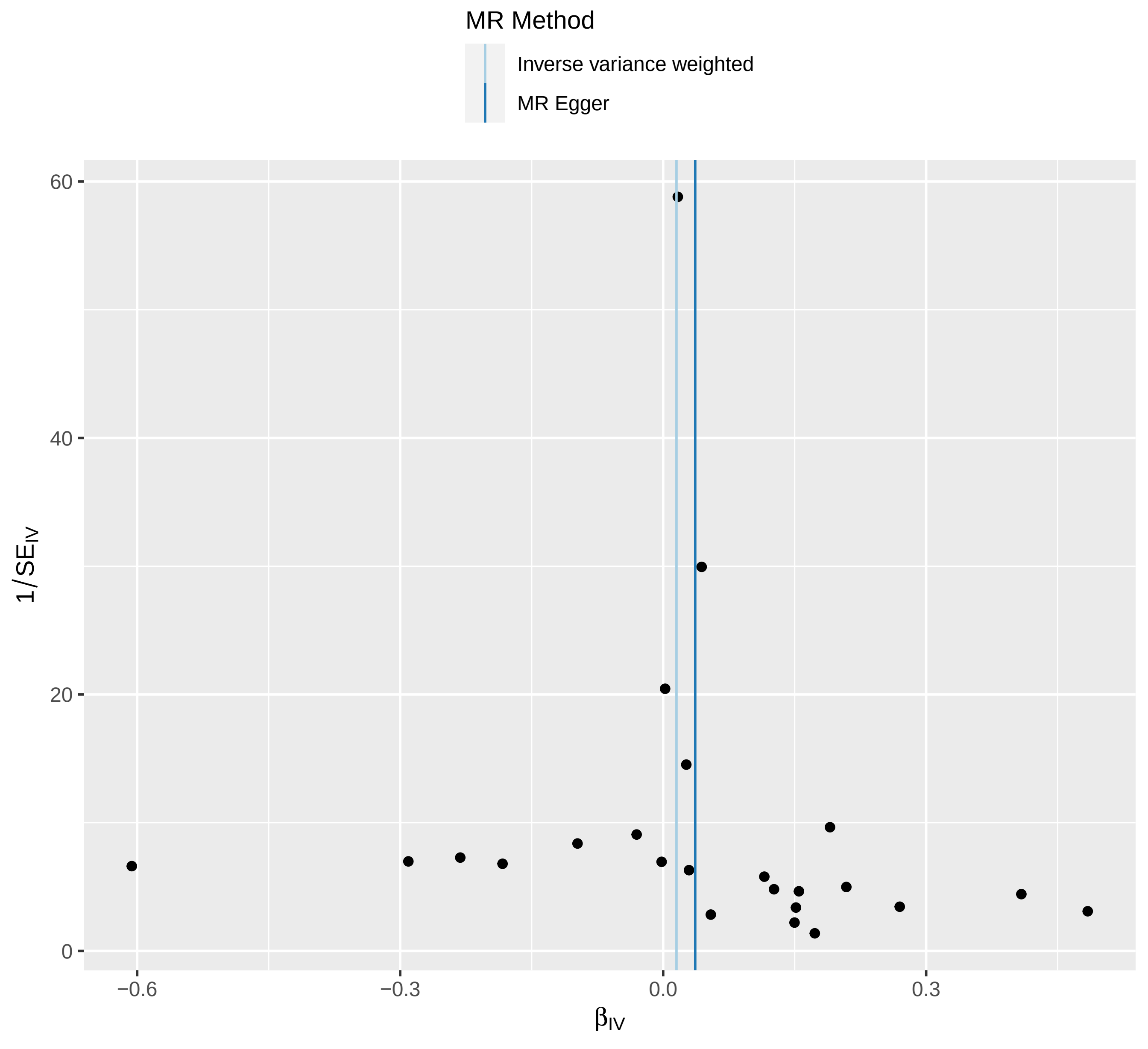

MR Method
Inverse variance weighted
MR Egger
60
40
20
0
−0.6
−0.3
0.0
0.3
β_{IV}
$1/SE_{IV}$

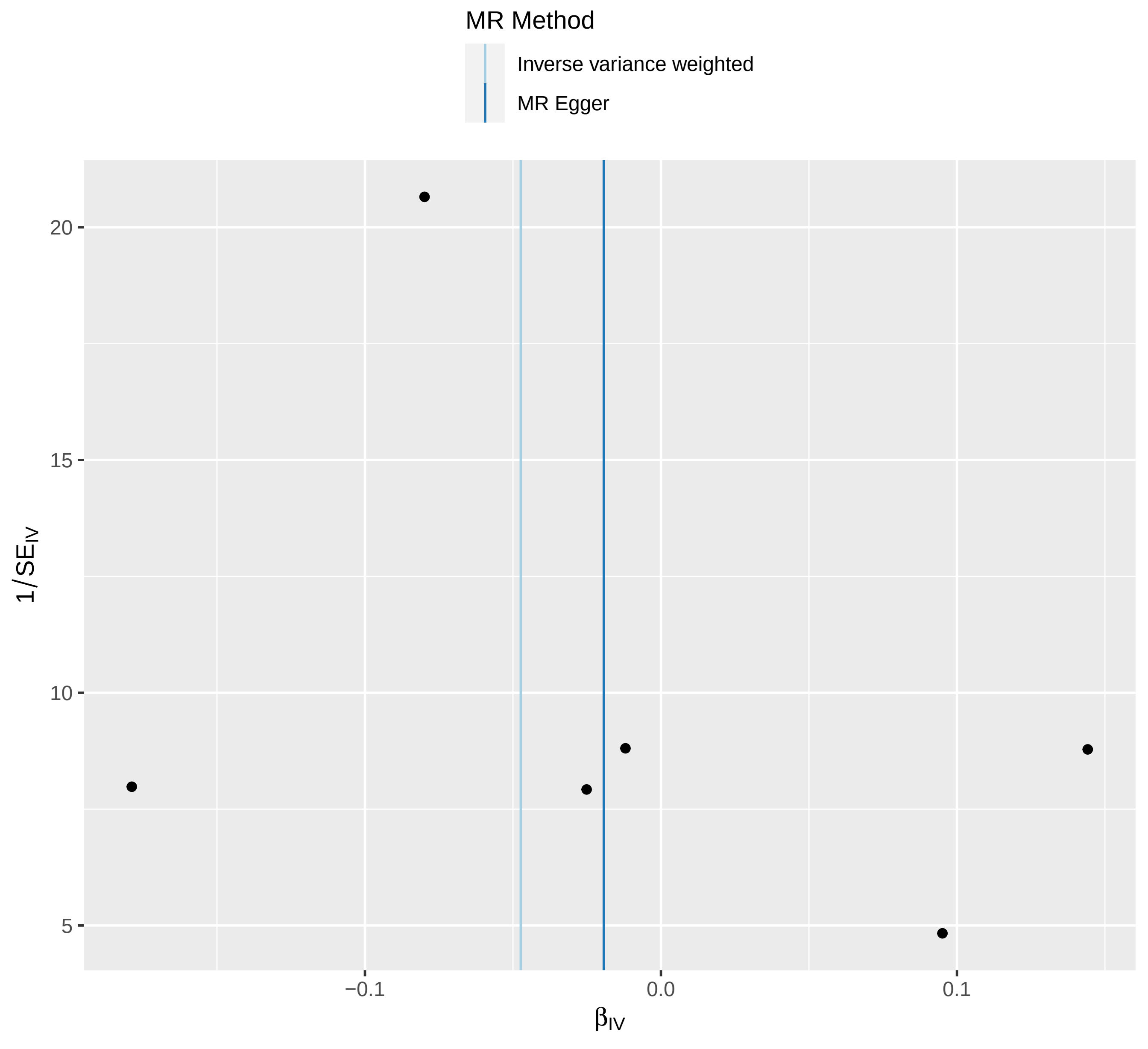
MR Method
Inverse variance weighted
MR Egger
20
15
10
5
1/SE_IV
−0.1
0.0
0.1
β_IV

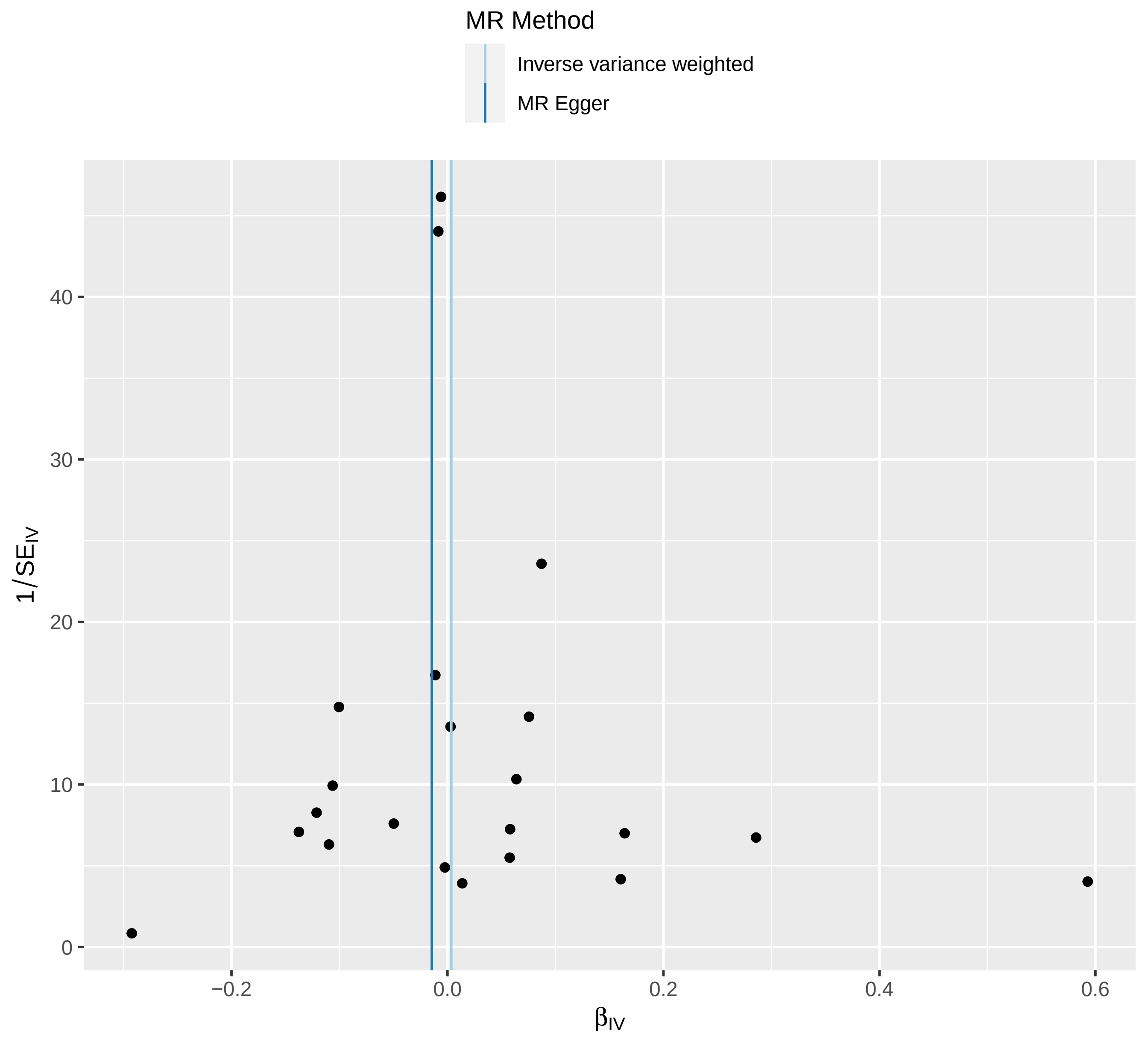
MR Method
Inverse variance weighted
MR Egger
40
30
20
10
0
−0.2
0.0
0.2
0.4
0.6
1/SE_IV
β_IV

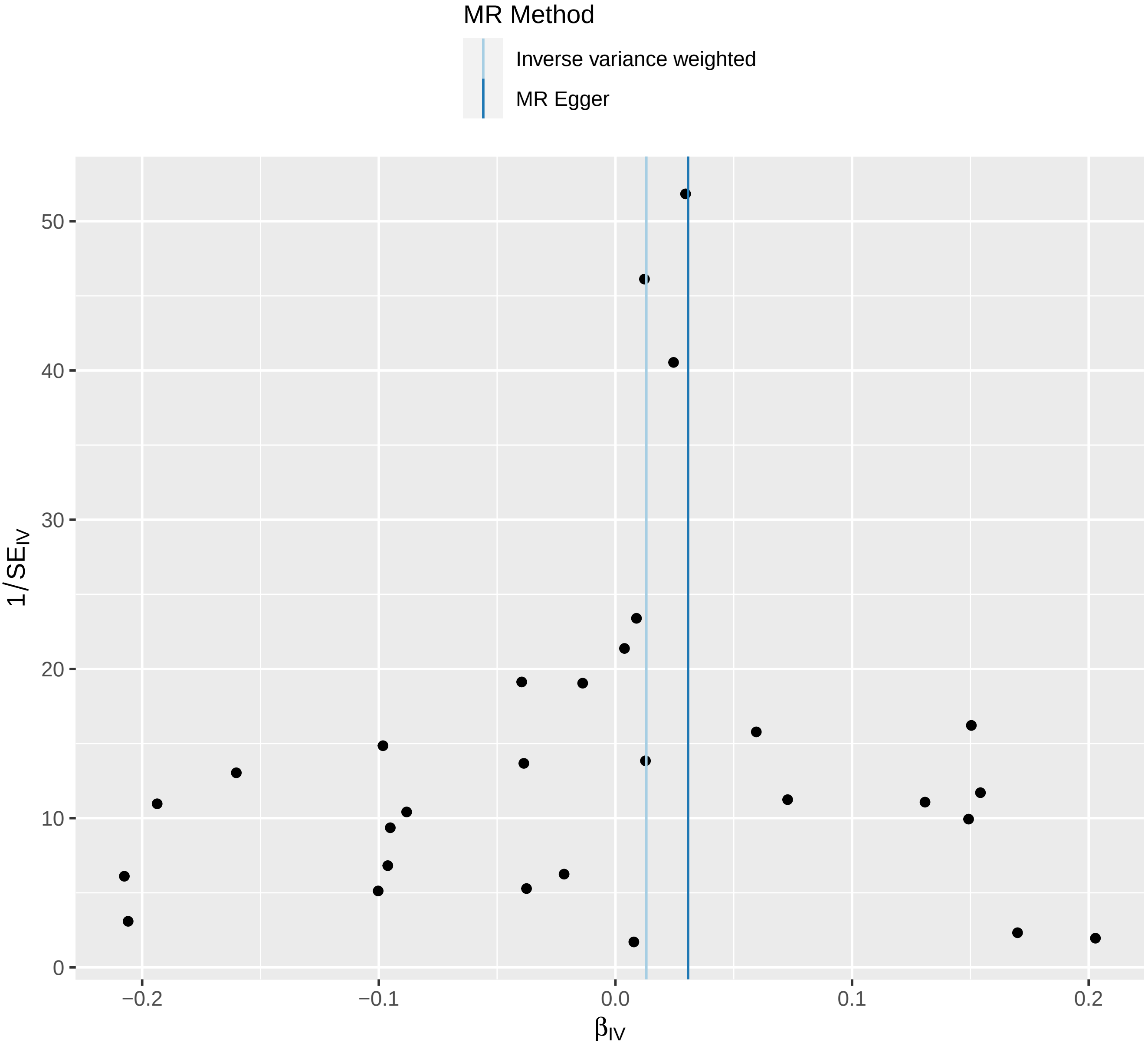

MR Method
Inverse variance weighted
MR Egger
1/SE_IV
50
40
30
20
10
0
−0.2
−0.1
0.0
0.1
0.2
β_IV

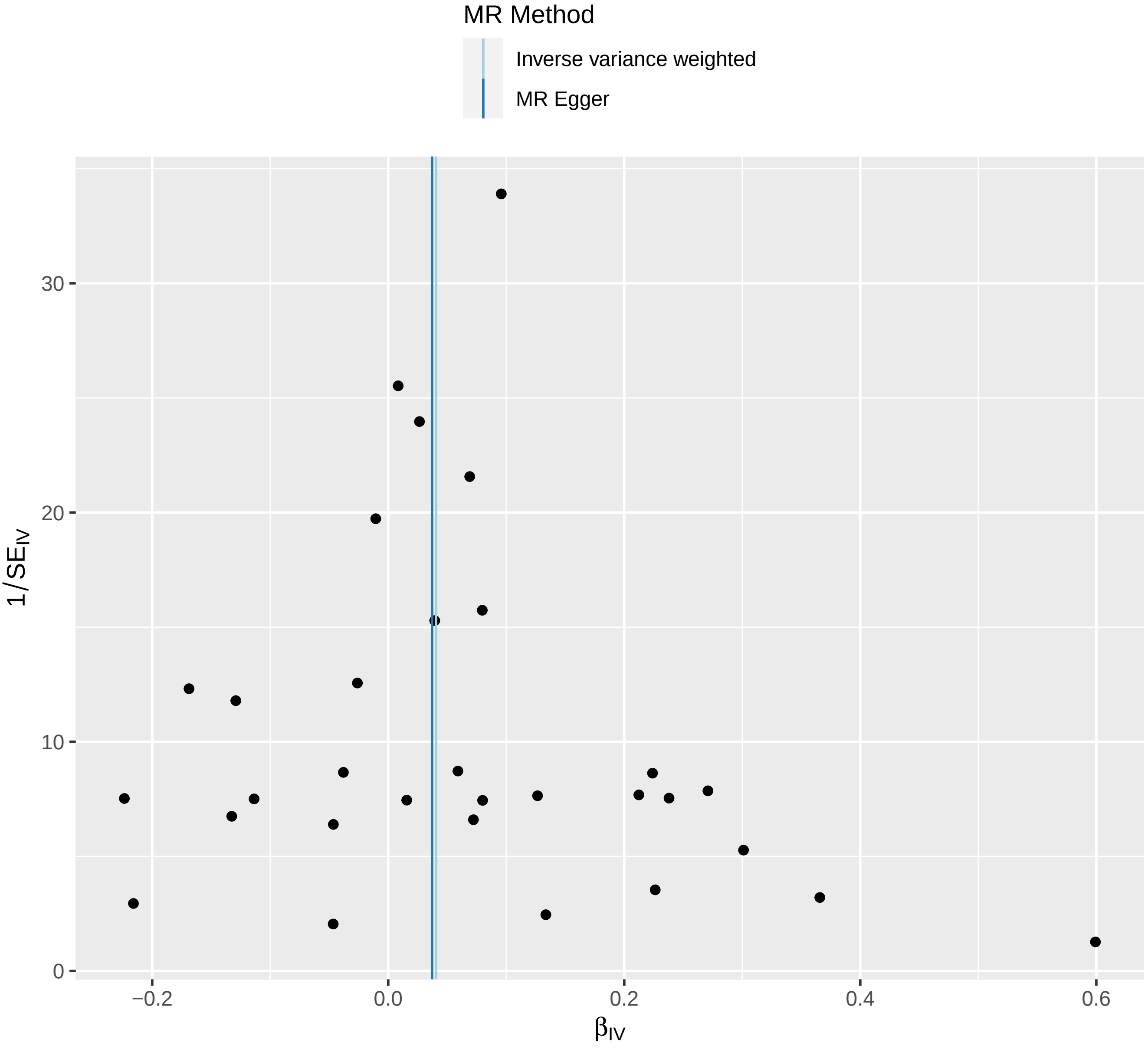

MR Method
Inverse variance weighted
MR Egger
30
20
10
0
1/SE$_{IV}$
−0.2
0.0
0.2
0.4
0.6
β$_{IV}$

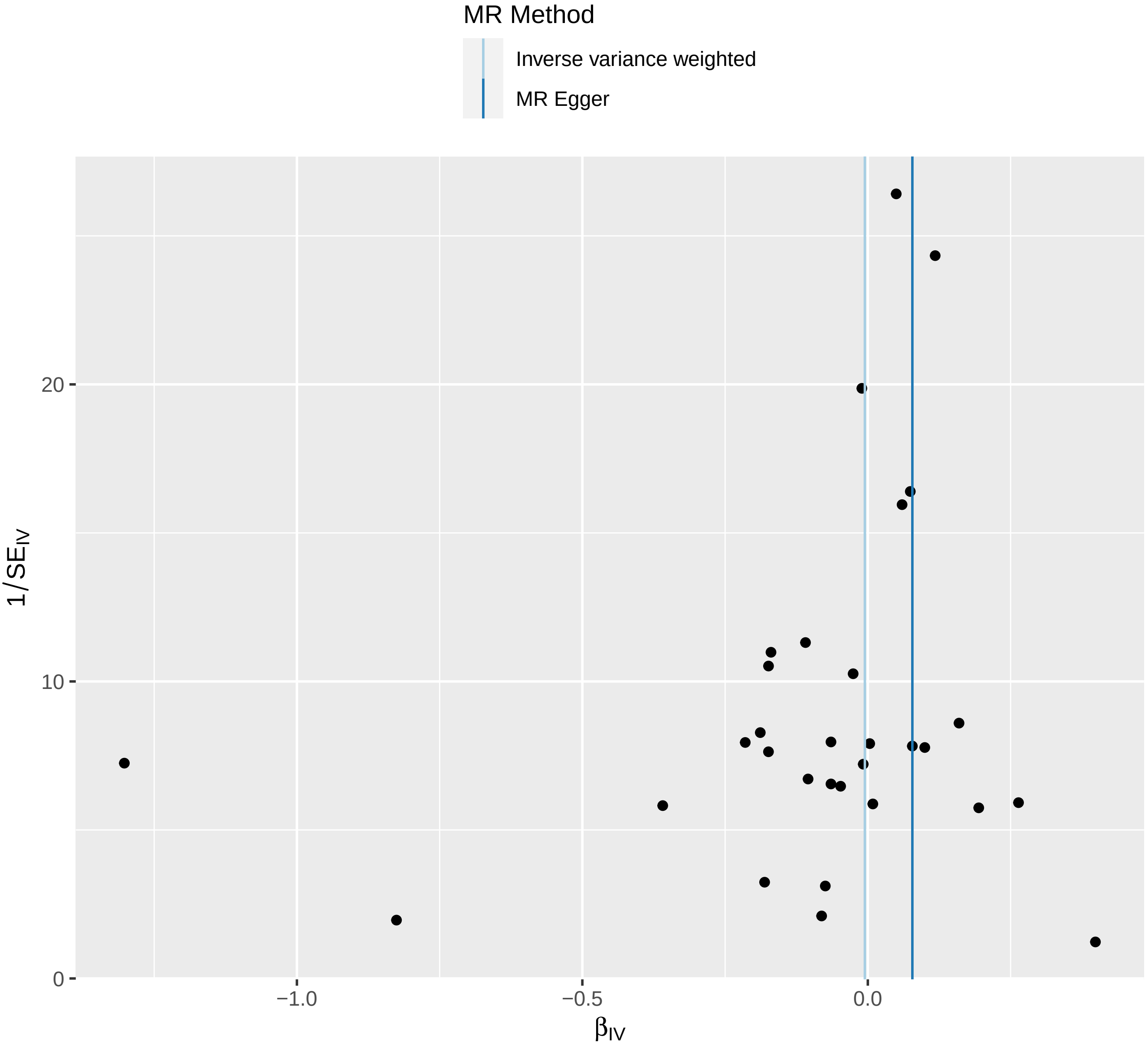

MR Method
Inverse variance weighted
MR Egger
1/SE$_{IV}$
20
10
0
−1.0
−0.5
0.0
β$_{IV}$

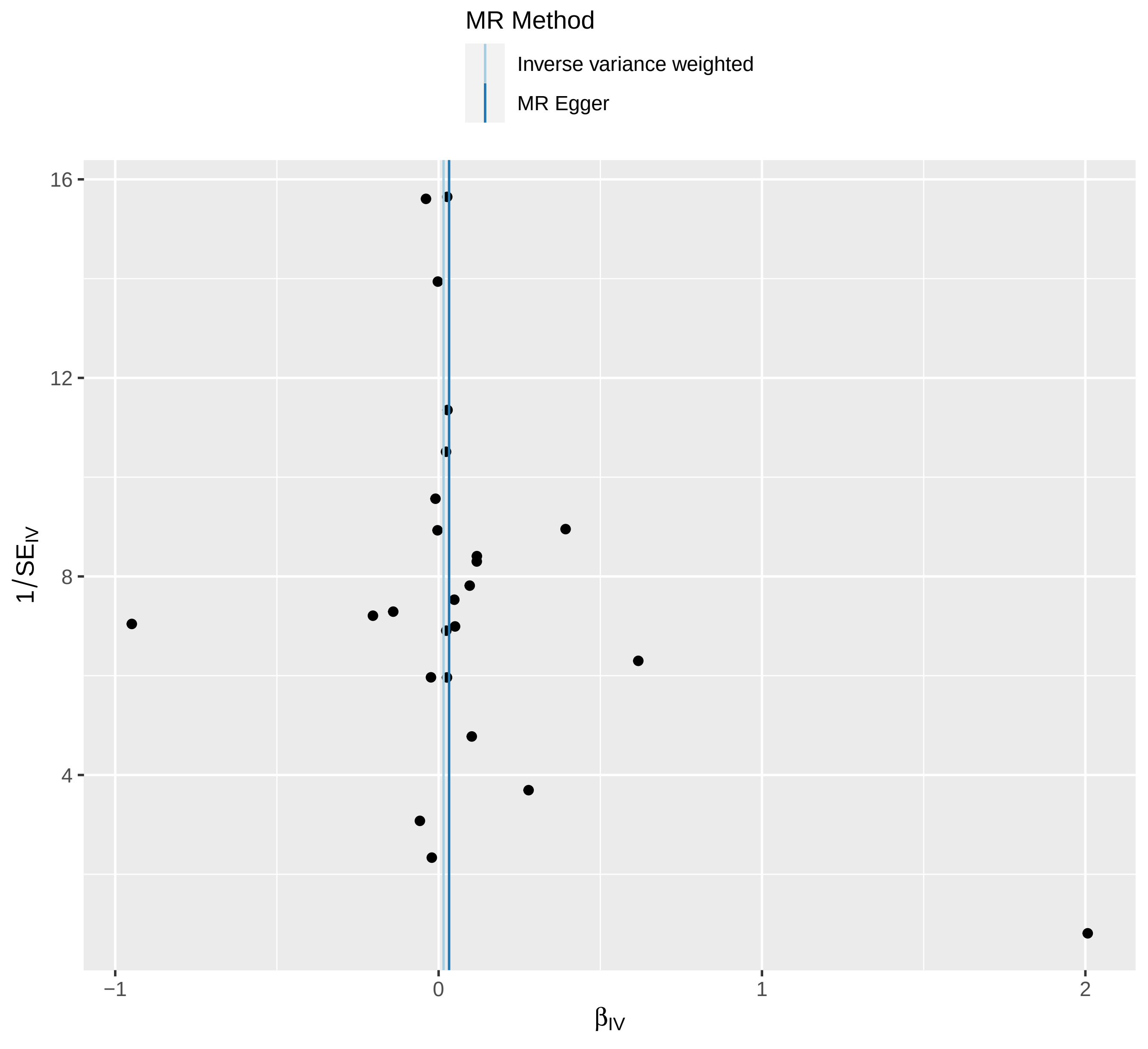
MR Method
Inverse variance weighted
MR Egger
16
12
8
4
$1/SE_{IV}$
−1
0
1
2
β_{IV}

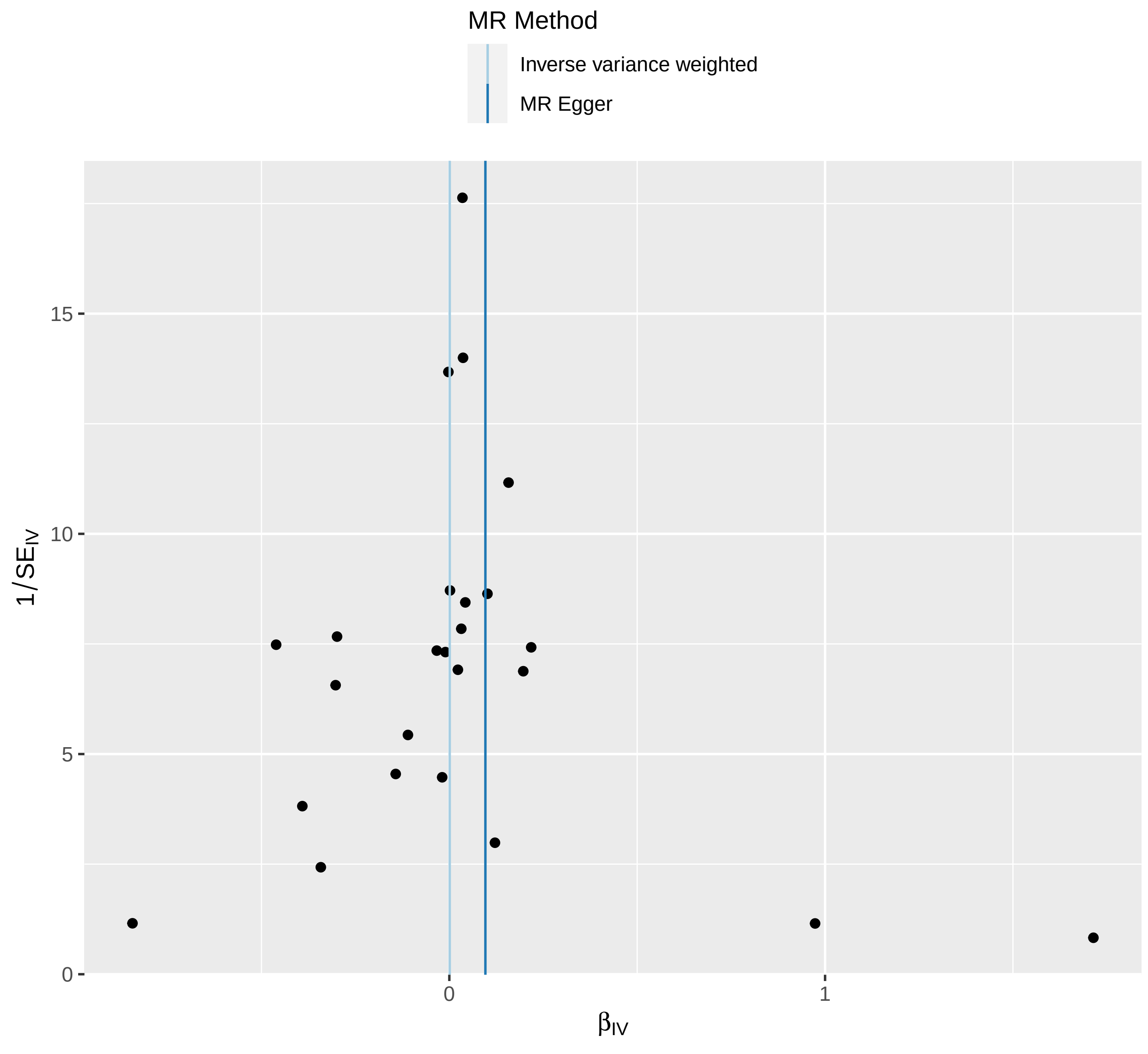

MR Method
Inverse variance weighted
MR Egger
15
10
5
0
$1/SE_{IV}$
0
1
β_{IV}

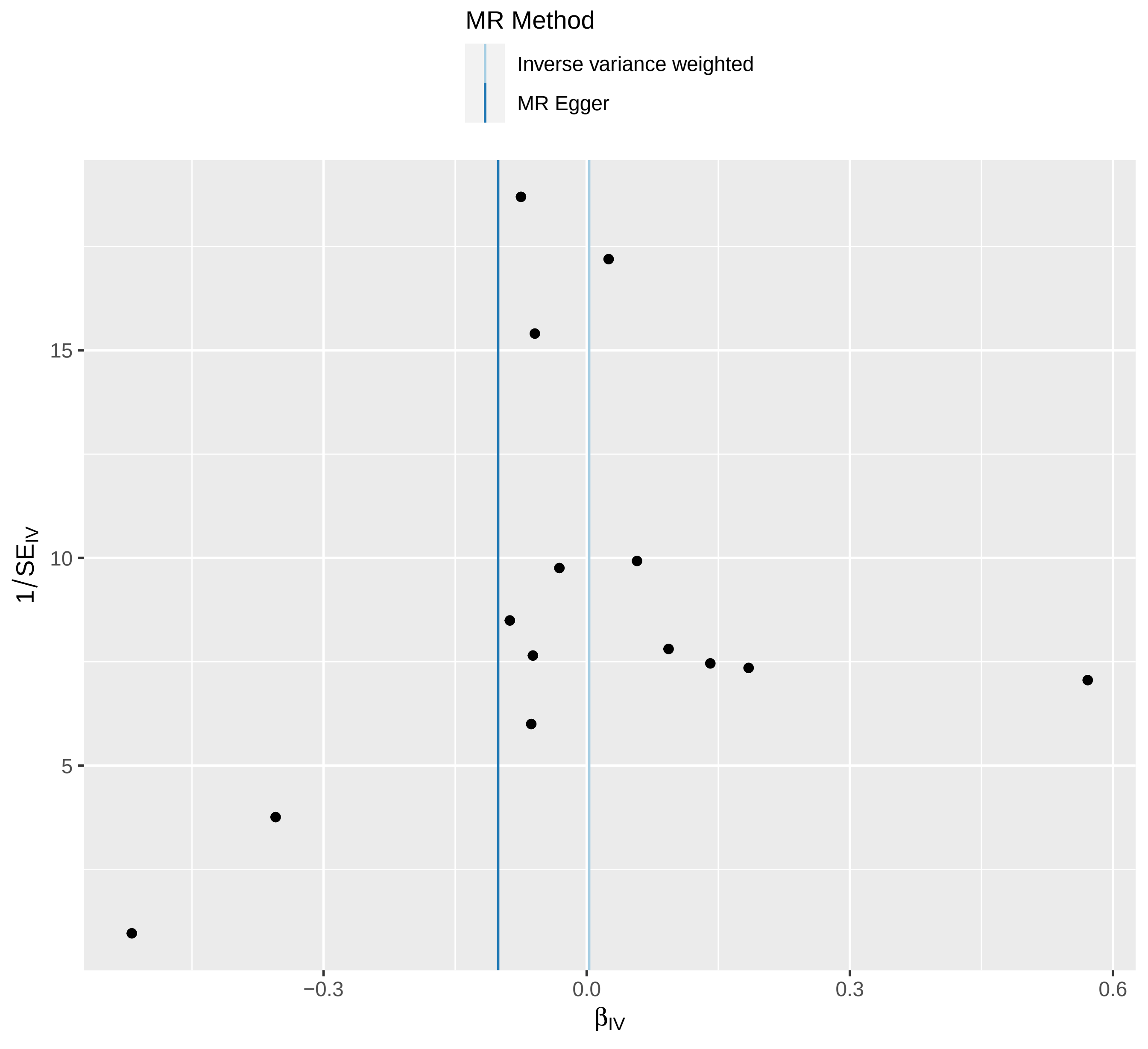
MR Method
Inverse variance weighted
MR Egger
15
10
5
$1/SE_{IV}$
−0.3
0.0
0.3
0.6
β_{IV}

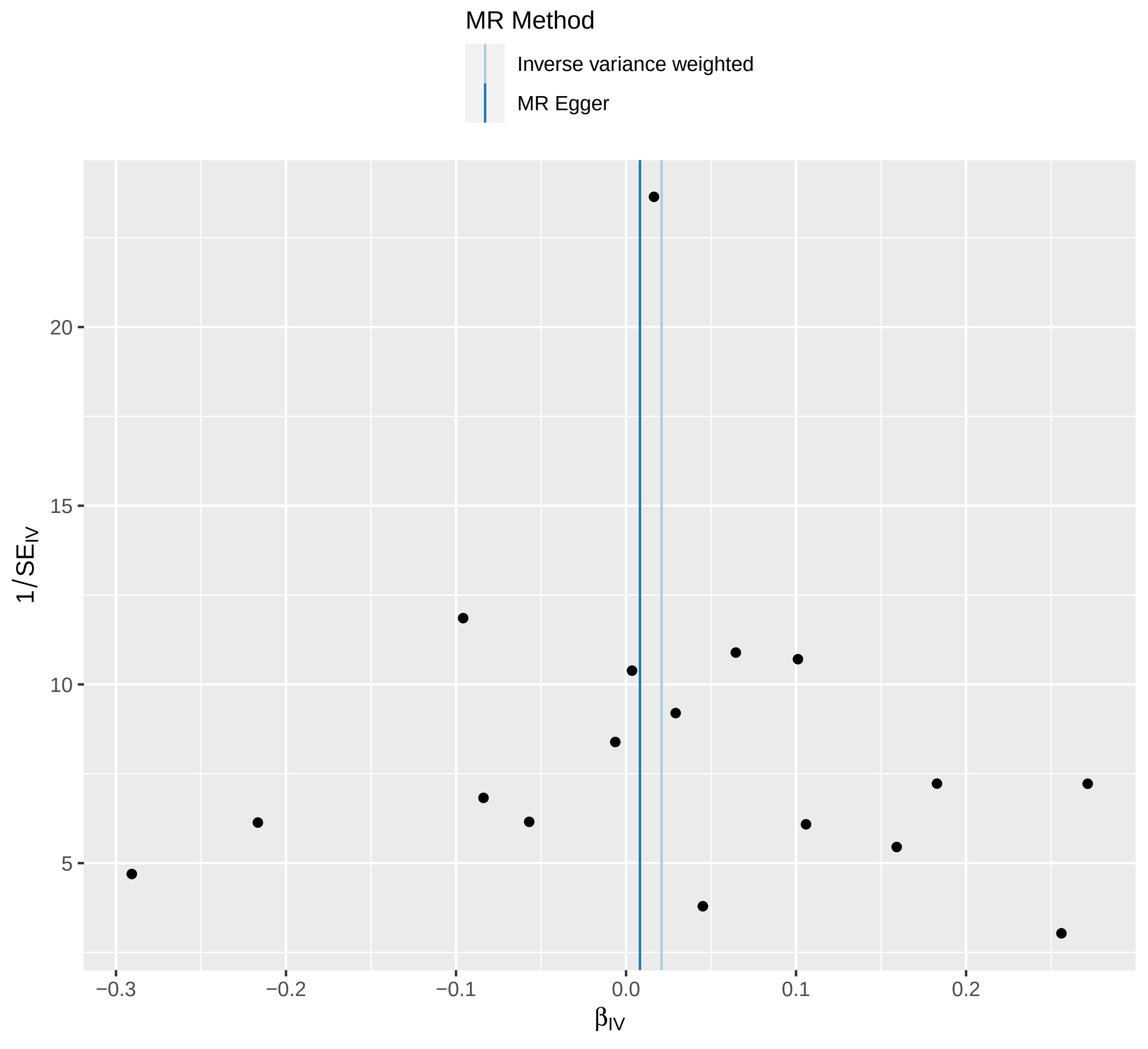
MR Method
Inverse variance weighted
MR Egger
20
15
10
5
$1/SE_{IV}$
−0.3
−0.2
−0.1
0.0
0.1
0.2
β_{IV}

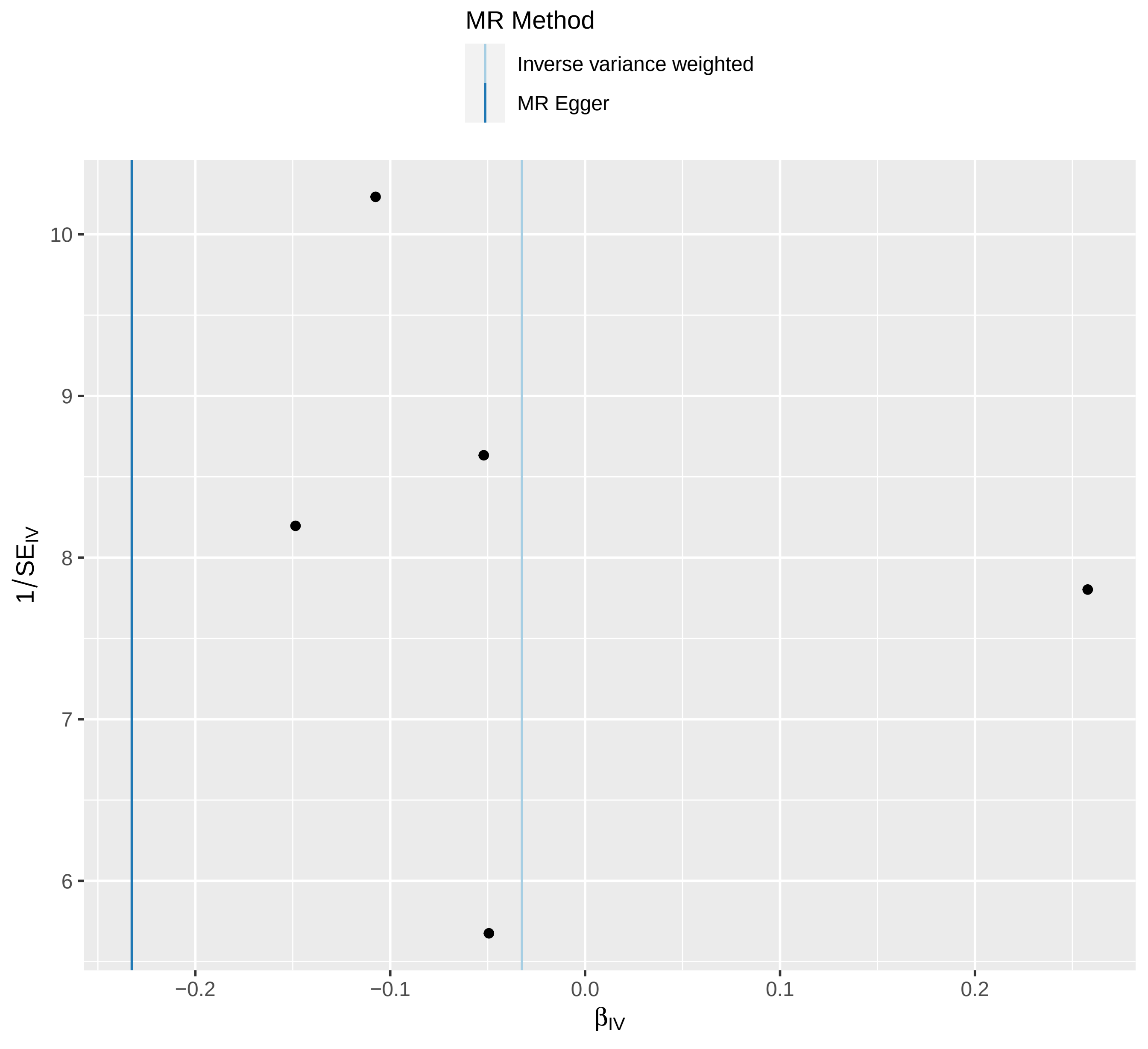

MR Method
Inverse variance weighted
MR Egger
10
9
8
7
6
1/SE_IV
−0.2
−0.1
0.0
0.1
0.2
β_IV

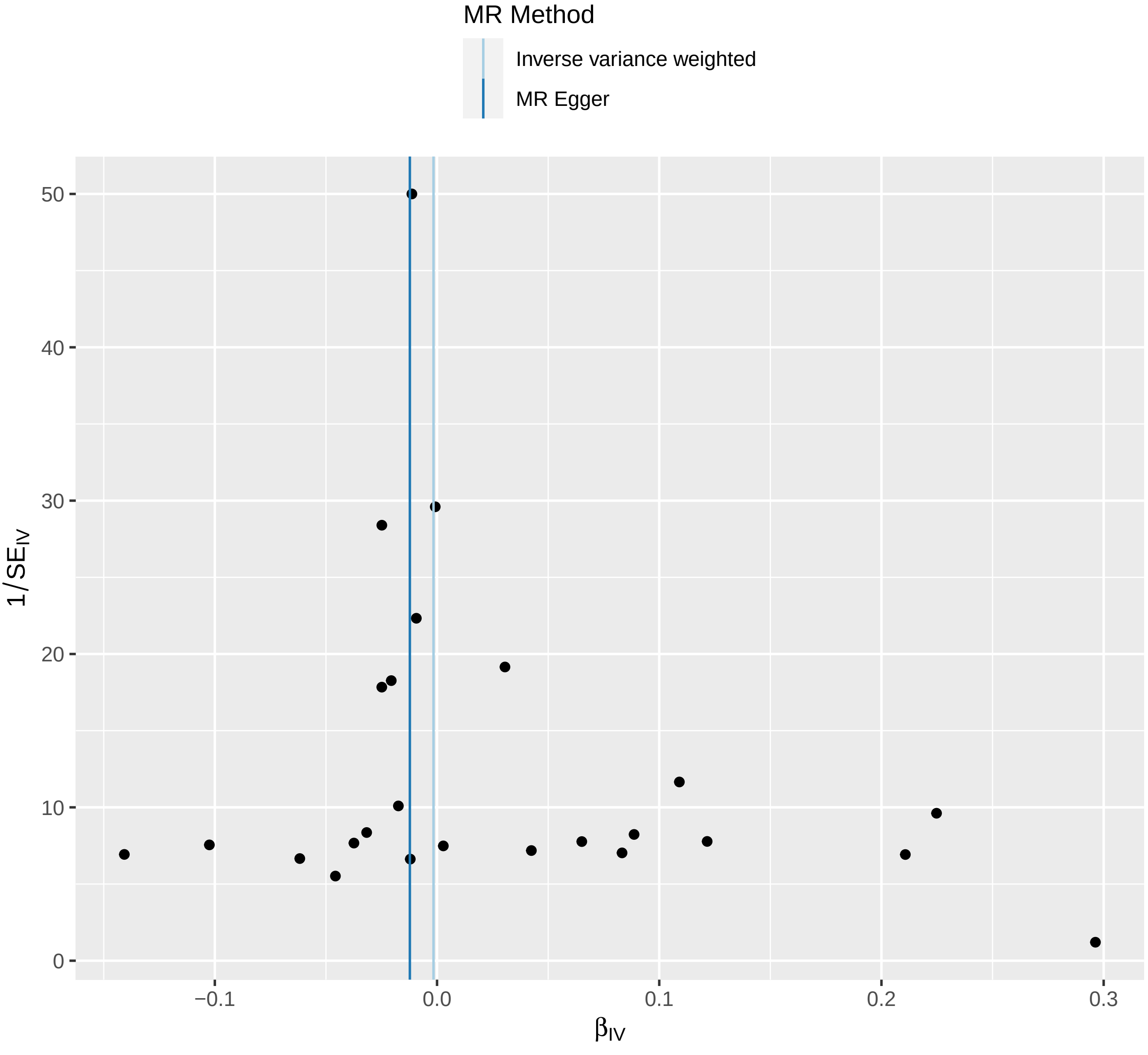

MR Method
Inverse variance weighted
MR Egger
1/SE$_{IV}$
β_{IV}
50
40
30
20
10
0
−0.1
0.0
0.1
0.2
0.3

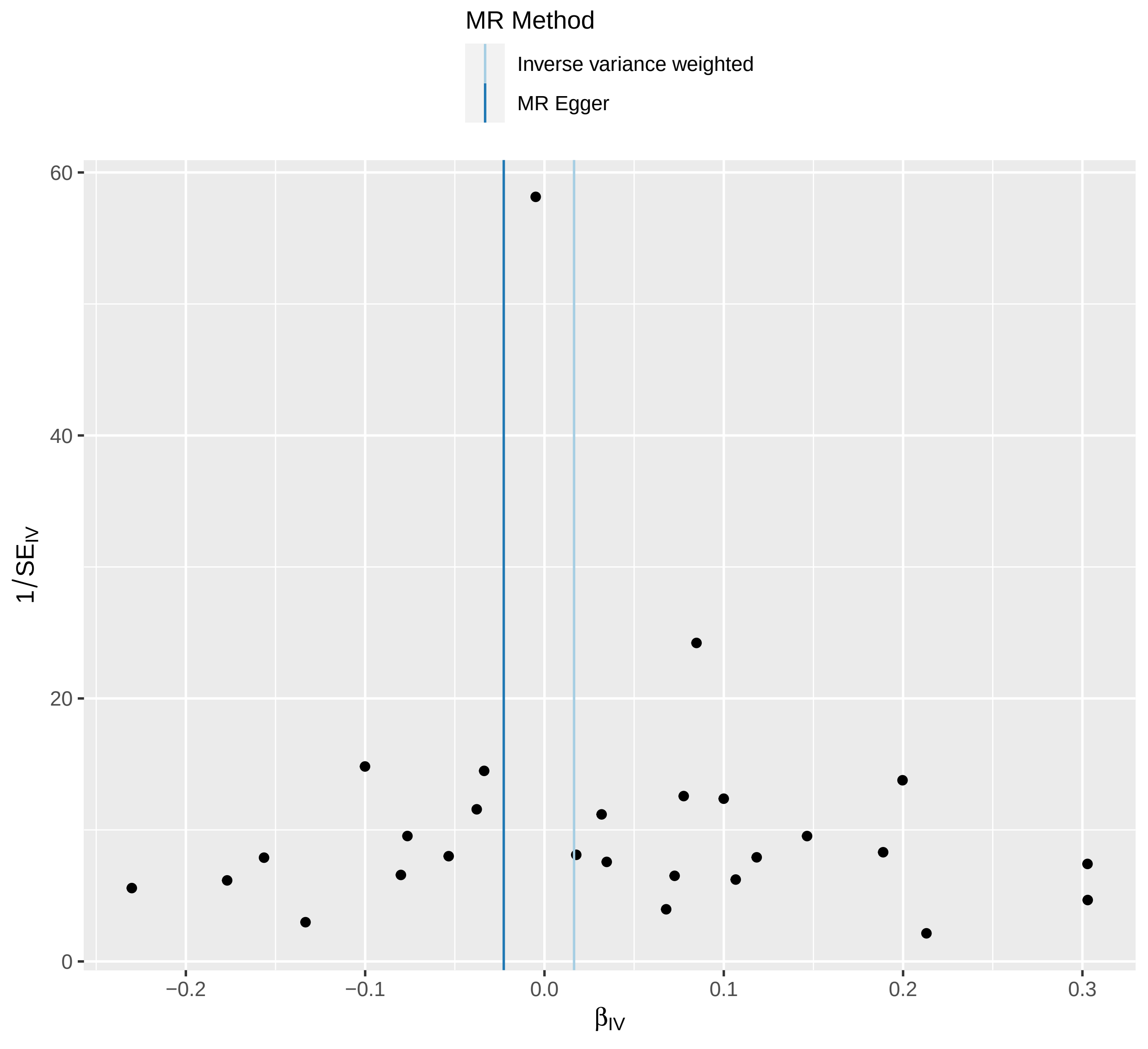
MR Method
Inverse variance weighted
MR Egger
60
40
20
0
1/SE$_{IV}$
−0.2
−0.1
0.0
0.1
0.2
0.3
β$_{IV}$

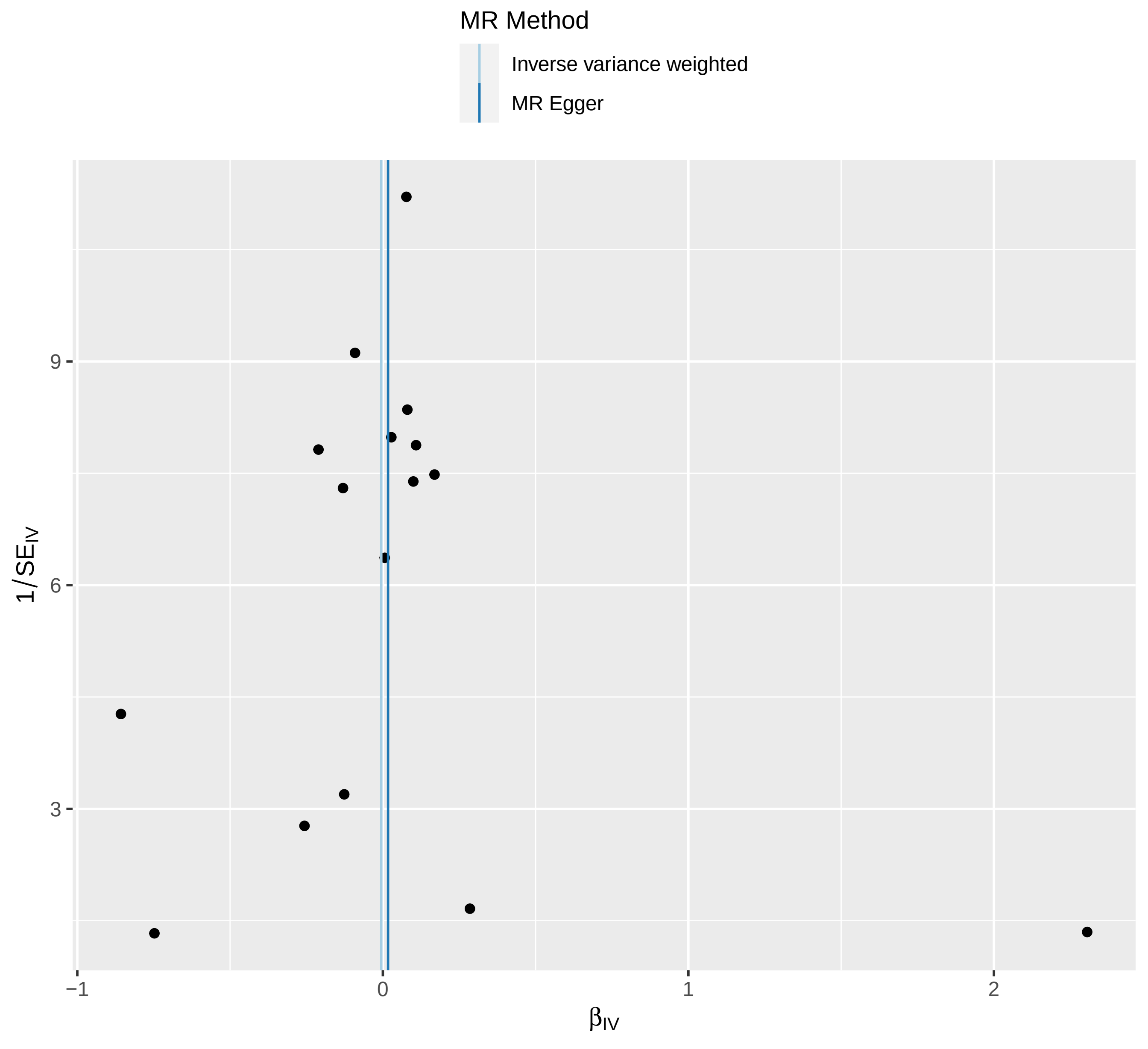

MR Method
Inverse variance weighted
MR Egger
9
6
3
1/SE$_{IV}$
−1
0
1
2
β$_{IV}$

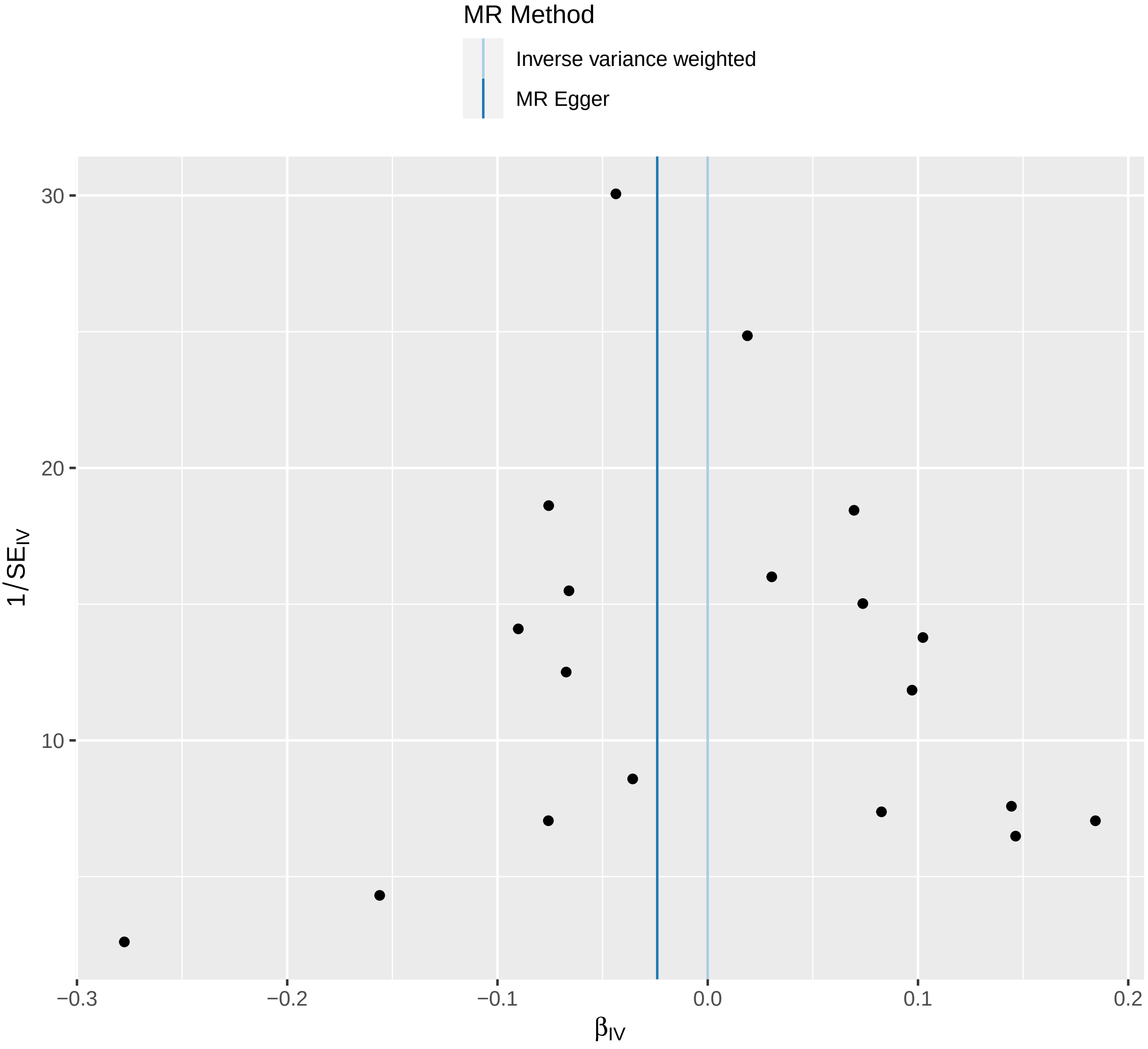
MR Method
Inverse variance weighted
MR Egger
30
20
10
1/SE_IV
−0.3
−0.2
−0.1
0.0
0.1
0.2
β_IV

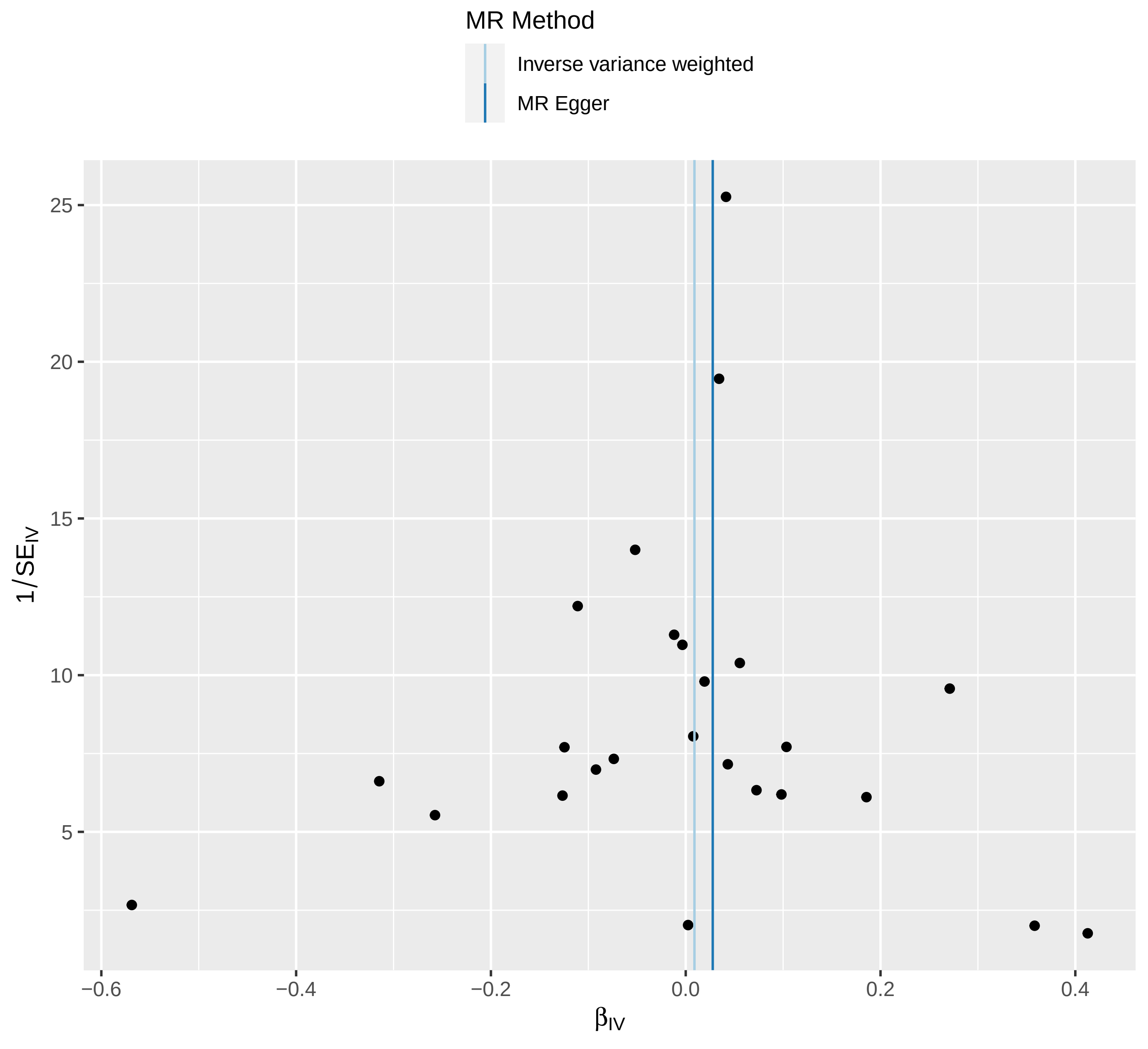

MR Method
Inverse variance weighted
MR Egger
25
20
15
10
5
$1/SE_{IV}$
−0.6
−0.4
−0.2
0.0
0.2
0.4
β_{IV}

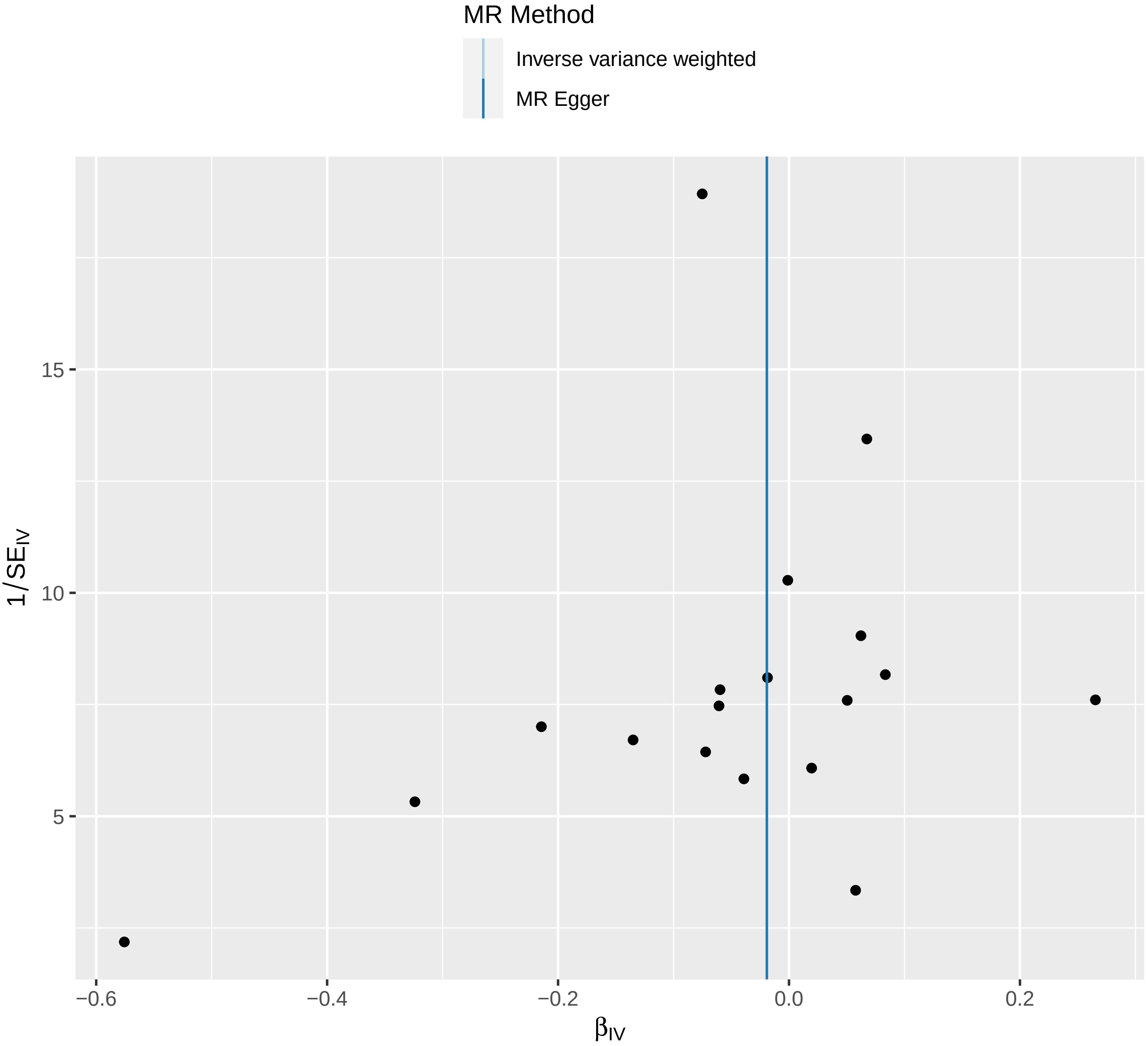
MR Method
Inverse variance weighted
MR Egger
15
10
5
1/SE_IV
−0.6
−0.4
−0.2
0.0
0.2
β_IV

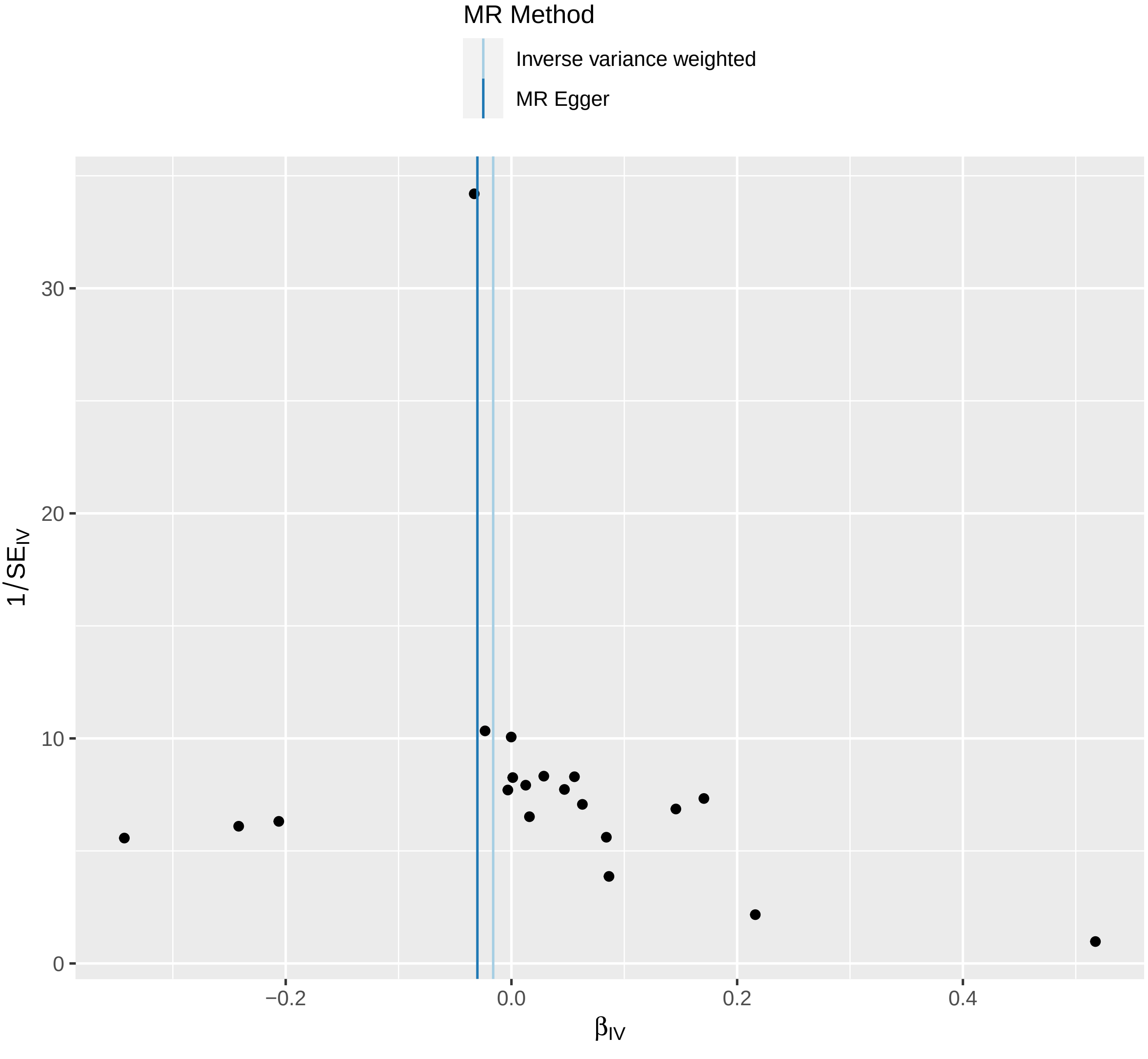
MR Method
Inverse variance weighted
MR Egger
$1/SE_{IV}$
β_{IV}
30
20
10
0
−0.2
0.0
0.2
0.4

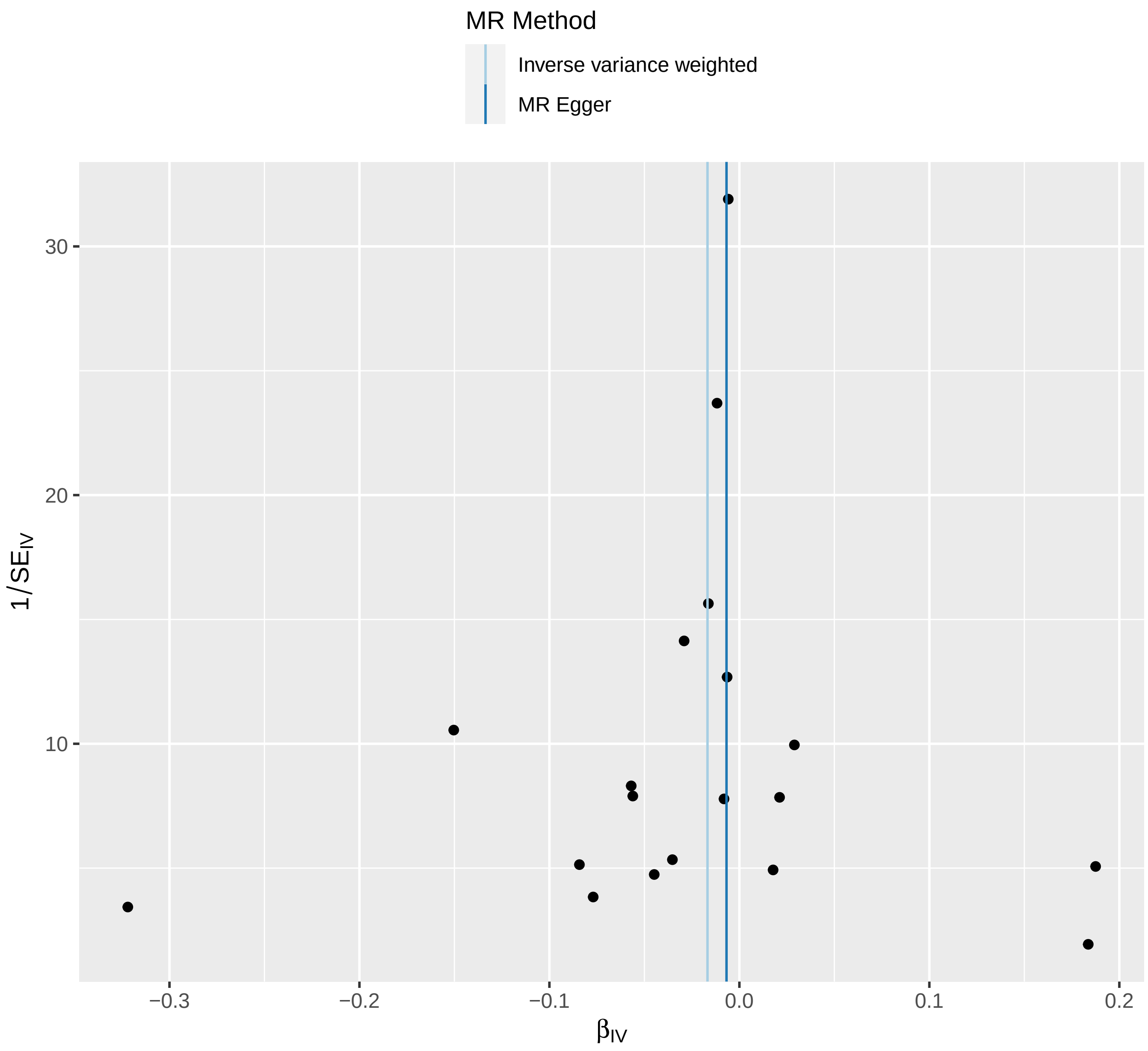
MR Method
Inverse variance weighted
MR Egger
30
20
10
1/SE$_{IV}$
−0.3
−0.2
−0.1
0.0
0.1
0.2
β$_{IV}$

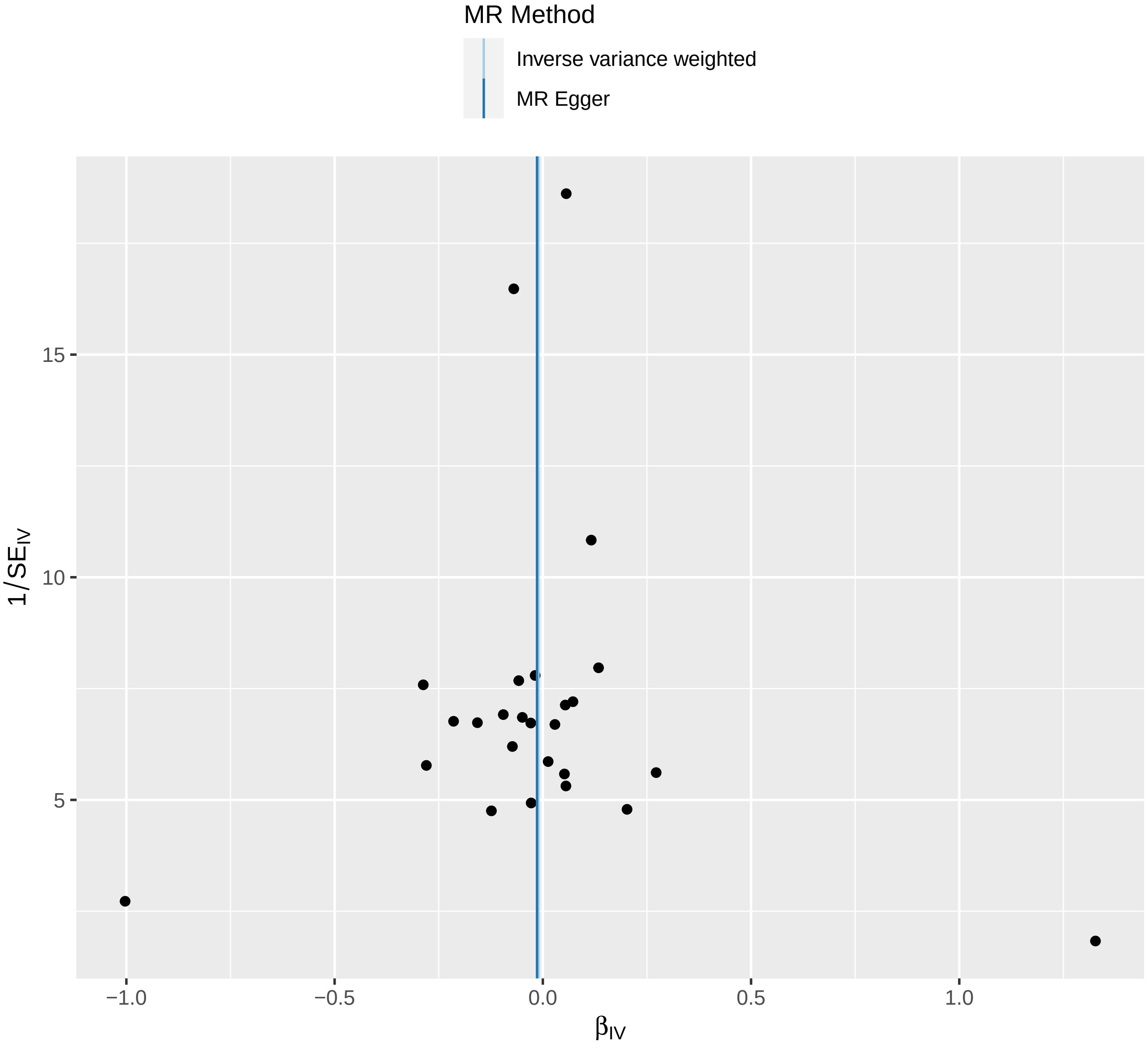

MR Method
Inverse variance weighted
MR Egger
15
10
5
1/SE_IV
−1.0
−0.5
0.0
0.5
1.0
β_IV

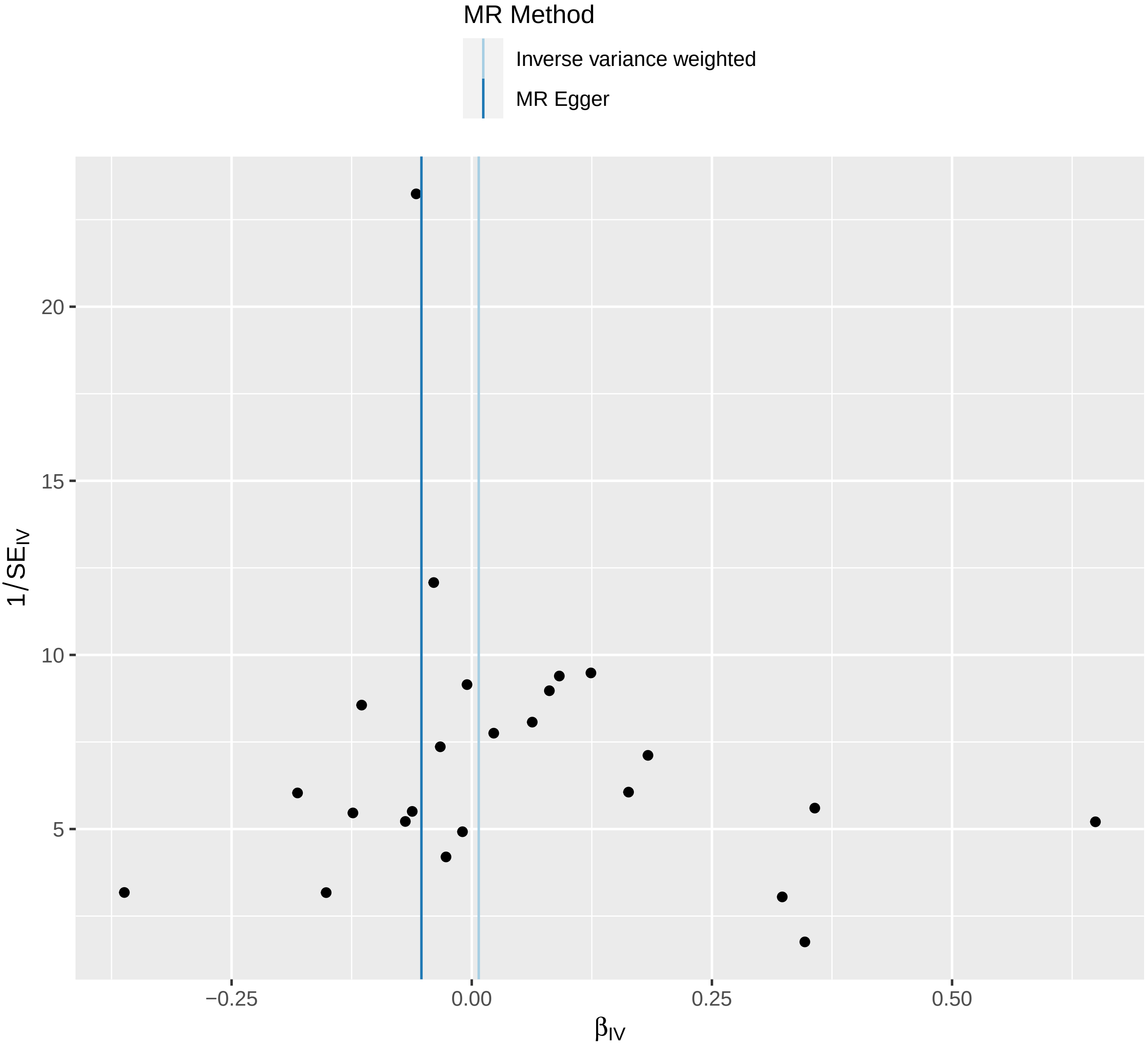
MR Method
Inverse variance weighted
MR Egger
20
15
10
5
−0.25
0.00
0.25
0.50
$1/SE_{IV}$
β_{IV}

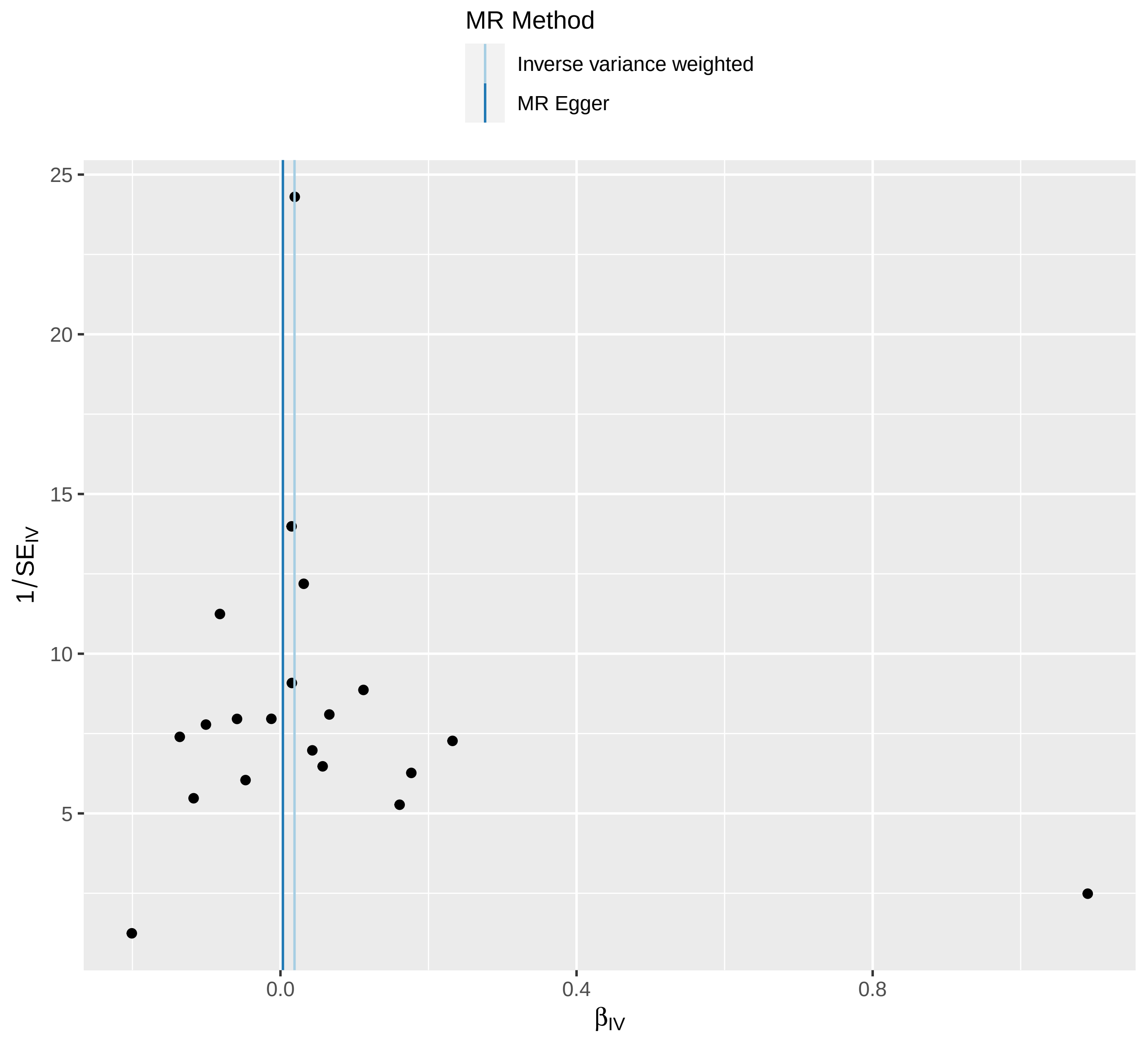

MR Method
Inverse variance weighted
MR Egger
25
20
15
10
5
1/SE_IV
0.0
0.4
0.8
β_IV

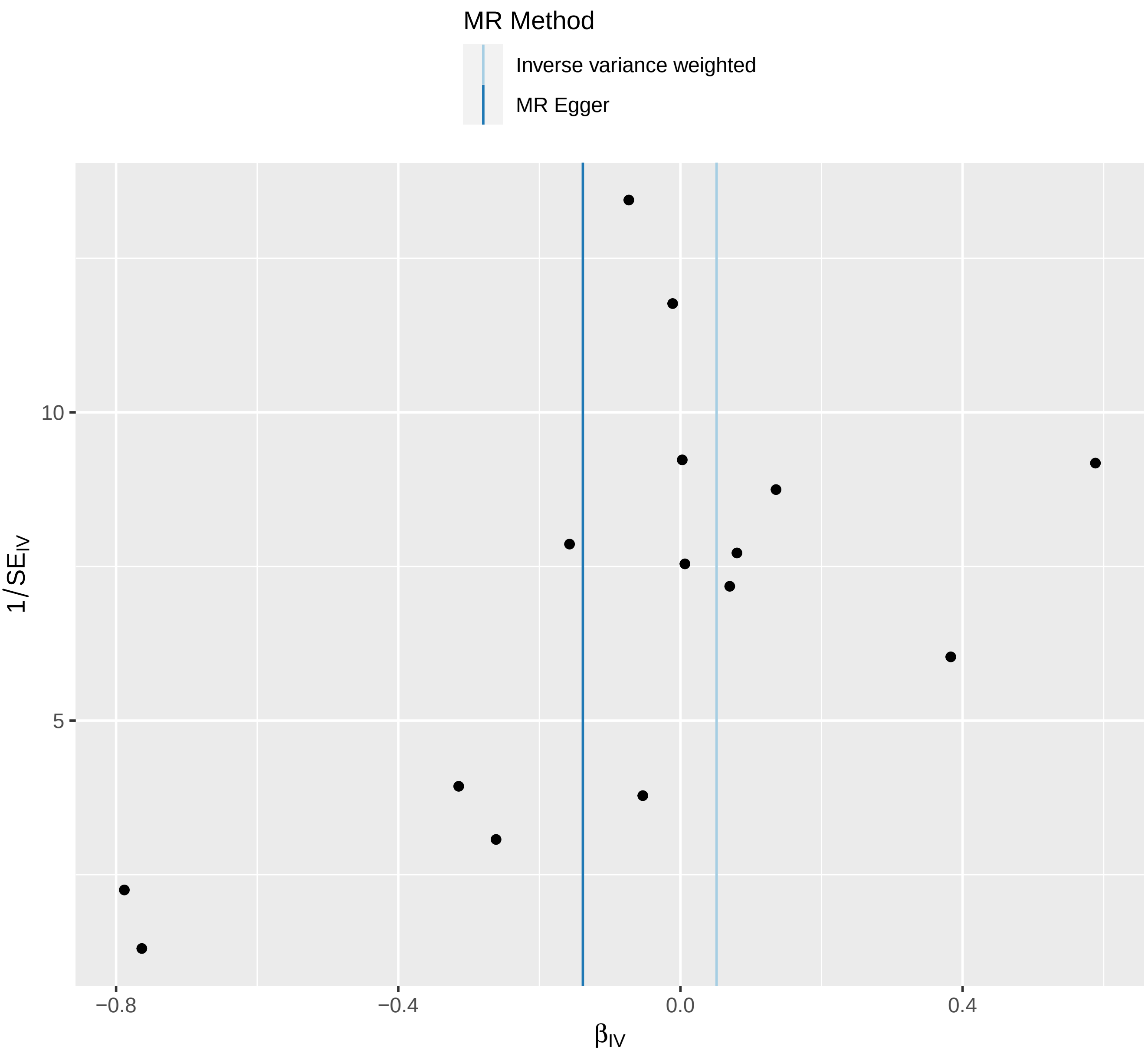
MR Method
Inverse variance weighted
MR Egger
10
5
1/SE_IV
−0.8
−0.4
0.0
0.4
β_IV

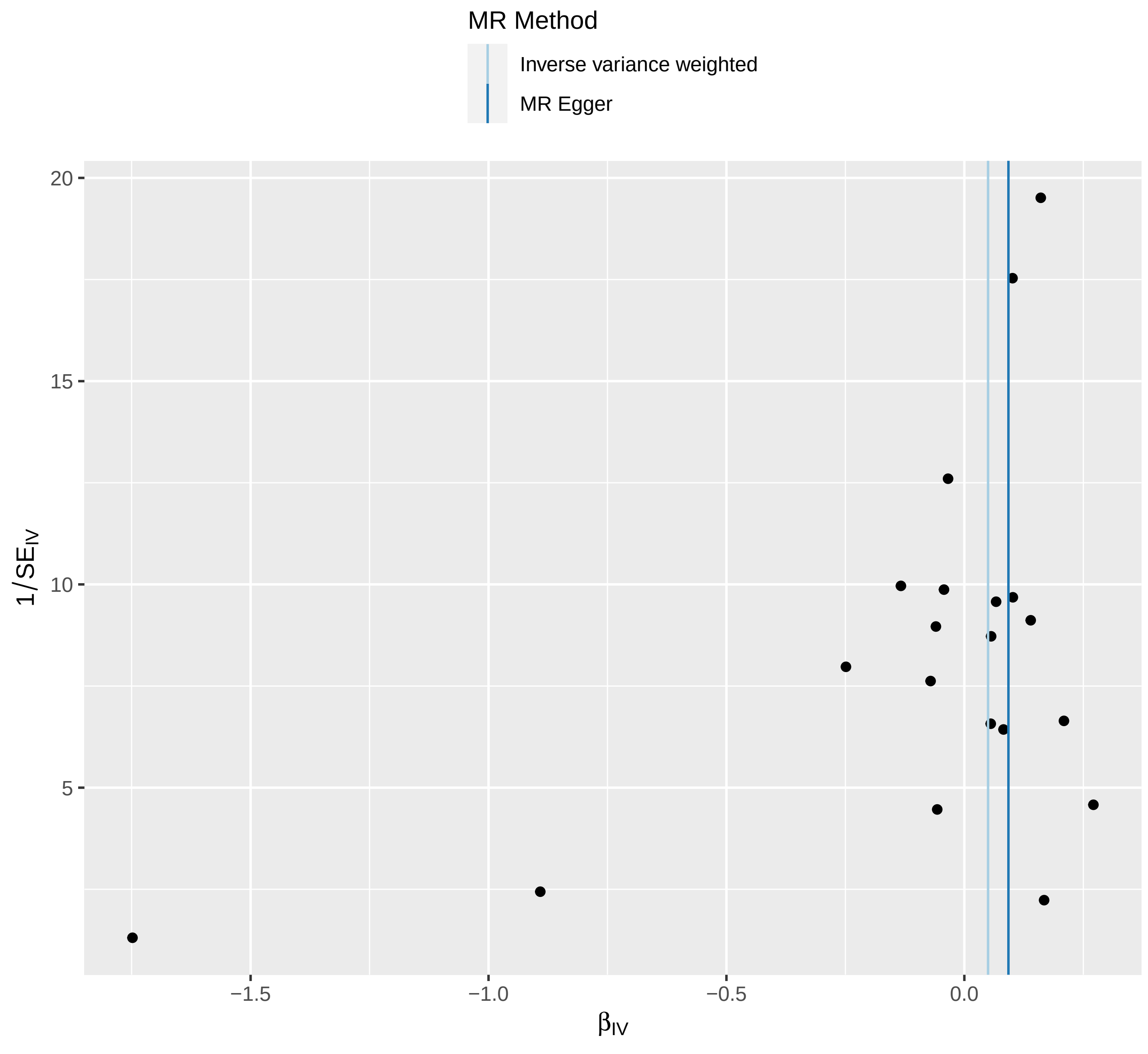

MR Method
Inverse variance weighted
MR Egger
20
15
10
5
1/SE$_{IV}$
−1.5
−1.0
−0.5
0.0
β$_{IV}$

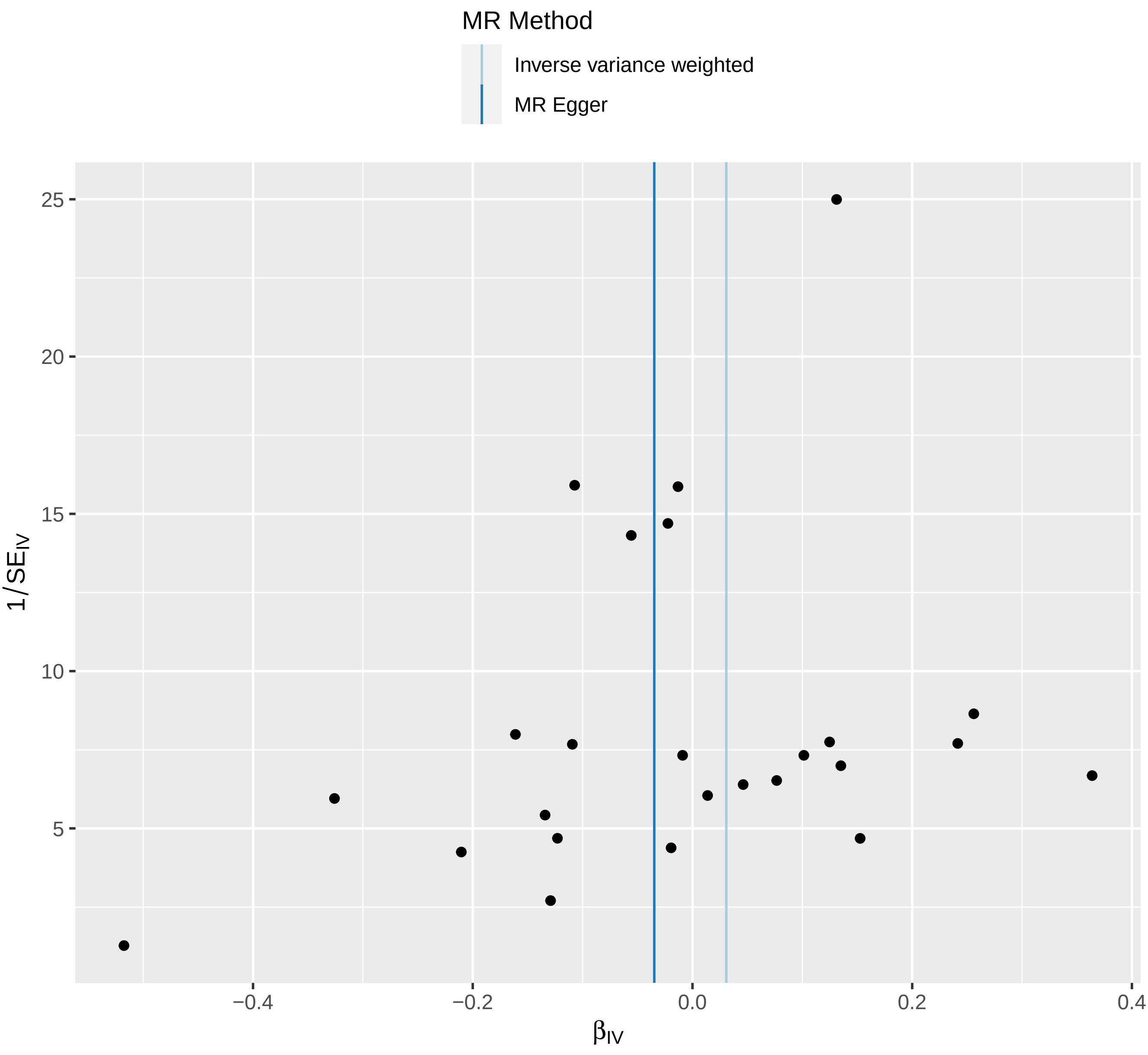

MR Method
Inverse variance weighted
MR Egger
25
20
15
10
5
1/SE_IV
−0.4
−0.2
0.0
0.2
0.4
β_IV

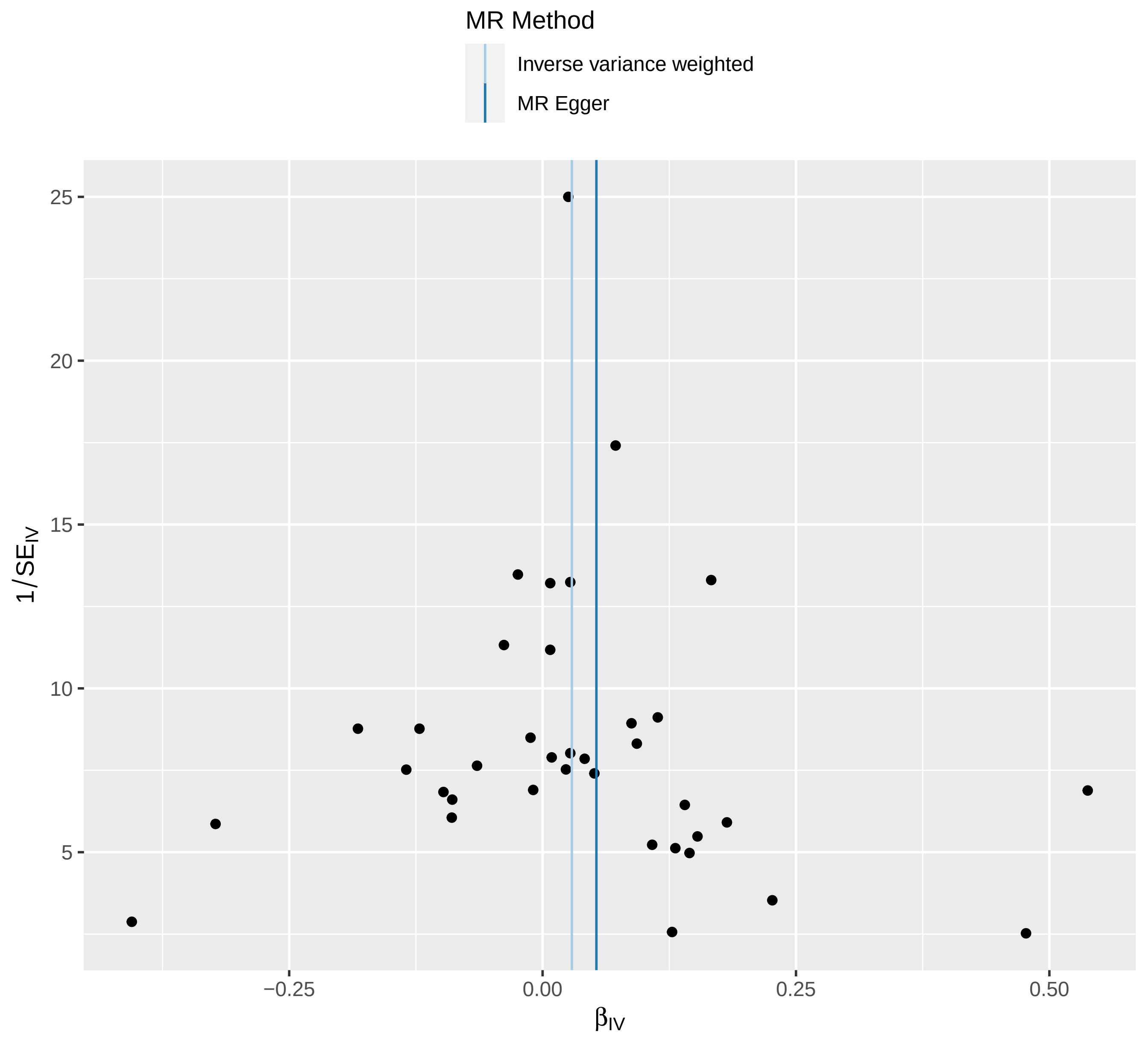
MR Method
Inverse variance weighted
MR Egger
25
20
15
10
5
1/SE_IV
−0.25
0.00
0.25
0.50
β_IV

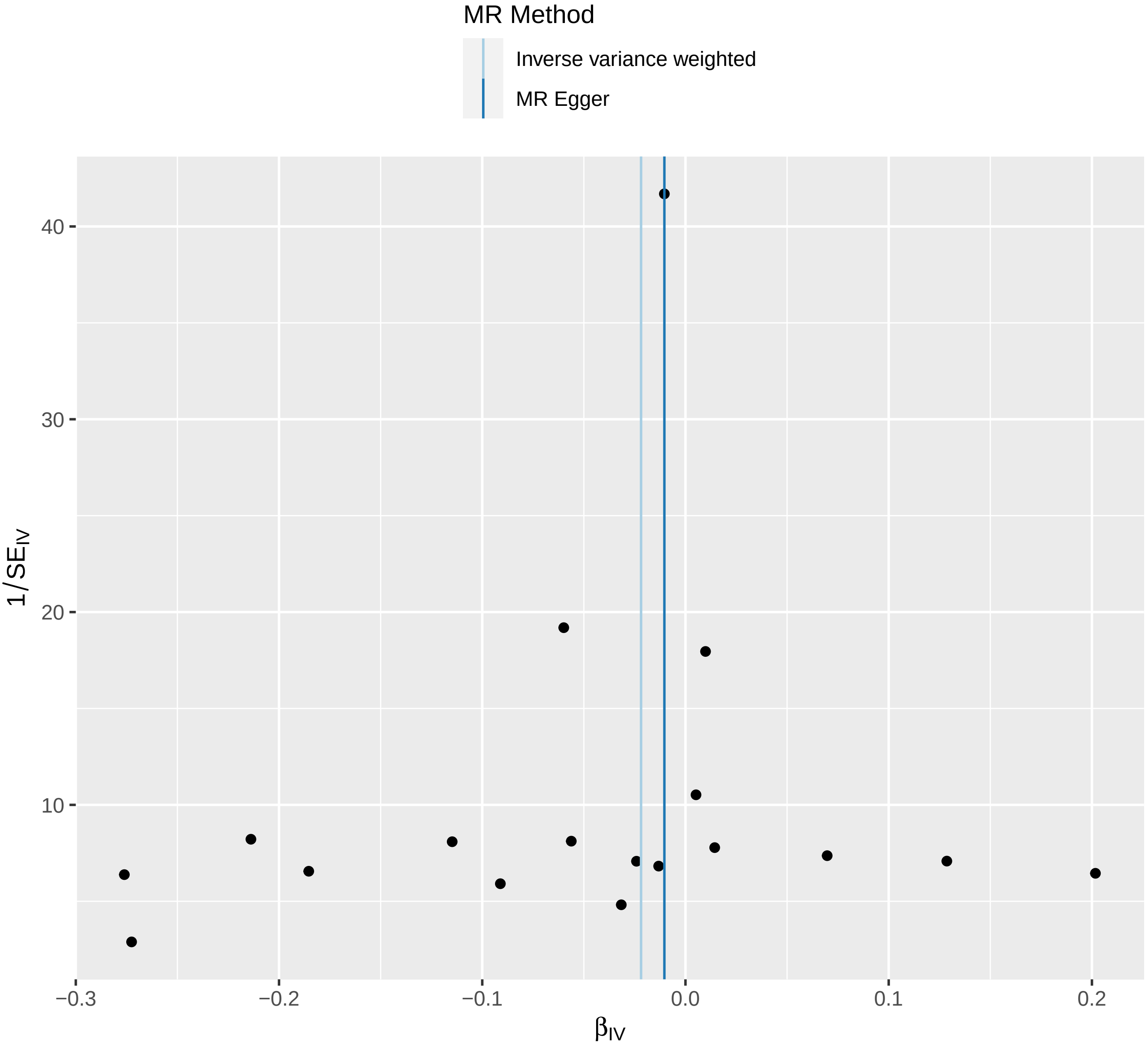
MR Method
Inverse variance weighted
MR Egger
40
30
20
10
1/SE_IV
−0.3
−0.2
−0.1
0.0
0.1
0.2
β_IV

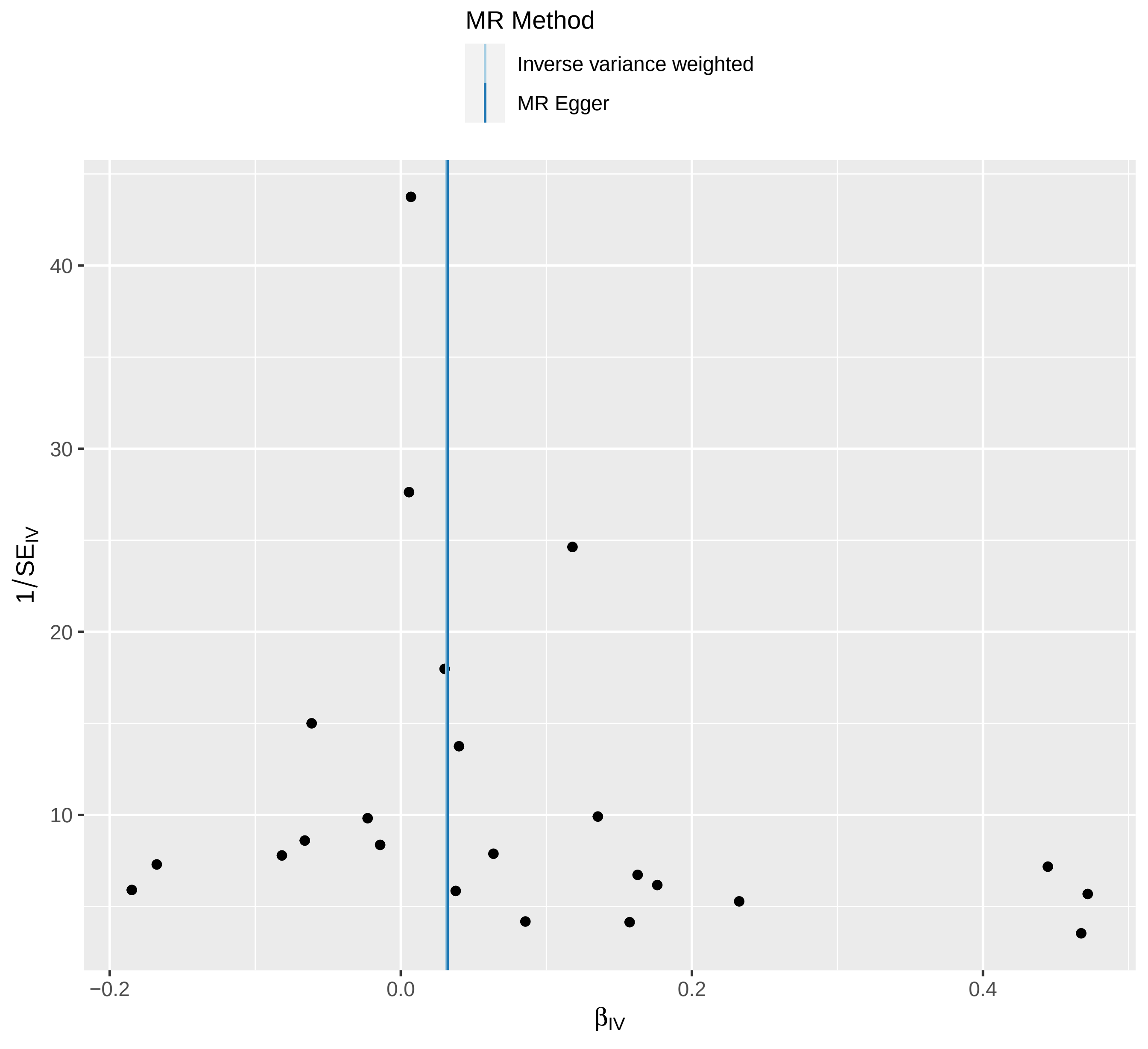

MR Method
Inverse variance weighted
MR Egger
40
30
20
10
$1/SE_{IV}$
−0.2
0.0
0.2
0.4
β_{IV}

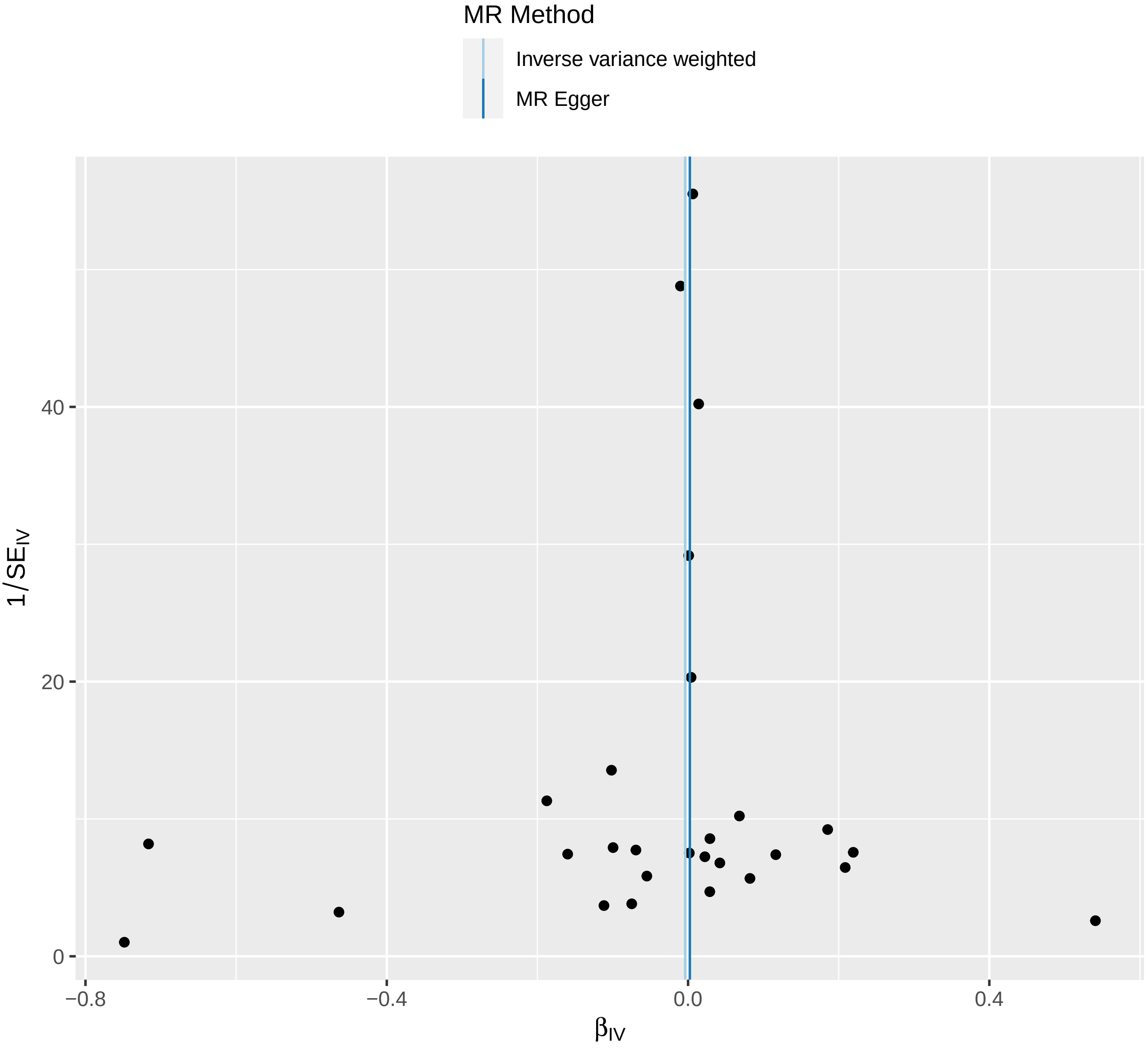

MR Method
Inverse variance weighted
MR Egger
40
20
0
1/SE$_{IV}$
−0.8
−0.4
0.0
0.4
β$_{IV}$

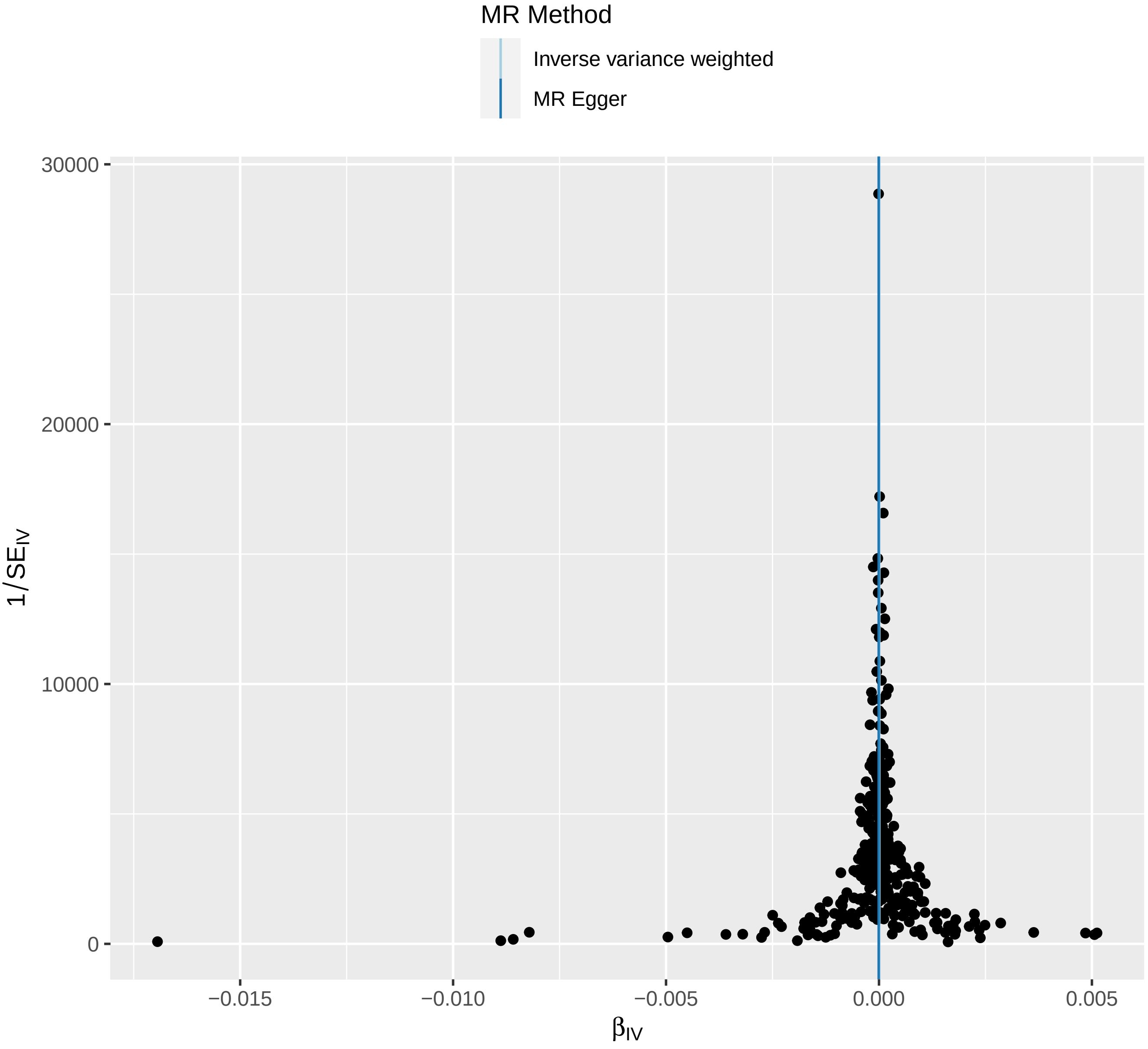
MR Method
Inverse variance weighted
MR Egger
30000
20000
10000
0
1/SE$_{IV}$
−0.015
−0.010
−0.005
0.000
0.005
β$_{IV}$

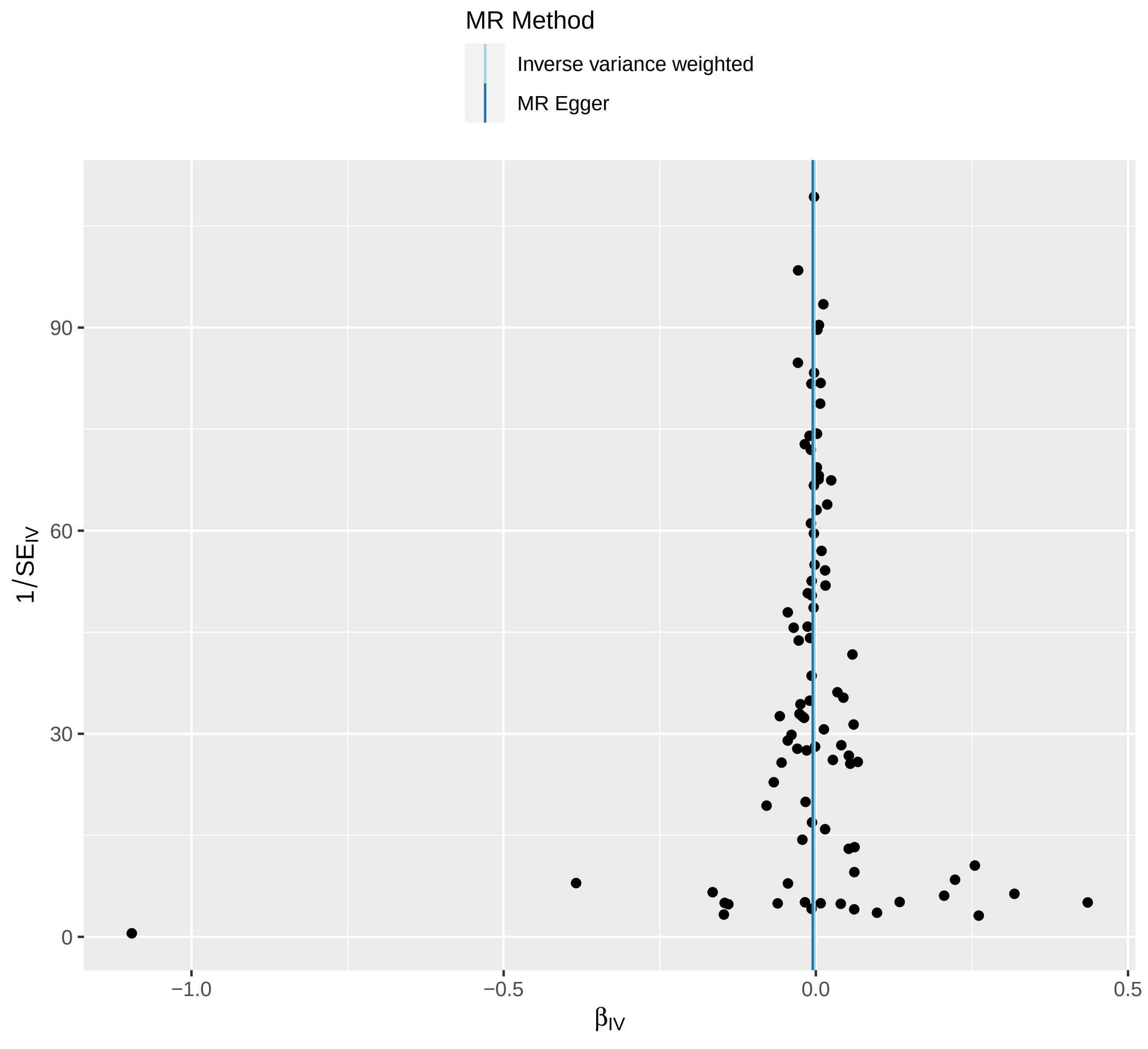
MR Method
Inverse variance weighted
MR Egger
90
60
30
0
$1/SE_{IV}$
−1.0
−0.5
0.0
0.5
β_{IV}

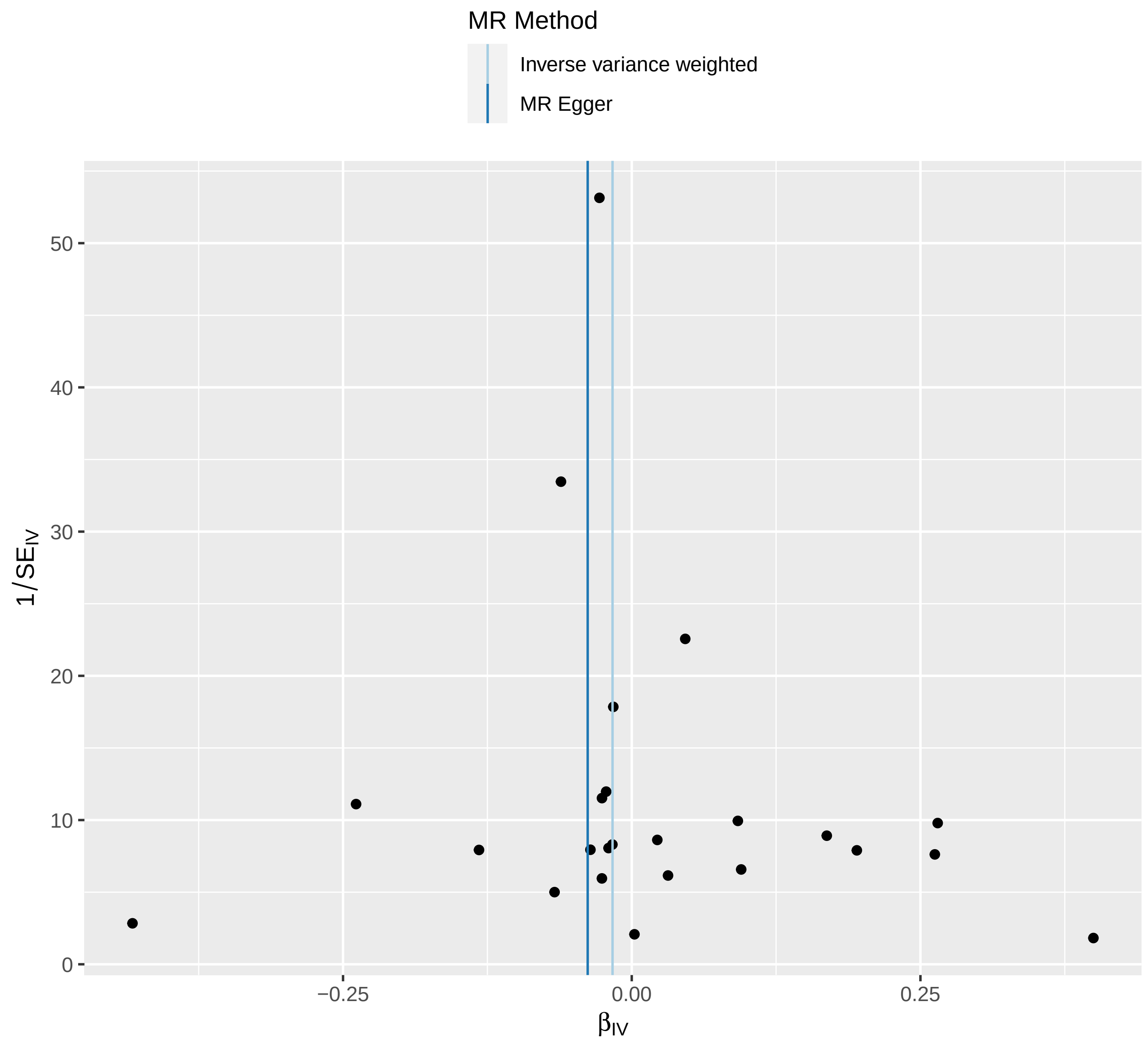

MR Method
Inverse variance weighted
MR Egger
1/SE$_{IV}$
50
40
30
20
10
0
−0.25
0.00
0.25
β$_{IV}$

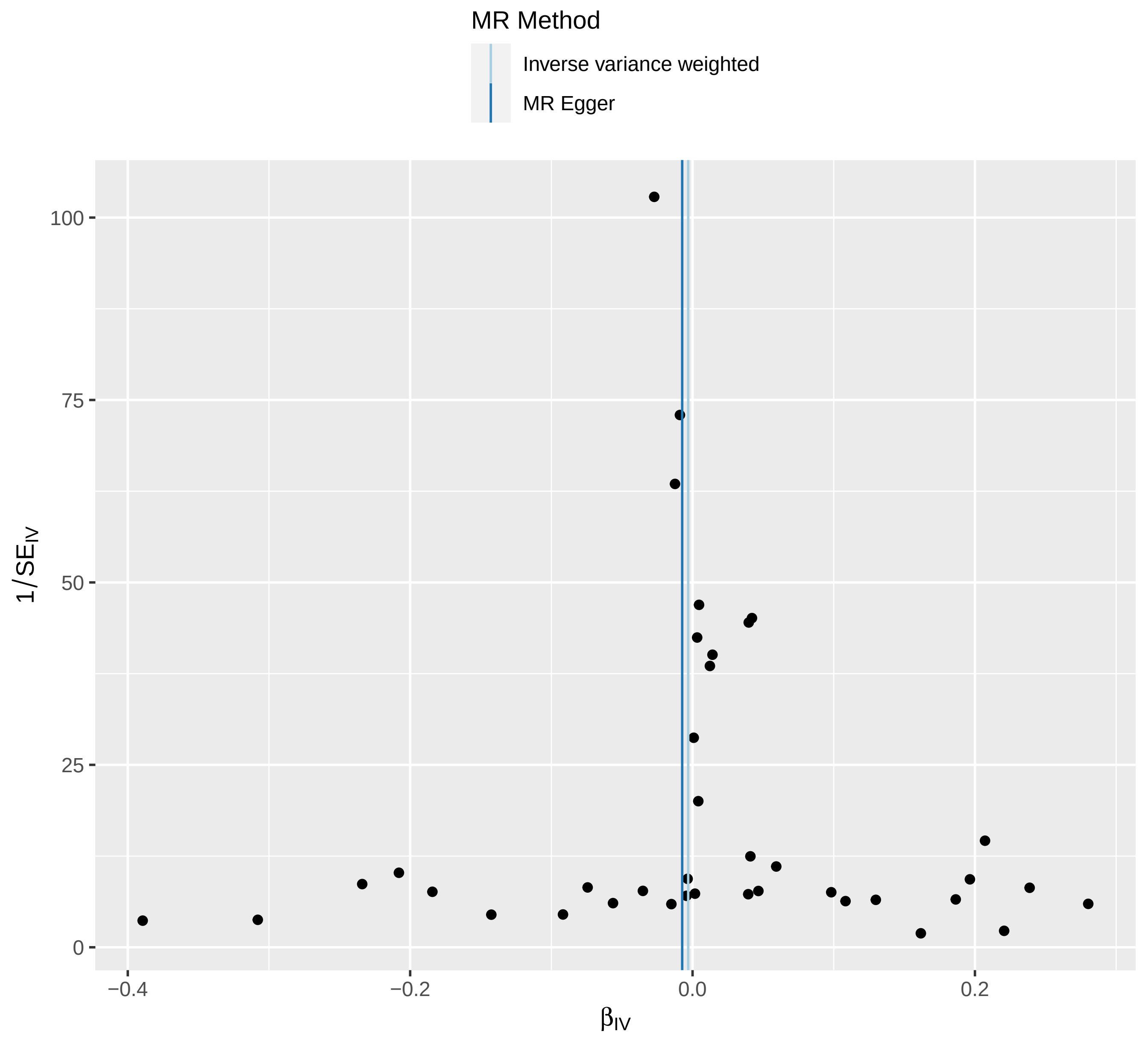

MR Method
Inverse variance weighted
MR Egger
1/SE_IV
β_IV
100
75
50
25
0
−0.4
−0.2
0.0
0.2

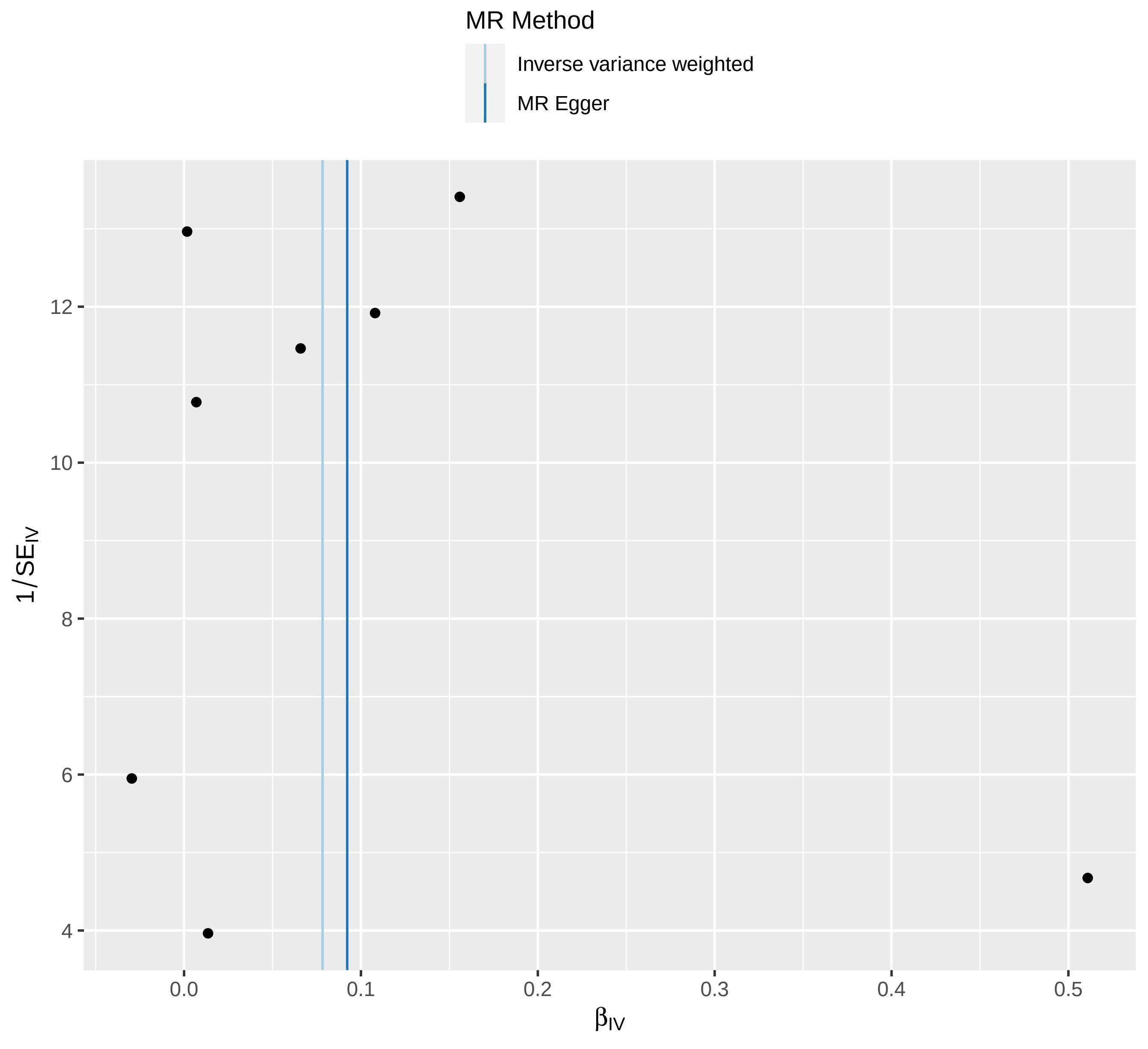

MR Method
Inverse variance weighted
MR Egger
12
10
8
6
4
1/SE$_{IV}$
0.0
0.1
0.2
0.3
0.4
0.5
β$_{IV}$

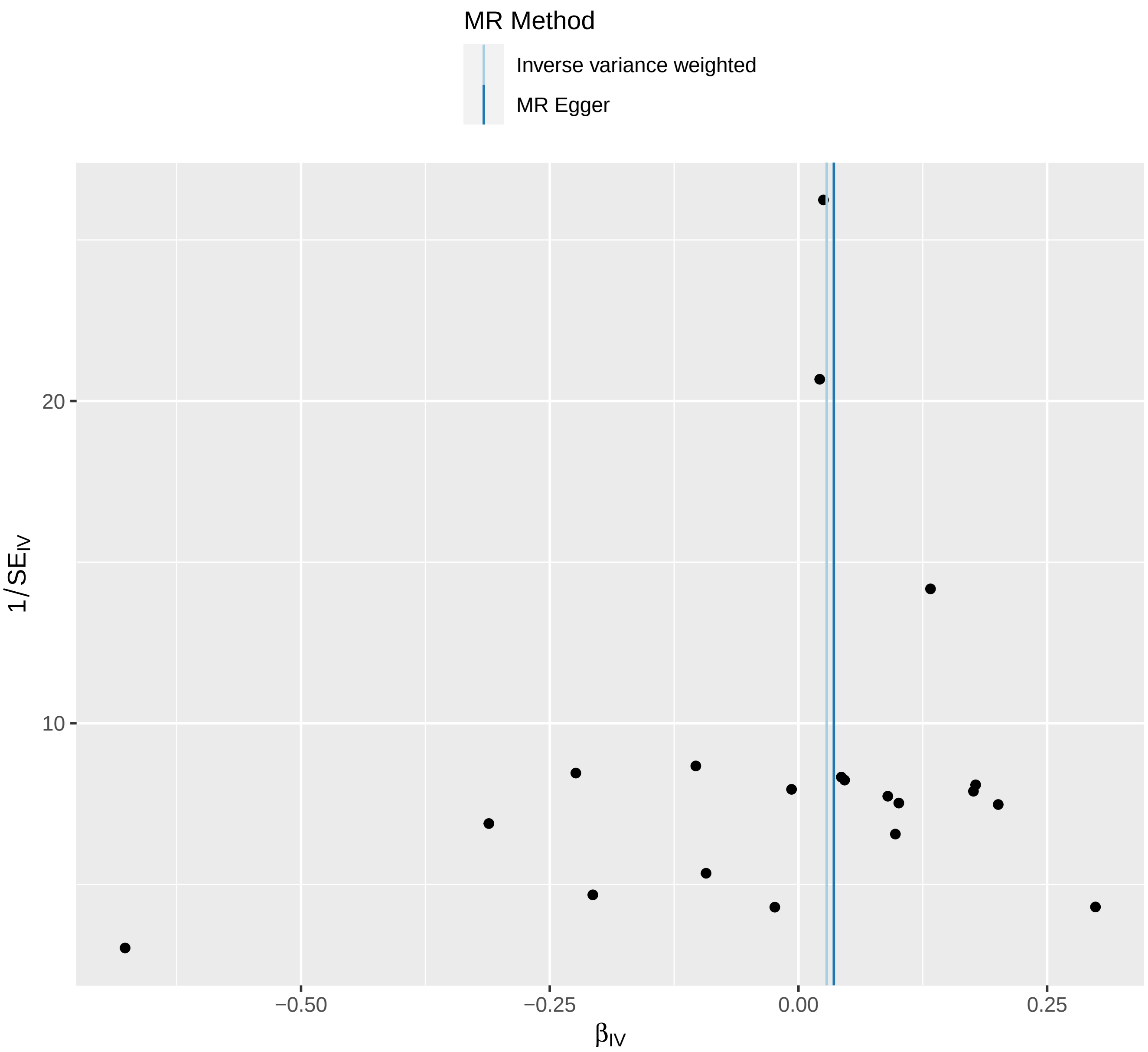

MR Method
Inverse variance weighted
MR Egger
20
10
1/SE_IV
−0.50
−0.25
0.00
0.25
β_IV

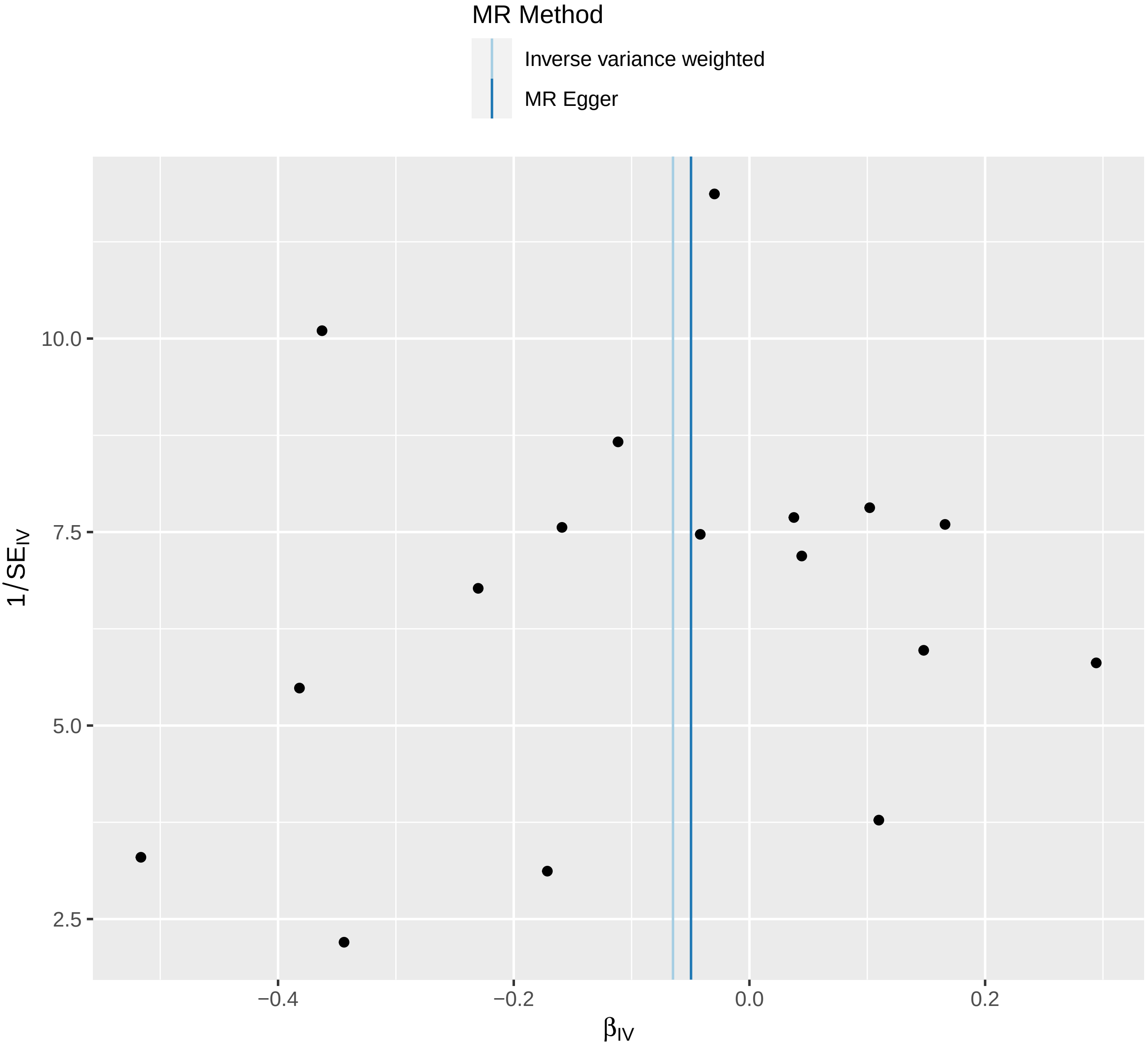

MR Method
Inverse variance weighted
MR Egger
10.0
7.5
5.0
2.5
$1/SE_{IV}$
−0.4
−0.2
0.0
0.2
β_{IV}

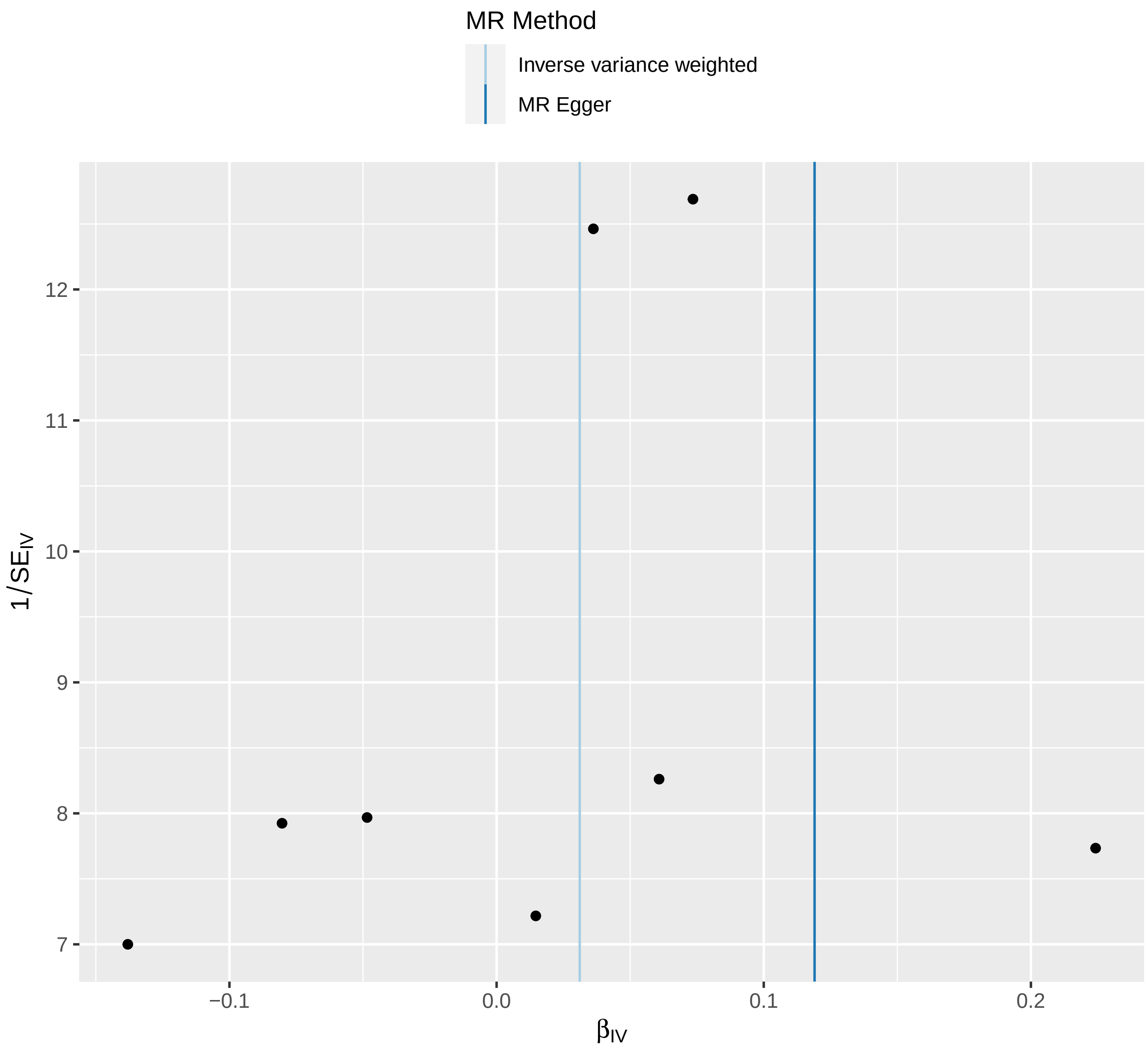
MR Method
Inverse variance weighted
MR Egger
12
11
10
9
8
7
1/SE_IV
−0.1
0.0
0.1
0.2
β_IV

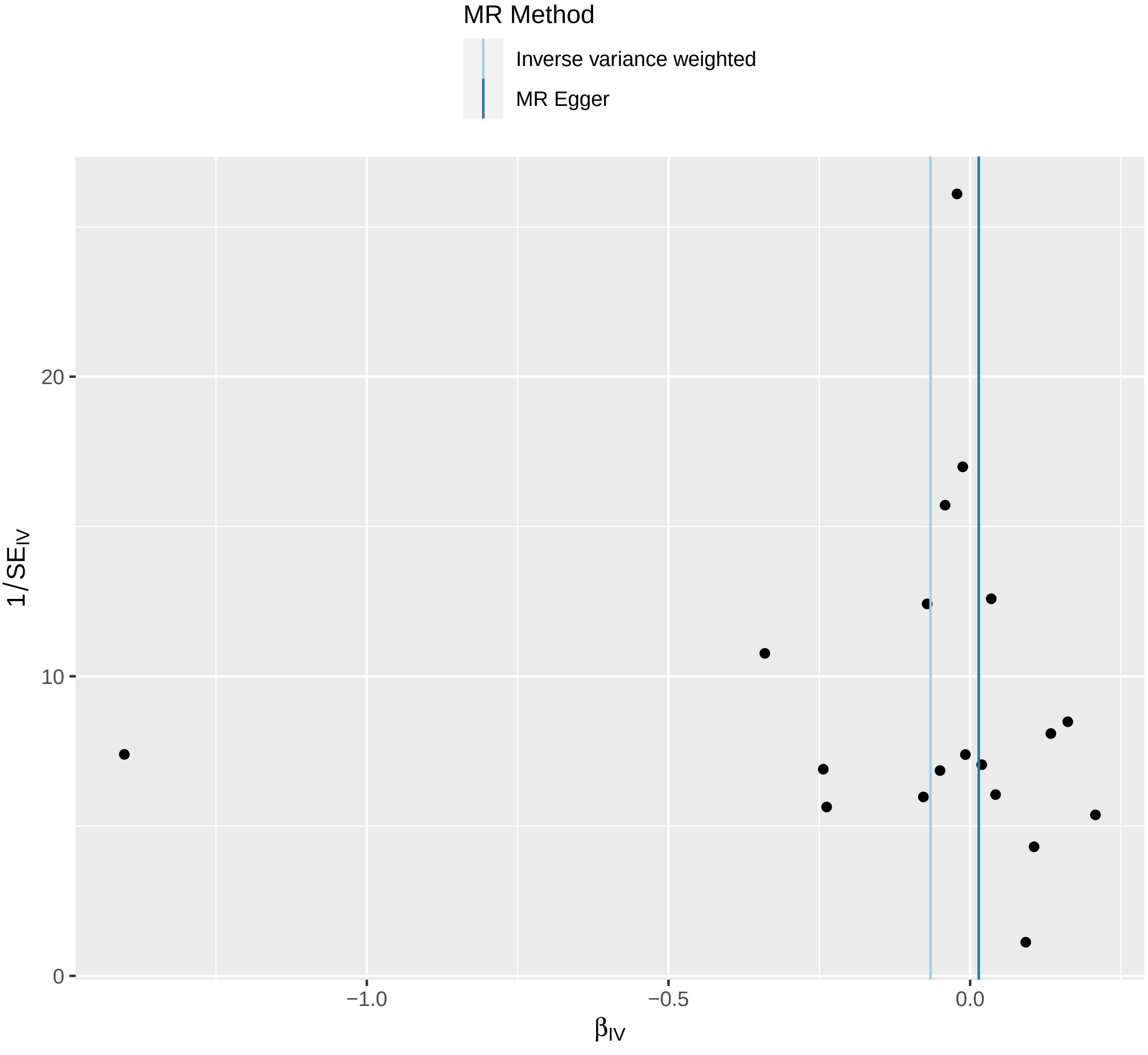

MR Method
Inverse variance weighted
MR Egger
20
10
0
1/SE$_{IV}$
−1.0
−0.5
0.0
β$_{IV}$

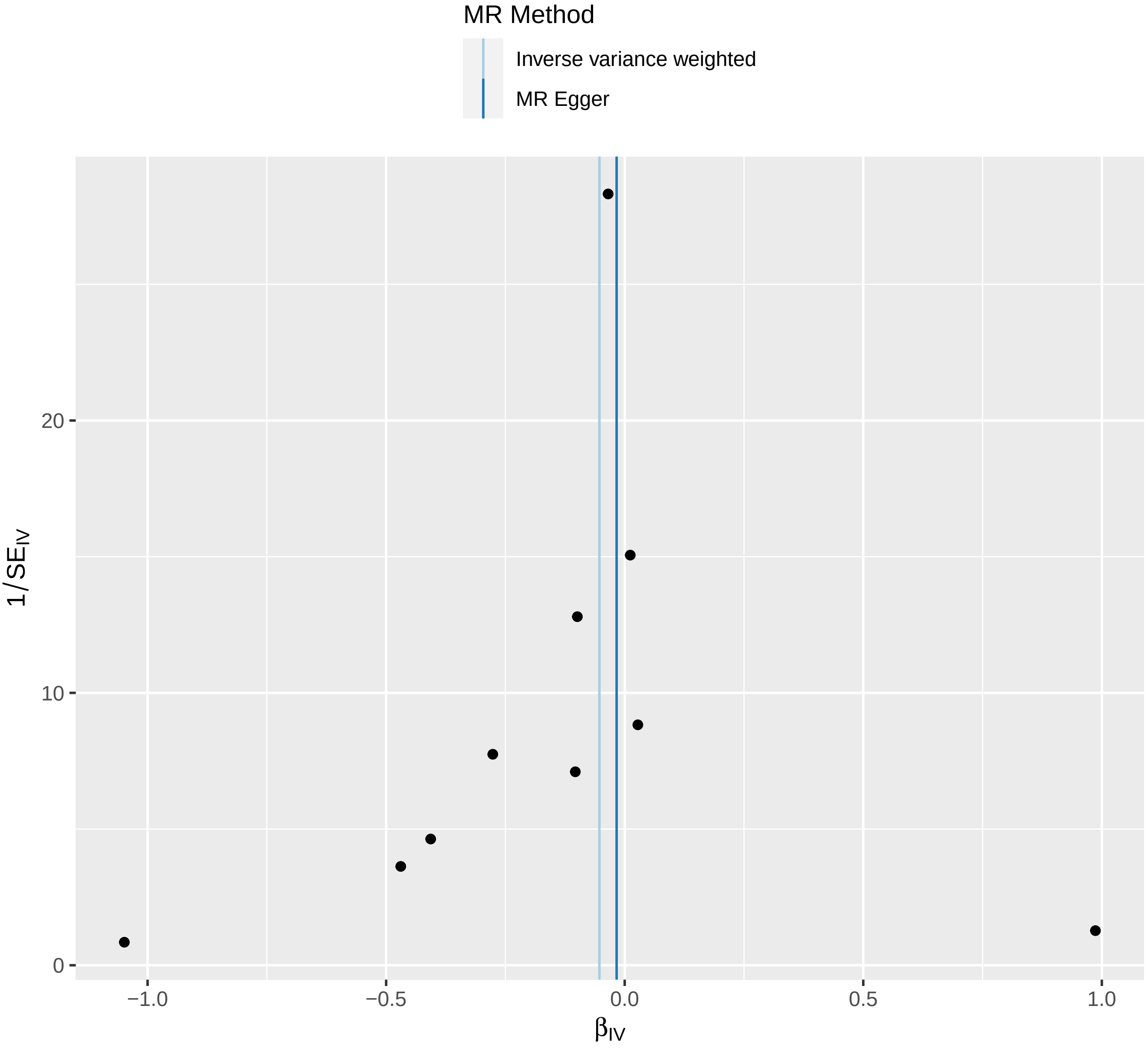

MR Method
Inverse variance weighted
MR Egger
20
10
0
−1.0
−0.5
0.0
0.5
1.0
$1/SE_{IV}$
β_{IV}

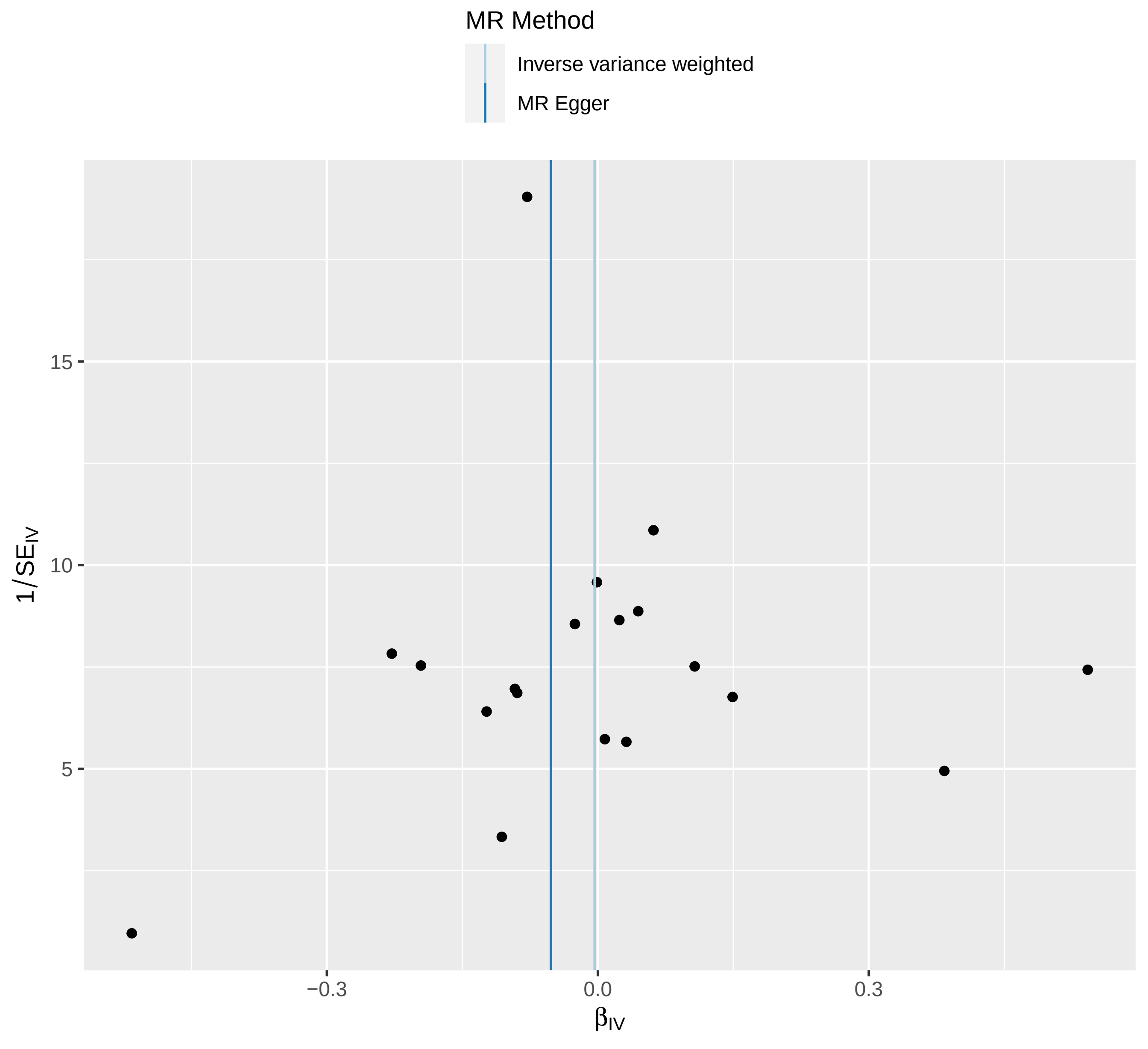

MR Method
Inverse variance weighted
MR Egger
15
10
5
1/SE_IV
−0.3
0.0
0.3
β_IV

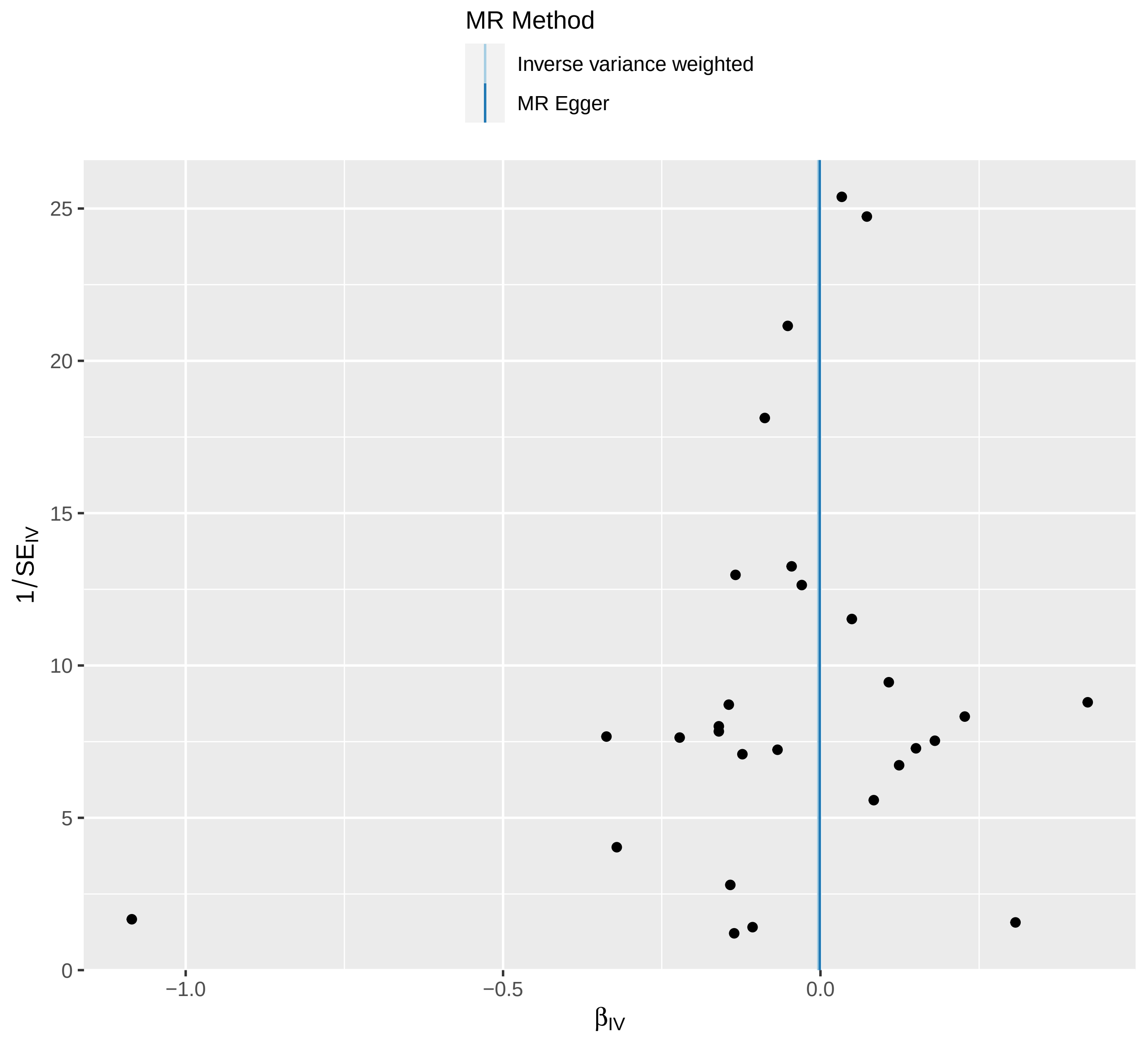
MR Method
Inverse variance weighted
MR Egger
1/SE$_{IV}$
25
20
15
10
5
0
−1.0
−0.5
0.0
β$_{IV}$

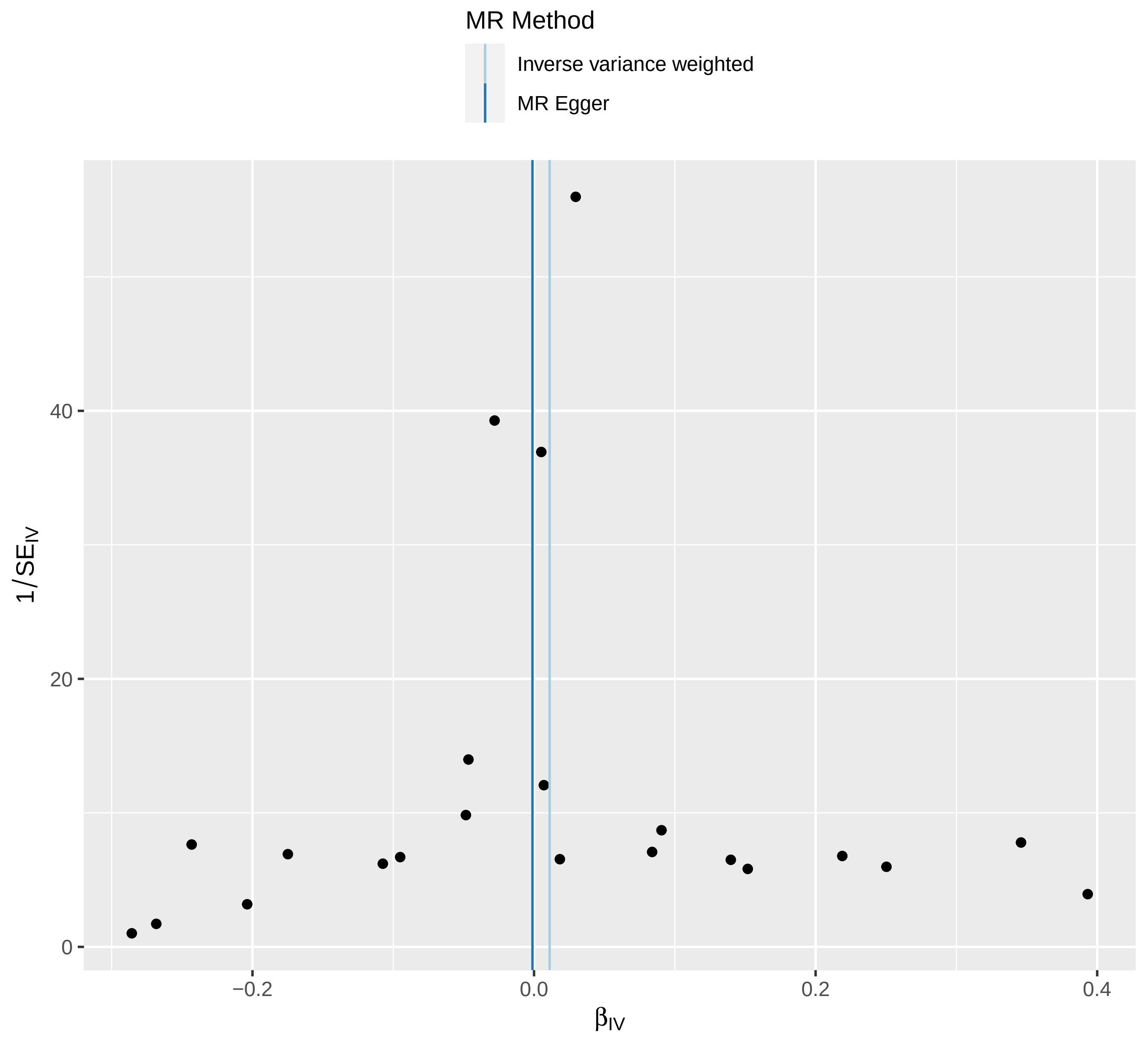

MR Method
Inverse variance weighted
MR Egger
40
20
0
1/SE$_{IV}$
−0.2
0.0
0.2
0.4
β$_{IV}$

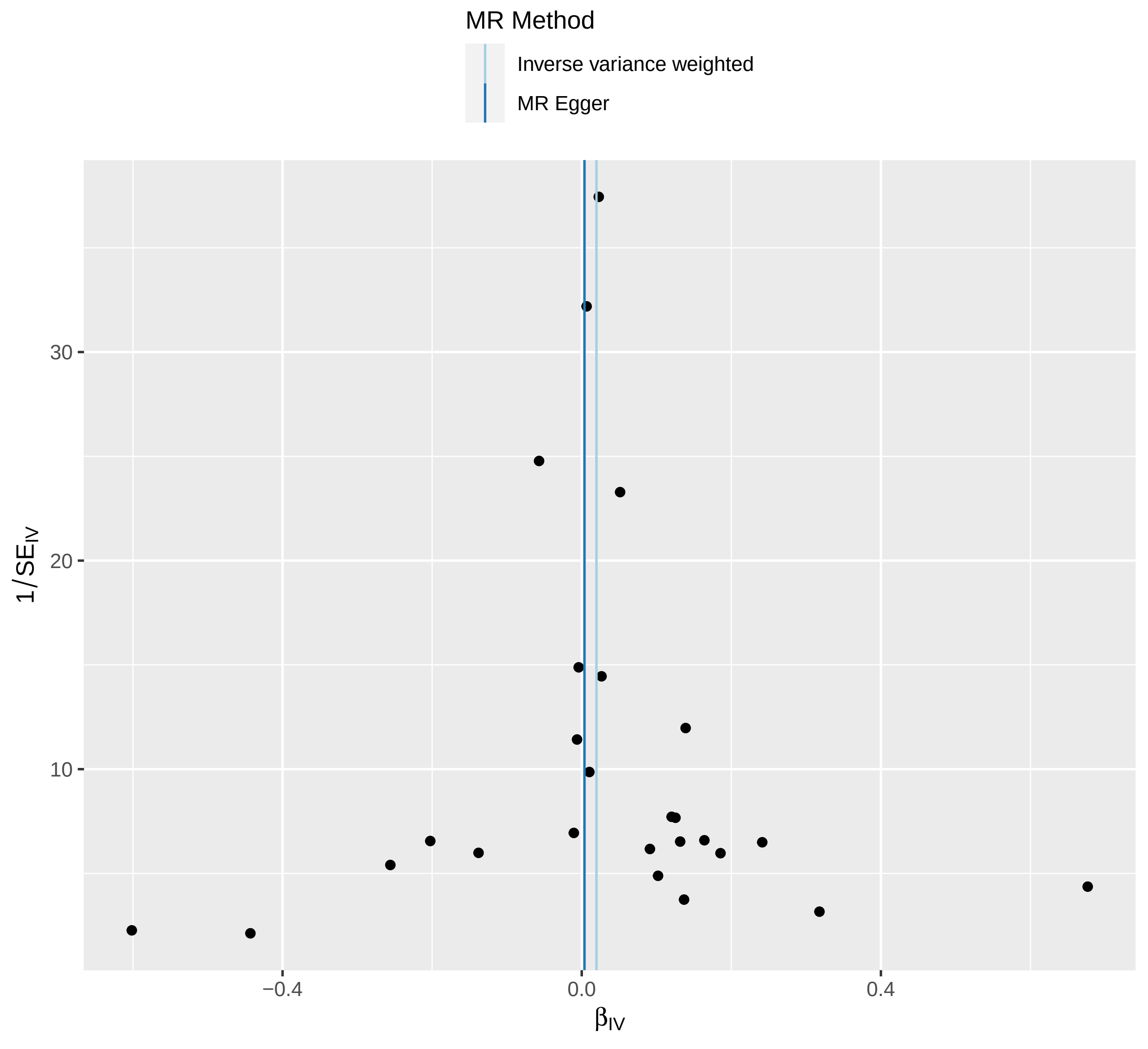
MR Method
Inverse variance weighted
MR Egger
30
20
10
1/SE$_{IV}$
−0.4
0.0
0.4
β$_{IV}$

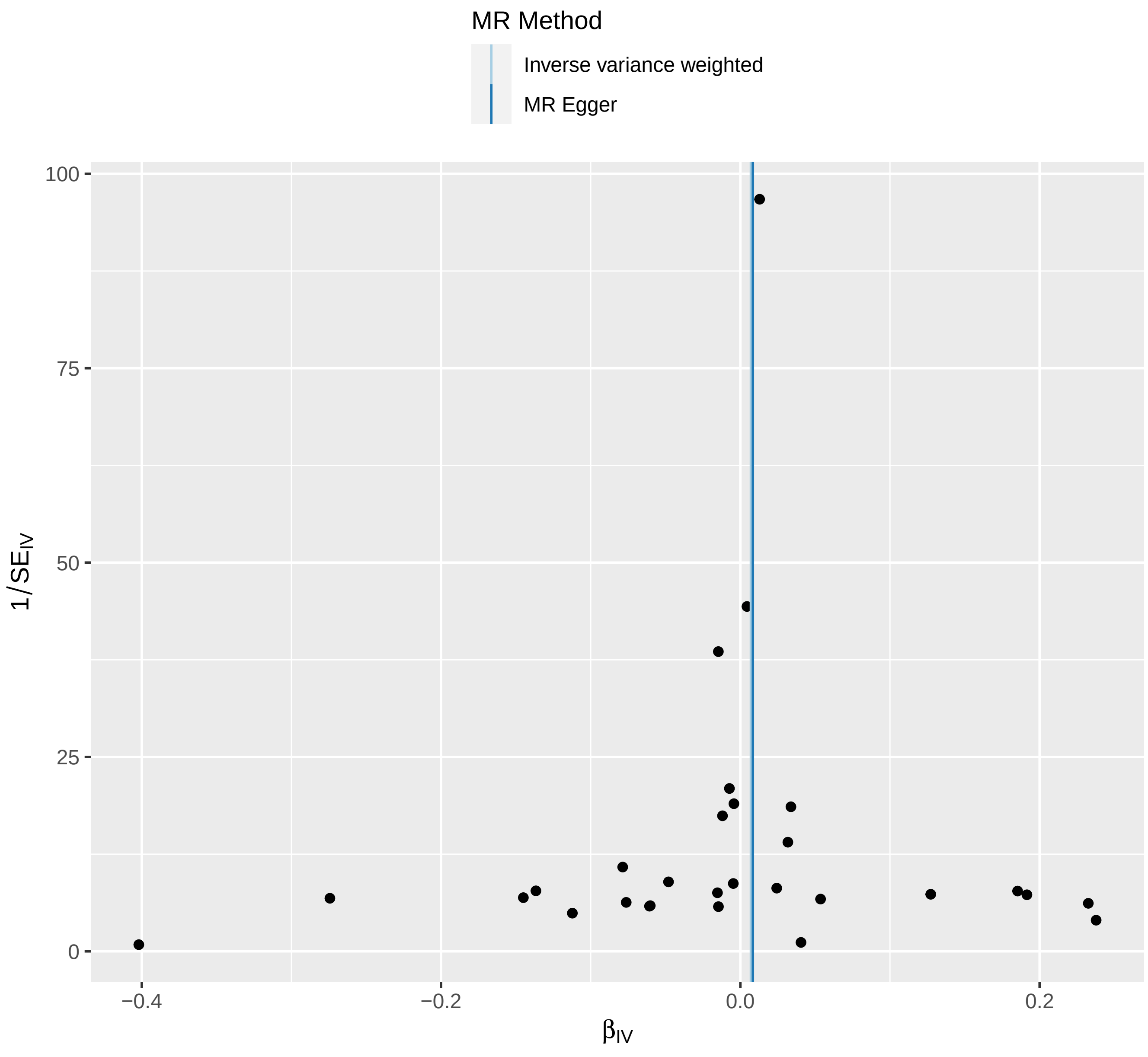

MR Method
Inverse variance weighted
MR Egger
100
75
50
25
0
1/SE$_{IV}$
−0.4
−0.2
0.0
0.2
β$_{IV}$

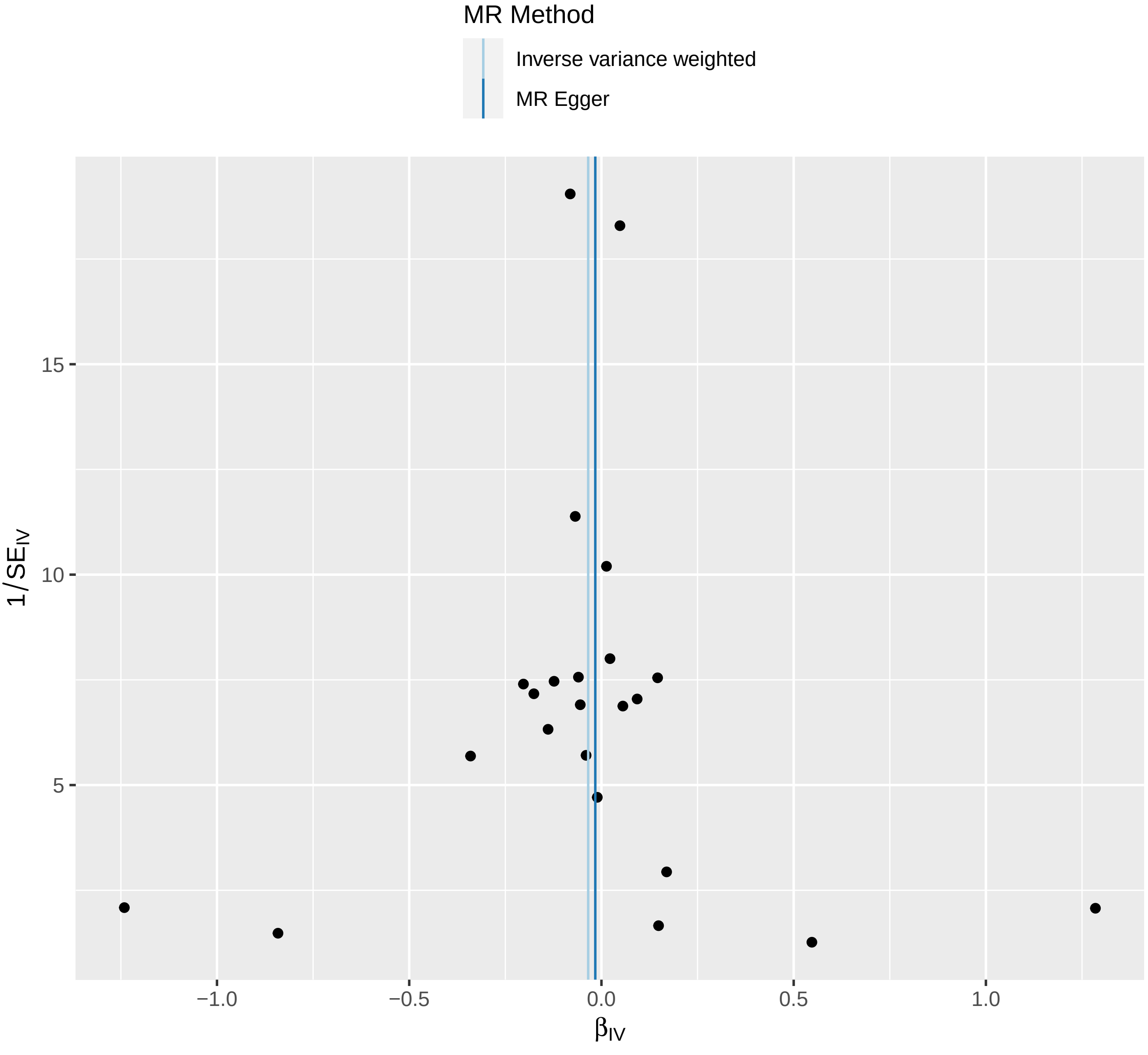
MR Method
Inverse variance weighted
MR Egger
15
10
5
1/SE$_{IV}$
−1.0
−0.5
0.0
0.5
1.0
β$_{IV}$

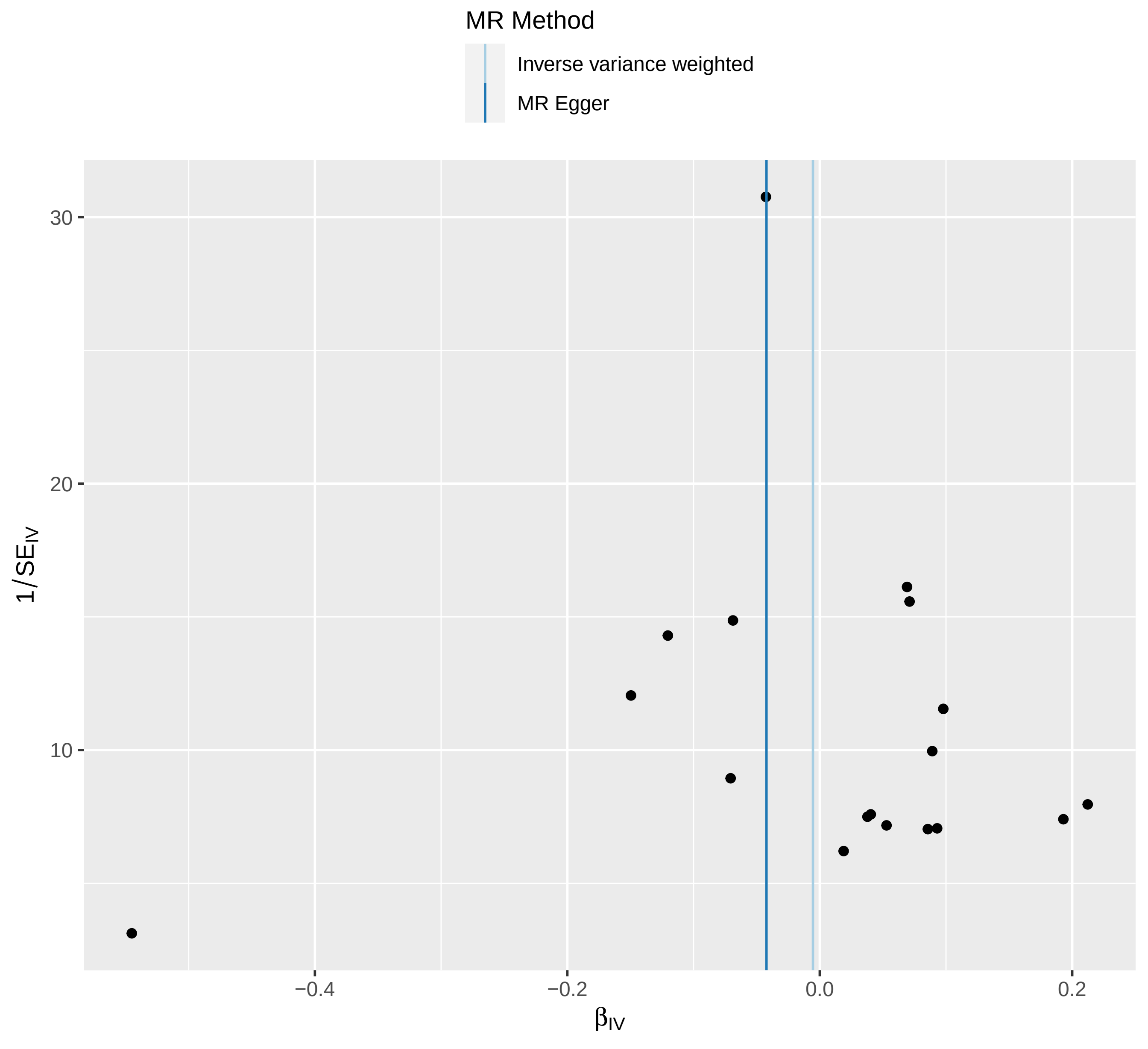

MR Method
Inverse variance weighted
MR Egger
30
20
10
1/SE$_{IV}$
−0.4
−0.2
0.0
0.2
β$_{IV}$

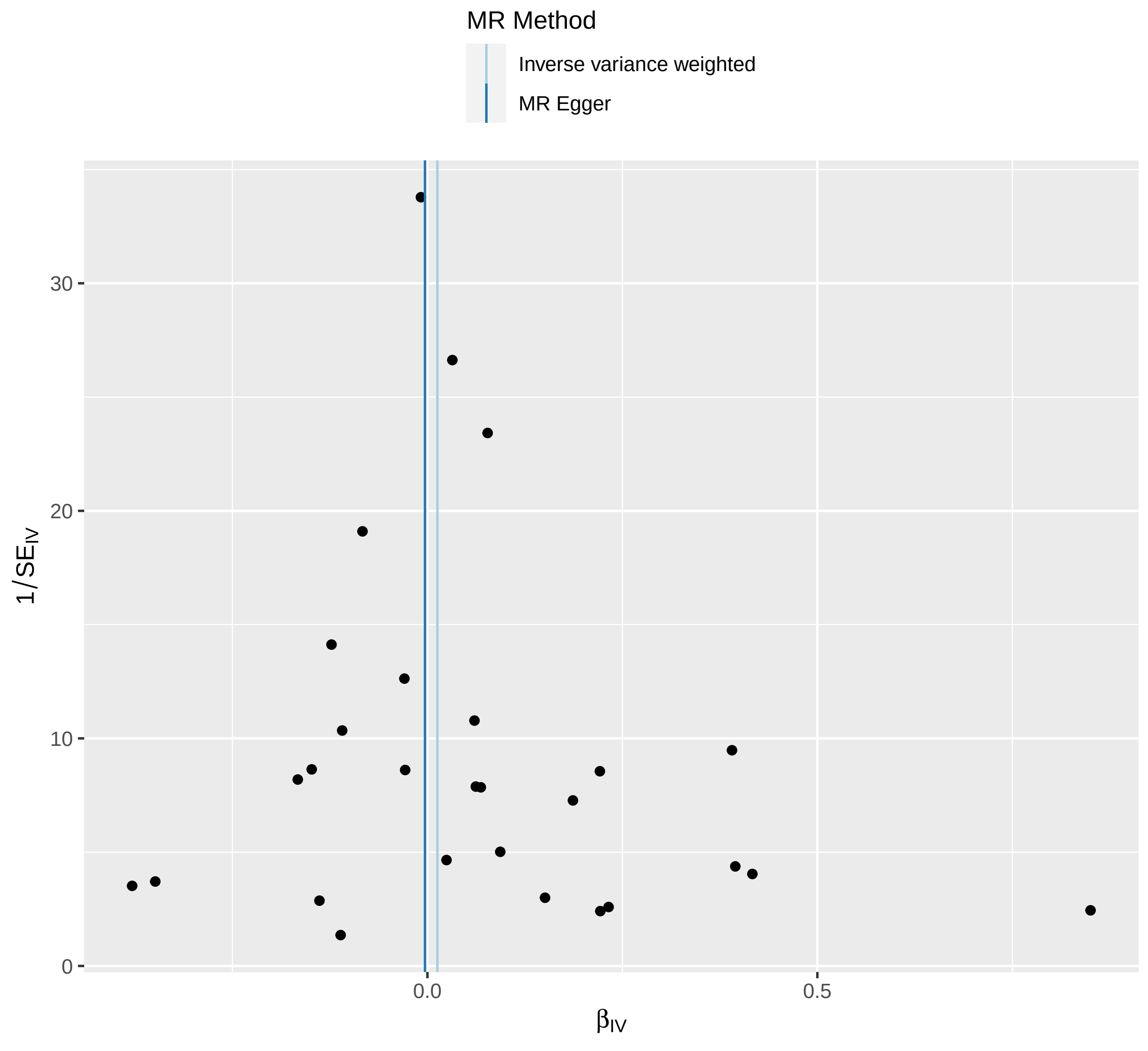
MR Method
Inverse variance weighted
MR Egger
30
20
10
0
0.0
0.5
1/SE$_{IV}$
β$_{IV}$

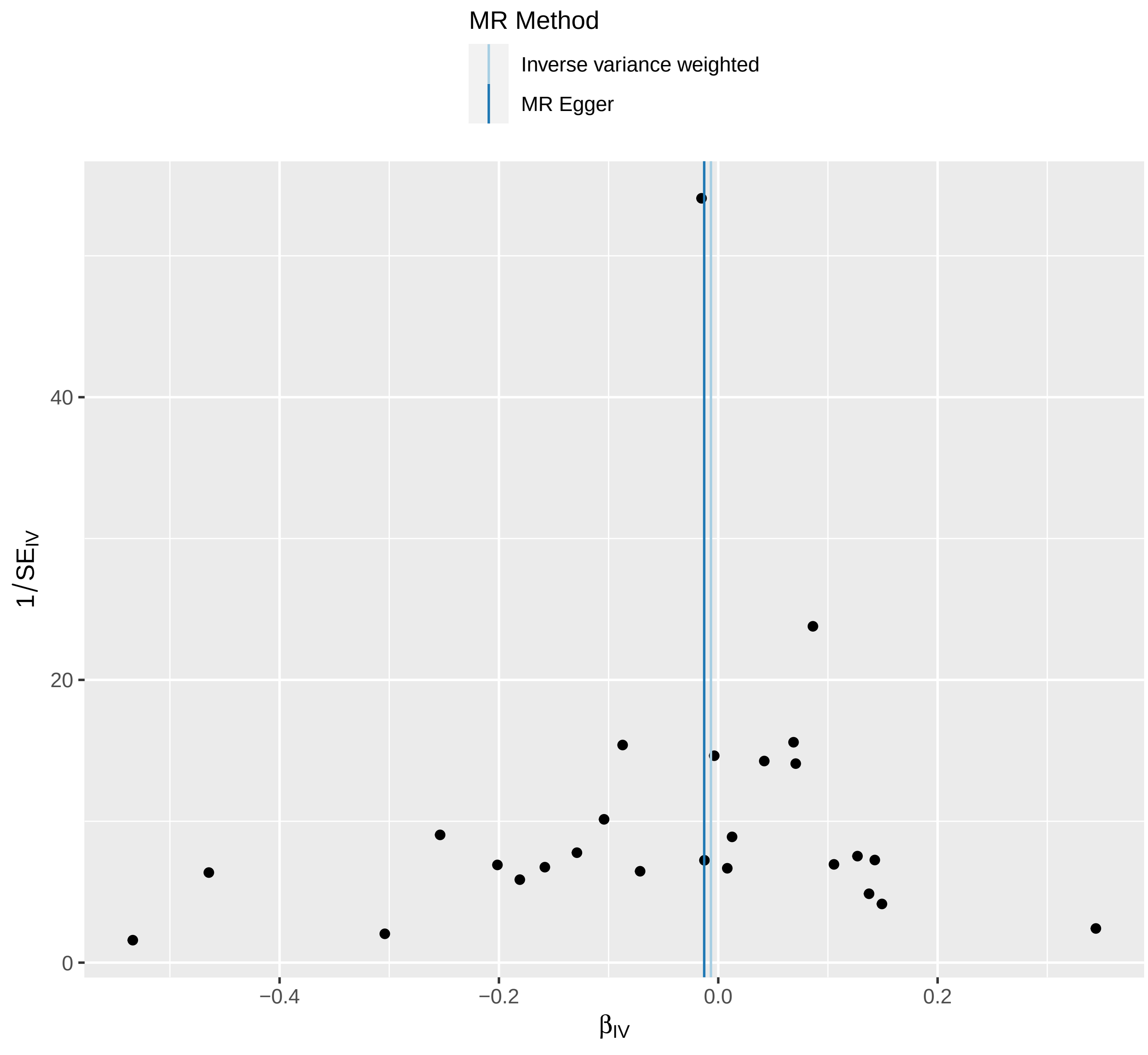

MR Method
Inverse variance weighted
MR Egger
40
20
0
1/SE$_{IV}$
−0.4
−0.2
0.0
0.2
β$_{IV}$

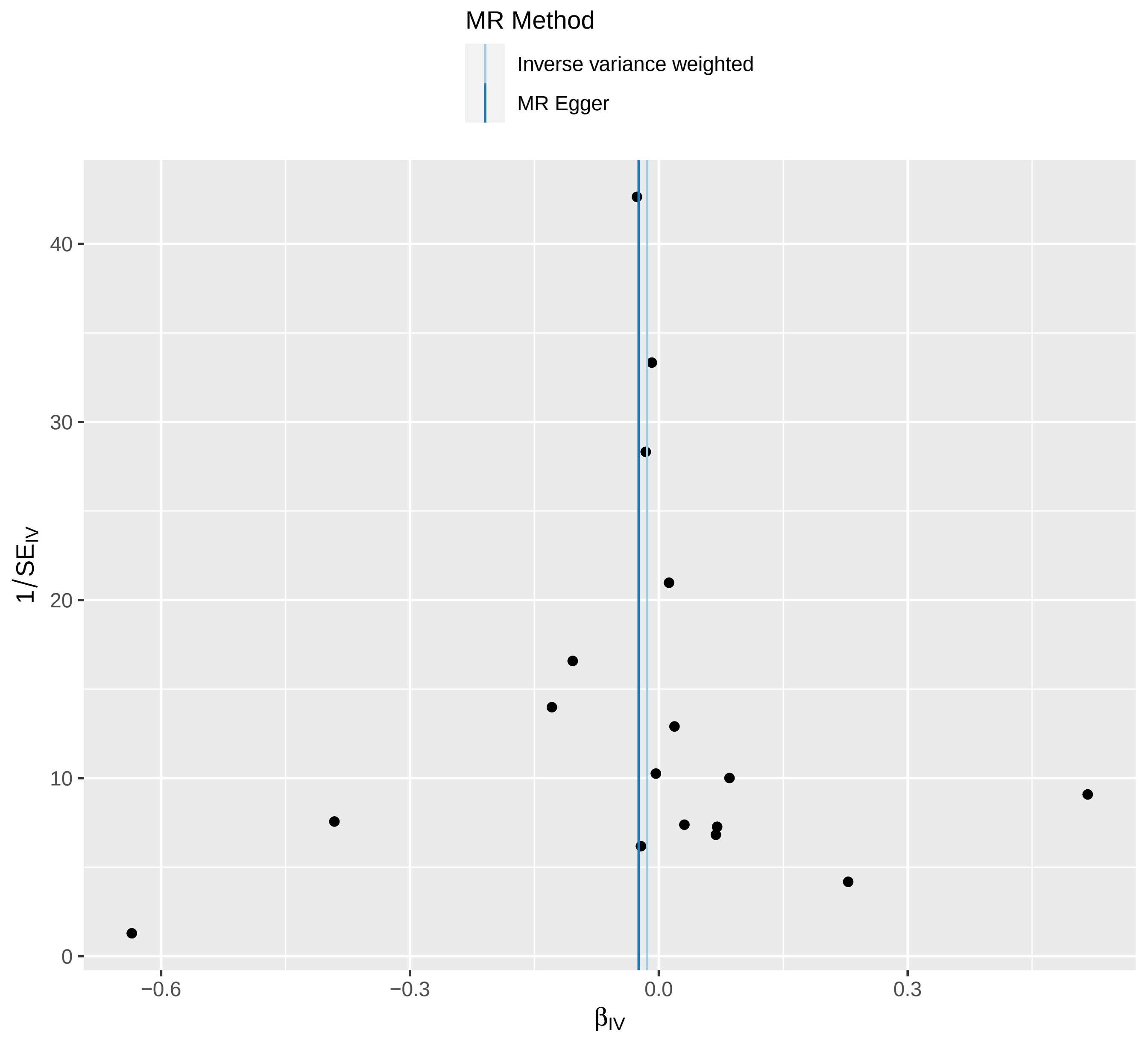

MR Method
Inverse variance weighted
MR Egger
40
30
20
10
0
1/SE_IV
−0.6
−0.3
0.0
0.3
β_IV

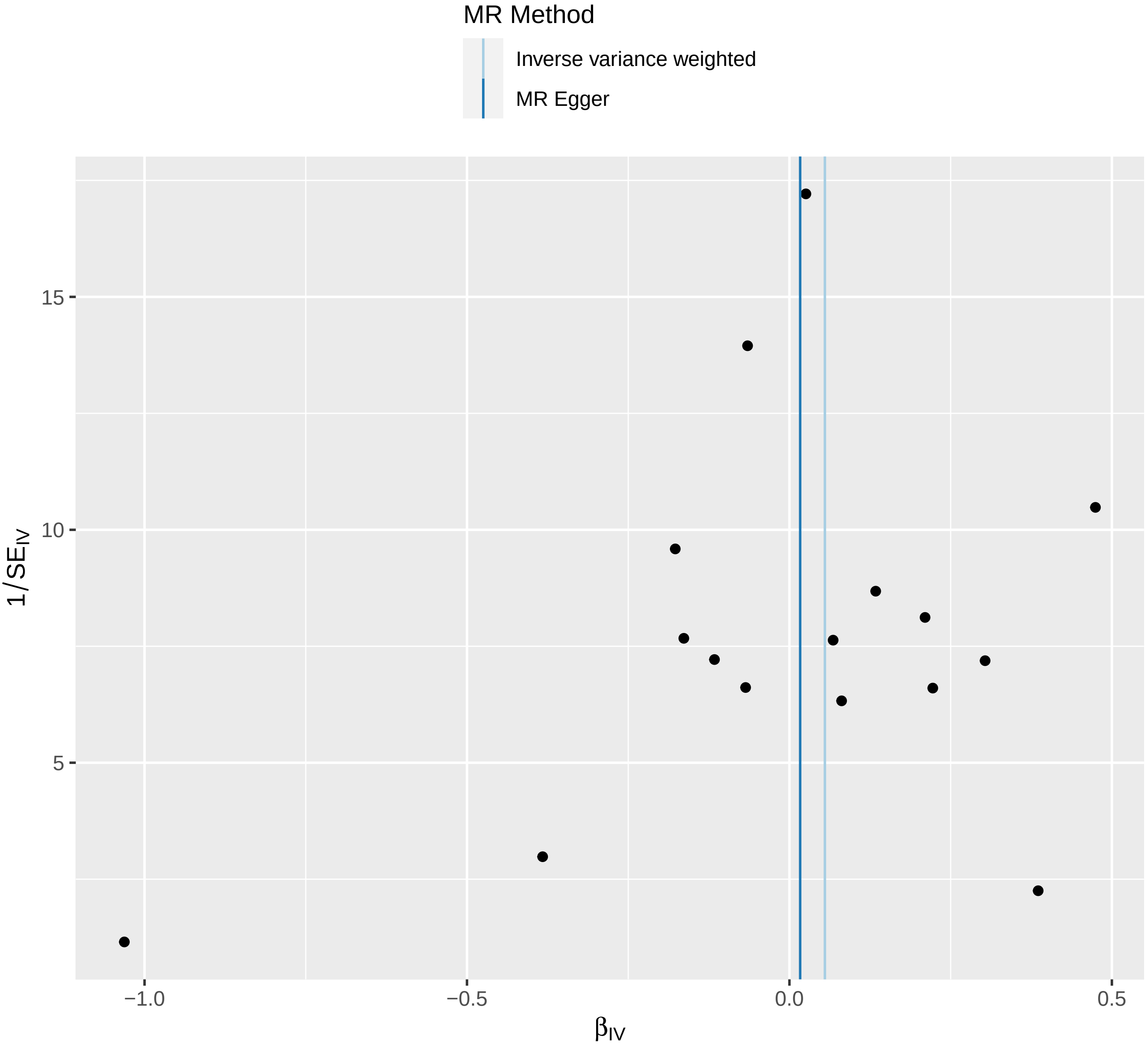
MR Method
Inverse variance weighted
MR Egger
15
10
5
$1/SE_{IV}$
−1.0
−0.5
0.0
0.5
β_{IV}

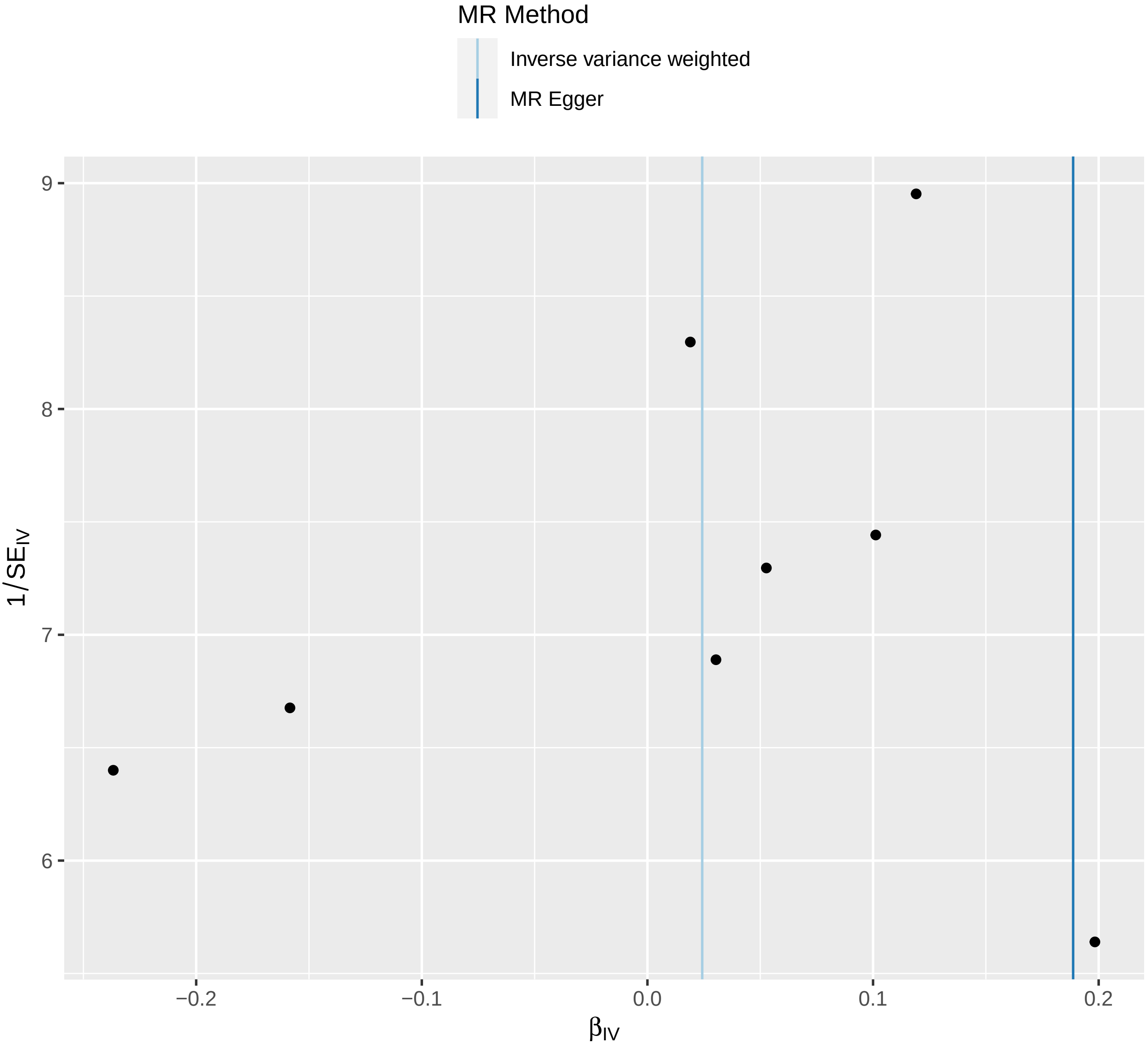
MR Method
Inverse variance weighted
MR Egger
9
8
7
6
1/SE_IV
−0.2
−0.1
0.0
0.1
0.2
β_IV

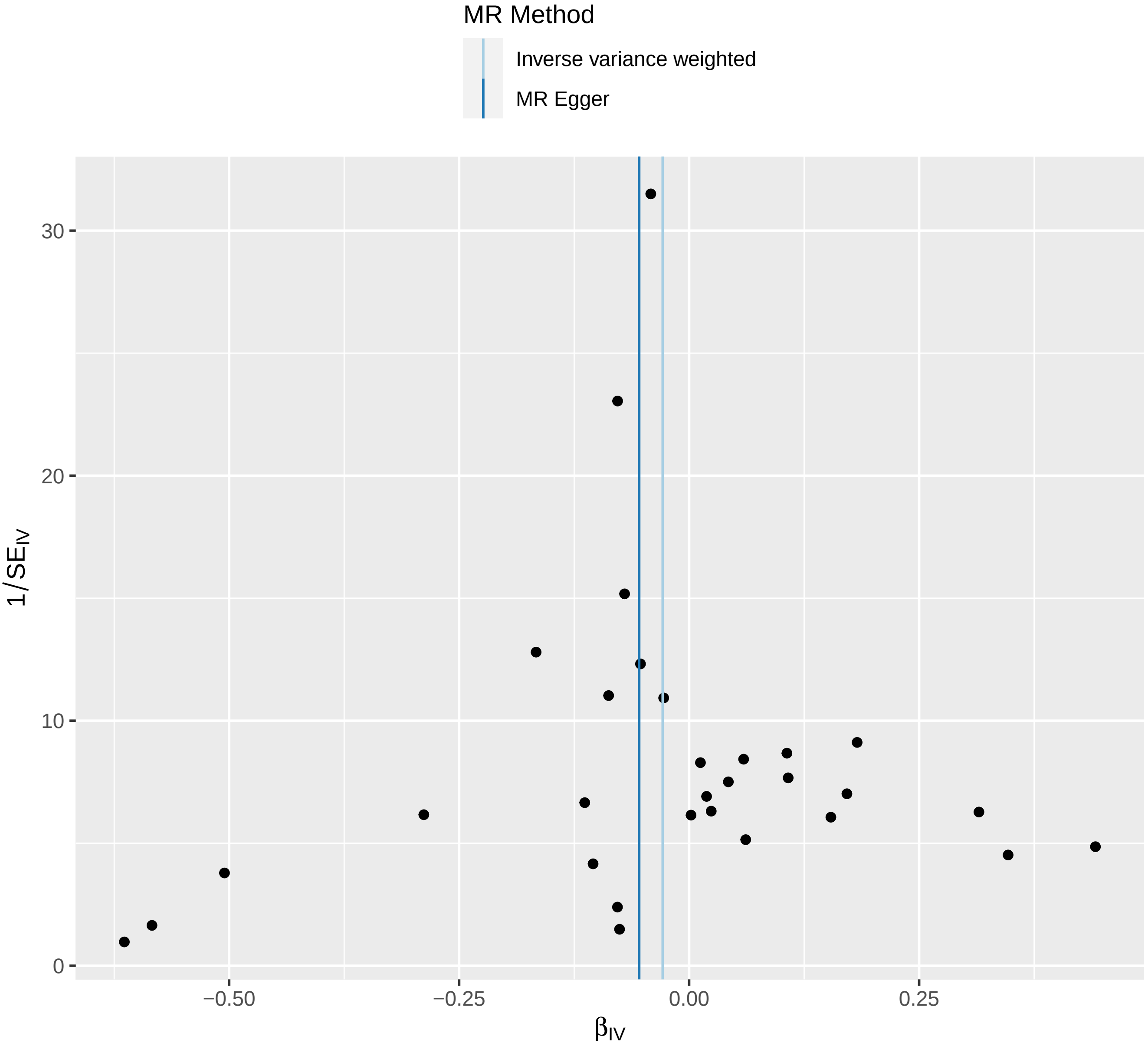
MR Method
Inverse variance weighted
MR Egger
30
20
10
0
1/SE$_{IV}$
−0.50
−0.25
0.00
0.25
β$_{IV}$

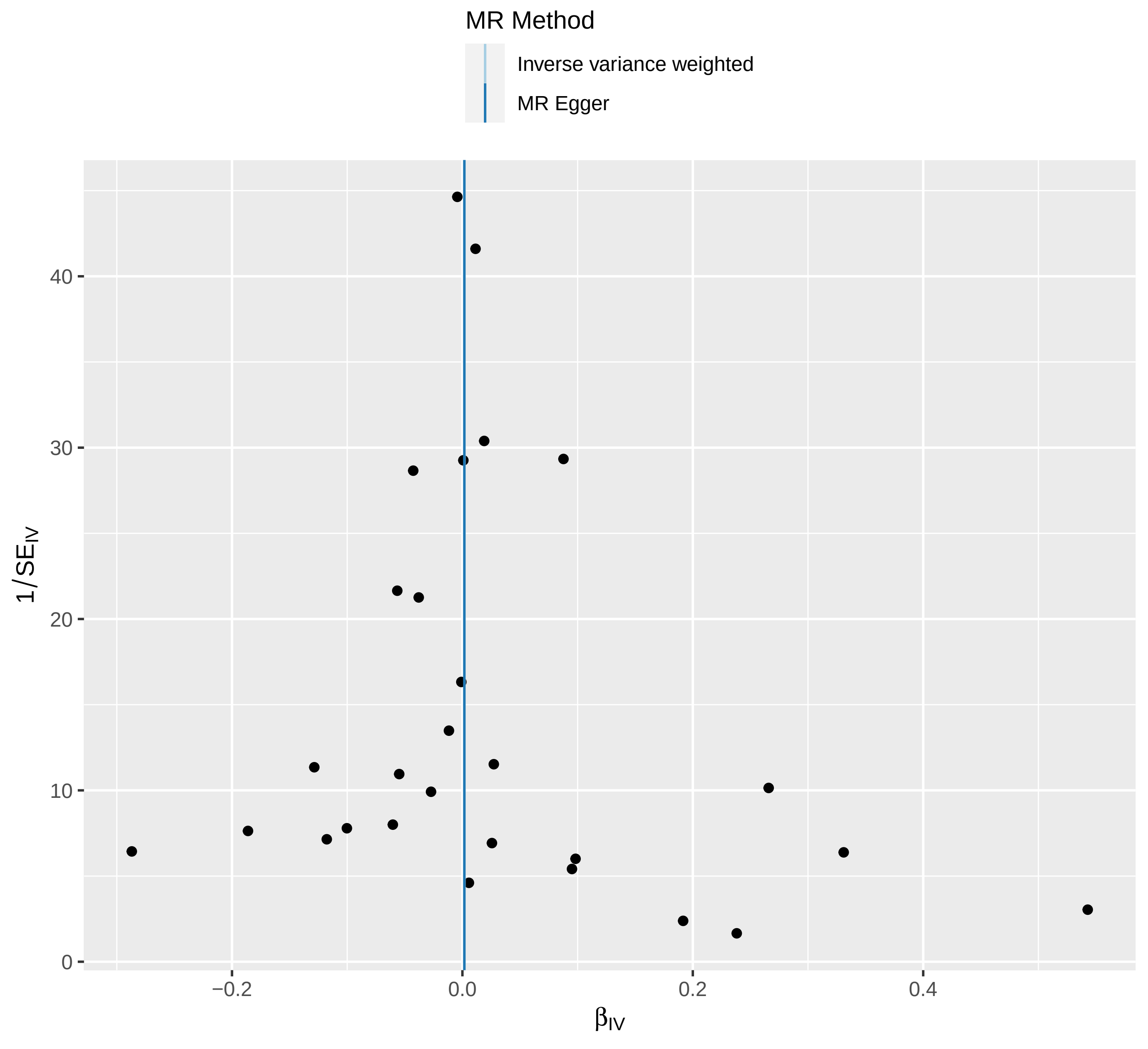
MR Method
Inverse variance weighted
MR Egger
40
30
20
10
0
−0.2
0.0
0.2
0.4
$1/SE_{IV}$
β_{IV}

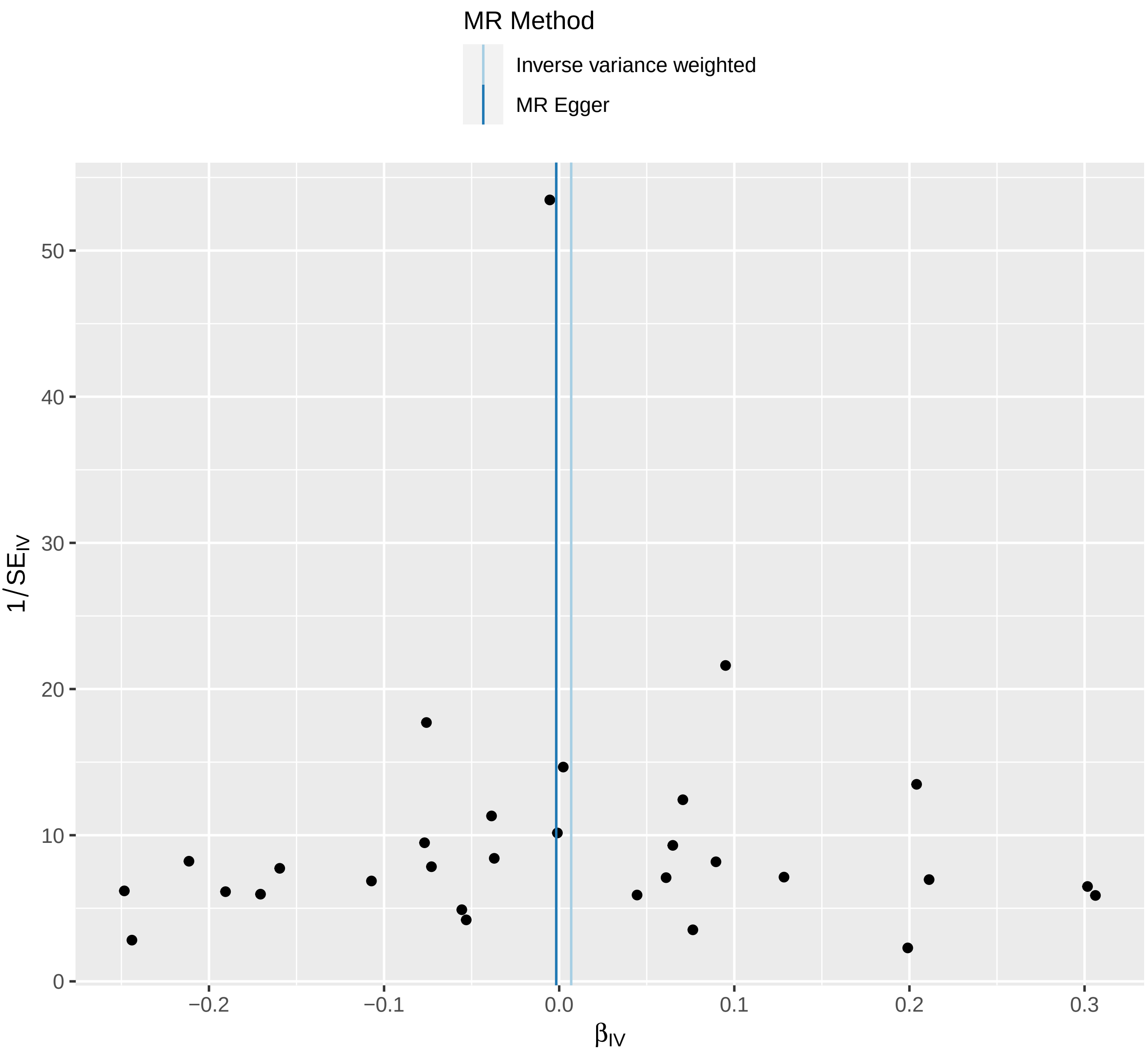
MR Method
Inverse variance weighted
MR Egger
1/SE_IV
β_IV
0
10
20
30
40
50
−0.2
−0.1
0.0
0.1
0.2
0.3

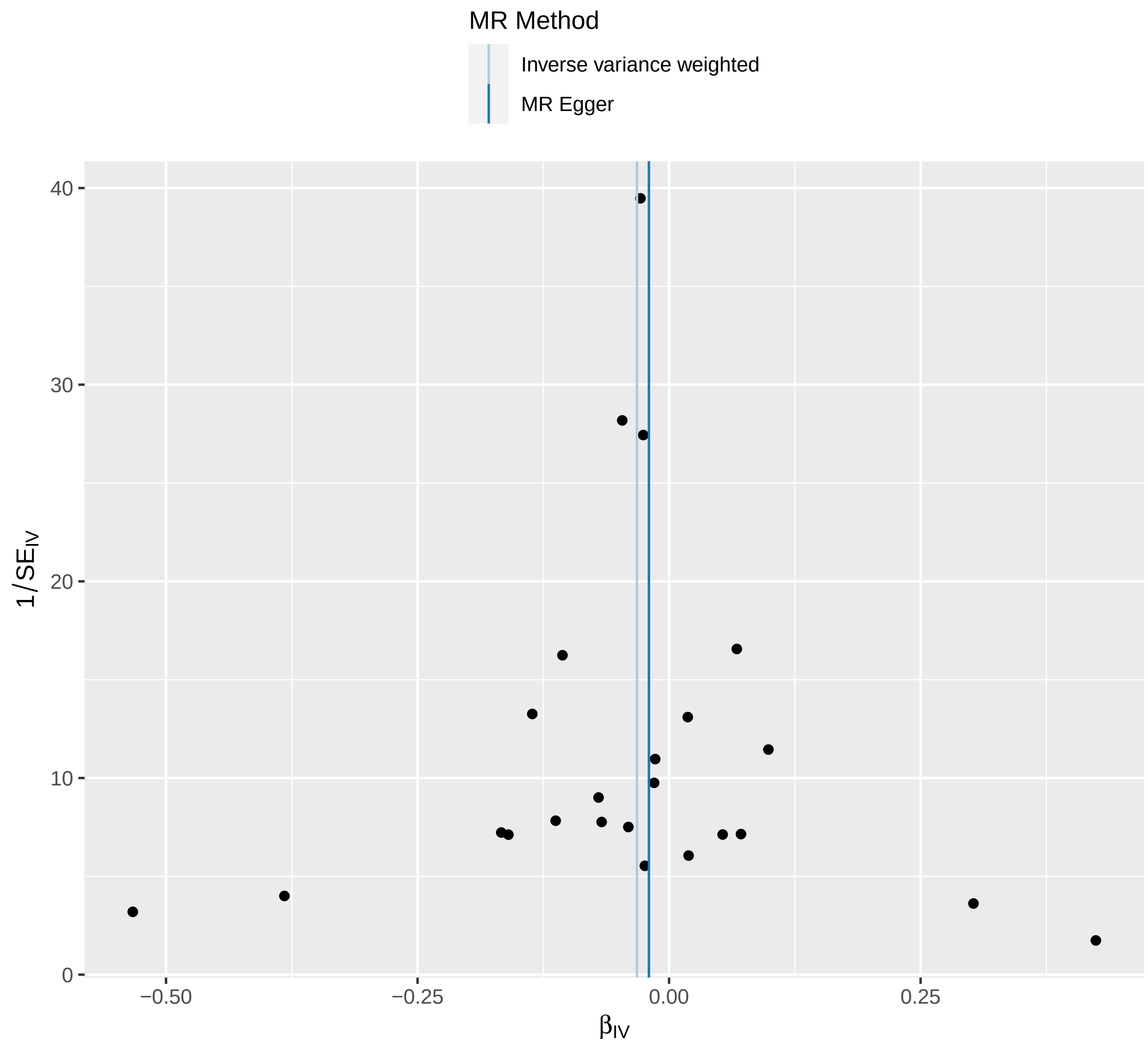

MR Method
Inverse variance weighted
MR Egger
40
30
20
10
0
$1/SE_{IV}$
−0.50
−0.25
0.00
0.25
β_{IV}

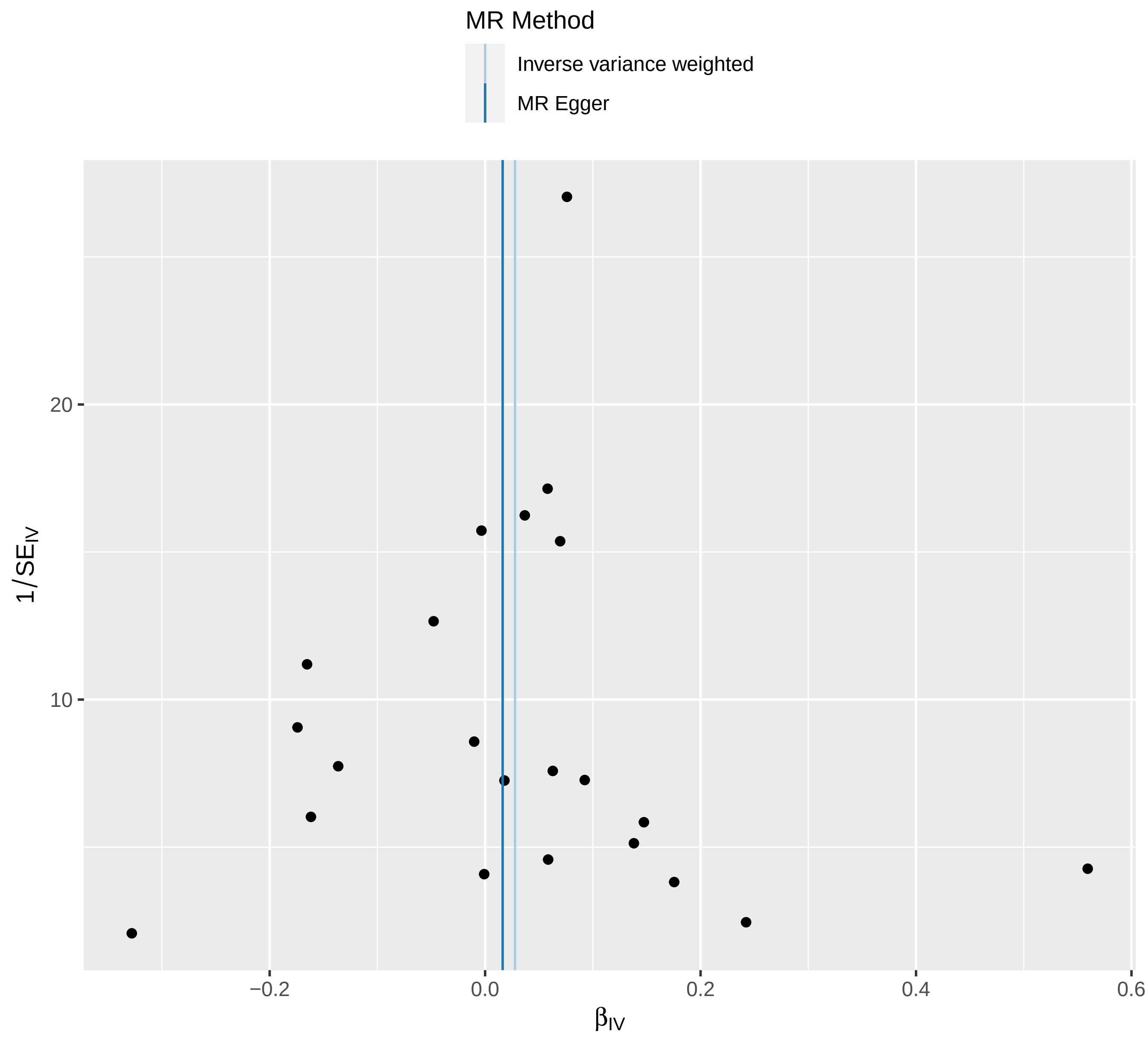

MR Method
Inverse variance weighted
MR Egger
20
10
$1/SE_{IV}$
−0.2
0.0
0.2
0.4
0.6
β_{IV}

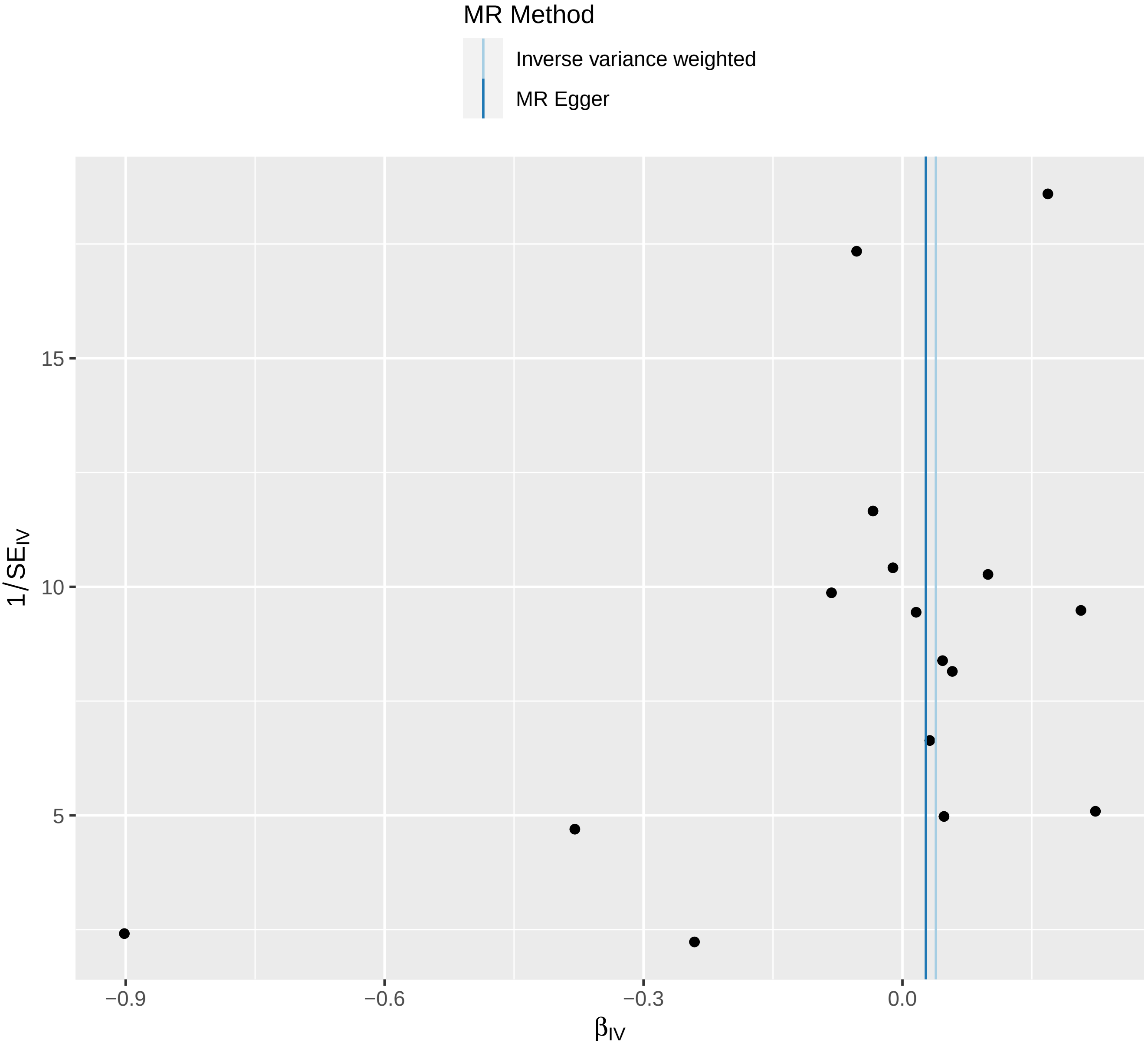
MR Method
Inverse variance weighted
MR Egger
1/SE$_{IV}$
15
10
5
−0.9
−0.6
−0.3
0.0
β$_{IV}$

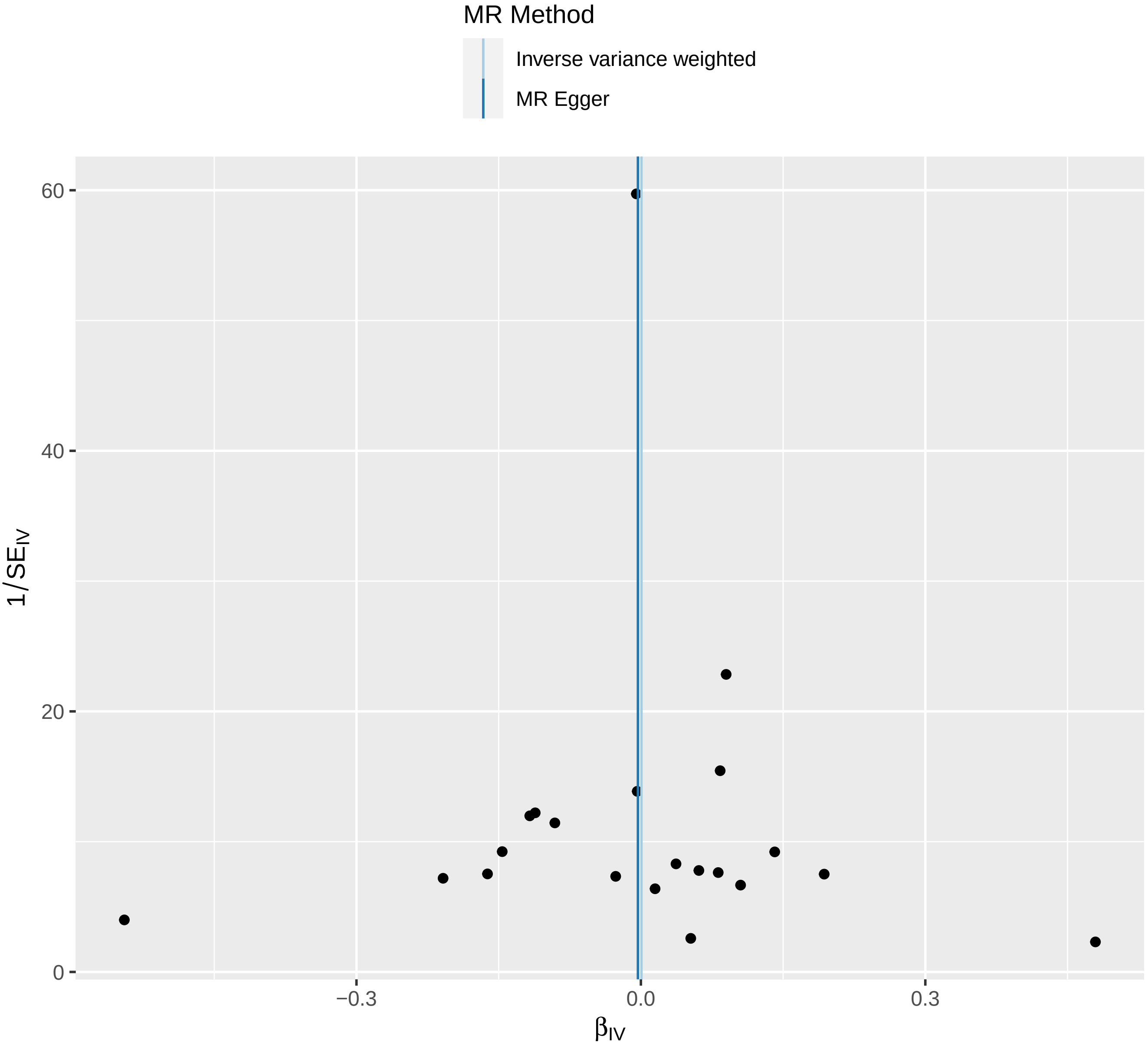

MR Method
Inverse variance weighted
MR Egger
60
40
20
0
1/SE$_{IV}$
−0.3
0.0
0.3
β$_{IV}$

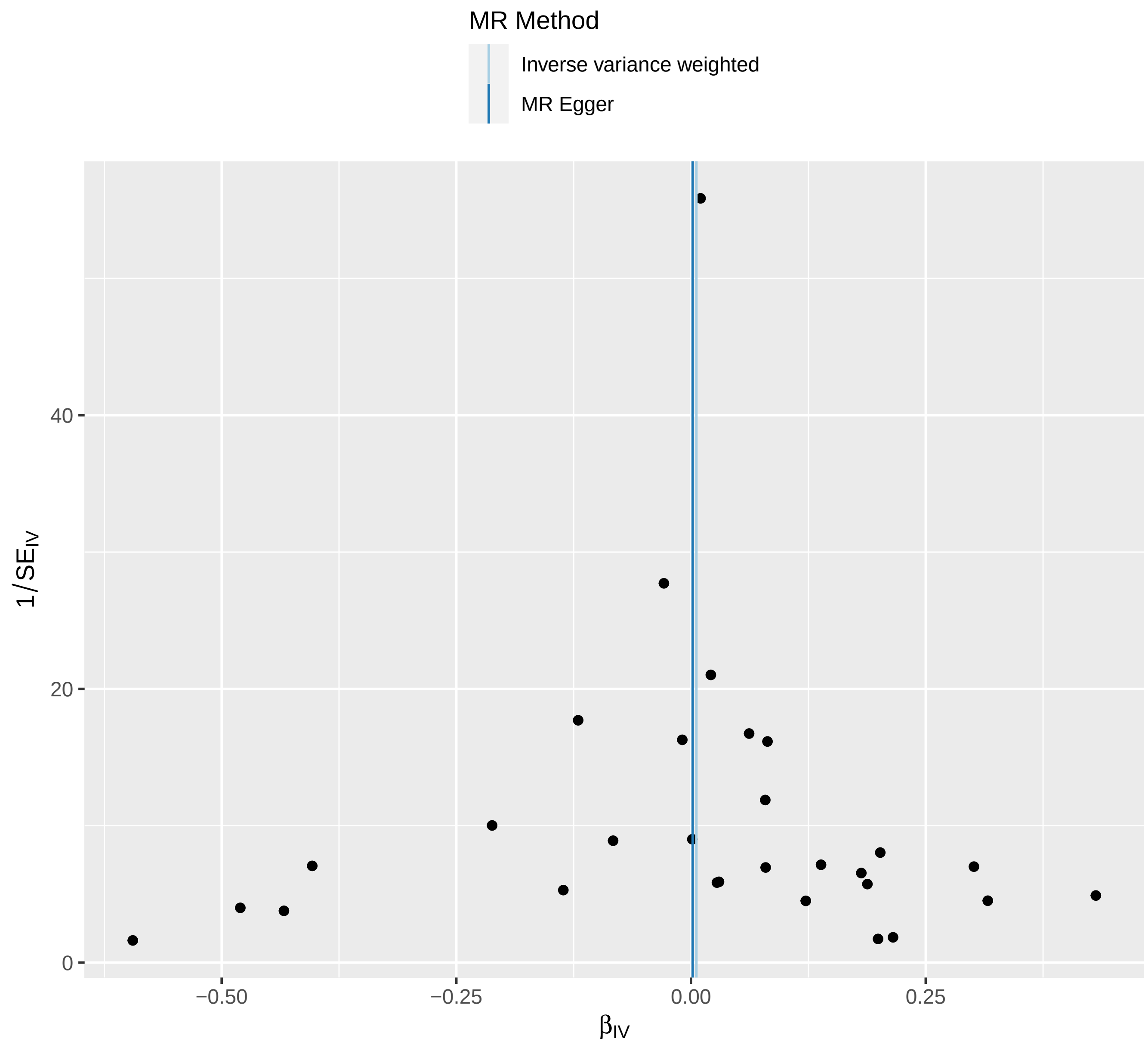
MR Method
Inverse variance weighted
MR Egger
40
20
0
1/SE$_{IV}$
−0.50
−0.25
0.00
0.25
β$_{IV}$

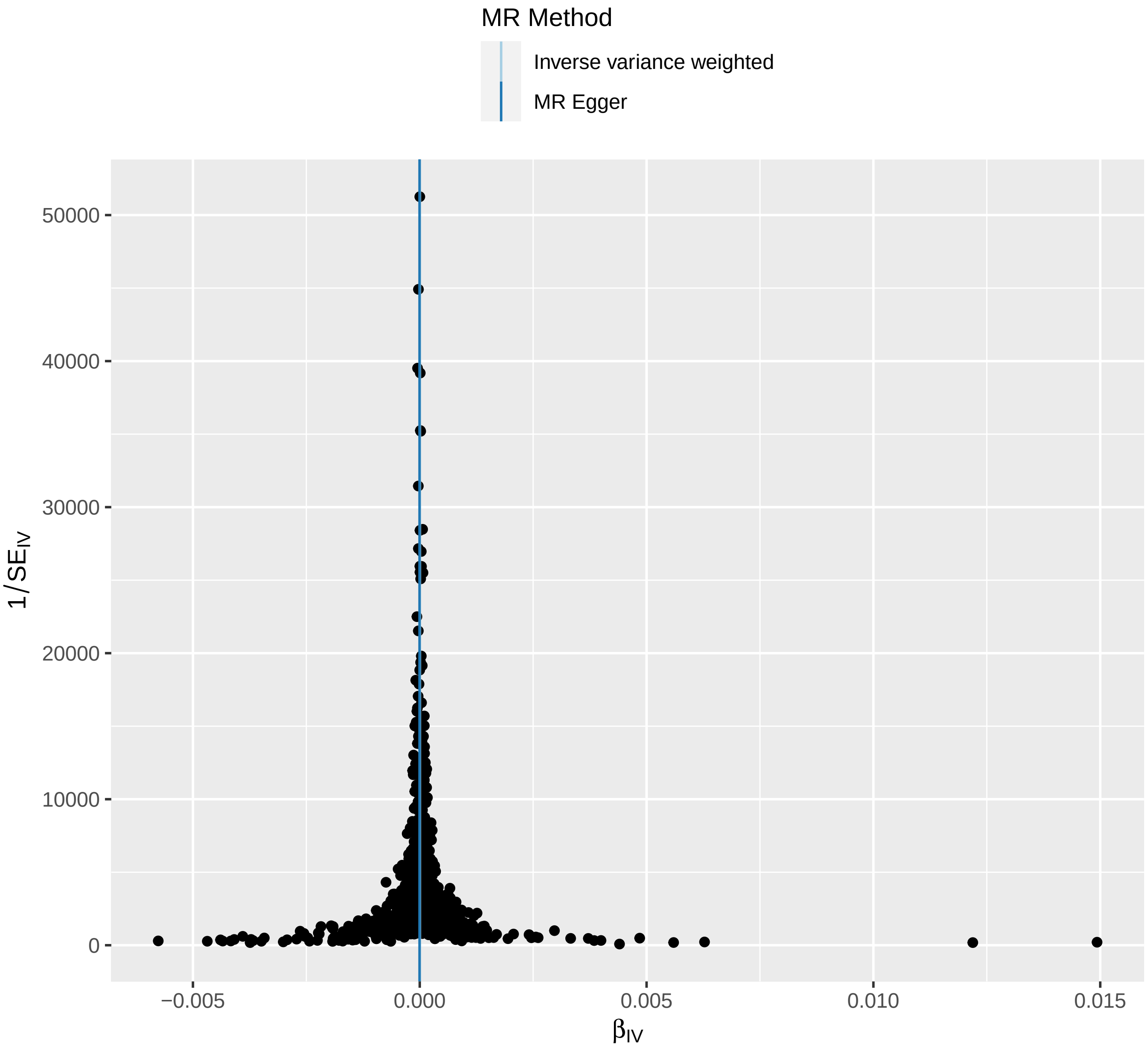

MR Method
Inverse variance weighted
MR Egger
50000
40000
30000
20000
10000
0
1/SE_IV
−0.005
0.000
0.005
0.010
0.015
β_IV

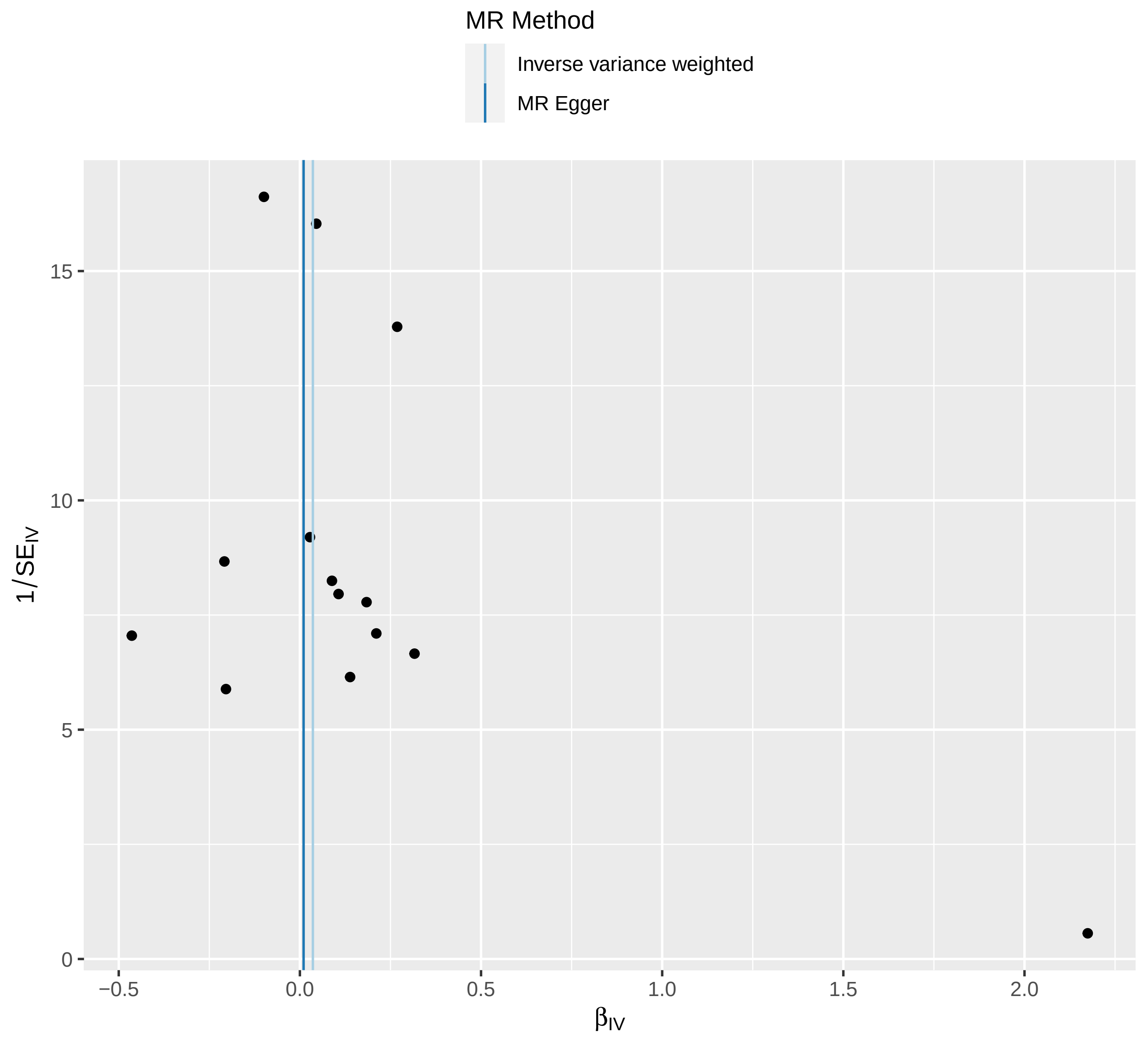
MR Method
Inverse variance weighted
MR Egger
15
10
5
0
1/SE_{IV}
−0.5
0.0
0.5
1.0
1.5
2.0
$β_{IV}$

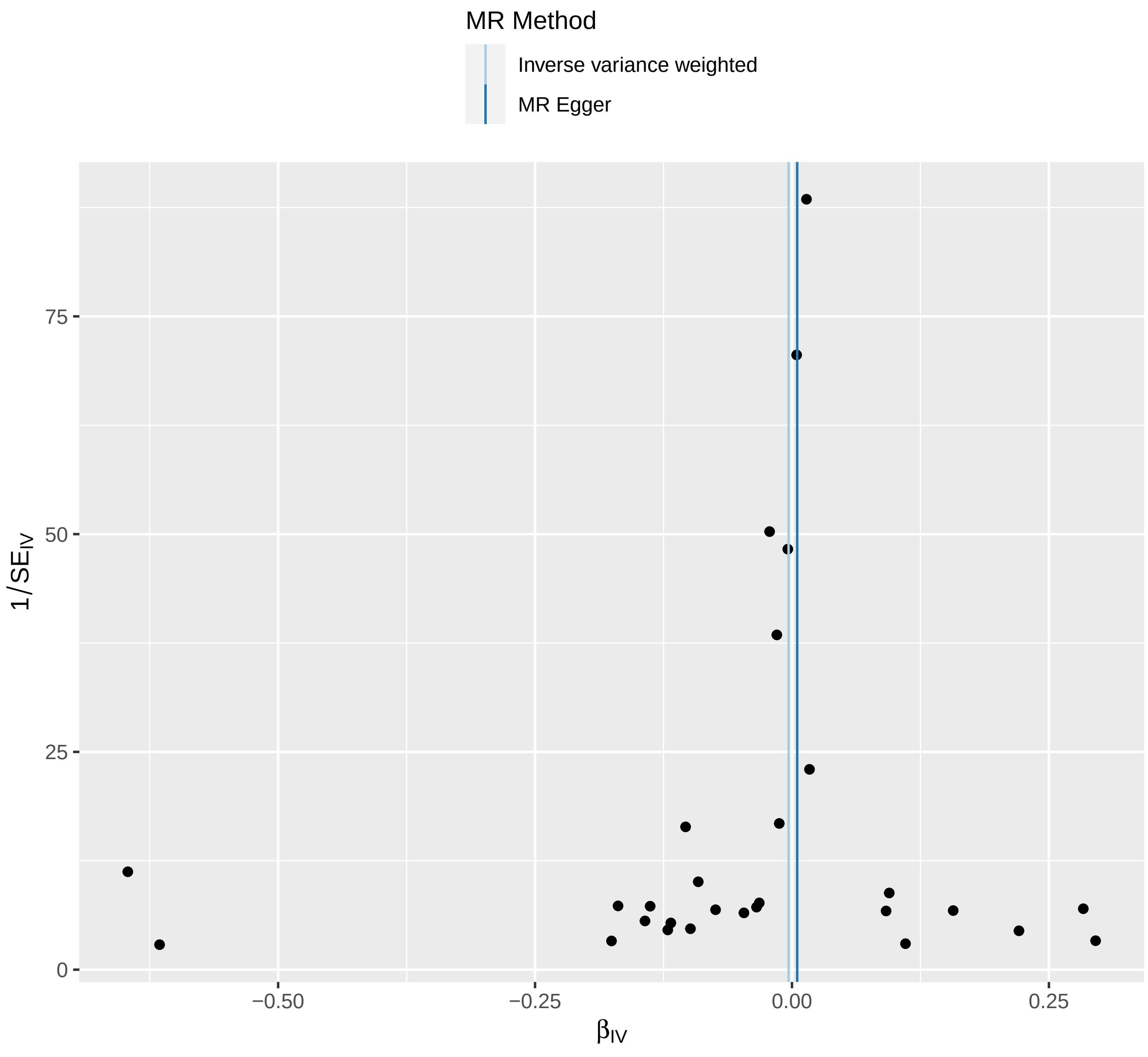

MR Method
Inverse variance weighted
MR Egger
75
50
25
0
1/SE_IV
−0.50
−0.25
0.00
0.25
β_IV

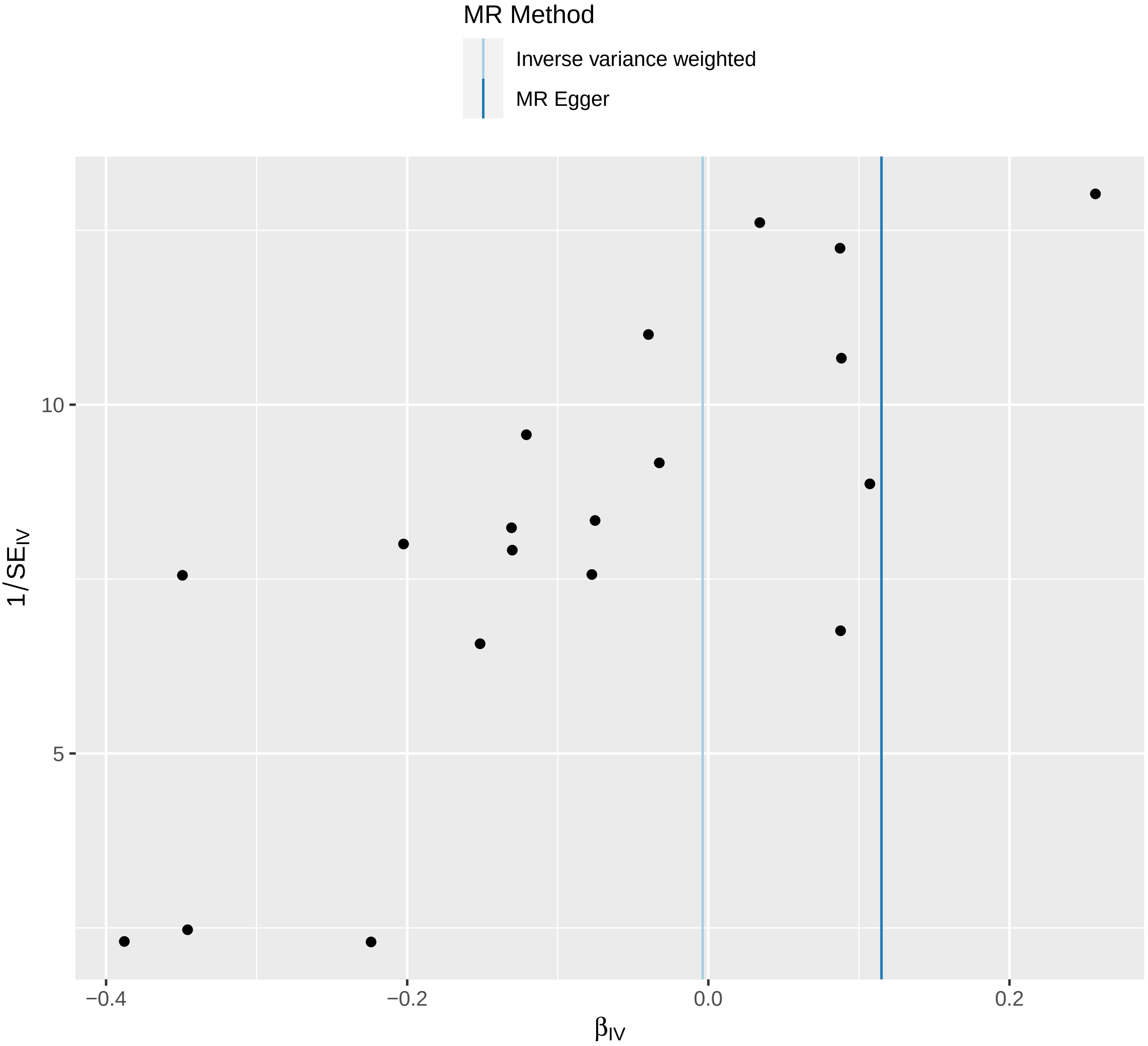

MR Method
Inverse variance weighted
MR Egger
10
5
−0.4
−0.2
0.0
0.2
β_{IV}
$1/SE_{IV}$

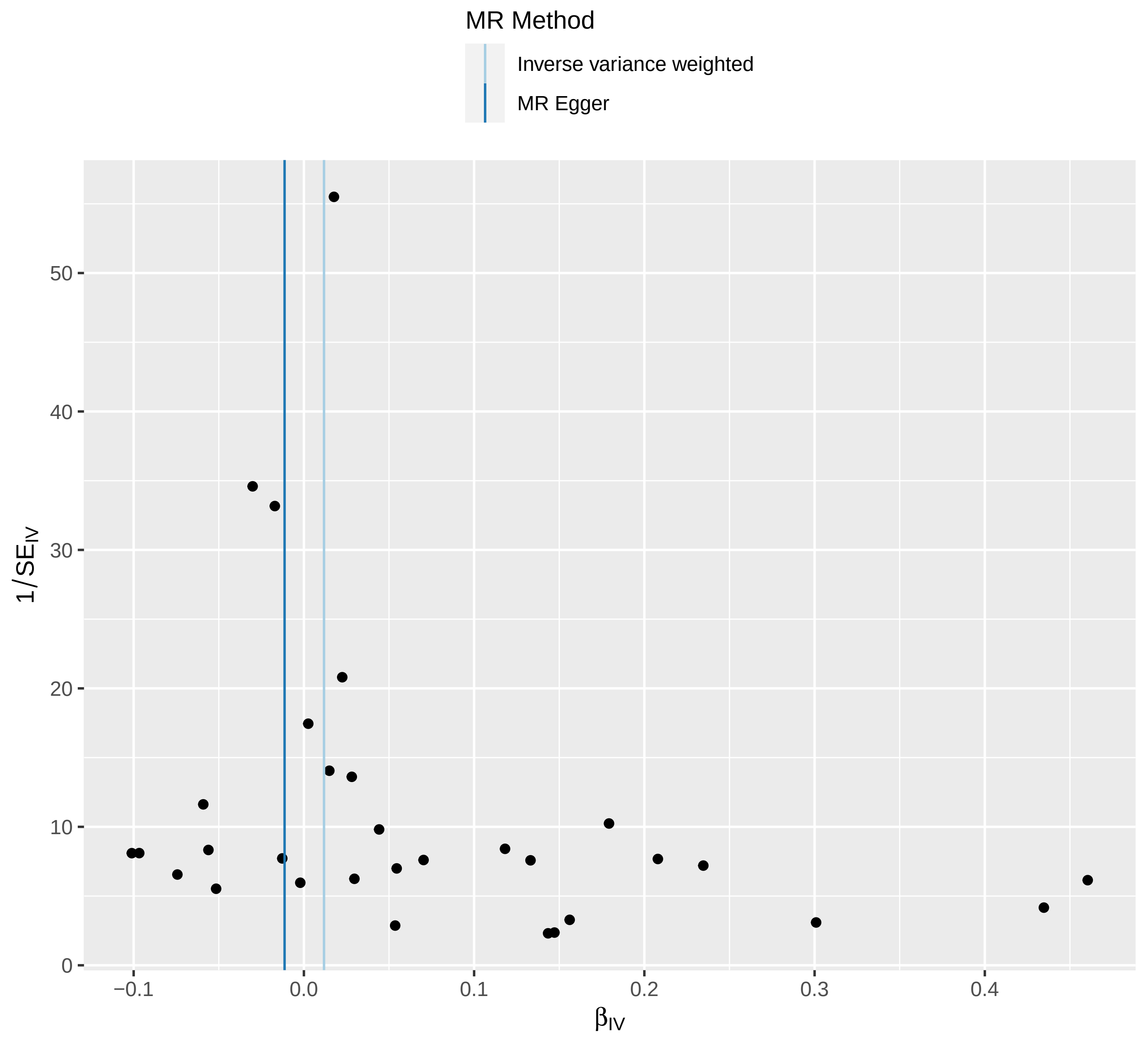

MR Method
Inverse variance weighted
MR Egger
50
40
30
20
10
0
1/SE_IV
−0.1
0.0
0.1
0.2
0.3
0.4
β_IV

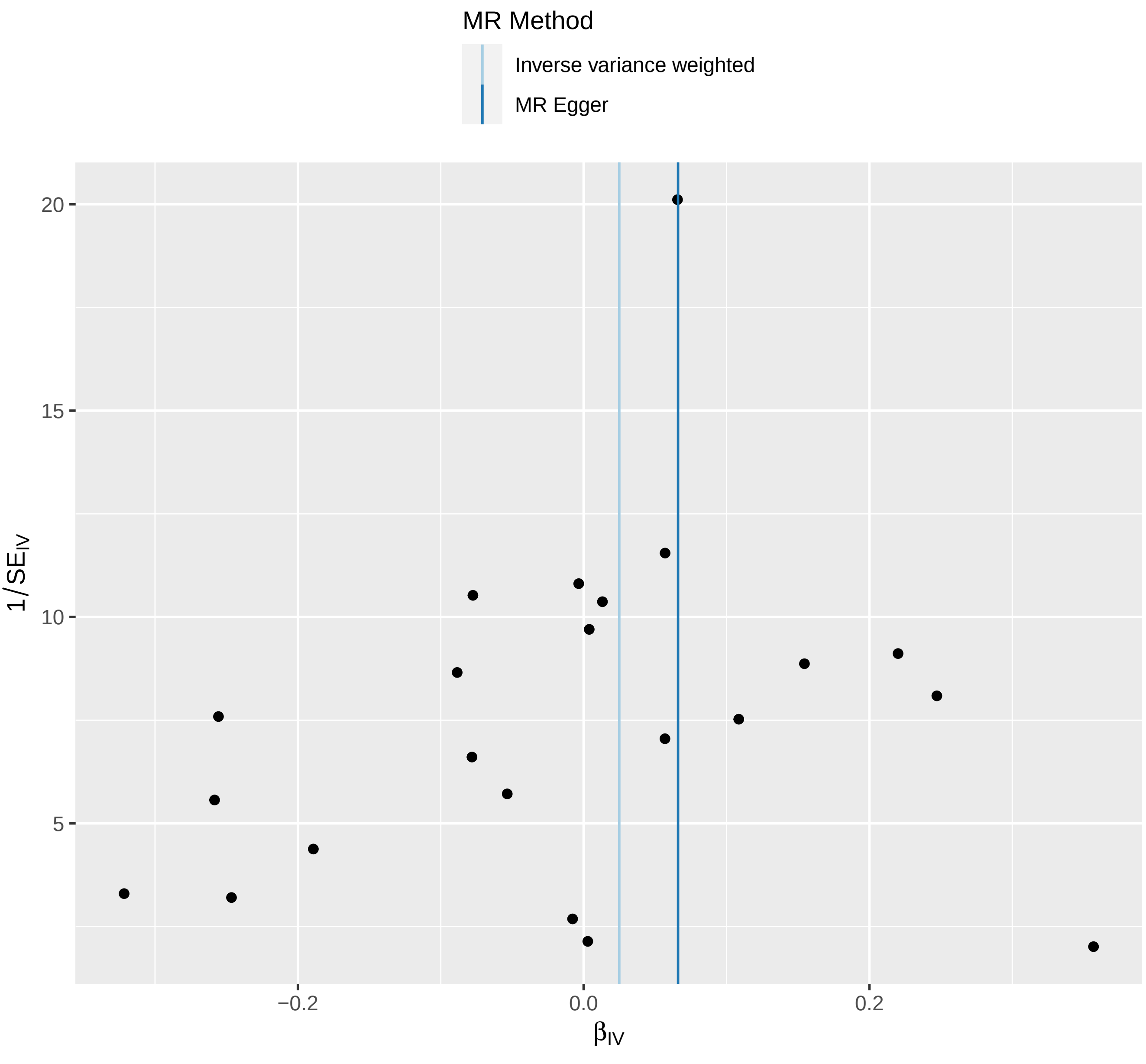

MR Method
Inverse variance weighted
MR Egger
20
15
10
5
1/SE_IV
−0.2
0.0
0.2
β_IV

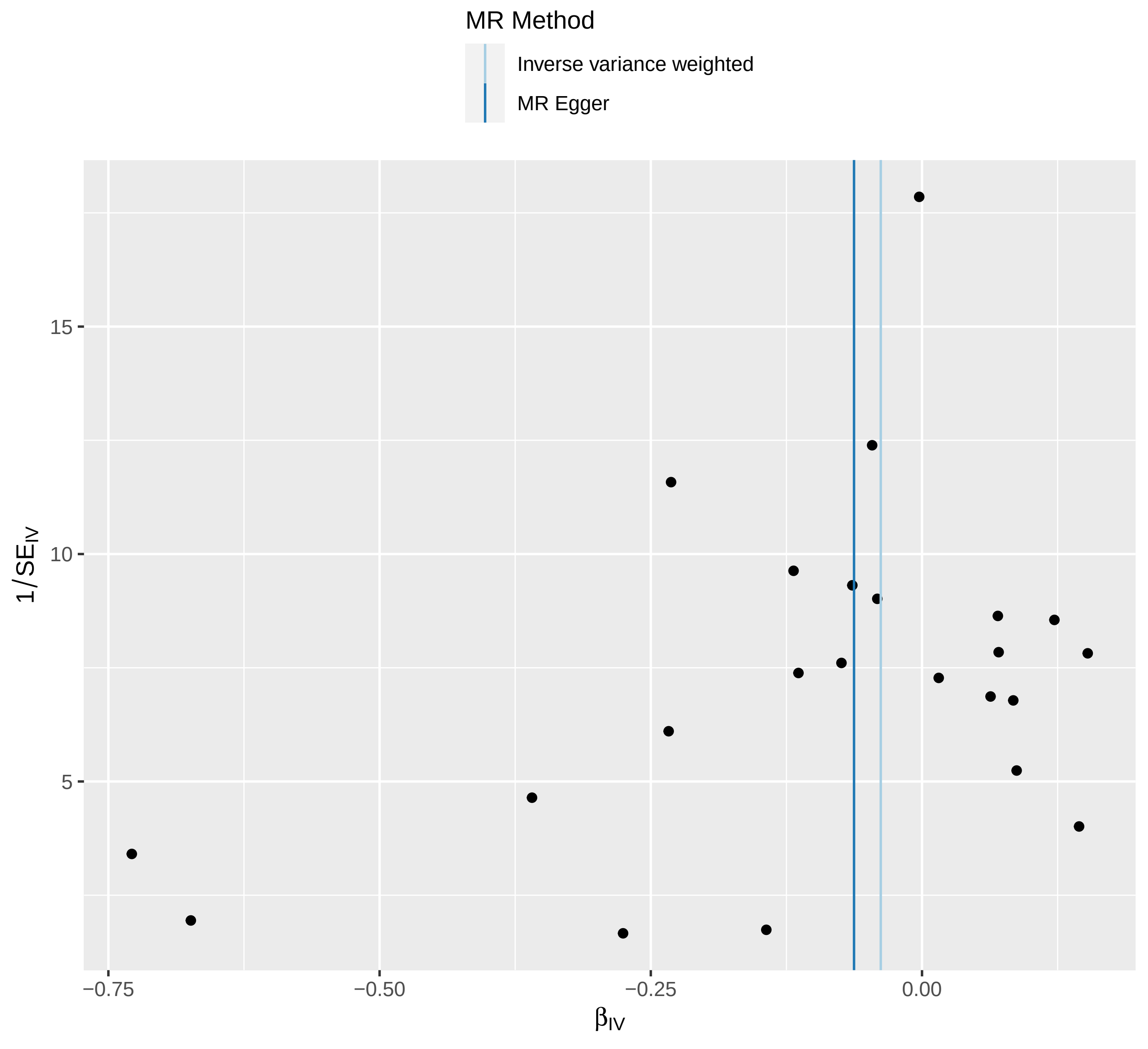
MR Method
Inverse variance weighted
MR Egger
15
10
5
1/SE_IV
−0.75
−0.50
−0.25
0.00
β_IV

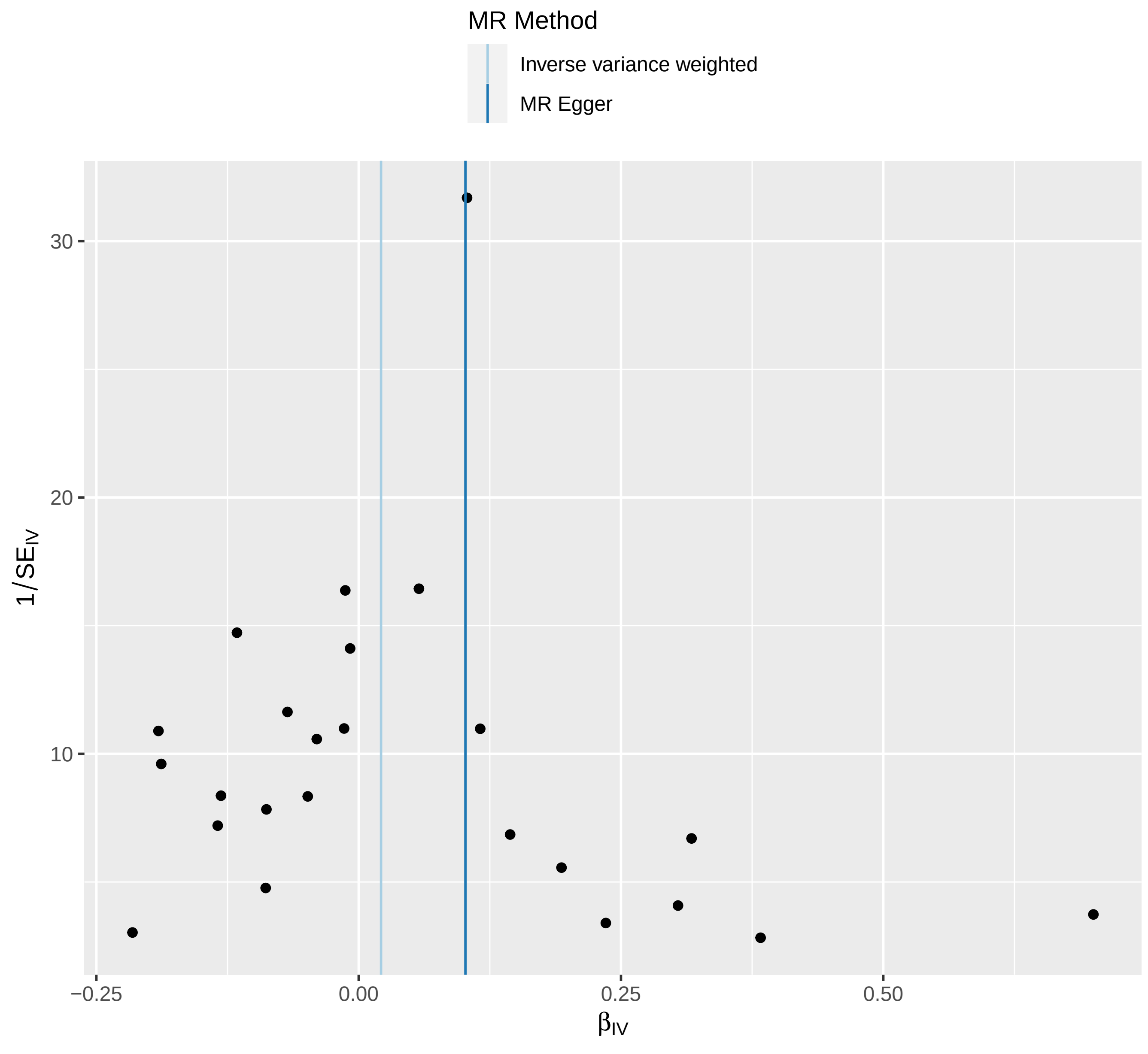

MR Method
Inverse variance weighted
MR Egger
30
20
10
1/SE$_{IV}$
−0.25
0.00
0.25
0.50
β$_{IV}$
