# Supplementary material for: Treg cells as a protective factor for Hashimoto`s thyroiditis: a Mendelian randomization study
Source: Front Endocrinol (Lausanne). 2024 Mar 8;15:1347695. doi: 10.3389/fendo.2024.1347695 (PMC10957564; doi:10.3389/fendo.2024.1347695)
Supplement: Supplementary file 4 [file Image_4.pdf]

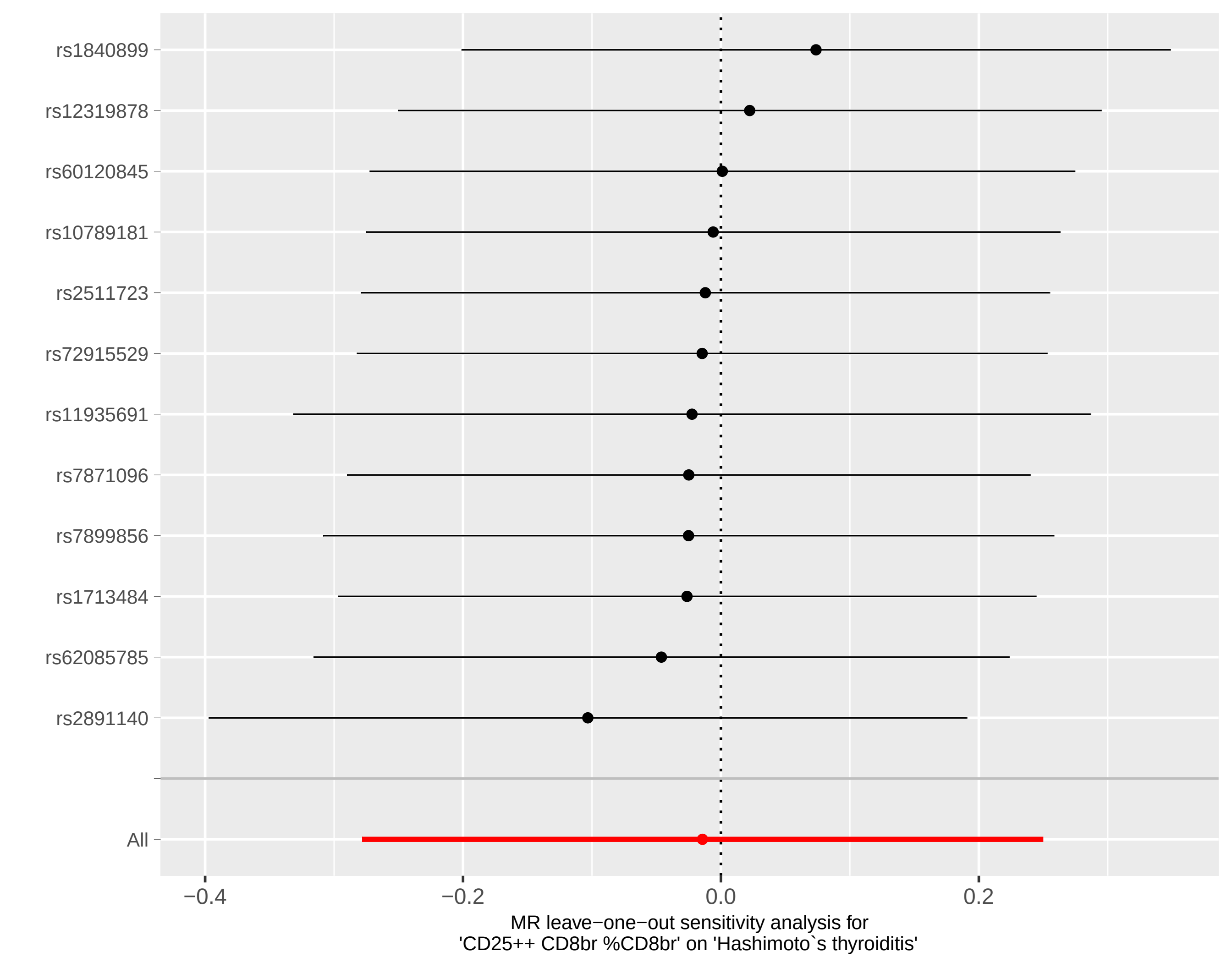
rs1840899
rs12319878
rs60120845
rs10789181
rs2511723
rs72915529
rs11935691
rs7871096
rs7899856
rs1713484
rs62085785
rs2891140
All
−0.4
−0.2
0.0
0.2
MR leave−one−out sensitivity analysis for
'CD25++ CD8br %CD8br' on 'Hashimoto`s thyroiditis'

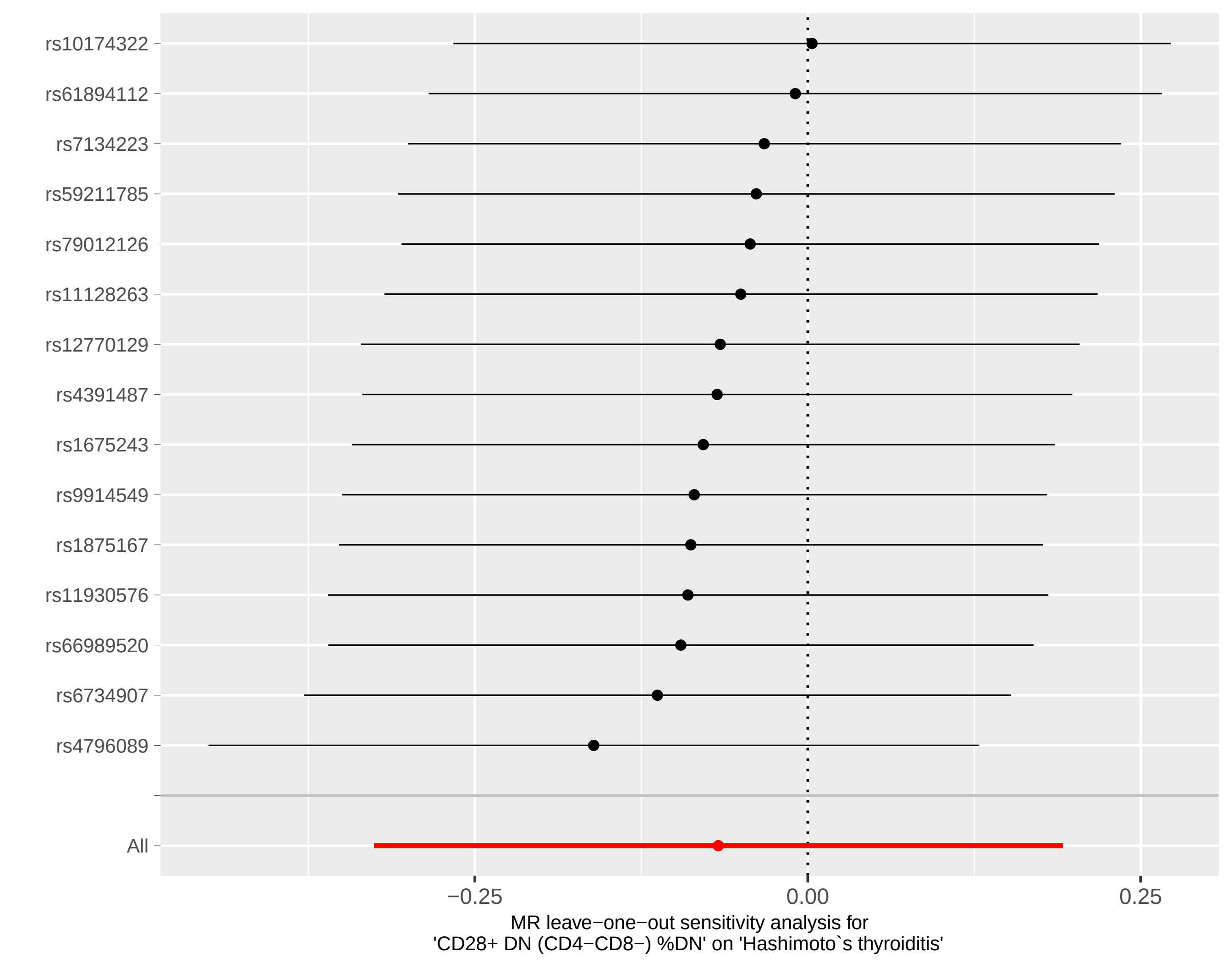

rs10174322
rs61894112
rs7134223
rs59211785
rs79012126
rs11128263
rs12770129
rs4391487
rs1675243
rs9914549
rs1875167
rs11930576
rs66989520
rs6734907
rs4796089
All
−0.25
0.00
0.25
MR leave−one−out sensitivity analysis for
'CD28+ DN (CD4−CD8−) %DN' on 'Hashimoto`s thyroiditis'

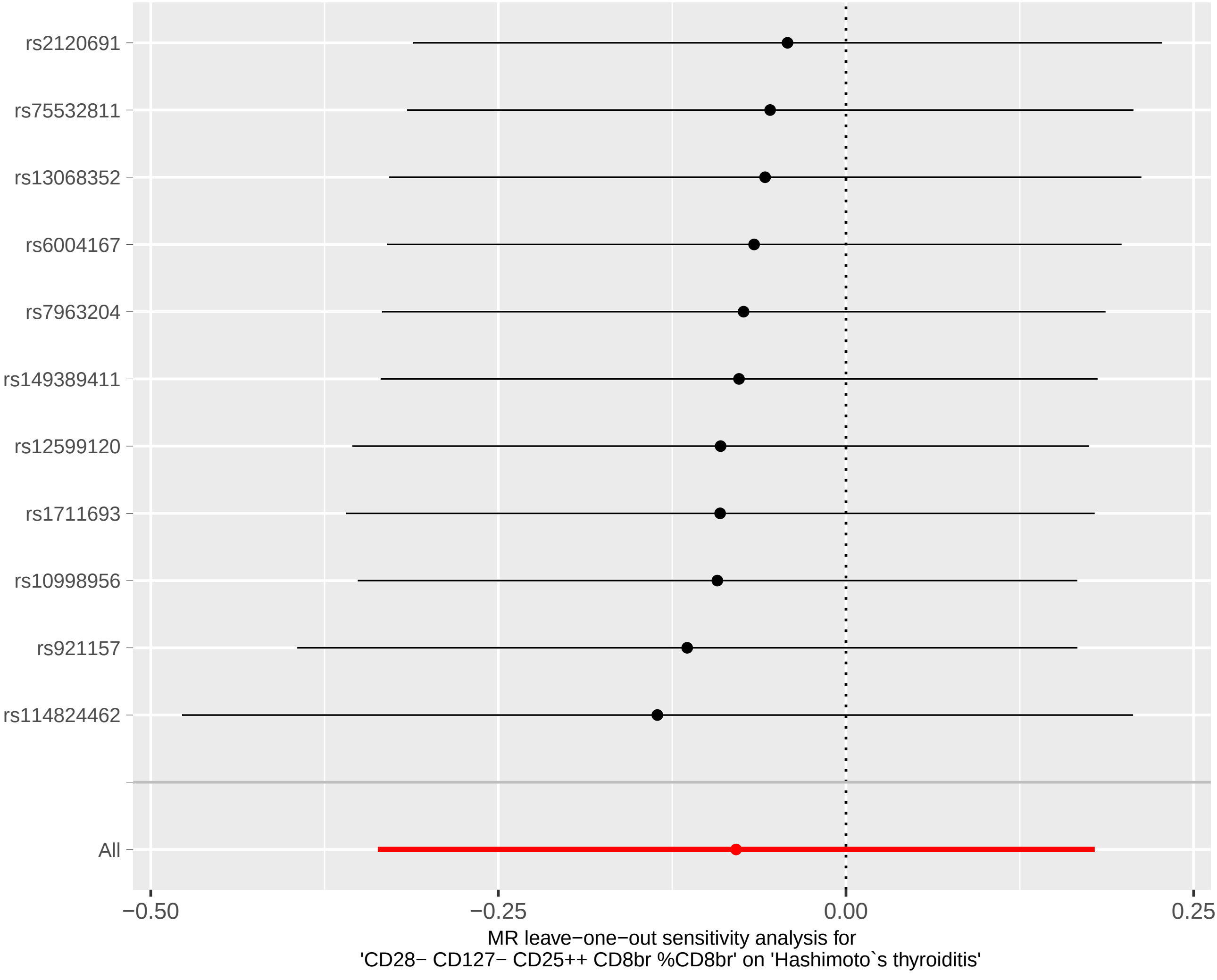
rs2120691
rs75532811
rs13068352
rs6004167
rs7963204
rs149389411
rs12599120
rs1711693
rs10998956
rs921157
rs114824462
All
−0.50
−0.25
0.00
0.25
MR leave−one−out sensitivity analysis for
'CD28− CD127− CD25++ CD8br %CD8br' on 'Hashimoto`s thyroiditis'

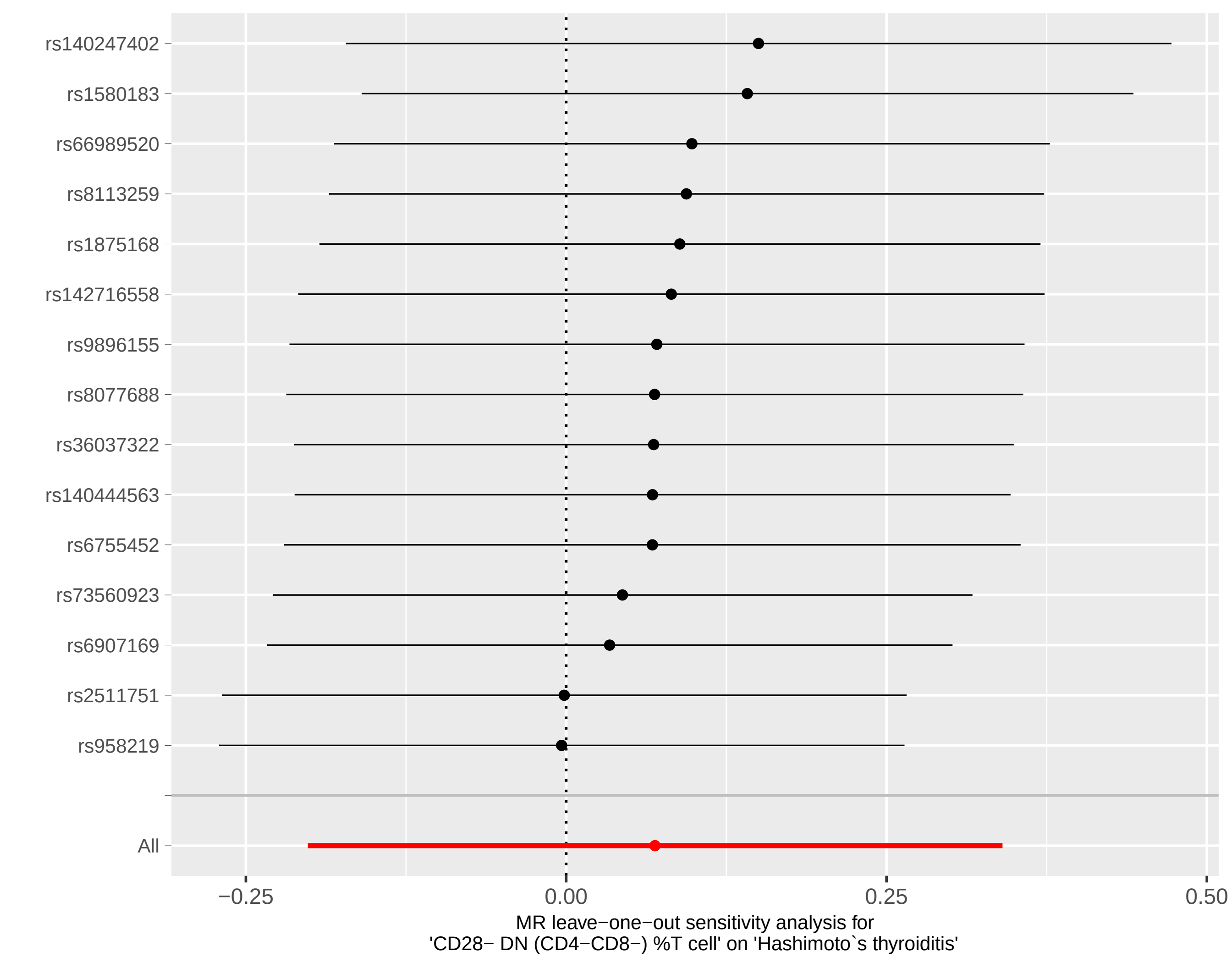
rs140247402
rs1580183
rs66989520
rs8113259
rs1875168
rs142716558
rs9896155
rs8077688
rs36037322
rs140444563
rs6755452
rs73560923
rs6907169
rs2511751
rs958219
All
−0.25
0.00
0.25
0.50
MR leave−one−out sensitivity analysis for
'CD28− DN (CD4−CD8−) %T cell' on 'Hashimoto`s thyroiditis'

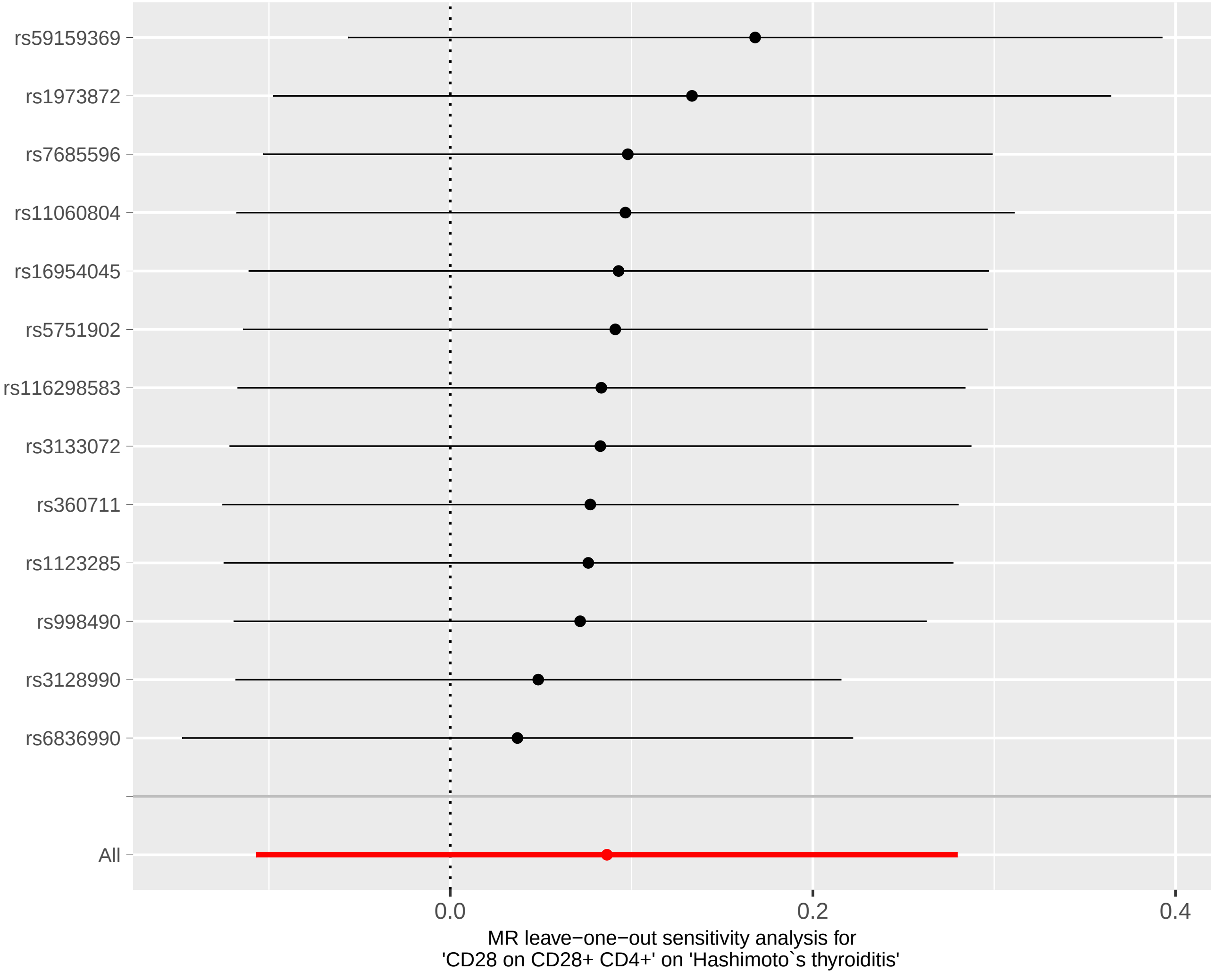
rs59159369
rs1973872
rs7685596
rs11060804
rs16954045
rs5751902
rs116298583
rs3133072
rs360711
rs1123285
rs998490
rs3128990
rs6836990
All
0.0
0.2
0.4
MR leave−one−out sensitivity analysis for
'CD28 on CD28+ CD4+' on 'Hashimoto`s thyroiditis'

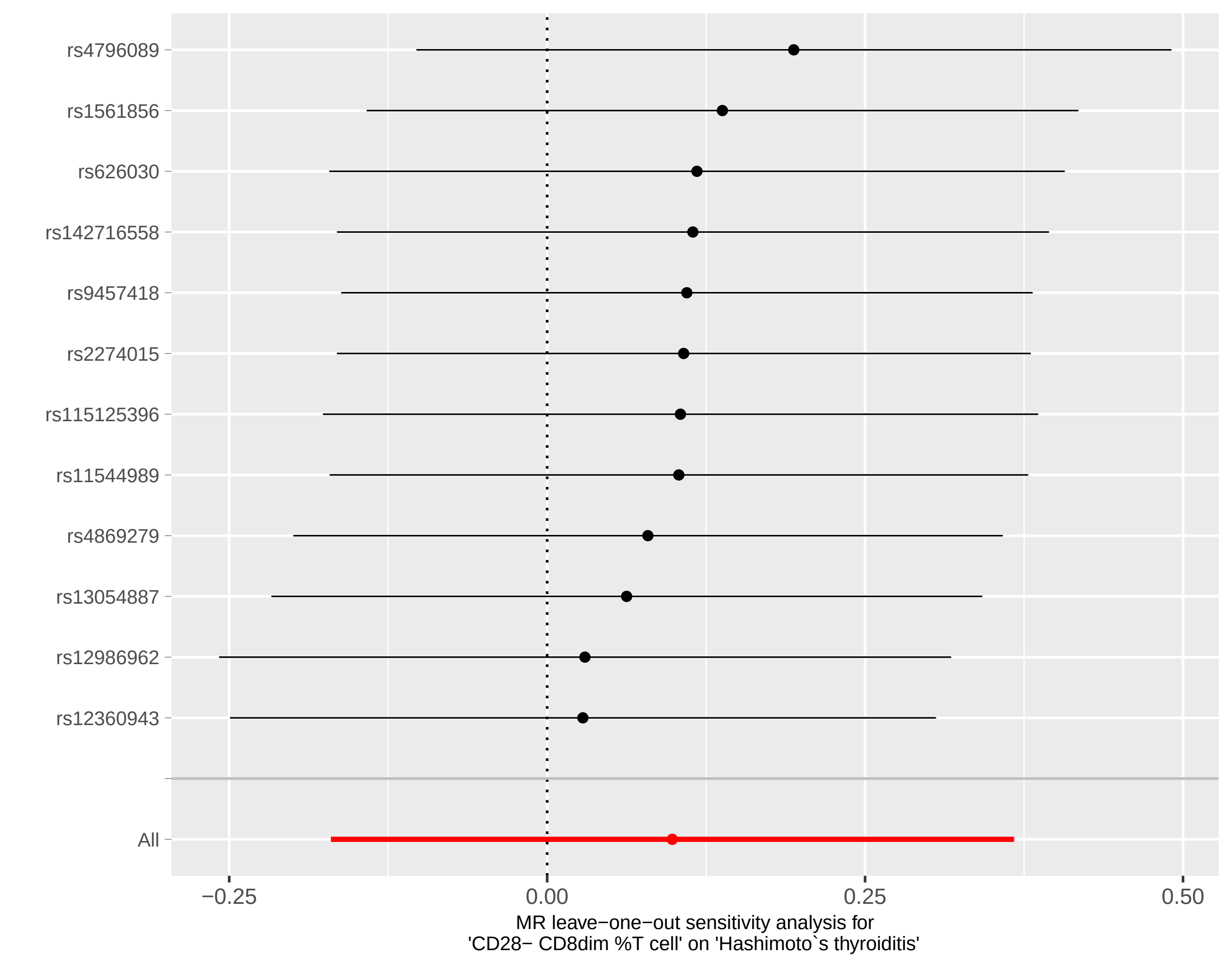
rs4796089
rs1561856
rs626030
rs142716558
rs9457418
rs2274015
rs115125396
rs11544989
rs4869279
rs13054887
rs12986962
rs12360943
All
−0.25
0.00
0.25
0.50
MR leave−one−out sensitivity analysis for
'CD28− CD8dim %T cell' on 'Hashimoto`s thyroiditis'

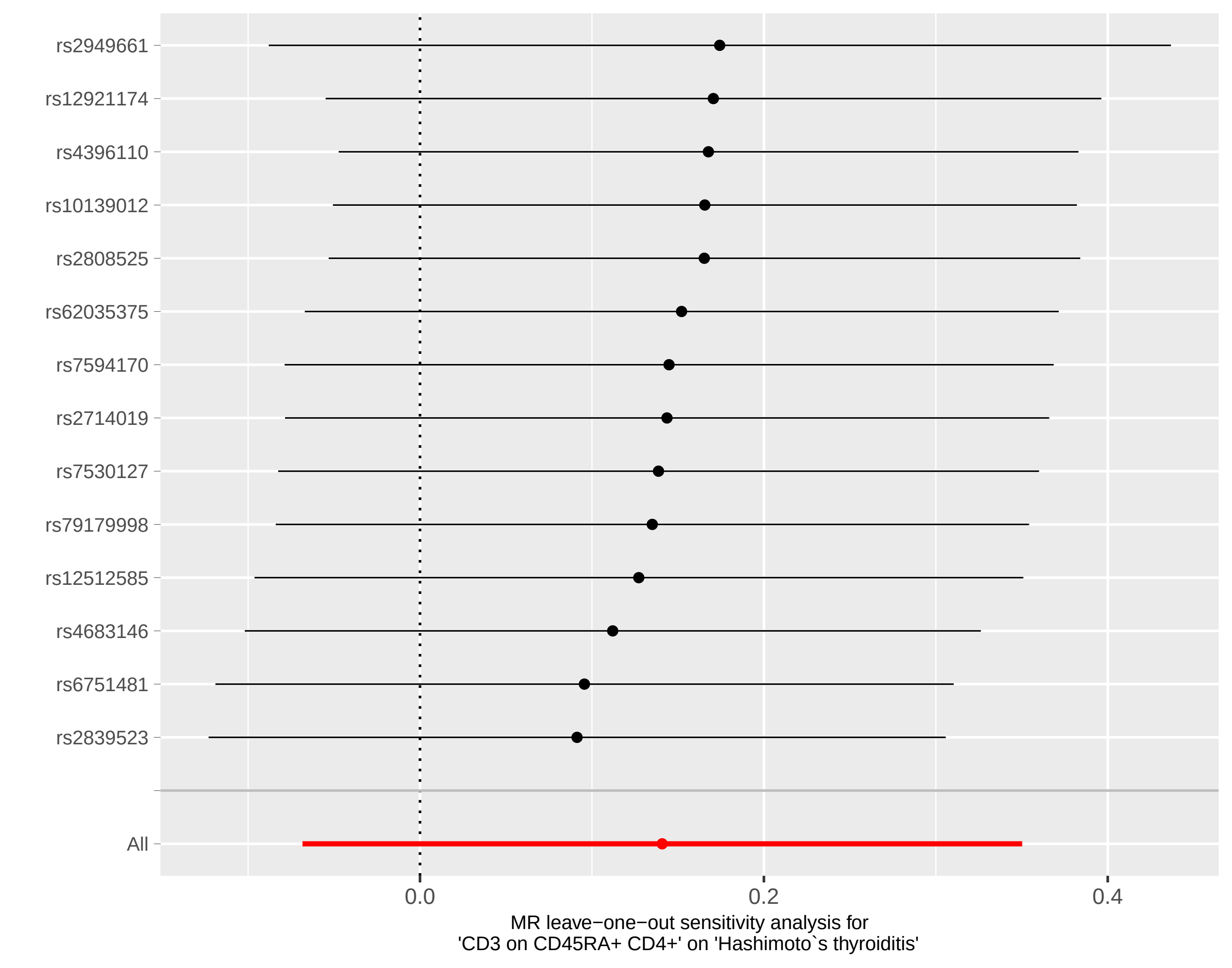
rs2949661
rs12921174
rs4396110
rs10139012
rs2808525
rs62035375
rs7594170
rs2714019
rs7530127
rs79179998
rs12512585
rs4683146
rs6751481
rs2839523
All
0.0
0.2
0.4
MR leave−one−out sensitivity analysis for
'CD3 on CD45RA+ CD4+' on 'Hashimoto`s thyroiditis'

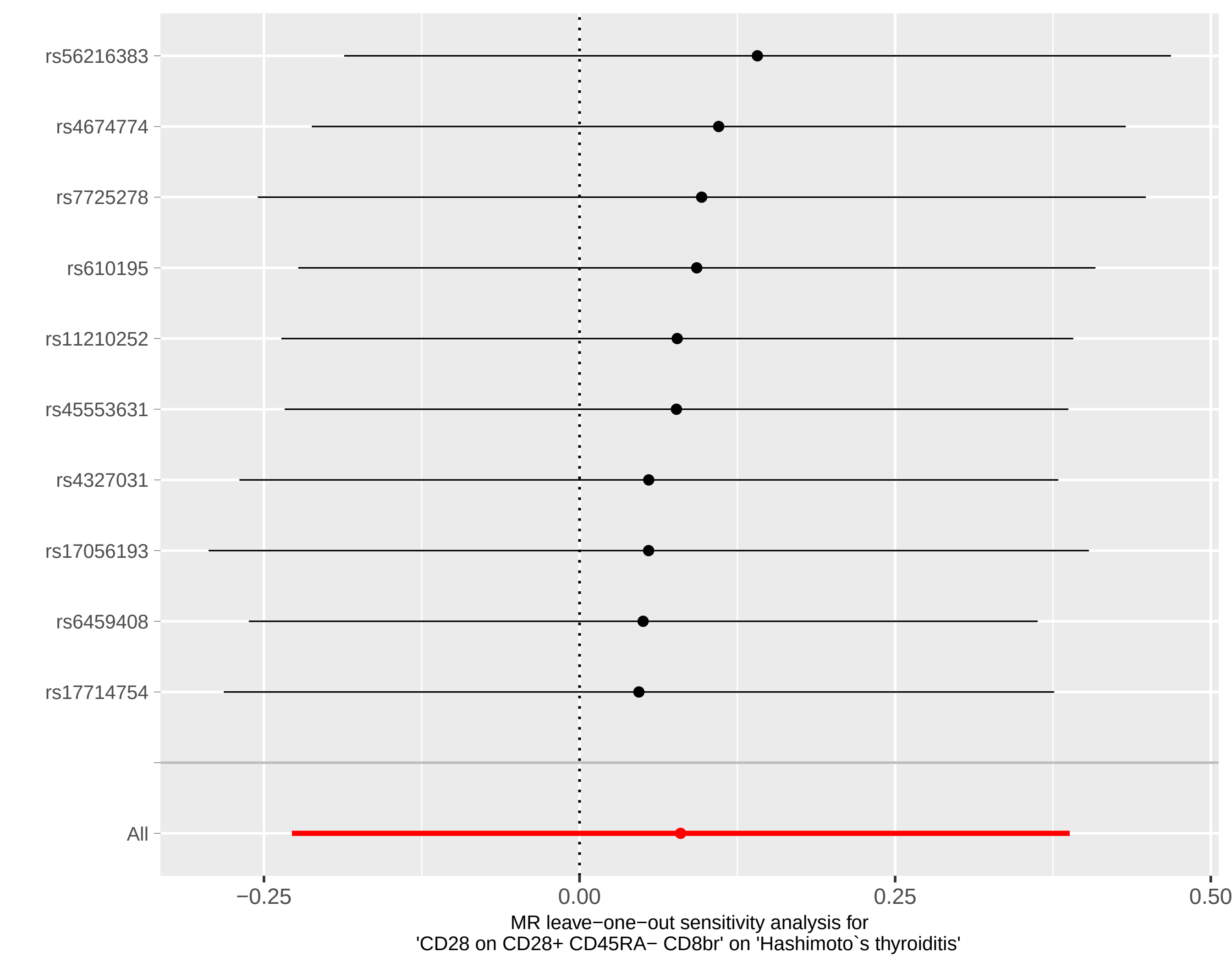
rs56216383
rs4674774
rs7725278
rs610195
rs11210252
rs45553631
rs4327031
rs17056193
rs6459408
rs17714754
All
−0.25
0.00
0.25
0.50
MR leave−one−out sensitivity analysis for
'CD28 on CD28+ CD45RA− CD8br' on 'Hashimoto`s thyroiditis'

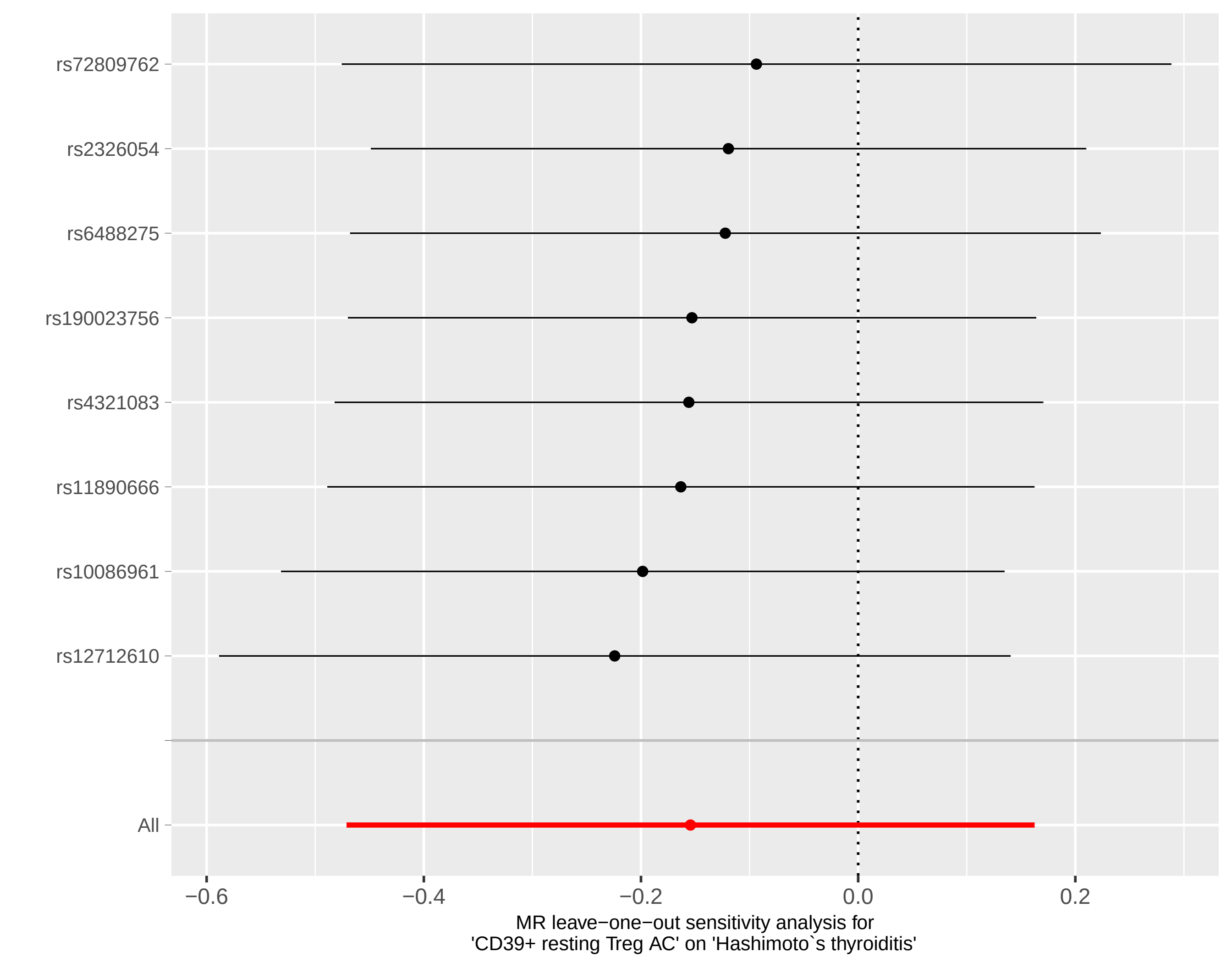
rs72809762
rs2326054
rs6488275
rs190023756
rs4321083
rs11890666
rs10086961
rs12712610
All
−0.6
−0.4
−0.2
0.0
0.2
MR leave−one−out sensitivity analysis for
'CD39+ resting Treg AC' on 'Hashimoto`s thyroiditis'

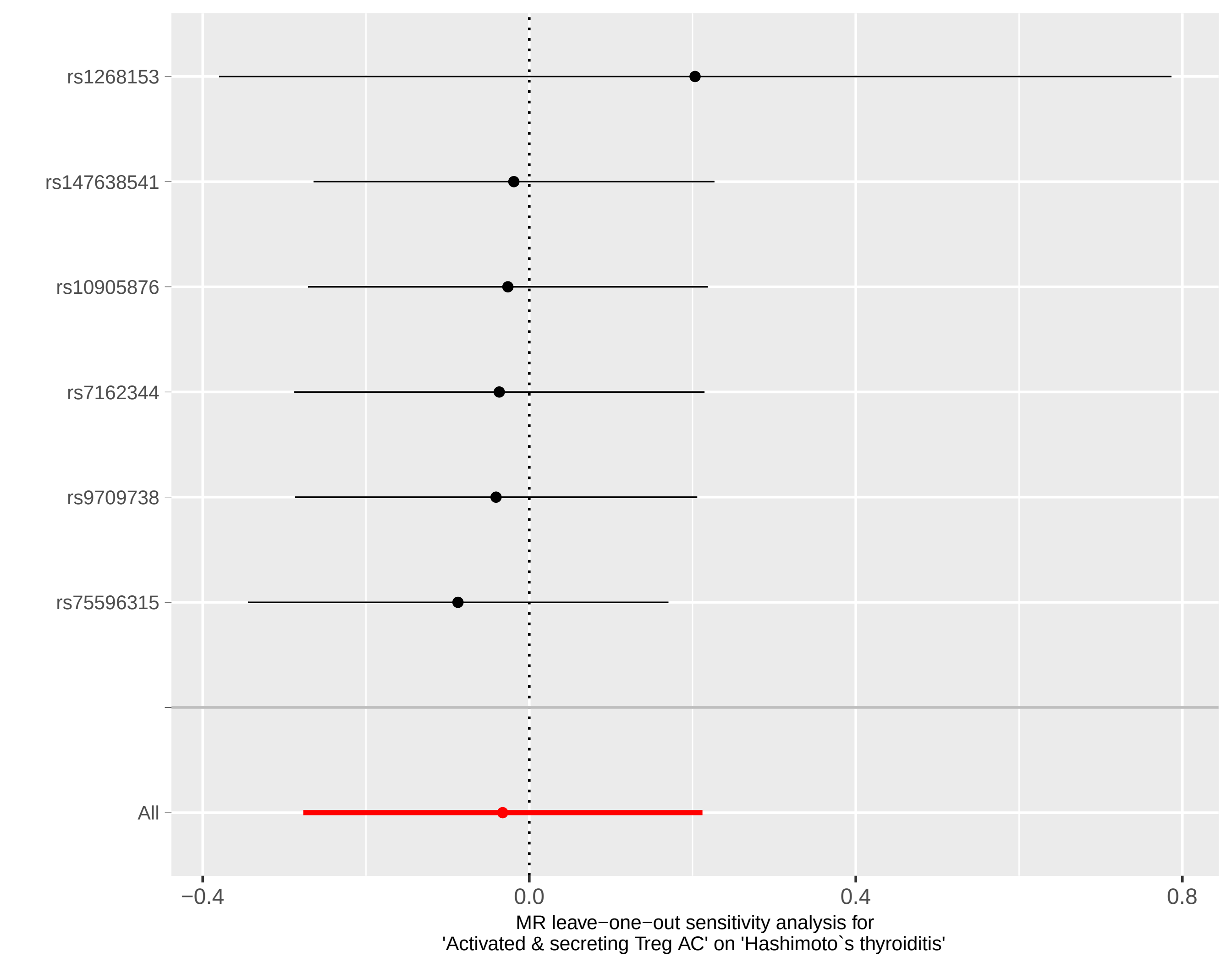
rs1268153
rs147638541
rs10905876
rs7162344
rs9709738
rs75596315
All
−0.4
0.0
0.4
0.8
MR leave−one−out sensitivity analysis for
'Activated & secreting Treg AC' on 'Hashimoto`s thyroiditis'

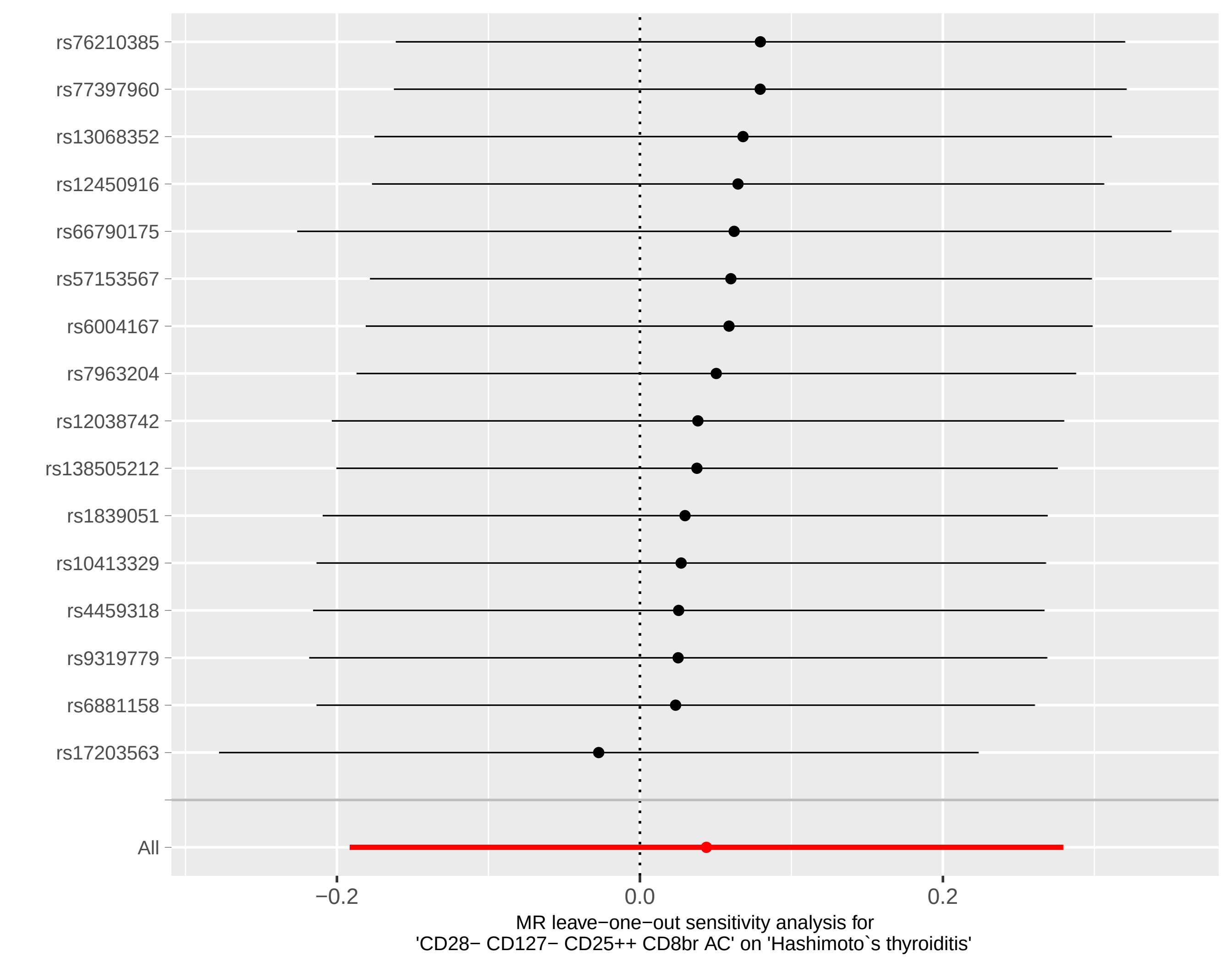

rs76210385
rs77397960
rs13068352
rs12450916
rs66790175
rs57153567
rs6004167
rs7963204
rs12038742
rs138505212
rs1839051
rs10413329
rs4459318
rs9319779
rs6881158
rs17203563
All
−0.2
0.0
0.2
MR leave−one−out sensitivity analysis for
'CD28− CD127− CD25++ CD8br AC' on 'Hashimoto`s thyroiditis'

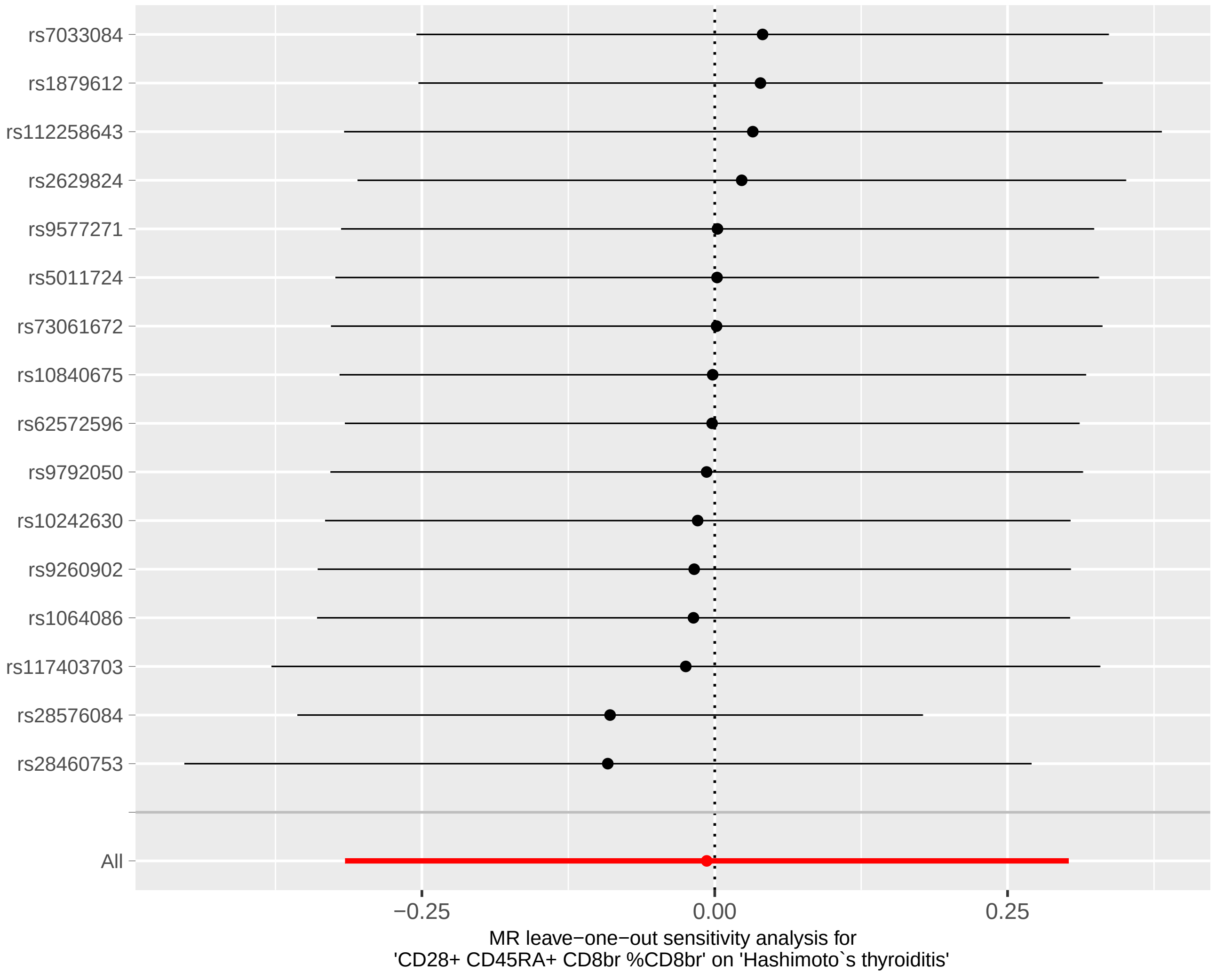
rs7033084
rs1879612
rs112258643
rs2629824
rs9577271
rs5011724
rs73061672
rs10840675
rs62572596
rs9792050
rs10242630
rs9260902
rs1064086
rs117403703
rs28576084
rs28460753
All
−0.25
0.00
0.25
MR leave−one−out sensitivity analysis for
'CD28+ CD45RA+ CD8br %CD8br' on 'Hashimoto`s thyroiditis'

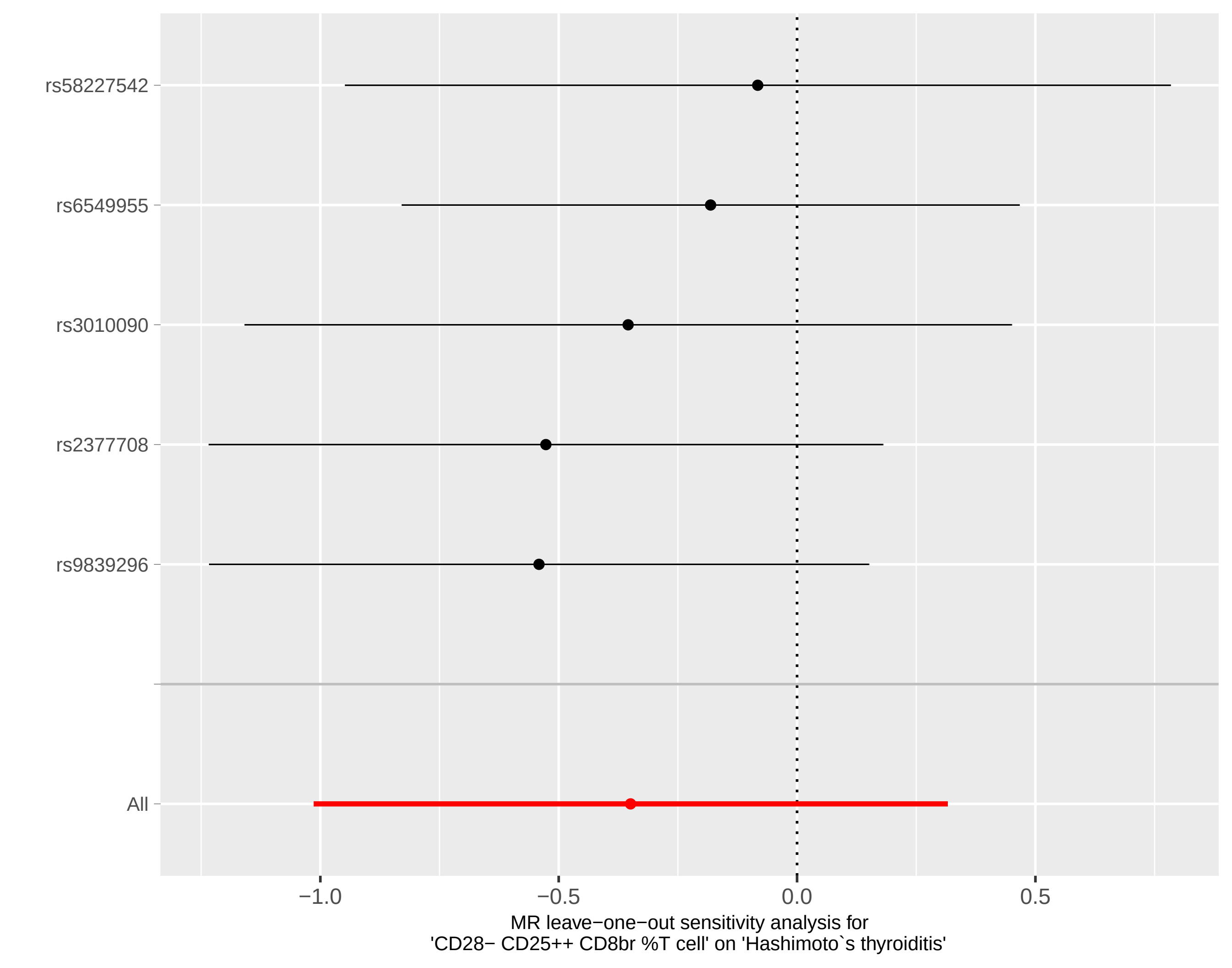
rs58227542
rs6549955
rs3010090
rs2377708
rs9839296
All
−1.0
−0.5
0.0
0.5
MR leave−one−out sensitivity analysis for
'CD28− CD25++ CD8br %T cell' on 'Hashimoto`s thyroiditis'

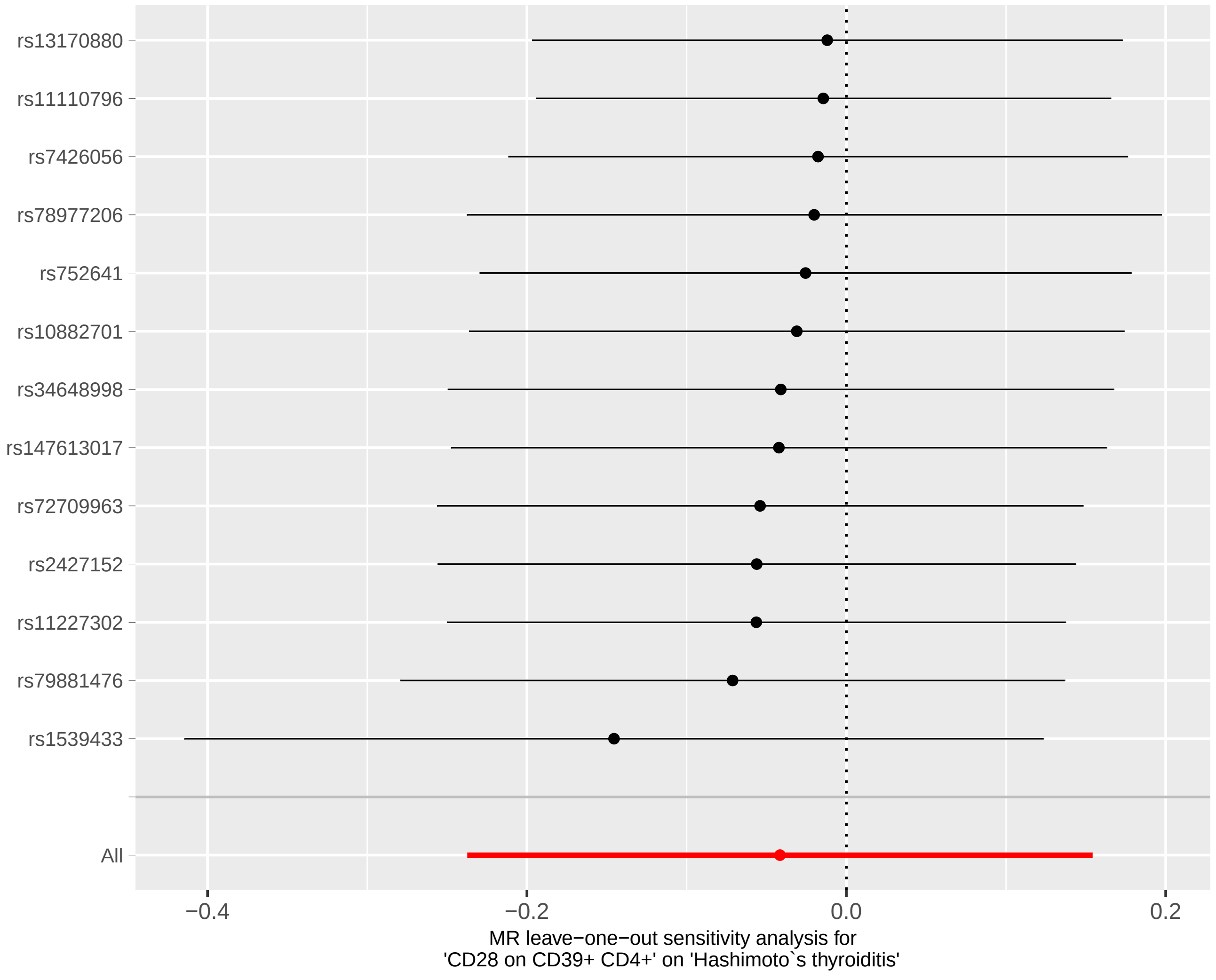
rs13170880
rs11110796
rs7426056
rs78977206
rs752641
rs10882701
rs34648998
rs147613017
rs72709963
rs2427152
rs11227302
rs79881476
rs1539433
All
−0.4
−0.2
0.0
0.2
MR leave−one−out sensitivity analysis for
'CD28 on CD39+ CD4+' on 'Hashimoto`s thyroiditis'

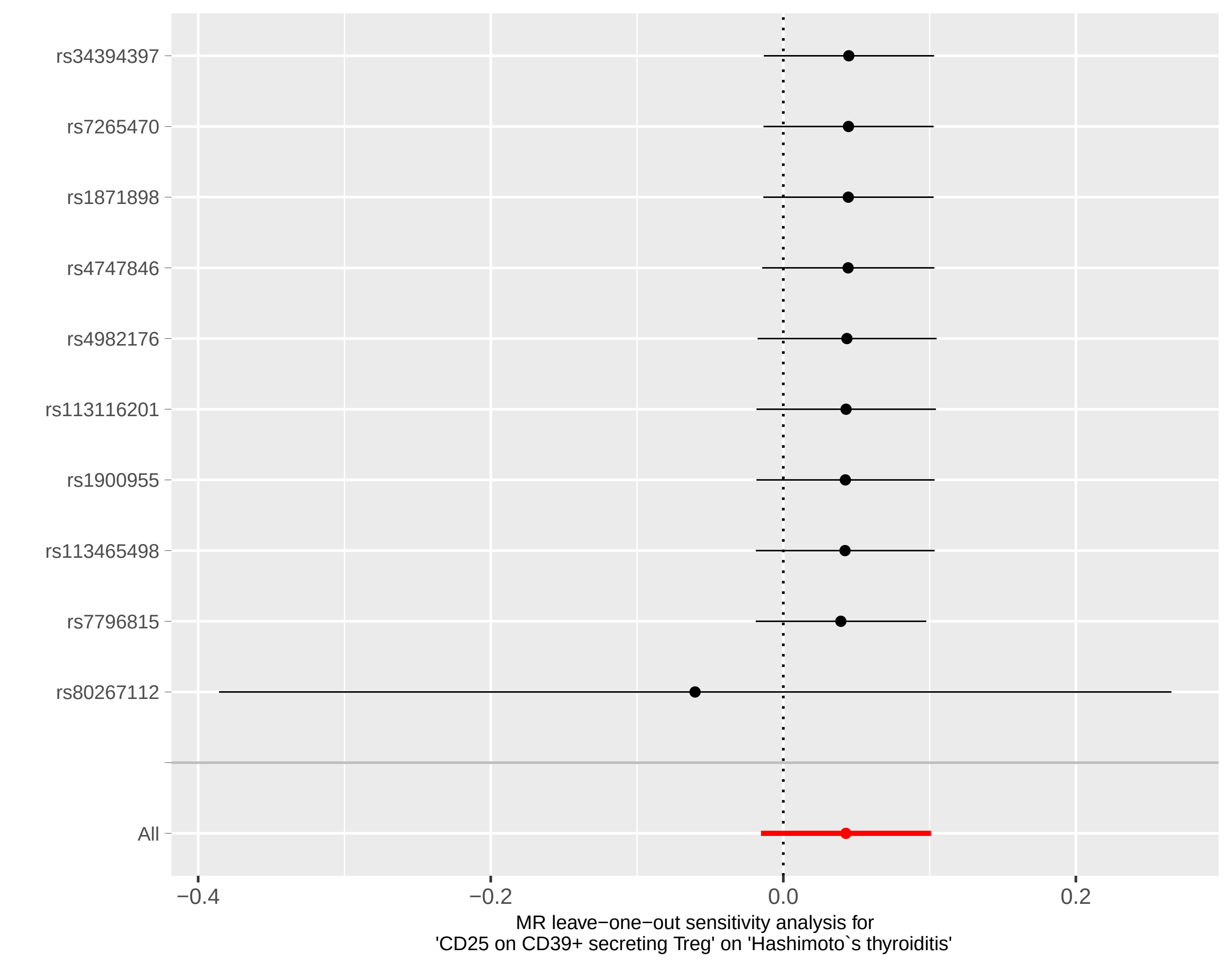
rs34394397
rs7265470
rs1871898
rs4747846
rs4982176
rs113116201
rs1900955
rs113465498
rs7796815
rs80267112
All
−0.4
−0.2
0.0
0.2
MR leave−one−out sensitivity analysis for
'CD25 on CD39+ secreting Treg' on 'Hashimoto`s thyroiditis'

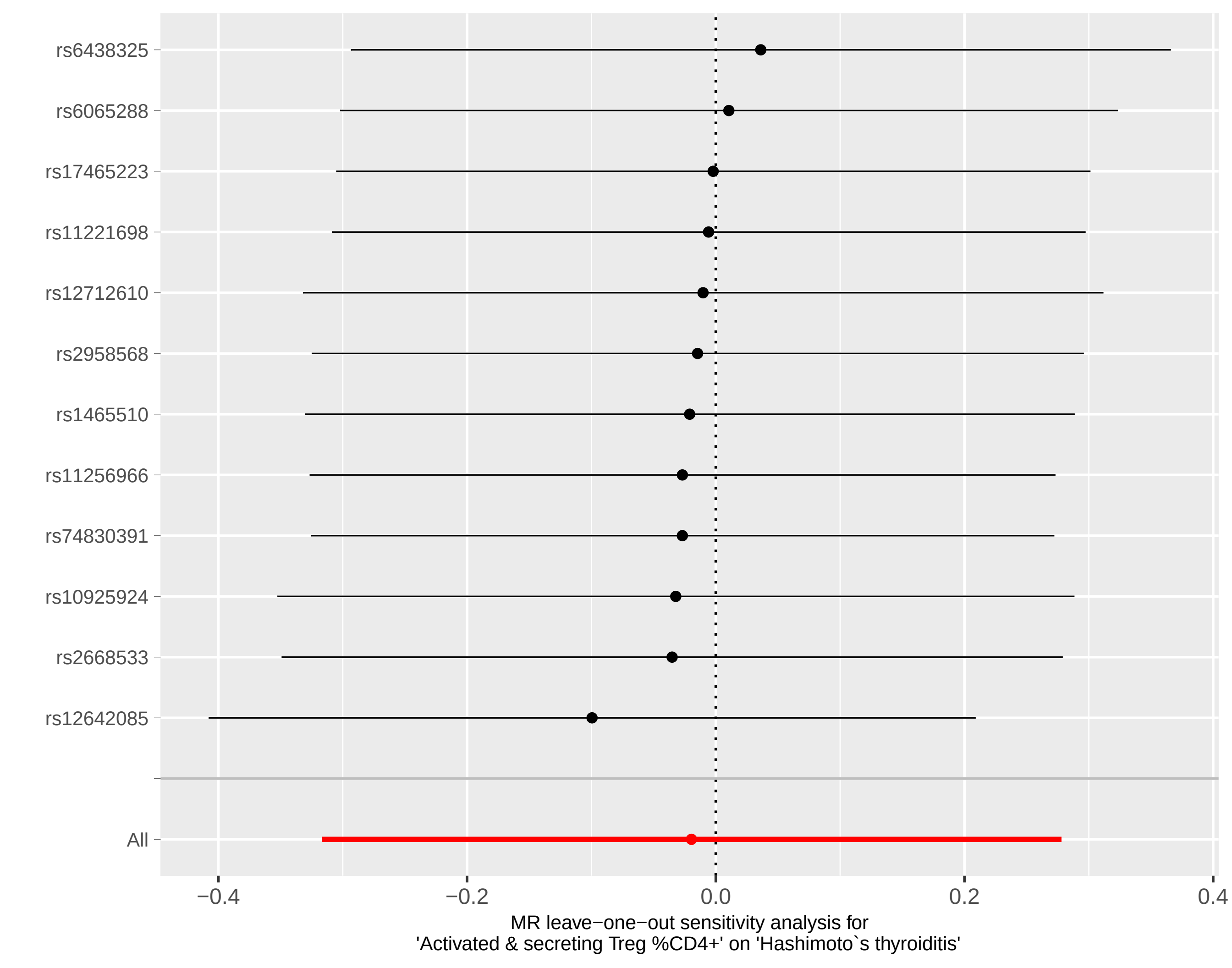
rs6438325
rs6065288
rs17465223
rs11221698
rs12712610
rs2958568
rs1465510
rs11256966
rs74830391
rs10925924
rs2668533
rs12642085
All
−0.4
−0.2
0.0
0.2
0.4
MR leave−one−out sensitivity analysis for
'Activated & secreting Treg %CD4+' on 'Hashimoto`s thyroiditis'

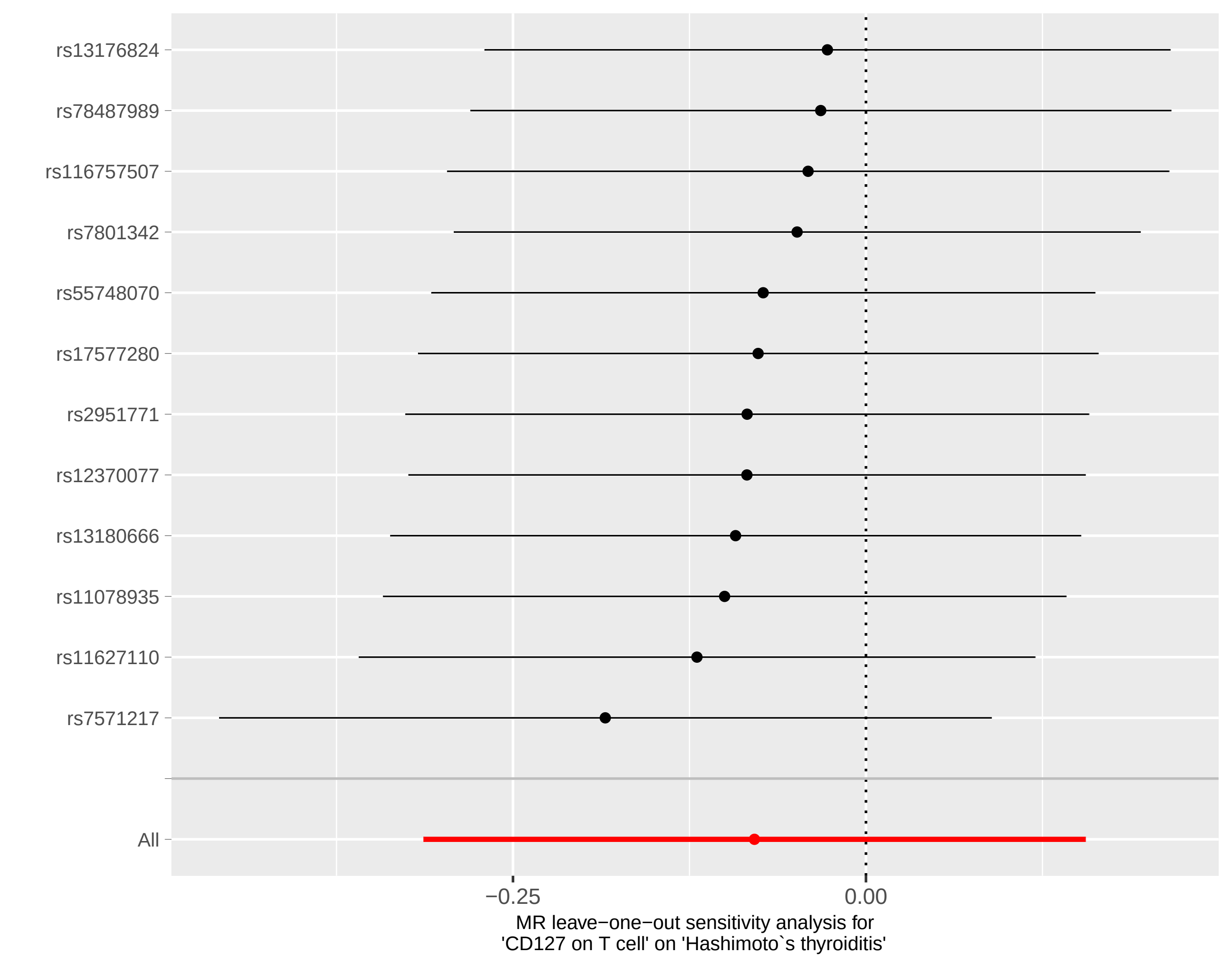
rs13176824
rs78487989
rs116757507
rs7801342
rs55748070
rs17577280
rs2951771
rs12370077
rs13180666
rs11078935
rs11627110
rs7571217
All
−0.25
0.00
MR leave−one−out sensitivity analysis for
'CD127 on T cell' on 'Hashimoto`s thyroiditis'

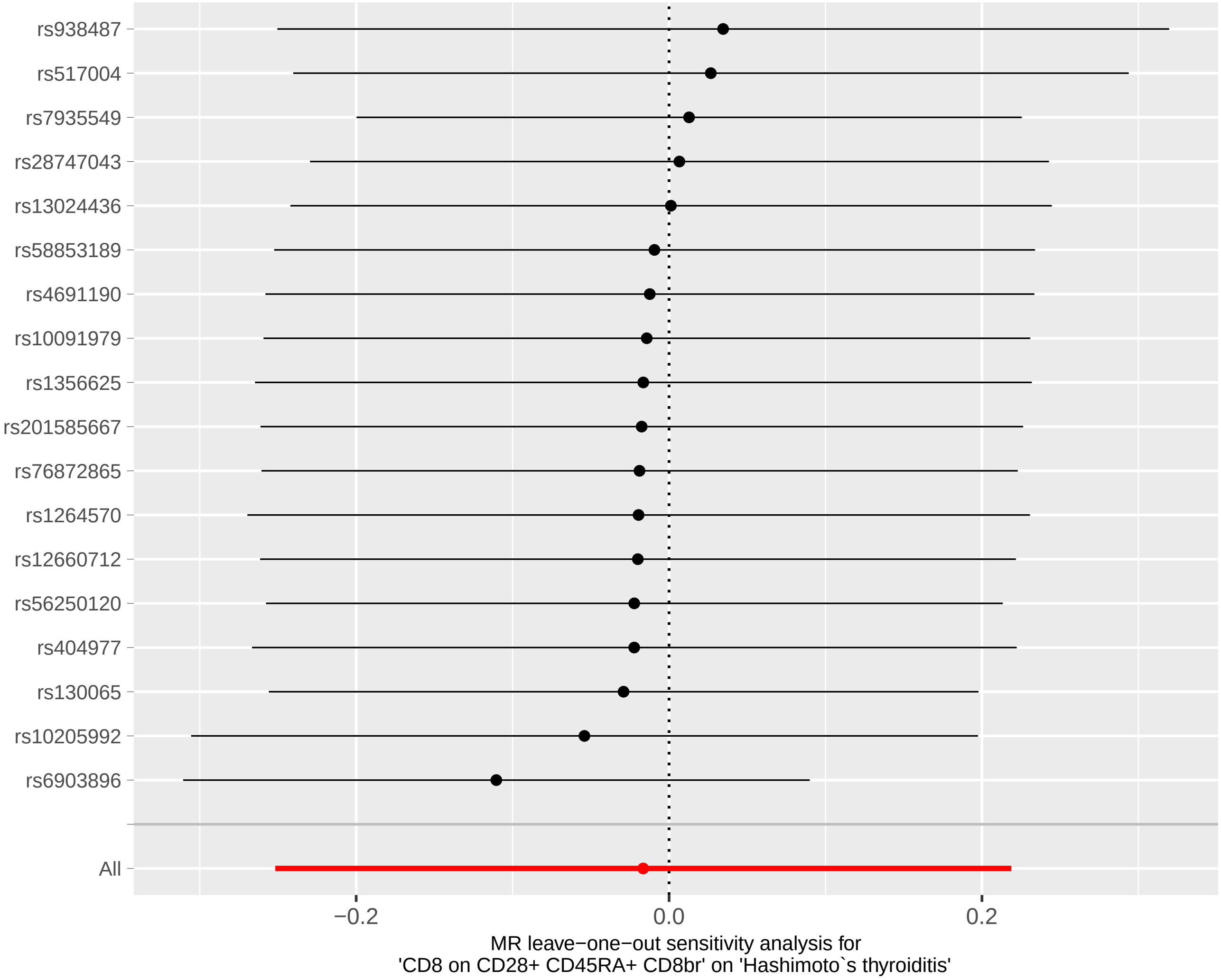
rs938487
rs517004
rs7935549
rs28747043
rs13024436
rs58853189
rs4691190
rs10091979
rs1356625
rs201585667
rs76872865
rs1264570
rs12660712
rs56250120
rs404977
rs130065
rs10205992
rs6903896
All
−0.2
0.0
0.2
MR leave−one−out sensitivity analysis for
'CD8 on CD28+ CD45RA+ CD8br' on 'Hashimoto`s thyroiditis'

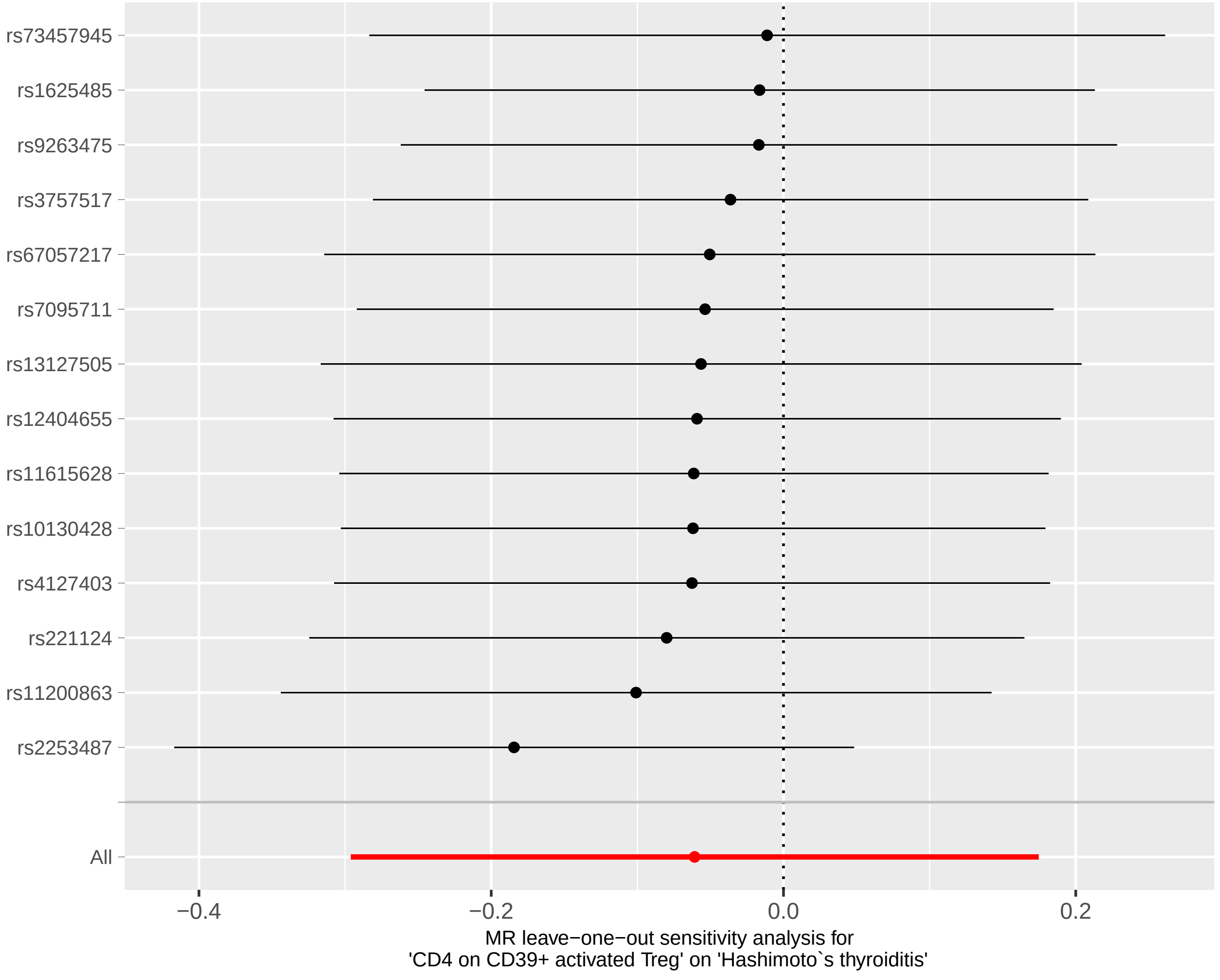
rs73457945
rs1625485
rs9263475
rs3757517
rs67057217
rs7095711
rs13127505
rs12404655
rs11615628
rs10130428
rs4127403
rs221124
rs11200863
rs2253487
All
−0.4
−0.2
0.0
0.2
MR leave−one−out sensitivity analysis for
'CD4 on CD39+ activated Treg' on 'Hashimoto`s thyroiditis'

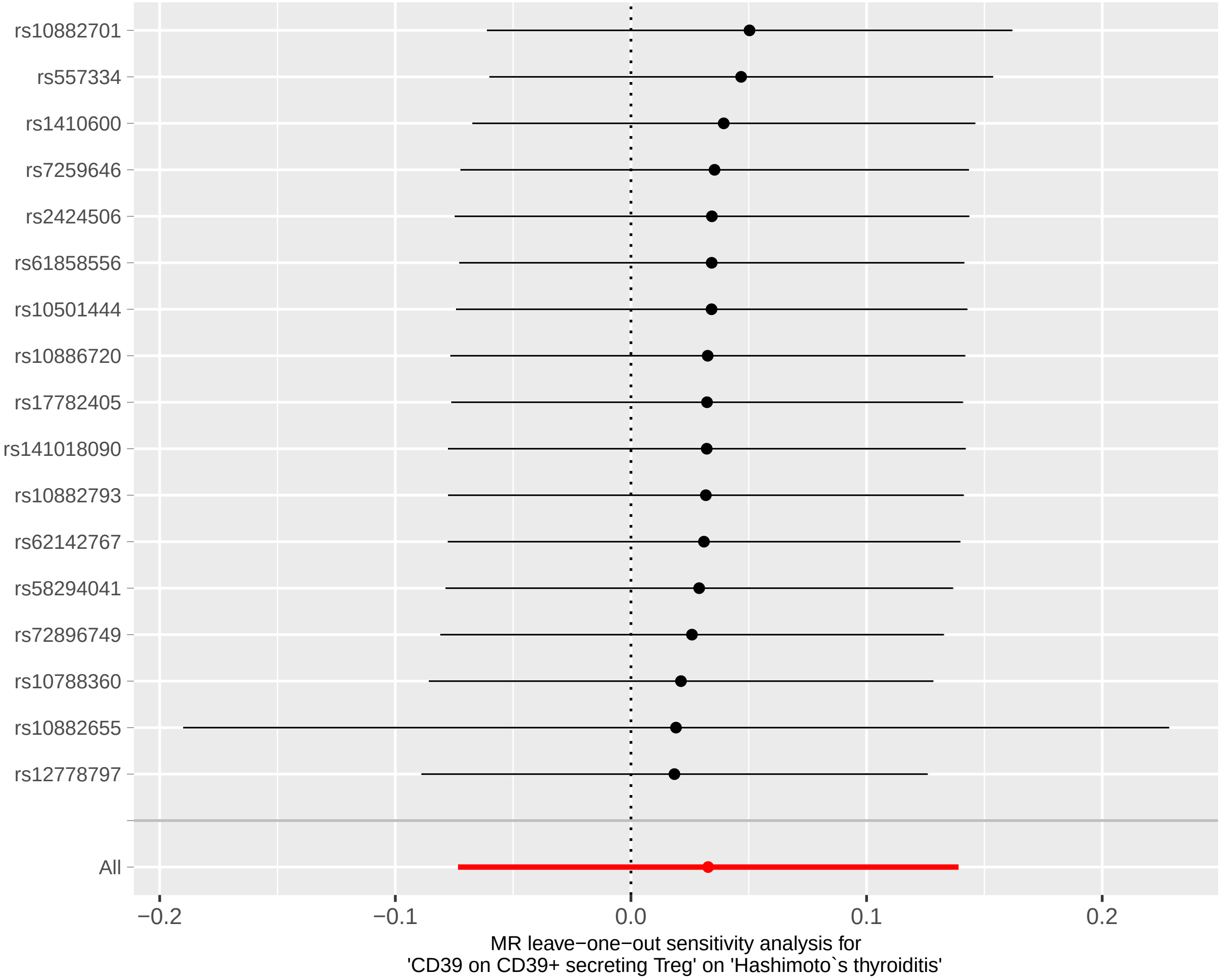
rs10882701
rs557334
rs1410600
rs7259646
rs2424506
rs61858556
rs10501444
rs10886720
rs17782405
rs141018090
rs10882793
rs62142767
rs58294041
rs72896749
rs10788360
rs10882655
rs12778797
All
−0.2
−0.1
0.0
0.1
0.2
MR leave−one−out sensitivity analysis for
'CD39 on CD39+ secreting Treg' on 'Hashimoto`s thyroiditis'

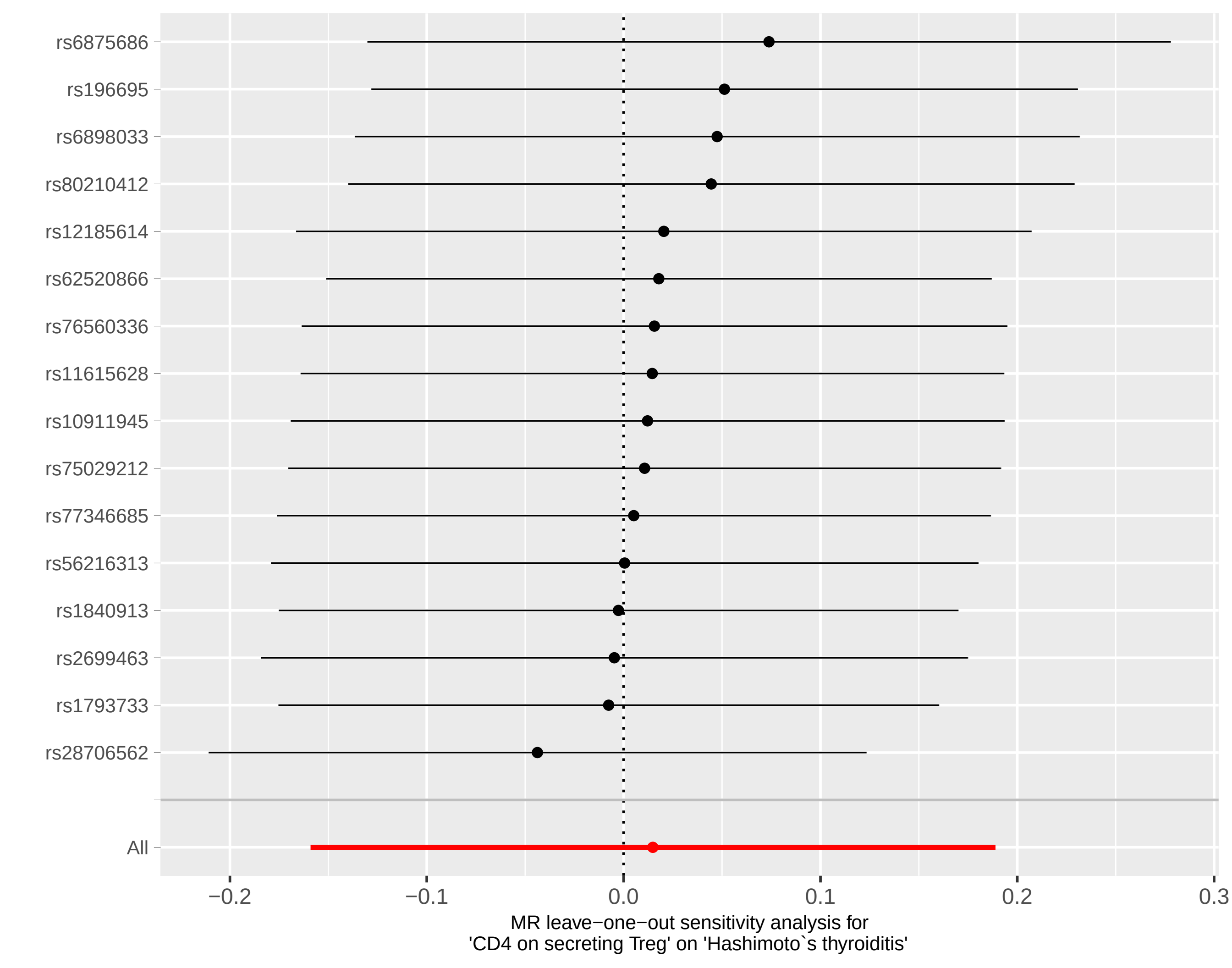
rs6875686
rs196695
rs6898033
rs80210412
rs12185614
rs62520866
rs76560336
rs11615628
rs10911945
rs75029212
rs77346685
rs56216313
rs1840913
rs2699463
rs1793733
rs28706562
All
−0.2
−0.1
0.0
0.1
0.2
0.3
MR leave−one−out sensitivity analysis for
'CD4 on secreting Treg' on 'Hashimoto`s thyroiditis'

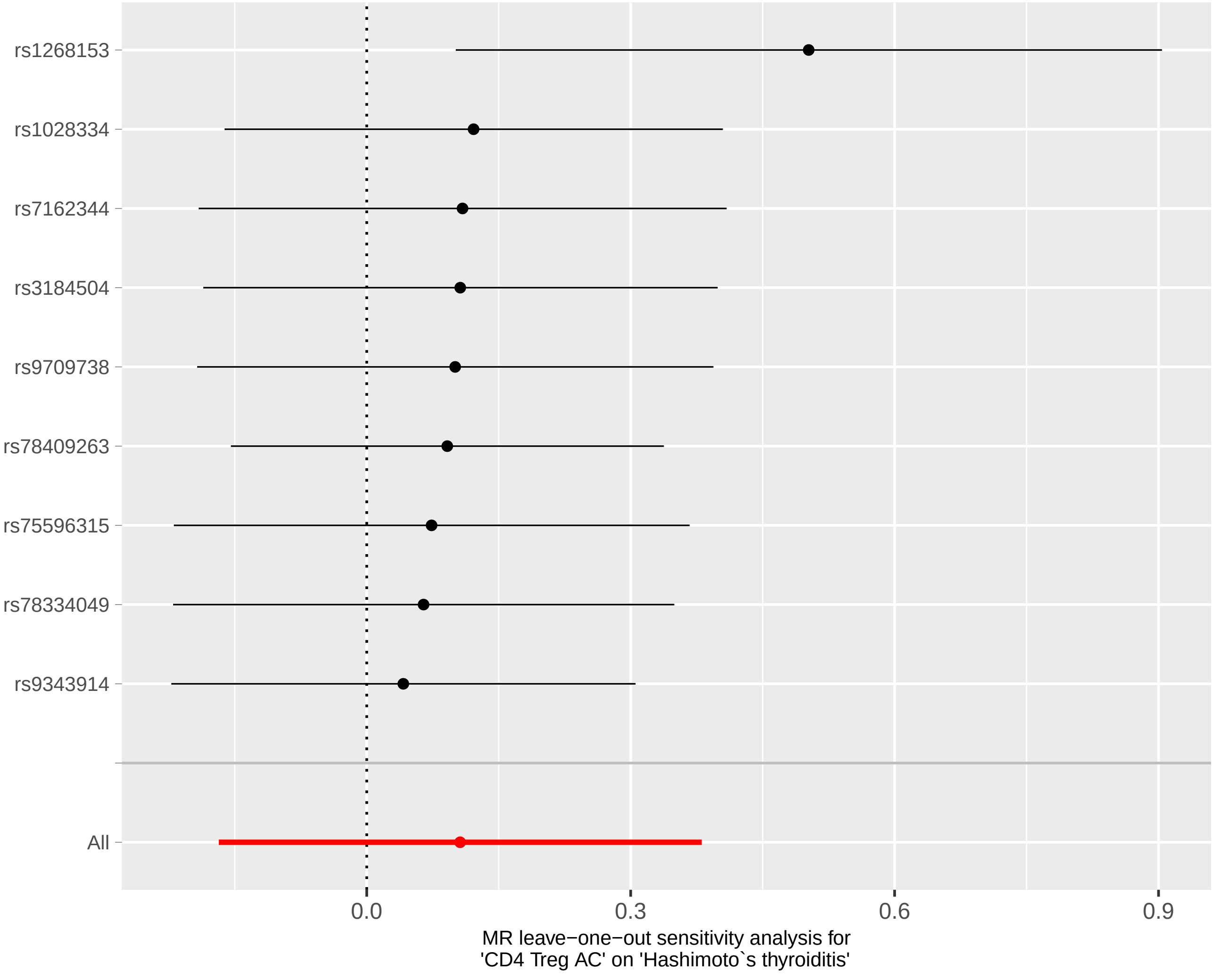
rs1268153
rs1028334
rs7162344
rs3184504
rs9709738
rs78409263
rs75596315
rs78334049
rs9343914
All
0.0
0.3
0.6
0.9
MR leave−one−out sensitivity analysis for
'CD4 Treg AC' on 'Hashimoto`s thyroiditis'

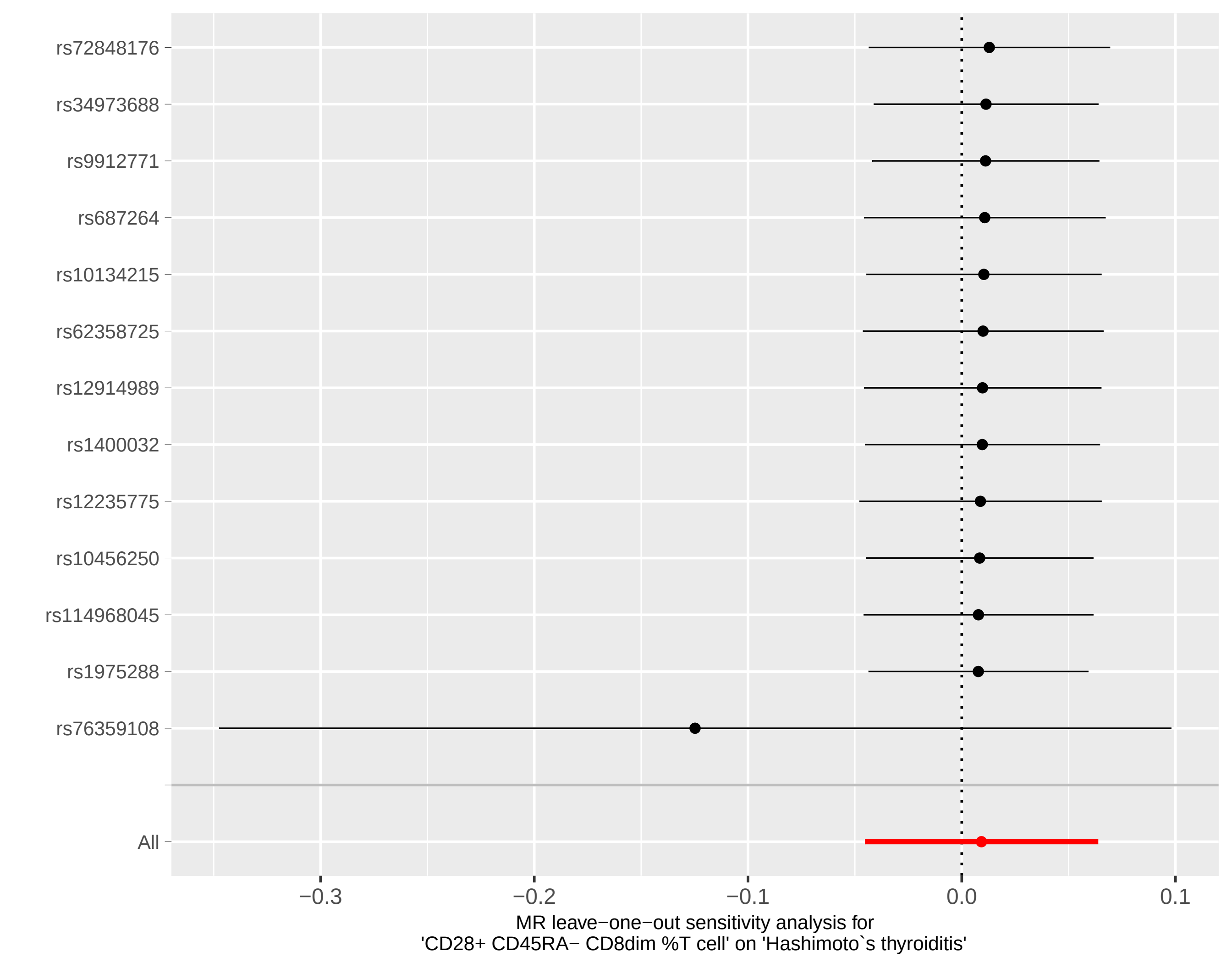

rs72848176
rs34973688
rs9912771
rs687264
rs10134215
rs62358725
rs12914989
rs1400032
rs12235775
rs10456250
rs114968045
rs1975288
rs76359108
All
−0.3
−0.2
−0.1
0.0
0.1
MR leave−one−out sensitivity analysis for
'CD28+ CD45RA− CD8dim %T cell' on 'Hashimoto`s thyroiditis'

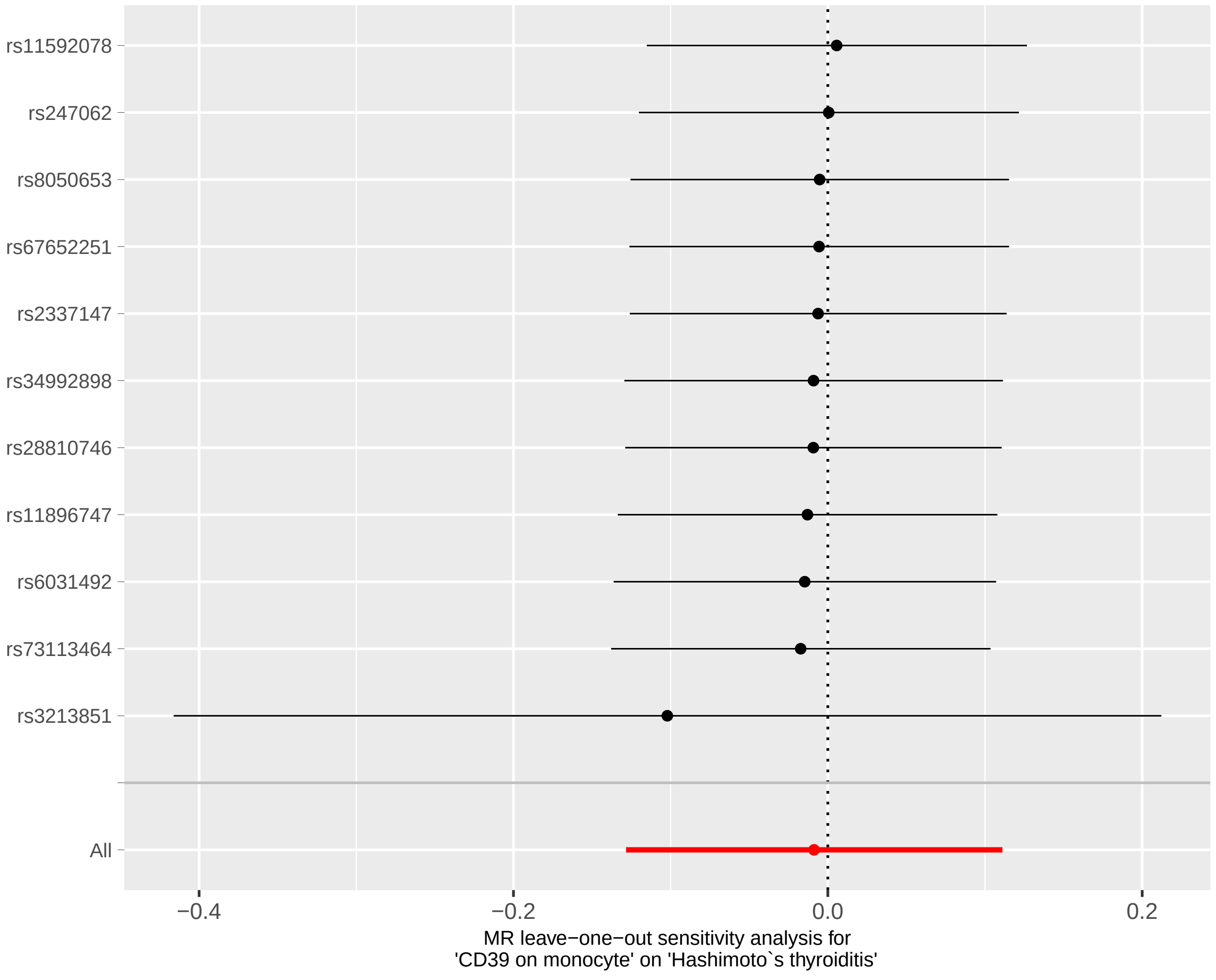
rs11592078
rs247062
rs8050653
rs67652251
rs2337147
rs34992898
rs28810746
rs11896747
rs6031492
rs73113464
rs3213851
All
−0.4
−0.2
0.0
0.2
MR leave−one−out sensitivity analysis for
'CD39 on monocyte' on 'Hashimoto`s thyroiditis'

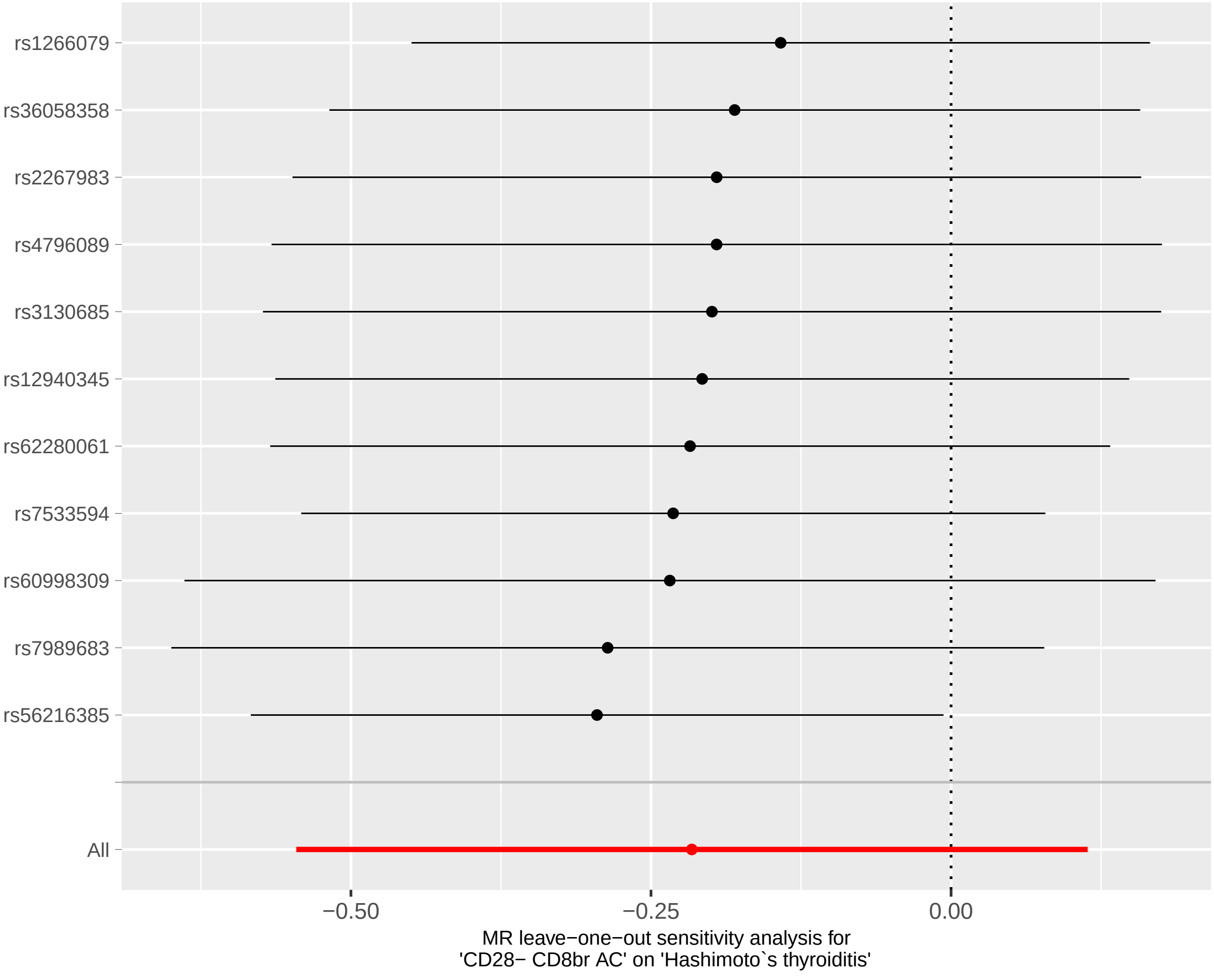
rs1266079
rs36058358
rs2267983
rs4796089
rs3130685
rs12940345
rs62280061
rs7533594
rs60998309
rs7989683
rs56216385
All
−0.50
−0.25
0.00
MR leave−one−out sensitivity analysis for
'CD28− CD8br AC' on 'Hashimoto`s thyroiditis'

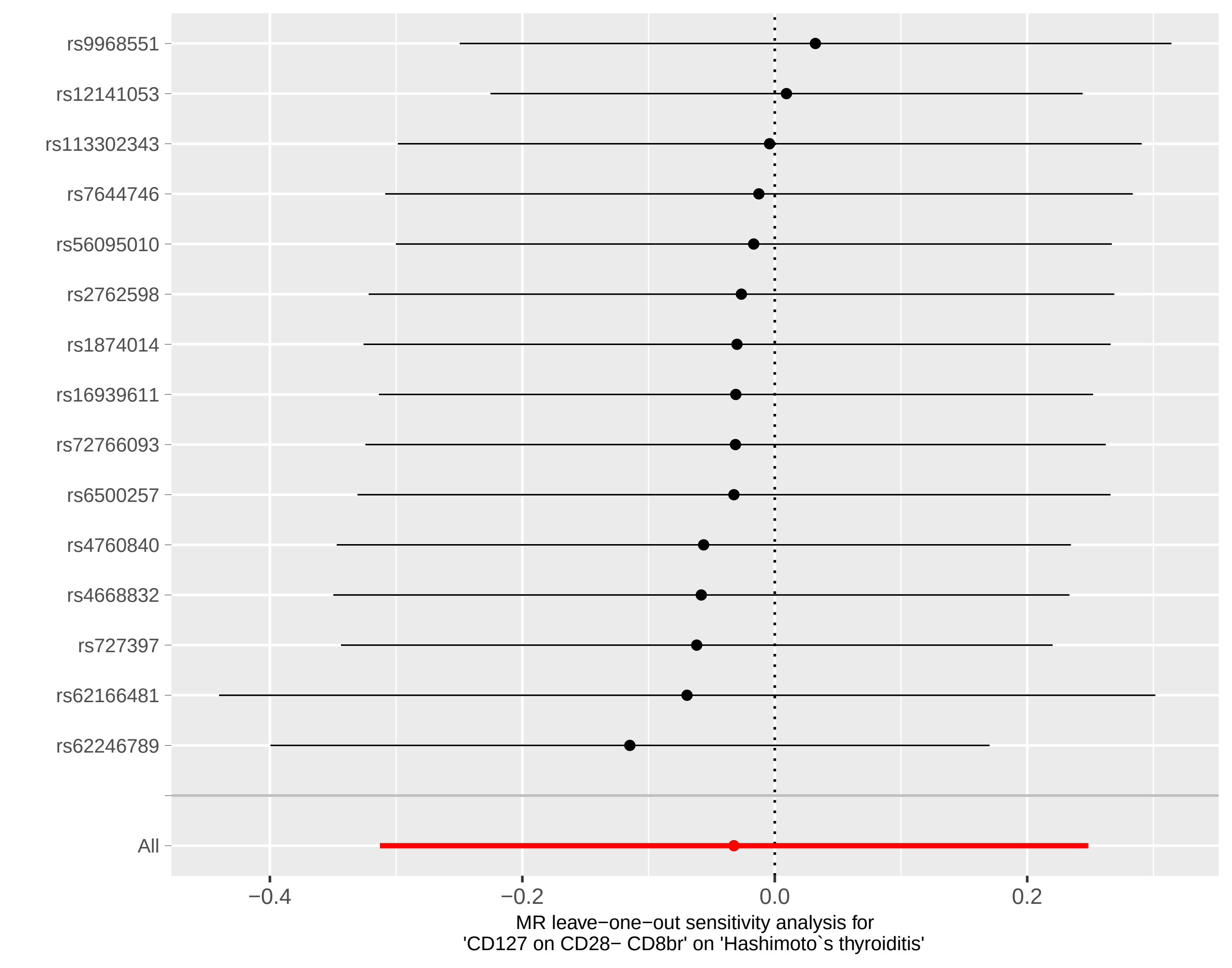
rs9968551
rs12141053
rs113302343
rs7644746
rs56095010
rs2762598
rs1874014
rs16939611
rs72766093
rs6500257
rs4760840
rs4668832
rs727397
rs62166481
rs62246789
All
−0.4
−0.2
0.0
0.2
MR leave−one−out sensitivity analysis for
'CD127 on CD28− CD8br' on 'Hashimoto`s thyroiditis'

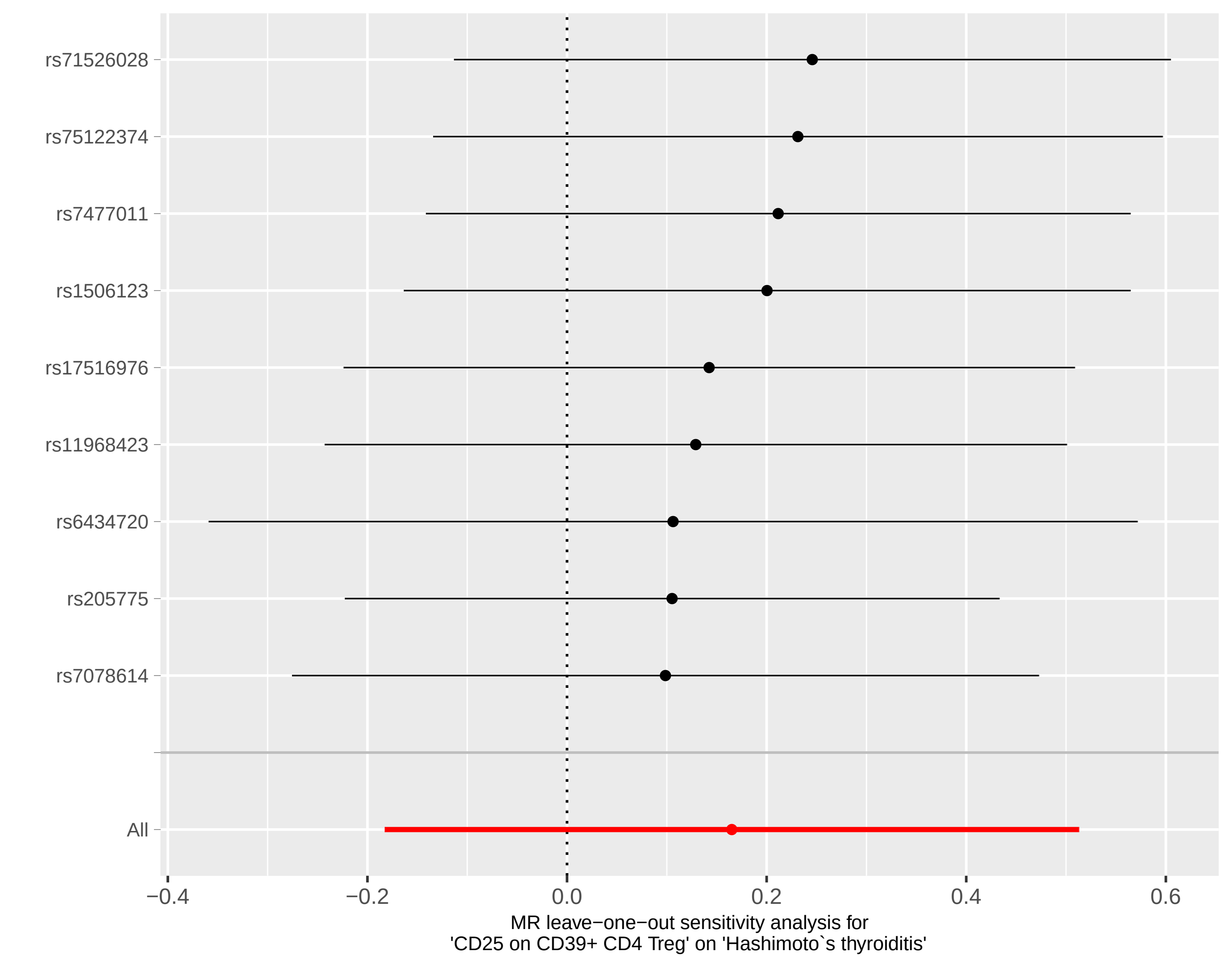
rs71526028
rs75122374
rs7477011
rs1506123
rs17516976
rs11968423
rs6434720
rs205775
rs7078614
All
−0.4
−0.2
0.0
0.2
0.4
0.6
MR leave−one−out sensitivity analysis for
'CD25 on CD39+ CD4 Treg' on 'Hashimoto`s thyroiditis'

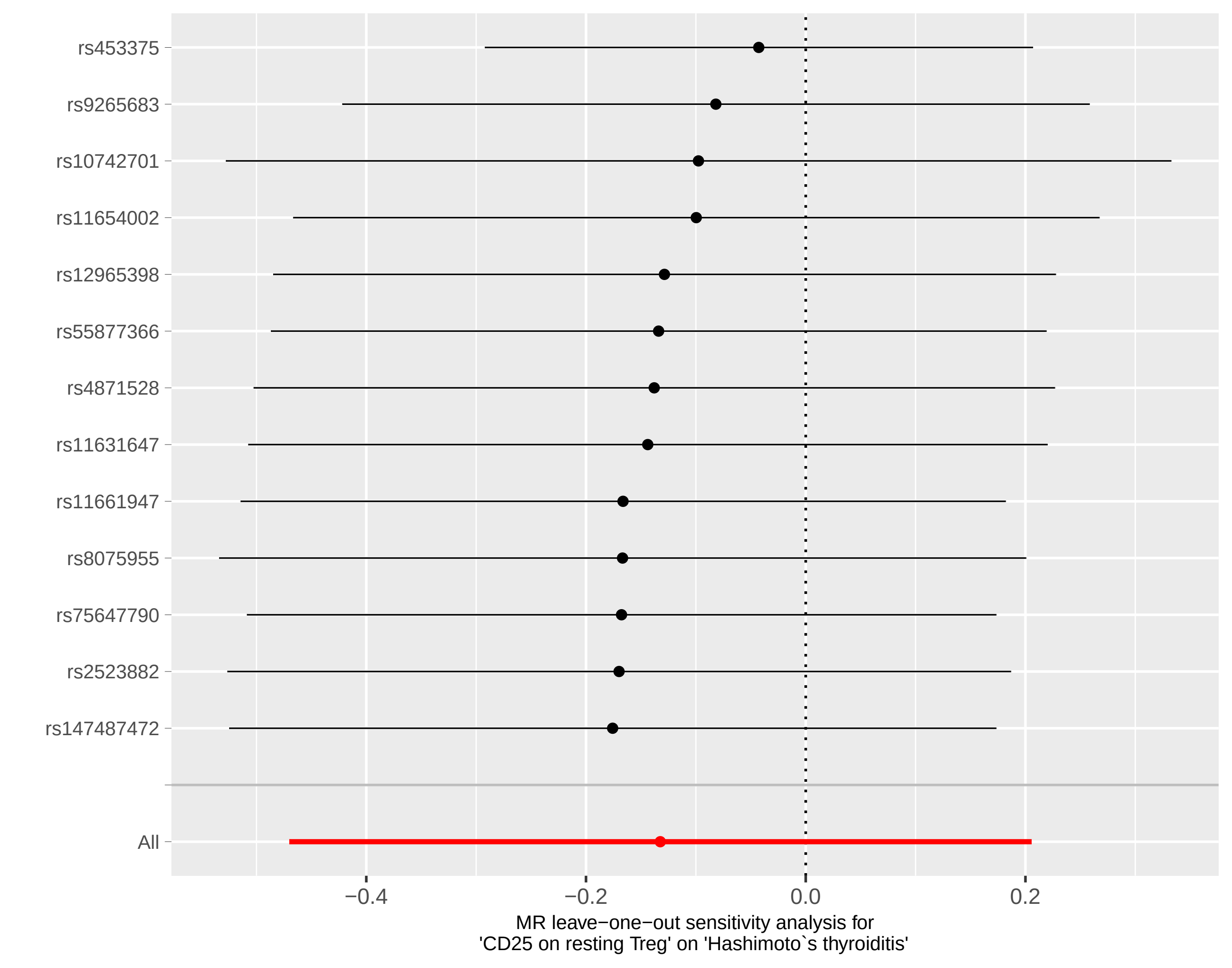

rs453375
rs9265683
rs10742701
rs11654002
rs12965398
rs55877366
rs4871528
rs11631647
rs11661947
rs8075955
rs75647790
rs2523882
rs147487472
All
−0.4
−0.2
0.0
0.2
MR leave−one−out sensitivity analysis for
'CD25 on resting Treg' on 'Hashimoto`s thyroiditis'

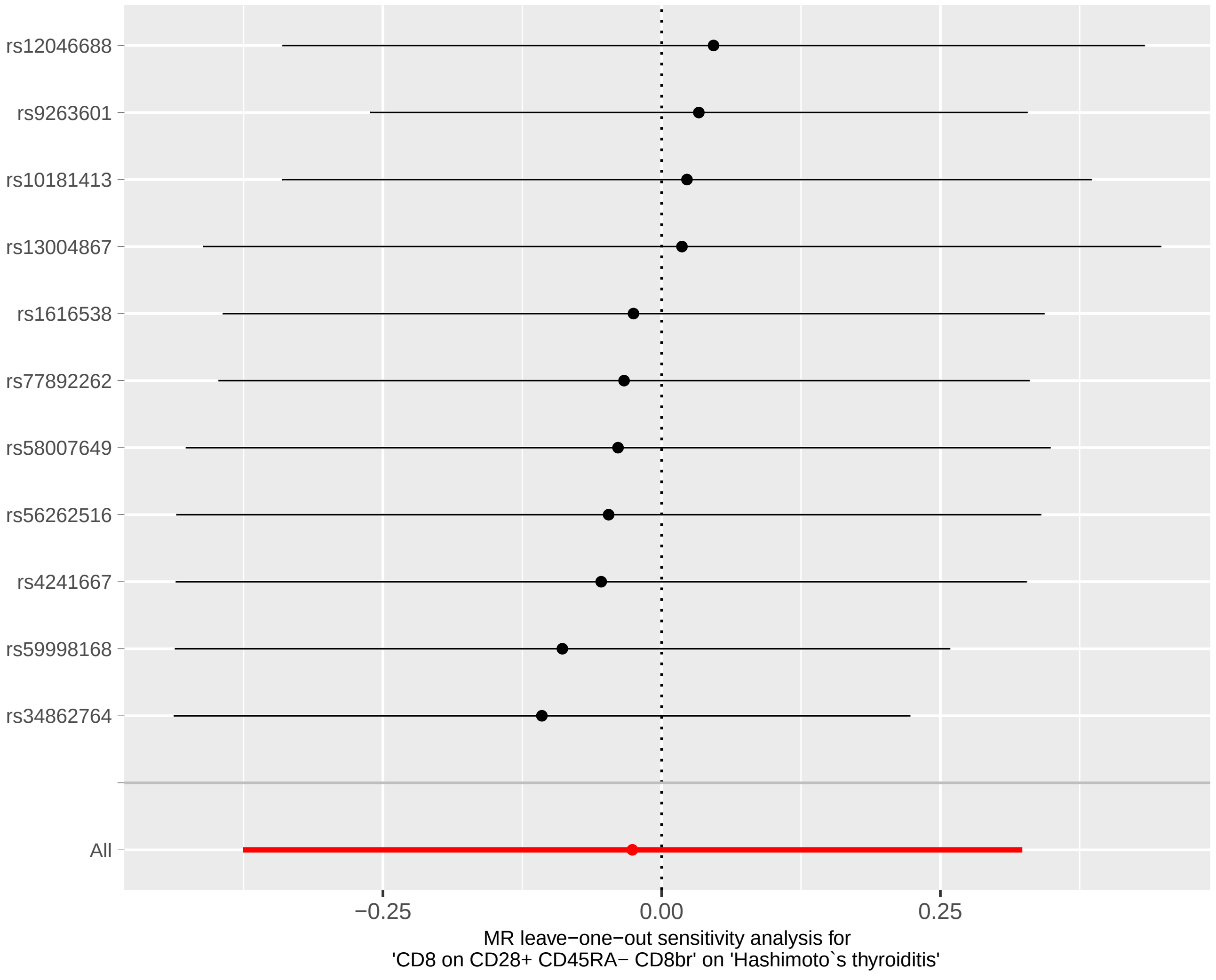
rs12046688
rs9263601
rs10181413
rs13004867
rs1616538
rs77892262
rs58007649
rs56262516
rs4241667
rs59998168
rs34862764
All
−0.25
0.00
0.25
MR leave−one−out sensitivity analysis for
'CD8 on CD28+ CD45RA− CD8br' on 'Hashimoto`s thyroiditis'

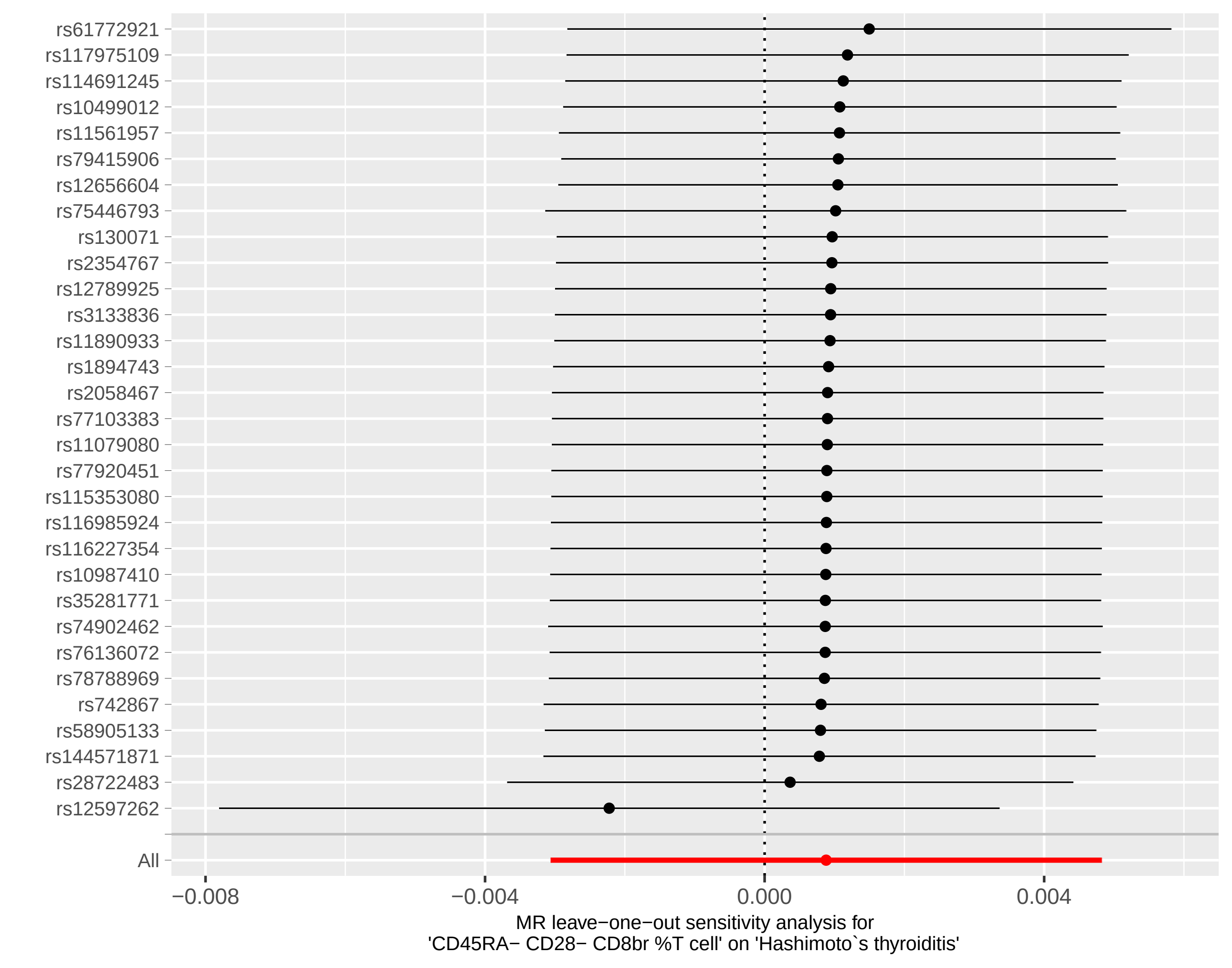
rs61772921
rs117975109
rs114691245
rs10499012
rs11561957
rs79415906
rs12656604
rs75446793
rs130071
rs2354767
rs12789925
rs3133836
rs11890933
rs1894743
rs2058467
rs77103383
rs11079080
rs77920451
rs115353080
rs116985924
rs116227354
rs10987410
rs35281771
rs74902462
rs76136072
rs78788969
rs742867
rs58905133
rs144571871
rs28722483
rs12597262
All
−0.008
−0.004
0.000
0.004
MR leave−one−out sensitivity analysis for
'CD45RA− CD28− CD8br %T cell' on 'Hashimoto`s thyroiditis'

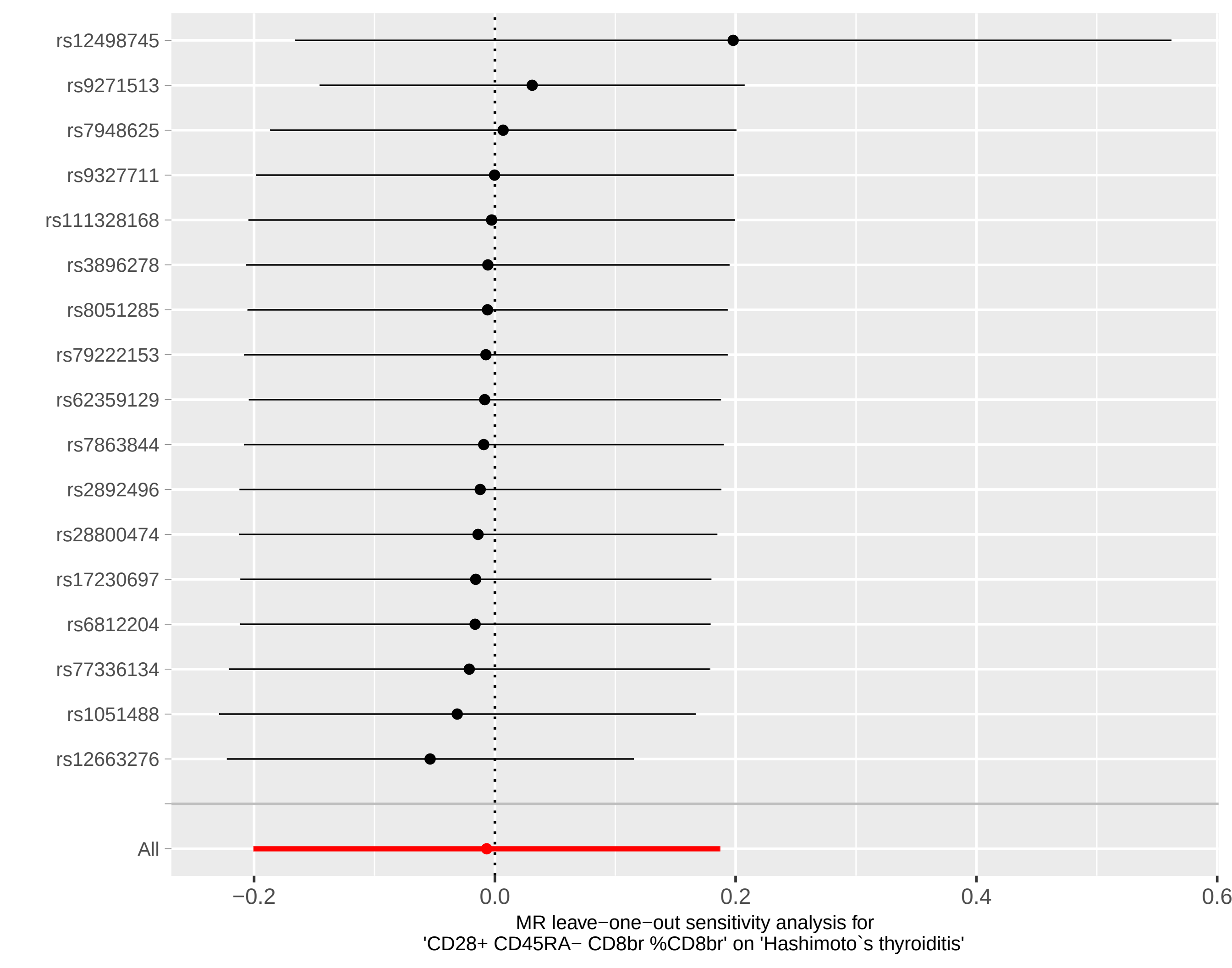

rs12498745
rs9271513
rs7948625
rs9327711
rs111328168
rs3896278
rs8051285
rs79222153
rs62359129
rs7863844
rs2892496
rs28800474
rs17230697
rs6812204
rs77336134
rs1051488
rs12663276
All
−0.2
0.0
0.2
0.4
0.6
MR leave−one−out sensitivity analysis for
'CD28+ CD45RA− CD8br %CD8br' on 'Hashimoto`s thyroiditis'

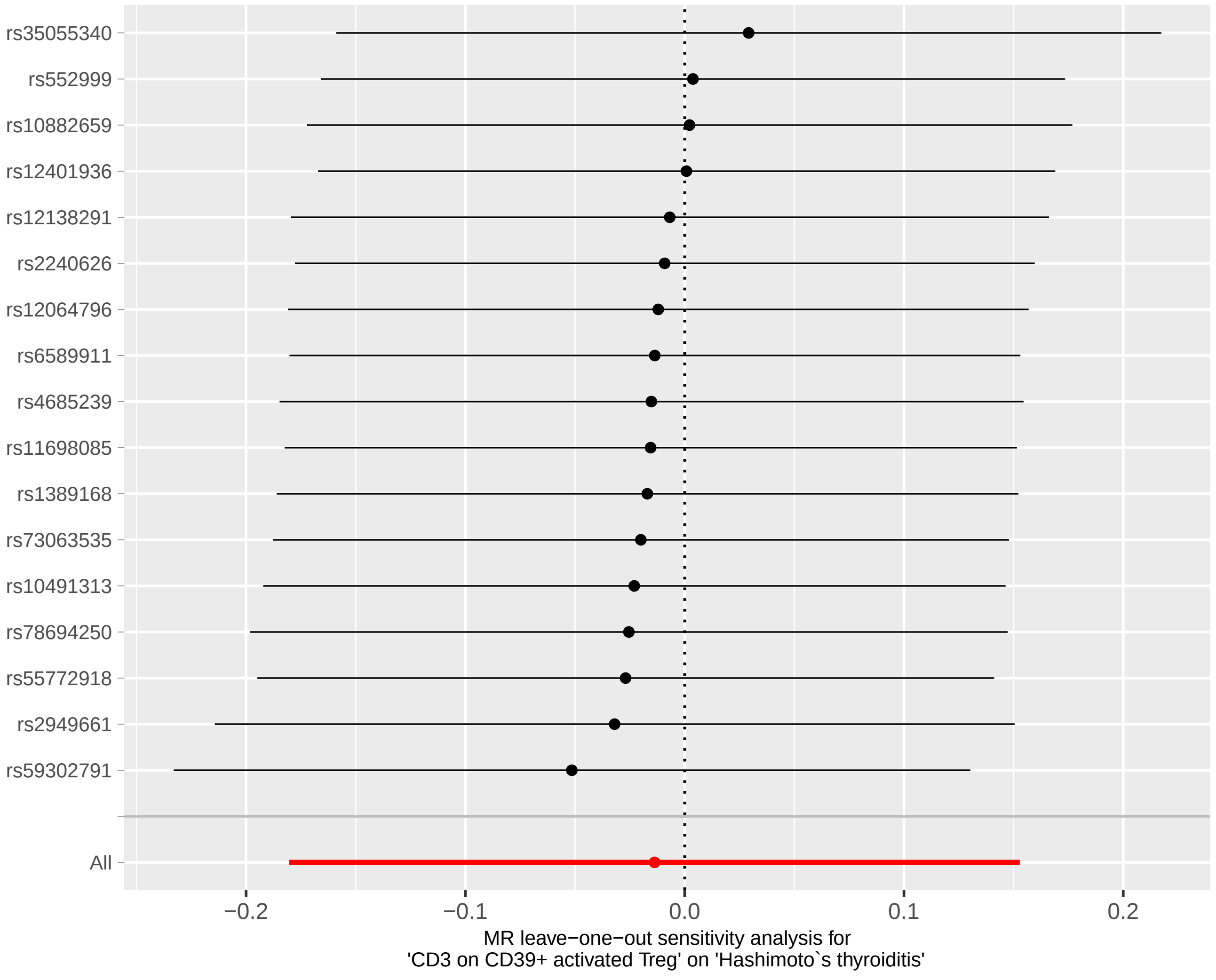
rs35055340
rs552999
rs10882659
rs12401936
rs12138291
rs2240626
rs12064796
rs6589911
rs4685239
rs11698085
rs1389168
rs73063535
rs10491313
rs78694250
rs55772918
rs2949661
rs59302791
All
−0.2
−0.1
0.0
0.1
0.2
MR leave−one−out sensitivity analysis for
'CD3 on CD39+ activated Treg' on 'Hashimoto`s thyroiditis'

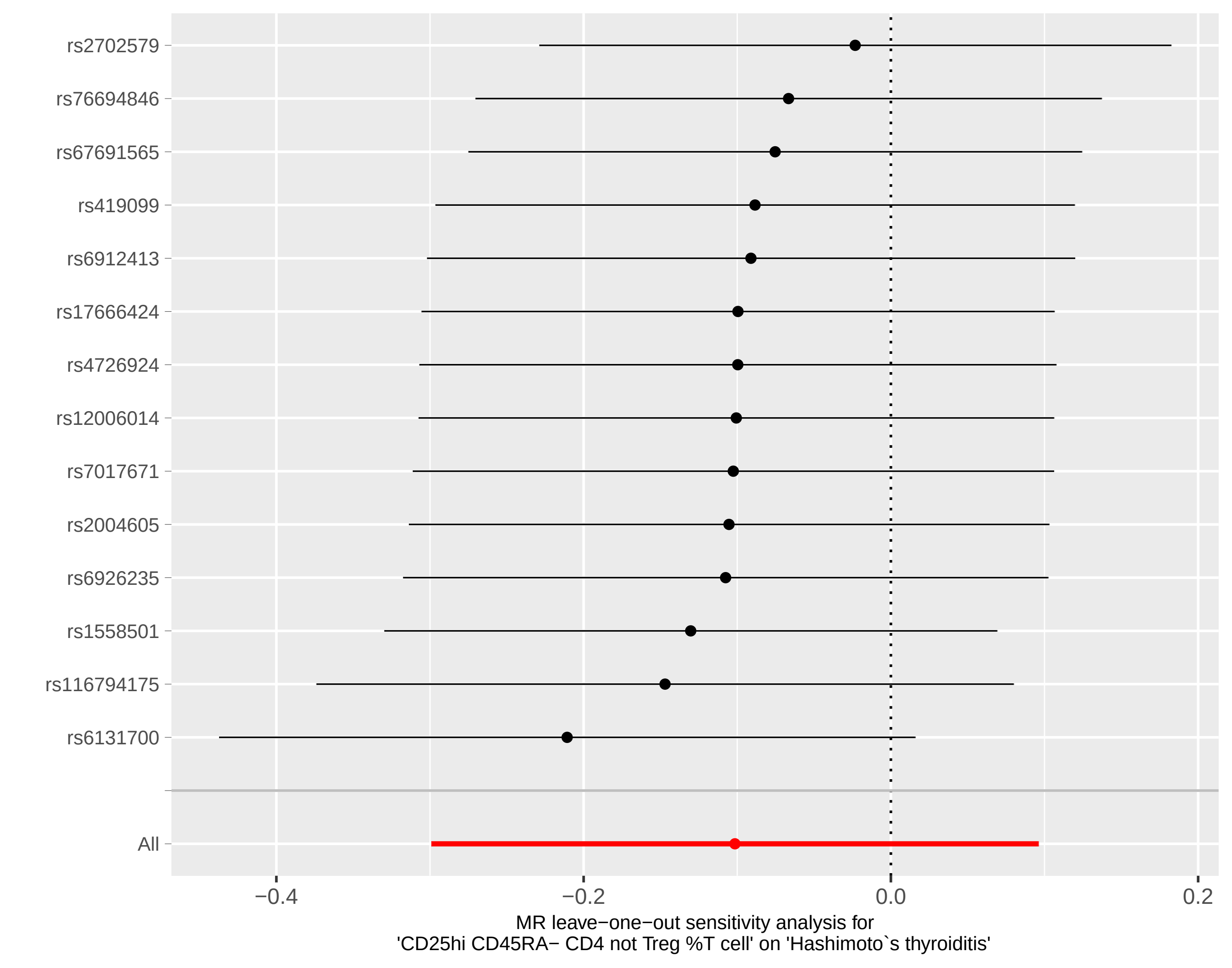
rs2702579
rs76694846
rs67691565
rs419099
rs6912413
rs17666424
rs4726924
rs12006014
rs7017671
rs2004605
rs6926235
rs1558501
rs116794175
rs6131700
All
−0.4
−0.2
0.0
0.2
MR leave−one−out sensitivity analysis for
'CD25hi CD45RA− CD4 not Treg %T cell' on 'Hashimoto`s thyroiditis'

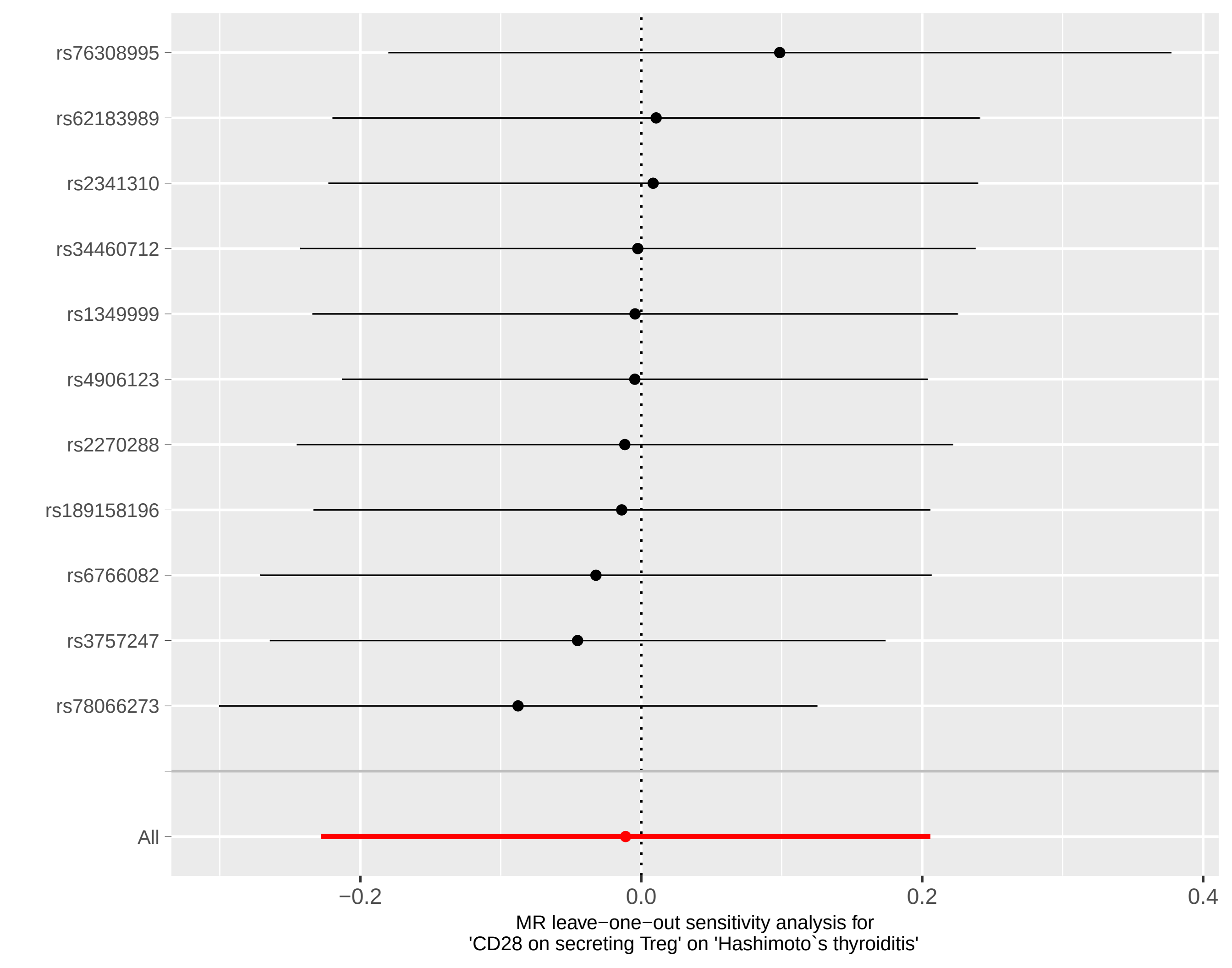
rs76308995
rs62183989
rs2341310
rs34460712
rs1349999
rs4906123
rs2270288
rs189158196
rs6766082
rs3757247
rs78066273
All
−0.2
0.0
0.2
0.4
MR leave−one−out sensitivity analysis for
'CD28 on secreting Treg' on 'Hashimoto`s thyroiditis'

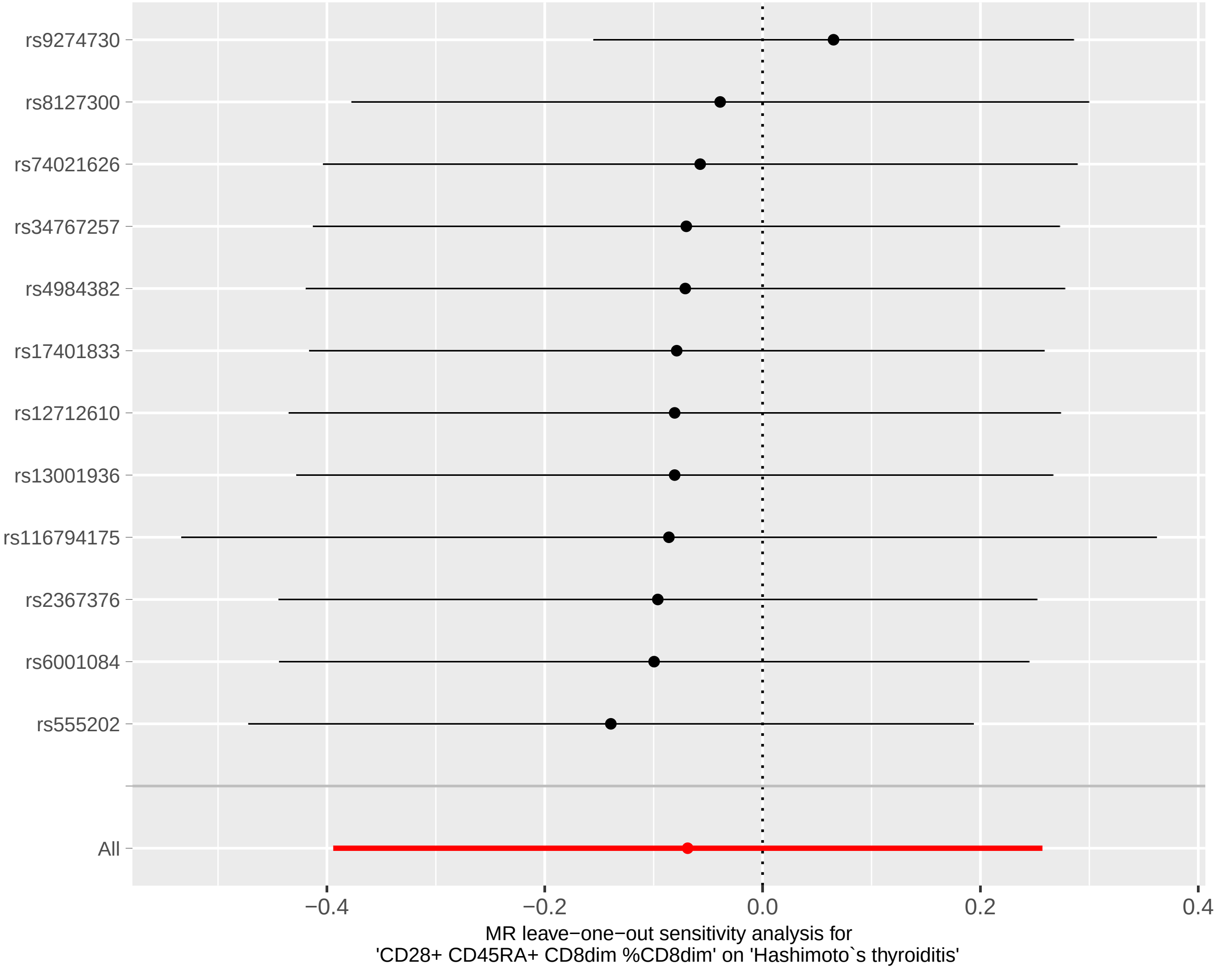

rs9274730
rs8127300
rs74021626
rs34767257
rs4984382
rs17401833
rs12712610
rs13001936
rs116794175
rs2367376
rs6001084
rs555202
All
−0.4
−0.2
0.0
0.2
0.4
MR leave−one−out sensitivity analysis for
'CD28+ CD45RA+ CD8dim %CD8dim' on 'Hashimoto`s thyroiditis'

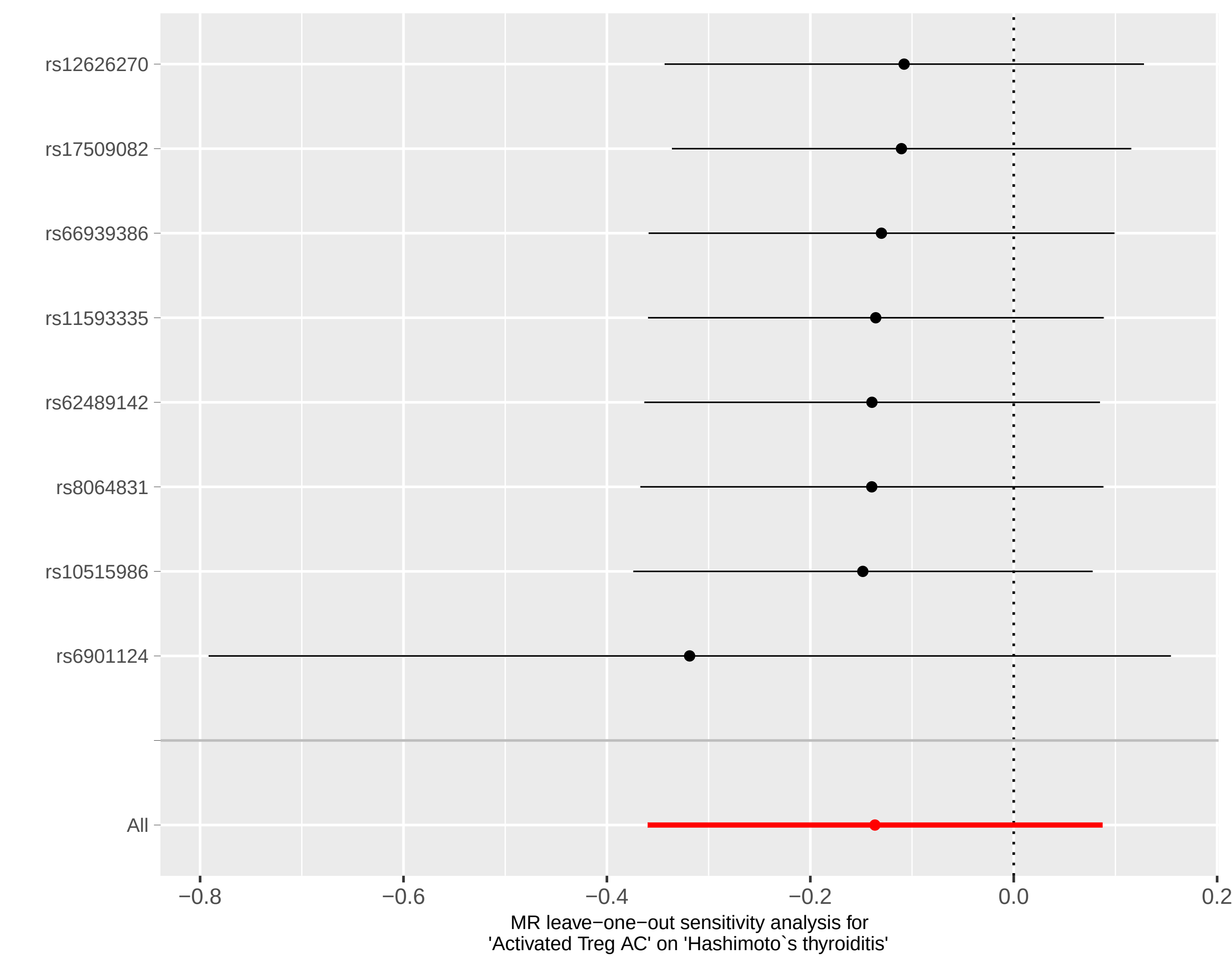

rs12626270
rs17509082
rs66939386
rs11593335
rs62489142
rs8064831
rs10515986
rs6901124
All
−0.8
−0.6
−0.4
−0.2
0.0
0.2
MR leave−one−out sensitivity analysis for
'Activated Treg AC' on 'Hashimoto`s thyroiditis'

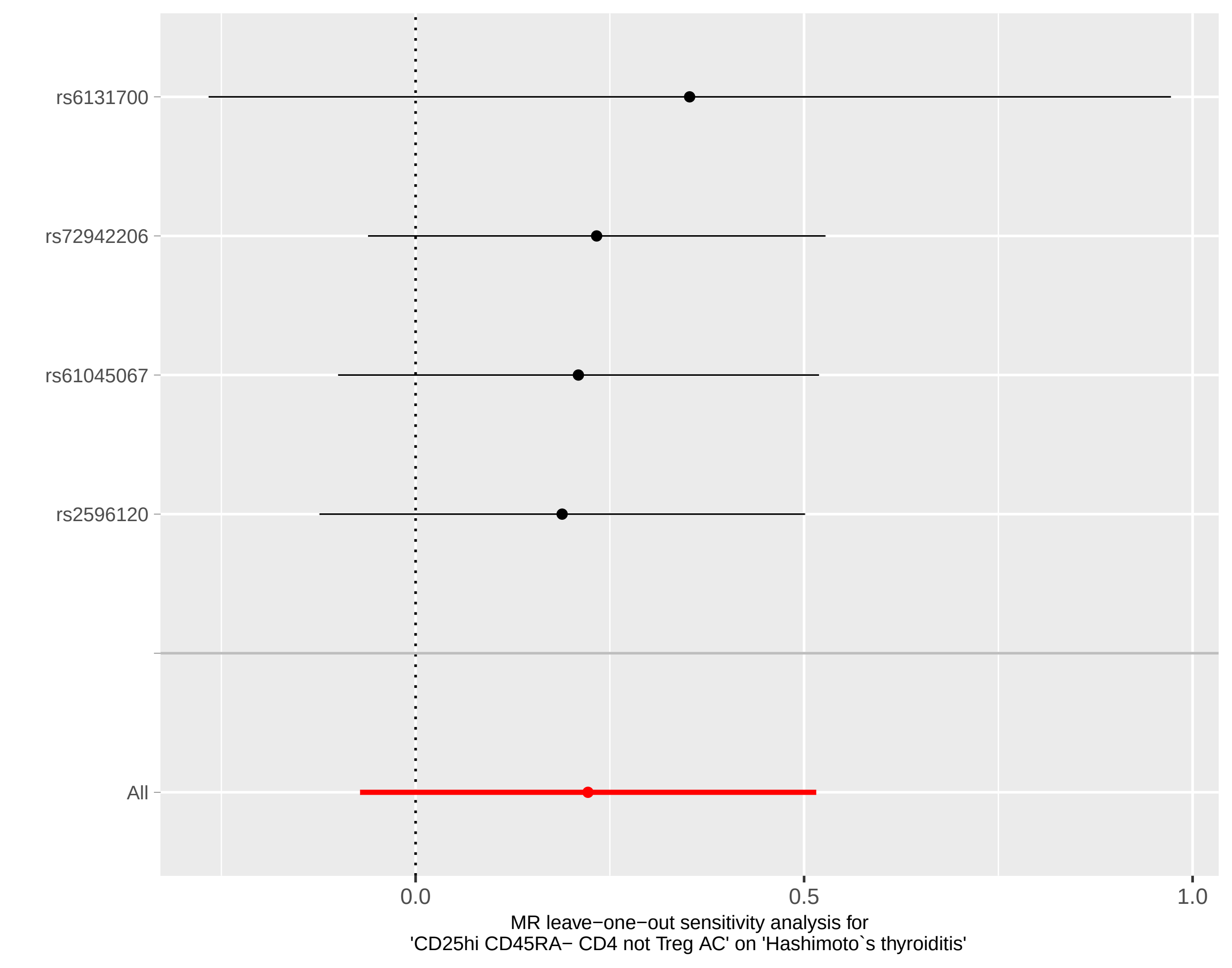
rs6131700
rs72942206
rs61045067
rs2596120
All
0.0
0.5
1.0
MR leave−one−out sensitivity analysis for
'CD25hi CD45RA− CD4 not Treg AC' on 'Hashimoto`s thyroiditis'

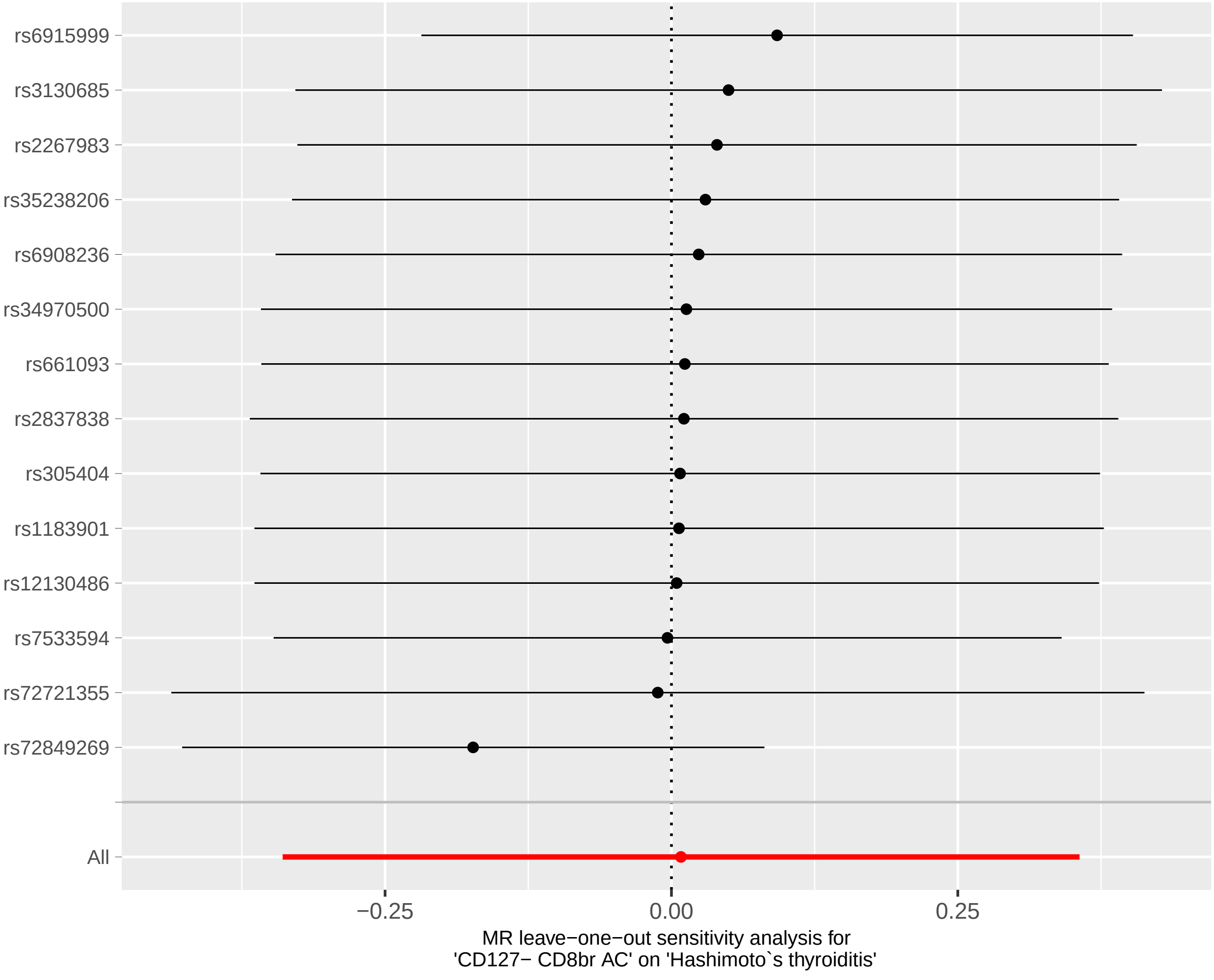
rs6915999
rs3130685
rs2267983
rs35238206
rs6908236
rs34970500
rs661093
rs2837838
rs305404
rs1183901
rs12130486
rs7533594
rs72721355
rs72849269
All
−0.25
0.00
0.25
MR leave−one−out sensitivity analysis for
'CD127− CD8br AC' on 'Hashimoto`s thyroiditis'

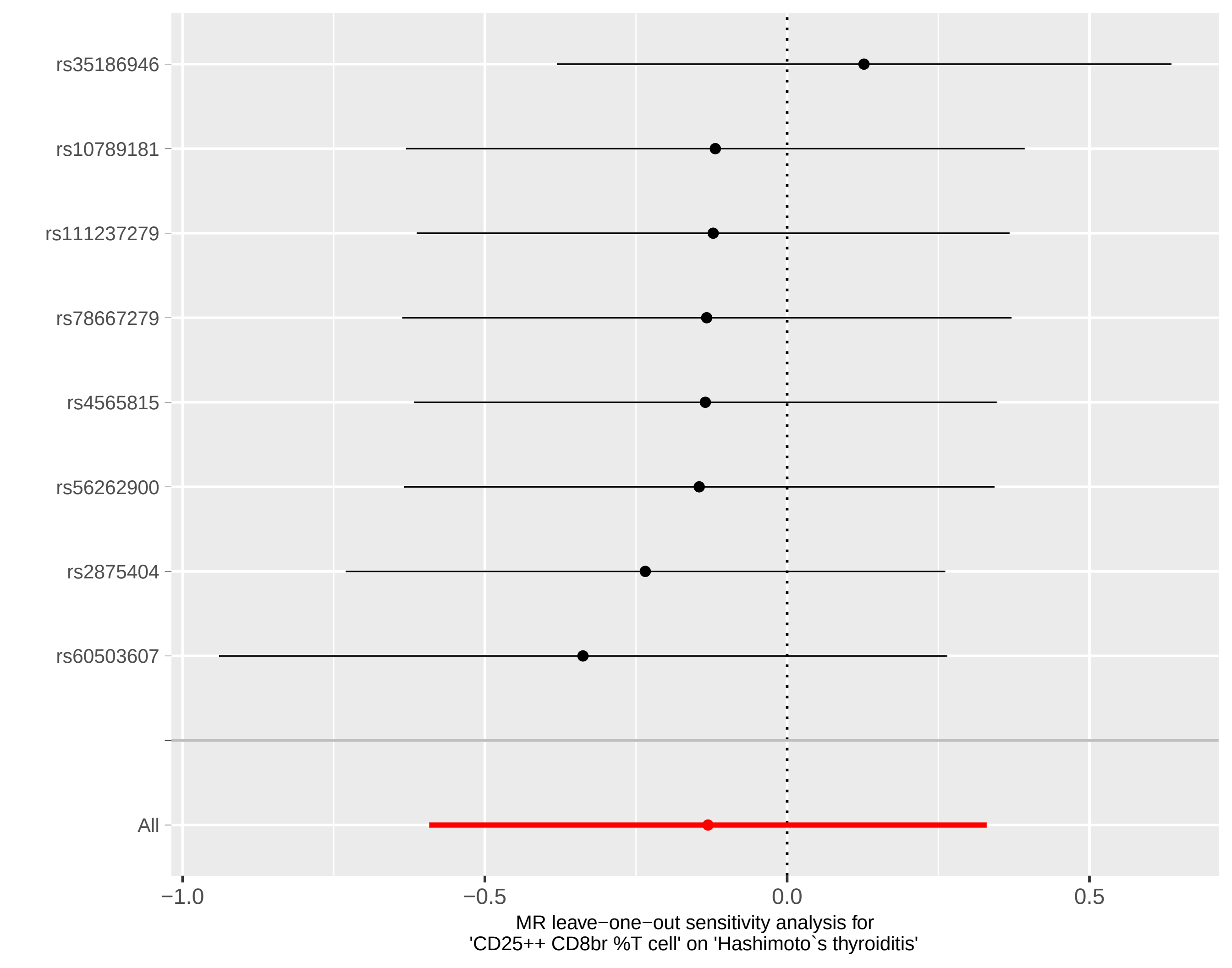
rs35186946
rs10789181
rs111237279
rs78667279
rs4565815
rs56262900
rs2875404
rs60503607
All
−1.0
−0.5
0.0
0.5
MR leave−one−out sensitivity analysis for
'CD25++ CD8br %T cell' on 'Hashimoto`s thyroiditis'

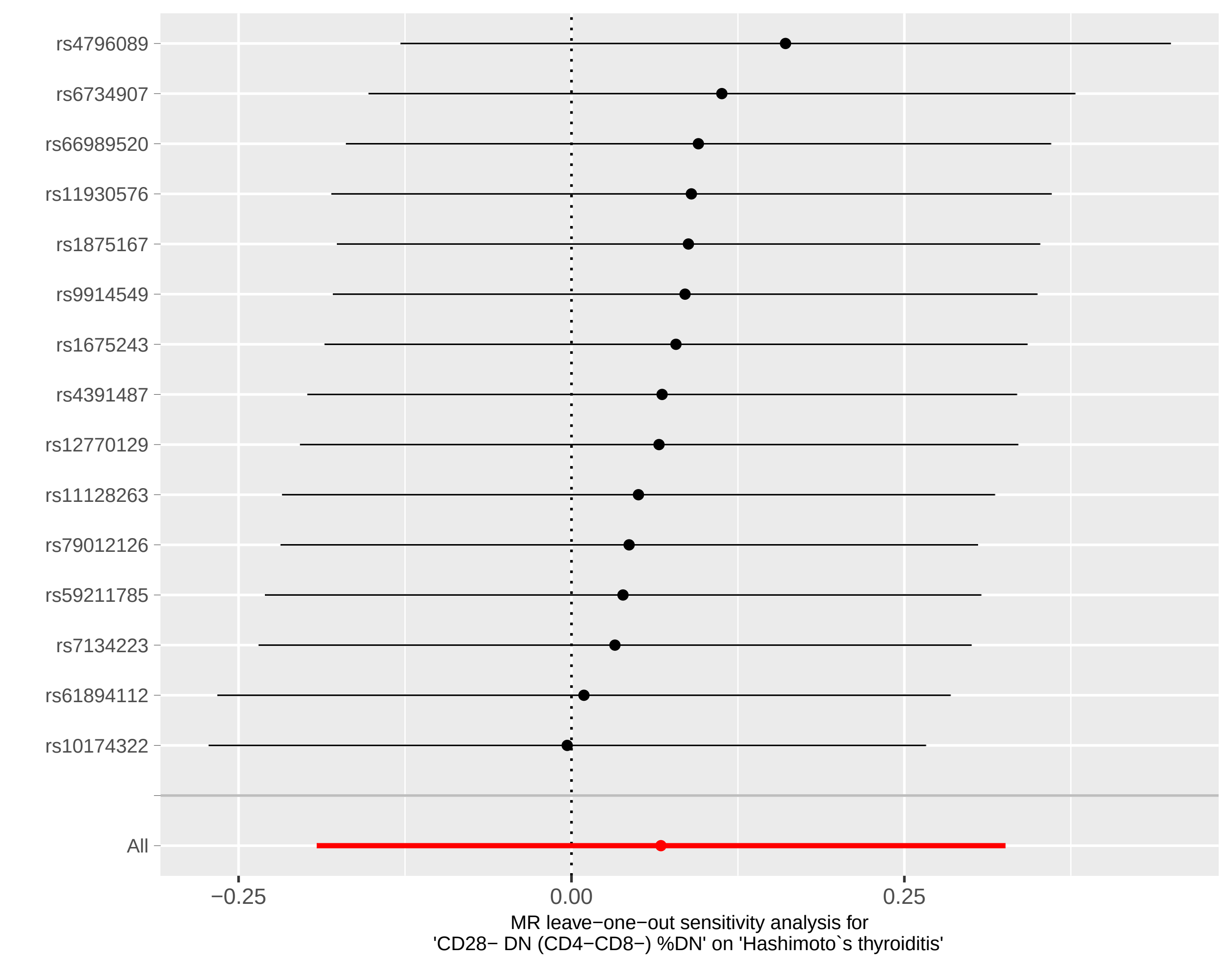

rs4796089
rs6734907
rs66989520
rs11930576
rs1875167
rs9914549
rs1675243
rs4391487
rs12770129
rs11128263
rs79012126
rs59211785
rs7134223
rs61894112
rs10174322
All
−0.25
0.00
0.25
MR leave−one−out sensitivity analysis for
'CD28− DN (CD4−CD8−) %DN' on 'Hashimoto`s thyroiditis'

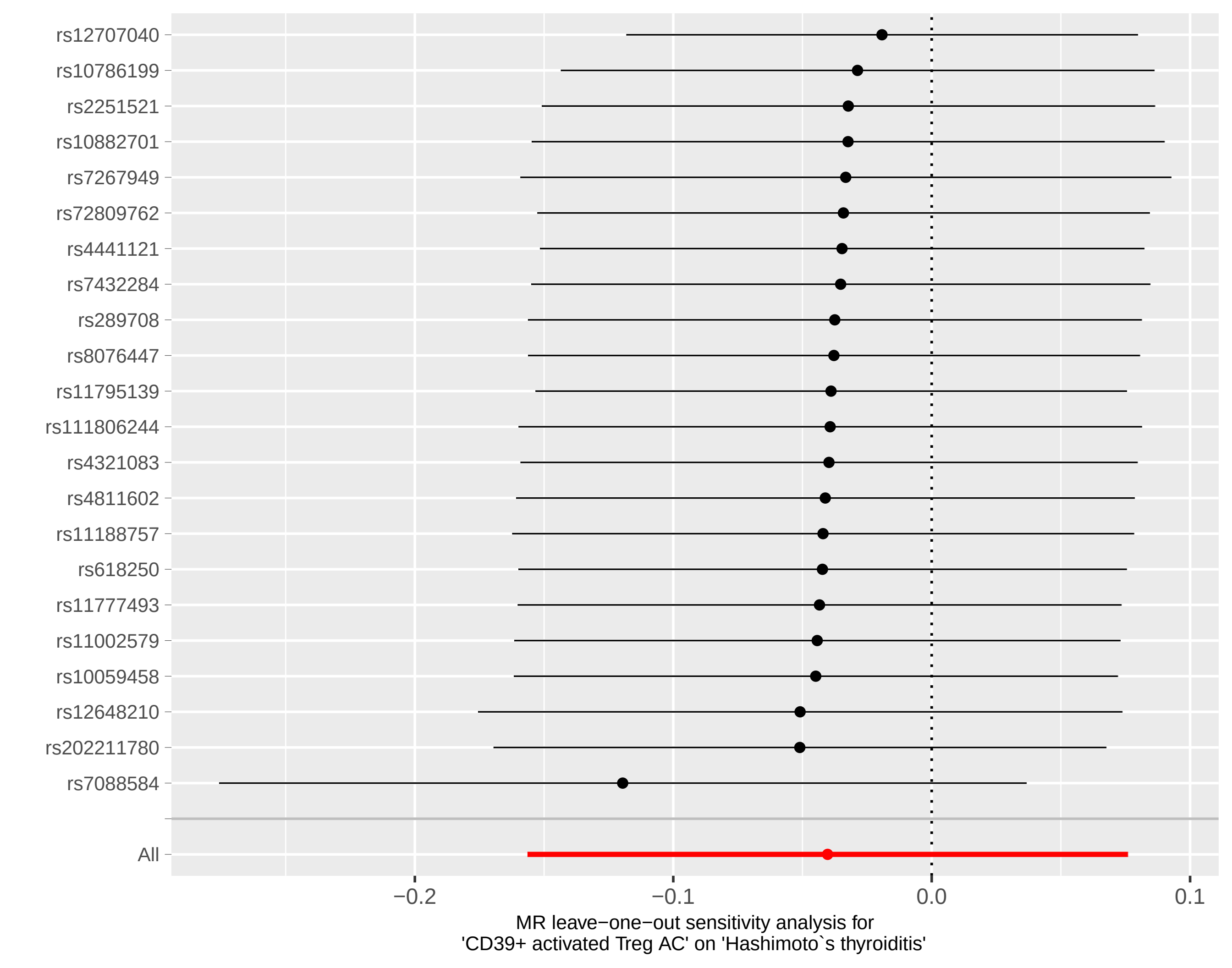

rs12707040
rs10786199
rs2251521
rs10882701
rs7267949
rs72809762
rs4441121
rs7432284
rs289708
rs8076447
rs11795139
rs111806244
rs4321083
rs4811602
rs11188757
rs618250
rs11777493
rs11002579
rs10059458
rs12648210
rs202211780
rs7088584
All
−0.2
−0.1
0.0
0.1
MR leave−one−out sensitivity analysis for
'CD39+ activated Treg AC' on 'Hashimoto`s thyroiditis'

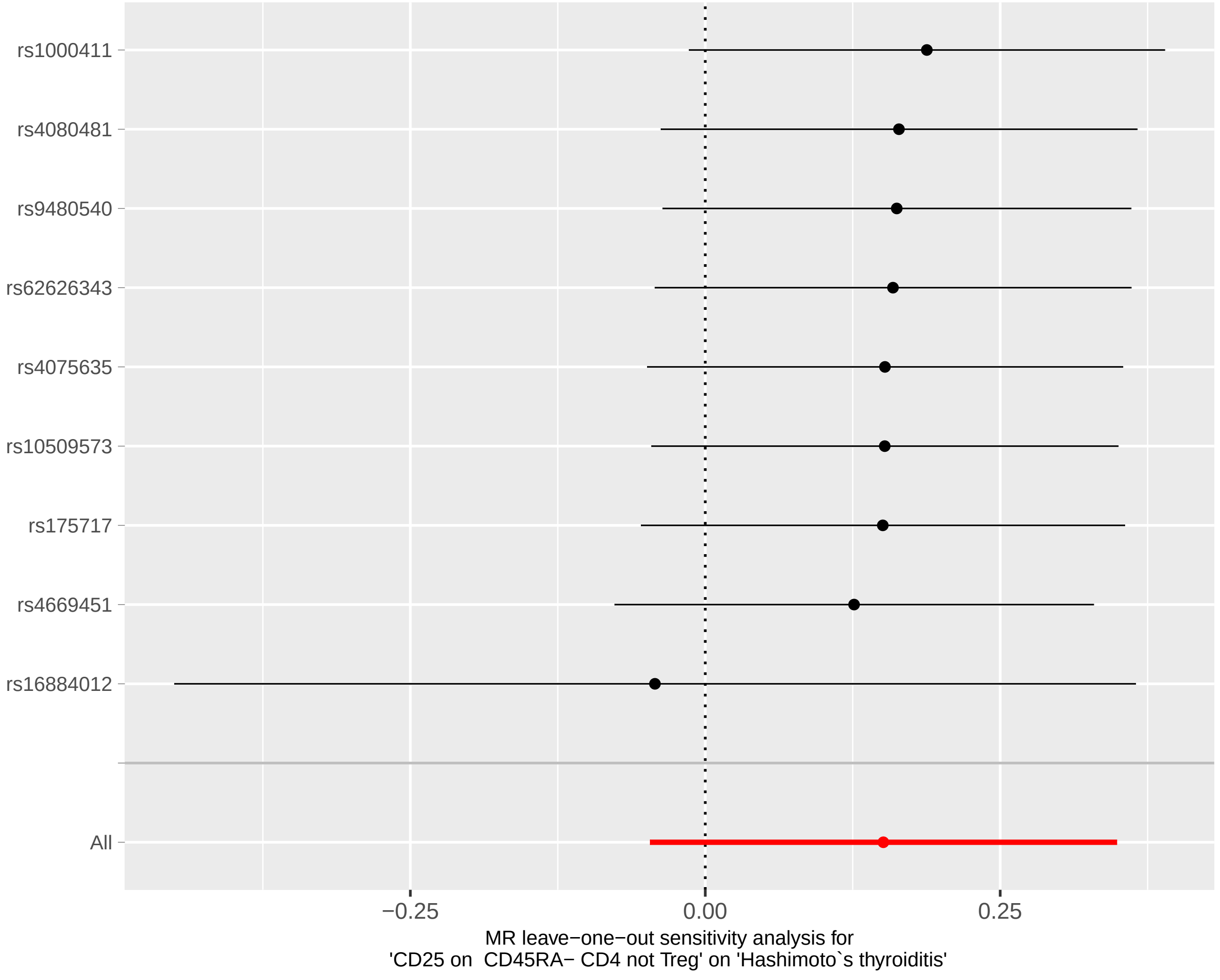
rs1000411
rs4080481
rs9480540
rs62626343
rs4075635
rs10509573
rs175717
rs4669451
rs16884012
All
−0.25
0.00
0.25
MR leave−one−out sensitivity analysis for
'CD25 on CD45RA− CD4 not Treg' on 'Hashimoto`s thyroiditis'

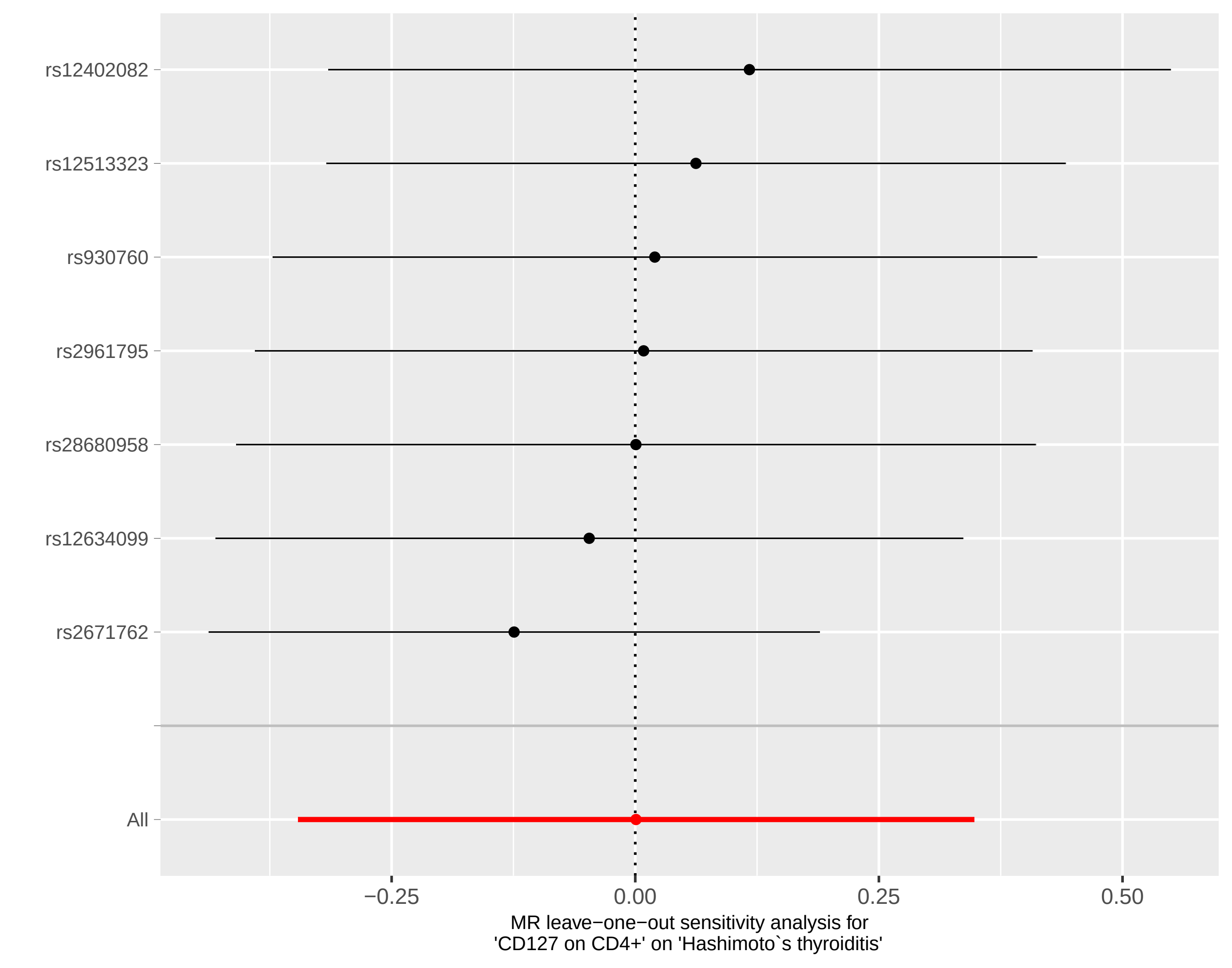
rs12402082
rs12513323
rs930760
rs2961795
rs28680958
rs12634099
rs2671762
All
−0.25
0.00
0.25
0.50
MR leave−one−out sensitivity analysis for
'CD127 on CD4+' on 'Hashimoto`s thyroiditis'

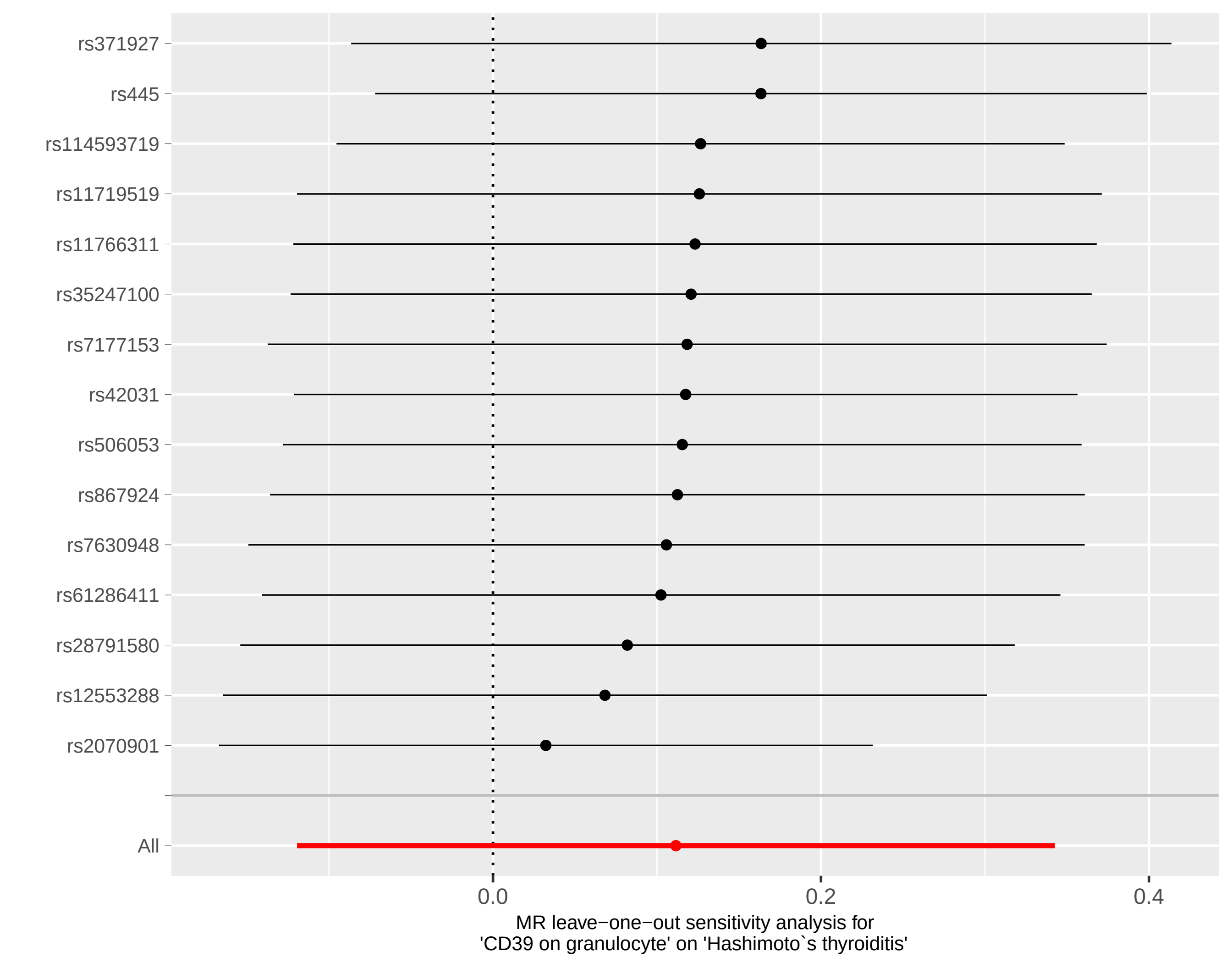
rs371927
rs445
rs114593719
rs11719519
rs11766311
rs35247100
rs7177153
rs42031
rs506053
rs867924
rs7630948
rs61286411
rs28791580
rs12553288
rs2070901
All
0.0
0.2
0.4
MR leave−one−out sensitivity analysis for
'CD39 on granulocyte' on 'Hashimoto`s thyroiditis'

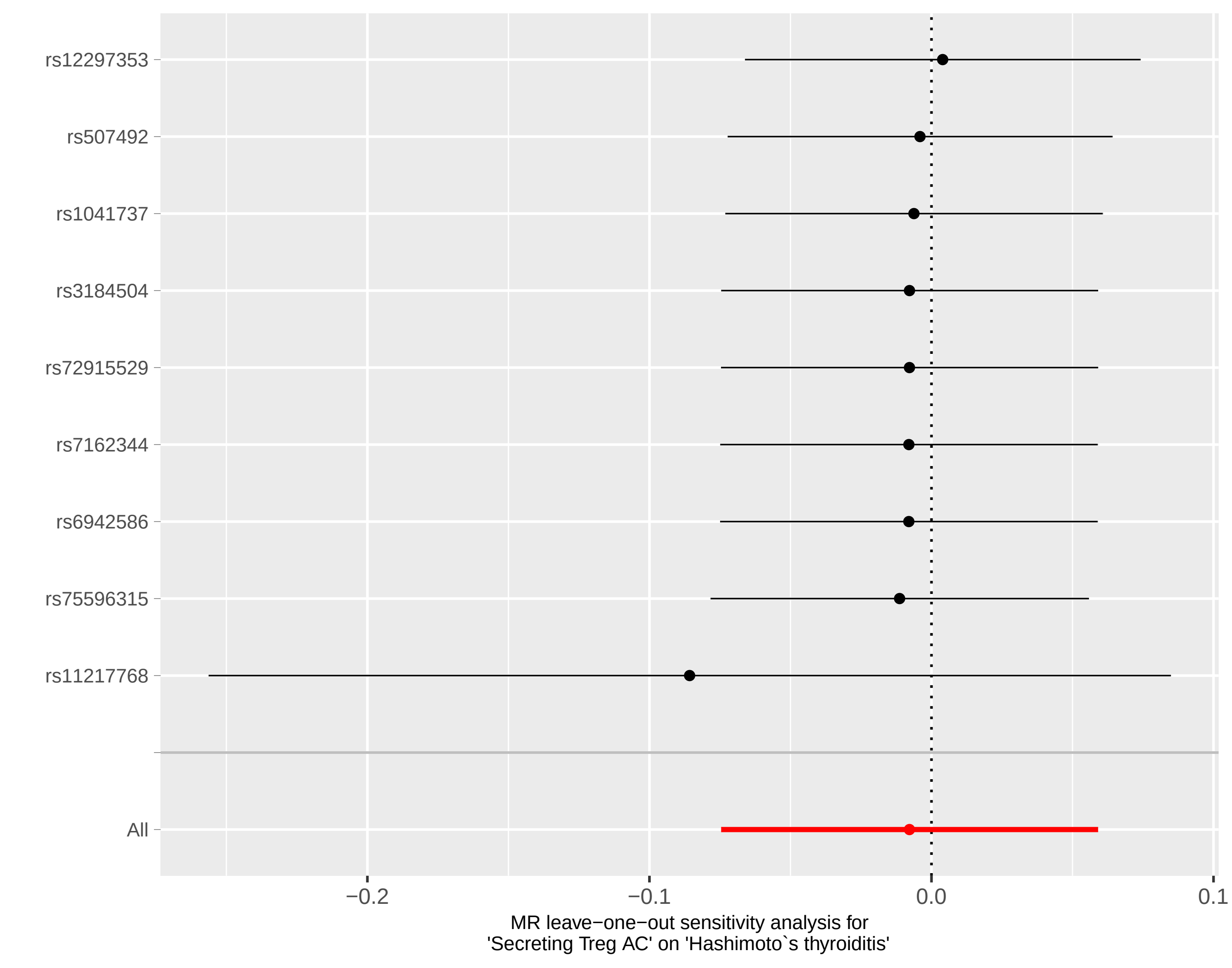
rs12297353
rs507492
rs1041737
rs3184504
rs72915529
rs7162344
rs6942586
rs75596315
rs11217768
All
−0.2
−0.1
0.0
0.1
MR leave−one−out sensitivity analysis for
'Secreting Treg AC' on 'Hashimoto`s thyroiditis'

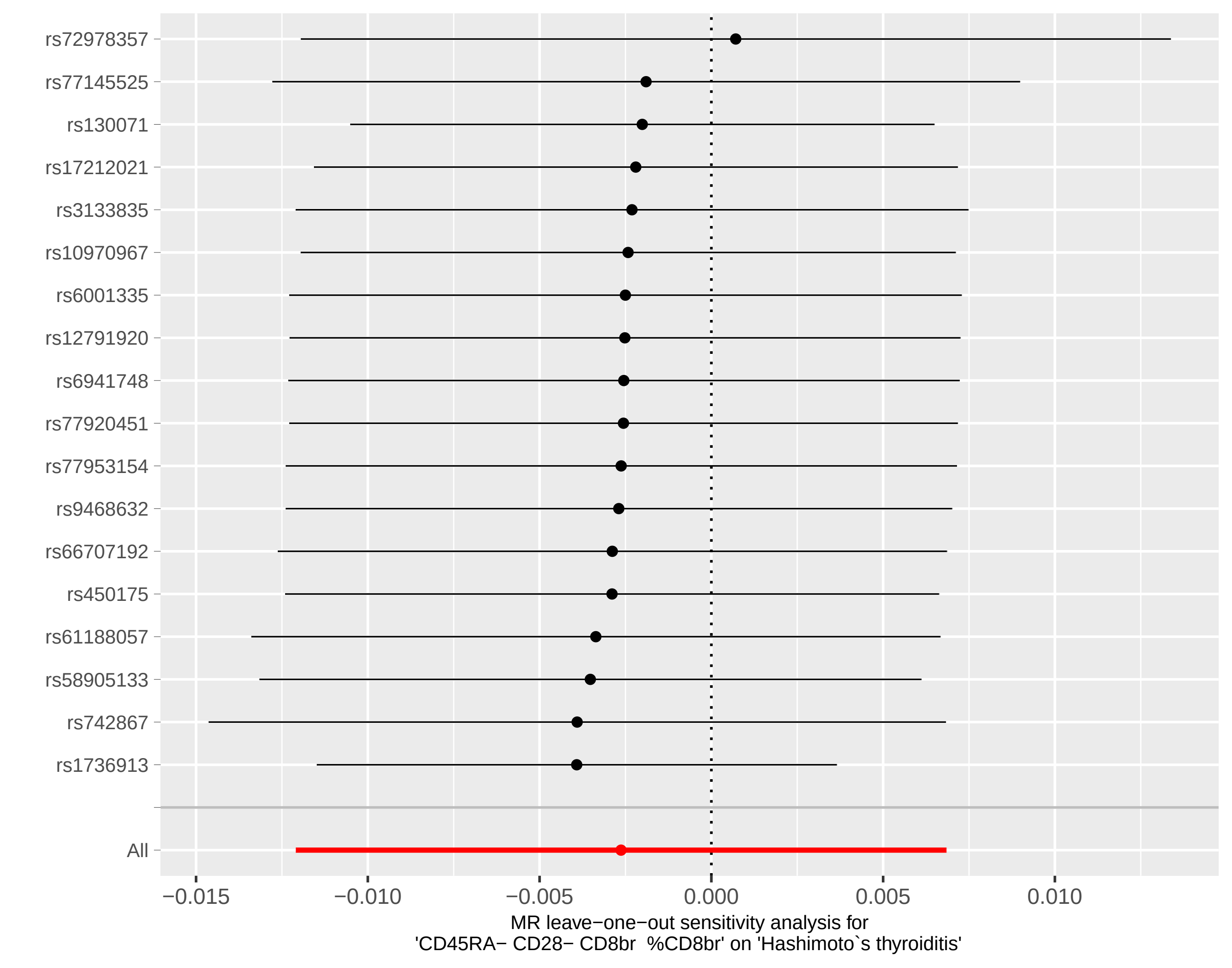

rs72978357
rs77145525
rs130071
rs17212021
rs3133835
rs10970967
rs6001335
rs12791920
rs6941748
rs77920451
rs77953154
rs9468632
rs66707192
rs450175
rs61188057
rs58905133
rs742867
rs1736913
All
−0.015
−0.010
−0.005
0.000
0.005
0.010
MR leave−one−out sensitivity analysis for
'CD45RA− CD28− CD8br %CD8br' on 'Hashimoto`s thyroiditis'

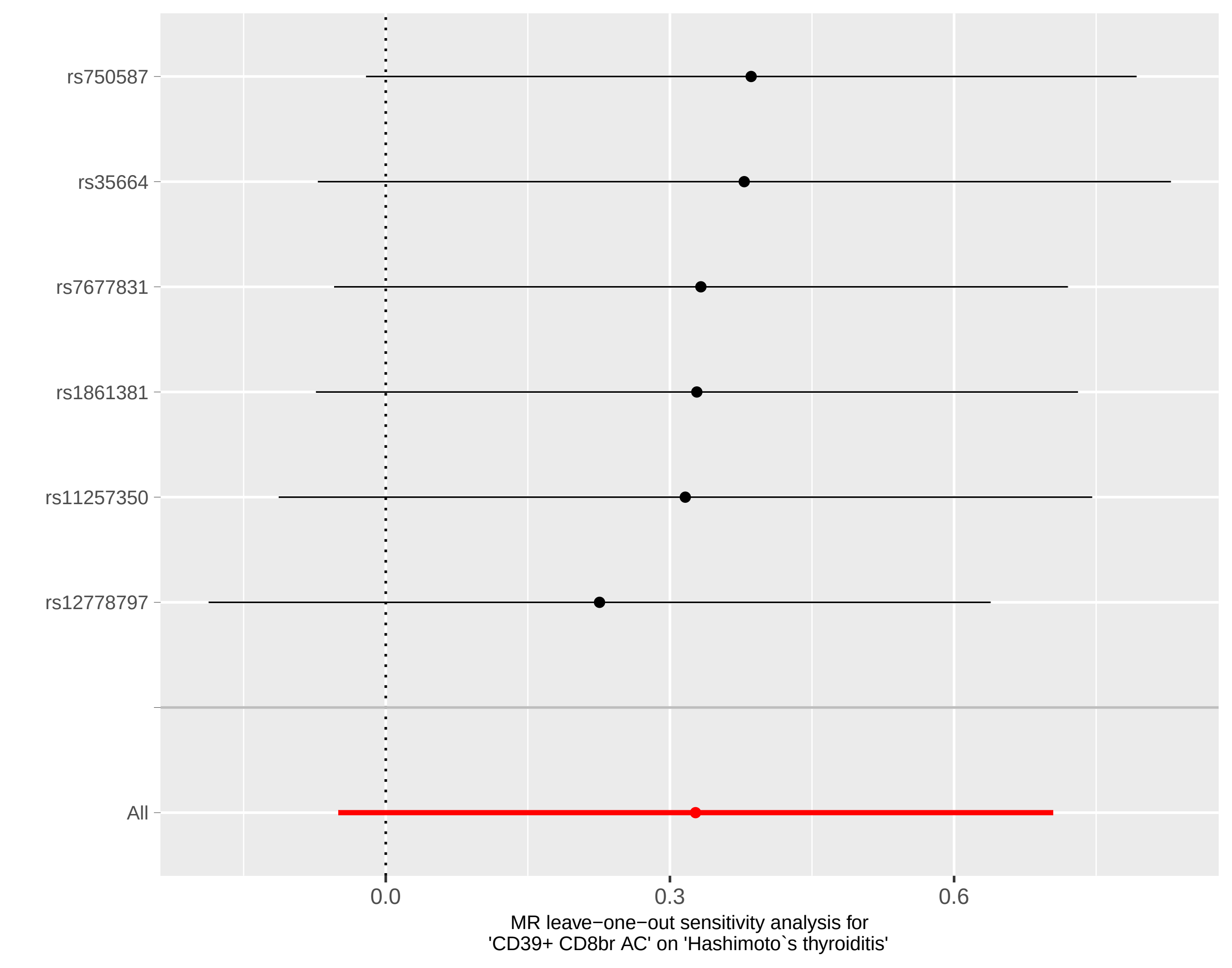

rs750587
rs35664
rs7677831
rs1861381
rs11257350
rs12778797
All
0.0
0.3
0.6
MR leave−one−out sensitivity analysis for
'CD39+ CD8br AC' on 'Hashimoto`s thyroiditis'

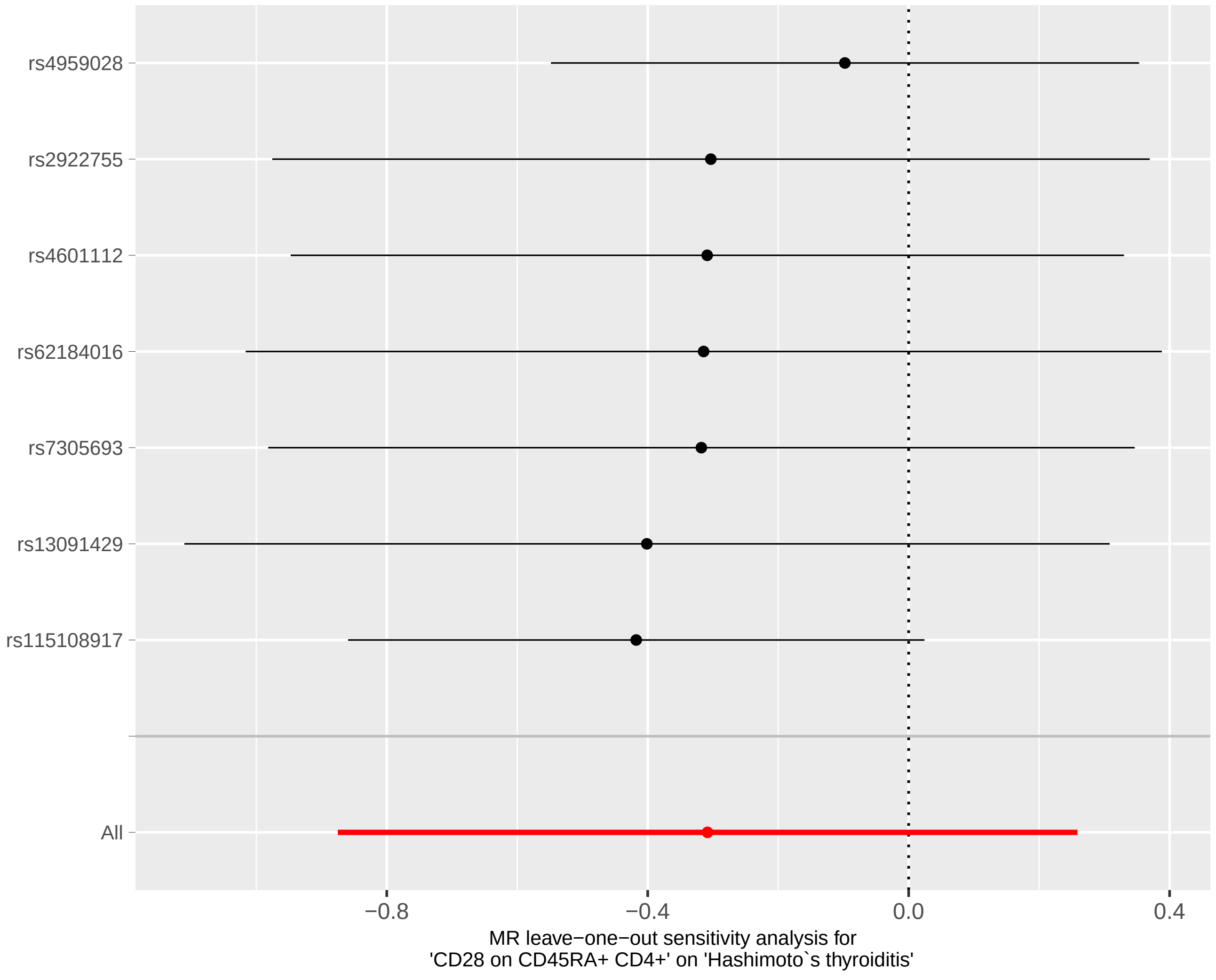
rs4959028
rs2922755
rs4601112
rs62184016
rs7305693
rs13091429
rs115108917
All
−0.8
−0.4
0.0
0.4
MR leave−one−out sensitivity analysis for
'CD28 on CD45RA+ CD4+' on 'Hashimoto`s thyroiditis'

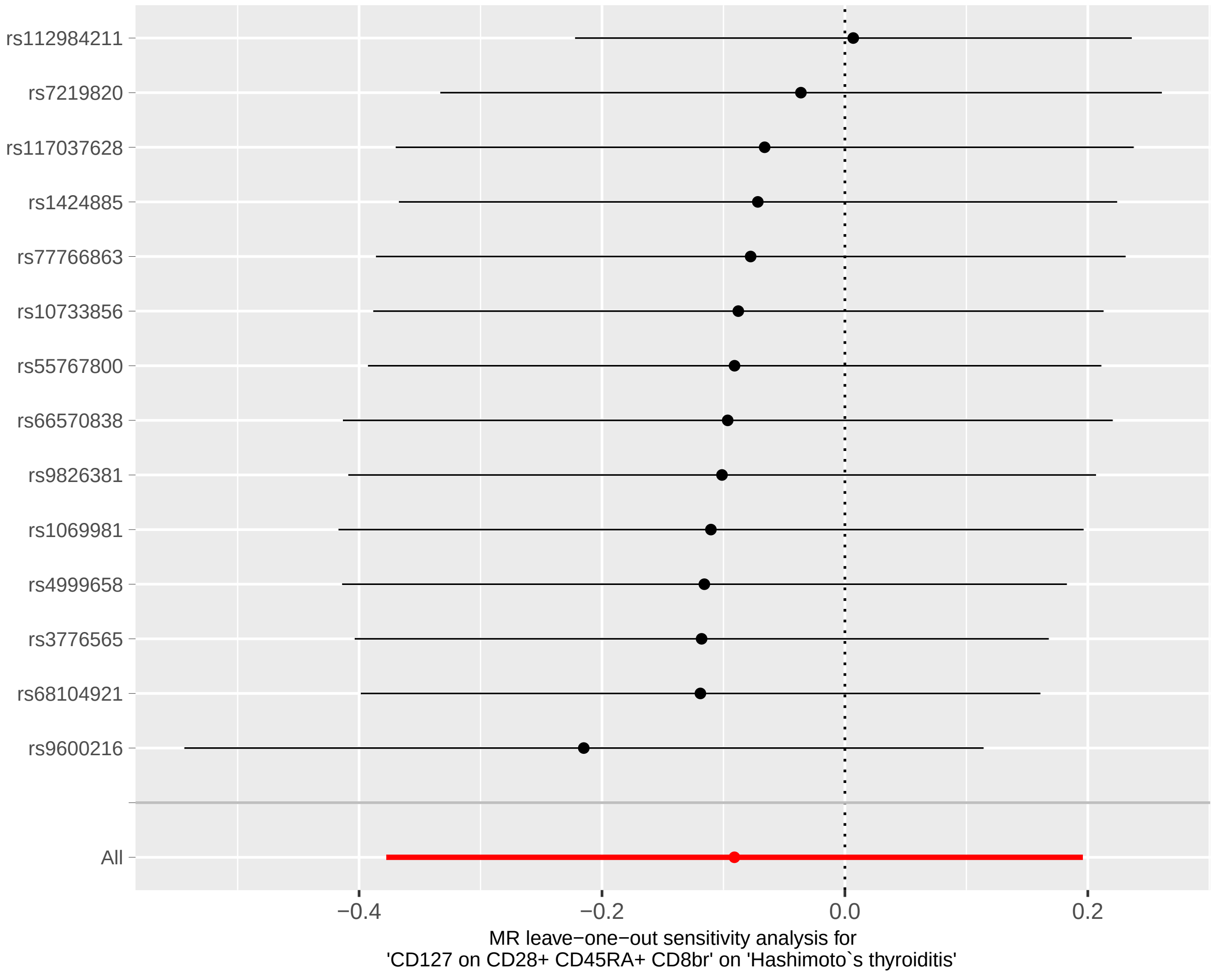
rs112984211
rs7219820
rs117037628
rs1424885
rs77766863
rs10733856
rs55767800
rs66570838
rs9826381
rs1069981
rs4999658
rs3776565
rs68104921
rs9600216
All
−0.4
−0.2
0.0
0.2
MR leave−one−out sensitivity analysis for
'CD127 on CD28+ CD45RA+ CD8br' on 'Hashimoto`s thyroiditis'

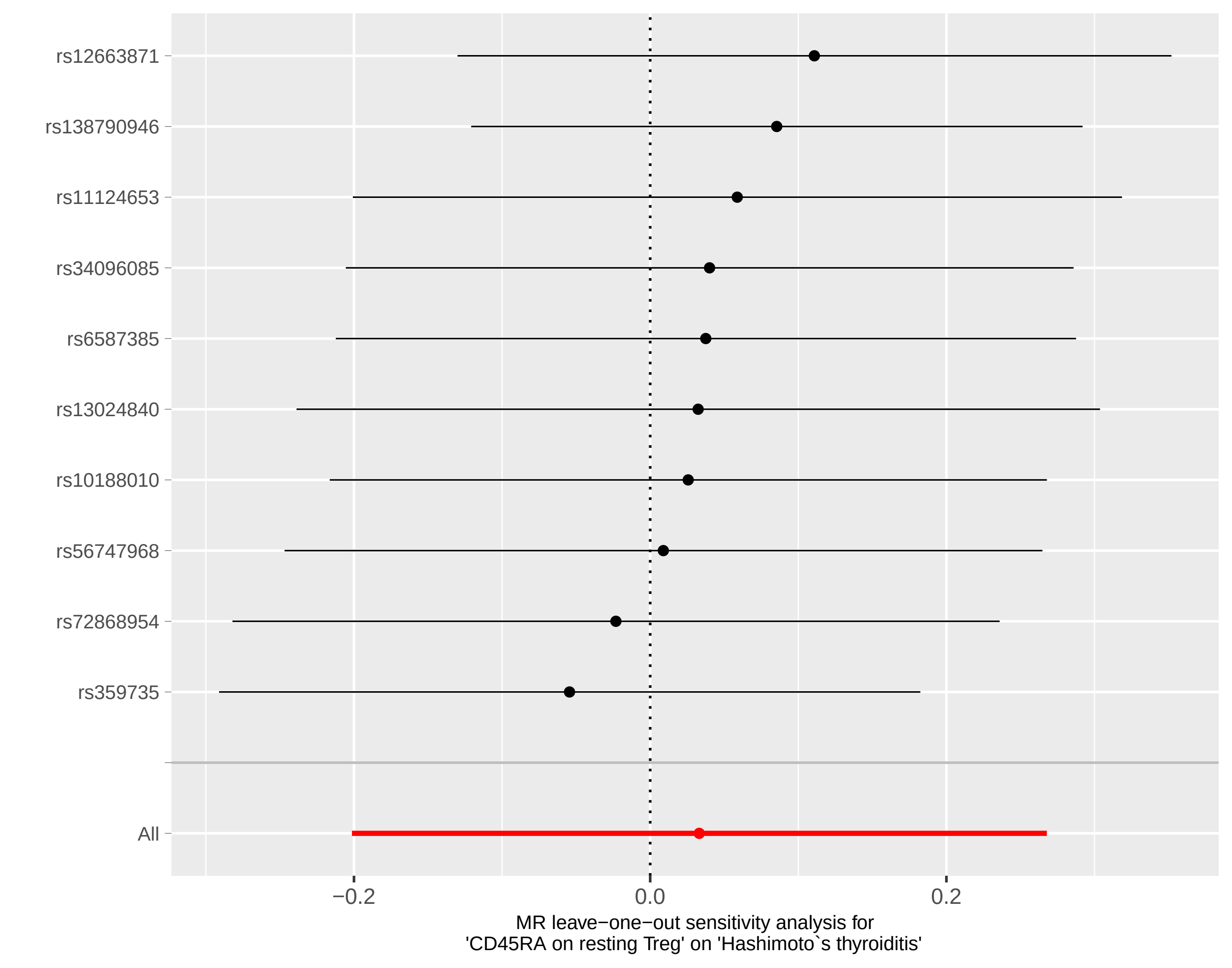
rs12663871
rs138790946
rs11124653
rs34096085
rs6587385
rs13024840
rs10188010
rs56747968
rs72868954
rs359735
All
−0.2
0.0
0.2
MR leave−one−out sensitivity analysis for
'CD45RA on resting Treg' on 'Hashimoto`s thyroiditis'

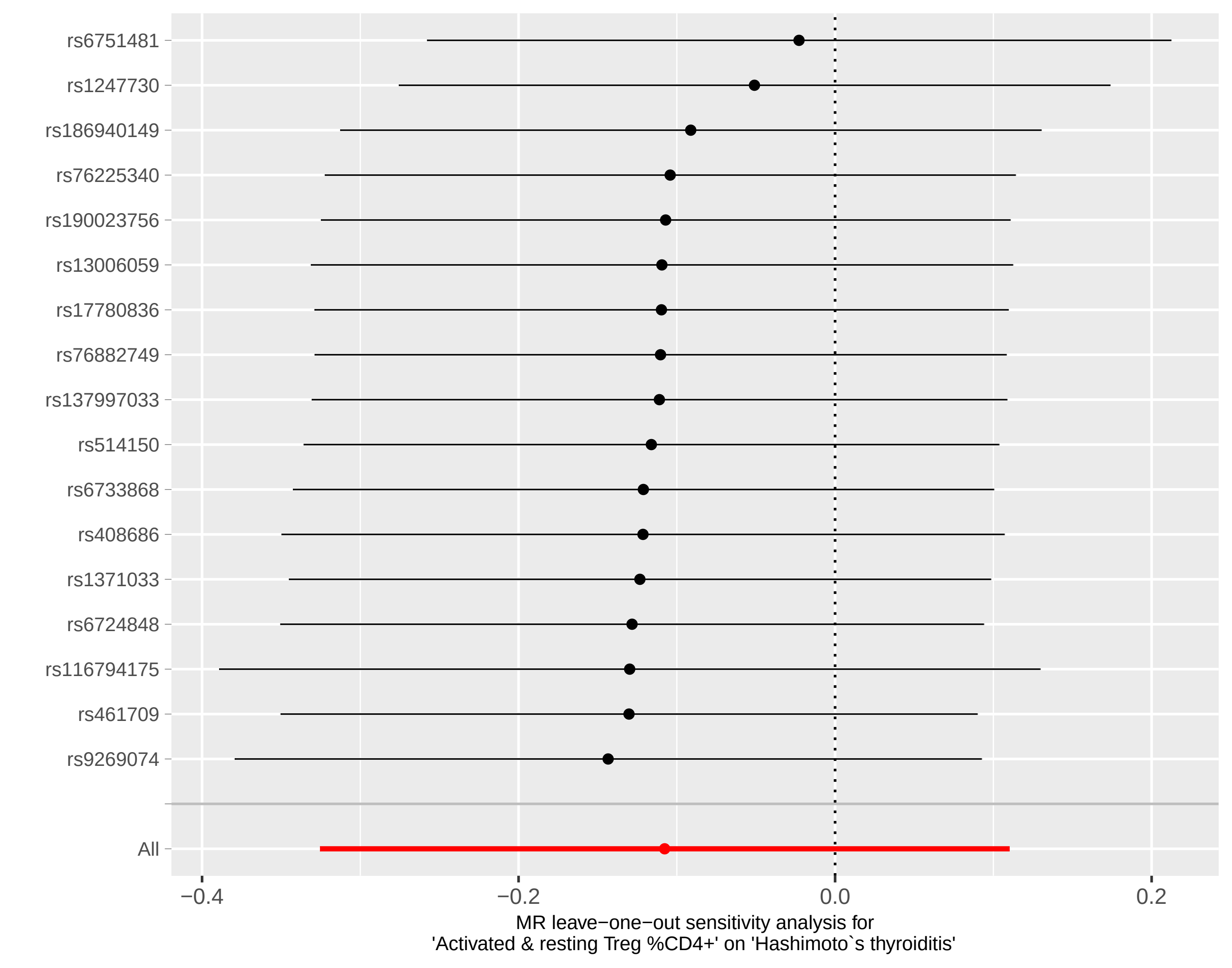
rs6751481
rs1247730
rs186940149
rs76225340
rs190023756
rs13006059
rs17780836
rs76882749
rs137997033
rs514150
rs6733868
rs408686
rs1371033
rs6724848
rs116794175
rs461709
rs9269074
All
−0.4
−0.2
0.0
0.2
MR leave−one−out sensitivity analysis for
'Activated & resting Treg %CD4+' on 'Hashimoto`s thyroiditis'

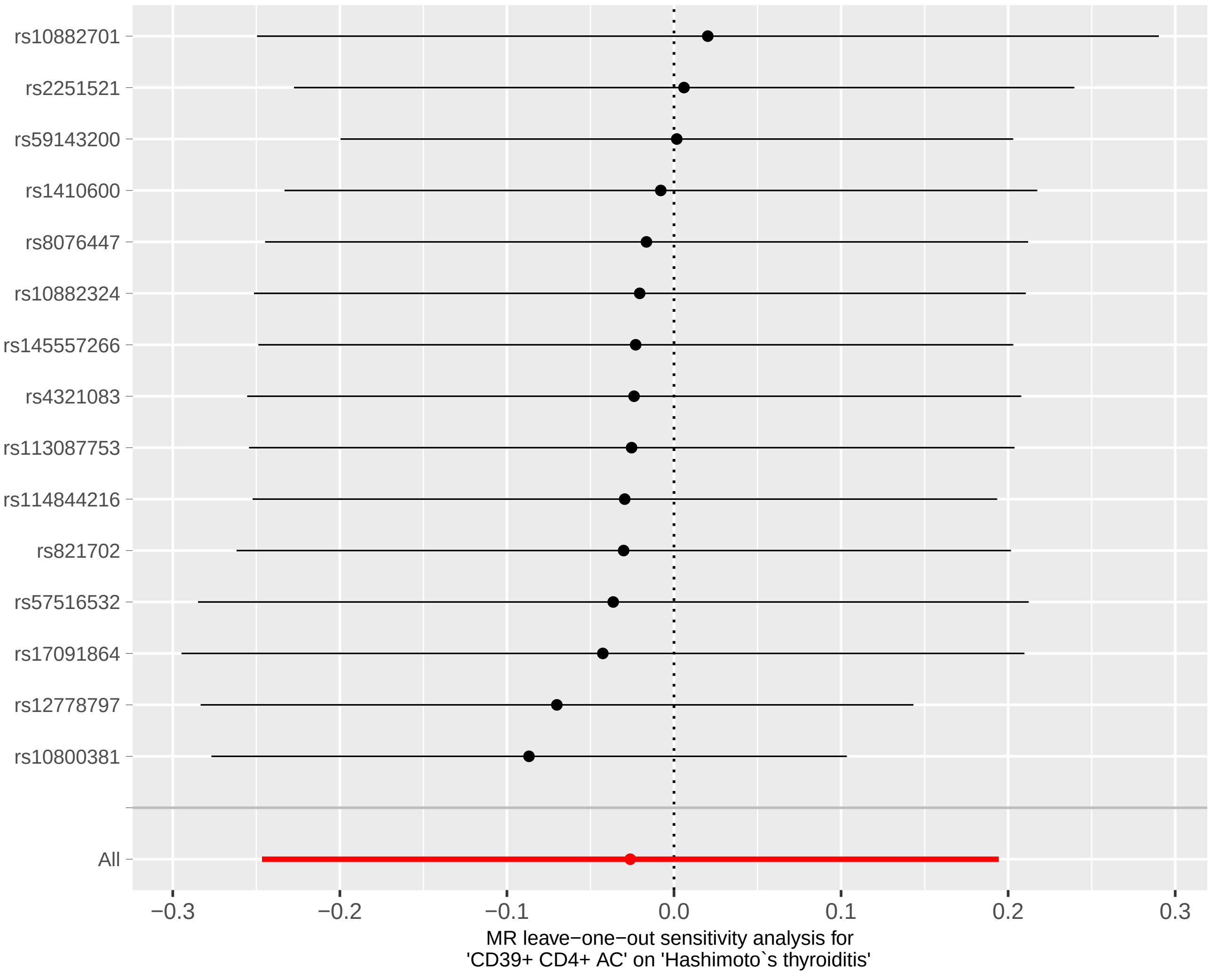

rs10882701
rs2251521
rs59143200
rs1410600
rs8076447
rs10882324
rs145557266
rs4321083
rs113087753
rs114844216
rs821702
rs57516532
rs17091864
rs12778797
rs10800381
All
−0.3
−0.2
−0.1
0.0
0.1
0.2
0.3
MR leave−one−out sensitivity analysis for
'CD39+ CD4+ AC' on 'Hashimoto`s thyroiditis'

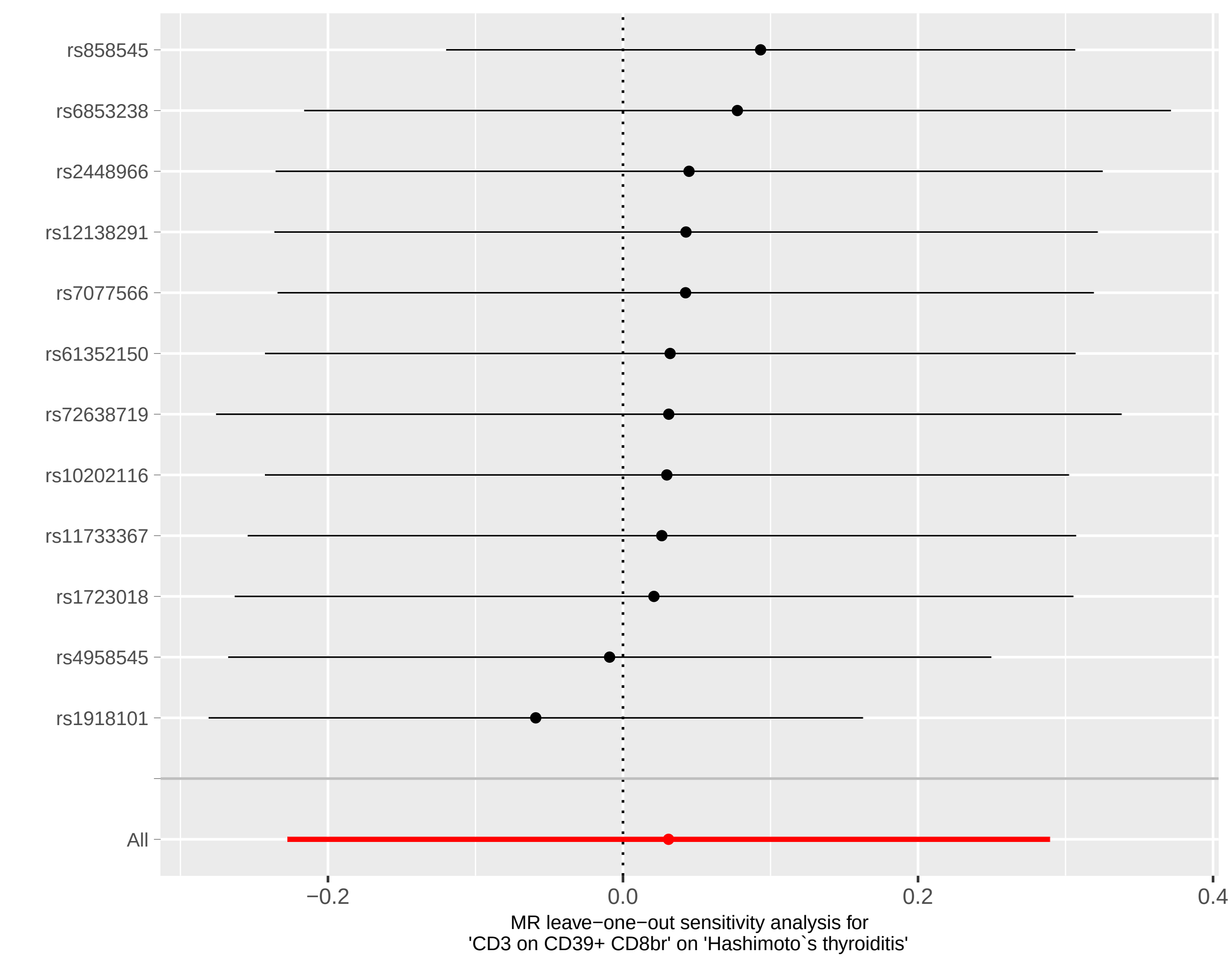
rs858545
rs6853238
rs2448966
rs12138291
rs7077566
rs61352150
rs72638719
rs10202116
rs11733367
rs1723018
rs4958545
rs1918101
All
−0.2
0.0
0.2
0.4
MR leave−one−out sensitivity analysis for
'CD3 on CD39+ CD8br' on 'Hashimoto`s thyroiditis'

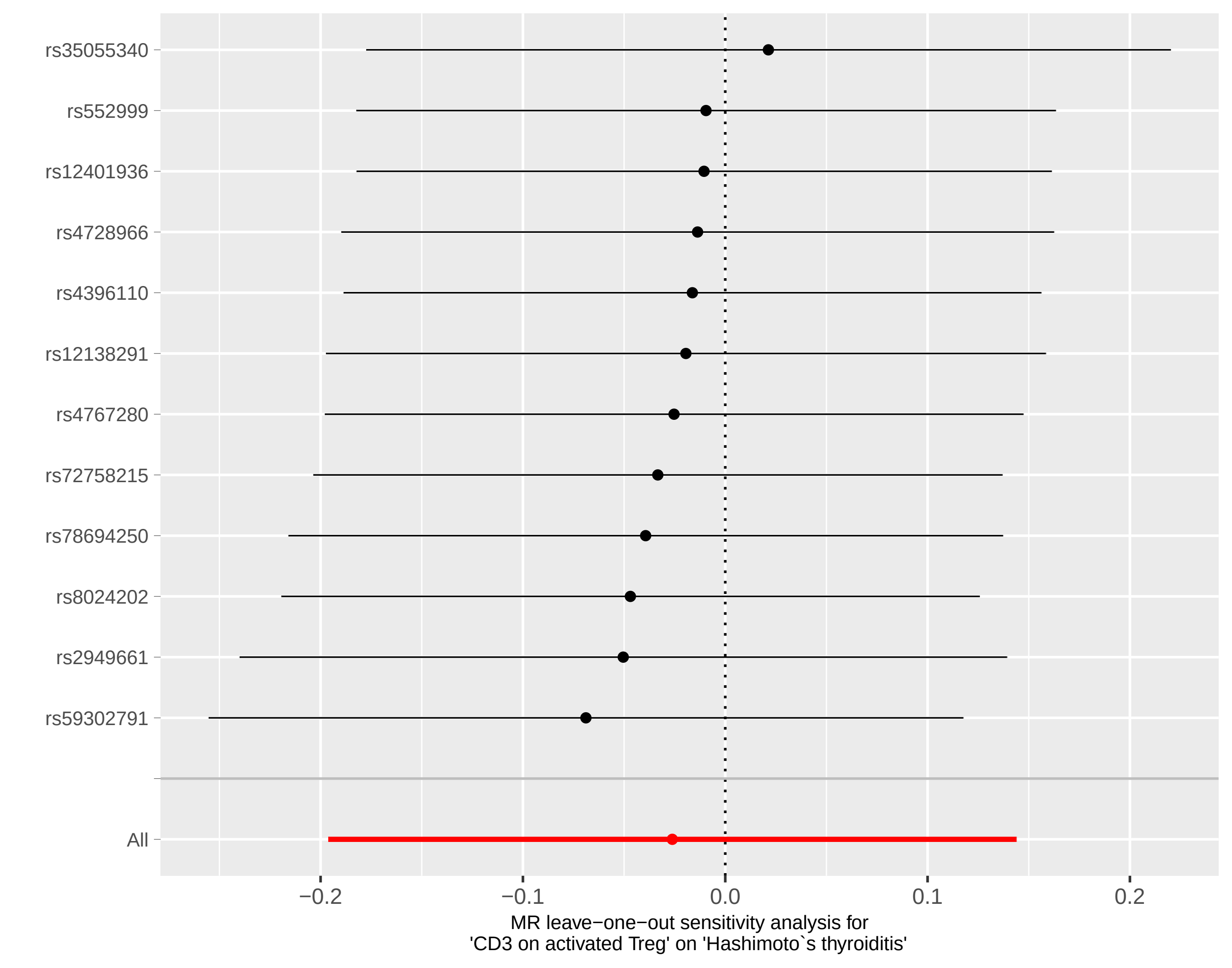
rs35055340
rs552999
rs12401936
rs4728966
rs4396110
rs12138291
rs4767280
rs72758215
rs78694250
rs8024202
rs2949661
rs59302791
All
−0.2
−0.1
0.0
0.1
0.2
MR leave−one−out sensitivity analysis for
'CD3 on activated Treg' on 'Hashimoto`s thyroiditis'

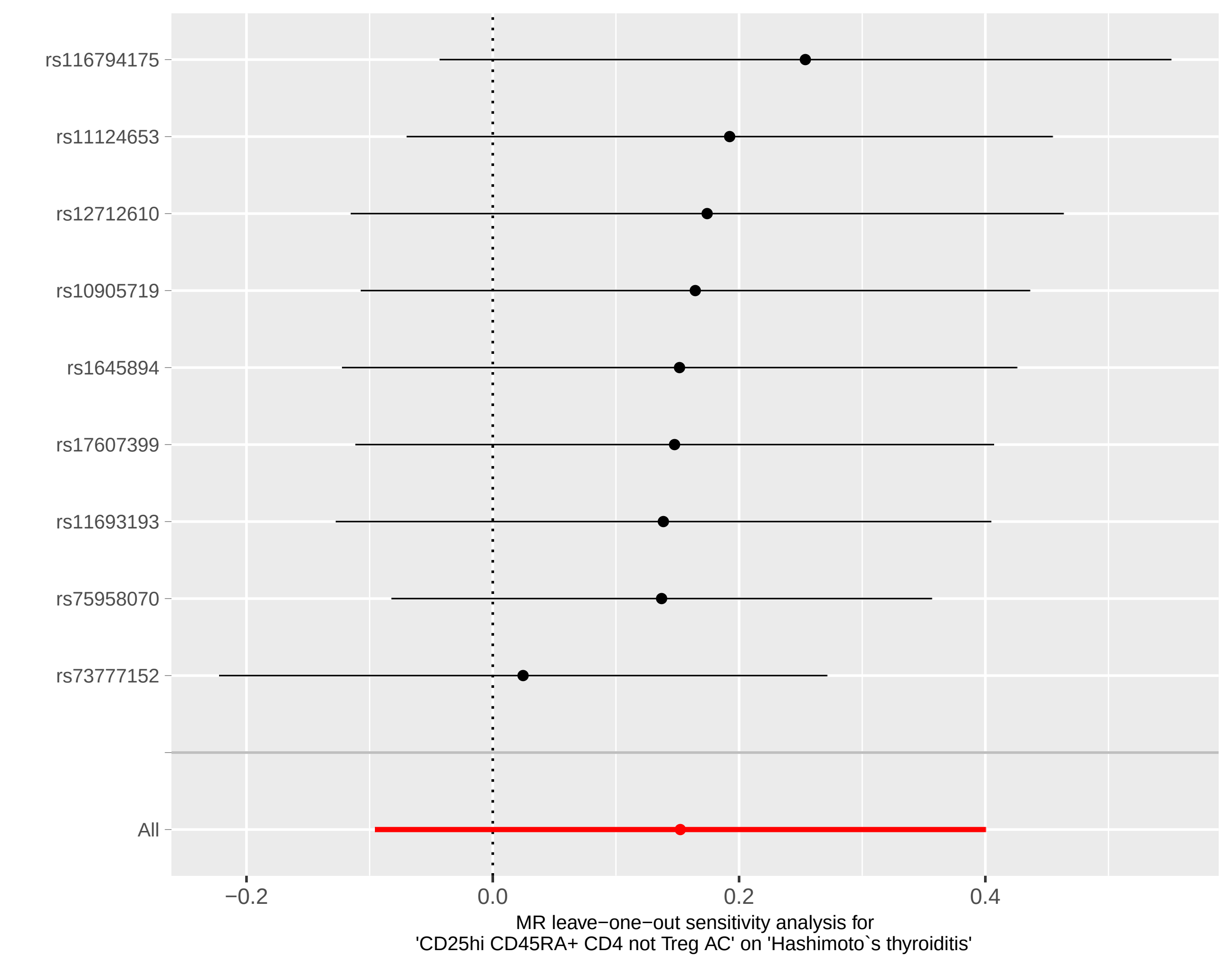

rs116794175
rs11124653
rs12712610
rs10905719
rs1645894
rs17607399
rs11693193
rs75958070
rs73777152
All
−0.2
0.0
0.2
0.4
MR leave−one−out sensitivity analysis for
'CD25hi CD45RA+ CD4 not Treg AC' on 'Hashimoto`s thyroiditis'

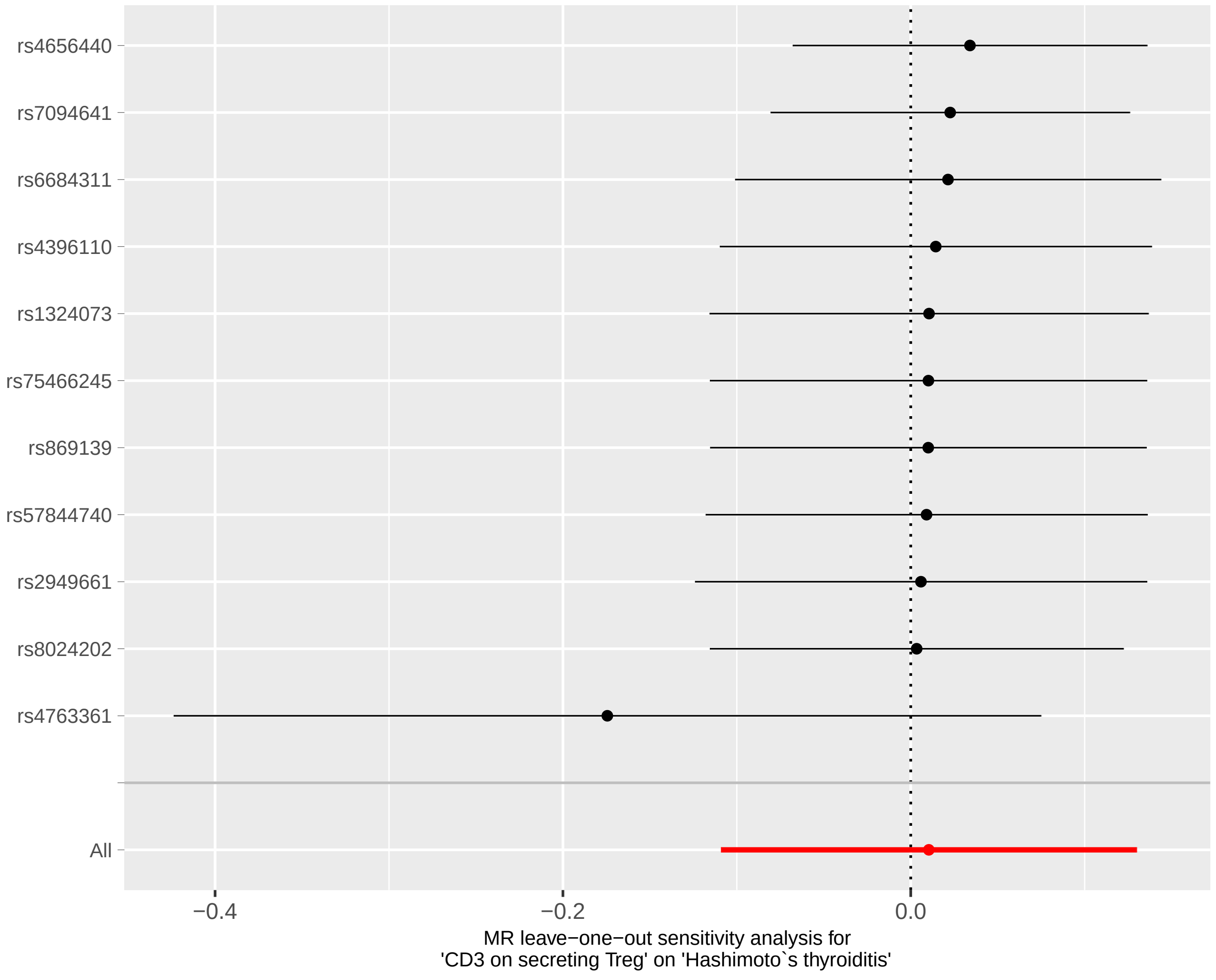

rs4656440
rs7094641
rs6684311
rs4396110
rs1324073
rs75466245
rs869139
rs57844740
rs2949661
rs8024202
rs4763361
All
−0.4
−0.2
0.0
MR leave−one−out sensitivity analysis for
'CD3 on secreting Treg' on 'Hashimoto`s thyroiditis'

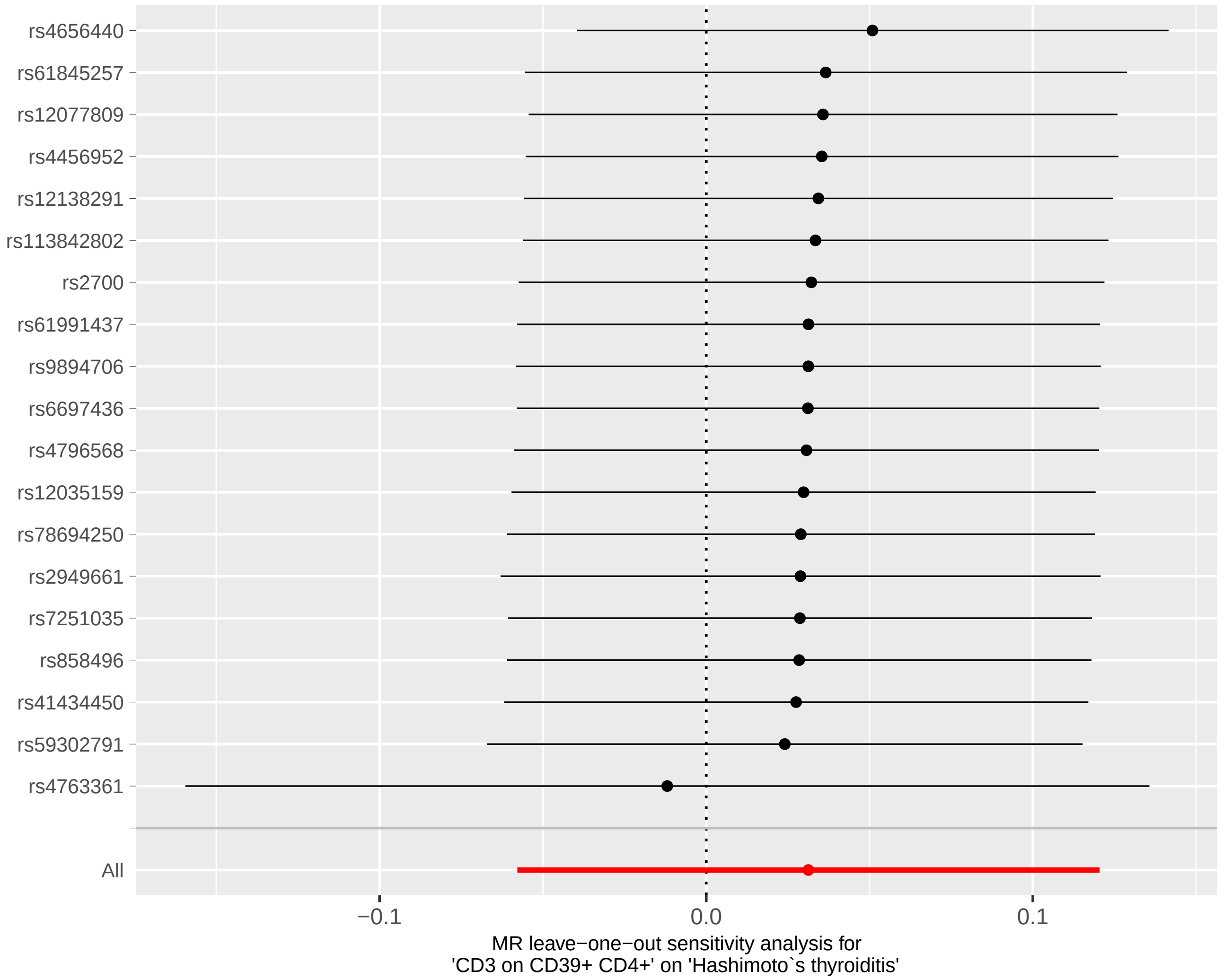
rs4656440
rs61845257
rs12077809
rs4456952
rs12138291
rs113842802
rs2700
rs61991437
rs9894706
rs6697436
rs4796568
rs12035159
rs78694250
rs2949661
rs7251035
rs858496
rs41434450
rs59302791
rs4763361
All
−0.1
0.0
0.1
MR leave−one−out sensitivity analysis for
'CD3 on CD39+ CD4+' on 'Hashimoto`s thyroiditis'

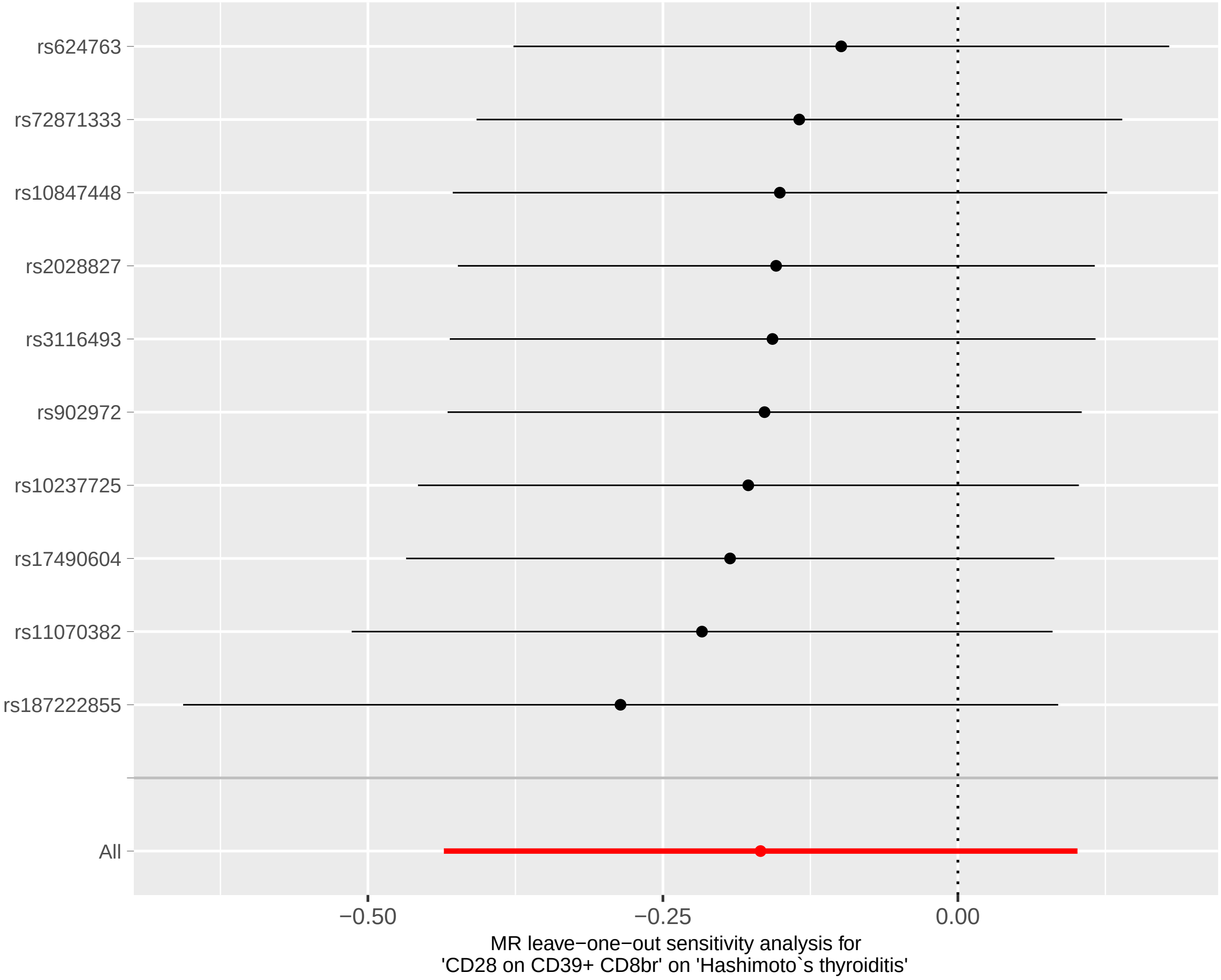
rs624763
rs72871333
rs10847448
rs2028827
rs3116493
rs902972
rs10237725
rs17490604
rs11070382
rs187222855
All
−0.50
−0.25
0.00
MR leave−one−out sensitivity analysis for
'CD28 on CD39+ CD8br' on 'Hashimoto`s thyroiditis'

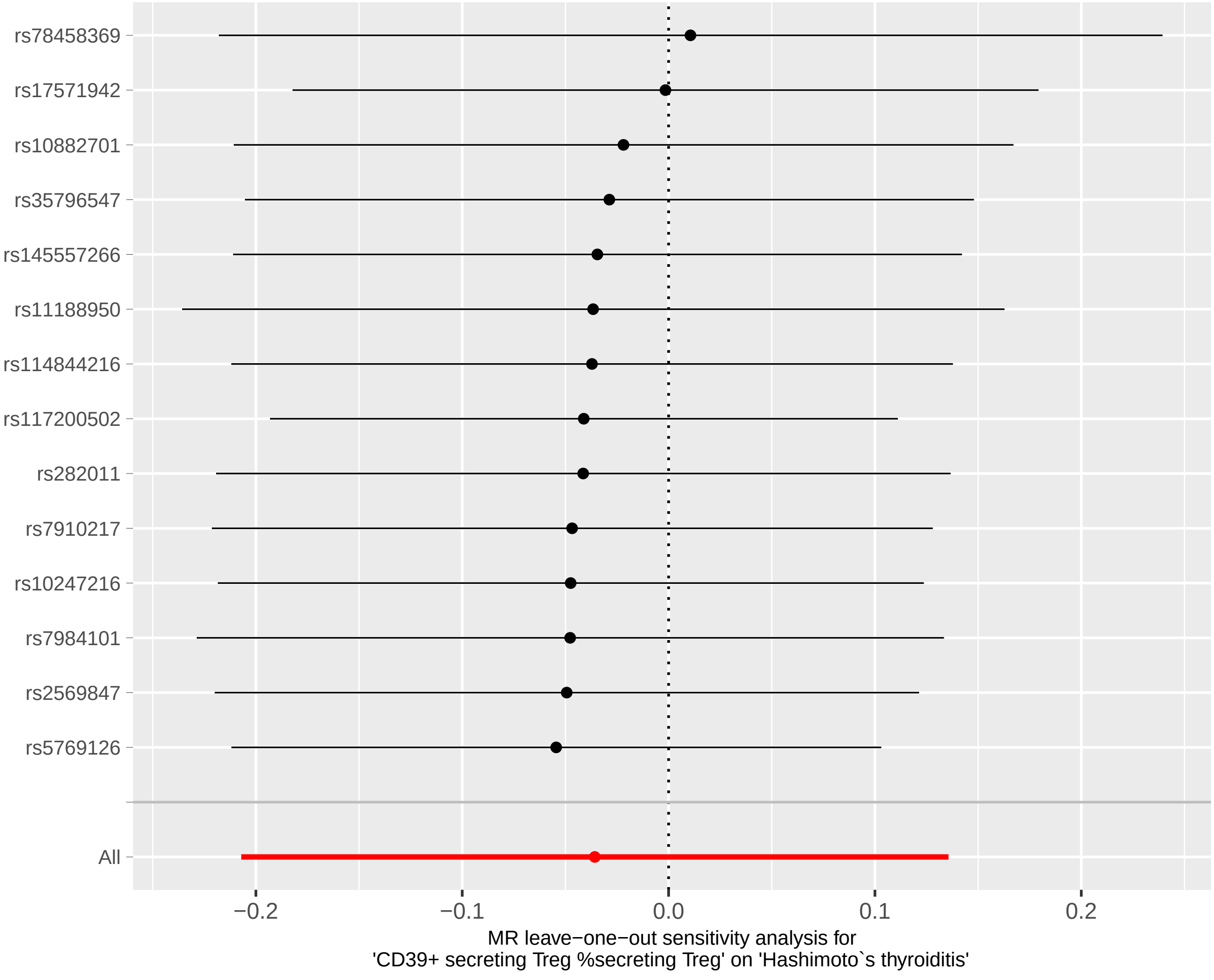
rs78458369
rs17571942
rs10882701
rs35796547
rs145557266
rs11188950
rs114844216
rs117200502
rs282011
rs7910217
rs10247216
rs7984101
rs2569847
rs5769126
All
−0.2
−0.1
0.0
0.1
0.2
MR leave−one−out sensitivity analysis for
'CD39+ secreting Treg %secreting Treg' on 'Hashimoto`s thyroiditis'

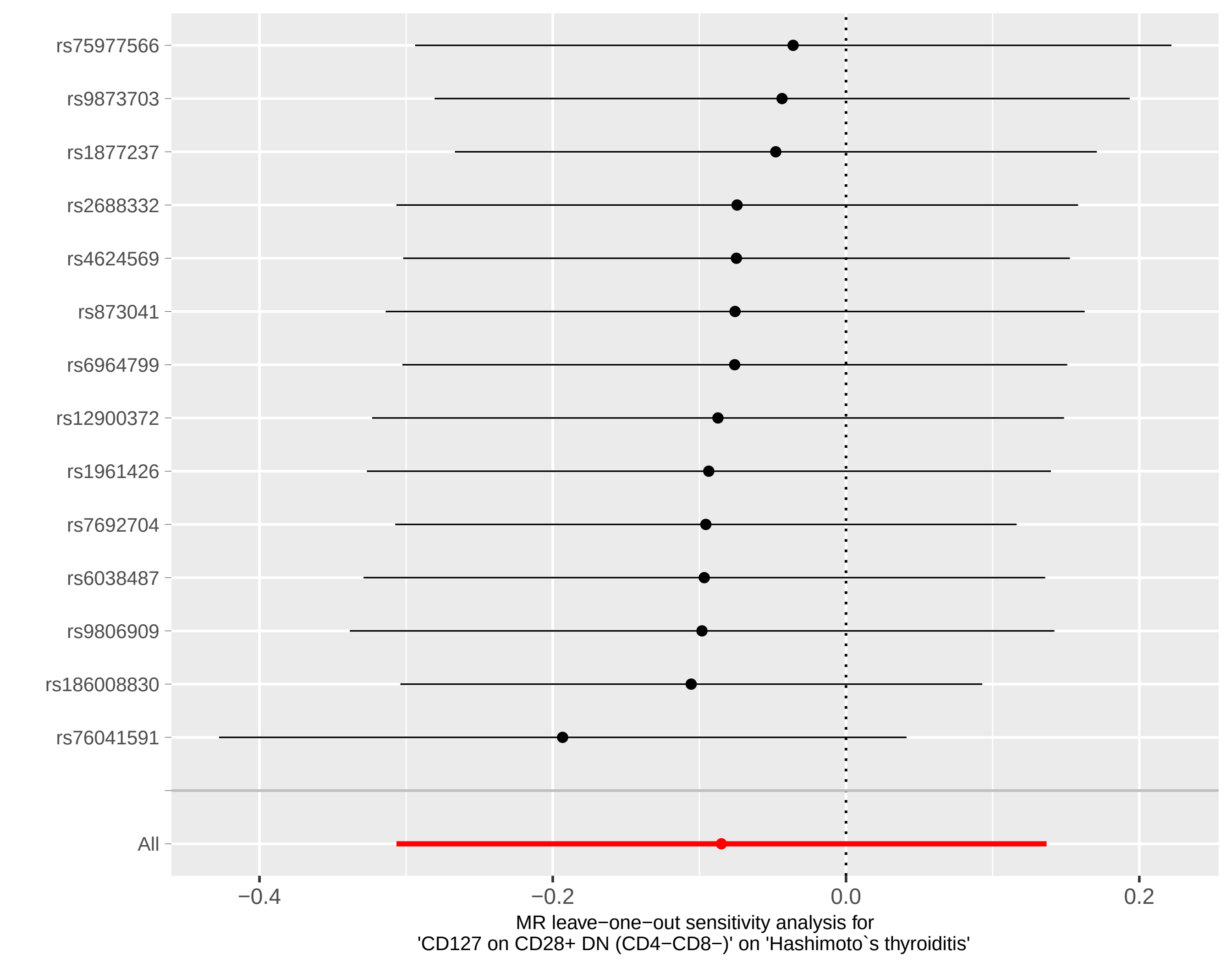
rs75977566
rs9873703
rs1877237
rs2688332
rs4624569
rs873041
rs6964799
rs12900372
rs1961426
rs7692704
rs6038487
rs9806909
rs186008830
rs76041591
All
−0.4
−0.2
0.0
0.2
MR leave−one−out sensitivity analysis for
'CD127 on CD28+ DN (CD4−CD8−)' on 'Hashimoto`s thyroiditis'

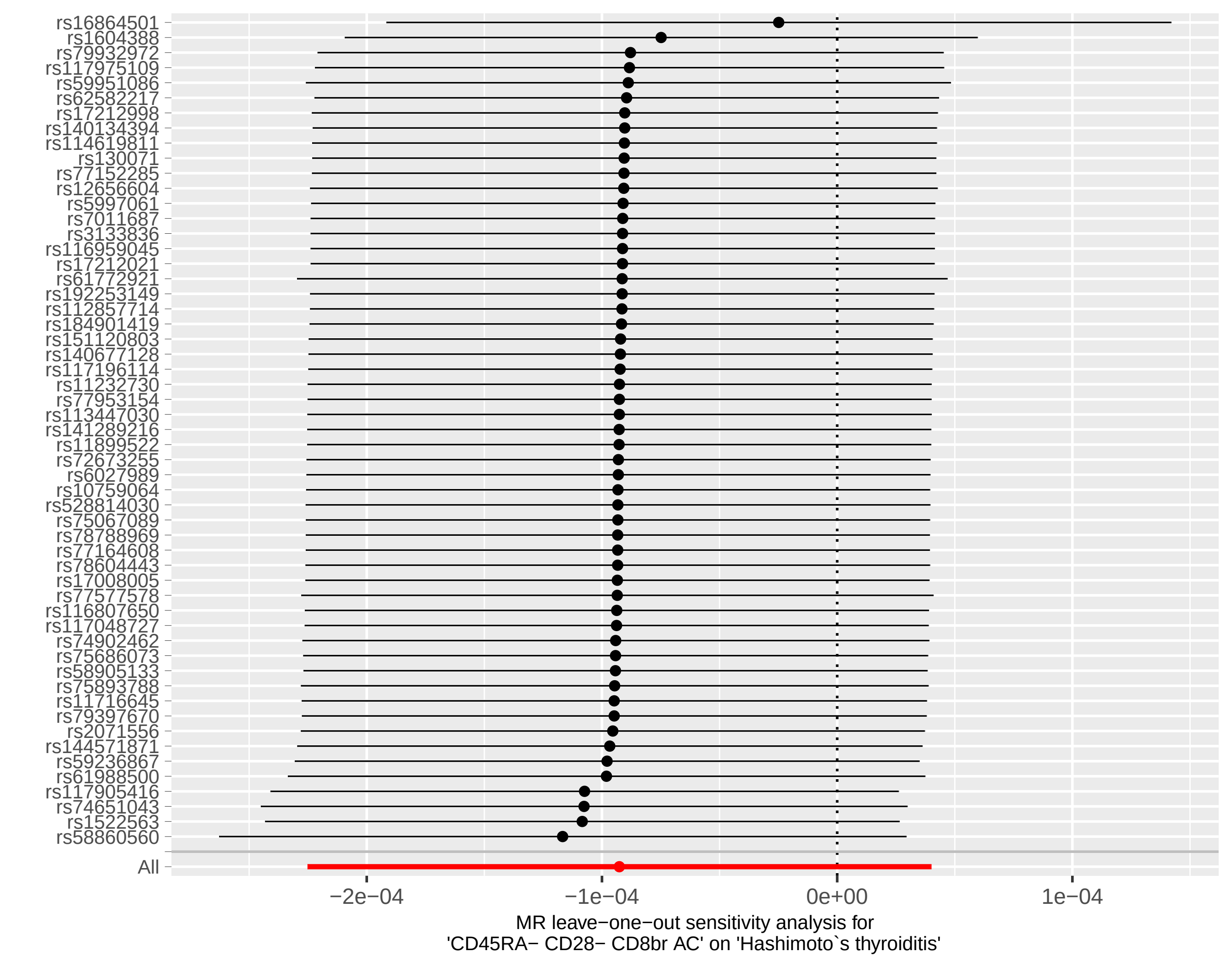
rs16864501
rs1604388
rs79932972
rs117975109
rs59951086
rs62582217
rs17212998
rs140134394
rs114619811
rs130071
rs77152285
rs12656604
rs5997061
rs7011687
rs3133836
rs116959045
rs17212021
rs61772921
rs192253149
rs112857714
rs184901419
rs151120803
rs140677128
rs117196114
rs11232730
rs77953154
rs113447030
rs141289216
rs11899522
rs72673255
rs6027989
rs10759064
rs528814030
rs75067089
rs78788969
rs77164608
rs78604443
rs17008005
rs77577578
rs116807650
rs117048727
rs74902462
rs75686073
rs58905133
rs75893788
rs11716645
rs79397670
rs2071556
rs144571871
rs59236867
rs61988500
rs117905416
rs74651043
rs1522563
rs58860560
All
−2e−04
−1e−04
0e+00
1e−04
MR leave−one−out sensitivity analysis for
'CD45RA− CD28− CD8br AC' on 'Hashimoto`s thyroiditis'

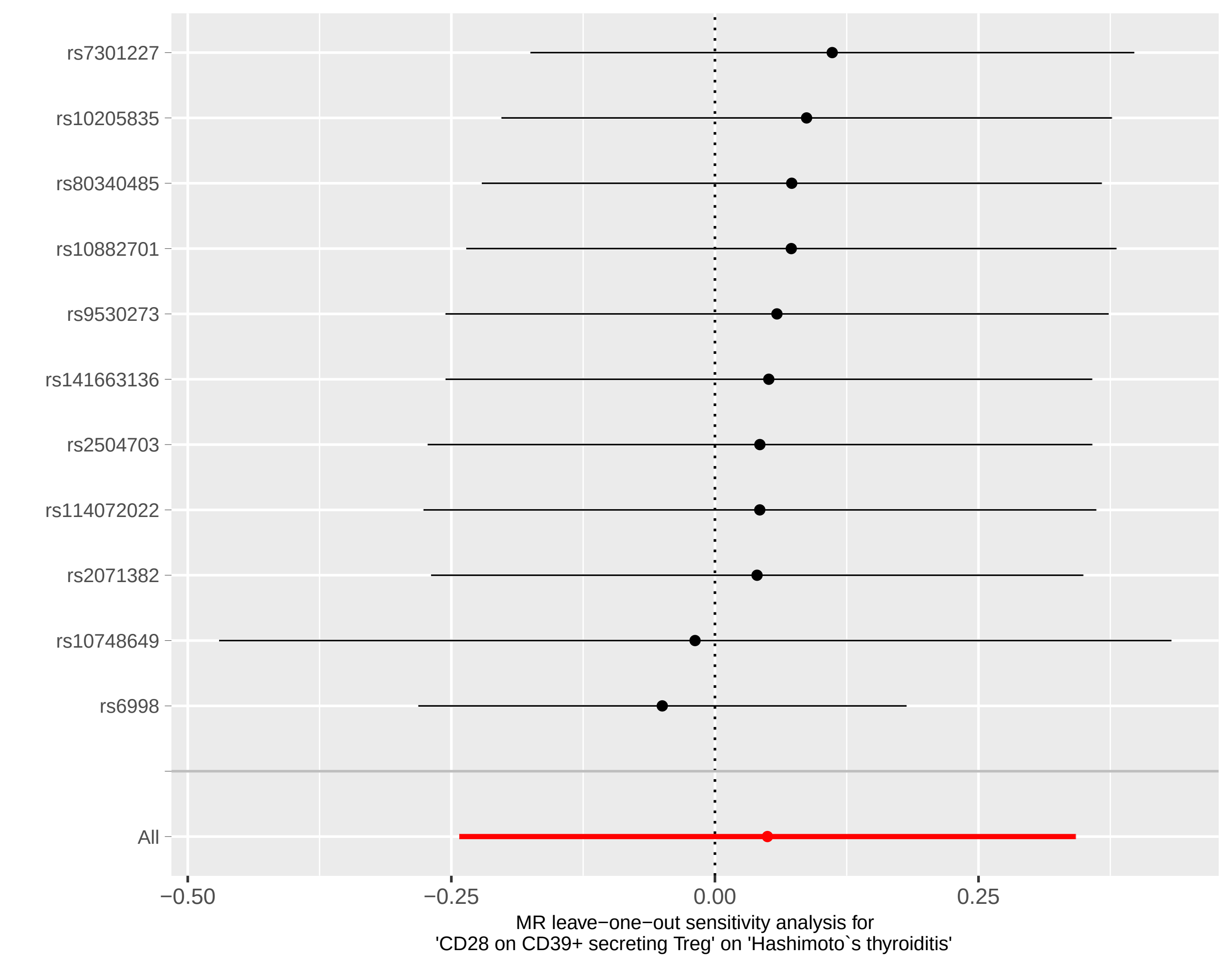
rs7301227
rs10205835
rs80340485
rs10882701
rs9530273
rs141663136
rs2504703
rs114072022
rs2071382
rs10748649
rs6998
All
−0.50
−0.25
0.00
0.25
MR leave−one−out sensitivity analysis for
'CD28 on CD39+ secreting Treg' on 'Hashimoto`s thyroiditis'

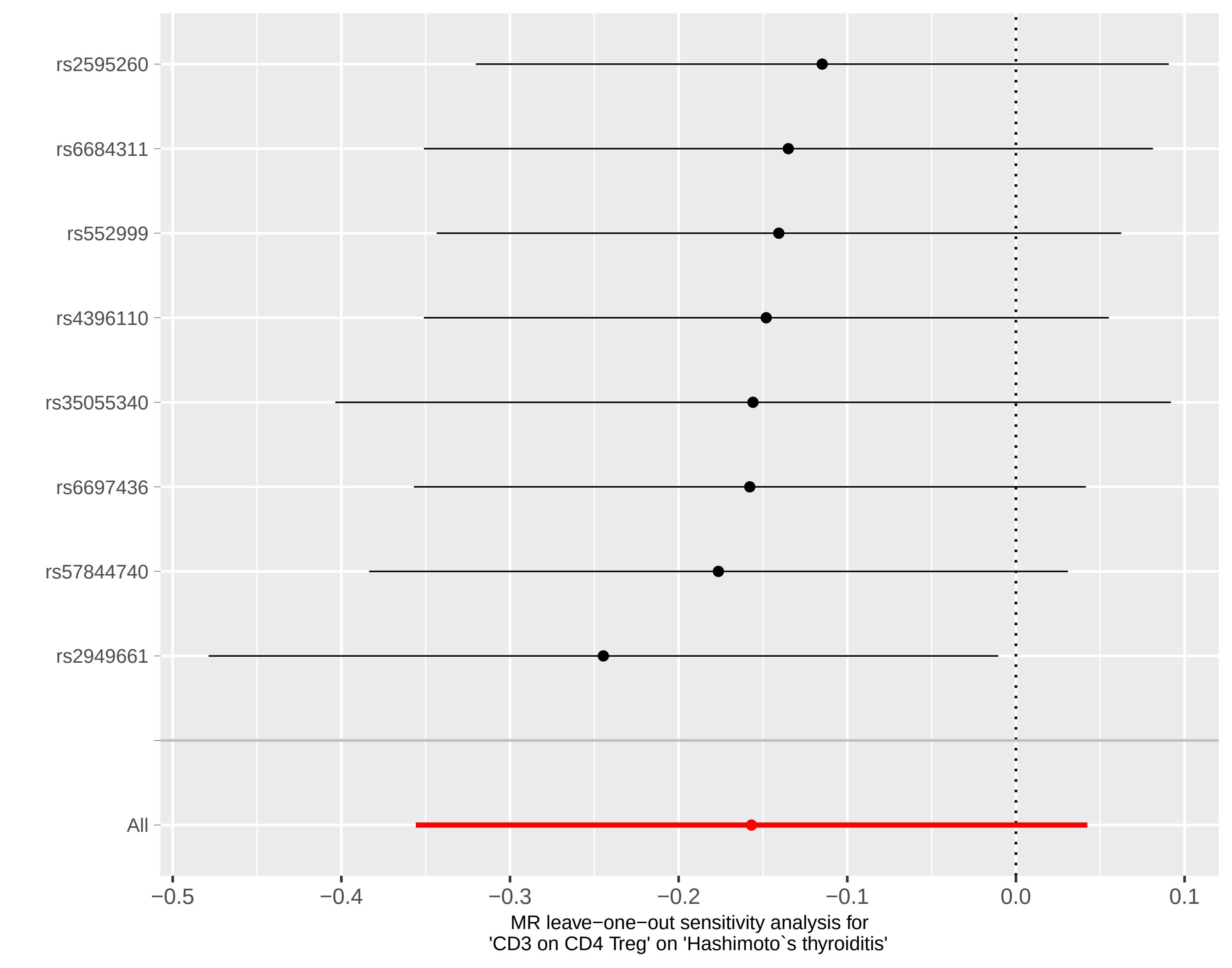

rs2595260
rs6684311
rs552999
rs4396110
rs35055340
rs6697436
rs57844740
rs2949661
All
−0.5
−0.4
−0.3
−0.2
−0.1
0.0
0.1
MR leave−one−out sensitivity analysis for
'CD3 on CD4 Treg' on 'Hashimoto`s thyroiditis'

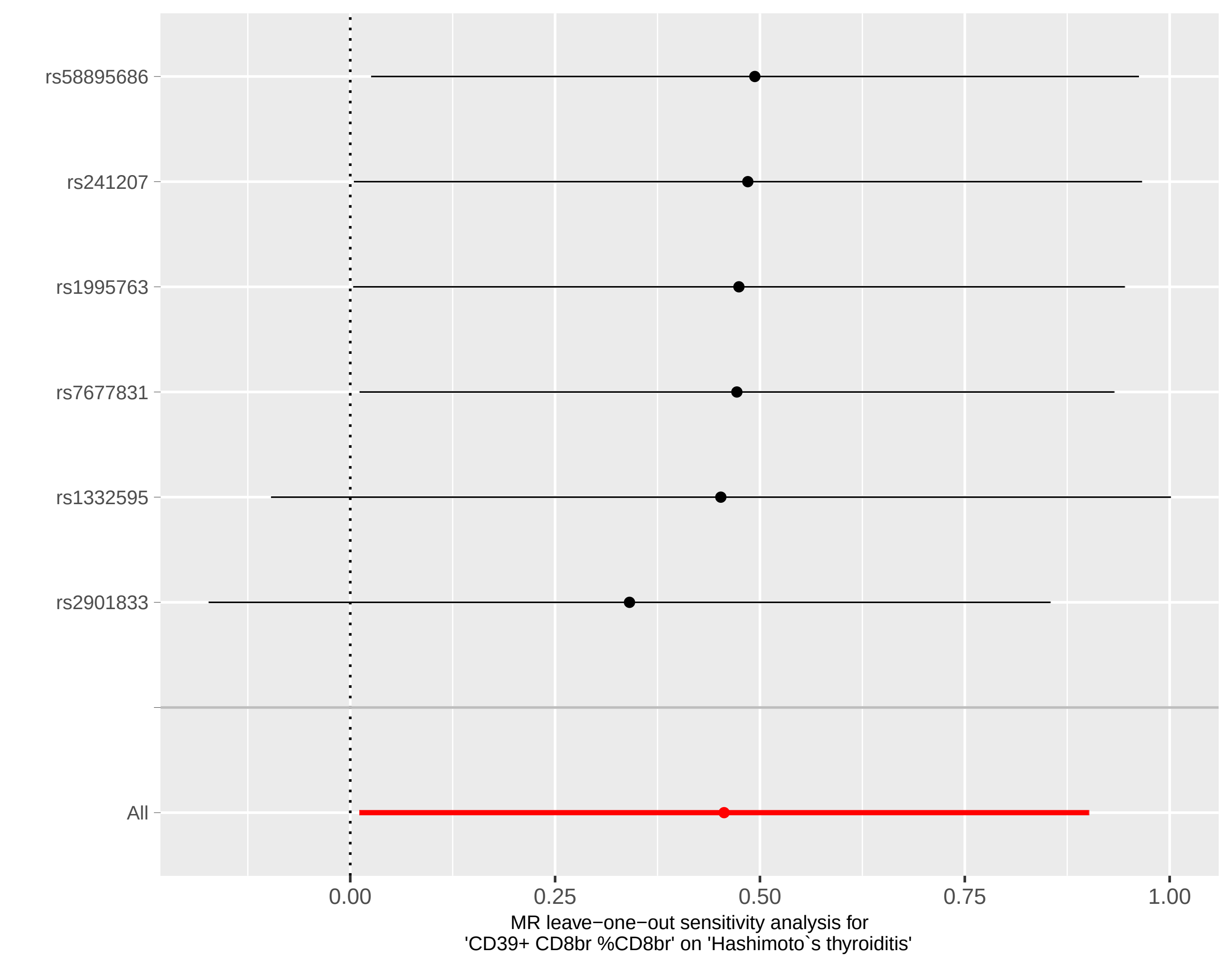
rs58895686
rs241207
rs1995763
rs7677831
rs1332595
rs2901833
All
0.00
0.25
0.50
0.75
1.00
MR leave−one−out sensitivity analysis for
'CD39+ CD8br %CD8br' on 'Hashimoto`s thyroiditis'

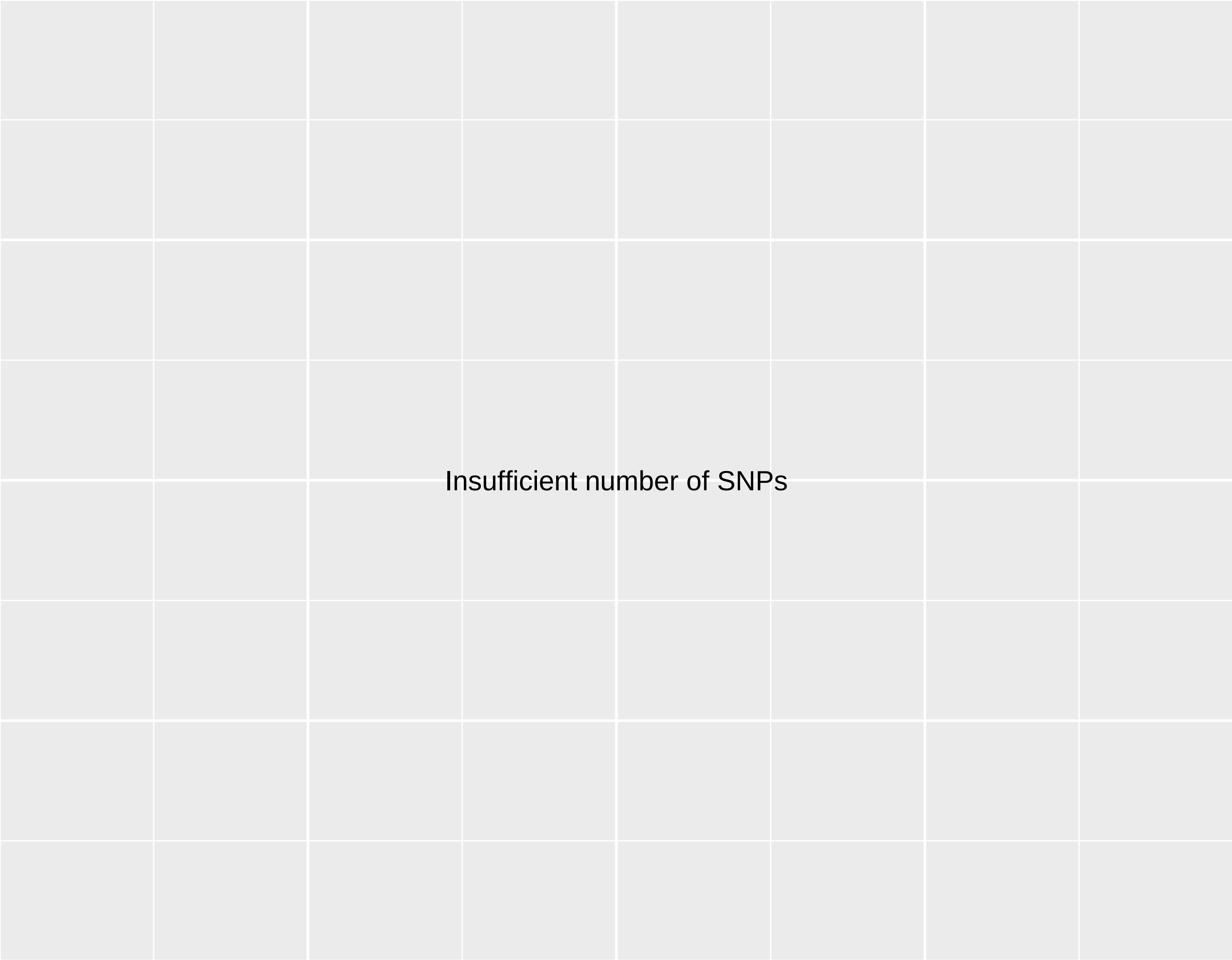
Insufficient number of SNPs

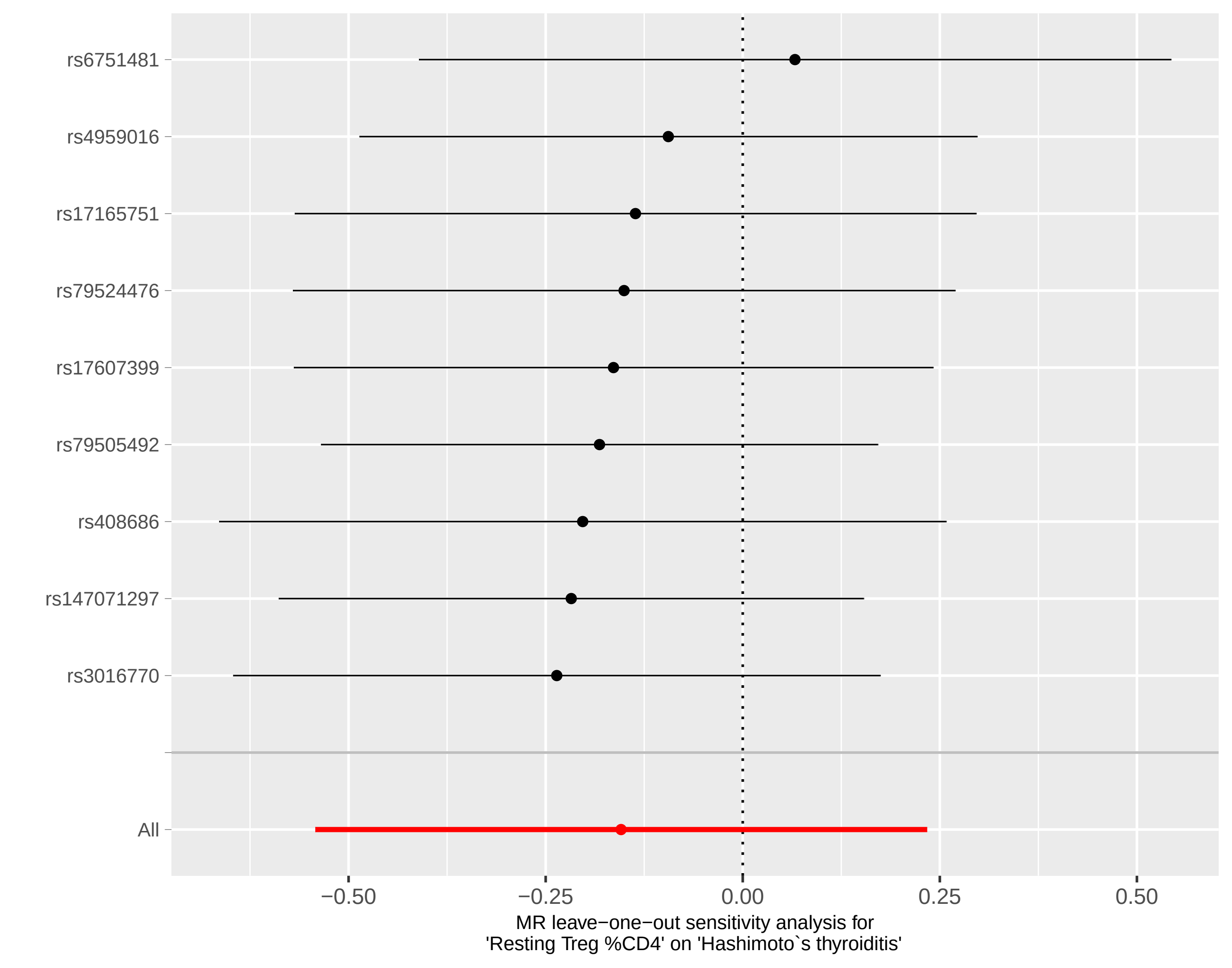

rs6751481
rs4959016
rs17165751
rs79524476
rs17607399
rs79505492
rs408686
rs147071297
rs3016770
All
−0.50
−0.25
0.00
0.25
0.50
MR leave−one−out sensitivity analysis for
'Resting Treg %CD4' on 'Hashimoto`s thyroiditis'

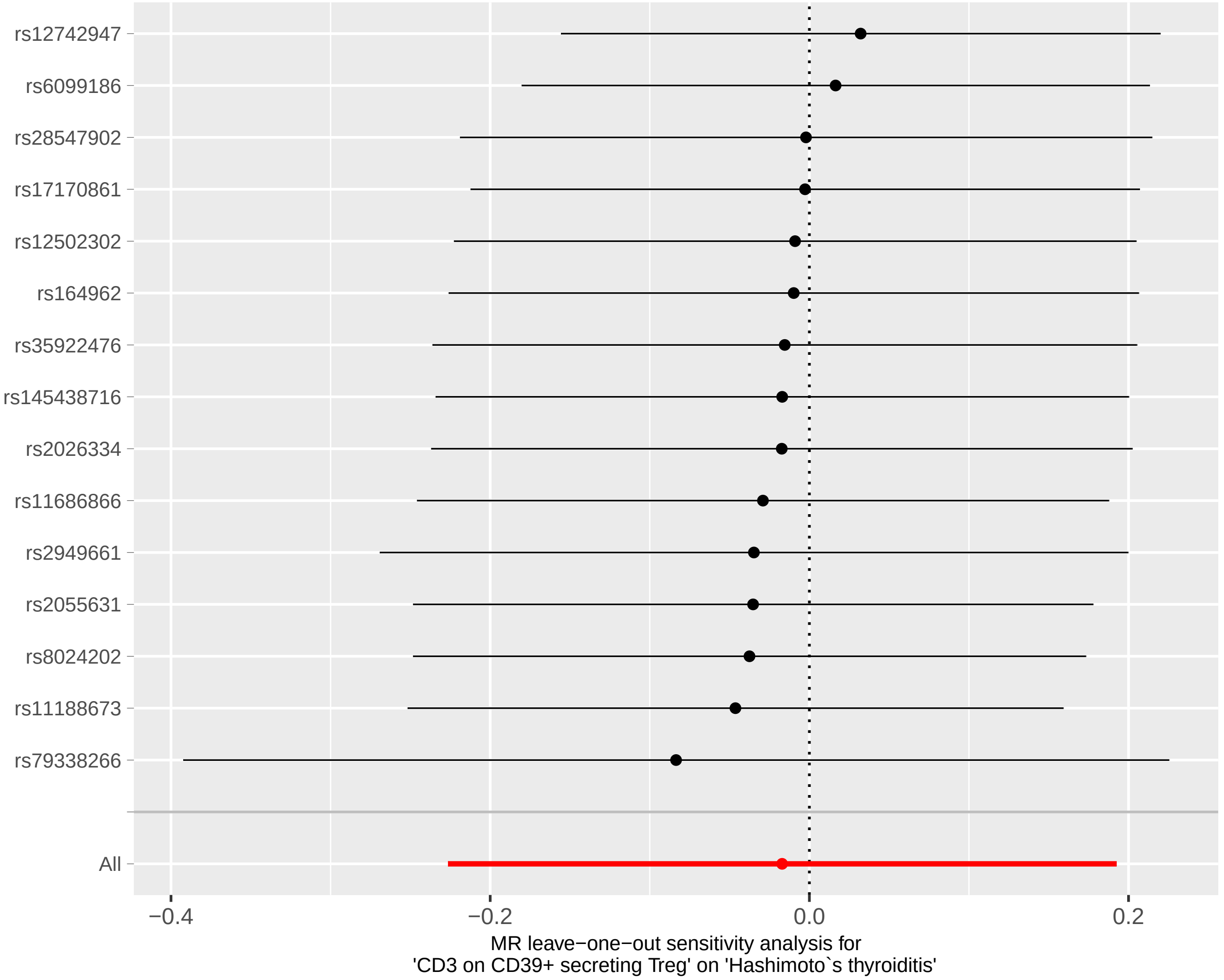
rs12742947
rs6099186
rs28547902
rs17170861
rs12502302
rs164962
rs35922476
rs145438716
rs2026334
rs11686866
rs2949661
rs2055631
rs8024202
rs11188673
rs79338266
All
−0.4
−0.2
0.0
0.2
MR leave−one−out sensitivity analysis for
'CD3 on CD39+ secreting Treg' on 'Hashimoto`s thyroiditis'

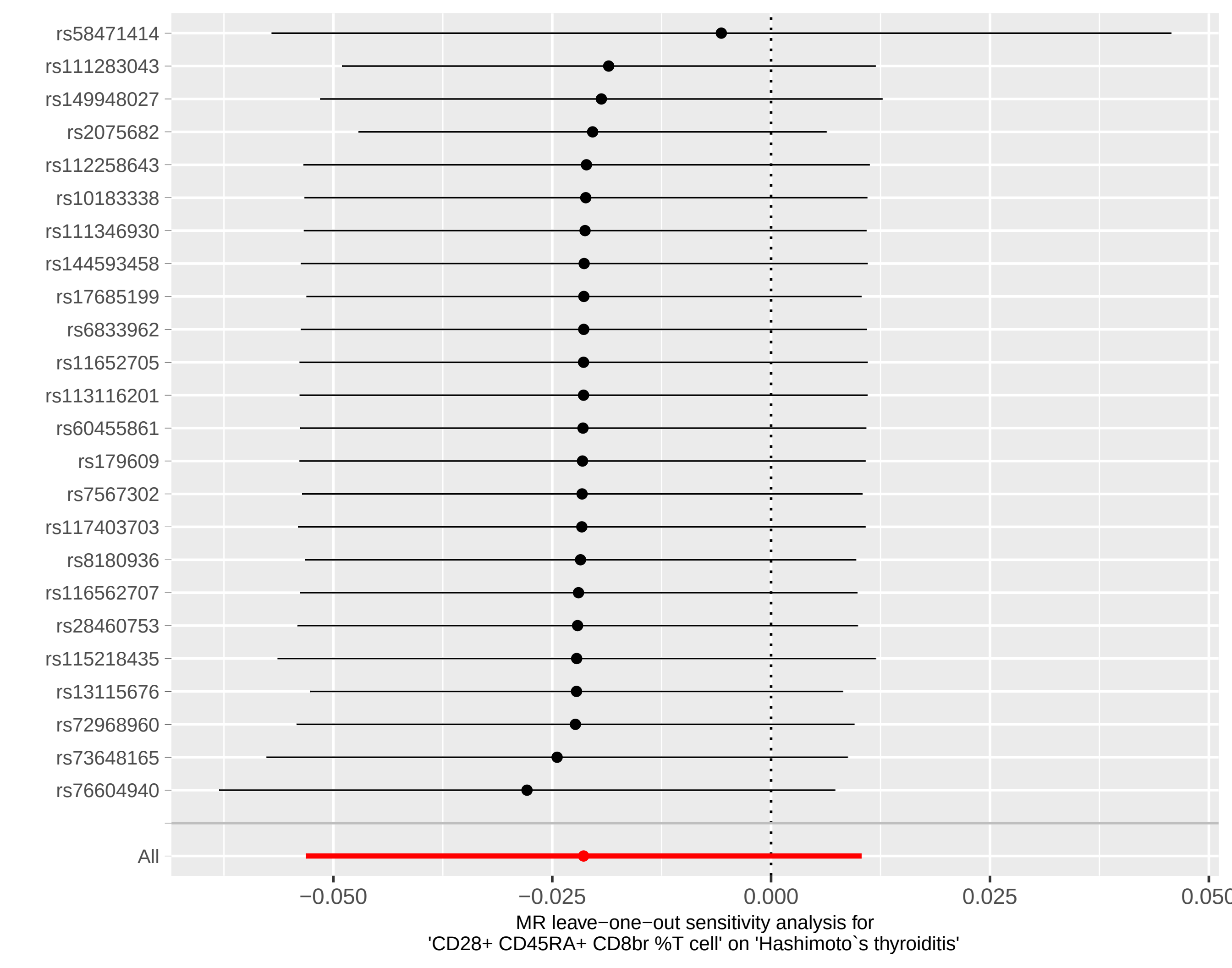
rs58471414
rs111283043
rs149948027
rs2075682
rs112258643
rs10183338
rs111346930
rs144593458
rs17685199
rs6833962
rs11652705
rs113116201
rs60455861
rs179609
rs7567302
rs117403703
rs8180936
rs116562707
rs28460753
rs115218435
rs13115676
rs72968960
rs73648165
rs76604940
All
−0.050
−0.025
0.000
0.025
0.050
MR leave−one−out sensitivity analysis for
'CD28+ CD45RA+ CD8br %T cell' on 'Hashimoto`s thyroiditis'

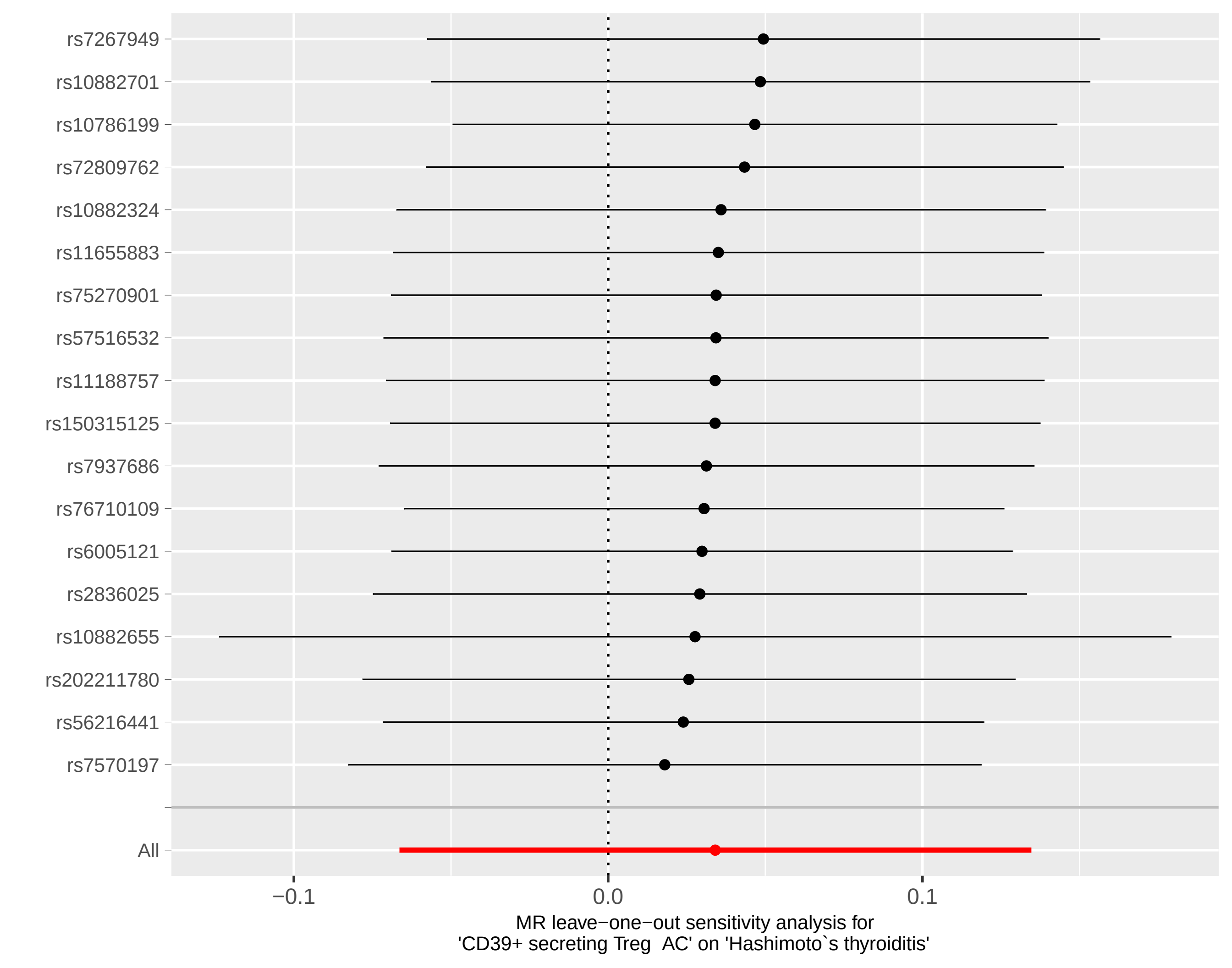

rs7267949
rs10882701
rs10786199
rs72809762
rs10882324
rs11655883
rs75270901
rs57516532
rs11188757
rs150315125
rs7937686
rs76710109
rs6005121
rs2836025
rs10882655
rs202211780
rs56216441
rs7570197
All
−0.1
0.0
0.1
MR leave−one−out sensitivity analysis for
'CD39+ secreting Treg AC' on 'Hashimoto`s thyroiditis'

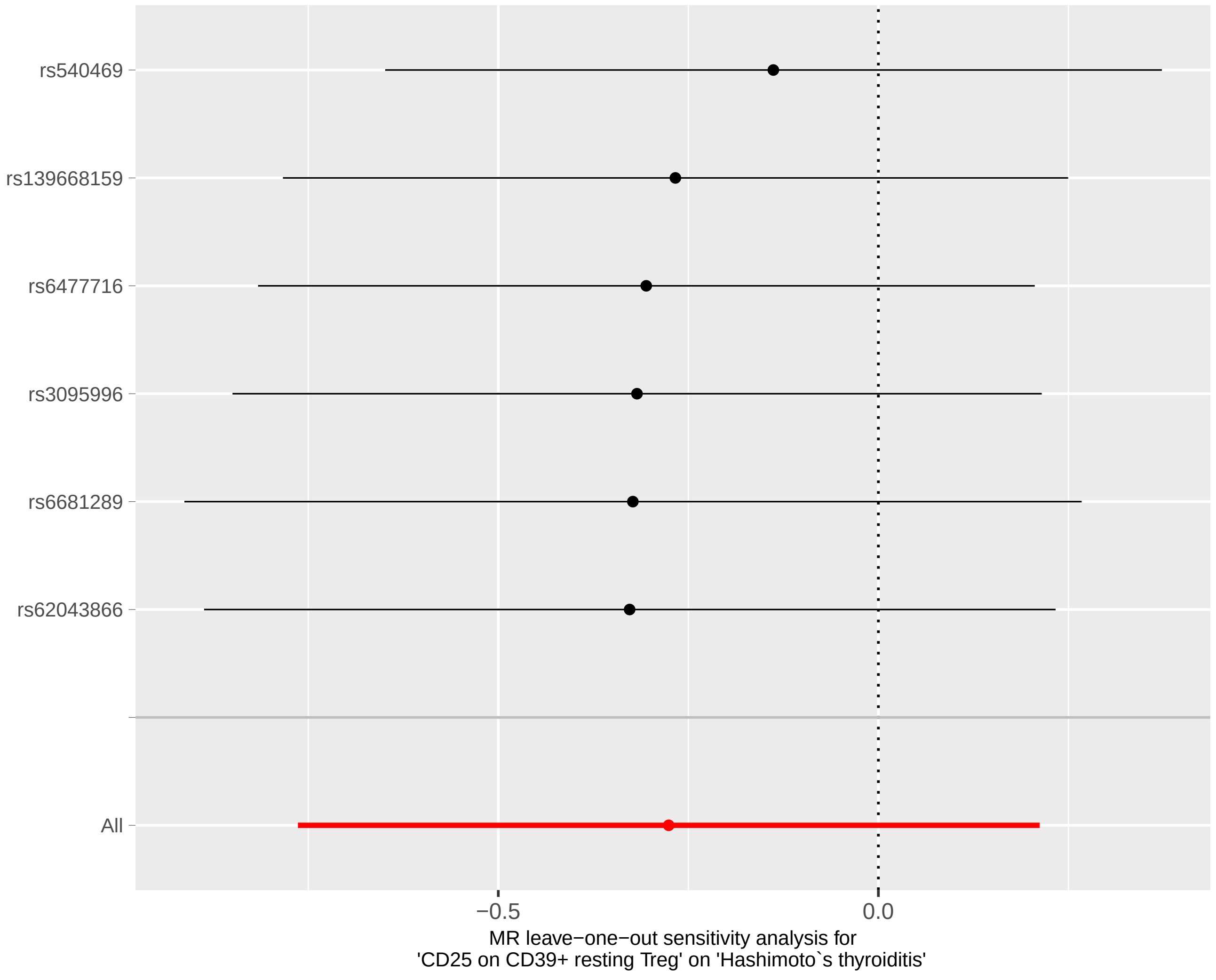
rs540469
rs139668159
rs6477716
rs3095996
rs6681289
rs62043866
All
−0.5
0.0
MR leave−one−out sensitivity analysis for
'CD25 on CD39+ resting Treg' on 'Hashimoto`s thyroiditis'

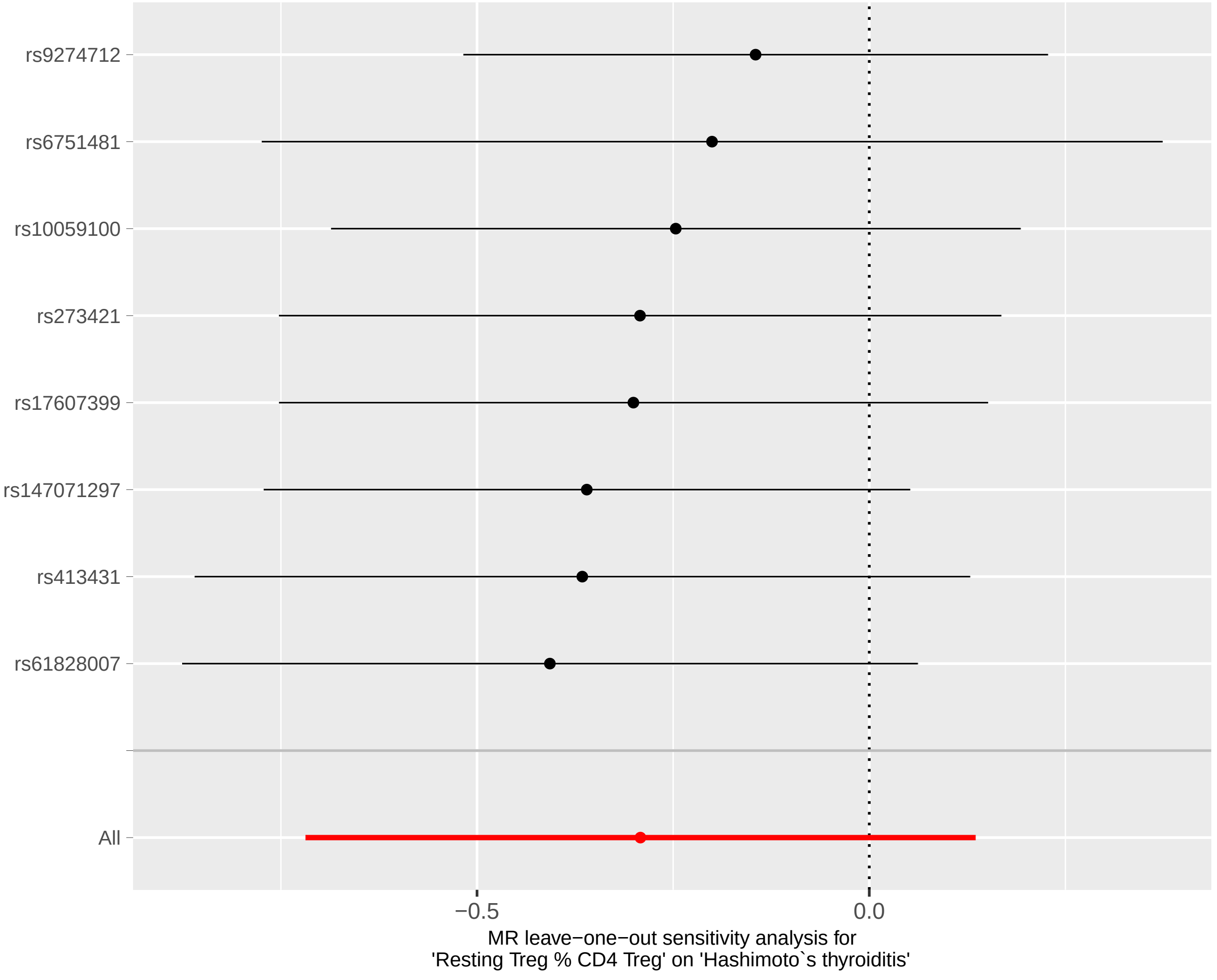
rs9274712
rs6751481
rs10059100
rs273421
rs17607399
rs147071297
rs413431
rs61828007
All
−0.5
0.0
MR leave−one−out sensitivity analysis for
'Resting Treg % CD4 Treg' on 'Hashimoto`s thyroiditis'

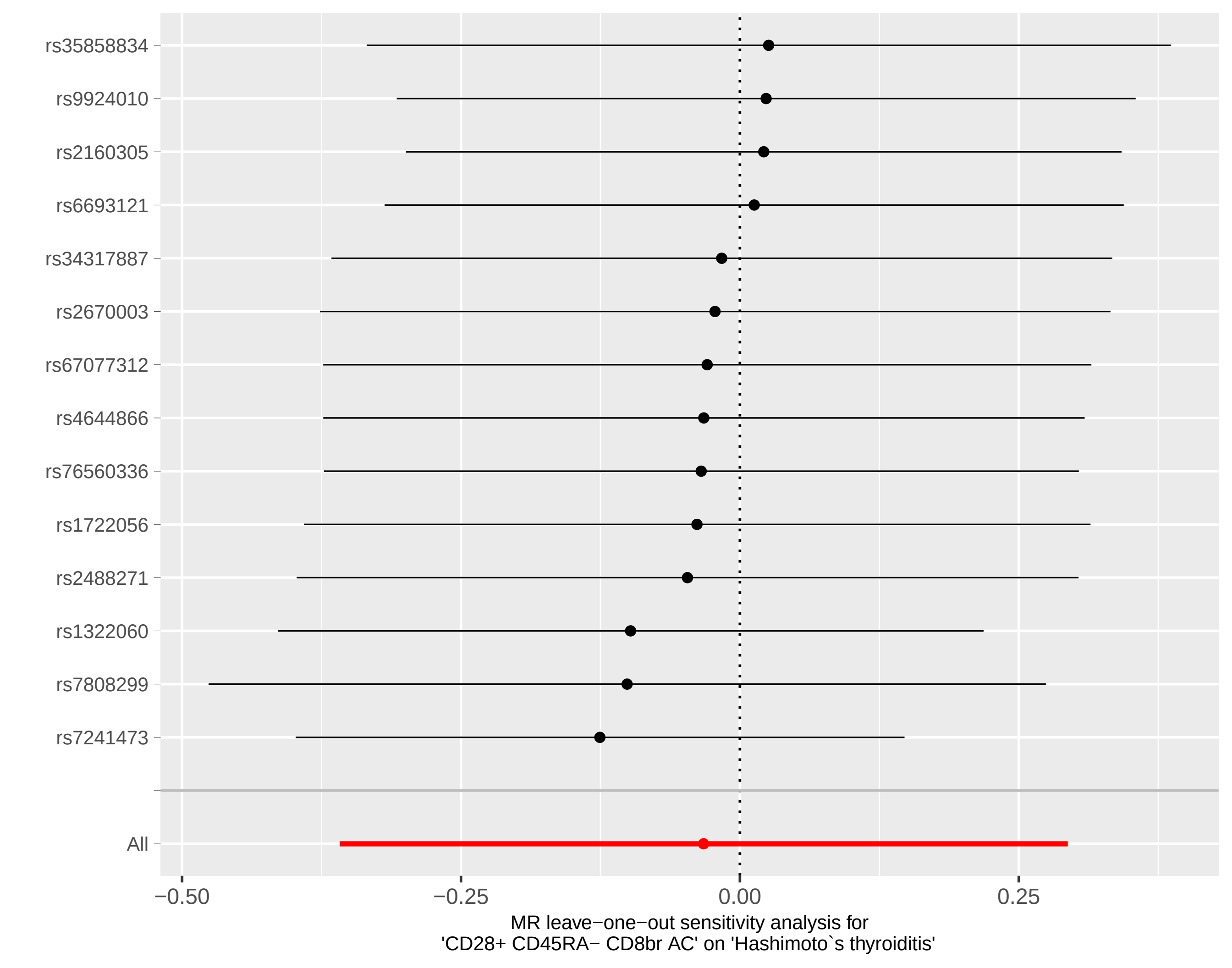
rs35858834
rs9924010
rs2160305
rs6693121
rs34317887
rs2670003
rs67077312
rs4644866
rs76560336
rs1722056
rs2488271
rs1322060
rs7808299
rs7241473
All
−0.50
−0.25
0.00
0.25
MR leave−one−out sensitivity analysis for
'CD28+ CD45RA− CD8br AC' on 'Hashimoto`s thyroiditis'

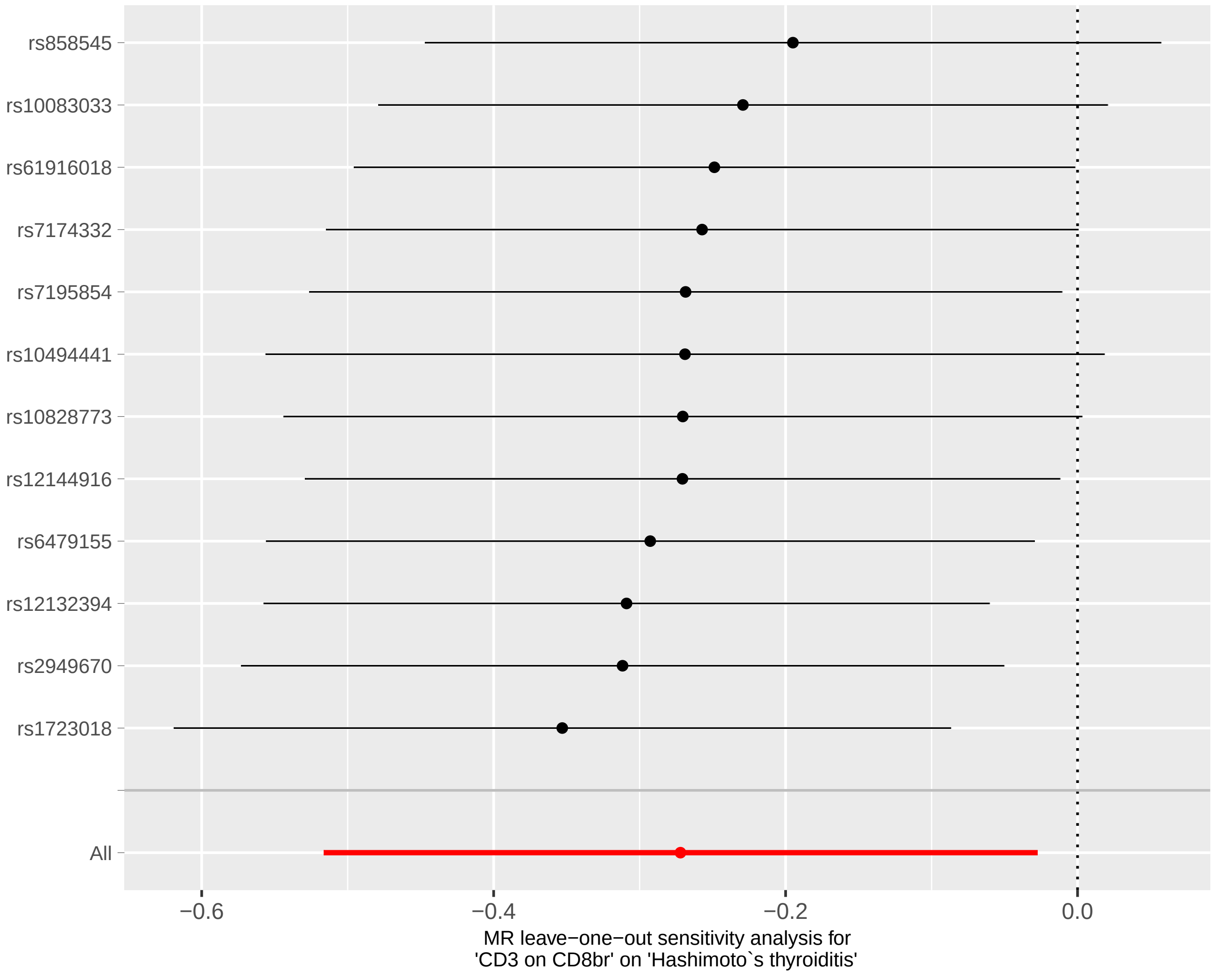
rs858545
rs10083033
rs61916018
rs7174332
rs7195854
rs10494441
rs10828773
rs12144916
rs6479155
rs12132394
rs2949670
rs1723018
All
−0.6
−0.4
−0.2
0.0
MR leave−one−out sensitivity analysis for
'CD3 on CD8br' on 'Hashimoto`s thyroiditis'

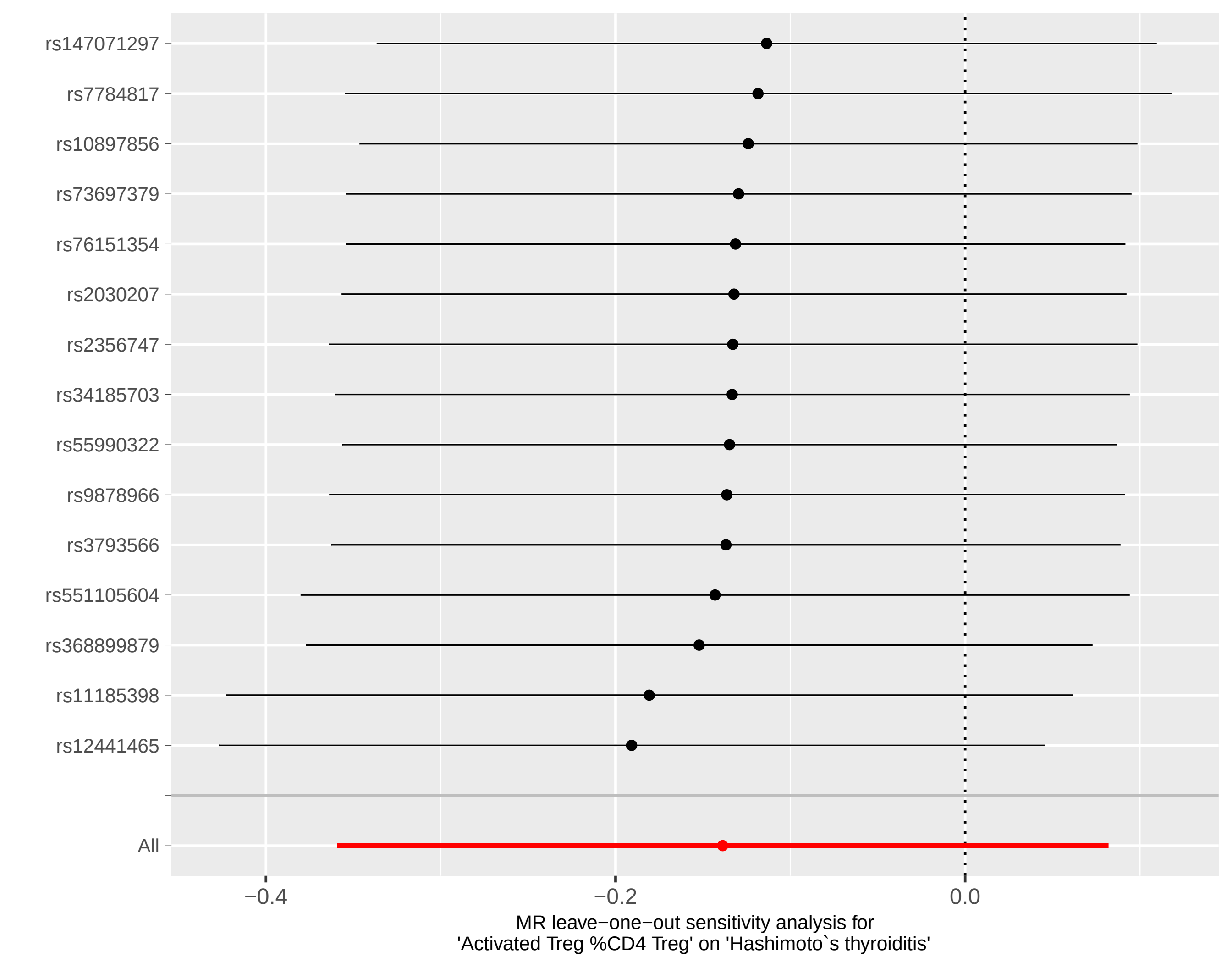
rs147071297
rs7784817
rs10897856
rs73697379
rs76151354
rs2030207
rs2356747
rs34185703
rs55990322
rs9878966
rs3793566
rs551105604
rs368899879
rs11185398
rs12441465
All
−0.4
−0.2
0.0
MR leave−one−out sensitivity analysis for
'Activated Treg %CD4 Treg' on 'Hashimoto`s thyroiditis'

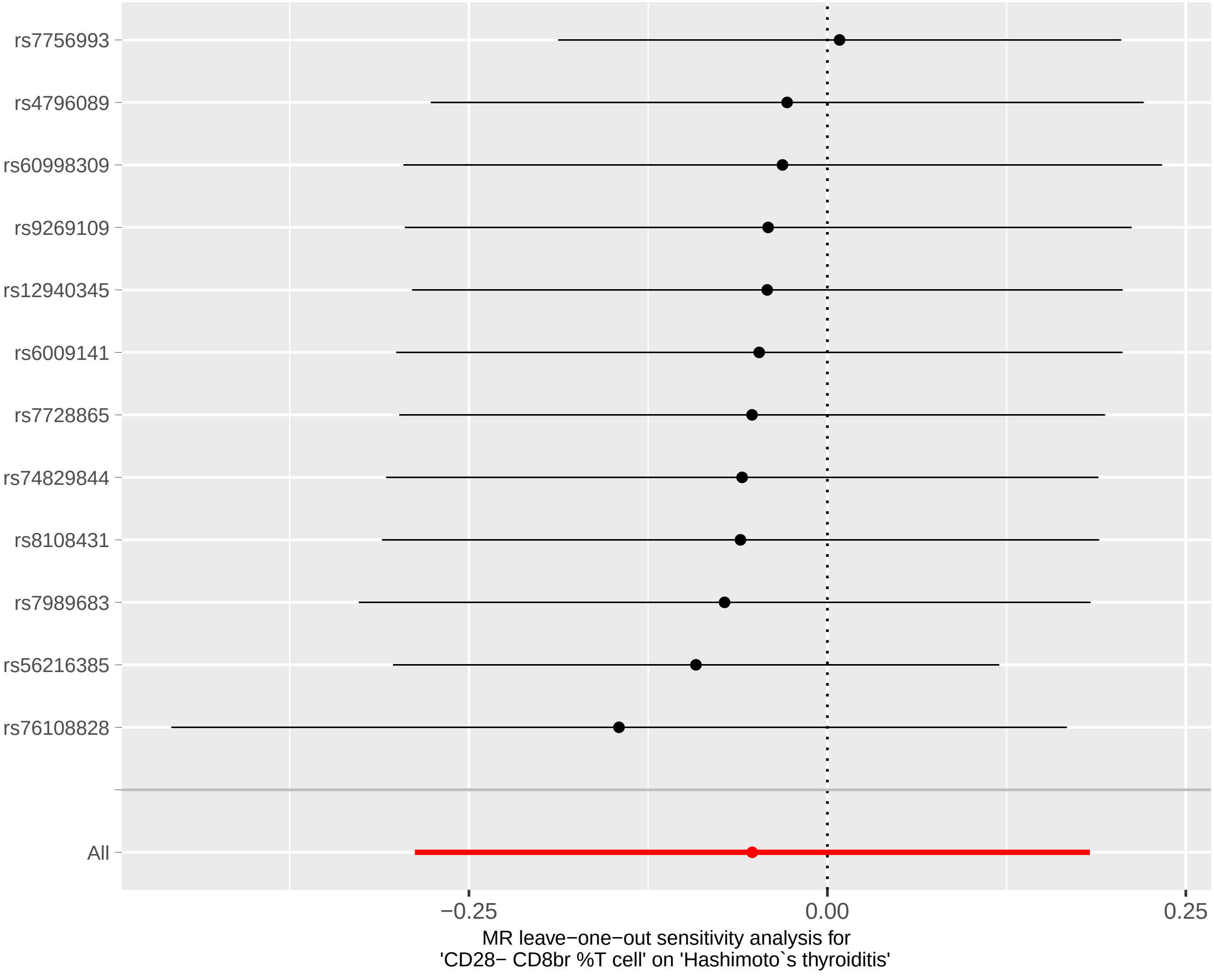
rs7756993
rs4796089
rs60998309
rs9269109
rs12940345
rs6009141
rs7728865
rs74829844
rs8108431
rs7989683
rs56216385
rs76108828
All
−0.25
0.00
0.25
MR leave−one−out sensitivity analysis for
'CD28− CD8br %T cell' on 'Hashimoto`s thyroiditis'

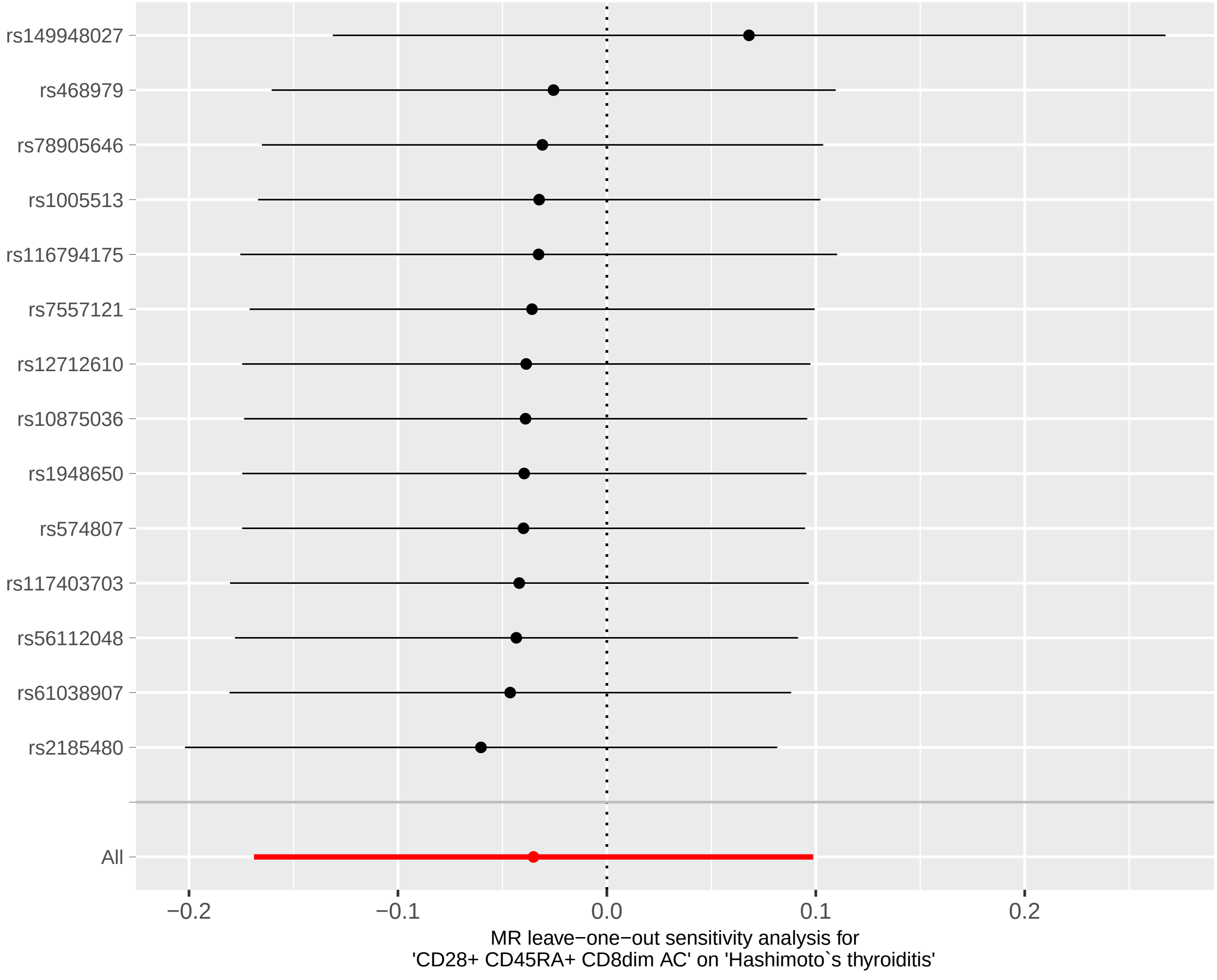
rs149948027
rs468979
rs78905646
rs1005513
rs116794175
rs7557121
rs12712610
rs10875036
rs1948650
rs574807
rs117403703
rs56112048
rs61038907
rs2185480
All
−0.2
−0.1
0.0
0.1
0.2
MR leave−one−out sensitivity analysis for
'CD28+ CD45RA+ CD8dim AC' on 'Hashimoto`s thyroiditis'

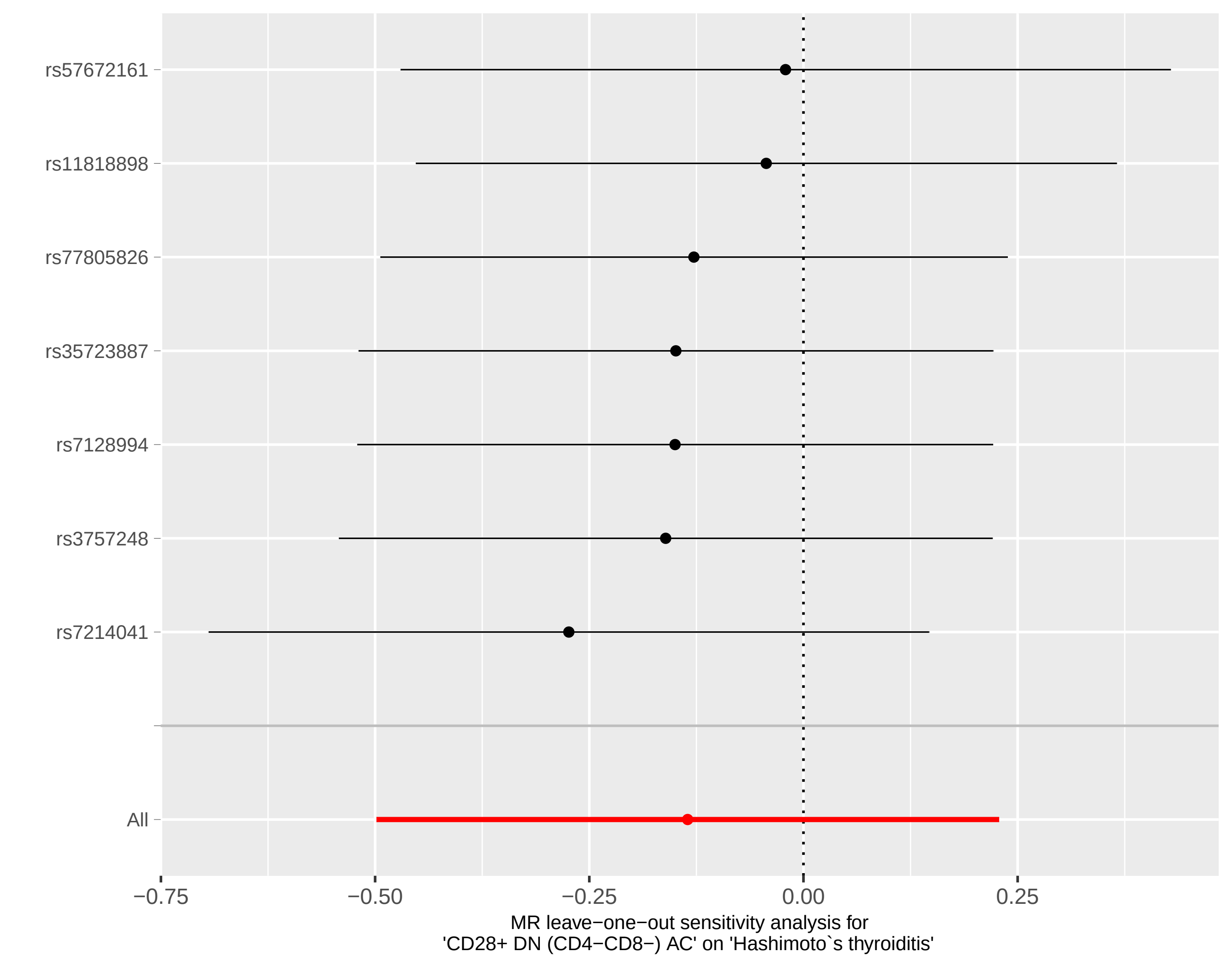
rs57672161
rs11818898
rs77805826
rs35723887
rs7128994
rs3757248
rs7214041
All
−0.75
−0.50
−0.25
0.00
0.25
MR leave−one−out sensitivity analysis for
'CD28+ DN (CD4−CD8−) AC' on 'Hashimoto`s thyroiditis'

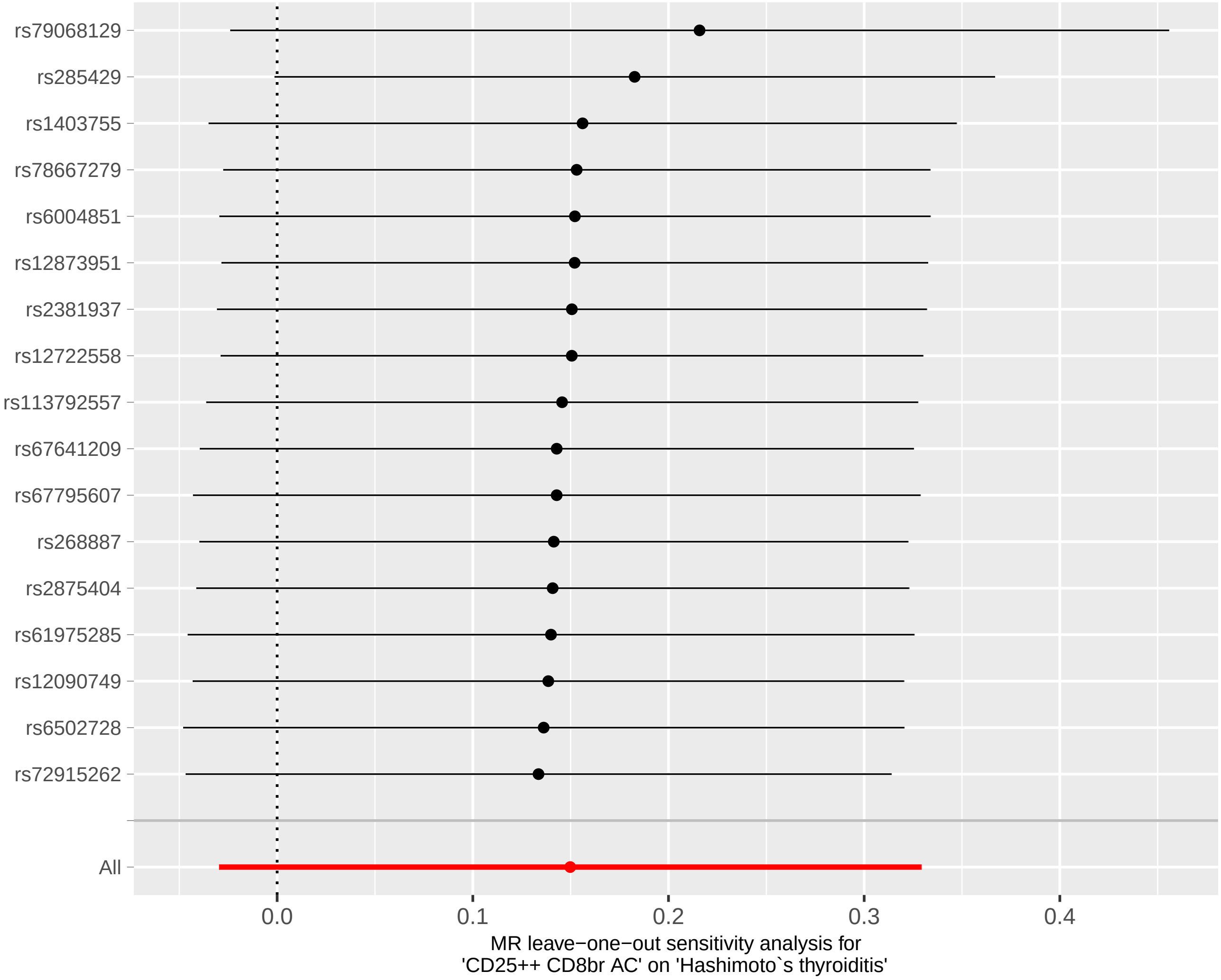

rs79068129
rs285429
rs1403755
rs78667279
rs6004851
rs12873951
rs2381937
rs12722558
rs113792557
rs67641209
rs67795607
rs268887
rs2875404
rs61975285
rs12090749
rs6502728
rs72915262
All
0.0
0.1
0.2
0.3
0.4
MR leave−one−out sensitivity analysis for
'CD25++ CD8br AC' on 'Hashimoto`s thyroiditis'

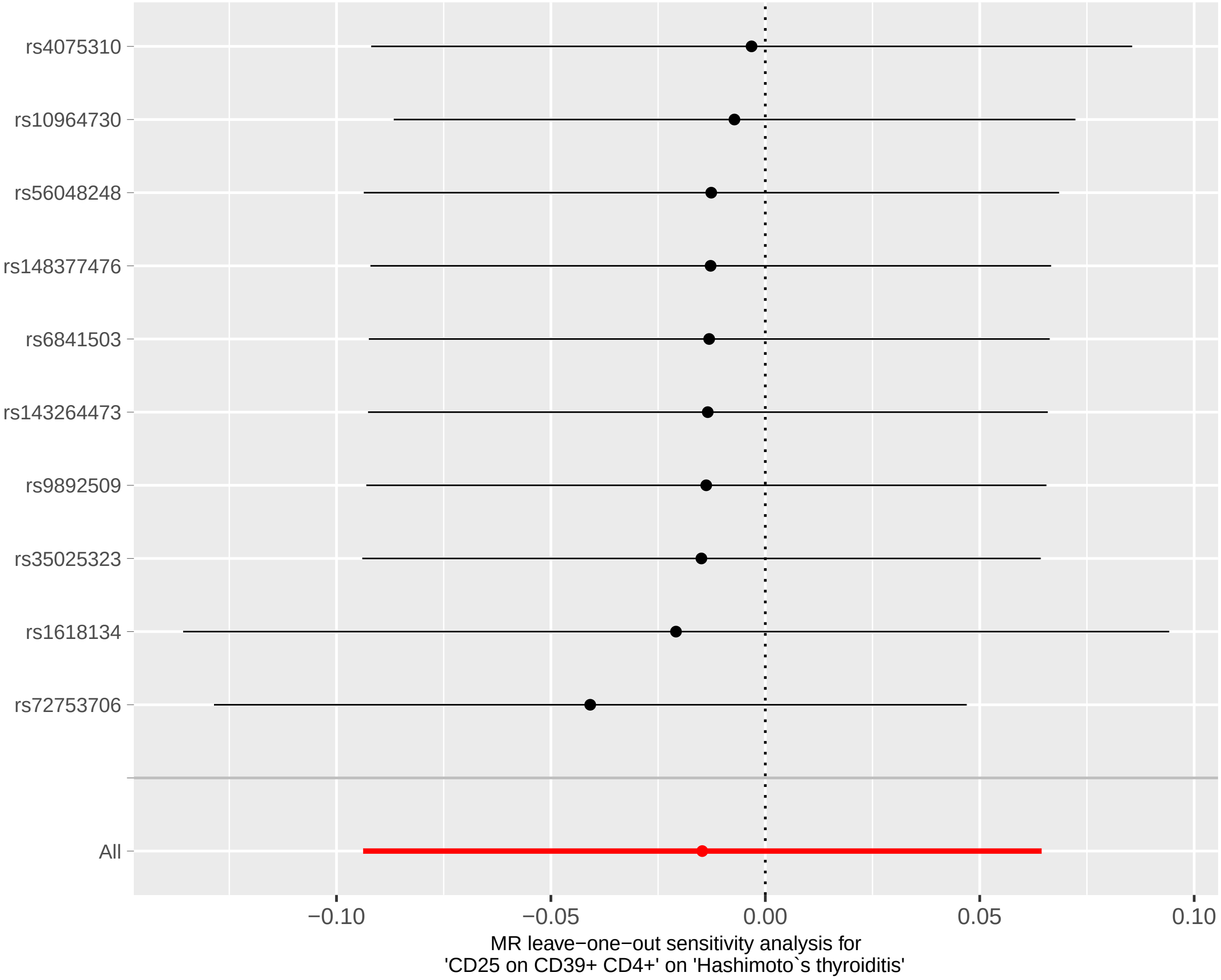
rs4075310
rs10964730
rs56048248
rs148377476
rs6841503
rs143264473
rs9892509
rs35025323
rs1618134
rs72753706
All
−0.10
−0.05
0.00
0.05
0.10
MR leave−one−out sensitivity analysis for
'CD25 on CD39+ CD4+' on 'Hashimoto`s thyroiditis'

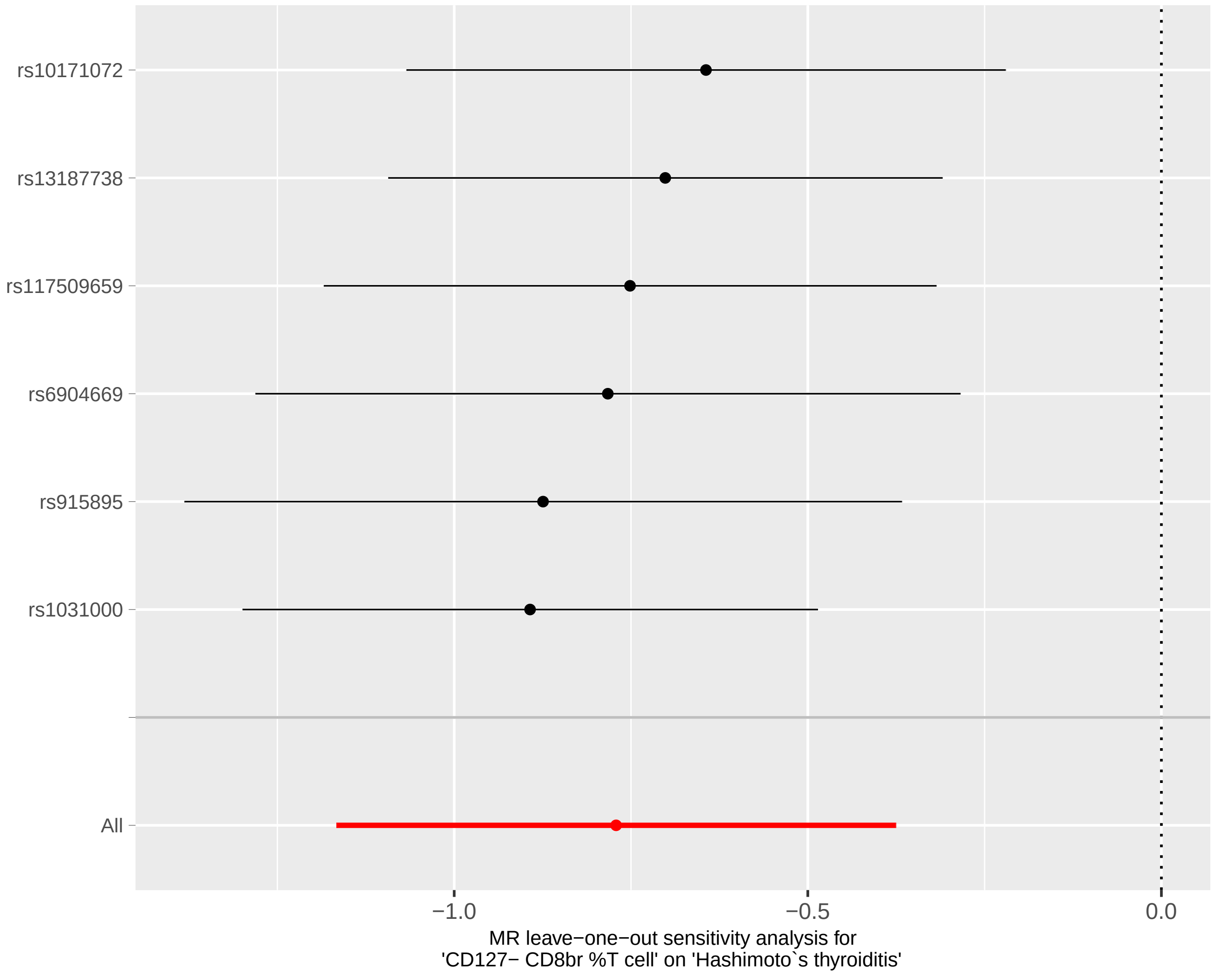
rs10171072
rs13187738
rs117509659
rs6904669
rs915895
rs1031000
All
−1.0
−0.5
0.0
MR leave−one−out sensitivity analysis for
'CD127− CD8br %T cell' on 'Hashimoto`s thyroiditis'

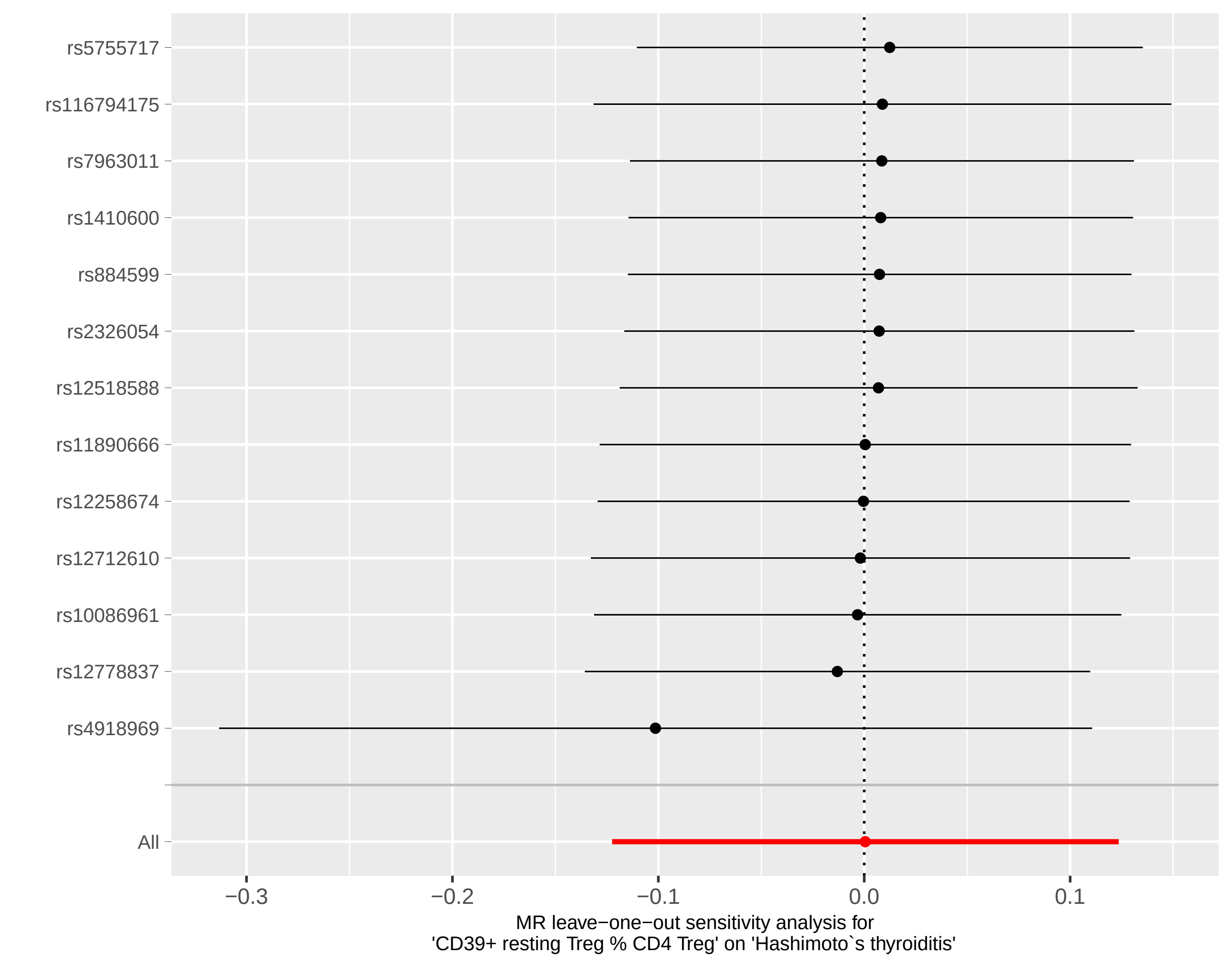
rs5755717
rs116794175
rs7963011
rs1410600
rs884599
rs2326054
rs12518588
rs11890666
rs12258674
rs12712610
rs10086961
rs12778837
rs4918969
All
−0.3
−0.2
−0.1
0.0
0.1
MR leave−one−out sensitivity analysis for
'CD39+ resting Treg % CD4 Treg' on 'Hashimoto`s thyroiditis'

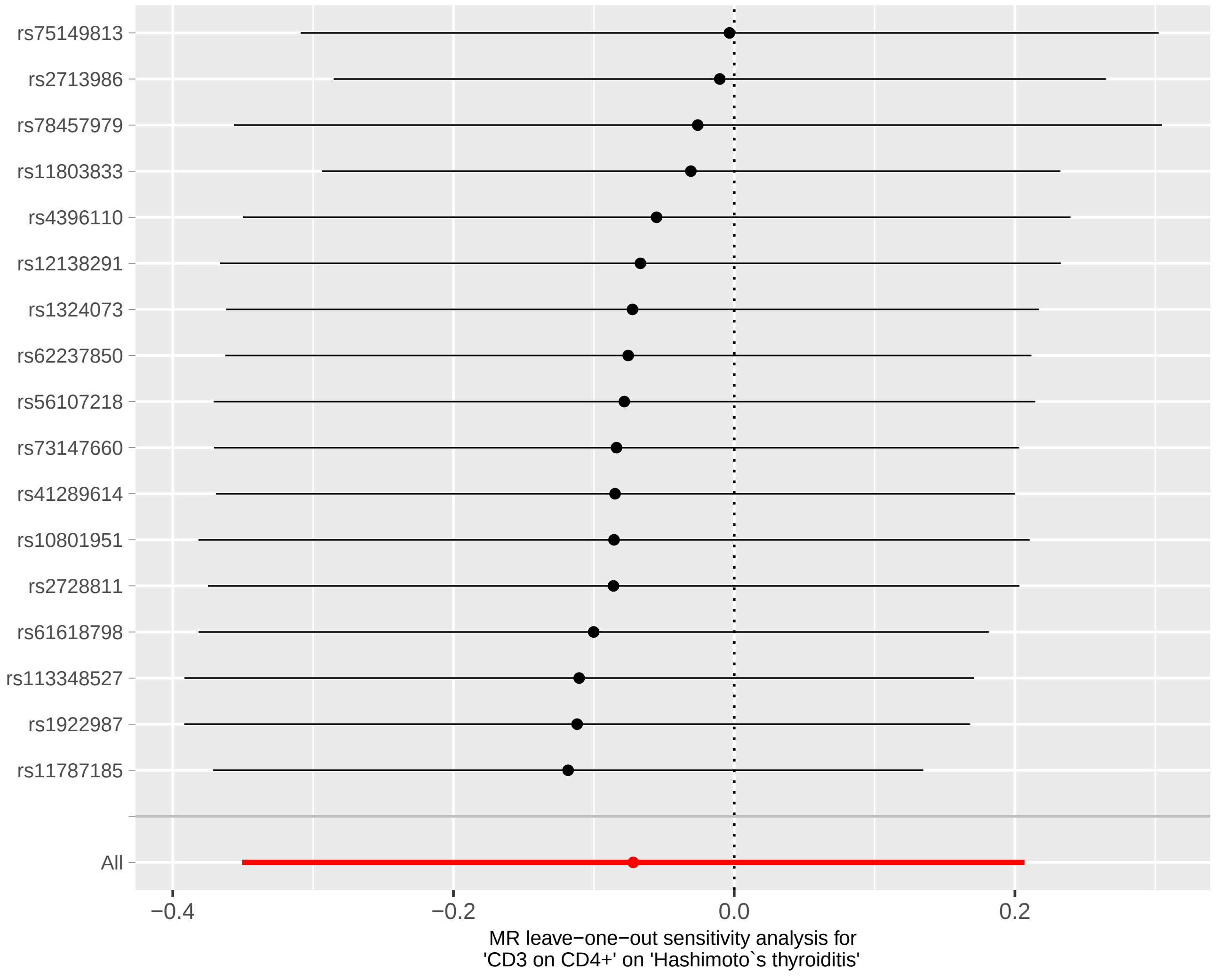
rs75149813
rs2713986
rs78457979
rs11803833
rs4396110
rs12138291
rs1324073
rs62237850
rs56107218
rs73147660
rs41289614
rs10801951
rs2728811
rs61618798
rs113348527
rs1922987
rs11787185
All
−0.4
−0.2
0.0
0.2
MR leave−one−out sensitivity analysis for
'CD3 on CD4+' on 'Hashimoto`s thyroiditis'

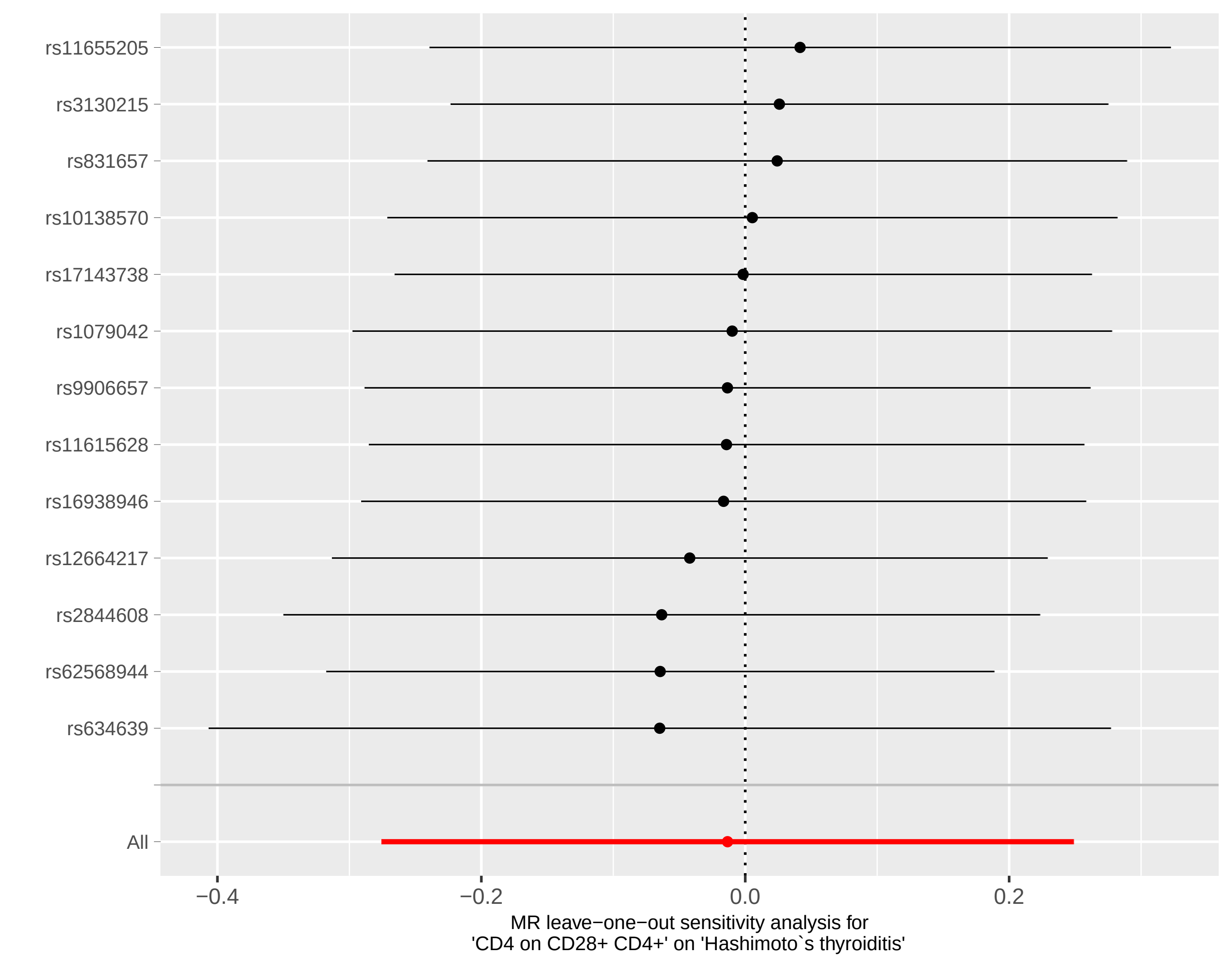
rs11655205
rs3130215
rs831657
rs10138570
rs17143738
rs1079042
rs9906657
rs11615628
rs16938946
rs12664217
rs2844608
rs62568944
rs634639
All
−0.4
−0.2
0.0
0.2
MR leave−one−out sensitivity analysis for
'CD4 on CD28+ CD4+' on 'Hashimoto`s thyroiditis'

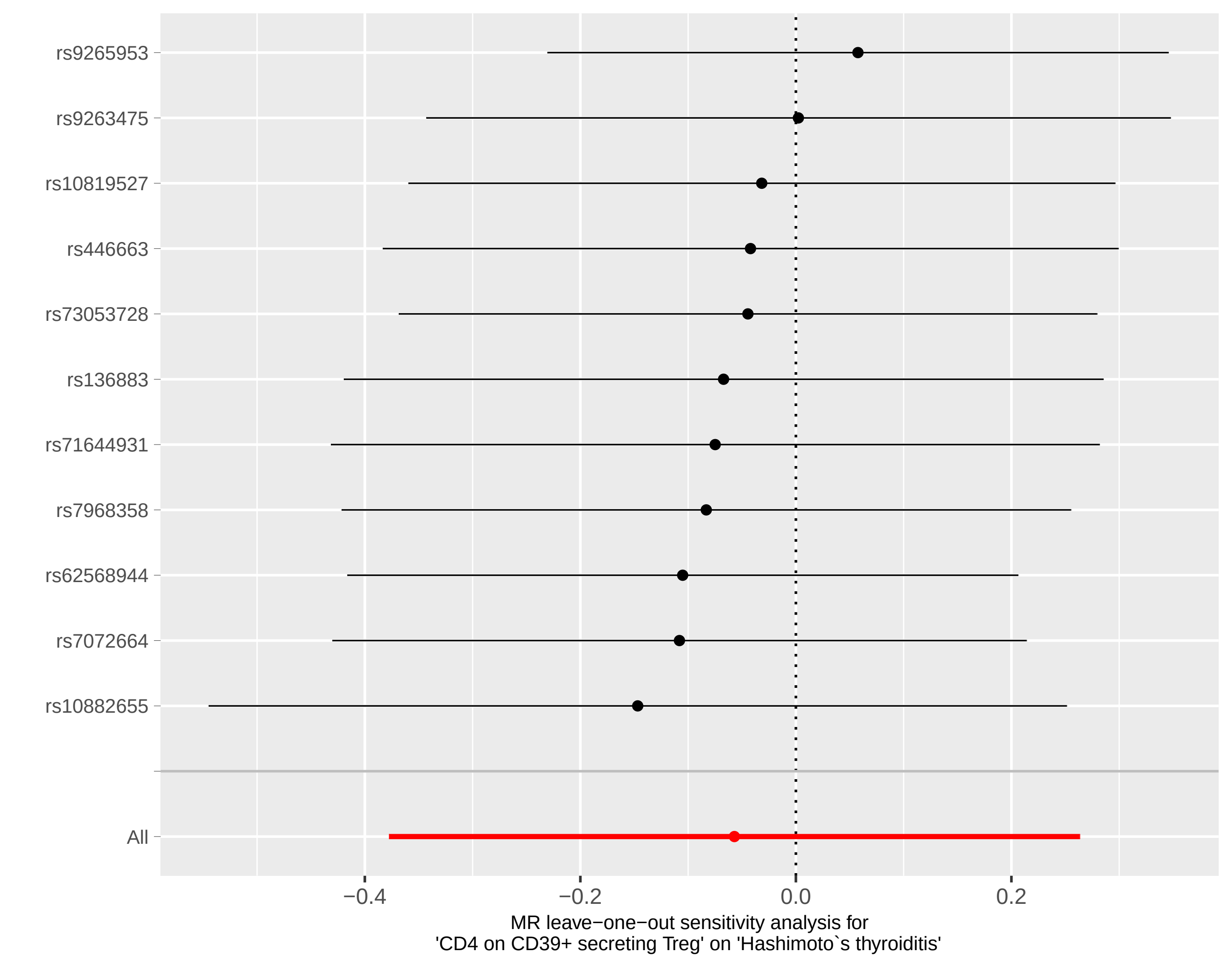
rs9265953
rs9263475
rs10819527
rs446663
rs73053728
rs136883
rs71644931
rs7968358
rs62568944
rs7072664
rs10882655
All
−0.4
−0.2
0.0
0.2
MR leave−one−out sensitivity analysis for
'CD4 on CD39+ secreting Treg' on 'Hashimoto`s thyroiditis'

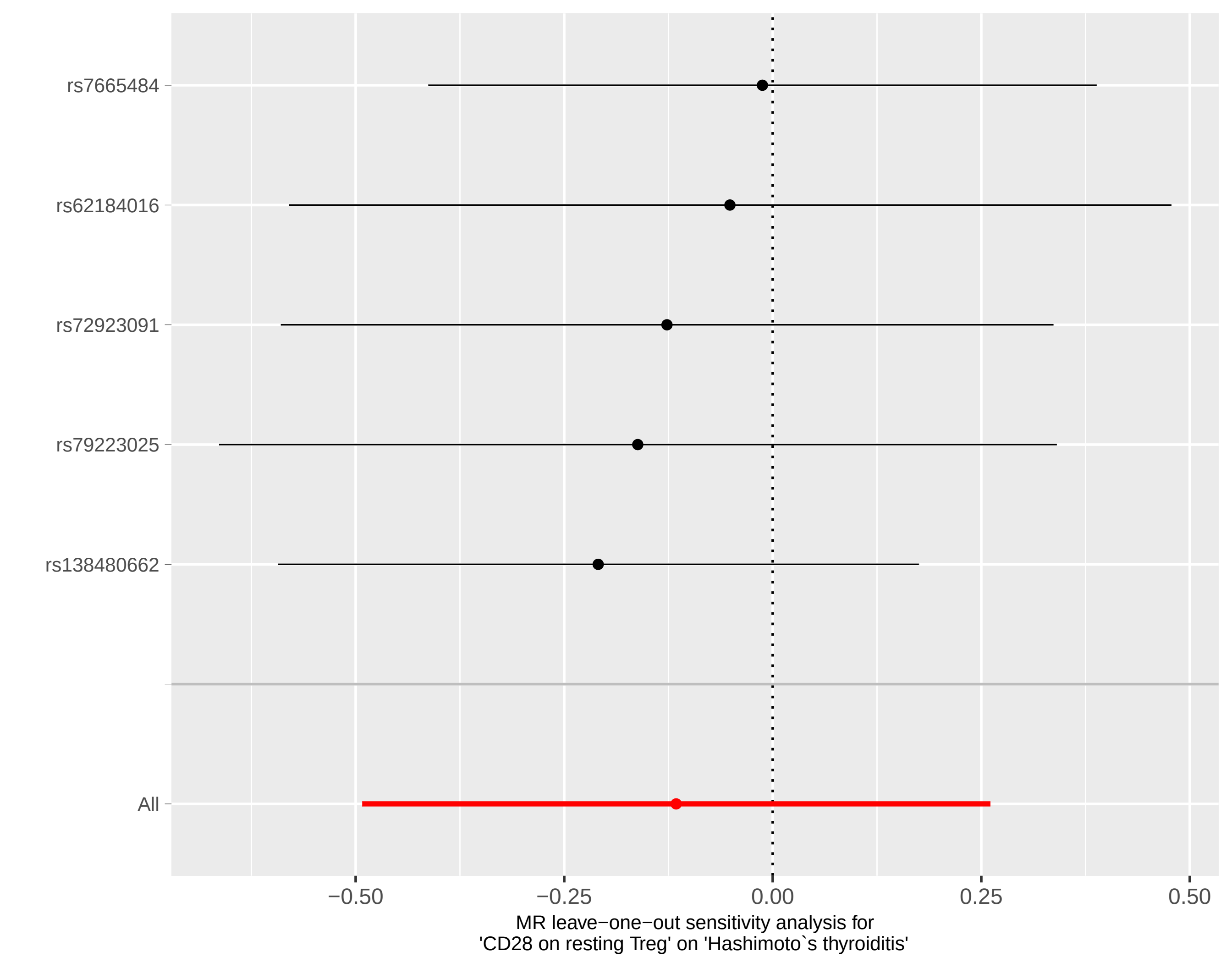
rs7665484
rs62184016
rs72923091
rs79223025
rs138480662
All
−0.50
−0.25
0.00
0.25
0.50
MR leave−one−out sensitivity analysis for
'CD28 on resting Treg' on 'Hashimoto`s thyroiditis'

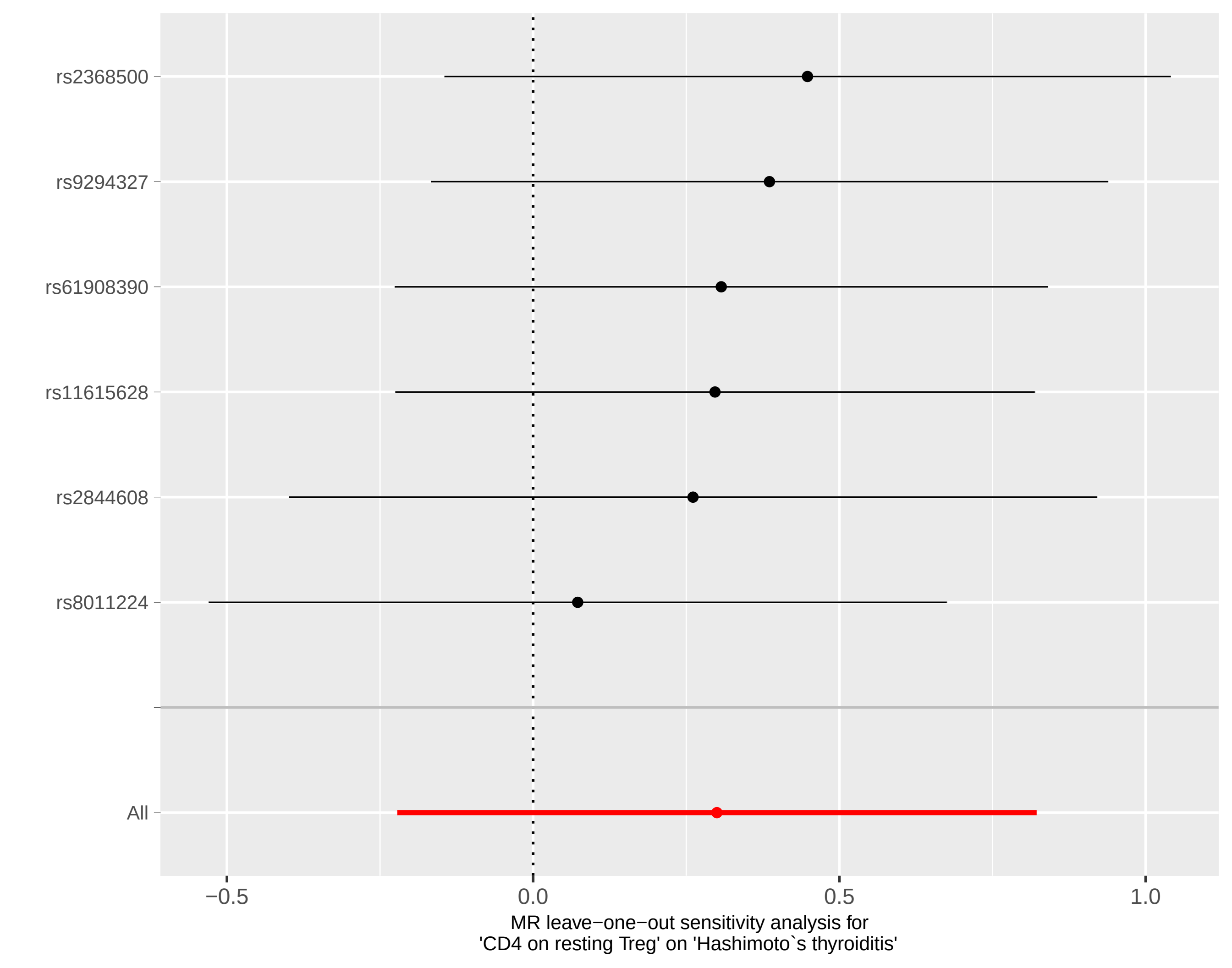
rs2368500
rs9294327
rs61908390
rs11615628
rs2844608
rs8011224
All
−0.5
0.0
0.5
1.0
MR leave−one−out sensitivity analysis for
'CD4 on resting Treg' on 'Hashimoto`s thyroiditis'

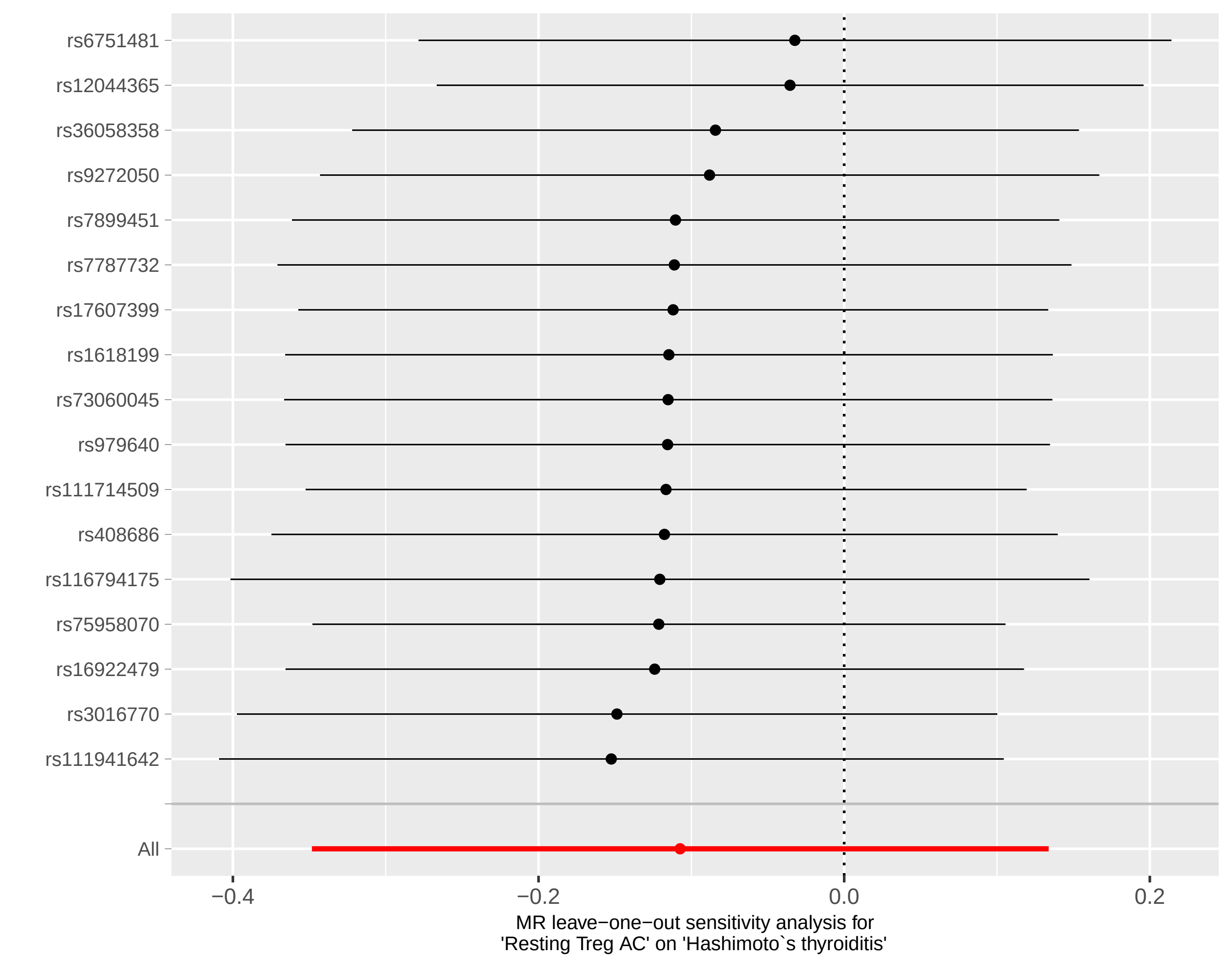

rs6751481
rs12044365
rs36058358
rs9272050
rs7899451
rs7787732
rs17607399
rs1618199
rs73060045
rs979640
rs111714509
rs408686
rs116794175
rs75958070
rs16922479
rs3016770
rs111941642
All
−0.4
−0.2
0.0
0.2
MR leave−one−out sensitivity analysis for
'Resting Treg AC' on 'Hashimoto`s thyroiditis'

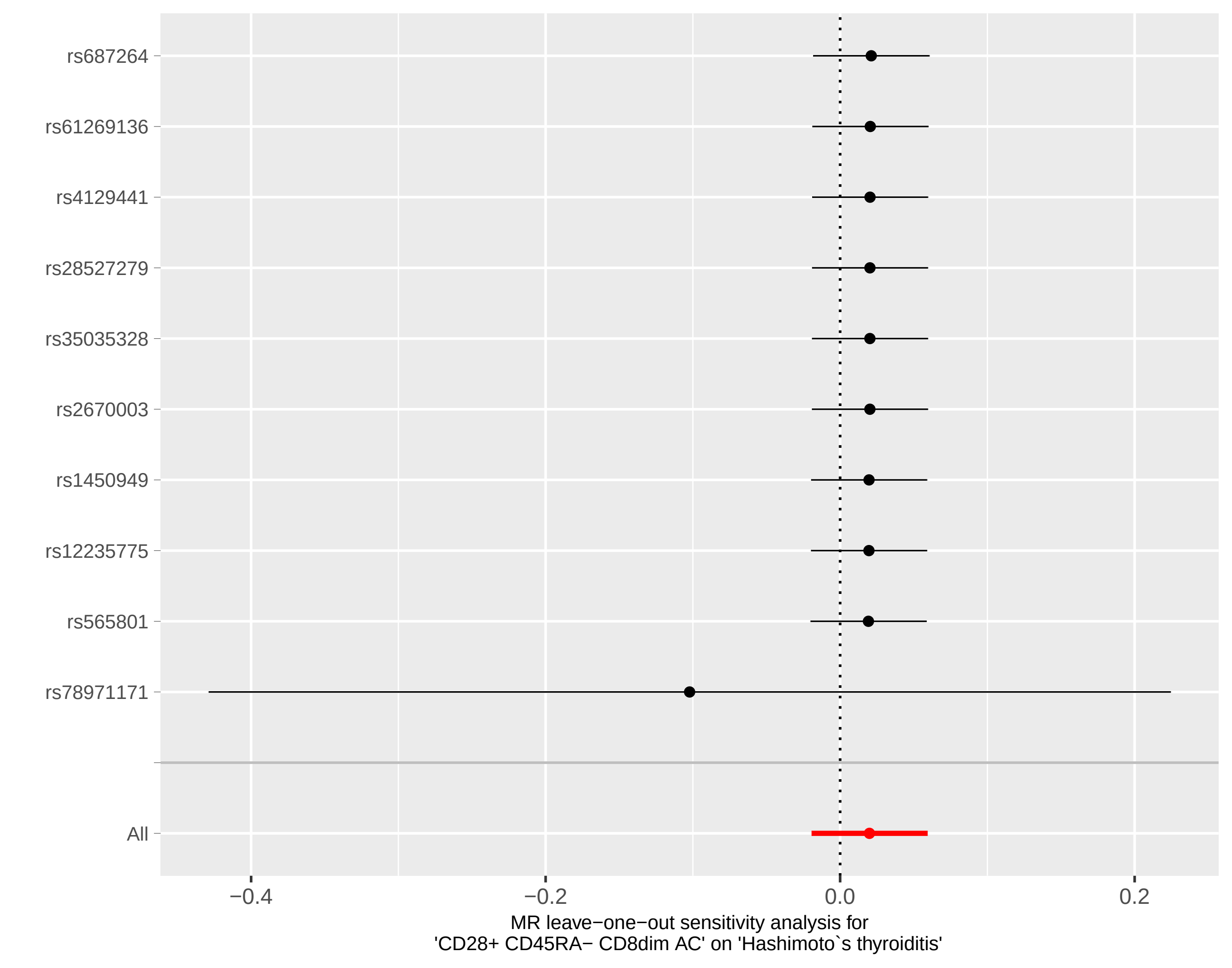
rs687264
rs61269136
rs4129441
rs28527279
rs35035328
rs2670003
rs1450949
rs12235775
rs565801
rs78971171
All
−0.4
−0.2
0.0
0.2
MR leave−one−out sensitivity analysis for
'CD28+ CD45RA− CD8dim AC' on 'Hashimoto`s thyroiditis'

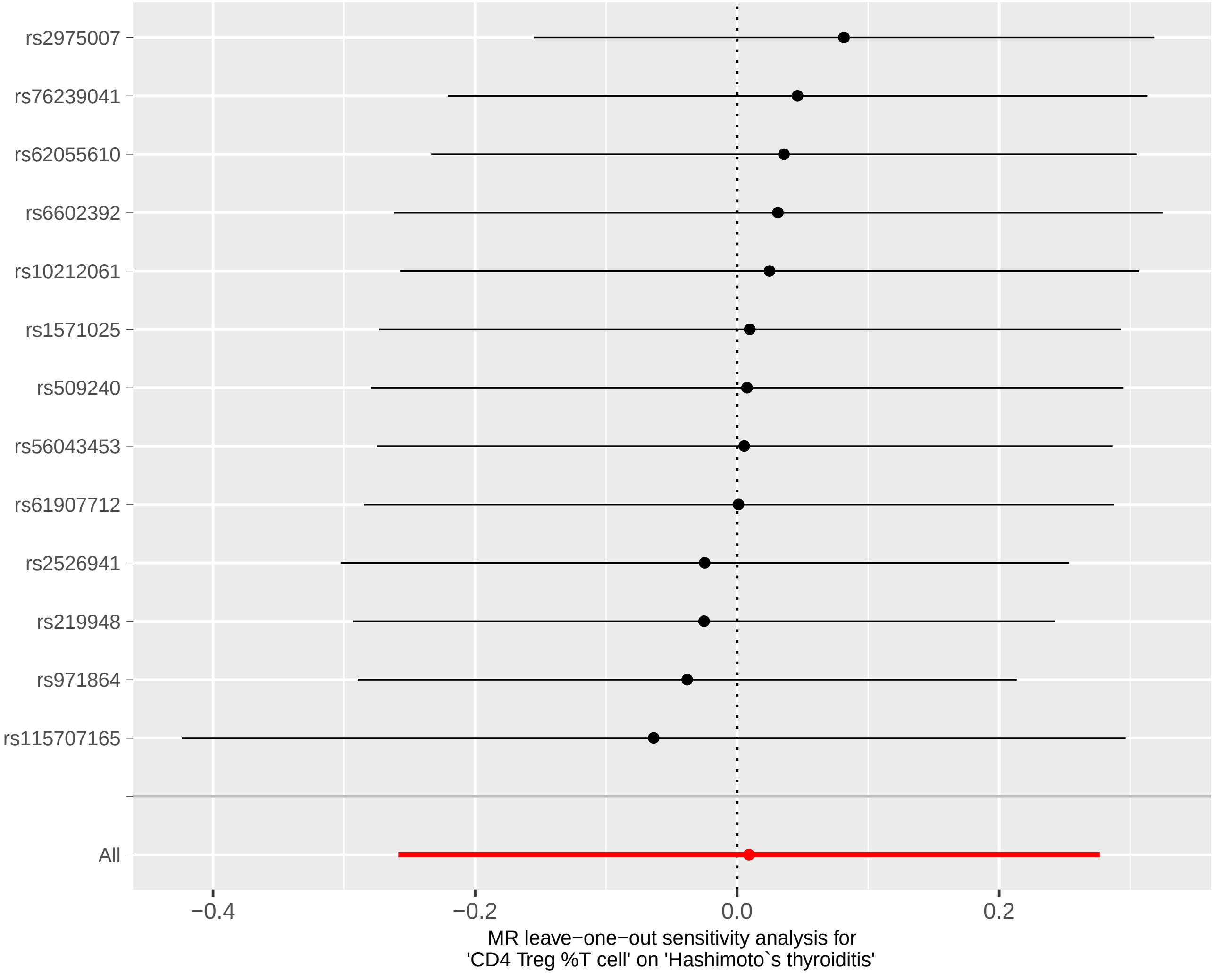

rs2975007
rs76239041
rs62055610
rs6602392
rs10212061
rs1571025
rs509240
rs56043453
rs61907712
rs2526941
rs219948
rs971864
rs115707165
All
−0.4
−0.2
0.0
0.2
MR leave−one−out sensitivity analysis for
'CD4 Treg %T cell' on 'Hashimoto`s thyroiditis'

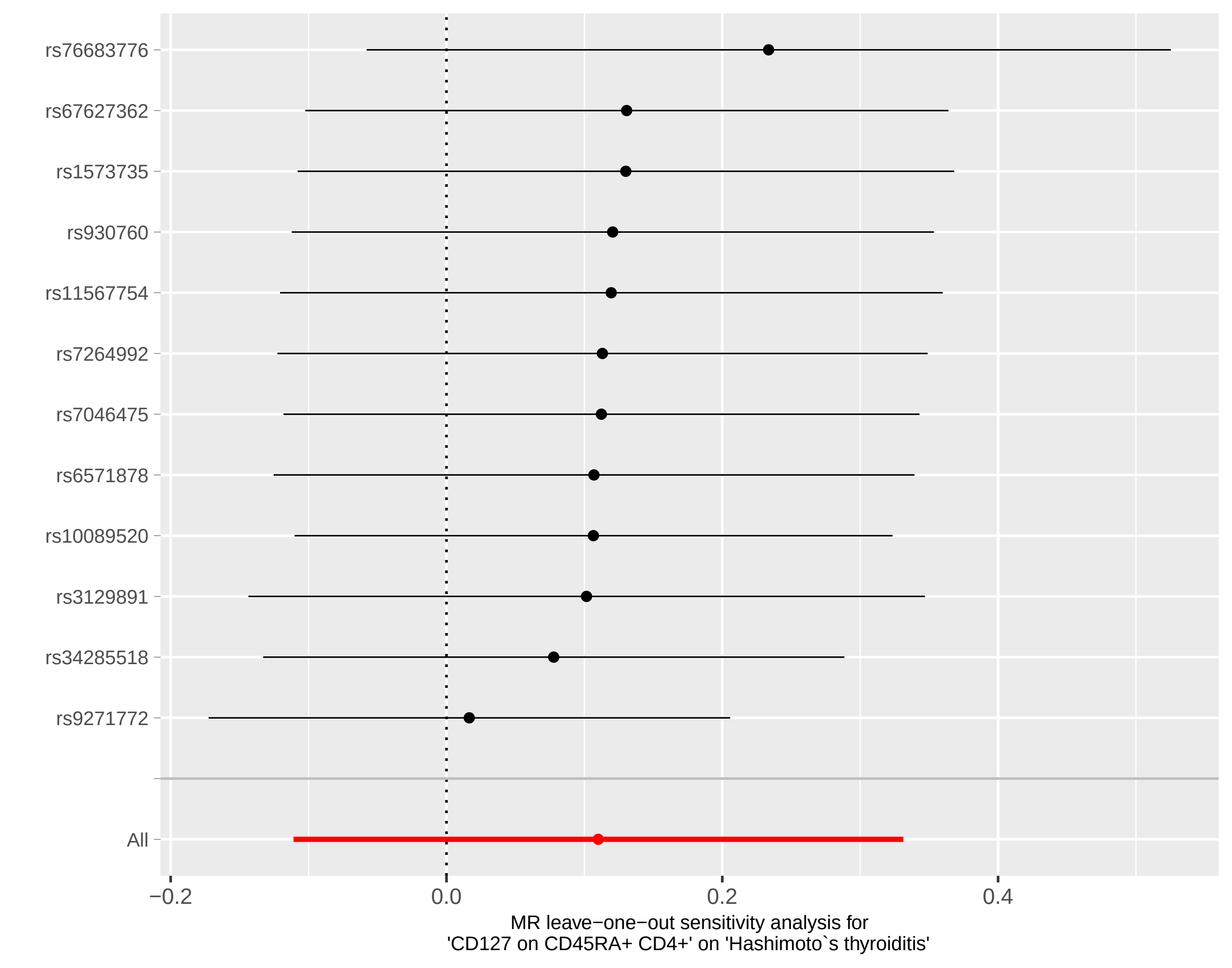

rs76683776
rs67627362
rs1573735
rs930760
rs11567754
rs7264992
rs7046475
rs6571878
rs10089520
rs3129891
rs34285518
rs9271772
All
−0.2
0.0
0.2
0.4
MR leave−one−out sensitivity analysis for
'CD127 on CD45RA+ CD4+' on 'Hashimoto`s thyroiditis'

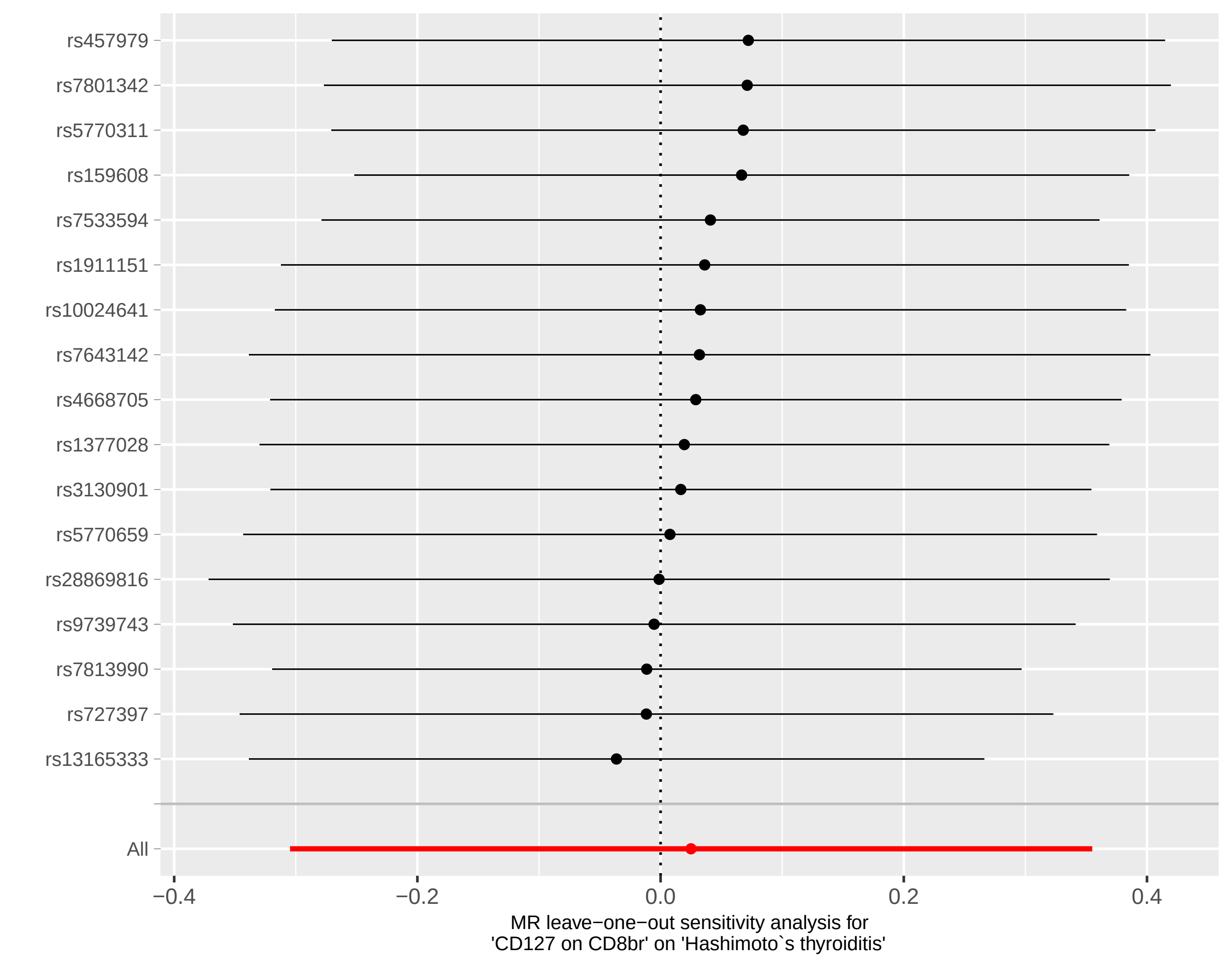
rs457979
rs7801342
rs5770311
rs159608
rs7533594
rs1911151
rs10024641
rs7643142
rs4668705
rs1377028
rs3130901
rs5770659
rs28869816
rs9739743
rs7813990
rs727397
rs13165333
All
−0.4
−0.2
0.0
0.2
0.4
MR leave−one−out sensitivity analysis for
'CD127 on CD8br' on 'Hashimoto`s thyroiditis'

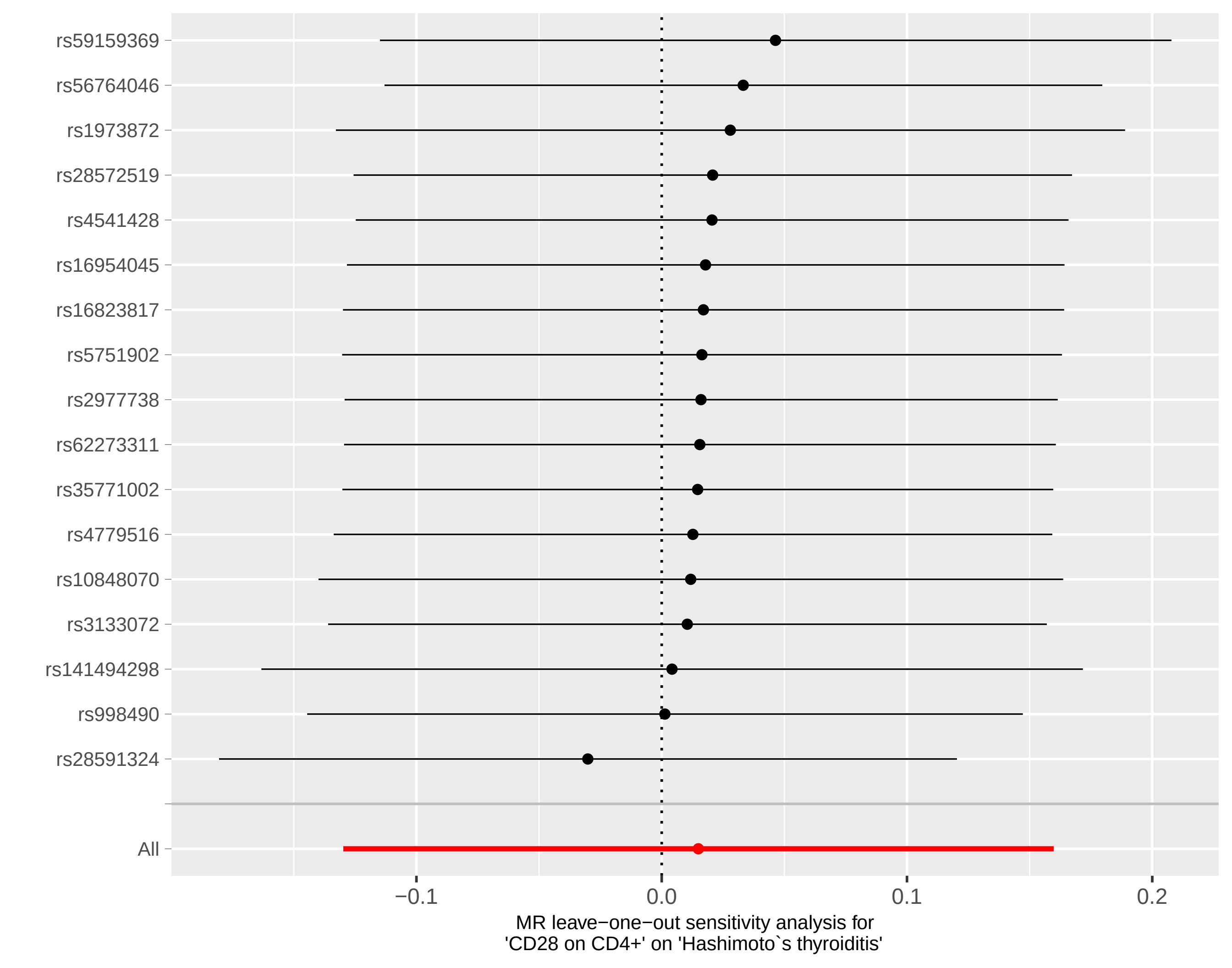
rs59159369
rs56764046
rs1973872
rs28572519
rs4541428
rs16954045
rs16823817
rs5751902
rs2977738
rs62273311
rs35771002
rs4779516
rs10848070
rs3133072
rs141494298
rs998490
rs28591324
All
−0.1
0.0
0.1
0.2
MR leave−one−out sensitivity analysis for
'CD28 on CD4+' on 'Hashimoto`s thyroiditis'

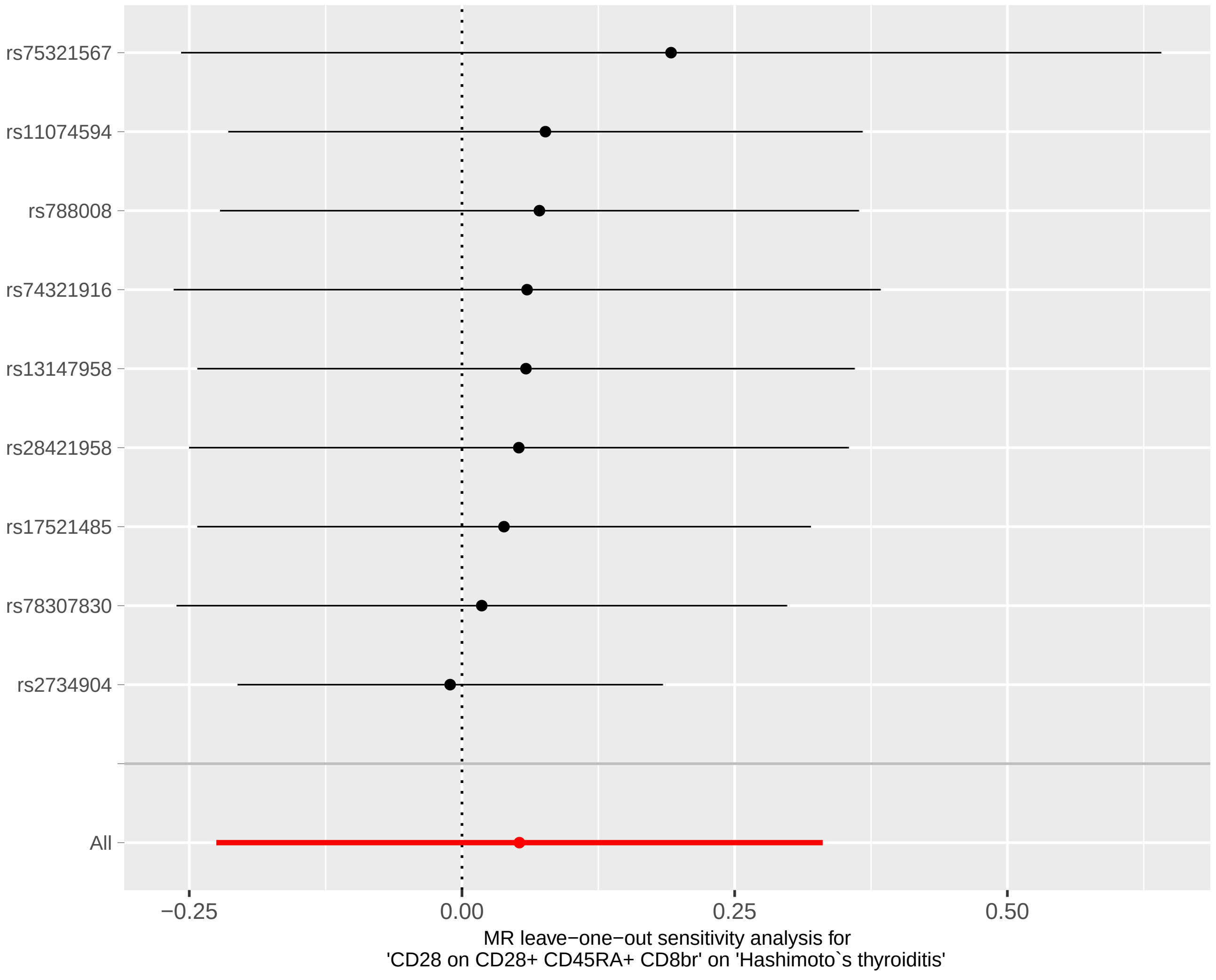

rs75321567
rs11074594
rs788008
rs74321916
rs13147958
rs28421958
rs17521485
rs78307830
rs2734904
All
−0.25
0.00
0.25
0.50
MR leave−one−out sensitivity analysis for
'CD28 on CD28+ CD45RA+ CD8br' on 'Hashimoto`s thyroiditis'

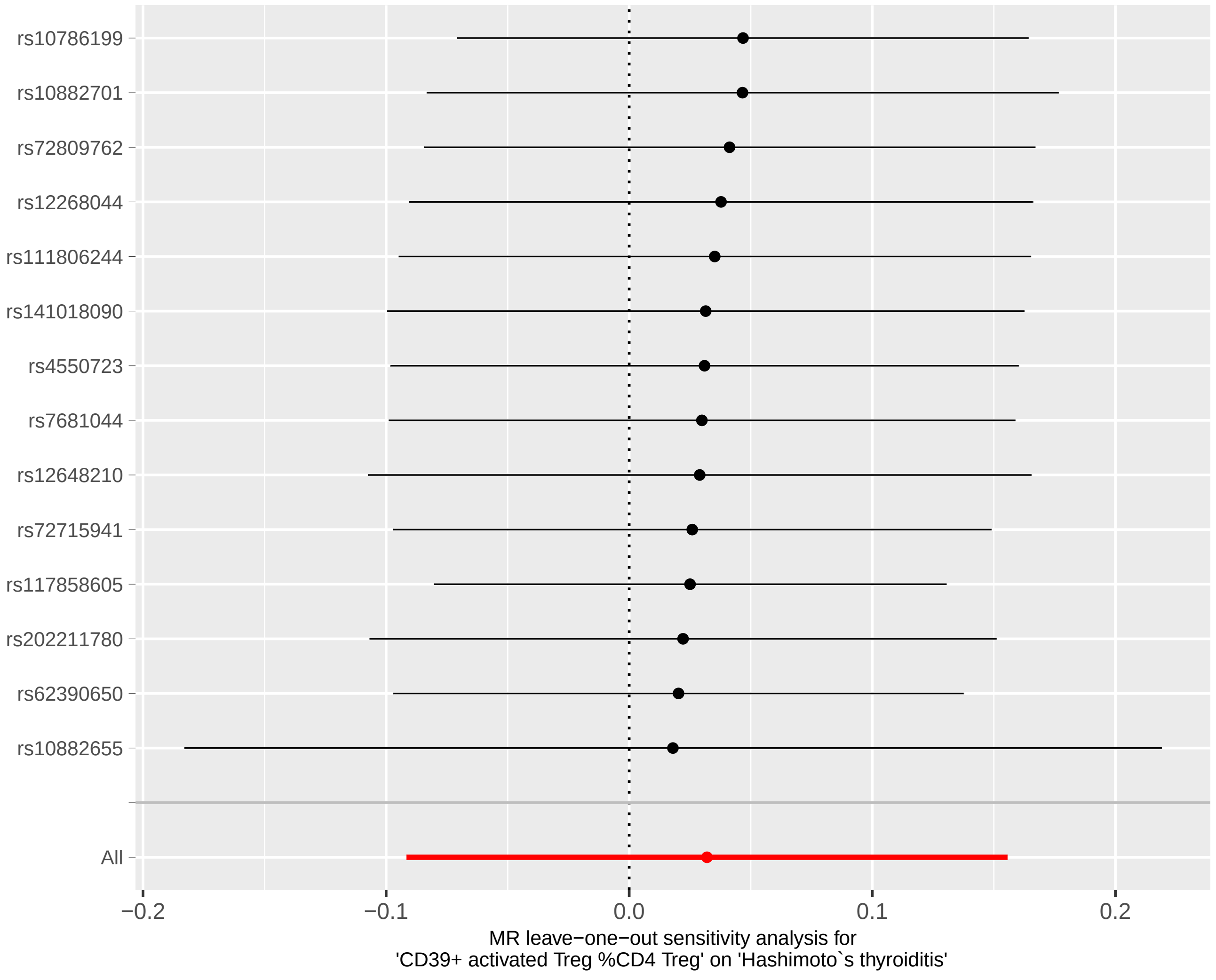

rs10786199
rs10882701
rs72809762
rs12268044
rs111806244
rs141018090
rs4550723
rs7681044
rs12648210
rs72715941
rs117858605
rs202211780
rs62390650
rs10882655
All
−0.2
−0.1
0.0
0.1
0.2
MR leave−one−out sensitivity analysis for
'CD39+ activated Treg %CD4 Treg' on 'Hashimoto`s thyroiditis'

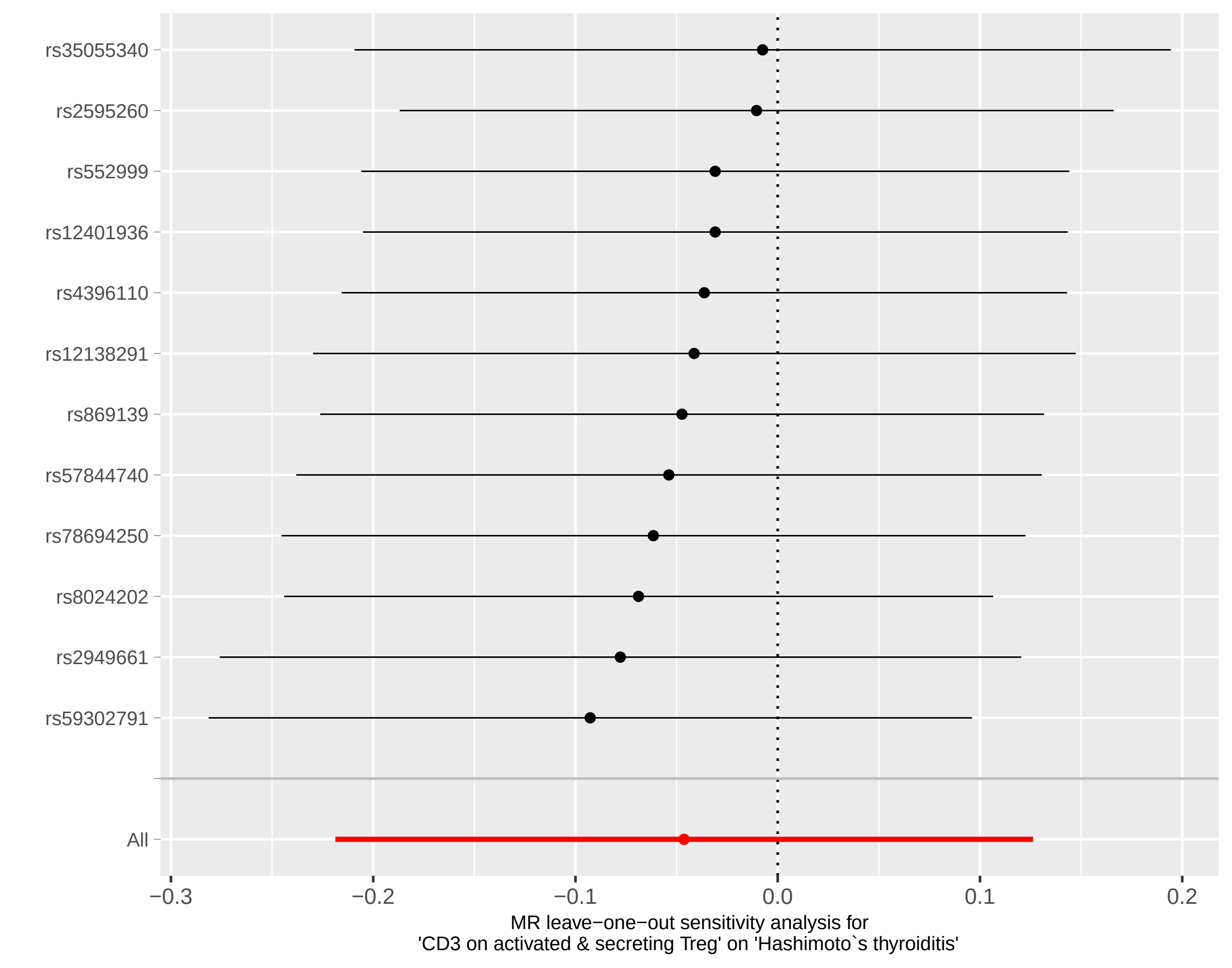

rs35055340
rs2595260
rs552999
rs12401936
rs4396110
rs12138291
rs869139
rs57844740
rs78694250
rs8024202
rs2949661
rs59302791
All
−0.3
−0.2
−0.1
0.0
0.1
0.2
MR leave−one−out sensitivity analysis for
'CD3 on activated & secreting Treg' on 'Hashimoto`s thyroiditis'

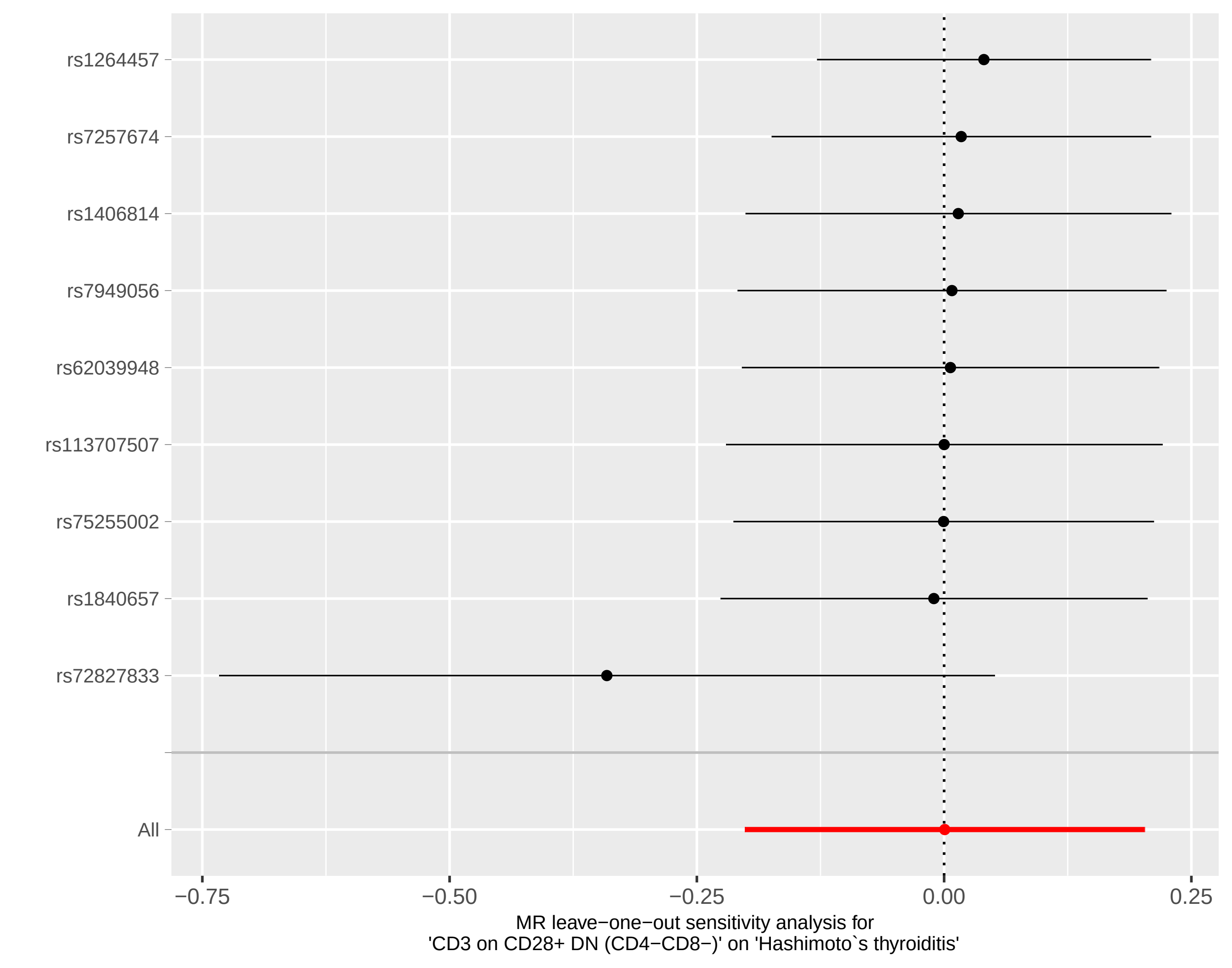
rs1264457
rs7257674
rs1406814
rs7949056
rs62039948
rs113707507
rs75255002
rs1840657
rs72827833
All
−0.75
−0.50
−0.25
0.00
0.25
MR leave−one−out sensitivity analysis for
'CD3 on CD28+ DN (CD4−CD8−)' on 'Hashimoto`s thyroiditis'

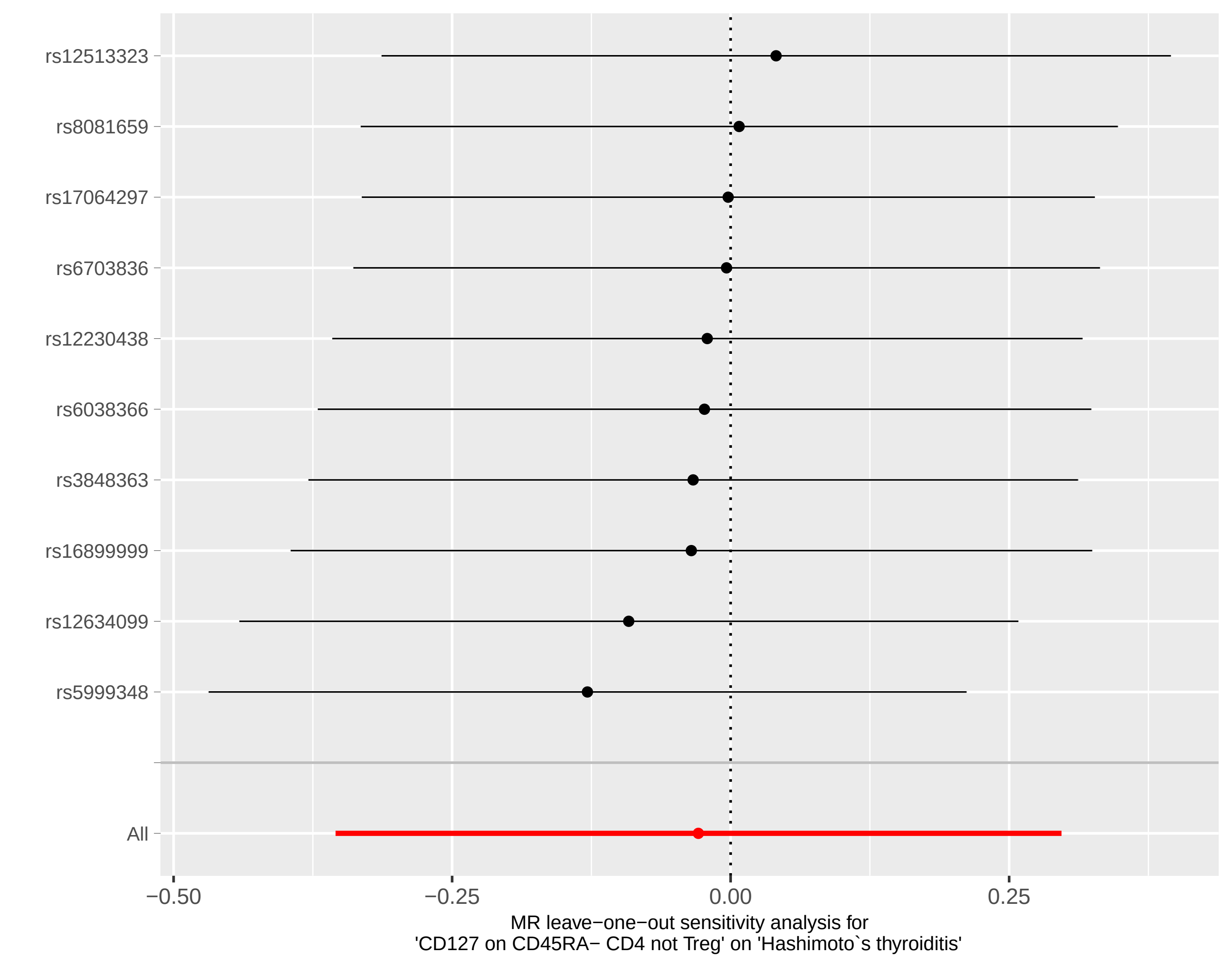
rs12513323
rs8081659
rs17064297
rs6703836
rs12230438
rs6038366
rs3848363
rs16899999
rs12634099
rs5999348
All
−0.50
−0.25
0.00
0.25
MR leave−one−out sensitivity analysis for
'CD127 on CD45RA− CD4 not Treg' on 'Hashimoto`s thyroiditis'

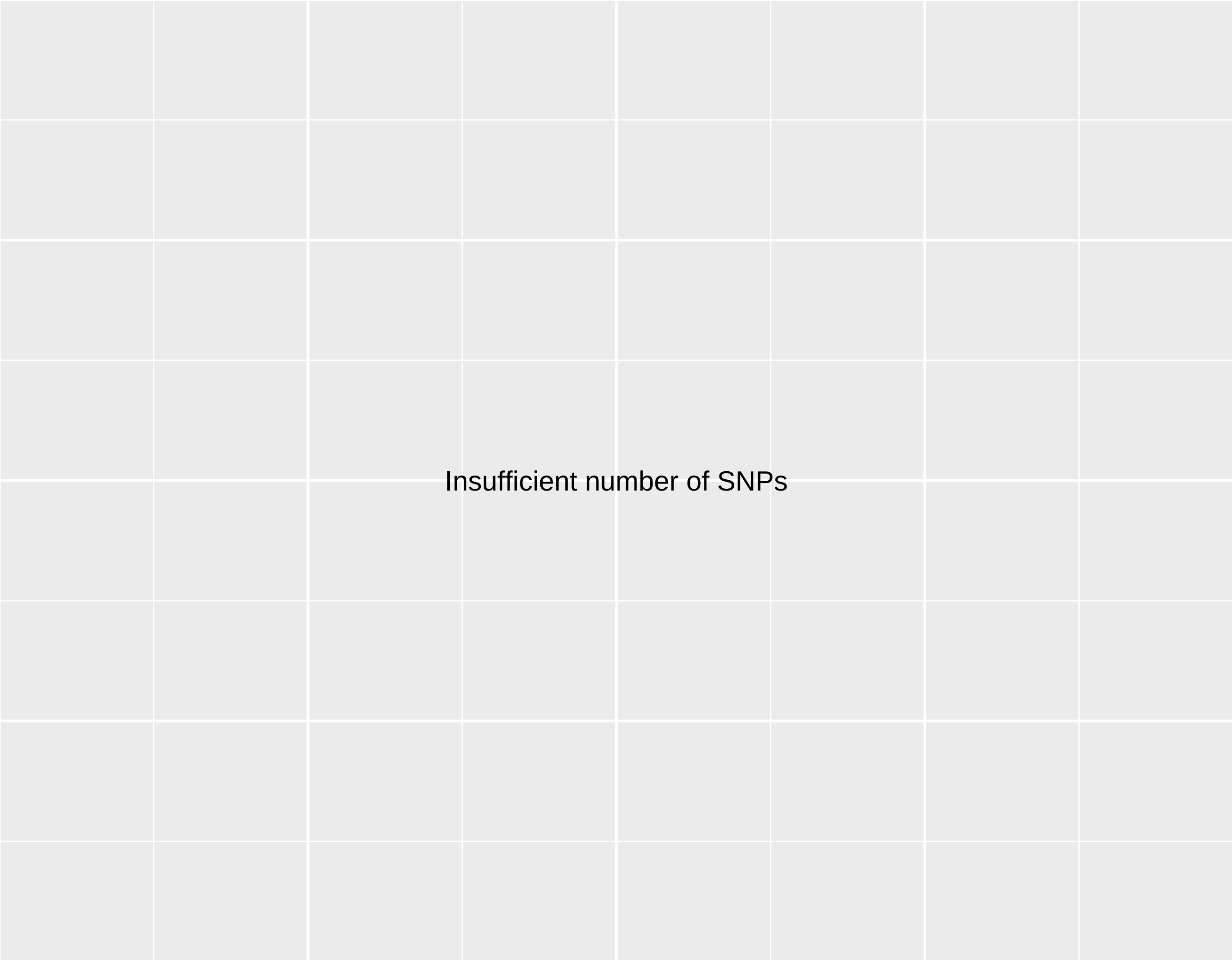
Insufficient number of SNPs

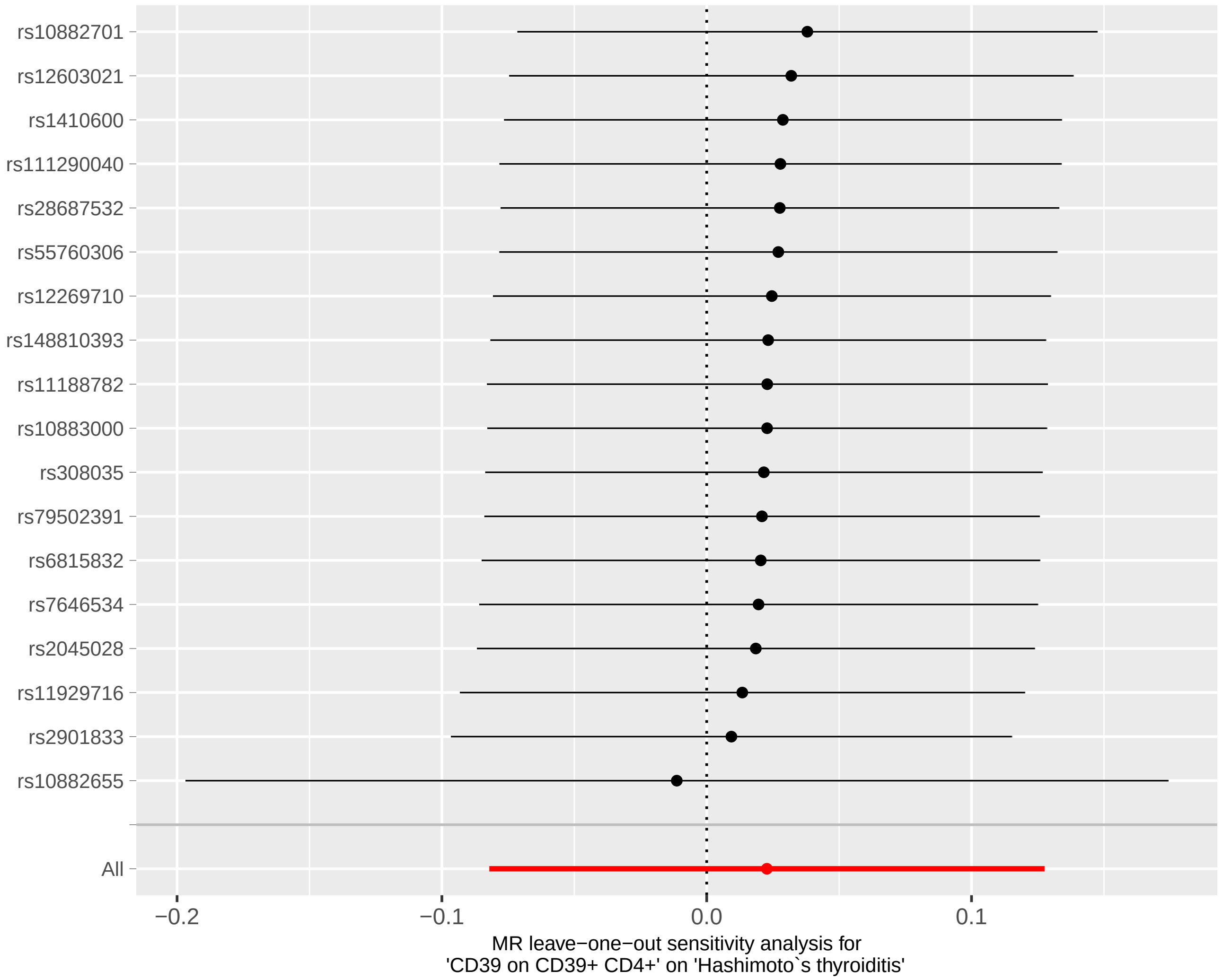
rs10882701
rs12603021
rs1410600
rs111290040
rs28687532
rs55760306
rs12269710
rs148810393
rs11188782
rs10883000
rs308035
rs79502391
rs6815832
rs7646534
rs2045028
rs11929716
rs2901833
rs10882655
All
−0.2
−0.1
0.0
0.1
MR leave−one−out sensitivity analysis for
'CD39 on CD39+ CD4+' on 'Hashimoto`s thyroiditis'

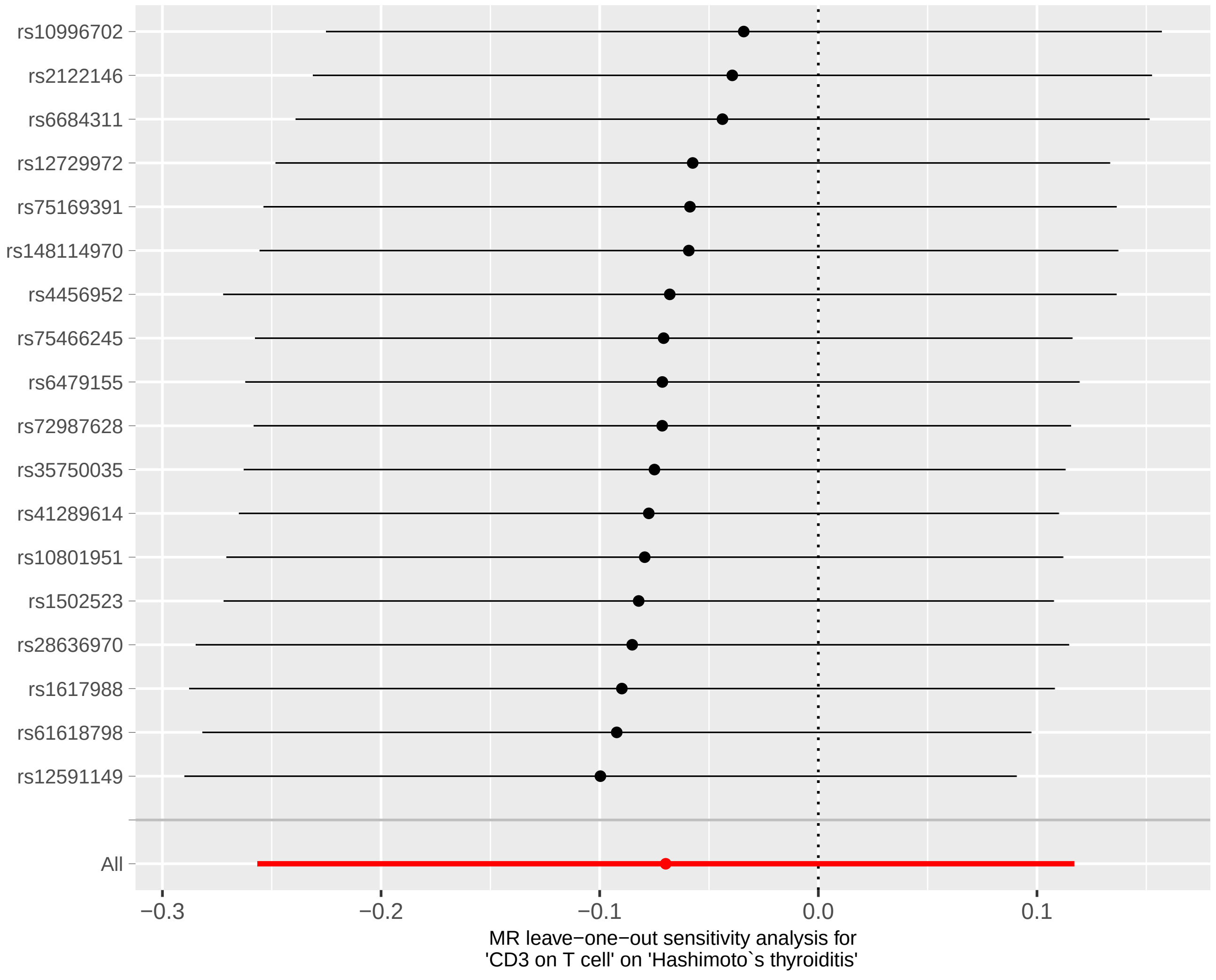

rs10996702
rs2122146
rs6684311
rs12729972
rs75169391
rs148114970
rs4456952
rs75466245
rs6479155
rs72987628
rs35750035
rs41289614
rs10801951
rs1502523
rs28636970
rs1617988
rs61618798
rs12591149
All
−0.3
−0.2
−0.1
0.0
0.1
MR leave−one−out sensitivity analysis for
'CD3 on T cell' on 'Hashimoto`s thyroiditis'

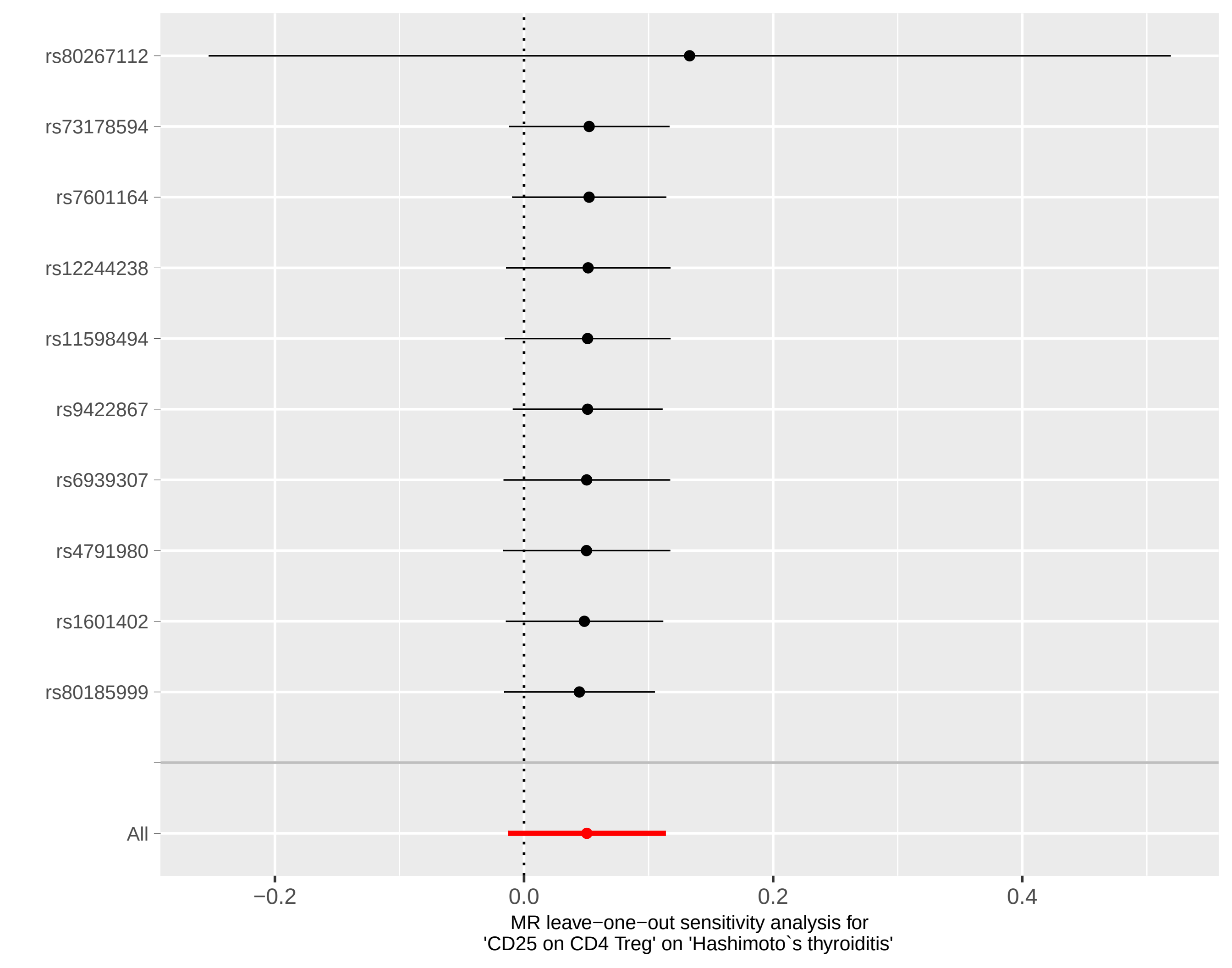
rs80267112
rs73178594
rs7601164
rs12244238
rs11598494
rs9422867
rs6939307
rs4791980
rs1601402
rs80185999
All
−0.2
0.0
0.2
0.4
MR leave−one−out sensitivity analysis for
'CD25 on CD4 Treg' on 'Hashimoto`s thyroiditis'

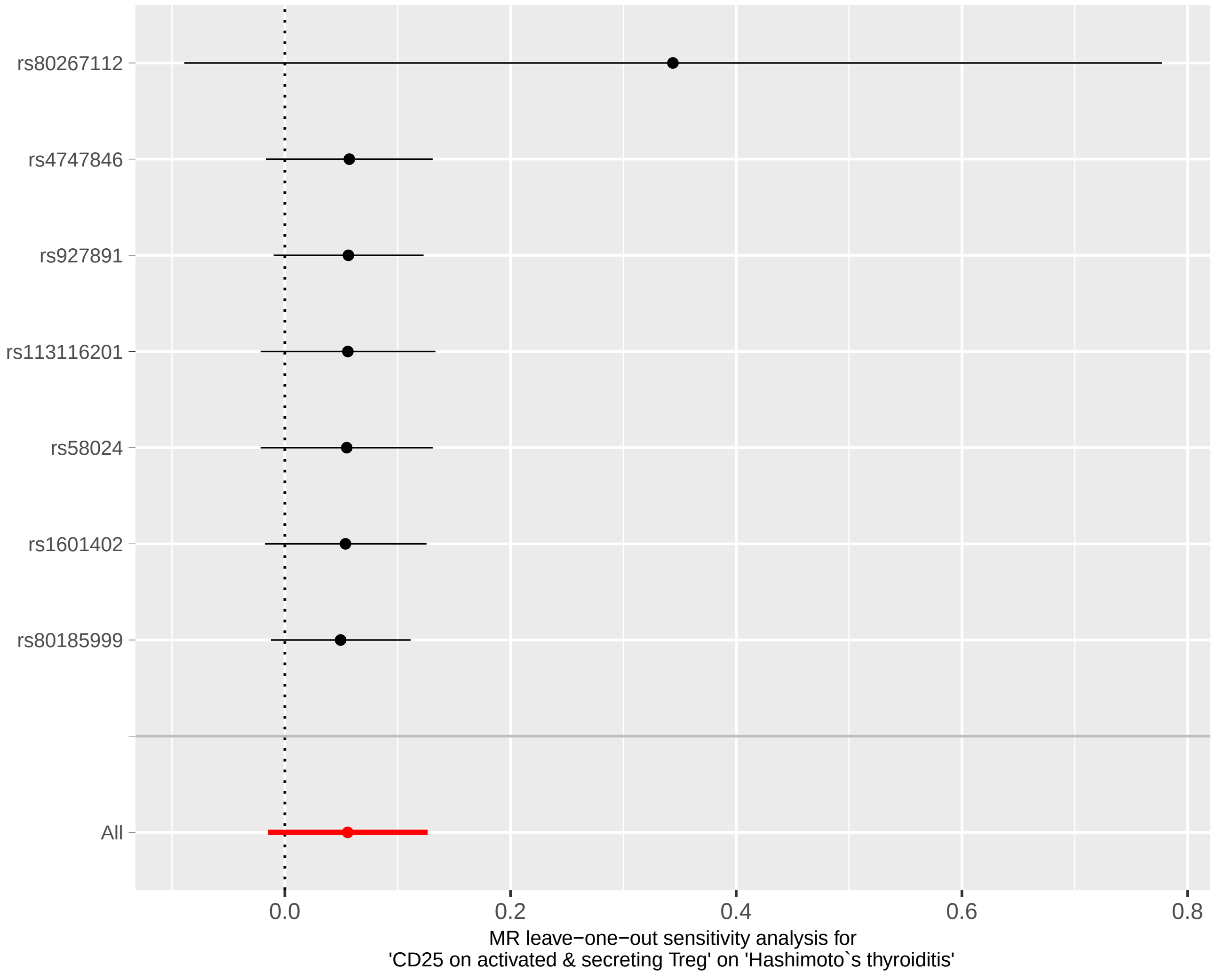
rs80267112
rs4747846
rs927891
rs113116201
rs58024
rs1601402
rs80185999
All
0.0
0.2
0.4
0.6
0.8
MR leave−one−out sensitivity analysis for
'CD25 on activated & secreting Treg' on 'Hashimoto`s thyroiditis'

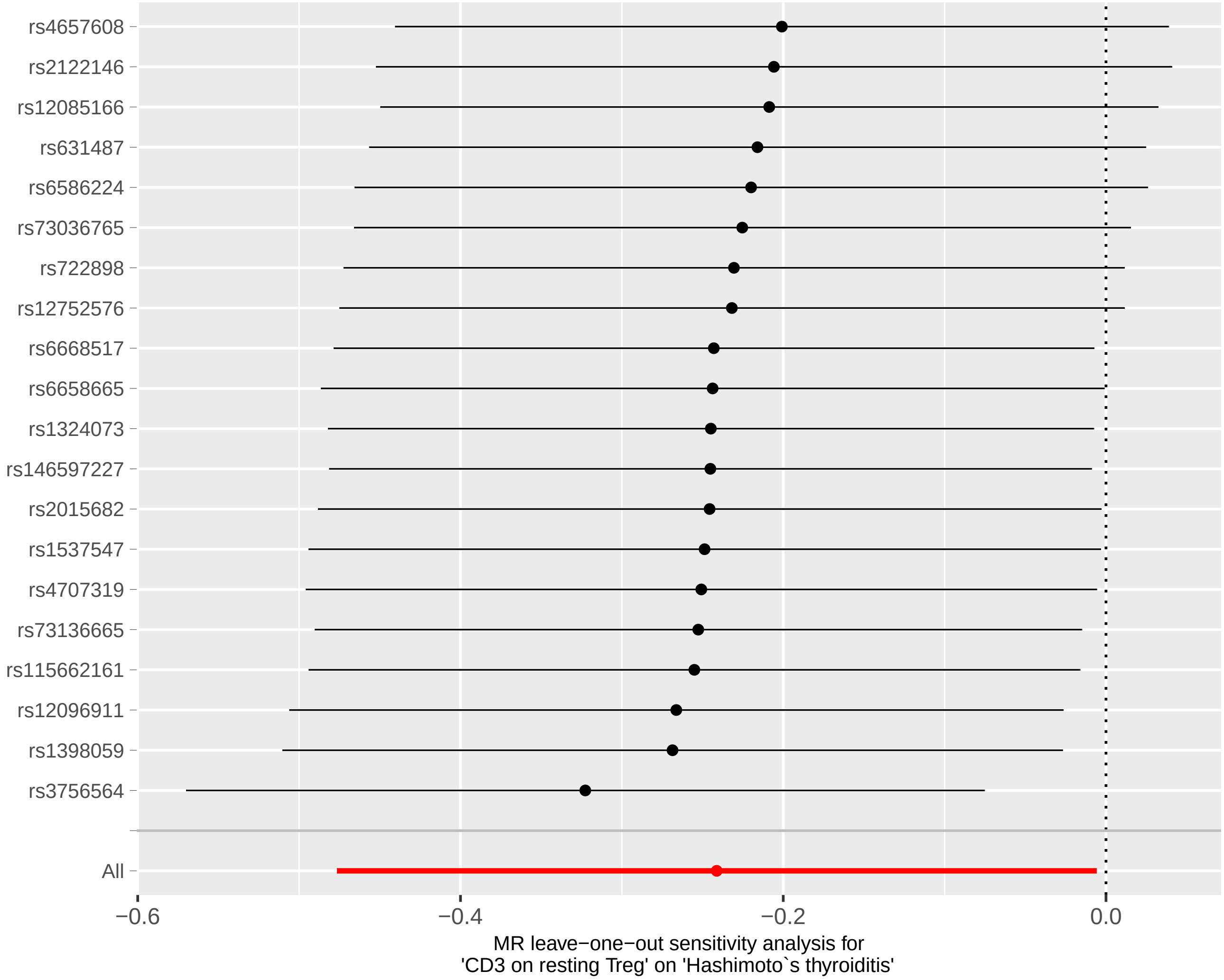

rs4657608
rs2122146
rs12085166
rs631487
rs6586224
rs73036765
rs722898
rs12752576
rs6668517
rs6658665
rs1324073
rs146597227
rs2015682
rs1537547
rs4707319
rs73136665
rs115662161
rs12096911
rs1398059
rs3756564
All
−0.6
−0.4
−0.2
0.0
MR leave−one−out sensitivity analysis for
'CD3 on resting Treg' on 'Hashimoto`s thyroiditis'

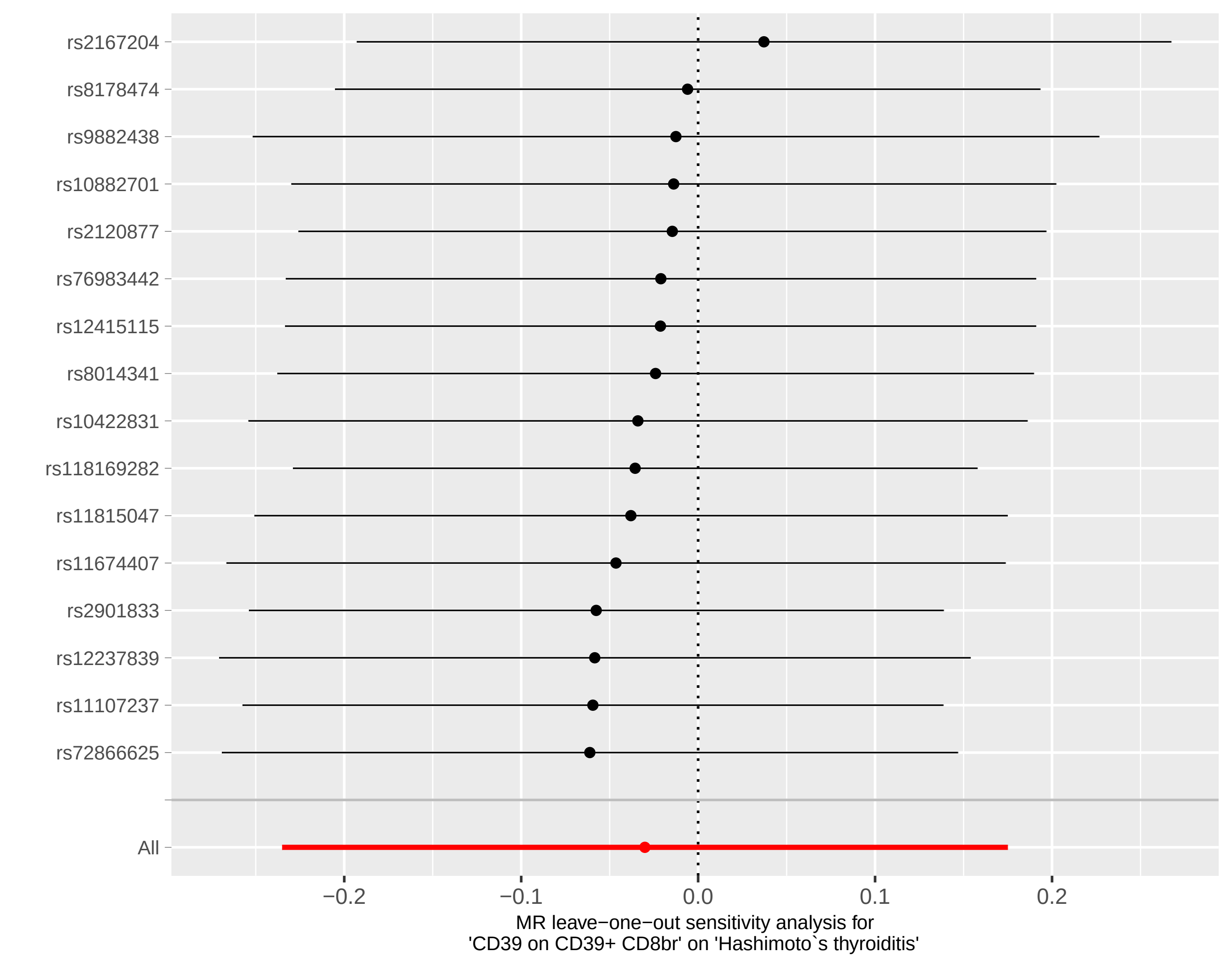
rs2167204
rs8178474
rs9882438
rs10882701
rs2120877
rs76983442
rs12415115
rs8014341
rs10422831
rs118169282
rs11815047
rs11674407
rs2901833
rs12237839
rs11107237
rs72866625
All
−0.2
−0.1
0.0
0.1
0.2
MR leave−one−out sensitivity analysis for
'CD39 on CD39+ CD8br' on 'Hashimoto`s thyroiditis'

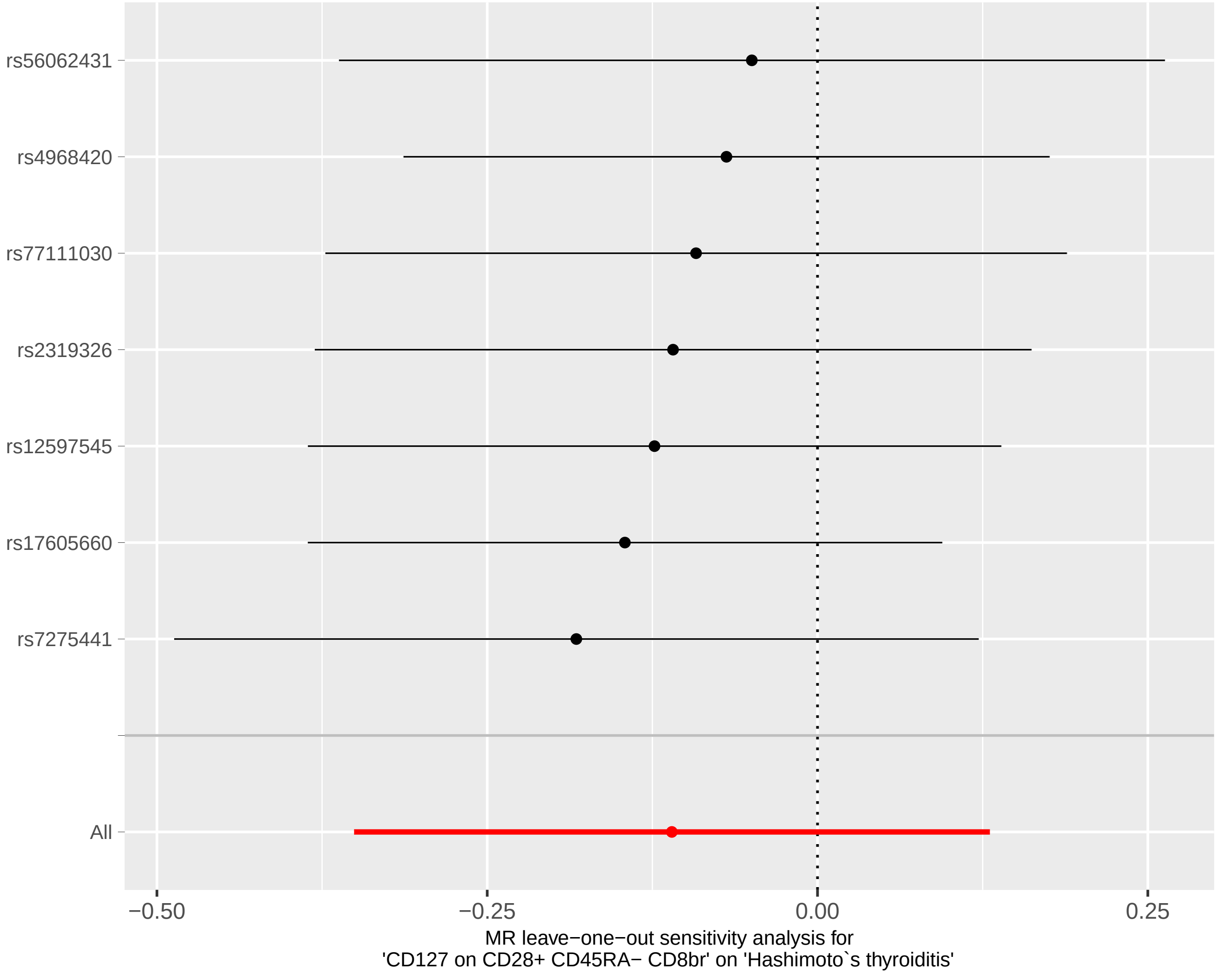

rs56062431
rs4968420
rs77111030
rs2319326
rs12597545
rs17605660
rs7275441
All
−0.50
−0.25
0.00
0.25
MR leave−one−out sensitivity analysis for
'CD127 on CD28+ CD45RA− CD8br' on 'Hashimoto`s thyroiditis'

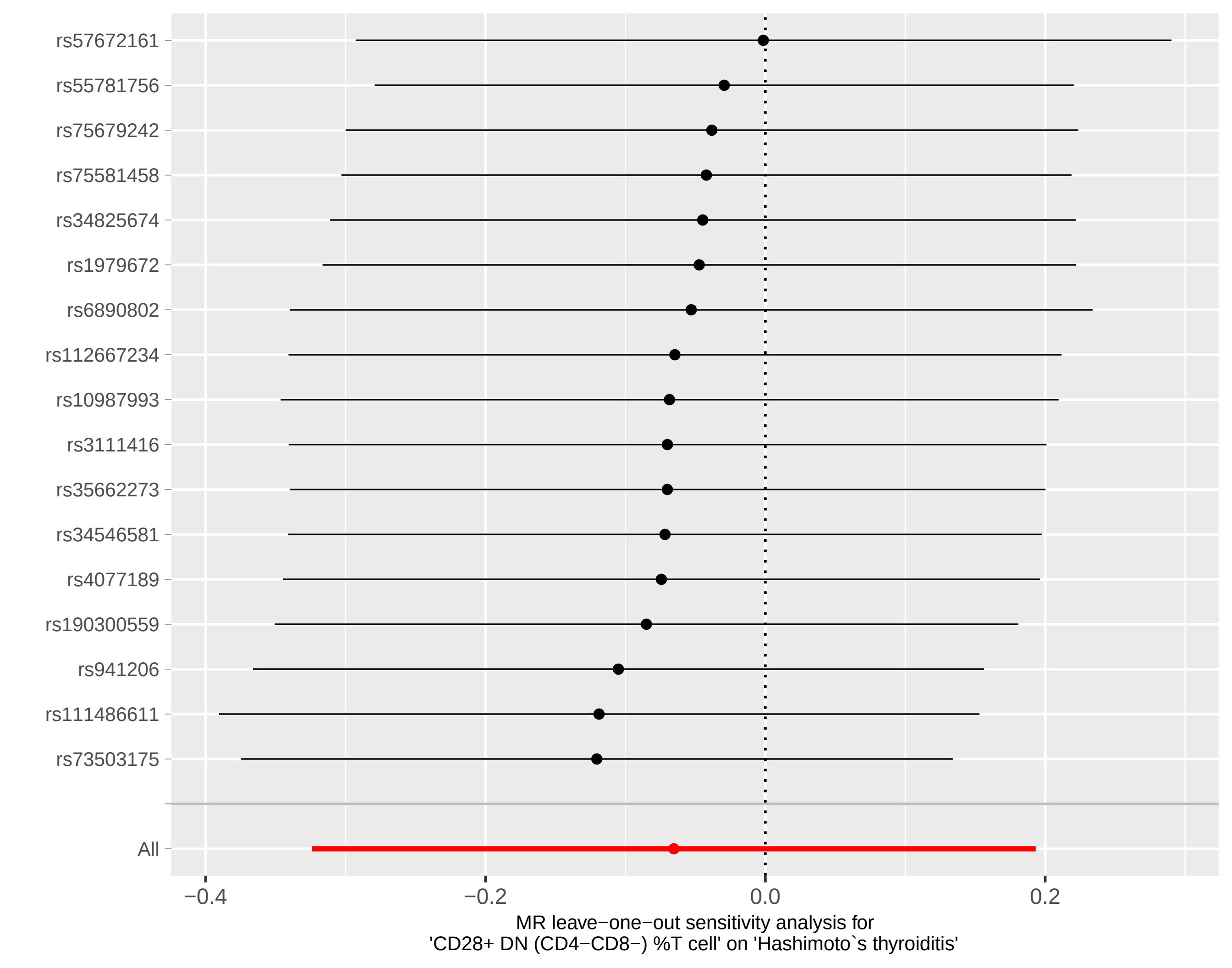
rs57672161
rs55781756
rs75679242
rs75581458
rs34825674
rs1979672
rs6890802
rs112667234
rs10987993
rs3111416
rs35662273
rs34546581
rs4077189
rs190300559
rs941206
rs111486611
rs73503175
All
−0.4
−0.2
0.0
0.2
MR leave−one−out sensitivity analysis for
'CD28+ DN (CD4−CD8−) %T cell' on 'Hashimoto`s thyroiditis'

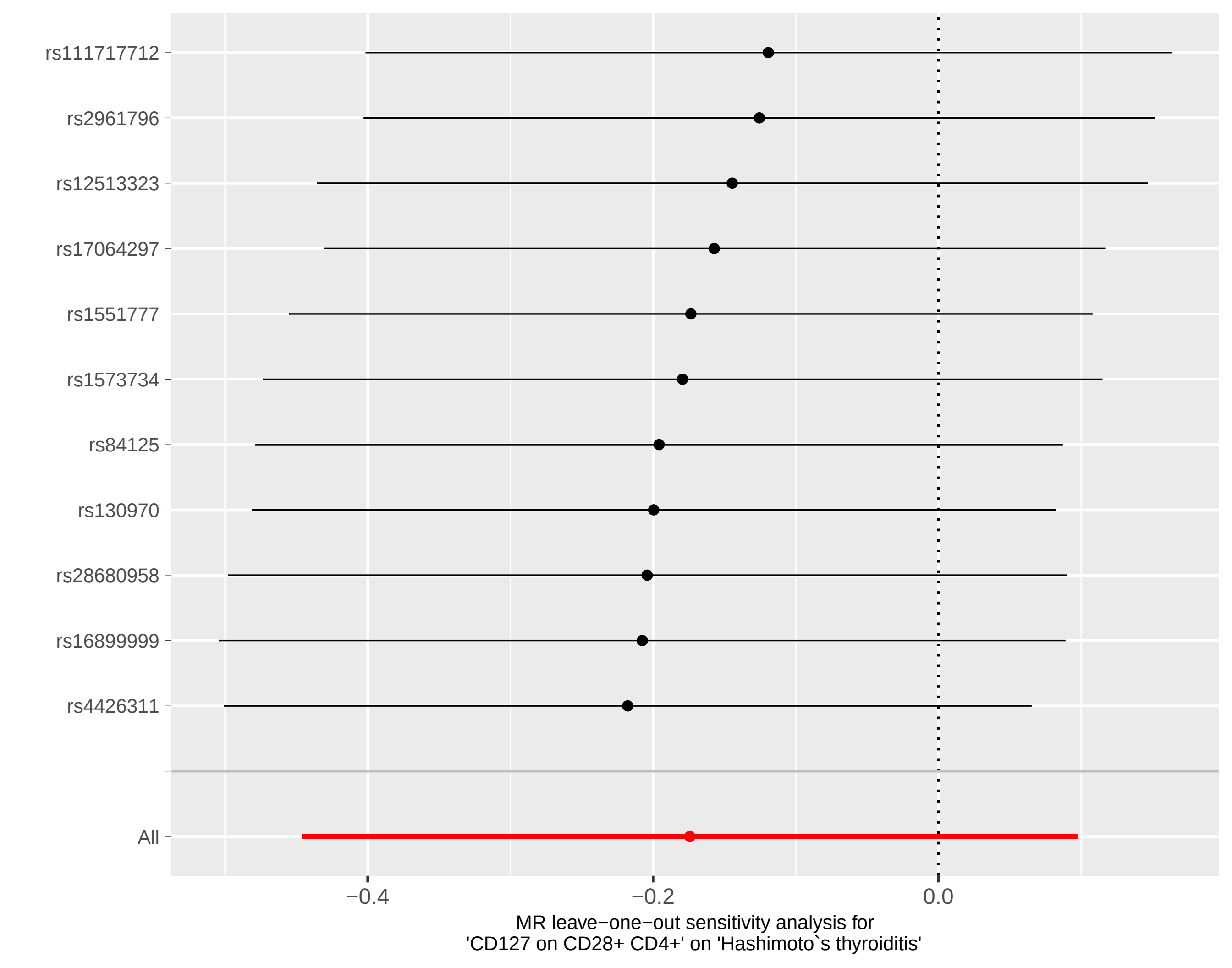
rs111717712
rs2961796
rs12513323
rs17064297
rs1551777
rs1573734
rs84125
rs130970
rs28680958
rs16899999
rs4426311
All
−0.4
−0.2
0.0
MR leave−one−out sensitivity analysis for
'CD127 on CD28+ CD4+' on 'Hashimoto`s thyroiditis'

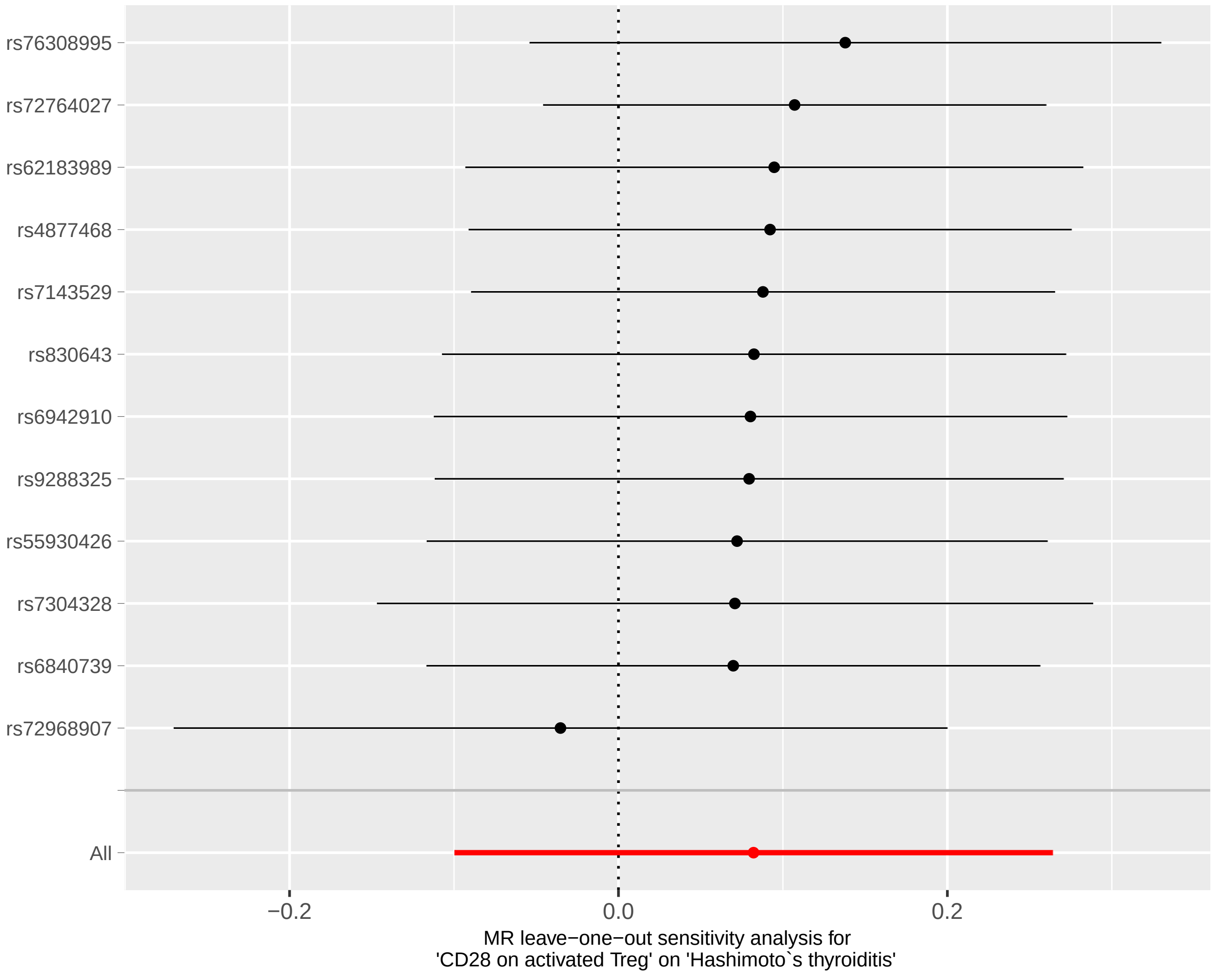

rs76308995
rs72764027
rs62183989
rs4877468
rs7143529
rs830643
rs6942910
rs9288325
rs55930426
rs7304328
rs6840739
rs72968907
All
−0.2
0.0
0.2
MR leave−one−out sensitivity analysis for
'CD28 on activated Treg' on 'Hashimoto`s thyroiditis'

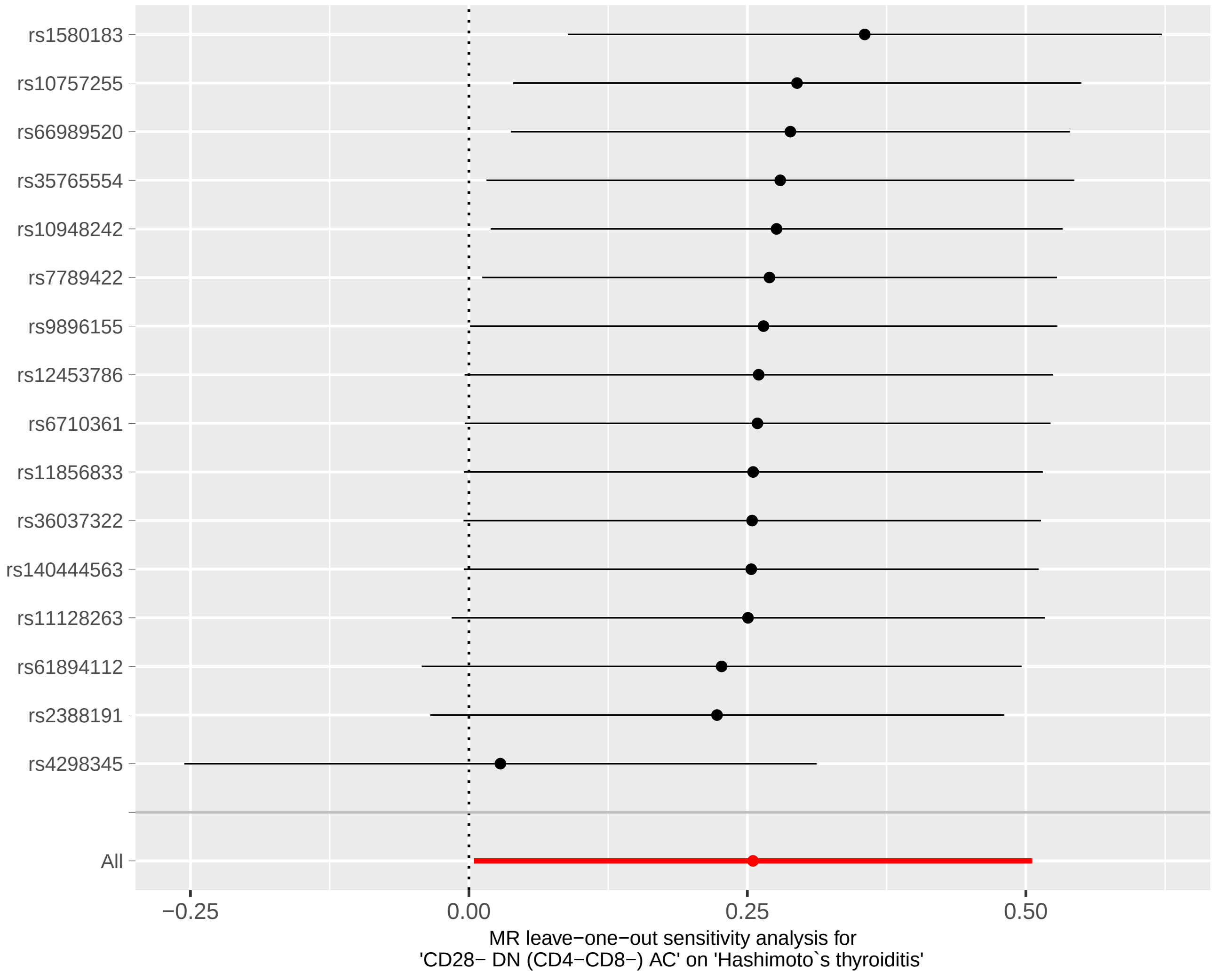
rs1580183
rs10757255
rs66989520
rs35765554
rs10948242
rs7789422
rs9896155
rs12453786
rs6710361
rs11856833
rs36037322
rs140444563
rs11128263
rs61894112
rs2388191
rs4298345
All
−0.25
0.00
0.25
0.50
MR leave−one−out sensitivity analysis for
'CD28− DN (CD4−CD8−) AC' on 'Hashimoto`s thyroiditis'

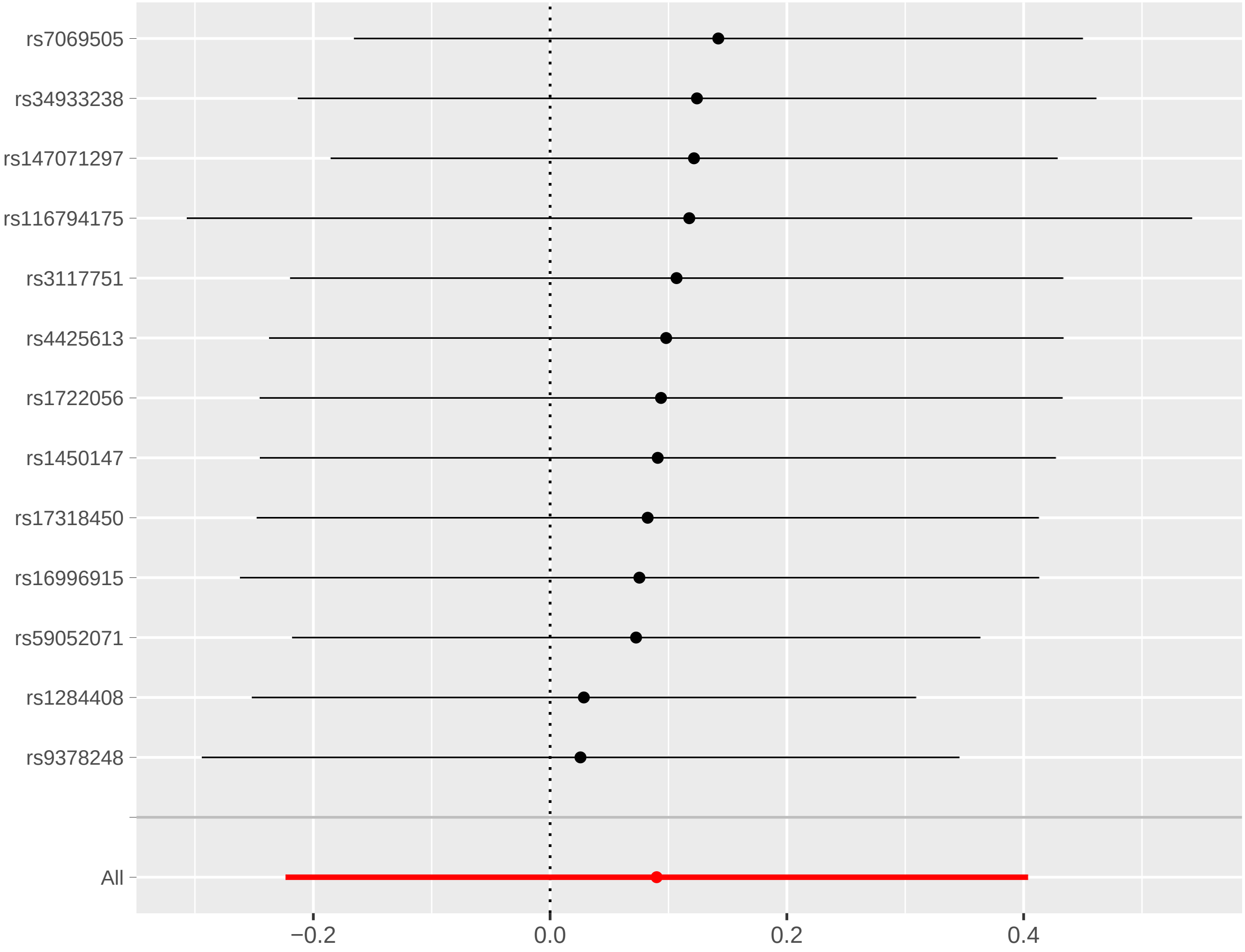

MR leave−one−out sensitivity analysis for
'CD28+ CD45RA− CD8br %T cell' on 'Hashimoto`s thyroiditis'

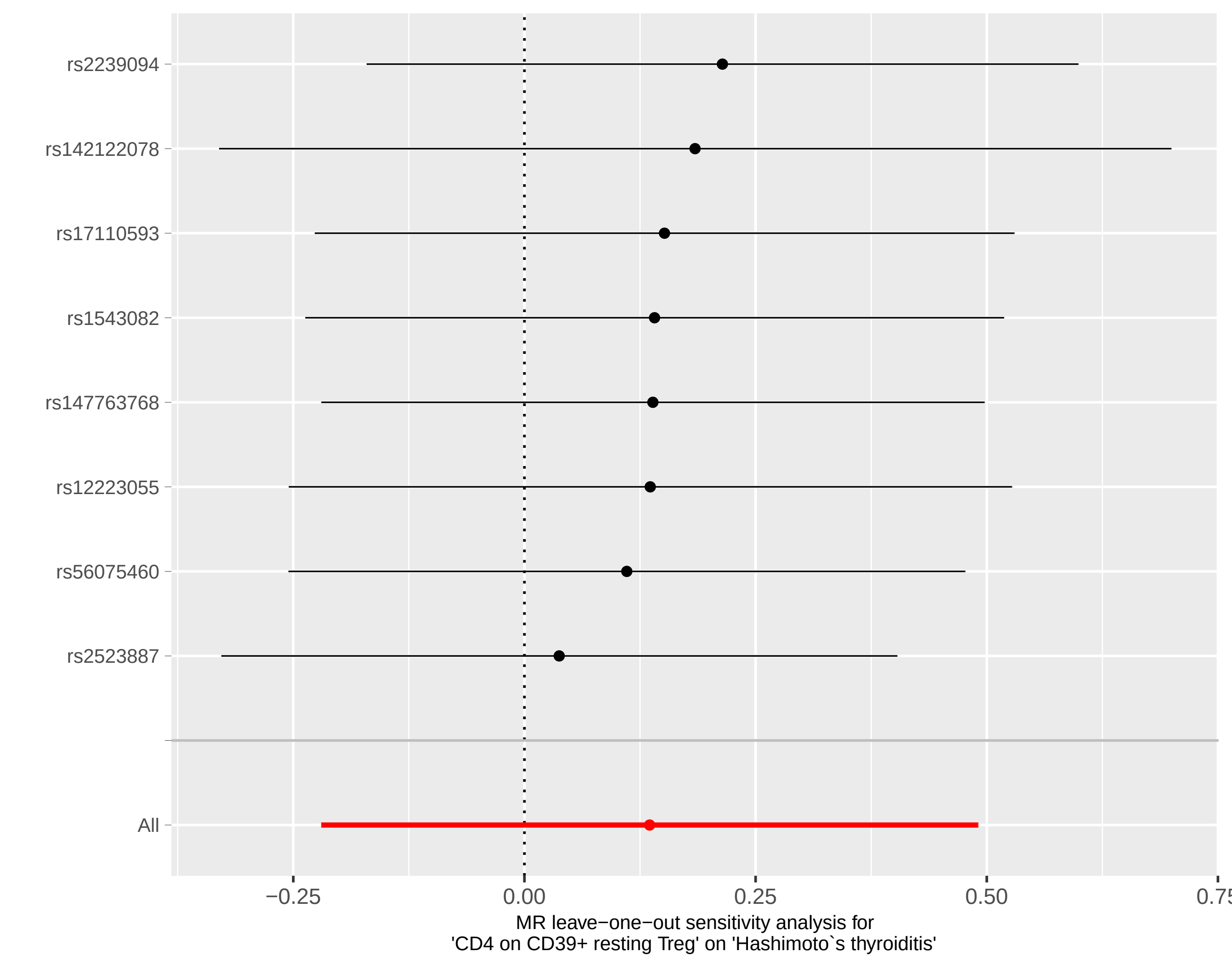
rs2239094
rs142122078
rs17110593
rs1543082
rs147763768
rs12223055
rs56075460
rs2523887
All
−0.25
0.00
0.25
0.50
0.75
MR leave−one−out sensitivity analysis for
'CD4 on CD39+ resting Treg' on 'Hashimoto`s thyroiditis'

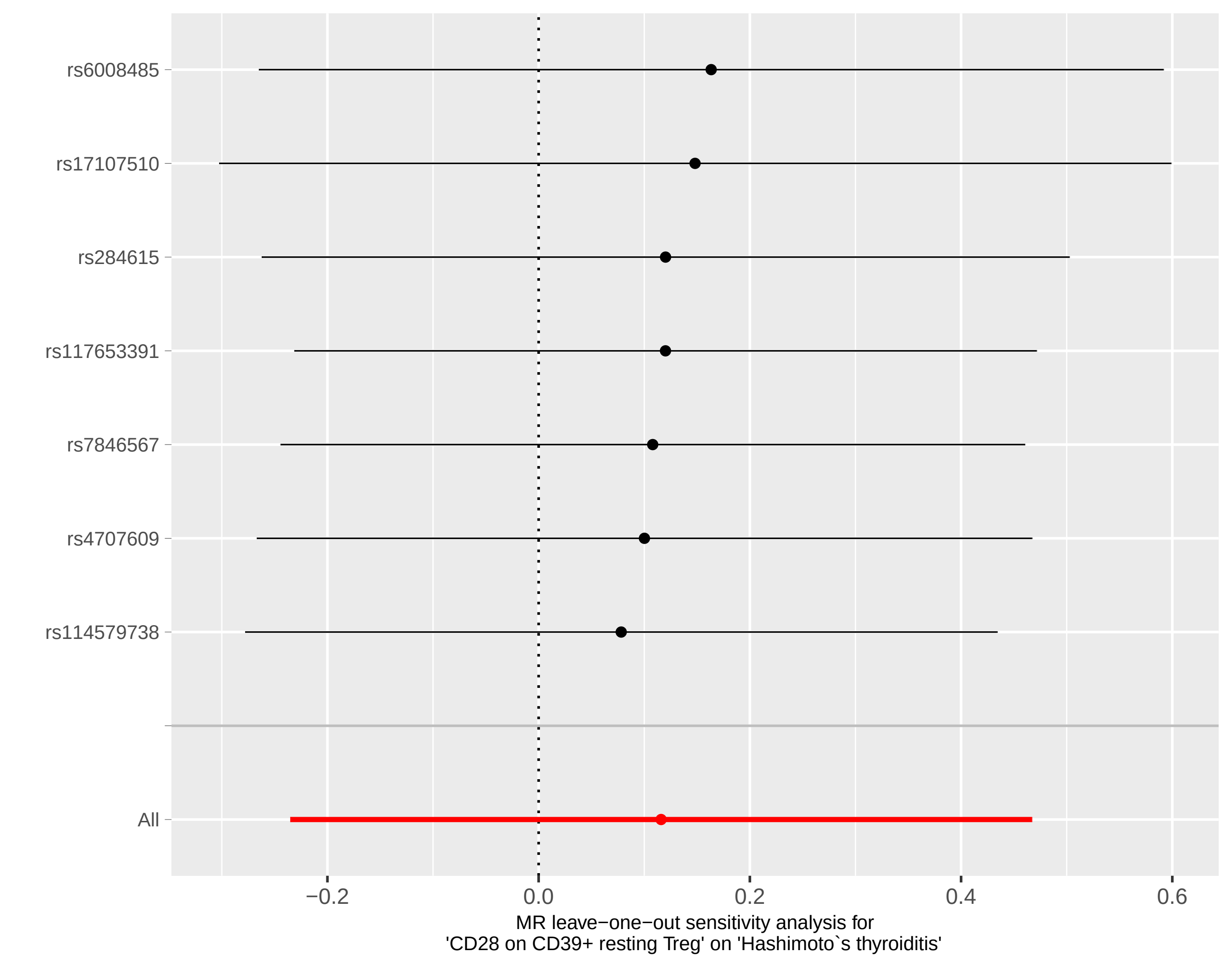
rs6008485
rs17107510
rs284615
rs117653391
rs7846567
rs4707609
rs114579738
All
−0.2
0.0
0.2
0.4
0.6
MR leave−one−out sensitivity analysis for
'CD28 on CD39+ resting Treg' on 'Hashimoto`s thyroiditis'

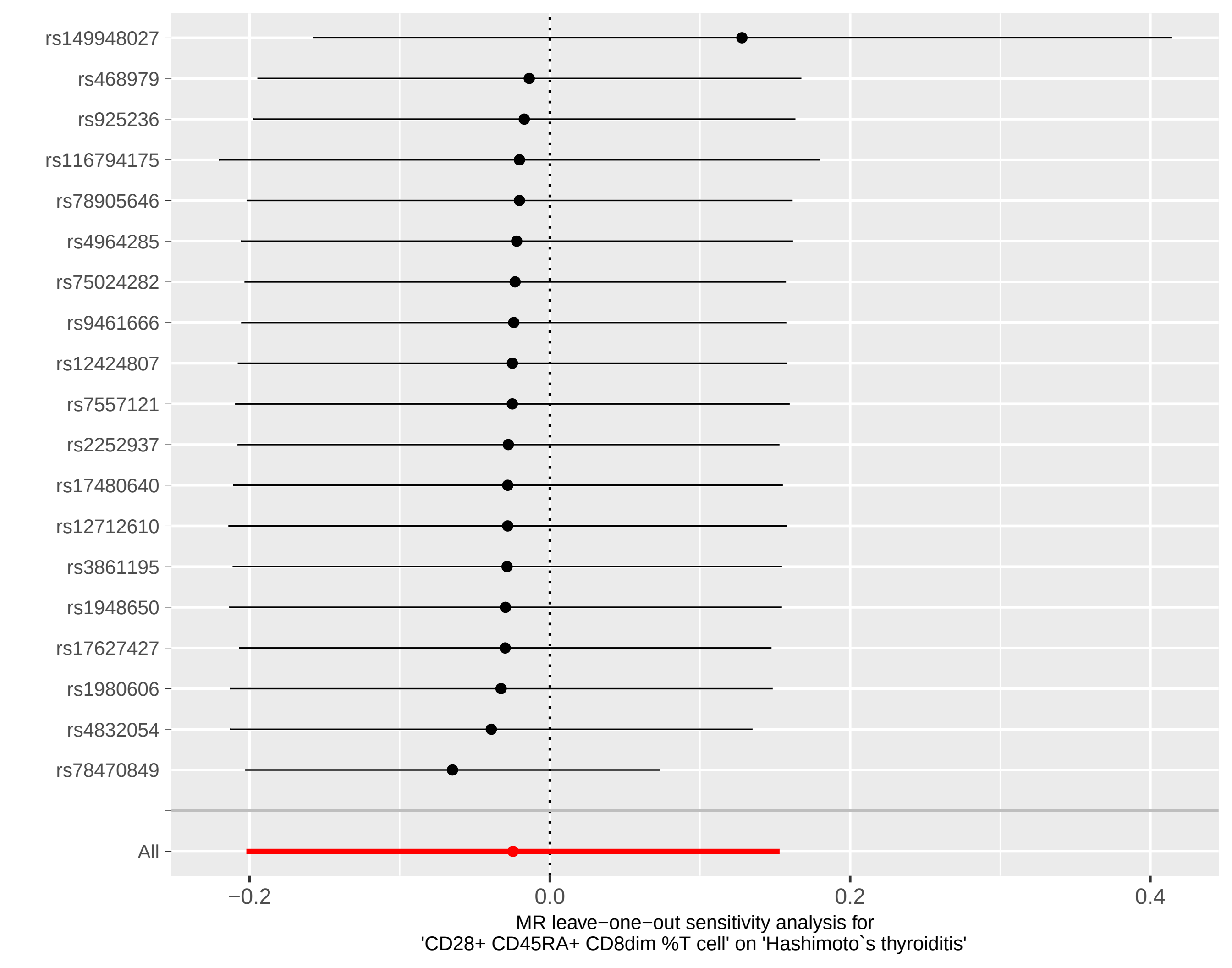

rs149948027
rs468979
rs925236
rs116794175
rs78905646
rs4964285
rs75024282
rs9461666
rs12424807
rs7557121
rs2252937
rs17480640
rs12712610
rs3861195
rs1948650
rs17627427
rs1980606
rs4832054
rs78470849
All
−0.2
0.0
0.2
0.4
MR leave−one−out sensitivity analysis for
'CD28+ CD45RA+ CD8dim %T cell' on 'Hashimoto`s thyroiditis'

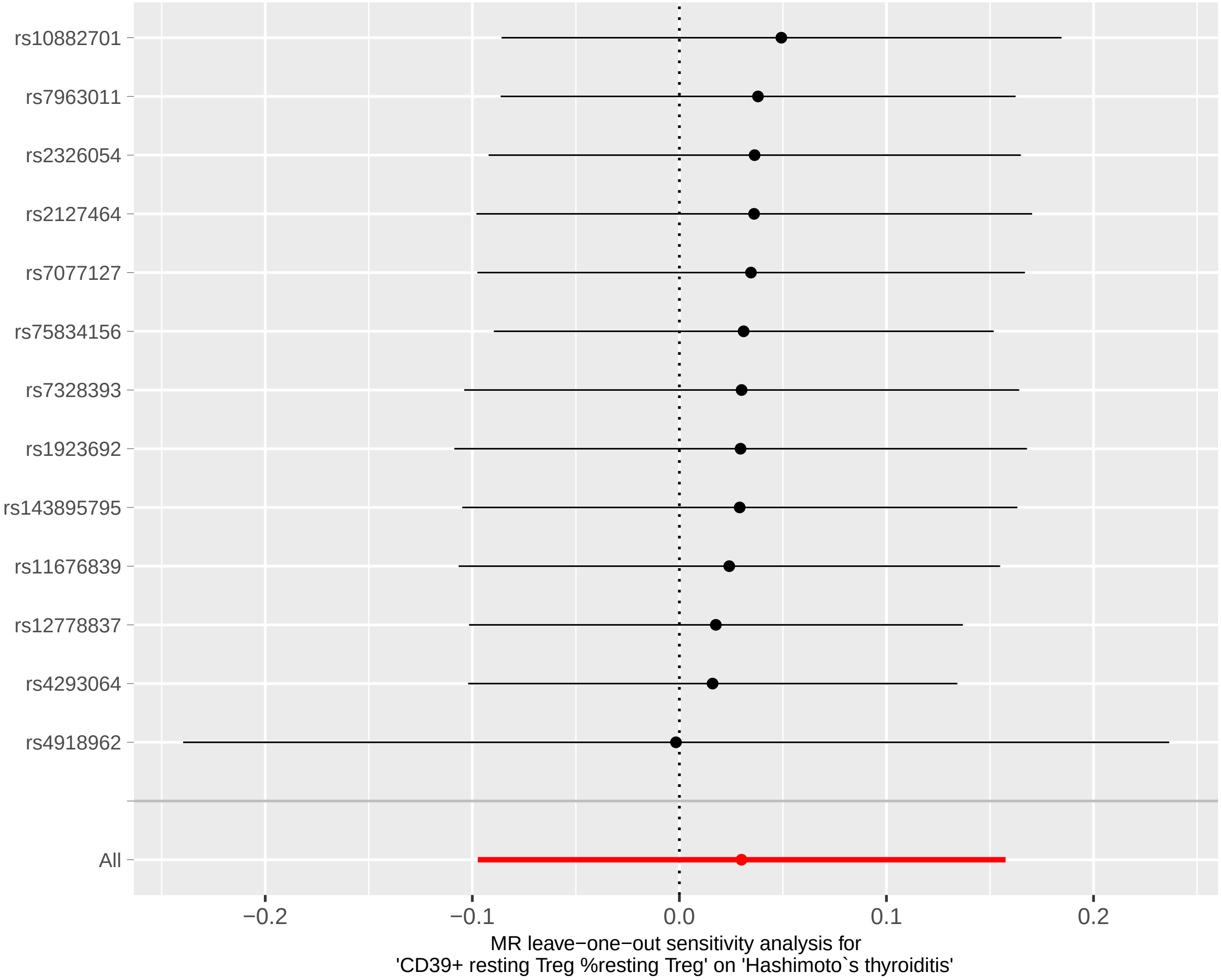
rs10882701
rs7963011
rs2326054
rs2127464
rs7077127
rs75834156
rs7328393
rs1923692
rs143895795
rs11676839
rs12778837
rs4293064
rs4918962
All
−0.2
−0.1
0.0
0.1
0.2
MR leave−one−out sensitivity analysis for
'CD39+ resting Treg %resting Treg' on 'Hashimoto`s thyroiditis'

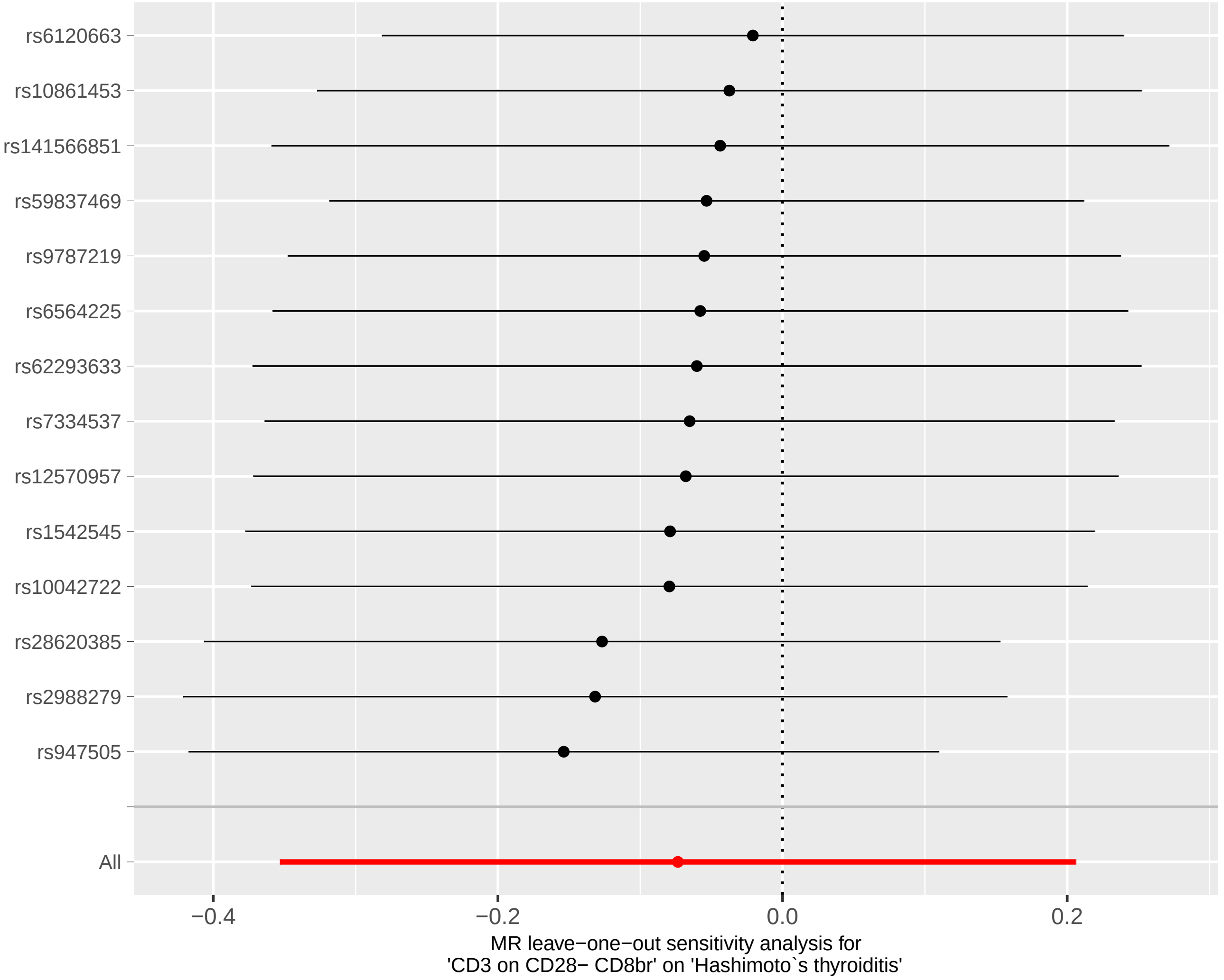
rs6120663
rs10861453
rs141566851
rs59837469
rs9787219
rs6564225
rs62293633
rs7334537
rs12570957
rs1542545
rs10042722
rs28620385
rs2988279
rs947505
All
−0.4
−0.2
0.0
0.2
MR leave−one−out sensitivity analysis for
'CD3 on CD28− CD8br' on 'Hashimoto`s thyroiditis'

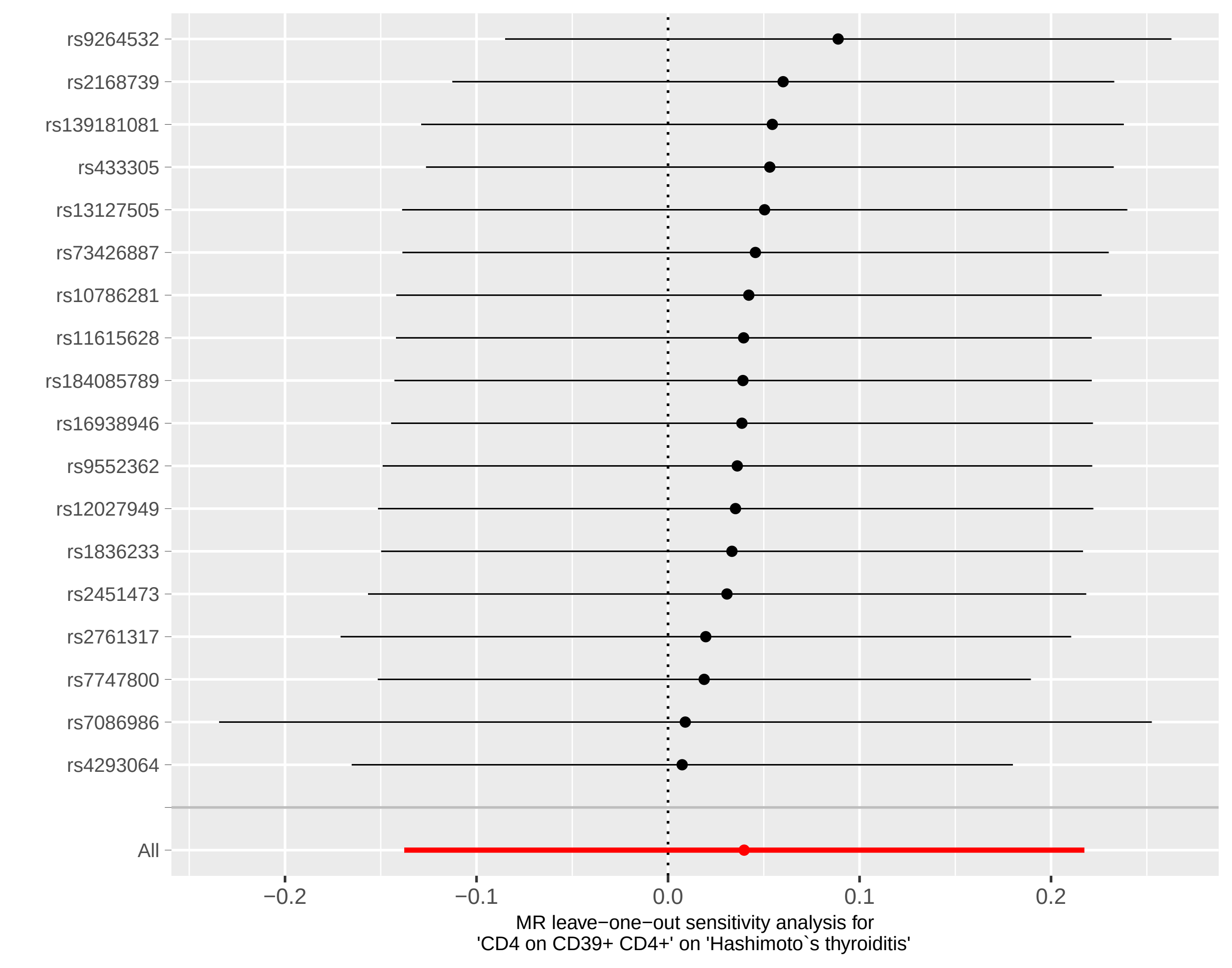
rs9264532
rs2168739
rs139181081
rs433305
rs13127505
rs73426887
rs10786281
rs11615628
rs184085789
rs16938946
rs9552362
rs12027949
rs1836233
rs2451473
rs2761317
rs7747800
rs7086986
rs4293064
All
−0.2
−0.1
0.0
0.1
0.2
MR leave−one−out sensitivity analysis for
'CD4 on CD39+ CD4+' on 'Hashimoto`s thyroiditis'

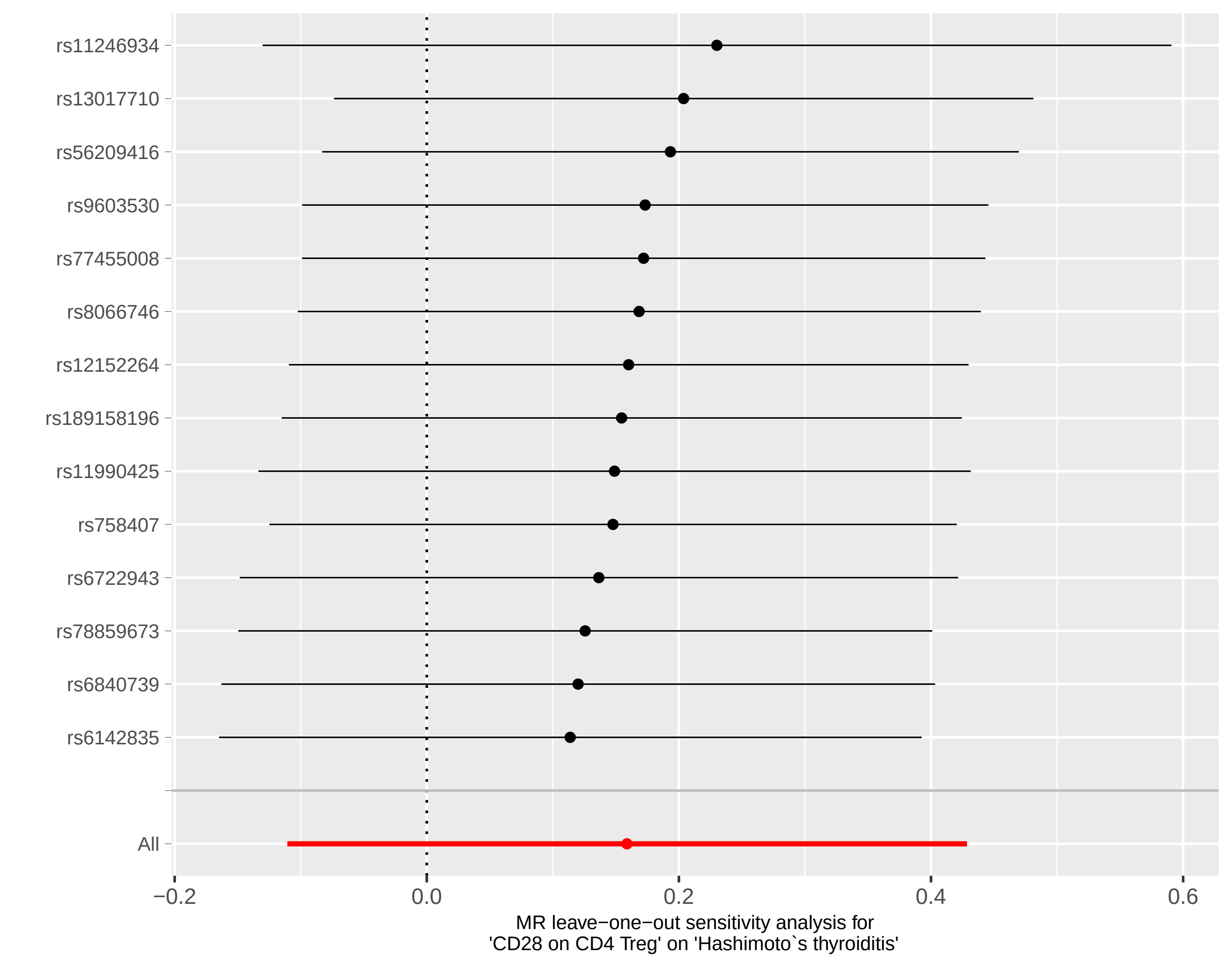
rs11246934
rs13017710
rs56209416
rs9603530
rs77455008
rs8066746
rs12152264
rs189158196
rs11990425
rs758407
rs6722943
rs78859673
rs6840739
rs6142835
All
−0.2
0.0
0.2
0.4
0.6
MR leave−one−out sensitivity analysis for
'CD28 on CD4 Treg' on 'Hashimoto`s thyroiditis'

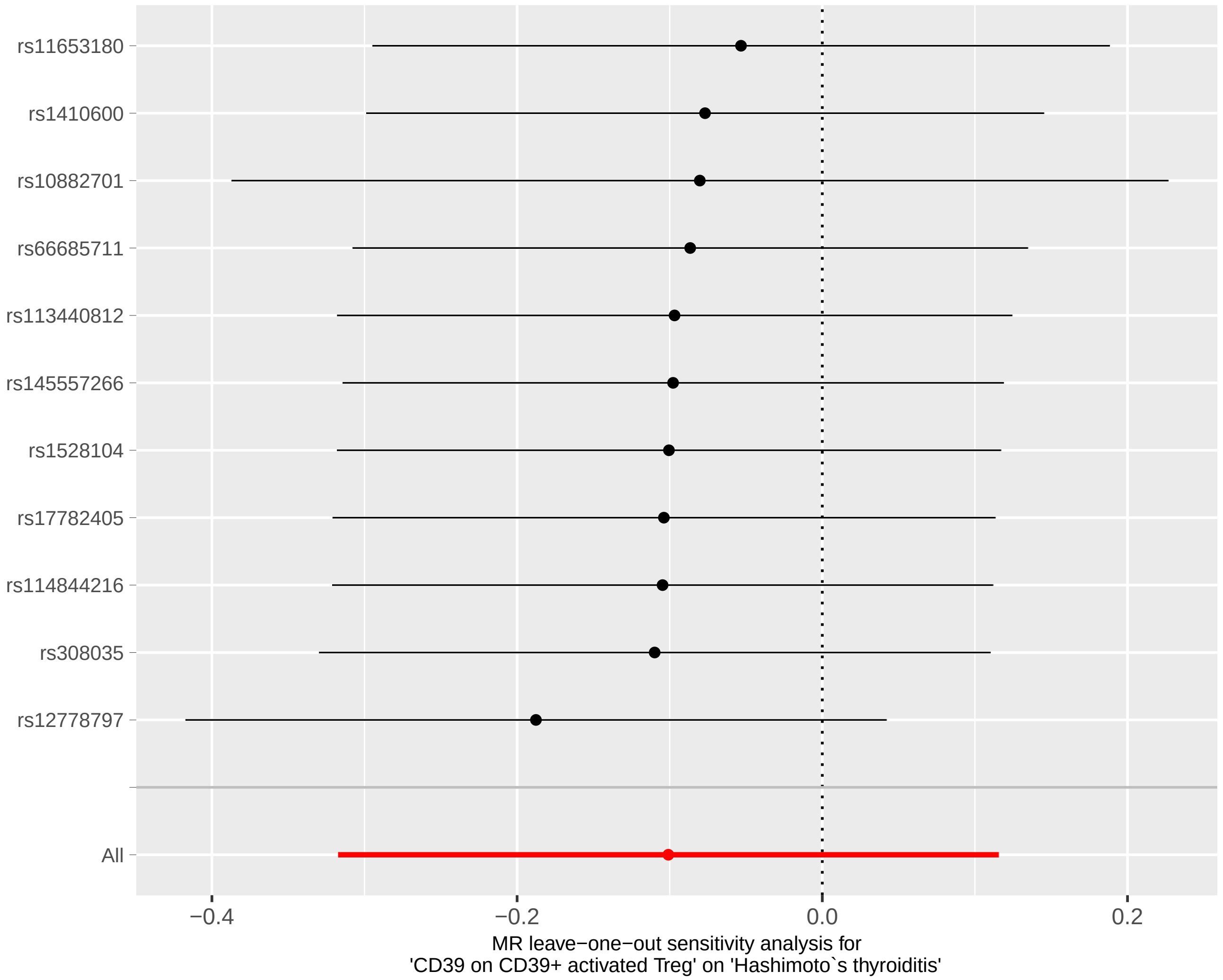
rs11653180
rs1410600
rs10882701
rs66685711
rs113440812
rs145557266
rs1528104
rs17782405
rs114844216
rs308035
rs12778797
All
−0.4
−0.2
0.0
0.2
MR leave−one−out sensitivity analysis for
'CD39 on CD39+ activated Treg' on 'Hashimoto`s thyroiditis'

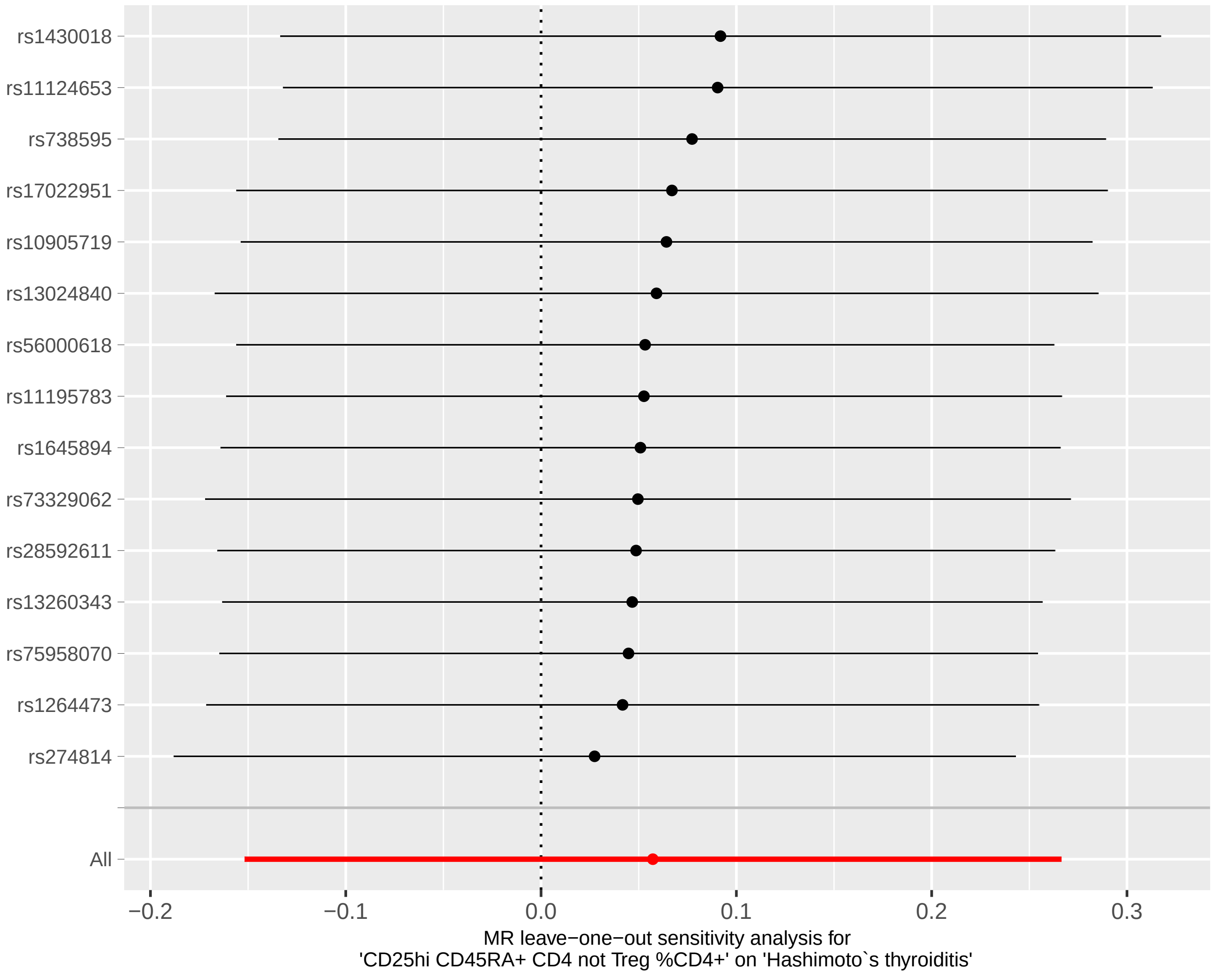
rs1430018
rs11124653
rs738595
rs17022951
rs10905719
rs13024840
rs56000618
rs11195783
rs1645894
rs73329062
rs28592611
rs13260343
rs75958070
rs1264473
rs274814
All
−0.2
−0.1
0.0
0.1
0.2
0.3
MR leave−one−out sensitivity analysis for
'CD25hi CD45RA+ CD4 not Treg %CD4+' on 'Hashimoto`s thyroiditis'

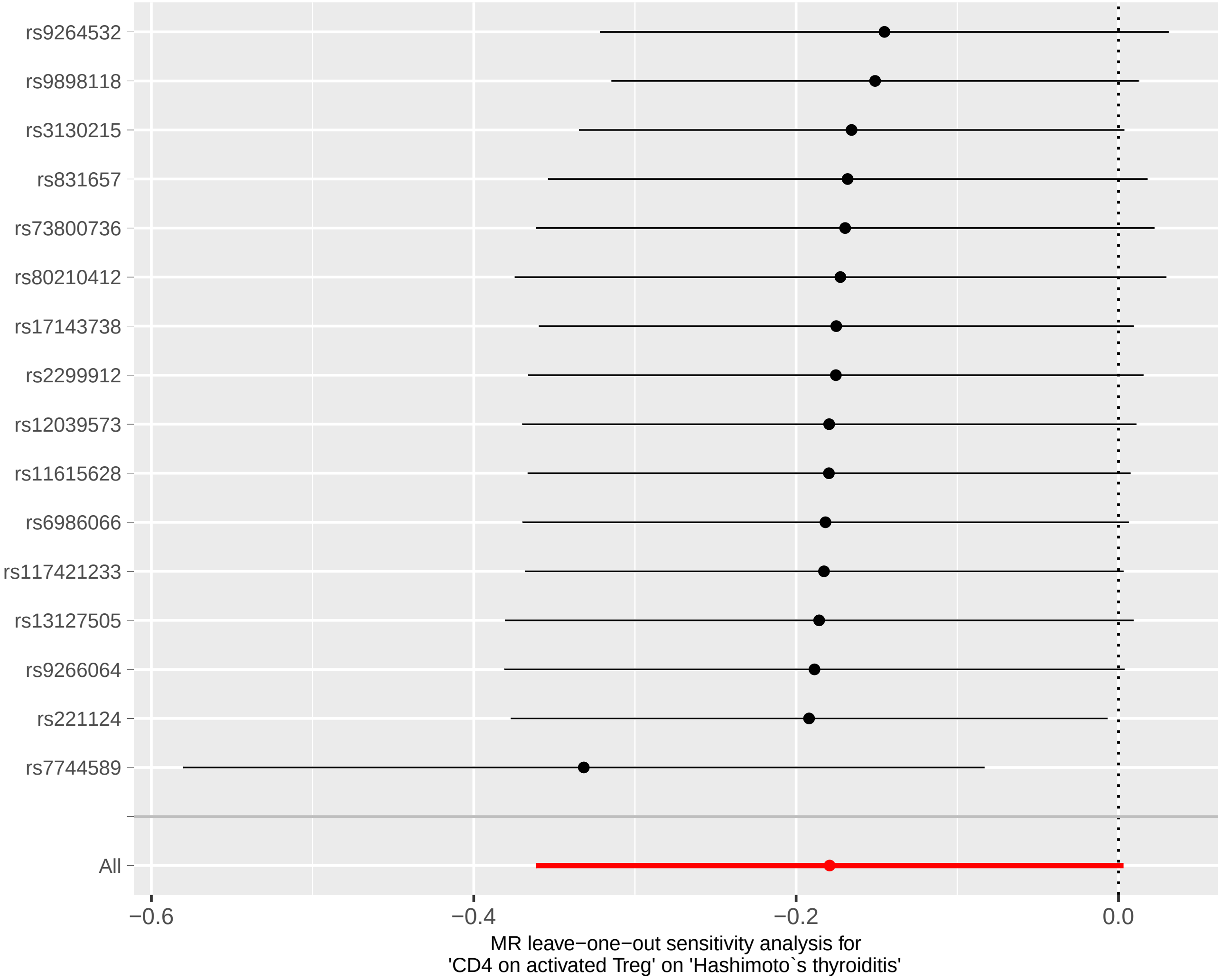

rs9264532
rs9898118
rs3130215
rs831657
rs73800736
rs80210412
rs17143738
rs2299912
rs12039573
rs11615628
rs6986066
rs117421233
rs13127505
rs9266064
rs221124
rs7744589
All
−0.6
−0.4
−0.2
0.0
MR leave−one−out sensitivity analysis for
'CD4 on activated Treg' on 'Hashimoto`s thyroiditis'

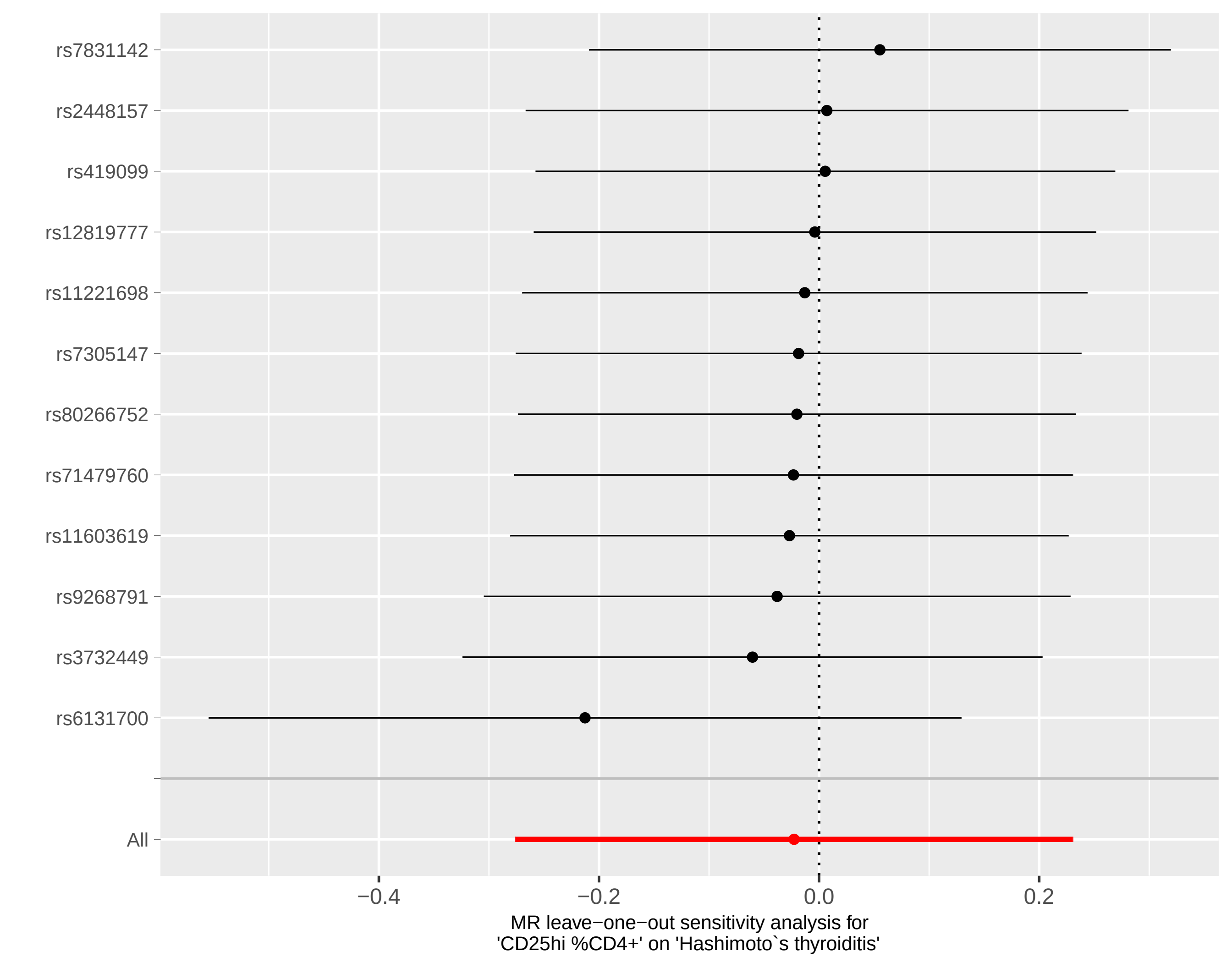
rs7831142
rs2448157
rs419099
rs12819777
rs11221698
rs7305147
rs80266752
rs71479760
rs11603619
rs9268791
rs3732449
rs6131700
All
−0.4
−0.2
0.0
0.2
MR leave−one−out sensitivity analysis for
'CD25hi %CD4+' on 'Hashimoto`s thyroiditis'

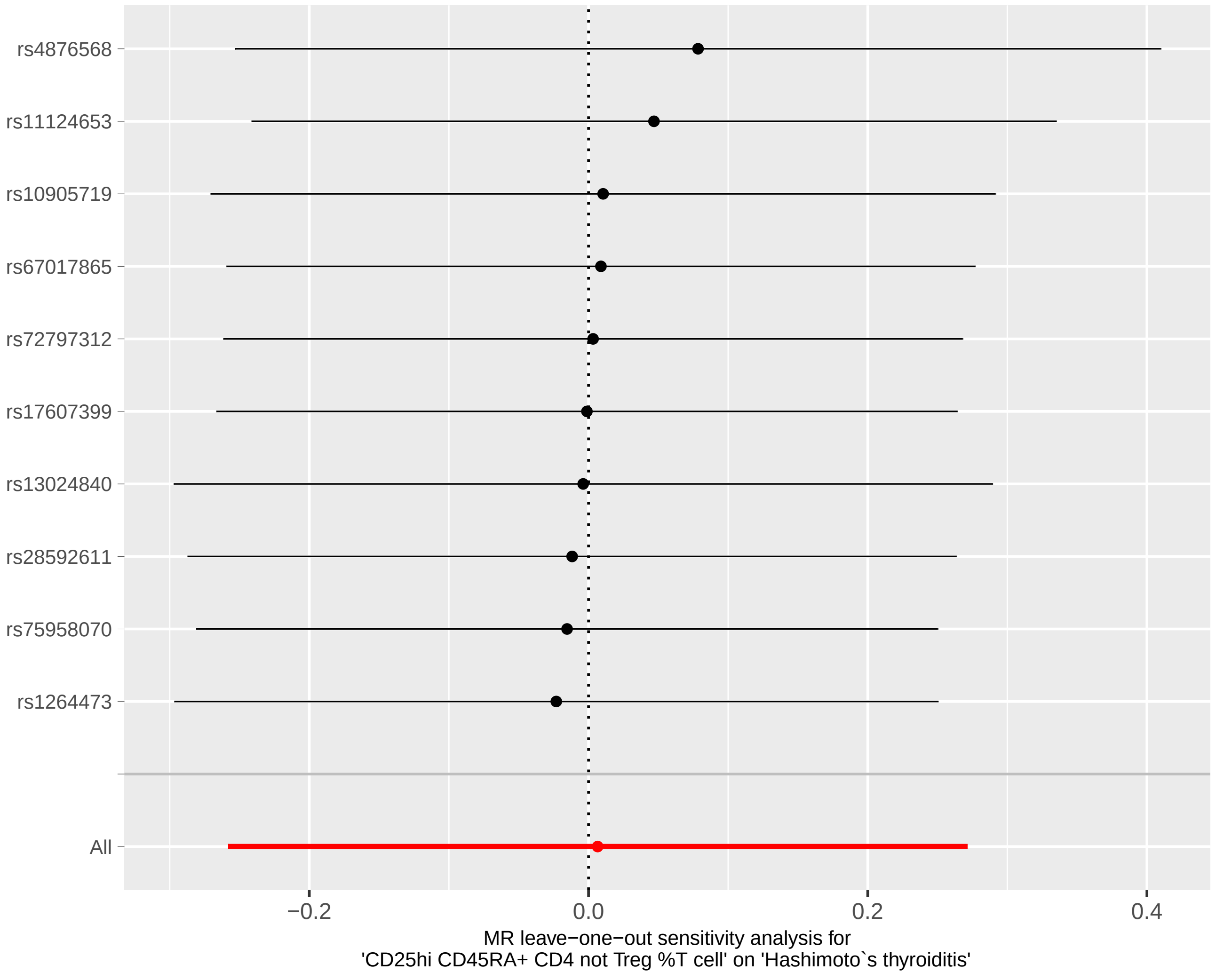
rs4876568
rs11124653
rs10905719
rs67017865
rs72797312
rs17607399
rs13024840
rs28592611
rs75958070
rs1264473
All
−0.2
0.0
0.2
0.4
MR leave−one−out sensitivity analysis for
'CD25hi CD45RA+ CD4 not Treg %T cell' on 'Hashimoto`s thyroiditis'

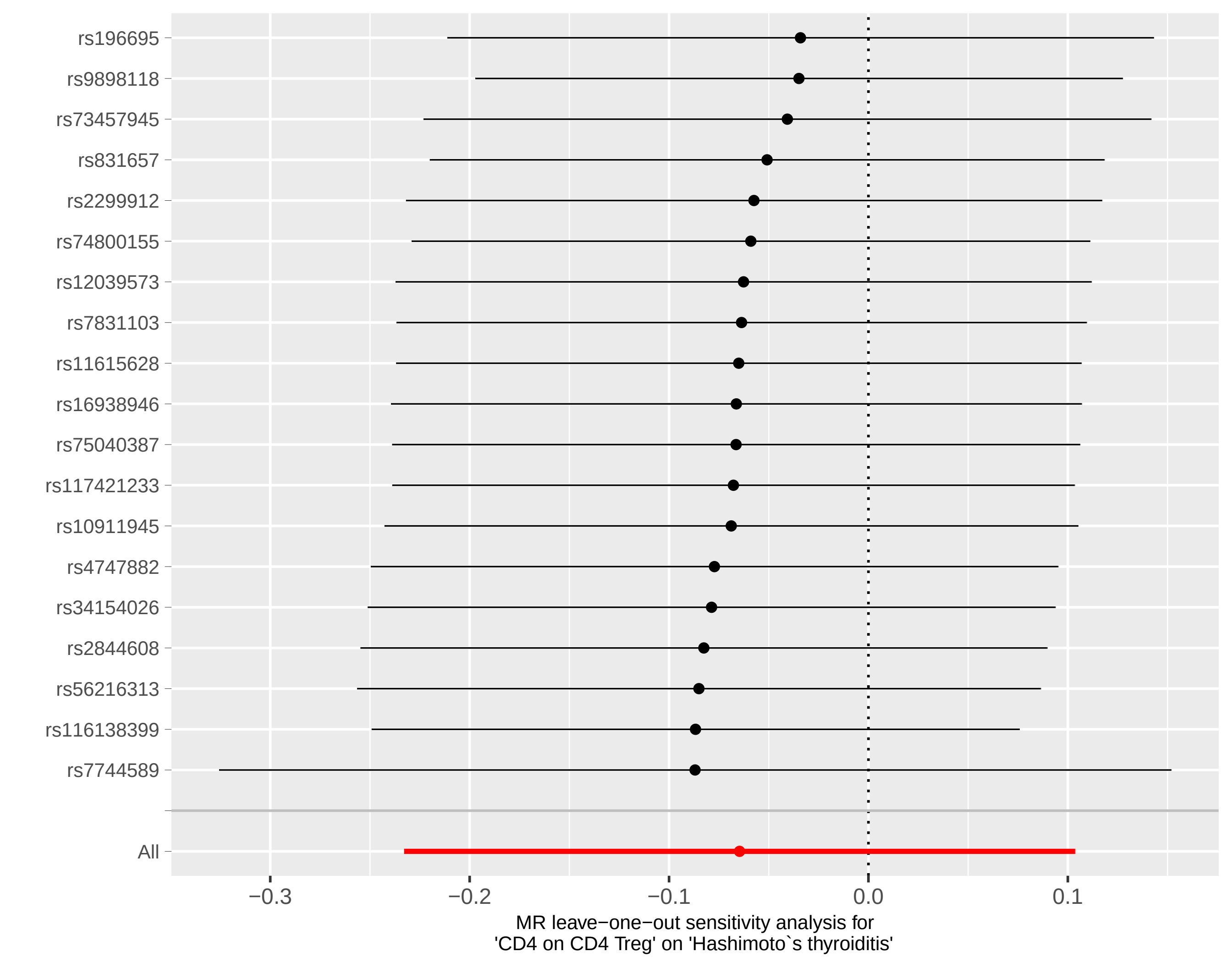

rs196695
rs9898118
rs73457945
rs831657
rs2299912
rs74800155
rs12039573
rs7831103
rs11615628
rs16938946
rs75040387
rs117421233
rs10911945
rs4747882
rs34154026
rs2844608
rs56216313
rs116138399
rs7744589
All
−0.3
−0.2
−0.1
0.0
0.1
MR leave−one−out sensitivity analysis for
'CD4 on CD4 Treg' on 'Hashimoto`s thyroiditis'

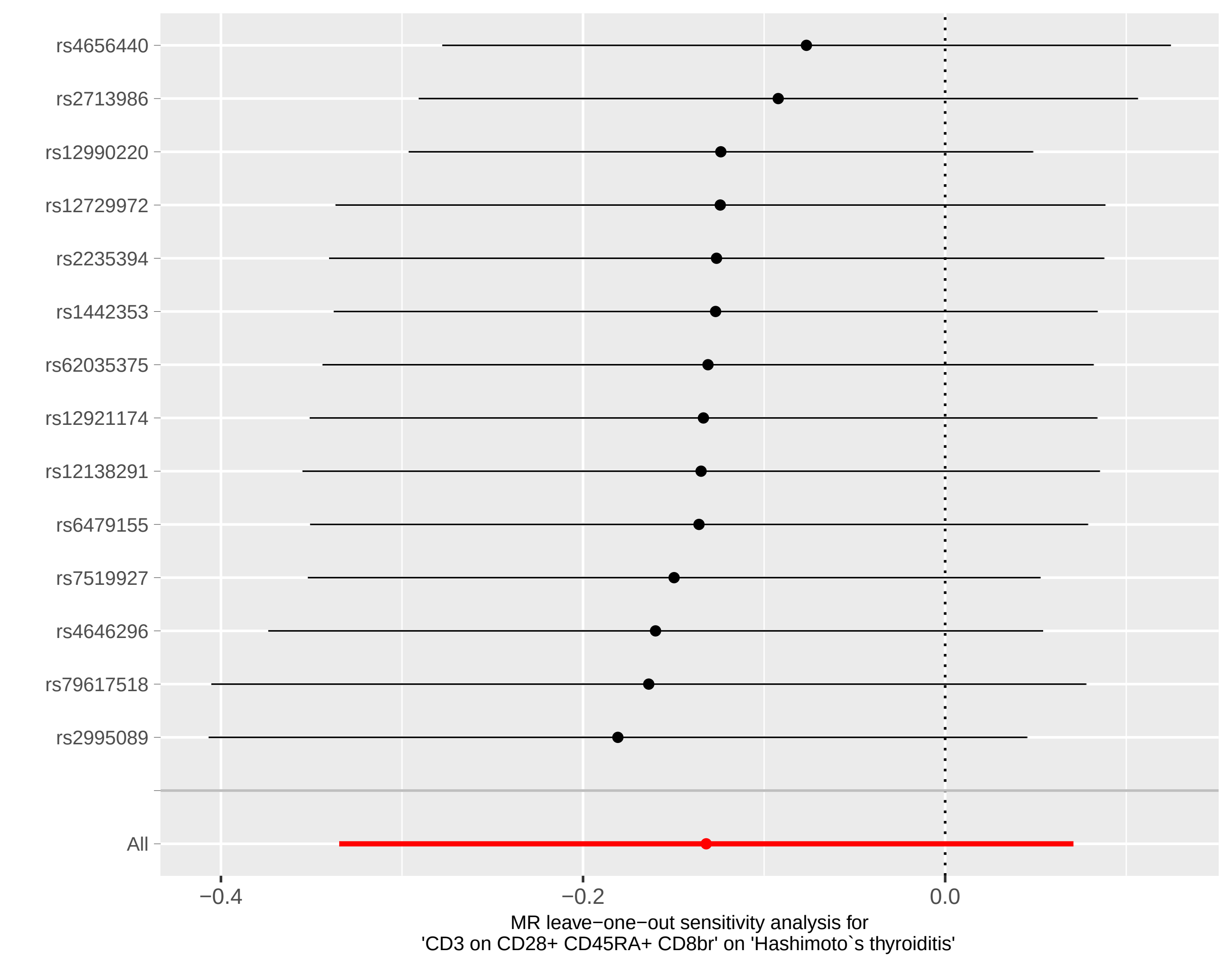
rs4656440
rs2713986
rs12990220
rs12729972
rs2235394
rs1442353
rs62035375
rs12921174
rs12138291
rs6479155
rs7519927
rs4646296
rs79617518
rs2995089
All
−0.4
−0.2
0.0
MR leave−one−out sensitivity analysis for
'CD3 on CD28+ CD45RA+ CD8br' on 'Hashimoto`s thyroiditis'

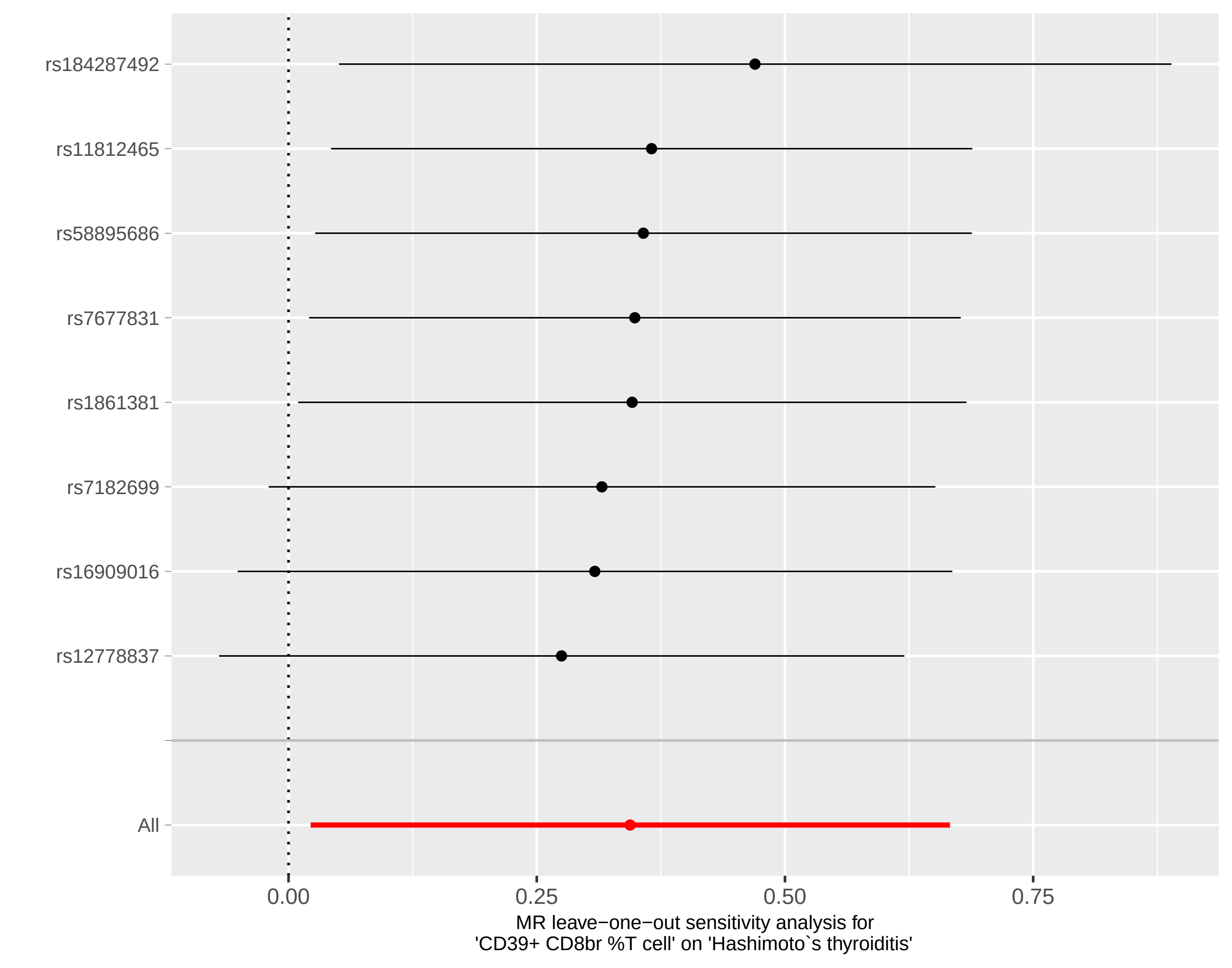

rs184287492
rs11812465
rs58895686
rs7677831
rs1861381
rs7182699
rs16909016
rs12778837
All
0.00
0.25
0.50
0.75
MR leave−one−out sensitivity analysis for
'CD39+ CD8br %T cell' on 'Hashimoto`s thyroiditis'

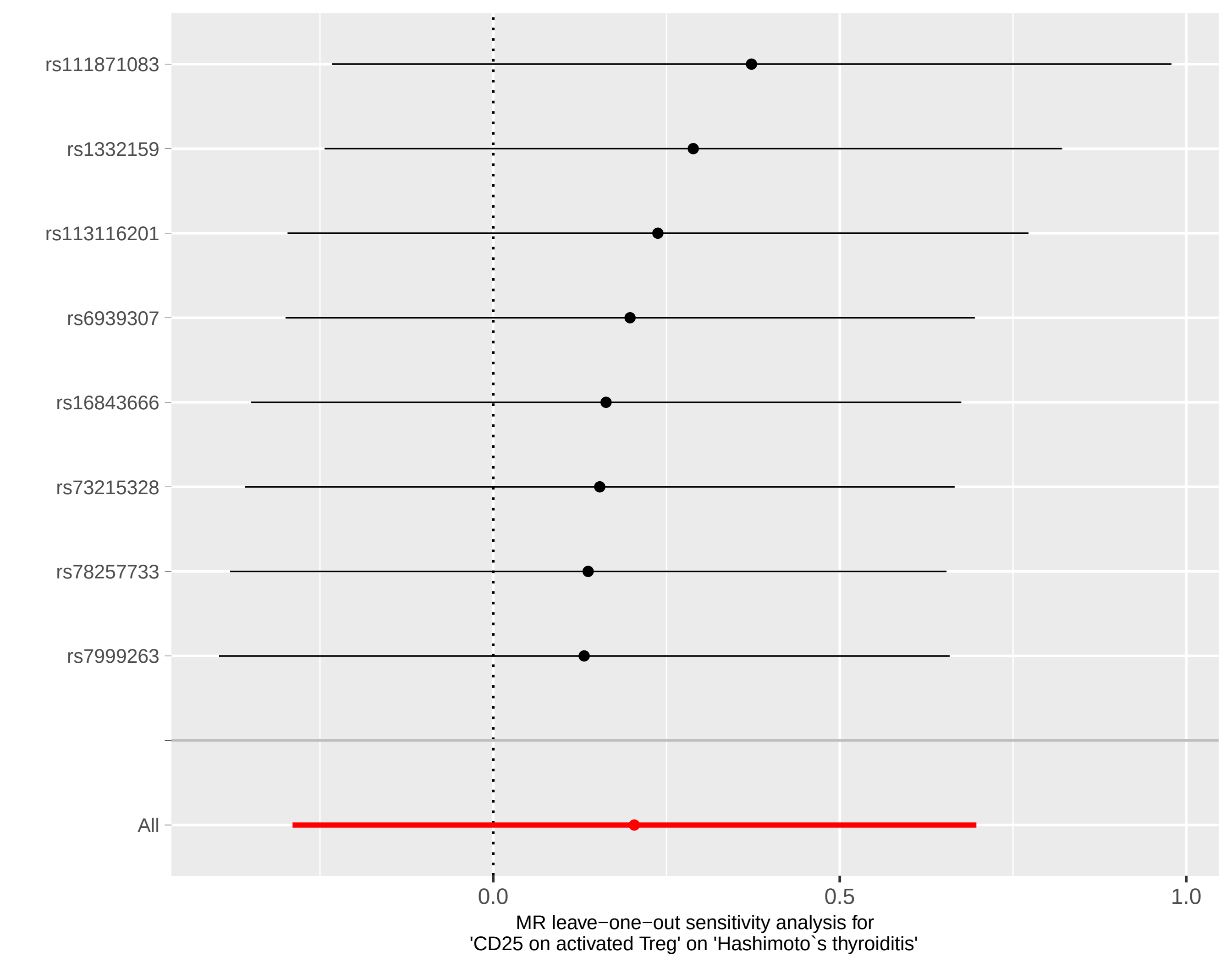
rs111871083
rs1332159
rs113116201
rs6939307
rs16843666
rs73215328
rs78257733
rs7999263
All
0.0
0.5
1.0
MR leave−one−out sensitivity analysis for
'CD25 on activated Treg' on 'Hashimoto`s thyroiditis'

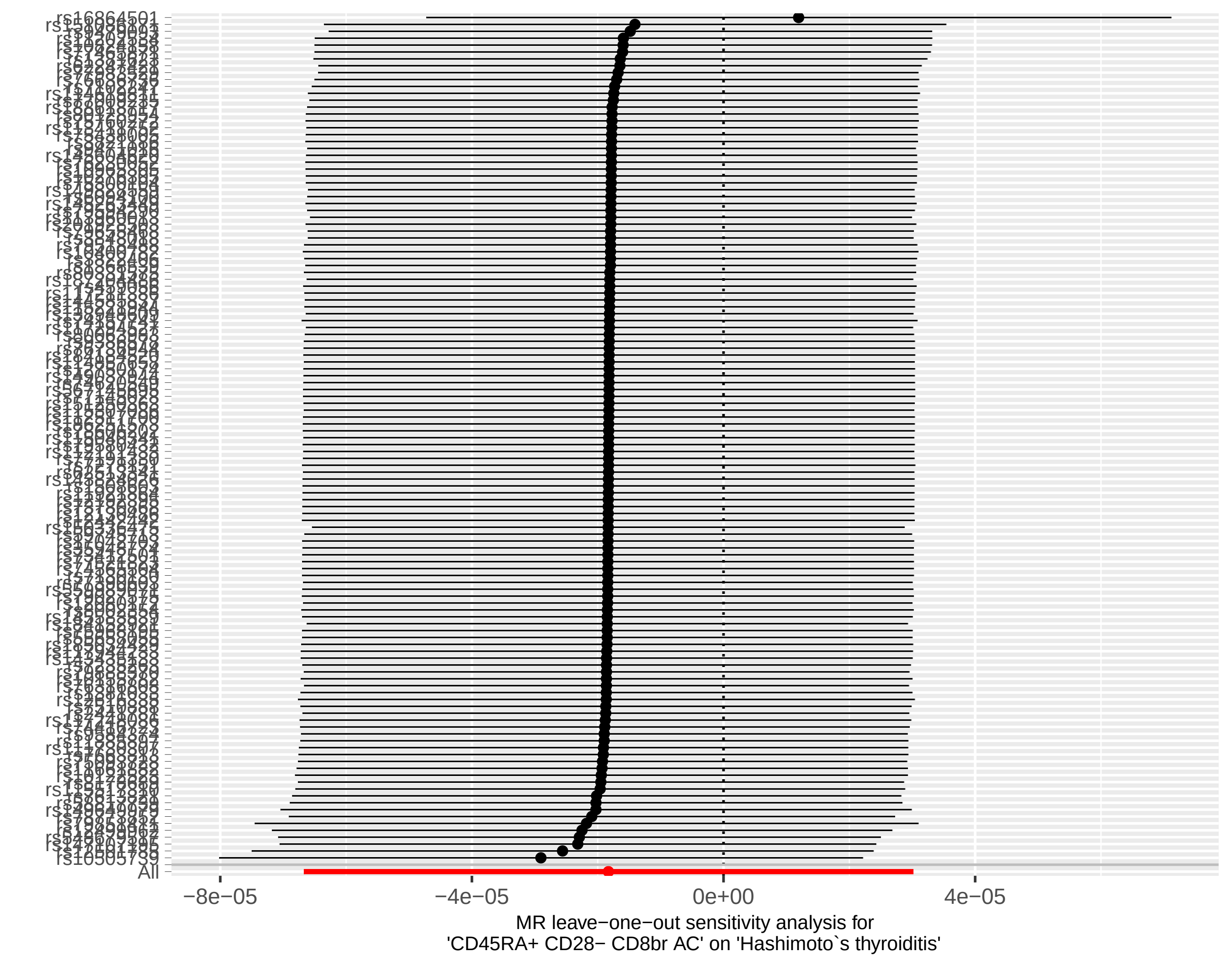
rs16864501
rs10505739
All
−8e−05
−4e−05
0e+00
4e−05
MR leave−one−out sensitivity analysis for
'CD45RA+ CD28− CD8br AC' on 'Hashimoto`s thyroiditis'

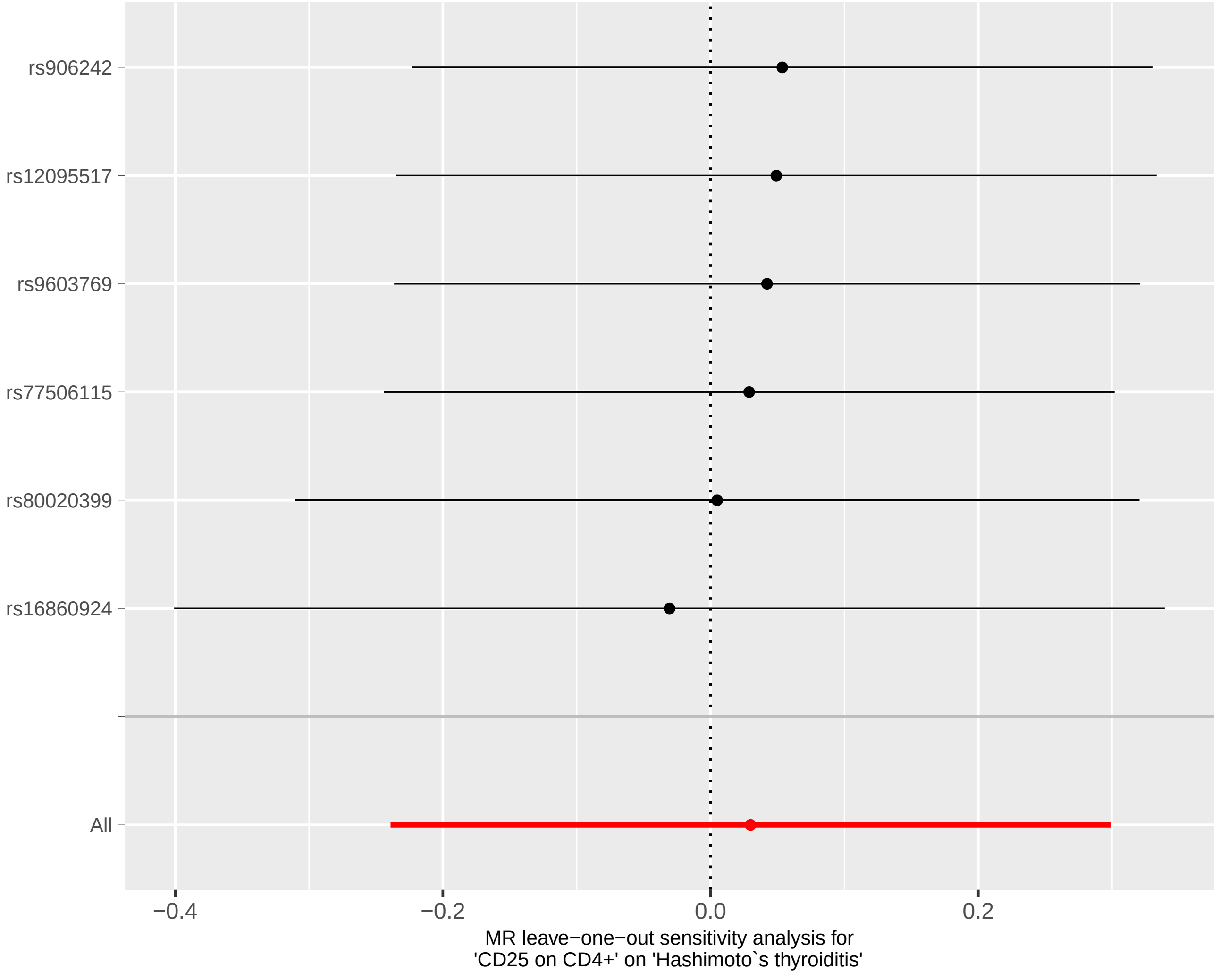

rs906242
rs12095517
rs9603769
rs77506115
rs80020399
rs16860924
All
−0.4
−0.2
0.0
0.2
MR leave−one−out sensitivity analysis for
'CD25 on CD4+' on 'Hashimoto`s thyroiditis'

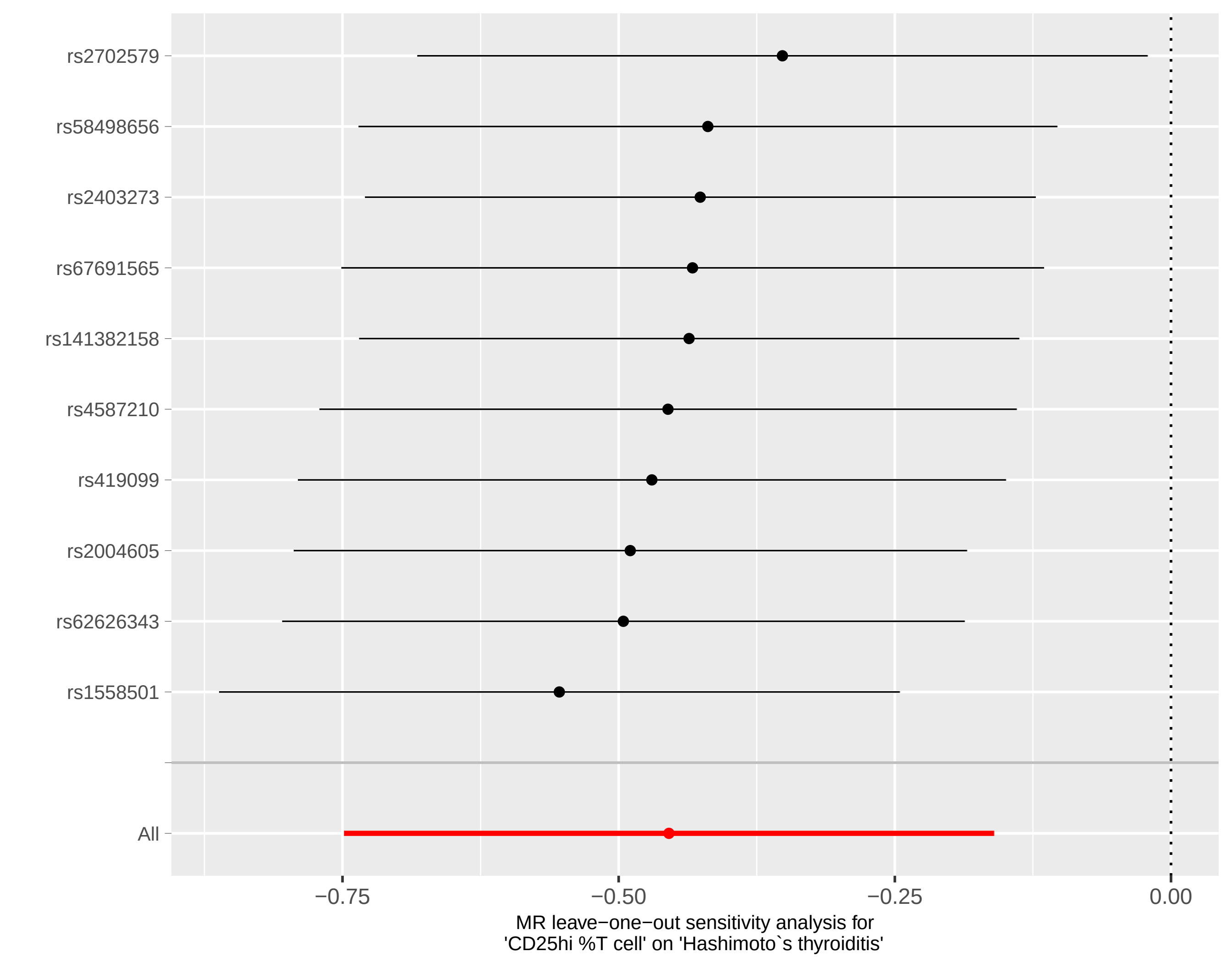

rs2702579
rs58498656
rs2403273
rs67691565
rs141382158
rs4587210
rs419099
rs2004605
rs62626343
rs1558501
All
−0.75
−0.50
−0.25
0.00
MR leave−one−out sensitivity analysis for
'CD25hi %T cell' on 'Hashimoto`s thyroiditis'

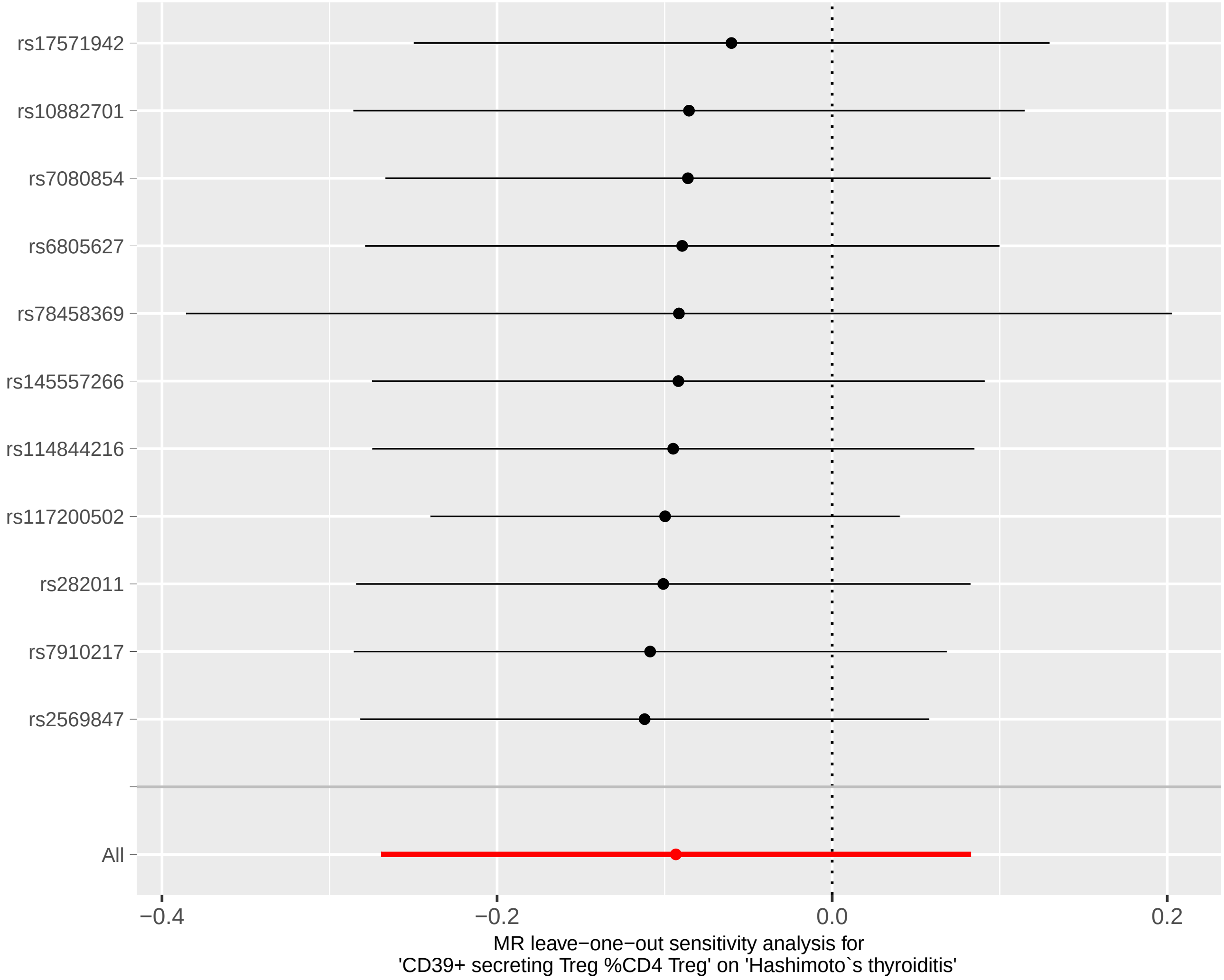
rs17571942
rs10882701
rs7080854
rs6805627
rs78458369
rs145557266
rs114844216
rs117200502
rs282011
rs7910217
rs2569847
All
−0.4
−0.2
0.0
0.2
MR leave−one−out sensitivity analysis for
'CD39+ secreting Treg %CD4 Treg' on 'Hashimoto`s thyroiditis'

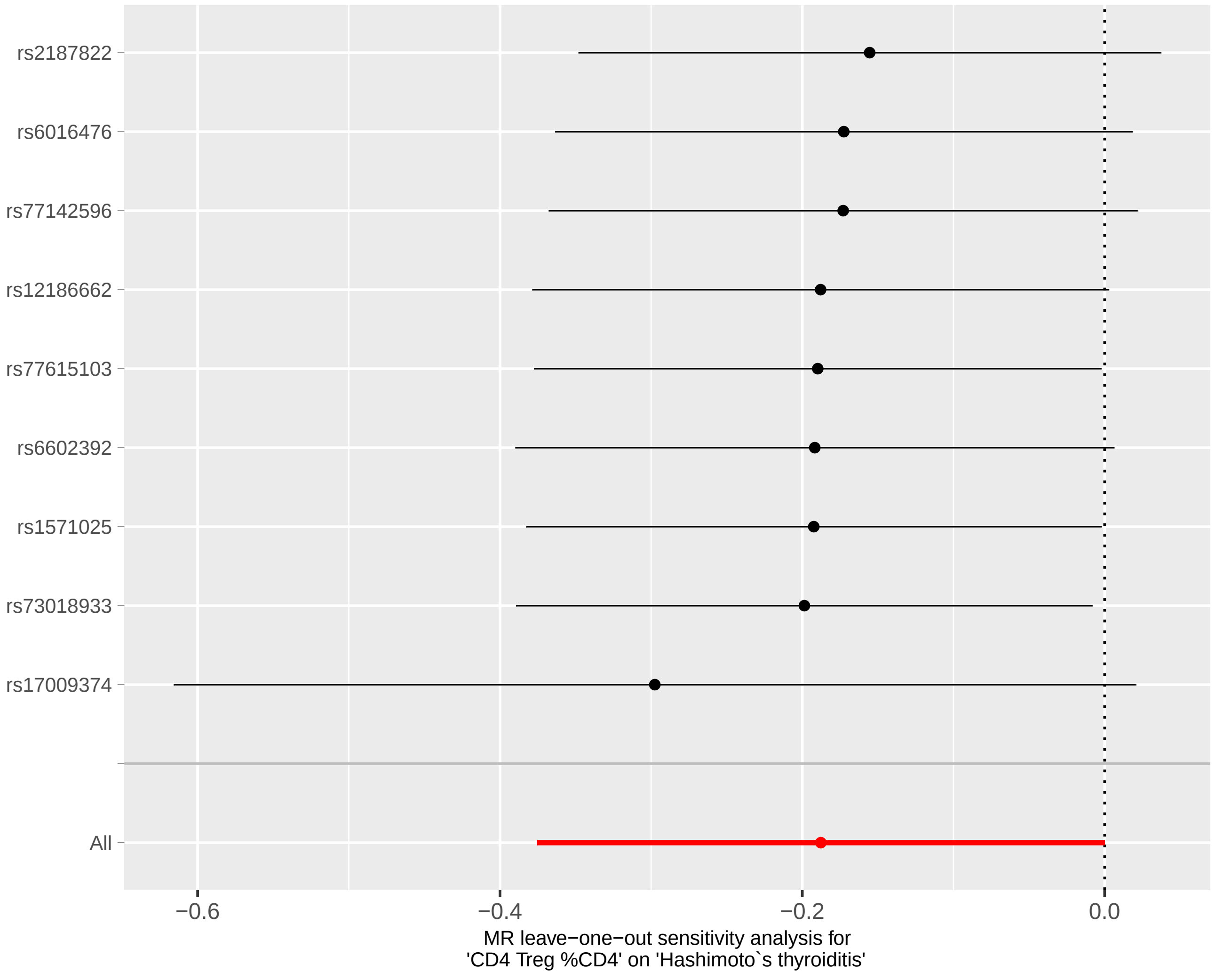

rs2187822
rs6016476
rs77142596
rs12186662
rs77615103
rs6602392
rs1571025
rs73018933
rs17009374
All
−0.6
−0.4
−0.2
0.0
MR leave−one−out sensitivity analysis for
'CD4 Treg %CD4' on 'Hashimoto`s thyroiditis'

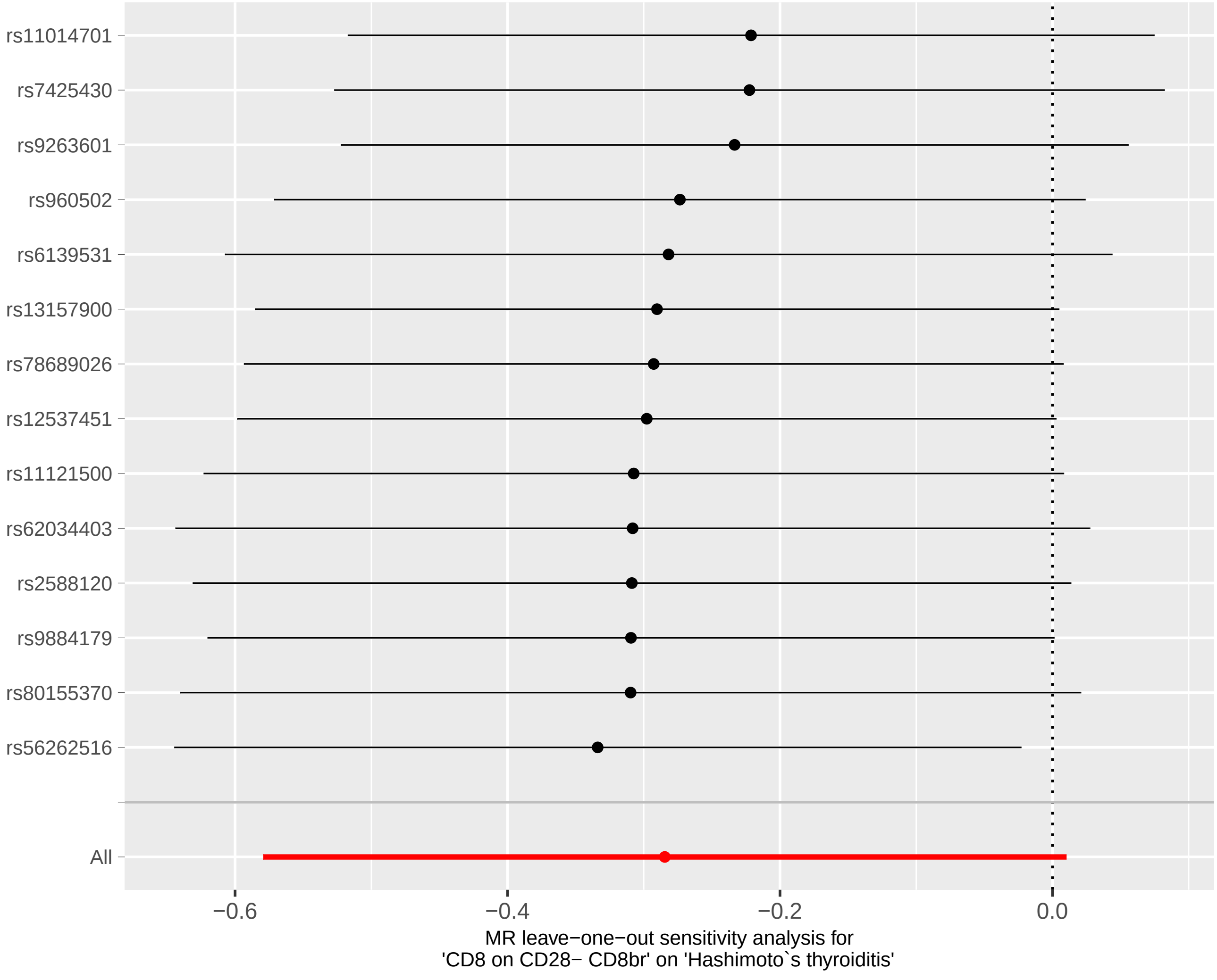
rs11014701
rs7425430
rs9263601
rs960502
rs6139531
rs13157900
rs78689026
rs12537451
rs11121500
rs62034403
rs2588120
rs9884179
rs80155370
rs56262516
All
−0.6
−0.4
−0.2
0.0
MR leave−one−out sensitivity analysis for
'CD8 on CD28− CD8br' on 'Hashimoto`s thyroiditis'

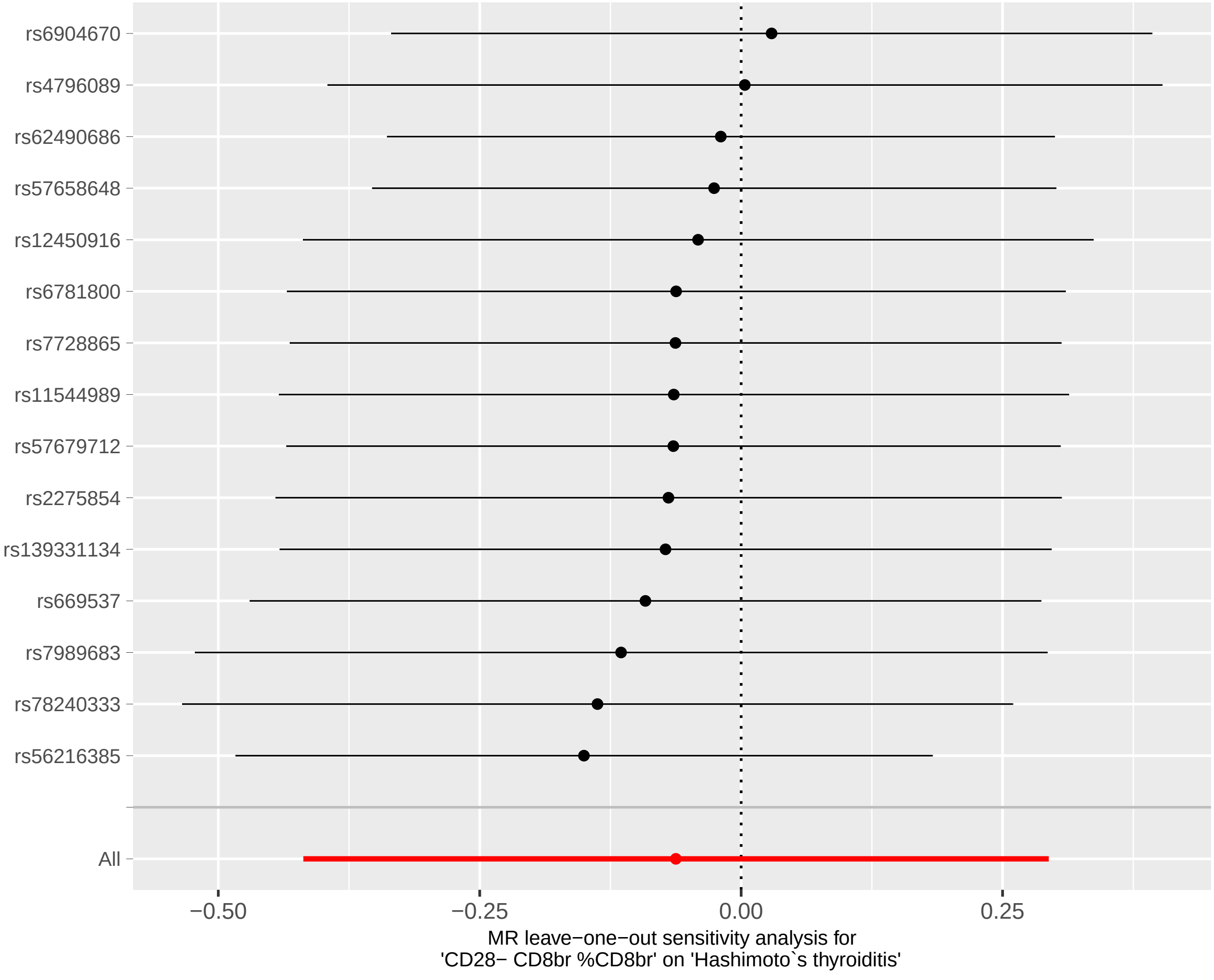

rs6904670
rs4796089
rs62490686
rs57658648
rs12450916
rs6781800
rs7728865
rs11544989
rs57679712
rs2275854
rs139331134
rs669537
rs7989683
rs78240333
rs56216385
All
−0.50
−0.25
0.00
0.25
MR leave−one−out sensitivity analysis for
'CD28− CD8br %CD8br' on 'Hashimoto`s thyroiditis'

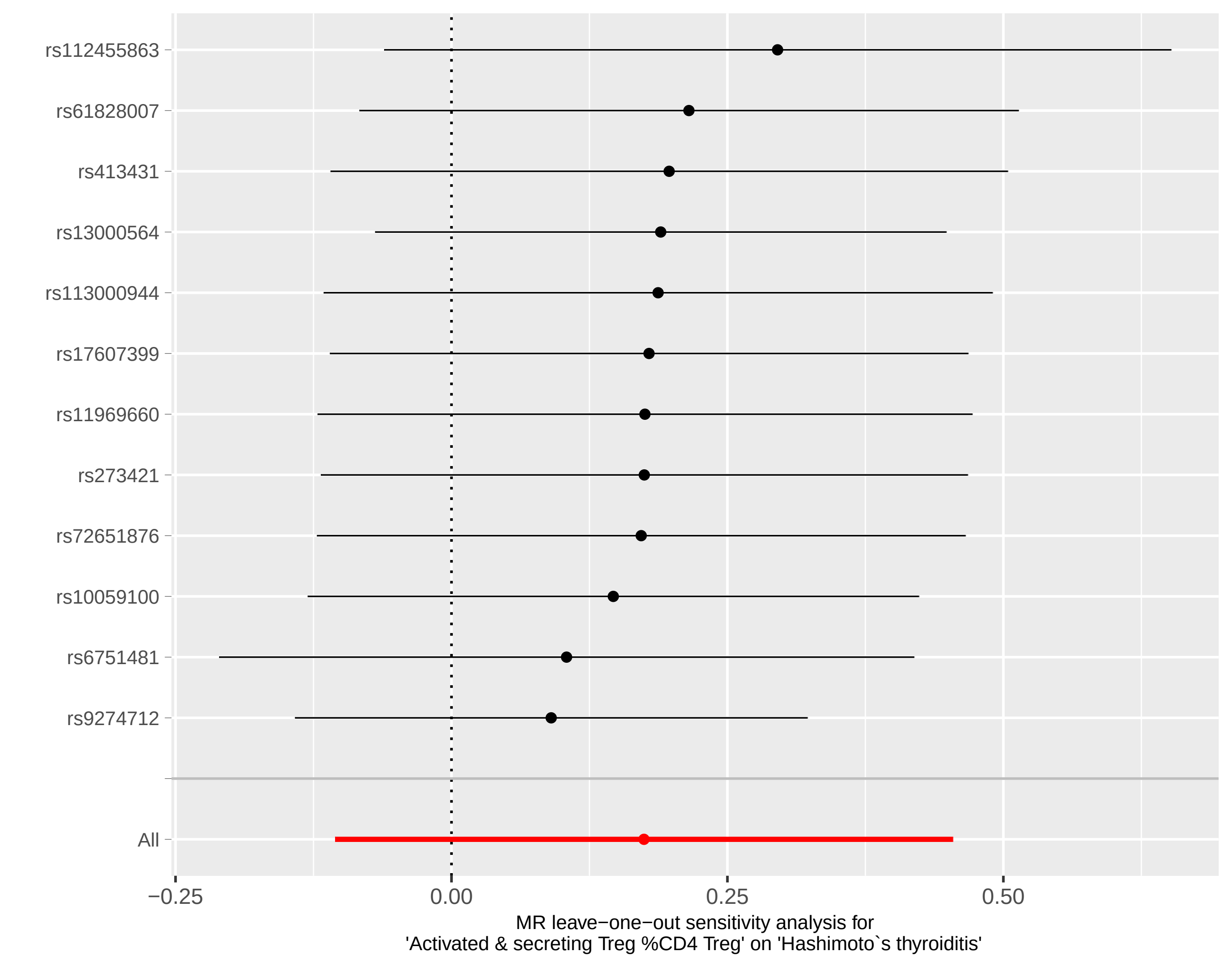
rs112455863
rs61828007
rs413431
rs13000564
rs113000944
rs17607399
rs11969660
rs273421
rs72651876
rs10059100
rs6751481
rs9274712
All
−0.25
0.00
0.25
0.50
MR leave−one−out sensitivity analysis for
'Activated & secreting Treg %CD4 Treg' on 'Hashimoto`s thyroiditis'

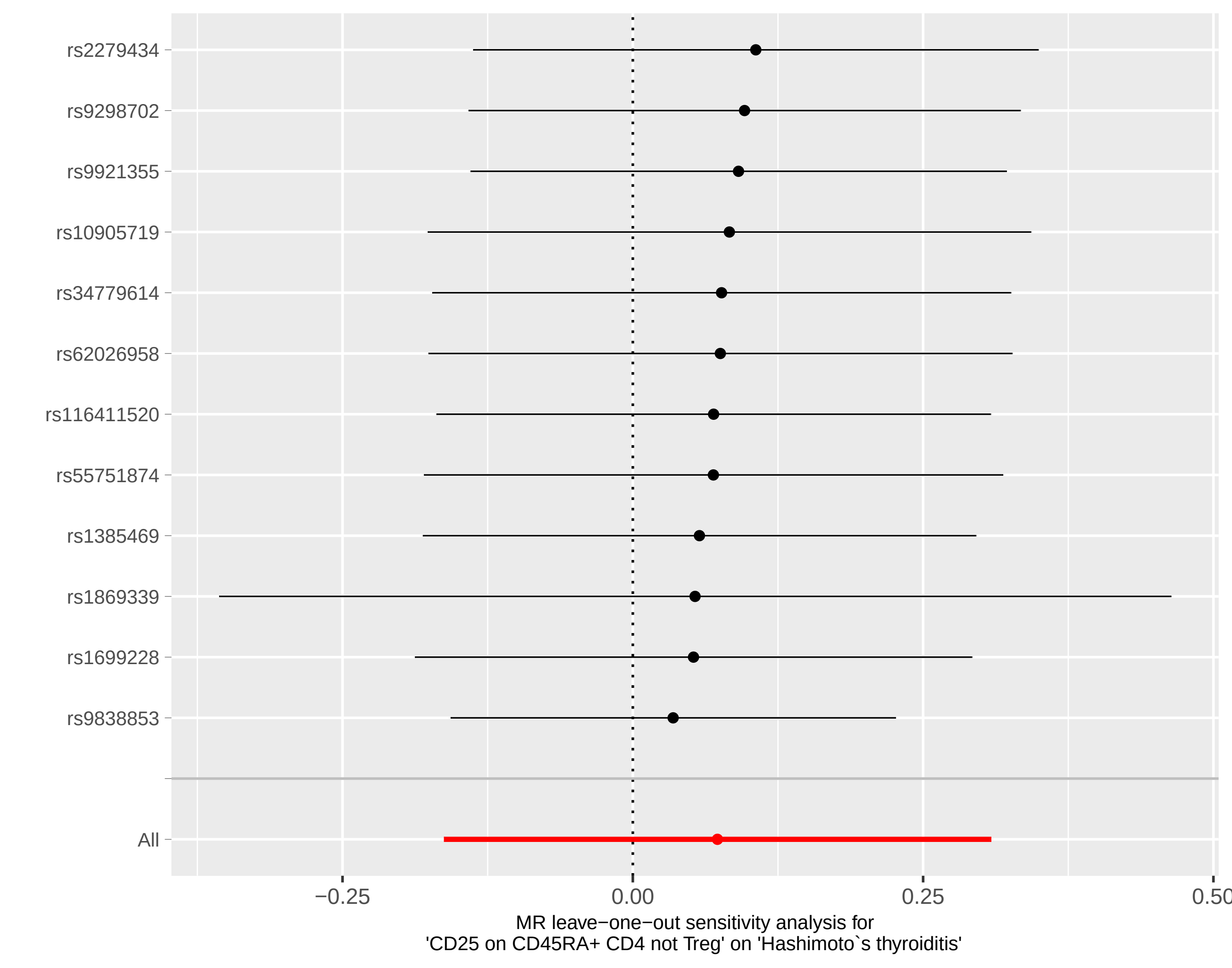
rs2279434
rs9298702
rs9921355
rs10905719
rs34779614
rs62026958
rs116411520
rs55751874
rs1385469
rs1869339
rs1699228
rs9838853
All
−0.25
0.00
0.25
0.50
MR leave−one−out sensitivity analysis for
'CD25 on CD45RA+ CD4 not Treg' on 'Hashimoto`s thyroiditis'

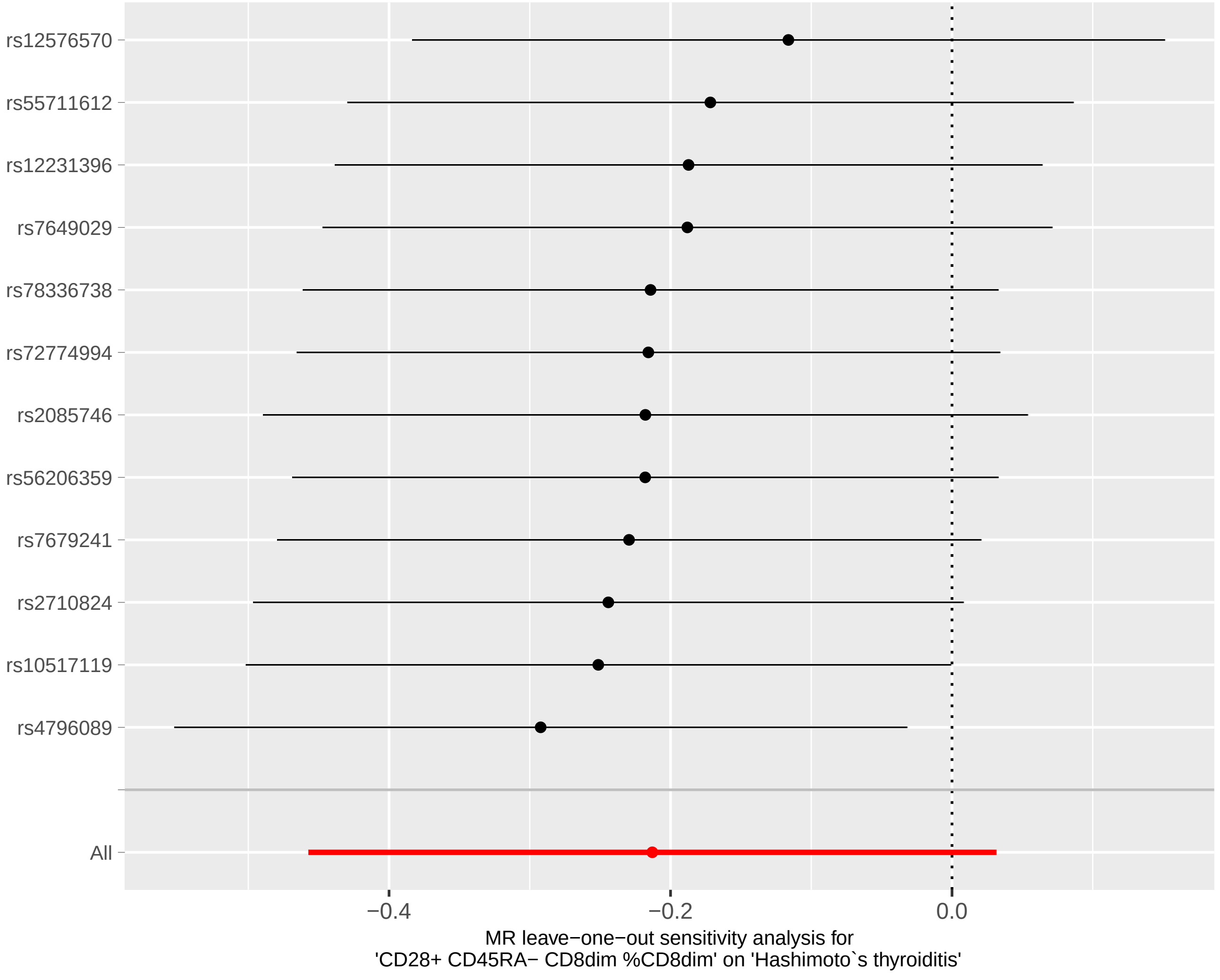
rs12576570
rs55711612
rs12231396
rs7649029
rs78336738
rs72774994
rs2085746
rs56206359
rs7679241
rs2710824
rs10517119
rs4796089
All
−0.4
−0.2
0.0
MR leave−one−out sensitivity analysis for
'CD28+ CD45RA− CD8dim %CD8dim' on 'Hashimoto`s thyroiditis'

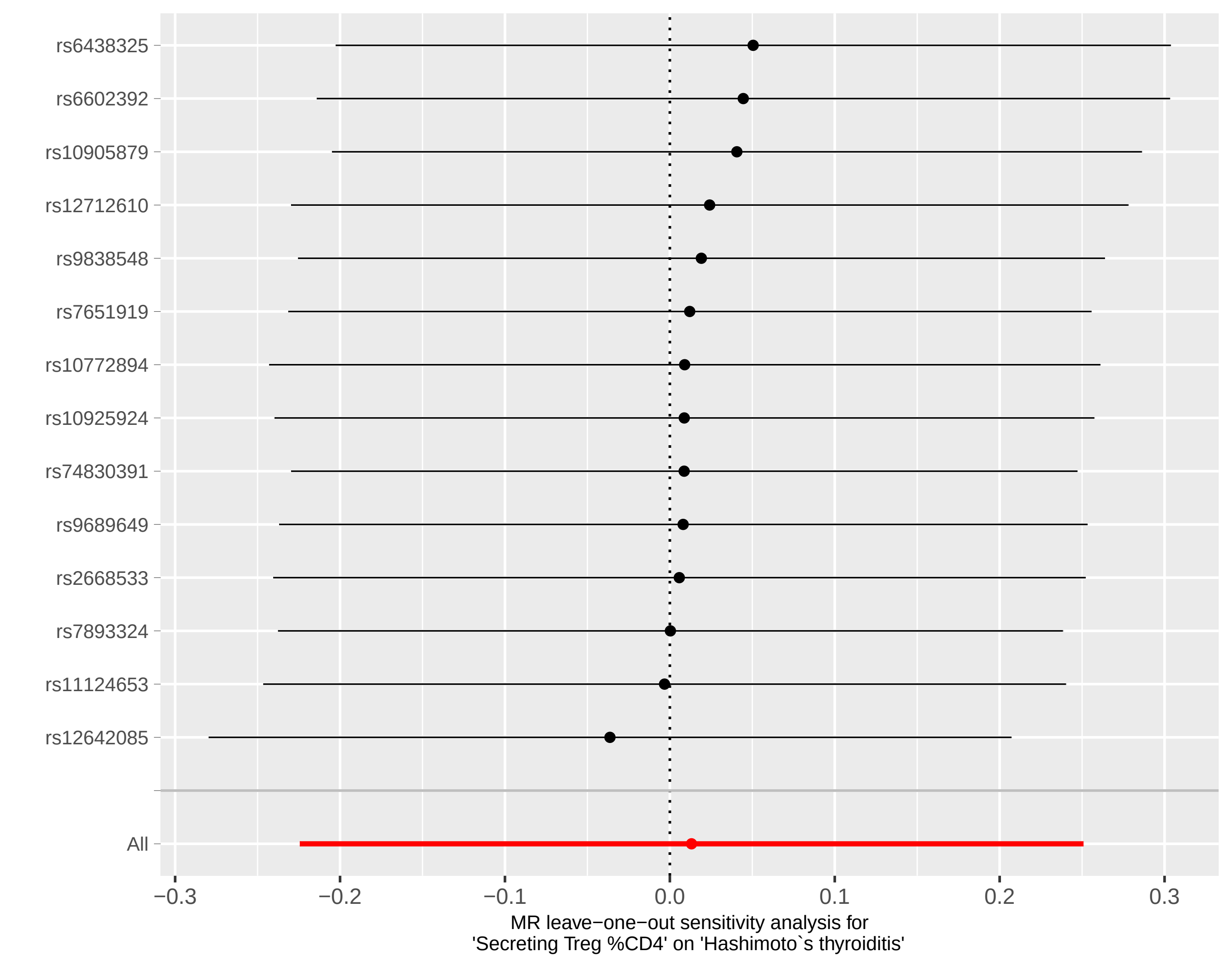

rs6438325
rs6602392
rs10905879
rs12712610
rs9838548
rs7651919
rs10772894
rs10925924
rs74830391
rs9689649
rs2668533
rs7893324
rs11124653
rs12642085
All
−0.3
−0.2
−0.1
0.0
0.1
0.2
0.3
MR leave−one−out sensitivity analysis for
'Secreting Treg %CD4' on 'Hashimoto`s thyroiditis'

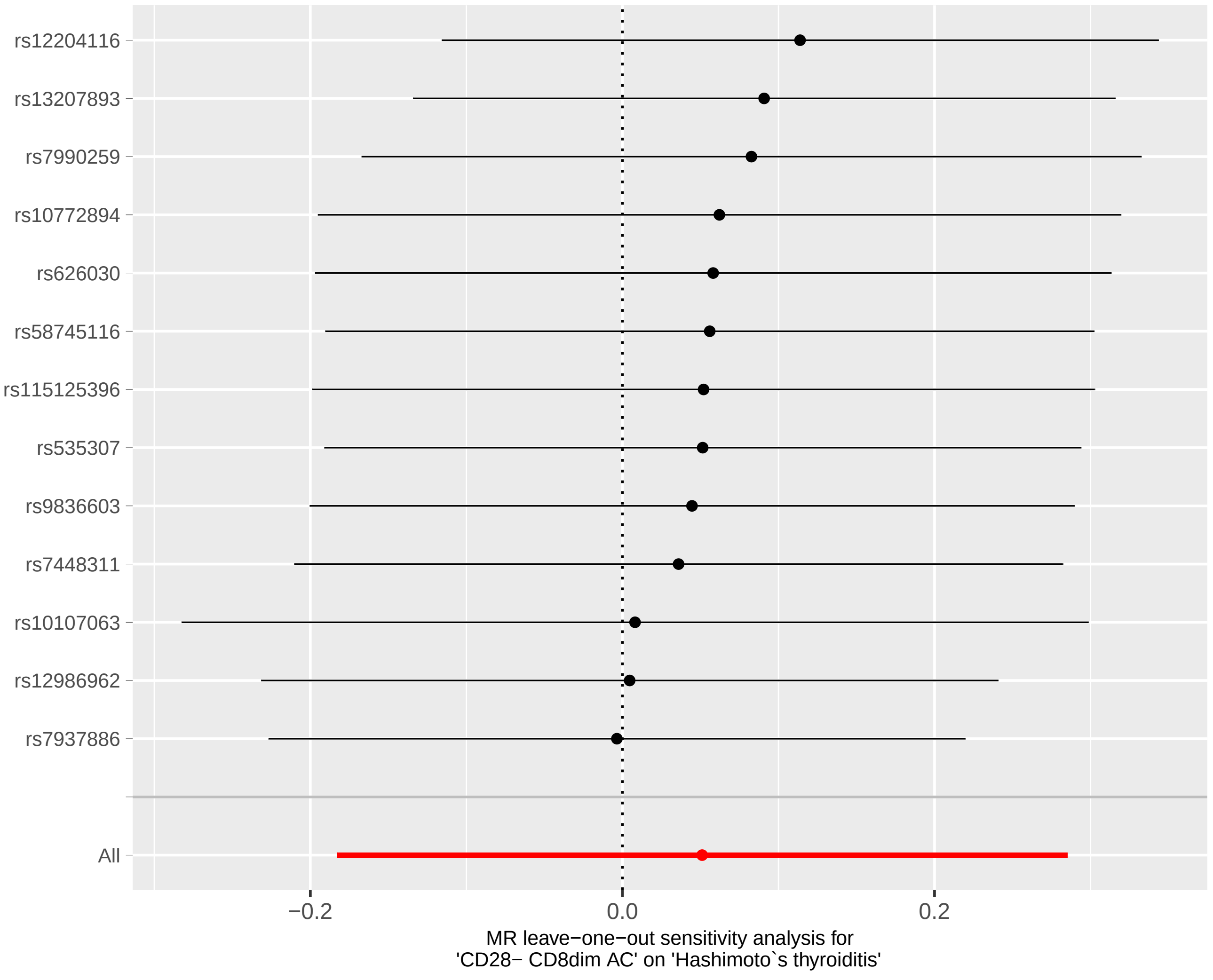

rs12204116
rs13207893
rs7990259
rs10772894
rs626030
rs58745116
rs115125396
rs535307
rs9836603
rs7448311
rs10107063
rs12986962
rs7937886
All
−0.2
0.0
0.2
MR leave−one−out sensitivity analysis for
'CD28− CD8dim AC' on 'Hashimoto`s thyroiditis'

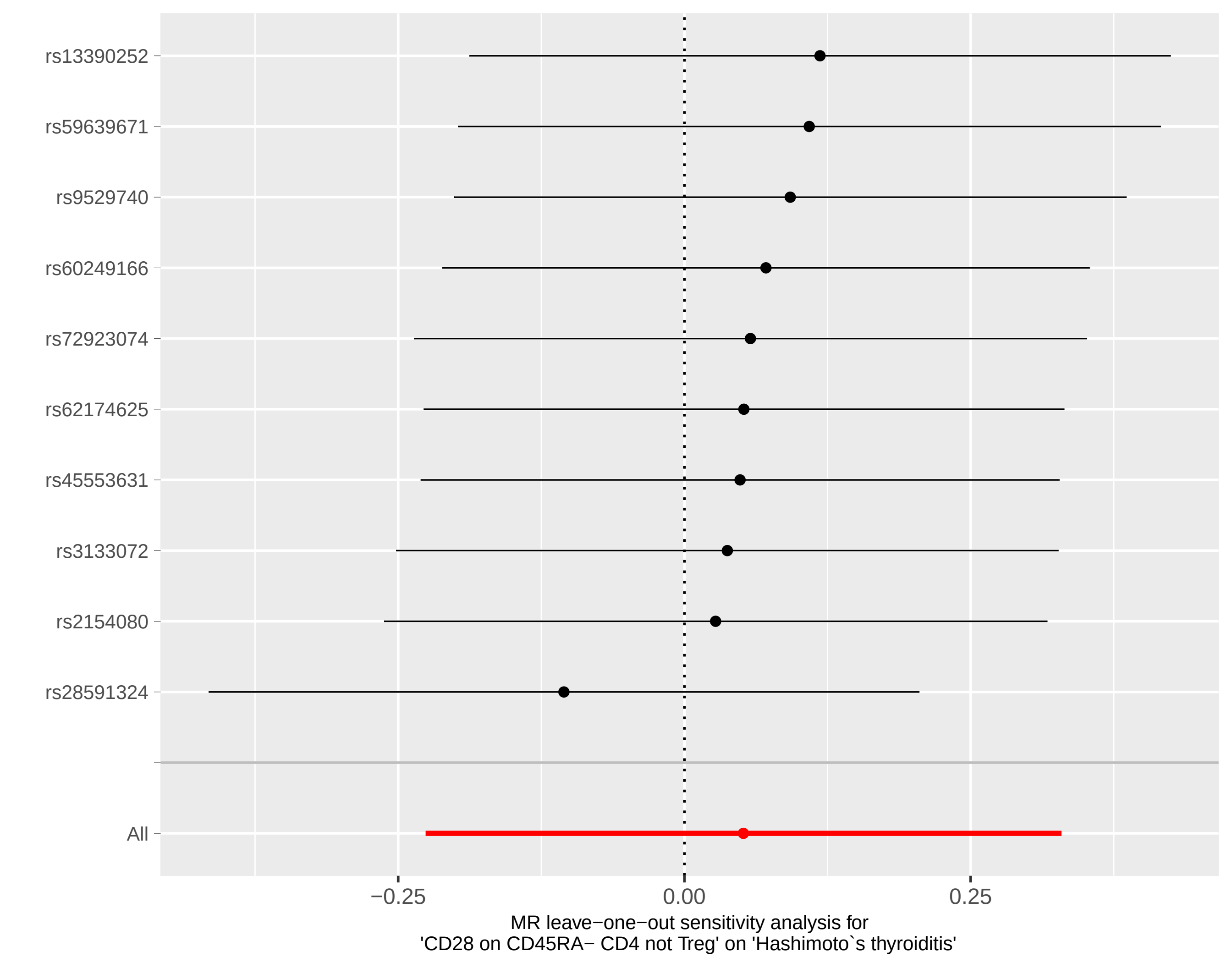

rs13390252
rs59639671
rs9529740
rs60249166
rs72923074
rs62174625
rs45553631
rs3133072
rs2154080
rs28591324
All
−0.25
0.00
0.25
MR leave−one−out sensitivity analysis for
'CD28 on CD45RA− CD4 not Treg' on 'Hashimoto`s thyroiditis'

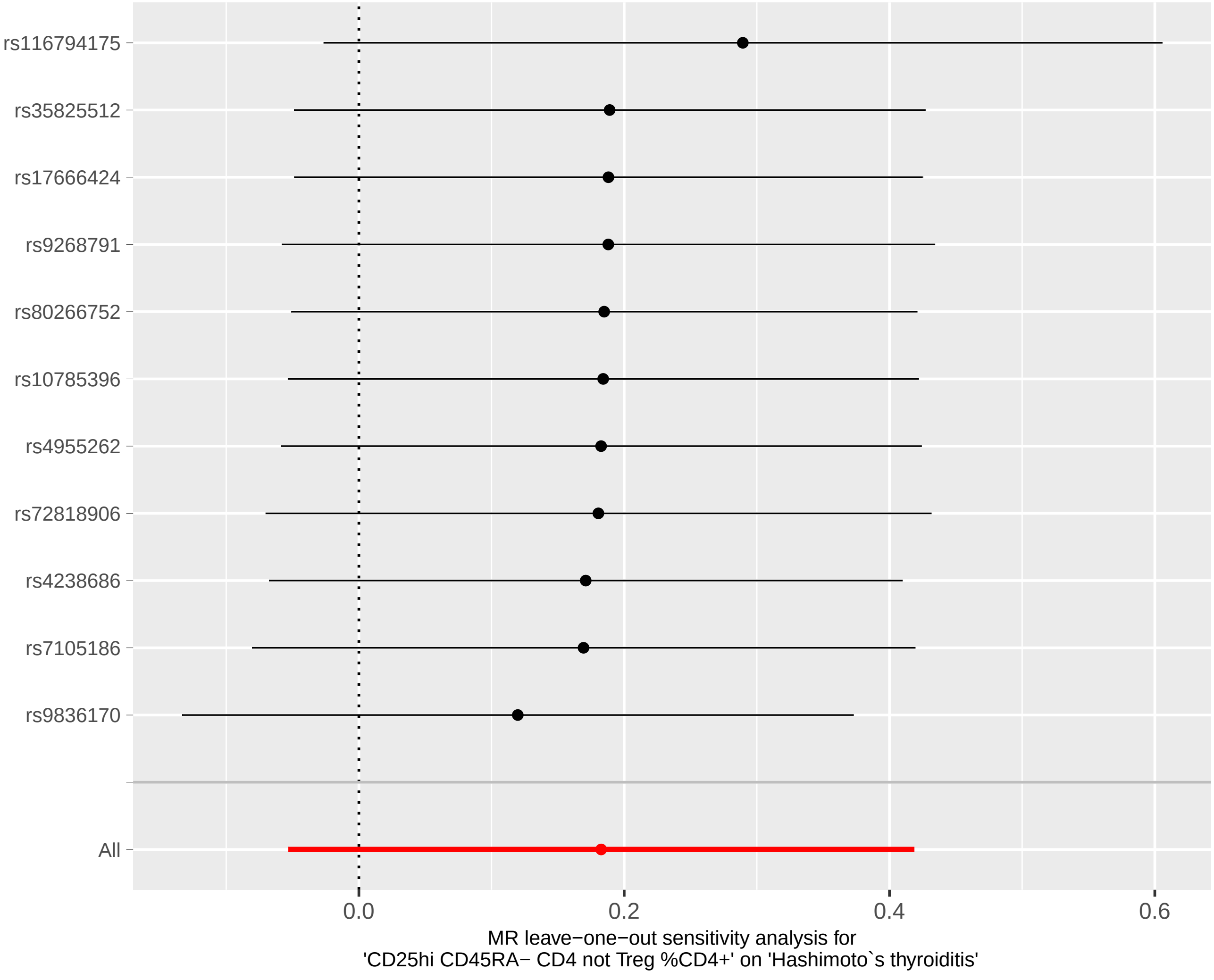
rs116794175
rs35825512
rs17666424
rs9268791
rs80266752
rs10785396
rs4955262
rs72818906
rs4238686
rs7105186
rs9836170
All
0.0
0.2
0.4
0.6
MR leave−one−out sensitivity analysis for
'CD25hi CD45RA− CD4 not Treg %CD4+' on 'Hashimoto`s thyroiditis'

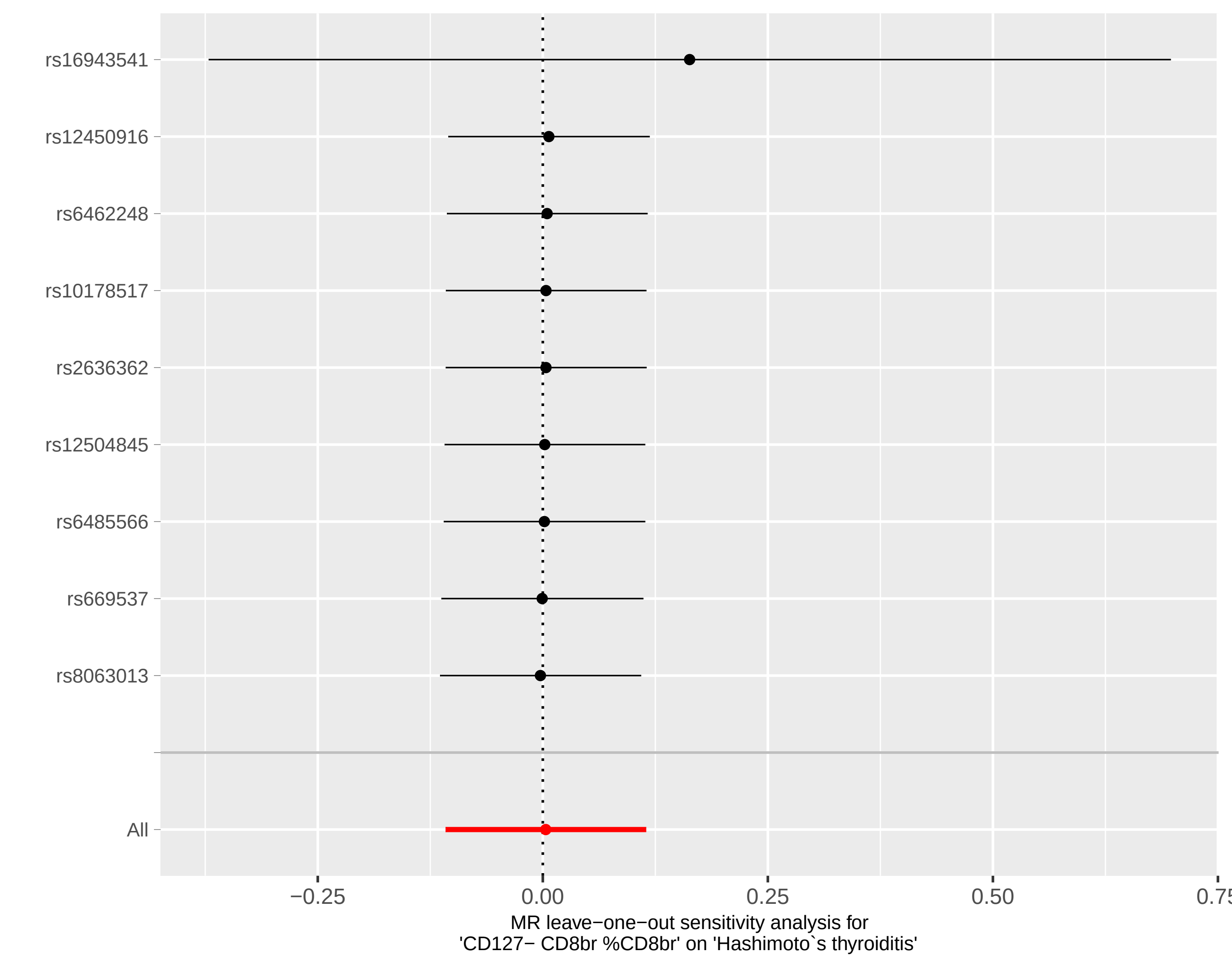

rs16943541
rs12450916
rs6462248
rs10178517
rs2636362
rs12504845
rs6485566
rs669537
rs8063013
All
−0.25
0.00
0.25
0.50
0.75
MR leave−one−out sensitivity analysis for
'CD127− CD8br %CD8br' on 'Hashimoto`s thyroiditis'

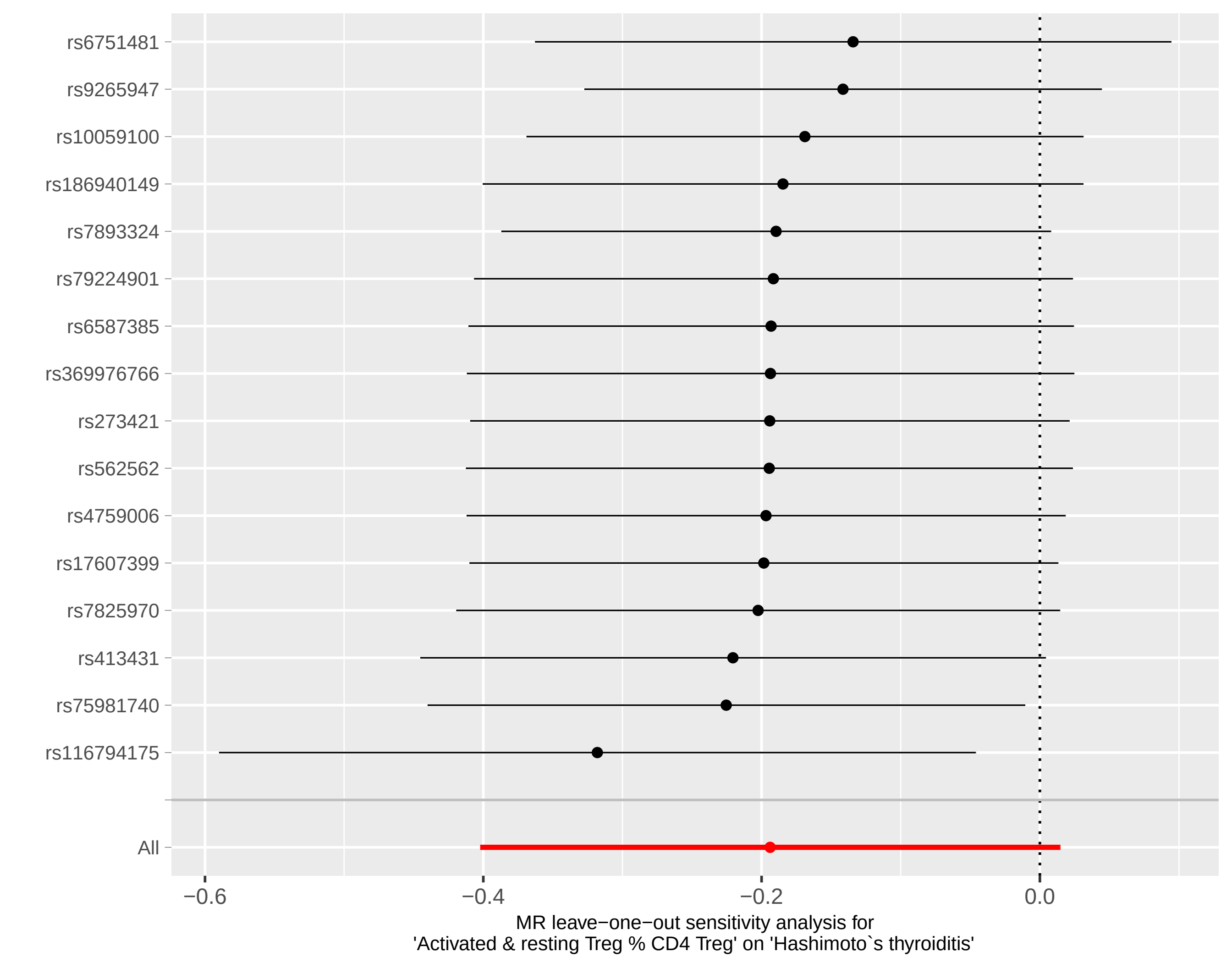
rs6751481
rs9265947
rs10059100
rs186940149
rs7893324
rs79224901
rs6587385
rs369976766
rs273421
rs562562
rs4759006
rs17607399
rs7825970
rs413431
rs75981740
rs116794175
All
−0.6
−0.4
−0.2
0.0
MR leave−one−out sensitivity analysis for
'Activated & resting Treg % CD4 Treg' on 'Hashimoto`s thyroiditis'

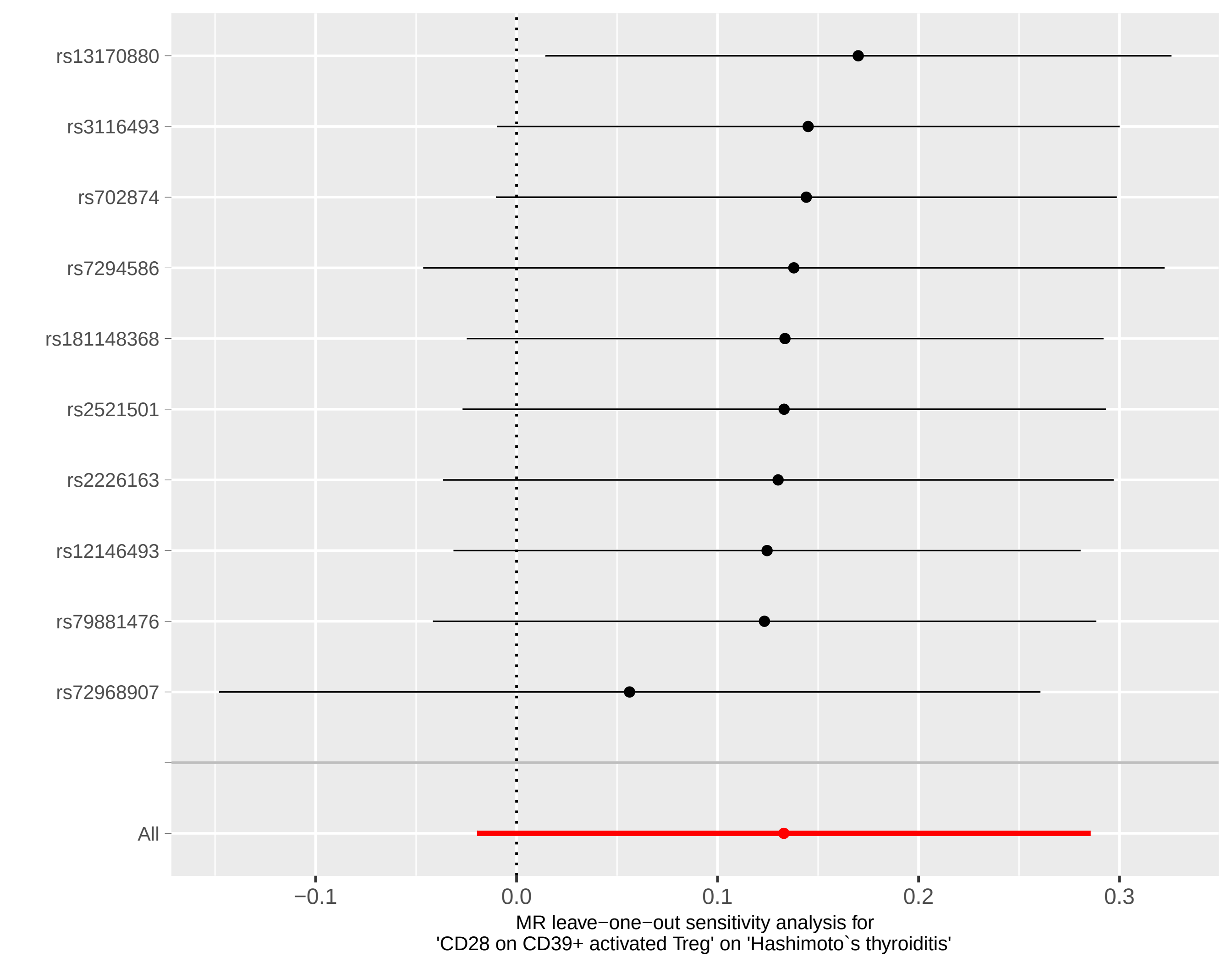
rs13170880
rs3116493
rs702874
rs7294586
rs181148368
rs2521501
rs2226163
rs12146493
rs79881476
rs72968907
All
−0.1
0.0
0.1
0.2
0.3
MR leave−one−out sensitivity analysis for
'CD28 on CD39+ activated Treg' on 'Hashimoto`s thyroiditis'

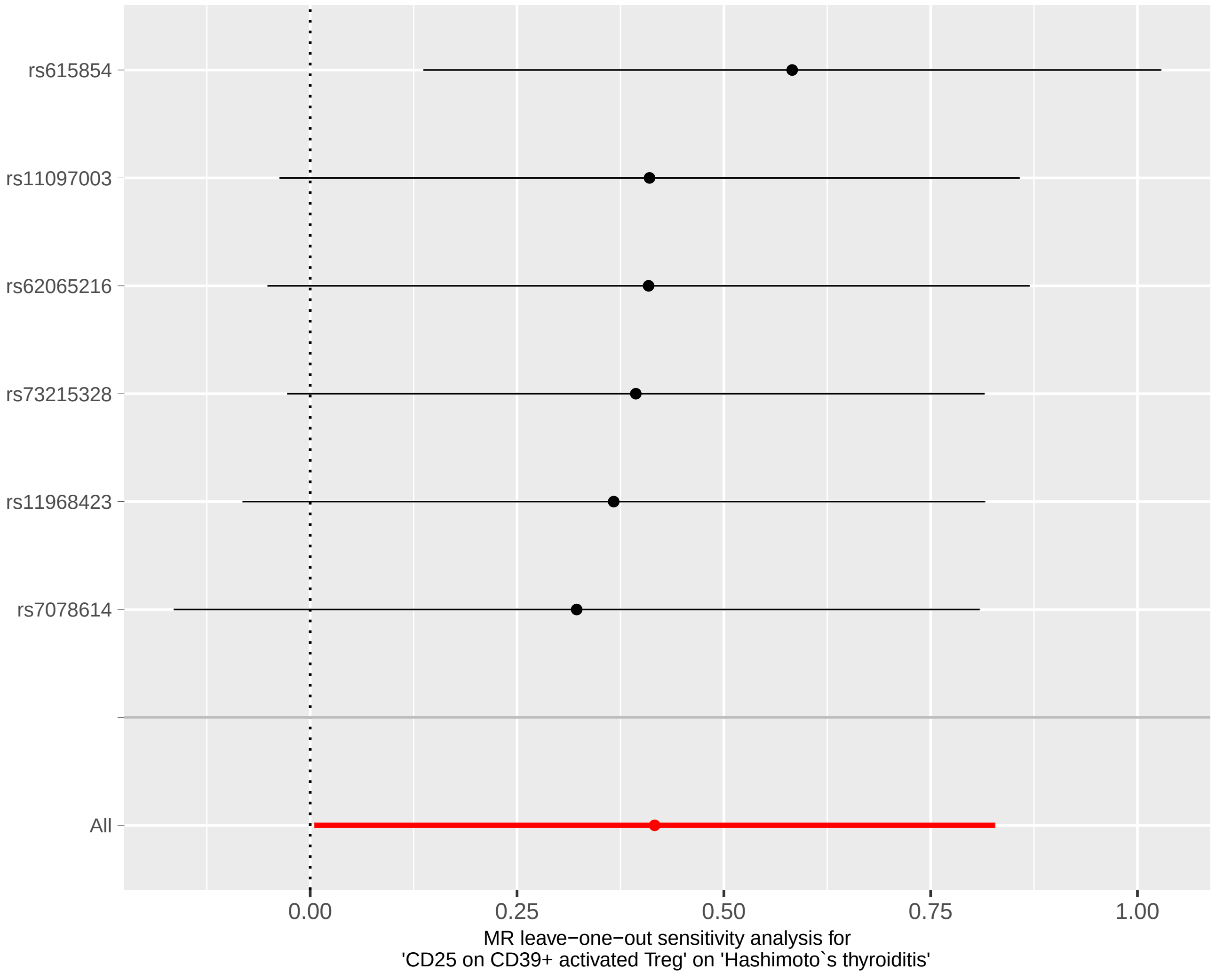
rs615854
rs11097003
rs62065216
rs73215328
rs11968423
rs7078614
All
0.00
0.25
0.50
0.75
1.00
MR leave−one−out sensitivity analysis for
'CD25 on CD39+ activated Treg' on 'Hashimoto`s thyroiditis'

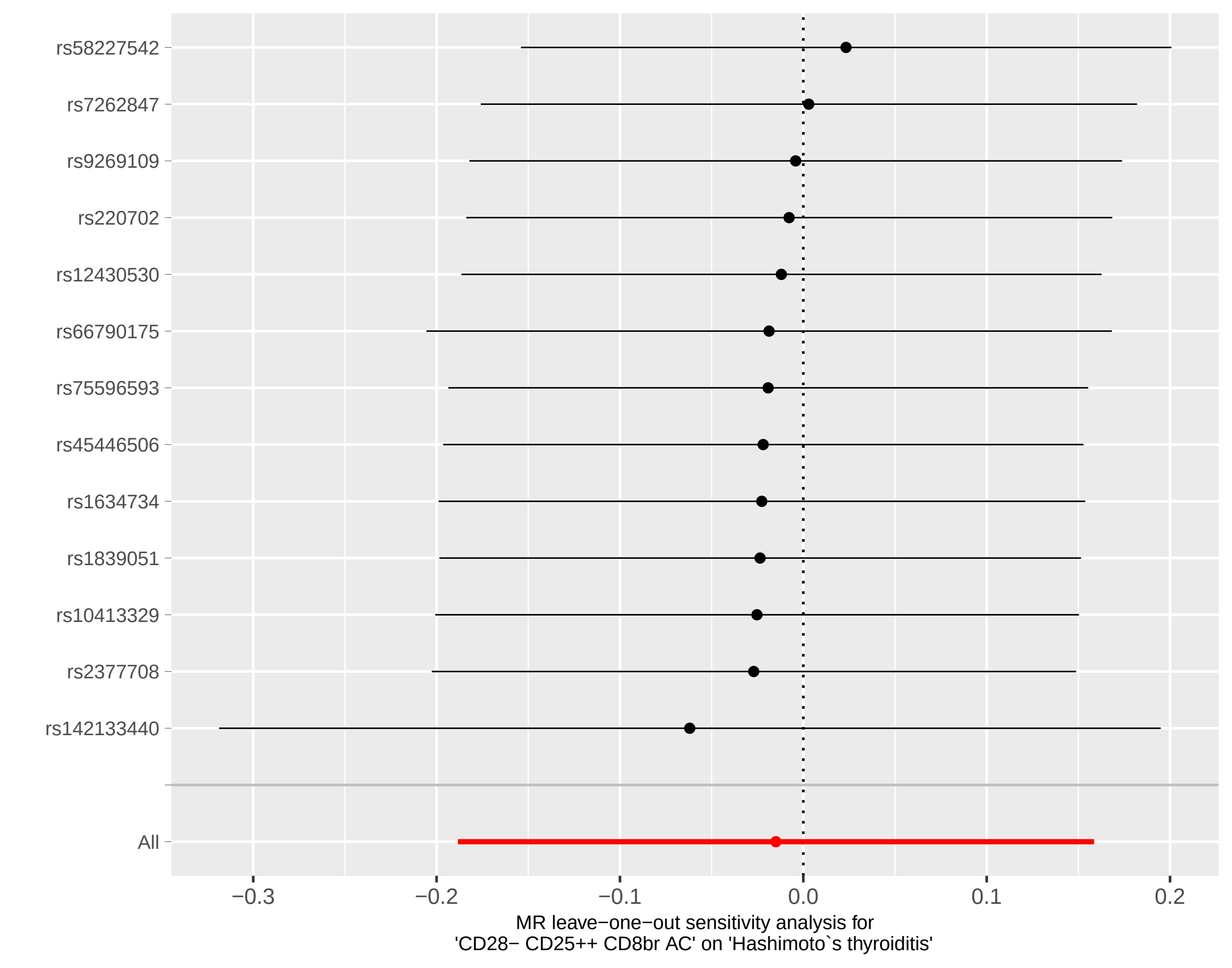
rs58227542
rs7262847
rs9269109
rs220702
rs12430530
rs66790175
rs75596593
rs45446506
rs1634734
rs1839051
rs10413329
rs2377708
rs142133440
All
−0.3
−0.2
−0.1
0.0
0.1
0.2
MR leave−one−out sensitivity analysis for
'CD28− CD25++ CD8br AC' on 'Hashimoto`s thyroiditis'

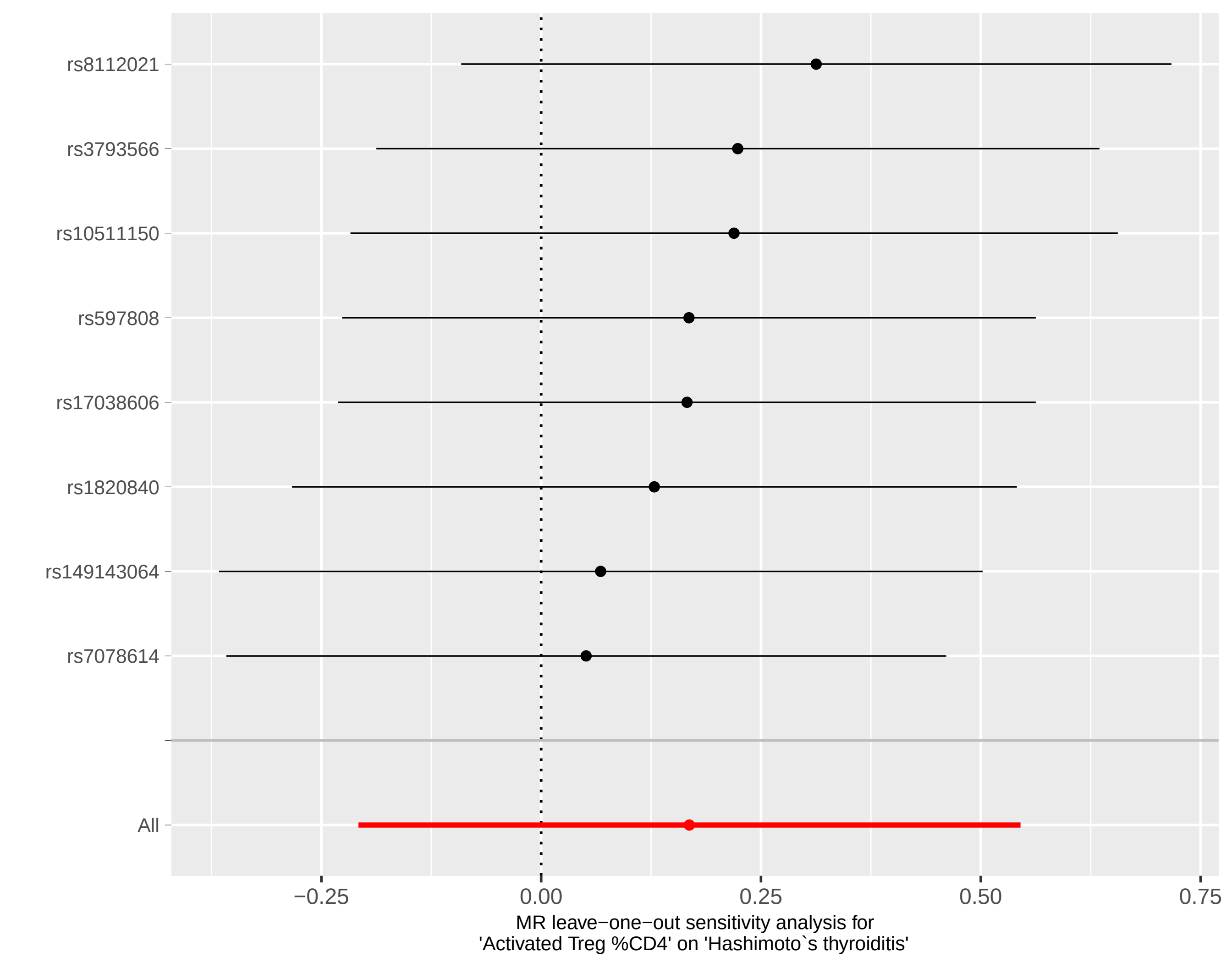
rs8112021
rs3793566
rs10511150
rs597808
rs17038606
rs1820840
rs149143064
rs7078614
All
−0.25
0.00
0.25
0.50
0.75
MR leave−one−out sensitivity analysis for
'Activated Treg %CD4' on 'Hashimoto`s thyroiditis'

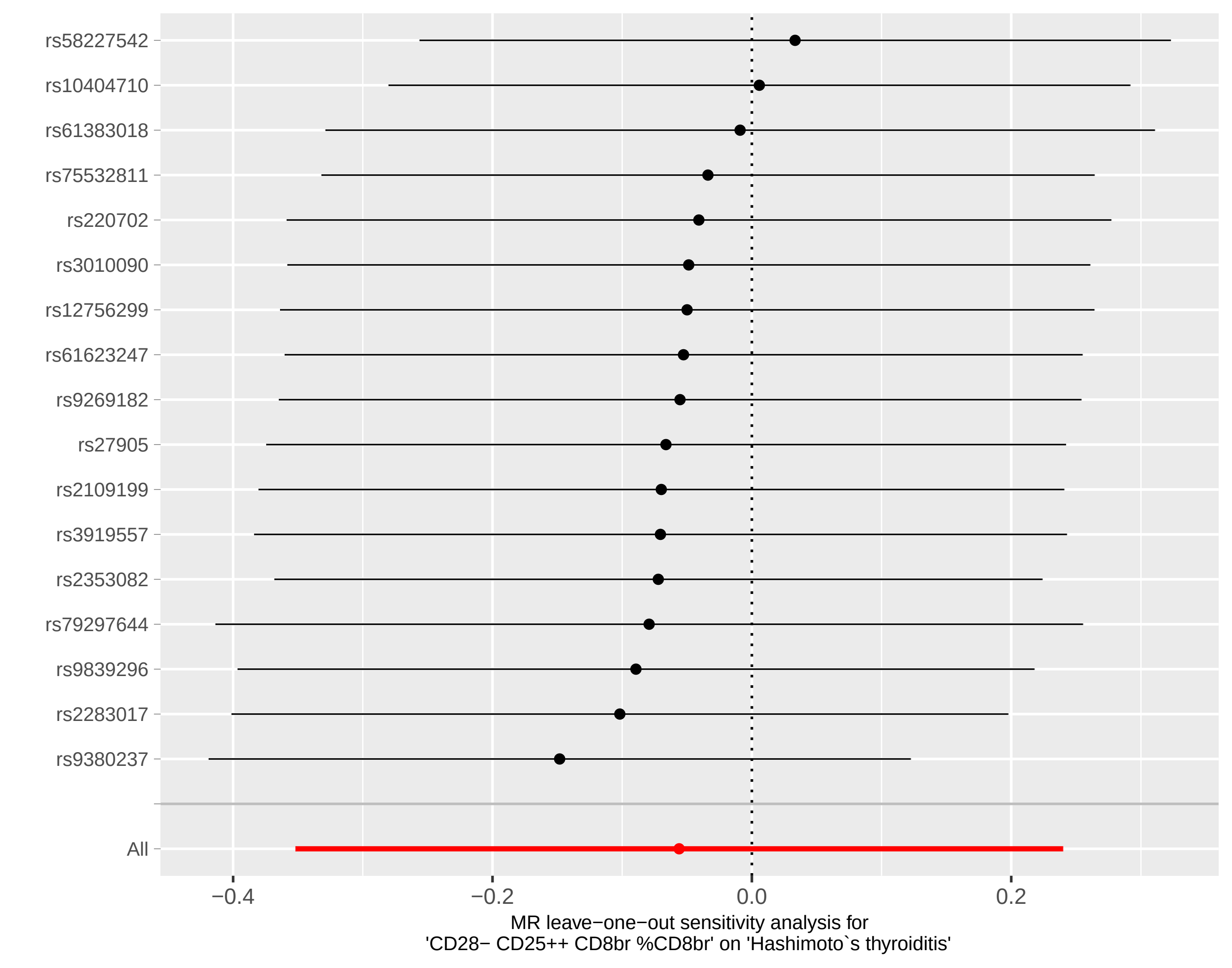
rs58227542
rs10404710
rs61383018
rs75532811
rs220702
rs3010090
rs12756299
rs61623247
rs9269182
rs27905
rs2109199
rs3919557
rs2353082
rs79297644
rs9839296
rs2283017
rs9380237
All
−0.4
−0.2
0.0
0.2
MR leave−one−out sensitivity analysis for
'CD28− CD25++ CD8br %CD8br' on 'Hashimoto`s thyroiditis'

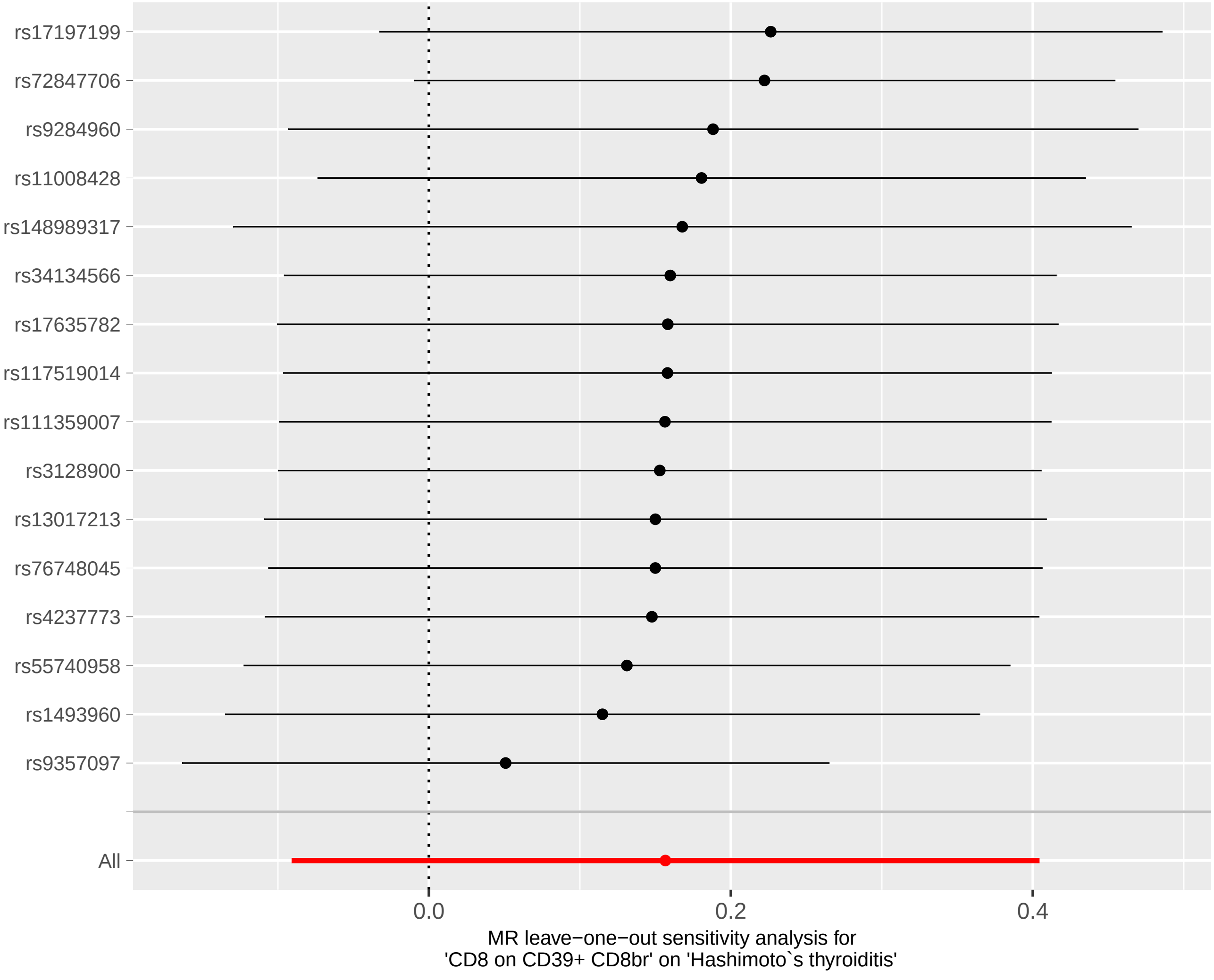

rs17197199
rs72847706
rs9284960
rs11008428
rs148989317
rs34134566
rs17635782
rs117519014
rs111359007
rs3128900
rs13017213
rs76748045
rs4237773
rs55740958
rs1493960
rs9357097
All
0.0
0.2
0.4
MR leave−one−out sensitivity analysis for
'CD8 on CD39+ CD8br' on 'Hashimoto`s thyroiditis'

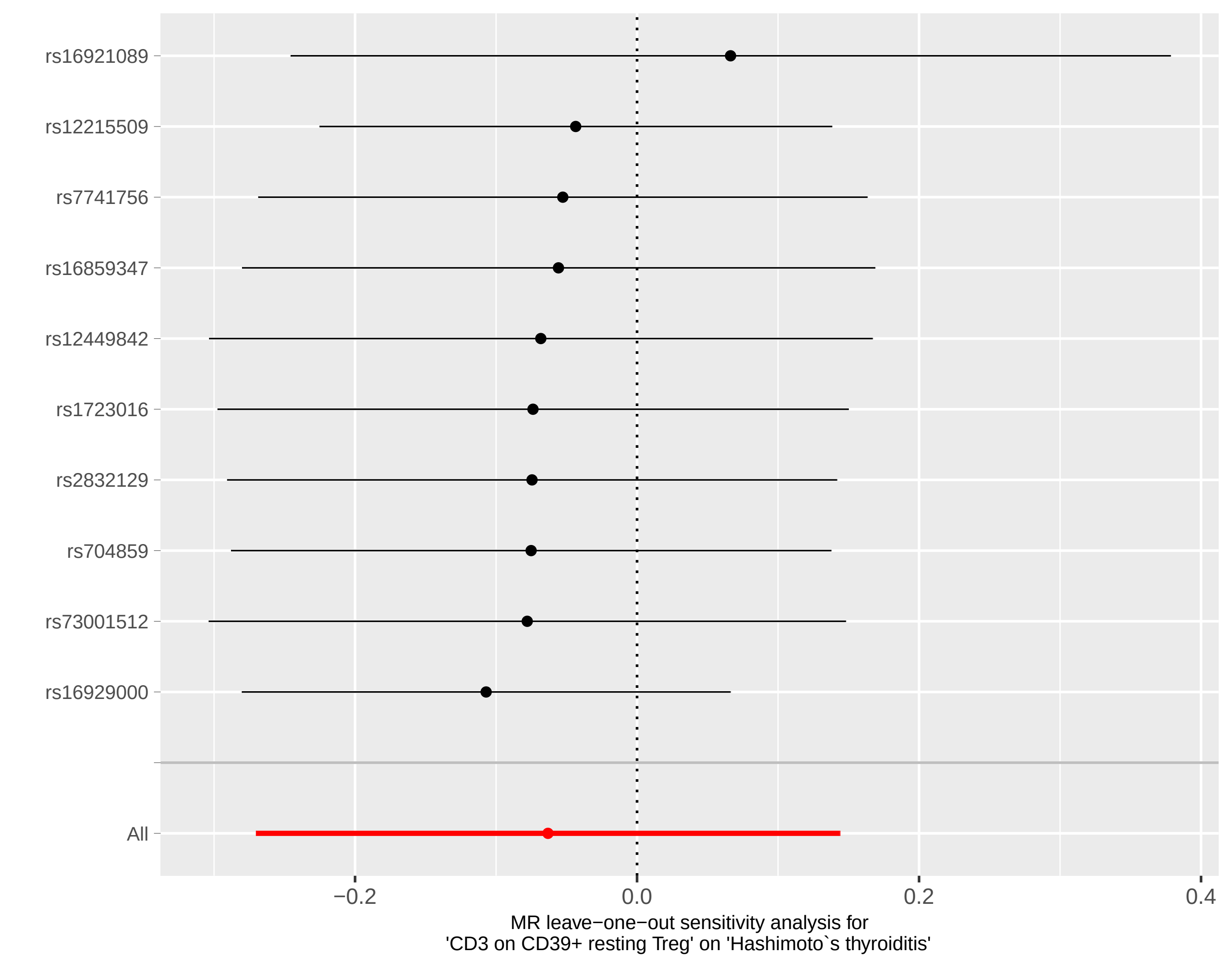
rs16921089
rs12215509
rs7741756
rs16859347
rs12449842
rs1723016
rs2832129
rs704859
rs73001512
rs16929000
All
−0.2
0.0
0.2
0.4
MR leave−one−out sensitivity analysis for
'CD3 on CD39+ resting Treg' on 'Hashimoto`s thyroiditis'

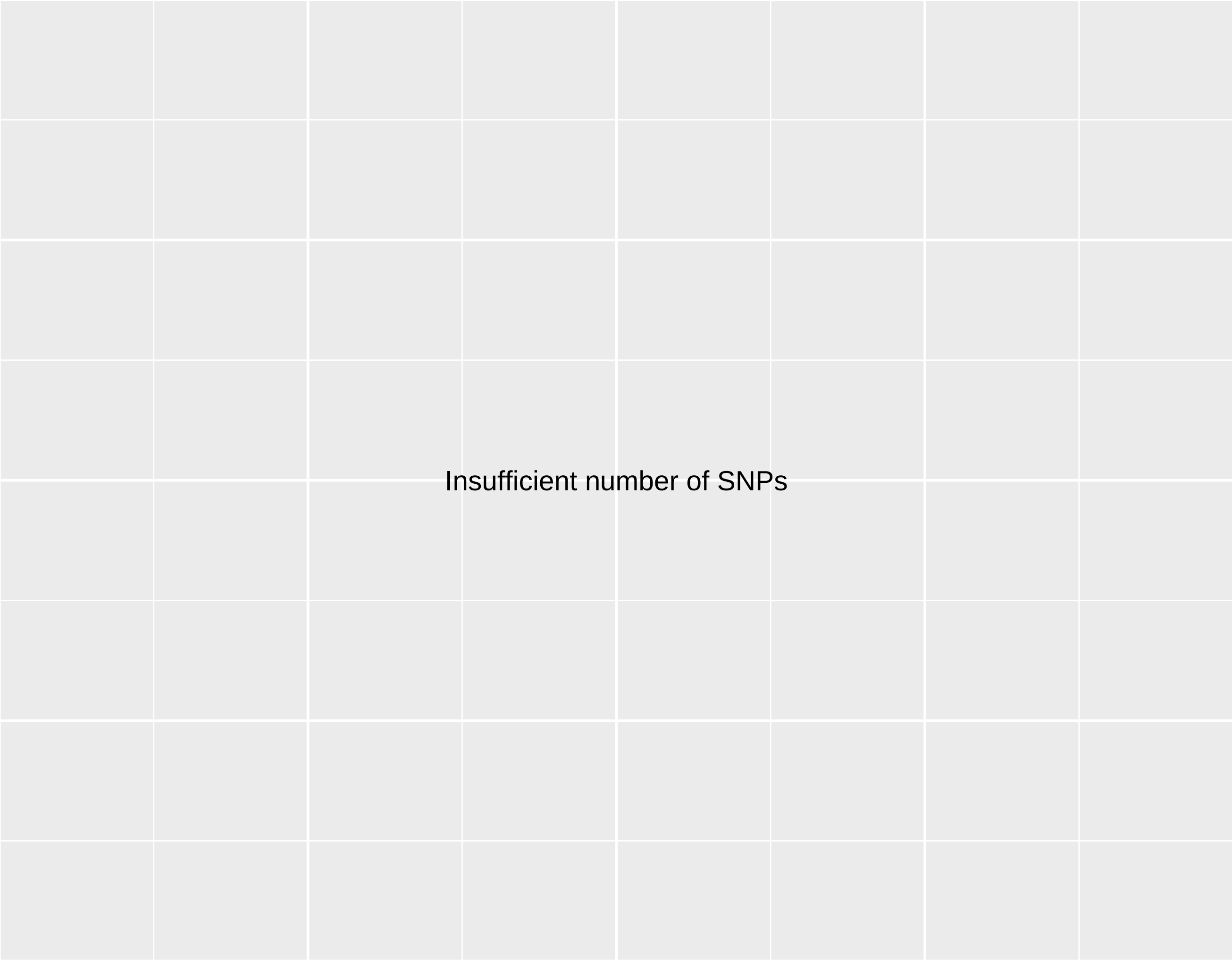
Insufficient number of SNPs

Insufficient number of SNPs

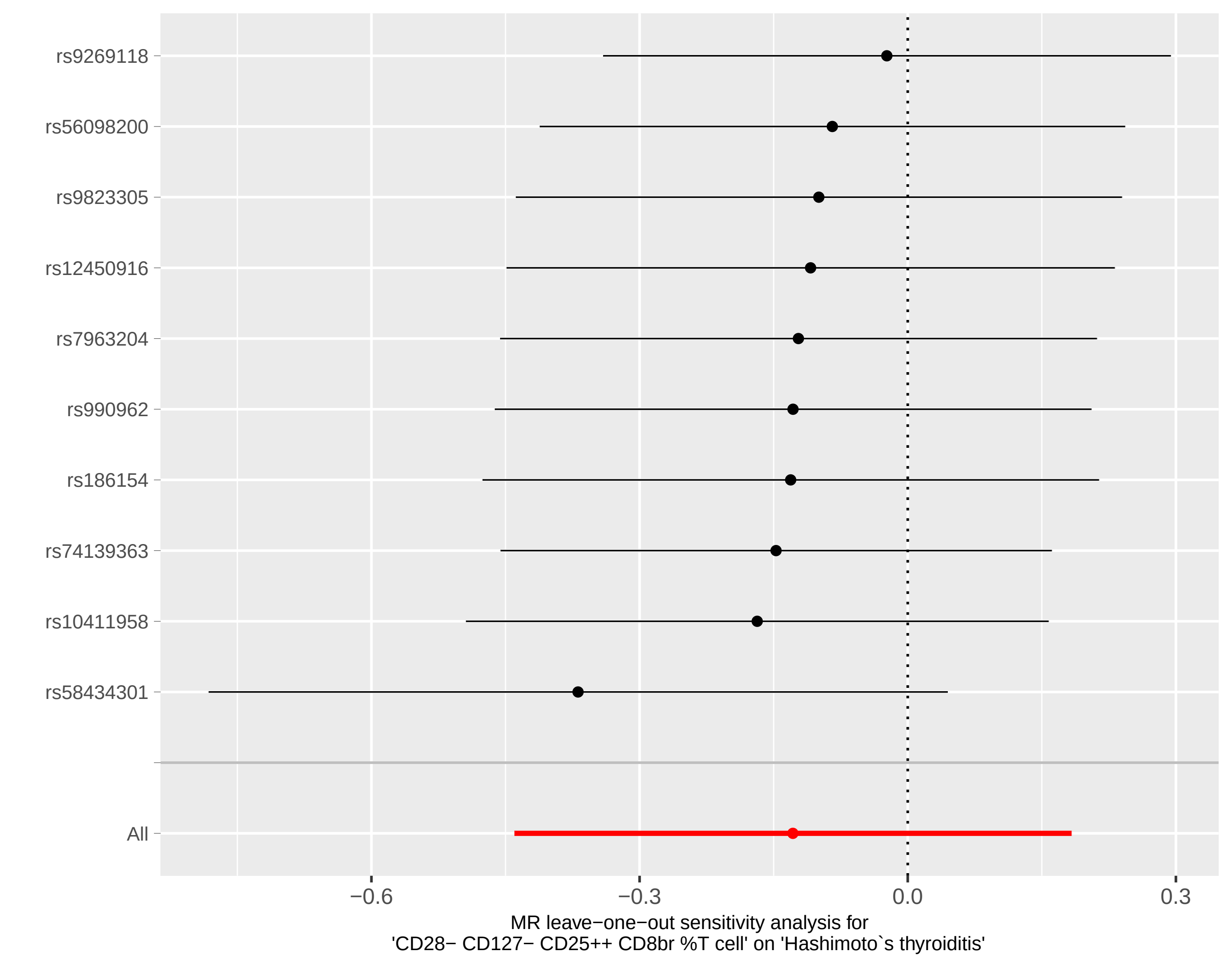
rs9269118
rs56098200
rs9823305
rs12450916
rs7963204
rs990962
rs186154
rs74139363
rs10411958
rs58434301
All
−0.6
−0.3
0.0
0.3
MR leave−one−out sensitivity analysis for
'CD28− CD127− CD25++ CD8br %T cell' on 'Hashimoto`s thyroiditis'

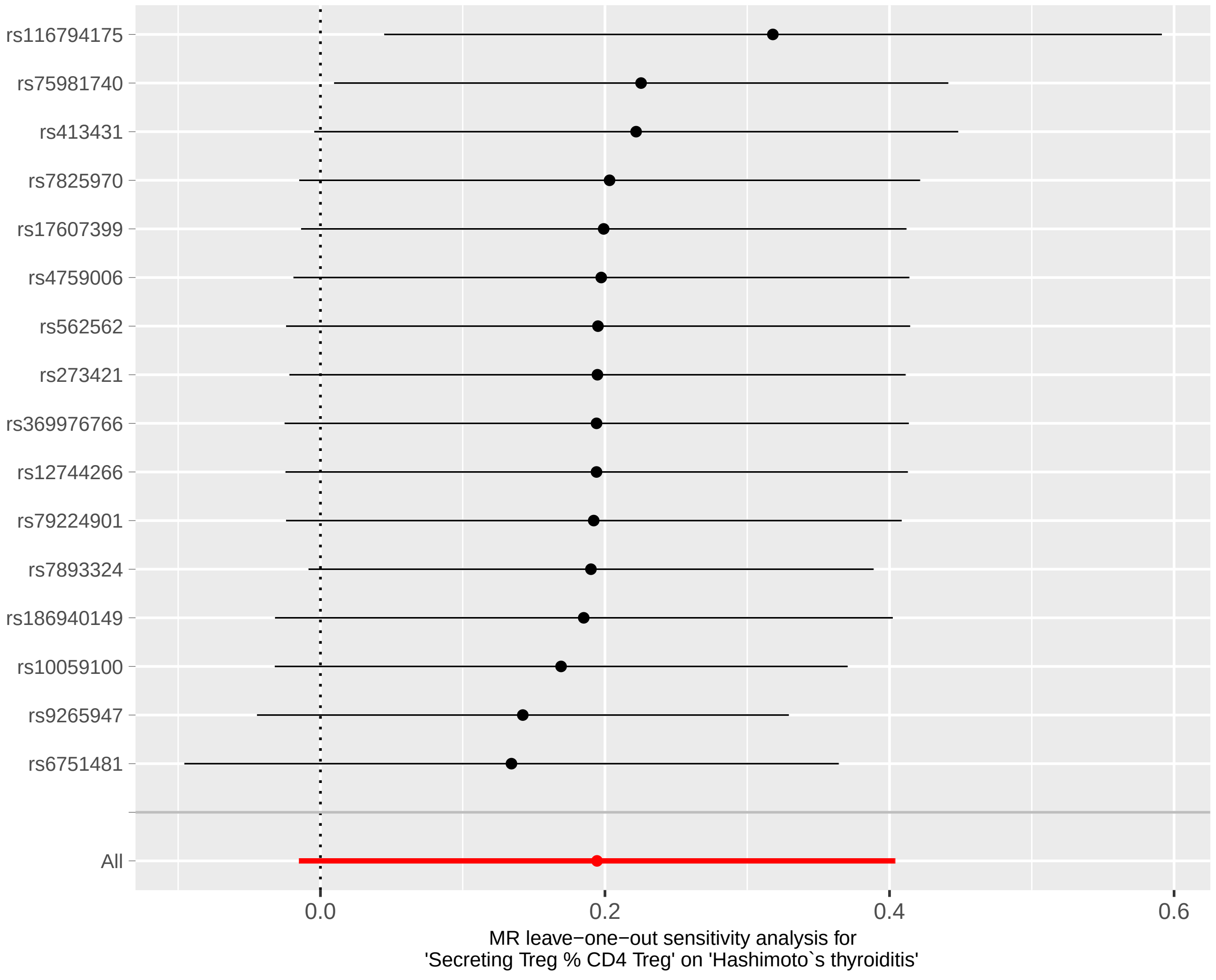
rs116794175
rs75981740
rs413431
rs7825970
rs17607399
rs4759006
rs562562
rs273421
rs369976766
rs12744266
rs79224901
rs7893324
rs186940149
rs10059100
rs9265947
rs6751481
All
0.0
0.2
0.4
0.6
MR leave−one−out sensitivity analysis for
'Secreting Treg % CD4 Treg' on 'Hashimoto`s thyroiditis'

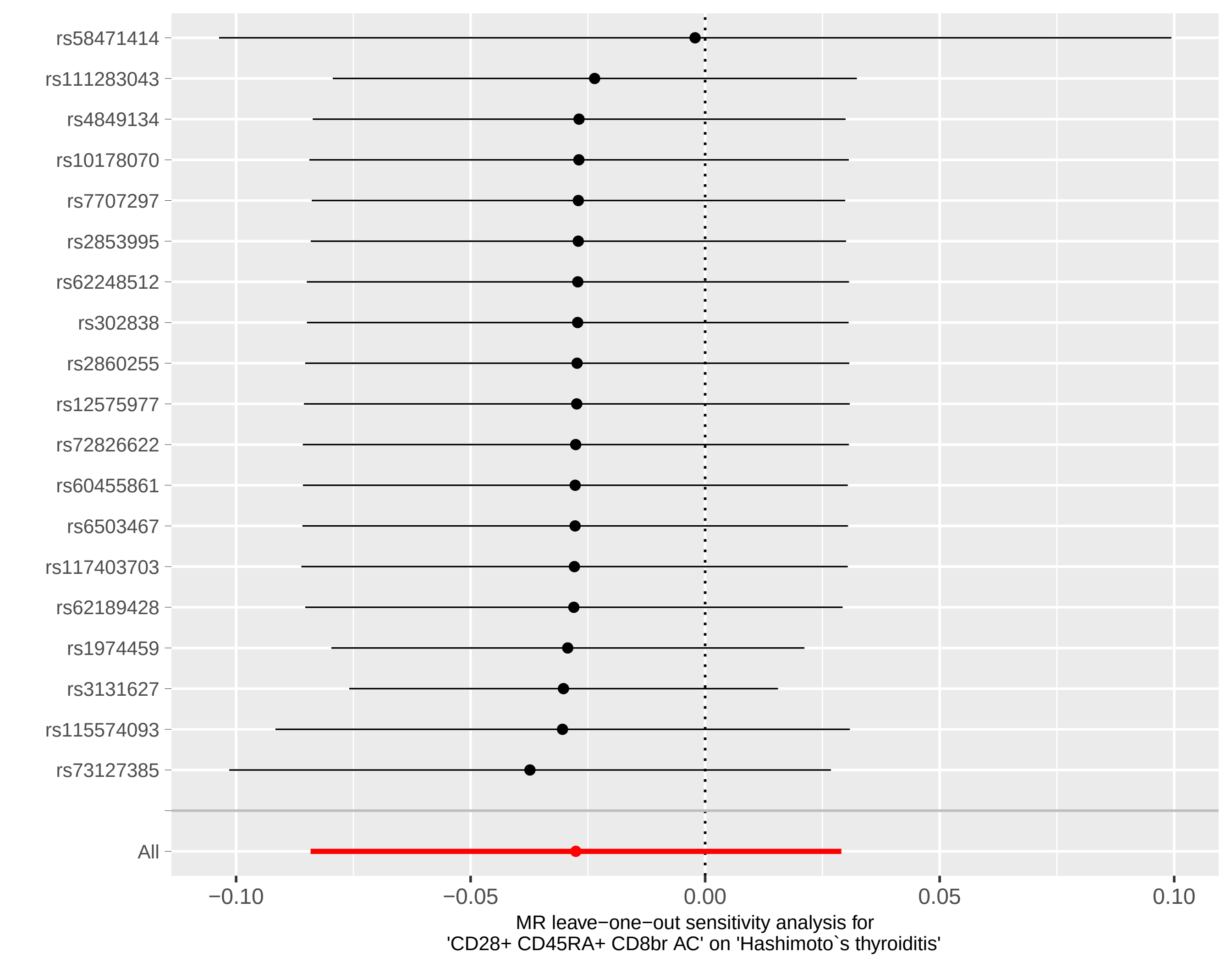

rs58471414
rs111283043
rs4849134
rs10178070
rs7707297
rs2853995
rs62248512
rs302838
rs2860255
rs12575977
rs72826622
rs60455861
rs6503467
rs117403703
rs62189428
rs1974459
rs3131627
rs115574093
rs73127385
All
−0.10
−0.05
0.00
0.05
0.10
MR leave−one−out sensitivity analysis for
'CD28+ CD45RA+ CD8br AC' on 'Hashimoto`s thyroiditis'

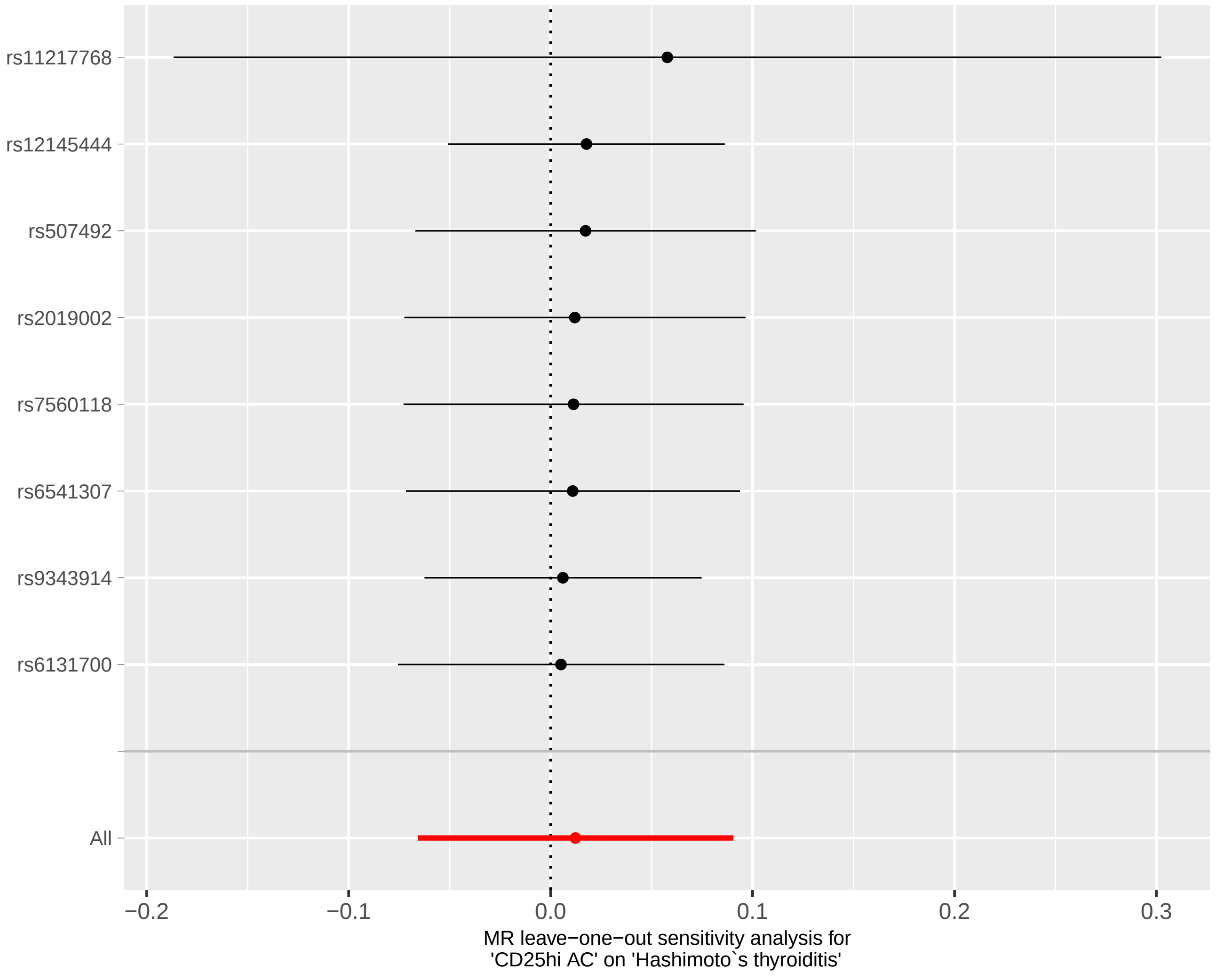
rs11217768
rs12145444
rs507492
rs2019002
rs7560118
rs6541307
rs9343914
rs6131700
All
−0.2
−0.1
0.0
0.1
0.2
0.3
MR leave−one−out sensitivity analysis for
'CD25hi AC' on 'Hashimoto`s thyroiditis'

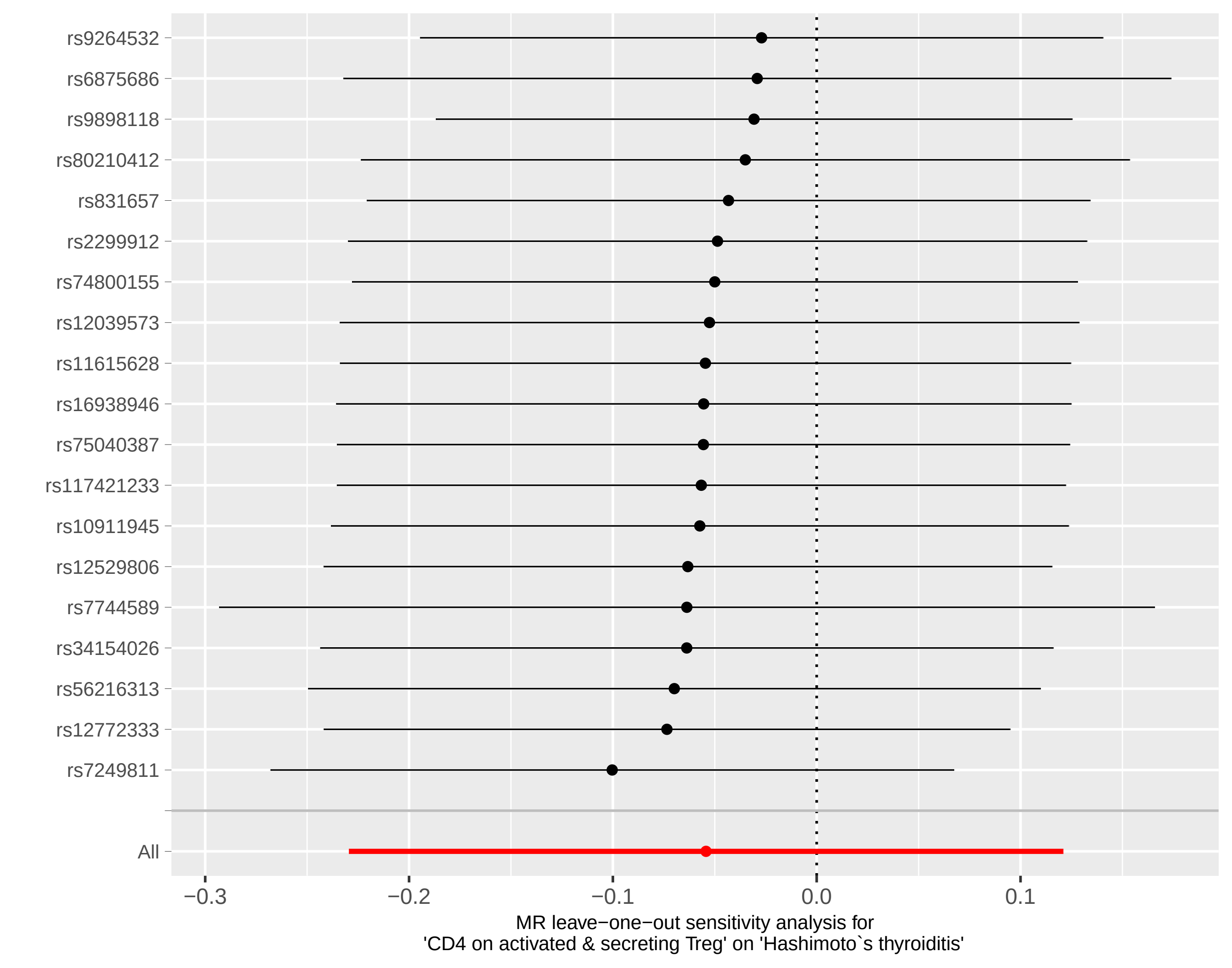
rs9264532
rs6875686
rs9898118
rs80210412
rs831657
rs2299912
rs74800155
rs12039573
rs11615628
rs16938946
rs75040387
rs117421233
rs10911945
rs12529806
rs7744589
rs34154026
rs56216313
rs12772333
rs7249811
All
−0.3
−0.2
−0.1
0.0
0.1
MR leave−one−out sensitivity analysis for
'CD4 on activated & secreting Treg' on 'Hashimoto`s thyroiditis'

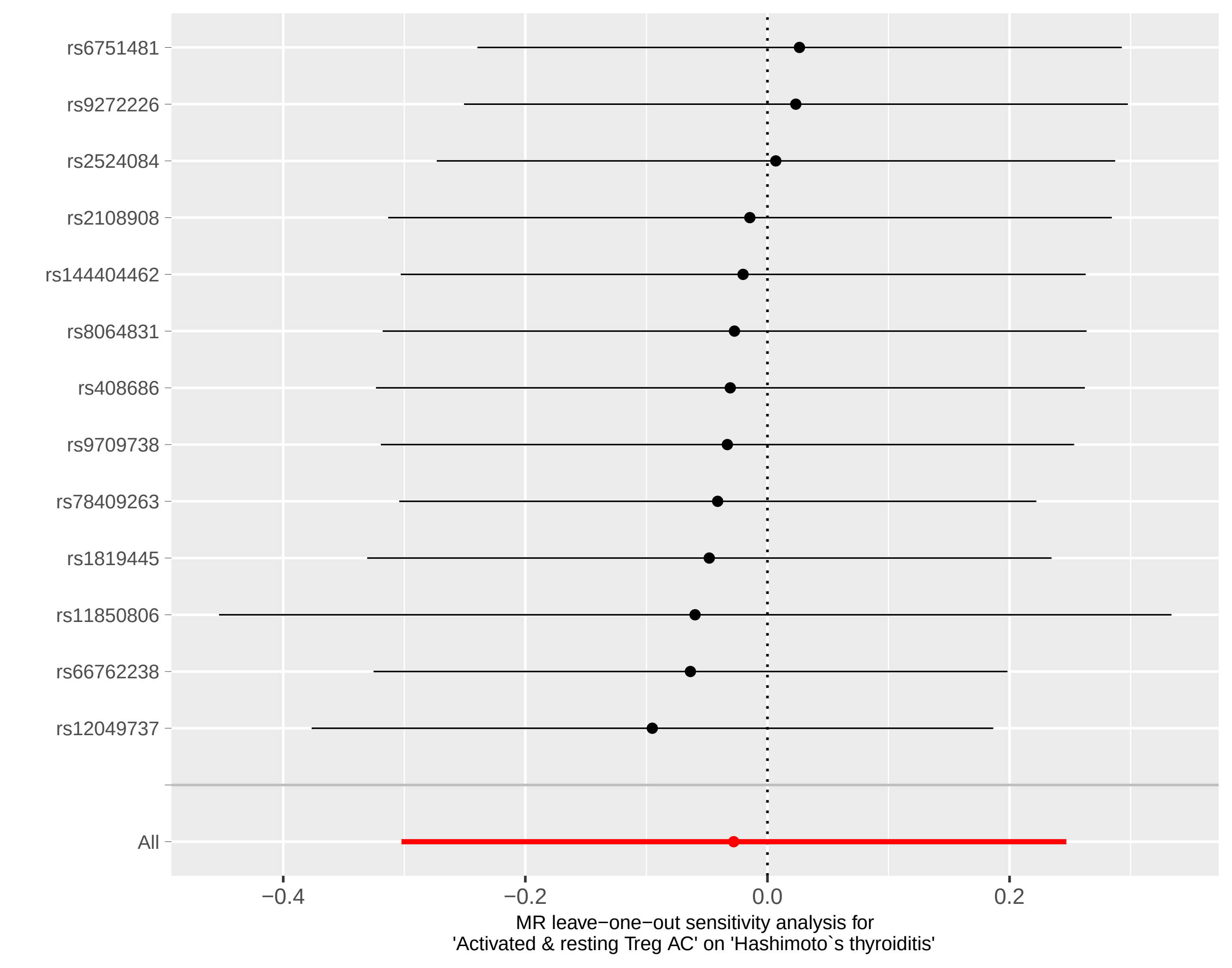
rs6751481
rs9272226
rs2524084
rs2108908
rs144404462
rs8064831
rs408686
rs9709738
rs78409263
rs1819445
rs11850806
rs66762238
rs12049737
All
−0.4
−0.2
0.0
0.2
MR leave−one−out sensitivity analysis for
'Activated & resting Treg AC' on 'Hashimoto`s thyroiditis'

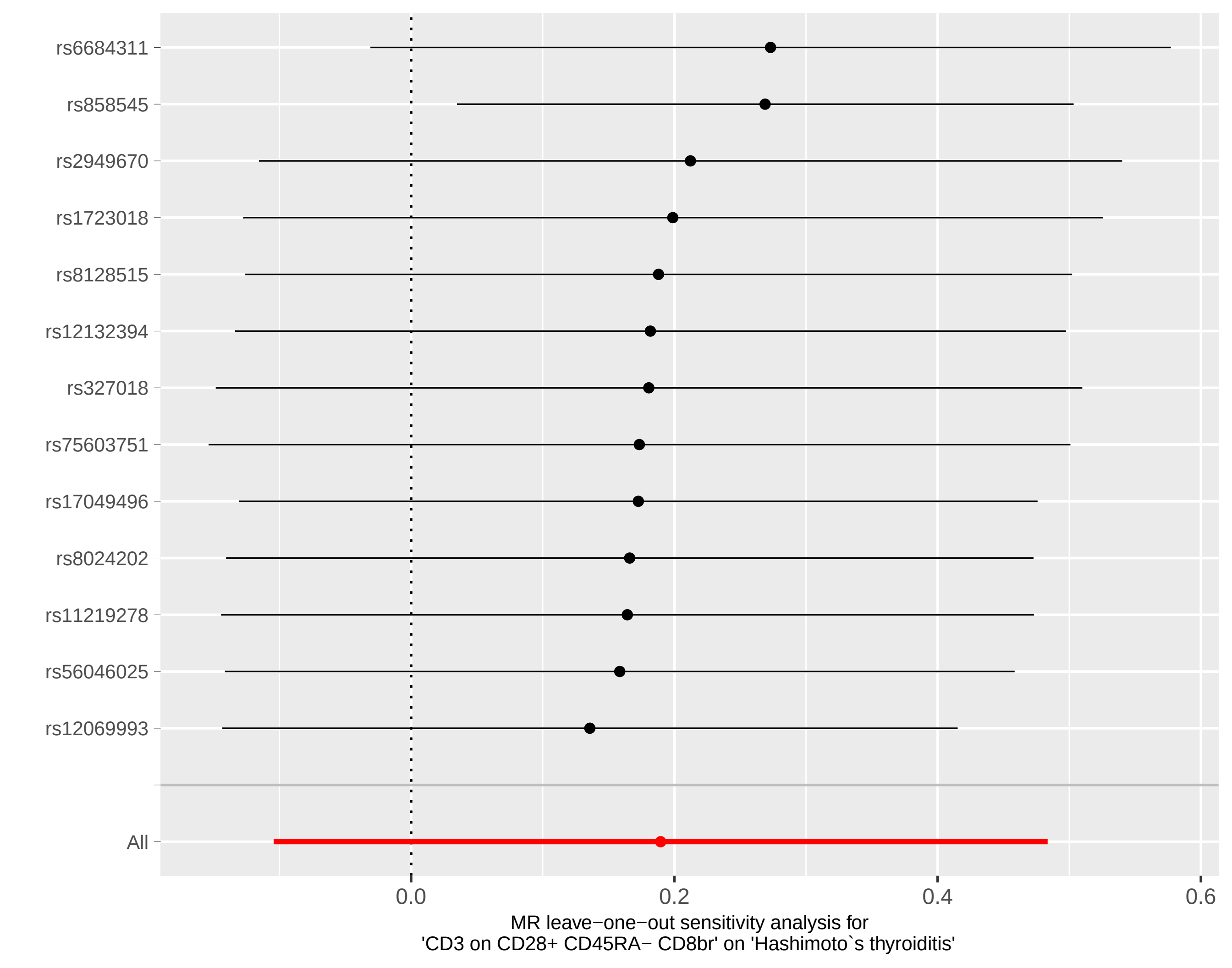
rs6684311
rs858545
rs2949670
rs1723018
rs8128515
rs12132394
rs327018
rs75603751
rs17049496
rs8024202
rs11219278
rs56046025
rs12069993
All
0.0
0.2
0.4
0.6
MR leave−one−out sensitivity analysis for
'CD3 on CD28+ CD45RA− CD8br' on 'Hashimoto`s thyroiditis'

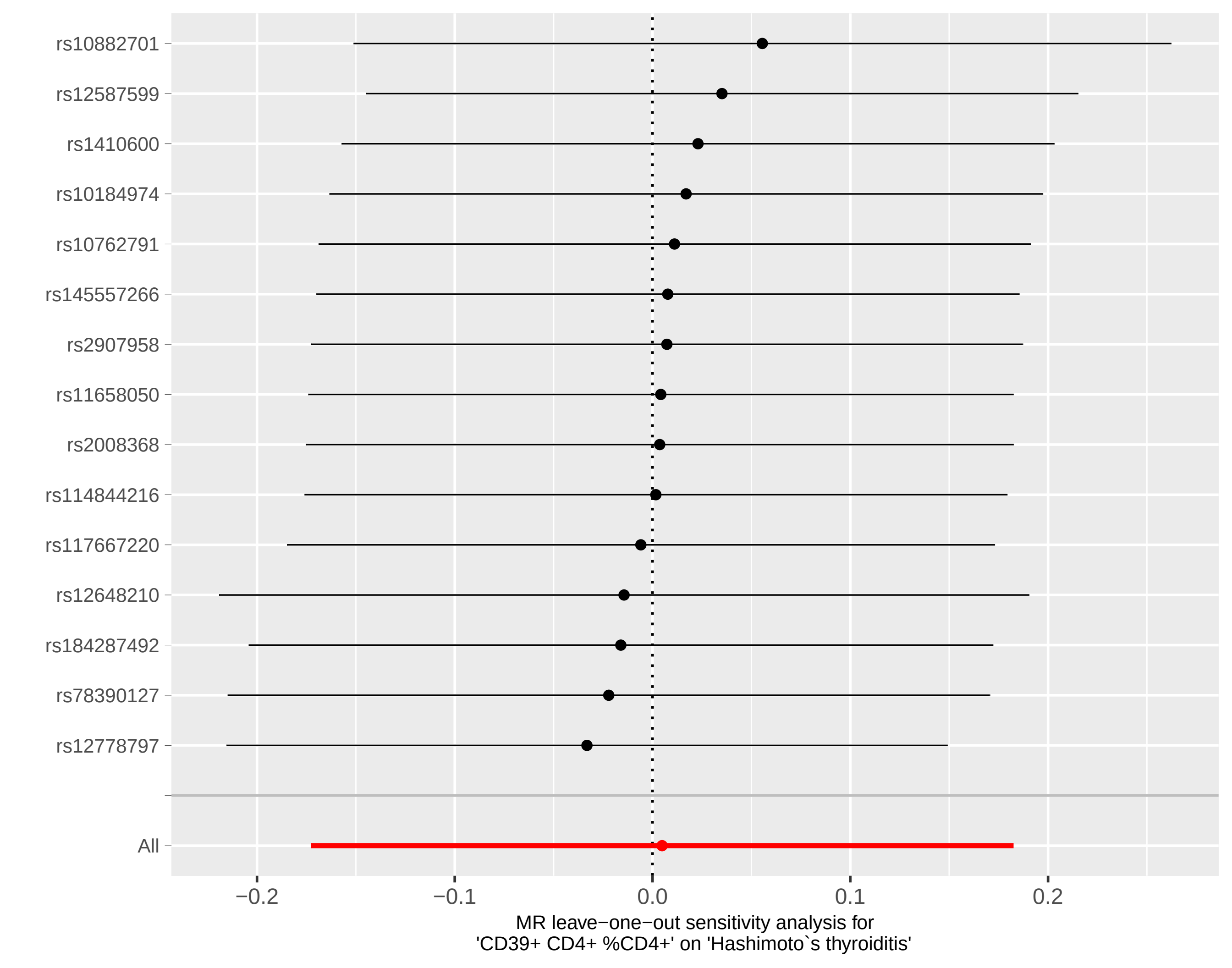
rs10882701
rs12587599
rs1410600
rs10184974
rs10762791
rs145557266
rs2907958
rs11658050
rs2008368
rs114844216
rs117667220
rs12648210
rs184287492
rs78390127
rs12778797
All
−0.2
−0.1
0.0
0.1
0.2
MR leave−one−out sensitivity analysis for
'CD39+ CD4+ %CD4+' on 'Hashimoto`s thyroiditis'

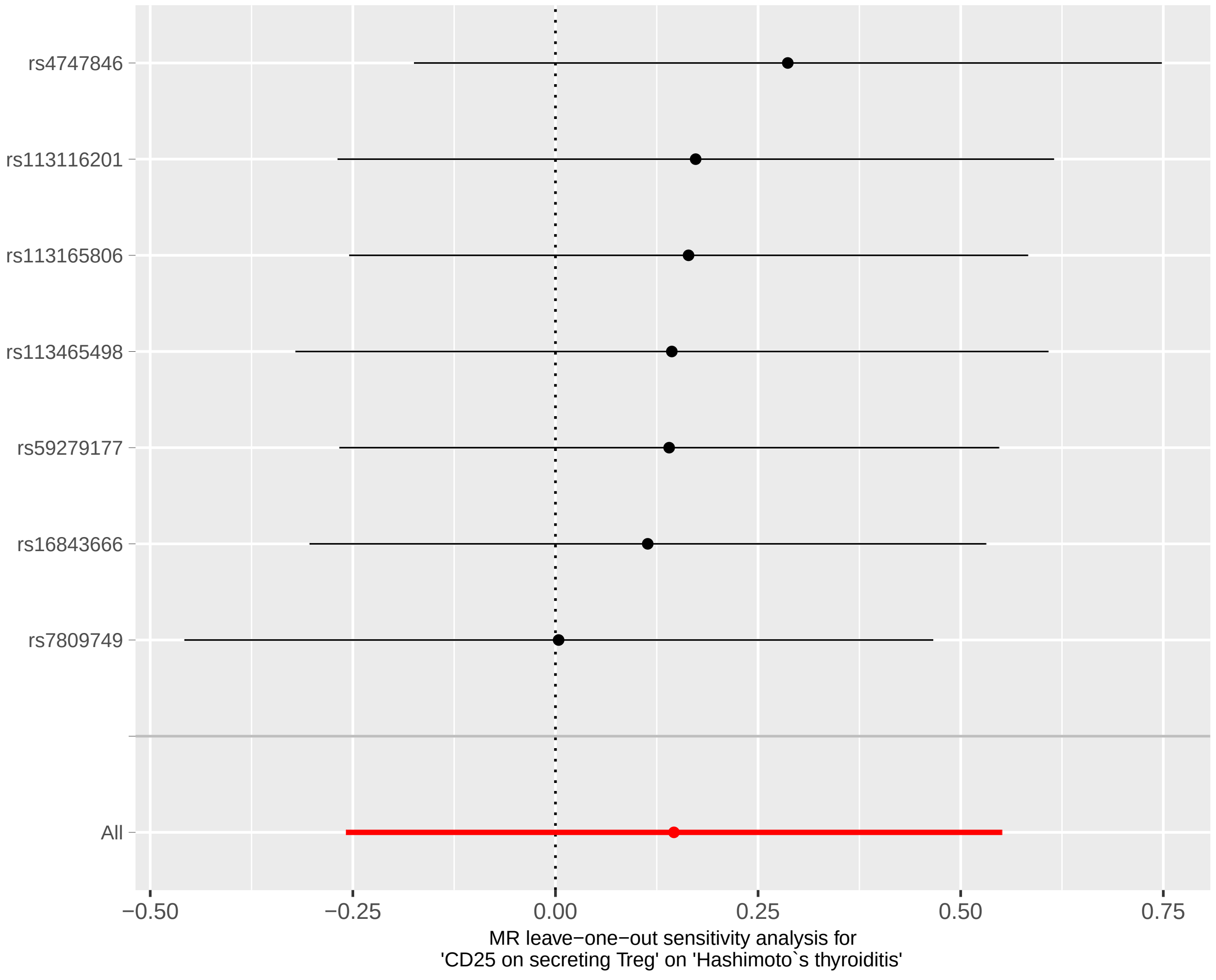

rs4747846
rs113116201
rs113165806
rs113465498
rs59279177
rs16843666
rs7809749
All
−0.50
−0.25
0.00
0.25
0.50
0.75
MR leave−one−out sensitivity analysis for
'CD25 on secreting Treg' on 'Hashimoto`s thyroiditis'

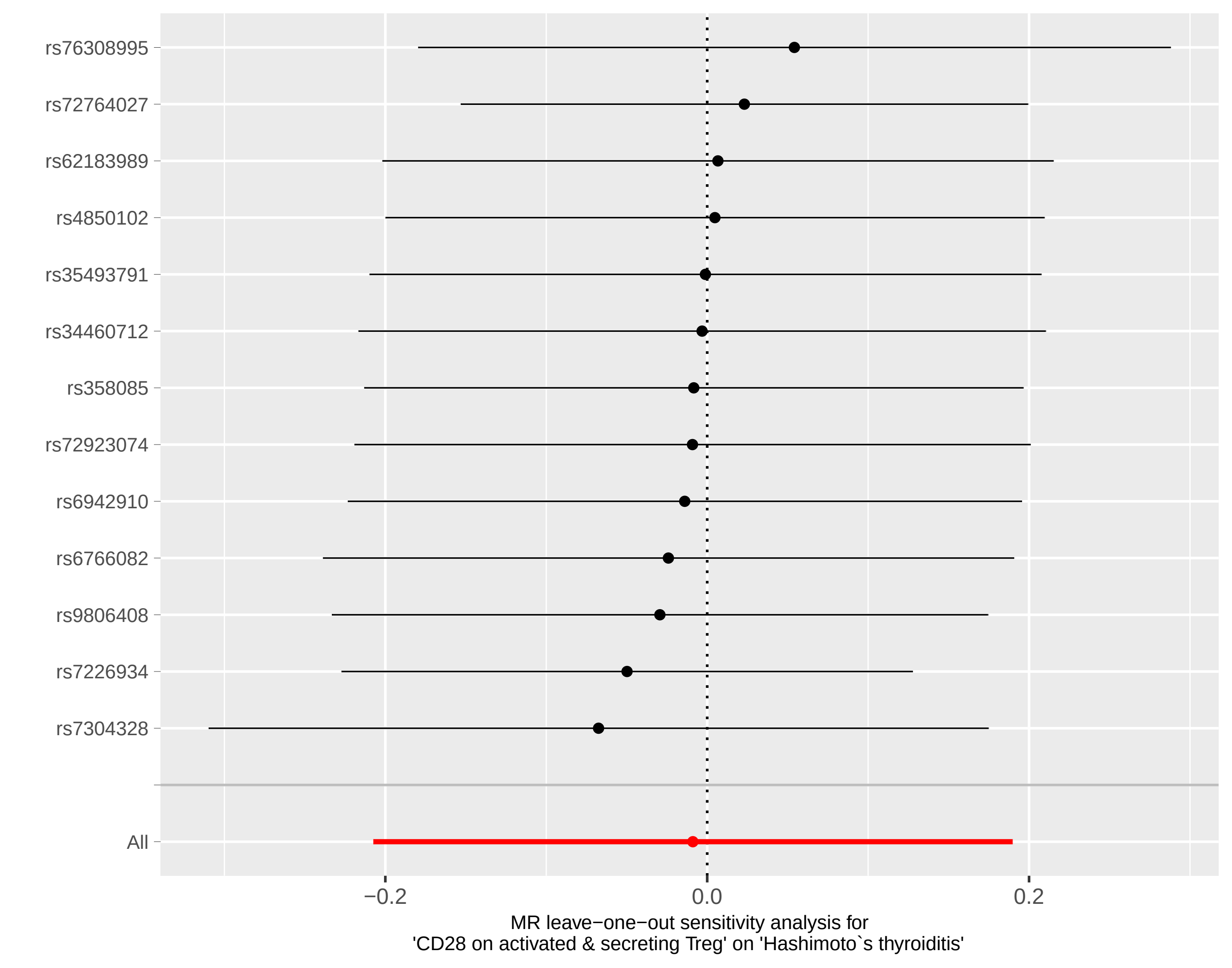
rs76308995
rs72764027
rs62183989
rs4850102
rs35493791
rs34460712
rs358085
rs72923074
rs6942910
rs6766082
rs9806408
rs7226934
rs7304328
All
−0.2
0.0
0.2
MR leave−one−out sensitivity analysis for
'CD28 on activated & secreting Treg' on 'Hashimoto`s thyroiditis'

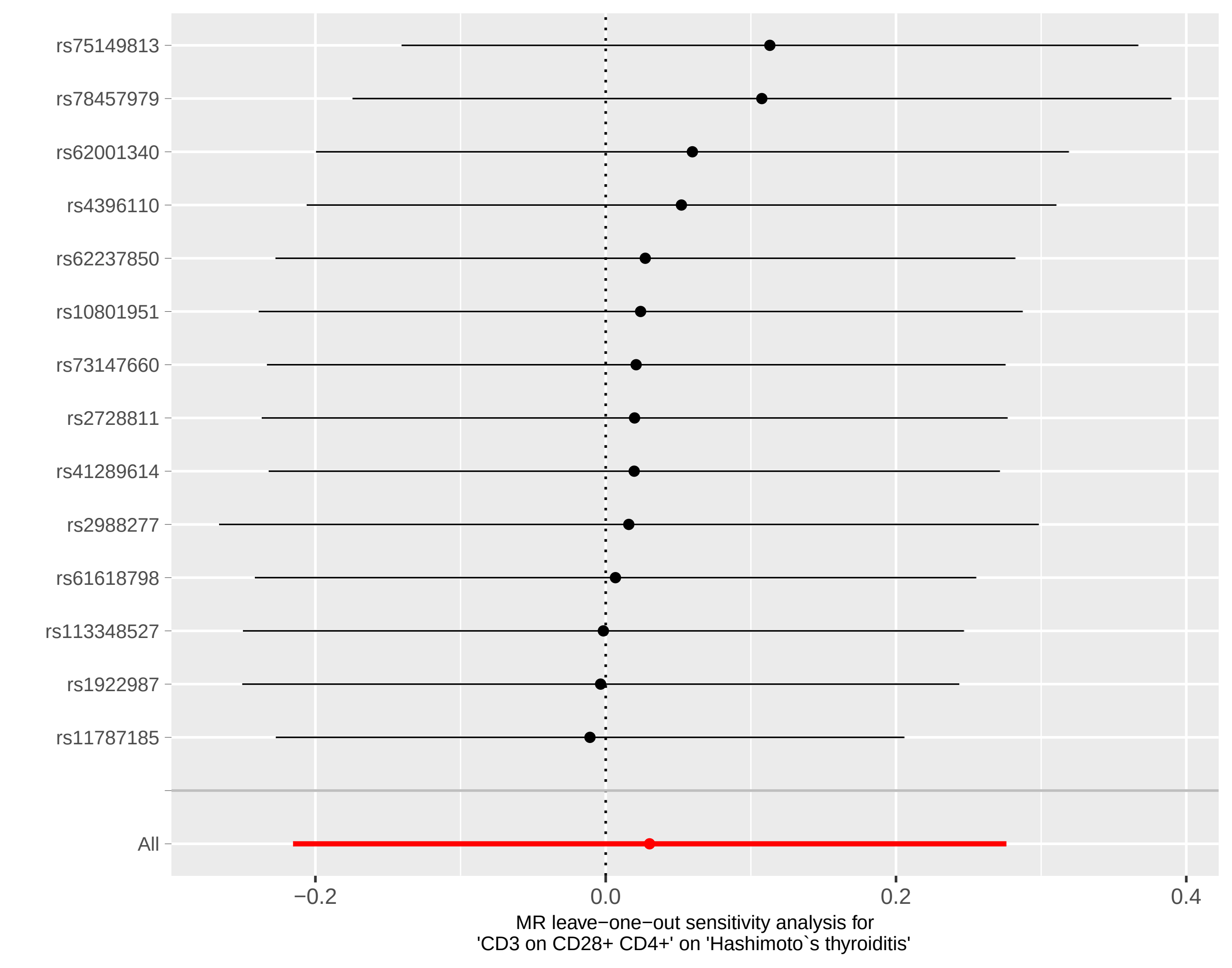

rs75149813
rs78457979
rs62001340
rs4396110
rs62237850
rs10801951
rs73147660
rs2728811
rs41289614
rs2988277
rs61618798
rs113348527
rs1922987
rs11787185
All
−0.2
0.0
0.2
0.4
MR leave−one−out sensitivity analysis for
'CD3 on CD28+ CD4+' on 'Hashimoto`s thyroiditis'

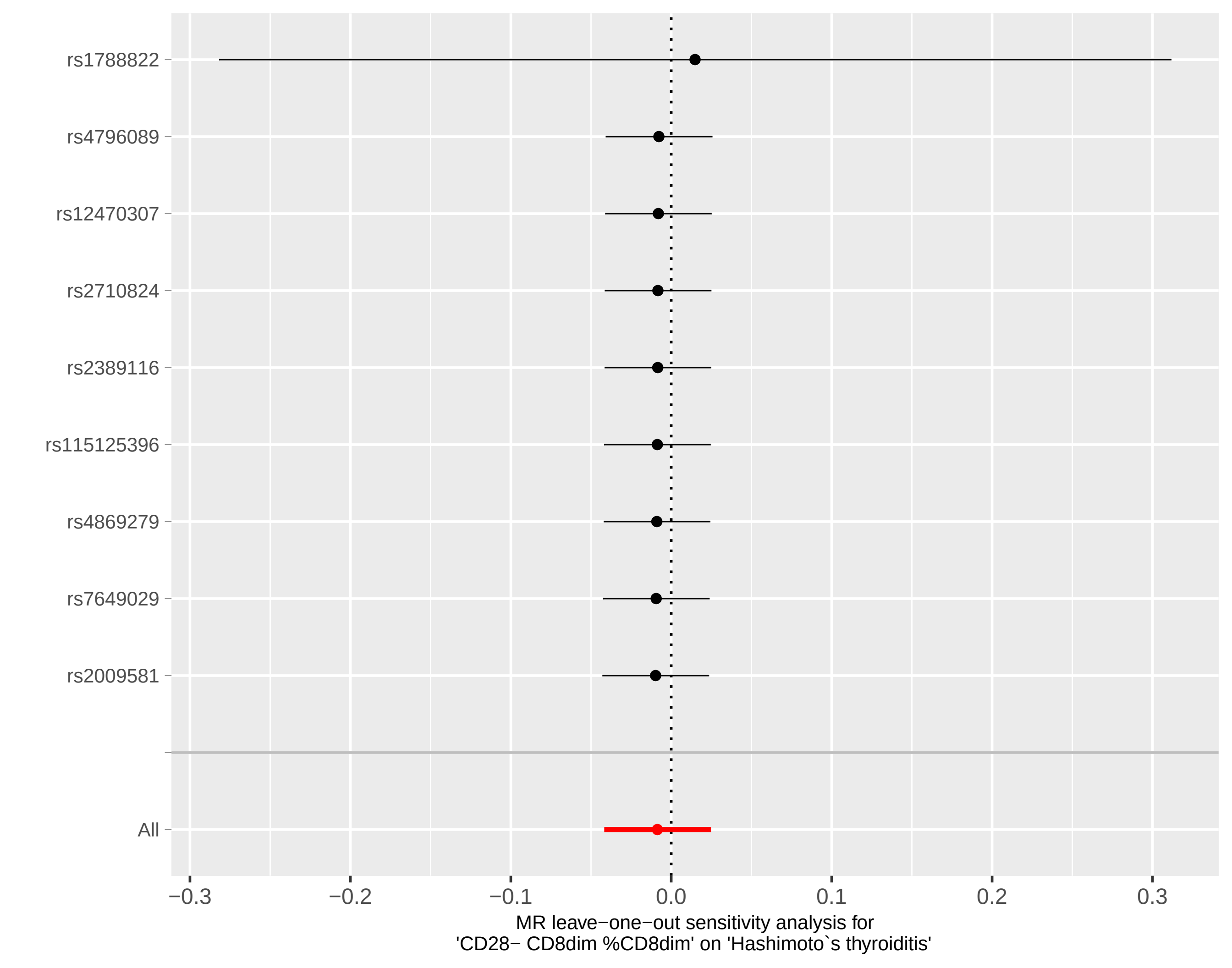
rs1788822
rs4796089
rs12470307
rs2710824
rs2389116
rs115125396
rs4869279
rs7649029
rs2009581
All
−0.3
−0.2
−0.1
0.0
0.1
0.2
0.3
MR leave−one−out sensitivity analysis for
'CD28− CD8dim %CD8dim' on 'Hashimoto`s thyroiditis'

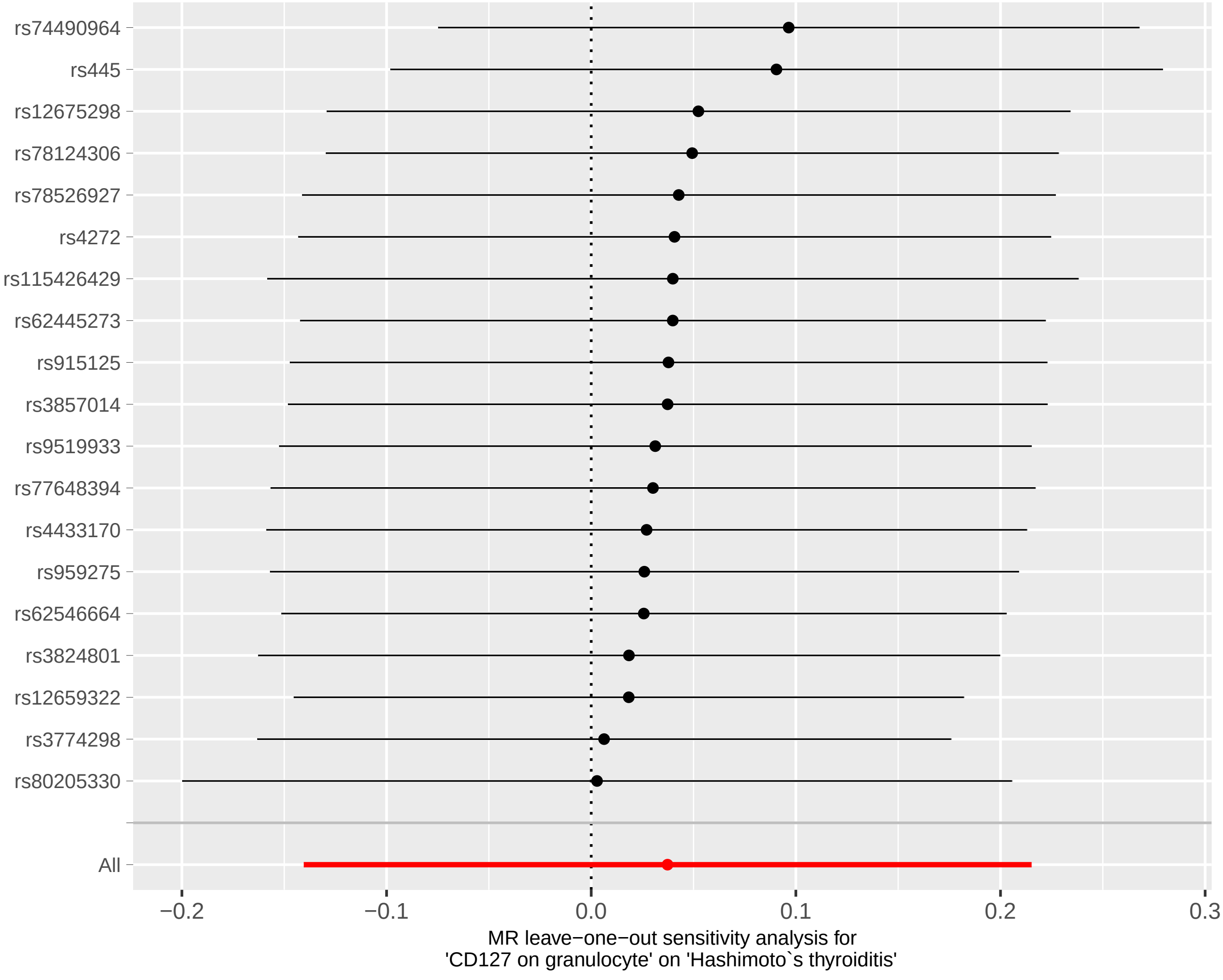

rs74490964
rs445
rs12675298
rs78124306
rs78526927
rs4272
rs115426429
rs62445273
rs915125
rs3857014
rs9519933
rs77648394
rs4433170
rs959275
rs62546664
rs3824801
rs12659322
rs3774298
rs80205330
All
−0.2
−0.1
0.0
0.1
0.2
0.3
MR leave−one−out sensitivity analysis for
'CD127 on granulocyte' on 'Hashimoto`s thyroiditis'

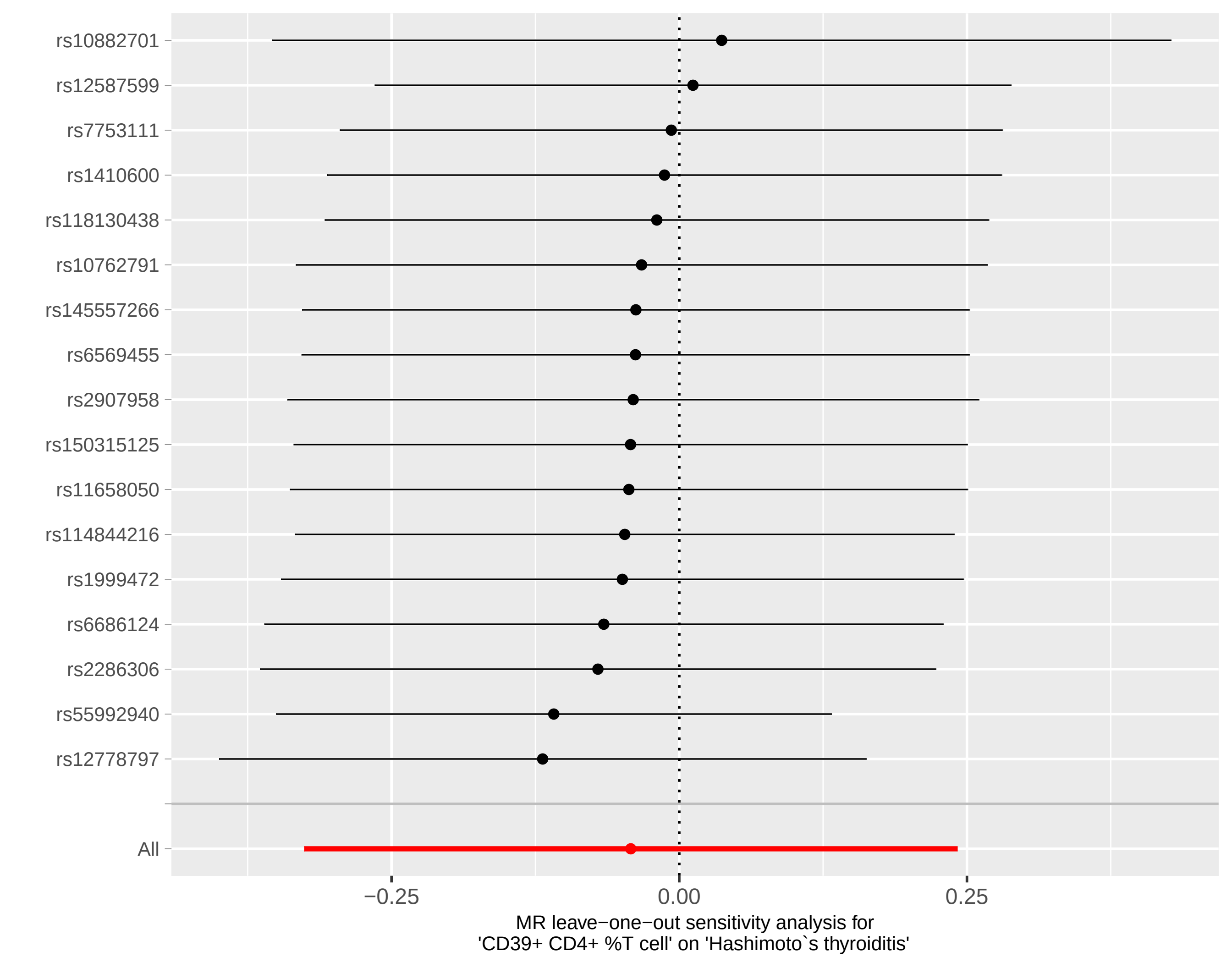
rs10882701
rs12587599
rs7753111
rs1410600
rs118130438
rs10762791
rs145557266
rs6569455
rs2907958
rs150315125
rs11658050
rs114844216
rs1999472
rs6686124
rs2286306
rs55992940
rs12778797
All
−0.25
0.00
0.25
MR leave−one−out sensitivity analysis for
'CD39+ CD4+ %T cell' on 'Hashimoto`s thyroiditis'
