# Supplementary material for: Treg cells as a protective factor for Hashimoto`s thyroiditis: a Mendelian randomization study
Source: Front Endocrinol (Lausanne). 2024 Mar 8;15:1347695. doi: 10.3389/fendo.2024.1347695 (PMC10957564; doi:10.3389/fendo.2024.1347695)
Supplement: Supplementary file 6 [file Image_6.pdf]

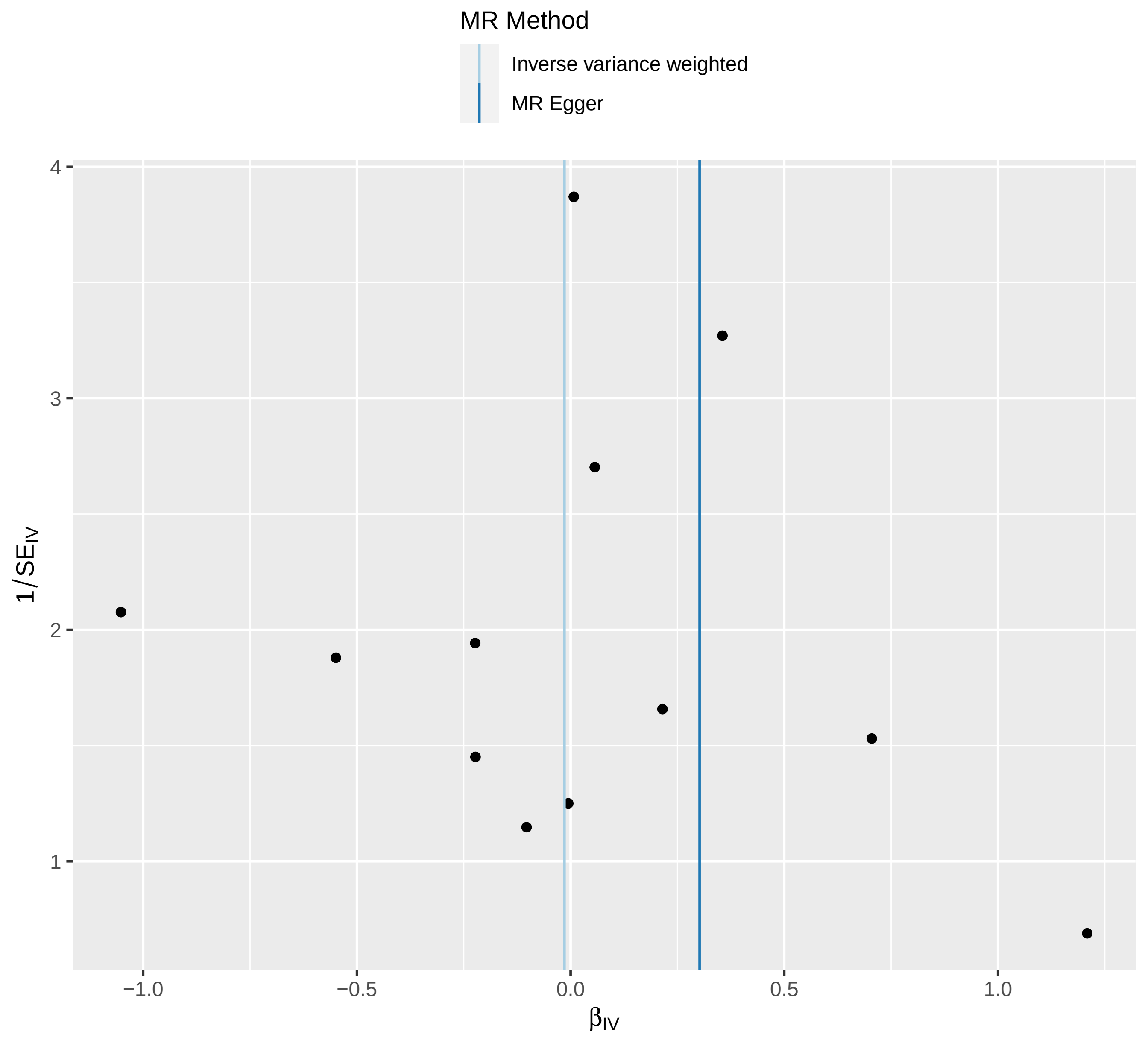
MR Method
Inverse variance weighted
MR Egger
4
3
2
1
1/SE$_{IV}$
−1.0
−0.5
0.0
0.5
1.0
β$_{IV}$

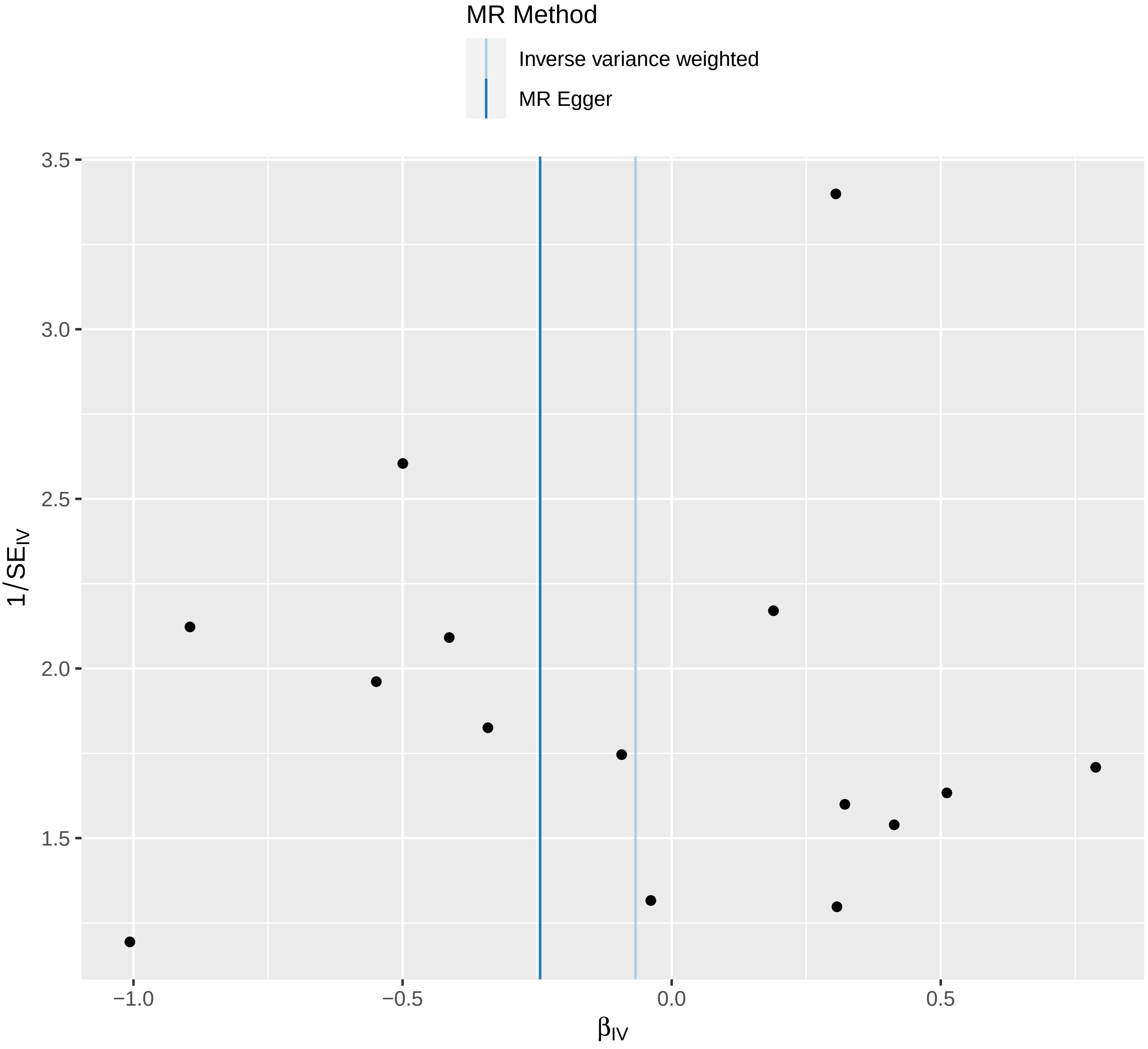

MR Method
Inverse variance weighted
MR Egger
3.5
3.0
2.5
2.0
1.5
1/SE$_{IV}$
−1.0
−0.5
0.0
0.5
β$_{IV}$

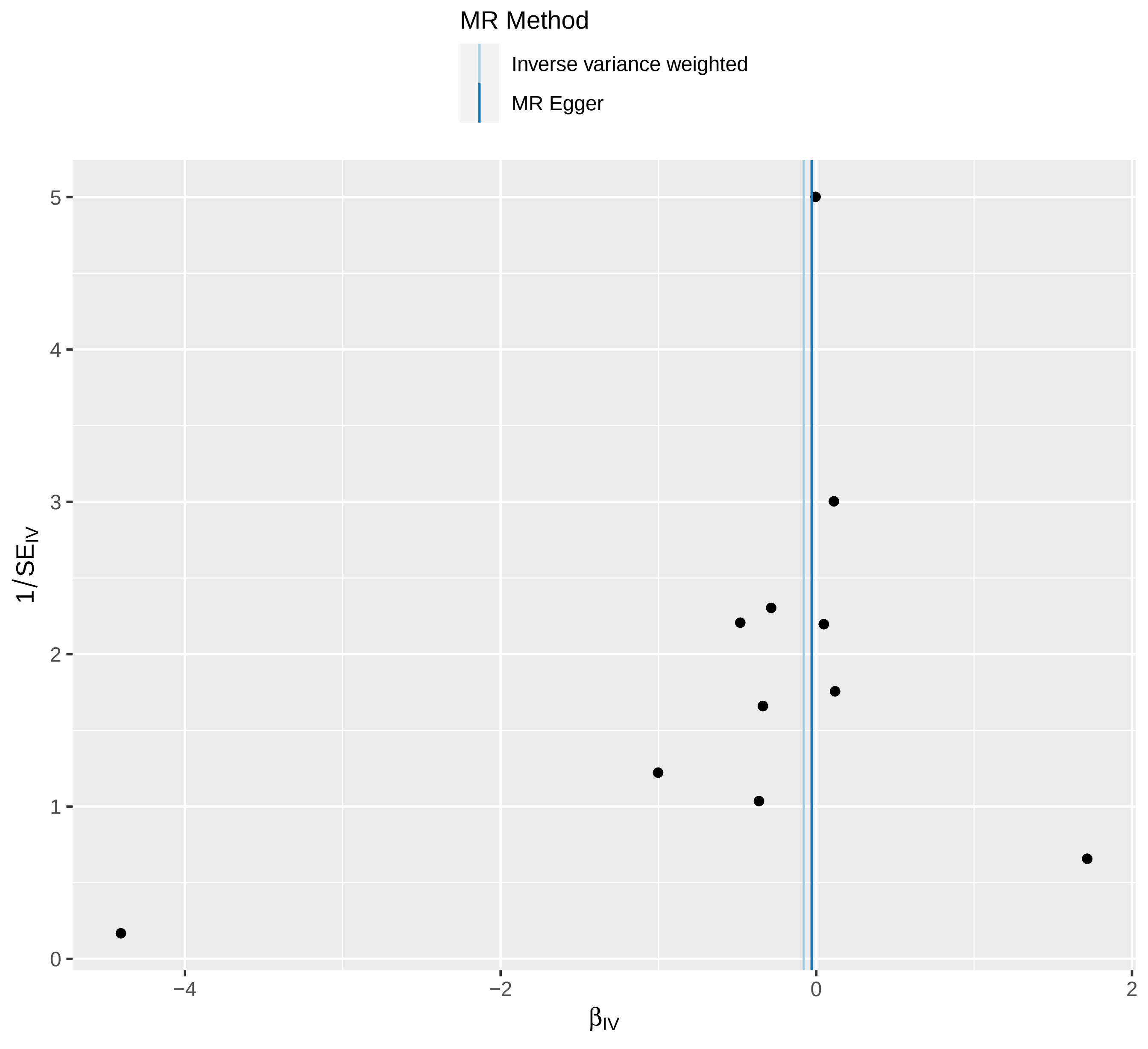

MR Method
Inverse variance weighted
MR Egger
5
4
3
2
1
0
−4
−2
0
2
$1/SE_{IV}$
β_{IV}

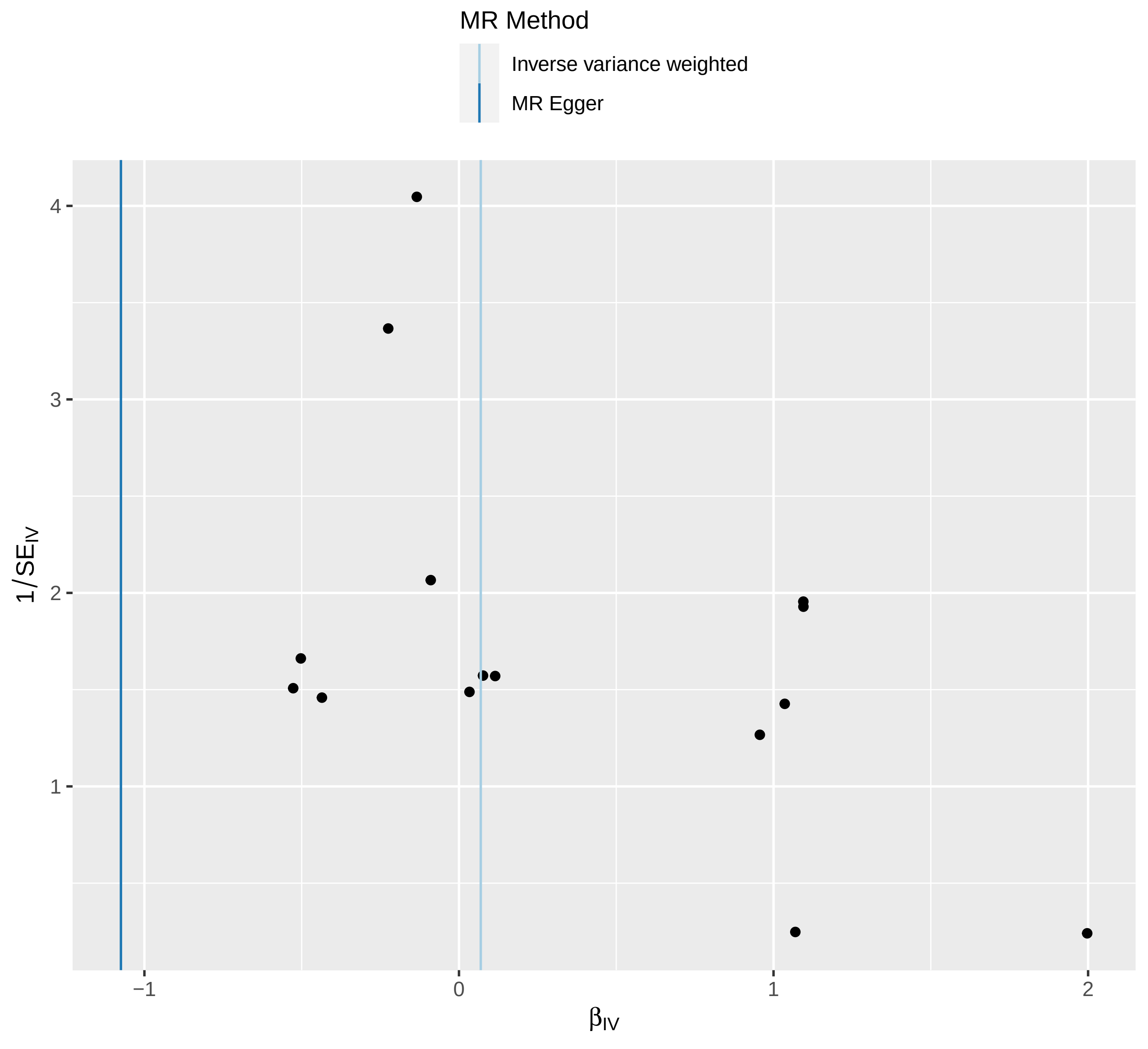

MR Method
Inverse variance weighted
MR Egger
4
3
2
1
1/SE_IV
−1
0
1
2
β_IV

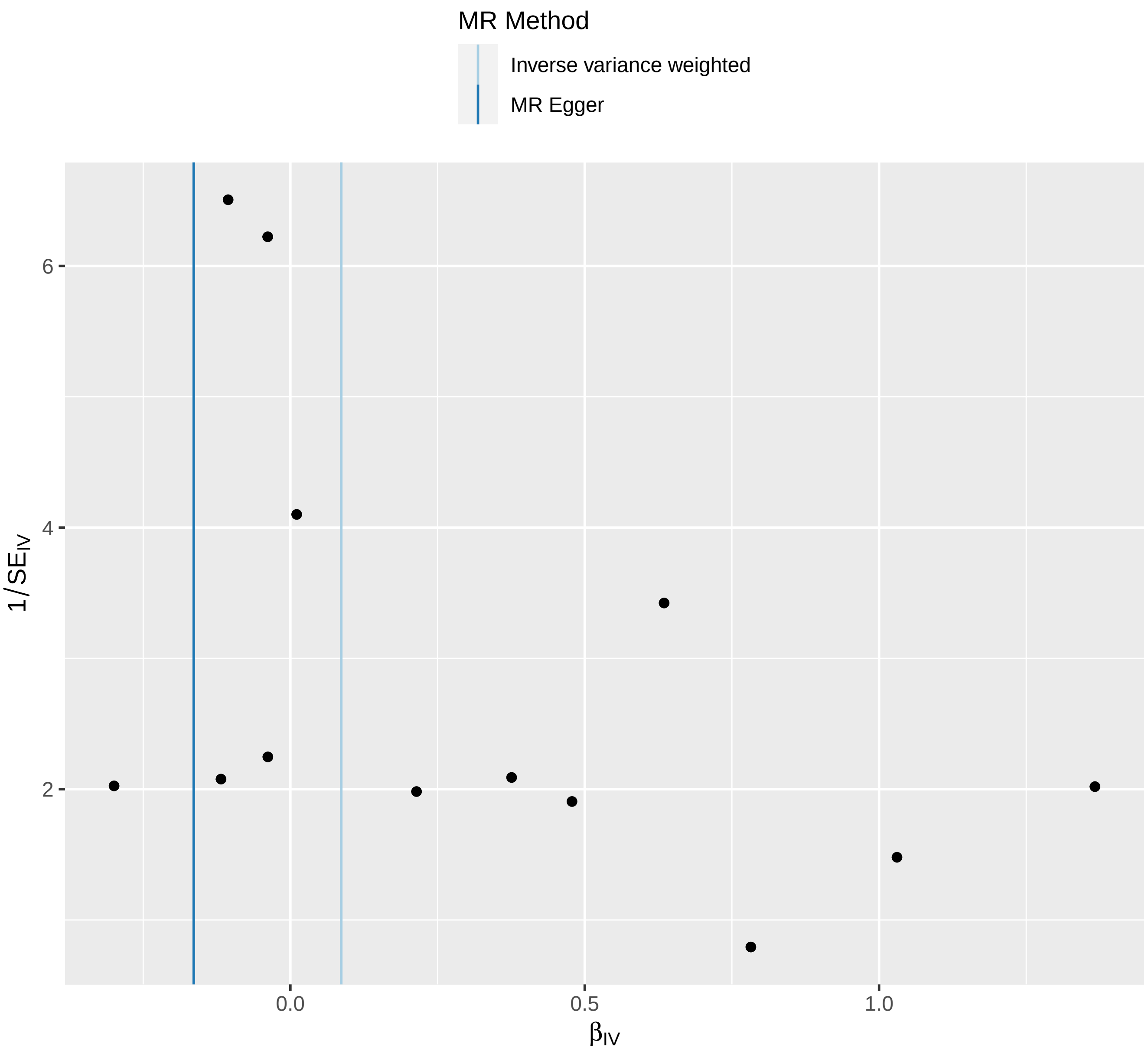

MR Method
Inverse variance weighted
MR Egger
6
4
2
1/SE$_{IV}$
0.0
0.5
1.0
β$_{IV}$

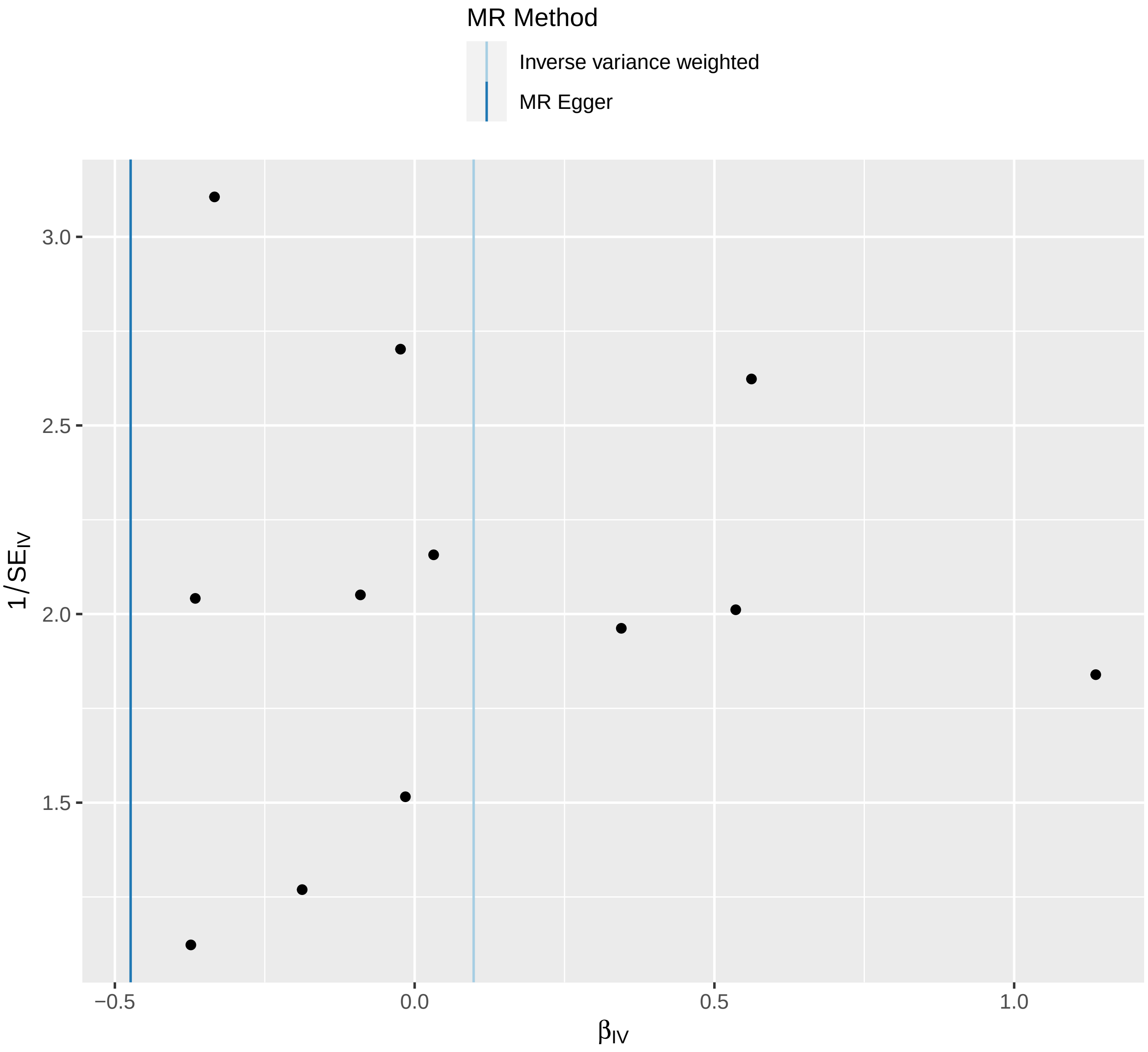

MR Method
Inverse variance weighted
MR Egger
3.0
2.5
2.0
1.5
$1/SE_{IV}$
−0.5
0.0
0.5
1.0
β_{IV}

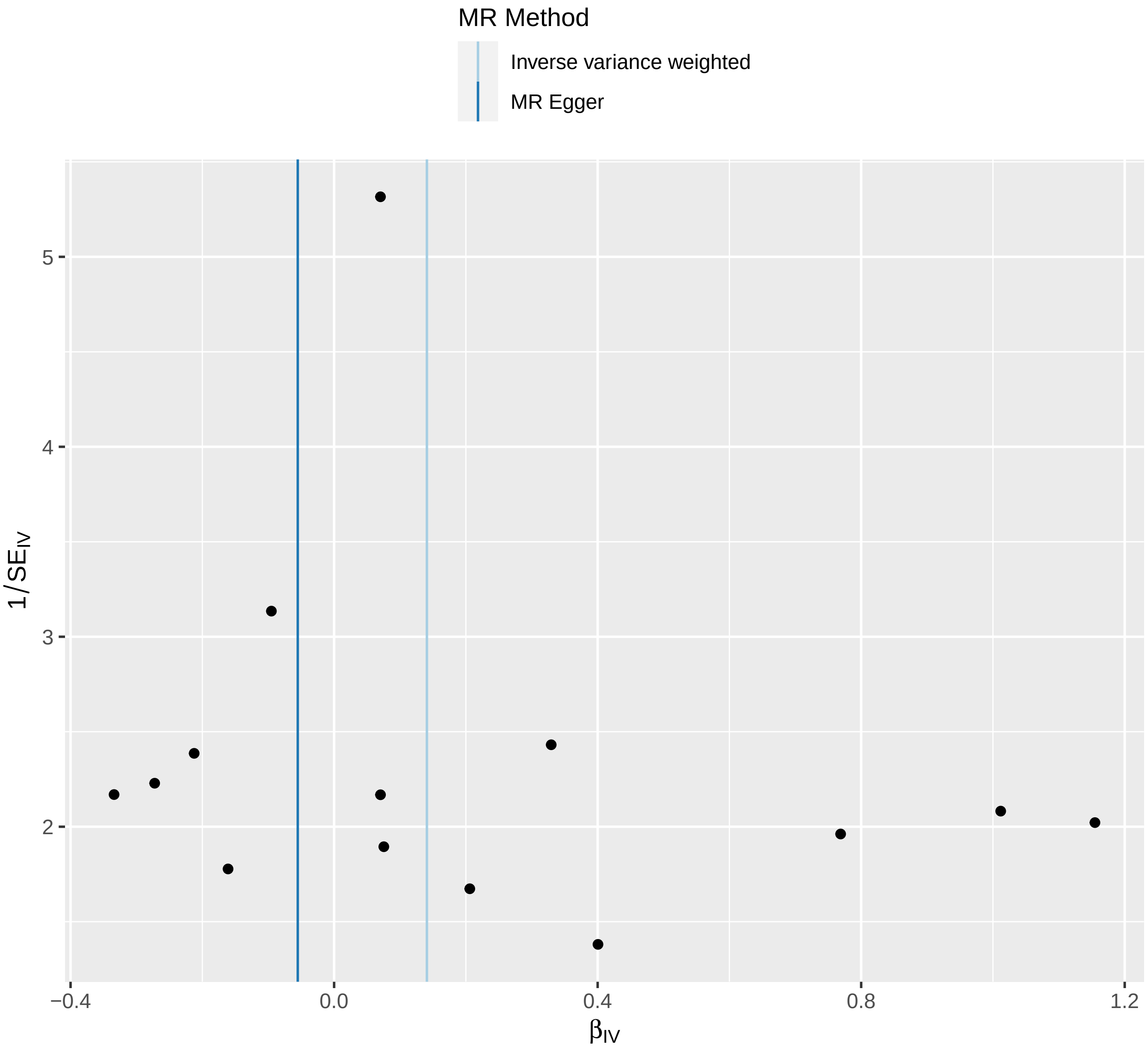

MR Method
Inverse variance weighted
MR Egger
1/SE_IV
5
4
3
2
−0.4
0.0
0.4
0.8
1.2
β_IV

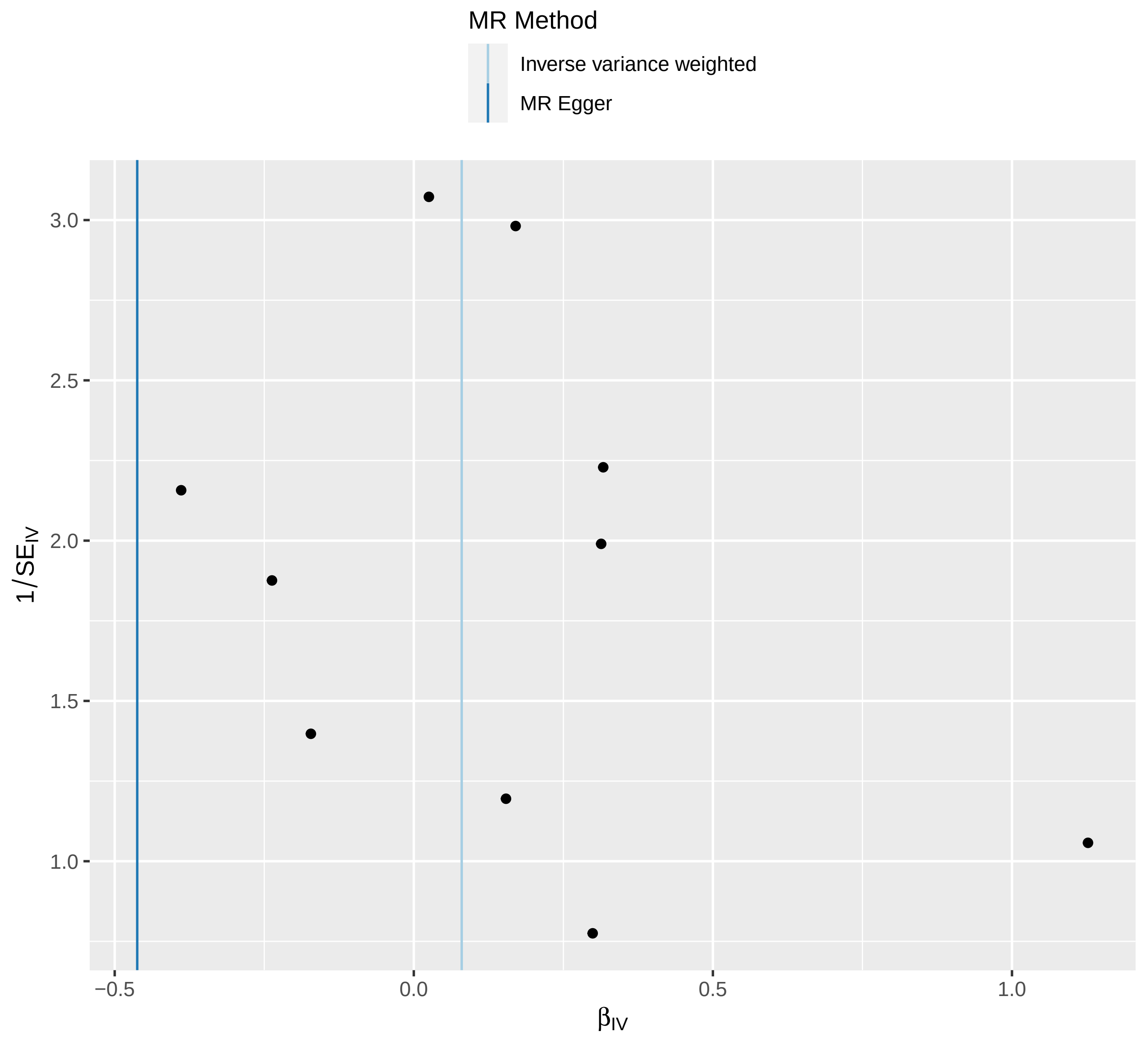
MR Method
Inverse variance weighted
MR Egger
3.0
2.5
2.0
1.5
1.0
1/SE_IV
−0.5
0.0
0.5
1.0
β_IV

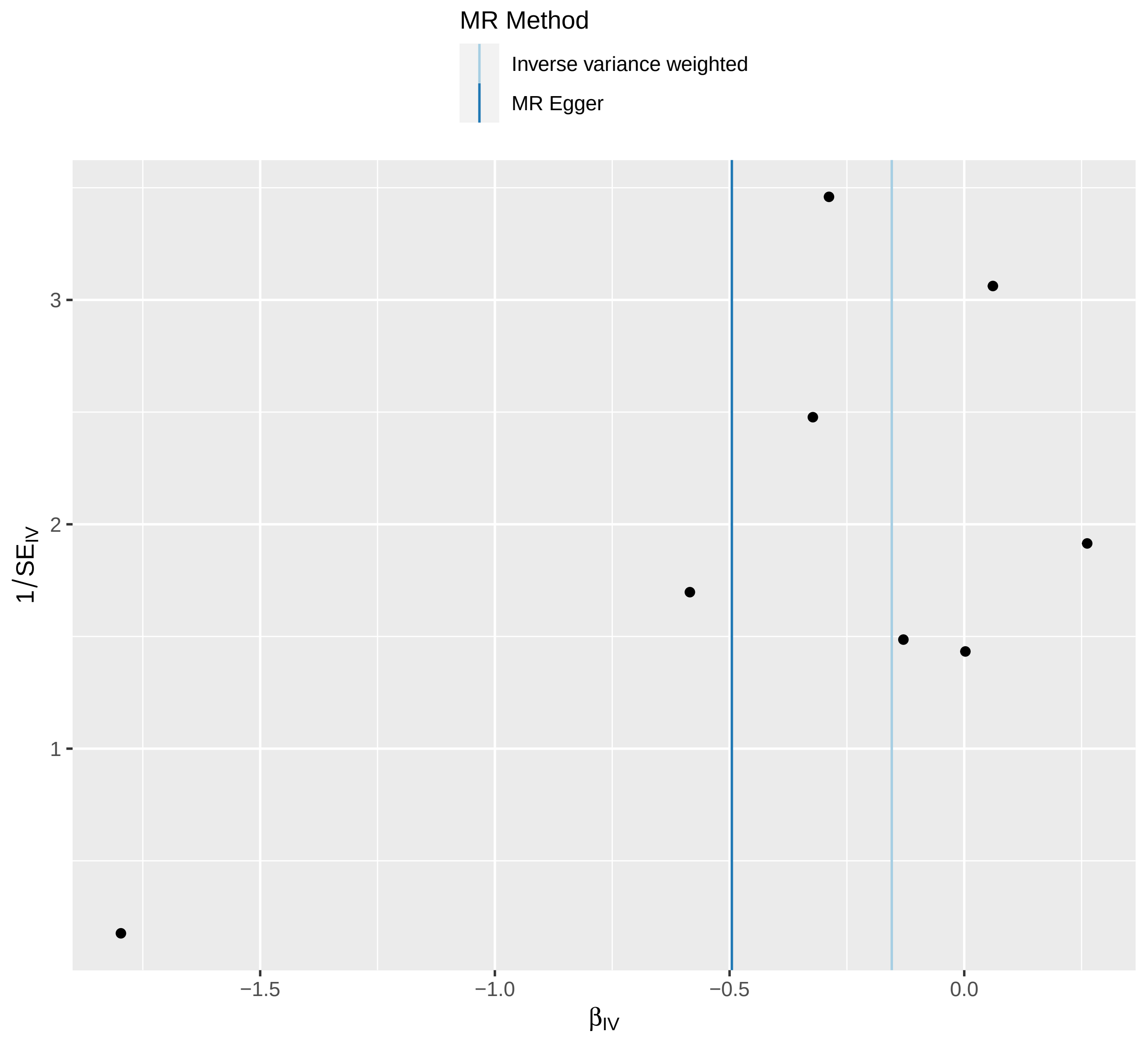

MR Method
Inverse variance weighted
MR Egger
3
2
1
1/SE_IV
−1.5
−1.0
−0.5
0.0
β_IV

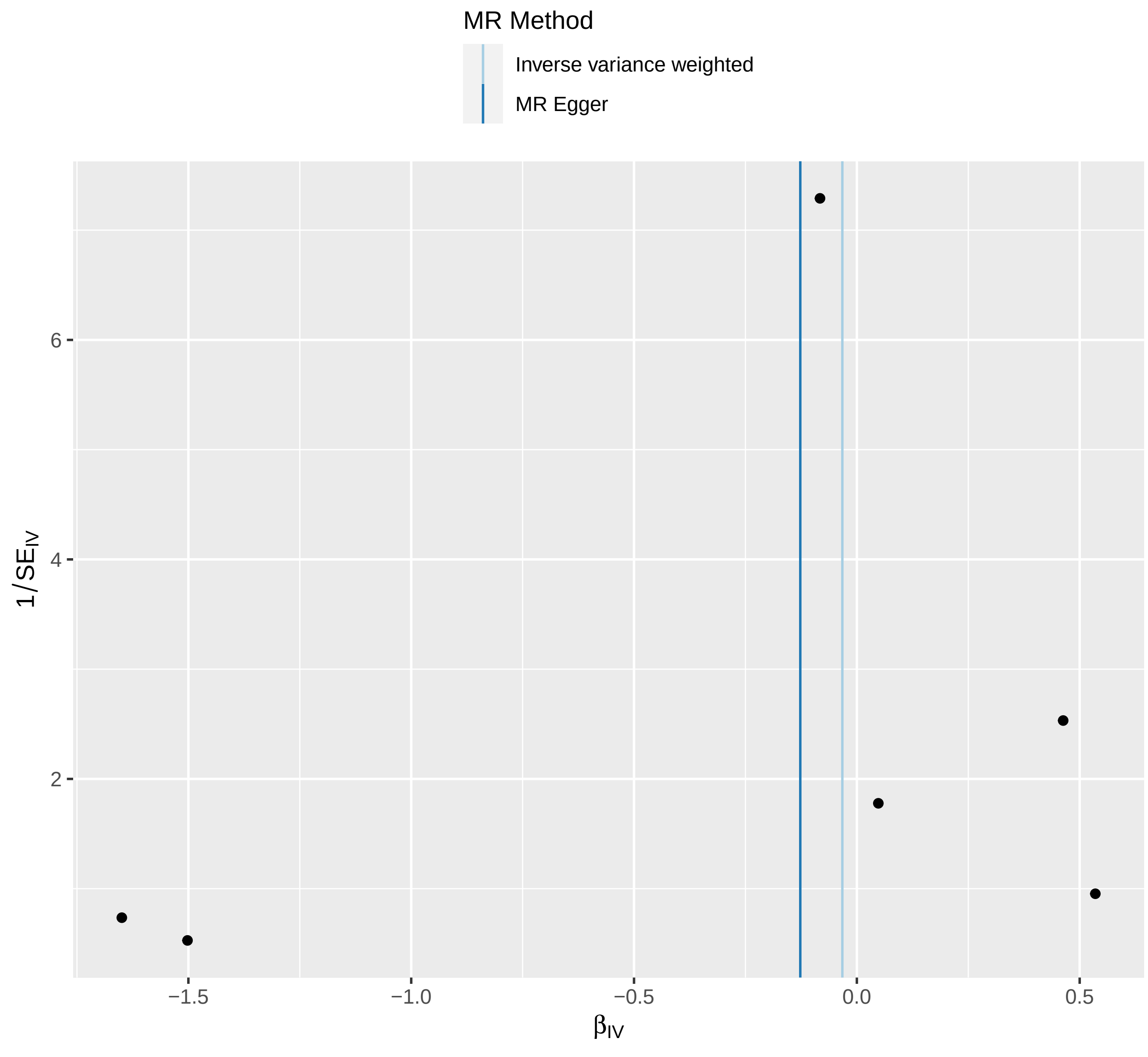

MR Method
Inverse variance weighted
MR Egger
6
4
2
1/SE$_{IV}$
−1.5
−1.0
−0.5
0.0
0.5
β$_{IV}$

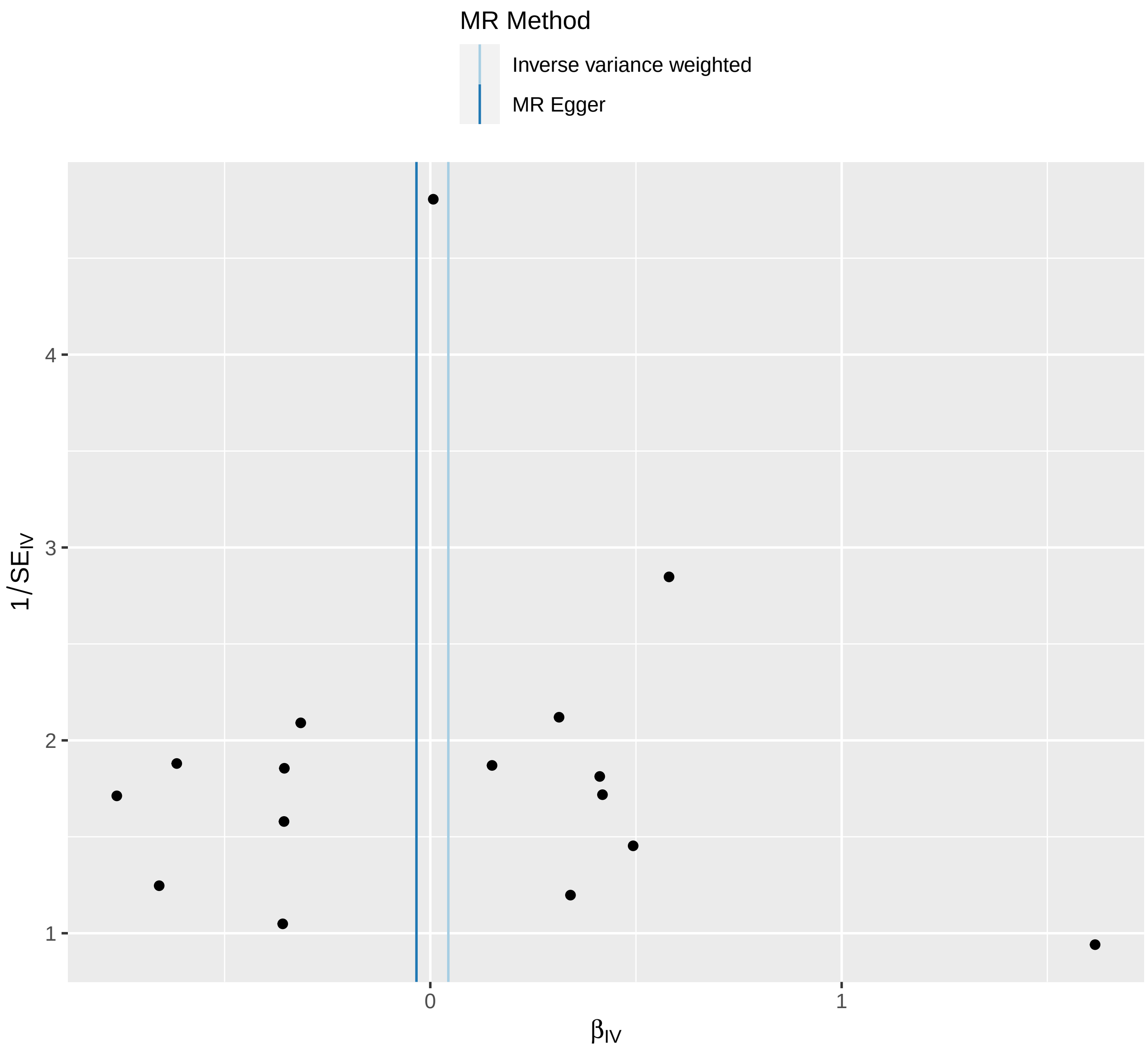

MR Method
Inverse variance weighted
MR Egger
4
3
2
1
1/SE_IV
0
1
β_IV

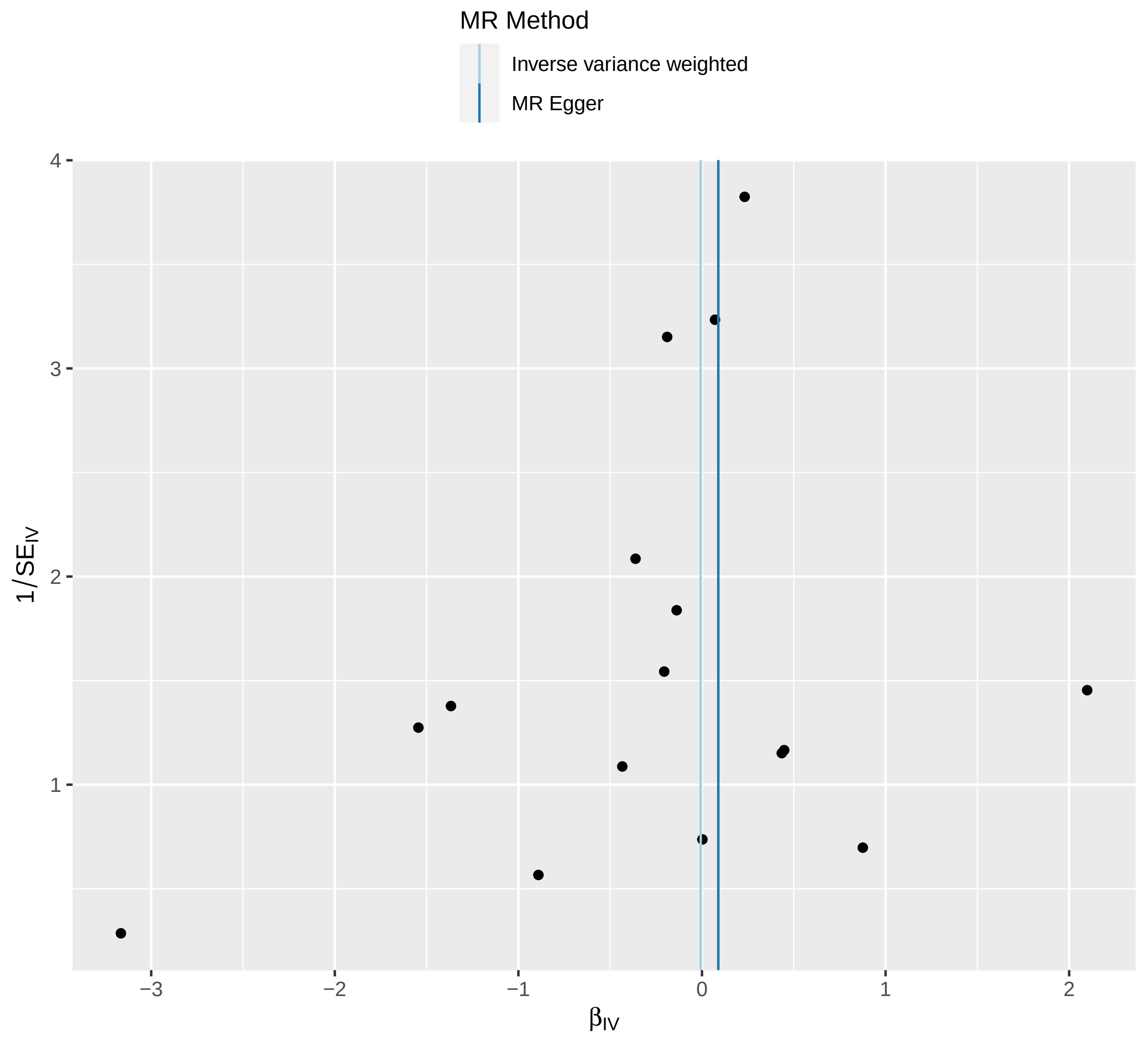
MR Method
Inverse variance weighted
MR Egger
4
3
2
1
1/SE$_{IV}$
−3
−2
−1
0
1
2
β$_{IV}$

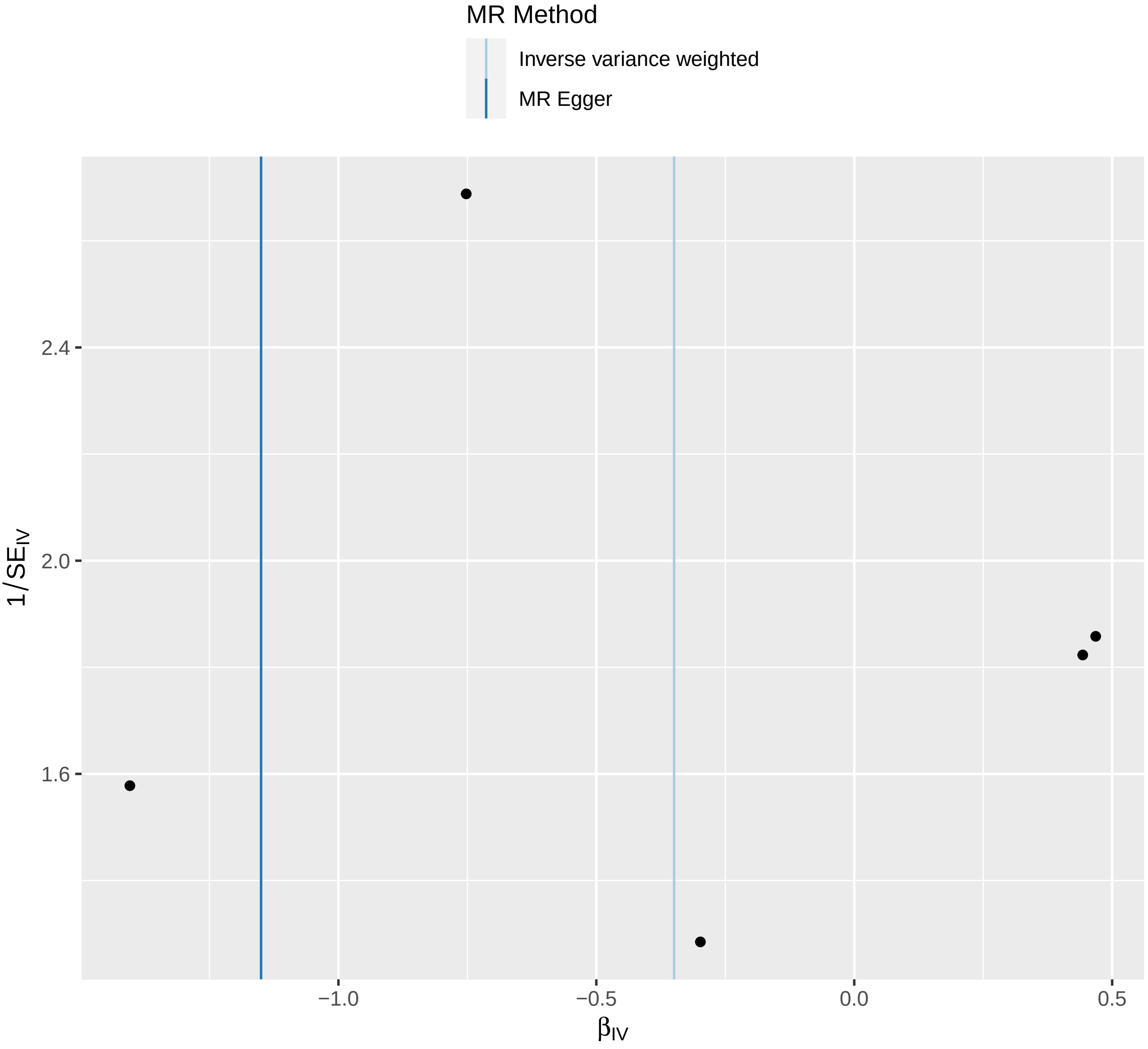

MR Method
Inverse variance weighted
MR Egger
2.4
2.0
1.6
1/SE_IV
−1.0
−0.5
0.0
0.5
β_IV

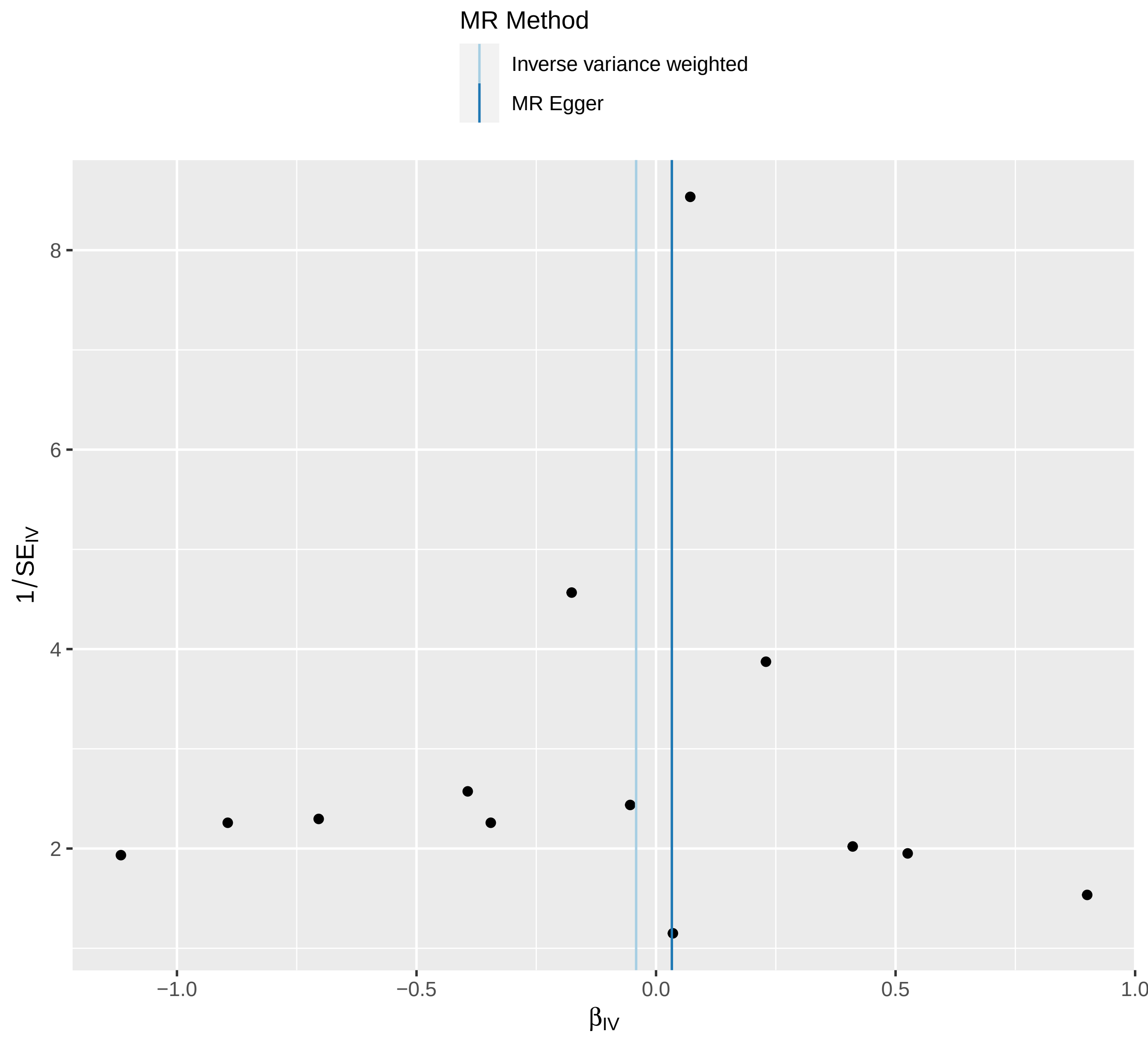
MR Method
Inverse variance weighted
MR Egger
1/SE$_{IV}$
8
6
4
2
−1.0
−0.5
0.0
0.5
1.0
β$_{IV}$

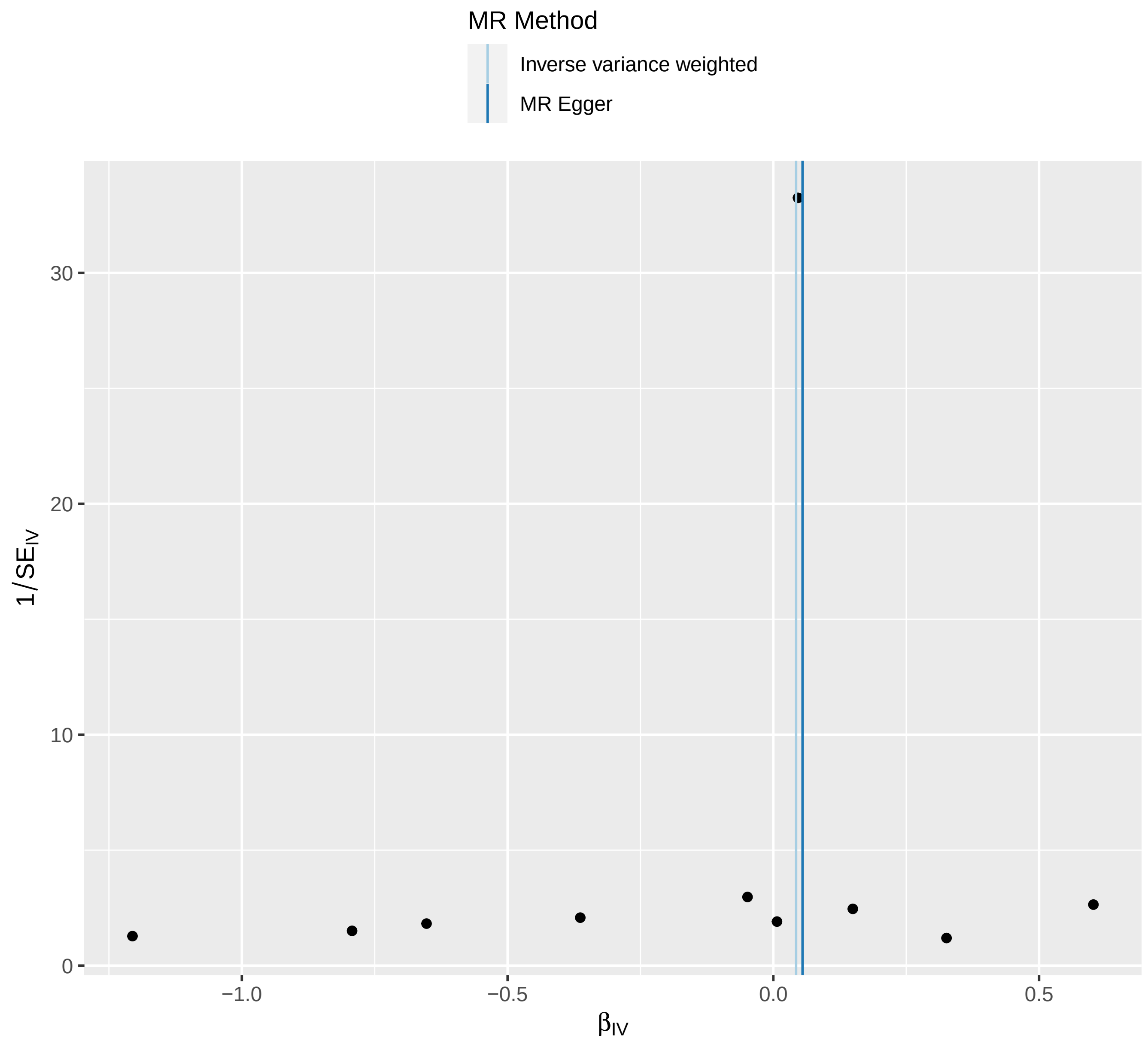

MR Method
Inverse variance weighted
MR Egger
1/SE_IV
β_IV
30
20
10
0
−1.0
−0.5
0.0
0.5

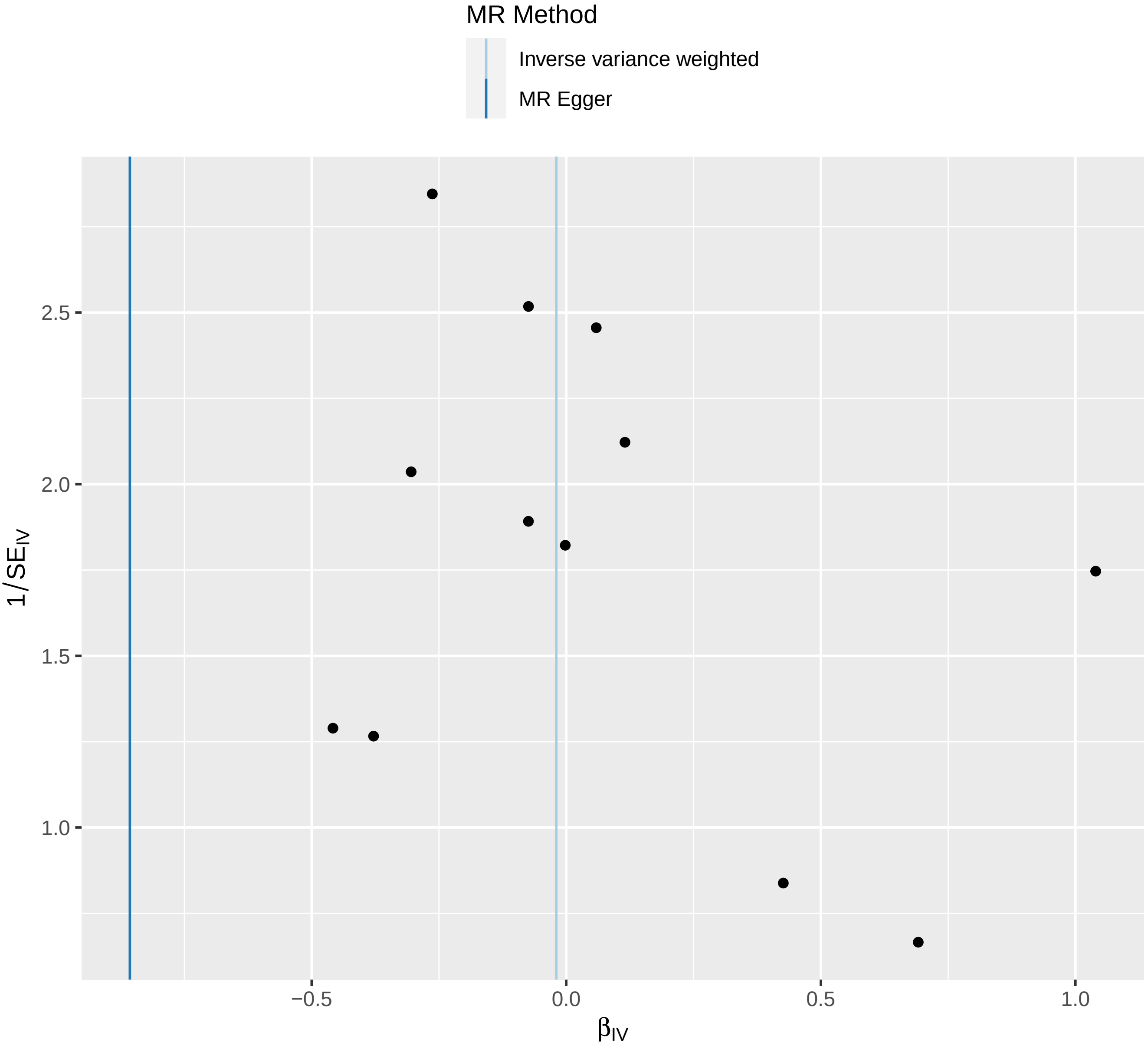

MR Method
Inverse variance weighted
MR Egger
2.5
2.0
1.5
1.0
−0.5
0.0
0.5
1.0
$1/SE_{IV}$
β_{IV}

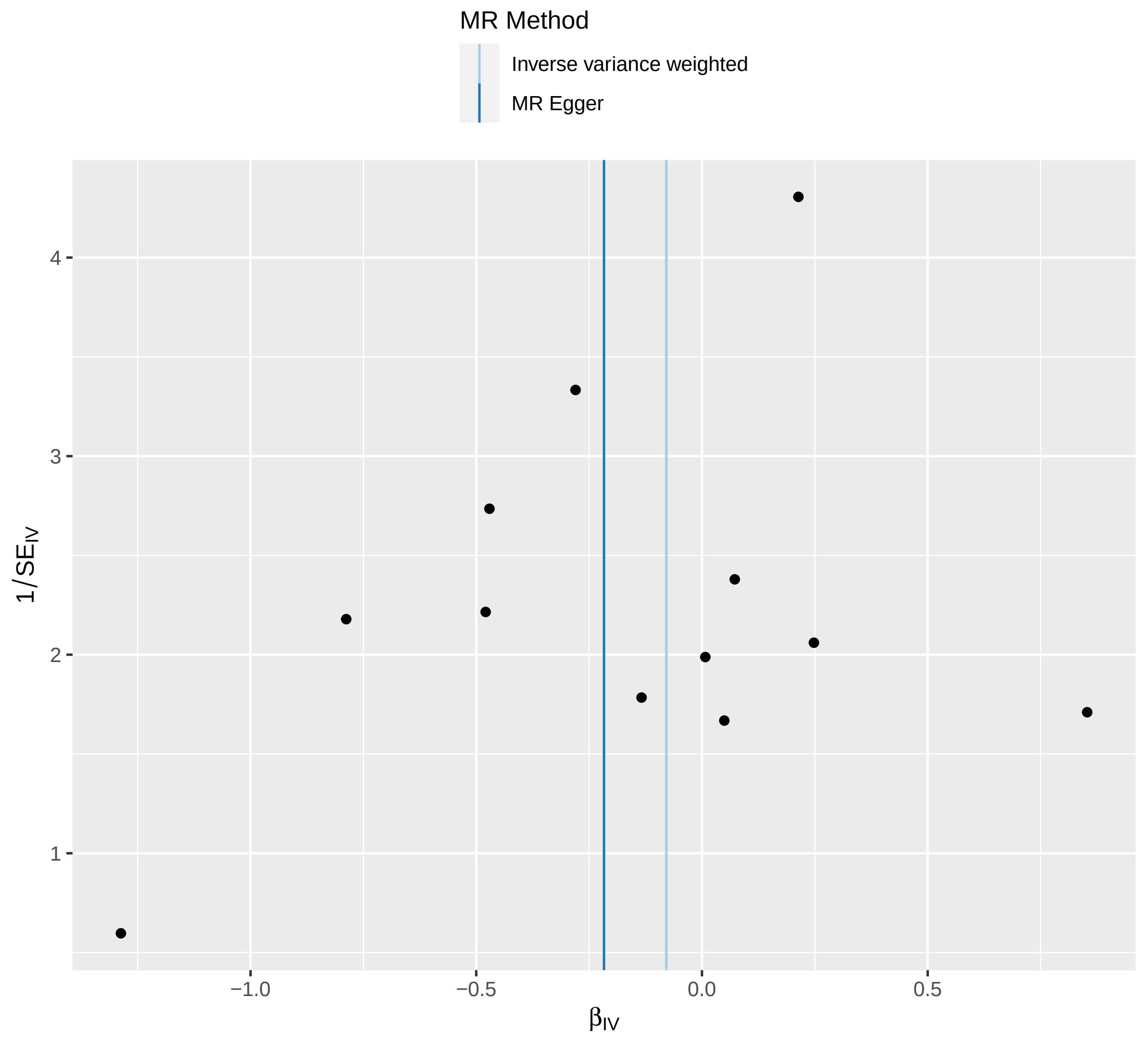

MR Method
Inverse variance weighted
MR Egger
4
3
2
1
1/SE$_{IV}$
−1.0
−0.5
0.0
0.5
β$_{IV}$

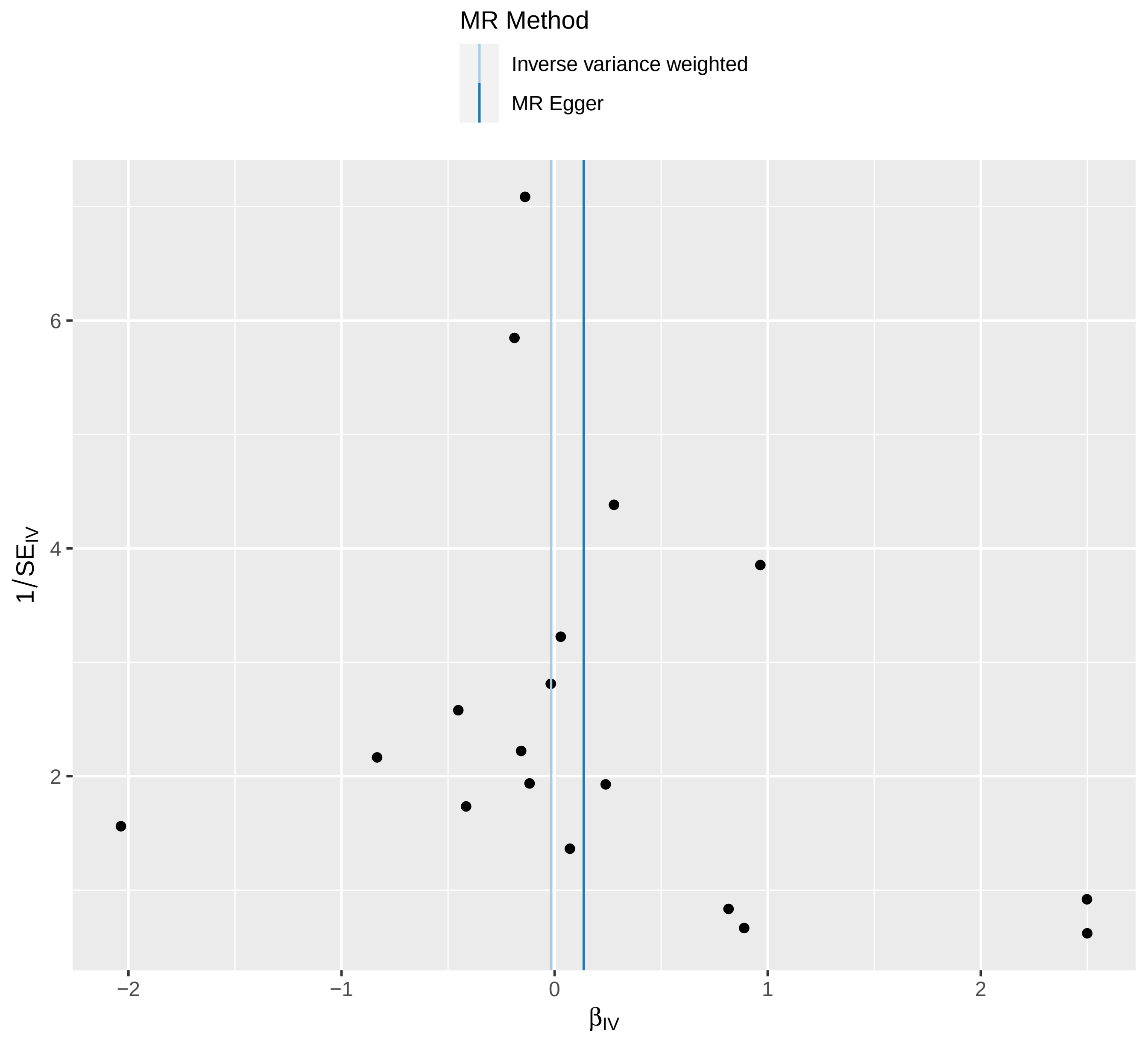

MR Method
Inverse variance weighted
MR Egger
6
4
2
1/SE$_{IV}$
−2
−1
0
1
2
β$_{IV}$

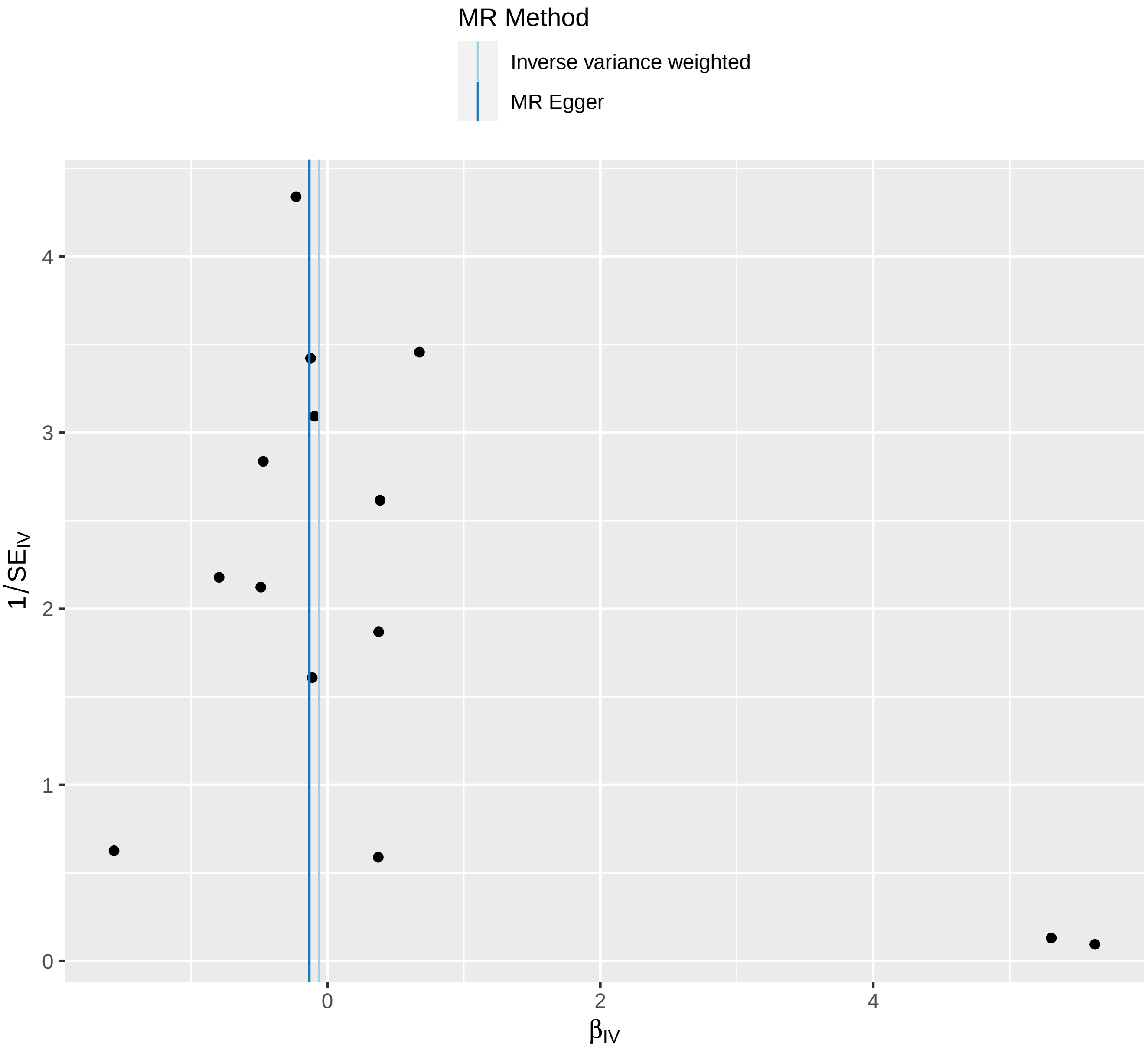

MR Method
Inverse variance weighted
MR Egger
1/SE$_{IV}$
β$_{IV}$
0
1
2
3
4
0
2
4

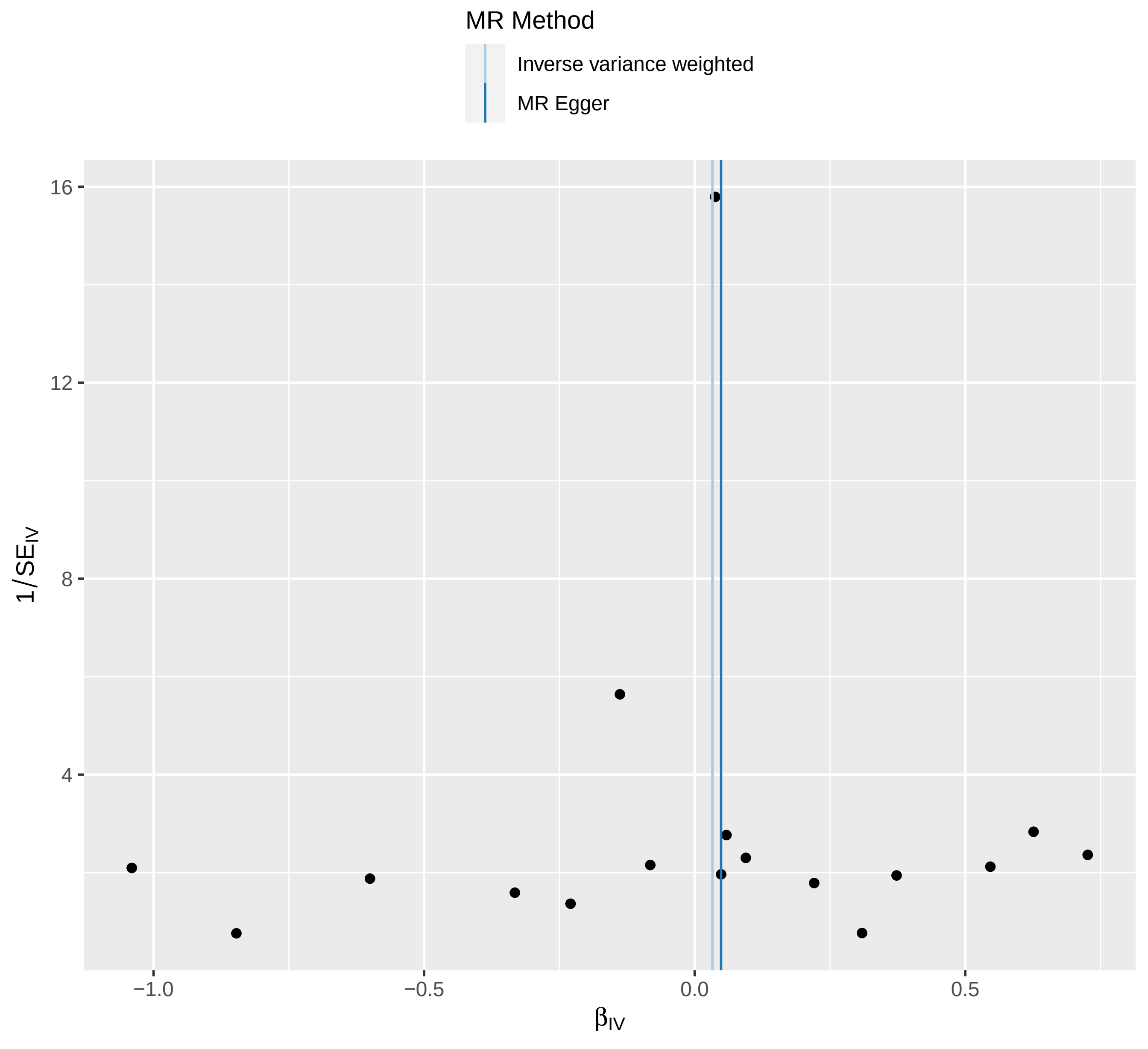

MR Method
Inverse variance weighted
MR Egger
16
12
8
4
1/SE_IV
−1.0
−0.5
0.0
0.5
β_IV

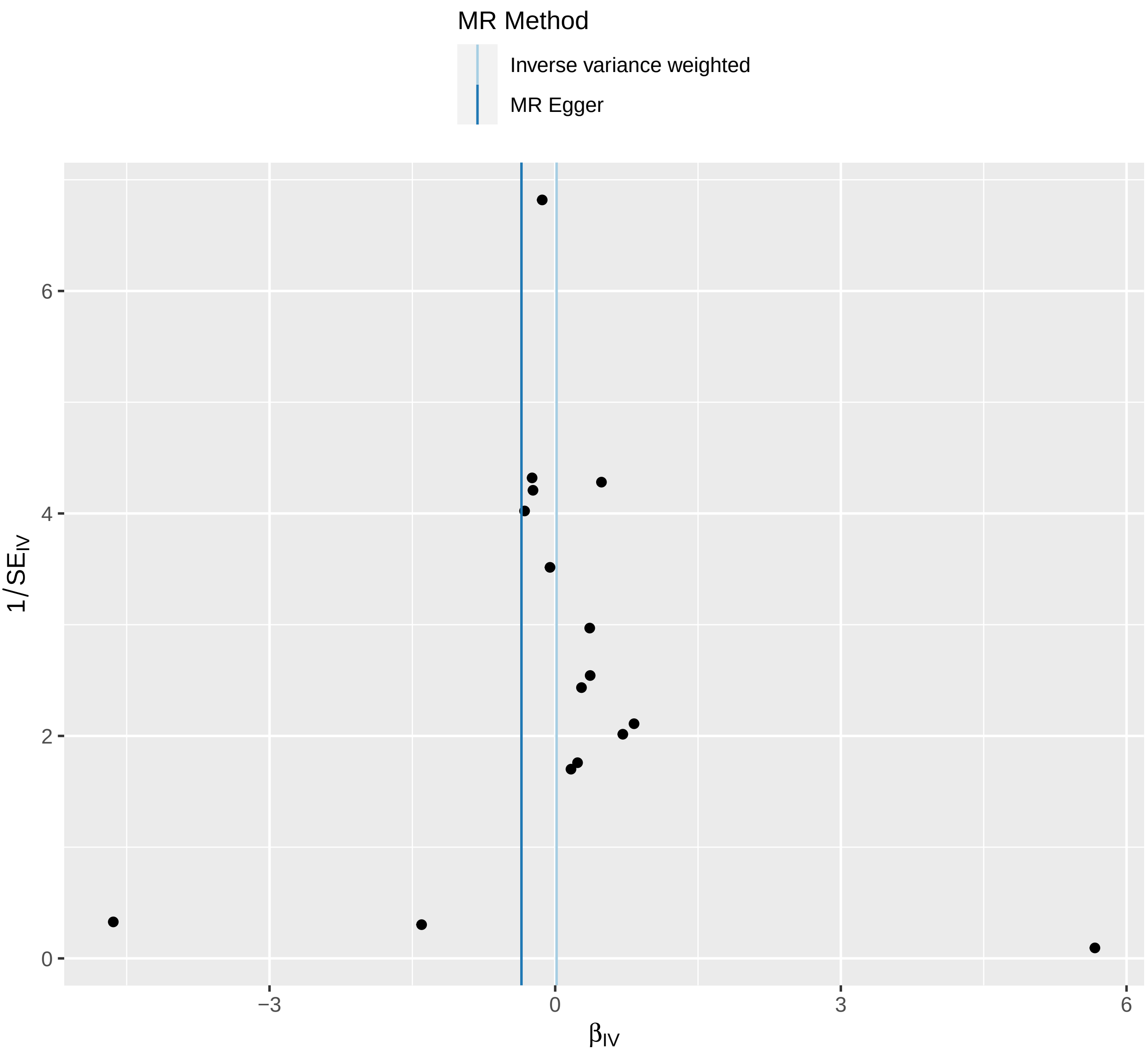
MR Method
Inverse variance weighted
MR Egger
1/SE_IV
β_IV
6
4
2
0
−3
0
3
6

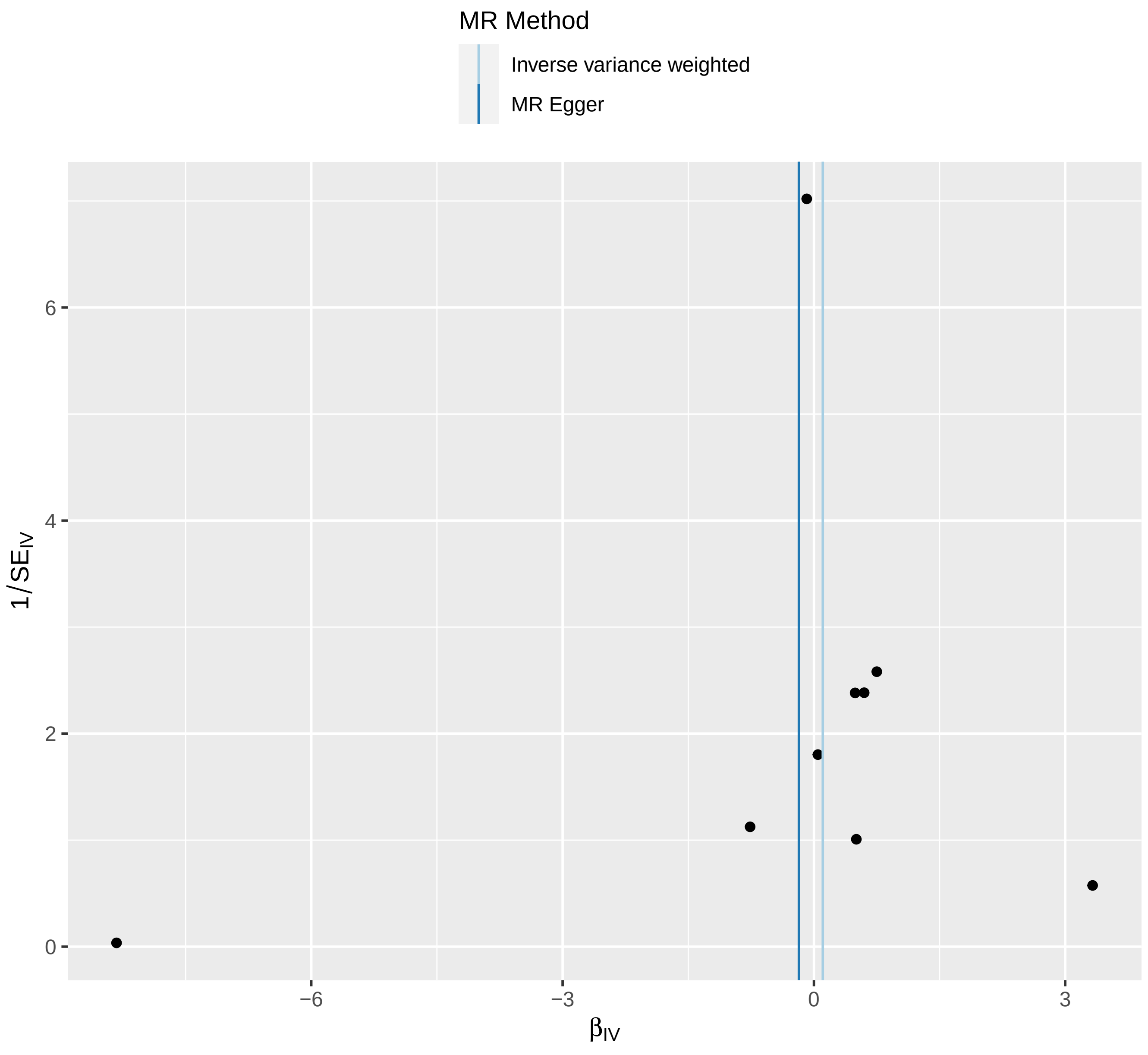
MR Method
Inverse variance weighted
MR Egger
1/SE_{IV}
β_{IV}
0
2
4
6
−6
−3
0
3

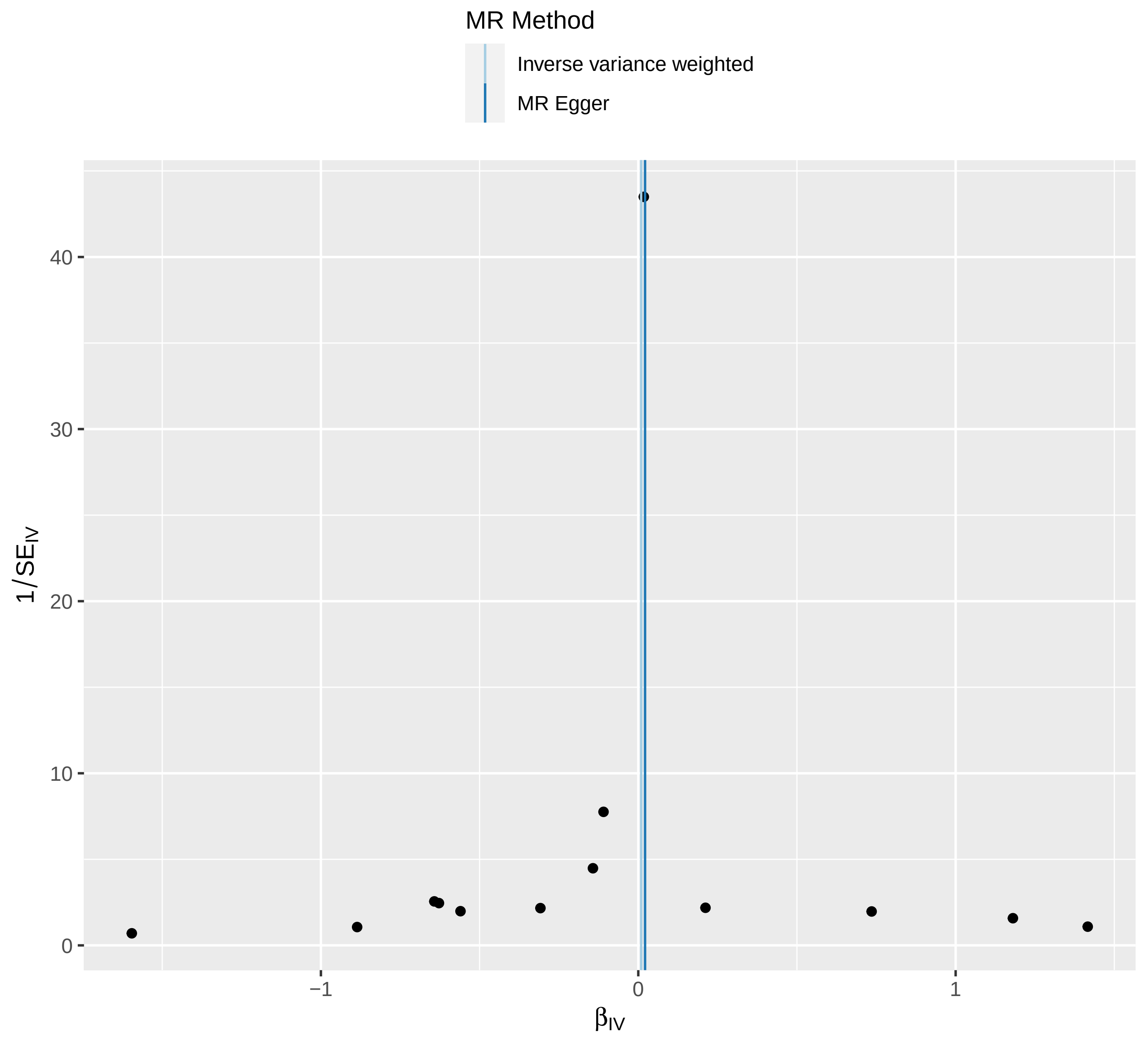
MR Method
Inverse variance weighted
MR Egger
40
30
20
10
0
1/SE$_{IV}$
−1
0
1
β$_{IV}$

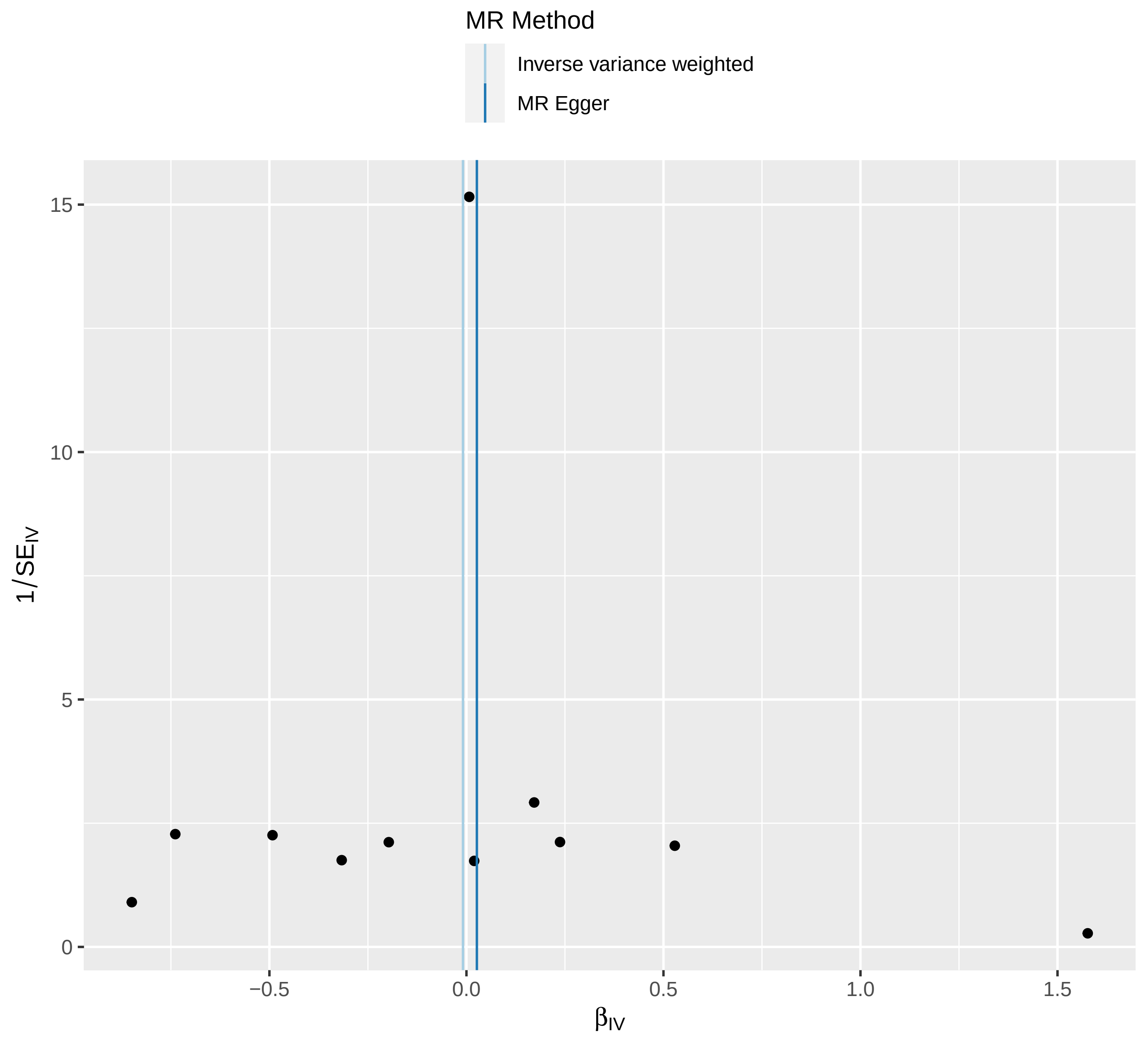

MR Method
Inverse variance weighted
MR Egger
15
10
5
0
1/SE_IV
−0.5
0.0
0.5
1.0
1.5
β_IV

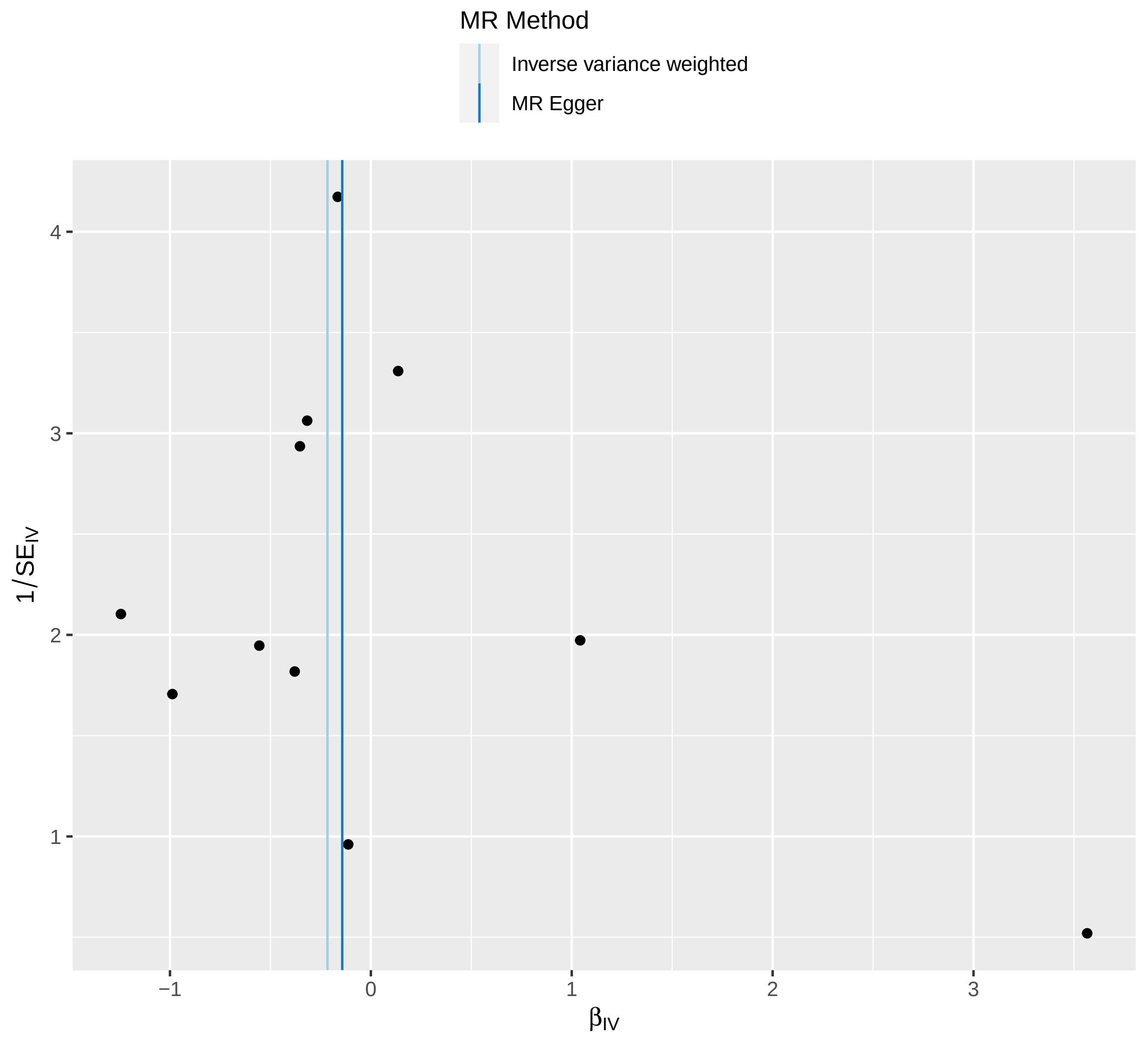
MR Method
Inverse variance weighted
MR Egger
1/SE_IV
β_IV
4
3
2
1
−1
0
1
2
3

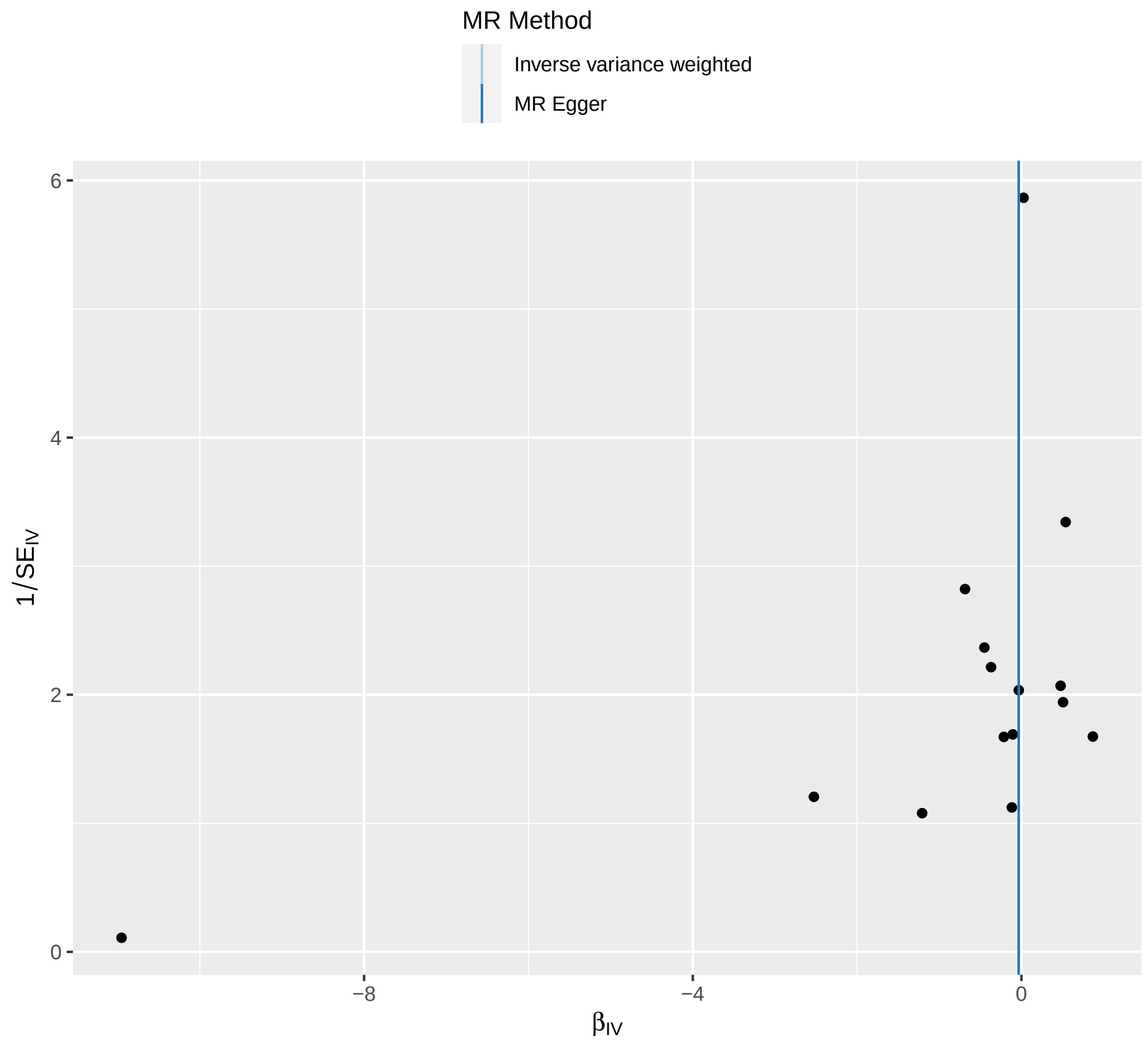

MR Method
Inverse variance weighted
MR Egger
6
4
2
0
1/SE_IV
−8
−4
0
β_IV

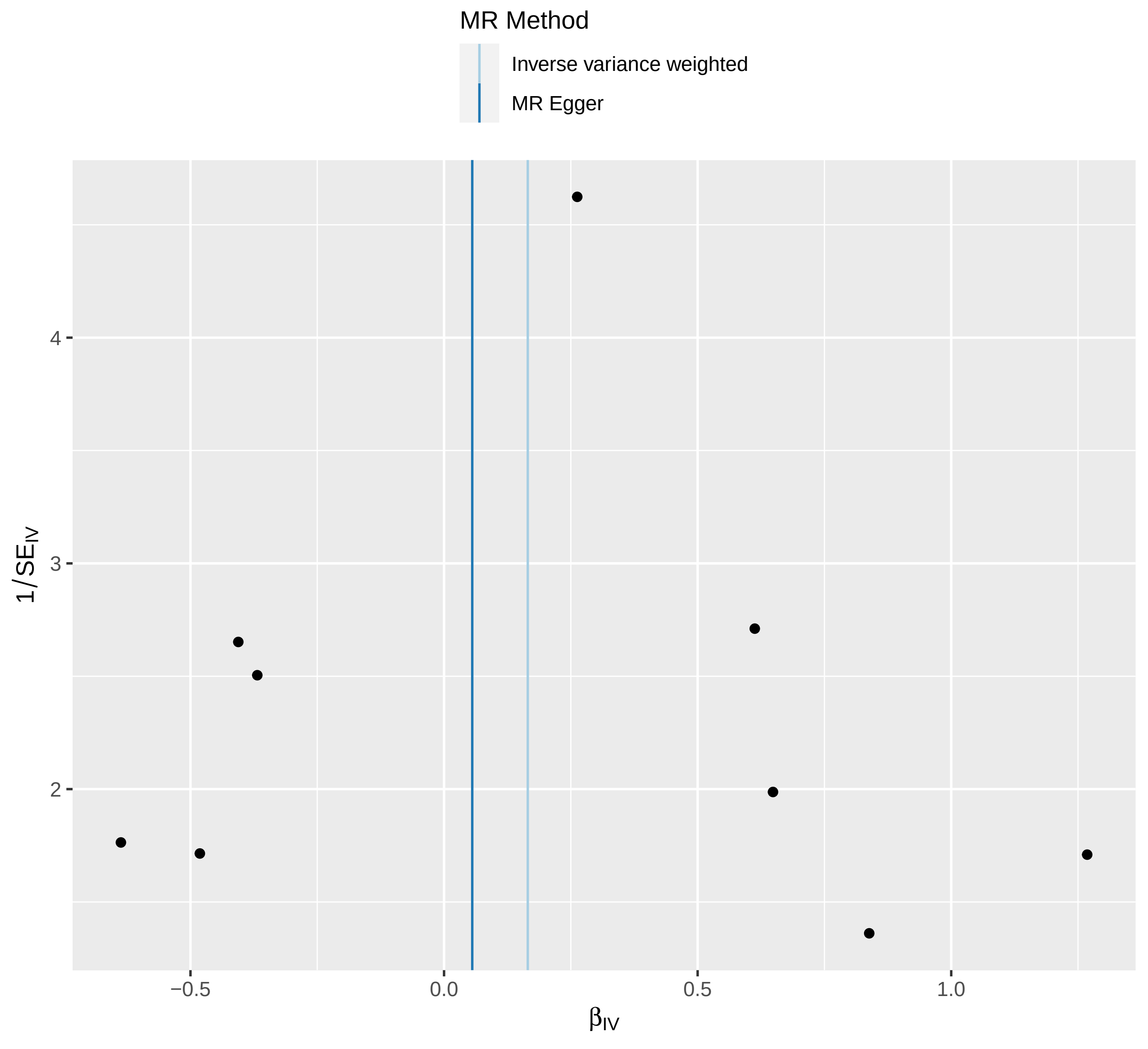

MR Method
Inverse variance weighted
MR Egger
4
3
2
1/SE$_{IV}$
−0.5
0.0
0.5
1.0
β$_{IV}$

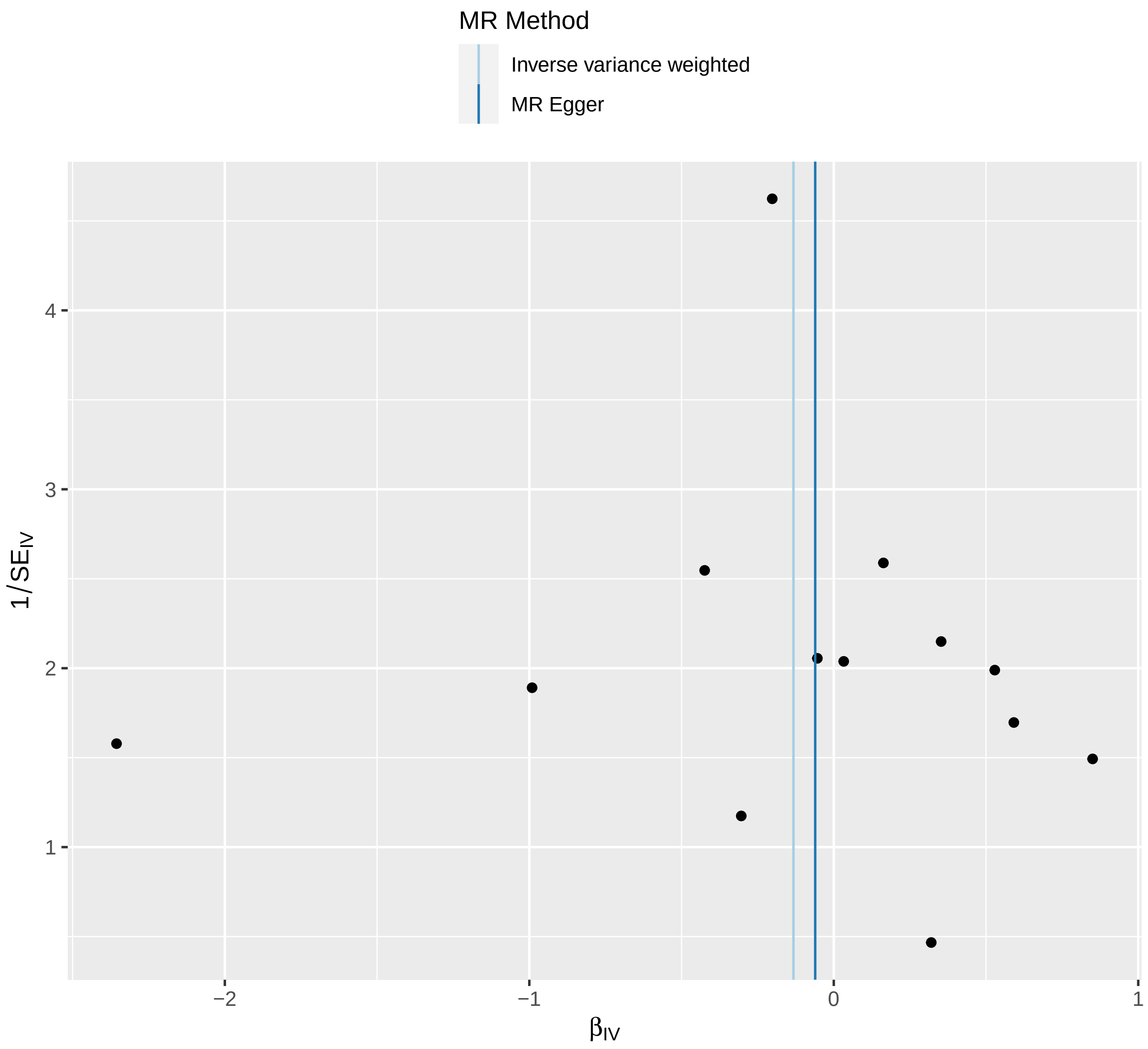

MR Method
Inverse variance weighted
MR Egger
4
3
2
1
1/SE_IV
−2
−1
0
1
β_IV

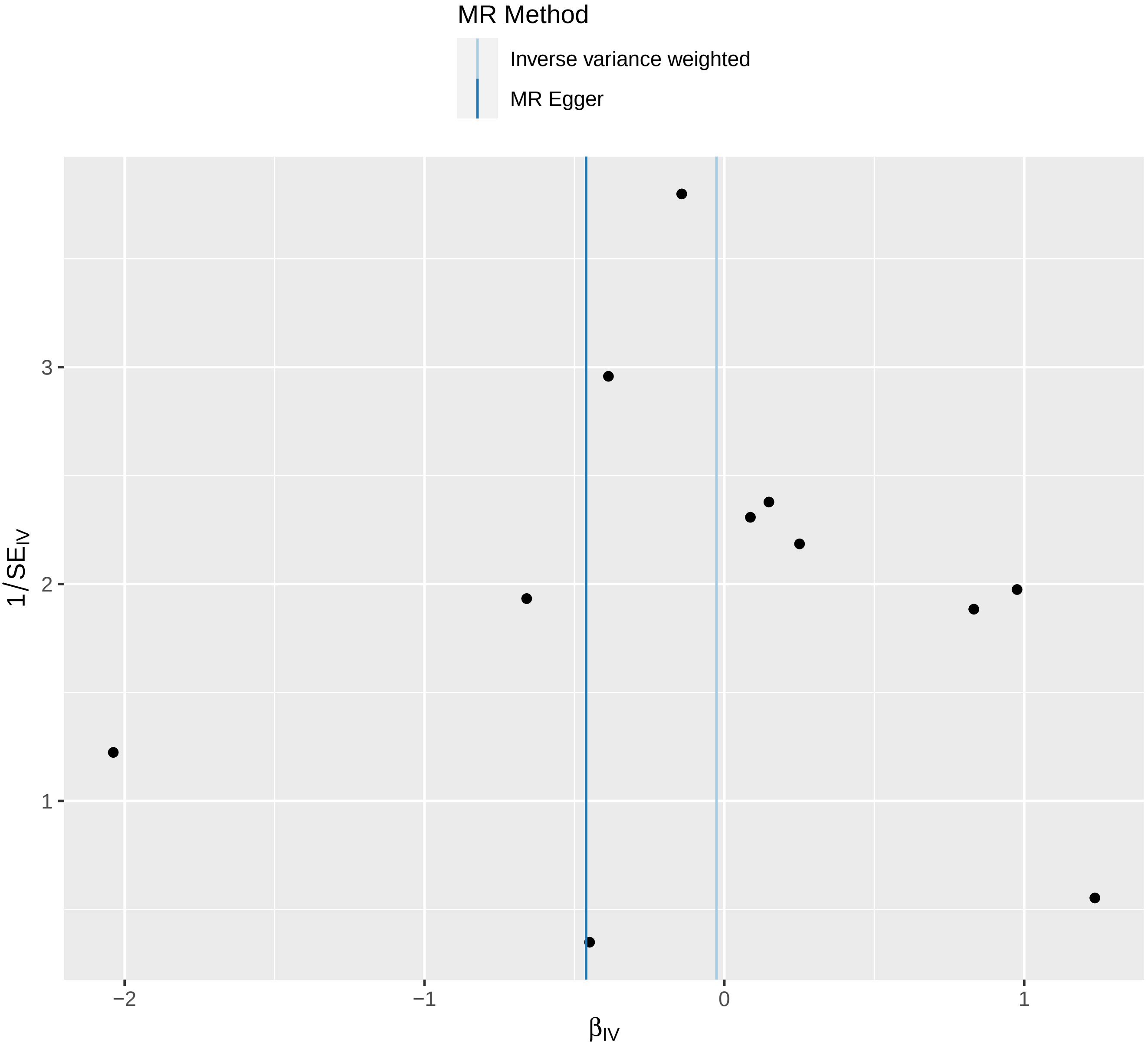

MR Method
Inverse variance weighted
MR Egger
3
2
1
1/SE$_{IV}$
−2
−1
0
1
β$_{IV}$

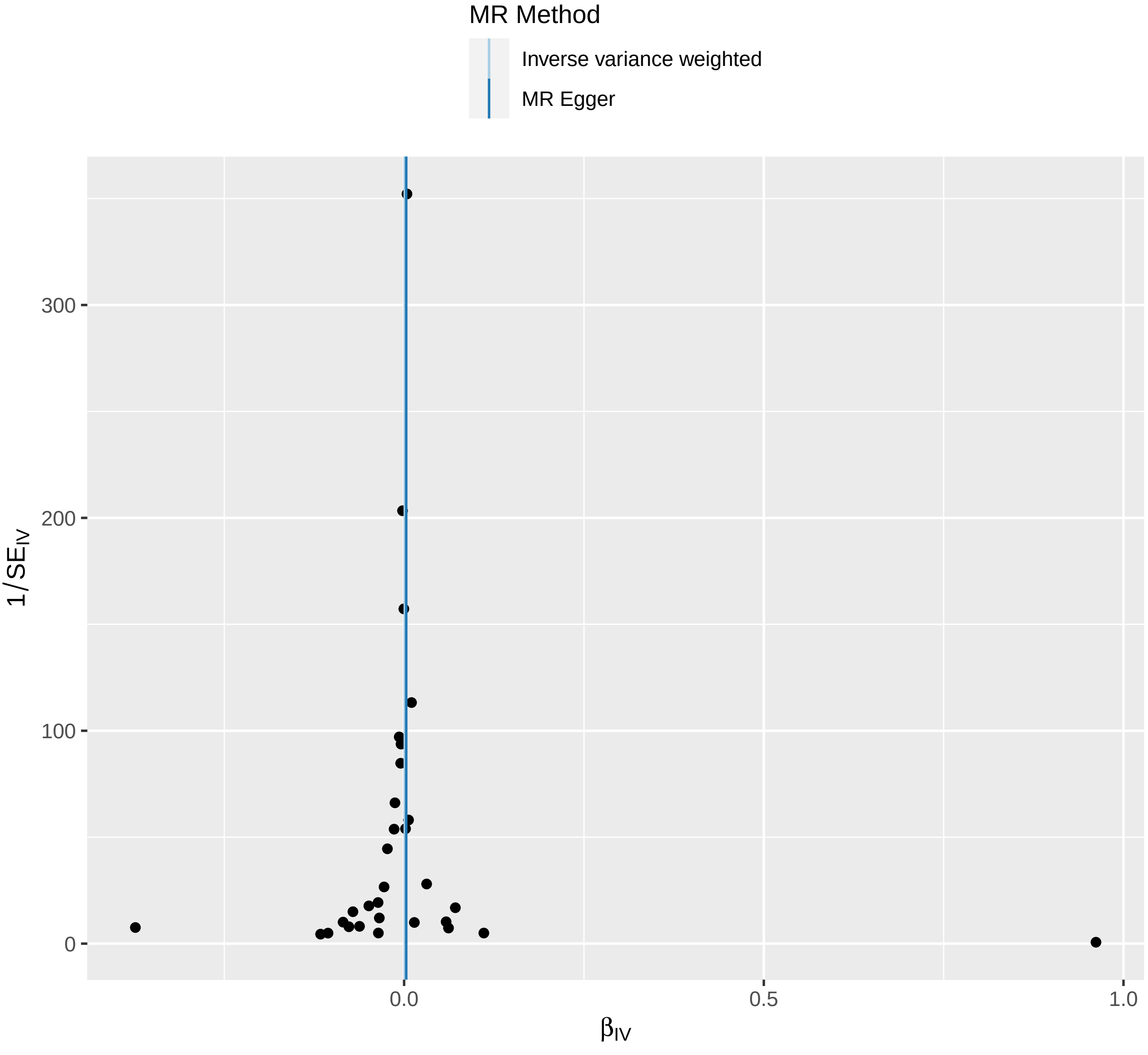
MR Method
Inverse variance weighted
MR Egger
300
200
100
0
0.0
0.5
1.0
$1/SE_{IV}$
β_{IV}

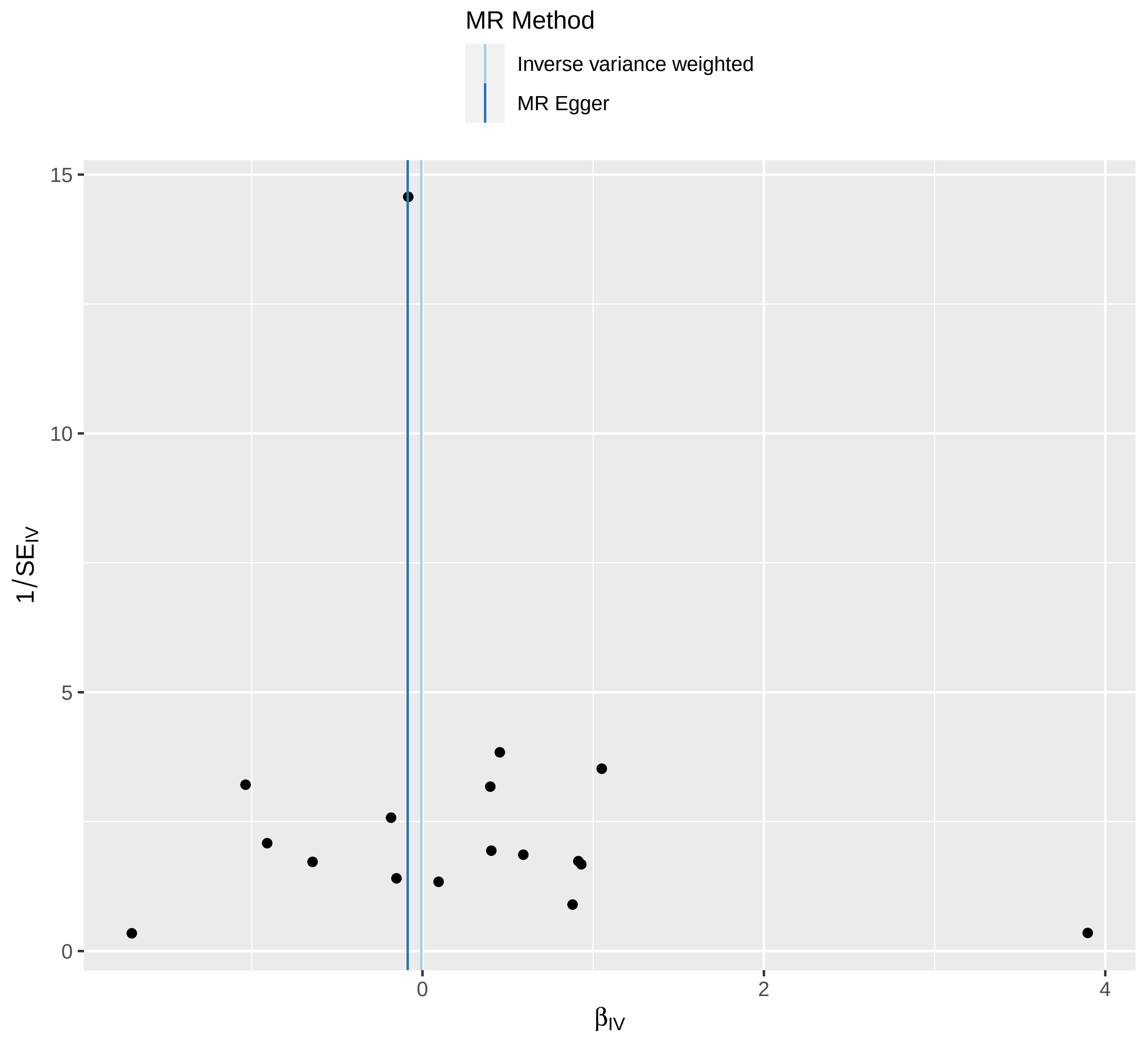
MR Method
Inverse variance weighted
MR Egger
15
10
5
0
1/SE_IV
0
2
4
β_IV

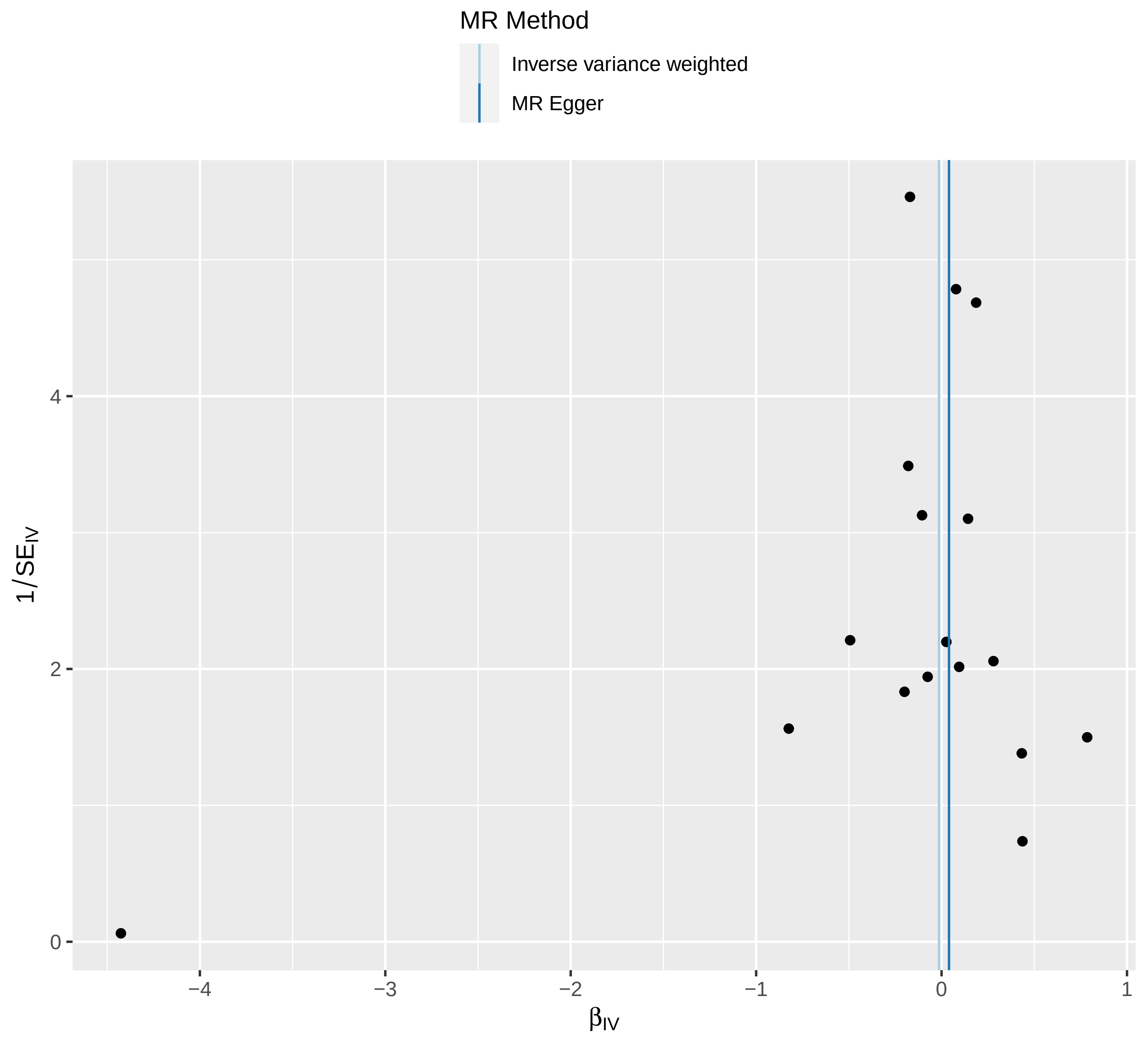
MR Method
Inverse variance weighted
MR Egger
1/SE_IV
β_IV
0
2
4
−4
−3
−2
−1
0
1

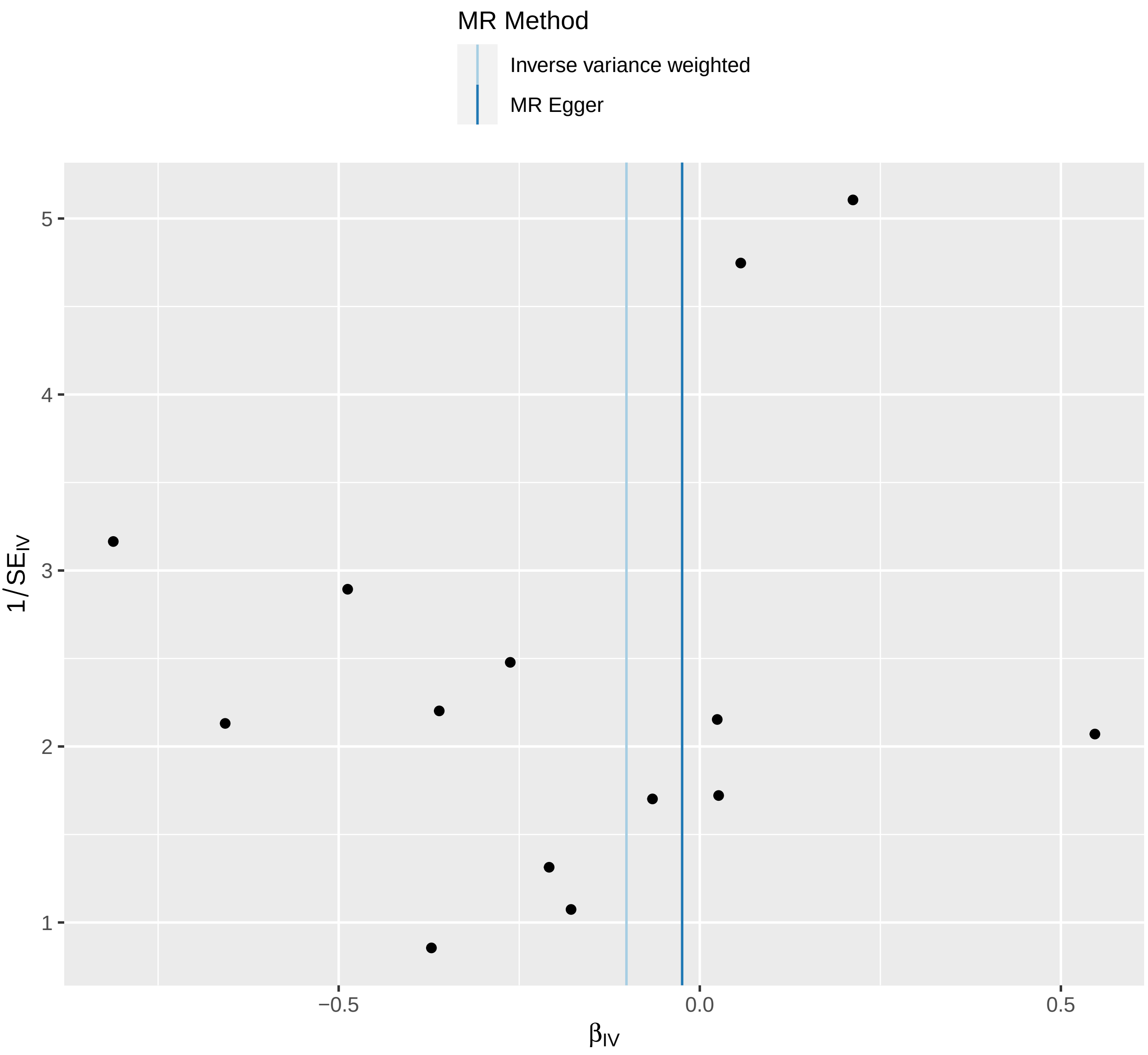

MR Method
Inverse variance weighted
MR Egger
5
4
3
2
1
1/SE_IV
−0.5
0.0
0.5
β_IV

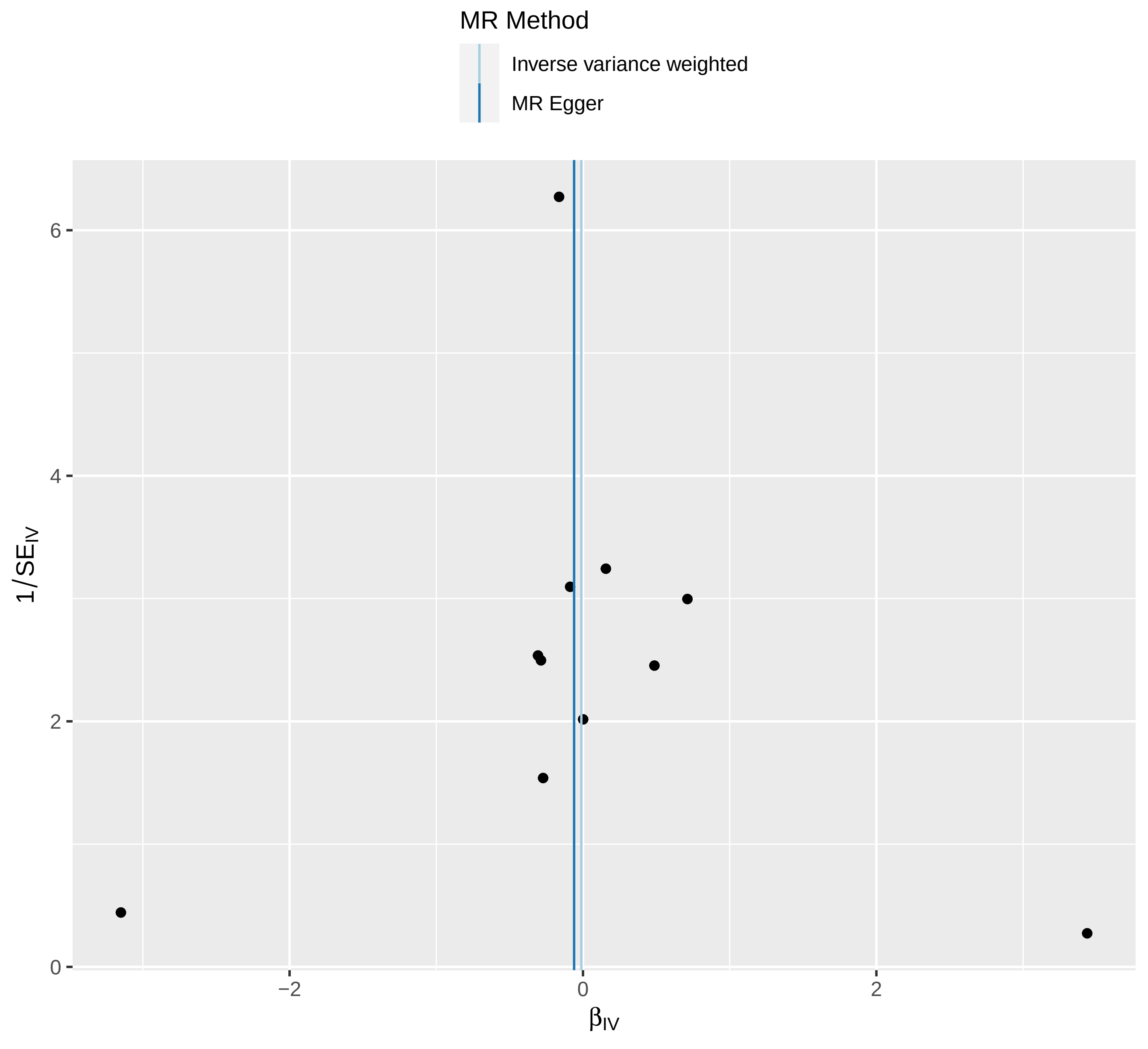

MR Method
Inverse variance weighted
MR Egger
1/SE_IV
6
4
2
0
−2
0
2
β_IV

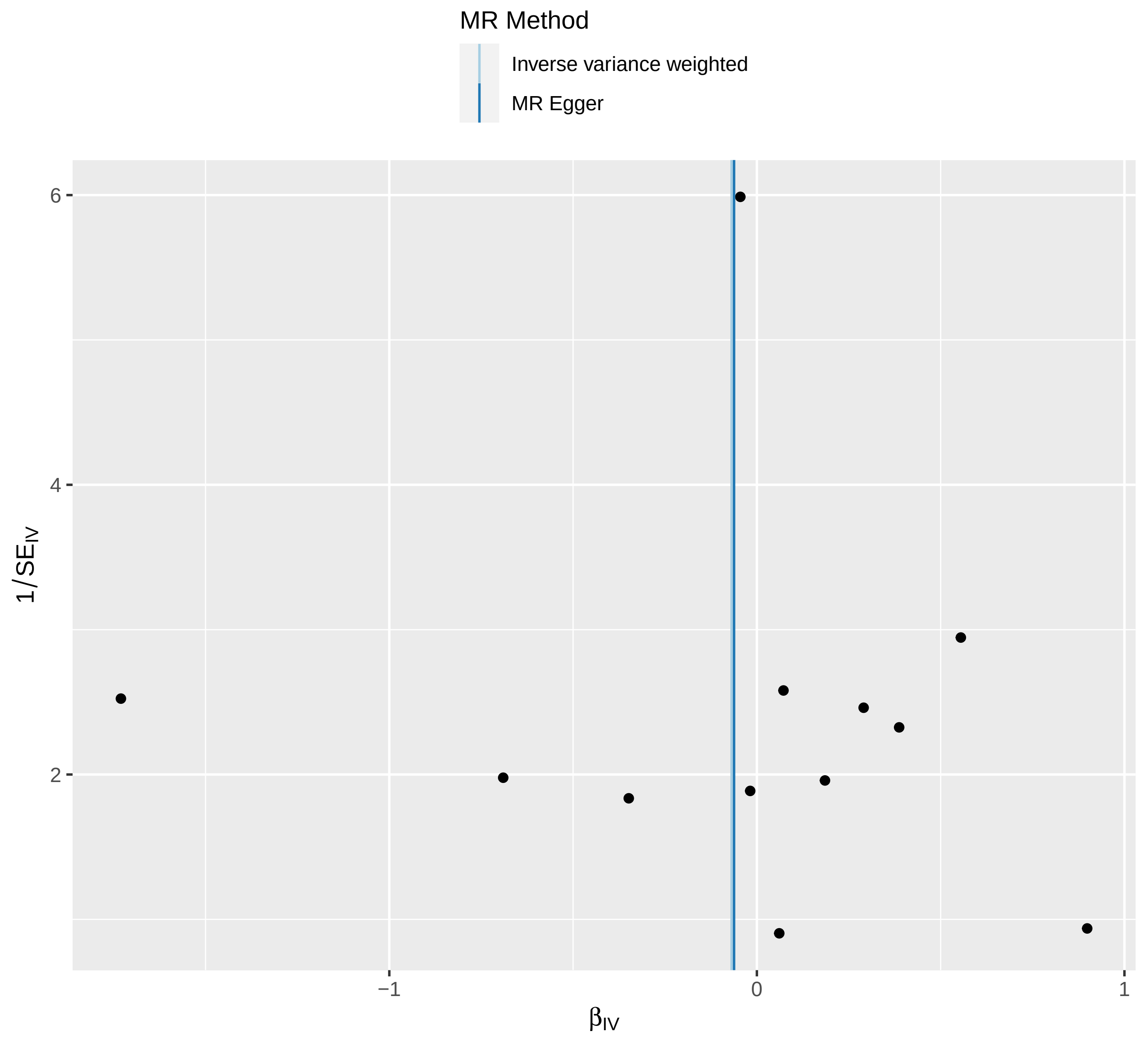
MR Method
Inverse variance weighted
MR Egger
1/SE_IV
β_IV
6
4
2
−1
0
1

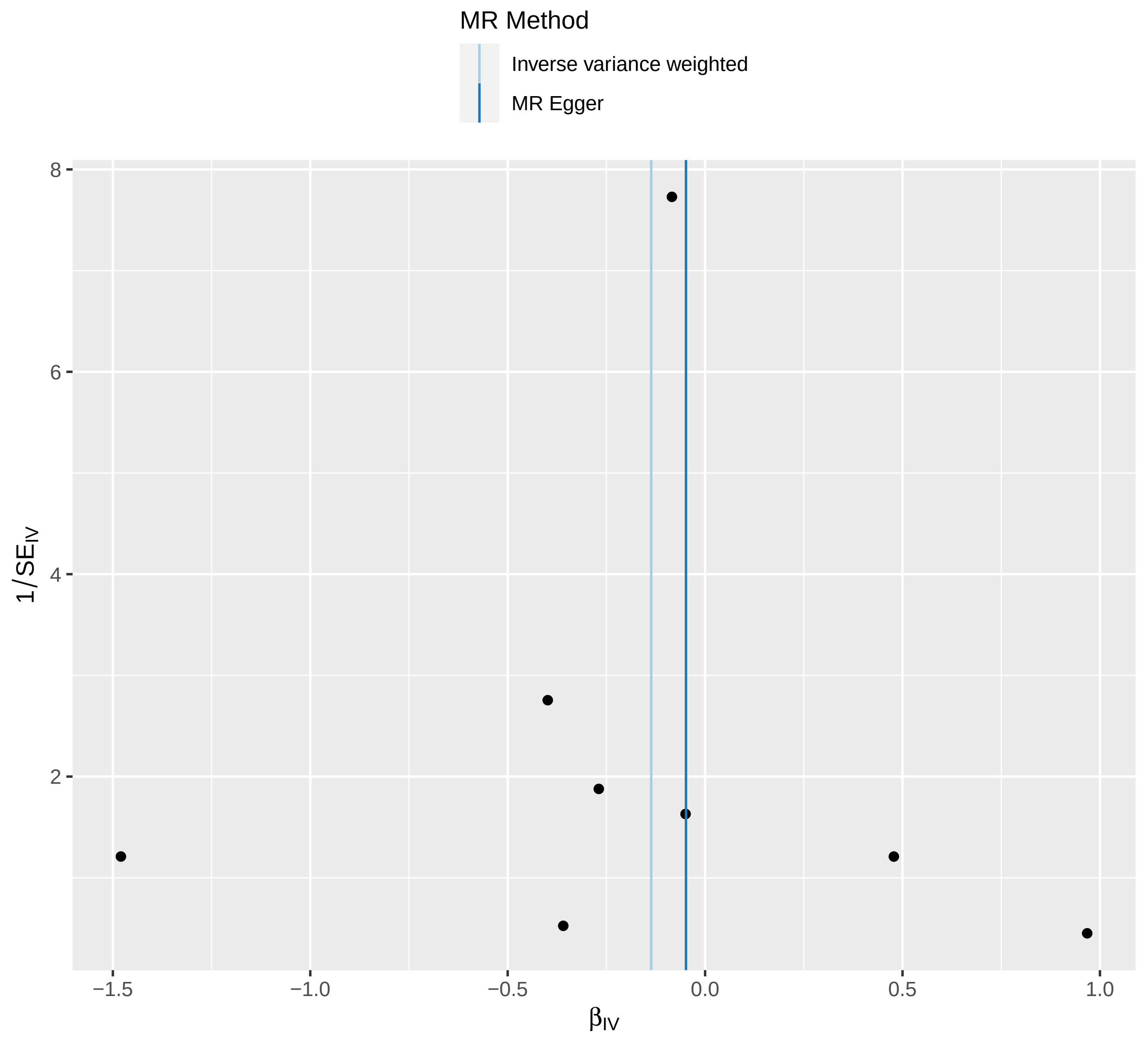
MR Method
Inverse variance weighted
MR Egger
1/SE_IV
β_IV
8
6
4
2
−1.5
−1.0
−0.5
0.0
0.5
1.0

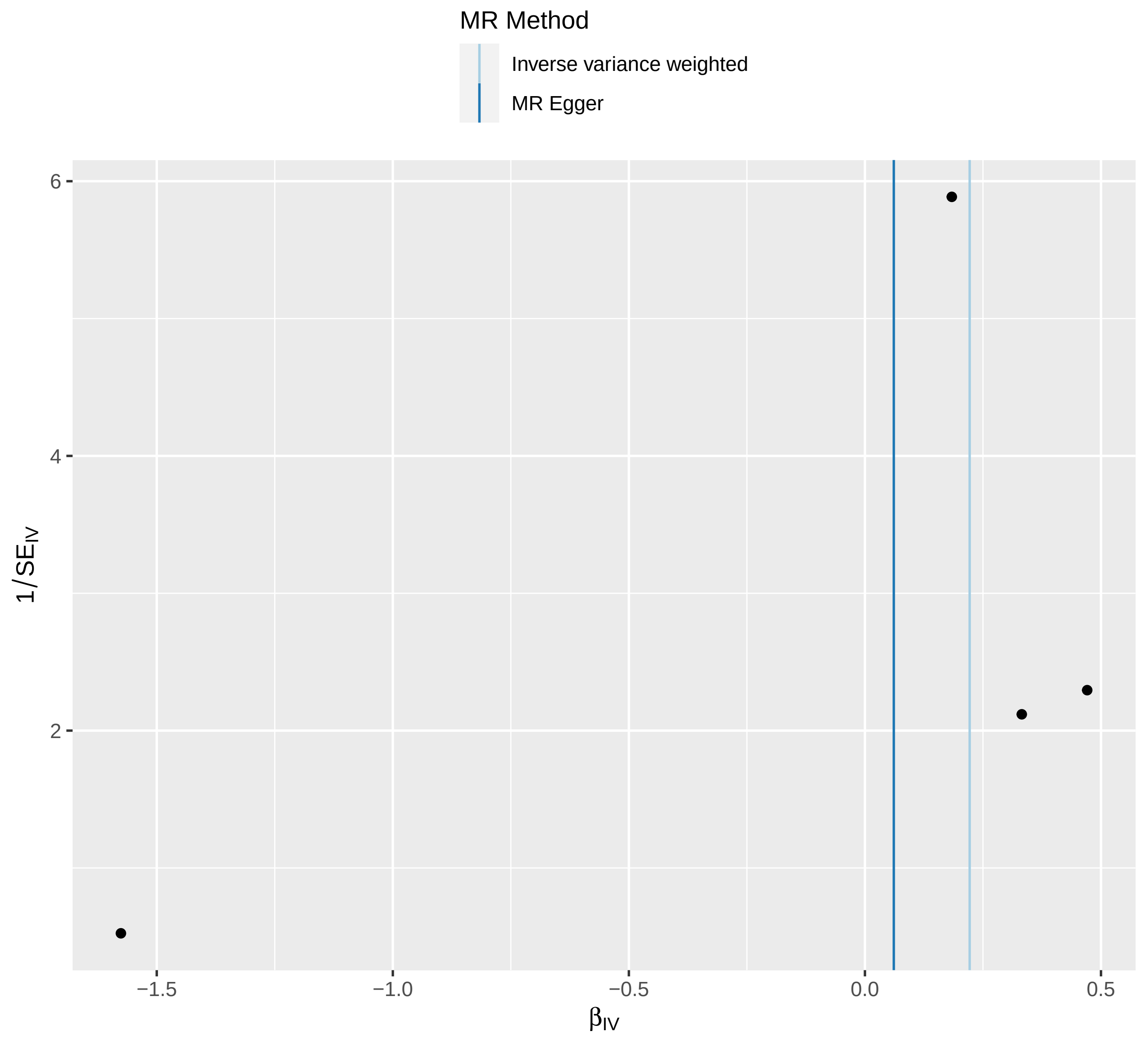
MR Method
Inverse variance weighted
MR Egger
6
4
2
1/SE$_{IV}$
−1.5
−1.0
−0.5
0.0
0.5
β$_{IV}$

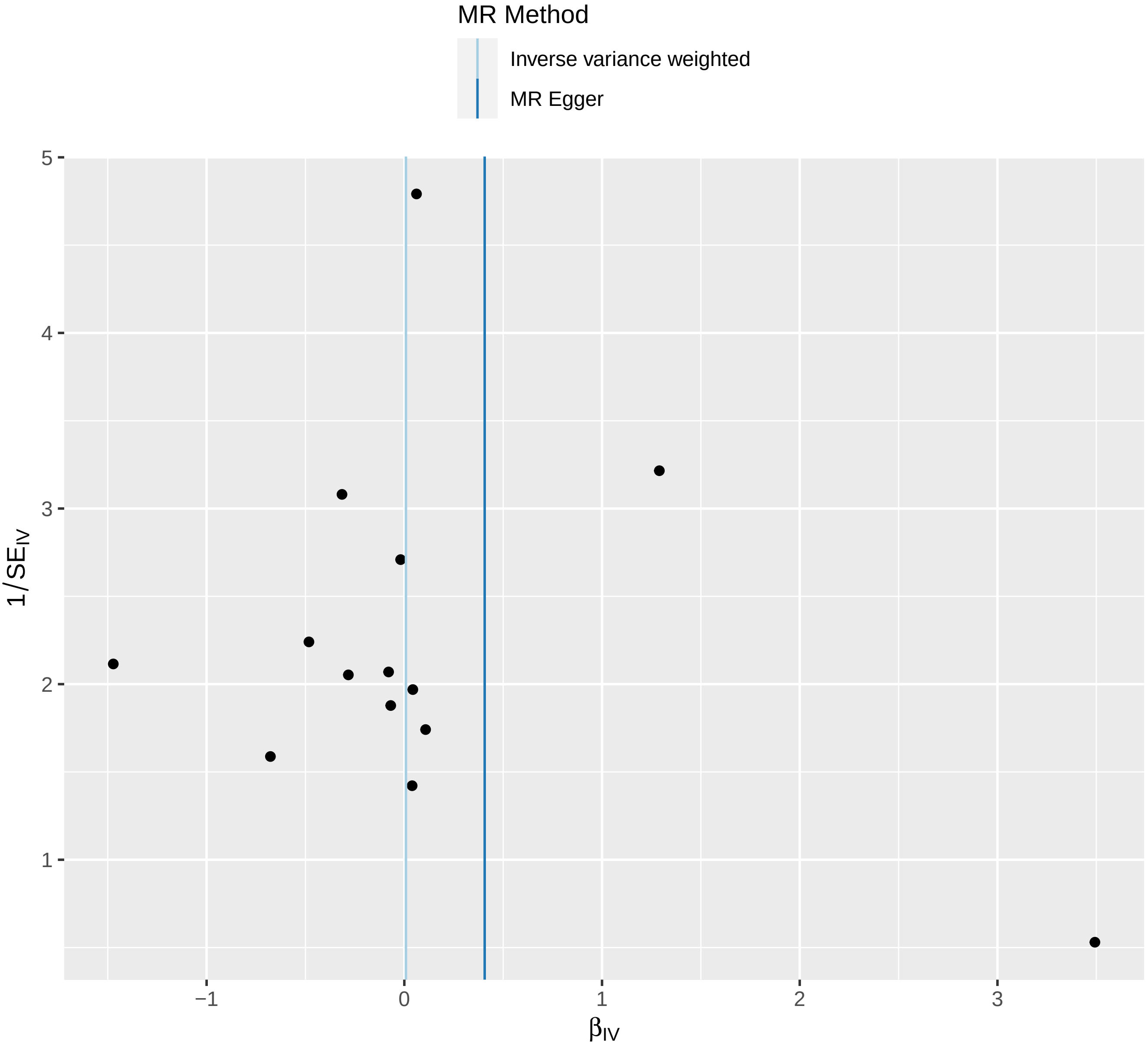

MR Method
Inverse variance weighted
MR Egger
5
4
3
2
1
1/SE_IV
−1
0
1
2
3
β_IV

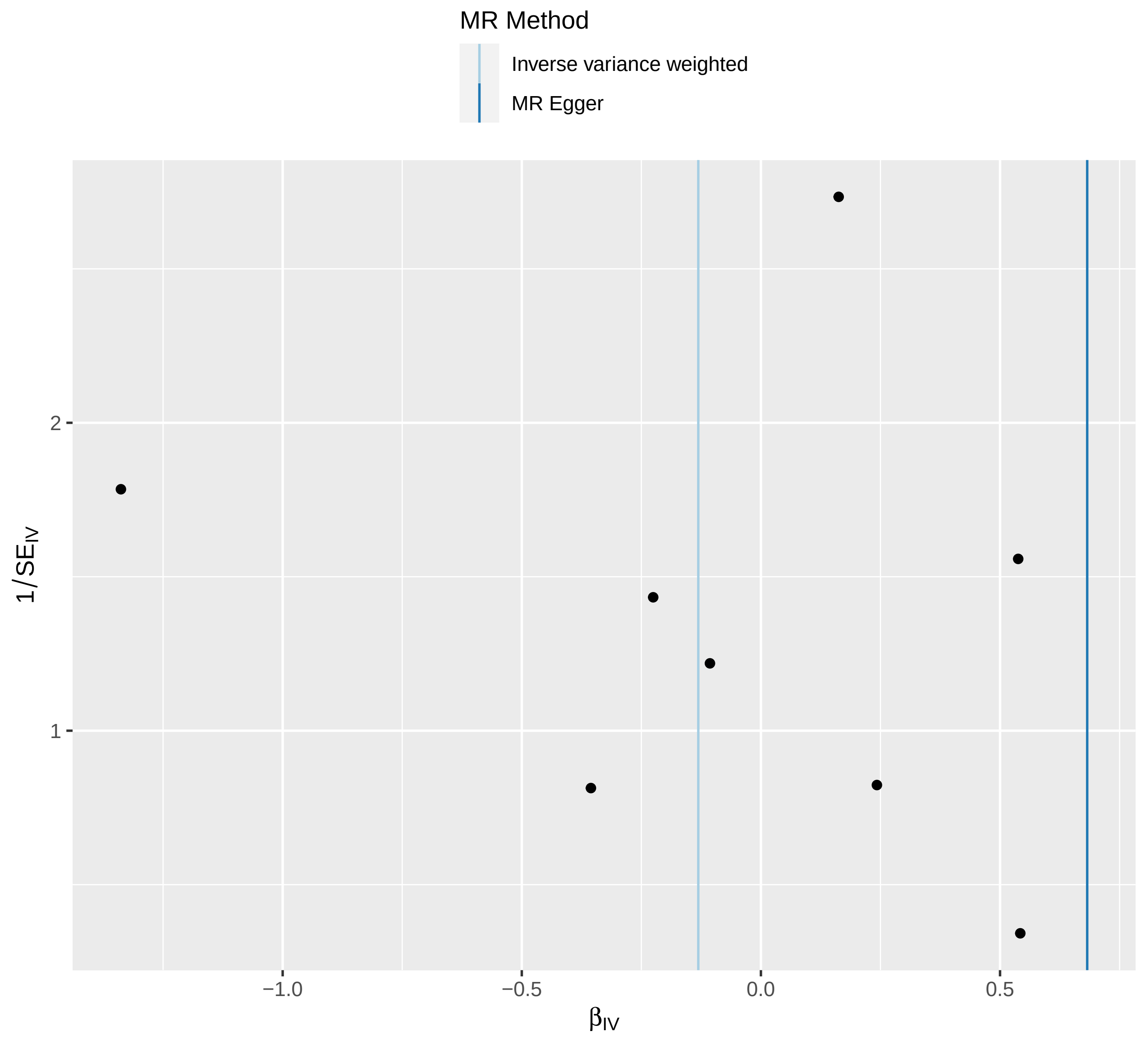

MR Method
Inverse variance weighted
MR Egger
2
1
1/SE_IV
−1.0
−0.5
0.0
0.5
β_IV

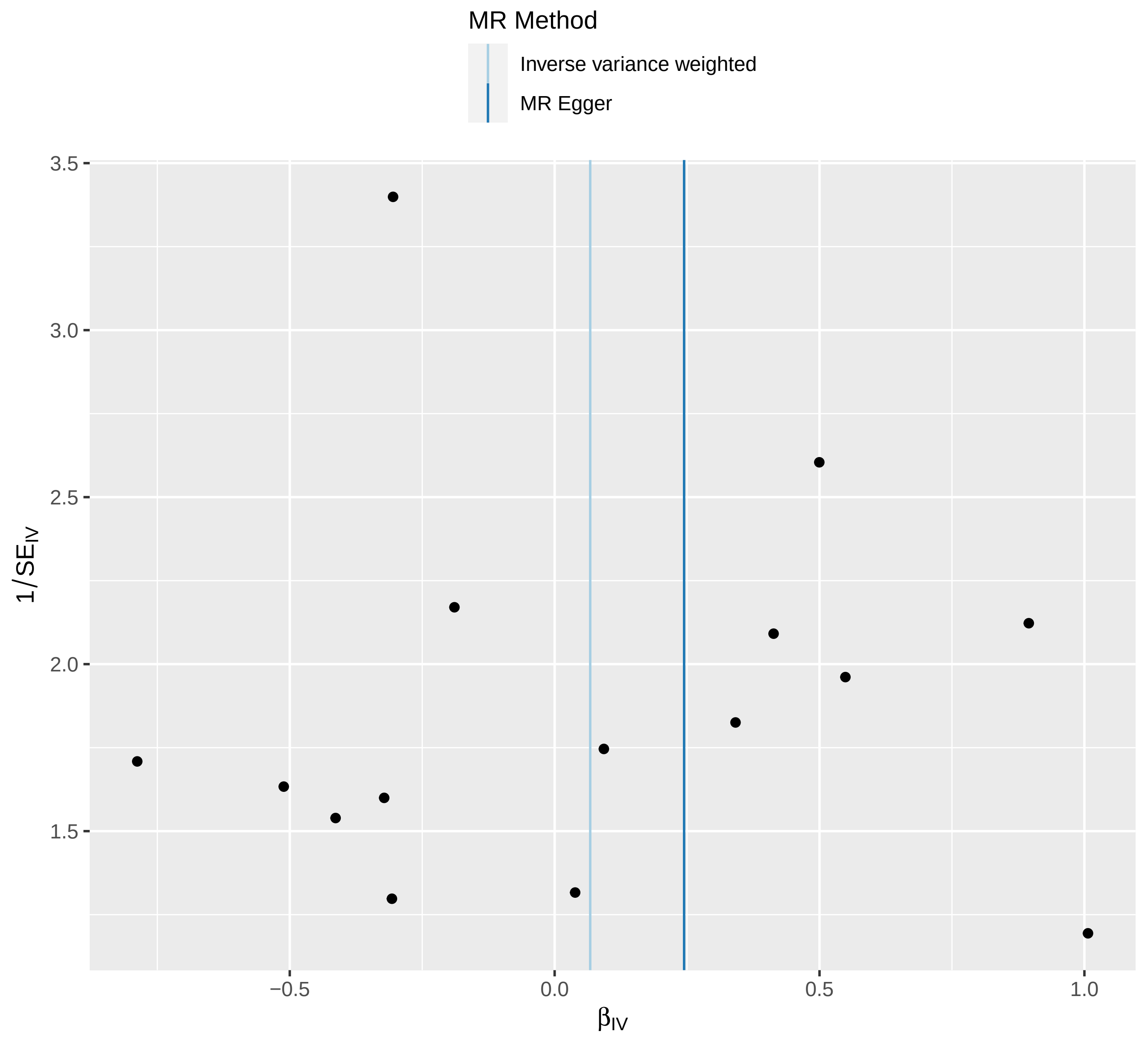

MR Method
Inverse variance weighted
MR Egger
3.5
3.0
2.5
2.0
1.5
1/SE$_{IV}$
−0.5
0.0
0.5
1.0
β$_{IV}$

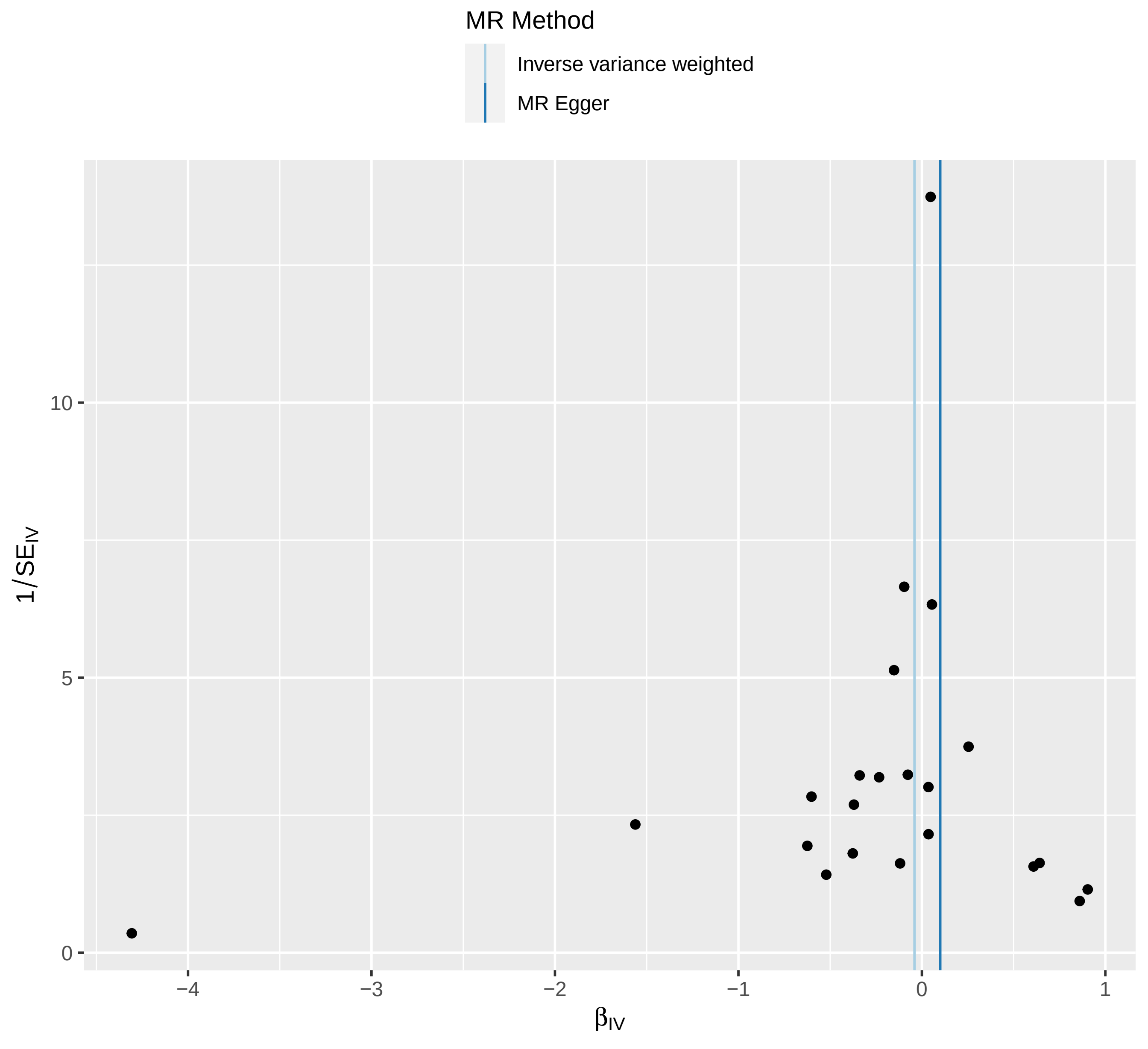
MR Method
Inverse variance weighted
MR Egger
1/SE$_{IV}$
10
5
0
−4
−3
−2
−1
0
1
β$_{IV}$

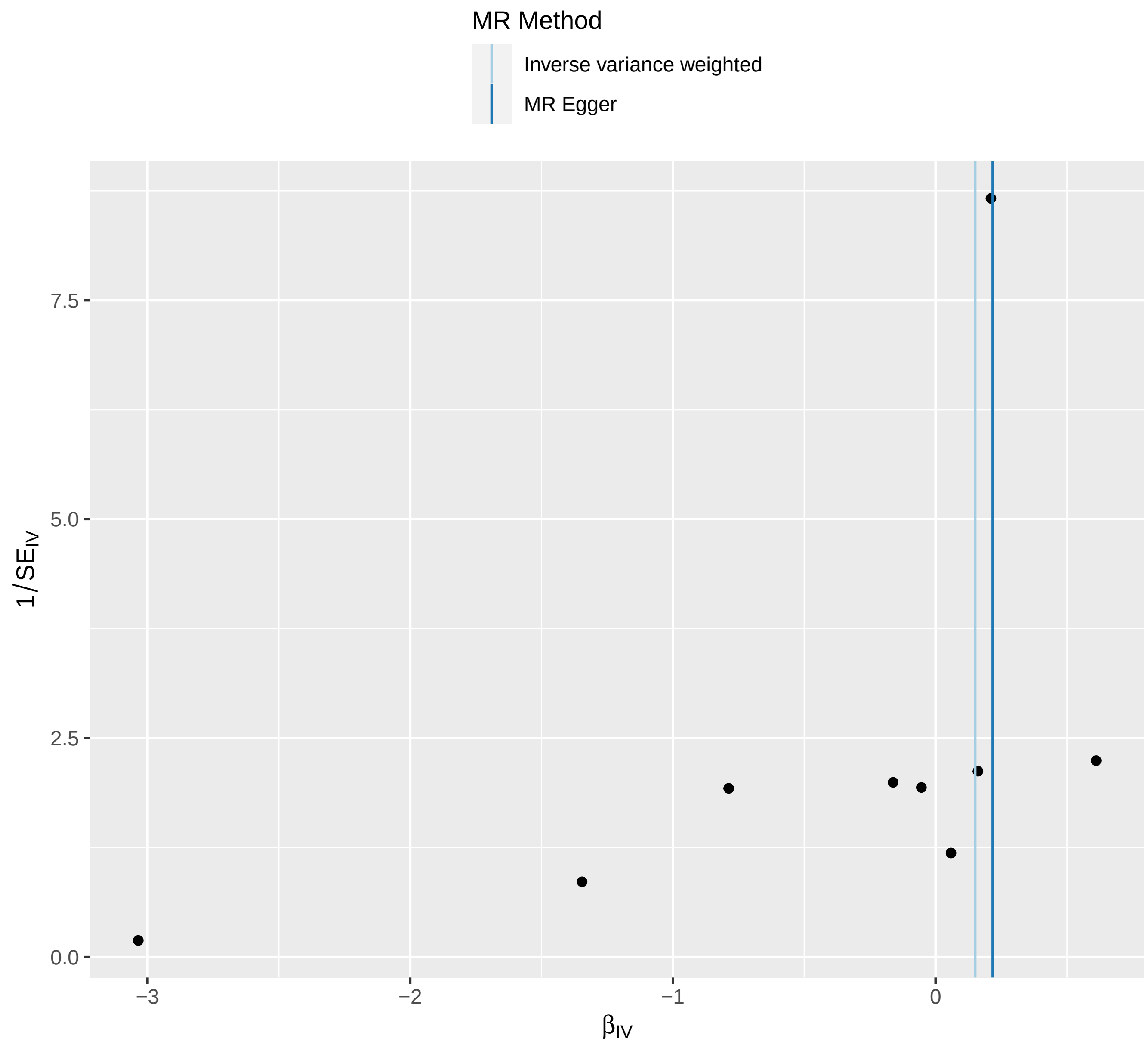
MR Method
Inverse variance weighted
MR Egger
7.5
5.0
2.5
0.0
1/SE$_{IV}$
−3
−2
−1
0
β$_{IV}$

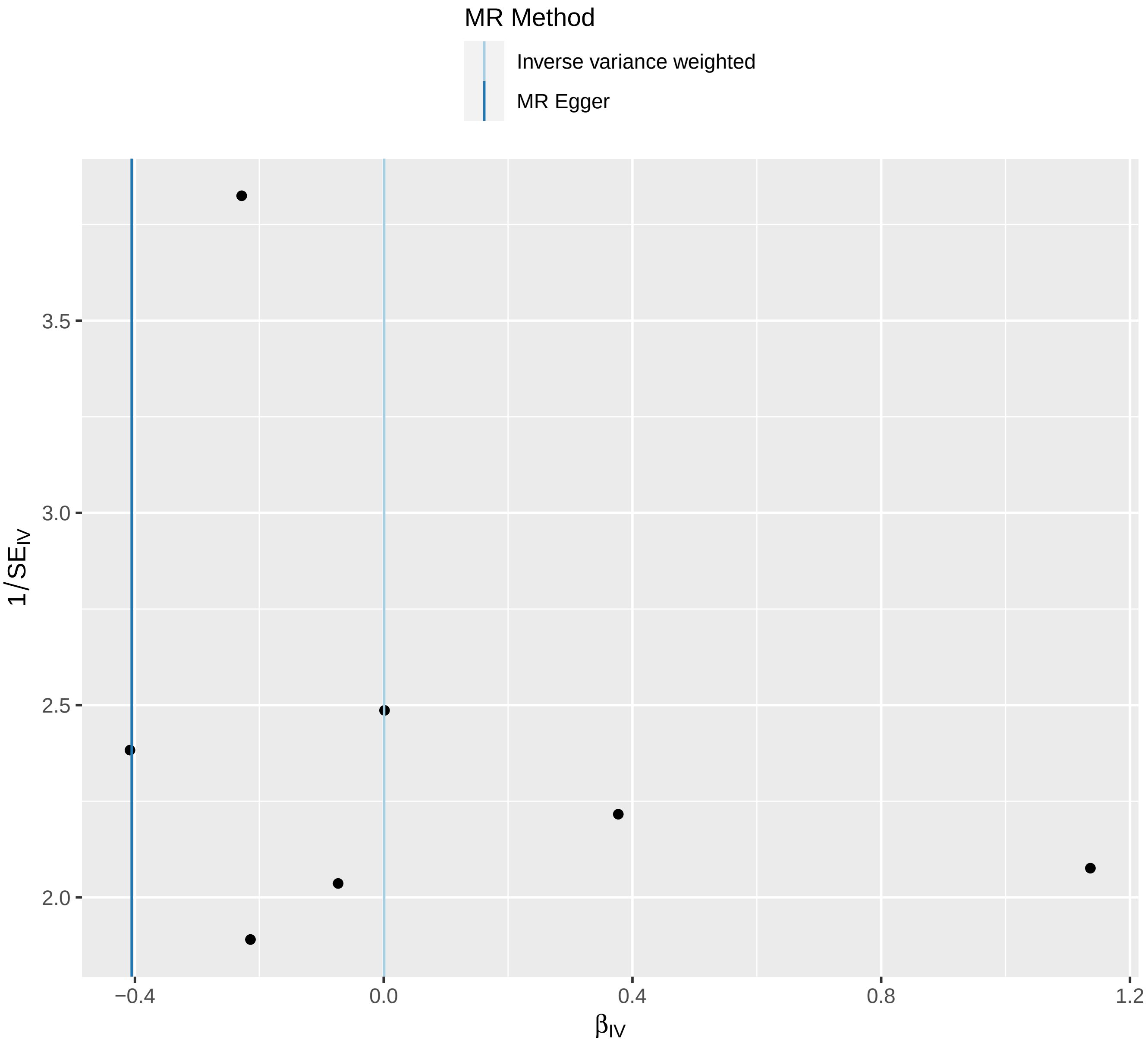

MR Method
Inverse variance weighted
MR Egger
3.5
3.0
2.5
2.0
1/SE_IV
−0.4
0.0
0.4
0.8
1.2
β_IV

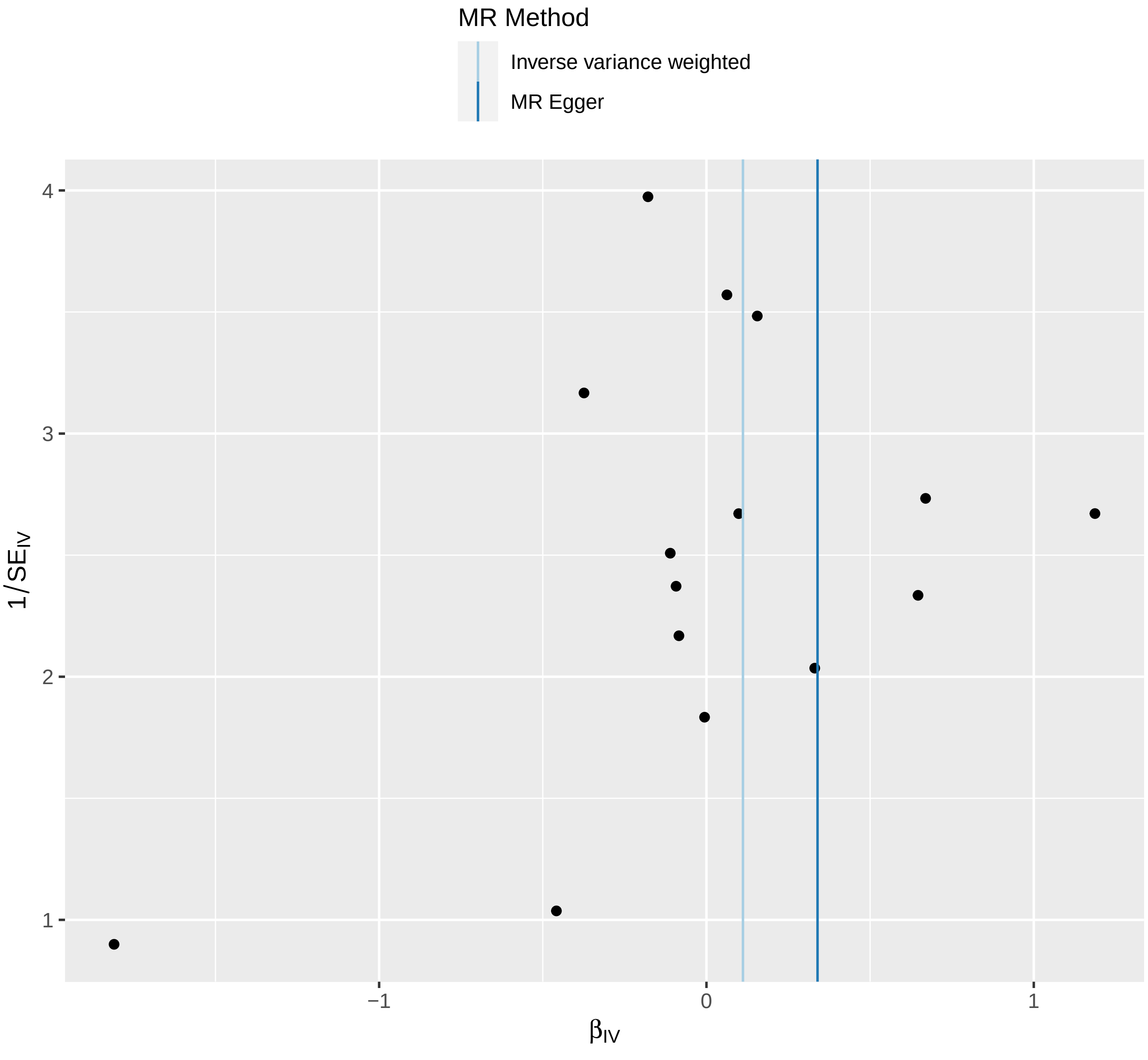
MR Method
Inverse variance weighted
MR Egger
4
3
2
1
$1/SE_{IV}$
−1
0
1
β_{IV}

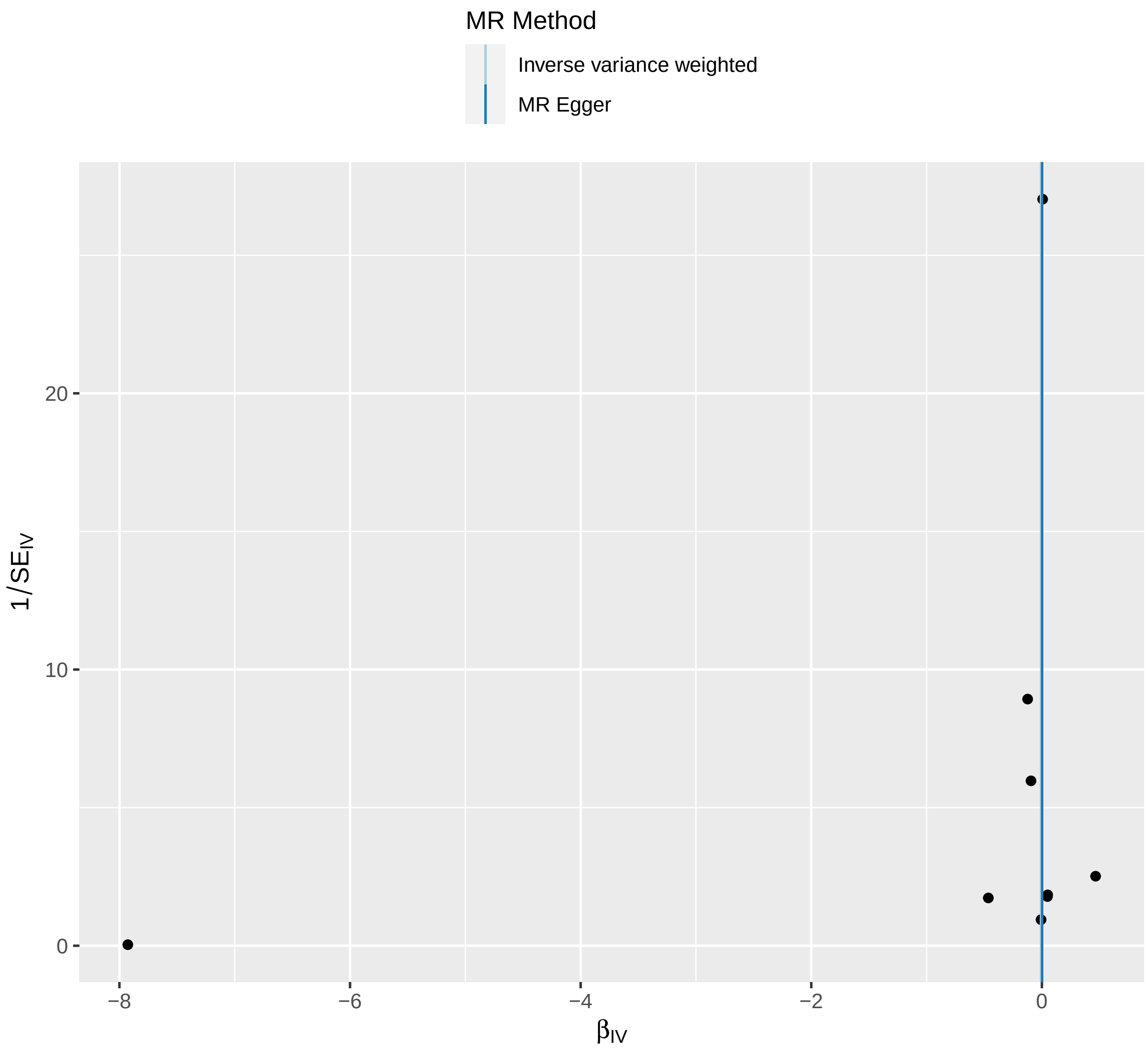
MR Method
Inverse variance weighted
MR Egger
20
10
0
−8
−6
−4
−2
0
1/SE_IV
β_IV

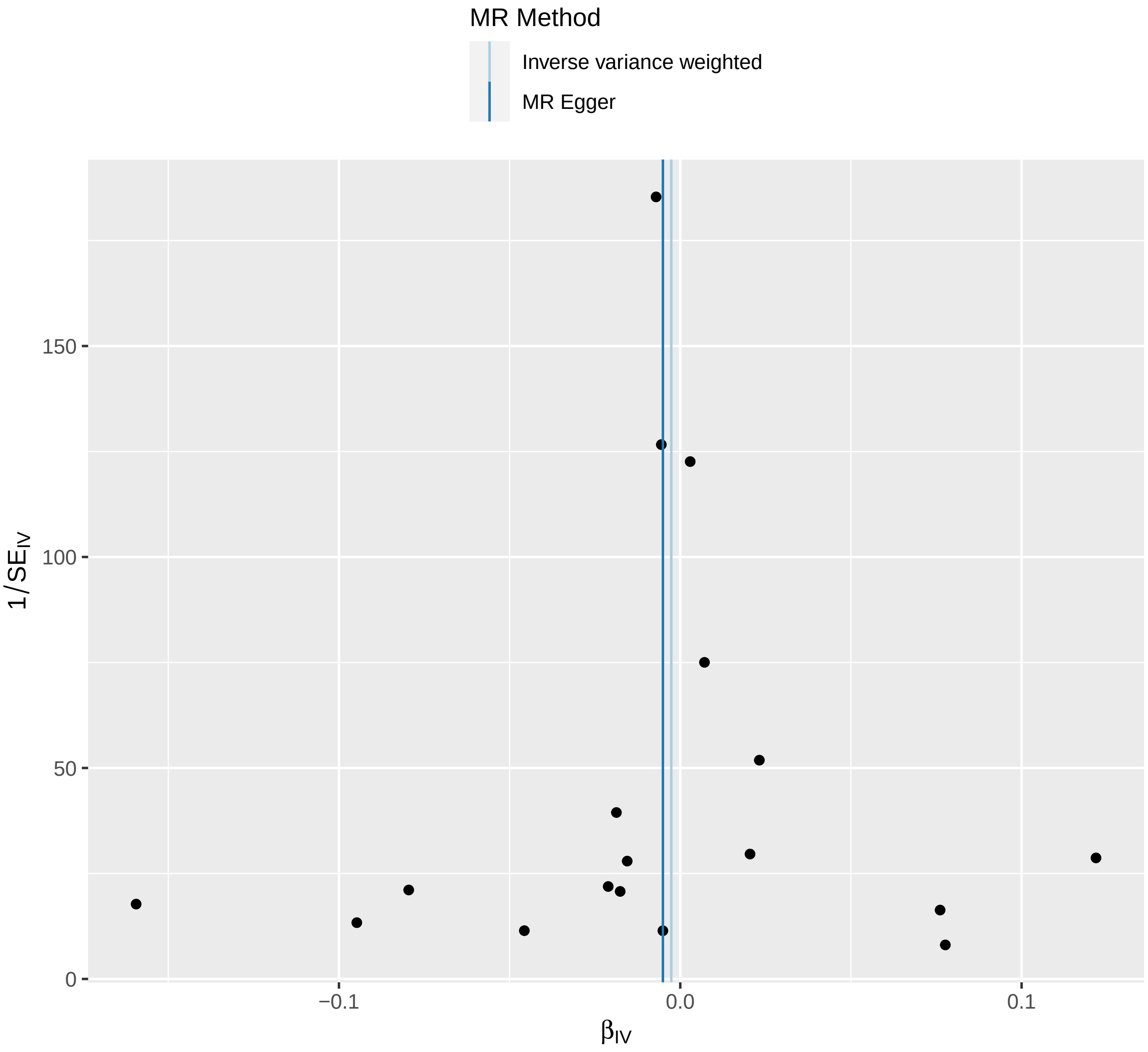
MR Method
Inverse variance weighted
MR Egger
150
100
50
0
−0.1
0.0
0.1
1/SE_IV
β_IV

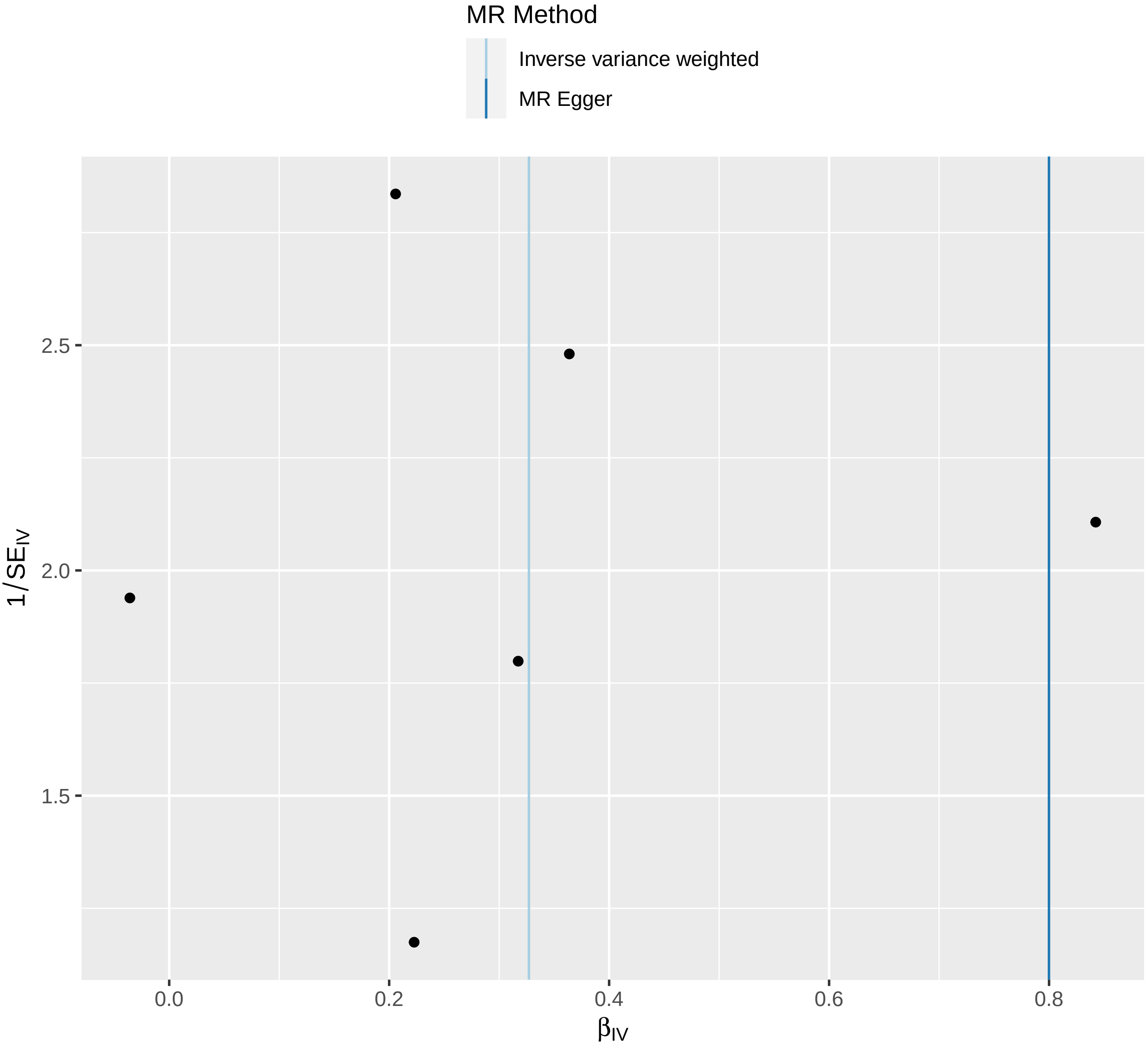
MR Method
Inverse variance weighted
MR Egger
2.5
2.0
1.5
1/SE_IV
0.0
0.2
0.4
0.6
0.8
β_IV

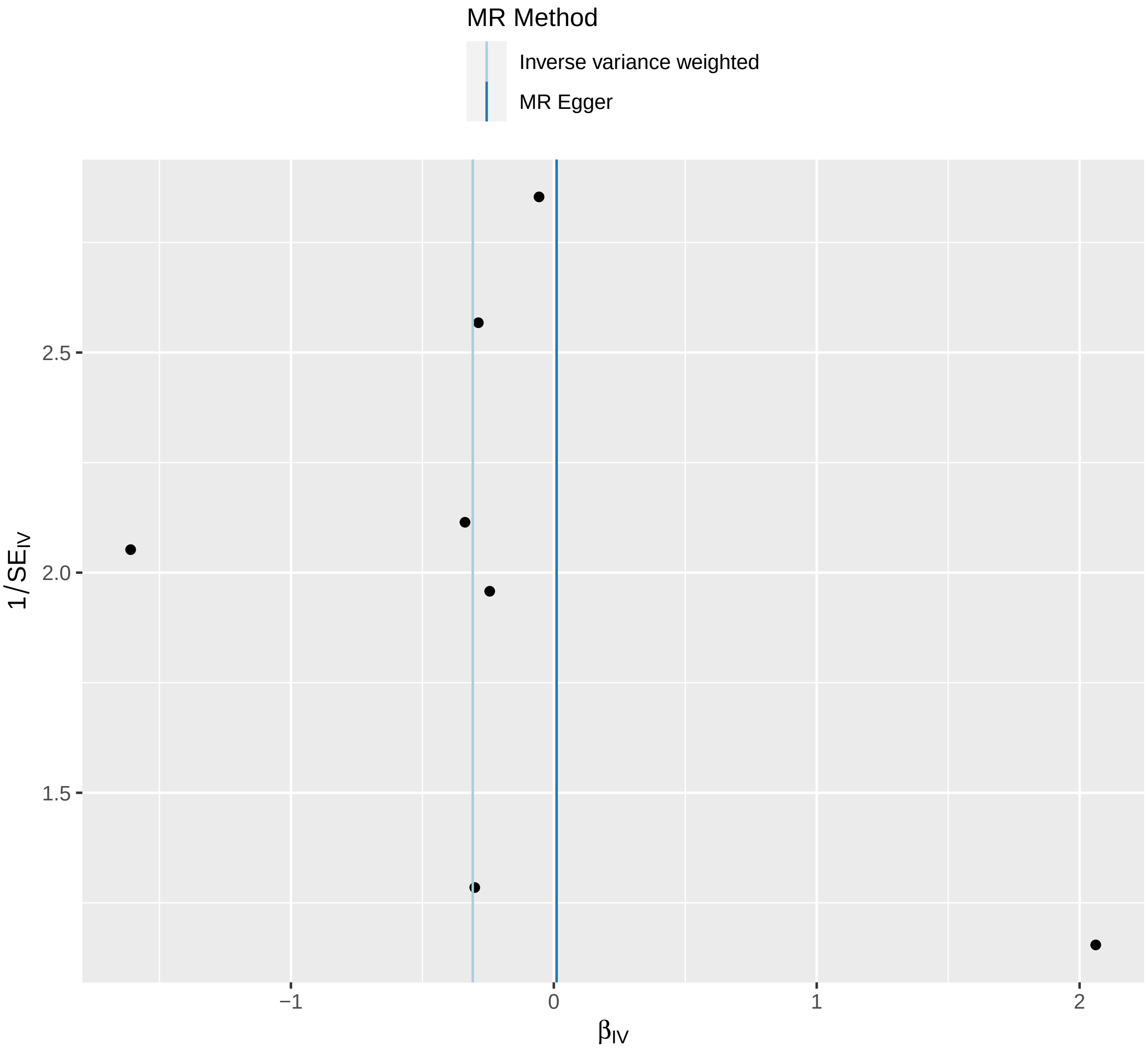

MR Method
Inverse variance weighted
MR Egger
2.5
2.0
1.5
1/SE$_{IV}$
−1
0
1
2
β$_{IV}$

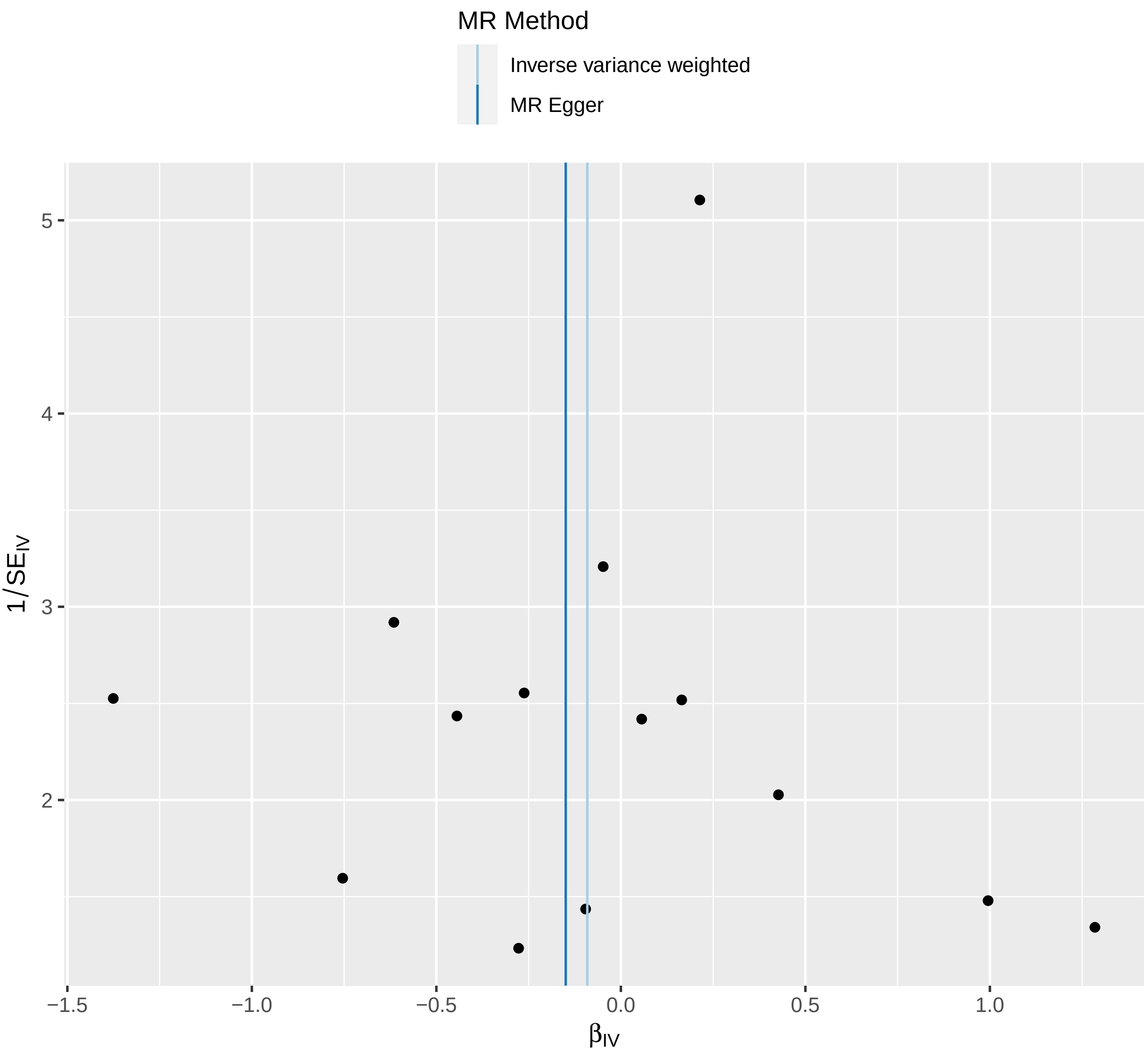

MR Method
Inverse variance weighted
MR Egger
5
4
3
2
1/SE_IV
−1.5
−1.0
−0.5
0.0
0.5
1.0
β_IV

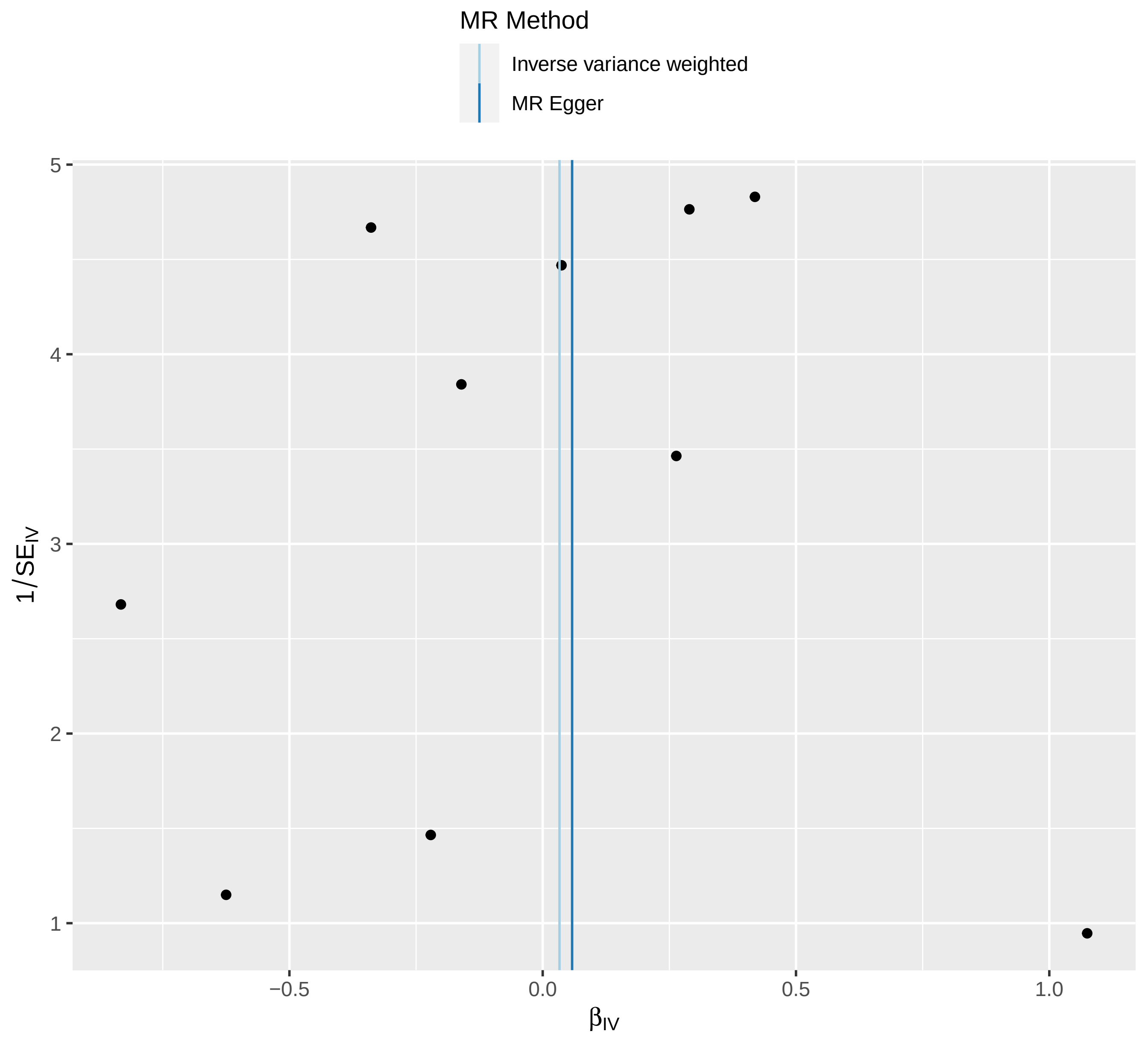
MR Method
Inverse variance weighted
MR Egger
1/SE_IV
β_IV
5
4
3
2
1
−0.5
0.0
0.5
1.0

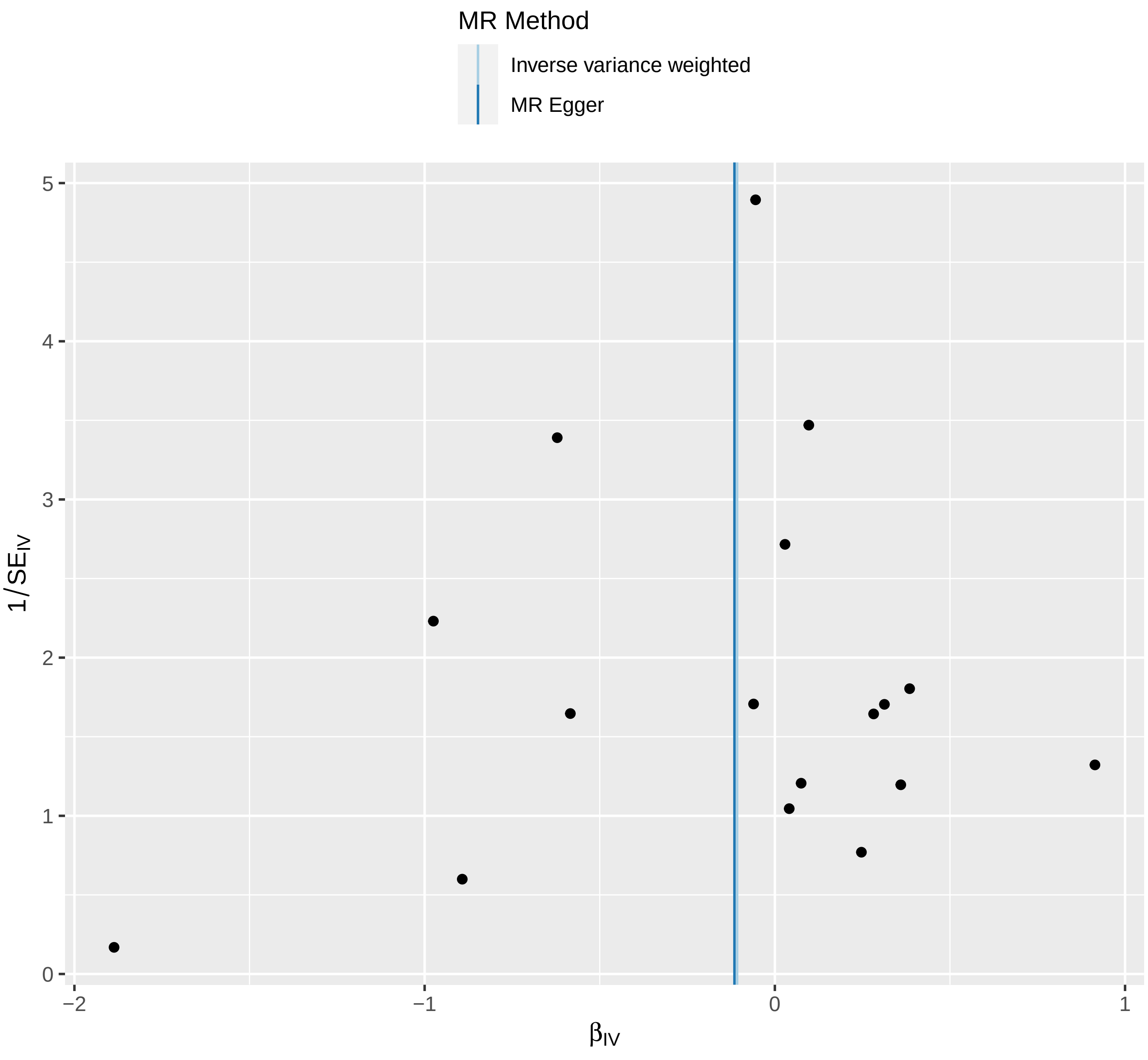
MR Method
Inverse variance weighted
MR Egger
5
4
3
2
1
0
−2
−1
0
1
1/SE_IV
β_IV

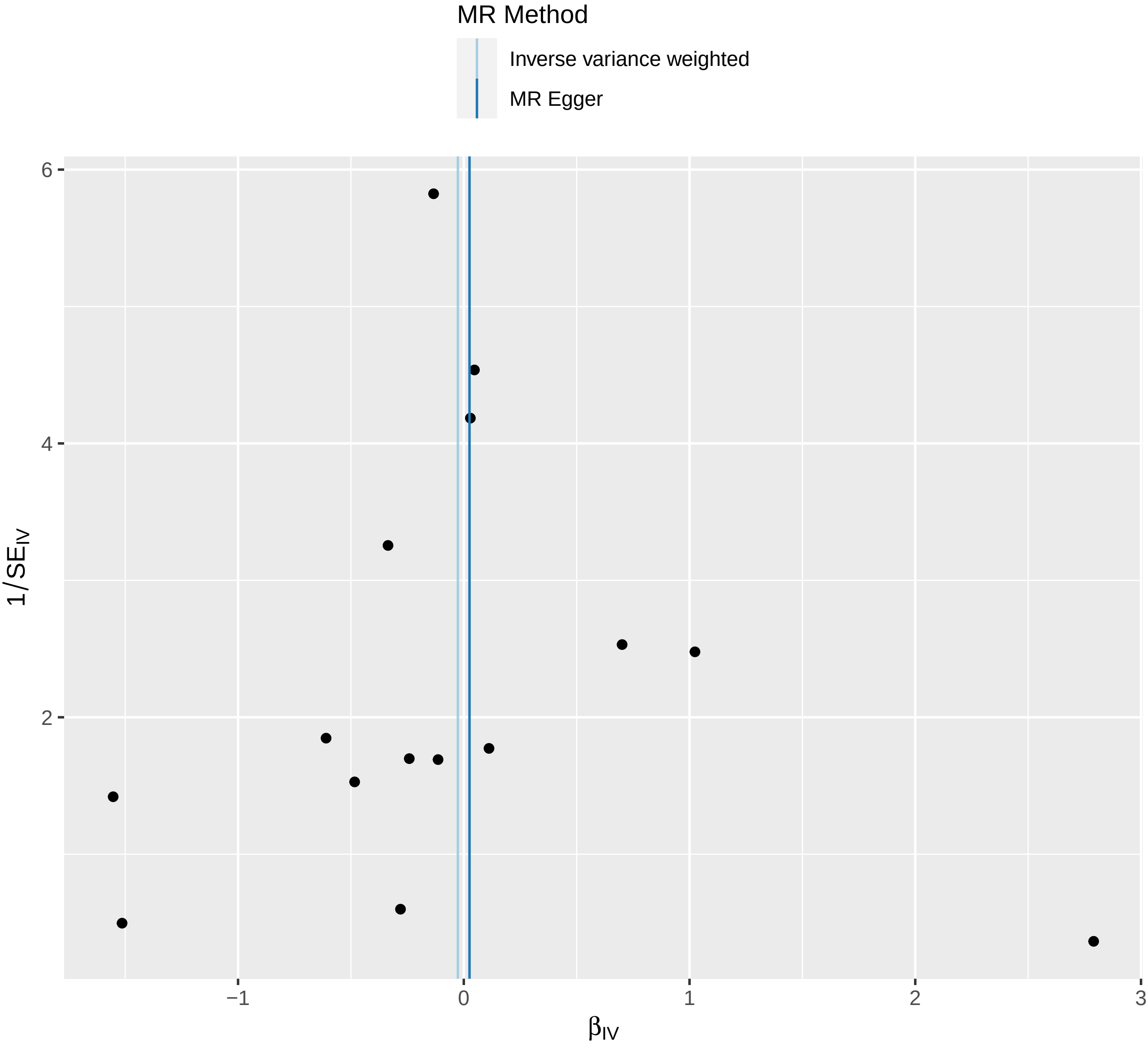

MR Method
Inverse variance weighted
MR Egger
6
4
2
1/SE$_{IV}$
−1
0
1
2
3
β$_{IV}$

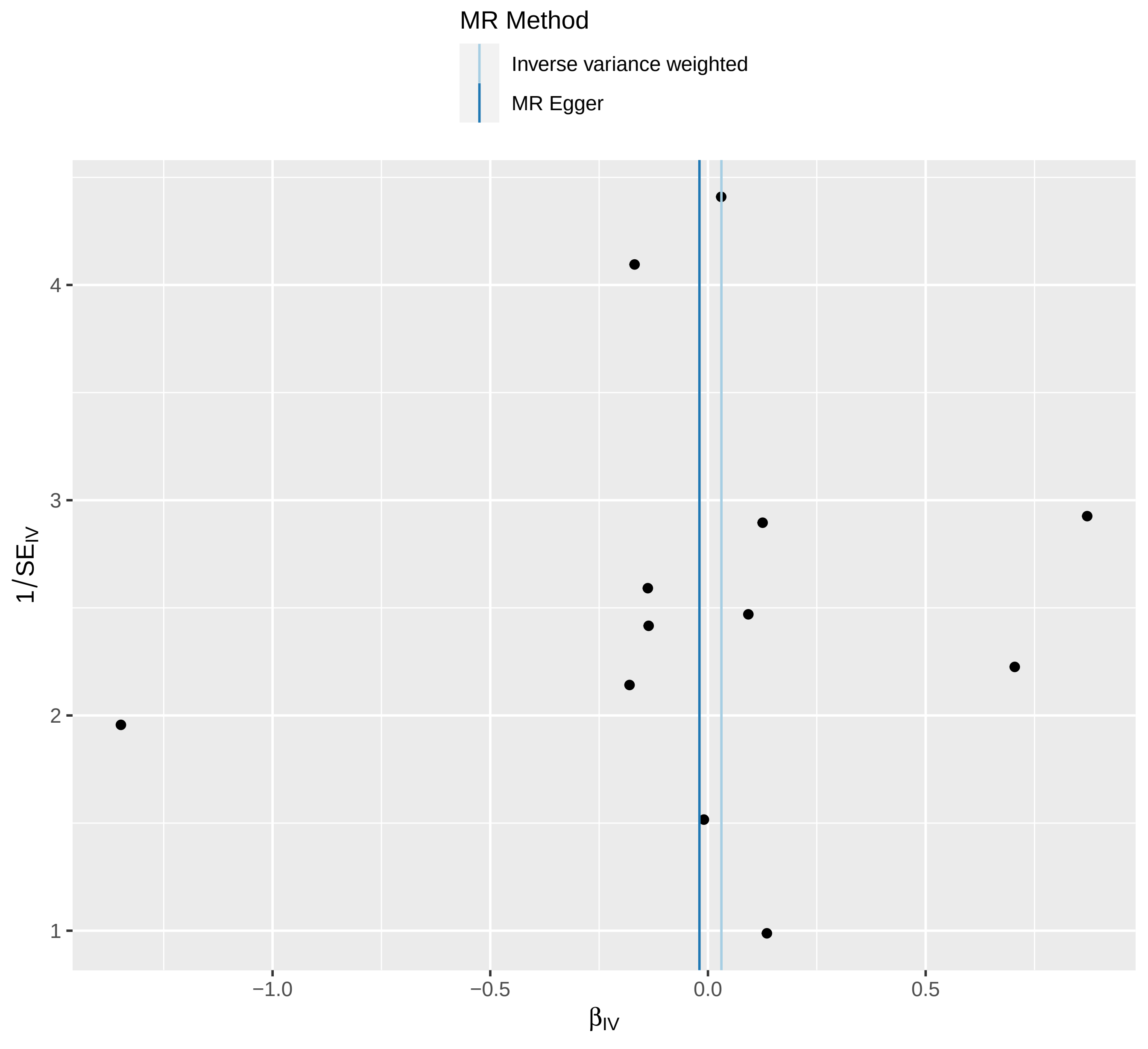

MR Method
Inverse variance weighted
MR Egger
4
3
2
1
1/SE_IV
−1.0
−0.5
0.0
0.5
β_IV

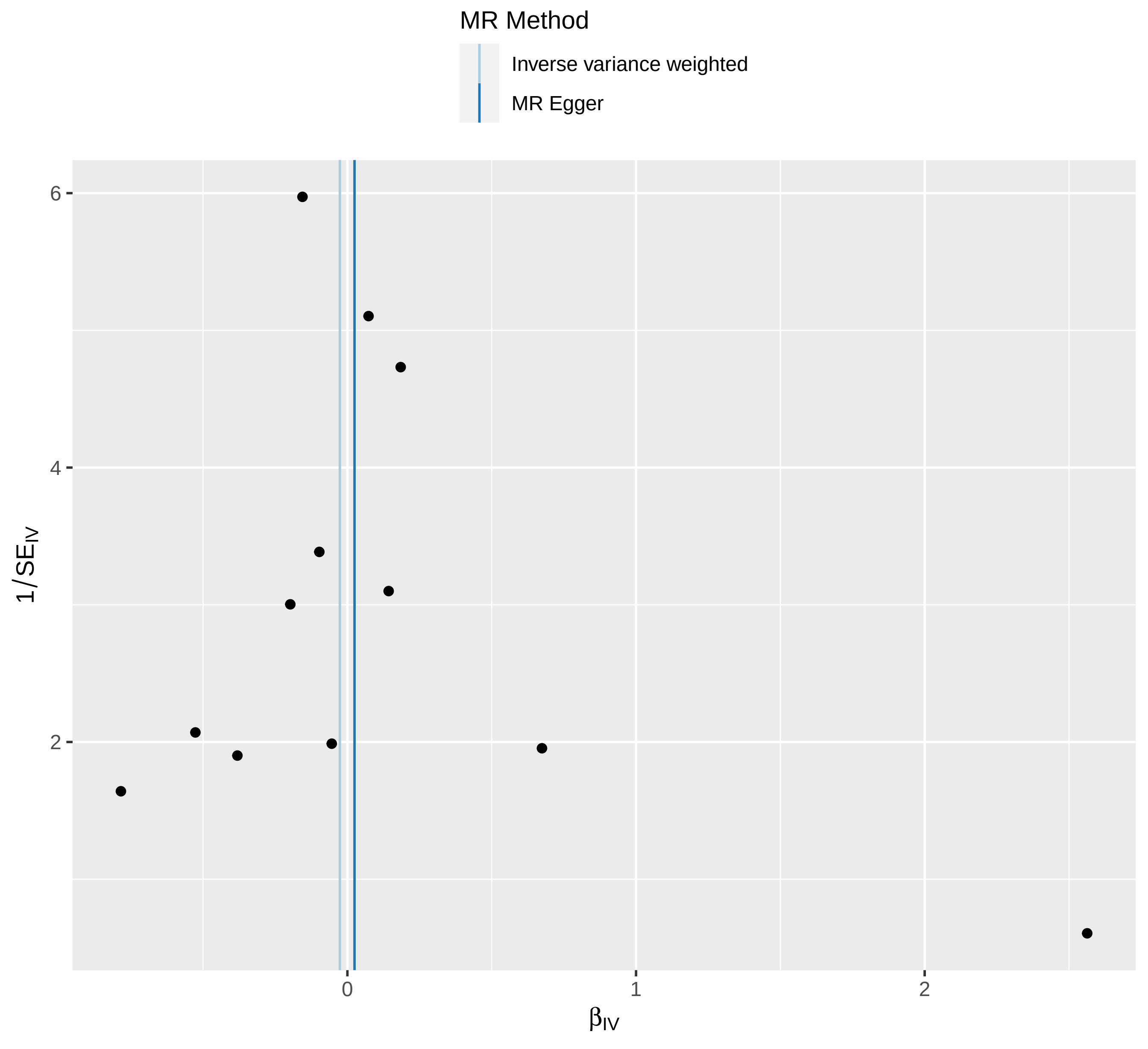

MR Method
Inverse variance weighted
MR Egger
6
4
2
0
1
2
$1/SE_{IV}$
β_{IV}

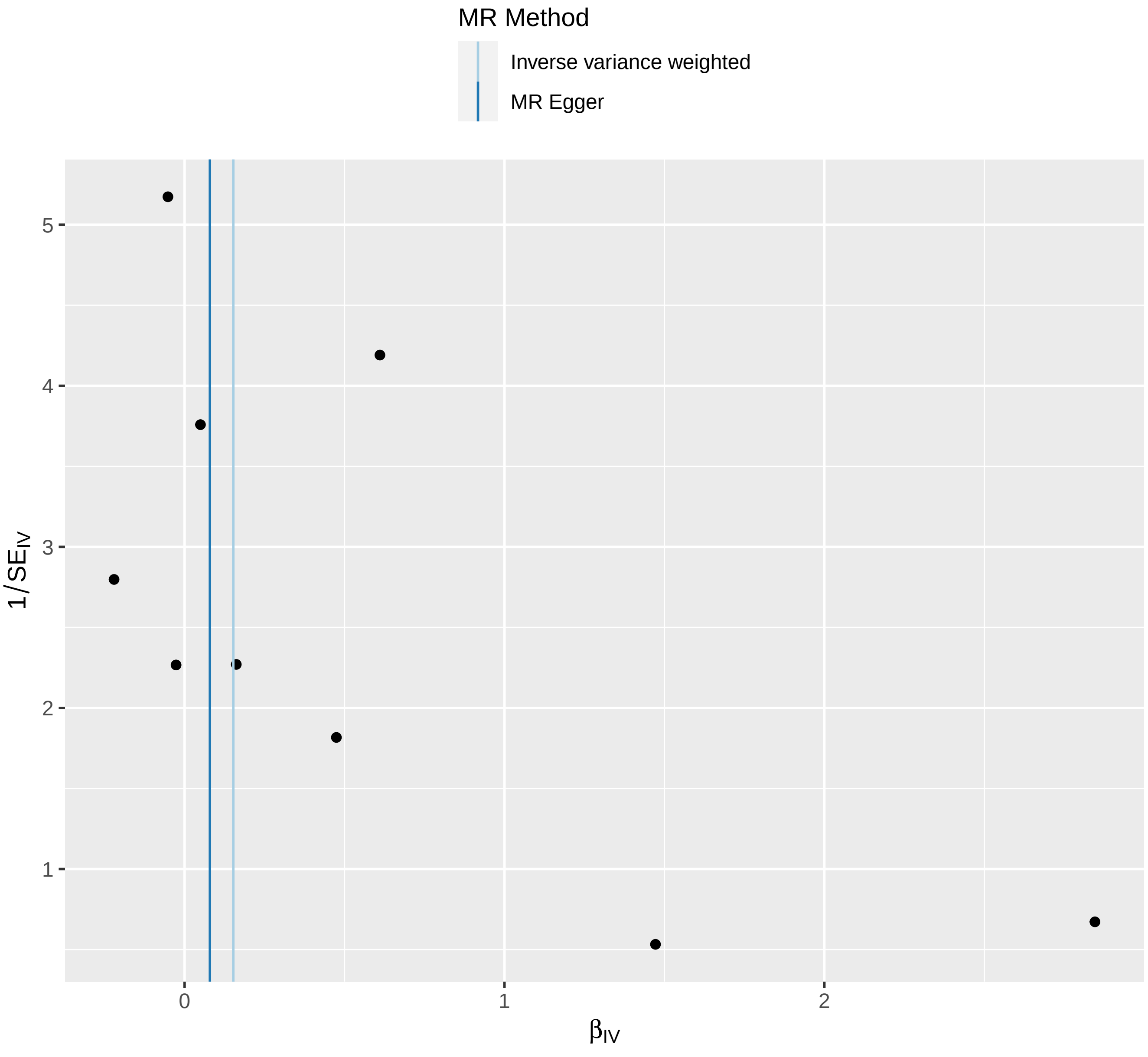
MR Method
Inverse variance weighted
MR Egger
1/SE_IV
β_IV
5
4
3
2
1
0
1
2

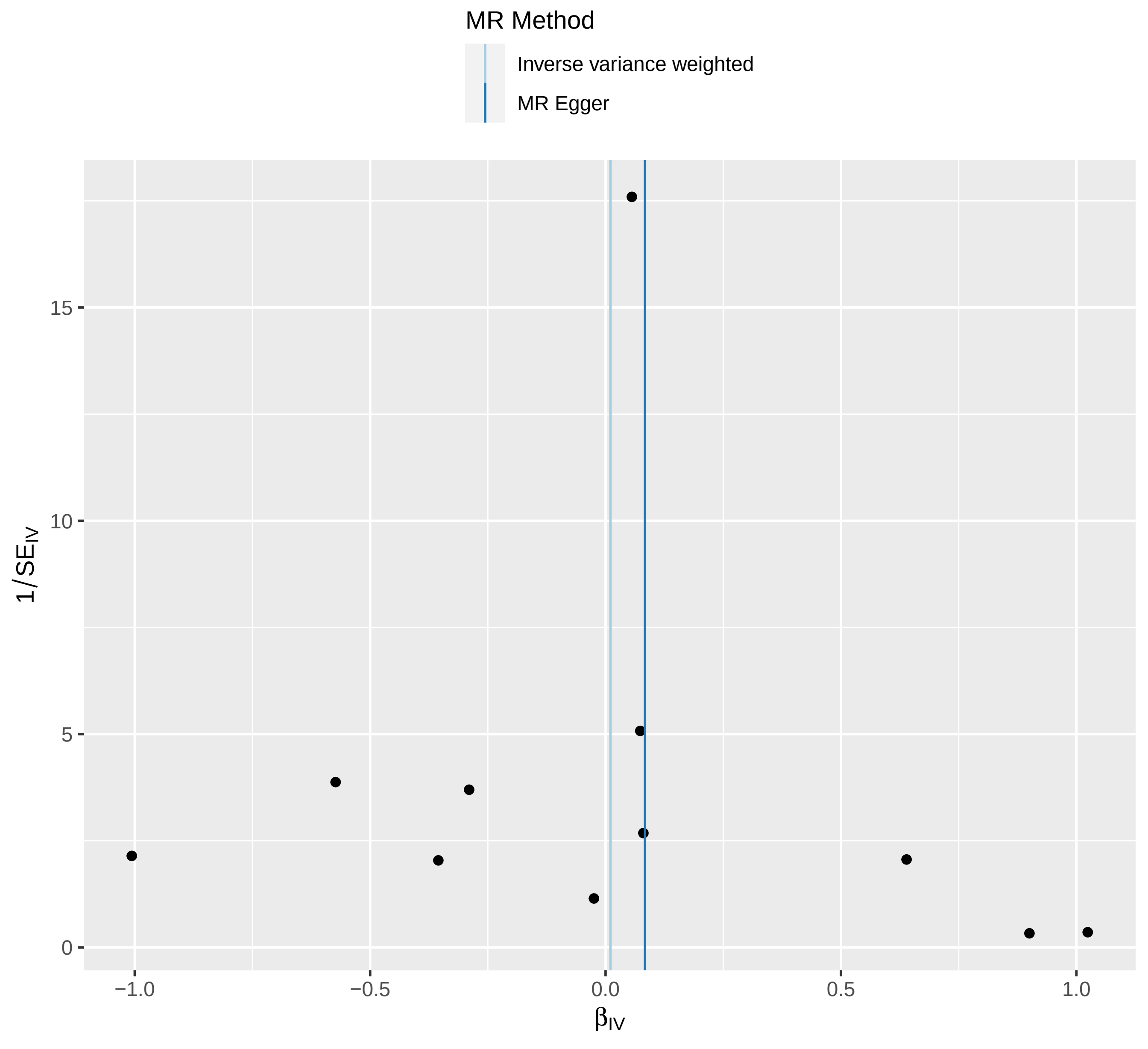

MR Method
Inverse variance weighted
MR Egger
15
10
5
0
1/SE$_{IV}$
−1.0
−0.5
0.0
0.5
1.0
β$_{IV}$

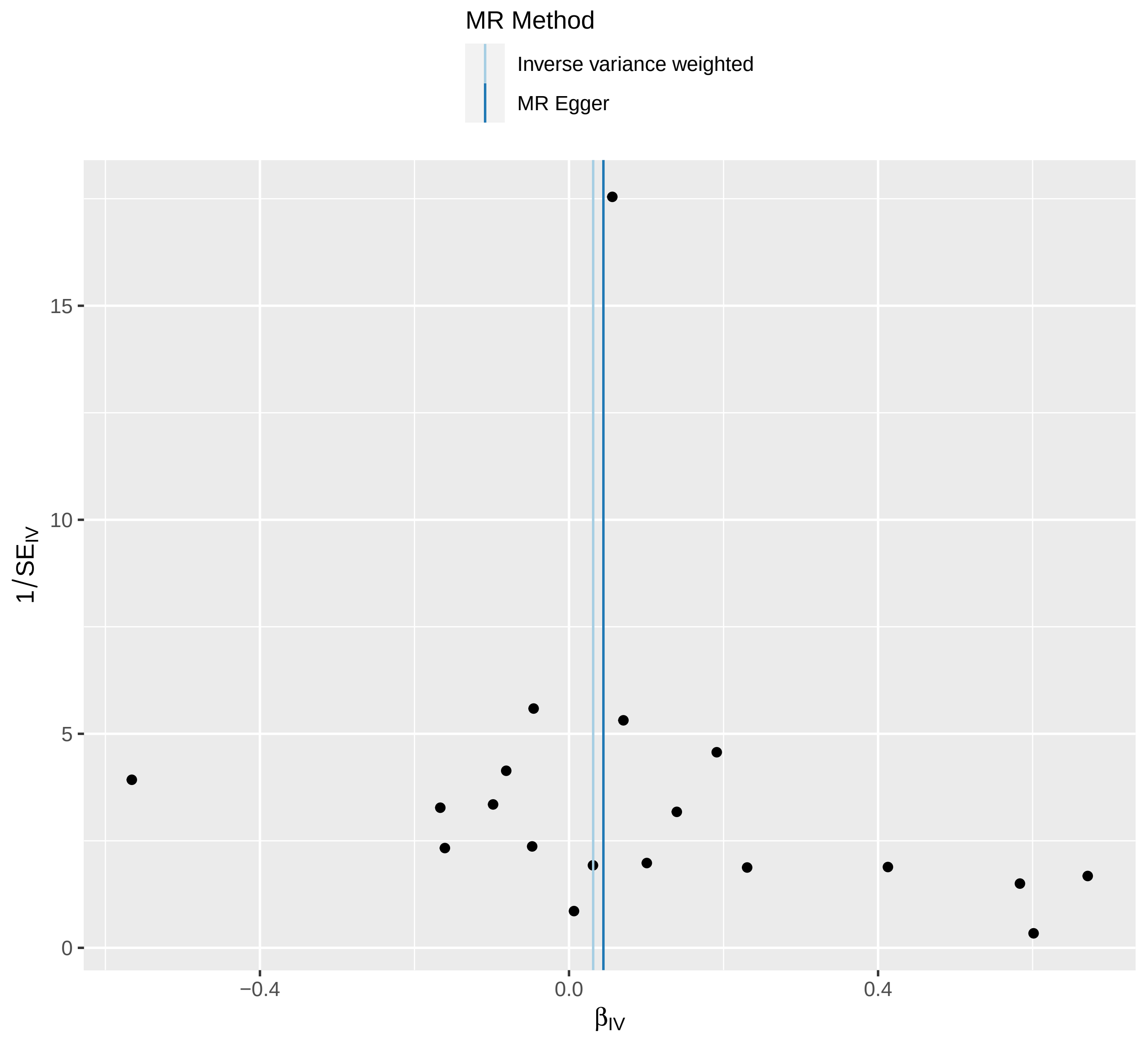

MR Method
Inverse variance weighted
MR Egger
15
10
5
0
1/SE_IV
−0.4
0.0
0.4
β_IV

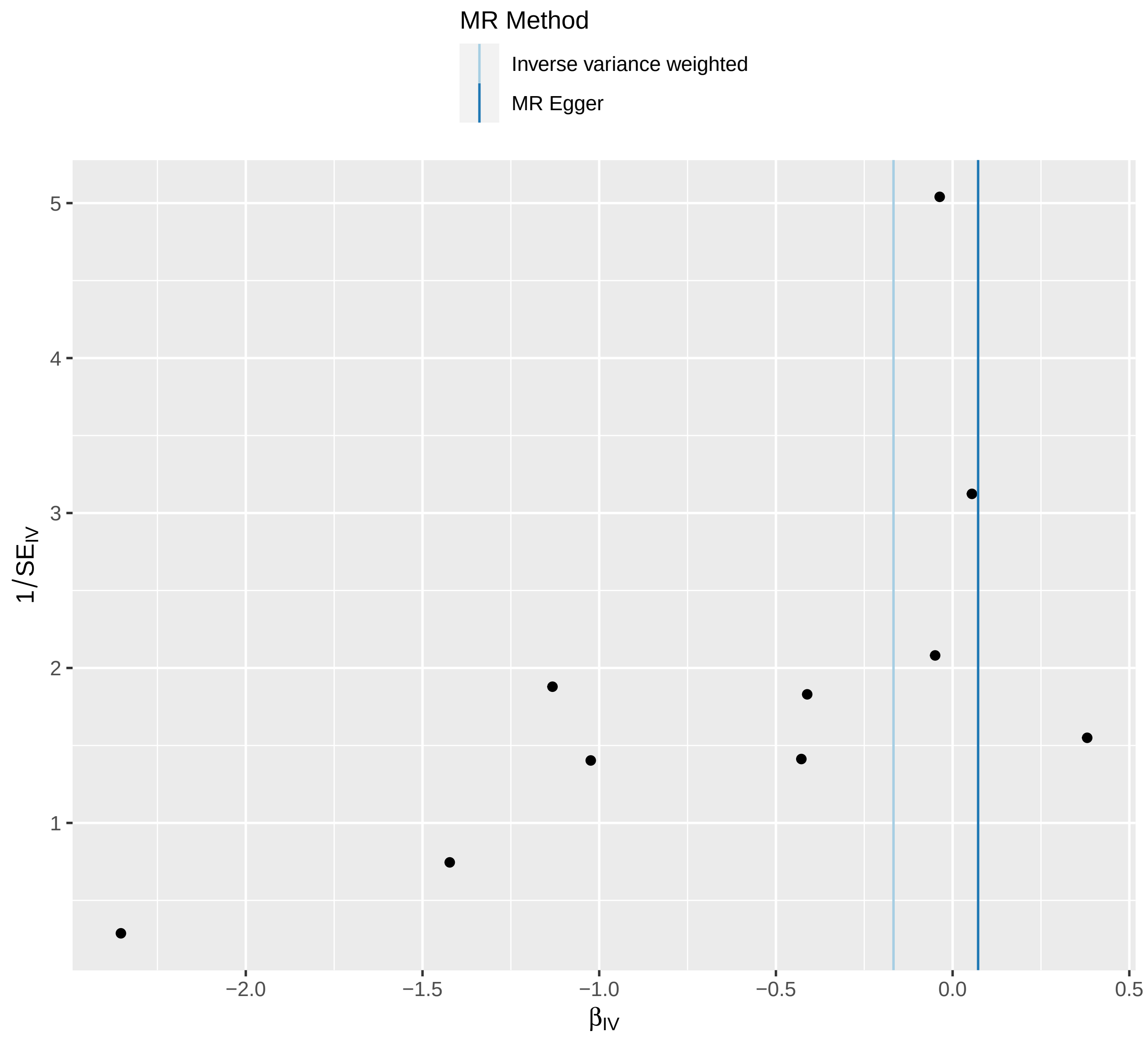
MR Method
Inverse variance weighted
MR Egger
1/SE$_{IV}$
5
4
3
2
1
−2.0
−1.5
−1.0
−0.5
0.0
0.5
β$_{IV}$

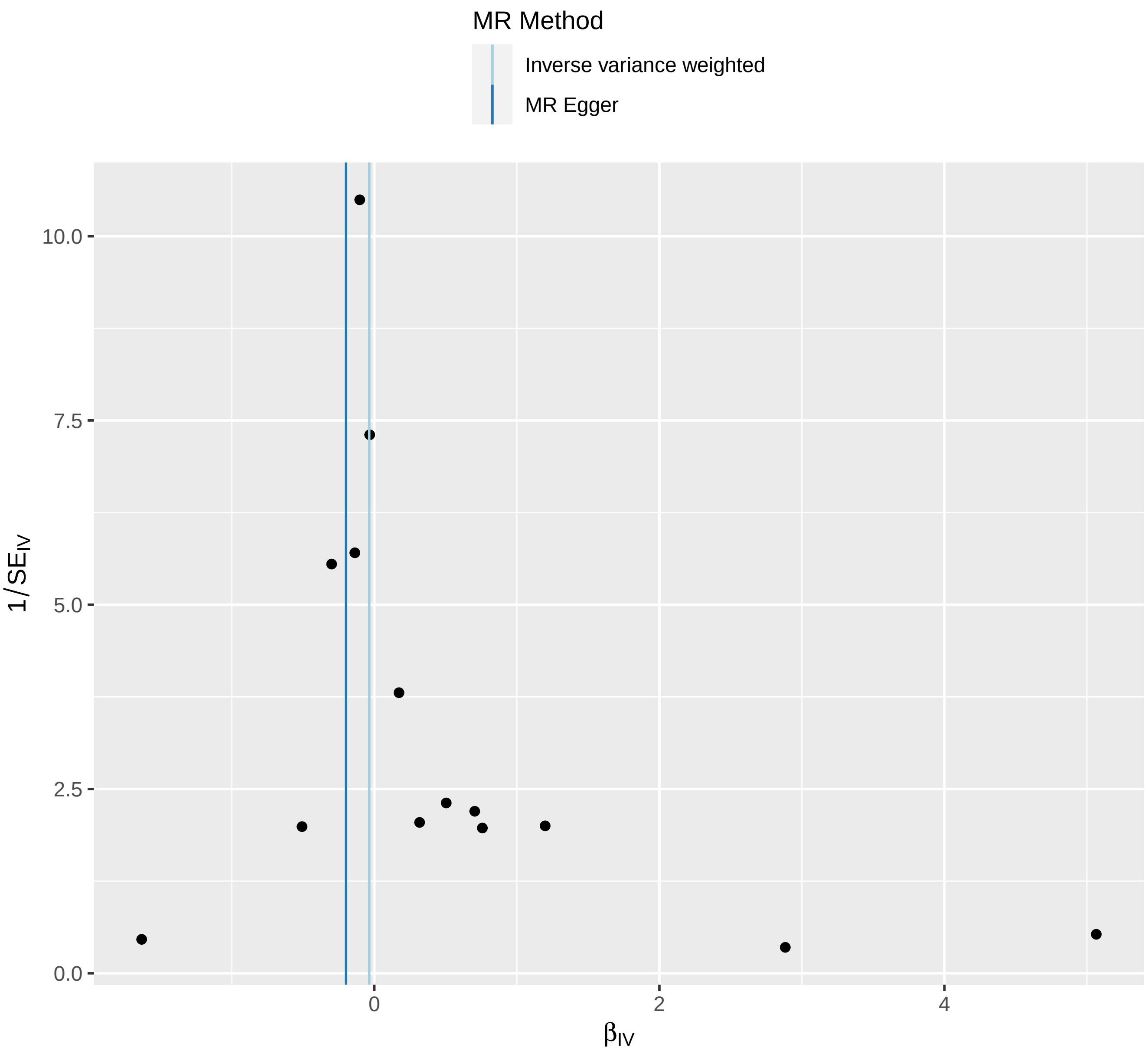

MR Method
Inverse variance weighted
MR Egger
10.0
7.5
5.0
2.5
0.0
1/SE_IV
0
2
4
β_IV

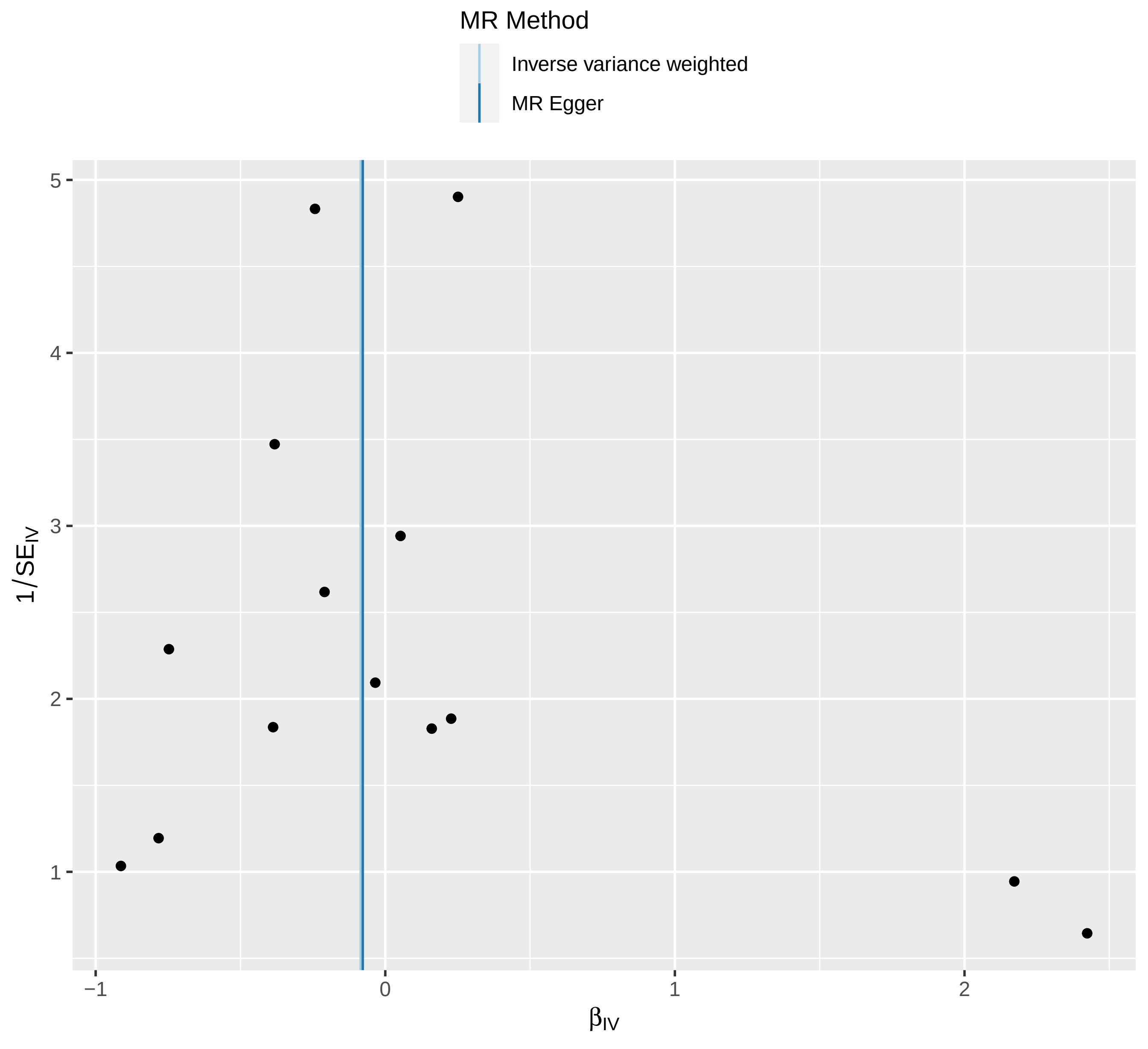
MR Method
Inverse variance weighted
MR Egger
1/SE$_{IV}$
β$_{IV}$
5
4
3
2
1
−1
0
1
2

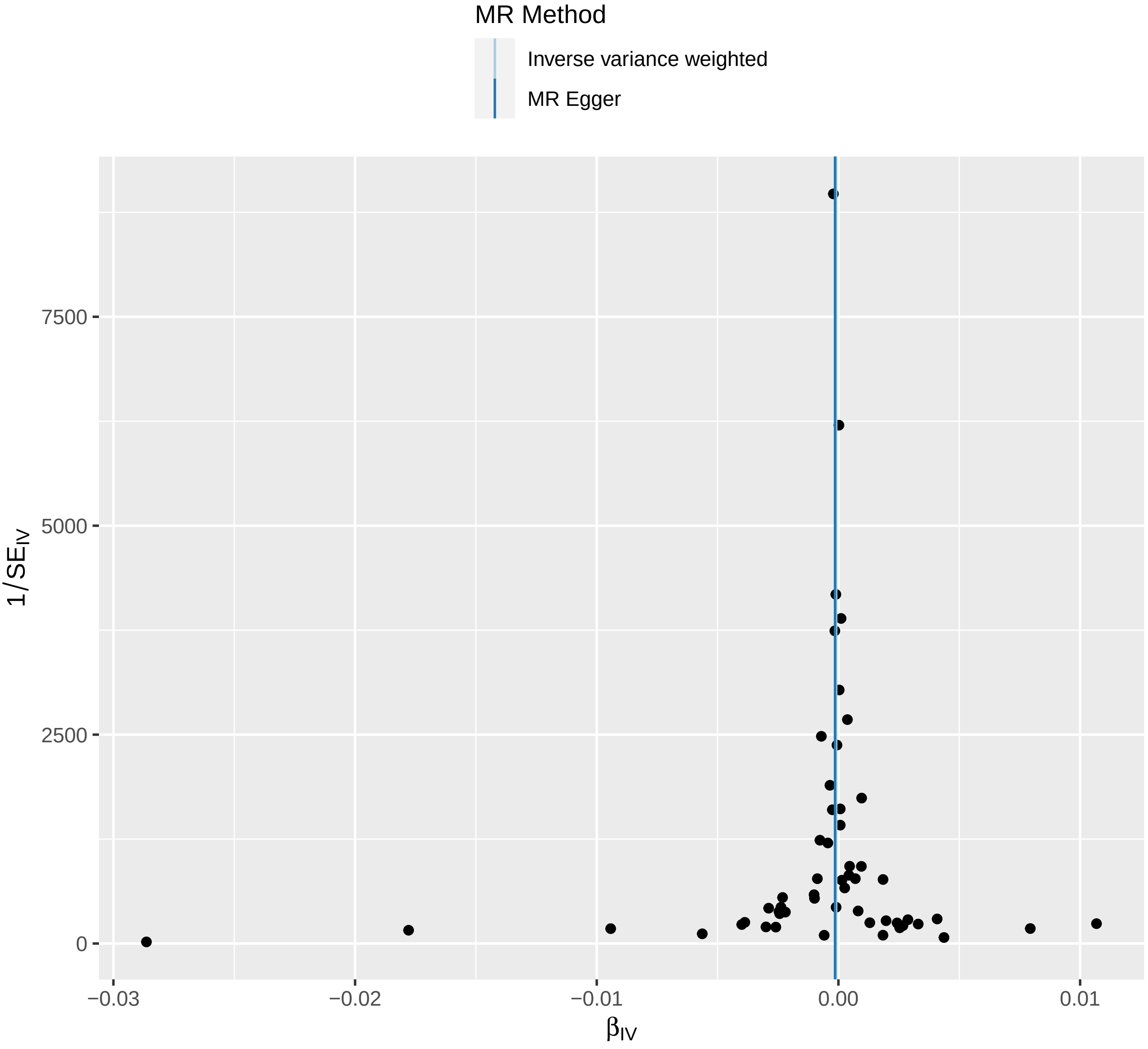

MR Method
Inverse variance weighted
MR Egger
7500
5000
2500
0
$1/SE_{IV}$
−0.03
−0.02
−0.01
0.00
0.01
β_{IV}

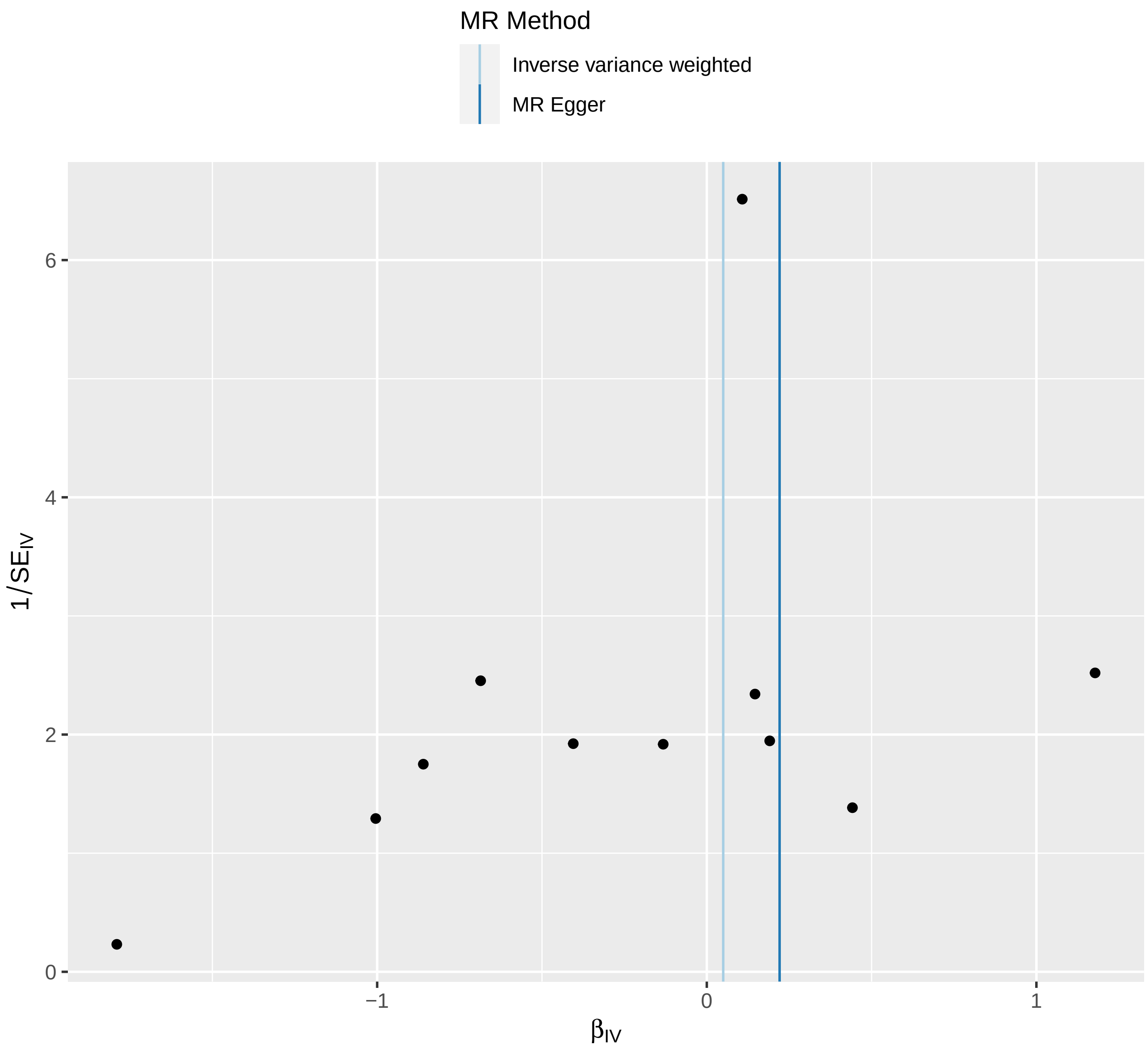

MR Method
Inverse variance weighted
MR Egger
6
4
2
0
−1
0
1
$1/SE_{IV}$
β_{IV}

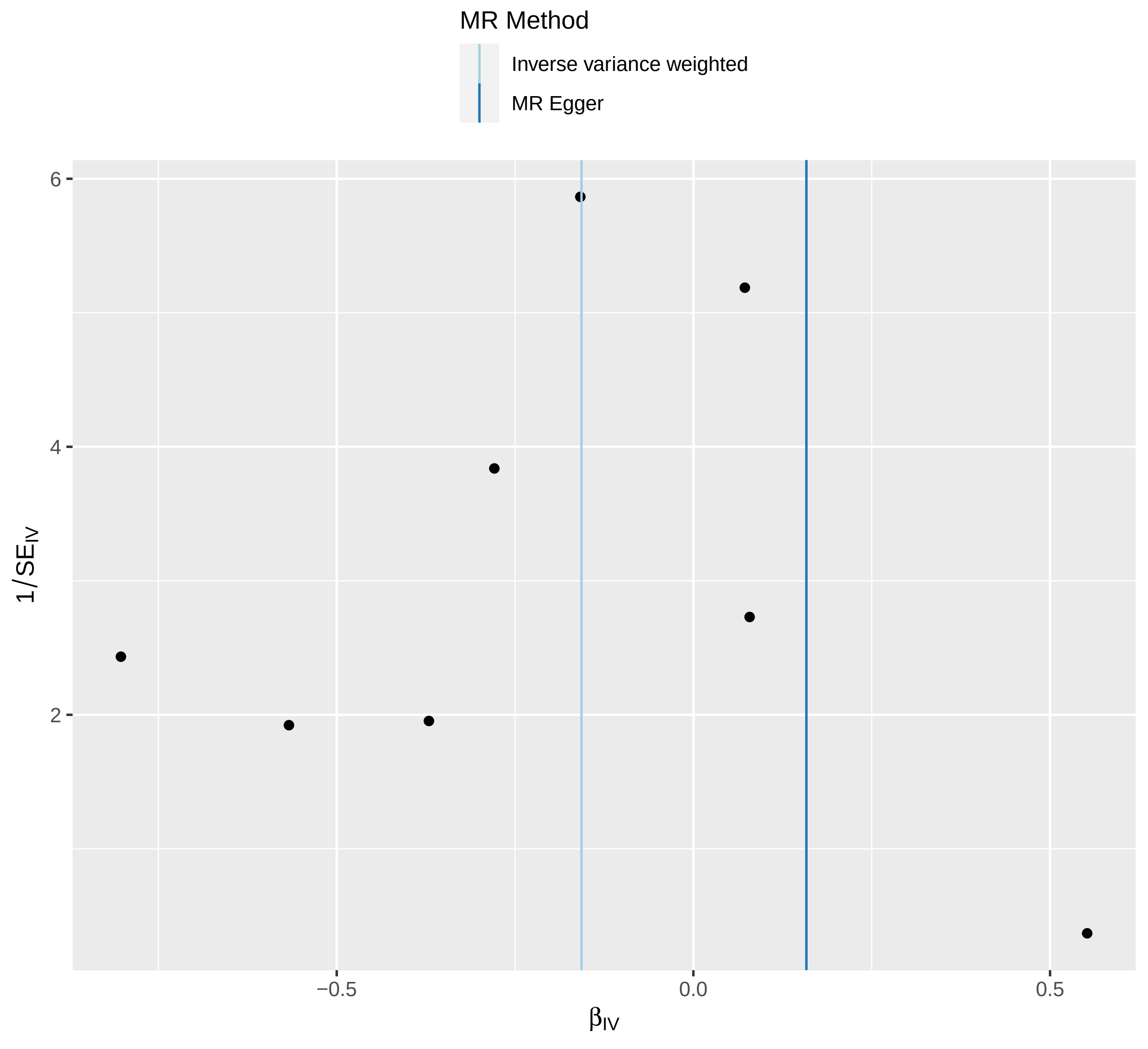
MR Method
Inverse variance weighted
MR Egger
6
4
2
1/SE_IV
−0.5
0.0
0.5
β_IV

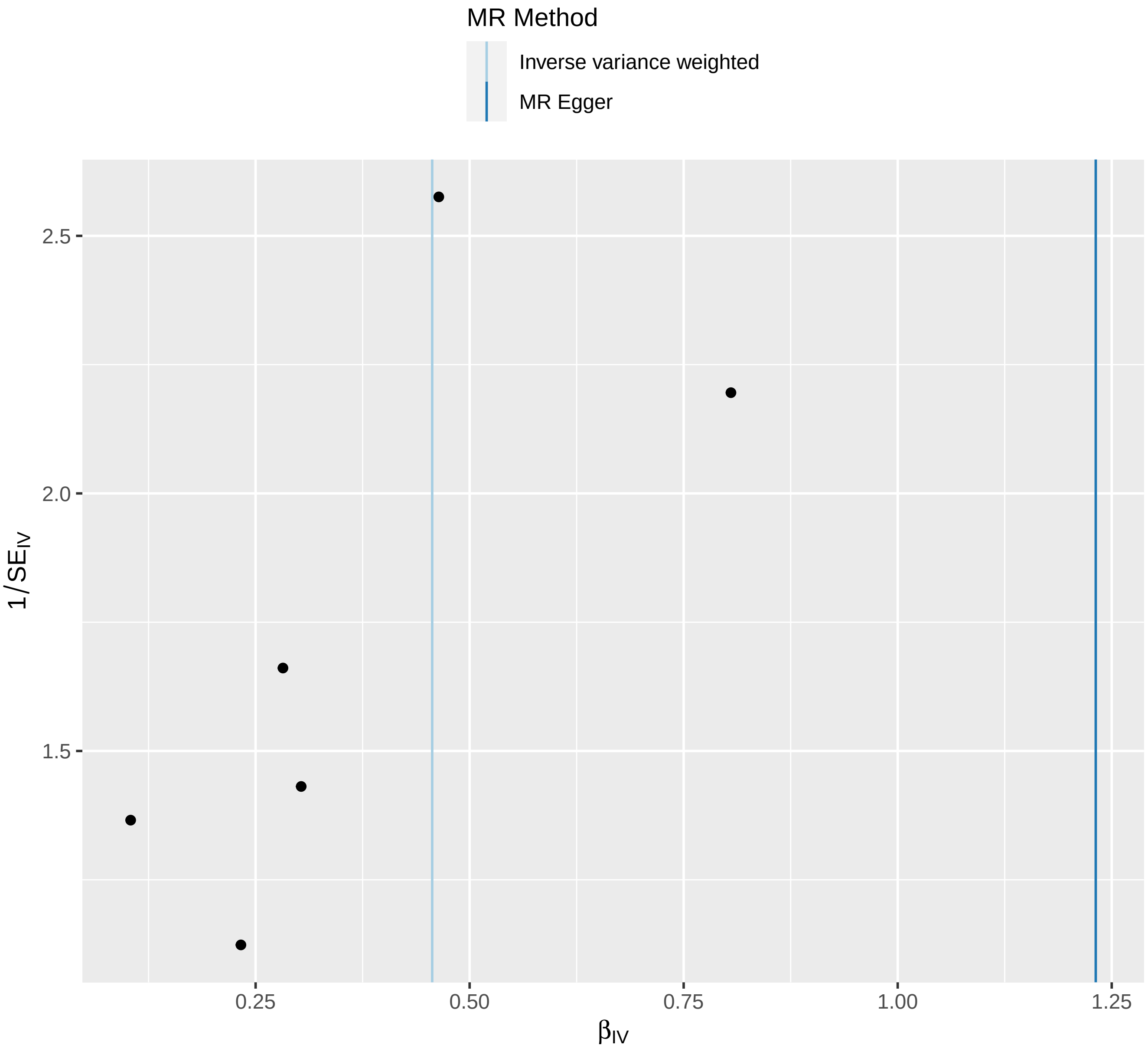

MR Method
Inverse variance weighted
MR Egger
2.5
2.0
1.5
1/SE_IV
0.25
0.50
0.75
1.00
1.25
β_IV

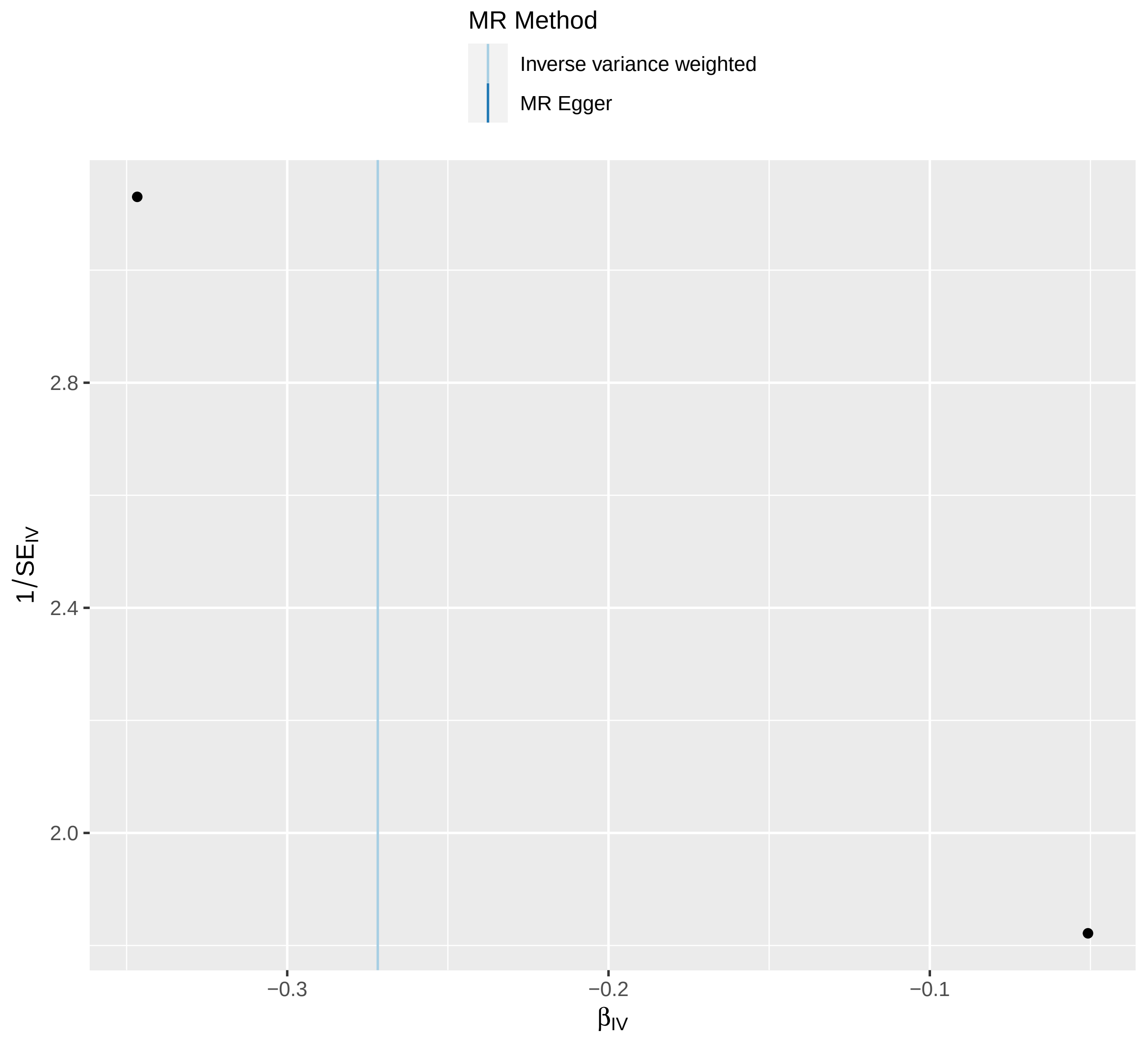
MR Method
Inverse variance weighted
MR Egger
2.8
2.4
2.0
1/SE$_{IV}$
−0.3
−0.2
−0.1
β$_{IV}$

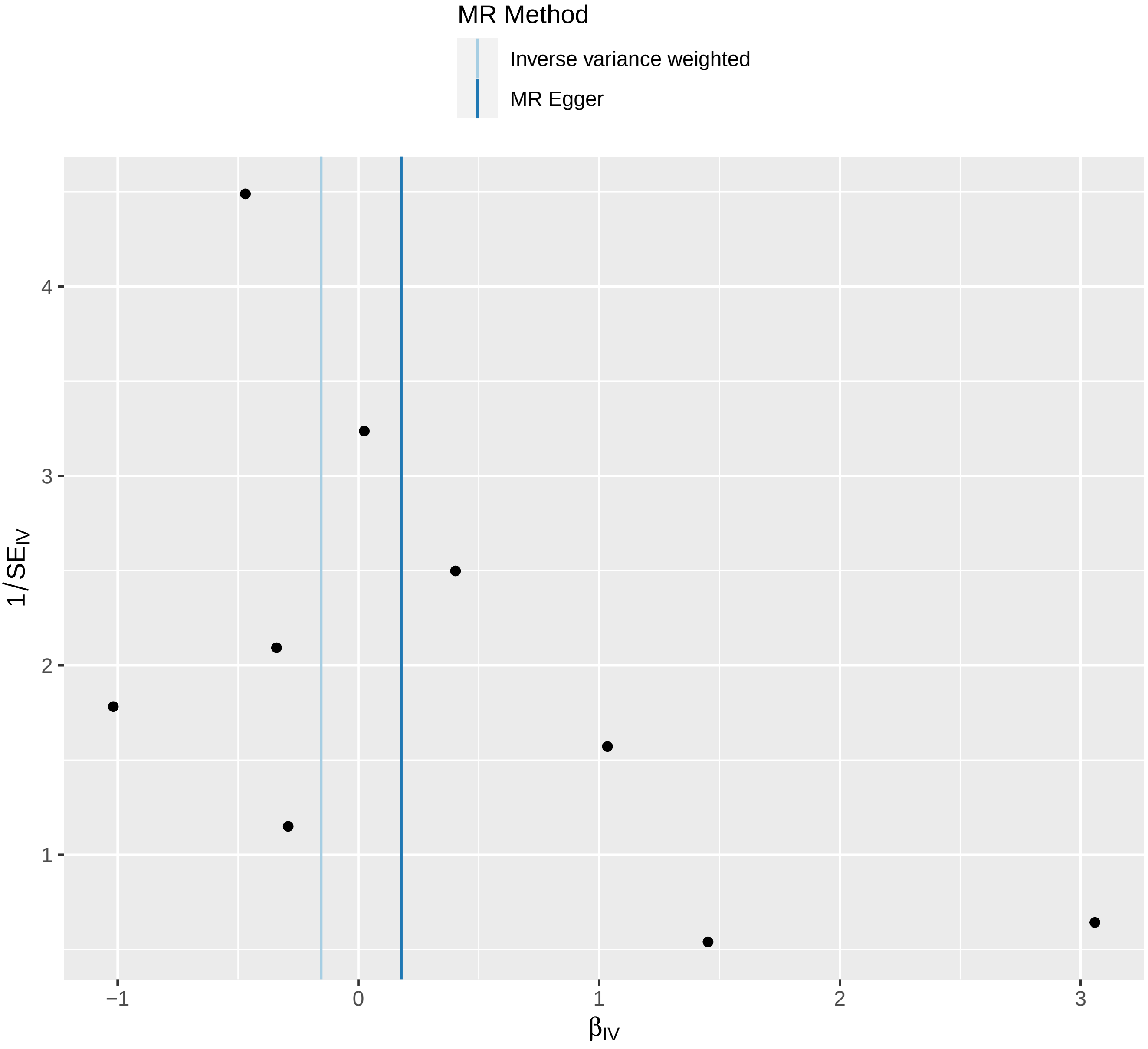
MR Method
Inverse variance weighted
MR Egger
1/SE$_{IV}$
4
3
2
1
−1
0
1
2
3
β$_{IV}$

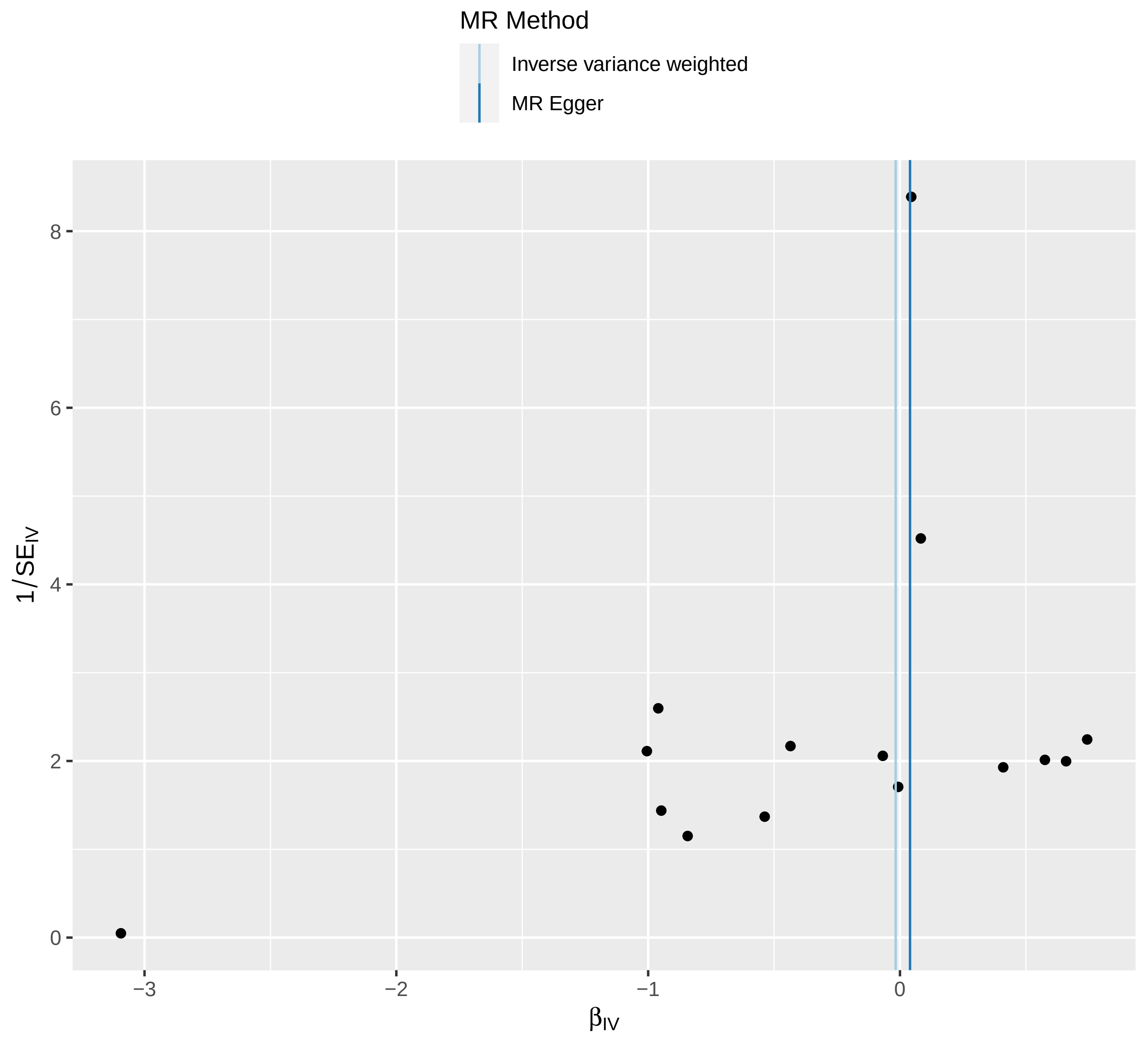

MR Method
Inverse variance weighted
MR Egger
8
6
4
2
0
−3
−2
−1
0
1/SE_IV
β_IV

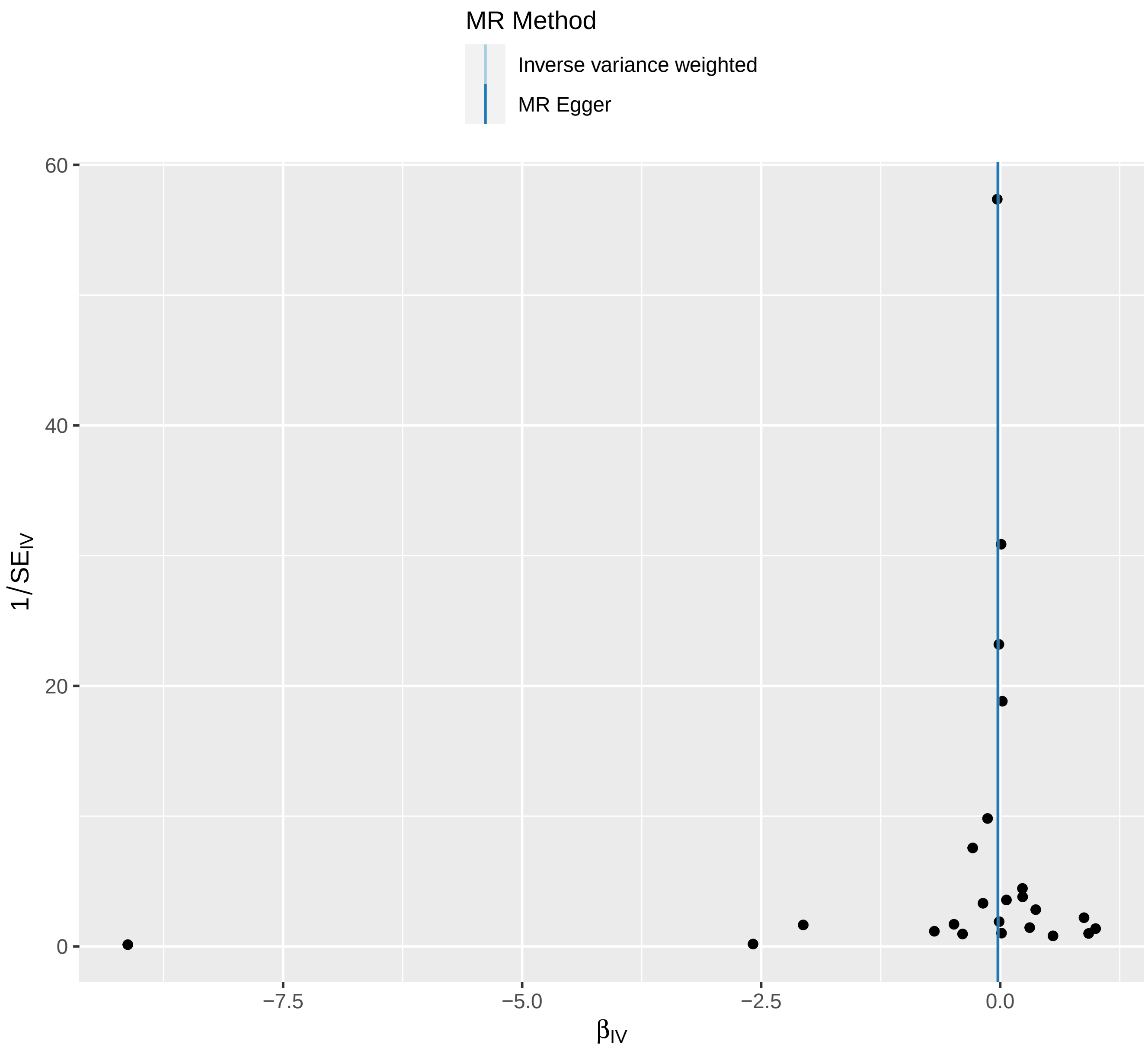

MR Method
Inverse variance weighted
MR Egger
60
40
20
0
1/SE$_{IV}$
−7.5
−5.0
−2.5
0.0
β$_{IV}$

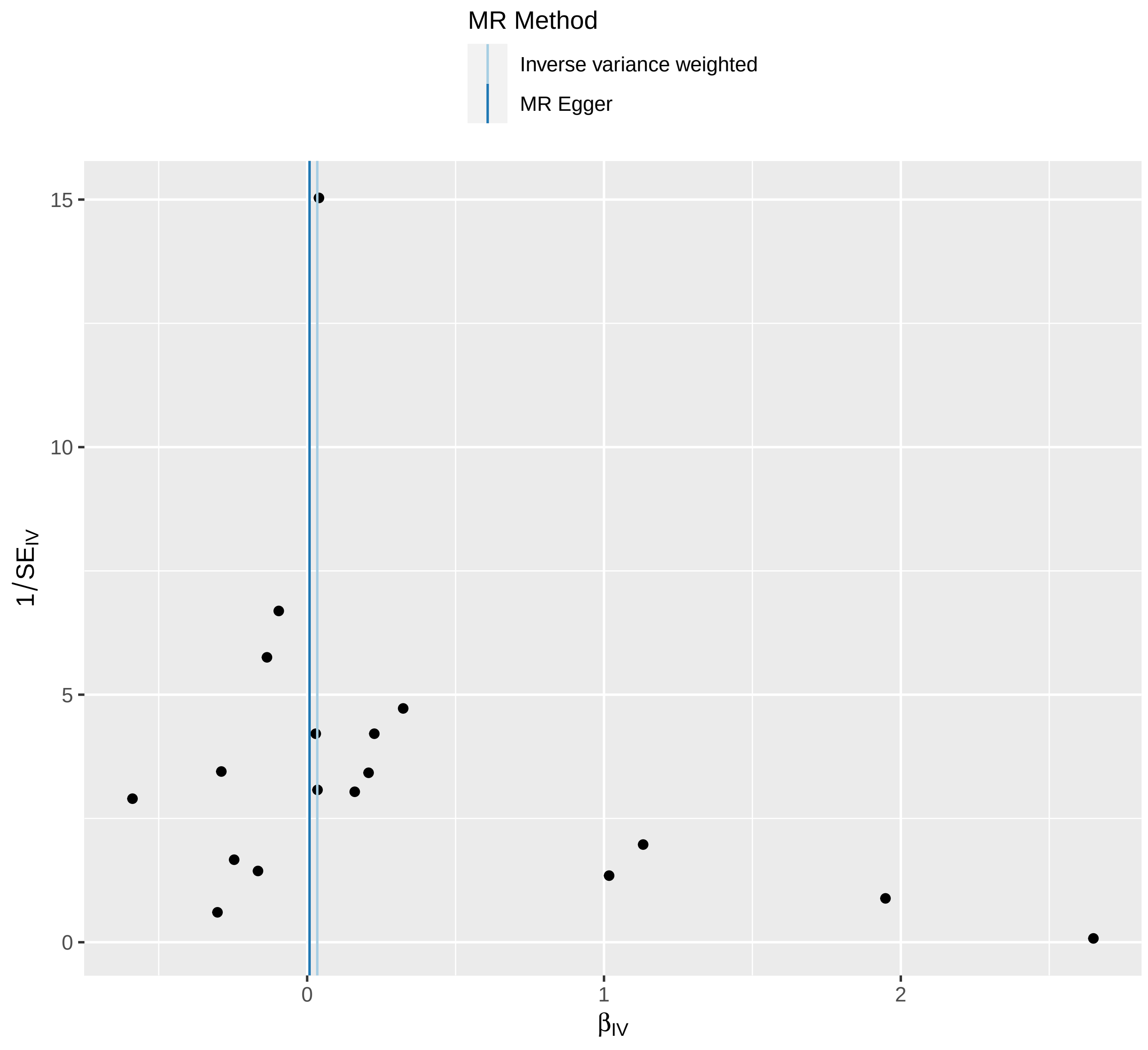

MR Method
Inverse variance weighted
MR Egger
15
10
5
0
1/SE$_{IV}$
0
1
2
β$_{IV}$

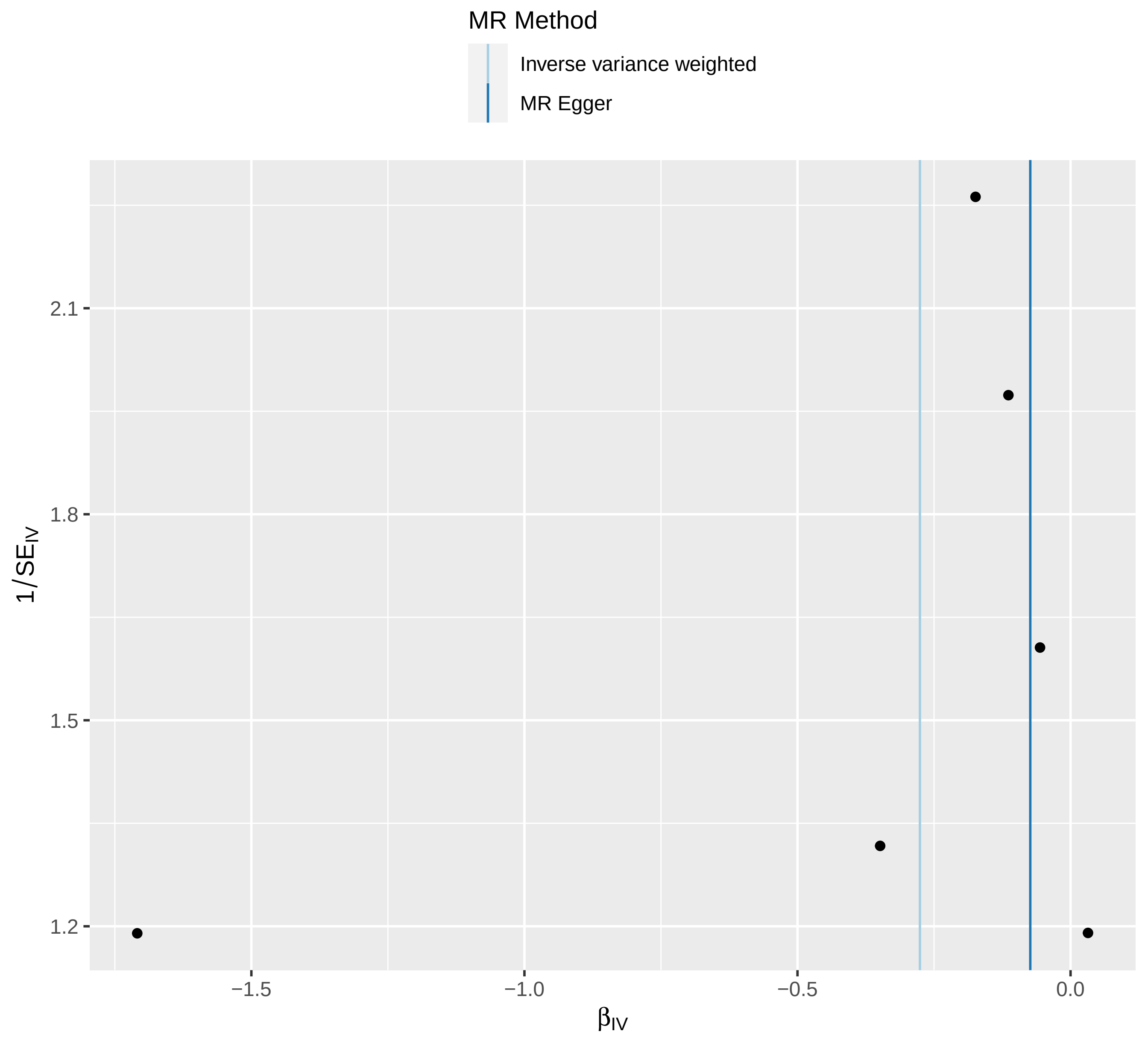

MR Method
Inverse variance weighted
MR Egger
2.1
1.8
1.5
1.2
1/SE$_{IV}$
−1.5
−1.0
−0.5
0.0
β$_{IV}$

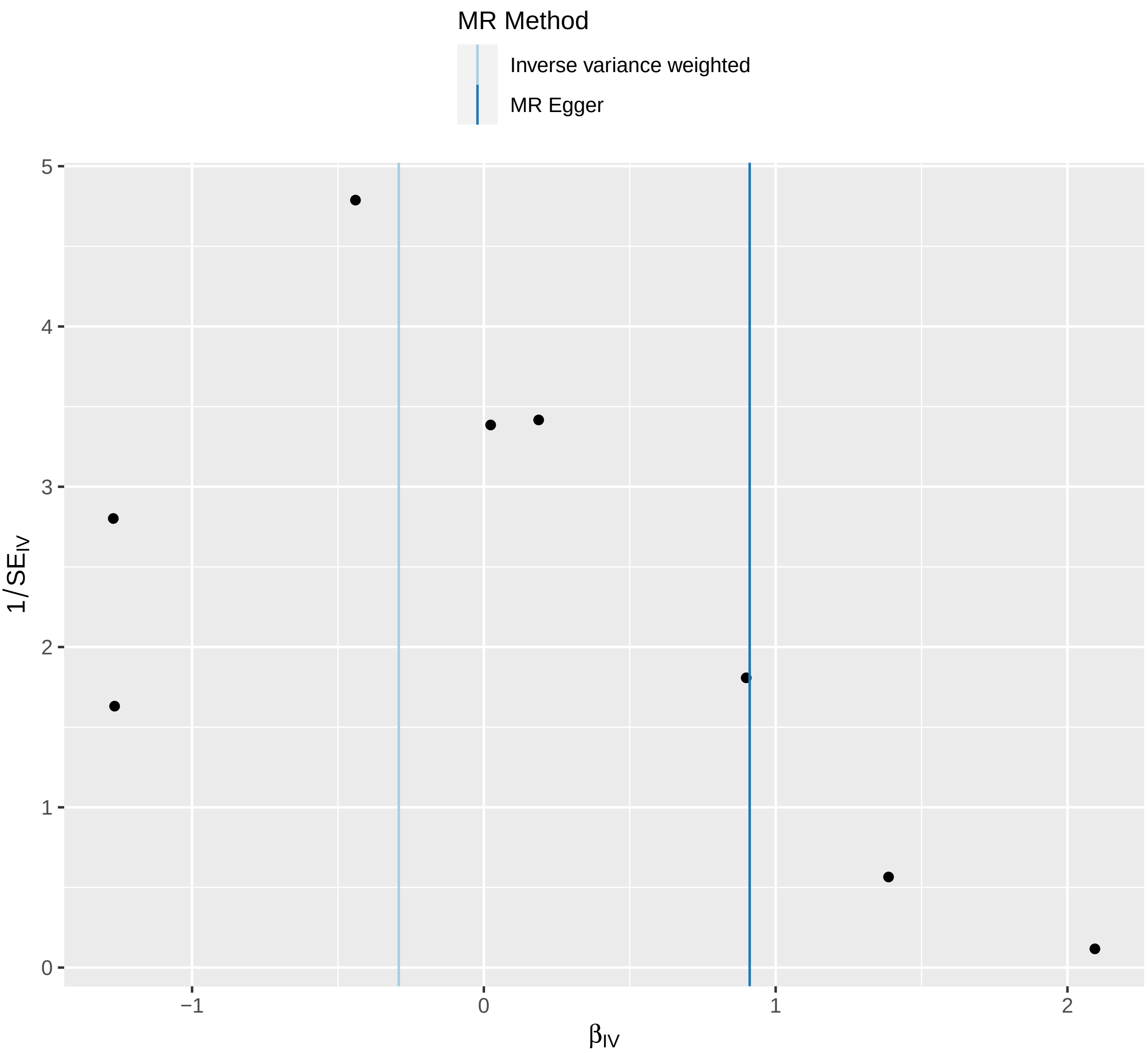
MR Method
Inverse variance weighted
MR Egger
5
4
3
2
1
0
1/SE_IV
−1
0
1
2
β_IV

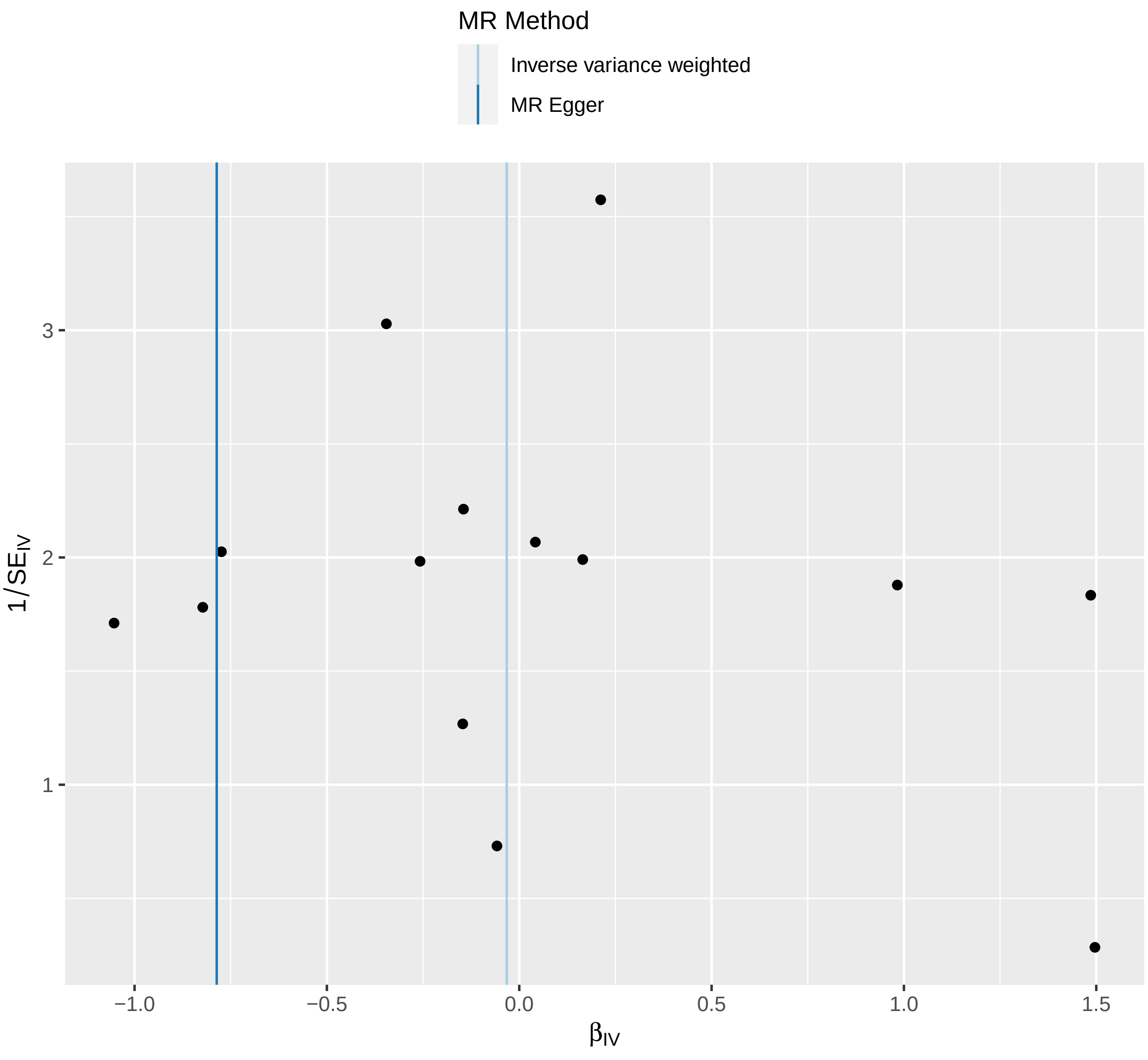

MR Method
Inverse variance weighted
MR Egger
1/SE_{IV}
β_{IV}
3
2
1
−1.0
−0.5
0.0
0.5
1.0
1.5

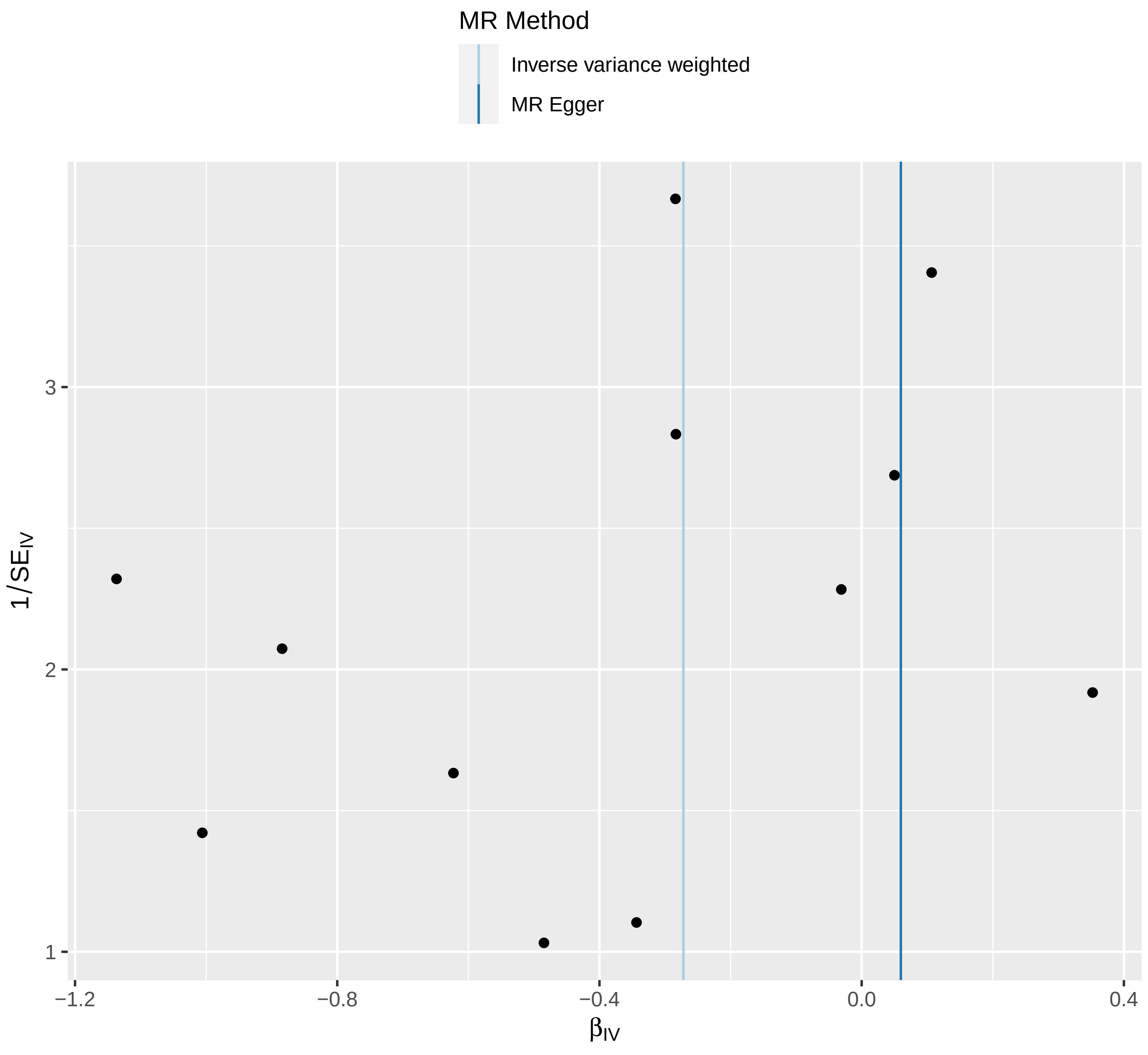
MR Method
Inverse variance weighted
MR Egger
$1/SE_{IV}$
3
2
1
−1.2
−0.8
−0.4
0.0
0.4
β_{IV}

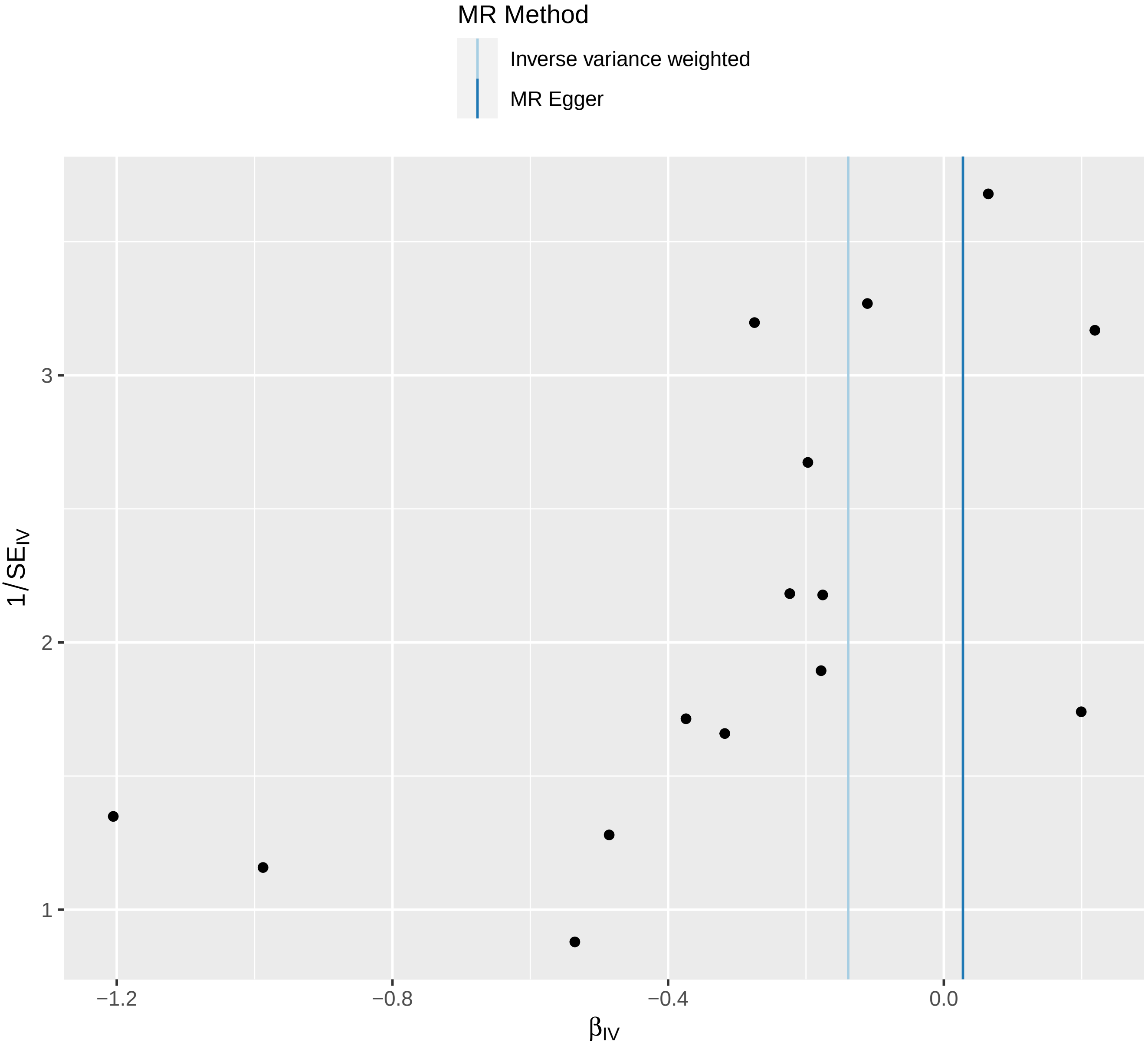
MR Method
Inverse variance weighted
MR Egger
1/SE$_{IV}$
3
2
1
−1.2
−0.8
−0.4
0.0
β$_{IV}$

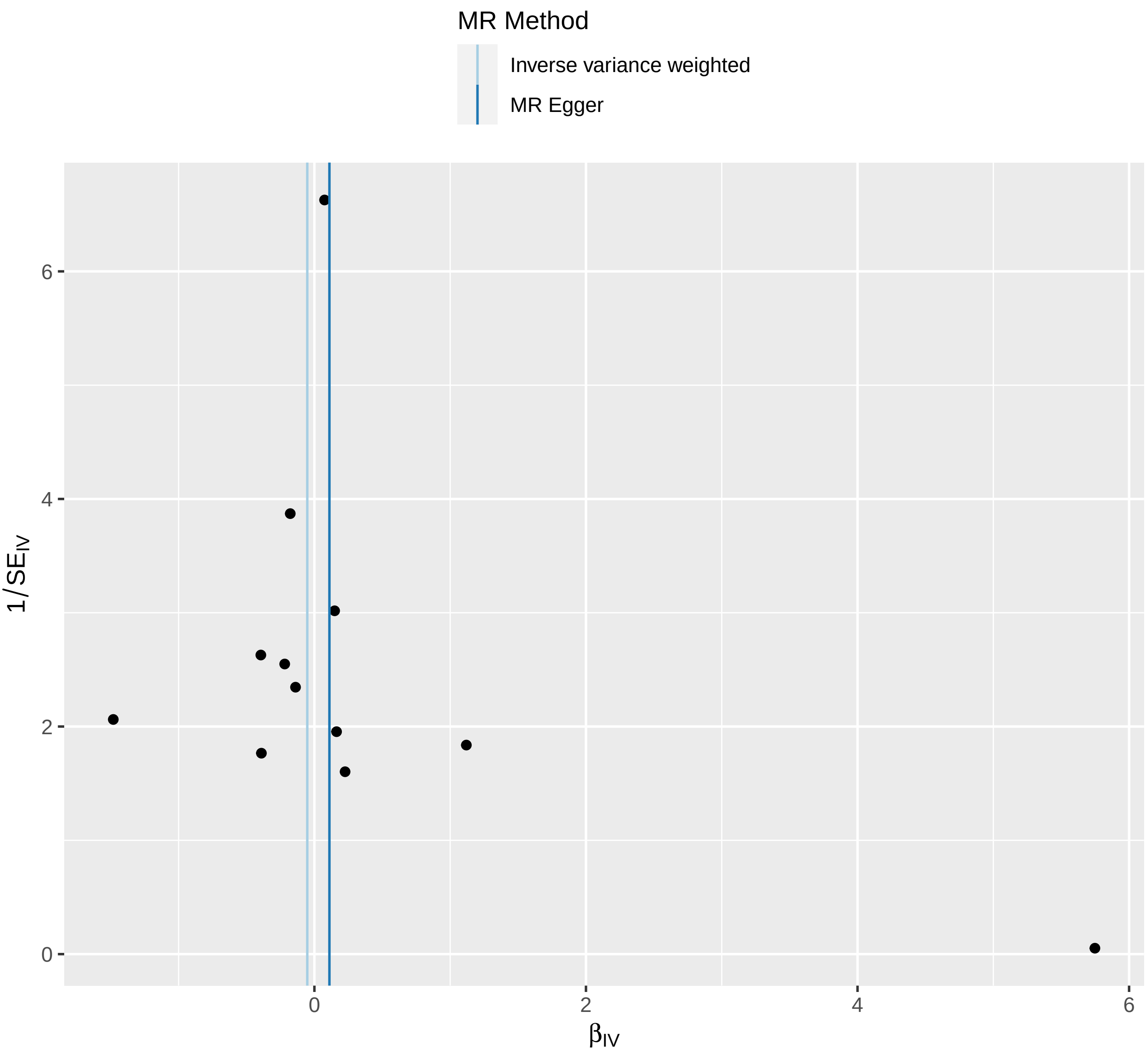
MR Method
Inverse variance weighted
MR Egger
6
4
2
0
0
2
4
6
$1/SE_{IV}$
β_{IV}

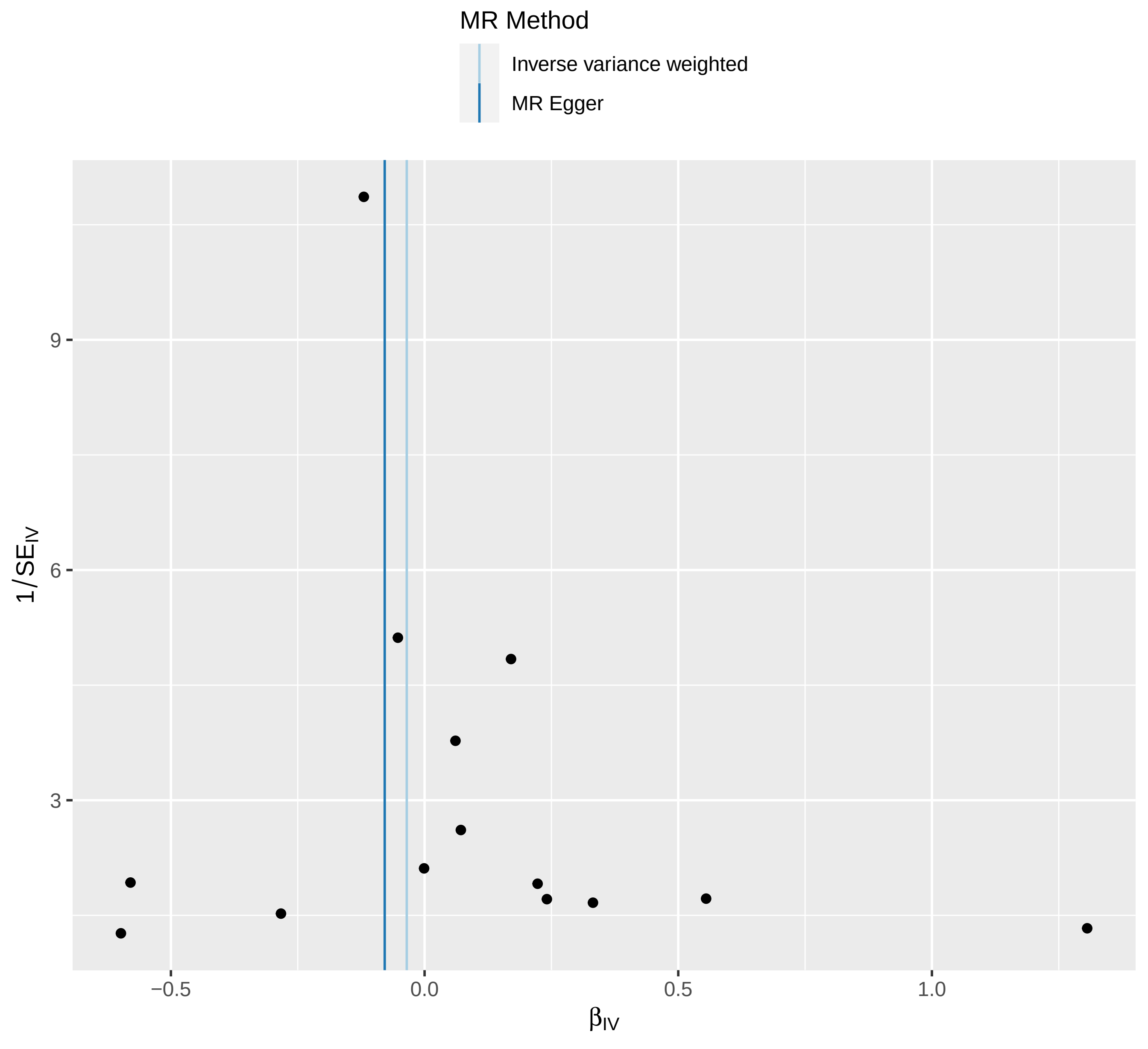

MR Method
Inverse variance weighted
MR Egger
9
6
3
1/SE$_{IV}$
−0.5
0.0
0.5
1.0
β$_{IV}$

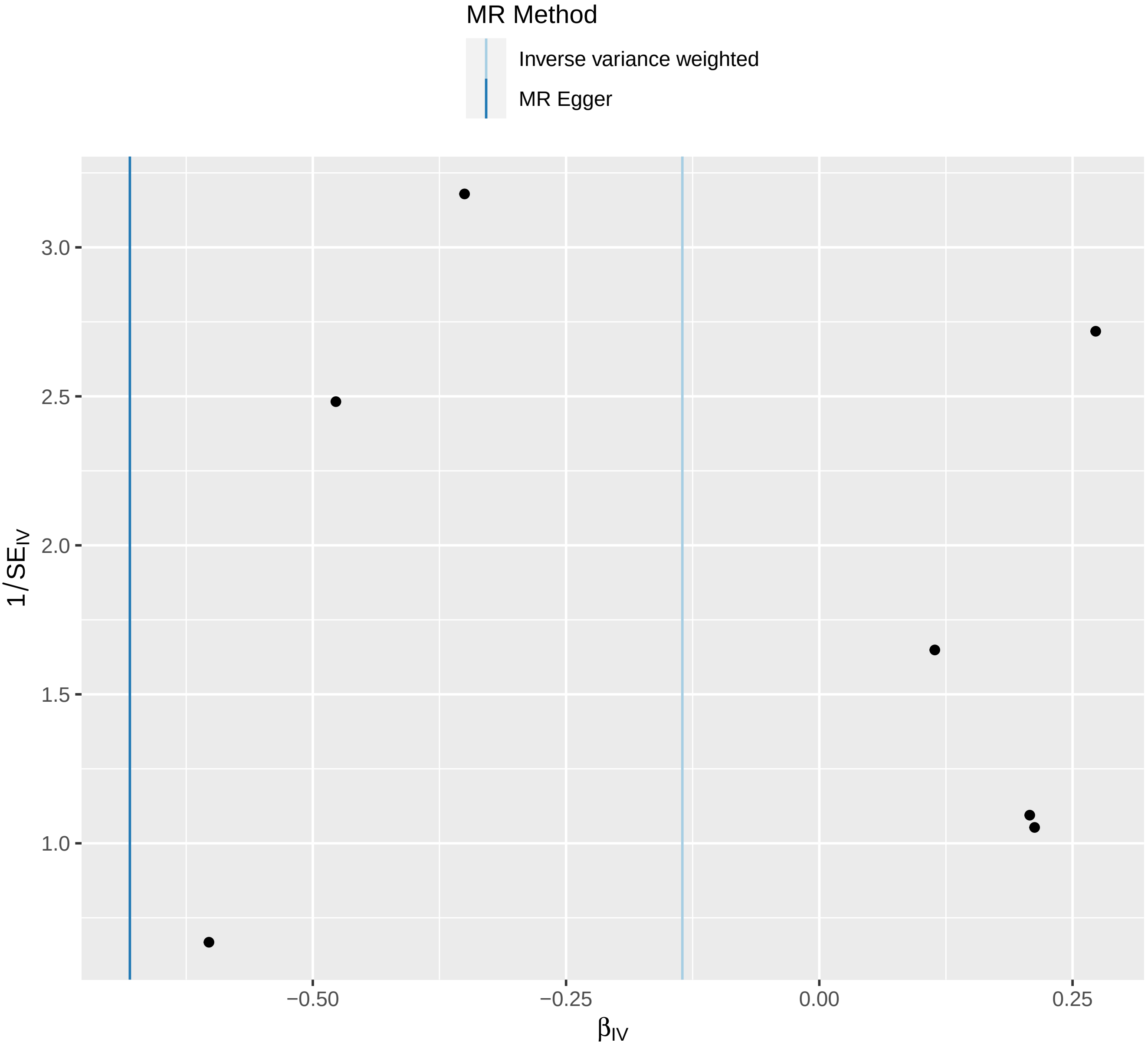

MR Method
Inverse variance weighted
MR Egger
3.0
2.5
2.0
1.5
1.0
$1/SE_{IV}$
−0.50
−0.25
0.00
0.25
β_{IV}

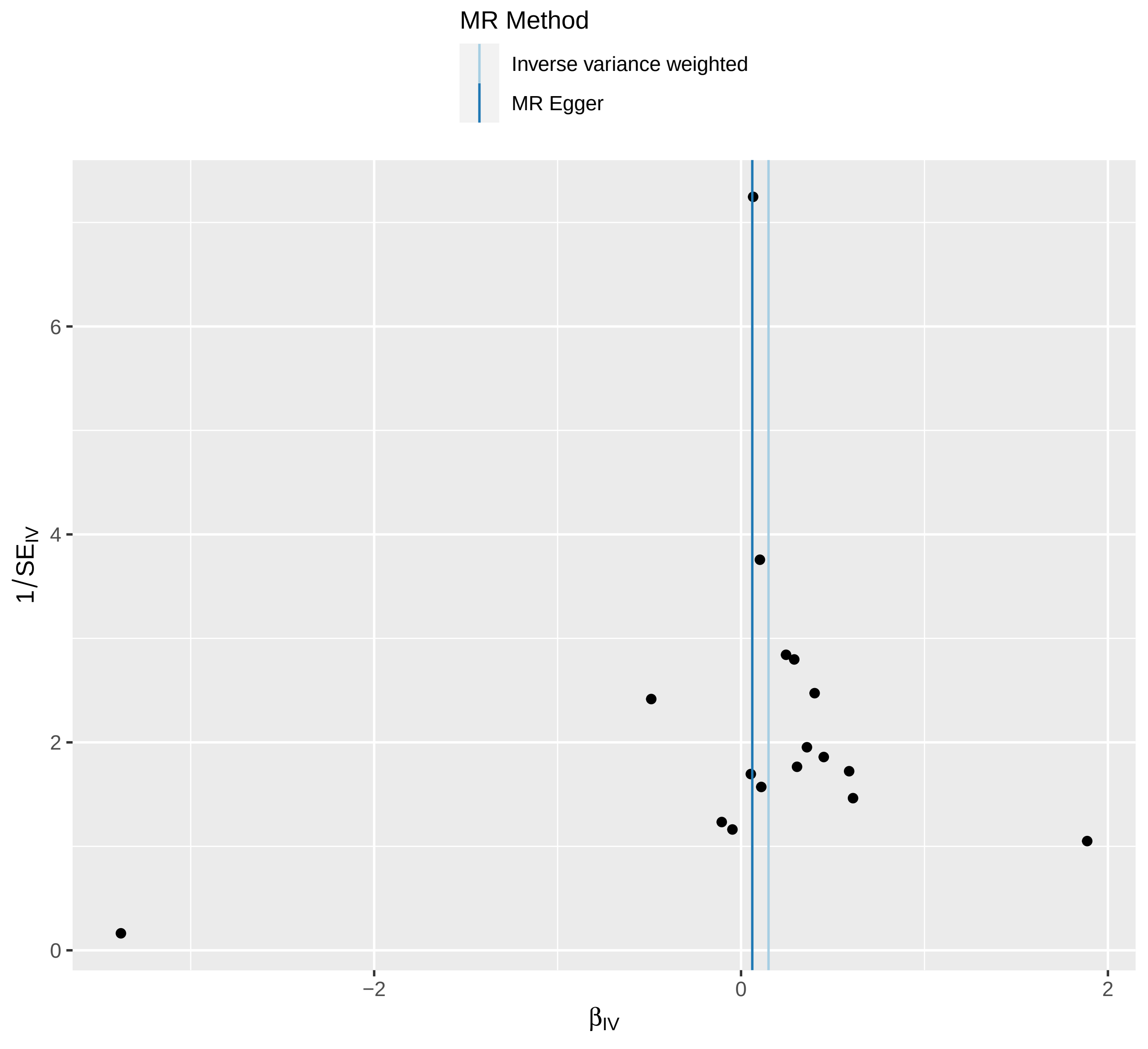
MR Method
Inverse variance weighted
MR Egger
6
4
2
0
1/SE_IV
−2
0
2
β_IV

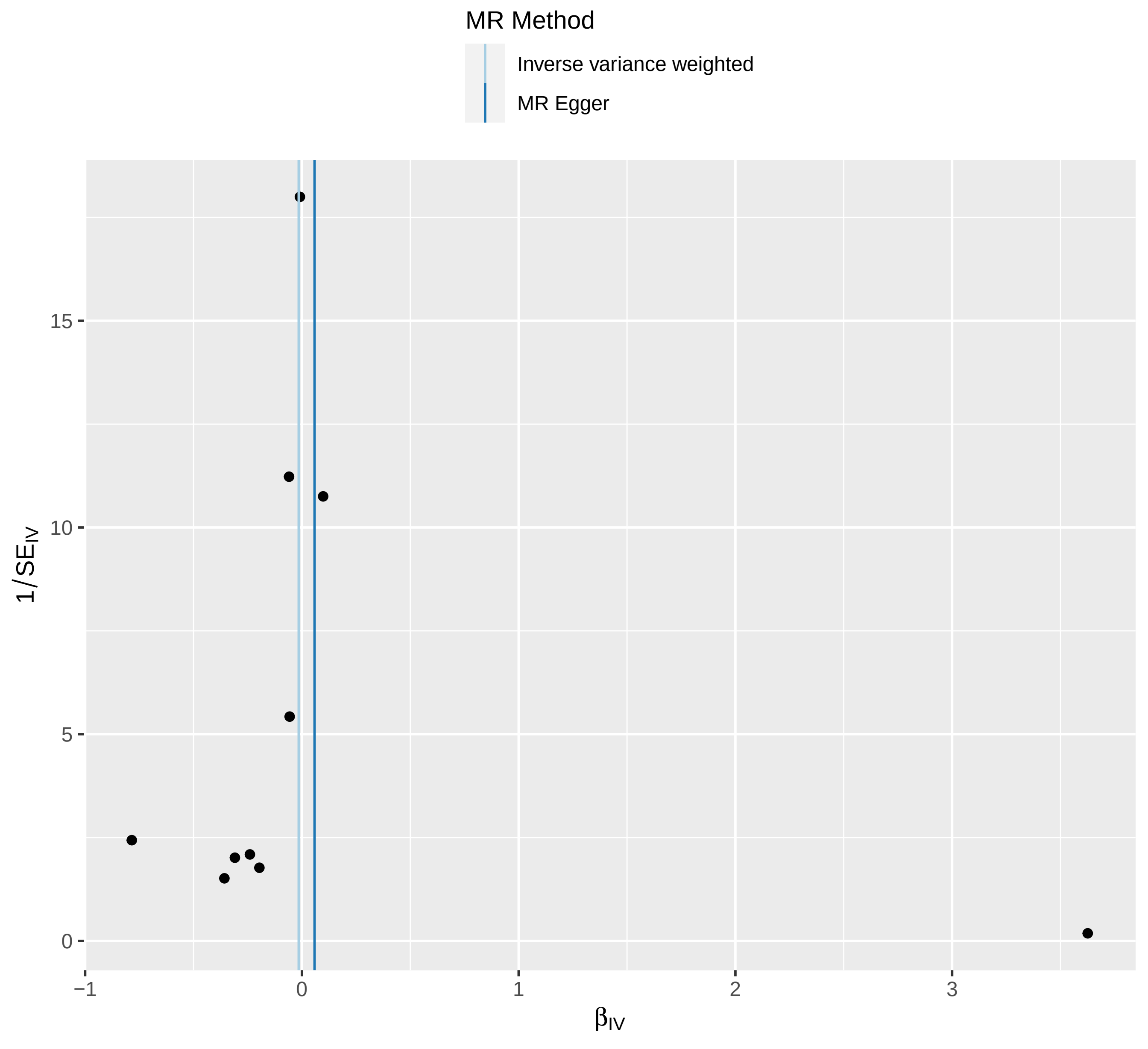

MR Method
Inverse variance weighted
MR Egger
1/SE_IV
β_IV
15
10
5
0
−1
0
1
2
3

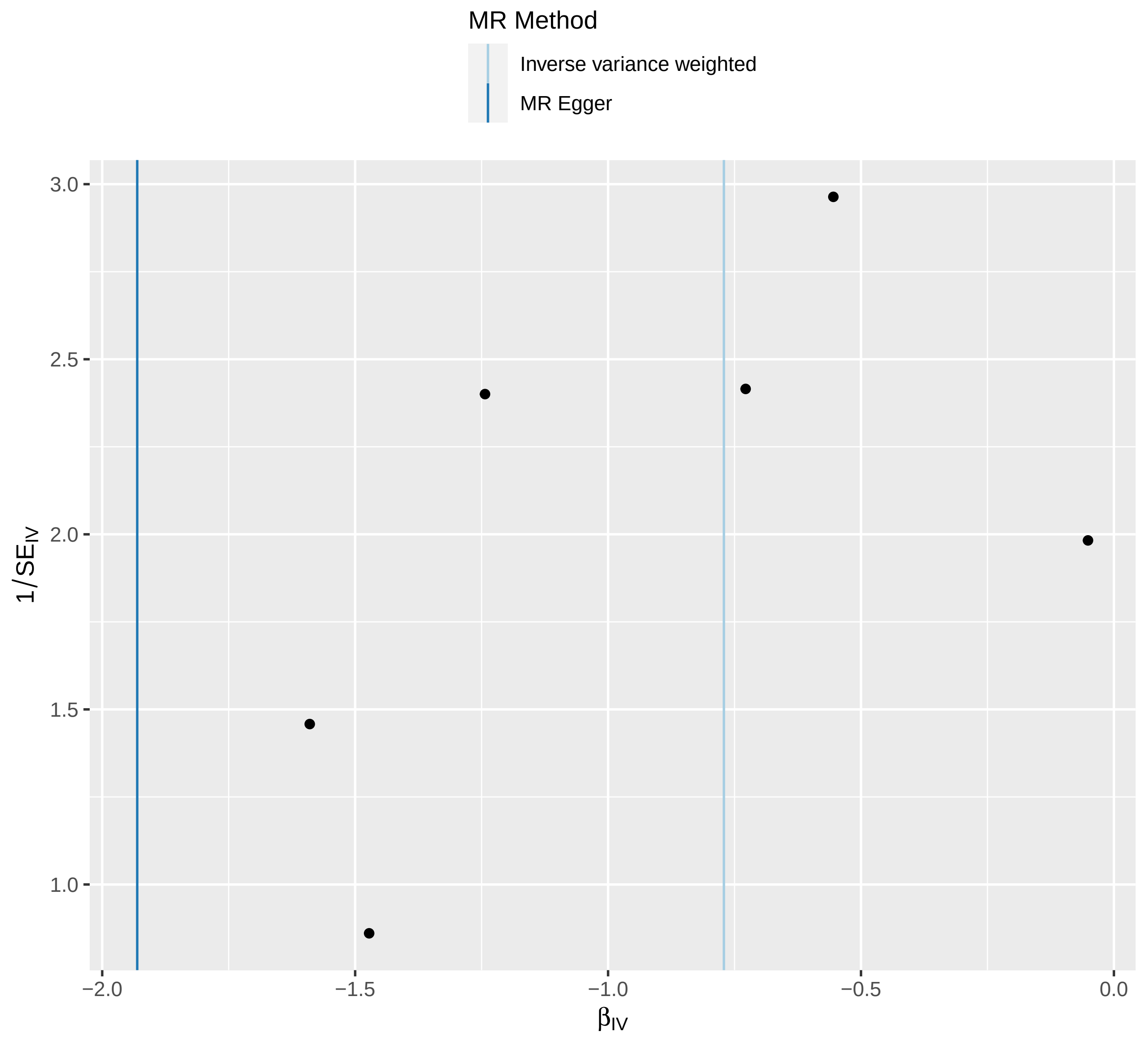

MR Method
Inverse variance weighted
MR Egger
3.0
2.5
2.0
1.5
1.0
1/SE_IV
−2.0
−1.5
−1.0
−0.5
0.0
β_IV

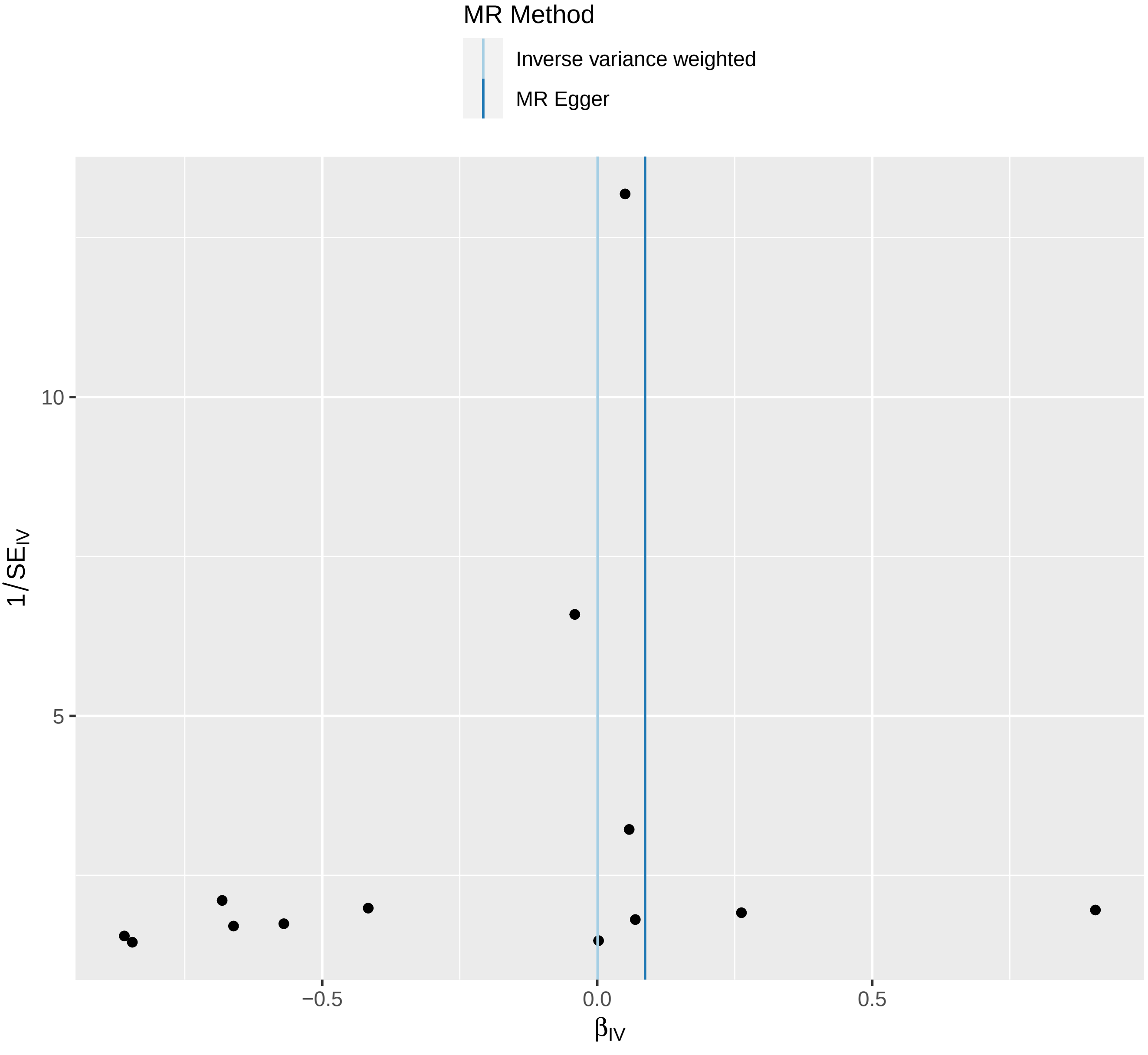
MR Method
Inverse variance weighted
MR Egger
10
5
1/SE$_{IV}$
−0.5
0.0
0.5
β$_{IV}$

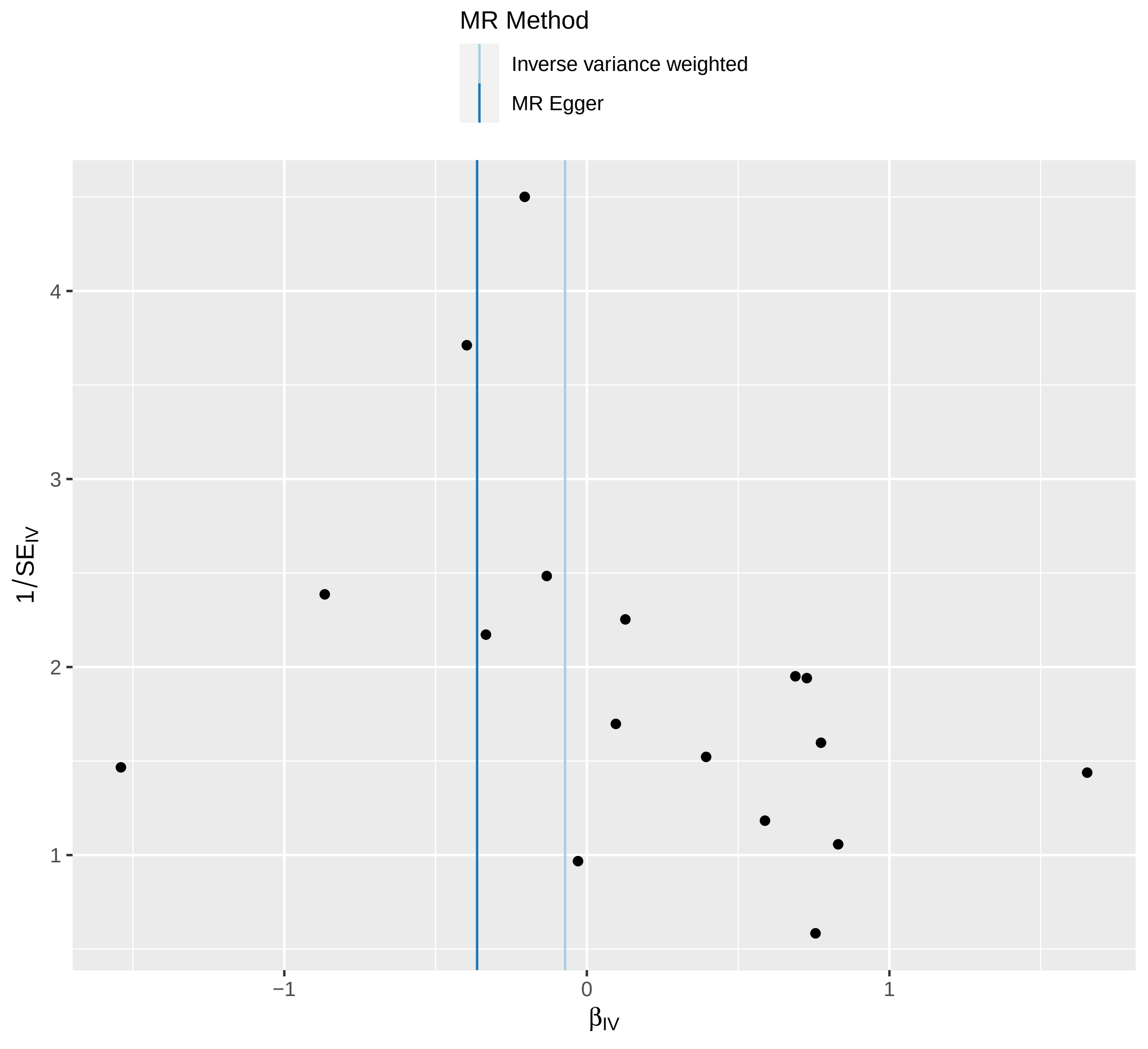
MR Method
Inverse variance weighted
MR Egger
1/SE_IV
4
3
2
1
−1
0
1
β_IV

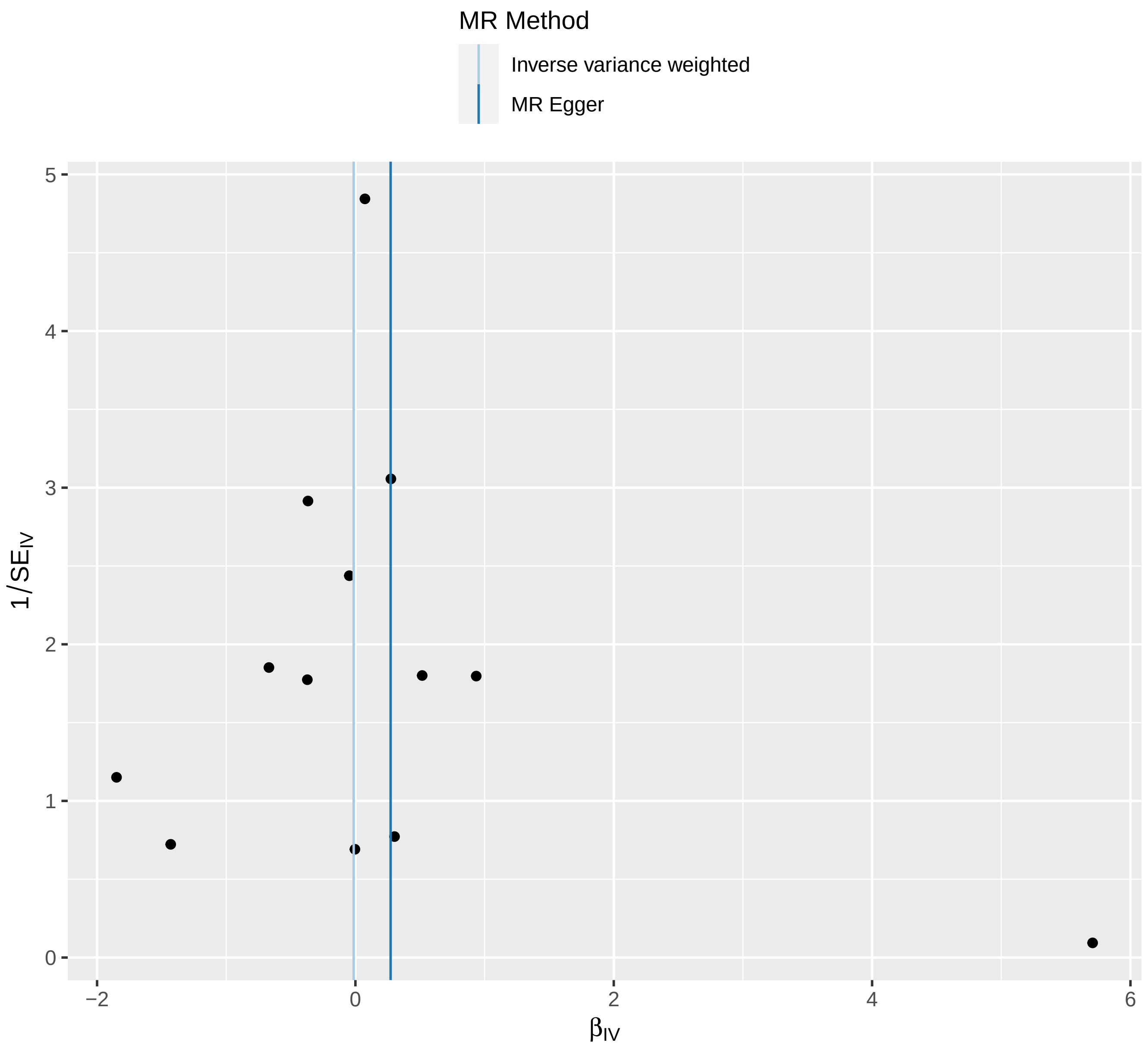
MR Method
Inverse variance weighted
MR Egger
1/SE_IV
β_IV
5
4
3
2
1
0
−2
0
2
4
6

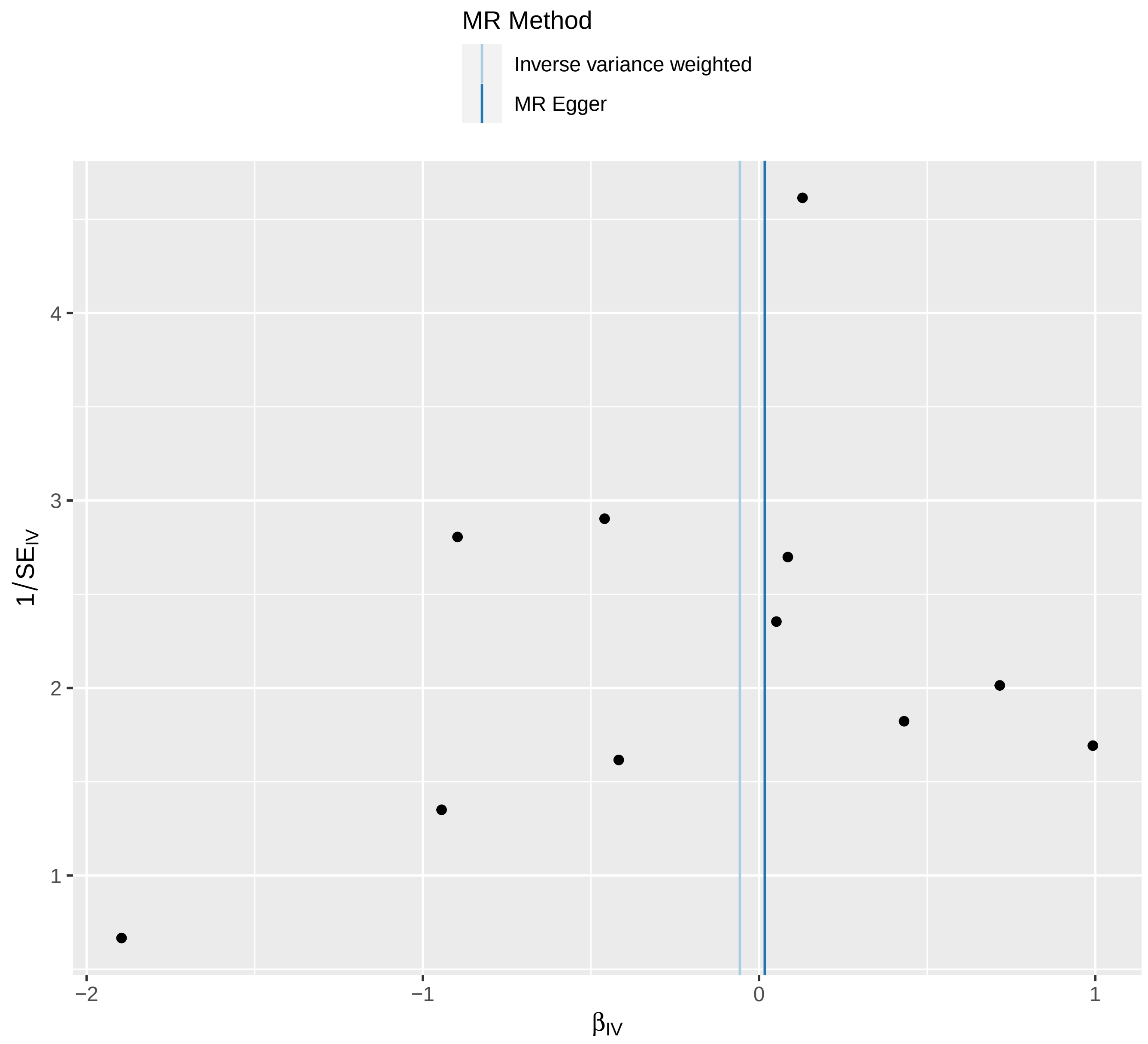
MR Method
Inverse variance weighted
MR Egger
1/SE_IV
4
3
2
1
−2
−1
0
1
β_IV

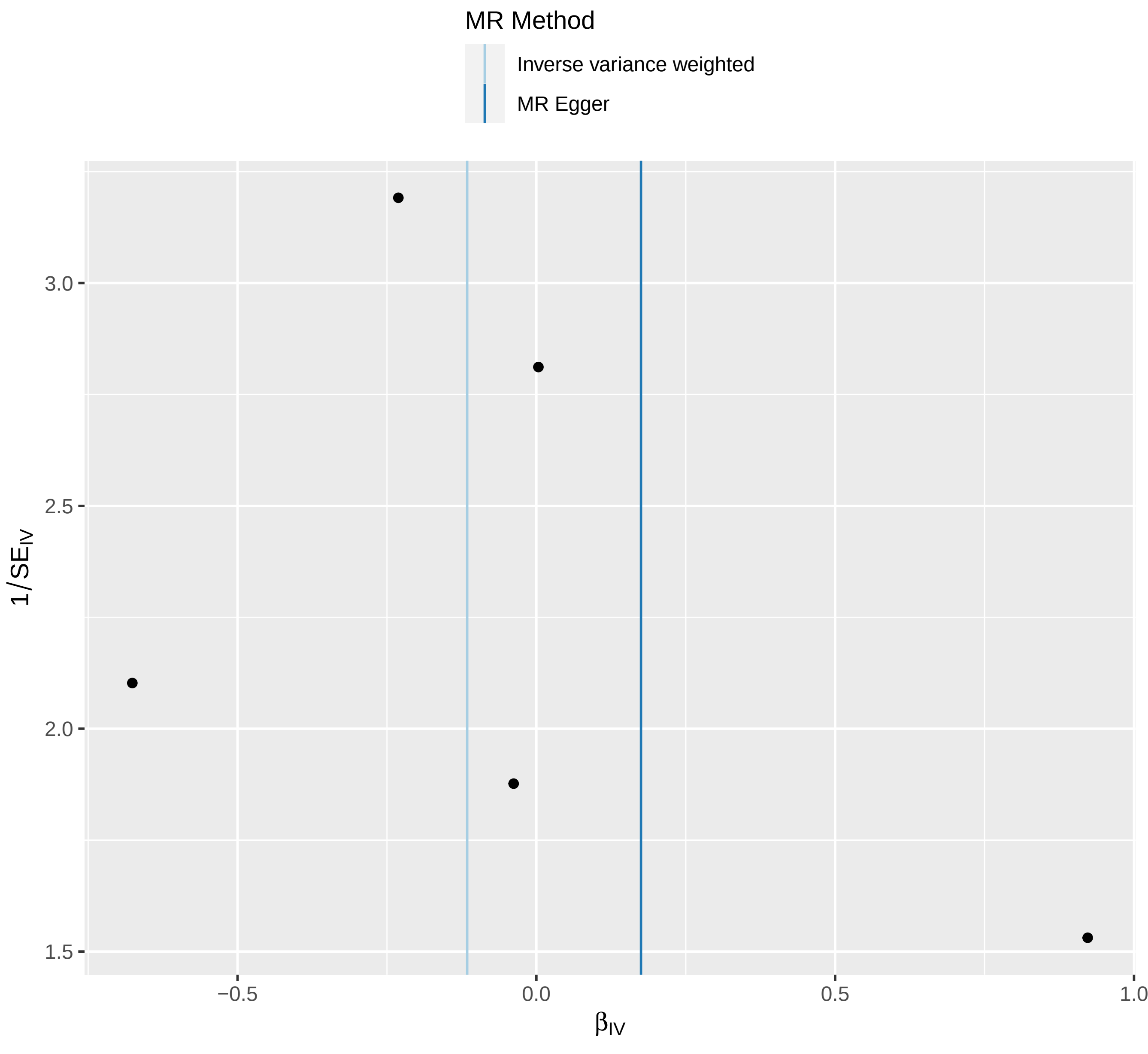

MR Method
Inverse variance weighted
MR Egger
3.0
2.5
2.0
1.5
1/SE_IV
−0.5
0.0
0.5
1.0
β_IV

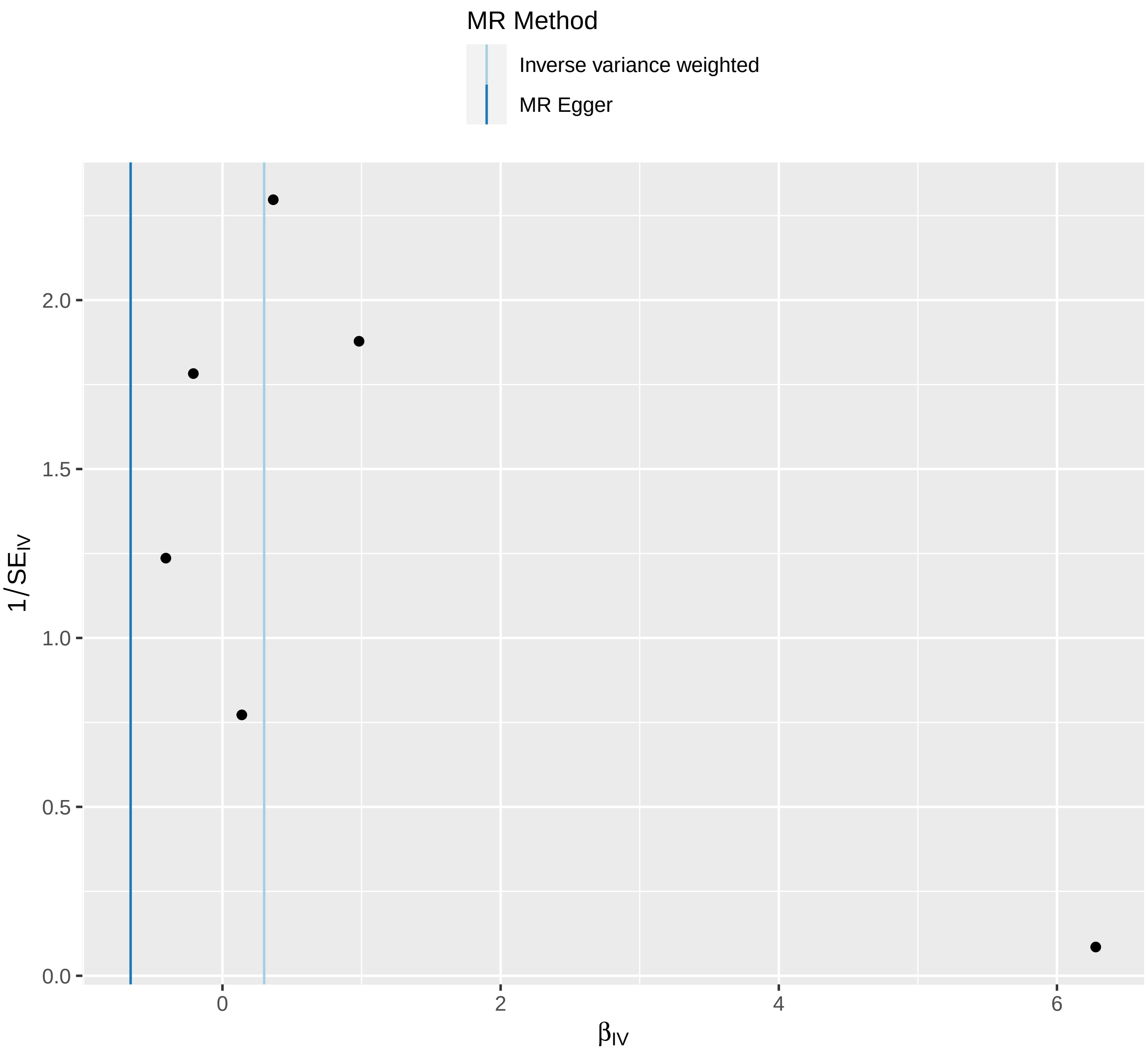
MR Method
Inverse variance weighted
MR Egger
1/SE_IV
β_IV
2.0
1.5
1.0
0.5
0.0
0
2
4
6

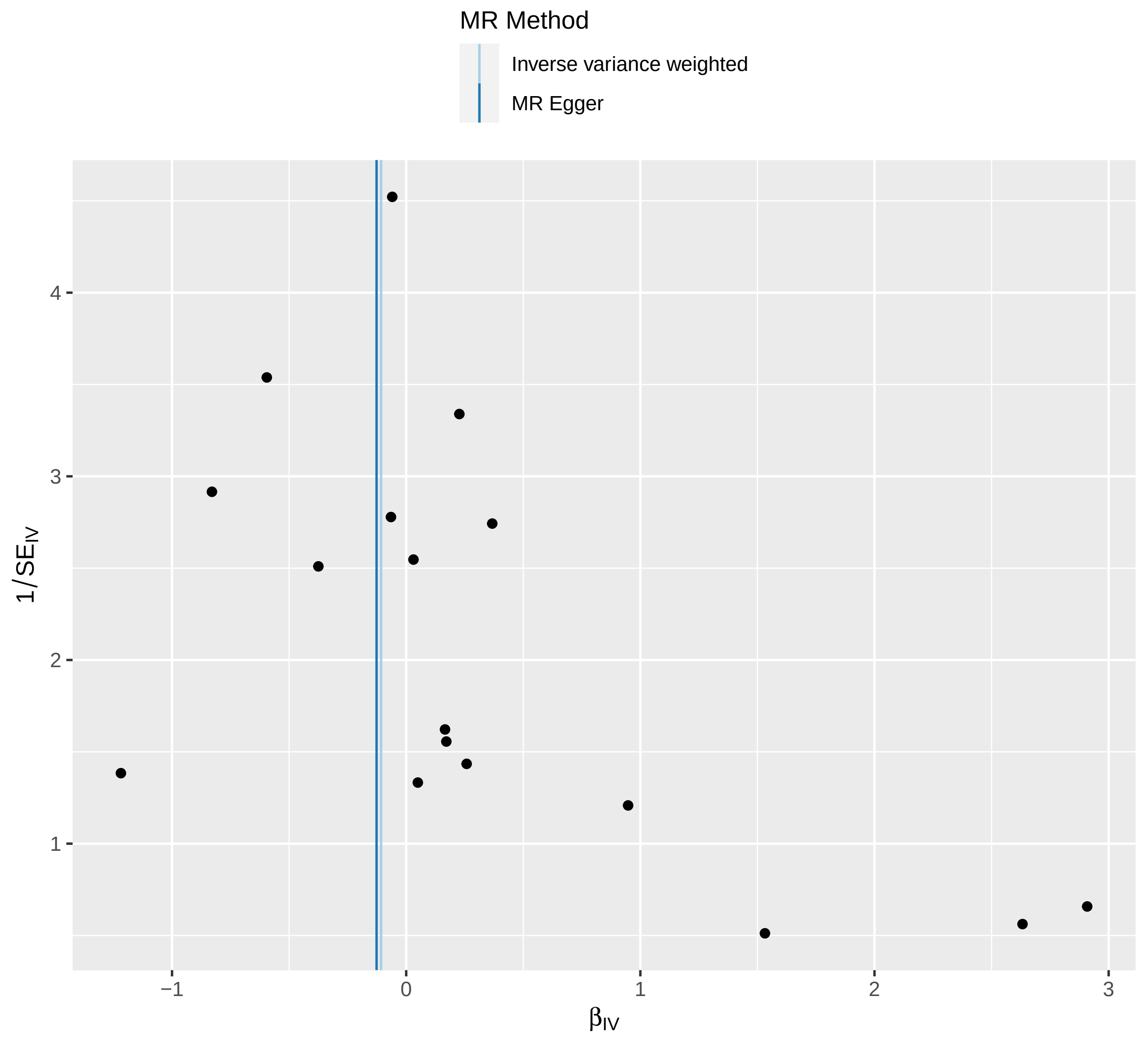

MR Method
Inverse variance weighted
MR Egger
4
3
2
1
1/SE$_{IV}$
−1
0
1
2
3
β$_{IV}$

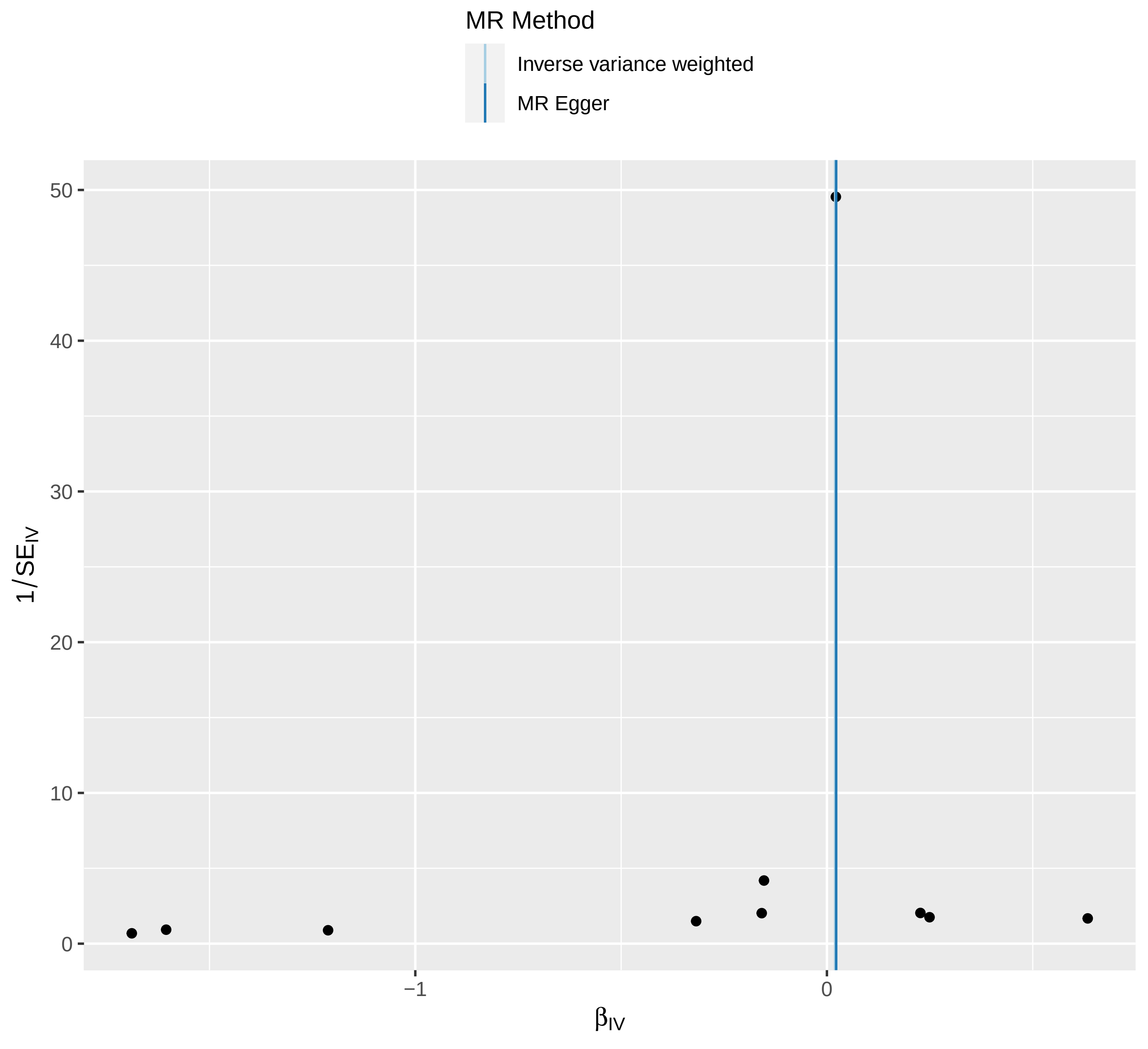

MR Method
Inverse variance weighted
MR Egger
1/SE$_{IV}$
50
40
30
20
10
0
−1
0
β$_{IV}$

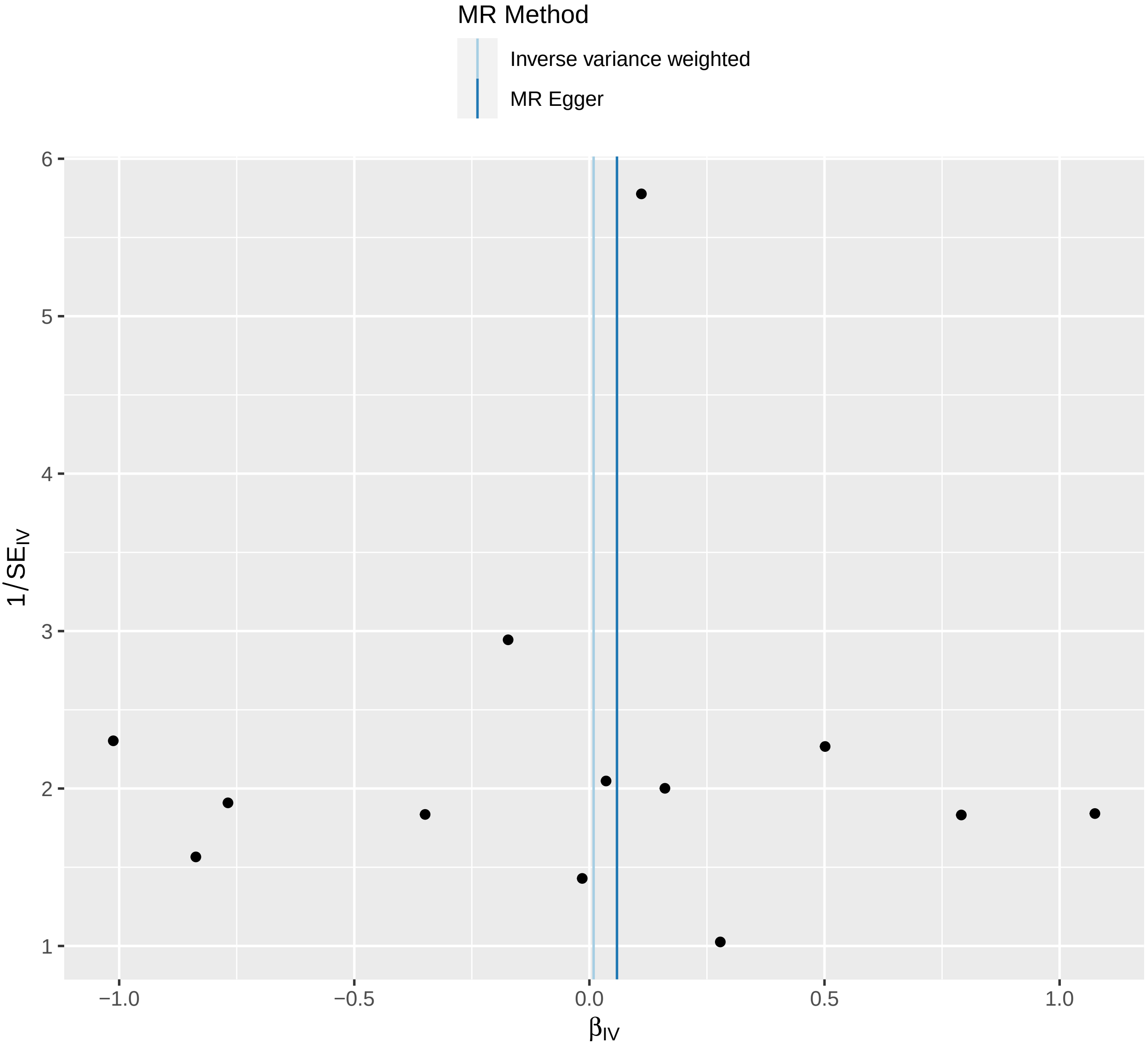
MR Method
Inverse variance weighted
MR Egger
$1/SE_{IV}$
β_{IV}
6
5
4
3
2
1
−1.0
−0.5
0.0
0.5
1.0

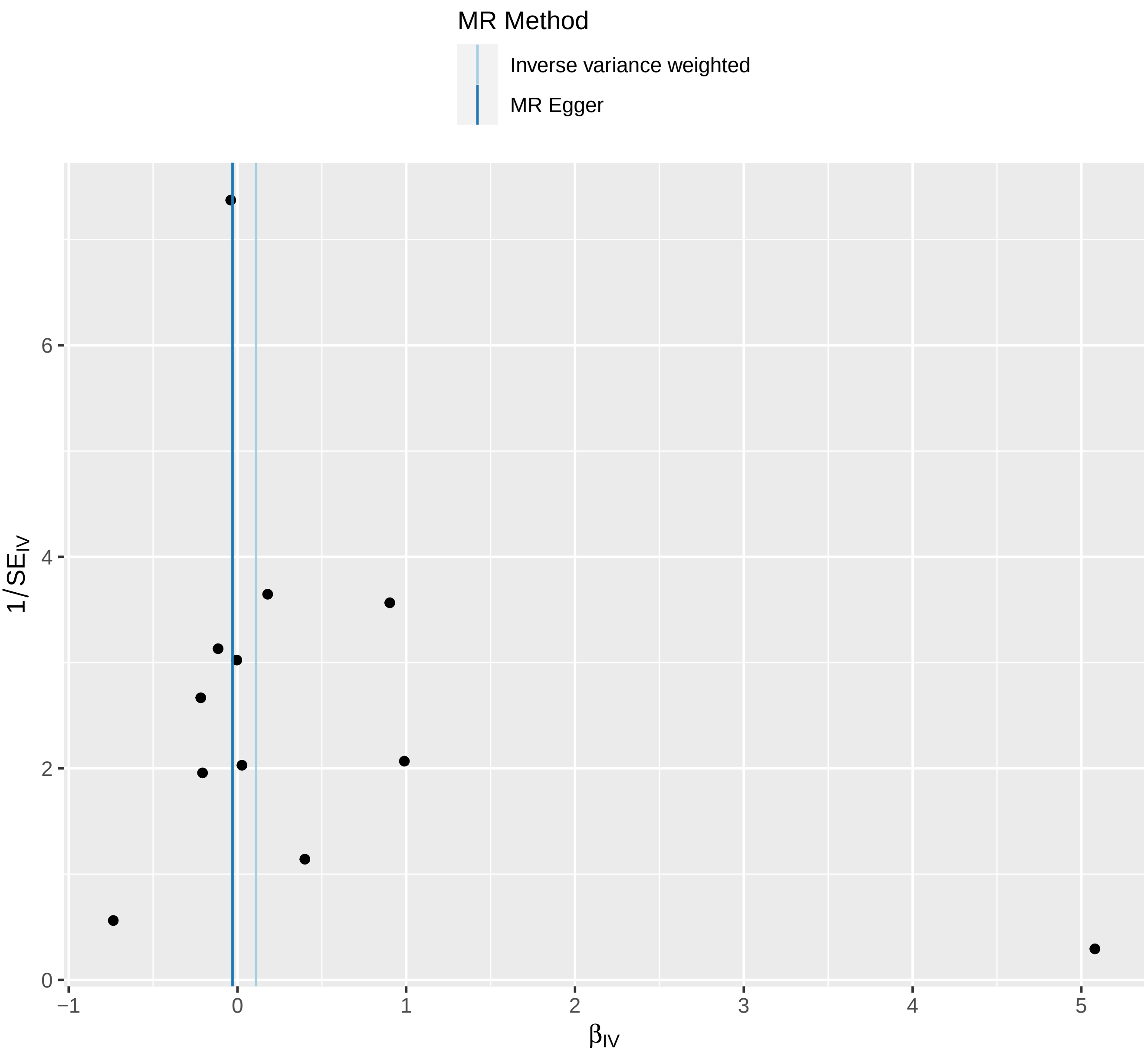
MR Method
Inverse variance weighted
MR Egger
1/SE_IV
β_IV
0
2
4
6
−1
0
1
2
3
4
5

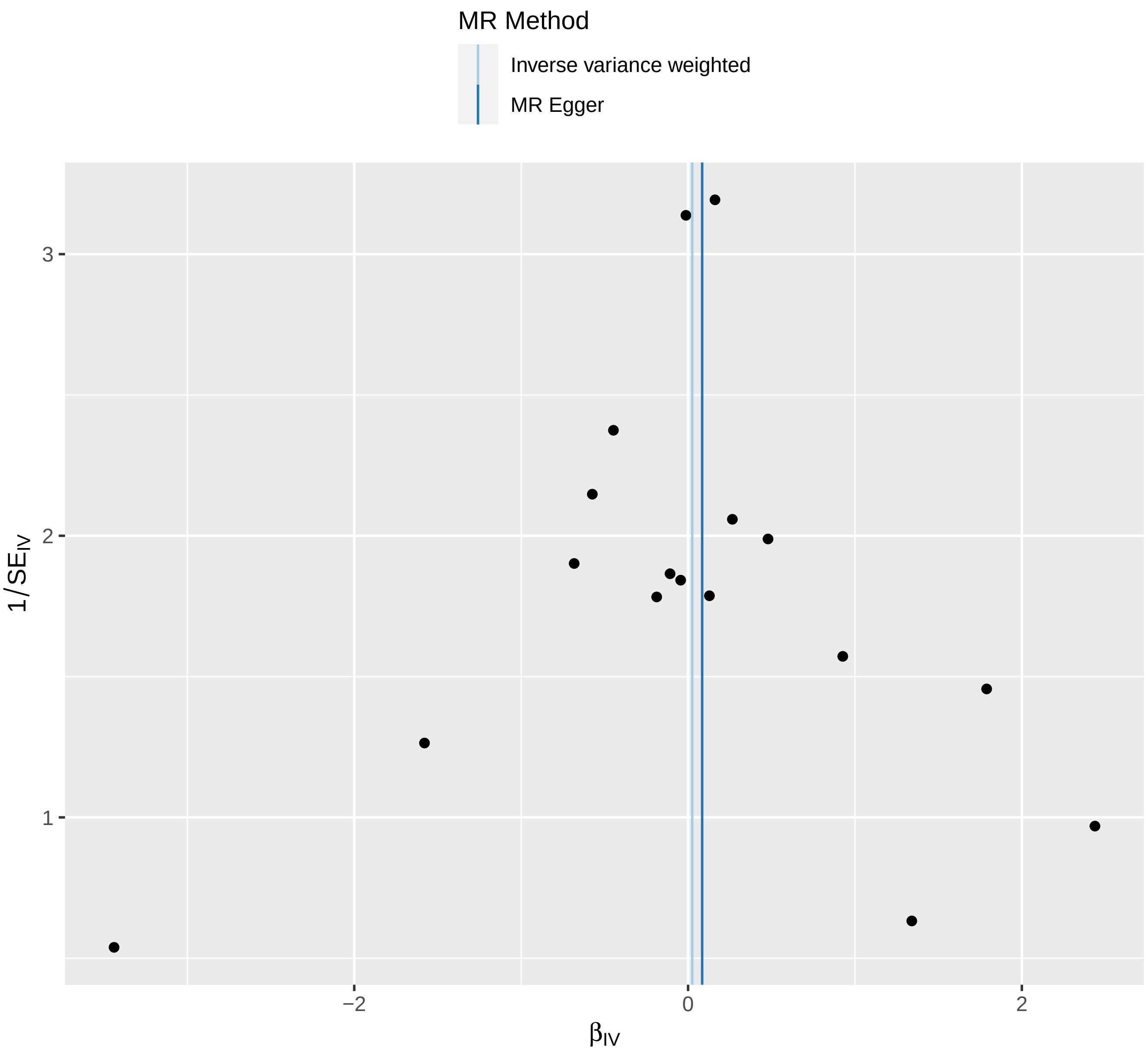

MR Method
Inverse variance weighted
MR Egger
3
2
1
1/SE$_{IV}$
−2
0
2
β$_{IV}$

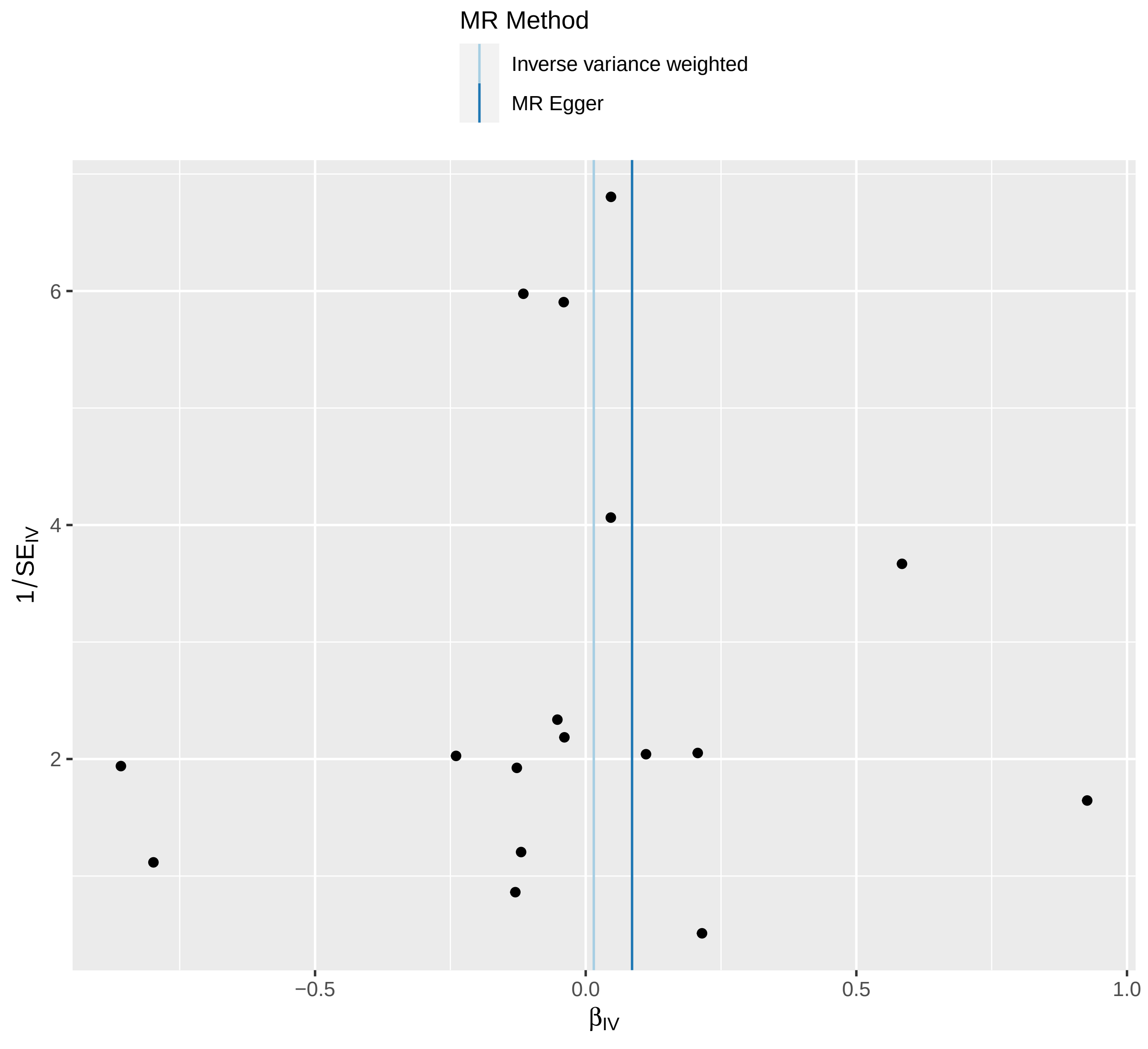

MR Method
Inverse variance weighted
MR Egger
6
4
2
1/SE_IV
−0.5
0.0
0.5
1.0
β_IV

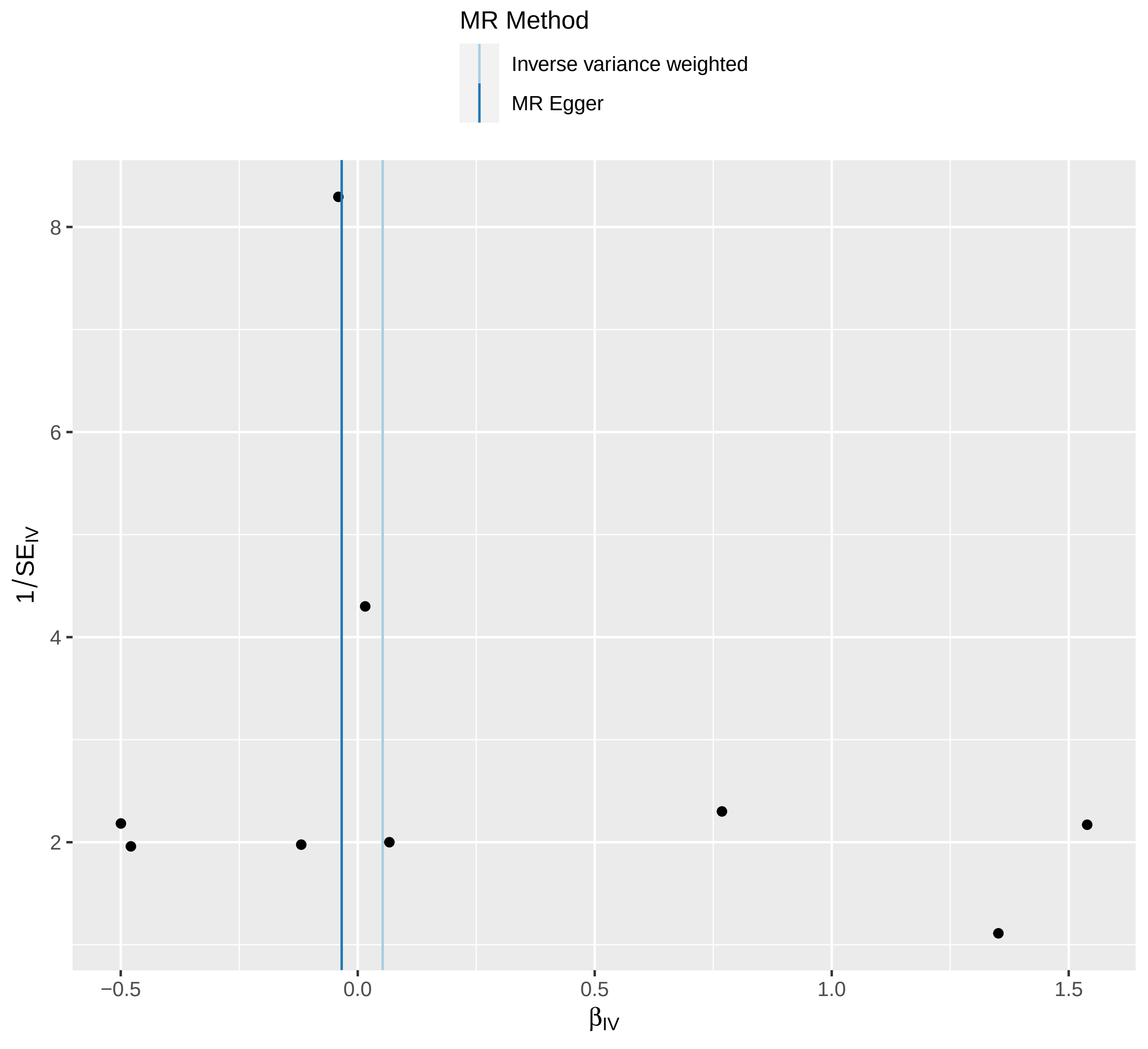

MR Method
Inverse variance weighted
MR Egger
8
6
4
2
$1/SE_{IV}$
−0.5
0.0
0.5
1.0
1.5
β_{IV}

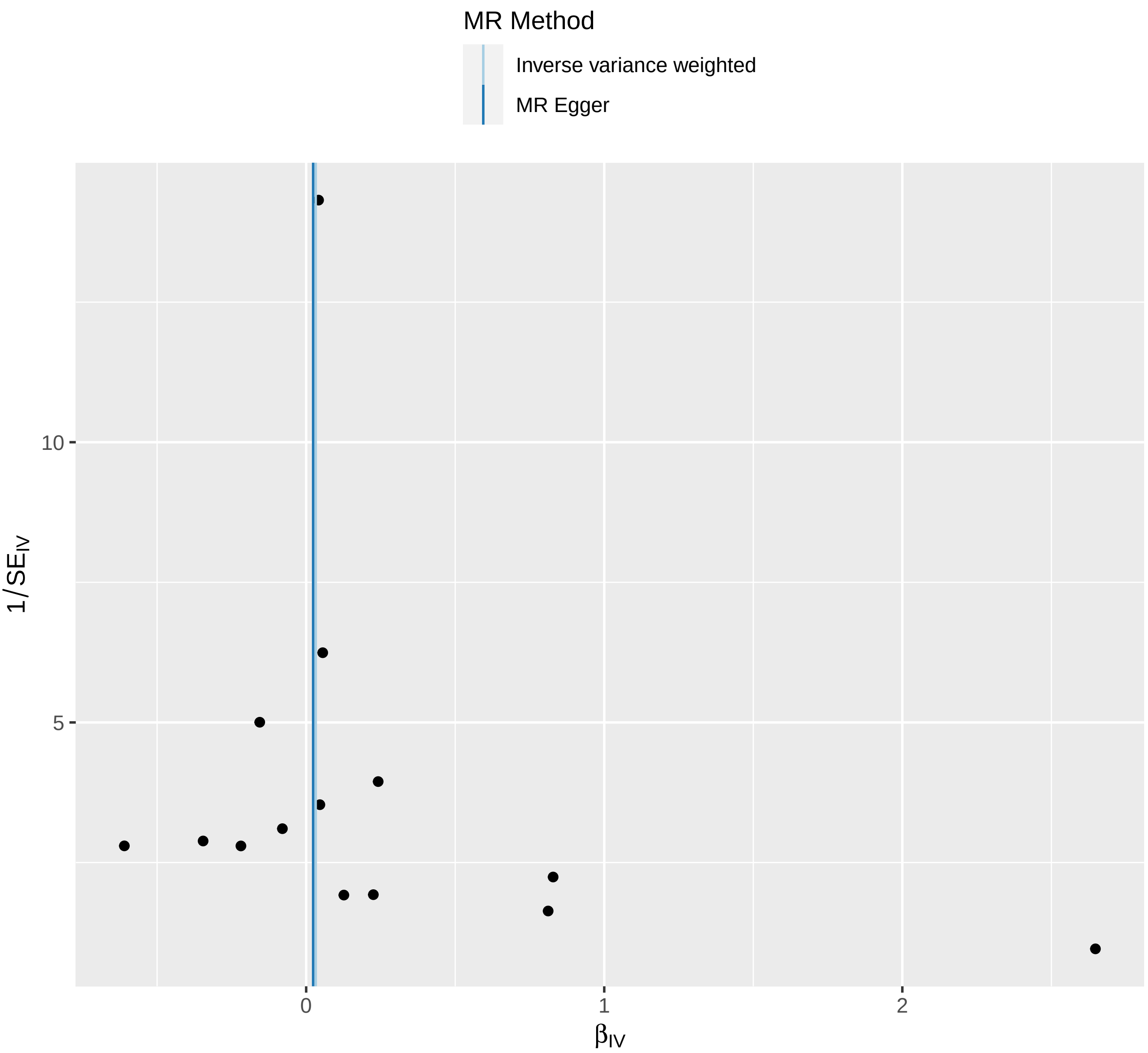

MR Method
Inverse variance weighted
MR Egger
10
5
0
1
2
1/SE$_{IV}$
β$_{IV}$

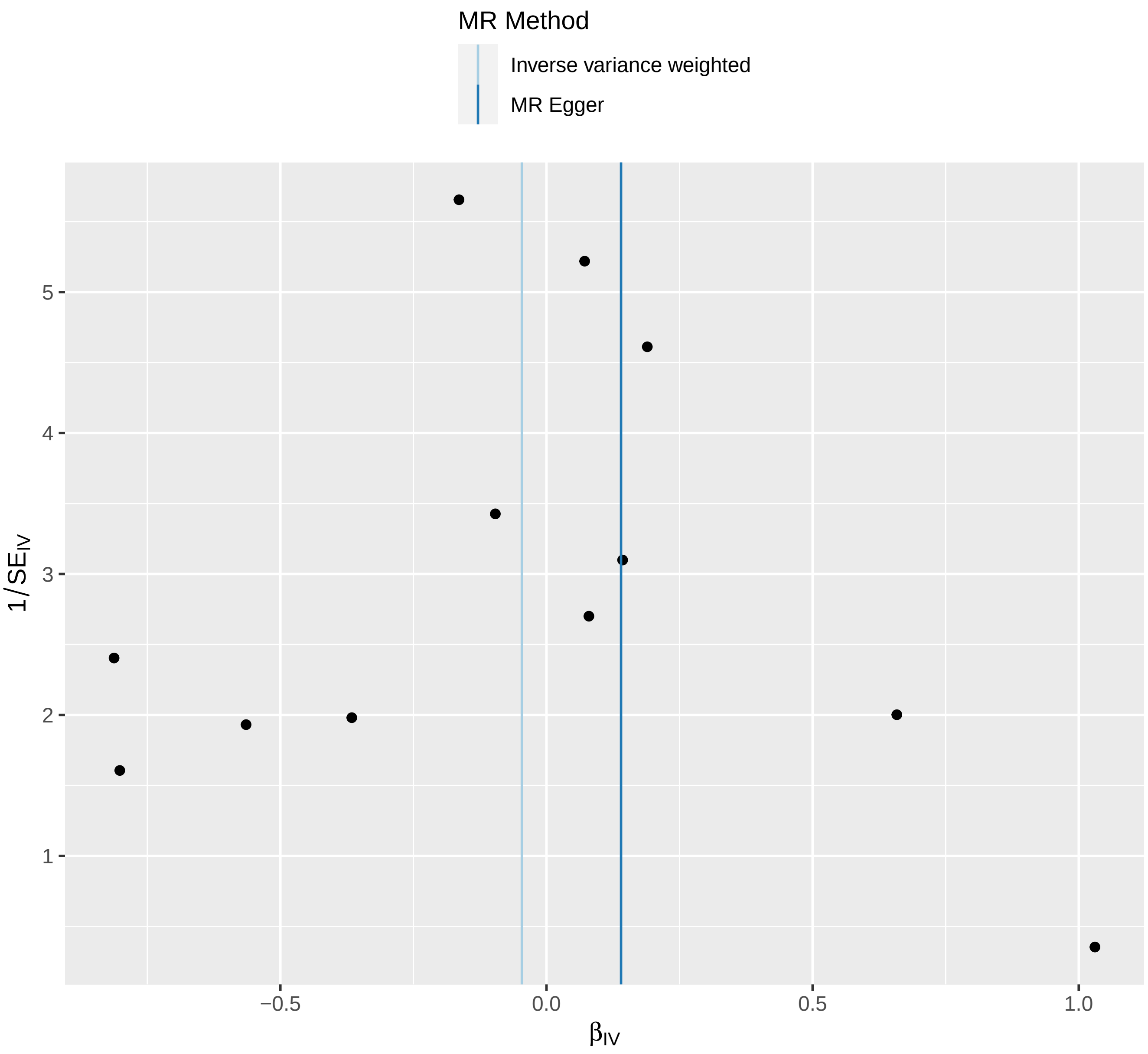

MR Method
Inverse variance weighted
MR Egger
5
4
3
2
1
1/SE$_{IV}$
−0.5
0.0
0.5
1.0
β$_{IV}$

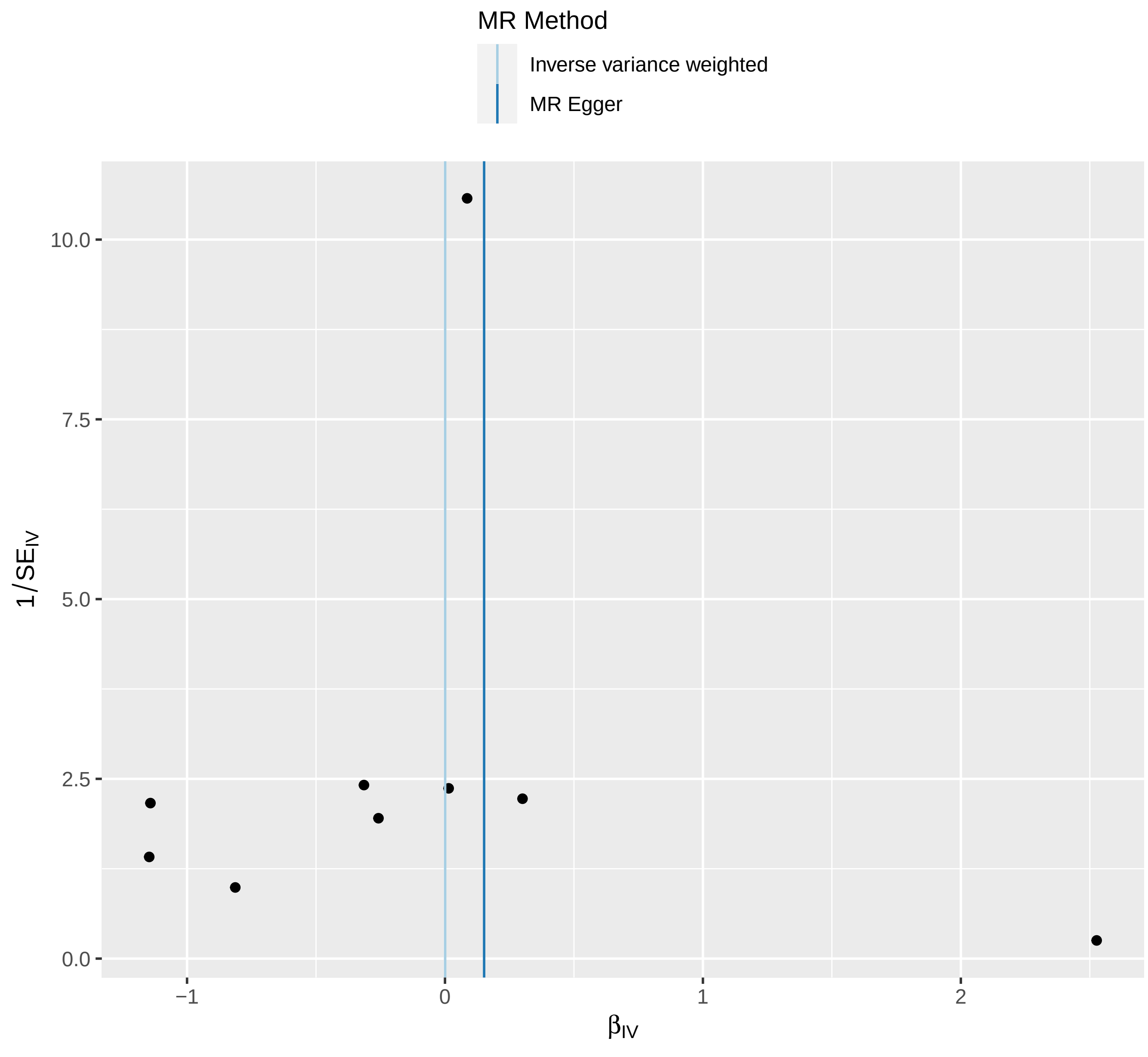

MR Method
Inverse variance weighted
MR Egger
10.0
7.5
5.0
2.5
0.0
1/SE$_{IV}$
−1
0
1
2
β$_{IV}$

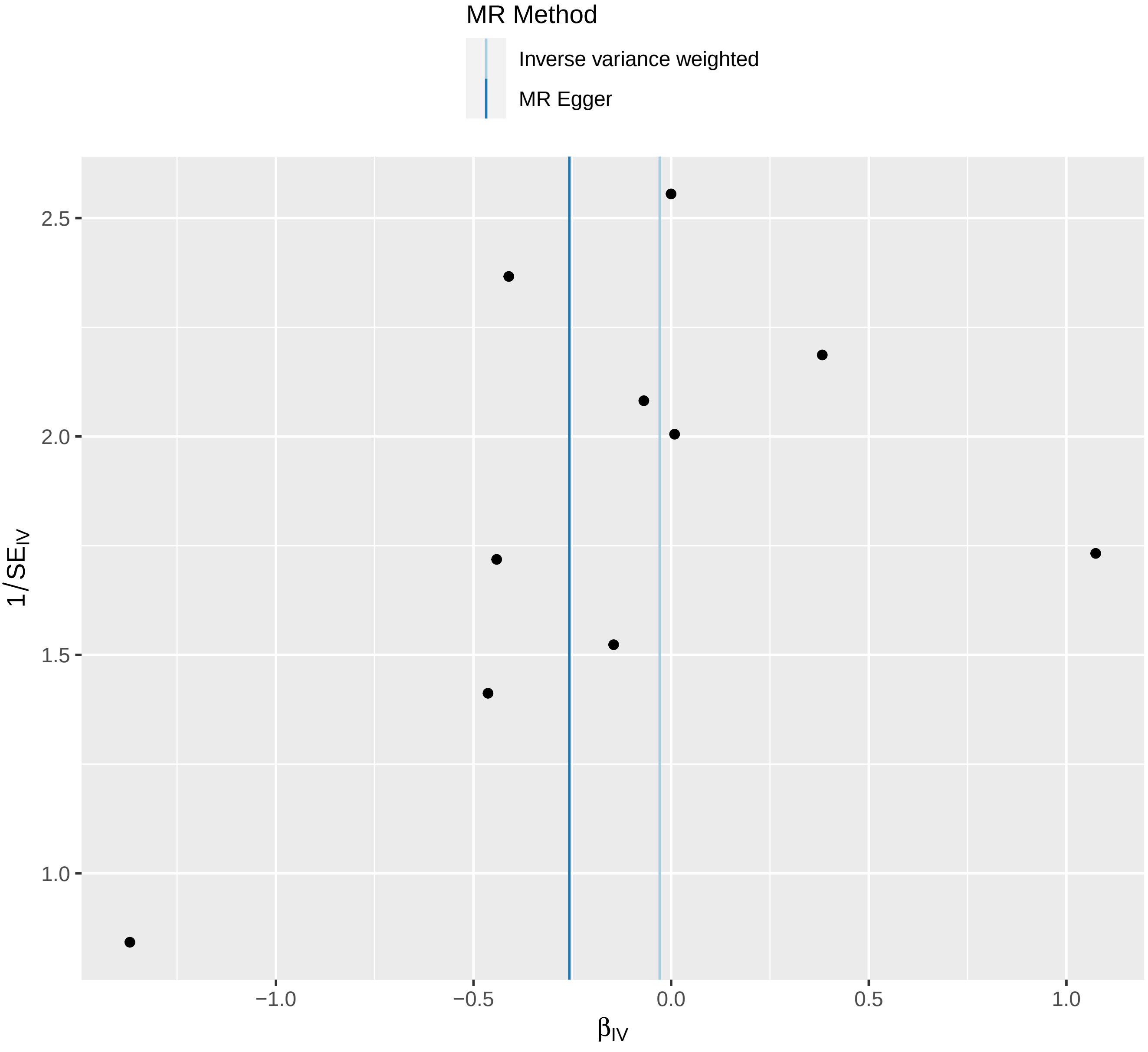
MR Method
Inverse variance weighted
MR Egger
2.5
2.0
1.5
1.0
1/SE_IV
−1.0
−0.5
0.0
0.5
1.0
β_IV

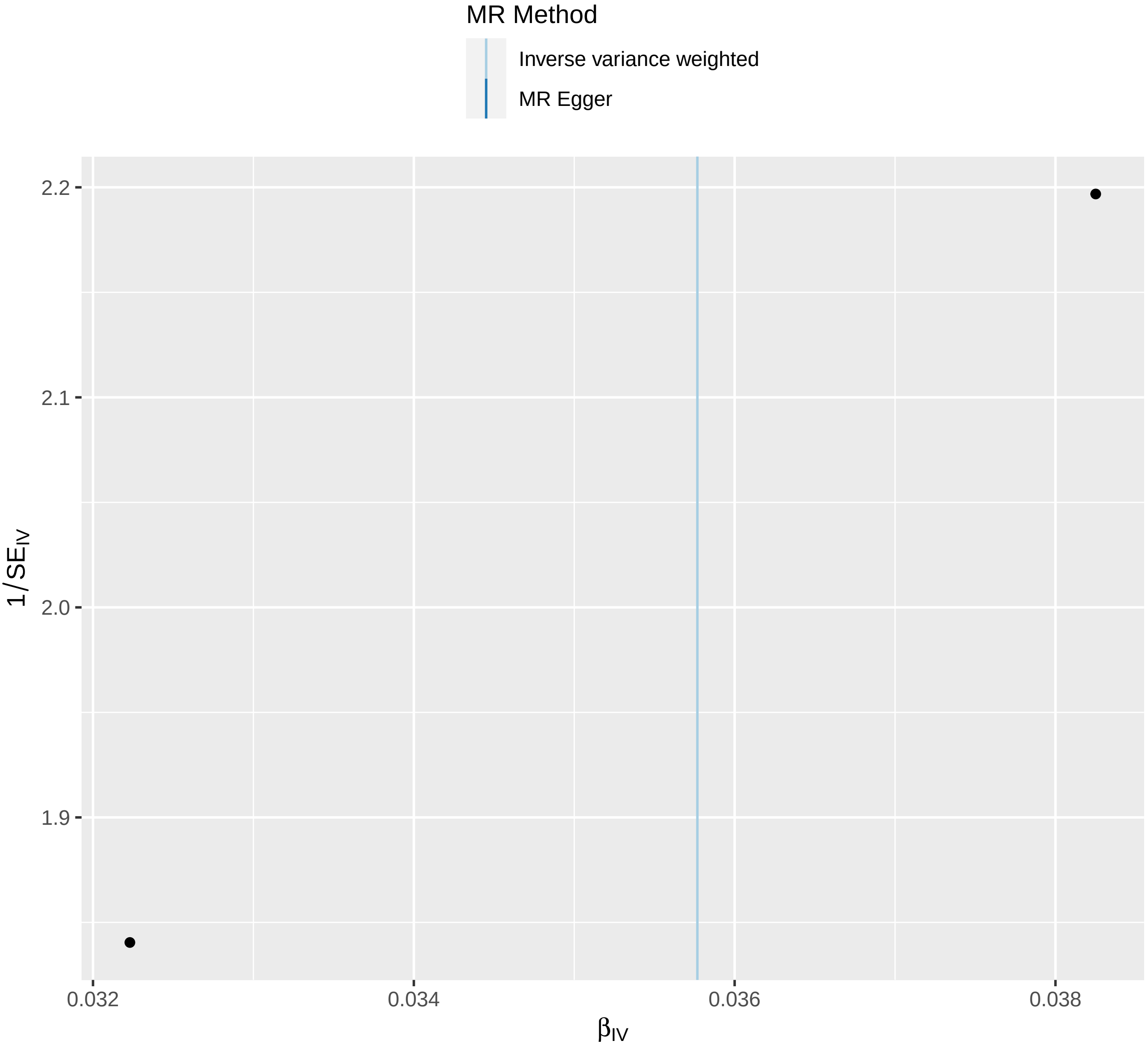

MR Method
Inverse variance weighted
MR Egger
2.2
2.1
2.0
1.9
1/SE_IV
0.032
0.034
0.036
0.038
β_IV

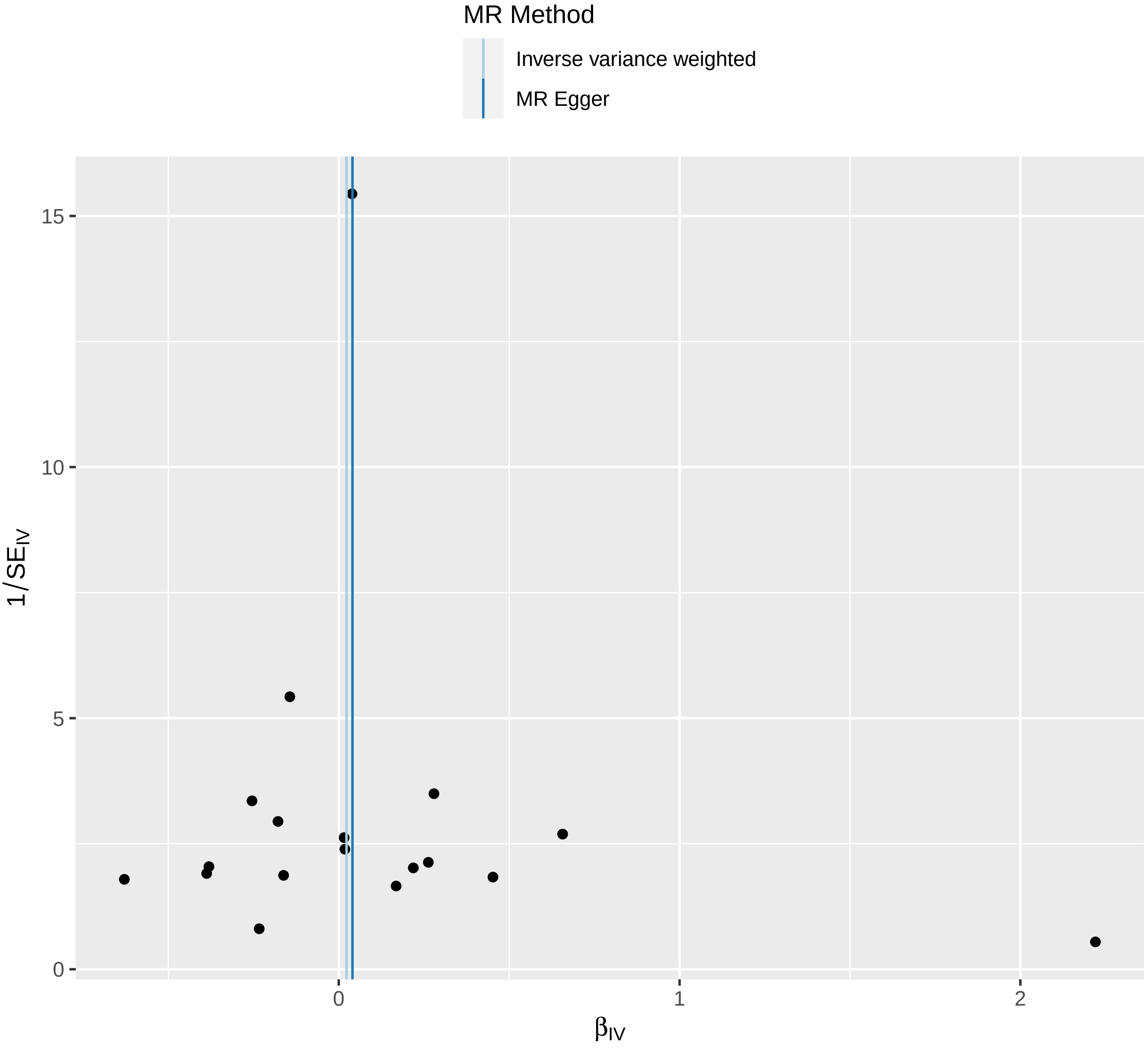

MR Method
Inverse variance weighted
MR Egger
15
10
5
0
1/SE$_{IV}$
0
1
2
β$_{IV}$

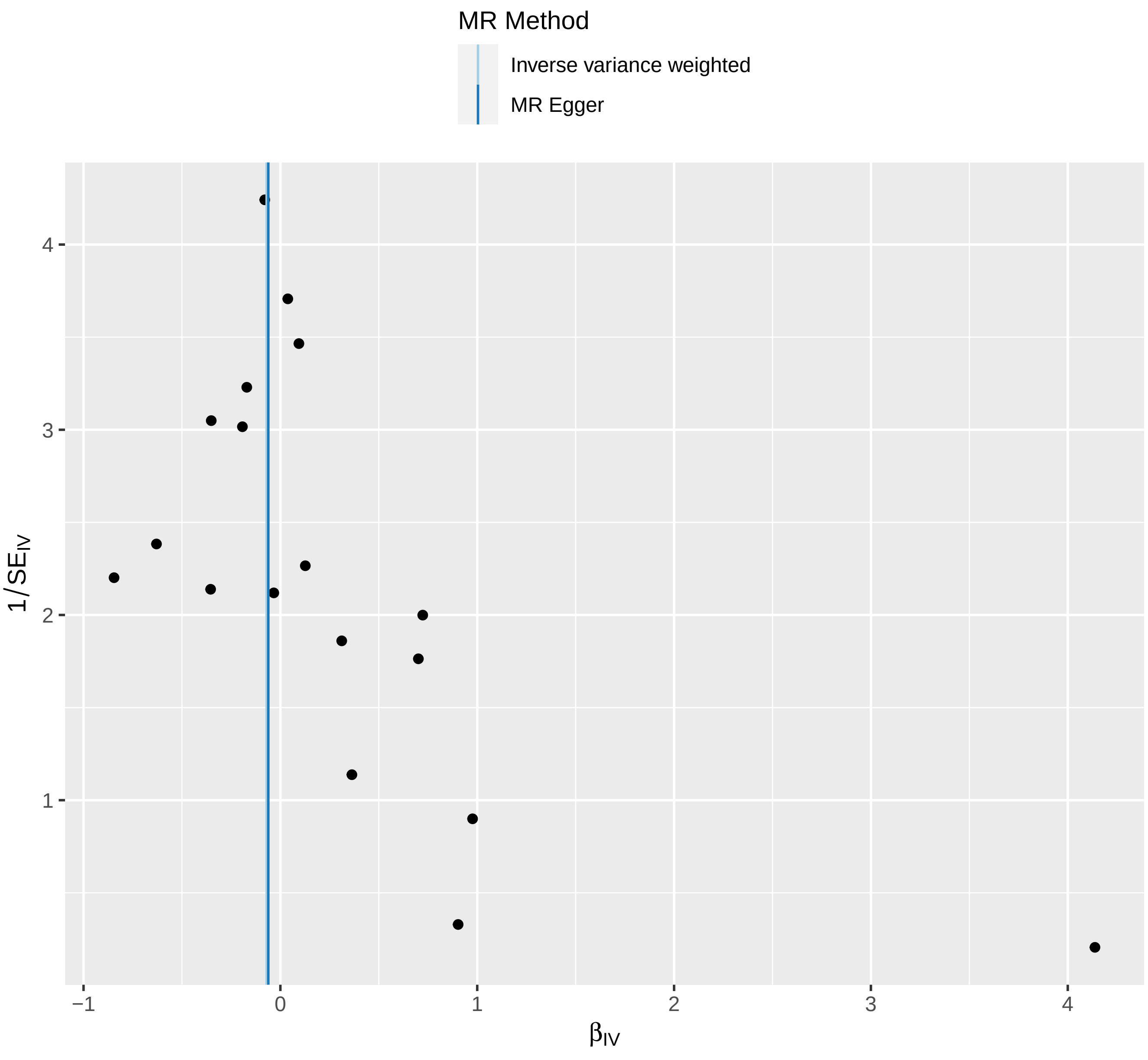
MR Method
Inverse variance weighted
MR Egger
1/SE_IV
β_IV
4
3
2
1
−1
0
1
2
3
4

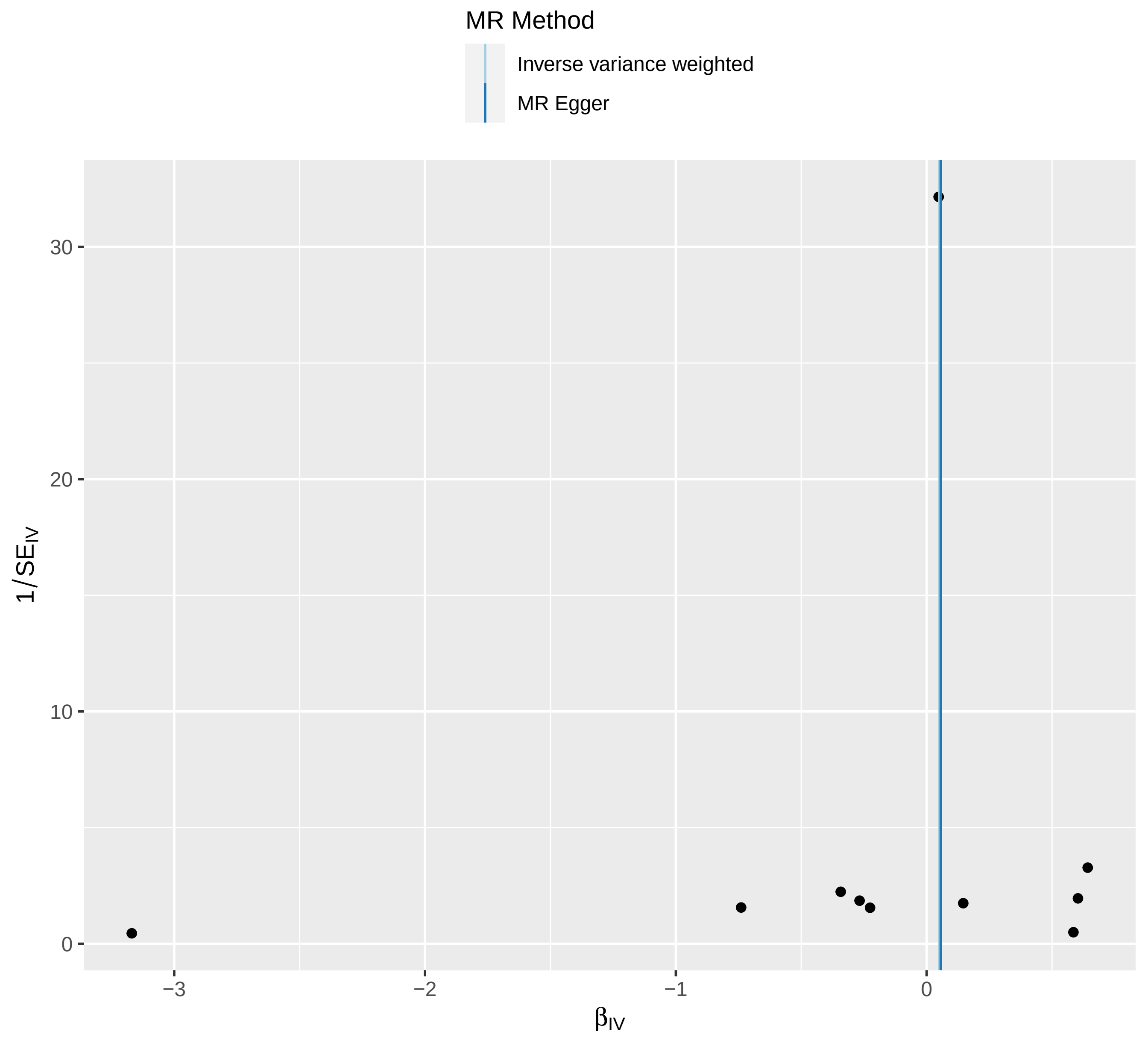

MR Method
Inverse variance weighted
MR Egger
30
20
10
0
1/SE_IV
−3
−2
−1
0
β_IV

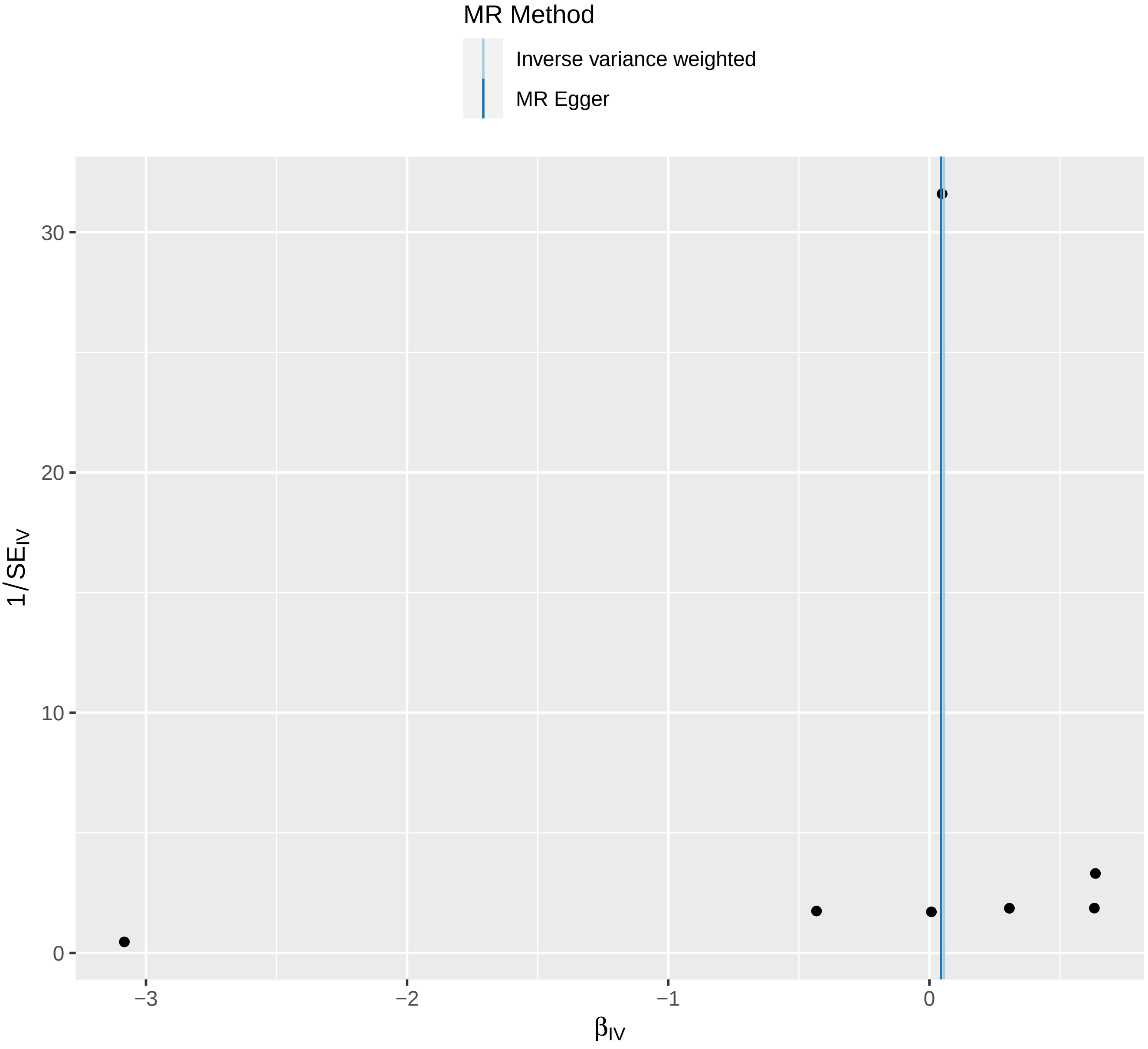

MR Method
Inverse variance weighted
MR Egger
30
20
10
0
1/SE_IV
−3
−2
−1
0
β_IV

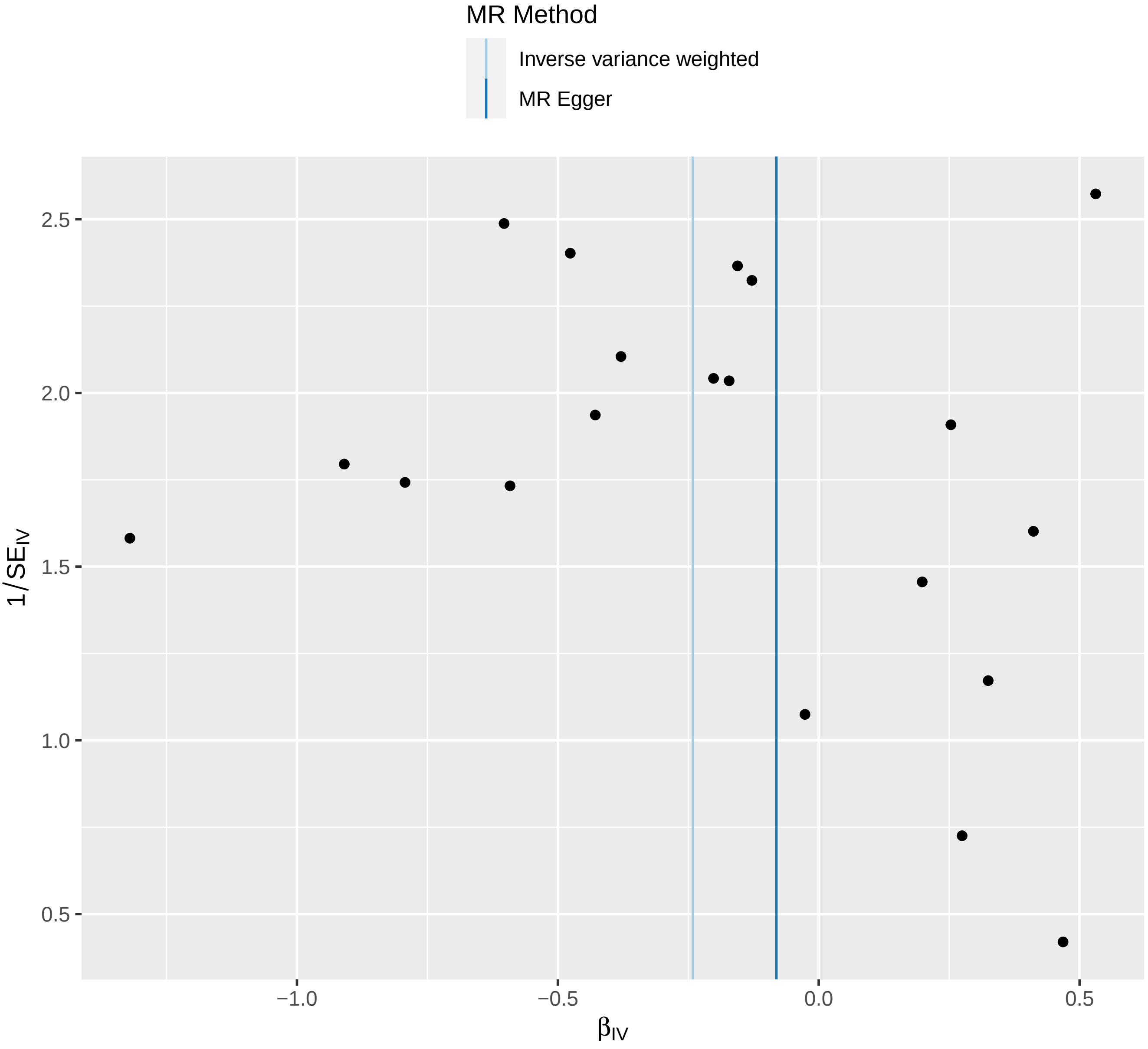
MR Method
Inverse variance weighted
MR Egger
2.5
2.0
1.5
1.0
0.5
1/SE$_{IV}$
−1.0
−0.5
0.0
0.5
β$_{IV}$

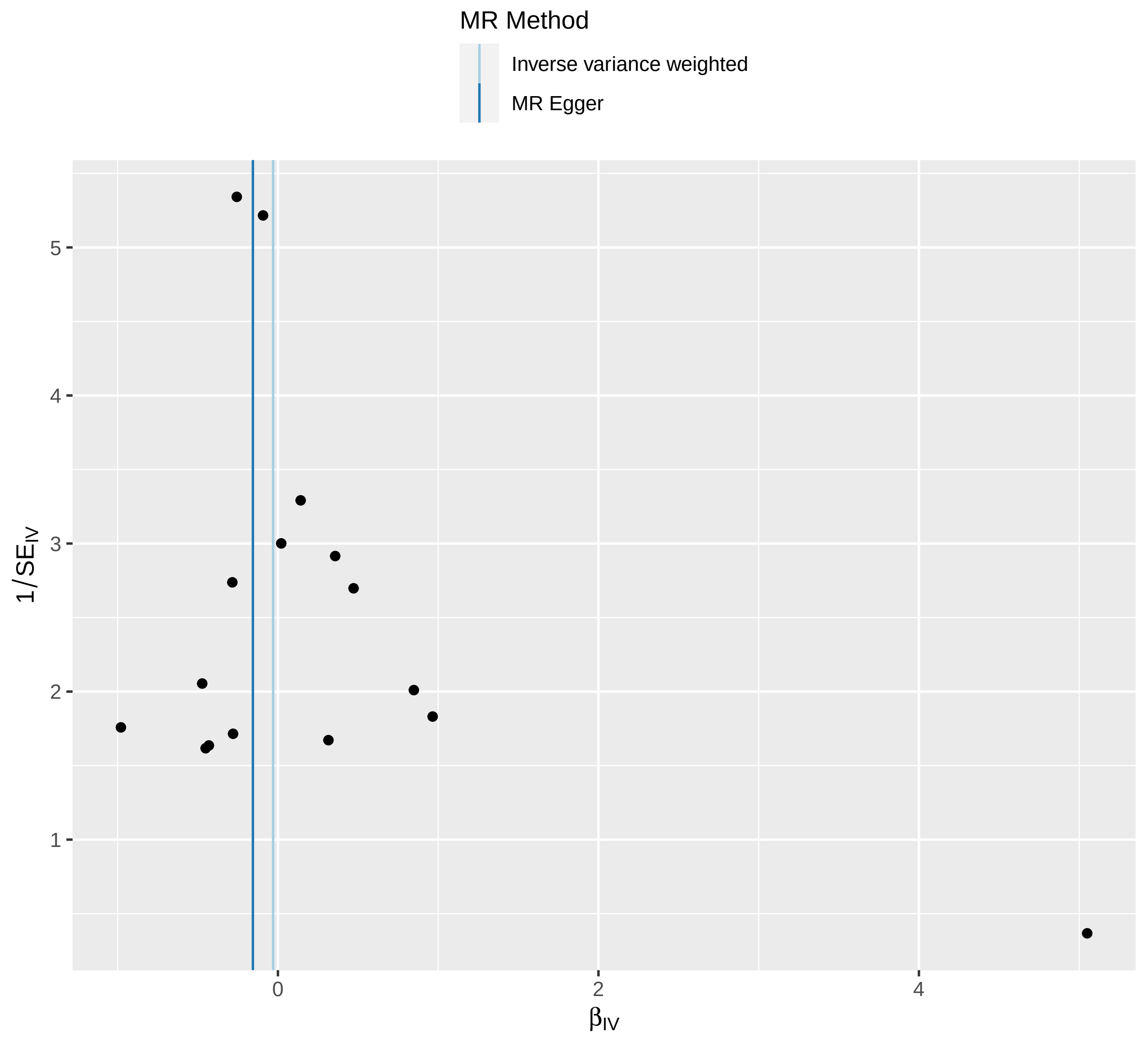
MR Method
Inverse variance weighted
MR Egger
1/SE$_{IV}$
β$_{IV}$
5
4
3
2
1
0
2
4

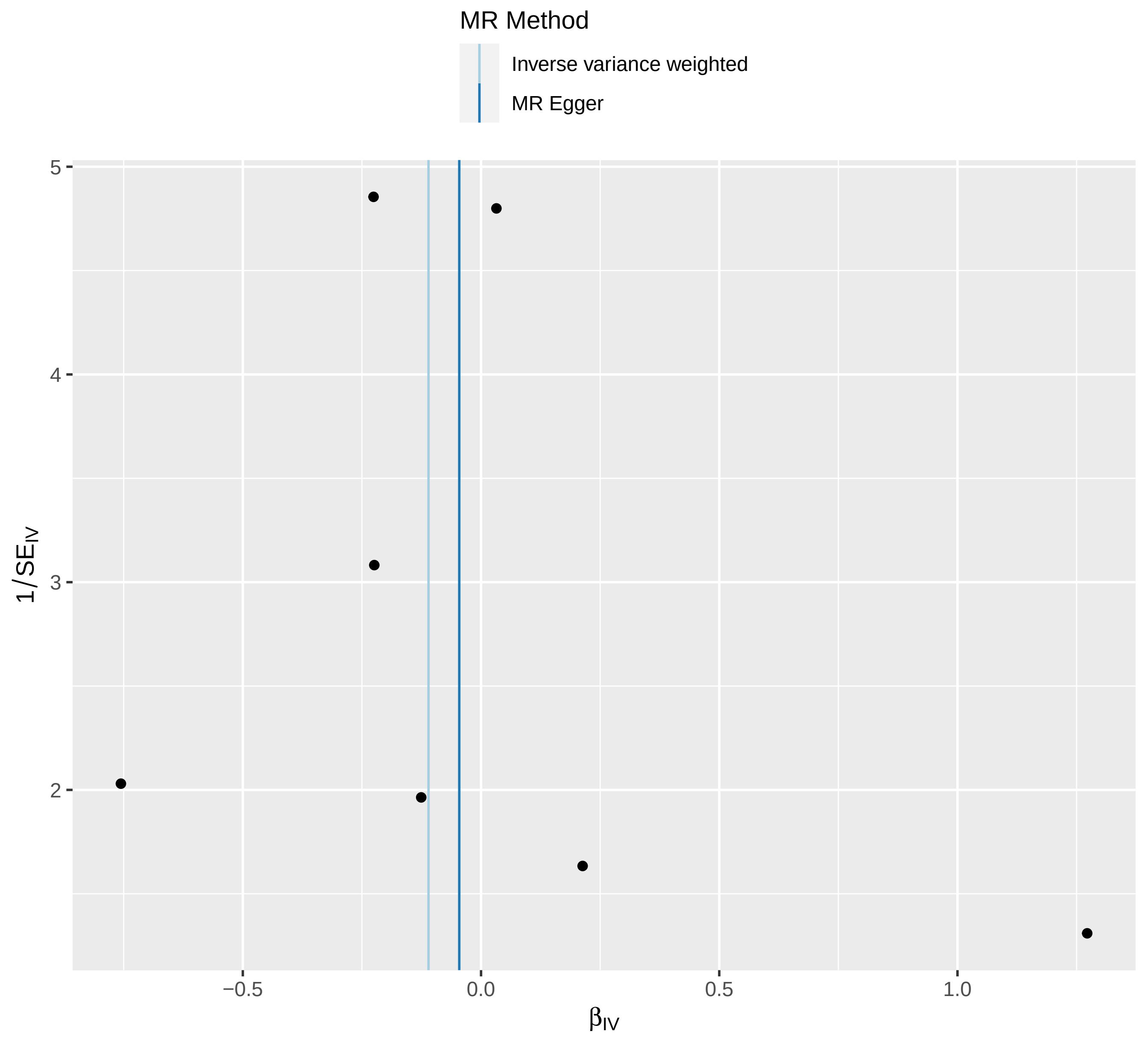
MR Method
Inverse variance weighted
MR Egger
5
4
3
2
1/SE_IV
−0.5
0.0
0.5
1.0
β_IV

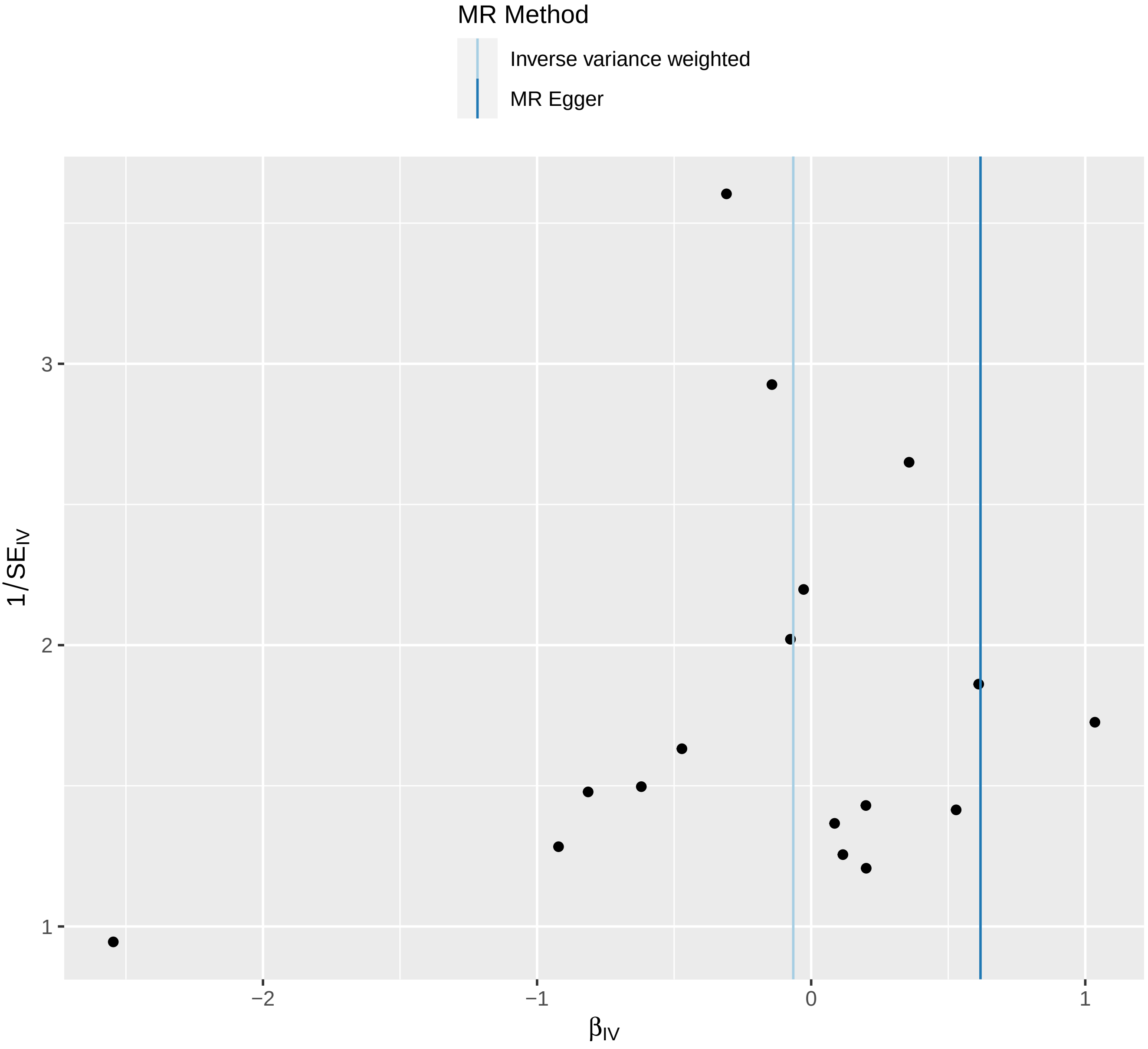
MR Method
Inverse variance weighted
MR Egger
1/SE_IV
3
2
1
−2
−1
0
1
β_IV

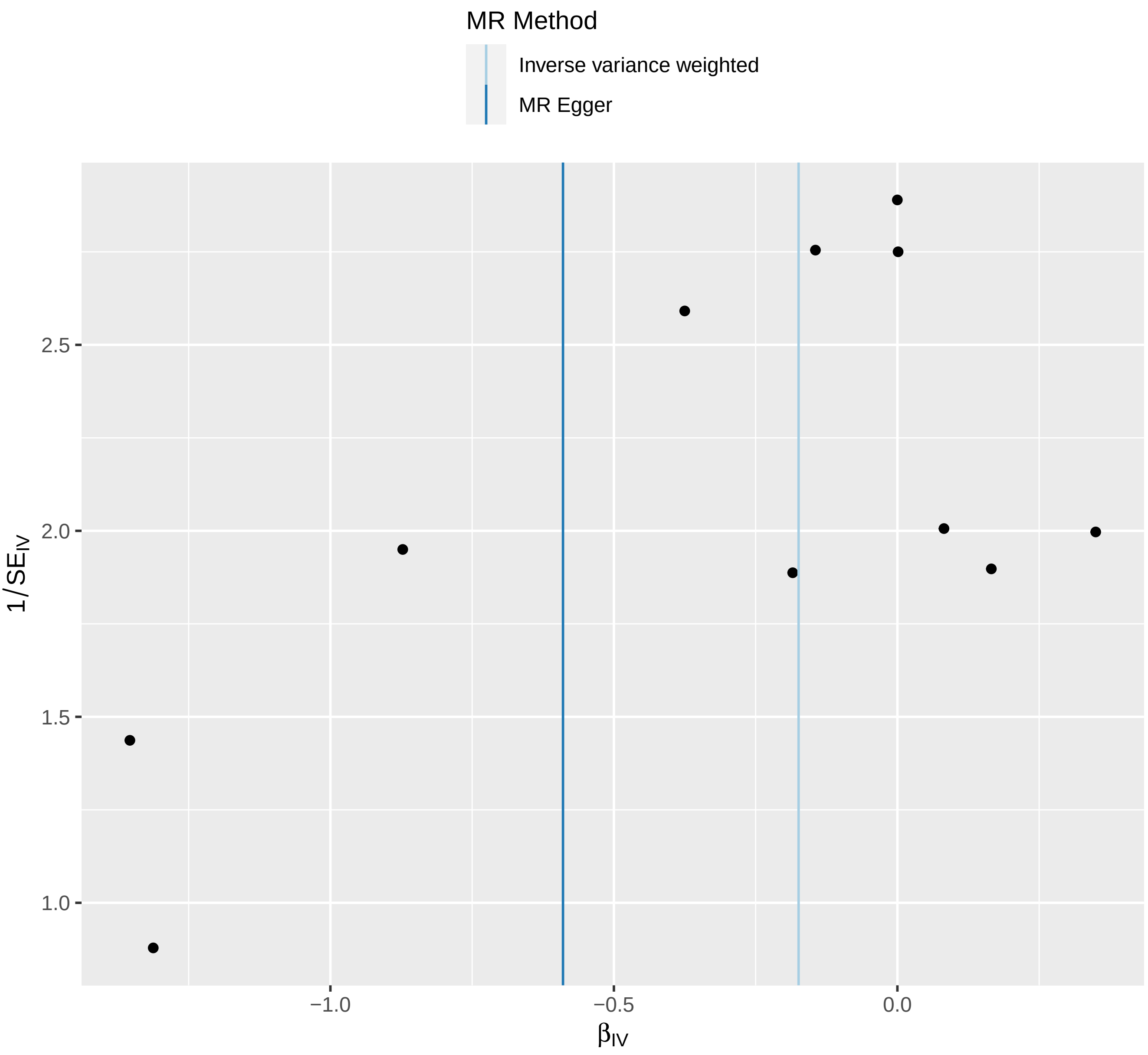

MR Method
Inverse variance weighted
MR Egger
2.5
2.0
1.5
1.0
1/SE_IV
−1.0
−0.5
0.0
β_IV

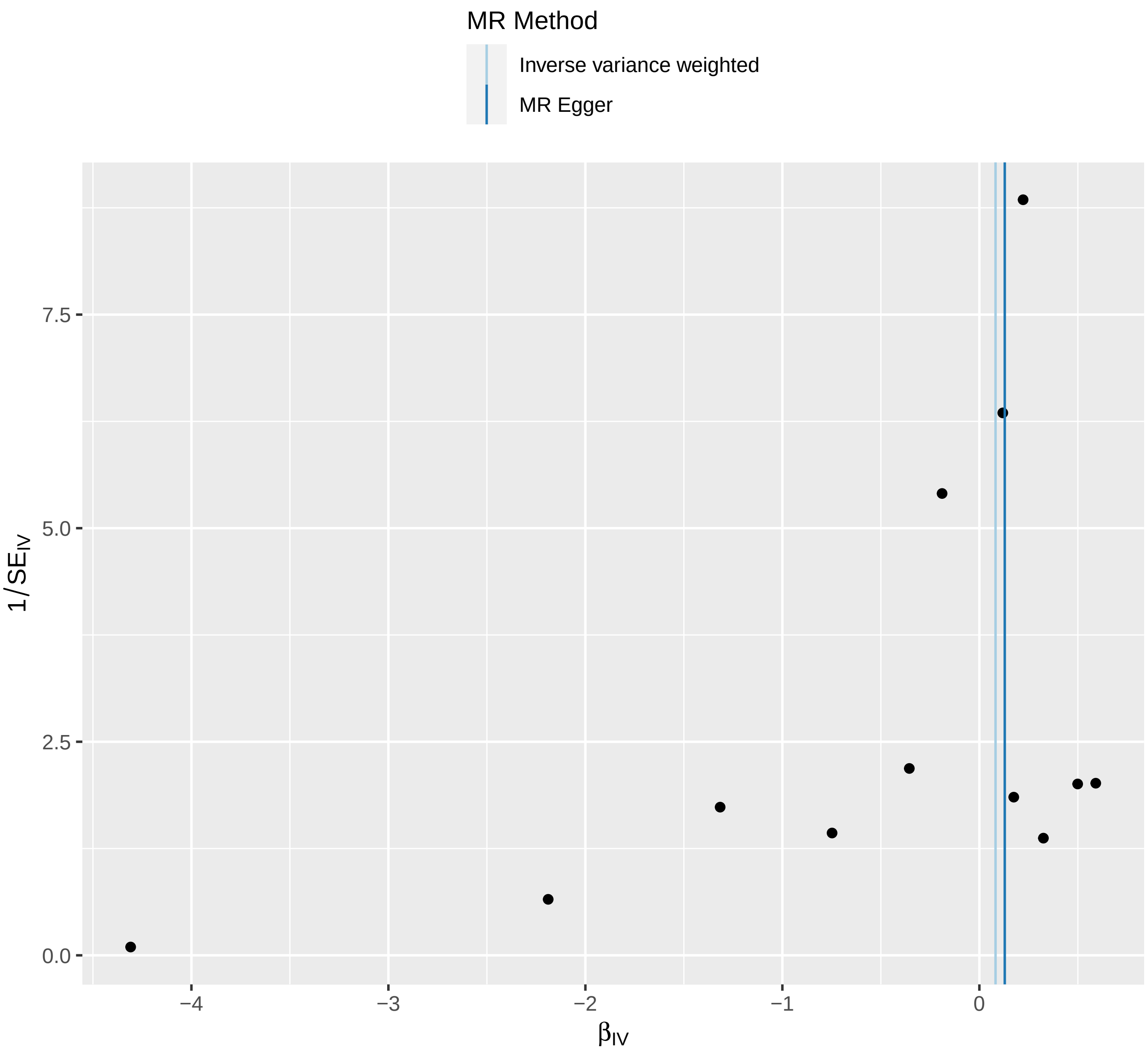
MR Method
Inverse variance weighted
MR Egger
1/SE_{IV}
0.0
2.5
5.0
7.5
−4
−3
−2
−1
0
β_{IV}

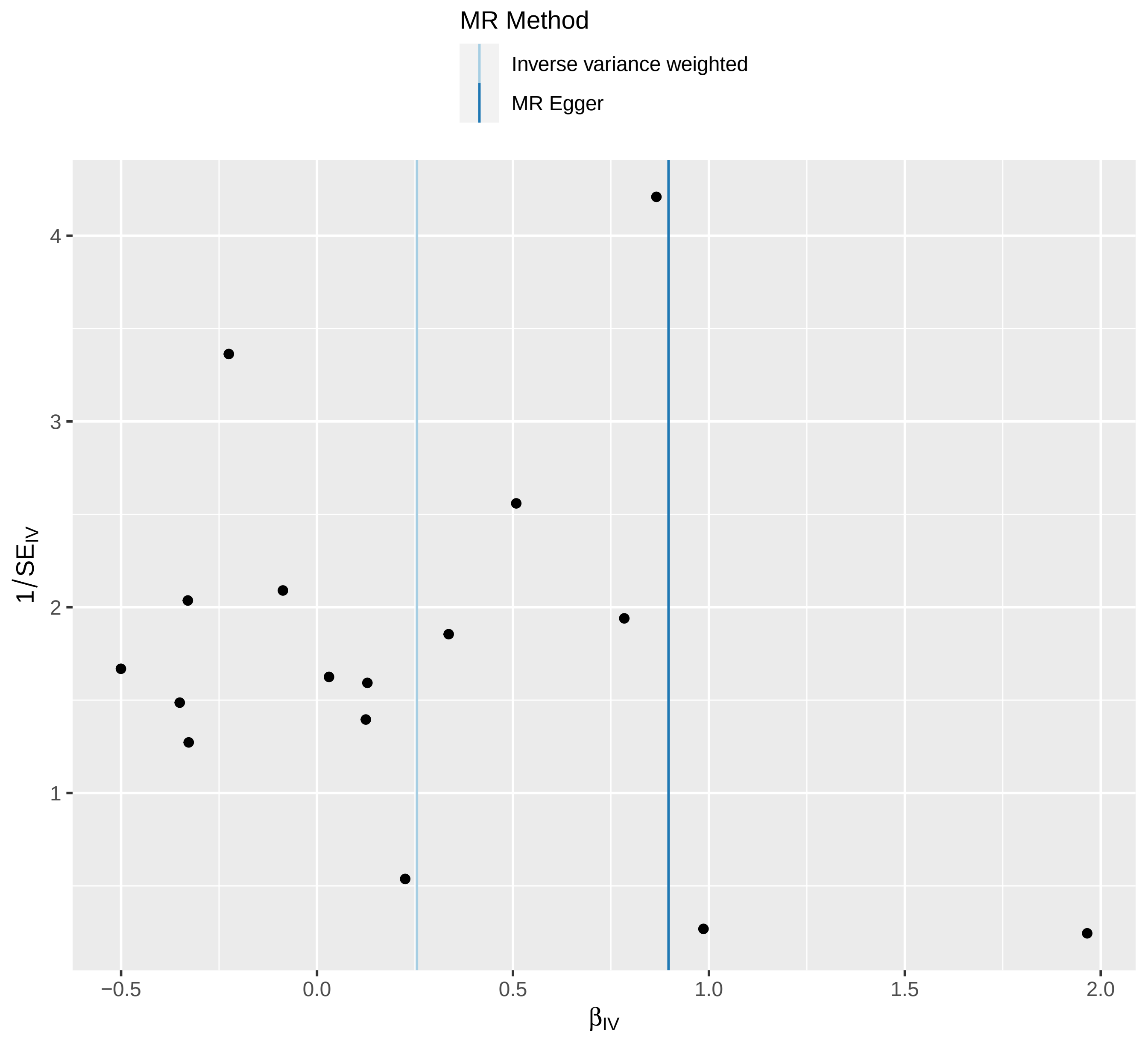
MR Method
Inverse variance weighted
MR Egger
4
3
2
1
1/SE$_{IV}$
−0.5
0.0
0.5
1.0
1.5
2.0
β$_{IV}$

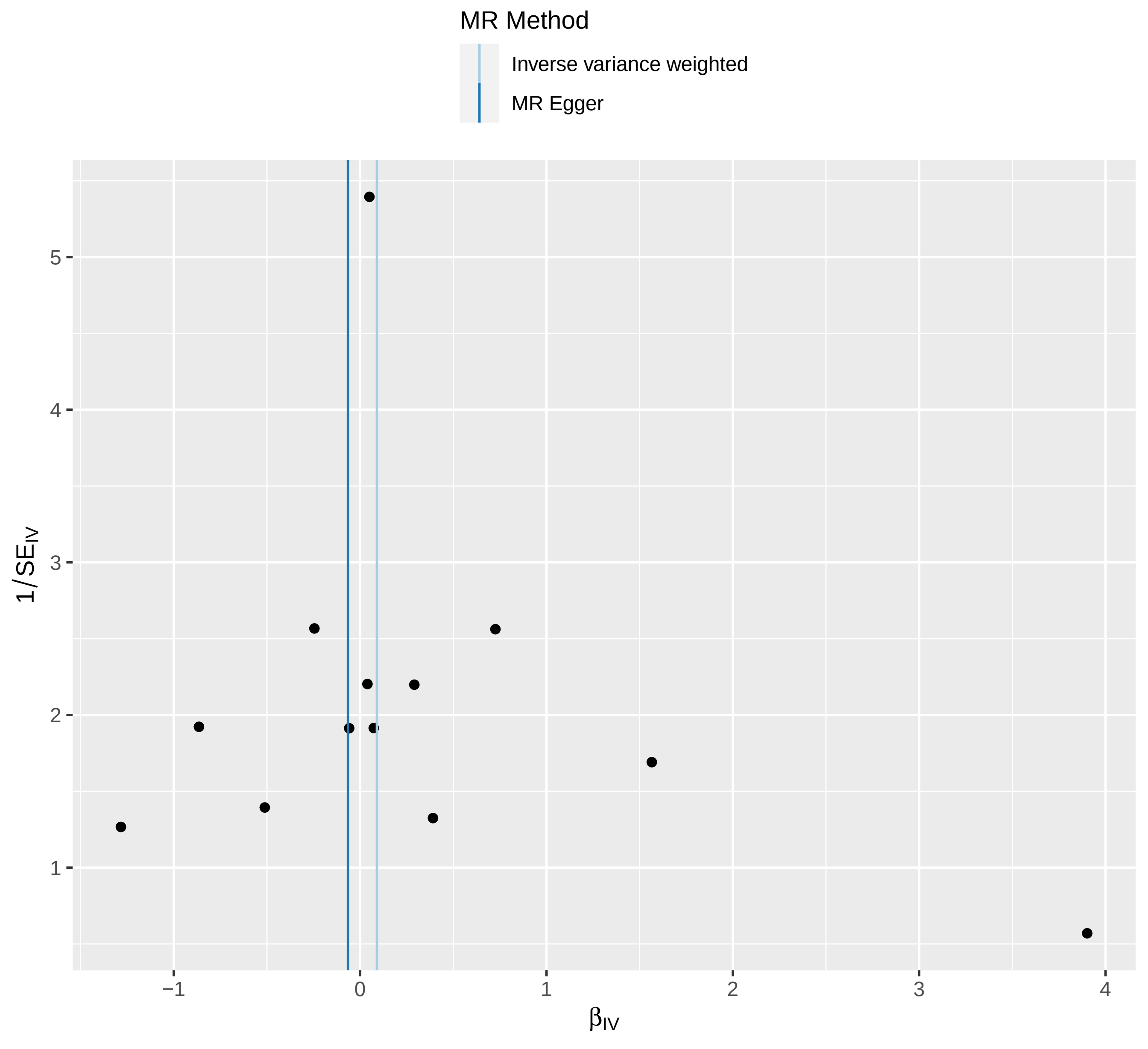

MR Method
Inverse variance weighted
MR Egger
1/SE_IV
β_IV
5
4
3
2
1
−1
0
1
2
3
4

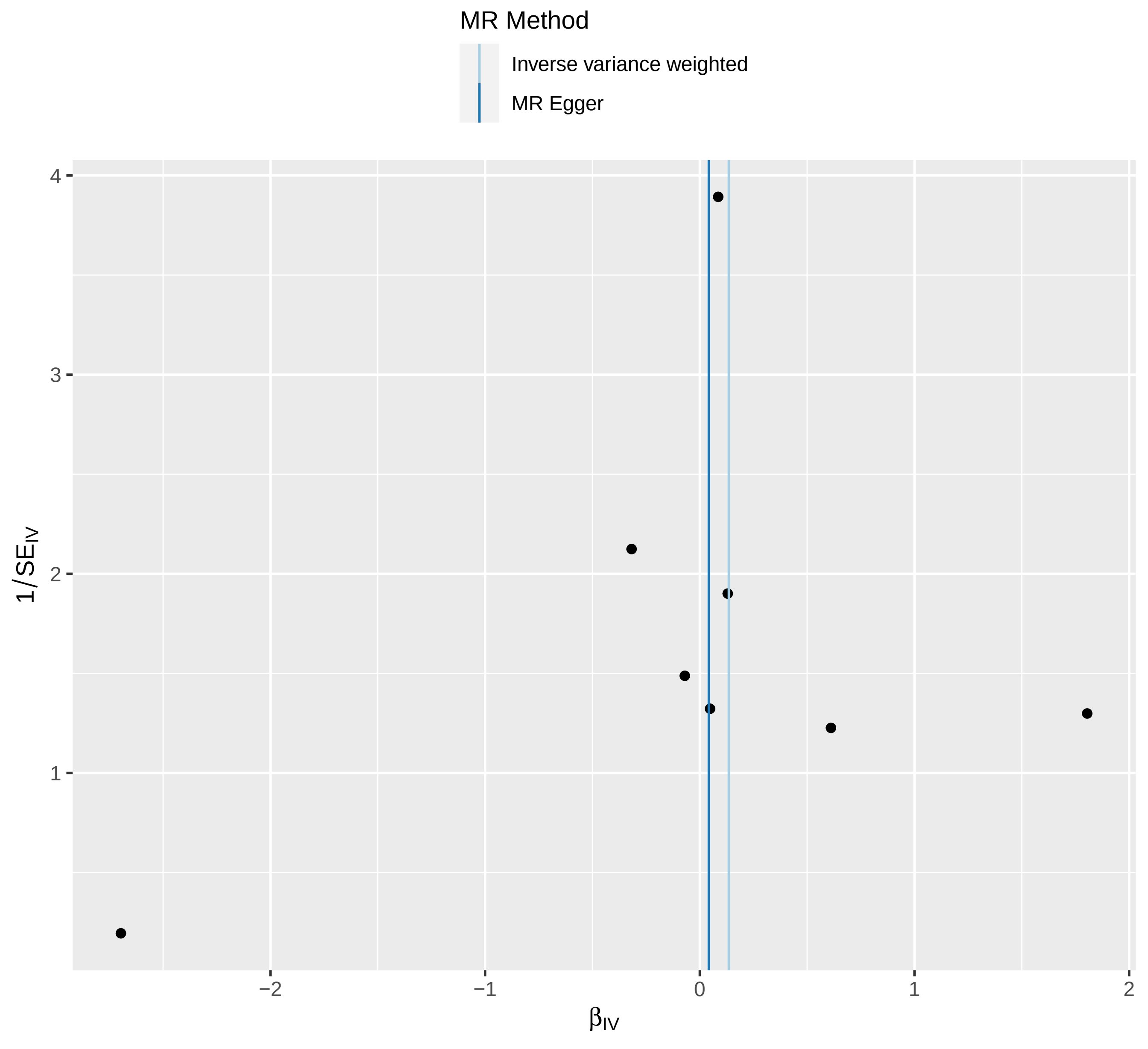
MR Method
Inverse variance weighted
MR Egger
1/SE$_{IV}$
4
3
2
1
−2
−1
0
1
2
β$_{IV}$

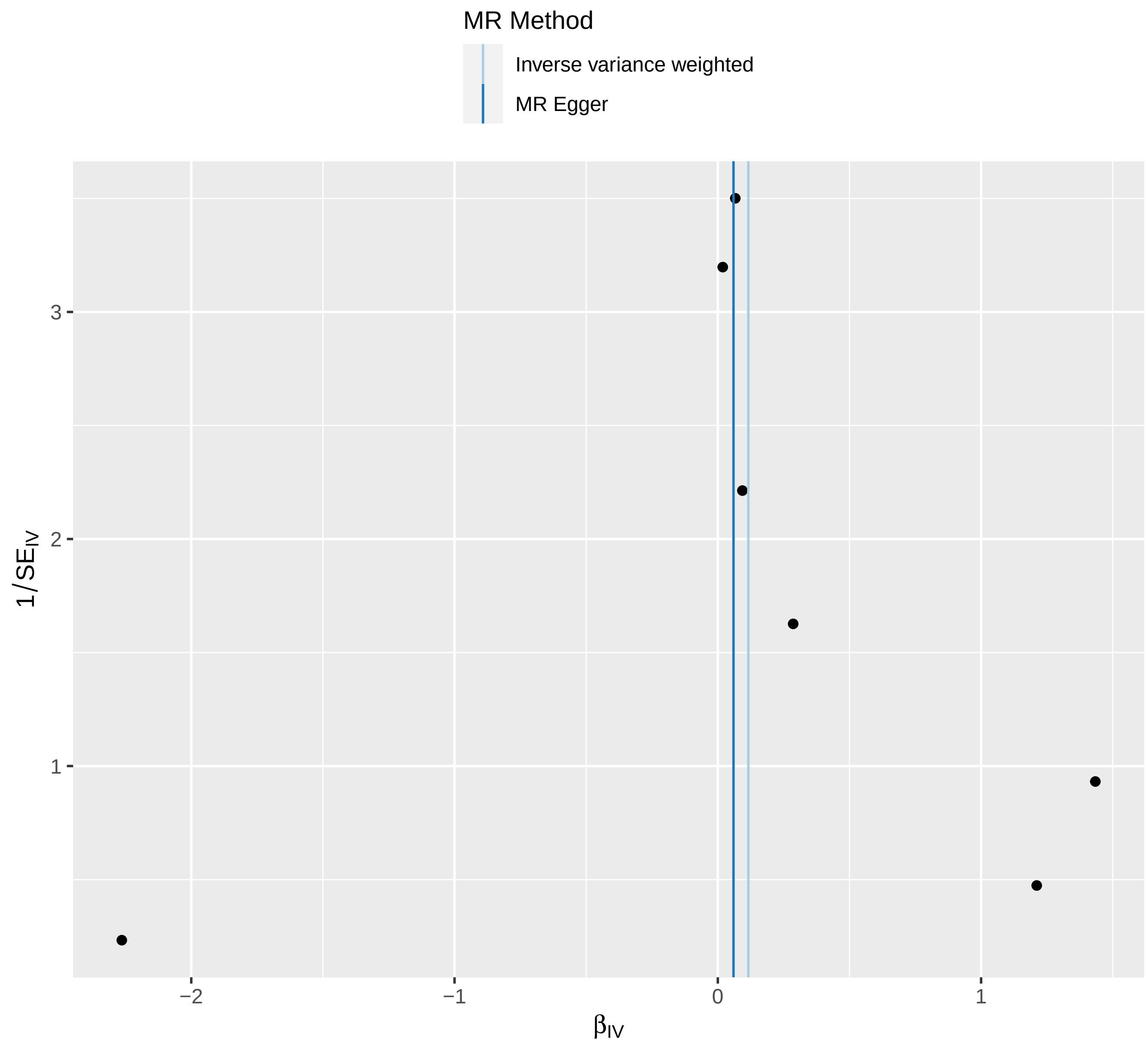
MR Method
Inverse variance weighted
MR Egger
1/SE$_{IV}$
3
2
1
−2
−1
0
1
β$_{IV}$

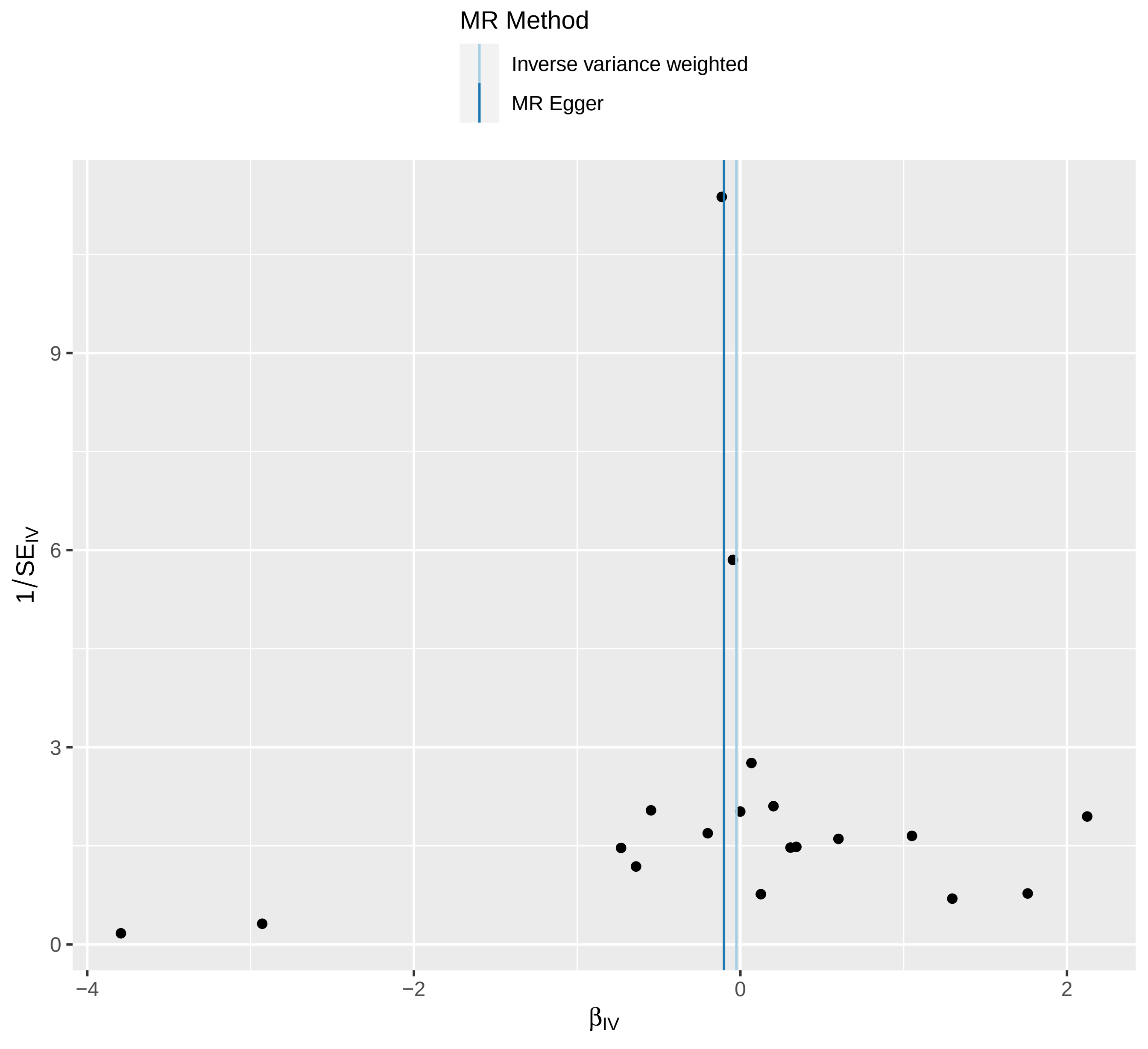

MR Method
Inverse variance weighted
MR Egger
9
6
3
0
1/SE_IV
−4
−2
0
2
β_IV

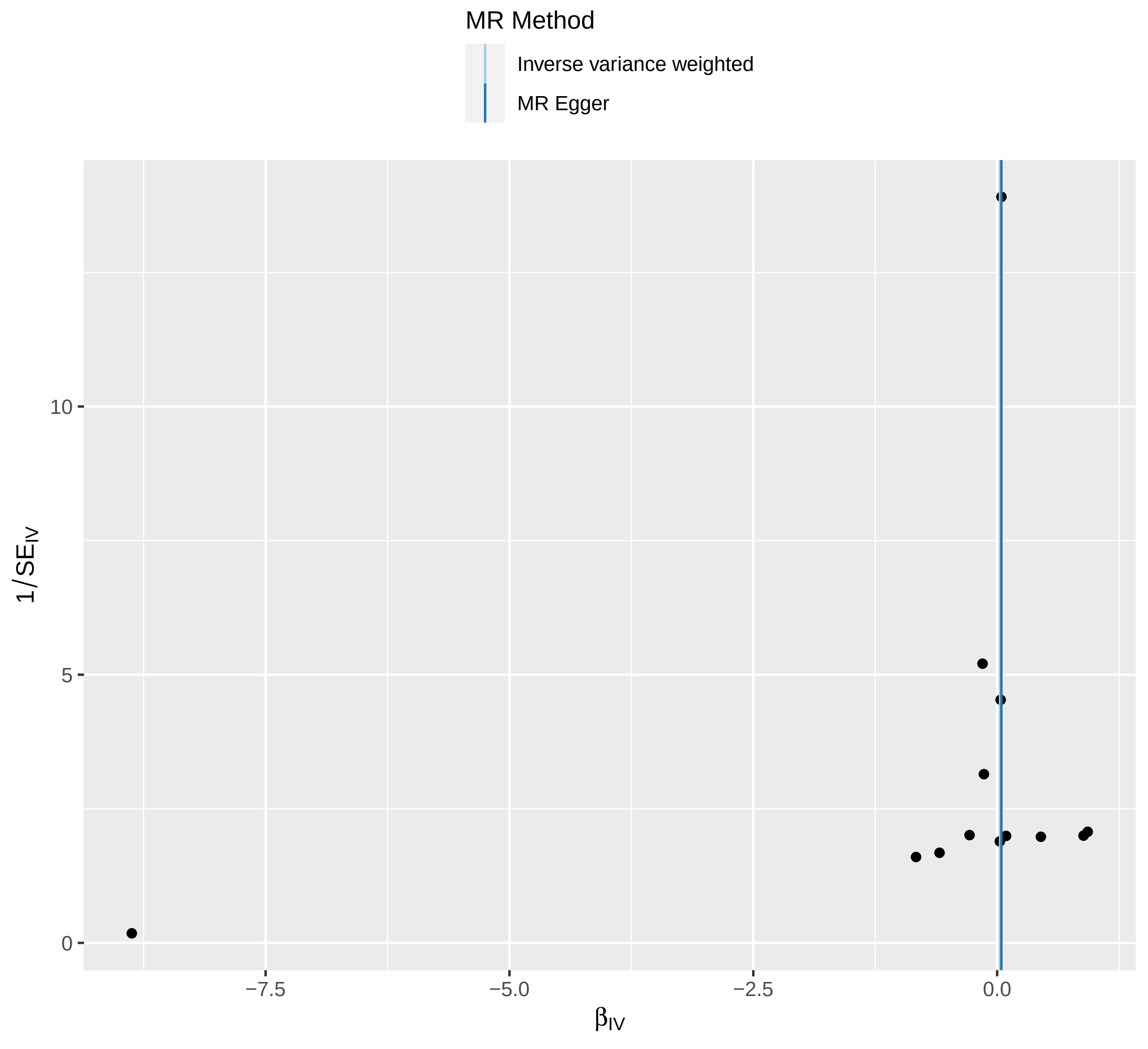

MR Method
Inverse variance weighted
MR Egger
1/SE_{IV}
10
5
0
−7.5
−5.0
−2.5
0.0
β_{IV}

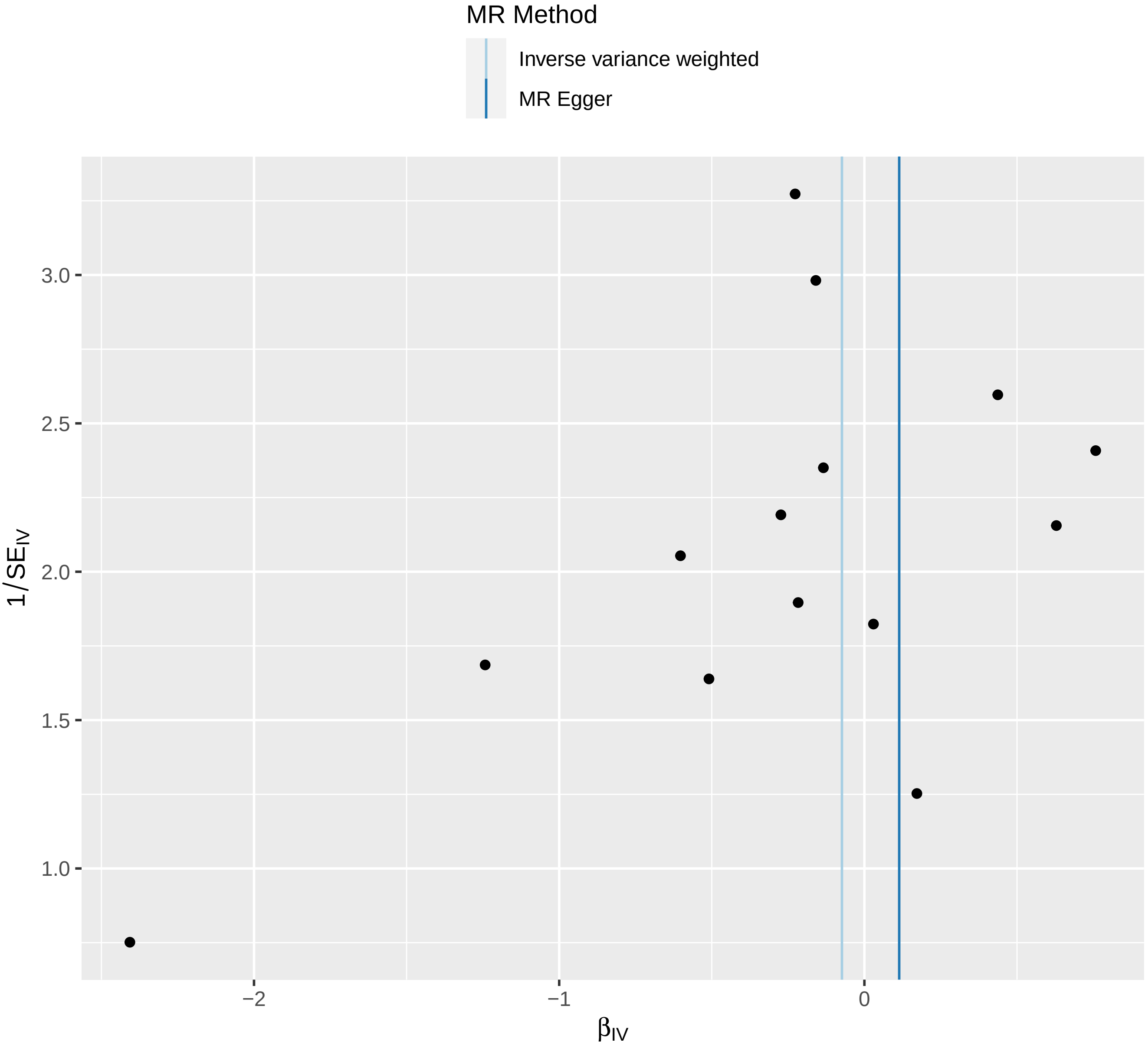
MR Method
Inverse variance weighted
MR Egger
3.0
2.5
2.0
1.5
1.0
$1/SE_{IV}$
−2
−1
0
β_{IV}

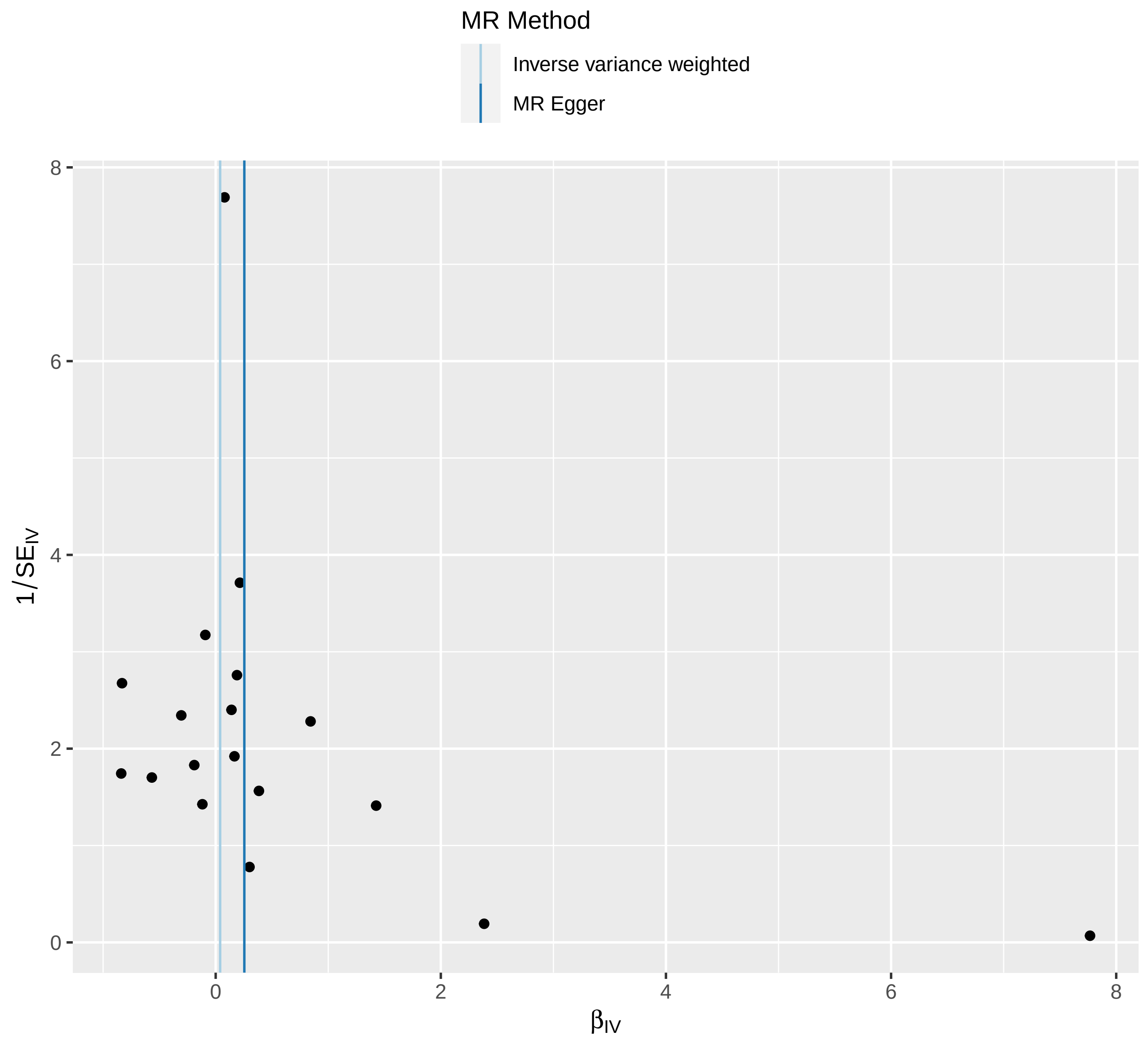
MR Method
Inverse variance weighted
MR Egger
1/SE$_{IV}$
β$_{IV}$
0
2
4
6
8
0
2
4
6
8

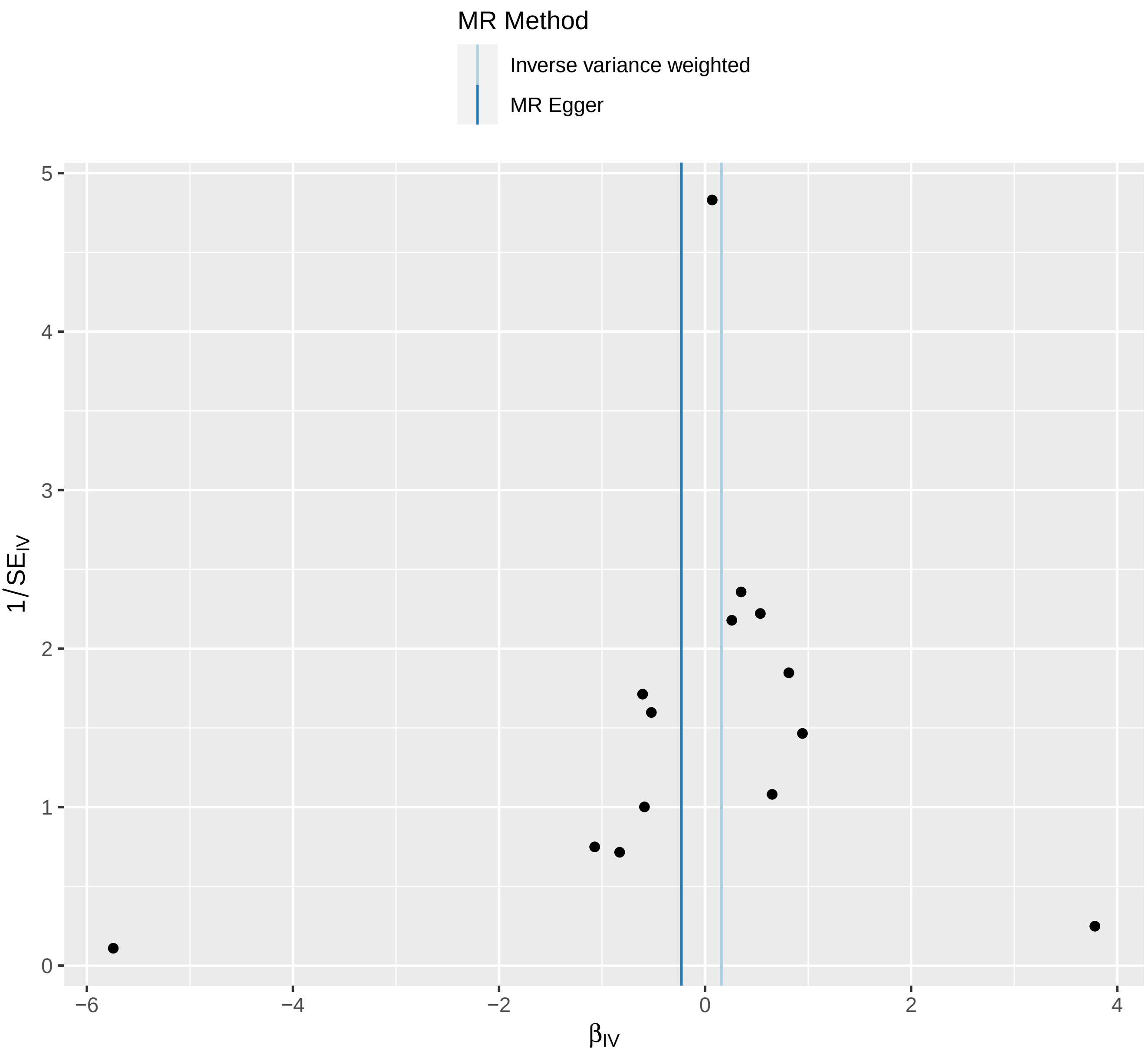
MR Method
Inverse variance weighted
MR Egger
5
4
3
2
1
0
$1/SE_{IV}$
−6
−4
−2
0
2
4
β_{IV}

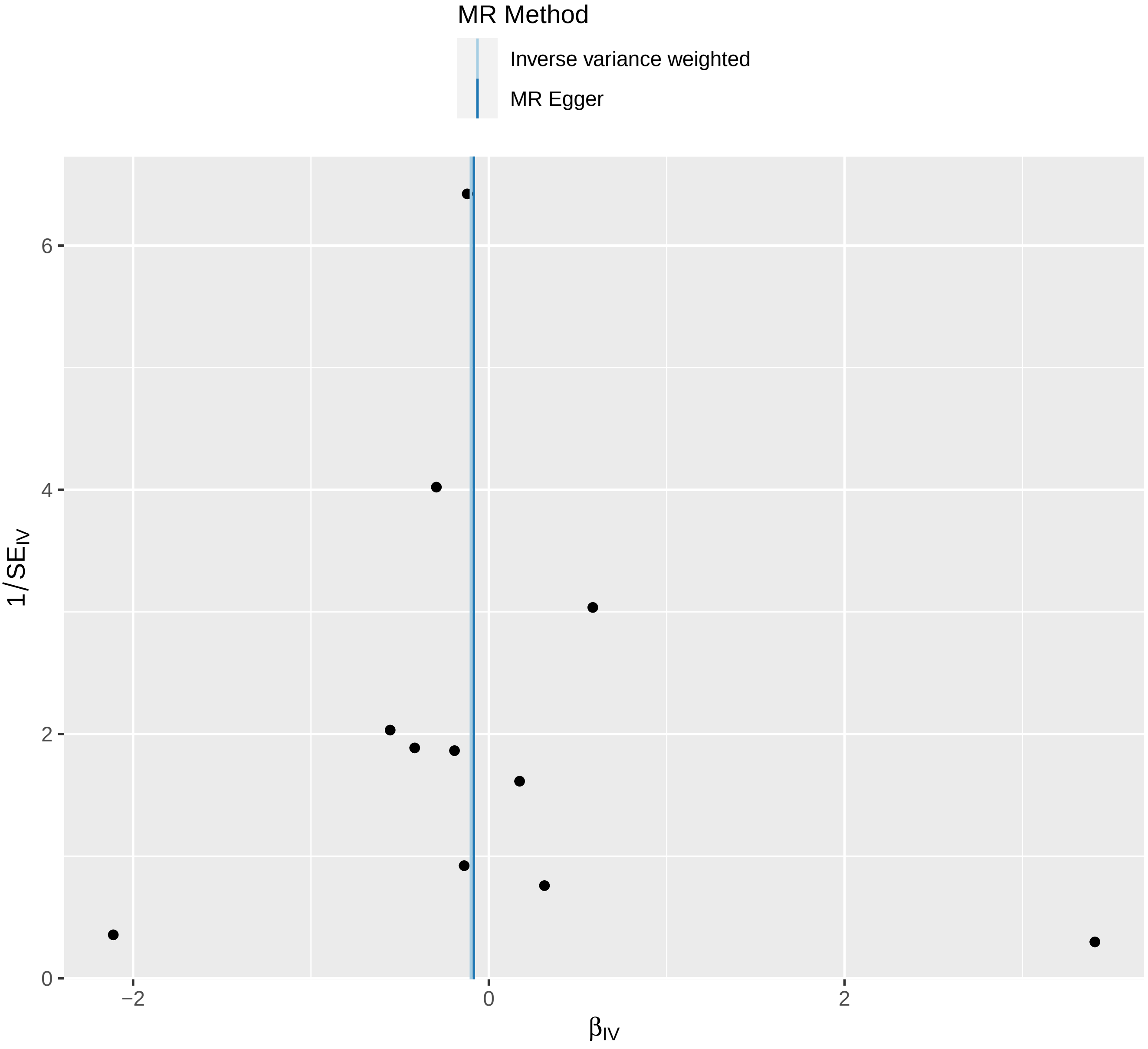

MR Method
Inverse variance weighted
MR Egger
6
4
2
0
1/SE_IV
−2
0
2
β_IV

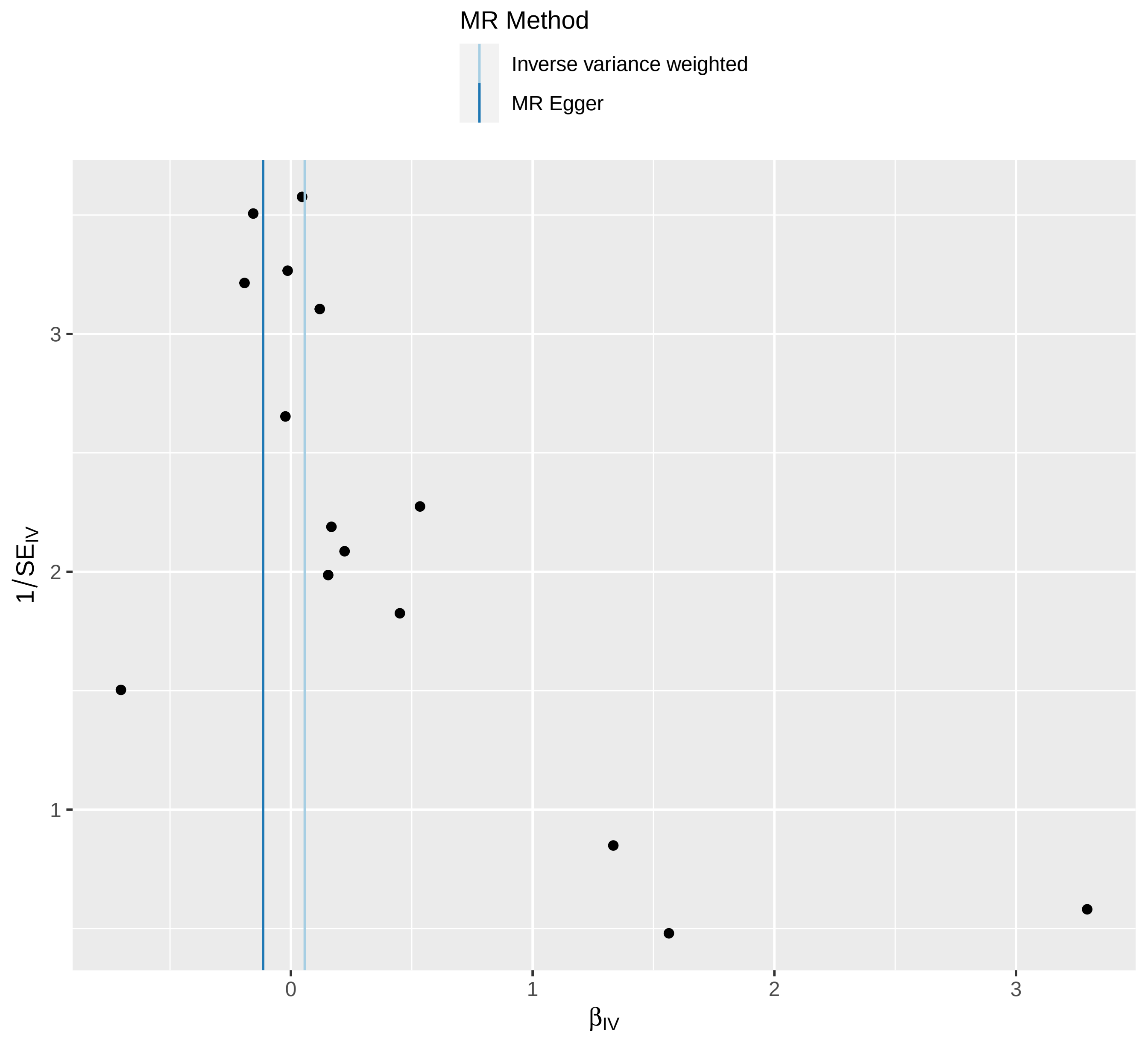

MR Method
Inverse variance weighted
MR Egger
3
2
1
0
1
2
3
$1/SE_{IV}$
β_{IV}

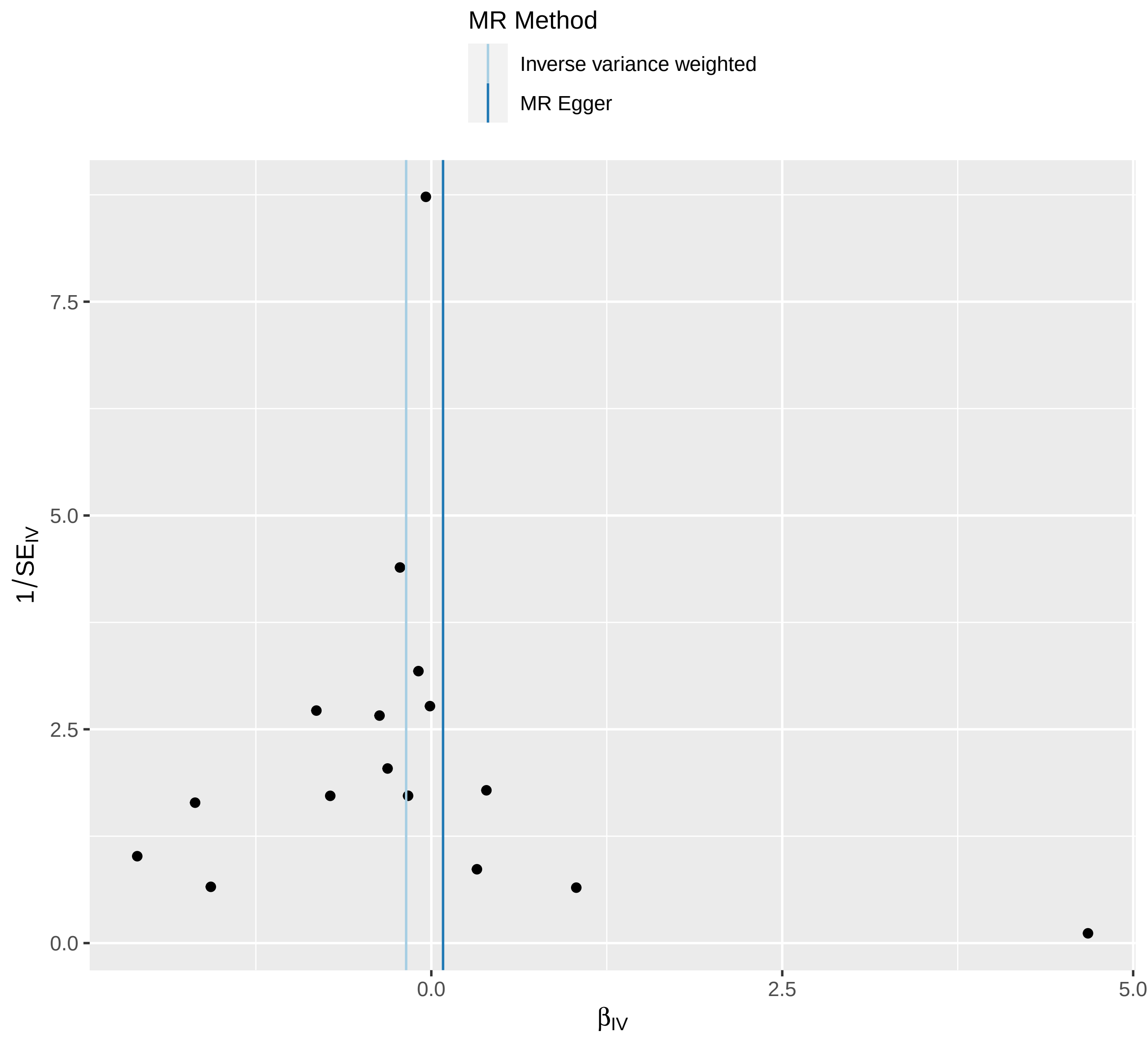
MR Method
Inverse variance weighted
MR Egger
7.5
5.0
2.5
0.0
1/SE_IV
0.0
2.5
5.0
β_IV

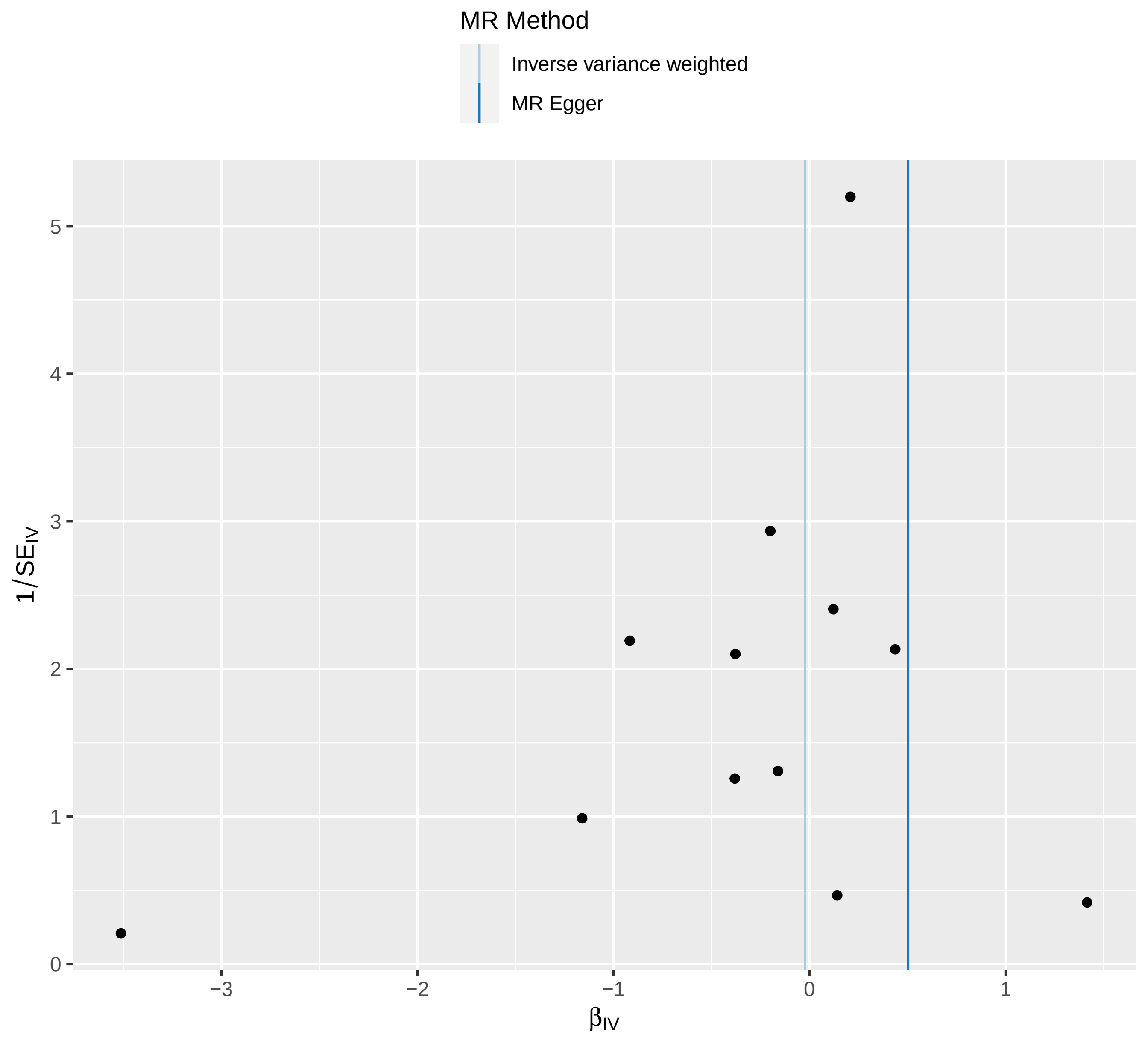
MR Method
Inverse variance weighted
MR Egger
5
4
3
2
1
0
−3
−2
−1
0
1
1/SE_IV
β_IV

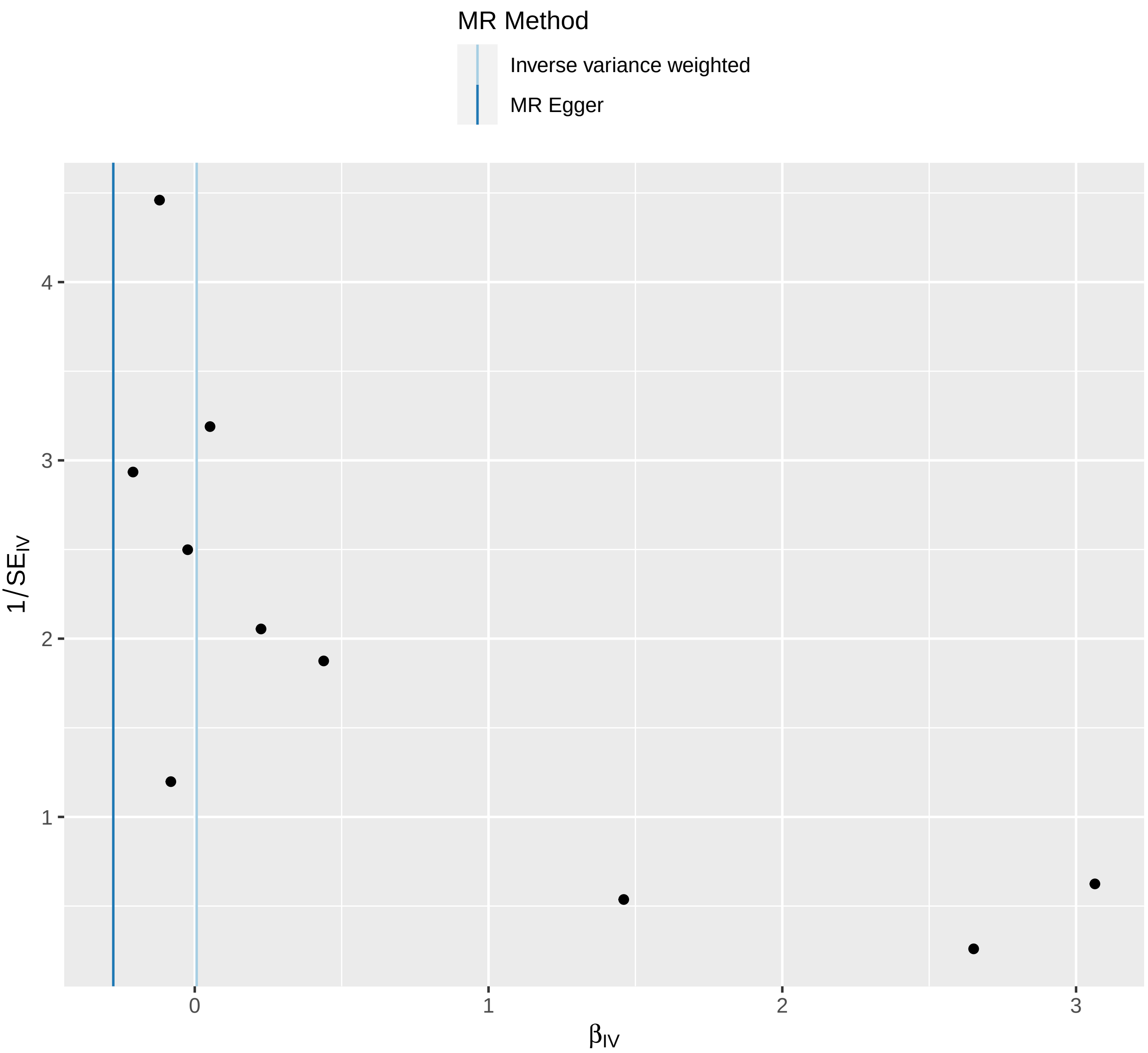

MR Method
Inverse variance weighted
MR Egger
4
3
2
1
1/SE_IV
0
1
2
3
β_IV

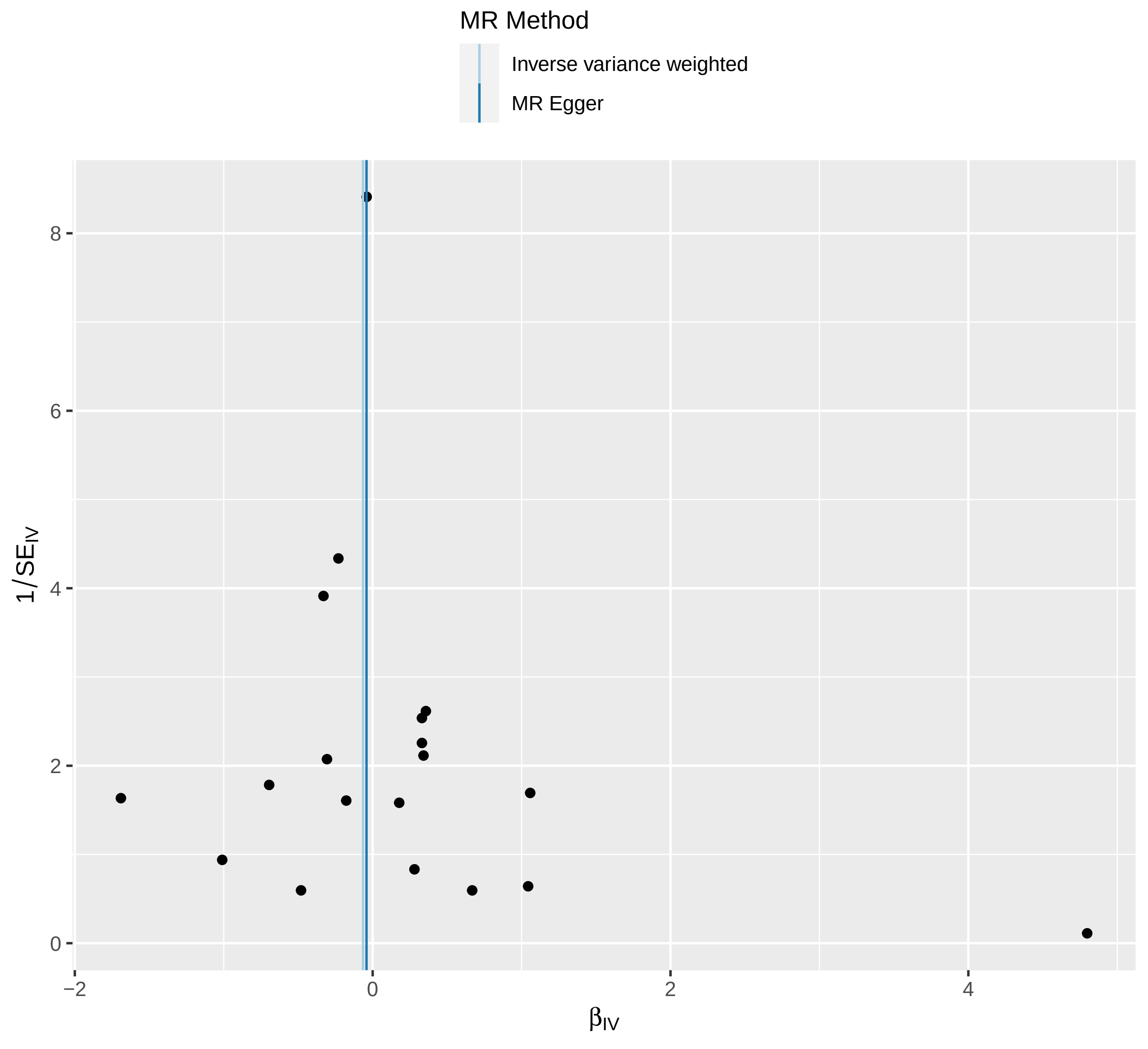

MR Method
Inverse variance weighted
MR Egger
8
6
4
2
0
1/SE$_{IV}$
−2
0
2
4
β$_{IV}$

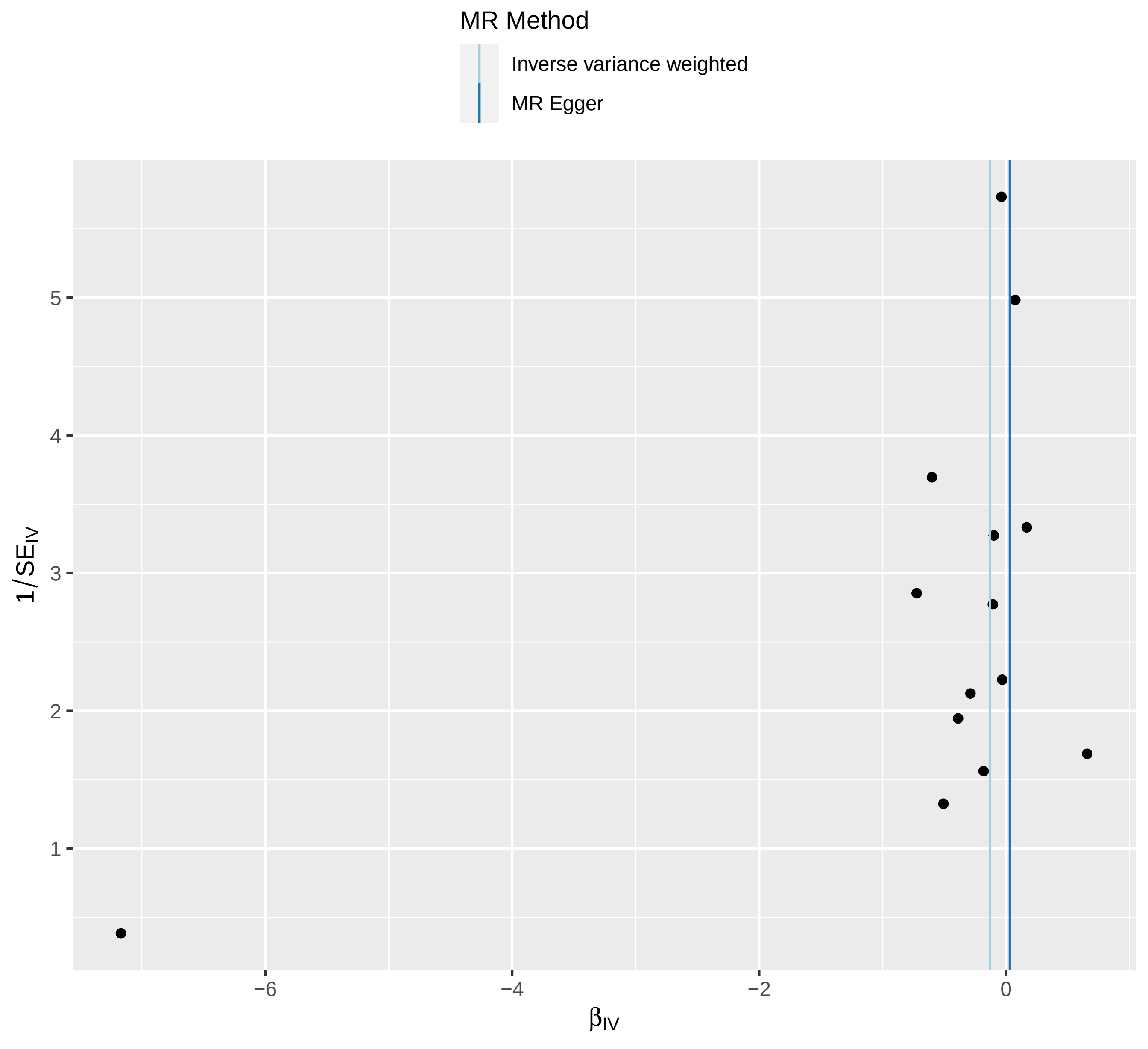
MR Method
Inverse variance weighted
MR Egger
1/SE_IV
β_IV
−6
−4
−2
0
1
2
3
4
5

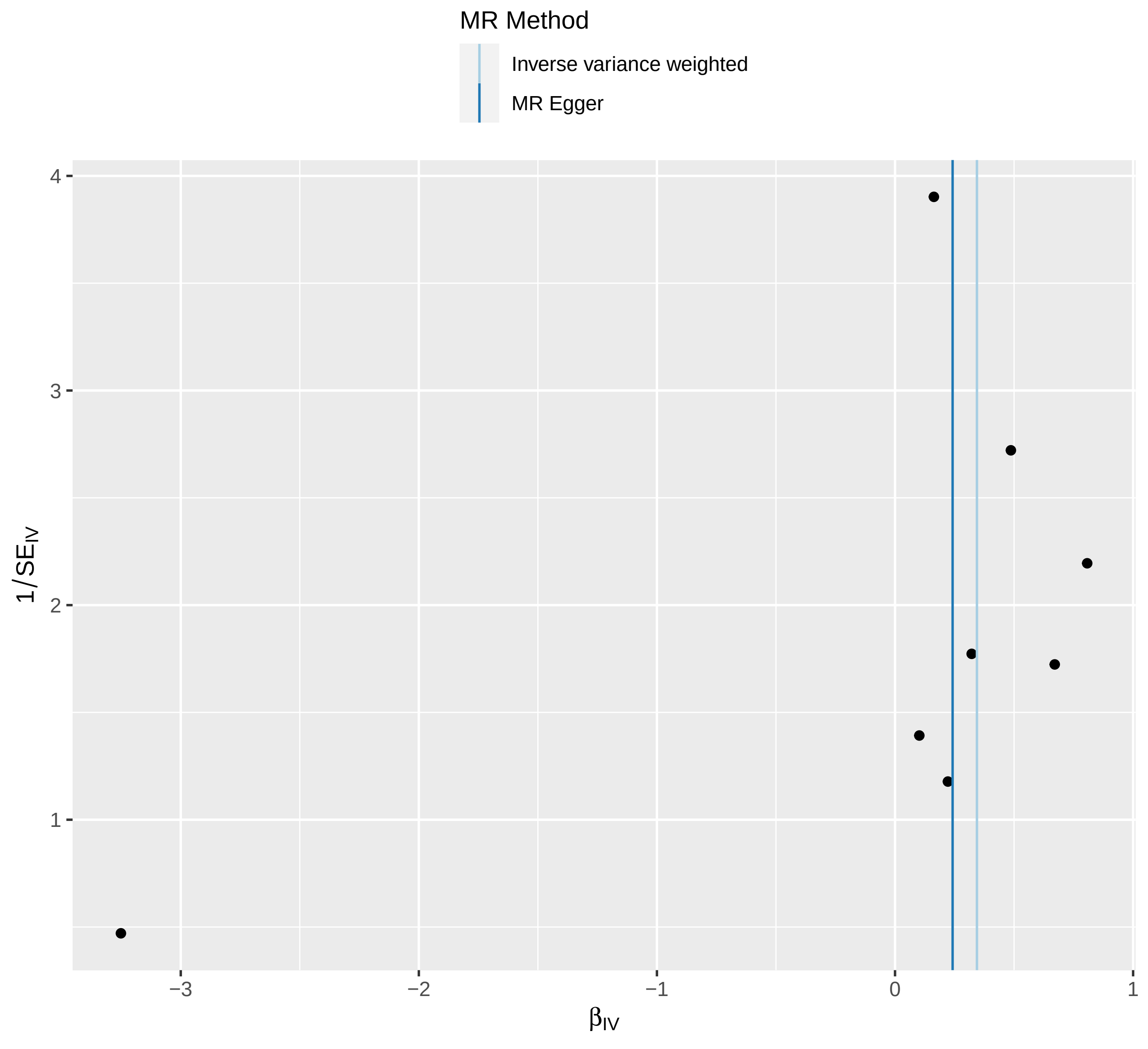
MR Method
Inverse variance weighted
MR Egger
4
3
2
1
1/SE_IV
−3
−2
−1
0
1
β_IV

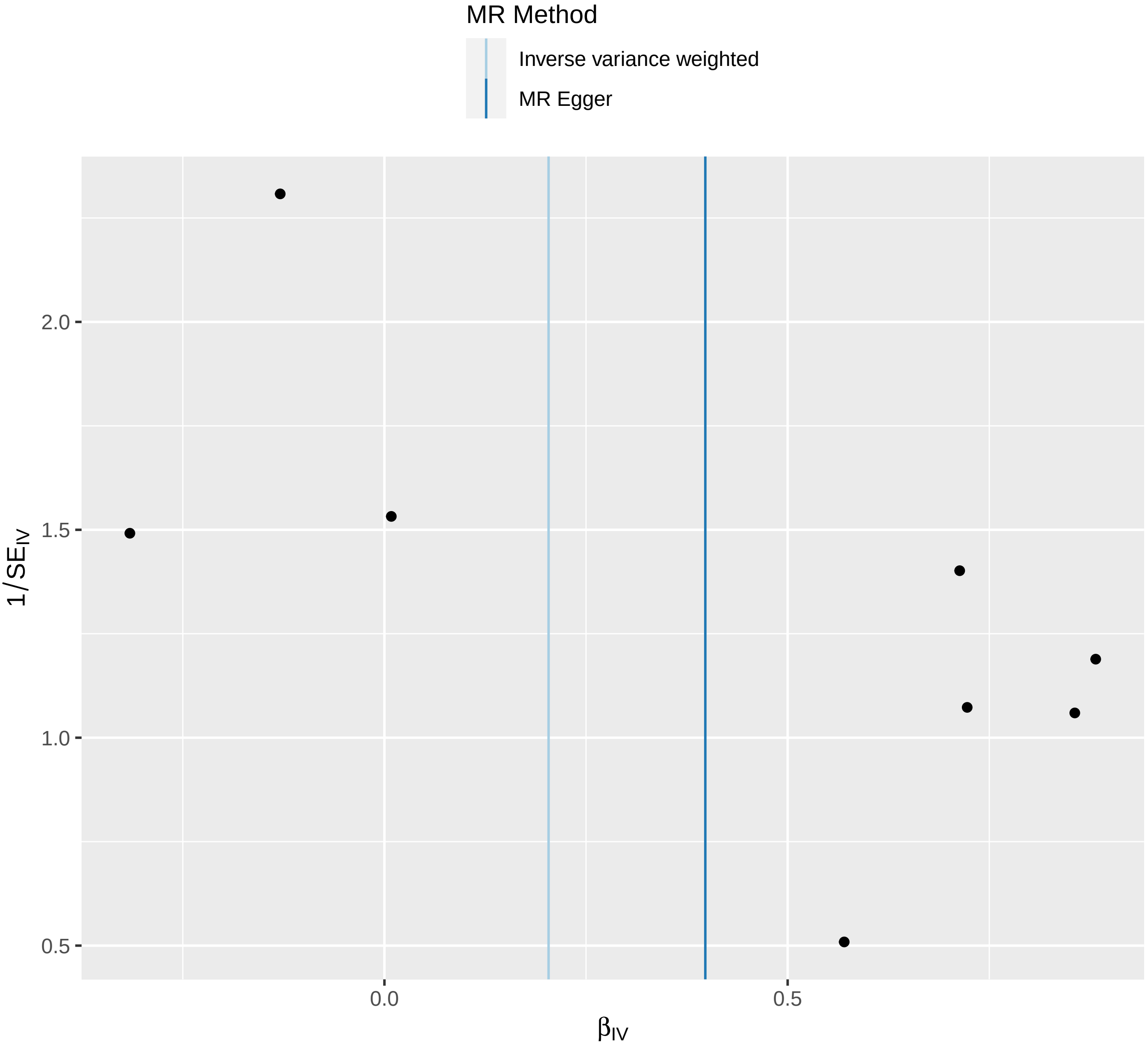

MR Method
Inverse variance weighted
MR Egger
2.0
1.5
1.0
0.5
1/SE_IV
0.0
0.5
β_IV

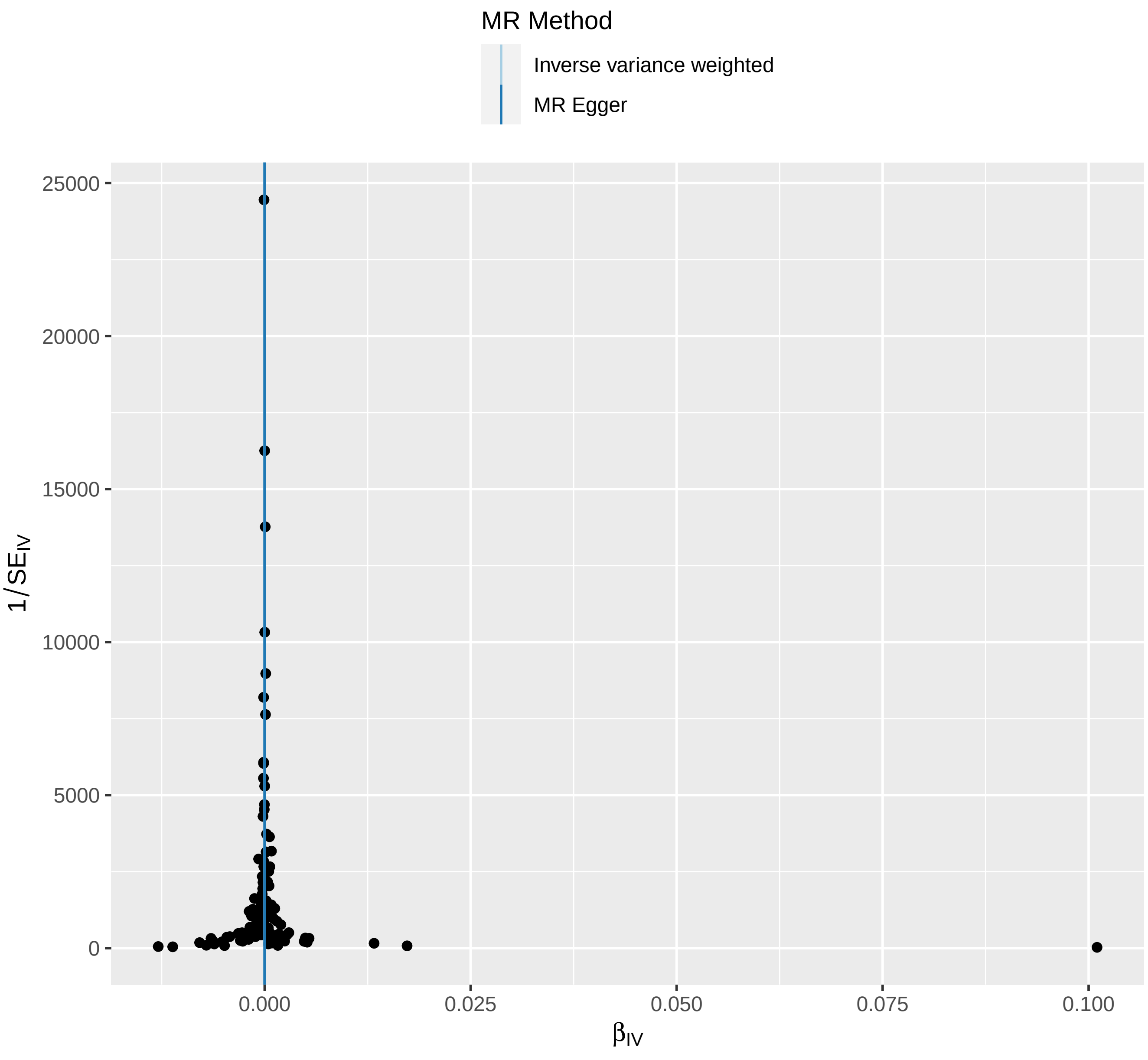

MR Method
Inverse variance weighted
MR Egger
25000
20000
15000
10000
5000
0
1/SE$_{IV}$
0.000
0.025
0.050
0.075
0.100
β$_{IV}$

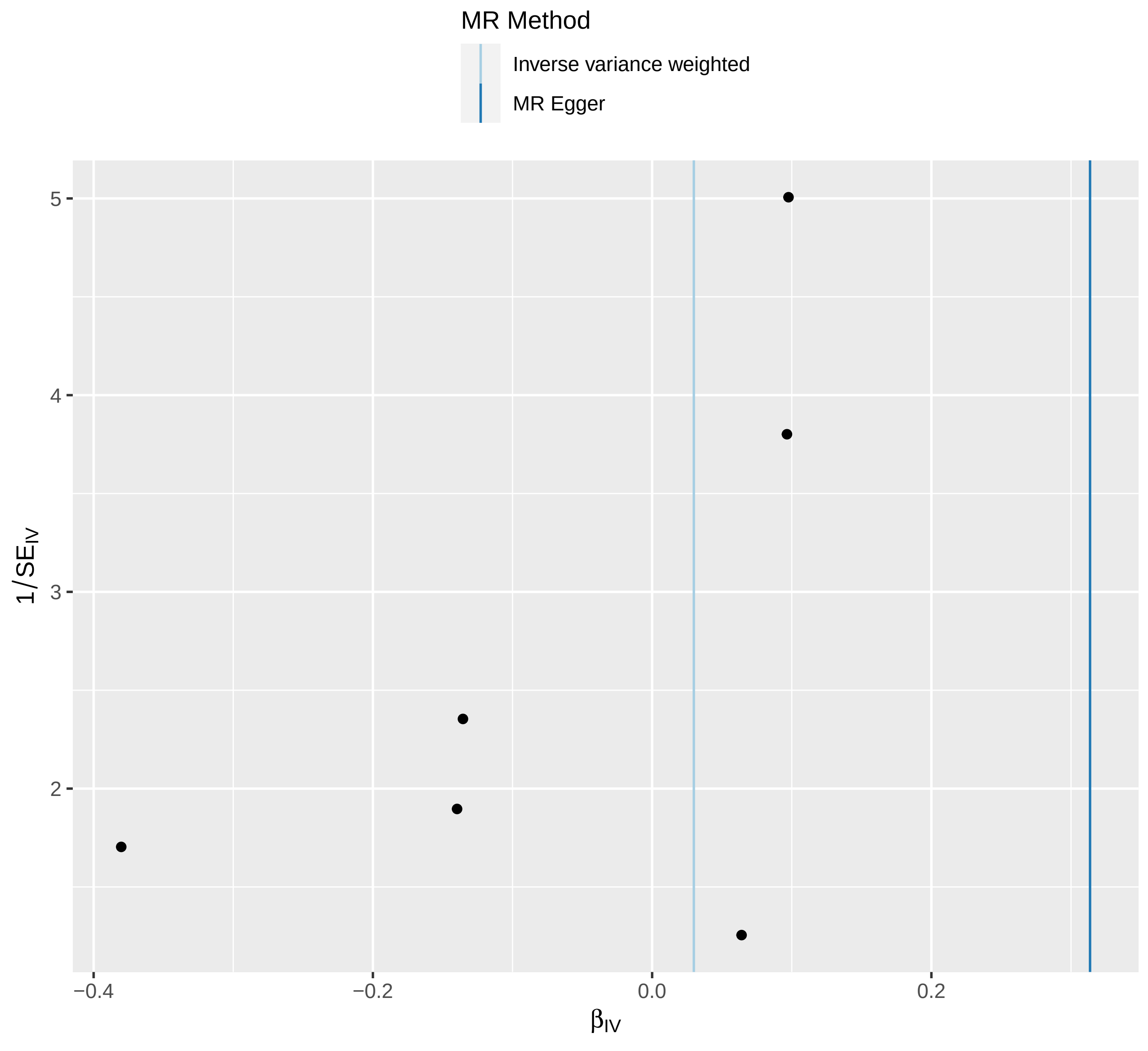
MR Method
Inverse variance weighted
MR Egger
1/SE$_{IV}$
5
4
3
2
−0.4
−0.2
0.0
0.2
β$_{IV}$

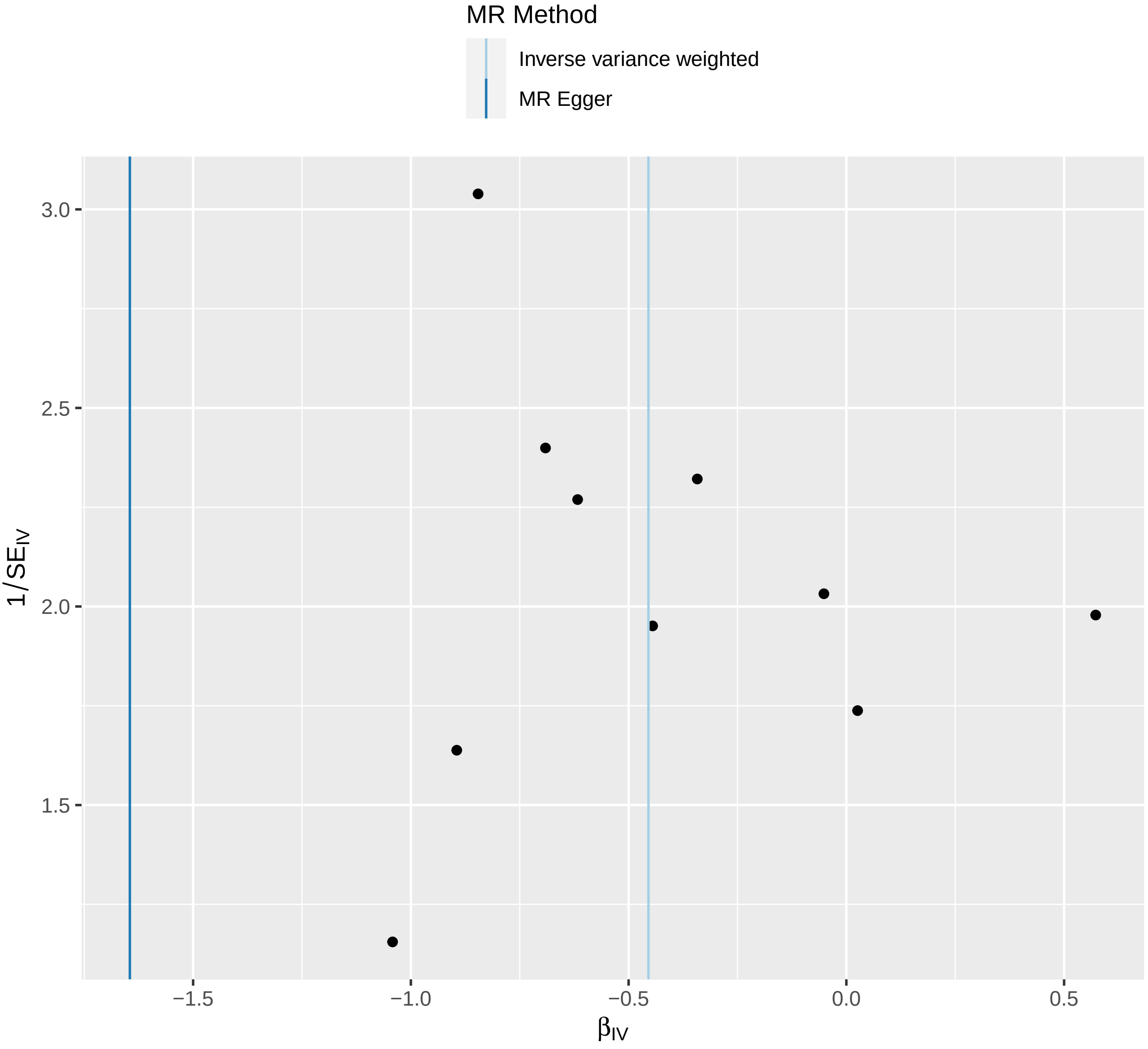

MR Method
Inverse variance weighted
MR Egger
1/SE_{IV}
3.0
2.5
2.0
1.5
−1.5
−1.0
−0.5
0.0
0.5
β_{IV}

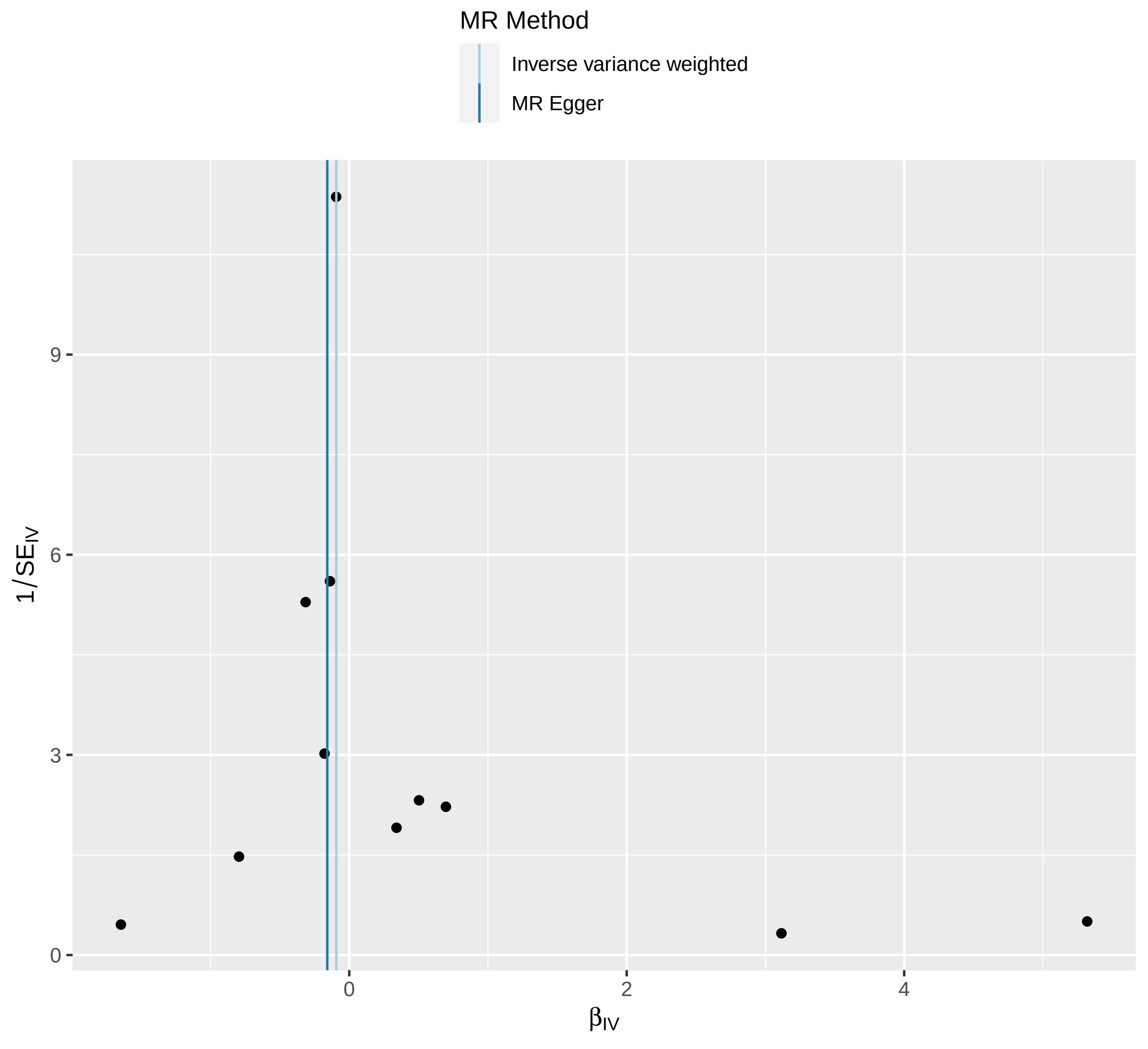
MR Method
Inverse variance weighted
MR Egger
1/SE$_{IV}$
9
6
3
0
0
2
4
β$_{IV}$

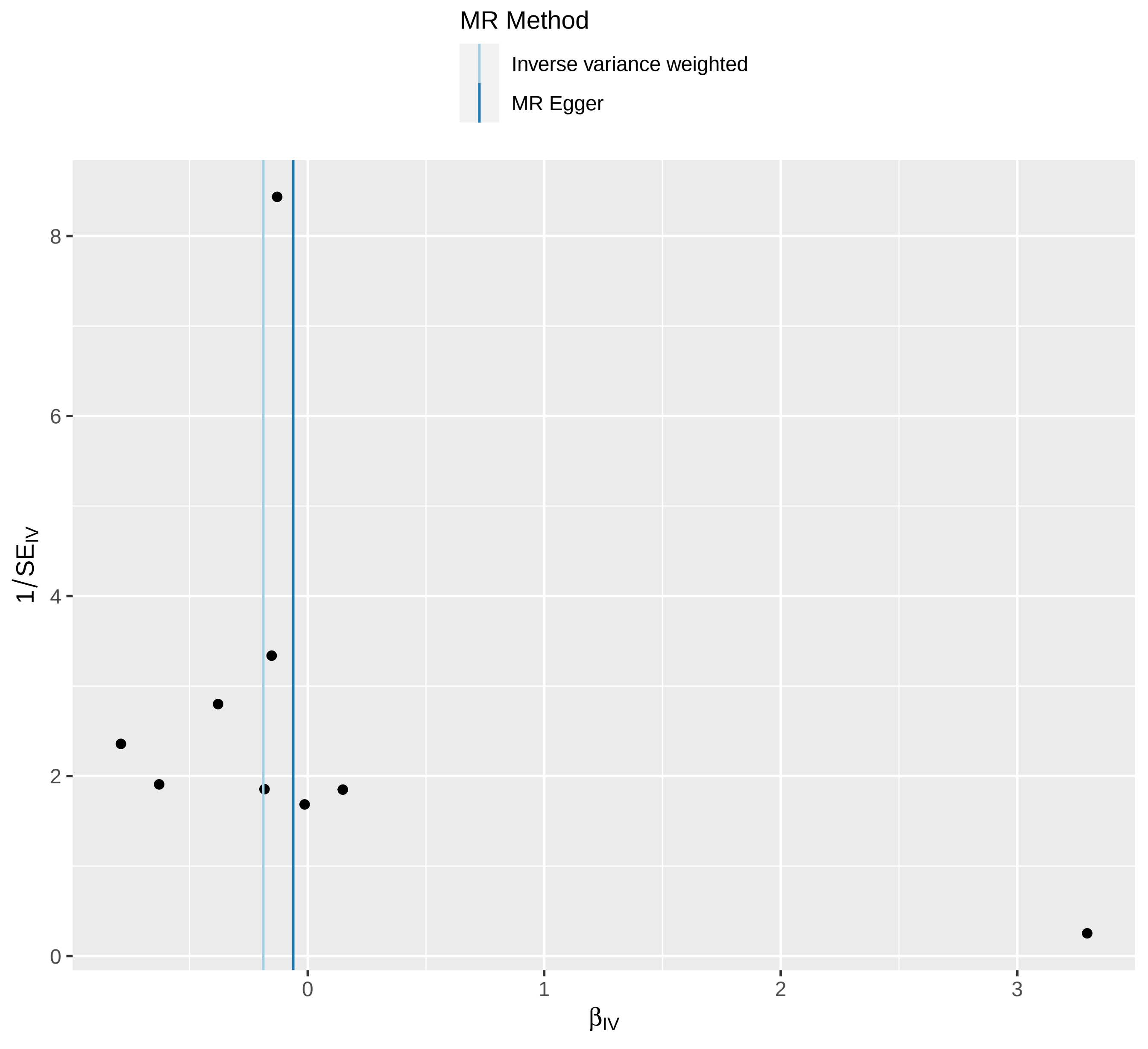

MR Method
Inverse variance weighted
MR Egger
8
6
4
2
0
1/SE_IV
0
1
2
3
β_IV

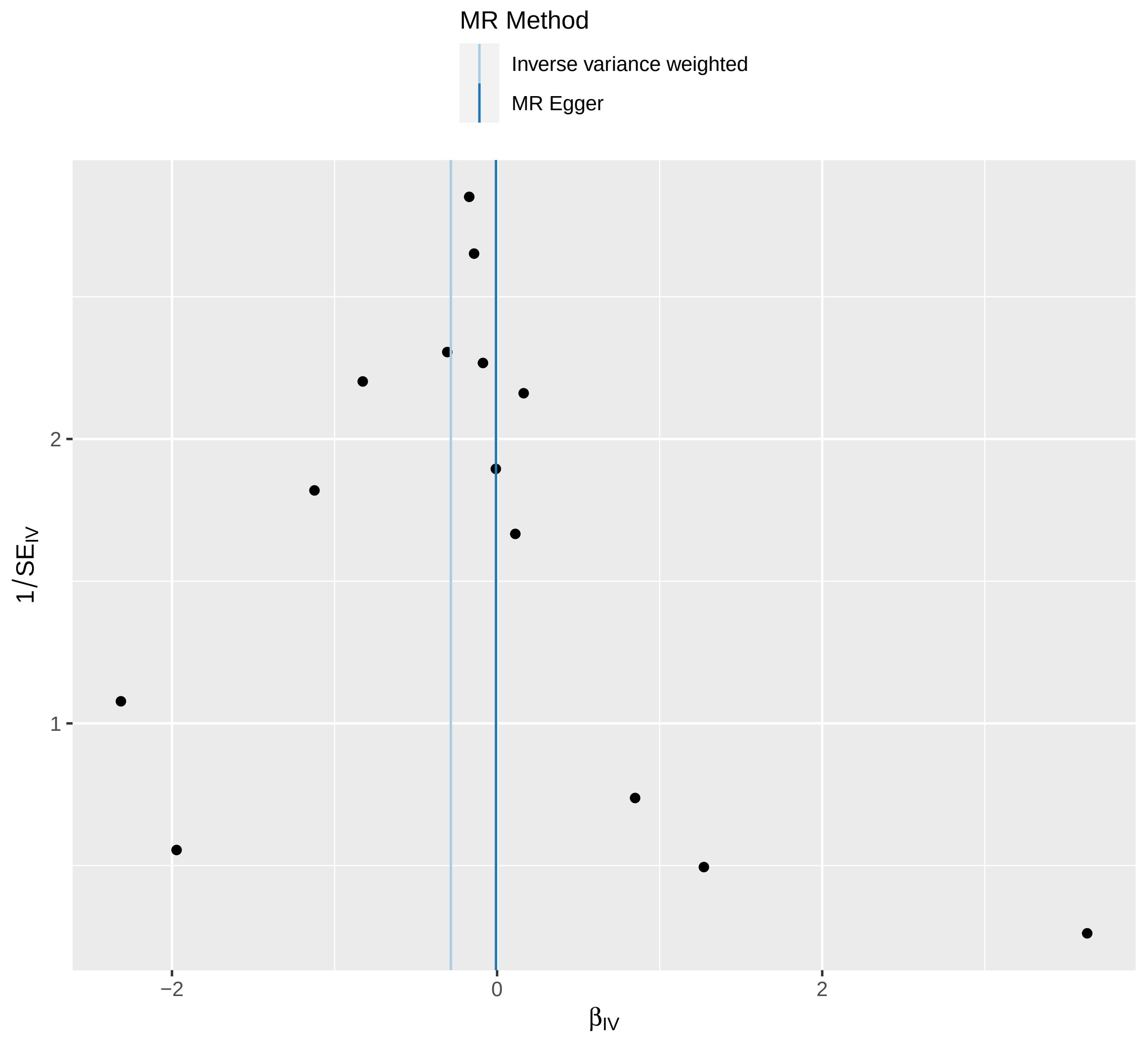

MR Method
Inverse variance weighted
MR Egger
2
1
1/SE_IV
−2
0
2
β_IV

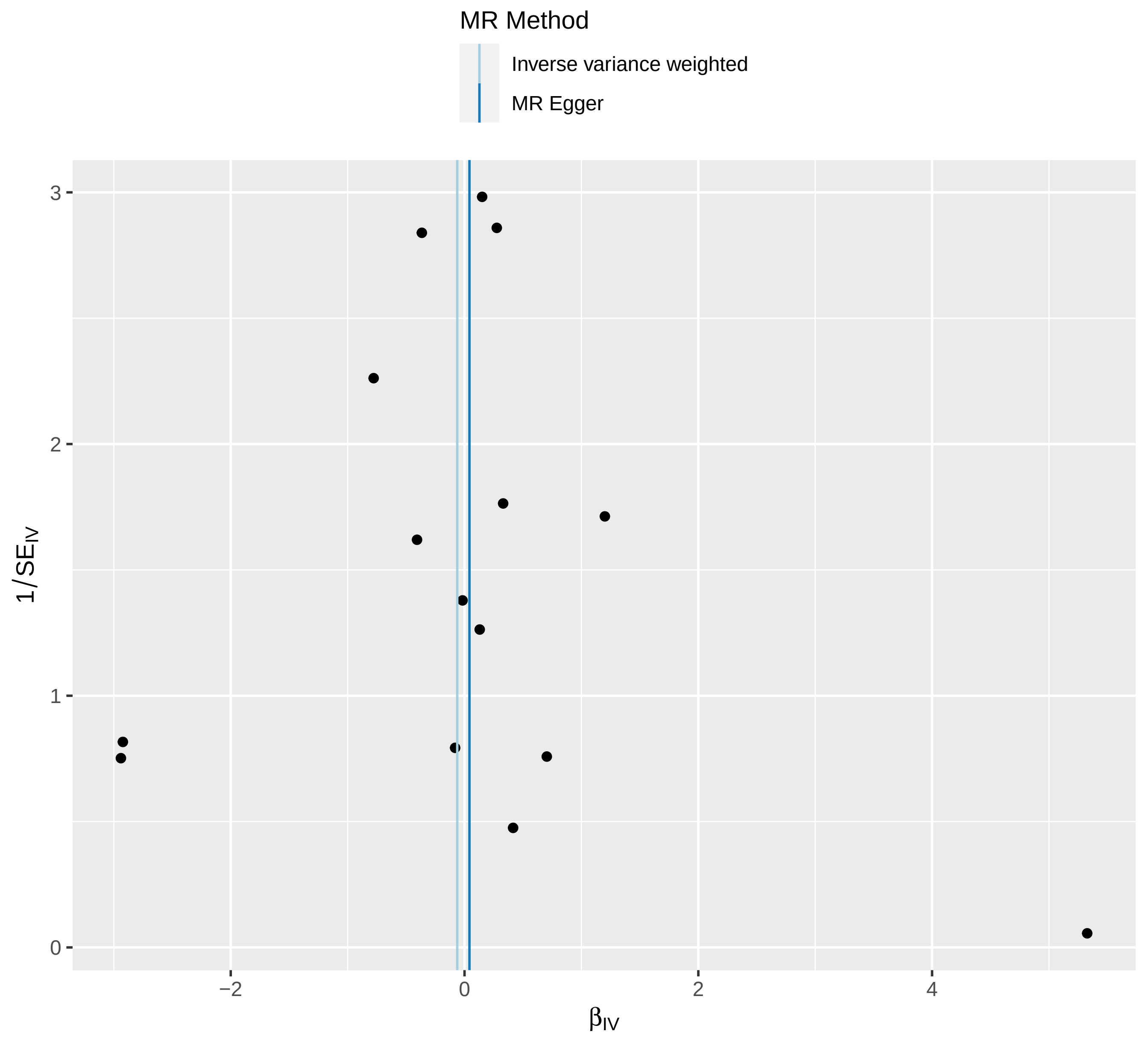

MR Method
Inverse variance weighted
MR Egger
3
2
1
0
$1/SE_{IV}$
−2
0
2
4
β_{IV}

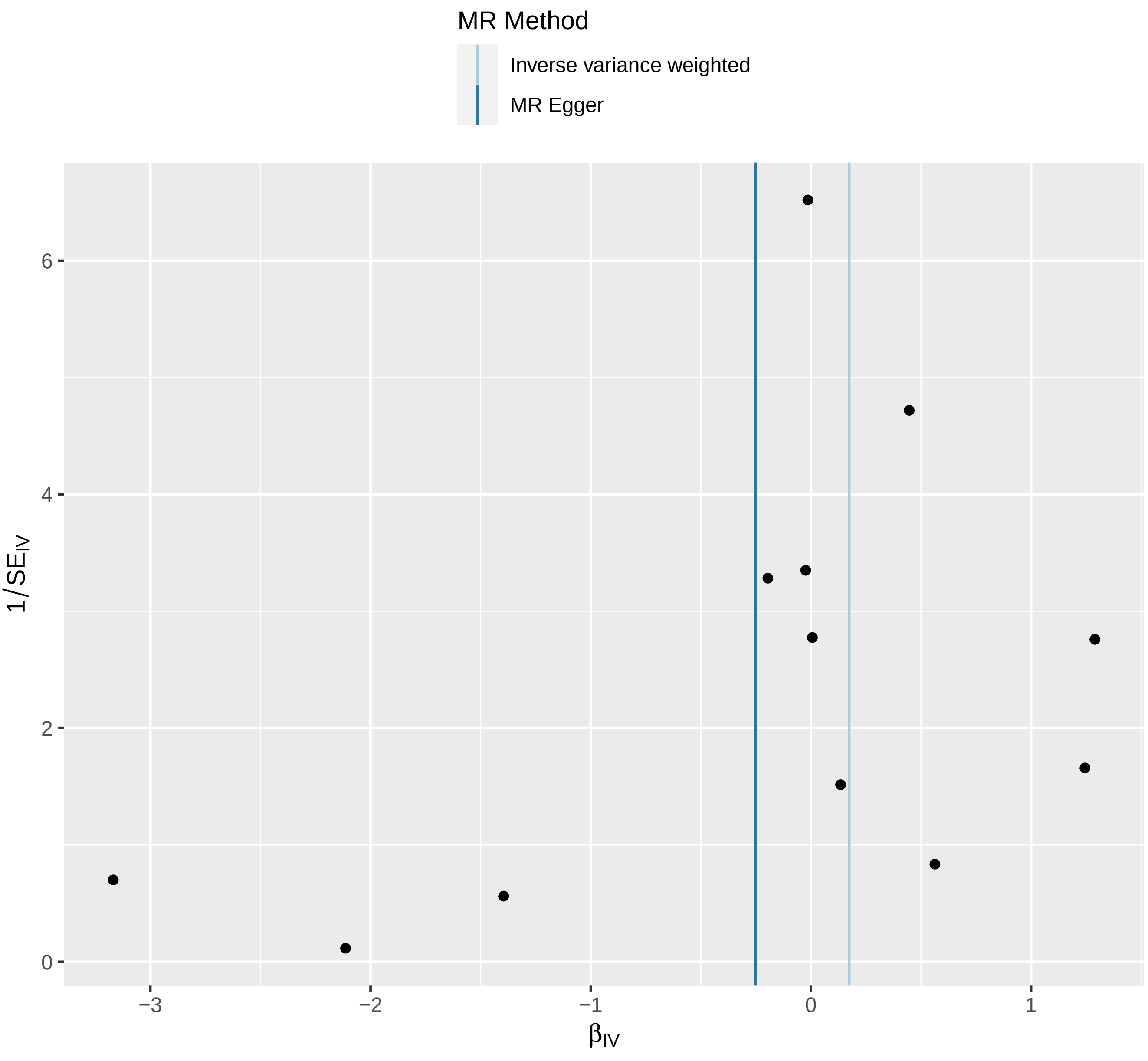
MR Method
Inverse variance weighted
MR Egger
1/SE$_{IV}$
β$_{IV}$
−3
−2
−1
0
1
0
2
4
6

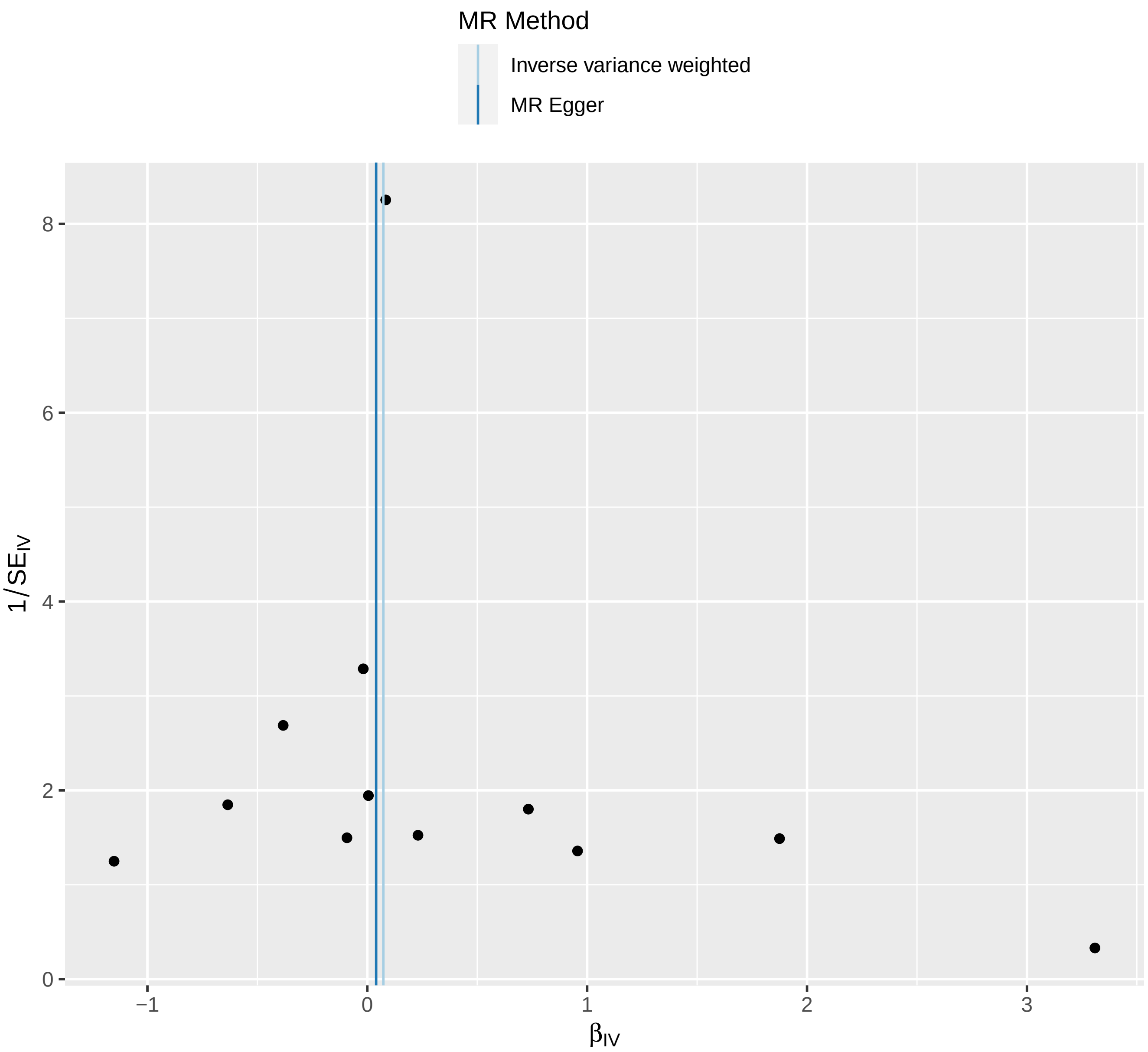

MR Method
Inverse variance weighted
MR Egger
1/SE$_{IV}$
β$_{IV}$
0
2
4
6
8
−1
0
1
2
3

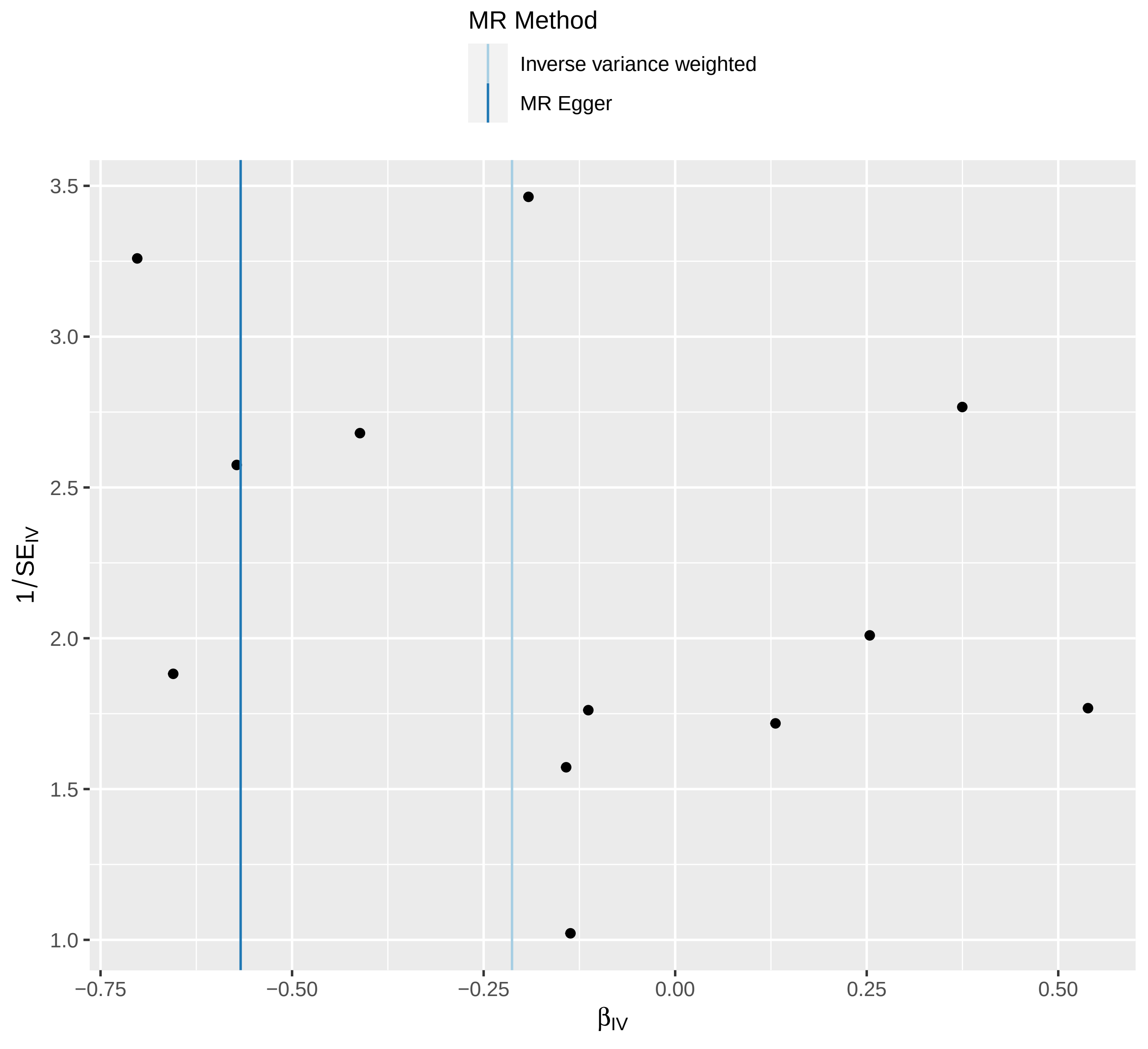

MR Method
Inverse variance weighted
MR Egger
3.5
3.0
2.5
2.0
1.5
1.0
$1/SE_{IV}$
−0.75
−0.50
−0.25
0.00
0.25
0.50
β_{IV}

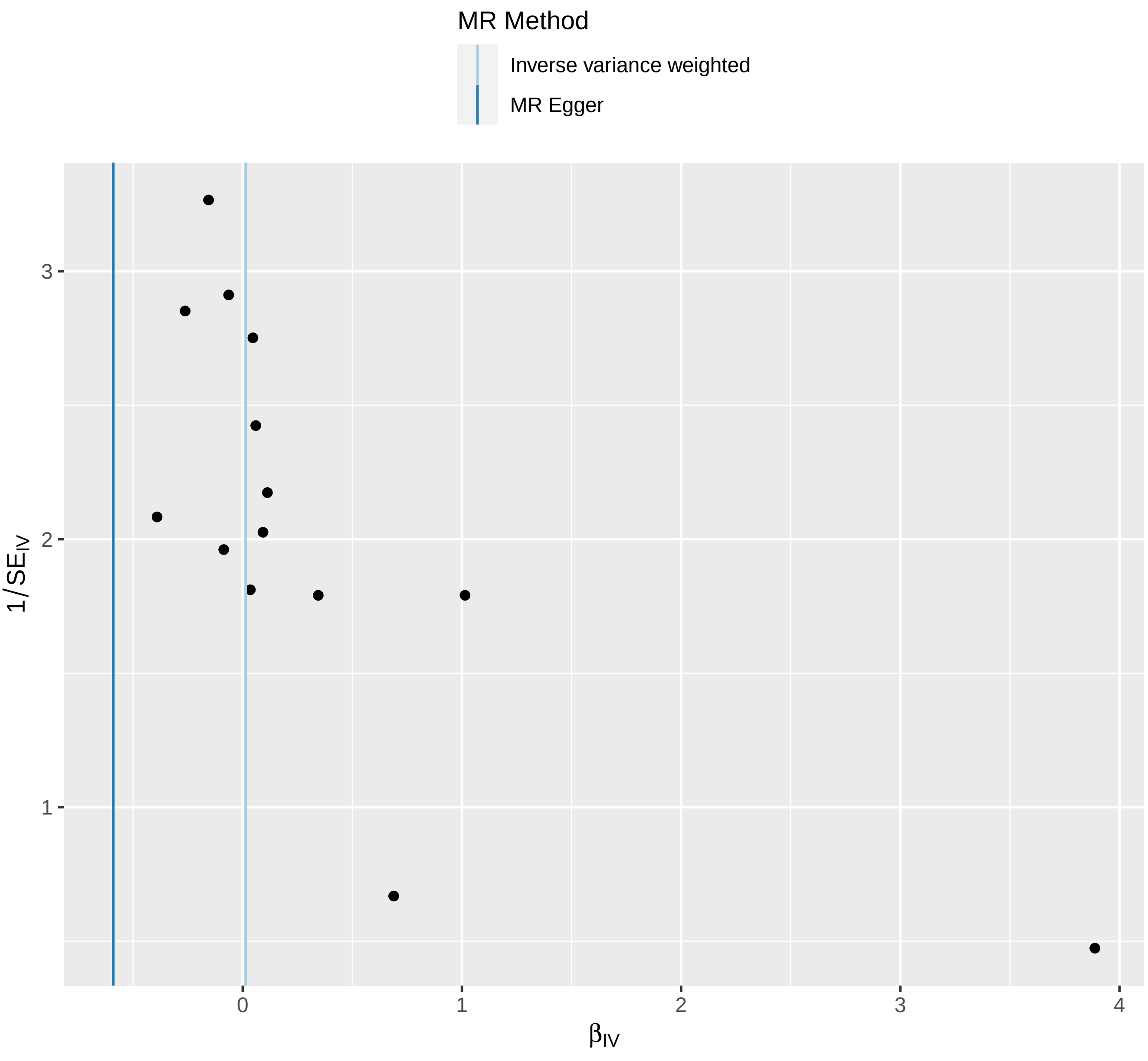
MR Method
Inverse variance weighted
MR Egger
3
2
1
$1/SE_{IV}$
0
1
2
3
4
β_{IV}

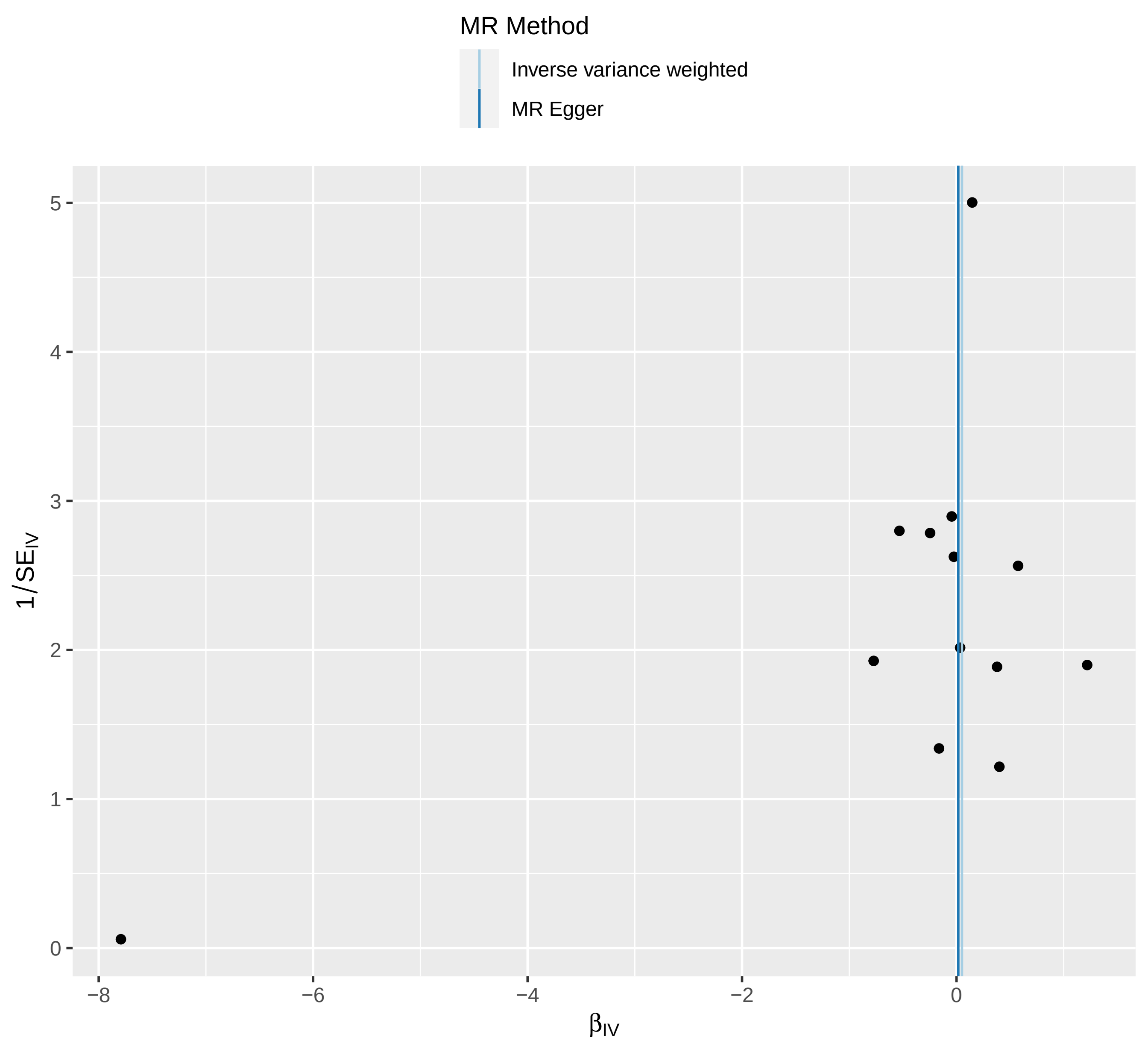

MR Method
Inverse variance weighted
MR Egger
5
4
3
2
1
0
1/SE$_{IV}$
−8
−6
−4
−2
0
β$_{IV}$

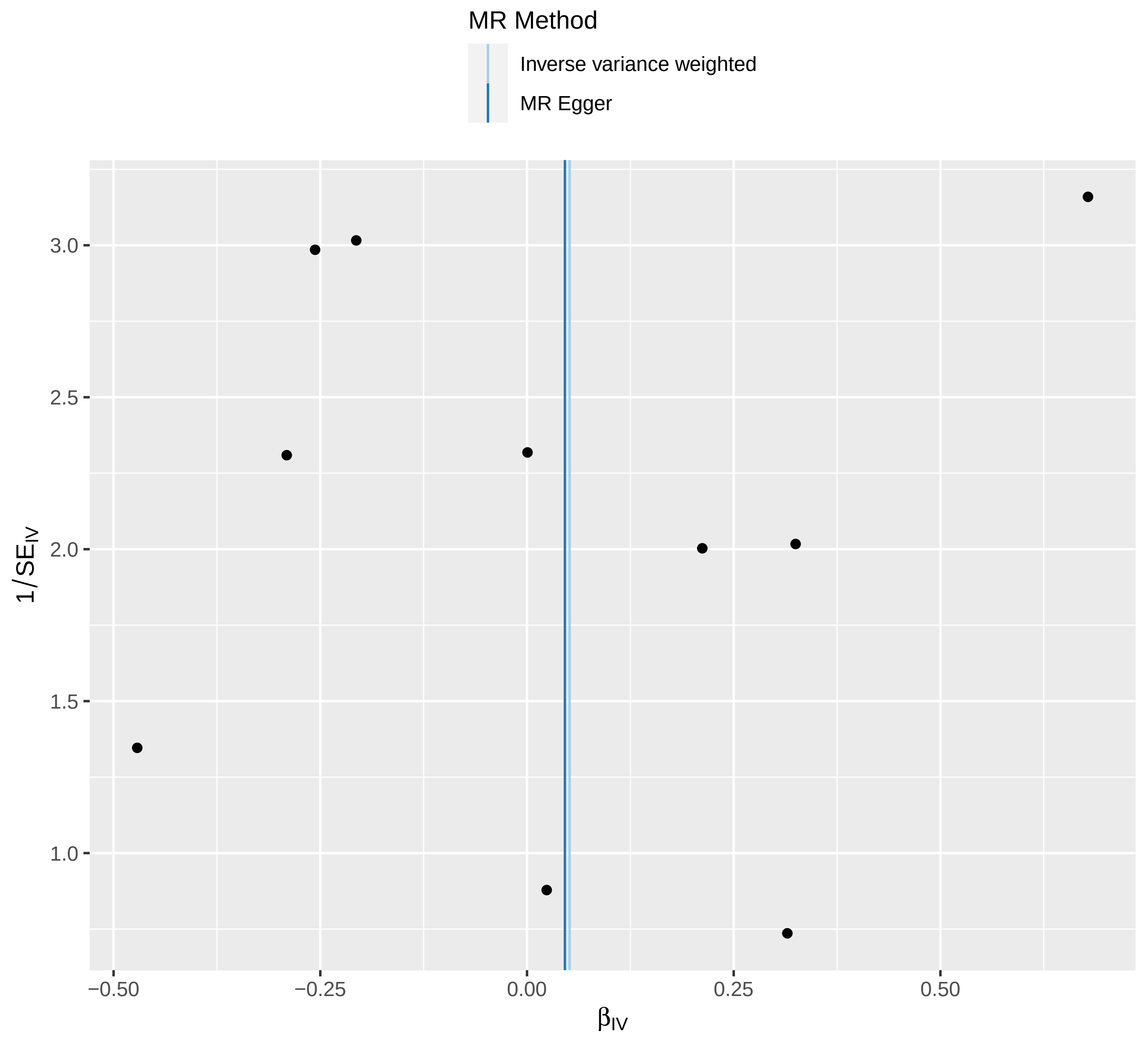

MR Method
Inverse variance weighted
MR Egger
3.0
2.5
2.0
1.5
1.0
1/SE$_{IV}$
−0.50
−0.25
0.00
0.25
0.50
β$_{IV}$

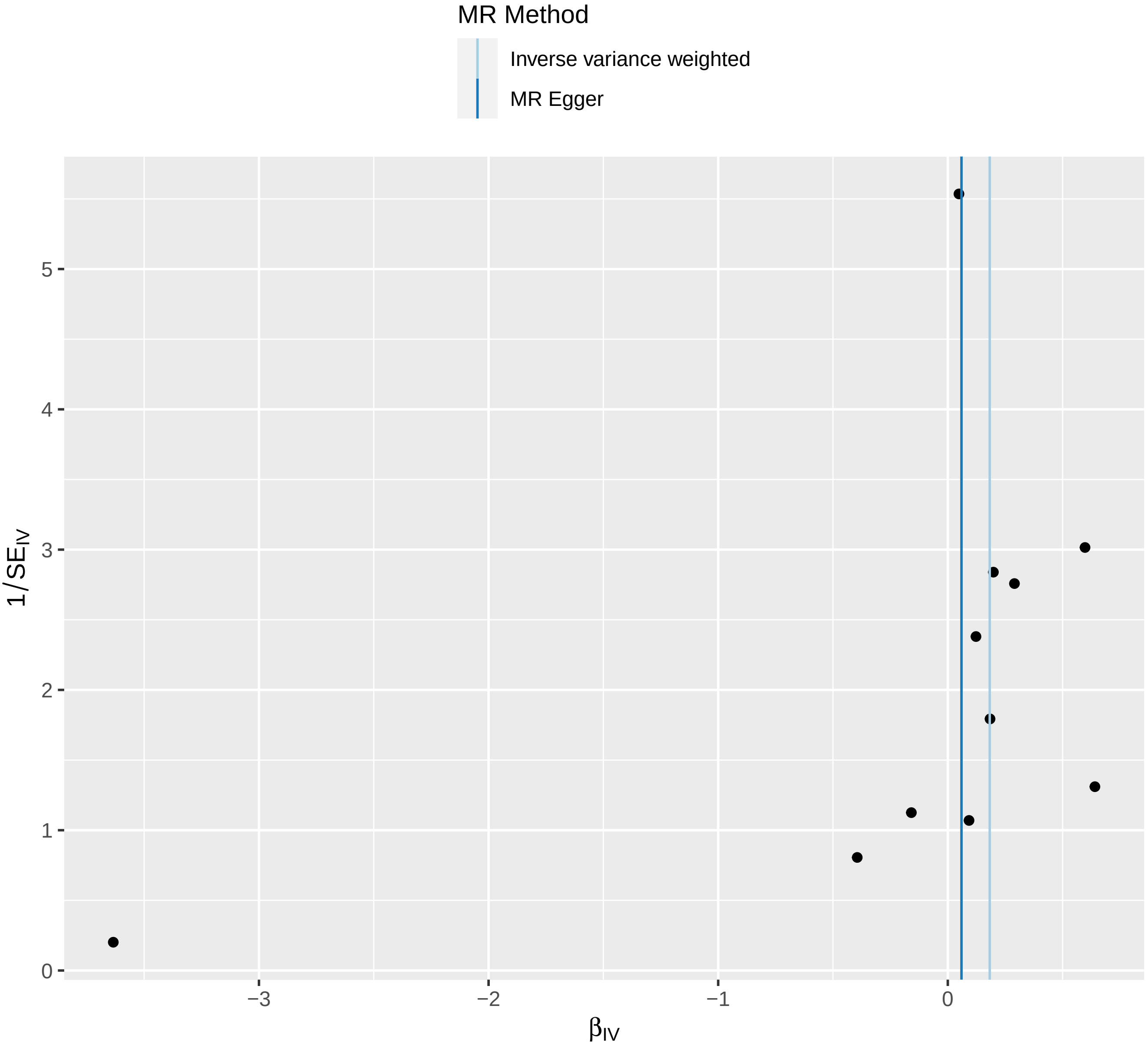
MR Method
Inverse variance weighted
MR Egger
1/SE_IV
5
4
3
2
1
0
−3
−2
−1
0
β_IV

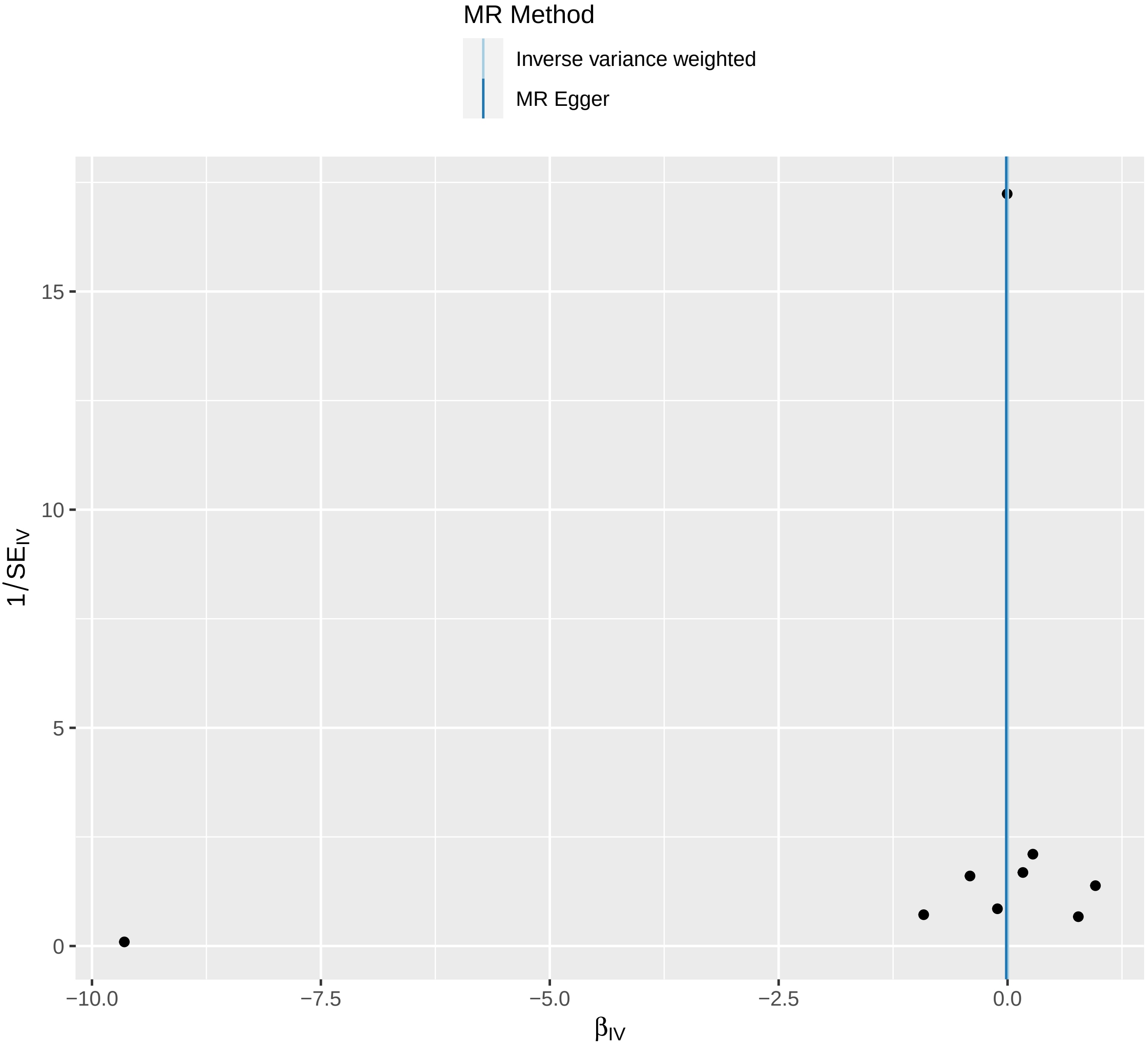
MR Method
Inverse variance weighted
MR Egger
15
10
5
0
1/SE_IV
−10.0
−7.5
−5.0
−2.5
0.0
β_IV

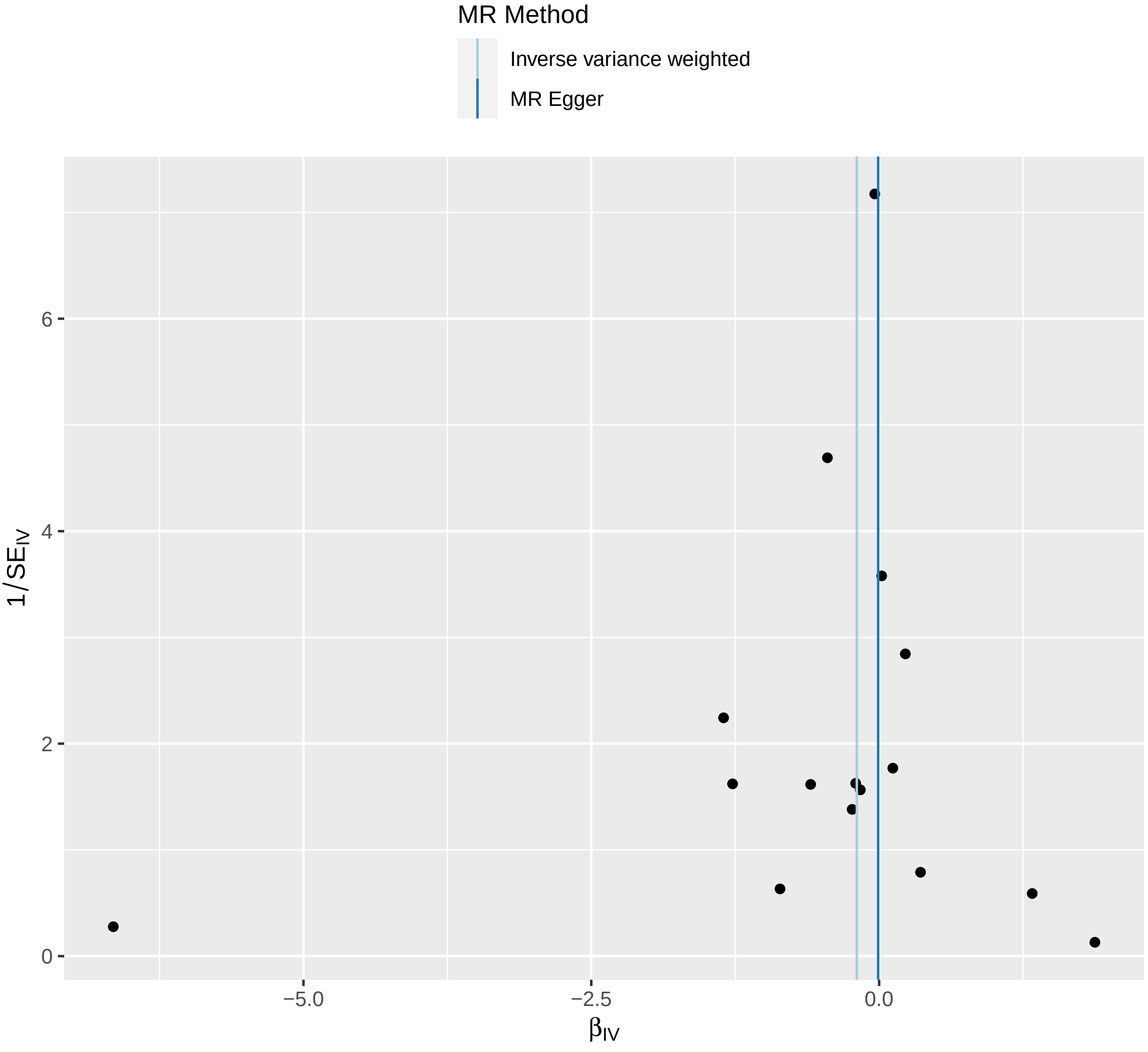

MR Method
Inverse variance weighted
MR Egger
6
4
2
0
$1/SE_{IV}$
−5.0
−2.5
0.0
β_{IV}

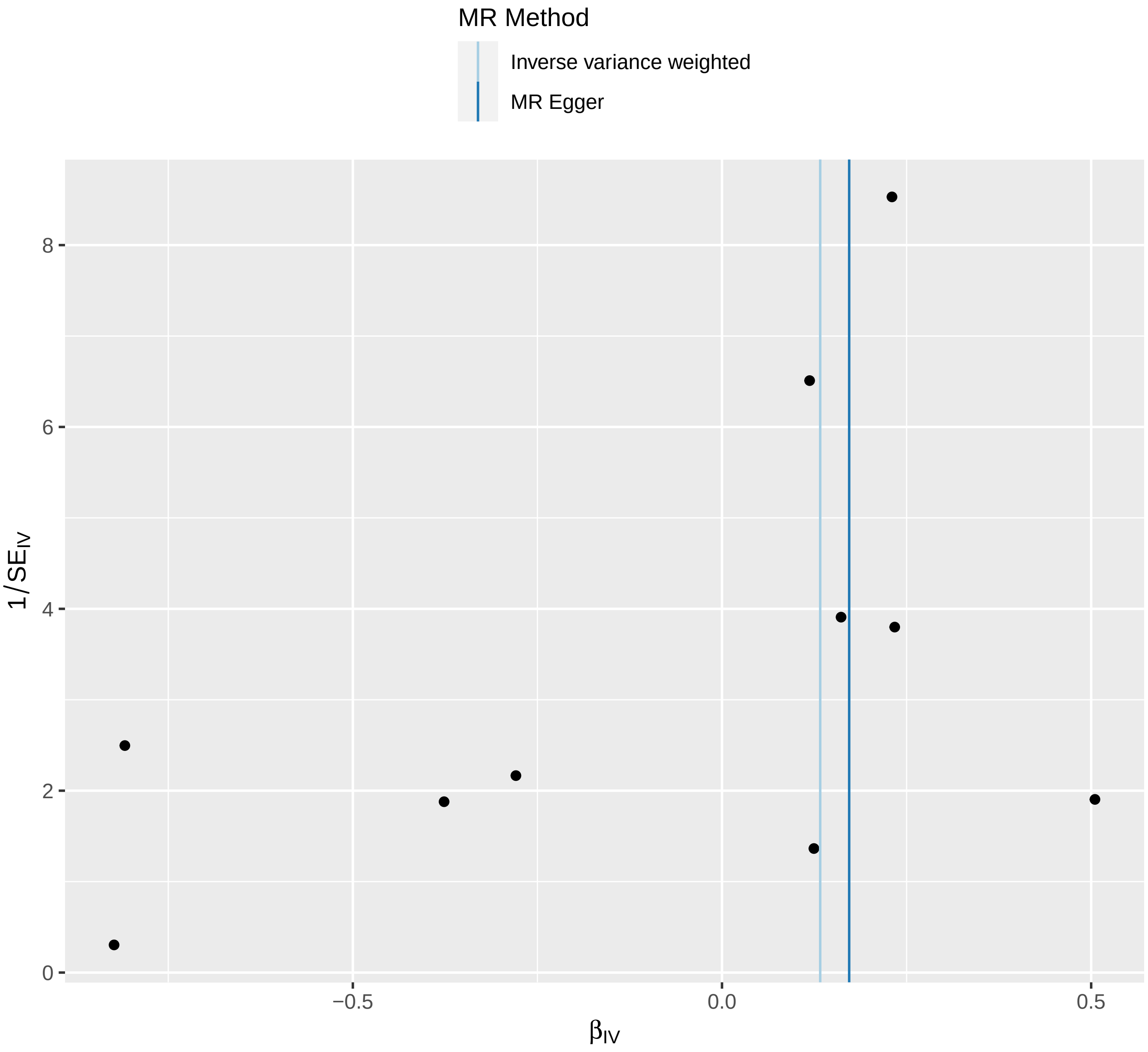

MR Method
Inverse variance weighted
MR Egger
8
6
4
2
0
$1/SE_{IV}$
−0.5
0.0
0.5
β_{IV}

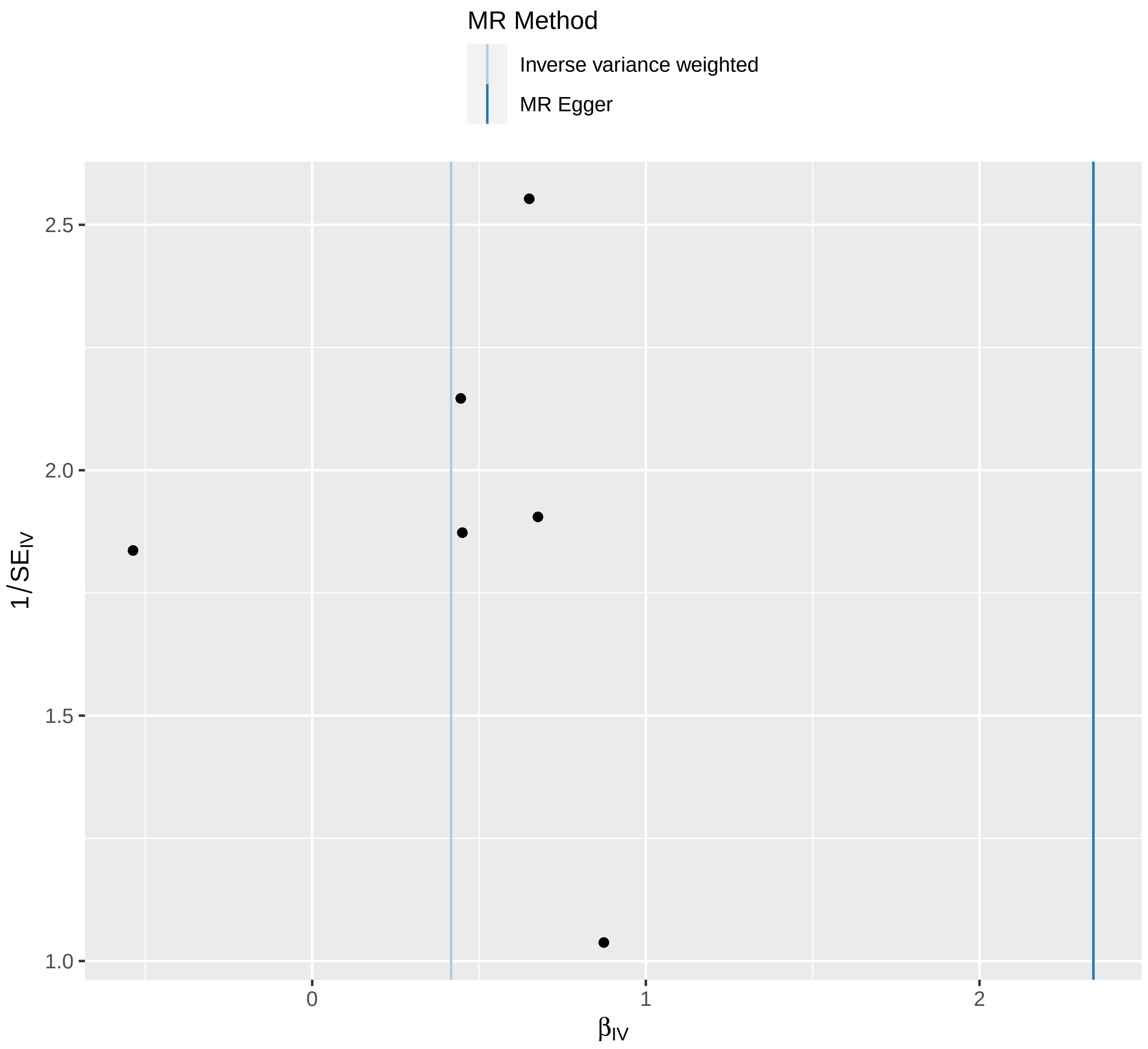
MR Method
Inverse variance weighted
MR Egger
2.5
2.0
1.5
1.0
0
1
2
$1/SE_{IV}$
β_{IV}

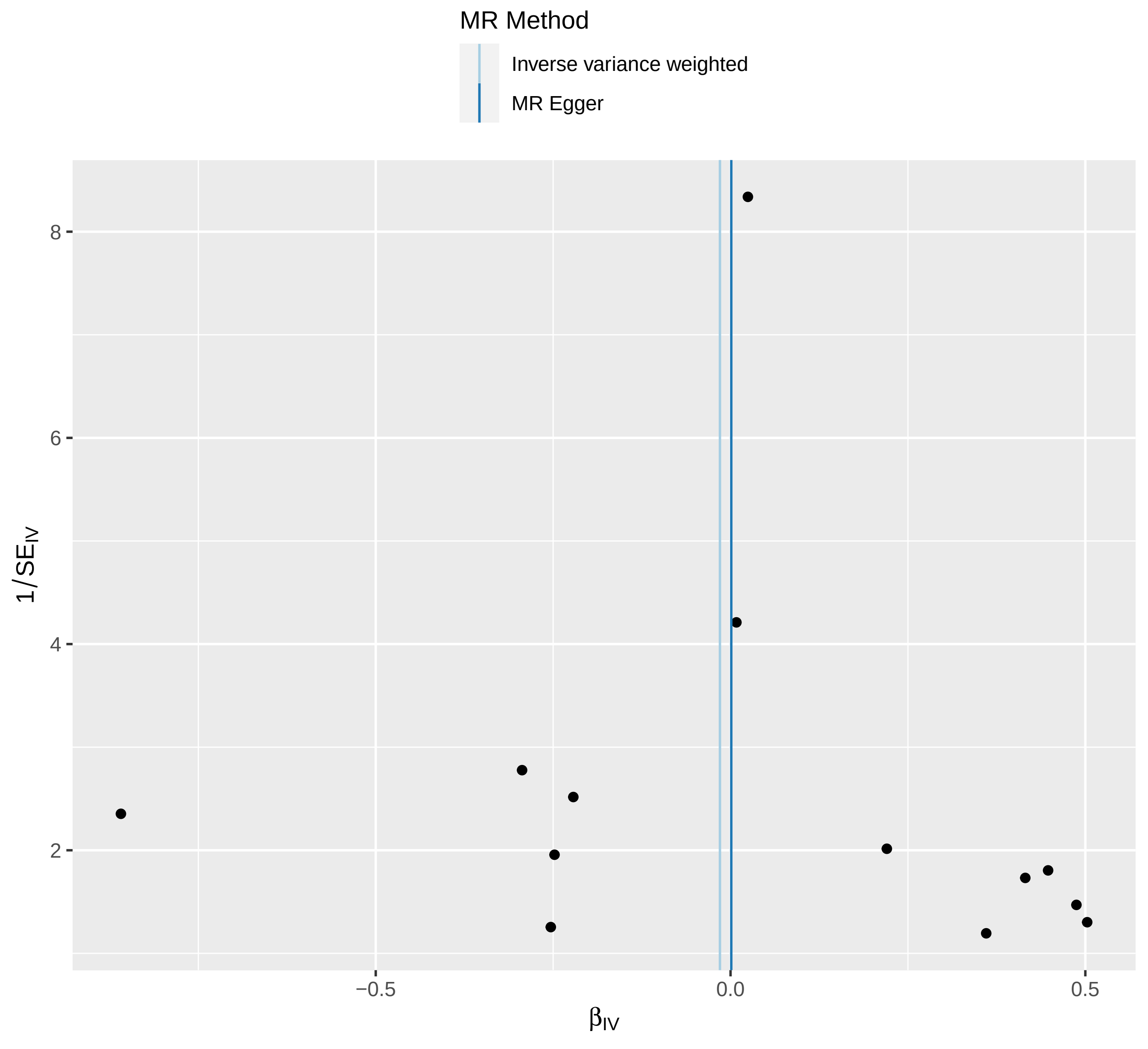

MR Method
Inverse variance weighted
MR Egger
8
6
4
2
1/SE$_{IV}$
−0.5
0.0
0.5
β$_{IV}$

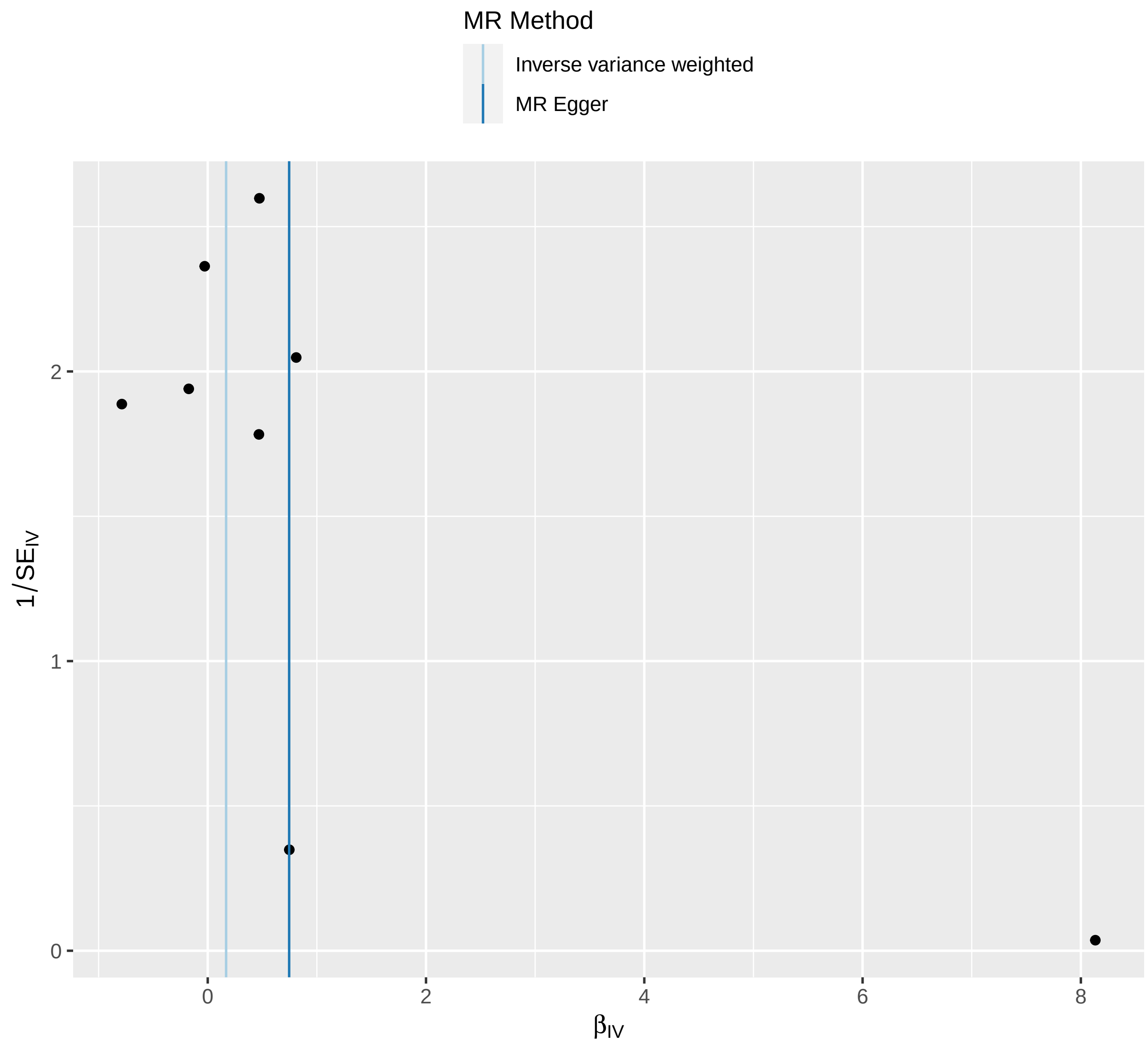
MR Method
Inverse variance weighted
MR Egger
1/SE_IV
0
1
2
0
2
4
6
8
β_IV

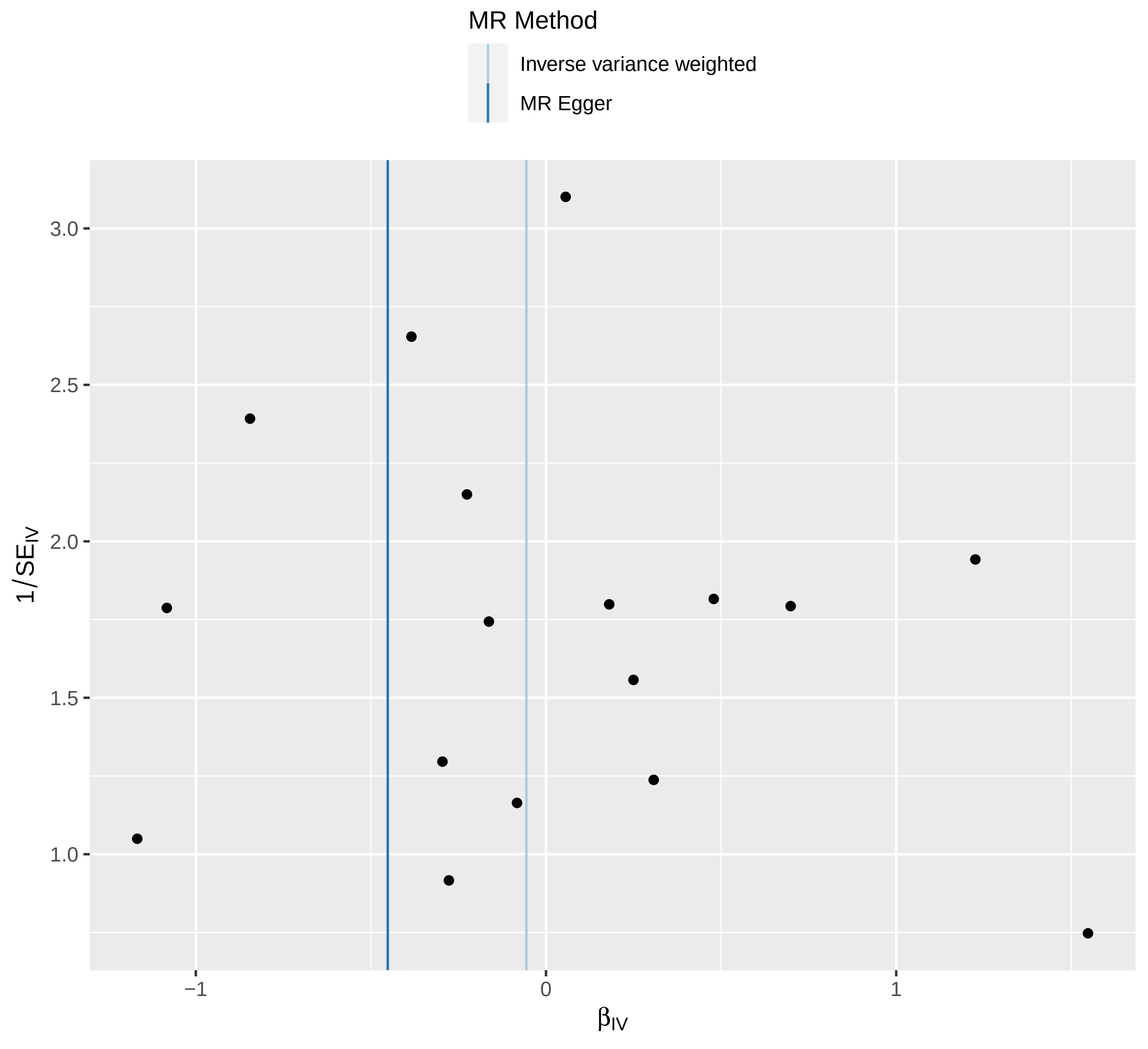
MR Method
Inverse variance weighted
MR Egger
3.0
2.5
2.0
1.5
1.0
$1/SE_{IV}$
−1
0
1
β_{IV}

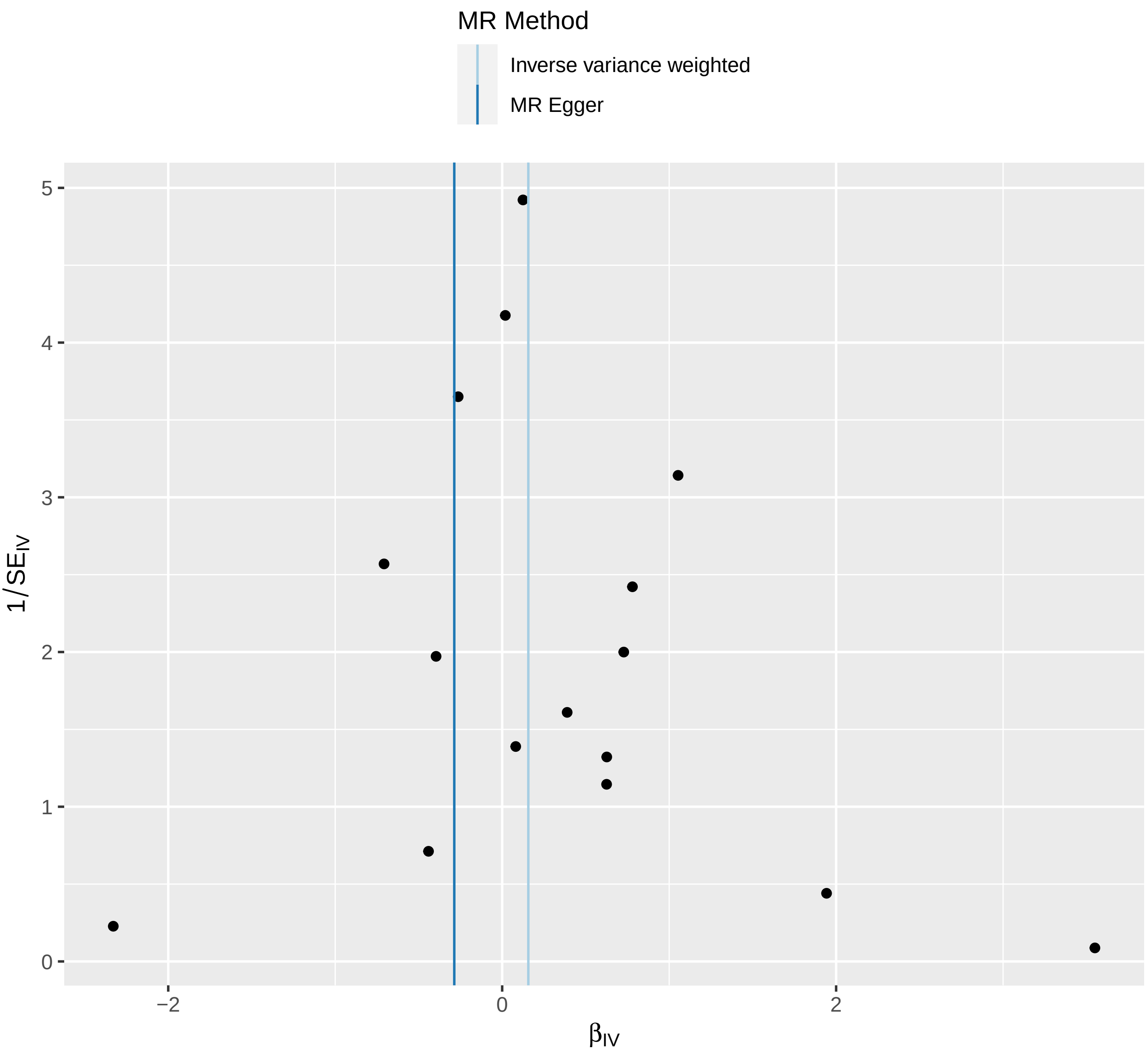

MR Method
Inverse variance weighted
MR Egger
5
4
3
2
1
0
1/SE$_{IV}$
−2
0
2
β$_{IV}$

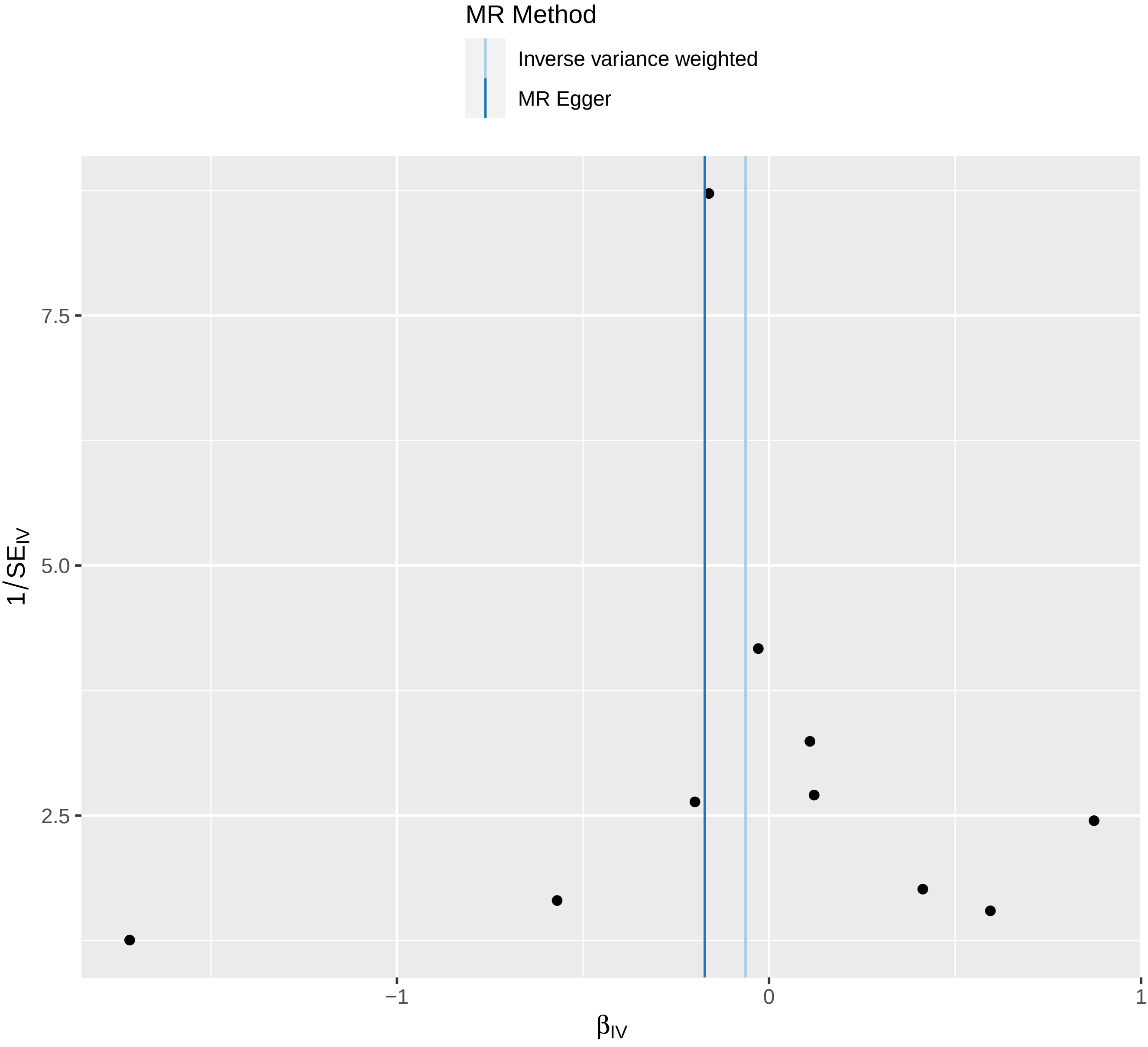

MR Method
Inverse variance weighted
MR Egger
7.5
5.0
2.5
1/SE_IV
−1
0
1
β_IV

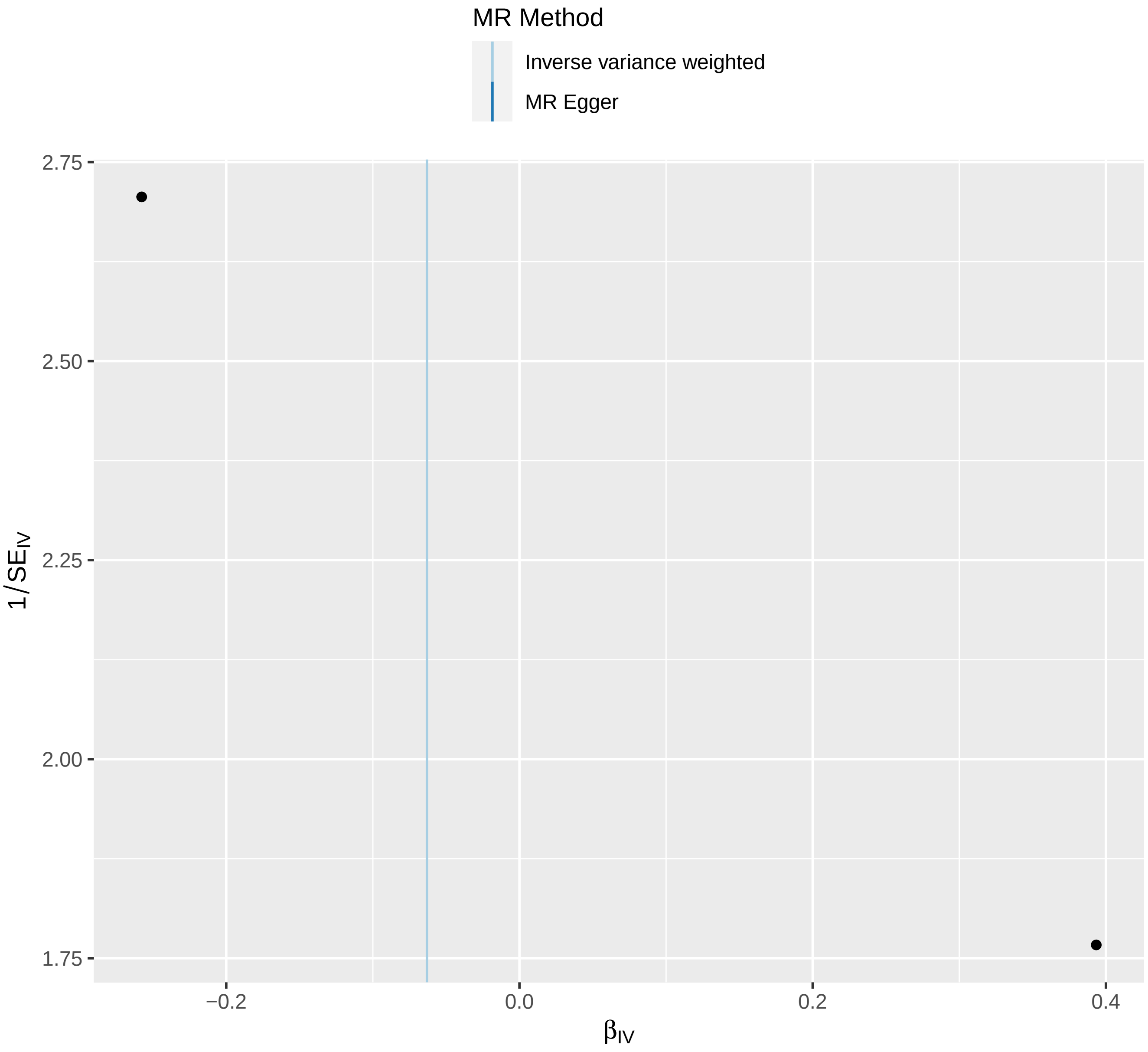

MR Method
Inverse variance weighted
MR Egger
2.75
2.50
2.25
2.00
1.75
1/SE$_{IV}$
−0.2
0.0
0.2
0.4
β$_{IV}$

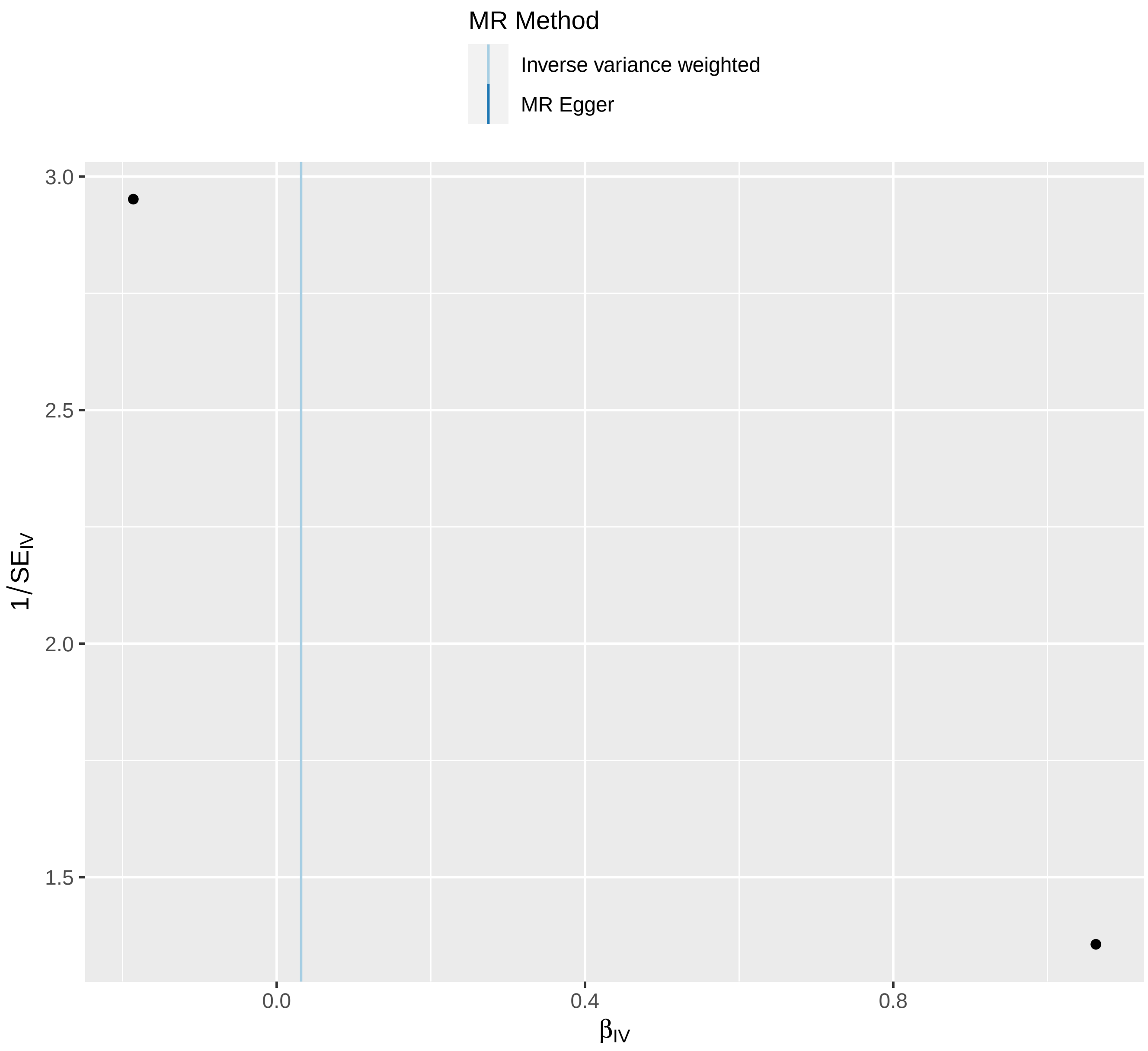
MR Method
Inverse variance weighted
MR Egger
3.0
2.5
2.0
1.5
1/SE_IV
0.0
0.4
0.8
β_IV

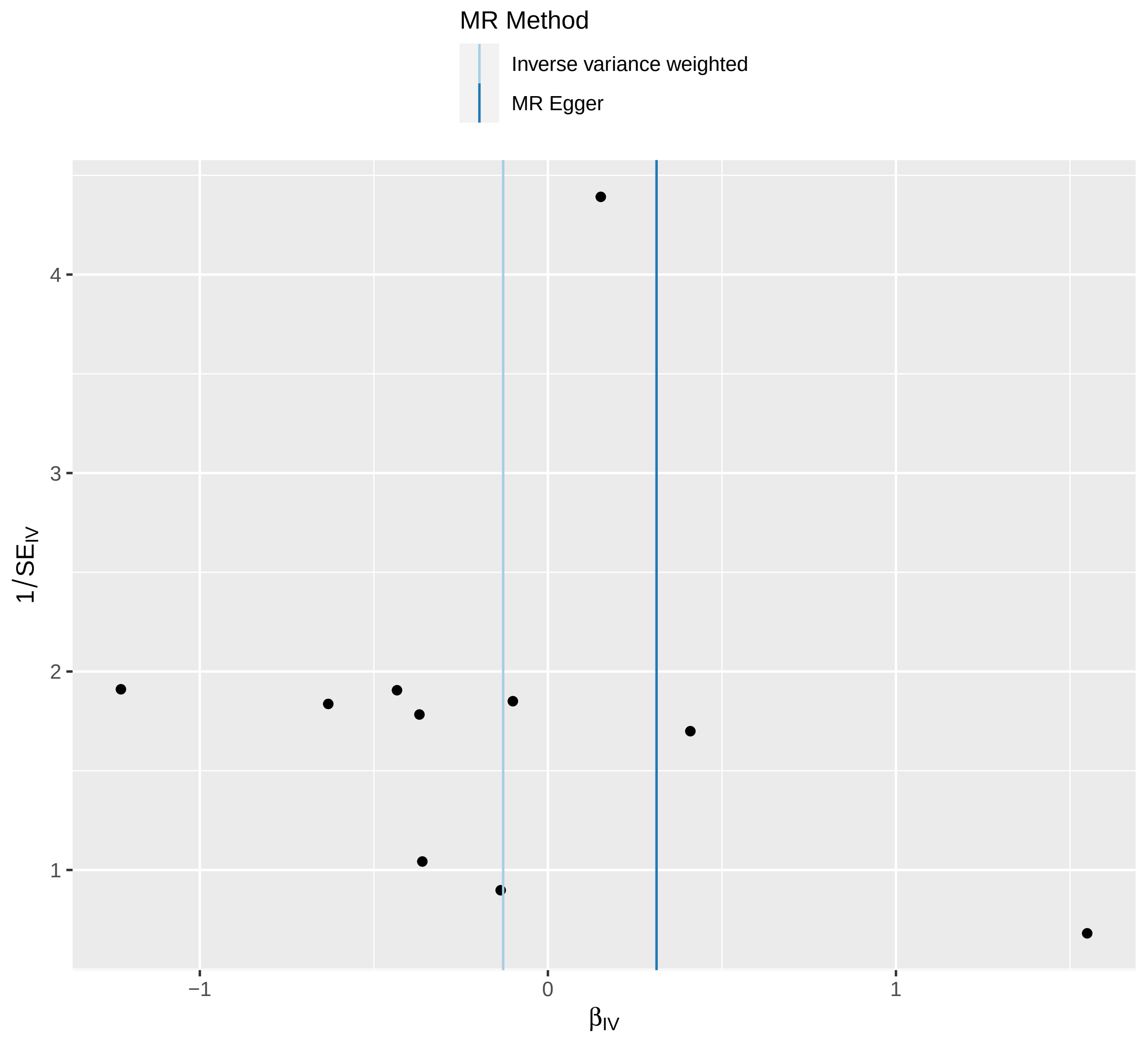
MR Method
Inverse variance weighted
MR Egger
4
3
2
1
−1
0
1
1/SE_IV
β_IV

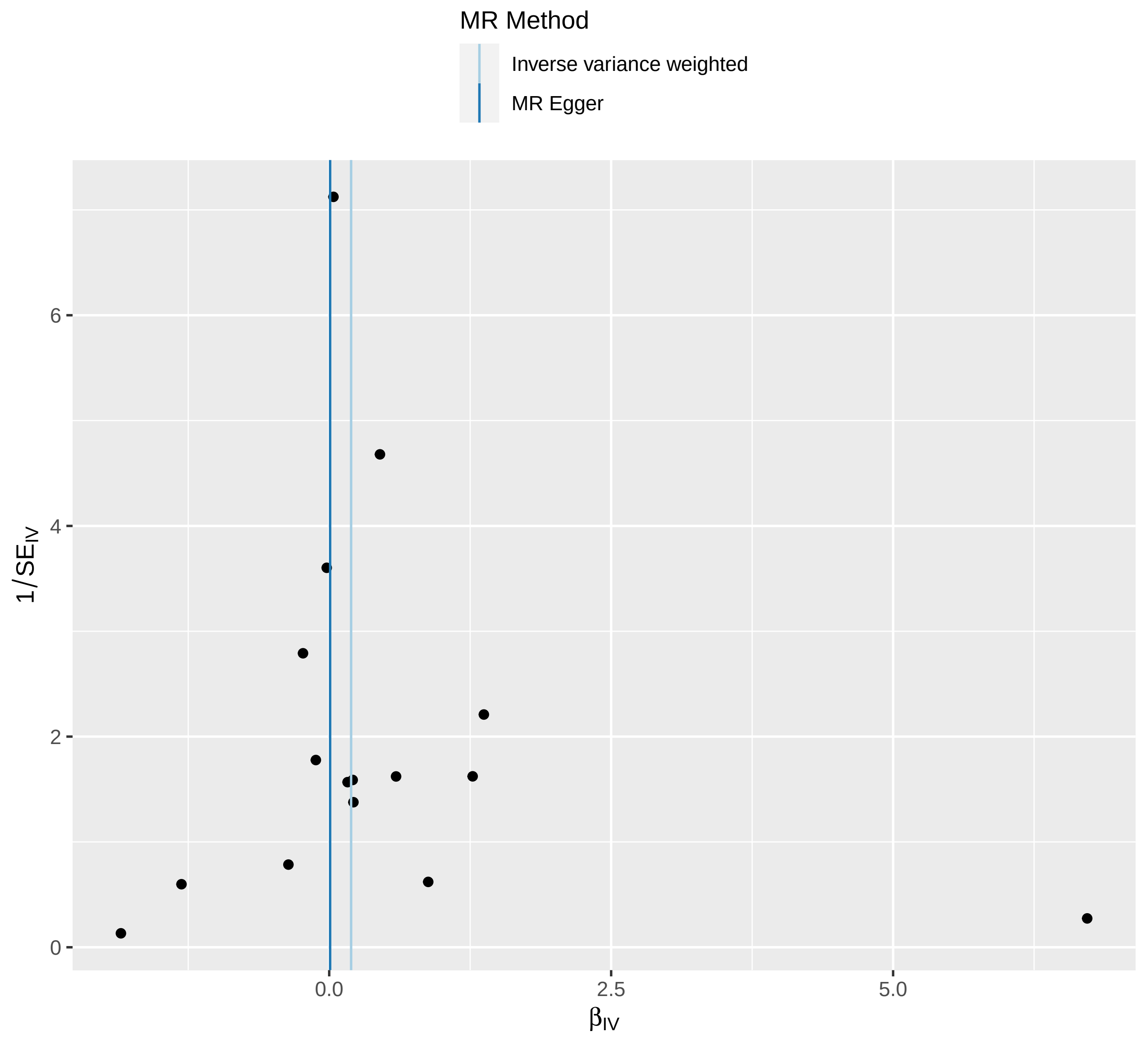
MR Method
Inverse variance weighted
MR Egger
1/SE_IV
0
2
4
6
0.0
2.5
5.0
β_IV

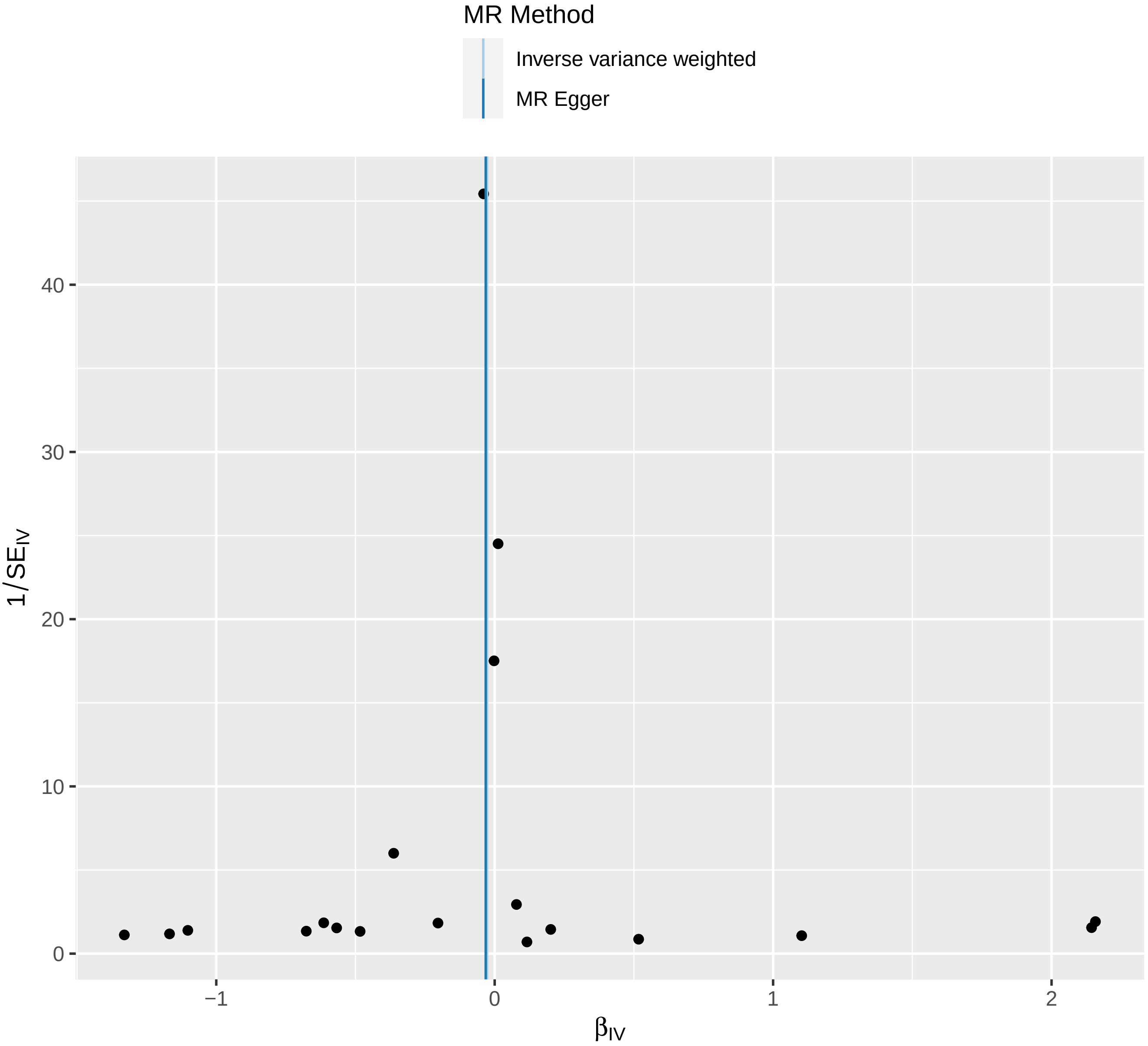

MR Method
Inverse variance weighted
MR Egger
40
30
20
10
0
−1
0
1
2
1/SE_IV
β_IV

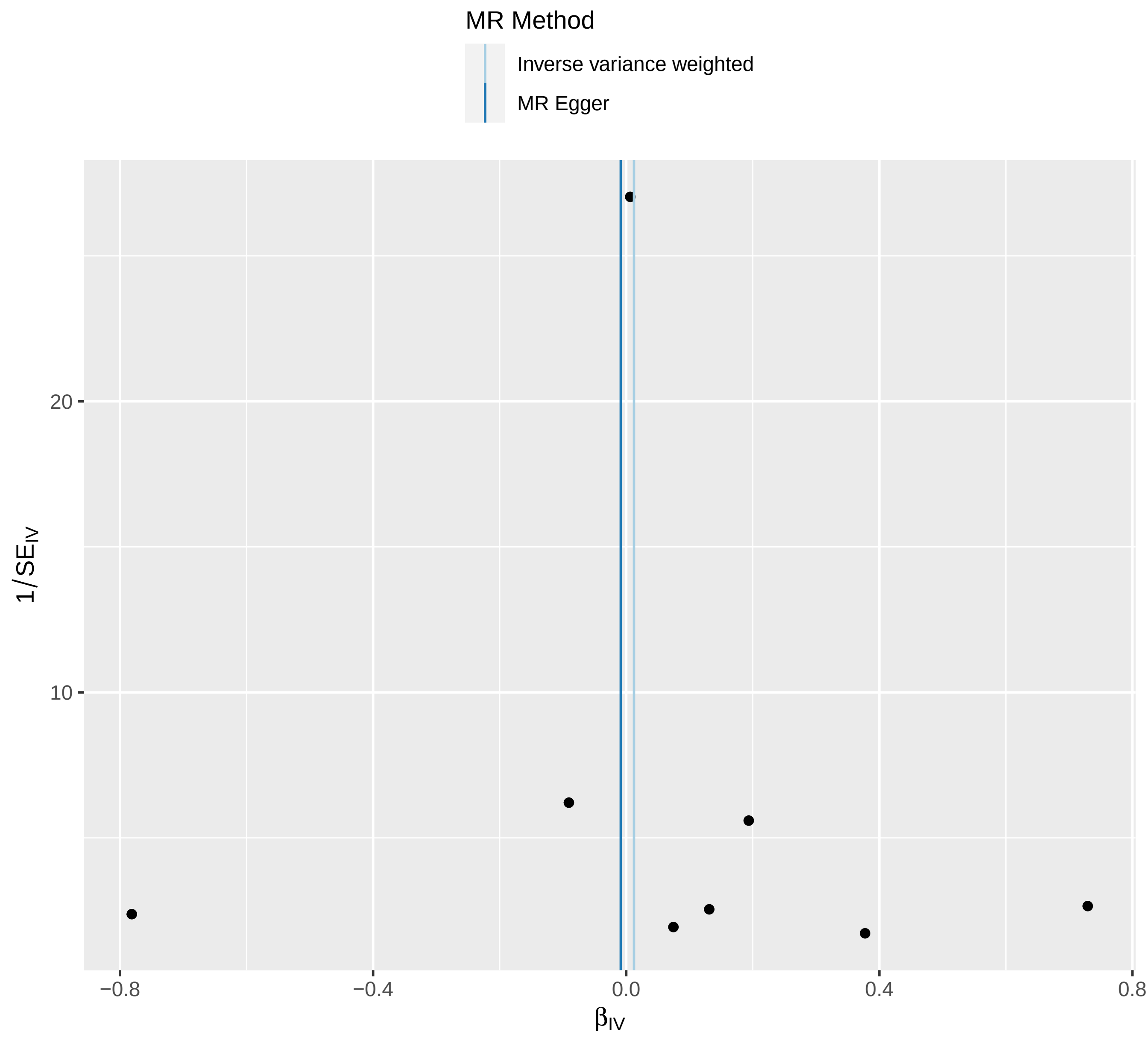

MR Method
Inverse variance weighted
MR Egger
1/SE_IV
20
10
−0.8
−0.4
0.0
0.4
0.8
β_IV

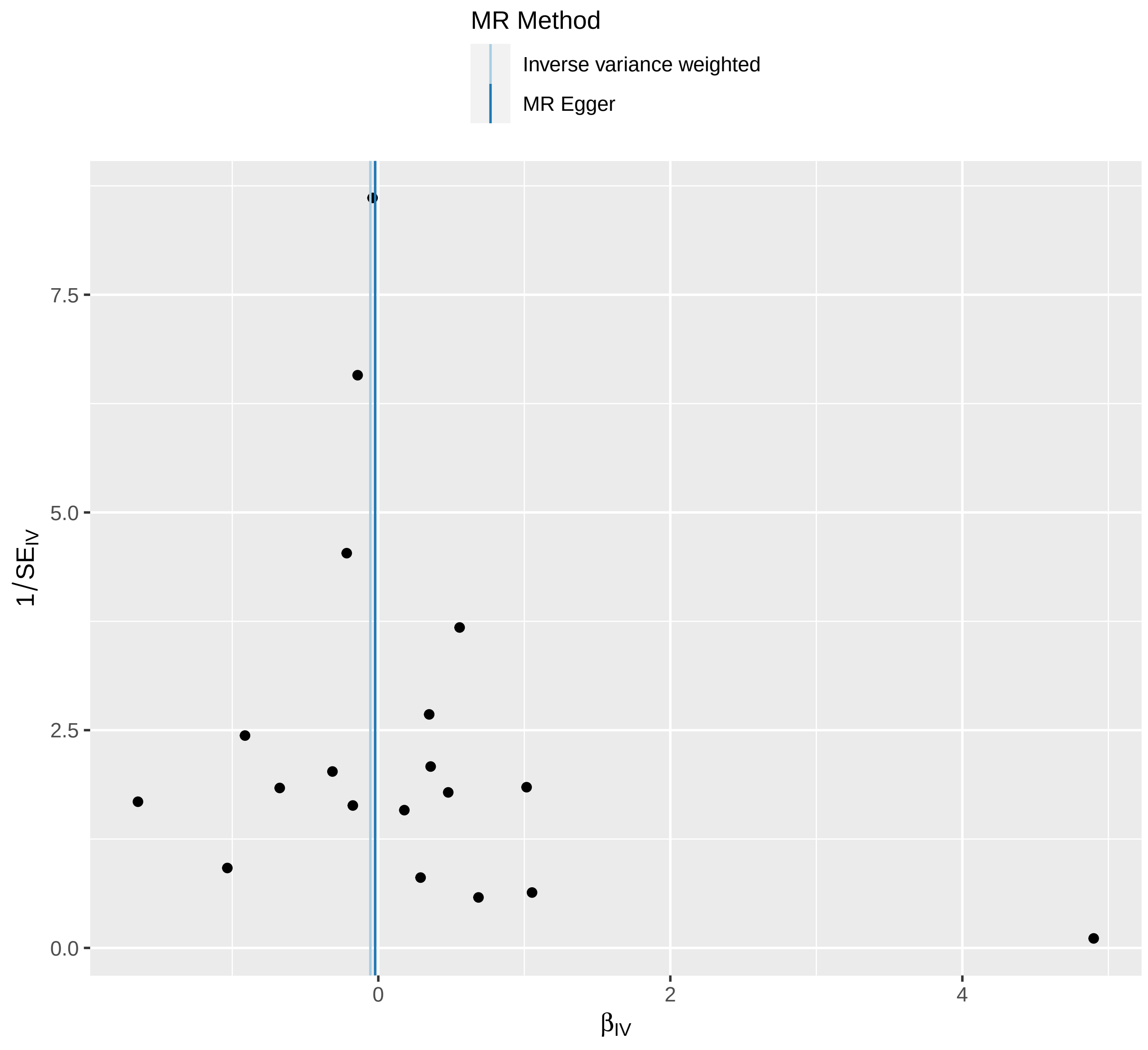

MR Method
Inverse variance weighted
MR Egger
1/SE$_{IV}$
7.5
5.0
2.5
0.0
0
2
4
β$_{IV}$

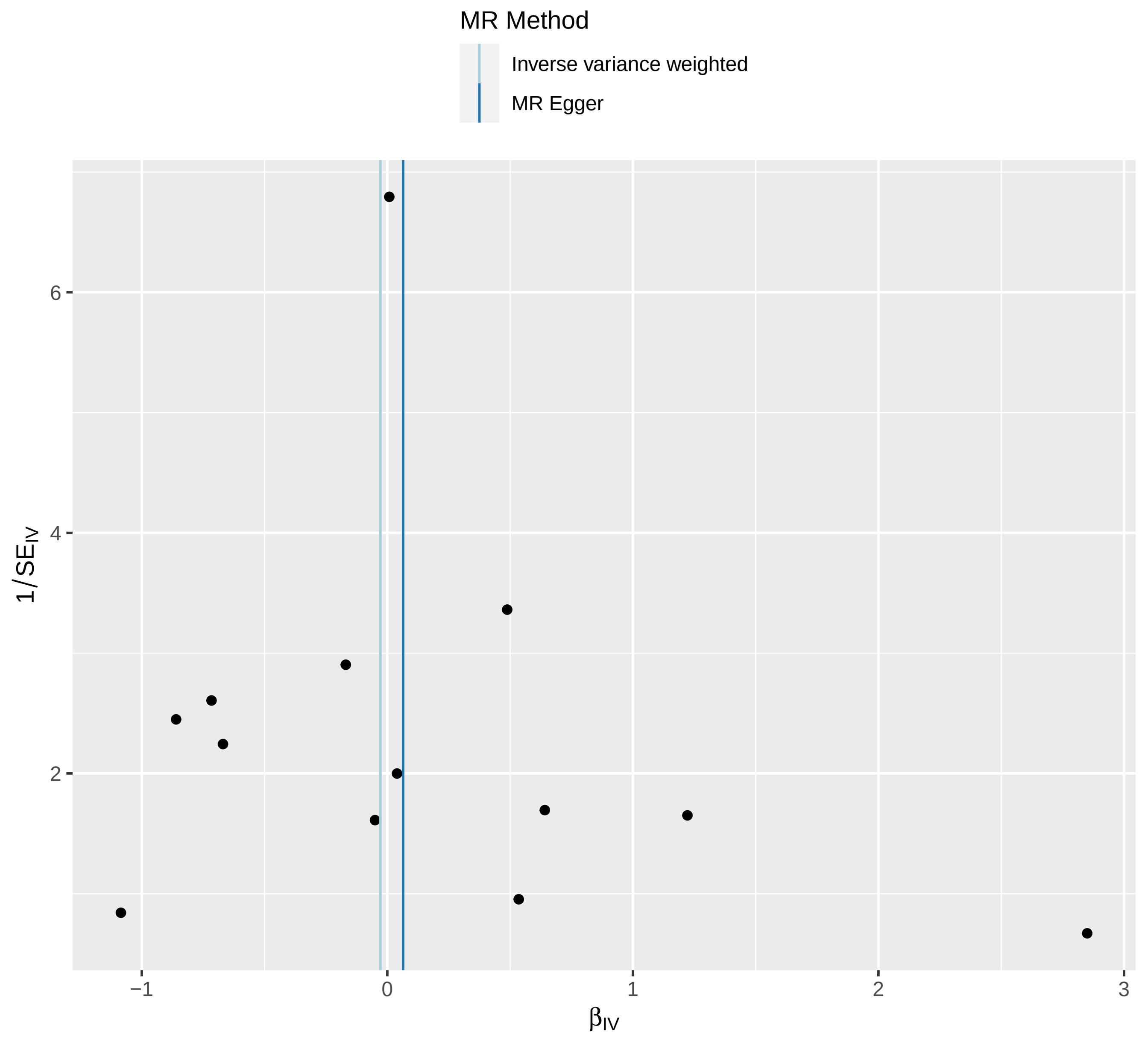
MR Method
Inverse variance weighted
MR Egger
6
4
2
1/SE$_{IV}$
−1
0
1
2
3
β$_{IV}$

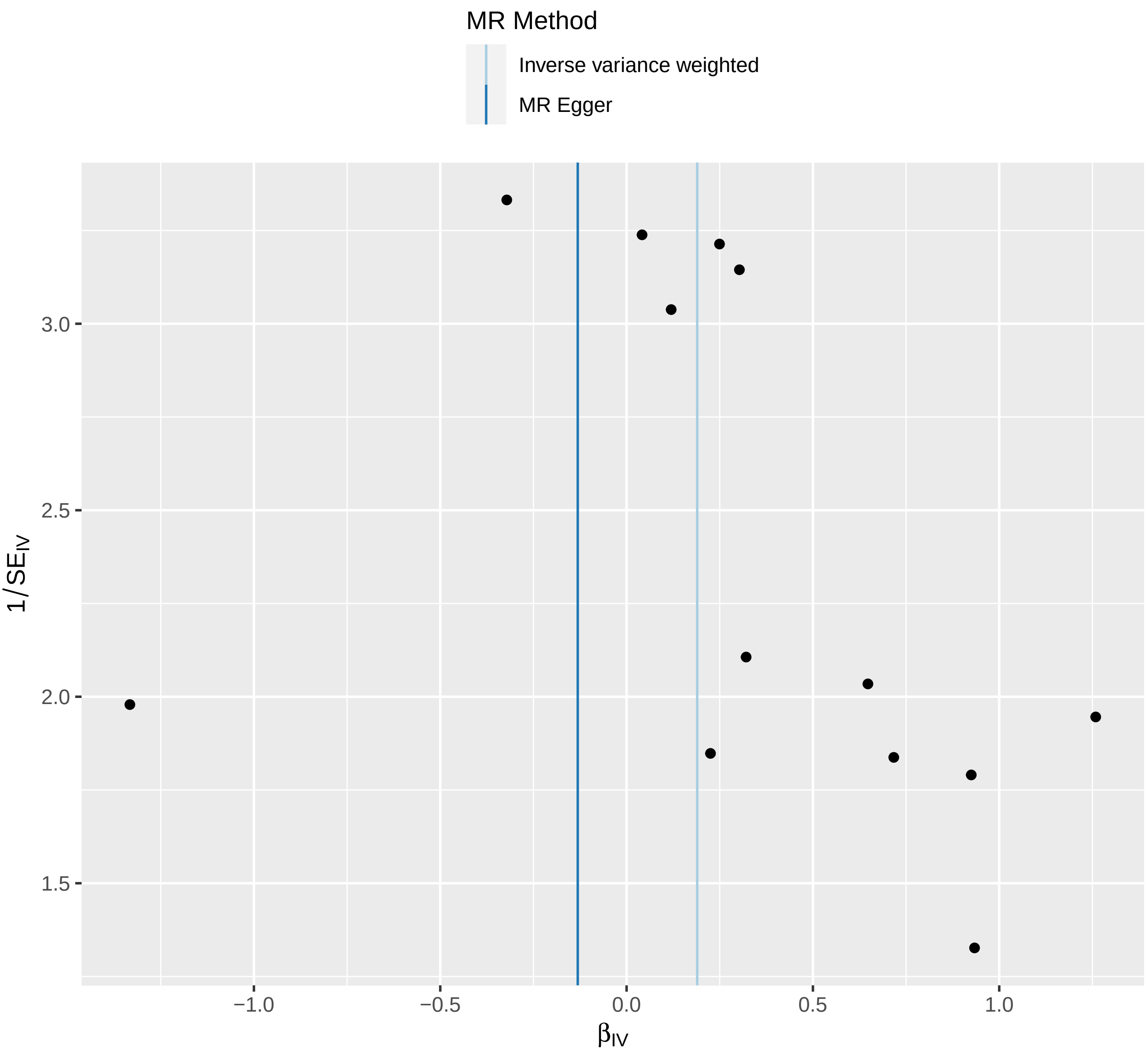
MR Method
Inverse variance weighted
MR Egger
3.0
2.5
2.0
1.5
1/SE$_{IV}$
−1.0
−0.5
0.0
0.5
1.0
β$_{IV}$

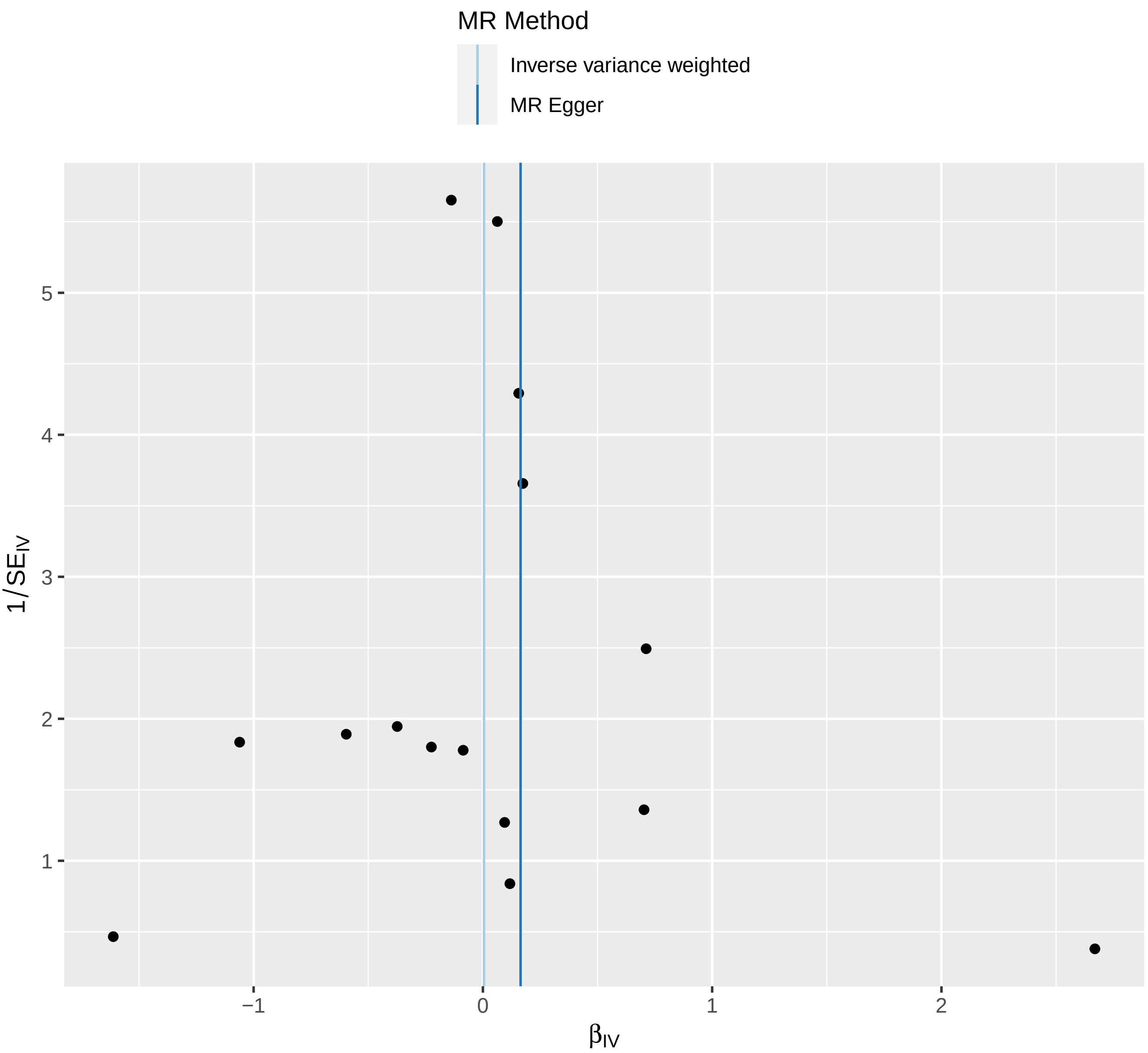
MR Method
Inverse variance weighted
MR Egger
1/SE_IV
β_IV
5
4
3
2
1
−1
0
1
2

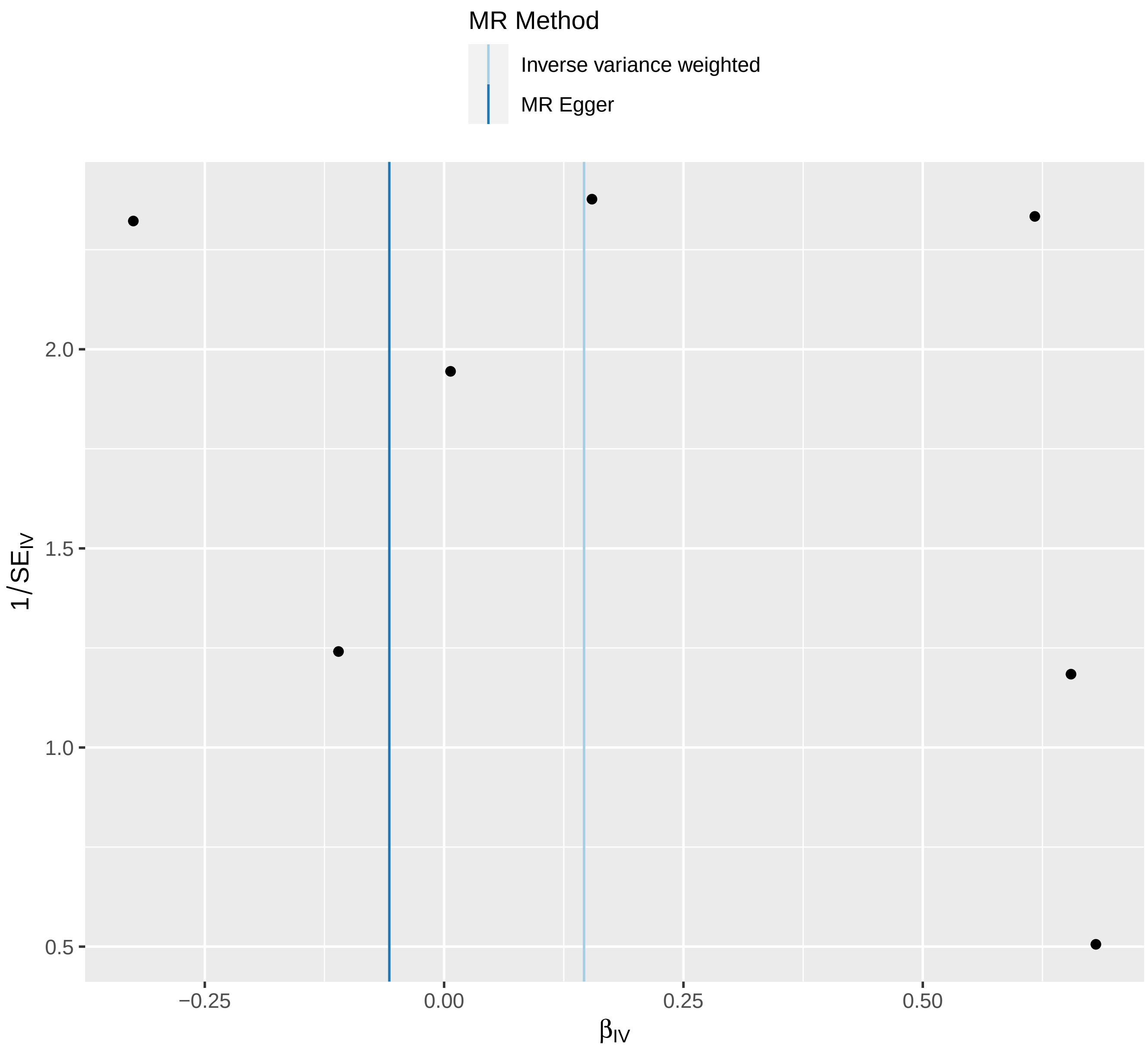

MR Method
Inverse variance weighted
MR Egger
2.0
1.5
1.0
0.5
1/SE_IV
−0.25
0.00
0.25
0.50
β_IV

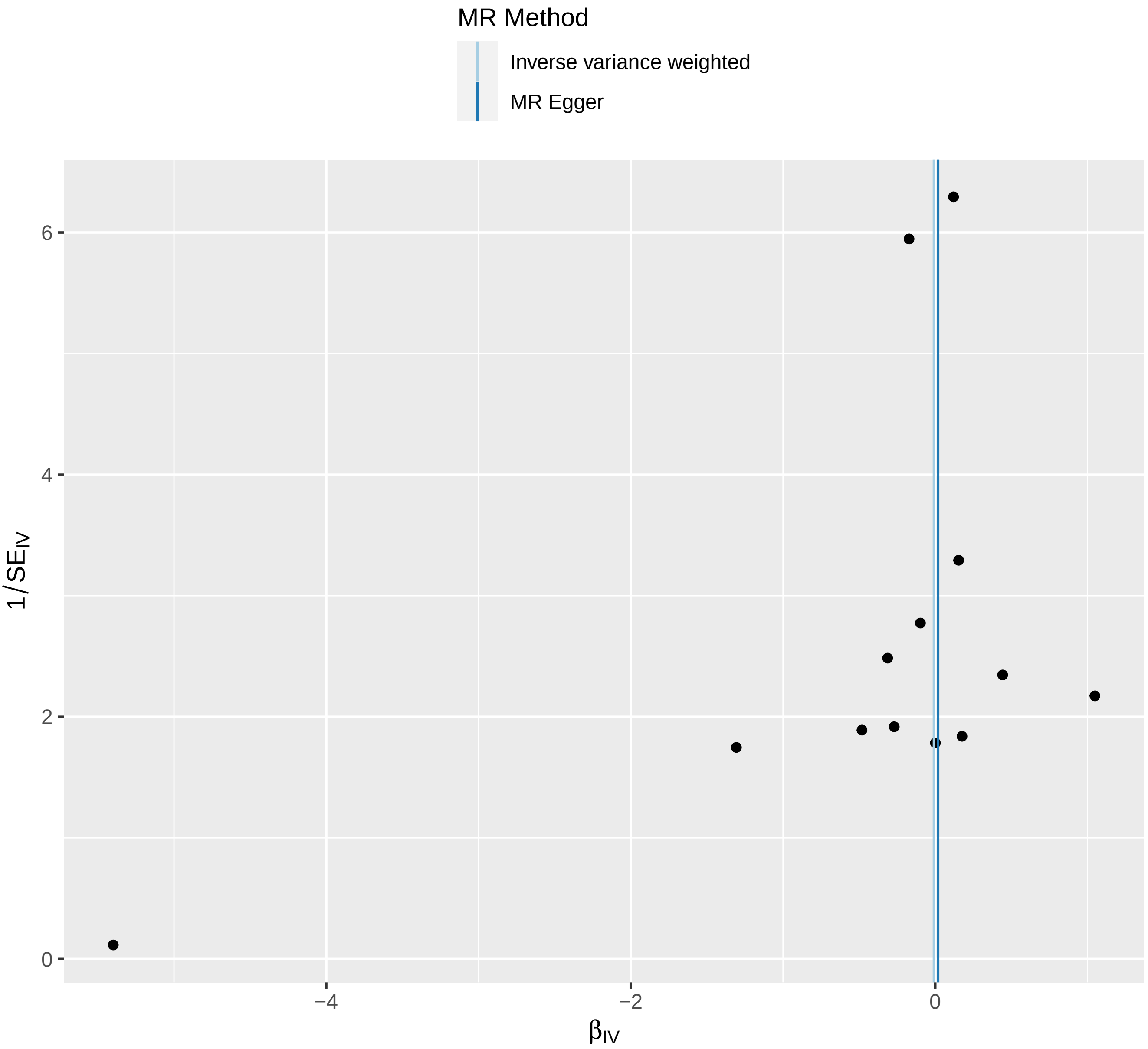

MR Method
Inverse variance weighted
MR Egger
6
4
2
0
1/SE$_{IV}$
−4
−2
0
β_{IV}

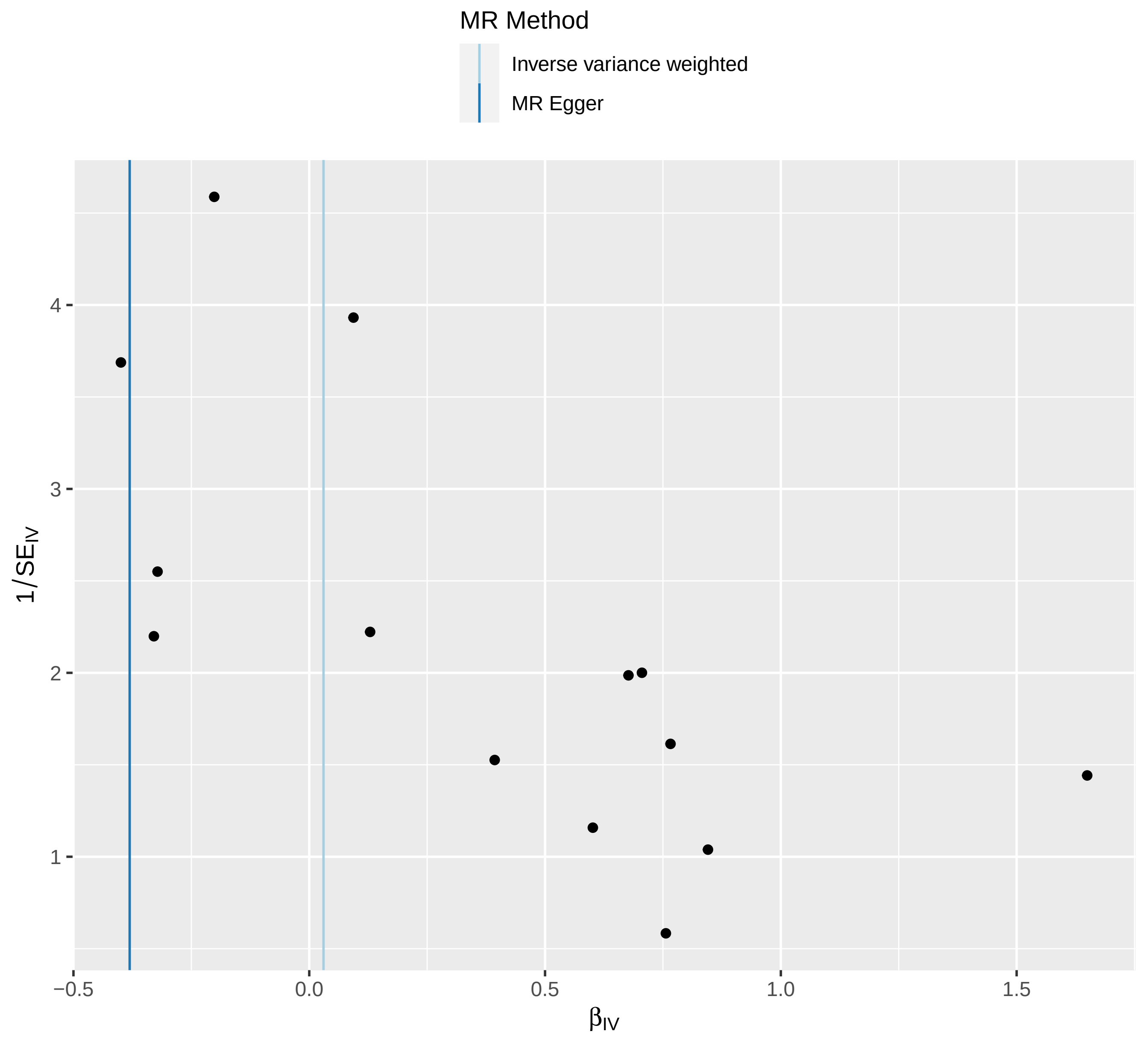
MR Method
Inverse variance weighted
MR Egger
1/SE_IV
4
3
2
1
−0.5
0.0
0.5
1.0
1.5
β_IV

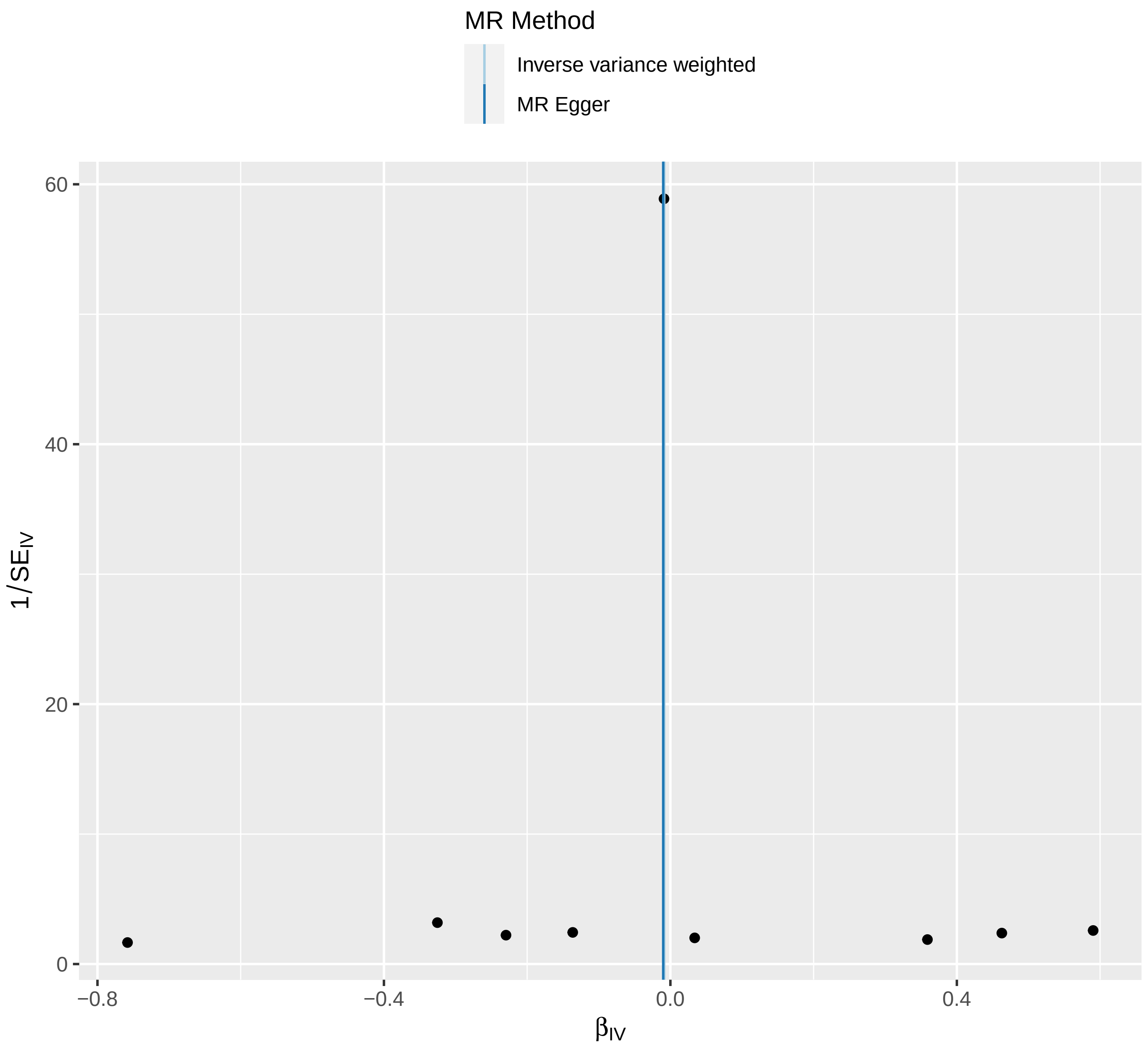
MR Method
Inverse variance weighted
MR Egger
60
40
20
0
1/SE$_{IV}$
−0.8
−0.4
0.0
0.4
β$_{IV}$

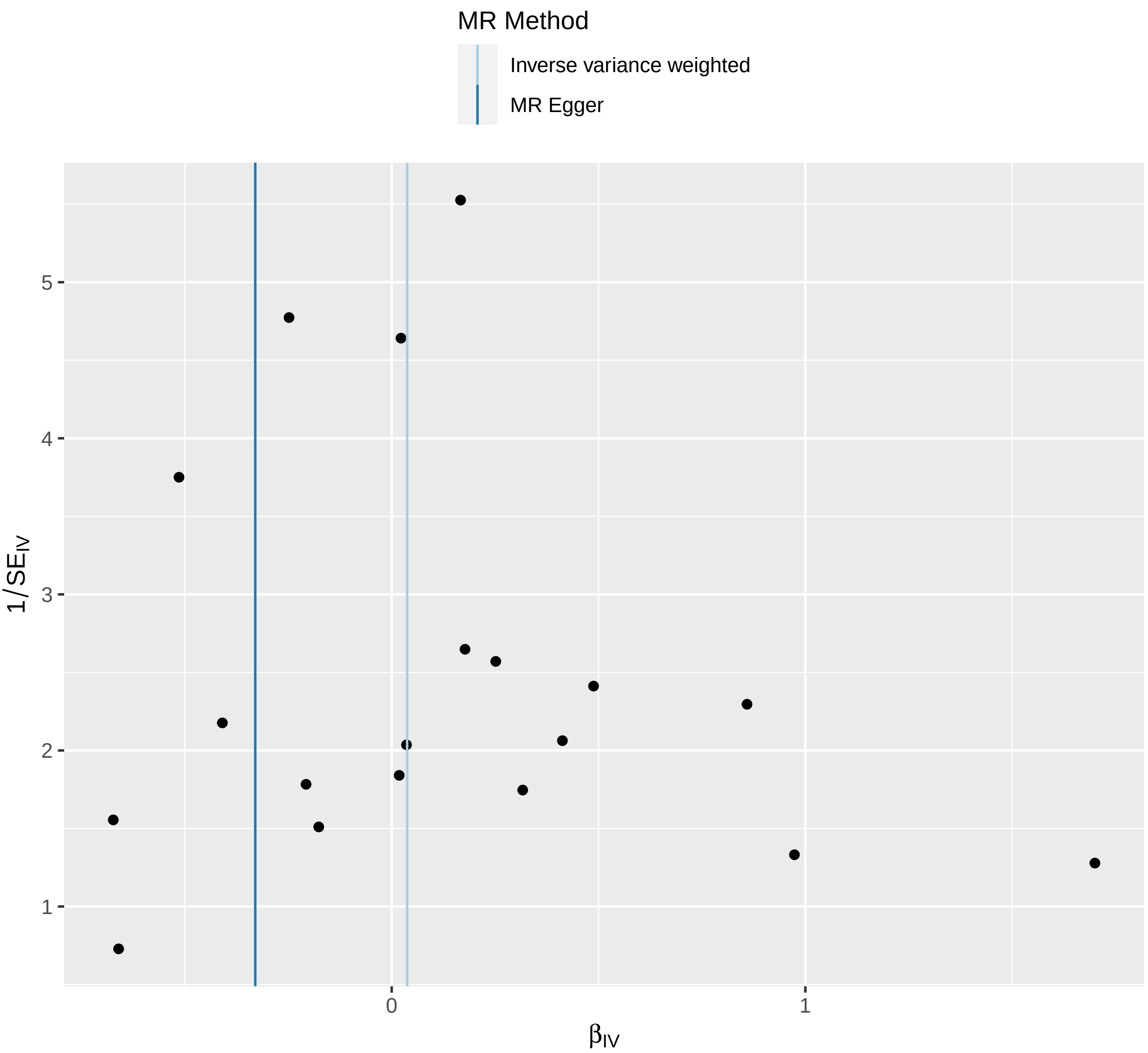
MR Method
Inverse variance weighted
MR Egger
1/SE_{IV}
5
4
3
2
1
0
1
β_{IV}

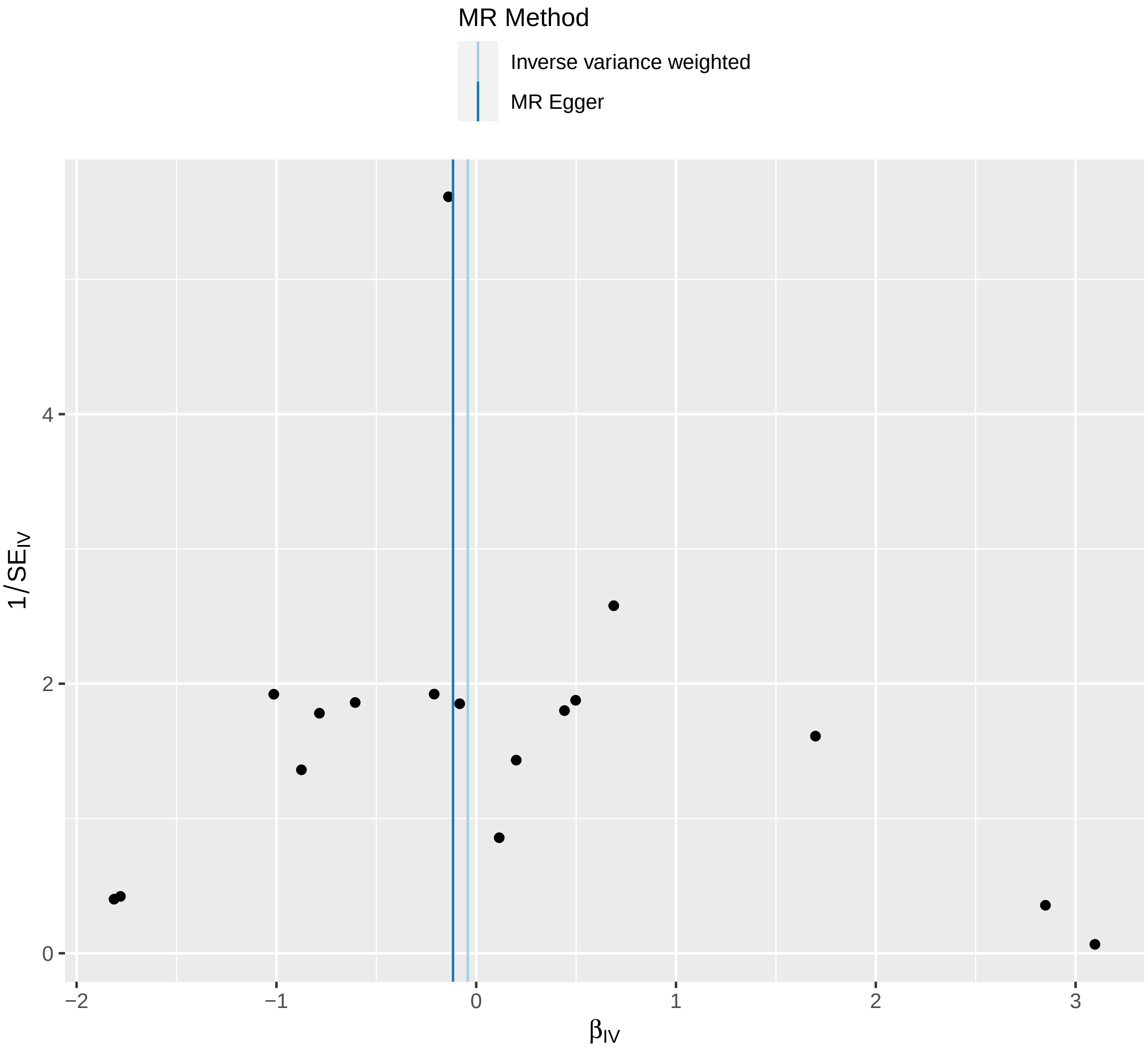

MR Method
Inverse variance weighted
MR Egger
1/SE_IV
4
2
0
−2
−1
0
1
2
3
β_IV
